# Membrane Proteins ClcB, PtsI, and YcaM Mediate the Bactericidal Effects of Colistin in *Escherichia coli*

**DOI:** 10.1021/acsinfecdis.5c00566

**Published:** 2025-10-30

**Authors:** Rhys Donafee, Mohammad Radi, Douglas Bruce Kell, Jesus Enrique Salcedo-Sora

**Affiliations:** † GeneMill Research Facility, Liverpool Shared Research Facilities, 4591 University of Liverpool, Crown Street, Liverpool L69 7ZB, U.K.; ‡ Department of Biochemistry, Cell and Systems Biology, Institute of Systems, Molecular and Integrated Biology, 4591 University of Liverpool, Crown Street, Liverpool L69 7ZB, U.K.; § 587234 The Novo Nordisk Foundation Center for Biosustainability, Søltofts Plads 200, Kgs. Lyngby 2800, Den-Mark

**Keywords:** polymyxins, colistin, membrane transporters, Gram-negative, *E. coli*, Greater wax moth larvae

## Abstract

Bacteria can be killed very effectively by targeting their first line of protection. The cell membranes (outer and inner membranes) of Gram-negative bacteria are directly targeted by antibiotics, such as polymyxins, via electrostatic interactions with their lipopolysaccharide (LPS) fraction. The downstream effects—preceding the cell death—of the disruptions of the bacterial membranes upon the intercalation of these antibiotics are unknown. By screening a set of *E. coli* membrane protein knockouts, the absence of three membrane transporters was shown to influence the susceptibility to colistin. Their involvement was corroborated with growth assays on gain-of-function strains, cytotoxic assays, and *in vivo* infections in an invertebrate animal model. This is the first-time evidence that the disruption caused to membrane proteins, such as the chloride channel ClcB, the sugar transport system component PtsI and the hypothetical glutamate:GABA antiporter YcaM, is part of the cytotoxic pathway that follows or is concomitant to the electrostatic intercalations of polymyxins with the Gram-negative bacterial membrane.

## Graphical abstract

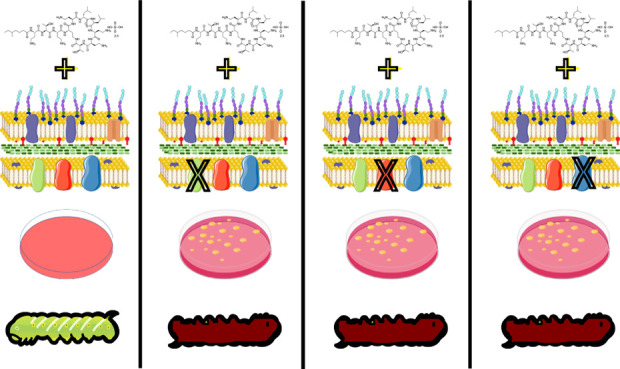

Colistin is currently used as a last resort antibiotic against infections by multidrug-resistant Gram-negative bacilli, such as carbapenemase-producing *Enterobacteriales*, *Pseudomonas aeruginosa*, and *Acinetobacter baumanni*.^1^ The World Health Organization (WHO) has listed colistin as critically important because of the increasing use of colistin to treat serious infections in humans in many parts of the world, the prevalence of *mcr* (mobile colistin resistance) genes, and the spread of colistin-resistant bacteria via the food chain.^2^ Polymyxins such as colistin, and other cell membrane-binding antibiotics, are also effective at targeting antibiotic persister bacteria (stochastic phenotypes that survive high concentrations of antibiotics).^3,4^

Colistin (polymyxin E) is a cyclic lipopolypeptide produced using a nonribosomal synthase by *Bacillus polymyxa* var. colistinus (previously *B. colistinus*).^5,6^ Colistin is a polycation that binds lipid A in the lipopolysaccharide (LPS) fraction of the cell outer membrane (OM) of Gram-negative bacteria, displaces the divalent cations calcium (Ca^2+^) and magnesium (Mg^2+^), and impairs the structure of LPS while inserting its hydrophobic terminal acyl chain. This causes an expansion of the OM, permeabilization of the OM and a further movement of colistin beyond the OM.^7–10^ This seems to lead to an increase in cell permeability and cell barrier disruptions, including destabilization of membranes and loss of cytoplasmic content.^11^

Upon exposure to colistin bacteria show some visible cell membrane disruptions within one^12^ and 2 h.^13^ Other morphological postexposure observations include the formation of bleb-like projections from the cell wall of *E. coli*,^8^ with cytoplasmic material detectable in the extracellular milieu after half an hour of exposure to polymyxins.^14^ However, other reports show cell aggregation rather than cell membrane disruption.^15^

Modes of resistance to colistin have substantiated the cell membrane-binding properties of polymyxins. A reduction in the net negative charge of lipid A affects polymyxin’s affinity for the OM. Enzymes that mediate chemical modifications of lipid A and cause a loss of sensitivity to polymyxins include plasmid-encoded *mcr* gene products,^16^ and two-component regulatory systems and sensor kinase systems such as pmrA/pmrB^17^ and phoP/phoQ.^11,18^ Inhibiting the production of fatty acids destined for lipid A biosynthesis overcomes resistance to colistin altogether.^19^

This said, the mechanisms underlying the bactericidal effects of polymyxins upon their interactions with the outer, and the inner membranes^20^ are not known. Furthermore, the cell permeabilisation and killing properties of polymyxins might not have a causal relationship. Modified versions of polymyxin B (polymyxin B nonapeptide) that still have cell membrane-binding properties and cause leakage of cytoplasmic contents, do not have antibacterial activity.^21^

Part of the answer could be in the role played by the proteins within the inner membrane (cell membrane) of Gram-negative bacteria.^22^ The often generally overlooked integral membrane proteins and membrane-associated proteins are the most abundant component by mass of the cell membranes,^23^ and responsible for the transport of any number of small molecules.^24–26^ It thus seems logical to investigate the involvement of membrane proteins in the bactericidal effects of a membrane-binding antibiotic such as colistin. We describe here that at least three membrane-associated proteins seem to mediate cell death caused by colistin. Specifically, the absence of these proteins caused tolerance while their presence sensitized *E. coli* to this antibiotic. The relevance of these findings to *in vivo* infections is illustrated in an invertebrate animal model.

## Results

### The Absence of Certain Membrane Transporters in *E. coli* Gene Knockouts Reduced Their Sensitivity to Colistin

A set of 534 strains lacking membrane transporters and other membrane associated proteins from the *E. coli* Keio collection of gene knock outs (KOs)^27^ was used to screen for tolerance to colistin. They were exposed to incremental concentrations of colistin up to 5 mg/L in complex media and ranked according to their growth in comparison to that of the reference strain for that collection, the *E. coli* BW25113 strain (Supporting Table S1). From that initial list, eight KO strains were selected for further growth inhibition assays (IC_50_): *ΔclcB, ΔfocB, ΔhisJ, ΔmalX, ΔptsI, ΔrhtC, ΔyadI* and *ΔycaM.* The IC_50_ data from four or more replicates for each of these strains showed that the KO strains *ΔclcB*, *ΔptsI*, *ΔyadI* and *ΔycaM* had IC_50_ median values six to 20-fold that of the reference strain BW25113 (Figure 1). A Welch-Anova analysis showed that there were significant differences in the IC_50_ values for colistin between all different strains (*p* = 2.2e-3). From the pairwise comparisons against the reference strain, the KOs strains *ΔclcB*, *ΔptsI*, *ΔyadI* and *ΔycaM* showed to have IC_50_ values significantly different (Figure 1).

**Figure 1:**
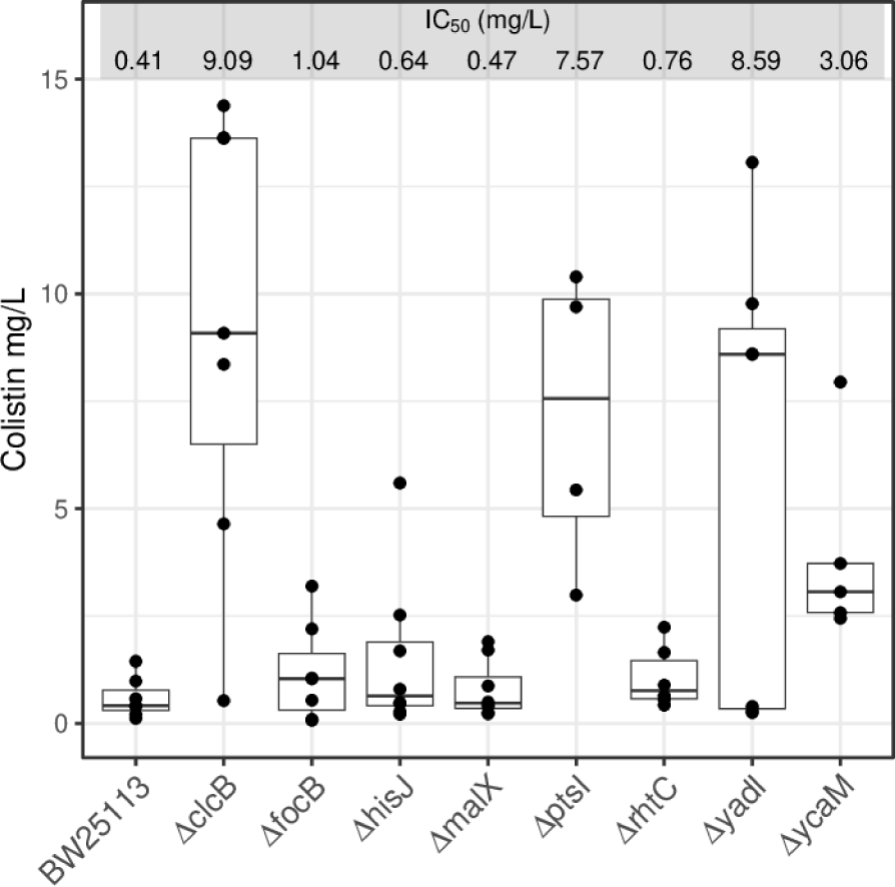
*E. coli* KOs with increased tolerance to colistin. The median for the IC_50_ values of the *E. coli* reference strain BW25113 is presented against those of the eight KO strains initially observed to have different levels of higher tolerance to colistin: *ΔclcB, ΔfocB, ΔhisJ, ΔmalX, ΔptsI, ΔrhtC, ΔyadI* and *ΔycaM.* Data represent at least four biological replicates (black dots). The median values for each IC_50_ range are listed as mg/L. The data had a normal distribution (Shapiro-Wilk’s test *p* = 9.3e-4) with non-homogenous variances between strains (Levene’s test *p* = 4.2e-4). Thus, the pairwise comparisons between BW25113 and *ΔclcB, ΔptsI, ΔyadI* and *ΔycaM* were carried out with the Game-Howell test showing respectively *p* values of 5.5e-3, 2e-2, 3e-2 and 2e-2.

### Expression of the Membrane Transporters *clcB, ptsI* and *ycaM* in the ASKA AG1 Strain Increased Their Sensitivity to Colistin

The availability of the ASKA collection of *E. coli* transformed with plasmids expressing most of the protein-encoding genes for these bacteria allowed us to assess if the opposite (increased sensitivity to colistin) was observed in strains expressing the genes *clcB, ptsI, yadI* and *ycaM*. The ASKA collection uses the *E. coli* K-12 derivative strain *AG1* carrying recombinant constructs in an IPTG-inducible *lacO*-T5 hybrid promoter expression system in the multicopy plasmid pCA24N (*CmR, lacIq*).^28^ The *E. coli* reference strain AG1 as used here was a true control transformed with an empty version of the pCA24N plasmid.

Strains with the recombinant plasmids carrying *clcB*, *ptsI*, *yadI* or *ycaM* (denoted as *AG1+pclcB*, *AG1+pptsI, AG1+ pyadI* and *AG1+pycaM*, respectively) showed IC_50_ median values similar to that of the reference AG1 strain (*AG1+pCA24N*) (Figure 2): 0.09 – 0.2 mg/L. However, when 0.1 mM IPTG was added, IC_50_ values decreased to half or less for those strains carrying recombinant plasmids: 0.05 – 0.07 mg/L; except for the strains carrying the empty vector (*AG1+pCA24N*), and the strain expressing *pyadI* (*AG1+pyadI*) (Figure 2). The latter construct was not included in the next following parts of this work due to the lack of consistent with the expected trends of sensitivity to colistin upon addition of IPTG (Figures 1 and 2).

**Figure 2:**
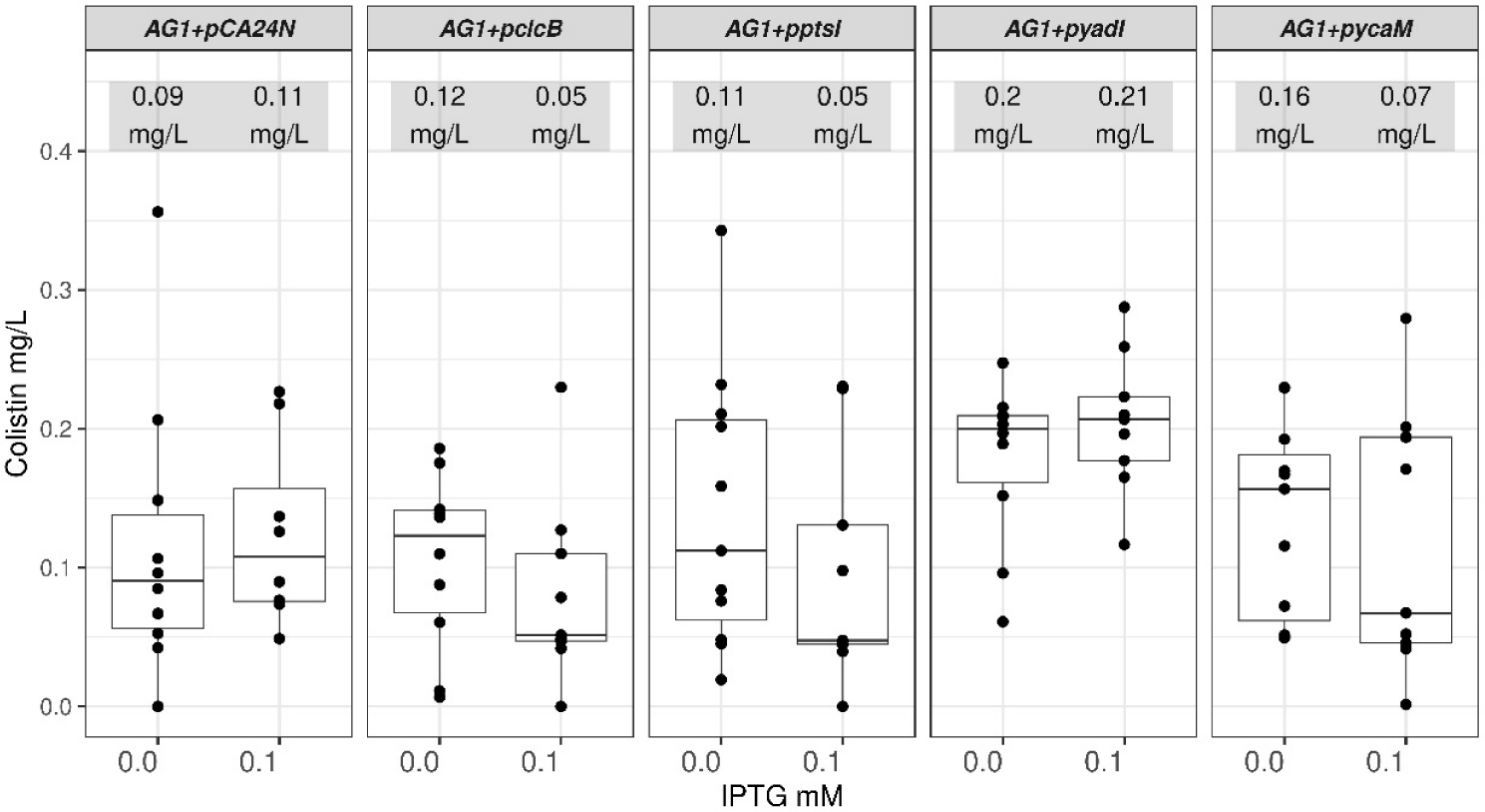
*E. coli* strains expressing *clcB*, *ptsI* and *ycaM* are more sensitive to colistin. The median values for the IC_50_ of the *E. coli* reference strain AG1 carrying the empty pCA24N (*AG1+pCA24N*) plasmid and those strains containing this plasmid with either of four genes were calculated from samples incubated in the absence or the presence of 0.1 mM IPTG. Three of those strains (*AG1+pclcB*, *AG1+pptsI* and *AG1+pycaM*) showed a trend of increased sensitivity to colistin when the cognate membrane transporter was induced. Data illustrated originated from at least eight biological repeats (black dots). The median values for each IC_50_ range are listed as mg/L. The pairwise comparisons within each strain in the absence versus the presence of IPTG had the following *p* values: *p* > 5e-2 for *AG1+pCA24N*, 3.9e-2 for *AG1+pclcB,* 2.8e-2 for *AG1+pptsI,* *p* > 5e-2 for *AG1+pyadI* and 3.9e-2 for *AG1+pycaM*.

Given the differences noted above, the *AG1+pclcB, AG1+pptsI and AG1+pycaM* strains were selected for kinetic growth assays (Methods). In the kinetic assays (Figures 3A and 4) cultures were allowed to grow before adding IPTG and colistin. A greater inoculum size allowed to have more cells expressing these membrane transporters upon addition of IPTG (0.05 mM) after 4 h of growth. After a window of IPTG-driven protein expression colistin was then added. At that time the membrane transporters were expected to have reached detectable protein levels induced by IPTG.^29^ When colistin was added the cell mass of most cultures had reached OD_600_ values up to 0.5, which explains the subsequent growth observed in some of the strains, even in the presence of high colistin concentrations (Figures 3A and 4). The attenuation of the bactericidal activity of colistin by inoculum size has been characterized.^30^

**Figure 3:**
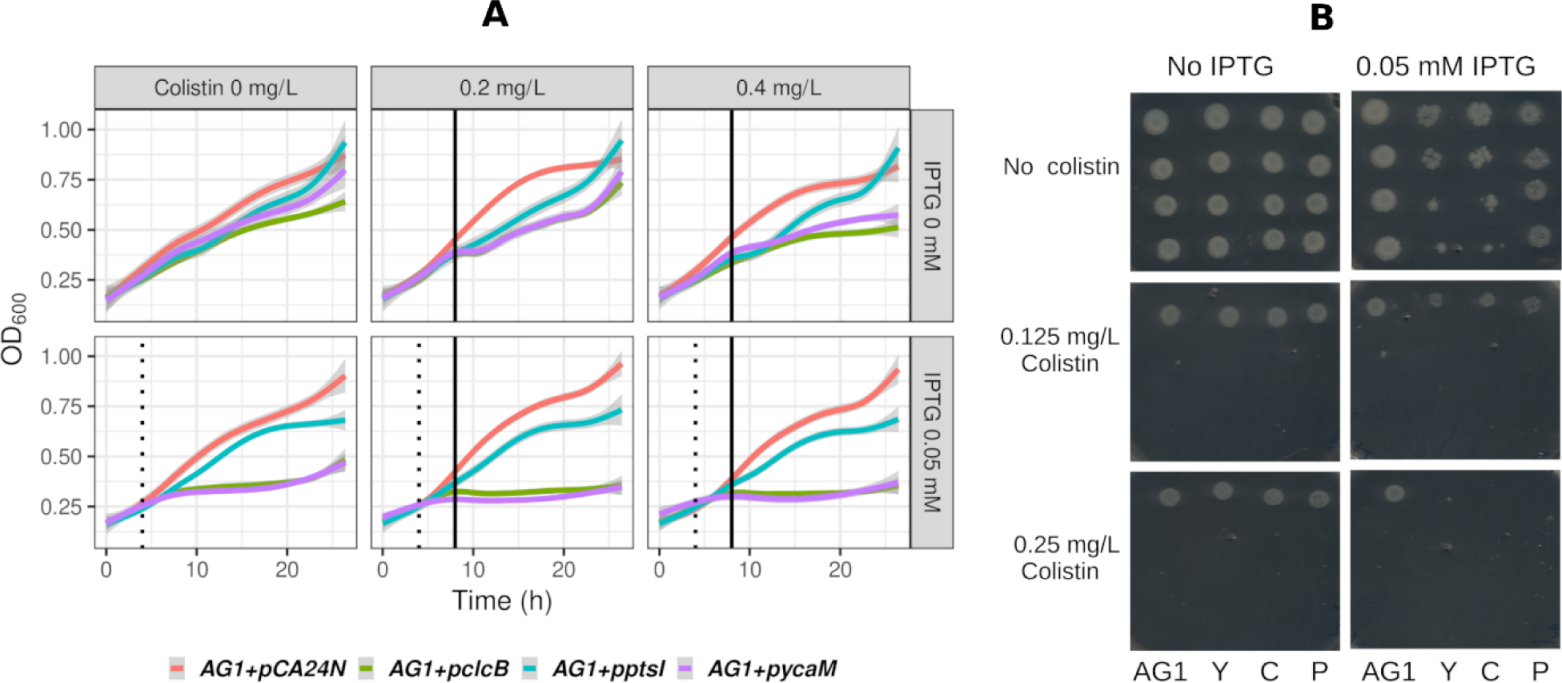
Growth inhibitory effects of colistin in *E. coli* expressing the membrane transporters *clcB*, *ptsI* and *ycaM*. (A) The abscissa represents time. The ordinate represents the culture media optical densities at 600 nm. The AG1 control and the recombinant samples are color-coded: *AG1+pCA24N* is red, *AG1+pclcB* is green, *AG1+pptsI* is blue and *AG1+pycaM* is magenta. Colistin is represented here in the 0.2 and 0.4 mg/L samples. The full range of colistin concentrations analyzed is presented in Table S2. The dotted vertical black line represents the addition of 0.05 mM IPTG at 4 h. The solid vertical black line represents the addition of colistin at 8 h. The data represented in this figure are tabulated in Supporting Table S2. (B).

**Figure 4:**
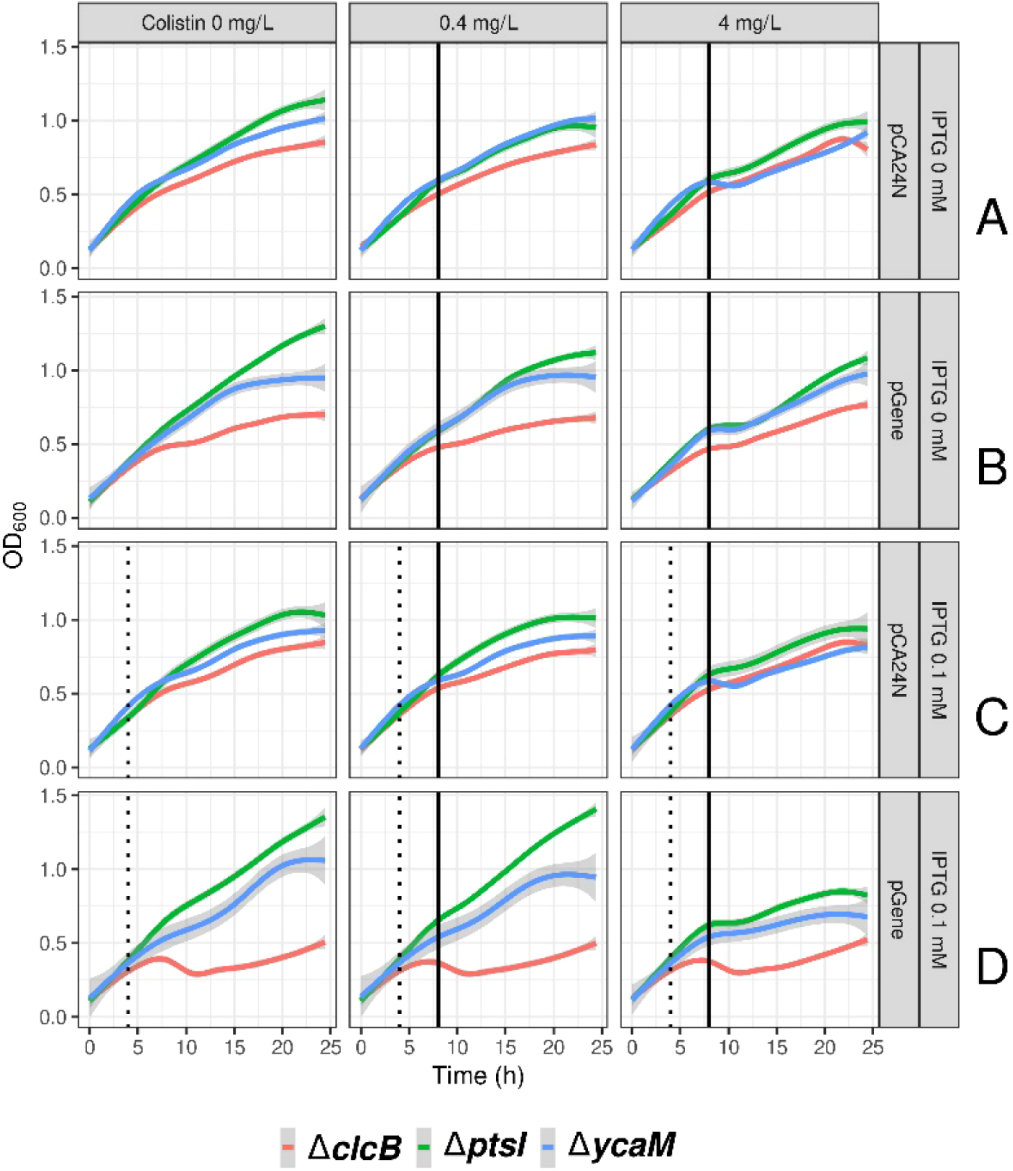
Expression of *ptsI* and *ycaM* resensitizes *E. coli* KO strains to colistin. No-colistin or colistin samples at 0.4 and 4 mg/L are presented column-wise. Rows A and B represent growth (absorbance at OD_600_) in the absence of IPTG. Rows C and D represent growth in the presence of 0.1 mM IPTG. Strains transformed with the empty pCA24N plasmid are in rows A and C. Strains transformed with the recombinant plasmids expressing their cognate genes (denoted as pGene) are in rows B and D. Each KO strain carrying either the empty plasmid or the recombinant plasmid is color coded: *ΔclcB* is red, *ΔptsI* is green and *ΔycaM* is blue. Dashed lines represent the time point for the addition of IPTG while the solid line represents the addition of colistin.

In the kinetic assays all three strains had visible lower culture densities (absorbance at OD_600_) than the *AG1+pCA24N* control in the presence of colistin. Those differences widened in the samples upon induction with 0.05 mM IPTG (Figure 3A and Supporting Table S2), particularly for the *AG1+pclcB* and *AG1+pycaM* samples. The growth of the *AG1+pptsI* samples seemed to have had an intermediate sensitivity to the inhibitory effects of colistin (Figure 3A).

### The Presence of *clcB*, *ptsI,* and *ycaM* Mediated the Growth Inhibition Caused by Colistin in *E. coli*

AG1 is the control strain carrying the empty plasmid pCA24N. Y, C, and P represent the AG1 strains carrying the plasmids *pycaM*. *pclcB* or *pptsI*, respectively. Each strain is represented by four row-wise 1:10 dilutions of culture broth used for spotting. Two different colistin concentrations are shown, in the absence or the presence of IPTG (0.05 mM).

The overexpression of membrane transporters is often toxic to the bacterial host.^31^ This was evident in the growth assays where *AG1+pclcB* and *AG1+pycaM* were visibly inhibited in the IPTG-induced samples in the absence of colistin (Figure 3). However, further assays based on growth in solid media substantiated, at two incremental doses, the observed sensitization to colistin caused by the expression of *clcB*, *ptsI* and *ycaM* additional to the baseline toxicity due to the expression of these proteins upon their induction with IPTG (Figure 4). Here, both colistin and IPTG were present in the solid media and therefore the inoculum for strain was exposed to colistin and IPTG from the beginning as opposed to the planktonic cultures where the initial cultures were allowed to grow beforehand (Figures 3A and 4).

### Gene Complementation Induced Sensitivity to Colistin in Otherwise Tolerant *E. coli* BW25113 KO Strain

Each of the *E. coli* KOs *ΔclcB*, *ΔptsI* and *ΔycaM* from the Keio collection were transformed with either an empty pCA24N plasmid or with a recombinant plasmid carrying their cognate gene *pclcB*, *pptsI* or *pycaM*. The identity of the recombinant constructs in pCA24N plasmids as well as the empty plasmid were all confirmed by DNA sequencing (sequence data available upon request). Equally, whole genome sequencing for the *ΔclcB*, *ΔptsI* and *ΔycaM* strains confirmed their loci to have been replaced by the *S. aureus* *aadD1* gene encoding for an aminoglycoside O-nucleotidyltransferase that confers the kanamycin resistance marker for the *E. coli* Keio KO collection.

The strains *ΔclcB*, *ΔptsI* and *ΔycaM* carrying the recombinant plasmids *pclcB*, *pptsI* and *pycaM*, respectively, had their growth affected in the absence of IPTG (Figure 5B) when compared against the empty plasmid controls (pCA24N, Figure 5A). We interpreted this to be the consequence of a leakier gene expression system in the *E. coli* BW25113 background strain in comparison to the *E. coli* AG1 strains (Figure 3). The BW25113-based strains seemed to have a higher basal lac operon activity, as is sometimes observed when it is assembled with the endogenous bacterial promoter T5,^29^ as is the case of the pCA24N plasmid. Growth was further affected by the presence of 0.1 mM IPTG, particularly for the strain carrying *pclcB* which impeded to observe any further growth inhibition upon addition of colistin in this case. Nevertheless, it was still possible to observe the growth inhibitory effect of colistin in relation to the heterologous expression of *ptsI* and *ycaM* (Figure 5D).

**Figure 5:**
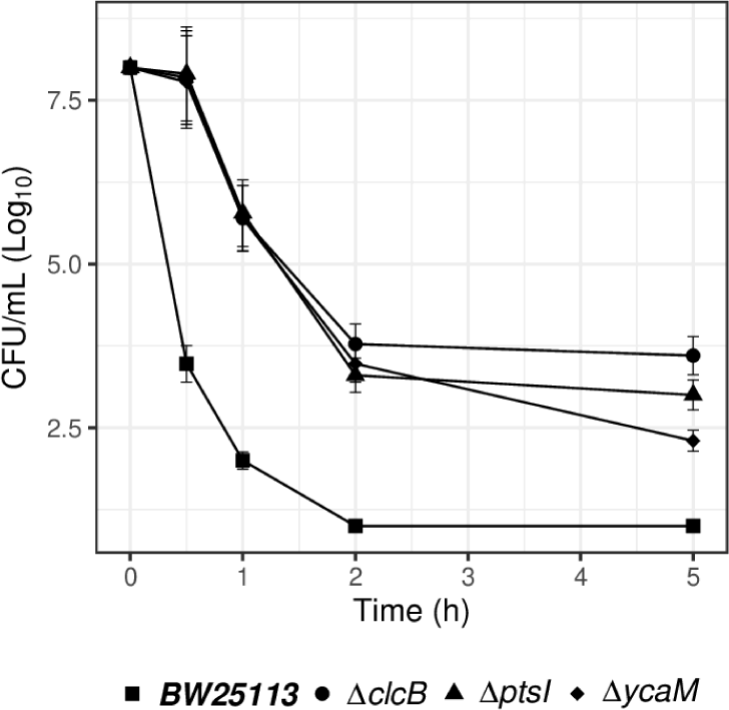
*E. coli* KOs increased tolerance to high concentrations of colistin. Viable cell counts as Log_10_ of CFU/mL are presented for bacteria exposed up to 5 h to 5 mg/L of colistin. The inoculum size is 10^8^ cfu/mL for all strains. The *E. coli* reference strain is BW25113. The three KO strains are *ΔclcB*, *ΔptsI* and *ΔycaM*. Data represent three biological replicates.

### Time-Kill Assays Support the Mediation of *clcB*, *ptsI,* and *ycaM* in the Action of Colistin

We measured the survival of the reference strain in comparison to that of *ΔclcB, ΔptsI* and *ΔycaM* upon exposure to 5 mg/L of colistin. A steep decline in viable cell counts (cfu/mL) indicated a bactericidal effect on BW25113 with a drop of 6 orders of magnitude in the first hour Figure 5). At the same time point the KO strains reduction in viable cells was approximately 3 3 orders of magnitude. By the second and fifth hour the cell viability was still 2 to 3 Log_10_ values higher in the KOs than in BW25113.

### The Absence of *clcB, ptsI,* and *ycaM* Reduces the Efficacy of Colistin *In Vivo*

The larvae of the Greater wax moth (*Galleria mellonella*) provide an invertebrate animal model that is often used to study bacterial infections.^32–35^ A positive correlation between the virulence and immune responses between mammalian models and the innate immune response of *G. mellonella* has been established for several infections.^32,36^ We used larvae of *G. mellonella* to determine potential differences, in the absence as well as the presence of colistin, in the survival of the larvae infected with the *E. coli* reference BW25113 strain or the KOs *ΔclcB, ΔptsI* and Δ*ycaM*. The data were represented as time-to-event analysis using Kaplan–Meier curves and using log rank tests to test for significance between strains. Each larva was injected with 50 ng of colistin. At an average weight of 250 mg, each larva received approximately 0.2 mg/kg of colistin.

In the absence of colistin, at day four the survival probability of larvae infected with *E. coli* BW25113 was 8.2% (Figure 6, Supporting Table S3). When the infected larvae were also injected with colistin, the survival trend increased to 47% for BW25113 at day four (Figure 6). Thus, the presence of colistin increased the survival of larvae infected with the reference strain by over 6-fold.

**Figure 6:**
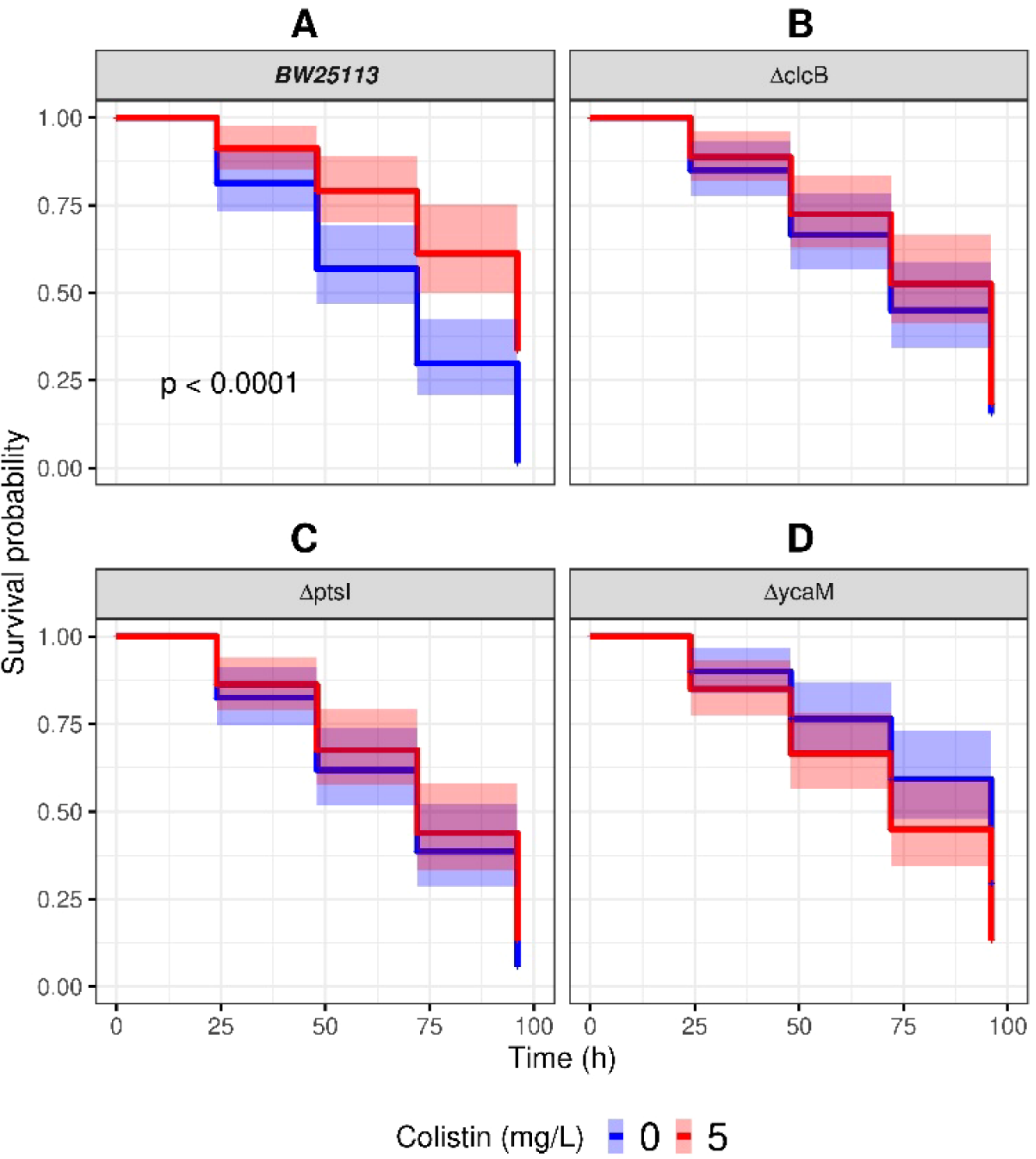
*Galleria mellonella* *larvae infected* *E. coli* KOs have reduced survival rates. Kaplan–Meier survival curves are shown for larvae first infected with the reference strain BW25113 (A) or either of the KOs *ΔclcB* (B), *ΔptsI* (C) and *ΔycaM* (D). Colistin control larvae (blue curves) were subsequently injected with PBS. Data of larvae subsequently injected with 5 mg/L of colistin are represented by the red curve. Data represents up to 80 larvae per group, from at least four different biological replicates. Shaded areas cover the 95% confidence intervals (Supporting Table S3).

The probabilities of survival for the larvae injected with the KOs were 14%, 10%, and 18% for *ΔclcB*, *ΔptsI* and *ΔycaM,* respectively, in the absence of colistin (Figure 6B,D, Supporting Table S3). In the presence of colistin, survival rates were 13%, 12%, and 8% for *ΔclcB*, *ΔptsI* and *ΔycaM,* respectively (Figure 6B,D, Supporting Table S3). Therefore, the presence of the antibiotic made no significant difference to those larvae infected with the KOs.

## Discussion

Membrane lipid composition and integrity can modulate the structure and activity of transport proteins.^37,38^ The derangement of the bacterial cell membranes after its interactions with polymyxins such as colistin^12–15,20,21^ are thus expected to alter the integrity and activity of at least some membrane proteins. Colistin also reaches the cytoplasmic side of the inner membrane whereby it can disrupt the initial movement of LPS into the cell membrane. This would be expected to add to the irreversible changes in the integrity of the bacterial membrane.^22^ This is corroborated by the reports of efflux inhibitors increasing the susceptibility of bacteria to colistin.^39,40^ Altogether, disruptions of cellular homeostasis are expected to be a primary cytotoxic effect caused by permeability disruption.^13^ Our findings here point to specific membrane proteins whose malfunction can affect this disruption of cell homeostasis. Given their location and roles, the dysregulation of PtsI, ClcB and YcaM provides evidence for specific membrane protein dysgenesis as a mediator of the colistin’s lethality.

ClcB is one of the two CLC-type voltage-gated chloride channels found in bacteria, although the function of ClcB is largely unknown.^41^ It is thought to act as an electrical shunt for an outwardly directed proton pump that is linked to amino acid decarboxylation, and is of importance in extreme acid resistance responses.^42^ PtsI is enzyme I of the phosphoenolpyruvate:carbohydrate phosphotransferase system (PTS).^43^ This system mediates the transport of sugars and their intracellular phosphorylation in bacteria. PtsI localizes to the inner surface of the cytoplasmic membrane where it interacts with sugar-specific inner-membrane permeases. PTS systems regulate carbon utilization in switching between the hierarchical (diauxic) and coutilization (nonspecific) use of sugars. YcaM has been classified by sequence similarity as a Glutamate:GABA antiporter, part of the amino acid-polyamine organocation superfamily.^44^ YcaM function has not been characterized. It seems to be expressed when bacteria are in the presence of abundant nutrients *in vitro*.^45^

The Keio collection of *E. coli* gene KO used here has been previously profiled against a set of antibiotics that included colistin.^46^ However, opposite to our approach, these authors assessed only the sensitivity of KOs to antibiotics (we screened for tolerance to colistin). This could explain why the membrane protein-encoding genes reported here do not appear in the list of genes reported in that work as conferring different levels of sensitivity to colistin.^46^ A subsequent study, although polymyxins were not included, showed other membrane transporters mediating the growth inhibitory effects of a number of antibiotics in a subset of the Keio collection.^47^

The dysregulation of ClcB can affect the pH and ionic homeostasis of bacteria. Alkaline pH has a synergistic effect with colistin across several species of Gram-negative.^48^ This observation was extended to intrinsically polymyxin-tolerant Gram-negative bacteria *Burkholderia spp.*.^48^ PtsI has been shown to be involved in ROS-mediated cellular responses to antimicrobials and other environmental stressors, where a lack of PtsI function allowed tolerance to different types of stressors. Such pan-tolerance in the absence of PtsI seems to be based on a reduced capacity for metabolic shifts between different central carbon metabolism pathways. This offers protection from stress-mediated ATP surges, with a concomitant reduced accumulation of ROS.^49^ The potential mechanisms by which YcaM would mediate the toxicity of a membrane-acting antibiotic such as colistin will require further investigation, particularly since the function of this membrane protein is itself still to be characterized. Interestingly, while ClcB and PtsI have homologues in other Gram-negative bacteria, YcaM seems to be conserved only in *E. coli* and missing even in closely related species such as *Salmonella* sp.^45^

The evidence presented here opens new views regarding the mechanism of action of polymyxins. These antibiotics kill the bacterial cell via a series of discernible steps as opposed to a single event of detergent-like cell lysis. In contrast to detergents, the interactions of polymyxins with the cell membranes, mainly with the LPS fraction, are driven by electrostatic interactions (i.e., not hydrophobic), which cause membrane disorganization by antibiotic intercalation and self-promoted permeation.^50^ It is evident that the ensuing cytotoxicity follows then a pathway that includes the disfunction of critical membrane transporters, represented here for *E. coli* by the chloride channel ClcB, the sugar transport system (at least PtsI) and the Glutamate:GABA antiporter YcaM. It is possible that these transporters are directly targeted by colistin. However, the genes encoding them do not seem to have been reported in gene mutation studies of colistin resistance.^51^ We would therefore argue that their disruption is more likely to be secondary to the membrane damage caused by polymyxins.

## Methods

### Strains

The *E. coli* Keio collection of gene KO strains was provided by the National Institute of Genetics, Mishima, Shizuoka, Japan.^27,52^ A subset of 534 strains from the Keio Collection whose cognate genes are annotated as encoding for membrane associated proteins were selected for this study (Supporting Table S1). Individual knockout strains highlighted in this work are as follows: **clcB** *(F-, Δ­(araD-araB)­567, ΔlacZ4787­(::rrnB-3), λ-, ΔclcB740:kan, rph-1, Δ­(rhaD-rhaB)­568, hsdR514);* **ptsI** *(F-, Δ­(araD-araB)­567, ΔlacZ4787­(::rrnB-3), λ-, ΔptsI745:kan, rph-1, Δ­(rhaD-rhaB)­568, hsdR514);* **ycaM** *(F-, Δ­(araD-araB)­567, ΔlacZ4787­(::rrnB-3), λ-, rph-1, ΔycaM723:kan, Δ­(rhaD-rhaB)­568, hsdR514)* ; **ydaI** *(F-, Δ­(araD-araB)­567, ΔlacZ4787­(::rrnB-3), λ-, ΔyadI746:kan, rph-1, Δ­(rhaD-rhaB)­568, hsdR514)*. Whole genome sequencing was used to verify the absence of the cognate genes in the *clcB*, *ptsI* and *ycaM* KO strains (Plasmidsaurus Inc.). We also used strain overexpressing *clcB*, *ptsI* and *ycaM* from the ASKA collection: *E. coli* K-12 derivative strain AG1 [*recA1 endA1 gyrA96 thi-1 hsdR17 (r K– m K+) supE44 relA1*] carrying recombinant constructs for those genes in the IPTG-inducible and multicopy plasmid pCA24N (*CmR, lacIq*).^28^ The composition of those recombinants in pCA24N plasmids was also confirmed by whole plasmid DNA sequencing (Plasmidsaurus Inc.; sequencing data available upon request). The genes pCA24N vectors carry in the ASKA strains have an amino-terminal His-tag. No other further tags (e.g., GFP) are present in these constructs.

### Inhibitory Concentration Assays

Bacterial cultures were carried out routinely in complex media (lysogeny broth, Merck LB 110285).^25^ Colistin was purchased from MP Biomedicals, cat number 194157, activity ≥ 15,000 U/mg. The screening of the Keio collection subset of 534 strains (Supporting Table S1) to incremental concentrations of colistin was carried out by replicating their glycerol stocks into 384-well plates with 50 μL of LB containing 30 μg/mL of kanamycin. These sealed plates were incubated overnight at 37 °C. The overnight cultures were diluted 1 in 100 in 50 μL of fresh LB without kanamycin in polystyrene 384-well plates with a transparent bottom (Sigma M6936–40EA). End-point growth inhibitory concentrations (IC_50_) were calculated from microtitration assays. Overnight cultures in LB were diluted 1 in 100 in fresh LB. Fifty μL of 2-fold dilutions of colistin were prepared in 96-well plates to which 50 μL of fresh bacterial culture were added. End point read outs of the media turbidity at 600 nm (OD_600_) were taken after 24 h at 37 °C.

### Kinetic Assays

Continuous growth assays to follow the effect of protein expression and subsequent exposure to colistin were carried out with freshly 1 in 100 diluted cultures in 180 μL at 30 °C in 96 microwell plates and shaking at 200 rpm. After 4 h protein expression was induced with 0.05 mM IPTG. At 8 h colistin was added to final concentrations 0.05, 0.1, 0.2, 0.4, 0.8, 1.6, 3.2 mg/L. OD_600_ readouts were collected every 15 min in a BMG LabTech CLARIOstar Plus plate reader for 27 h.

### Time-Kill Assays

The inoculum originated from overnight cultures diluted 1:500 in 5 mL of complex media and allowed to grow for 2h, 37 °C and shaking at 250 rpm. An inoculum of 10^8^ cfu/mL showed at least one hundred viable cells in the reference strain BW25113 throughout the 5 h of exposure to 5 mg/L of colistin. Freshly inoculated 5 mL cultures for BW25113 and KO strains were exposed to colistin for up to 5 h. Samples of 0.02 mL were taken from each culture at 0.5, 1, 2, and 5 h. Those volumes were diluted 1:10 in 0.180 mL of complex media, prepared for each strain row-wise in a 96-well plate. Diluted aliquots were plated out in solid complex media in standard Petri dishes and incubated overnight at 37 °C. Viable cells were counted manually from plates with colonies clearly visible and separated from each other

### Gene Complementation

*E. coli* KO strains *ΔclcB, ΔptsI* and *ΔycaM* were transformed with the recombinant plasmids pCA24N carrying either their cognates genes, or empty plasmids as controls. The presence of those plasmids in their respective strains was verified by enzyme restriction analysis before assessing their antibiotic sensitivity. The IC_50_ assays for colistin were carried out as described above.

### Larval Survival Assay

Larvae of the Greater wax moth *Galleria mellonella* were purchased from Livefoods UK Ltd. (Somerset, UK). Larvae were injected the same day upon delivery. Healthy large larvae (approximately 250 – 350 mg body weight) were selected. They had a uniform cream color without spots or marks indicating melanisation. Ten larvae per condition were injected with 10^4^ cells of *E. coli* (for both reference strain and KO strains) in10 μL in either of the penultimate prolegs, using a 10 μL Hamilton 750 syringe (Hamilton Company, UK). After injections, the larvae were placed into Petri dishes and incubated at 37 °C. Syringes were cleaned with 70% ethanol between groups of injection. Larvae mortality was followed for 96 h by counting death individuals (black or dark brown in color) every 24 h. Colistin was injected in the opposite penultimate proleg, 30 min after they had been injected with bacteria. The control group was injected with phosphate buffer saline (PBS).

### Data Analysis

The inhibitory concentrations of colistin that kills half of the bacteria population (IC_50_) were calculated with the four-parameter logistic model as implemented in the R package *drc* ^53,54^ and python package py50,^55^ following the guidelines for relative calculations according to the spread of the data.^56^ The Shapiro-Wilk test, Levene’s test, Welch-Anova and Games-Howell’s posthoc tests (pairwise comparisons) were carried out using the required packages in R.^53^ Visualization and annotation of bacterial genomes and plasmid sequences used Proksee.^57^ Survival analyses of larvae were carried out with the time to event model Kaplan–Meier which estimates survival differences in survival times between compared groups as implemented in the R packages survival and survminer.

## Supplementary Material



## Notes

Three tables have been included in this section. Table S1 contains the screening with increasing concentrations of colistin of the *E. coli* Keio collection of 534 of knockouts of genes encoding membrane proteins. Table S2 is a large table with the data for the kinetic growth assays of *E. coli* ASKA strains expressing *clcB*, *pstI* and *ycaM* and exposed to increasing concentrations of colistin. Table S3 contains the data for the survival assays carried out in larvae as presented in Figure 6 in the main text of the manuscript (PDF)

## ⊥

Department of Tropical Disease Biology, Liverpool School of Tropical Medicine, Liverpool, L3 5QA, U.K

## Abbreviations

ClcB: CLC-type voltage-gated chloride channel
PtsI: enzyme I of the phosphoenolpyruvate:carbohydrate phosphotransferase system
YcaM: Glutamate:GABA antiporter, part of the amino acid-polyamine organocation superfamily

## References

[1] Andrade F. F., Silva D., Rodrigues A., Pina-Vaz C. (2020). Colistin Update on
Its Mechanism of Action and Resistance, Present and Future Challenges. Microorganisms.

[2] World Health Organization (WHO) WHO List of Medically Important Antimicrobia: antimicrobial Resistance Division (AMR) Department of Global Coordination and Partnership of Antimicrobial Resistance; WHO: Geneva, Switzerland, 2024.

[3] Salcedo-Sora J. E., Kell D. B. (2020). A Quantitative
Survey of Bacterial Persistence in the
Presence of Antibiotics: Towards Antipersister Antimicrobial Discovery. Antibiotics.

[4] Cui P., Niu H., Shi W., Zhang S., Zhang H., Margolick J., Zhang W., Zhang Y. (2016). Disruption of Membrane
by Colistin
Kills Uropathogenic *Escherichia coli* Persisters and Enhances Killing of Other Antibiotics. Antimicrob. Agents Chemother..

[5] Ito M., Aida K., Uemura T. (1970). Biosynthesis
of colistin by Bacillus
colistinus Koyama. Biochim. Biophys. Acta, Protein
Struct. Mol. Enzymol..

[6] Tambadou F., Caradec T., Gagez A. L., Bonnet A., Sopena V., Bridiau N., Thiery V., Didelot S., Barthelemy C., Chevrot R. (2015). Characterization of
the colistin (polymyxin E1 and
E2) biosynthetic gene cluster. Arch. Microbiol..

[7] Davis S. D., Iannetta A., Wedgwood R. J. (1971). Activity of colistin against *Pseudomonas aeruginosa*: inhibition by calcium. J. Infect. Dis..

[8] Koike M., Iida K., Matsuo T. (1969). Electron microscopic
studies on mode
of action of polymyxin. J. Bacteriol..

[9] Cooperstock M. S. (1974). Inactivation
of endotoxin by polymyxin B. Antimicrob. Agents
Chemother..

[10] Li J., Nation R. L., Milne R. W., Turnidge J. D., Coulthard K. (2005). Evaluation
of colistin as an agent against multi-resistant Gram-negative bacteria. Int. J. Antimicrob. Agents.

[11] Hussein N. H., Al-Kadmy I. M. S., Taha B. M., Hussein J. D. (2021). Mobilized colistin
resistance (mcr) genes from 1 to 10: a comprehensive review. Mol. Biol. Rep..

[12] Mohamed Y. F., Abou-Shleib H. M., Khalil A. M., El-Guink N. M., El-Nakeeb M. A. (2016). Membrane
permeabilization of colistin toward pan-drug resistant Gram-negative
isolates. Braz. J. Microbiol..

[13] Kaye J. J., Chapman G. B. (1963). Cytological Aspects
of Antimicrobial Antibiosis. Iii.
Cytologically Distinguishable Stages in Antibiotic Action of Colistin
Sulfate on *Escherichia Coli*. J. Bacteriol..

[14] Ito M., Koyama Y. (1972). Jolipeptin, a new peptide
antibiotic. II. The mode
of action of jolipeptin. J. Antibiot. (Tokyo).

[15] O’Driscoll N. H., Cushnie T. P. T., Matthews K. H., Lamb A. J. (2018). Colistin causes
profound morphological alteration but minimal cytoplasmic membrane
perforation in populations of *Escherichia coli* and *Pseudomonas aeruginosa*. Arch. Microbiol..

[16] Sun J., Zhang H., Liu Y. H., Feng Y. (2018). Towards Understanding
MCR-like Colistin Resistance. Trends Microbiol..

[17] McPhee J. B., Lewenza S., Hancock R. E. (2003). Cationic antimicrobial peptides activate
a two-component regulatory system, PmrA-PmrB, that regulates resistance
to polymyxin B and cationic antimicrobial peptides in *Pseudomonas
aeruginosa*. Mol. Microbiol..

[18] Macfarlane E. L. A., Kwasnicka A., Hancock R. E. W. (2000). Role of *Pseudomonas aeruginosa* PhoP-phoQ in resistance to antimicrobial cationic peptides and aminoglycosides. Microbiology.

[19] Carfrae L. A., Rachwalski K., French S., Gordzevich R., Seidel L., Tsai C. N., Tu M. M., MacNair C. R., Ovchinnikova O. G., Clarke B. R. (2023). Inhibiting fatty acid
synthesis overcomes colistin resistance. Nat.
Microbiol..

[20] Hancock R. E., Chapple D. S. (1999). Peptide antibiotics. Antimicrob.
Agents Chemother..

[21] Dixon R. A., Chopra I. (1986). Polymyxin B and polymyxin
B nonapeptide alter cytoplasmic
membrane permeability in *Escherichia coli*. J. Antimicrob. Chemother..

[22] Sabnis A., Hagart K. L., Klockner A., Becce M., Evans L. E., Furniss R. C. D., Mavridou D. A., Murphy R., Stevens M. M., Davies J. C. (2021). Colistin
kills bacteria by targeting lipopolysaccharide
in the cytoplasmic membrane. eLife.

[23] Kell D. B. (1986). On the
lateral mobility of proteins in prokaryotic membranes. Biochem. Soc. Trans..

[24] Jindal S., Yang L., Day P. J., Kell D. B. (2019). Involvement of multiple
influx and efflux transporters in the accumulation of cationic fluorescent
dyes by *Escherichia coli*. BMC Microbiol..

[25] Salcedo-Sora J. E., Jindal S., O’Hagan S., Kell D. B. (2021). A palette of fluorophores
that are differentially accumulated by wild-type and mutant strains
of *Escherichia coli*: surrogate ligands
for profiling bacterial membrane transporters. Microbiology.

[26] Kell D. B. (2021). The Transporter-Mediated
Cellular Uptake and Efflux of Pharmaceutical Drugs and Biotechnology
Products: How and Why Phospholipid Bilayer Transport Is Negligible
in Real Biomembranes. Molecules.

[27] Baba T., Ara T., Hasegawa M., Takai Y., Okumura Y., Baba M., Datsenko K. A., Tomita M., Wanner B. L., Mori H. (2006). Construction
of *Escherichia coli* K-12 in-frame,
single-gene knockout mutants: the Keio collection. Mol. Syst. Biol..

[28] Kitagawa M., Ara T., Arifuzzaman M., Ioka-Nakamichi T., Inamoto E., Toyonaga H., Mori H. (2006). Complete set of ORF clones of *Escherichia coli* ASKA library (a complete set of *E. coli* K-12 ORF archive): unique resources for biological research. DNA Res..

[29] Nielsen B. L., Willis V. C., Lin C. Y. (2007). Western blot analysis to illustrate
relative control levels of the lac and ara promoters in *Escherichia coli*. Biochem.
Mol. Biol. Educ..

[30] Bulitta J. B., Yang J. C., Yohonn L., Ly N. S., Brown S. V., D’Hondt R. E., Jusko W. J., Forrest A., Tsuji B. T. (2010). Attenuation
of colistin bactericidal activity by high inoculum of *Pseudomonas
aeruginosa* characterized by a new mechanism-based population
pharmacodynamic model. Antimicrob. Agents Chemother..

[31] Gubellini F., Verdon G., Karpowich N. K., Luff J. D., Boel G., Gauthier N., Handelman S. K., Ades S. E., Hunt J. F. (2011). Physiological
response to membrane protein overexpression in *E. coli*. Mol. Cell. Proteomics.

[32] Menard G., Rouillon A., Cattoir V., Donnio P. Y. (2021). *Galleria
mellonella* as a Suitable Model of Bacterial Infection: Past,
Present and Future. Front. Cell. Infect. Microbiol..

[33] Asai M., Li Y., Newton S. M., Robertson B. D., Langford P. R. (2023). *Galleria
mellonella*-intracellular bacteria pathogen infection models:
the ins and outs. FEMS Microbiol. Rev..

[34] Pereira M. F., Rossi C. C., da Silva G. C., Rosa J. N., Bazzolli D. M. S. (2020). *Galleria mellonella* as an infection model: an in-depth look
at why it works and practical considerations for successful application. Pathog. Dis..

[35] Tsai C. J., Loh J. M., Proft T. (2016). *Galleria mellonella* infection models for the study of bacterial diseases and for antimicrobial
drug testing. Virulence.

[36] Hesketh-Best P. J., Mouritzen M. V., Shandley-Edwards K., Billington R. A., Upton M. (2021). *Galleria mellonella* larvae exhibit a weight-dependent
lethal median dose when infected with methicillin-resistant *Staphylococcus aureus*. Pathog. Dis..

[37] Stieger B., Steiger J., Locher K. P. (2021). Membrane
lipids and transporter function. Biochim. Biophys.
Acta, Mol. Basis Dis..

[38] Gupta K., Donlan J. A. C., Hopper J. T. S., Uzdavinys P., Landreh M., Struwe W. B., Drew D., Baldwin A. J., Stansfeld P. J., Robinson C. V. (2017). The role of interfacial lipids in
stabilizing membrane protein oligomers. Nature.

[39] Park Y. K., Ko K. S. (2015). Effect of carbonyl cyanide 3-chlorophenylhydrazone (CCCP) on killing
Acinetobacter baumannii by colistin. J. Microbiol..

[40] Baron S. A., Rolain J. M. (2018). Efflux pump inhibitor CCCP to rescue colistin susceptibility
in mcr-1 plasmid-mediated colistin-resistant strains and Gram-negative
bacteria. J. Antimicrob. Chemother..

[41] Kim M., Choi N., Choi E., Lee E. J. (2023). ClC Chloride Channels
in Gram-Negative Bacteria and Its Role in the Acid Resistance Systems. J. Microbiol. Biotechnol..

[42] Iyer R., Iverson T. M., Accardi A., Miller C. (2002). A biological role for
prokaryotic ClC chloride channels. Nature.

[43] Lim S., Seo H. S., Jeong J., Yoon H. (2019). Understanding the multifaceted
roles of the phosphoenolpyruvate: Phosphotransferase system in regulation
of *Salmonella* virulence using a mutant
defective in ptsI and crr expression. Microbiol.
Res..

[44] Saier M. H., Reddy V. S., Tamang D. G., Vastermark A. (2014). The transporter
classification database. Nucl Acids Res..

[45] Serina S., Nozza F., Nicastro G., Faggioni F., Mottl H., Deho G., Polissi A. (2004). Scanning the *Escherichia
coli* chromosome by random transposon mutagenesis and
multiple phenotypic screening. Res. Microbiol..

[46] Liu A., Tran L., Becket E., Lee K., Chinn L., Park E., Tran K., Miller J. H. (2010). Antibiotic sensitivity
profiles determined with an *Escherichia coli* gene knockout collection: generating an antibiotic bar code. Antimicrob. Agents Chemother..

[47] Munro L. J., Kell D. B. (2022). Analysis of a Library
of *Escherichia
coli* Transporter Knockout Strains to Identify Transport
Pathways of Antibiotics. Antibiotics.

[48] Panta P. R., Doerrler W. T. (2021). A link between pH
homeostasis and colistin resistance
in bacteria. Sci. Rep..

[49] Zeng J., Hong Y., Zhao N., Liu Q., Zhu W., Xiao L., Wang W., Chen M., Hong S., Wu L. (2022). A broadly applicable,
stress-mediated bacterial death
pathway regulated by the phosphotransferase system (PTS) and the cAMP-Crp
cascade. Proc. Natl. Acad. Sci. U. S. A..

[50] Cetuk H., Anishkin A., Scott A. J., Rempe S. B., Ernst R. K., Sukharev S. (2021). Partitioning of Seven Different Classes of Antibiotics
into LPS Monolayers Supports Three Different Permeation Mechanisms
through the Outer Bacterial Membrane. Langmuir.

[51] Abavisani M., Bostanghadiri N., Ghahramanpour H., Kodori M., Akrami F., Fathizadeh H., Hashemi A., Rastegari-Pouyani M. (2023). Colistin resistance
mechanisms in Gram-negative bacteria: a Focus on *Escherichia
coli*. Lett. Appl. Microbiol..

[52] Yamamoto N., Nakahigashi K., Nakamichi T., Yoshino M., Takai Y., Touda Y., Furubayashi A., Kinjyo S., Dose H., Hasegawa M. (2009). Update on the Keio collection of *Escherichia coli* single-gene deletion mutants. Mol. Syst. Biol..

[53] R Core Team R: A Language and Environment for Statistical Computing. https://www.R-project.org/. accessed December 2024, 2020.

[54] Ritz C., Baty F., Streibig J. C., Gerhard D. (2015). Dose-Response Analysis
Using R. PLoS One.

[55] Lin, T. E. py50: generate Dose-Response Curves (v1.0.4) Zenodo 2024 10.5281/zenodo.11195022

[56] Sebaugh J. L. (2011). Guidelines
for accurate EC50/IC50 estimation. Pharm. Stat..

[57] Grant J. R., Enns E., Marinier E., Mandal A., Herman E. K., Chen C. Y., Graham M., Van Domselaar G., Stothard P. (2023). Proksee: in-depth characterization
and visualization
of bacterial genomes. Nucleic Acids Res..

