# Supplementary material for: Membrane Proteins ClcB, PtsI, and YcaM Mediate the Bactericidal Effects of Colistin in Escherichia coli
Source: ACS Infect Dis. 2025 Oct 30;11(11):3166–74. doi: 10.1021/acsinfecdis.5c00566 (PMC12624714; doi:10.1021/acsinfecdis.5c00566)
Supplement: Supplementary file 1 [file id5c00566_si_001.pdf]

Supporting Information

Membrane proteins ClcB, PtsI and YcaM mediate the bactericidal effects of colistin in *Escherichia coli*

Rhys Donafee [2], Mohammad Radi [3], Douglas Bruce Kell [1,2,3] and Jesus Enrique Salcedo-Sora [1,4,*]

1 GeneMill Research Facility, Liverpool Shared Research Facilities, University of Liverpool, Crown Street, Liverpool, UK, L69 7ZB

2 Department of Biochemistry, Cell and Systems Biology; Institute of Systems, Molecular and Integrated Biology, University of Liverpool, Crown Street, Liverpool, UK, L69 7ZB; dbk@liverpool.ac.uk [ORCID 0000-0001-5838-7963]

3 The Novo Nordisk Foundation Center for Biosustainability, Søltofts Plads 200, 2800 Kgs. Lyngby, Den-mark

4 Current affiliation: Department of Tropical Disease Biology, Liverpool School of Tropical Medicine, Liverpool, UK, L3 5QA; [ORCID 0000-0002-8756-0080]

* Correspondence to: J. Enrique Salcedo-Sora, enrique.salcedo-sora@lstmed.ac.uk

	Table of Contents	Page
**Table S1**	Growth as OD600 in *E. coli* KO strains	2
**Table S2**	Growth data for the *E. coli* ASKA strains	8
**Table S3**	Survival in larvae exposed to *E. coli* KO strains	806

Table S1. Growth as OD600 in *E. coli* KO strains

Strain	Growth_LB_Kan	Growth_LB_colistin_1ug-ml	Growth_LB_colistin_1ug-ml_again	Growth_LB_colistin_2.5ug-ml	Growth_LB_colistin_5ug-ml
aroP	1.678	0.861	1.695	0.358	1.905
clcB	1.485	1.259	1.768	1.716	1.757
yadI	1.959	0.975	1.718	1.808	1.625
ycaM	1.459	0.88	0.971	0.941	1.218
rhtC	2.126	1.151	1.439	0.695	1.144
focB	1.426	0.984	1.134	1.05	1.074
ptsI	1.96	1.13	1.69	1.527	0.897
ycaD	1.912	0.955	0.62	0.82	0.782
yraQ	1.658	0.243	0.347	0.504	0.727
crr	2.022	0.971	1.544	0.296	0.428
phnK	1.574	0.948	0.203	0.35	0.424
sapC	1.555	0.936	0.242	0.338	0.421
oppB	1.607	0.655	0.302	0.368	0.402
ybhG	1.44	0.797	1.593	0.36	0.396
ulaB	1.842	1	1.58	0.3	0.383
artI	1.94	0.76	0.934	0.297	0.359
yidE	1.767	0.814	0.256	0.278	0.356
btuF	2.081	0.557	0.263	0.282	0.336
ydcT	1.839	0.958	1.764	0.288	0.326
gadC	1.782	0.821	1.49	0.729	0.324
pitA	2.222	0.358	0.22	0.296	0.322
ybhS	2.036	0.334	0.255	0.274	0.32
oppD	1.834	0.918	1.466	0.279	0.311
yicE	0.761	0.948	0.291	0.27	0.311
mgtA	1.928	0.999	0.273	0.289	0.308
putP	1.764	0.878	1.258	0.301	0.306
marA	0.989	0.964	0.249	0.302	0.301
uhpC	2.016	0.968	0.261	0.288	0.297
sapB	1.793	0.895	0.253	0.296	0.294
atpF	1.514	0.994	0.275	0.291	0.288
betT	1.932	0.892	0.28	0.282	0.288
yieG	2.353	0.46	0.239	0.329	0.287
ynjB	1.75	0.838	0.223	0.276	0.287
chaA	1.68	0.812	1.457	0.312	0.285
glvC	2.29	1.11	0.24	0.258	0.283
mtlA	2.088	0.86	0.228	0.288	0.279
trkD	2.201	0.861	1.355	0.276	0.276
narK	1.921	1.069	0.224	0.253	0.274
frwB	1.64	0.974	0.223	0.382	0.258
proP	2.046	0.664	1.584	0.247	0.257
frvB	2.396	0.501	0.224	0.282	0.256
fieF	1.912	1.04	1.542	0.479	0.249
yigM	2.302	0.932	1.615	0.306	0.239
hsrA	2.055	0.749	0	0	0
WT	0.044	0.517	0	0	0
garP	2.126	0.435	0	0	0
rbsB	0.1	0.261	0	0	0
yfhD	1.432	0.232	0	0	0
tolQ	1.37	0.198	0	0	0
yijE	1.696	0.195	0	0	0
wzxE	1.826	0.194	0	0	0
caiT	1.806	0.192	0	0	0
ybhF	2.245	0.18	0	0	0
ugpC	2.133	0.178	0	0	0
atoS	0.076	0.175	0	0	0
guaB	1.327	0.172	0	0	0
sbp	1.975	0.168	0	0	0
dppC	1.856	0.162	0	0	0
ydhK	1.97	0.154	0	0	0
malX	1.994	0.142	0	0	0
agaV	1.903	0.141	0	0	0
ybgH	1.942	0.135	0	0	0
yicL	2.03	0.134	0	0	0
ycjP	2.213	0.13	0	0	0
barA	2.233	0.126	0	0	0
yicG	1.75	0.126	0	0	0
dppD	1.614	0.124	0	0	0
yjiO	2.124	0.123	0	0	0
gntT	0.13	0.123	0	0	0
WT	0.08	0.122	0	0	0
oppA	2.11	0.12	0	0	0
ynfA	1.857	0.119	0	0	0
alsC	1.735	0.118	0	0	0
gntP	0.102	0.118	0	0	0
ulaA	2.216	0.117	0	0	0
rarD	1.804	0.115	0	0	0
rbbA	1.775	0.115	0	0	0
mhpT	1.645	0.115	0	0	0
yeiJ	2.119	0.113	0	0	0
emrD	1.991	0.113	0	0	0
emrB	1.56	0.113	0	0	0
bglF	1.847	0.111	0	0	0
cmtB	1.624	0.111	0	0	0
potH	0.052	0.111	0	0	0
ybaL	2	0.11	0	0	0
feoB	2.226	0.109	0	0	0
ydjK	2.055	0.108	0	0	0
mdtI	1.97	0.108	0	0	0
yejE	1.95	0.108	0	0	0
pheP	1.726	0.108	0	0	0
hisP	1.698	0.108	0	0	0

yedA	1.648	0.108	0	0	0
manX	1.611	0.108	0	0	0
yehZ	1.979	0.107	0	0	0
yqcE	1.789	0.107	0	0	0
ydeE	1.738	0.107	0	0	0
yddA	1.692	0.107	0	0	0
ybbM	1.427	0.107	0	0	0
yhdW	1.9	0.106	0	0	0
yhaO	1.785	0.106	0	0	0
ydhP	1.527	0.106	0	0	0
yfiK	1.918	0.105	0	0	0
cusA	1.789	0.105	0	0	0
yqeG	1.677	0.105	0	0	0
fsr	2.232	0.104	0	0	0
yphF	1.994	0.104	0	0	0
gntU	1.945	0.104	0	0	0
atoE	1.764	0.103	0	0	0
tsgA	1.646	0.103	0	0	0
ytfQ	1.609	0.103	0	0	0
ynjD	1.591	0.103	0	0	0
yebQ	1.54	0.103	0	0	0
ygcS	2.077	0.102	0	0	0
rhtB	2.023	0.102	0	0	0
pstA	1.901	0.102	0	0	0
uhpT	1.886	0.102	0	0	0
oppC	1.8	0.102	0	0	0
agaC	1.778	0.102	0	0	0
ycjO	1.583	0.102	0	0	0
livK	1.488	0.102	0	0	0
ygiS	0.111	0.102	0	0	0
yjcQ	1.919	0.101	0	0	0
dppF	1.772	0.101	0	0	0
manZ	1.509	0.101	0	0	0
hisM	1.416	0.101	0	0	0
ybbP	1.977	0.1	0	0	0
sufC	1.905	0.1	0	0	0
araE	1.899	0.1	0	0	0
acrE	1.788	0.1	0	0	0
alx	1.756	0.1	0	0	0
sufD	1.656	0.1	0	0	0
yrbE	1.552	0.1	0	0	0
ydgG	1.489	0.1	0	0	0
yhdX	1.453	0.1	0	0	0
yjjK	1.416	0.1	0	0	0
mdtD	1.988	0.099	0	0	0
ymjB	1.897	0.099	0	0	0
uraA	1.868	0.099	0	0	0
actP	1.531	0.099	0	0	0
yfdC	1.41	0.099	0	0	0
ygdQ	0.042	0.099	0	0	0
mdtA	2.015	0.098	0	0	0
yhjE	1.895	0.098	0	0	0
ydjE	1.847	0.098	0	0	0
yihN	1.809	0.098	0	0	0
chbA	1.793	0.098	0	0	0
frwD	1.782	0.098	0	0	0
nikA	1.745	0.098	0	0	0
agaB	1.639	0.098	0	0	0
yaaJ	1.621	0.098	0	0	0
ytfF	1.586	0.098	0	0	0
ybbY	1.32	0.098	0	0	0
argT	2.102	0.097	0	0	0
kefC	2.024	0.097	0	0	0
yhiP	1.794	0.097	0	0	0
sugE	1.765	0.097	0	0	0
dsdX	1.762	0.097	0	0	0
malG	1.719	0.097	0	0	0
metI	1.594	0.097	0	0	0
manY	1.584	0.097	0	0	0
tdcC	2.105	0.096	0	0	0
panF	2.033	0.096	0	0	0
yeeO	1.95	0.096	0	0	0
ptsA	1.81	0.096	0	0	0
fepB	1.79	0.096	0	0	0
yebA	1.786	0.096	0	0	0
kefB	1.761	0.096	0	0	0
livF	1.734	0.096	0	0	0
treB	1.69	0.096	0	0	0
yfeH	1.683	0.096	0	0	0
rbsC	1.659	0.096	0	0	0
yiaO	1.561	0.096	0	0	0
yhdZ	1.539	0.096	0	0	0
ygfO	1.522	0.096	0	0	0
gudP	1.289	0.096	0	0	0
ygjI	1.278	0.096	0	0	0
yicO	2.054	0.095	0	0	0
yehX	2.029	0.095	0	0	0
yeeF	1.943	0.095	0	0	0
yajR	1.908	0.095	0	0	0
malE	1.869	0.095	0	0	0
xylH	1.847	0.095	0	0	0
sapA	1.844	0.095	0	0	0
yjeH	1.697	0.095	0	0	0

tehA	1.558	0.095	0	0	0
lysP	1.455	0.095	0	0	0
gltS	1.373	0.095	0	0	0
ybbA	1.311	0.095	0	0	0
eamA	2.33	0.094	0	0	0
ybjL	2.088	0.094	0	0	0
hisQ	1.97	0.094	0	0	0
ygbN	1.968	0.094	0	0	0
ytfT	1.954	0.094	0	0	0
narU	1.897	0.094	0	0	0
mdlA	1.89	0.094	0	0	0
cycA	1.756	0.094	0	0	0
yfgO	1.731	0.094	0	0	0
cmtA	1.726	0.094	0	0	0
pstC	1.724	0.094	0	0	0
srlA	1.709	0.094	0	0	0
modA	1.704	0.094	0	0	0
ydgR	1.649	0.094	0	0	0
gltL	1.641	0.094	0	0	0
ddpB	1.637	0.094	0	0	0
thiP	1.6	0.094	0	0	0
setC	1.553	0.094	0	0	0
mdtL	1.551	0.094	0	0	0
ddpF	1.519	0.094	0	0	0
ynjC	1.486	0.094	0	0	0
cadB	1.431	0.094	0	0	0
gltJ	1.342	0.094	0	0	0
atpD	0.059	0.094	0	0	0
potI	0.042	0.094	0	0	0
ygfU	2.192	0.093	0	0	0
ydiK	2.161	0.093	0	0	0
yhdY	2.116	0.093	0	0	0
ydcS	1.971	0.093	0	0	0
yhhS	1.962	0.093	0	0	0
yfbJ	1.891	0.093	0	0	0
ddpA	1.871	0.093	0	0	0
yhhT	1.865	0.093	0	0	0
trkH	1.797	0.093	0	0	0
ynfM	1.787	0.093	0	0	0
mscS	1.772	0.093	0	0	0
dcuA	1.759	0.093	0	0	0
glpF	1.581	0.093	0	0	0
ydcO	1.569	0.093	0	0	0
yeaN	1.561	0.093	0	0	0
dctA	1.546	0.093	0	0	0
ypdG	1.49	0.093	0	0	0
acrA	1.293	0.093	0	0	0
kdpD	1.208	0.093	0	0	0
yjbB	2.097	0.092	0	0	0
kdgT	2.072	0.092	0	0	0
focA	1.973	0.092	0	0	0
ydeA	1.961	0.092	0	0	0
potA	1.961	0.092	0	0	0
yiaN	1.944	0.092	0	0	0
artM	1.94	0.092	0	0	0
yjfF	1.921	0.092	0	0	0
mngA	1.892	0.092	0	0	0
tbpA	1.89	0.092	0	0	0
setA	1.885	0.092	0	0	0
ycjN	1.864	0.092	0	0	0
ydiN	1.831	0.092	0	0	0
exbD	1.814	0.092	0	0	0
macB	1.782	0.092	0	0	0
ychM	1.774	0.092	0	0	0
kch	1.752	0.092	0	0	0
sdaC	1.728	0.092	0	0	0
yehY	1.718	0.092	0	0	0
codB	1.681	0.092	0	0	0
ybhN	1.662	0.092	0	0	0
shiA	1.656	0.092	0	0	0
murP	1.628	0.092	0	0	0
dcuC	1.618	0.092	0	0	0
yliC	1.597	0.092	0	0	0
araF	1.472	0.092	0	0	0
glnP	1.472	0.092	0	0	0
cysU	1.426	0.092	0	0	0
yjeP	1.369	0.092	0	0	0
mdlB	2.218	0.091	0	0	0
ytfL	2.157	0.091	0	0	0
yjcD	2.076	0.091	0	0	0
ydgI	2.05	0.091	0	0	0
nupC	1.973	0.091	0	0	0
nlpA	1.927	0.091	0	0	0
ybiR	1.881	0.091	0	0	0
yidK	1.861	0.091	0	0	0
livM	1.789	0.091	0	0	0
ydcV	1.786	0.091	0	0	0
ydcZ	1.75	0.091	0	0	0
lsrB	1.746	0.091	0	0	0
potD	1.696	0.091	0	0	0
xylF	1.684	0.091	0	0	0
ego	1.661	0.091	0	0	0
ygiE	1.636	0.091	0	0	0

nirC	1.627	0.091	0	0	0
ddpC	1.626	0.091	0	0	0
yoaE	1.592	0.091	0	0	0
artJ	1.591	0.091	0	0	0
ydhJ	1.587	0.091	0	0	0
yhjX	1.585	0.091	0	0	0
gltP	1.55	0.091	0	0	0
chbB	1.513	0.091	0	0	0
citT	1.508	0.091	0	0	0
modC	1.409	0.091	0	0	0
atpI	2.25	0.09	0	0	0
exbB	2.214	0.09	0	0	0
gltK	2.093	0.09	0	0	0
glnH	2.092	0.09	0	0	0
yheS	2.074	0.09	0	0	0
metN	2.067	0.09	0	0	0
lldP	2.046	0.09	0	0	0
ssuC	2.022	0.09	0	0	0
galP	2.018	0.09	0	0	0
fruB	2.006	0.09	0	0	0
ybhR	1.93	0.09	0	0	0
yjdL	1.922	0.09	0	0	0
yhfK	1.916	0.09	0	0	0
tatB	1.892	0.09	0	0	0
dcuD	1.888	0.09	0	0	0
yaaU	1.887	0.09	0	0	0
chbC	1.885	0.09	0	0	0
yeaV	1.852	0.09	0	0	0
ybhI	1.85	0.09	0	0	0
kdpC	1.848	0.09	0	0	0
mglC	1.844	0.09	0	0	0
yjcE	1.829	0.09	0	0	0
yojI	1.819	0.09	0	0	0
yegT	1.768	0.09	0	0	0
dppB	1.722	0.09	0	0	0
araH	1.719	0.09	0	0	0
dppA	1.705	0.09	0	0	0
yadS	1.694	0.09	0	0	0
yliA	1.687	0.09	0	0	0
potE	1.665	0.09	0	0	0
potF	1.649	0.09	0	0	0
mtr	1.633	0.09	0	0	0
ydcU	1.616	0.09	0	0	0
aqpZ	1.612	0.09	0	0	0
yegH	1.588	0.09	0	0	0
yfcJ	1.575	0.09	0	0	0
ptsP	1.573	0.09	0	0	0
ugpA	1.569	0.09	0	0	0
cysW	1.553	0.09	0	0	0
xapB	1.522	0.09	0	0	0
puuP	1.486	0.09	0	0	0
ptsN	1.447	0.09	0	0	0
hisJ	1.435	0.09	0	0	0
rbsA	1.338	0.09	0	0	0
artQ	1.334	0.09	0	0	0
yohN	1.161	0.09	0	0	0
yhbE	0.049	0.09	0	0	0
yhhJ	2.115	0.089	0	0	0
ccmA	1.954	0.089	0	0	0
phnC	1.92	0.089	0	0	0
yejB	1.906	0.089	0	0	0
mdtK	1.892	0.089	0	0	0
aaeB	1.889	0.089	0	0	0
btuC	1.821	0.089	0	0	0
ybiO	1.797	0.089	0	0	0
srlB	1.762	0.089	0	0	0
yphD	1.725	0.089	0	0	0
yneE	1.72	0.089	0	0	0
yrbF	1.713	0.089	0	0	0
livG	1.683	0.089	0	0	0
fruA	1.674	0.089	0	0	0
nupG	1.668	0.089	0	0	0
agaD	1.655	0.089	0	0	0
zitB	1.64	0.089	0	0	0
ssuA	1.625	0.089	0	0	0
yohM	1.594	0.089	0	0	0
yhjV	1.583	0.089	0	0	0
emrE	1.574	0.089	0	0	0
yliD	1.515	0.089	0	0	0
pstB	1.508	0.089	0	0	0
mdtE	1.47	0.089	0	0	0
potG	1.461	0.089	0	0	0
nagE	1.456	0.089	0	0	0
btuD	1.447	0.089	0	0	0
atpB	1.447	0.089	0	0	0
tnaB	1.431	0.089	0	0	0
lacY	1.417	0.089	0	0	0
cvrA	1.417	0.089	0	0	0
nikB	1.402	0.089	0	0	0
frlA	1.38	0.089	0	0	0
phnL	1.338	0.089	0	0	0
idnT	1.335	0.089	0	0	0
pstS	1.322	0.089	0	0	0

kgtP	1.288	0.089	0	0	0
hofC	2.037	0.088	0	0	0
ugpE	1.971	0.088	0	0	0
ydiM	1.953	0.088	0	0	0
yiaM	1.95	0.088	0	0	0
yicM	1.93	0.088	0	0	0
malK	1.925	0.088	0	0	0
cydD	1.904	0.088	0	0	0
brnQ	1.868	0.088	0	0	0
yjeM	1.816	0.088	0	0	0
sstT	1.812	0.088	0	0	0
ypdD	1.803	0.088	0	0	0
yadH	1.774	0.088	0	0	0
proY	1.762	0.088	0	0	0
yejA	1.751	0.088	0	0	0
mdtF	1.75	0.088	0	0	0
cysP	1.749	0.088	0	0	0
ampG	1.745	0.088	0	0	0
ygfQ	1.709	0.088	0	0	0
yeiM	1.688	0.088	0	0	0
kdpA	1.686	0.088	0	0	0
mdtG	1.655	0.088	0	0	0
proW	1.647	0.088	0	0	0
livH	1.644	0.088	0	0	0
bcr	1.627	0.088	0	0	0
malF	1.619	0.088	0	0	0
fepD	1.588	0.088	0	0	0
gltI	1.578	0.088	0	0	0
yrbD	1.53	0.088	0	0	0
acrD	1.51	0.088	0	0	0
nikD	1.505	0.088	0	0	0
nikC	1.497	0.088	0	0	0
lsrC	1.477	0.088	0	0	0
nanT	1.438	0.088	0	0	0
uup	1.393	0.088	0	0	0
clcA	1.392	0.088	0	0	0
nikE	1.382	0.088	0	0	0
ybaT	1.356	0.088	0	0	0
yfbS	1.316	0.088	0	0	0
dhaH	1.31	0.088	0	0	0
corA	1.307	0.088	0	0	0
torT	1.3	0.088	0	0	0
ycdG	1.276	0.088	0	0	0
yeaS	1.248	0.088	0	0	0
srlE	1.141	0.088	0	0	0
tatC	0.996	0.088	0	0	0
modB	0.913	0.088	0	0	0
dgoT	0.174	0.088	0	0	0
ccmC	1.906	0.087	0	0	0
yfjD	1.894	0.087	0	0	0
hcaT	1.846	0.087	0	0	0
gabP	1.813	0.087	0	0	0
yihP	1.778	0.087	0	0	0
mscL	1.763	0.087	0	0	0
alsA	1.759	0.087	0	0	0
kdpB	1.751	0.087	0	0	0
fepG	1.75	0.087	0	0	0
phnD	1.698	0.087	0	0	0
metQ	1.694	0.087	0	0	0
thiQ	1.682	0.087	0	0	0
ybiT	1.65	0.087	0	0	0
exuT	1.643	0.087	0	0	0
ansP	1.609	0.087	0	0	0
acrF	1.597	0.087	0	0	0
frvA	1.578	0.087	0	0	0
ybbW	1.57	0.087	0	0	0
yrbG	1.566	0.087	0	0	0
mglA	1.552	0.087	0	0	0
ulaC	1.547	0.087	0	0	0
yphE	1.513	0.087	0	0	0
glnQ	1.509	0.087	0	0	0
yehW	1.491	0.087	0	0	0
fepC	1.463	0.087	0	0	0
glpT	1.458	0.087	0	0	0
xylE	1.419	0.087	0	0	0
yahN	1.394	0.087	0	0	0
mglB	1.308	0.087	0	0	0
proV	1.245	0.087	0	0	0
yccS	0.834	0.087	0	0	0
phoR	0.066	0.087	0	0	0
amtB	0.048	0.087	0	0	0
ygeD	2.234	0.086	0	0	0
mdtH	2.041	0.086	0	0	0
mdtC	2.027	0.086	0	0	0
fucP	1.909	0.086	0	0	0
livJ	1.829	0.086	0	0	0
emrY	1.814	0.086	0	0	0
uidB	1.791	0.086	0	0	0
ygjE	1.749	0.086	0	0	0
yejF	1.744	0.086	0	0	0
yggT	1.743	0.086	0	0	0
yliB	1.695	0.086	0	0	0
yfaV	1.692	0.086	0	0	0

ygaZ	1.69	0.086	0	0	0
ascF	1.679	0.086	0	0	0
ypdH	1.635	0.086	0	0	0
proX	1.629	0.086	0	0	0
xylG	1.612	0.086	0	0	0
dcuB	1.608	0.086	0	0	0
ybbL	1.581	0.086	0	0	0
setB	1.553	0.086	0	0	0
aaeA	1.455	0.086	0	0	0
cysA	1.451	0.086	0	0	0
frwC	1.413	0.086	0	0	0
artP	1.319	0.086	0	0	0
sapF	1.309	0.086	0	0	0
yjjP	1.02	0.086	0	0	0
WT	0.06	0.086	0	0	0
melB	0.054	0.086	0	0	0
cmr	0.052	0.086	0	0	0
ssuB	0.049	0.086	0	0	0
lsrD	1.908	0.085	0	0	0
araJ	1.824	0.085	0	0	0
alsB	1.8	0.085	0	0	0
acrB	1.738	0.085	0	0	0
mdtB	1.702	0.085	0	0	0
argO	1.558	0.085	0	0	0
glvB	1.489	0.085	0	0	0
rhaT	1.478	0.085	0	0	0
tyrP	1.422	0.085	0	0	0
kefA	1.418	0.085	0	0	0
oppF	1.375	0.085	0	0	0
potB	1.313	0.085	0	0	0
nhaB	1.288	0.085	0	0	0
arsB	1.137	0.085	0	0	0
tolC	0.89	0.085	0	0	0
ybjJ	0.748	0.085	0	0	0
atpH	0.668	0.085	0	0	0
yddG	0.068	0.085	0	0	0
atpA	0.047	0.085	0	0	0
yfdV	1.862	0.084	0	0	0
zntA	1.843	0.084	0	0	0
yjcR	1.824	0.084	0	0	0
modF	1.769	0.084	0	0	0
adiC	1.639	0.084	0	0	0
ccmB	1.611	0.084	0	0	0
cynX	1.54	0.084	0	0	0
copA	1.415	0.084	0	0	0
sapD	1.365	0.084	0	0	0
yihO	1.184	0.084	0	0	0
atpC	0.512	0.084	0	0	0
ugpB	0.085	0.084	0	0	0
rhtA	0.07	0.084	0	0	0
ddpD	0.061	0.084	0	0	0
ompF	0.048	0.084	0	0	0
yfbW	0.047	0.084	0	0	0
tsr	0.046	0.084	0	0	0
yadG	1.559	0.083	0	0	0
znuB	1.526	0.083	0	0	0
ptsG	1.342	0.083	0	0	0
yrbC	1.163	0.083	0	0	0
sufB	0.849	0.083	0	0	0
atpG	0.712	0.083	0	0	0
tolR	0.565	0.083	0	0	0
WT	0.278	0.083	0	0	0
ydjN	0.07	0.083	0	0	0
RtolC	0.045	0.083	0	0	0
yhbG	0.996	0.082	0	0	0
ygaH	0.12	0.082	0	0	0
mdtJ	0.086	0.082	0	0	0
WT	0.082	0.082	0	0	0
ytfR	0.07	0.082	0	0	0
yeeA	0.052	0.082	0	0	0
yifK	0.052	0.082	0	0	0
yicJ	0.05	0.082	0	0	0
WT	0.046	0.082	0	0	0
WT	0.046	0.082	0	0	0
ompC	0.044	0.082	0	0	0
potC	0.59	0.081	0	0	0
WT	0.044	0.081	0	0	0
dinF	0.043	0.081	0	0	0
ompA	0.041	0.081	0	0	0
WT	0.044	0.08	0	0	0
WT	0.04	0.08	0	0	0

Table S2: Growth data for the *E. coli* ASKA strains

Colistin	IPTG	Strain	Repeat	Time	OD600
0	0	ptsI	2	0	0.199
0	0	clcB	2	0	0.206
0	0	ycaM	2	0	0.213
0	0	yadI	2	0	0.222
0	0	AG1	2	0	0.228
0	0	ptsI	2	0.25	0.202
0	0	clcB	2	0.25	0.207
0	0	ycaM	2	0.25	0.206
0	0	yadI	2	0.25	0.213
0	0	AG1	2	0.25	0.222
0	0	ptsI	2	0.5	0.199
0	0	clcB	2	0.5	0.204
0	0	ycaM	2	0.5	0.205
0	0	yadI	2	0.5	0.214
0	0	AG1	2	0.5	0.219
0	0	ptsI	2	0.75	0.2
0	0	clcB	2	0.75	0.205
0	0	ycaM	2	0.75	0.206
0	0	yadI	2	0.75	0.215
0	0	AG1	2	0.75	0.22
0	0	ptsI	2	1	0.209
0	0	clcB	2	1	0.21
0	0	ycaM	2	1	0.212
0	0	yadI	2	1	0.222
0	0	AG1	2	1	0.225
0	0	ptsI	2	1.25	0.212
0	0	clcB	2	1.25	0.21
0	0	ycaM	2	1.25	0.214
0	0	yadI	2	1.25	0.231
0	0	AG1	2	1.25	0.23
0	0	ptsI	2	1.5	0.21
0	0	clcB	2	1.5	0.213
0	0	ycaM	2	1.5	0.219
0	0	yadI	2	1.5	0.234
0	0	AG1	2	1.5	0.238
0	0	ptsI	2	1.75	0.214
0	0	clcB	2	1.75	0.216
0	0	ycaM	2	1.75	0.222
0	0	yadI	2	1.75	0.242
0	0	AG1	2	1.75	0.246
0	0	ptsI	2	2	0.218
0	0	clcB	2	2	0.219
0	0	ycaM	2	2	0.228
0	0	yadI	2	2	0.252
0	0	AG1	2	2	0.258
0	0	ptsI	2	2.25	0.224
0	0	clcB	2	2.25	0.224
0	0	ycaM	2	2.25	0.235
0	0	yadI	2	2.25	0.262
0	0	AG1	2	2.25	0.274
0	0	ptsI	2	2.5	0.24

0	0	clcB	2	2.5	0.231
0	0	ycaM	2	2.5	0.241
0	0	yadI	2	2.5	0.272
0	0	AG1	2	2.5	0.287
0	0	ptsI	2	2.75	0.243
0	0	clcB	2	2.75	0.234
0	0	ycaM	2	2.75	0.249
0	0	yadI	2	2.75	0.29
0	0	AG1	2	2.75	0.303
0	0	ptsI	2	3	0.248
0	0	clcB	2	3	0.238
0	0	ycaM	2	3	0.256
0	0	yadI	2	3	0.3
0	0	AG1	2	3	0.308
0	0	ptsI	2	3.25	0.26
0	0	clcB	2	3.25	0.246
0	0	ycaM	2	3.25	0.267
0	0	yadI	2	3.25	0.318
0	0	AG1	2	3.25	0.324
0	0	ptsI	2	3.5	0.258
0	0	clcB	2	3.5	0.254
0	0	ycaM	2	3.5	0.275
0	0	yadI	2	3.5	0.334
0	0	AG1	2	3.5	0.33
0	0	ptsI	2	3.75	0.271
0	0	clcB	2	3.75	0.26
0	0	ycaM	2	3.75	0.29
0	0	yadI	2	3.75	0.353
0	0	AG1	2	3.75	0.341
0	0	ptsI	2	4	0.286
0	0	clcB	2	4	0.267
0	0	ycaM	2	4	0.298
0	0	yadI	2	4	0.37
0	0	AG1	2	4	0.353
0	0	ptsI	2	4.38333	0.291
0	0	clcB	2	4.38333	0.278
0	0	ycaM	2	4.38333	0.317
0	0	yadI	2	4.38333	0.405
0	0	AG1	2	4.38333	0.378
0	0	ptsI	2	4.63333	0.286
0	0	clcB	2	4.63333	0.27
0	0	ycaM	2	4.63333	0.31
0	0	yadI	2	4.63333	0.411
0	0	AG1	2	4.63333	0.368
0	0	ptsI	2	4.88333	0.295
0	0	clcB	2	4.88333	0.275
0	0	ycaM	2	4.88333	0.324
0	0	yadI	2	4.88333	0.43
0	0	AG1	2	4.88333	0.382
0	0	ptsI	2	5.13333	0.36
0	0	clcB	2	5.13333	0.281
0	0	ycaM	2	5.13333	0.333
0	0	yadI	2	5.13333	0.445

0	0	AG1	2	5.13333	0.39
0	0	ptsI	2	5.38333	0.308
0	0	clcB	2	5.38333	0.289
0	0	ycaM	2	5.38333	0.342
0	0	yadI	2	5.38333	0.458
0	0	AG1	2	5.38333	0.401
0	0	ptsI	2	5.63333	0.318
0	0	clcB	2	5.63333	0.302
0	0	ycaM	2	5.63333	0.354
0	0	yadI	2	5.63333	0.473
0	0	AG1	2	5.63333	0.412
0	0	ptsI	2	5.88333	0.324
0	0	clcB	2	5.88333	0.307
0	0	ycaM	2	5.88333	0.363
0	0	yadI	2	5.88333	0.484
0	0	AG1	2	5.88333	0.422
0	0	ptsI	2	6.13333	0.333
0	0	clcB	2	6.13333	0.311
0	0	ycaM	2	6.13333	0.373
0	0	yadI	2	6.13333	0.496
0	0	AG1	2	6.13333	0.435
0	0	ptsI	2	6.38333	0.343
0	0	clcB	2	6.38333	0.321
0	0	ycaM	2	6.38333	0.385
0	0	yadI	2	6.38333	0.51
0	0	AG1	2	6.38333	0.446
0	0	ptsI	2	6.63333	0.347
0	0	clcB	2	6.63333	0.327
0	0	ycaM	2	6.63333	0.395
0	0	yadI	2	6.63333	0.527
0	0	AG1	2	6.63333	0.458
0	0	ptsI	2	6.88333	0.359
0	0	clcB	2	6.88333	0.338
0	0	ycaM	2	6.88333	0.414
0	0	yadI	2	6.88333	0.55
0	0	AG1	2	6.88333	0.474
0	0	ptsI	2	7.13333	0.363
0	0	clcB	2	7.13333	0.338
0	0	ycaM	2	7.13333	0.416
0	0	yadI	2	7.13333	0.544
0	0	AG1	2	7.13333	0.476
0	0	ptsI	2	7.38333	0.373
0	0	clcB	2	7.38333	0.35
0	0	ycaM	2	7.38333	0.427
0	0	yadI	2	7.38333	0.558
0	0	AG1	2	7.38333	0.489
0	0	ptsI	2	7.63333	0.382
0	0	clcB	2	7.63333	0.361
0	0	ycaM	2	7.63333	0.438
0	0	yadI	2	7.63333	0.576
0	0	AG1	2	7.63333	0.505
0	0	ptsI	2	7.88333	0.404
0	0	clcB	2	7.88333	0.371

0	0	ycaM	2	7.88333	0.446
0	0	yadI	2	7.88333	0.59
0	0	AG1	2	7.88333	0.515
0	0	ptsI	2	8.13333	0.401
0	0	clcB	2	8.13333	0.372
0	0	ycaM	2	8.13333	0.456
0	0	yadI	2	8.13333	0.598
0	0	AG1	2	8.13333	0.526
0	0	ptsI	2	8.38333	0.408
0	0	clcB	2	8.38333	0.384
0	0	ycaM	2	8.38333	0.46
0	0	yadI	2	8.38333	0.608
0	0	AG1	2	8.38333	0.54
0	0	ptsI	2	8.91667	0.423
0	0	clcB	2	8.91667	0.387
0	0	ycaM	2	8.91667	0.489
0	0	yadI	2	8.91667	0.632
0	0	AG1	2	8.91667	0.361
0	0	ptsI	2	9.16667	0.429
0	0	clcB	2	9.16667	0.394
0	0	ycaM	2	9.16667	0.483
0	0	yadI	2	9.16667	0.613
0	0	AG1	2	9.16667	0.356
0	0	ptsI	2	9.41667	0.443
0	0	clcB	2	9.41667	0.412
0	0	ycaM	2	9.41667	0.489
0	0	yadI	2	9.41667	0.624
0	0	AG1	2	9.41667	0.365
0	0	ptsI	2	9.66667	0.454
0	0	clcB	2	9.66667	0.419
0	0	ycaM	2	9.66667	0.498
0	0	yadI	2	9.66667	0.63
0	0	AG1	2	9.66667	0.371
0	0	ptsI	2	9.91667	0.467
0	0	clcB	2	9.91667	0.426
0	0	ycaM	2	9.91667	0.508
0	0	yadI	2	9.91667	0.642
0	0	AG1	2	9.91667	0.379
0	0	ptsI	2	10.1667	0.479
0	0	clcB	2	10.1667	0.434
0	0	ycaM	2	10.1667	0.526
0	0	yadI	2	10.1667	0.658
0	0	AG1	2	10.1667	0.386
0	0	ptsI	2	10.4167	0.494
0	0	clcB	2	10.4167	0.445
0	0	ycaM	2	10.4167	0.537
0	0	yadI	2	10.4167	0.67
0	0	AG1	2	10.4167	0.393
0	0	ptsI	2	10.6667	0.494
0	0	clcB	2	10.6667	0.456
0	0	ycaM	2	10.6667	0.548
0	0	yadI	2	10.6667	0.688
0	0	AG1	2	10.6667	0.399

0	0	ptsI	2	10.9167	0.505
0	0	clcB	2	10.9167	0.459
0	0	ycaM	2	10.9167	0.558
0	0	yadI	2	10.9167	0.695
0	0	AG1	2	10.9167	0.407
0	0	ptsI	2	11.1667	0.541
0	0	clcB	2	11.1667	0.474
0	0	ycaM	2	11.1667	0.573
0	0	yadI	2	11.1667	0.709
0	0	AG1	2	11.1667	0.42
0	0	ptsI	2	11.4167	0.517
0	0	clcB	2	11.4167	0.476
0	0	ycaM	2	11.4167	0.587
0	0	yadI	2	11.4167	0.717
0	0	AG1	2	11.4167	0.417
0	0	ptsI	2	11.6667	0.565
0	0	clcB	2	11.6667	0.486
0	0	ycaM	2	11.6667	0.598
0	0	yadI	2	11.6667	0.731
0	0	AG1	2	11.6667	0.432
0	0	ptsI	2	11.9167	0.525
0	0	clcB	2	11.9167	0.496
0	0	ycaM	2	11.9167	0.62
0	0	yadI	2	11.9167	0.741
0	0	AG1	2	11.9167	0.444
0	0	ptsI	2	12.1667	0.554
0	0	clcB	2	12.1667	0.511
0	0	ycaM	2	12.1667	0.642
0	0	yadI	2	12.1667	0.756
0	0	AG1	2	12.1667	0.452
0	0	ptsI	2	12.4167	0.576
0	0	clcB	2	12.4167	0.523
0	0	ycaM	2	12.4167	0.654
0	0	yadI	2	12.4167	0.762
0	0	AG1	2	12.4167	0.471
0	0	ptsI	2	12.6667	0.554
0	0	clcB	2	12.6667	0.521
0	0	ycaM	2	12.6667	0.652
0	0	yadI	2	12.6667	0.772
0	0	AG1	2	12.6667	0.464
0	0	ptsI	2	12.9167	0.573
0	0	clcB	2	12.9167	0.528
0	0	ycaM	2	12.9167	0.672
0	0	yadI	2	12.9167	0.791
0	0	AG1	2	12.9167	0.476
0	0	ptsI	2	13.1667	0.601
0	0	clcB	2	13.1667	0.534
0	0	ycaM	2	13.1667	0.687
0	0	yadI	2	13.1667	0.802
0	0	AG1	2	13.1667	0.502
0	0	ptsI	2	13.4167	0.583
0	0	clcB	2	13.4167	0.527
0	0	ycaM	2	13.4167	0.672

0	0	yadI	2	13.4167	0.803
0	0	AG1	2	13.4167	0.504
0	0	ptsI	2	13.6667	0.618
0	0	clcB	2	13.6667	0.538
0	0	ycaM	2	13.6667	0.688
0	0	yadI	2	13.6667	0.818
0	0	AG1	2	13.6667	0.56
0	0	ptsI	2	13.9167	0.633
0	0	clcB	2	13.9167	0.546
0	0	ycaM	2	13.9167	0.675
0	0	yadI	2	13.9167	0.823
0	0	AG1	2	13.9167	0.553
0	0	ptsI	2	14.1667	0.642
0	0	clcB	2	14.1667	0.542
0	0	ycaM	2	14.1667	0.68
0	0	yadI	2	14.1667	0.836
0	0	AG1	2	14.1667	0.563
0	0	ptsI	2	14.4167	0.672
0	0	clcB	2	14.4167	0.538
0	0	ycaM	2	14.4167	0.674
0	0	yadI	2	14.4167	0.836
0	0	AG1	2	14.4167	0.564
0	0	ptsI	2	14.6667	0.686
0	0	clcB	2	14.6667	0.549
0	0	ycaM	2	14.6667	0.68
0	0	yadI	2	14.6667	0.841
0	0	AG1	2	14.6667	0.582
0	0	ptsI	2	14.9167	0.701
0	0	clcB	2	14.9167	0.56
0	0	ycaM	2	14.9167	0.662
0	0	yadI	2	14.9167	0.844
0	0	AG1	2	14.9167	0.587
0	0	ptsI	2	15.1667	0.726
0	0	clcB	2	15.1667	0.543
0	0	ycaM	2	15.1667	0.661
0	0	yadI	2	15.1667	0.862
0	0	AG1	2	15.1667	0.628
0	0	ptsI	2	15.4167	0.759
0	0	clcB	2	15.4167	0.561
0	0	ycaM	2	15.4167	0.671
0	0	yadI	2	15.4167	0.869
0	0	AG1	2	15.4167	0.656
0	0	ptsI	2	15.6667	0.742
0	0	clcB	2	15.6667	0.564
0	0	ycaM	2	15.6667	0.662
0	0	yadI	2	15.6667	0.868
0	0	AG1	2	15.6667	0.672
0	0	ptsI	2	15.9167	0.728
0	0	clcB	2	15.9167	0.571
0	0	ycaM	2	15.9167	0.671
0	0	yadI	2	15.9167	0.878
0	0	AG1	2	15.9167	0.67
0	0	ptsI	2	16.1667	0.769

0	0	clcB	2	16.1667	0.561
0	0	ycaM	2	16.1667	0.689
0	0	yadI	2	16.1667	0.884
0	0	AG1	2	16.1667	0.704
0	0	ptsI	2	16.4167	0.754
0	0	clcB	2	16.4167	0.559
0	0	ycaM	2	16.4167	0.697
0	0	yadI	2	16.4167	0.884
0	0	AG1	2	16.4167	0.706
0	0	ptsI	2	16.6667	0.763
0	0	clcB	2	16.6667	0.587
0	0	ycaM	2	16.6667	0.711
0	0	yadI	2	16.6667	0.889
0	0	AG1	2	16.6667	0.71
0	0	ptsI	2	16.9167	0.77
0	0	clcB	2	16.9167	0.564
0	0	ycaM	2	16.9167	0.702
0	0	yadI	2	16.9167	0.891
0	0	AG1	2	16.9167	0.729
0	0	ptsI	2	17.1667	0.799
0	0	clcB	2	17.1667	0.56
0	0	ycaM	2	17.1667	0.72
0	0	yadI	2	17.1667	0.909
0	0	AG1	2	17.1667	0.746
0	0	ptsI	2	17.4167	0.786
0	0	clcB	2	17.4167	0.608
0	0	ycaM	2	17.4167	0.727
0	0	yadI	2	17.4167	0.916
0	0	AG1	2	17.4167	0.761
0	0	ptsI	2	17.6667	0.81
0	0	clcB	2	17.6667	0.588
0	0	ycaM	2	17.6667	0.715
0	0	yadI	2	17.6667	0.916
0	0	AG1	2	17.6667	0.758
0	0	ptsI	2	17.9167	0.809
0	0	clcB	2	17.9167	0.575
0	0	ycaM	2	17.9167	0.717
0	0	yadI	2	17.9167	0.914
0	0	AG1	2	17.9167	0.771
0	0	ptsI	2	18.1667	0.835
0	0	clcB	2	18.1667	0.595
0	0	ycaM	2	18.1667	0.724
0	0	yadI	2	18.1667	0.926
0	0	AG1	2	18.1667	0.768
0	0	ptsI	2	18.4167	0.818
0	0	clcB	2	18.4167	0.586
0	0	ycaM	2	18.4167	0.738
0	0	yadI	2	18.4167	0.945
0	0	AG1	2	18.4167	0.776
0	0	ptsI	2	18.6667	0.822
0	0	clcB	2	18.6667	0.582
0	0	ycaM	2	18.6667	0.742
0	0	yadI	2	18.6667	0.938

0	0	AG1	2	18.6667	0.775
0	0	ptsI	2	18.9167	0.837
0	0	clcB	2	18.9167	0.612
0	0	ycaM	2	18.9167	0.748
0	0	yadI	2	18.9167	0.93
0	0	AG1	2	18.9167	0.781
0	0	ptsI	2	19.1667	0.821
0	0	clcB	2	19.1667	0.6
0	0	ycaM	2	19.1667	0.745
0	0	yadI	2	19.1667	0.939
0	0	AG1	2	19.1667	0.778
0	0	ptsI	2	19.4167	0.834
0	0	clcB	2	19.4167	0.601
0	0	ycaM	2	19.4167	0.752
0	0	yadI	2	19.4167	0.948
0	0	AG1	2	19.4167	0.788
0	0	ptsI	2	19.6667	0.882
0	0	clcB	2	19.6667	0.598
0	0	ycaM	2	19.6667	0.753
0	0	yadI	2	19.6667	0.943
0	0	AG1	2	19.6667	0.792
0	0	ptsI	2	19.9167	0.862
0	0	clcB	2	19.9167	0.621
0	0	ycaM	2	19.9167	0.77
0	0	yadI	2	19.9167	0.958
0	0	AG1	2	19.9167	0.815
0	0	ptsI	2	20.1667	0.859
0	0	clcB	2	20.1667	0.598
0	0	ycaM	2	20.1667	0.763
0	0	yadI	2	20.1667	0.954
0	0	AG1	2	20.1667	0.806
0	0	ptsI	2	20.4167	0.844
0	0	clcB	2	20.4167	0.602
0	0	ycaM	2	20.4167	0.757
0	0	yadI	2	20.4167	0.953
0	0	AG1	2	20.4167	0.807
0	0	ptsI	2	20.6667	0.854
0	0	clcB	2	20.6667	0.61
0	0	ycaM	2	20.6667	0.768
0	0	yadI	2	20.6667	0.962
0	0	AG1	2	20.6667	0.811
0	0	ptsI	2	20.9167	0.907
0	0	clcB	2	20.9167	0.604
0	0	ycaM	2	20.9167	0.766
0	0	yadI	2	20.9167	0.96
0	0	AG1	2	20.9167	0.815
0	0	ptsI	2	21.1667	0.849
0	0	clcB	2	21.1667	0.598
0	0	ycaM	2	21.1667	0.772
0	0	yadI	2	21.1667	0.959
0	0	AG1	2	21.1667	0.819
0	0	ptsI	2	21.4167	0.856
0	0	clcB	2	21.4167	0.603

0	0	ycaM	2	21.4167	0.78
0	0	yadI	2	21.4167	0.965
0	0	AG1	2	21.4167	0.834
0	0	ptsI	2	21.6667	0.876
0	0	clcB	2	21.6667	0.614
0	0	ycaM	2	21.6667	0.791
0	0	yadI	2	21.6667	0.979
0	0	AG1	2	21.6667	0.838
0	0	ptsI	2	21.9167	0.878
0	0	clcB	2	21.9167	0.622
0	0	ycaM	2	21.9167	0.789
0	0	yadI	2	21.9167	0.98
0	0	AG1	2	21.9167	0.844
0	0	ptsI	2	22.1667	0.884
0	0	clcB	2	22.1667	0.622
0	0	ycaM	2	22.1667	0.793
0	0	yadI	2	22.1667	0.984
0	0	AG1	2	22.1667	0.845
0	0	ptsI	2	22.4167	0.884
0	0	clcB	2	22.4167	0.613
0	0	ycaM	2	22.4167	0.793
0	0	yadI	2	22.4167	0.981
0	0	AG1	2	22.4167	0.852
0	0	ptsI	2	22.6667	0.885
0	0	clcB	2	22.6667	0.626
0	0	ycaM	2	22.6667	0.795
0	0	yadI	2	22.6667	0.979
0	0	AG1	2	22.6667	0.851
0	0	ptsI	2	22.9167	0.9
0	0	clcB	2	22.9167	0.624
0	0	ycaM	2	22.9167	0.807
0	0	yadI	2	22.9167	0.984
0	0	AG1	2	22.9167	0.865
0	0	ptsI	2	23.1667	0.895
0	0	clcB	2	23.1667	0.625
0	0	ycaM	2	23.1667	0.797
0	0	yadI	2	23.1667	0.971
0	0	AG1	2	23.1667	0.844
0	0	ptsI	2	23.4167	0.906
0	0	clcB	2	23.4167	0.632
0	0	ycaM	2	23.4167	0.804
0	0	yadI	2	23.4167	0.988
0	0	AG1	2	23.4167	0.855
0	0	ptsI	2	23.6667	0.912
0	0	clcB	2	23.6667	0.632
0	0	ycaM	2	23.6667	0.806
0	0	yadI	2	23.6667	0.989
0	0	AG1	2	23.6667	0.859
0	0	ptsI	2	23.9167	0.955
0	0	clcB	2	23.9167	0.638
0	0	ycaM	2	23.9167	0.812
0	0	yadI	2	23.9167	0.991
0	0	AG1	2	23.9167	0.863

0	0	ptsI	2	24.1667	0.95
0	0	clcB	2	24.1667	0.635
0	0	ycaM	2	24.1667	0.812
0	0	yadI	2	24.1667	0.991
0	0	AG1	2	24.1667	0.864
0	0	ptsI	2	24.4167	0.929
0	0	clcB	2	24.4167	0.639
0	0	ycaM	2	24.4167	0.82
0	0	yadI	2	24.4167	0.996
0	0	AG1	2	24.4167	0.869
0	0	ptsI	2	24.6667	0.915
0	0	clcB	2	24.6667	0.644
0	0	ycaM	2	24.6667	0.822
0	0	yadI	2	24.6667	0.991
0	0	AG1	2	24.6667	0.875
0	0	ptsI	2	24.9167	0.964
0	0	clcB	2	24.9167	0.646
0	0	ycaM	2	24.9167	0.82
0	0	yadI	2	24.9167	0.992
0	0	AG1	2	24.9167	0.88
0	0	ptsI	2	25.1667	0.936
0	0	clcB	2	25.1667	0.646
0	0	ycaM	2	25.1667	0.825
0	0	yadI	2	25.1667	0.999
0	0	AG1	2	25.1667	0.884
0	0	ptsI	2	25.4167	0.943
0	0	clcB	2	25.4167	0.654
0	0	ycaM	2	25.4167	0.832
0	0	yadI	2	25.4167	0.989
0	0	AG1	2	25.4167	0.898
0	0	ptsI	2	25.6667	0.953
0	0	clcB	2	25.6667	0.651
0	0	ycaM	2	25.6667	0.831
0	0	yadI	2	25.6667	0.989
0	0	AG1	2	25.6667	0.897
0	0	ptsI	2	25.9167	0.957
0	0	clcB	2	25.9167	0.648
0	0	ycaM	2	25.9167	0.833
0	0	yadI	2	25.9167	1.008
0	0	AG1	2	25.9167	0.904
0	0	ptsI	2	26.1667	0.957
0	0	clcB	2	26.1667	0.65
0	0	ycaM	2	26.1667	0.835
0	0	yadI	2	26.1667	0.989
0	0	AG1	2	26.1667	0.91
0	0	ptsI	2	26.4167	0.964
0	0	clcB	2	26.4167	0.661
0	0	ycaM	2	26.4167	0.844
0	0	yadI	2	26.4167	0.998
0	0	AG1	2	26.4167	0.925
0	0	ptsI	3	0	0.155
0	0	clcB	3	0	0.162
0	0	ycaM	3	0	0.151

0	0	yadI	3	0	0.165
0	0	AG1	3	0	0.165
0	0	ptsI	3	0.25	0.151
0	0	clcB	3	0.25	0.162
0	0	ycaM	3	0.25	0.148
0	0	yadI	3	0.25	0.163
0	0	AG1	3	0.25	0.165
0	0	ptsI	3	0.5	0.15
0	0	clcB	3	0.5	0.162
0	0	ycaM	3	0.5	0.149
0	0	yadI	3	0.5	0.162
0	0	AG1	3	0.5	0.166
0	0	ptsI	3	0.75	0.151
0	0	clcB	3	0.75	0.16
0	0	ycaM	3	0.75	0.149
0	0	yadI	3	0.75	0.179
0	0	AG1	3	0.75	0.166
0	0	ptsI	3	1	0.152
0	0	clcB	3	1	0.162
0	0	ycaM	3	1	0.149
0	0	yadI	3	1	0.163
0	0	AG1	3	1	0.168
0	0	ptsI	3	1.25	0.152
0	0	clcB	3	1.25	0.162
0	0	ycaM	3	1.25	0.149
0	0	yadI	3	1.25	0.164
0	0	AG1	3	1.25	0.169
0	0	ptsI	3	1.5	0.154
0	0	clcB	3	1.5	0.163
0	0	ycaM	3	1.5	0.151
0	0	yadI	3	1.5	0.168
0	0	AG1	3	1.5	0.172
0	0	ptsI	3	1.75	0.155
0	0	clcB	3	1.75	0.164
0	0	ycaM	3	1.75	0.152
0	0	yadI	3	1.75	0.17
0	0	AG1	3	1.75	0.175
0	0	ptsI	3	2	0.156
0	0	clcB	3	2	0.166
0	0	ycaM	3	2	0.154
0	0	yadI	3	2	0.174
0	0	AG1	3	2	0.178
0	0	ptsI	3	2.25	0.159
0	0	clcB	3	2.25	0.168
0	0	ycaM	3	2.25	0.156
0	0	yadI	3	2.25	0.178
0	0	AG1	3	2.25	0.18
0	0	ptsI	3	2.5	0.16
0	0	clcB	3	2.5	0.168
0	0	ycaM	3	2.5	0.159
0	0	yadI	3	2.5	0.182
0	0	AG1	3	2.5	0.183
0	0	ptsI	3	2.75	0.164

0	0	clcB	3	2.75	0.17
0	0	ycaM	3	2.75	0.162
0	0	yadI	3	2.75	0.187
0	0	AG1	3	2.75	0.188
0	0	ptsI	3	3	0.166
0	0	clcB	3	3	0.172
0	0	ycaM	3	3	0.164
0	0	yadI	3	3	0.192
0	0	AG1	3	3	0.19
0	0	ptsI	3	3.25	0.169
0	0	clcB	3	3.25	0.173
0	0	ycaM	3	3.25	0.165
0	0	yadI	3	3.25	0.197
0	0	AG1	3	3.25	0.194
0	0	ptsI	3	3.5	0.173
0	0	clcB	3	3.5	0.178
0	0	ycaM	3	3.5	0.169
0	0	yadI	3	3.5	0.205
0	0	AG1	3	3.5	0.199
0	0	ptsI	3	3.75	0.177
0	0	clcB	3	3.75	0.18
0	0	ycaM	3	3.75	0.173
0	0	yadI	3	3.75	0.212
0	0	AG1	3	3.75	0.204
0	0	ptsI	3	4	0.181
0	0	clcB	3	4	0.183
0	0	ycaM	3	4	0.176
0	0	yadI	3	4	0.221
0	0	AG1	3	4	0.213
0	0	ptsI	3	4.41667	0.183
0	0	clcB	3	4.41667	0.196
0	0	ycaM	3	4.41667	0.191
0	0	yadI	3	4.41667	0.232
0	0	AG1	3	4.41667	0.218
0	0	ptsI	3	4.66667	0.182
0	0	clcB	3	4.66667	0.187
0	0	ycaM	3	4.66667	0.189
0	0	yadI	3	4.66667	0.225
0	0	AG1	3	4.66667	0.209
0	0	ptsI	3	4.91667	0.187
0	0	clcB	3	4.91667	0.192
0	0	ycaM	3	4.91667	0.19
0	0	yadI	3	4.91667	0.234
0	0	AG1	3	4.91667	0.213
0	0	ptsI	3	5.16667	0.191
0	0	clcB	3	5.16667	0.196
0	0	ycaM	3	5.16667	0.195
0	0	yadI	3	5.16667	0.246
0	0	AG1	3	5.16667	0.218
0	0	ptsI	3	5.41667	0.196
0	0	clcB	3	5.41667	0.202
0	0	ycaM	3	5.41667	0.201
0	0	yadI	3	5.41667	0.262

0	0	AG1	3	5.41667	0.227
0	0	ptsI	3	5.66667	0.202
0	0	clcB	3	5.66667	0.206
0	0	ycaM	3	5.66667	0.209
0	0	yadI	3	5.66667	0.283
0	0	AG1	3	5.66667	0.236
0	0	ptsI	3	5.91667	0.209
0	0	clcB	3	5.91667	0.212
0	0	ycaM	3	5.91667	0.218
0	0	yadI	3	5.91667	0.309
0	0	AG1	3	5.91667	0.252
0	0	ptsI	3	6.16667	0.217
0	0	clcB	3	6.16667	0.22
0	0	ycaM	3	6.16667	0.229
0	0	yadI	3	6.16667	0.33
0	0	AG1	3	6.16667	0.264
0	0	ptsI	3	6.41667	0.225
0	0	clcB	3	6.41667	0.227
0	0	ycaM	3	6.41667	0.24
0	0	yadI	3	6.41667	0.35
0	0	AG1	3	6.41667	0.276
0	0	ptsI	3	6.66667	0.238
0	0	clcB	3	6.66667	0.236
0	0	ycaM	3	6.66667	0.256
0	0	yadI	3	6.66667	0.369
0	0	AG1	3	6.66667	0.29
0	0	ptsI	3	6.91667	0.25
0	0	clcB	3	6.91667	0.245
0	0	ycaM	3	6.91667	0.268
0	0	yadI	3	6.91667	0.383
0	0	AG1	3	6.91667	0.296
0	0	ptsI	3	7.16667	0.259
0	0	clcB	3	7.16667	0.256
0	0	ycaM	3	7.16667	0.274
0	0	yadI	3	7.16667	0.405
0	0	AG1	3	7.16667	0.303
0	0	ptsI	3	7.41667	0.268
0	0	clcB	3	7.41667	0.268
0	0	ycaM	3	7.41667	0.285
0	0	yadI	3	7.41667	0.42
0	0	AG1	3	7.41667	0.312
0	0	ptsI	3	7.66667	0.28
0	0	clcB	3	7.66667	0.283
0	0	ycaM	3	7.66667	0.295
0	0	yadI	3	7.66667	0.432
0	0	AG1	3	7.66667	0.324
0	0	ptsI	3	7.91667	0.286
0	0	clcB	3	7.91667	0.294
0	0	ycaM	3	7.91667	0.302
0	0	yadI	3	7.91667	0.45
0	0	AG1	3	7.91667	0.33
0	0	ptsI	3	8.16667	0.296
0	0	clcB	3	8.16667	0.301

0	0	ycaM	3	8.16667	0.312
0	0	yadI	3	8.16667	0.466
0	0	AG1	3	8.16667	0.337
0	0	ptsI	3	8.41667	0.31
0	0	clcB	3	8.41667	0.311
0	0	ycaM	3	8.41667	0.324
0	0	yadI	3	8.41667	0.488
0	0	AG1	3	8.41667	0.349
0	0	ptsI	3	9.05	0.267
0	0	clcB	3	9.05	0.255
0	0	ycaM	3	9.05	0.294
0	0	yadI	3	9.05	0.401
0	0	AG1	3	9.05	0.339
0	0	ptsI	3	9.3	0.267
0	0	clcB	3	9.3	0.251
0	0	ycaM	3	9.3	0.304
0	0	yadI	3	9.3	0.408
0	0	AG1	3	9.3	0.34
0	0	ptsI	3	9.55	0.278
0	0	clcB	3	9.55	0.259
0	0	ycaM	3	9.55	0.317
0	0	yadI	3	9.55	0.415
0	0	AG1	3	9.55	0.35
0	0	ptsI	3	9.8	0.284
0	0	clcB	3	9.8	0.267
0	0	ycaM	3	9.8	0.328
0	0	yadI	3	9.8	0.426
0	0	AG1	3	9.8	0.357
0	0	ptsI	3	10.05	0.288
0	0	clcB	3	10.05	0.274
0	0	ycaM	3	10.05	0.335
0	0	yadI	3	10.05	0.439
0	0	AG1	3	10.05	0.366
0	0	ptsI	3	10.3	0.299
0	0	clcB	3	10.3	0.278
0	0	ycaM	3	10.3	0.343
0	0	yadI	3	10.3	0.451
0	0	AG1	3	10.3	0.375
0	0	ptsI	3	10.55	0.308
0	0	clcB	3	10.55	0.287
0	0	ycaM	3	10.55	0.356
0	0	yadI	3	10.55	0.47
0	0	AG1	3	10.55	0.388
0	0	ptsI	3	10.8	0.316
0	0	clcB	3	10.8	0.292
0	0	ycaM	3	10.8	0.359
0	0	yadI	3	10.8	0.475
0	0	AG1	3	10.8	0.394
0	0	ptsI	3	11.05	0.322
0	0	clcB	3	11.05	0.301
0	0	ycaM	3	11.05	0.366
0	0	yadI	3	11.05	0.487
0	0	AG1	3	11.05	0.405

0	0	ptsI	3	11.3	0.331
0	0	clcB	3	11.3	0.31
0	0	ycaM	3	11.3	0.37
0	0	yadI	3	11.3	0.514
0	0	AG1	3	11.3	0.411
0	0	ptsI	3	11.55	0.339
0	0	clcB	3	11.55	0.317
0	0	ycaM	3	11.55	0.372
0	0	yadI	3	11.55	0.517
0	0	AG1	3	11.55	0.421
0	0	ptsI	3	11.8	0.348
0	0	clcB	3	11.8	0.328
0	0	ycaM	3	11.8	0.374
0	0	yadI	3	11.8	0.519
0	0	AG1	3	11.8	0.433
0	0	ptsI	3	12.05	0.354
0	0	clcB	3	12.05	0.336
0	0	ycaM	3	12.05	0.378
0	0	yadI	3	12.05	0.537
0	0	AG1	3	12.05	0.441
0	0	ptsI	3	12.3	0.367
0	0	clcB	3	12.3	0.348
0	0	ycaM	3	12.3	0.39
0	0	yadI	3	12.3	0.551
0	0	AG1	3	12.3	0.457
0	0	ptsI	3	12.55	0.379
0	0	clcB	3	12.55	0.358
0	0	ycaM	3	12.55	0.402
0	0	yadI	3	12.55	0.573
0	0	AG1	3	12.55	0.474
0	0	ptsI	3	12.8	0.385
0	0	clcB	3	12.8	0.364
0	0	ycaM	3	12.8	0.408
0	0	yadI	3	12.8	0.602
0	0	AG1	3	12.8	0.482
0	0	ptsI	3	13.05	0.392
0	0	clcB	3	13.05	0.362
0	0	ycaM	3	13.05	0.408
0	0	yadI	3	13.05	0.575
0	0	AG1	3	13.05	0.484
0	0	ptsI	3	13.3	0.396
0	0	clcB	3	13.3	0.365
0	0	ycaM	3	13.3	0.415
0	0	yadI	3	13.3	0.588
0	0	AG1	3	13.3	0.495
0	0	ptsI	3	13.55	0.41
0	0	clcB	3	13.55	0.377
0	0	ycaM	3	13.55	0.426
0	0	yadI	3	13.55	0.621
0	0	AG1	3	13.55	0.508
0	0	ptsI	3	13.8	0.423
0	0	clcB	3	13.8	0.39
0	0	ycaM	3	13.8	0.438

0	0	yadI	3	13.8	0.637
0	0	AG1	3	13.8	0.525
0	0	ptsI	3	14.05	0.426
0	0	clcB	3	14.05	0.39
0	0	ycaM	3	14.05	0.443
0	0	yadI	3	14.05	0.617
0	0	AG1	3	14.05	0.529
0	0	ptsI	3	14.3	0.437
0	0	clcB	3	14.3	0.403
0	0	ycaM	3	14.3	0.45
0	0	yadI	3	14.3	0.628
0	0	AG1	3	14.3	0.542
0	0	ptsI	3	14.55	0.441
0	0	clcB	3	14.55	0.411
0	0	ycaM	3	14.55	0.46
0	0	yadI	3	14.55	0.646
0	0	AG1	3	14.55	0.546
0	0	ptsI	3	14.8	0.466
0	0	clcB	3	14.8	0.431
0	0	ycaM	3	14.8	0.473
0	0	yadI	3	14.8	0.669
0	0	AG1	3	14.8	0.56
0	0	ptsI	3	15.05	0.47
0	0	clcB	3	15.05	0.438
0	0	ycaM	3	15.05	0.477
0	0	yadI	3	15.05	0.682
0	0	AG1	3	15.05	0.568
0	0	ptsI	3	15.3	0.477
0	0	clcB	3	15.3	0.446
0	0	ycaM	3	15.3	0.474
0	0	yadI	3	15.3	0.684
0	0	AG1	3	15.3	0.573
0	0	ptsI	3	15.55	0.474
0	0	clcB	3	15.55	0.446
0	0	ycaM	3	15.55	0.479
0	0	yadI	3	15.55	0.686
0	0	AG1	3	15.55	0.574
0	0	ptsI	3	15.8	0.49
0	0	clcB	3	15.8	0.456
0	0	ycaM	3	15.8	0.489
0	0	yadI	3	15.8	0.698
0	0	AG1	3	15.8	0.599
0	0	ptsI	3	16.05	0.485
0	0	clcB	3	16.05	0.459
0	0	ycaM	3	16.05	0.489
0	0	yadI	3	16.05	0.699
0	0	AG1	3	16.05	0.586
0	0	ptsI	3	16.3	0.494
0	0	clcB	3	16.3	0.461
0	0	ycaM	3	16.3	0.492
0	0	yadI	3	16.3	0.699
0	0	AG1	3	16.3	0.589
0	0	ptsI	3	16.55	0.512

0	0	clcB	3	16.55	0.47
0	0	ycaM	3	16.55	0.504
0	0	yadI	3	16.55	0.717
0	0	AG1	3	16.55	0.609
0	0	ptsI	3	16.8	0.532
0	0	clcB	3	16.8	0.483
0	0	ycaM	3	16.8	0.508
0	0	yadI	3	16.8	0.733
0	0	AG1	3	16.8	0.627
0	0	ptsI	3	17.05	0.543
0	0	clcB	3	17.05	0.486
0	0	ycaM	3	17.05	0.508
0	0	yadI	3	17.05	0.734
0	0	AG1	3	17.05	0.642
0	0	ptsI	3	17.3	0.539
0	0	clcB	3	17.3	0.494
0	0	ycaM	3	17.3	0.523
0	0	yadI	3	17.3	0.741
0	0	AG1	3	17.3	0.637
0	0	ptsI	3	17.55	0.548
0	0	clcB	3	17.55	0.494
0	0	ycaM	3	17.55	0.516
0	0	yadI	3	17.55	0.747
0	0	AG1	3	17.55	0.648
0	0	ptsI	3	17.8	0.56
0	0	clcB	3	17.8	0.498
0	0	ycaM	3	17.8	0.522
0	0	yadI	3	17.8	0.755
0	0	AG1	3	17.8	0.645
0	0	ptsI	3	18.05	0.562
0	0	clcB	3	18.05	0.501
0	0	ycaM	3	18.05	0.527
0	0	yadI	3	18.05	0.781
0	0	AG1	3	18.05	0.649
0	0	ptsI	3	18.3	0.57
0	0	clcB	3	18.3	0.507
0	0	ycaM	3	18.3	0.535
0	0	yadI	3	18.3	0.778
0	0	AG1	3	18.3	0.667
0	0	ptsI	3	18.55	0.571
0	0	clcB	3	18.55	0.511
0	0	ycaM	3	18.55	0.542
0	0	yadI	3	18.55	0.772
0	0	AG1	3	18.55	0.675
0	0	ptsI	3	18.8	0.577
0	0	clcB	3	18.8	0.523
0	0	ycaM	3	18.8	0.547
0	0	yadI	3	18.8	0.799
0	0	AG1	3	18.8	0.664
0	0	ptsI	3	19.05	0.594
0	0	clcB	3	19.05	0.528
0	0	ycaM	3	19.05	0.556
0	0	yadI	3	19.05	0.79

0	0	AG1	3	19.05	0.702
0	0	ptsI	3	19.3	0.603
0	0	clcB	3	19.3	0.538
0	0	ycaM	3	19.3	0.578
0	0	yadI	3	19.3	0.804
0	0	AG1	3	19.3	0.699
0	0	ptsI	3	19.55	0.619
0	0	clcB	3	19.55	0.555
0	0	ycaM	3	19.55	0.595
0	0	yadI	3	19.55	0.843
0	0	AG1	3	19.55	0.711
0	0	ptsI	3	19.8	0.612
0	0	clcB	3	19.8	0.551
0	0	ycaM	3	19.8	0.583
0	0	yadI	3	19.8	0.81
0	0	AG1	3	19.8	0.722
0	0	ptsI	3	20.05	0.63
0	0	clcB	3	20.05	0.562
0	0	ycaM	3	20.05	0.588
0	0	yadI	3	20.05	0.832
0	0	AG1	3	20.05	0.728
0	0	ptsI	3	20.3	0.621
0	0	clcB	3	20.3	0.554
0	0	ycaM	3	20.3	0.582
0	0	yadI	3	20.3	0.828
0	0	AG1	3	20.3	0.73
0	0	ptsI	3	20.55	0.63
0	0	clcB	3	20.55	0.567
0	0	ycaM	3	20.55	0.597
0	0	yadI	3	20.55	0.839
0	0	AG1	3	20.55	0.749
0	0	ptsI	3	20.8	0.638
0	0	clcB	3	20.8	0.573
0	0	ycaM	3	20.8	0.607
0	0	yadI	3	20.8	0.859
0	0	AG1	3	20.8	0.747
0	0	ptsI	3	21.05	0.648
0	0	clcB	3	21.05	0.572
0	0	ycaM	3	21.05	0.602
0	0	yadI	3	21.05	0.869
0	0	AG1	3	21.05	0.74
0	0	ptsI	3	21.3	0.65
0	0	clcB	3	21.3	0.574
0	0	ycaM	3	21.3	0.605
0	0	yadI	3	21.3	0.862
0	0	AG1	3	21.3	0.744
0	0	ptsI	3	21.55	0.674
0	0	clcB	3	21.55	0.584
0	0	ycaM	3	21.55	0.62
0	0	yadI	3	21.55	0.871
0	0	AG1	3	21.55	0.757
0	0	ptsI	3	21.8	0.683
0	0	clcB	3	21.8	0.587

0	0	ycaM	3	21.8	0.624
0	0	yadI	3	21.8	0.88
0	0	AG1	3	21.8	0.764
0	0	ptsI	3	22.05	0.704
0	0	clcB	3	22.05	0.586
0	0	ycaM	3	22.05	0.626
0	0	yadI	3	22.05	0.894
0	0	AG1	3	22.05	0.773
0	0	ptsI	3	22.3	0.72
0	0	clcB	3	22.3	0.591
0	0	ycaM	3	22.3	0.637
0	0	yadI	3	22.3	0.913
0	0	AG1	3	22.3	0.783
0	0	ptsI	3	22.55	0.737
0	0	clcB	3	22.55	0.592
0	0	ycaM	3	22.55	0.642
0	0	yadI	3	22.55	0.902
0	0	AG1	3	22.55	0.793
0	0	ptsI	3	22.8	0.744
0	0	clcB	3	22.8	0.598
0	0	ycaM	3	22.8	0.641
0	0	yadI	3	22.8	0.904
0	0	AG1	3	22.8	0.801
0	0	ptsI	3	23.05	0.761
0	0	clcB	3	23.05	0.601
0	0	ycaM	3	23.05	0.651
0	0	yadI	3	23.05	0.928
0	0	AG1	3	23.05	0.804
0	0	ptsI	3	23.3	0.776
0	0	clcB	3	23.3	0.607
0	0	ycaM	3	23.3	0.653
0	0	yadI	3	23.3	0.928
0	0	AG1	3	23.3	0.814
0	0	ptsI	3	23.55	0.812
0	0	clcB	3	23.55	0.628
0	0	ycaM	3	23.55	0.678
0	0	yadI	3	23.55	0.936
0	0	AG1	3	23.55	0.827
0	0	ptsI	3	23.8	0.801
0	0	clcB	3	23.8	0.618
0	0	ycaM	3	23.8	0.675
0	0	yadI	3	23.8	0.942
0	0	AG1	3	23.8	0.826
0	0	ptsI	3	24.05	0.809
0	0	clcB	3	24.05	0.62
0	0	ycaM	3	24.05	0.679
0	0	yadI	3	24.05	0.959
0	0	AG1	3	24.05	0.83
0	0	ptsI	3	24.3	0.794
0	0	clcB	3	24.3	0.623
0	0	ycaM	3	24.3	0.672
0	0	yadI	3	24.3	0.938
0	0	AG1	3	24.3	0.815

0	0	ptsI	4	0	0.182
0	0	clcB	4	0	0.196
0	0	ycaM	4	0	0.195
0	0	yadI	4	0	0.212
0	0	AG1	4	0	0.202
0	0	ptsI	4	0.25	0.181
0	0	clcB	4	0.25	0.192
0	0	ycaM	4	0.25	0.193
0	0	yadI	4	0.25	0.21
0	0	AG1	4	0.25	0.204
0	0	ptsI	4	0.5	0.184
0	0	clcB	4	0.5	0.193
0	0	ycaM	4	0.5	0.193
0	0	yadI	4	0.5	0.212
0	0	AG1	4	0.5	0.207
0	0	ptsI	4	0.75	0.189
0	0	clcB	4	0.75	0.196
0	0	ycaM	4	0.75	0.196
0	0	yadI	4	0.75	0.215
0	0	AG1	4	0.75	0.211
0	0	ptsI	4	1	0.195
0	0	clcB	4	1	0.201
0	0	ycaM	4	1	0.199
0	0	yadI	4	1	0.219
0	0	AG1	4	1	0.216
0	0	ptsI	4	1.25	0.206
0	0	clcB	4	1.25	0.207
0	0	ycaM	4	1.25	0.21
0	0	yadI	4	1.25	0.225
0	0	AG1	4	1.25	0.221
0	0	ptsI	4	1.5	0.205
0	0	clcB	4	1.5	0.212
0	0	ycaM	4	1.5	0.212
0	0	yadI	4	1.5	0.23
0	0	AG1	4	1.5	0.224
0	0	ptsI	4	1.75	0.22
0	0	clcB	4	1.75	0.224
0	0	ycaM	4	1.75	0.221
0	0	yadI	4	1.75	0.241
0	0	AG1	4	1.75	0.236
0	0	ptsI	4	2	0.233
0	0	clcB	4	2	0.235
0	0	ycaM	4	2	0.23
0	0	yadI	4	2	0.254
0	0	AG1	4	2	0.248
0	0	ptsI	4	2.25	0.246
0	0	clcB	4	2.25	0.249
0	0	ycaM	4	2.25	0.239
0	0	yadI	4	2.25	0.266
0	0	AG1	4	2.25	0.26
0	0	ptsI	4	2.5	0.254
0	0	clcB	4	2.5	0.259
0	0	ycaM	4	2.5	0.25

0	0	yadI	4	2.5	0.28
0	0	AG1	4	2.5	0.267
0	0	ptsI	4	2.75	0.272
0	0	clcB	4	2.75	0.269
0	0	ycaM	4	2.75	0.264
0	0	yadI	4	2.75	0.299
0	0	AG1	4	2.75	0.28
0	0	ptsI	4	3	0.284
0	0	clcB	4	3	0.292
0	0	ycaM	4	3	0.28
0	0	yadI	4	3	0.318
0	0	AG1	4	3	0.298
0	0	ptsI	4	3.25	0.297
0	0	clcB	4	3.25	0.301
0	0	ycaM	4	3.25	0.297
0	0	yadI	4	3.25	0.332
0	0	AG1	4	3.25	0.311
0	0	ptsI	4	3.5	0.319
0	0	clcB	4	3.5	0.32
0	0	ycaM	4	3.5	0.312
0	0	yadI	4	3.5	0.355
0	0	AG1	4	3.5	0.33
0	0	ptsI	4	3.75	0.338
0	0	clcB	4	3.75	0.326
0	0	ycaM	4	3.75	0.333
0	0	yadI	4	3.75	0.372
0	0	AG1	4	3.75	0.343
0	0	ptsI	4	4	0.351
0	0	clcB	4	4	0.35
0	0	ycaM	4	4	0.35
0	0	yadI	4	4	0.401
0	0	AG1	4	4	0.364
0	0	ptsI	4	4.35	0.38
0	0	clcB	4	4.35	0.293
0	0	ycaM	4	4.35	0.415
0	0	yadI	4	4.35	0.348
0	0	AG1	4	4.35	0.406
0	0	ptsI	4	4.6	0.377
0	0	clcB	4	4.6	0.288
0	0	ycaM	4	4.6	0.405
0	0	yadI	4	4.6	0.324
0	0	AG1	4	4.6	0.407
0	0	ptsI	4	4.85	0.39
0	0	clcB	4	4.85	0.291
0	0	ycaM	4	4.85	0.415
0	0	yadI	4	4.85	0.332
0	0	AG1	4	4.85	0.417
0	0	ptsI	4	5.1	0.403
0	0	clcB	4	5.1	0.301
0	0	ycaM	4	5.1	0.428
0	0	yadI	4	5.1	0.344
0	0	AG1	4	5.1	0.431
0	0	ptsI	4	5.35	0.416

0	0	clcB	4	5.35	0.312
0	0	ycaM	4	5.35	0.44
0	0	yadI	4	5.35	0.359
0	0	AG1	4	5.35	0.447
0	0	ptsI	4	5.6	0.433
0	0	clcB	4	5.6	0.317
0	0	ycaM	4	5.6	0.455
0	0	yadI	4	5.6	0.369
0	0	AG1	4	5.6	0.458
0	0	ptsI	4	5.85	0.452
0	0	clcB	4	5.85	0.328
0	0	ycaM	4	5.85	0.466
0	0	yadI	4	5.85	0.383
0	0	AG1	4	5.85	0.474
0	0	ptsI	4	6.1	0.469
0	0	clcB	4	6.1	0.345
0	0	ycaM	4	6.1	0.485
0	0	yadI	4	6.1	0.399
0	0	AG1	4	6.1	0.485
0	0	ptsI	4	6.35	0.484
0	0	clcB	4	6.35	0.35
0	0	ycaM	4	6.35	0.496
0	0	yadI	4	6.35	0.414
0	0	AG1	4	6.35	0.497
0	0	ptsI	4	6.6	0.502
0	0	clcB	4	6.6	0.364
0	0	ycaM	4	6.6	0.509
0	0	yadI	4	6.6	0.433
0	0	AG1	4	6.6	0.512
0	0	ptsI	4	6.85	0.515
0	0	clcB	4	6.85	0.374
0	0	ycaM	4	6.85	0.519
0	0	yadI	4	6.85	0.441
0	0	AG1	4	6.85	0.528
0	0	ptsI	4	7.1	0.526
0	0	clcB	4	7.1	0.383
0	0	ycaM	4	7.1	0.531
0	0	yadI	4	7.1	0.456
0	0	AG1	4	7.1	0.536
0	0	ptsI	4	7.35	0.533
0	0	clcB	4	7.35	0.388
0	0	ycaM	4	7.35	0.536
0	0	yadI	4	7.35	0.457
0	0	AG1	4	7.35	0.547
0	0	ptsI	4	7.6	0.541
0	0	clcB	4	7.6	0.398
0	0	ycaM	4	7.6	0.545
0	0	yadI	4	7.6	0.473
0	0	AG1	4	7.6	0.558
0	0	ptsI	4	7.85	0.546
0	0	clcB	4	7.85	0.404
0	0	ycaM	4	7.85	0.546
0	0	yadI	4	7.85	0.476

0	0	AG1	4	7.85	0.565
0	0	ptsI	4	8.1	0.56
0	0	clcB	4	8.1	0.422
0	0	ycaM	4	8.1	0.563
0	0	yadI	4	8.1	0.496
0	0	AG1	4	8.1	0.578
0	0	ptsI	4	8.35	0.572
0	0	clcB	4	8.35	0.421
0	0	ycaM	4	8.35	0.564
0	0	yadI	4	8.35	0.502
0	0	AG1	4	8.35	0.586
0	0	ptsI	4	8.83333	0.379
0	0	clcB	4	8.83333	0.394
0	0	ycaM	4	8.83333	0.397
0	0	yadI	4	8.83333	0.53
0	0	AG1	4	8.83333	0.619
0	0	ptsI	4	9.08333	0.37
0	0	clcB	4	9.08333	0.386
0	0	ycaM	4	9.08333	0.389
0	0	yadI	4	9.08333	0.522
0	0	AG1	4	9.08333	0.62
0	0	ptsI	4	9.33333	0.378
0	0	clcB	4	9.33333	0.392
0	0	ycaM	4	9.33333	0.394
0	0	yadI	4	9.33333	0.533
0	0	AG1	4	9.33333	0.628
0	0	ptsI	4	9.58333	0.382
0	0	clcB	4	9.58333	0.395
0	0	ycaM	4	9.58333	0.402
0	0	yadI	4	9.58333	0.543
0	0	AG1	4	9.58333	0.64
0	0	ptsI	4	9.83333	0.389
0	0	clcB	4	9.83333	0.4
0	0	ycaM	4	9.83333	0.4
0	0	yadI	4	9.83333	0.548
0	0	AG1	4	9.83333	0.65
0	0	ptsI	4	10.0833	0.397
0	0	clcB	4	10.0833	0.406
0	0	ycaM	4	10.0833	0.407
0	0	yadI	4	10.0833	0.557
0	0	AG1	4	10.0833	0.66
0	0	ptsI	4	10.3333	0.407
0	0	clcB	4	10.3333	0.414
0	0	ycaM	4	10.3333	0.415
0	0	yadI	4	10.3333	0.566
0	0	AG1	4	10.3333	0.669
0	0	ptsI	4	10.5833	0.418
0	0	clcB	4	10.5833	0.427
0	0	ycaM	4	10.5833	0.426
0	0	yadI	4	10.5833	0.578
0	0	AG1	4	10.5833	0.684
0	0	ptsI	4	10.8333	0.417
0	0	clcB	4	10.8333	0.43

0	0	ycaM	4	10.8333	0.425
0	0	yadI	4	10.8333	0.583
0	0	AG1	4	10.8333	0.686
0	0	ptsI	4	11.0833	0.431
0	0	clcB	4	11.0833	0.425
0	0	ycaM	4	11.0833	0.424
0	0	yadI	4	11.0833	0.584
0	0	AG1	4	11.0833	0.698
0	0	ptsI	4	11.3333	0.437
0	0	clcB	4	11.3333	0.449
0	0	ycaM	4	11.3333	0.442
0	0	yadI	4	11.3333	0.6
0	0	AG1	4	11.3333	0.71
0	0	ptsI	4	11.5833	0.436
0	0	clcB	4	11.5833	0.442
0	0	ycaM	4	11.5833	0.439
0	0	yadI	4	11.5833	0.603
0	0	AG1	4	11.5833	0.713
0	0	ptsI	4	11.8333	0.442
0	0	clcB	4	11.8333	0.443
0	0	ycaM	4	11.8333	0.437
0	0	yadI	4	11.8333	0.602
0	0	AG1	4	11.8333	0.721
0	0	ptsI	4	12.0833	0.448
0	0	clcB	4	12.0833	0.452
0	0	ycaM	4	12.0833	0.448
0	0	yadI	4	12.0833	0.614
0	0	AG1	4	12.0833	0.729
0	0	ptsI	4	12.3333	0.456
0	0	clcB	4	12.3333	0.462
0	0	ycaM	4	12.3333	0.456
0	0	yadI	4	12.3333	0.615
0	0	AG1	4	12.3333	0.734
0	0	ptsI	4	12.5833	0.468
0	0	clcB	4	12.5833	0.462
0	0	ycaM	4	12.5833	0.458
0	0	yadI	4	12.5833	0.619
0	0	AG1	4	12.5833	0.738
0	0	ptsI	4	12.8333	0.466
0	0	clcB	4	12.8333	0.463
0	0	ycaM	4	12.8333	0.46
0	0	yadI	4	12.8333	0.623
0	0	AG1	4	12.8333	0.748
0	0	ptsI	4	13.0833	0.479
0	0	clcB	4	13.0833	0.474
0	0	ycaM	4	13.0833	0.466
0	0	yadI	4	13.0833	0.627
0	0	AG1	4	13.0833	0.751
0	0	ptsI	4	13.3333	0.491
0	0	clcB	4	13.3333	0.483
0	0	ycaM	4	13.3333	0.474
0	0	yadI	4	13.3333	0.634
0	0	AG1	4	13.3333	0.758

0	0	ptsI	4	13.5833	0.489
0	0	clcB	4	13.5833	0.481
0	0	ycaM	4	13.5833	0.474
0	0	yadI	4	13.5833	0.634
0	0	AG1	4	13.5833	0.757
0	0	ptsI	4	13.8333	0.489
0	0	clcB	4	13.8333	0.487
0	0	ycaM	4	13.8333	0.484
0	0	yadI	4	13.8333	0.642
0	0	AG1	4	13.8333	0.764
0	0	ptsI	4	14.0833	0.49
0	0	clcB	4	14.0833	0.491
0	0	ycaM	4	14.0833	0.487
0	0	yadI	4	14.0833	0.644
0	0	AG1	4	14.0833	0.769
0	0	ptsI	4	14.3333	0.495
0	0	clcB	4	14.3333	0.496
0	0	ycaM	4	14.3333	0.498
0	0	yadI	4	14.3333	0.654
0	0	AG1	4	14.3333	0.788
0	0	ptsI	4	14.5833	0.501
0	0	clcB	4	14.5833	0.499
0	0	ycaM	4	14.5833	0.491
0	0	yadI	4	14.5833	0.648
0	0	AG1	4	14.5833	0.777
0	0	ptsI	4	14.8333	0.515
0	0	clcB	4	14.8333	0.499
0	0	ycaM	4	14.8333	0.498
0	0	yadI	4	14.8333	0.654
0	0	AG1	4	14.8333	0.784
0	0	ptsI	4	15.0833	0.513
0	0	clcB	4	15.0833	0.497
0	0	ycaM	4	15.0833	0.497
0	0	yadI	4	15.0833	0.653
0	0	AG1	4	15.0833	0.783
0	0	ptsI	4	15.3333	0.512
0	0	clcB	4	15.3333	0.496
0	0	ycaM	4	15.3333	0.507
0	0	yadI	4	15.3333	0.663
0	0	AG1	4	15.3333	0.799
0	0	ptsI	4	15.5833	0.53
0	0	clcB	4	15.5833	0.513
0	0	ycaM	4	15.5833	0.512
0	0	yadI	4	15.5833	0.663
0	0	AG1	4	15.5833	0.797
0	0	ptsI	4	15.8333	0.514
0	0	clcB	4	15.8333	0.501
0	0	ycaM	4	15.8333	0.512
0	0	yadI	4	15.8333	0.67
0	0	AG1	4	15.8333	0.796
0	0	ptsI	4	16.0833	0.534
0	0	clcB	4	16.0833	0.517
0	0	ycaM	4	16.0833	0.519

0	0	yadI	4	16.0833	0.668
0	0	AG1	4	16.0833	0.801
0	0	ptsI	4	16.3333	0.524
0	0	clcB	4	16.3333	0.502
0	0	ycaM	4	16.3333	0.512
0	0	yadI	4	16.3333	0.678
0	0	AG1	4	16.3333	0.803
0	0	ptsI	4	16.5833	0.524
0	0	clcB	4	16.5833	0.502
0	0	ycaM	4	16.5833	0.517
0	0	yadI	4	16.5833	0.678
0	0	AG1	4	16.5833	0.81
0	0	ptsI	4	16.8333	0.54
0	0	clcB	4	16.8333	0.515
0	0	ycaM	4	16.8333	0.521
0	0	yadI	4	16.8333	0.674
0	0	AG1	4	16.8333	0.803
0	0	ptsI	4	17.0833	0.535
0	0	clcB	4	17.0833	0.509
0	0	ycaM	4	17.0833	0.52
0	0	yadI	4	17.0833	0.676
0	0	AG1	4	17.0833	0.801
0	0	ptsI	4	17.3333	0.535
0	0	clcB	4	17.3333	0.512
0	0	ycaM	4	17.3333	0.528
0	0	yadI	4	17.3333	0.689
0	0	AG1	4	17.3333	0.817
0	0	ptsI	4	17.5833	0.542
0	0	clcB	4	17.5833	0.518
0	0	ycaM	4	17.5833	0.525
0	0	yadI	4	17.5833	0.678
0	0	AG1	4	17.5833	0.807
0	0	ptsI	4	17.8333	0.545
0	0	clcB	4	17.8333	0.515
0	0	ycaM	4	17.8333	0.524
0	0	yadI	4	17.8333	0.678
0	0	AG1	4	17.8333	0.808
0	0	ptsI	4	18.0833	0.55
0	0	clcB	4	18.0833	0.52
0	0	ycaM	4	18.0833	0.529
0	0	yadI	4	18.0833	0.687
0	0	AG1	4	18.0833	0.808
0	0	ptsI	4	18.3333	0.545
0	0	clcB	4	18.3333	0.518
0	0	ycaM	4	18.3333	0.537
0	0	yadI	4	18.3333	0.686
0	0	AG1	4	18.3333	0.814
0	0	ptsI	4	18.5833	0.556
0	0	clcB	4	18.5833	0.523
0	0	ycaM	4	18.5833	0.529
0	0	yadI	4	18.5833	0.689
0	0	AG1	4	18.5833	0.814
0	0	ptsI	4	18.8333	0.557

0	0	clcB	4	18.8333	0.516
0	0	ycaM	4	18.8333	0.53
0	0	yadI	4	18.8333	0.691
0	0	AG1	4	18.8333	0.819
0	0	ptsI	4	19.0833	0.549
0	0	clcB	4	19.0833	0.526
0	0	ycaM	4	19.0833	0.532
0	0	yadI	4	19.0833	0.693
0	0	AG1	4	19.0833	0.818
0	0	ptsI	4	19.3333	0.556
0	0	clcB	4	19.3333	0.519
0	0	ycaM	4	19.3333	0.534
0	0	yadI	4	19.3333	0.707
0	0	AG1	4	19.3333	0.829
0	0	ptsI	4	19.5833	0.57
0	0	clcB	4	19.5833	0.536
0	0	ycaM	4	19.5833	0.541
0	0	yadI	4	19.5833	0.704
0	0	AG1	4	19.5833	0.829
0	0	ptsI	4	19.8333	0.57
0	0	clcB	4	19.8333	0.532
0	0	ycaM	4	19.8333	0.533
0	0	yadI	4	19.8333	0.694
0	0	AG1	4	19.8333	0.823
0	0	ptsI	4	20.0833	0.566
0	0	clcB	4	20.0833	0.53
0	0	ycaM	4	20.0833	0.544
0	0	yadI	4	20.0833	0.707
0	0	AG1	4	20.0833	0.824
0	0	ptsI	4	20.3333	0.572
0	0	clcB	4	20.3333	0.534
0	0	ycaM	4	20.3333	0.548
0	0	yadI	4	20.3333	0.706
0	0	AG1	4	20.3333	0.828
0	0	ptsI	4	20.5833	0.565
0	0	clcB	4	20.5833	0.53
0	0	ycaM	4	20.5833	0.543
0	0	yadI	4	20.5833	0.712
0	0	AG1	4	20.5833	0.828
0	0	ptsI	4	20.8333	0.568
0	0	clcB	4	20.8333	0.531
0	0	ycaM	4	20.8333	0.551
0	0	yadI	4	20.8333	0.713
0	0	AG1	4	20.8333	0.839
0	0	ptsI	4	21.0833	0.581
0	0	clcB	4	21.0833	0.536
0	0	ycaM	4	21.0833	0.554
0	0	yadI	4	21.0833	0.711
0	0	AG1	4	21.0833	0.836
0	0	ptsI	4	21.3333	0.573
0	0	clcB	4	21.3333	0.534
0	0	ycaM	4	21.3333	0.543
0	0	yadI	4	21.3333	0.718

0	0	AG1	4	21.3333	0.84
0	0	ptsI	4	21.5833	0.582
0	0	clcB	4	21.5833	0.541
0	0	ycaM	4	21.5833	0.558
0	0	yadI	4	21.5833	0.712
0	0	AG1	4	21.5833	0.842
0	0	ptsI	4	21.8333	0.571
0	0	clcB	4	21.8333	0.529
0	0	ycaM	4	21.8333	0.546
0	0	yadI	4	21.8333	0.708
0	0	AG1	4	21.8333	0.837
0	0	ptsI	4	22.0833	0.582
0	0	clcB	4	22.0833	0.539
0	0	ycaM	4	22.0833	0.556
0	0	yadI	4	22.0833	0.717
0	0	AG1	4	22.0833	0.841
0	0	ptsI	4	22.3333	0.594
0	0	clcB	4	22.3333	0.549
0	0	ycaM	4	22.3333	0.566
0	0	yadI	4	22.3333	0.713
0	0	AG1	4	22.3333	0.842
0	0	ptsI	4	22.5833	0.579
0	0	clcB	4	22.5833	0.539
0	0	ycaM	4	22.5833	0.561
0	0	yadI	4	22.5833	0.72
0	0	AG1	4	22.5833	0.838
0	0	ptsI	4	22.8333	0.584
0	0	clcB	4	22.8333	0.543
0	0	ycaM	4	22.8333	0.555
0	0	yadI	4	22.8333	0.719
0	0	AG1	4	22.8333	0.84
0	0	ptsI	4	23.0833	0.594
0	0	clcB	4	23.0833	0.547
0	0	ycaM	4	23.0833	0.566
0	0	yadI	4	23.0833	0.722
0	0	AG1	4	23.0833	0.843
0	0	ptsI	4	23.3333	0.583
0	0	clcB	4	23.3333	0.54
0	0	ycaM	4	23.3333	0.558
0	0	yadI	4	23.3333	0.728
0	0	AG1	4	23.3333	0.848
0	0	ptsI	4	23.5833	0.589
0	0	clcB	4	23.5833	0.541
0	0	ycaM	4	23.5833	0.557
0	0	yadI	4	23.5833	0.73
0	0	AG1	4	23.5833	0.846
0	0	ptsI	4	23.8333	0.593
0	0	clcB	4	23.8333	0.543
0	0	ycaM	4	23.8333	0.56
0	0	yadI	4	23.8333	0.733
0	0	AG1	4	23.8333	0.844
0	0	ptsI	4	24.0833	0.596
0	0	clcB	4	24.0833	0.543

0	0	ycaM	4	24.0833	0.561
0	0	yadI	4	24.0833	0.734
0	0	AG1	4	24.0833	0.848
0	0	ptsI	5	0	0.156
0	0	clcB	5	0	0.163
0	0	ycaM	5	0	0.158
0	0	yadI	5	0	0.181
0	0	AG1	5	0	0.171
0	0	ptsI	5	0.35	0.154
0	0	clcB	5	0.35	0.158
0	0	ycaM	5	0.35	0.153
0	0	yadI	5	0.35	0.174
0	0	AG1	5	0.35	0.168
0	0	ptsI	5	0.6	0.157
0	0	clcB	5	0.6	0.161
0	0	ycaM	5	0.6	0.152
0	0	yadI	5	0.6	0.173
0	0	AG1	5	0.6	0.17
0	0	ptsI	5	0.85	0.152
0	0	clcB	5	0.85	0.16
0	0	ycaM	5	0.85	0.152
0	0	yadI	5	0.85	0.177
0	0	AG1	5	0.85	0.17
0	0	ptsI	5	1.1	0.158
0	0	clcB	5	1.1	0.164
0	0	ycaM	5	1.1	0.155
0	0	yadI	5	1.1	0.178
0	0	AG1	5	1.1	0.172
0	0	ptsI	5	1.35	0.157
0	0	clcB	5	1.35	0.165
0	0	ycaM	5	1.35	0.154
0	0	yadI	5	1.35	0.182
0	0	AG1	5	1.35	0.175
0	0	ptsI	5	1.6	0.164
0	0	clcB	5	1.6	0.17
0	0	ycaM	5	1.6	0.159
0	0	yadI	5	1.6	0.185
0	0	AG1	5	1.6	0.18
0	0	ptsI	5	1.85	0.164
0	0	clcB	5	1.85	0.172
0	0	ycaM	5	1.85	0.161
0	0	yadI	5	1.85	0.191
0	0	AG1	5	1.85	0.185
0	0	ptsI	5	2.1	0.168
0	0	clcB	5	2.1	0.173
0	0	ycaM	5	2.1	0.165
0	0	yadI	5	2.1	0.196
0	0	AG1	5	2.1	0.188
0	0	ptsI	5	2.35	0.176
0	0	clcB	5	2.35	0.183
0	0	ycaM	5	2.35	0.172
0	0	yadI	5	2.35	0.203
0	0	AG1	5	2.35	0.201

0	0	ptsI	5	2.6	0.174
0	0	clcB	5	2.6	0.184
0	0	ycaM	5	2.6	0.176
0	0	yadI	5	2.6	0.212
0	0	AG1	5	2.6	0.203
0	0	ptsI	5	2.85	0.185
0	0	clcB	5	2.85	0.197
0	0	ycaM	5	2.85	0.184
0	0	yadI	5	2.85	0.219
0	0	AG1	5	2.85	0.215
0	0	ptsI	5	3.1	0.194
0	0	clcB	5	3.1	0.21
0	0	ycaM	5	3.1	0.191
0	0	yadI	5	3.1	0.228
0	0	AG1	5	3.1	0.23
0	0	ptsI	5	3.35	0.202
0	0	clcB	5	3.35	0.225
0	0	ycaM	5	3.35	0.201
0	0	yadI	5	3.35	0.241
0	0	AG1	5	3.35	0.251
0	0	ptsI	5	3.6	0.21
0	0	clcB	5	3.6	0.24
0	0	ycaM	5	3.6	0.209
0	0	yadI	5	3.6	0.259
0	0	AG1	5	3.6	0.266
0	0	ptsI	5	3.85	0.22
0	0	clcB	5	3.85	0.253
0	0	ycaM	5	3.85	0.223
0	0	yadI	5	3.85	0.276
0	0	AG1	5	3.85	0.283
0	0	ptsI	5	4.38333	0.214
0	0	clcB	5	4.38333	0.245
0	0	ycaM	5	4.38333	0.242
0	0	yadI	5	4.38333	0.288
0	0	AG1	5	4.38333	0.289
0	0	ptsI	5	4.63333	0.224
0	0	clcB	5	4.63333	0.249
0	0	ycaM	5	4.63333	0.239
0	0	yadI	5	4.63333	0.281
0	0	AG1	5	4.63333	0.294
0	0	ptsI	5	4.88333	0.232
0	0	clcB	5	4.88333	0.261
0	0	ycaM	5	4.88333	0.252
0	0	yadI	5	4.88333	0.298
0	0	AG1	5	4.88333	0.3
0	0	ptsI	5	5.13333	0.24
0	0	clcB	5	5.13333	0.273
0	0	ycaM	5	5.13333	0.264
0	0	yadI	5	5.13333	0.311
0	0	AG1	5	5.13333	0.312
0	0	ptsI	5	5.38333	0.248
0	0	clcB	5	5.38333	0.286
0	0	ycaM	5	5.38333	0.279

0	0	yadI	5	5.38333	0.322
0	0	AG1	5	5.38333	0.324
0	0	ptsI	5	5.63333	0.26
0	0	clcB	5	5.63333	0.301
0	0	ycaM	5	5.63333	0.29
0	0	yadI	5	5.63333	0.338
0	0	AG1	5	5.63333	0.336
0	0	ptsI	5	5.88333	0.271
0	0	clcB	5	5.88333	0.31
0	0	ycaM	5	5.88333	0.3
0	0	yadI	5	5.88333	0.355
0	0	AG1	5	5.88333	0.35
0	0	ptsI	5	6.13333	0.29
0	0	clcB	5	6.13333	0.33
0	0	ycaM	5	6.13333	0.317
0	0	yadI	5	6.13333	0.38
0	0	AG1	5	6.13333	0.375
0	0	ptsI	5	6.38333	0.293
0	0	clcB	5	6.38333	0.336
0	0	ycaM	5	6.38333	0.326
0	0	yadI	5	6.38333	0.401
0	0	AG1	5	6.38333	0.405
0	0	ptsI	5	6.63333	0.305
0	0	clcB	5	6.63333	0.347
0	0	ycaM	5	6.63333	0.34
0	0	yadI	5	6.63333	0.421
0	0	AG1	5	6.63333	0.43
0	0	ptsI	5	6.88333	0.316
0	0	clcB	5	6.88333	0.359
0	0	ycaM	5	6.88333	0.356
0	0	yadI	5	6.88333	0.439
0	0	AG1	5	6.88333	0.454
0	0	ptsI	5	7.13333	0.326
0	0	clcB	5	7.13333	0.37
0	0	ycaM	5	7.13333	0.373
0	0	yadI	5	7.13333	0.46
0	0	AG1	5	7.13333	0.471
0	0	ptsI	5	7.38333	0.342
0	0	clcB	5	7.38333	0.387
0	0	ycaM	5	7.38333	0.394
0	0	yadI	5	7.38333	0.486
0	0	AG1	5	7.38333	0.496
0	0	ptsI	5	7.63333	0.353
0	0	clcB	5	7.63333	0.396
0	0	ycaM	5	7.63333	0.404
0	0	yadI	5	7.63333	0.501
0	0	AG1	5	7.63333	0.52
0	0	ptsI	5	7.88333	0.364
0	0	clcB	5	7.88333	0.407
0	0	ycaM	5	7.88333	0.416
0	0	yadI	5	7.88333	0.511
0	0	AG1	5	7.88333	0.531
0	0	ptsI	5	8.13333	0.378

0	0	clcB	5	8.13333	0.414
0	0	ycaM	5	8.13333	0.427
0	0	yadI	5	8.13333	0.528
0	0	AG1	5	8.13333	0.552
0	0	ptsI	5	8.38333	0.39
0	0	clcB	5	8.38333	0.428
0	0	ycaM	5	8.38333	0.444
0	0	yadI	5	8.38333	0.537
0	0	AG1	5	8.38333	0.564
0	0	ptsI	5	8.88333	0.318
0	0	clcB	5	8.88333	0.361
0	0	ycaM	5	8.88333	0.382
0	0	yadI	5	8.88333	0.433
0	0	AG1	5	8.88333	0.402
0	0	ptsI	5	9.13333	0.319
0	0	clcB	5	9.13333	0.364
0	0	ycaM	5	9.13333	0.382
0	0	yadI	5	9.13333	0.436
0	0	AG1	5	9.13333	0.404
0	0	ptsI	5	9.38333	0.325
0	0	clcB	5	9.38333	0.369
0	0	ycaM	5	9.38333	0.389
0	0	yadI	5	9.38333	0.443
0	0	AG1	5	9.38333	0.407
0	0	ptsI	5	9.63333	0.339
0	0	clcB	5	9.63333	0.381
0	0	ycaM	5	9.63333	0.4
0	0	yadI	5	9.63333	0.458
0	0	AG1	5	9.63333	0.424
0	0	ptsI	5	9.88333	0.354
0	0	clcB	5	9.88333	0.394
0	0	ycaM	5	9.88333	0.413
0	0	yadI	5	9.88333	0.474
0	0	AG1	5	9.88333	0.435
0	0	ptsI	5	10.1333	0.362
0	0	clcB	5	10.1333	0.402
0	0	ycaM	5	10.1333	0.416
0	0	yadI	5	10.1333	0.472
0	0	AG1	5	10.1333	0.443
0	0	ptsI	5	10.3833	0.369
0	0	clcB	5	10.3833	0.408
0	0	ycaM	5	10.3833	0.419
0	0	yadI	5	10.3833	0.482
0	0	AG1	5	10.3833	0.449
0	0	ptsI	5	10.6333	0.384
0	0	clcB	5	10.6333	0.414
0	0	ycaM	5	10.6333	0.423
0	0	yadI	5	10.6333	0.487
0	0	AG1	5	10.6333	0.457
0	0	ptsI	5	10.8833	0.397
0	0	clcB	5	10.8833	0.427
0	0	ycaM	5	10.8833	0.434
0	0	yadI	5	10.8833	0.503

0	0	AG1	5	10.8833	0.47
0	0	ptsI	5	11.1333	0.402
0	0	clcB	5	11.1333	0.425
0	0	ycaM	5	11.1333	0.431
0	0	yadI	5	11.1333	0.505
0	0	AG1	5	11.1333	0.473
0	0	ptsI	5	11.3833	0.411
0	0	clcB	5	11.3833	0.43
0	0	ycaM	5	11.3833	0.435
0	0	yadI	5	11.3833	0.516
0	0	AG1	5	11.3833	0.486
0	0	ptsI	5	11.6333	0.419
0	0	clcB	5	11.6333	0.438
0	0	ycaM	5	11.6333	0.444
0	0	yadI	5	11.6333	0.52
0	0	AG1	5	11.6333	0.49
0	0	ptsI	5	11.8833	0.426
0	0	clcB	5	11.8833	0.441
0	0	ycaM	5	11.8833	0.445
0	0	yadI	5	11.8833	0.532
0	0	AG1	5	11.8833	0.496
0	0	ptsI	5	12.1333	0.444
0	0	clcB	5	12.1333	0.456
0	0	ycaM	5	12.1333	0.458
0	0	yadI	5	12.1333	0.543
0	0	AG1	5	12.1333	0.511
0	0	ptsI	5	12.3833	0.436
0	0	clcB	5	12.3833	0.449
0	0	ycaM	5	12.3833	0.451
0	0	yadI	5	12.3833	0.538
0	0	AG1	5	12.3833	0.504
0	0	ptsI	5	12.6333	0.451
0	0	clcB	5	12.6333	0.461
0	0	ycaM	5	12.6333	0.462
0	0	yadI	5	12.6333	0.545
0	0	AG1	5	12.6333	0.518
0	0	ptsI	5	12.8833	0.464
0	0	clcB	5	12.8833	0.476
0	0	ycaM	5	12.8833	0.474
0	0	yadI	5	12.8833	0.561
0	0	AG1	5	12.8833	0.538
0	0	ptsI	5	13.1333	0.463
0	0	clcB	5	13.1333	0.469
0	0	ycaM	5	13.1333	0.467
0	0	yadI	5	13.1333	0.557
0	0	AG1	5	13.1333	0.531
0	0	ptsI	5	13.3833	0.475
0	0	clcB	5	13.3833	0.478
0	0	ycaM	5	13.3833	0.472
0	0	yadI	5	13.3833	0.561
0	0	AG1	5	13.3833	0.536
0	0	ptsI	5	13.6333	0.48
0	0	clcB	5	13.6333	0.479

0	0	ycaM	5	13.6333	0.48
0	0	yadI	5	13.6333	0.574
0	0	AG1	5	13.6333	0.541
0	0	ptsI	5	13.8833	0.484
0	0	clcB	5	13.8833	0.478
0	0	ycaM	5	13.8833	0.474
0	0	yadI	5	13.8833	0.573
0	0	AG1	5	13.8833	0.543
0	0	ptsI	5	14.1333	0.497
0	0	clcB	5	14.1333	0.485
0	0	ycaM	5	14.1333	0.484
0	0	yadI	5	14.1333	0.582
0	0	AG1	5	14.1333	0.555
0	0	ptsI	5	14.3833	0.502
0	0	clcB	5	14.3833	0.508
0	0	ycaM	5	14.3833	0.502
0	0	yadI	5	14.3833	0.597
0	0	AG1	5	14.3833	0.566
0	0	ptsI	5	14.6333	0.508
0	0	clcB	5	14.6333	0.505
0	0	ycaM	5	14.6333	0.501
0	0	yadI	5	14.6333	0.599
0	0	AG1	5	14.6333	0.568
0	0	ptsI	5	14.8833	0.522
0	0	clcB	5	14.8833	0.508
0	0	ycaM	5	14.8833	0.501
0	0	yadI	5	14.8833	0.6
0	0	AG1	5	14.8833	0.571
0	0	ptsI	5	15.1333	0.541
0	0	clcB	5	15.1333	0.514
0	0	ycaM	5	15.1333	0.507
0	0	yadI	5	15.1333	0.609
0	0	AG1	5	15.1333	0.577
0	0	ptsI	5	15.3833	0.534
0	0	clcB	5	15.3833	0.516
0	0	ycaM	5	15.3833	0.515
0	0	yadI	5	15.3833	0.613
0	0	AG1	5	15.3833	0.579
0	0	ptsI	5	15.6333	0.543
0	0	clcB	5	15.6333	0.513
0	0	ycaM	5	15.6333	0.513
0	0	yadI	5	15.6333	0.611
0	0	AG1	5	15.6333	0.577
0	0	ptsI	5	15.8833	0.548
0	0	clcB	5	15.8833	0.513
0	0	ycaM	5	15.8833	0.517
0	0	yadI	5	15.8833	0.615
0	0	AG1	5	15.8833	0.577
0	0	ptsI	5	16.1333	0.545
0	0	clcB	5	16.1333	0.511
0	0	ycaM	5	16.1333	0.519
0	0	yadI	5	16.1333	0.611
0	0	AG1	5	16.1333	0.577

0	0	ptsI	5	16.3833	0.542
0	0	clcB	5	16.3833	0.508
0	0	ycaM	5	16.3833	0.517
0	0	yadI	5	16.3833	0.612
0	0	AG1	5	16.3833	0.581
0	0	ptsI	5	16.6333	0.551
0	0	clcB	5	16.6333	0.524
0	0	ycaM	5	16.6333	0.523
0	0	yadI	5	16.6333	0.62
0	0	AG1	5	16.6333	0.591
0	0	ptsI	5	16.8833	0.561
0	0	clcB	5	16.8833	0.535
0	0	ycaM	5	16.8833	0.535
0	0	yadI	5	16.8833	0.635
0	0	AG1	5	16.8833	0.601
0	0	ptsI	5	17.1333	0.565
0	0	clcB	5	17.1333	0.526
0	0	ycaM	5	17.1333	0.537
0	0	yadI	5	17.1333	0.63
0	0	AG1	5	17.1333	0.595
0	0	ptsI	5	17.3833	0.565
0	0	clcB	5	17.3833	0.525
0	0	ycaM	5	17.3833	0.524
0	0	yadI	5	17.3833	0.627
0	0	AG1	5	17.3833	0.594
0	0	ptsI	5	17.6333	0.566
0	0	clcB	5	17.6333	0.525
0	0	ycaM	5	17.6333	0.536
0	0	yadI	5	17.6333	0.632
0	0	AG1	5	17.6333	0.594
0	0	ptsI	5	17.8833	0.572
0	0	clcB	5	17.8833	0.525
0	0	ycaM	5	17.8833	0.544
0	0	yadI	5	17.8833	0.634
0	0	AG1	5	17.8833	0.594
0	0	ptsI	5	18.1333	0.576
0	0	clcB	5	18.1333	0.539
0	0	ycaM	5	18.1333	0.543
0	0	yadI	5	18.1333	0.646
0	0	AG1	5	18.1333	0.608
0	0	ptsI	5	18.3833	0.589
0	0	clcB	5	18.3833	0.527
0	0	ycaM	5	18.3833	0.545
0	0	yadI	5	18.3833	0.637
0	0	AG1	5	18.3833	0.601
0	0	ptsI	5	18.6333	0.582
0	0	clcB	5	18.6333	0.527
0	0	ycaM	5	18.6333	0.55
0	0	yadI	5	18.6333	0.636
0	0	AG1	5	18.6333	0.603
0	0	ptsI	5	18.8833	0.583
0	0	clcB	5	18.8833	0.534
0	0	ycaM	5	18.8833	0.546

0	0	yadI	5	18.8833	0.647
0	0	AG1	5	18.8833	0.609
0	0	ptsI	5	19.1333	0.595
0	0	clcB	5	19.1333	0.532
0	0	ycaM	5	19.1333	0.554
0	0	yadI	5	19.1333	0.645
0	0	AG1	5	19.1333	0.608
0	0	ptsI	5	19.3833	0.596
0	0	clcB	5	19.3833	0.534
0	0	ycaM	5	19.3833	0.559
0	0	yadI	5	19.3833	0.651
0	0	AG1	5	19.3833	0.61
0	0	ptsI	5	19.6333	0.595
0	0	clcB	5	19.6333	0.539
0	0	ycaM	5	19.6333	0.554
0	0	yadI	5	19.6333	0.654
0	0	AG1	5	19.6333	0.616
0	0	ptsI	5	19.8833	0.596
0	0	clcB	5	19.8833	0.536
0	0	ycaM	5	19.8833	0.557
0	0	yadI	5	19.8833	0.648
0	0	AG1	5	19.8833	0.61
0	0	ptsI	5	20.1333	0.608
0	0	clcB	5	20.1333	0.536
0	0	ycaM	5	20.1333	0.566
0	0	yadI	5	20.1333	0.65
0	0	AG1	5	20.1333	0.612
0	0	ptsI	5	20.3833	0.605
0	0	clcB	5	20.3833	0.538
0	0	ycaM	5	20.3833	0.572
0	0	yadI	5	20.3833	0.65
0	0	AG1	5	20.3833	0.613
0	0	ptsI	5	20.6333	0.604
0	0	clcB	5	20.6333	0.536
0	0	ycaM	5	20.6333	0.565
0	0	yadI	5	20.6333	0.654
0	0	AG1	5	20.6333	0.618
0	0	ptsI	5	20.8833	0.614
0	0	clcB	5	20.8833	0.538
0	0	ycaM	5	20.8833	0.572
0	0	yadI	5	20.8833	0.656
0	0	AG1	5	20.8833	0.619
0	0	ptsI	5	21.1333	0.613
0	0	clcB	5	21.1333	0.539
0	0	ycaM	5	21.1333	0.571
0	0	yadI	5	21.1333	0.656
0	0	AG1	5	21.1333	0.62
0	0	ptsI	5	21.3833	0.623
0	0	clcB	5	21.3833	0.544
0	0	ycaM	5	21.3833	0.575
0	0	yadI	5	21.3833	0.666
0	0	AG1	5	21.3833	0.626
0	0	ptsI	5	21.6333	0.613

0	0	clcB	5	21.6333	0.553
0	0	ycaM	5	21.6333	0.568
0	0	yadI	5	21.6333	0.673
0	0	AG1	5	21.6333	0.635
0	0	ptsI	5	21.8833	0.632
0	0	clcB	5	21.8833	0.556
0	0	ycaM	5	21.8833	0.583
0	0	yadI	5	21.8833	0.674
0	0	AG1	5	21.8833	0.637
0	0	ptsI	5	22.1333	0.623
0	0	clcB	5	22.1333	0.543
0	0	ycaM	5	22.1333	0.579
0	0	yadI	5	22.1333	0.665
0	0	AG1	5	22.1333	0.621
0	0	ptsI	5	22.3833	0.628
0	0	clcB	5	22.3833	0.546
0	0	ycaM	5	22.3833	0.58
0	0	yadI	5	22.3833	0.664
0	0	AG1	5	22.3833	0.623
0	0	ptsI	5	22.6333	0.623
0	0	clcB	5	22.6333	0.555
0	0	ycaM	5	22.6333	0.578
0	0	yadI	5	22.6333	0.681
0	0	AG1	5	22.6333	0.639
0	0	ptsI	5	22.8833	0.626
0	0	clcB	5	22.8833	0.555
0	0	ycaM	5	22.8833	0.578
0	0	yadI	5	22.8833	0.678
0	0	AG1	5	22.8833	0.635
0	0	ptsI	5	23.1333	0.626
0	0	clcB	5	23.1333	0.558
0	0	ycaM	5	23.1333	0.582
0	0	yadI	5	23.1333	0.676
0	0	AG1	5	23.1333	0.636
0	0	ptsI	5	23.3833	0.633
0	0	clcB	5	23.3833	0.548
0	0	ycaM	5	23.3833	0.589
0	0	yadI	5	23.3833	0.685
0	0	AG1	5	23.3833	0.634
0	0	ptsI	5	23.6333	0.631
0	0	clcB	5	23.6333	0.549
0	0	ycaM	5	23.6333	0.59
0	0	yadI	5	23.6333	0.686
0	0	AG1	5	23.6333	0.633
0	0	ptsI	5	23.8833	0.632
0	0	clcB	5	23.8833	0.55
0	0	ycaM	5	23.8833	0.589
0	0	yadI	5	23.8833	0.686
0	0	AG1	5	23.8833	0.633
0	0	ptsI	5	24.1333	0.647
0	0	clcB	5	24.1333	0.554
0	0	ycaM	5	24.1333	0.593
0	0	yadI	5	24.1333	0.689

0	0	AG1	5	24.1333	0.644
0	0	ptsI	5	24.3833	0.634
0	0	clcB	5	24.3833	0.546
0	0	ycaM	5	24.3833	0.587
0	0	yadI	5	24.3833	0.686
0	0	AG1	5	24.3833	0.63
0.05	0	ptsI	1	0	0.234
0.05	0	clcB	1	0	0.237
0.05	0	ycaM	1	0	0.223
0.05	0	yadI	1	0	0.253
0.05	0	AG1	1	0	0.255
0.05	0	ptsI	1	0.25	0.226
0.05	0	clcB	1	0.25	0.226
0.05	0	ycaM	1	0.25	0.214
0.05	0	yadI	1	0.25	0.245
0.05	0	AG1	1	0.25	0.251
0.05	0	ptsI	1	0.5	0.223
0.05	0	clcB	1	0.5	0.223
0.05	0	ycaM	1	0.5	0.211
0.05	0	yadI	1	0.5	0.242
0.05	0	AG1	1	0.5	0.246
0.05	0	ptsI	1	0.75	0.223
0.05	0	clcB	1	0.75	0.221
0.05	0	ycaM	1	0.75	0.21
0.05	0	yadI	1	0.75	0.238
0.05	0	AG1	1	0.75	0.246
0.05	0	ptsI	1	1	0.23
0.05	0	clcB	1	1	0.222
0.05	0	ycaM	1	1	0.213
0.05	0	yadI	1	1	0.242
0.05	0	AG1	1	1	0.245
0.05	0	ptsI	1	1.25	0.232
0.05	0	clcB	1	1.25	0.218
0.05	0	ycaM	1	1.25	0.211
0.05	0	yadI	1	1.25	0.245
0.05	0	AG1	1	1.25	0.247
0.05	0	ptsI	1	1.5	0.238
0.05	0	clcB	1	1.5	0.221
0.05	0	ycaM	1	1.5	0.217
0.05	0	yadI	1	1.5	0.251
0.05	0	AG1	1	1.5	0.249
0.05	0	ptsI	1	1.75	0.241
0.05	0	clcB	1	1.75	0.218
0.05	0	ycaM	1	1.75	0.22
0.05	0	yadI	1	1.75	0.255
0.05	0	AG1	1	1.75	0.256
0.05	0	ptsI	1	2	0.246
0.05	0	clcB	1	2	0.22
0.05	0	ycaM	1	2	0.226
0.05	0	yadI	1	2	0.265
0.05	0	AG1	1	2	0.264
0.05	0	ptsI	1	2.25	0.252
0.05	0	clcB	1	2.25	0.22

0.05	0	ycaM	1	2.25	0.229
0.05	0	yadI	1	2.25	0.272
0.05	0	AG1	1	2.25	0.272
0.05	0	ptsI	1	2.5	0.258
0.05	0	clcB	1	2.5	0.223
0.05	0	ycaM	1	2.5	0.236
0.05	0	yadI	1	2.5	0.28
0.05	0	AG1	1	2.5	0.28
0.05	0	ptsI	1	2.75	0.264
0.05	0	clcB	1	2.75	0.223
0.05	0	ycaM	1	2.75	0.24
0.05	0	yadI	1	2.75	0.294
0.05	0	AG1	1	2.75	0.291
0.05	0	ptsI	1	3	0.27
0.05	0	clcB	1	3	0.227
0.05	0	ycaM	1	3	0.253
0.05	0	yadI	1	3	0.308
0.05	0	AG1	1	3	0.301
0.05	0	ptsI	1	3.25	0.278
0.05	0	clcB	1	3.25	0.228
0.05	0	ycaM	1	3.25	0.255
0.05	0	yadI	1	3.25	0.328
0.05	0	AG1	1	3.25	0.312
0.05	0	ptsI	1	3.5	0.286
0.05	0	clcB	1	3.5	0.23
0.05	0	ycaM	1	3.5	0.262
0.05	0	yadI	1	3.5	0.348
0.05	0	AG1	1	3.5	0.318
0.05	0	ptsI	1	3.75	0.298
0.05	0	clcB	1	3.75	0.234
0.05	0	ycaM	1	3.75	0.274
0.05	0	yadI	1	3.75	0.374
0.05	0	AG1	1	3.75	0.333
0.05	0	ptsI	1	4	0.308
0.05	0	clcB	1	4	0.238
0.05	0	ycaM	1	4	0.29
0.05	0	yadI	1	4	0.398
0.05	0	AG1	1	4	0.331
0.05	0	ptsI	1	4.25	0.321
0.05	0	clcB	1	4.25	0.24
0.05	0	ycaM	1	4.25	0.301
0.05	0	yadI	1	4.25	0.42
0.05	0	AG1	1	4.25	0.359
0.05	0	ptsI	1	4.5	0.325
0.05	0	clcB	1	4.5	0.243
0.05	0	ycaM	1	4.5	0.311
0.05	0	yadI	1	4.5	0.442
0.05	0	AG1	1	4.5	0.373
0.05	0	ptsI	1	4.75	0.334
0.05	0	clcB	1	4.75	0.247
0.05	0	ycaM	1	4.75	0.32
0.05	0	yadI	1	4.75	0.459
0.05	0	AG1	1	4.75	0.386

0.05	0	ptsI	1	5	0.343
0.05	0	clcB	1	5	0.254
0.05	0	ycaM	1	5	0.333
0.05	0	yadI	1	5	0.479
0.05	0	AG1	1	5	0.407
0.05	0	ptsI	1	5.25	0.353
0.05	0	clcB	1	5.25	0.258
0.05	0	ycaM	1	5.25	0.344
0.05	0	yadI	1	5.25	0.489
0.05	0	AG1	1	5.25	0.456
0.05	0	ptsI	1	5.5	0.362
0.05	0	clcB	1	5.5	0.268
0.05	0	ycaM	1	5.5	0.357
0.05	0	yadI	1	5.5	0.493
0.05	0	AG1	1	5.5	0.416
0.05	0	ptsI	1	5.75	0.37
0.05	0	clcB	1	5.75	0.266
0.05	0	ycaM	1	5.75	0.368
0.05	0	yadI	1	5.75	0.508
0.05	0	AG1	1	5.75	0.424
0.05	0	ptsI	1	6	0.39
0.05	0	clcB	1	6	0.274
0.05	0	ycaM	1	6	0.377
0.05	0	yadI	1	6	0.517
0.05	0	AG1	1	6	0.436
0.05	0	ptsI	1	6.25	0.405
0.05	0	clcB	1	6.25	0.285
0.05	0	ycaM	1	6.25	0.404
0.05	0	yadI	1	6.25	0.533
0.05	0	AG1	1	6.25	0.459
0.05	0	ptsI	1	6.5	0.418
0.05	0	clcB	1	6.5	0.289
0.05	0	ycaM	1	6.5	0.41
0.05	0	yadI	1	6.5	0.538
0.05	0	AG1	1	6.5	0.462
0.05	0	ptsI	1	6.75	0.419
0.05	0	clcB	1	6.75	0.297
0.05	0	ycaM	1	6.75	0.419
0.05	0	yadI	1	6.75	0.56
0.05	0	AG1	1	6.75	0.468
0.05	0	ptsI	1	7	0.426
0.05	0	clcB	1	7	0.306
0.05	0	ycaM	1	7	0.429
0.05	0	yadI	1	7	0.573
0.05	0	AG1	1	7	0.482
0.05	0	ptsI	1	7.25	0.436
0.05	0	clcB	1	7.25	0.316
0.05	0	ycaM	1	7.25	0.44
0.05	0	yadI	1	7.25	0.579
0.05	0	AG1	1	7.25	0.492
0.05	0	ptsI	1	7.5	0.443
0.05	0	clcB	1	7.5	0.327
0.05	0	ycaM	1	7.5	0.446

0.05	0	yadI	1	7.5	0.593
0.05	0	AG1	1	7.5	0.504
0.05	0	ptsI	1	7.75	0.452
0.05	0	clcB	1	7.75	0.339
0.05	0	ycaM	1	7.75	0.455
0.05	0	yadI	1	7.75	0.606
0.05	0	AG1	1	7.75	0.515
0.05	0	ptsI	1	8	0.478
0.05	0	clcB	1	8	0.348
0.05	0	ycaM	1	8	0.463
0.05	0	yadI	1	8	0.617
0.05	0	AG1	1	8	0.523
0.05	0	ptsI	1	8.25	0.477
0.05	0	clcB	1	8.25	0.36
0.05	0	ycaM	1	8.25	0.469
0.05	0	yadI	1	8.25	0.629
0.05	0	AG1	1	8.25	0.534
0.05	0	ptsI	1	8.5	0.493
0.05	0	clcB	1	8.5	0.368
0.05	0	ycaM	1	8.5	0.48
0.05	0	yadI	1	8.5	0.646
0.05	0	AG1	1	8.5	0.551
0.05	0	ptsI	1	8.75	0.495
0.05	0	clcB	1	8.75	0.375
0.05	0	ycaM	1	8.75	0.482
0.05	0	yadI	1	8.75	0.65
0.05	0	AG1	1	8.75	0.57
0.05	0	ptsI	1	9	0.507
0.05	0	clcB	1	9	0.382
0.05	0	ycaM	1	9	0.487
0.05	0	yadI	1	9	0.661
0.05	0	AG1	1	9	0.588
0.05	0	ptsI	1	9.25	0.522
0.05	0	clcB	1	9.25	0.393
0.05	0	ycaM	1	9.25	0.493
0.05	0	yadI	1	9.25	0.678
0.05	0	AG1	1	9.25	0.618
0.05	0	ptsI	1	9.5	0.551
0.05	0	clcB	1	9.5	0.406
0.05	0	ycaM	1	9.5	0.509
0.05	0	yadI	1	9.5	0.714
0.05	0	AG1	1	9.5	0.614
0.05	0	ptsI	1	9.75	0.548
0.05	0	clcB	1	9.75	0.41
0.05	0	ycaM	1	9.75	0.515
0.05	0	yadI	1	9.75	0.714
0.05	0	AG1	1	9.75	0.623
0.05	0	ptsI	1	10	0.569
0.05	0	clcB	1	10	0.418
0.05	0	ycaM	1	10	0.52
0.05	0	yadI	1	10	0.723
0.05	0	AG1	1	10	0.625
0.05	0	ptsI	1	10.25	0.575

0.05	0	clcB	1	10.25	0.422
0.05	0	ycaM	1	10.25	0.531
0.05	0	yadI	1	10.25	0.726
0.05	0	AG1	1	10.25	0.628
0.05	0	ptsI	1	10.5	0.604
0.05	0	clcB	1	10.5	0.425
0.05	0	ycaM	1	10.5	0.539
0.05	0	yadI	1	10.5	0.732
0.05	0	AG1	1	10.5	0.656
0.05	0	ptsI	1	10.75	0.653
0.05	0	clcB	1	10.75	0.44
0.05	0	ycaM	1	10.75	0.551
0.05	0	yadI	1	10.75	0.749
0.05	0	AG1	1	10.75	0.625
0.05	0	ptsI	1	11	0.634
0.05	0	clcB	1	11	0.462
0.05	0	ycaM	1	11	0.572
0.05	0	yadI	1	11	0.762
0.05	0	AG1	1	11	0.632
0.05	0	ptsI	1	11.25	0.659
0.05	0	clcB	1	11.25	0.465
0.05	0	ycaM	1	11.25	0.563
0.05	0	yadI	1	11.25	0.765
0.05	0	AG1	1	11.25	0.621
0.05	0	ptsI	1	11.5	0.689
0.05	0	clcB	1	11.5	0.481
0.05	0	ycaM	1	11.5	0.56
0.05	0	yadI	1	11.5	0.779
0.05	0	AG1	1	11.5	0.622
0.05	0	ptsI	1	11.75	0.721
0.05	0	clcB	1	11.75	0.497
0.05	0	ycaM	1	11.75	0.571
0.05	0	yadI	1	11.75	0.809
0.05	0	AG1	1	11.75	0.636
0.05	0	ptsI	1	12	0.71
0.05	0	clcB	1	12	0.497
0.05	0	ycaM	1	12	0.58
0.05	0	yadI	1	12	0.796
0.05	0	AG1	1	12	0.644
0.05	0	ptsI	1	12.25	0.717
0.05	0	clcB	1	12.25	0.517
0.05	0	ycaM	1	12.25	0.58
0.05	0	yadI	1	12.25	0.808
0.05	0	AG1	1	12.25	0.669
0.05	0	ptsI	1	12.5	0.733
0.05	0	clcB	1	12.5	0.519
0.05	0	ycaM	1	12.5	0.584
0.05	0	yadI	1	12.5	0.823
0.05	0	AG1	1	12.5	0.668
0.05	0	ptsI	1	12.75	0.745
0.05	0	clcB	1	12.75	0.519
0.05	0	ycaM	1	12.75	0.591
0.05	0	yadI	1	12.75	0.818

0.05	0	AG1	1	12.75	0.698
0.05	0	ptsI	1	13	0.76
0.05	0	clcB	1	13	0.526
0.05	0	ycaM	1	13	0.598
0.05	0	yadI	1	13	0.825
0.05	0	AG1	1	13	0.7
0.05	0	ptsI	1	13.25	0.763
0.05	0	clcB	1	13.25	0.543
0.05	0	ycaM	1	13.25	0.606
0.05	0	yadI	1	13.25	0.83
0.05	0	AG1	1	13.25	0.705
0.05	0	ptsI	1	13.5	0.773
0.05	0	clcB	1	13.5	0.551
0.05	0	ycaM	1	13.5	0.611
0.05	0	yadI	1	13.5	0.847
0.05	0	AG1	1	13.5	0.726
0.05	0	ptsI	1	13.75	0.783
0.05	0	clcB	1	13.75	0.562
0.05	0	ycaM	1	13.75	0.61
0.05	0	yadI	1	13.75	0.859
0.05	0	AG1	1	13.75	0.72
0.05	0	ptsI	1	14	0.808
0.05	0	clcB	1	14	0.563
0.05	0	ycaM	1	14	0.613
0.05	0	yadI	1	14	0.871
0.05	0	AG1	1	14	0.754
0.05	0	ptsI	1	14.25	0.81
0.05	0	clcB	1	14.25	0.566
0.05	0	ycaM	1	14.25	0.622
0.05	0	yadI	1	14.25	0.864
0.05	0	AG1	1	14.25	0.748
0.05	0	ptsI	1	14.5	0.816
0.05	0	clcB	1	14.5	0.569
0.05	0	ycaM	1	14.5	0.618
0.05	0	yadI	1	14.5	0.872
0.05	0	AG1	1	14.5	0.757
0.05	0	ptsI	1	14.75	0.821
0.05	0	clcB	1	14.75	0.579
0.05	0	ycaM	1	14.75	0.619
0.05	0	yadI	1	14.75	0.88
0.05	0	AG1	1	14.75	0.768
0.05	0	ptsI	1	15	0.811
0.05	0	clcB	1	15	0.59
0.05	0	ycaM	1	15	0.62
0.05	0	yadI	1	15	0.876
0.05	0	AG1	1	15	0.771
0.05	0	ptsI	1	15.25	0.841
0.05	0	clcB	1	15.25	0.616
0.05	0	ycaM	1	15.25	0.645
0.05	0	yadI	1	15.25	0.873
0.05	0	AG1	1	15.25	0.782
0.05	0	ptsI	1	15.5	0.838
0.05	0	clcB	1	15.5	0.648

0.05	0	ycaM	1	15.5	0.659
0.05	0	yadI	1	15.5	0.881
0.05	0	AG1	1	15.5	0.761
0.05	0	ptsI	1	15.75	0.829
0.05	0	clcB	1	15.75	0.636
0.05	0	ycaM	1	15.75	0.644
0.05	0	yadI	1	15.75	0.889
0.05	0	AG1	1	15.75	0.784
0.05	0	ptsI	1	16	0.831
0.05	0	clcB	1	16	0.676
0.05	0	ycaM	1	16	0.653
0.05	0	yadI	1	16	0.887
0.05	0	AG1	1	16	0.776
0.05	0	ptsI	1	16.25	0.848
0.05	0	clcB	1	16.25	0.653
0.05	0	ycaM	1	16.25	0.652
0.05	0	yadI	1	16.25	0.88
0.05	0	AG1	1	16.25	0.81
0.05	0	ptsI	1	16.5	0.856
0.05	0	clcB	1	16.5	0.67
0.05	0	ycaM	1	16.5	0.678
0.05	0	yadI	1	16.5	0.884
0.05	0	AG1	1	16.5	0.789
0.05	0	ptsI	1	17.0667	0.906
0.05	0	clcB	1	17.0667	0.662
0.05	0	ycaM	1	17.0667	0.744
0.05	0	yadI	1	17.0667	0.952
0.05	0	AG1	1	17.0667	0.811
0.05	0	ptsI	1	17.3167	0.81
0.05	0	clcB	1	17.3167	0.642
0.05	0	ycaM	1	17.3167	0.686
0.05	0	yadI	1	17.3167	0.914
0.05	0	AG1	1	17.3167	0.792
0.05	0	ptsI	1	17.5667	0.823
0.05	0	clcB	1	17.5667	0.658
0.05	0	ycaM	1	17.5667	0.707
0.05	0	yadI	1	17.5667	0.9
0.05	0	AG1	1	17.5667	0.8
0.05	0	ptsI	1	17.8167	0.853
0.05	0	clcB	1	17.8167	0.736
0.05	0	ycaM	1	17.8167	0.697
0.05	0	yadI	1	17.8167	0.909
0.05	0	AG1	1	17.8167	0.832
0.05	0	ptsI	1	18.0667	0.85
0.05	0	clcB	1	18.0667	0.644
0.05	0	ycaM	1	18.0667	0.704
0.05	0	yadI	1	18.0667	0.909
0.05	0	AG1	1	18.0667	0.779
0.05	0	ptsI	1	18.3167	0.853
0.05	0	clcB	1	18.3167	0.668
0.05	0	ycaM	1	18.3167	0.712
0.05	0	yadI	1	18.3167	0.917
0.05	0	AG1	1	18.3167	0.802

0.05	0	ptsI	1	18.5667	0.866
0.05	0	clcB	1	18.5667	0.697
0.05	0	ycaM	1	18.5667	0.725
0.05	0	yadI	1	18.5667	0.916
0.05	0	AG1	1	18.5667	0.822
0.05	0	ptsI	1	18.8167	0.872
0.05	0	clcB	1	18.8167	0.711
0.05	0	ycaM	1	18.8167	0.735
0.05	0	yadI	1	18.8167	0.918
0.05	0	AG1	1	18.8167	0.826
0.05	0	ptsI	1	19.0667	0.88
0.05	0	clcB	1	19.0667	0.708
0.05	0	ycaM	1	19.0667	0.733
0.05	0	yadI	1	19.0667	0.93
0.05	0	AG1	1	19.0667	0.823
0.05	0	ptsI	1	19.3167	0.887
0.05	0	clcB	1	19.3167	0.709
0.05	0	ycaM	1	19.3167	0.73
0.05	0	yadI	1	19.3167	0.931
0.05	0	AG1	1	19.3167	0.819
0.05	0	ptsI	1	19.5667	0.894
0.05	0	clcB	1	19.5667	0.742
0.05	0	ycaM	1	19.5667	0.748
0.05	0	yadI	1	19.5667	0.934
0.05	0	AG1	1	19.5667	0.838
0.05	0	ptsI	1	19.8167	0.9
0.05	0	clcB	1	19.8167	0.731
0.05	0	ycaM	1	19.8167	0.744
0.05	0	yadI	1	19.8167	0.937
0.05	0	AG1	1	19.8167	0.835
0.05	0	ptsI	1	20.0667	0.915
0.05	0	clcB	1	20.0667	0.743
0.05	0	ycaM	1	20.0667	0.747
0.05	0	yadI	1	20.0667	0.94
0.05	0	AG1	1	20.0667	0.84
0.05	0	ptsI	1	20.3167	0.917
0.05	0	clcB	1	20.3167	0.737
0.05	0	ycaM	1	20.3167	0.752
0.05	0	yadI	1	20.3167	0.95
0.05	0	AG1	1	20.3167	0.831
0.05	0	ptsI	1	20.5667	0.921
0.05	0	clcB	1	20.5667	0.726
0.05	0	ycaM	1	20.5667	0.748
0.05	0	yadI	1	20.5667	0.947
0.05	0	AG1	1	20.5667	0.826
0.05	0	ptsI	1	20.8167	0.936
0.05	0	clcB	1	20.8167	0.728
0.05	0	ycaM	1	20.8167	0.749
0.05	0	yadI	1	20.8167	0.953
0.05	0	AG1	1	20.8167	0.817
0.05	0	ptsI	1	21.0667	0.952
0.05	0	clcB	1	21.0667	0.73
0.05	0	ycaM	1	21.0667	0.764

0.05	0	yadI	1	21.0667	0.952
0.05	0	AG1	1	21.0667	0.811
0.05	0	ptsI	1	21.3167	0.956
0.05	0	clcB	1	21.3167	0.738
0.05	0	ycaM	1	21.3167	0.787
0.05	0	yadI	1	21.3167	0.959
0.05	0	AG1	1	21.3167	0.82
0.05	0	ptsI	1	21.5667	0.972
0.05	0	clcB	1	21.5667	0.735
0.05	0	ycaM	1	21.5667	0.769
0.05	0	yadI	1	21.5667	0.961
0.05	0	AG1	1	21.5667	0.823
0.05	0	ptsI	1	21.8167	0.985
0.05	0	clcB	1	21.8167	0.738
0.05	0	ycaM	1	21.8167	0.775
0.05	0	yadI	1	21.8167	0.966
0.05	0	AG1	1	21.8167	0.82
0.05	0	ptsI	1	22.0667	1.007
0.05	0	clcB	1	22.0667	0.745
0.05	0	ycaM	1	22.0667	0.792
0.05	0	yadI	1	22.0667	0.97
0.05	0	AG1	1	22.0667	0.82
0.05	0	ptsI	1	22.9333	1.125
0.05	0	clcB	1	22.9333	0.701
0.05	0	ycaM	1	22.9333	0.744
0.05	0	yadI	1	22.9333	1.088
0.05	0	AG1	1	22.9333	0.812
0.05	0	ptsI	1	23.1833	1.088
0.05	0	clcB	1	23.1833	0.665
0.05	0	ycaM	1	23.1833	0.716
0.05	0	yadI	1	23.1833	1.039
0.05	0	AG1	1	23.1833	0.776
0.05	0	ptsI	1	23.4333	1.073
0.05	0	clcB	1	23.4333	0.706
0.05	0	ycaM	1	23.4333	0.745
0.05	0	yadI	1	23.4333	1.035
0.05	0	AG1	1	23.4333	0.779
0.05	0	ptsI	1	23.6833	1.068
0.05	0	clcB	1	23.6833	0.685
0.05	0	ycaM	1	23.6833	0.739
0.05	0	yadI	1	23.6833	1.043
0.05	0	AG1	1	23.6833	0.802
0.05	0	ptsI	1	23.9333	1.066
0.05	0	clcB	1	23.9333	0.674
0.05	0	ycaM	1	23.9333	0.732
0.05	0	yadI	1	23.9333	1.042
0.05	0	AG1	1	23.9333	0.805
0.05	0	ptsI	1	24.1833	1.067
0.05	0	clcB	1	24.1833	0.686
0.05	0	ycaM	1	24.1833	0.748
0.05	0	yadI	1	24.1833	1.052
0.05	0	AG1	1	24.1833	0.807
0.05	0	ptsI	1	24.4333	1.074

0.05	0	clcB	1	24.4333	0.696
0.05	0	ycaM	1	24.4333	0.751
0.05	0	yadI	1	24.4333	1.051
0.05	0	AG1	1	24.4333	0.808
0.05	0	ptsI	1	24.6833	1.066
0.05	0	clcB	1	24.6833	0.699
0.05	0	ycaM	1	24.6833	0.75
0.05	0	yadI	1	24.6833	1.053
0.05	0	AG1	1	24.6833	0.814
0.05	0	ptsI	1	24.9333	1.07
0.05	0	clcB	1	24.9333	0.7
0.05	0	ycaM	1	24.9333	0.755
0.05	0	yadI	1	24.9333	1.065
0.05	0	AG1	1	24.9333	0.832
0.05	0	ptsI	1	25.1833	1.072
0.05	0	clcB	1	25.1833	0.706
0.05	0	ycaM	1	25.1833	0.753
0.05	0	yadI	1	25.1833	1.063
0.05	0	AG1	1	25.1833	0.823
0.05	0	ptsI	1	25.4333	1.079
0.05	0	clcB	1	25.4333	0.715
0.05	0	ycaM	1	25.4333	0.771
0.05	0	yadI	1	25.4333	1.074
0.05	0	AG1	1	25.4333	0.836
0.05	0	ptsI	1	25.6833	1.08
0.05	0	clcB	1	25.6833	0.719
0.05	0	ycaM	1	25.6833	0.775
0.05	0	yadI	1	25.6833	1.074
0.05	0	AG1	1	25.6833	0.836
0.05	0	ptsI	1	25.9333	1.078
0.05	0	clcB	1	25.9333	0.731
0.05	0	ycaM	1	25.9333	0.778
0.05	0	yadI	1	25.9333	1.073
0.05	0	AG1	1	25.9333	0.841
0.05	0	ptsI	1	26.1833	1.088
0.05	0	clcB	1	26.1833	0.746
0.05	0	ycaM	1	26.1833	0.793
0.05	0	yadI	1	26.1833	1.081
0.05	0	AG1	1	26.1833	0.851
0.05	0	ptsI	1	26.4333	1.087
0.05	0	clcB	1	26.4333	0.752
0.05	0	ycaM	1	26.4333	0.788
0.05	0	yadI	1	26.4333	1.08
0.05	0	AG1	1	26.4333	0.845
0.05	0	ptsI	1	26.6833	1.091
0.05	0	clcB	1	26.6833	0.754
0.05	0	ycaM	1	26.6833	0.789
0.05	0	yadI	1	26.6833	1.087
0.05	0	AG1	1	26.6833	0.847
0.05	0	ptsI	1	26.9333	1.092
0.05	0	clcB	1	26.9333	0.762
0.05	0	ycaM	1	26.9333	0.797
0.05	0	yadI	1	26.9333	1.087

0.05	0	AG1	1	26.9333	0.85
0.05	0	ptsI	1	27.1833	1.095
0.05	0	clcB	1	27.1833	0.768
0.05	0	ycaM	1	27.1833	0.802
0.05	0	yadI	1	27.1833	1.094
0.05	0	AG1	1	27.1833	0.857
0.05	0	ptsI	1	27.4333	1.097
0.05	0	clcB	1	27.4333	0.761
0.05	0	ycaM	1	27.4333	0.799
0.05	0	yadI	1	27.4333	1.097
0.05	0	AG1	1	27.4333	0.85
0.05	0	ptsI	1	27.6833	1.103
0.05	0	clcB	1	27.6833	0.776
0.05	0	ycaM	1	27.6833	0.806
0.05	0	yadI	1	27.6833	1.104
0.05	0	AG1	1	27.6833	0.861
0.05	0	ptsI	1	27.9333	1.105
0.05	0	clcB	1	27.9333	0.772
0.05	0	ycaM	1	27.9333	0.8
0.05	0	yadI	1	27.9333	1.101
0.05	0	AG1	1	27.9333	0.853
0.05	0	ptsI	1	28.1833	1.11
0.05	0	clcB	1	28.1833	0.791
0.05	0	ycaM	1	28.1833	0.812
0.05	0	yadI	1	28.1833	1.108
0.05	0	AG1	1	28.1833	0.865
0.05	0	ptsI	1	28.4333	1.114
0.05	0	clcB	1	28.4333	0.788
0.05	0	ycaM	1	28.4333	0.812
0.05	0	yadI	1	28.4333	1.104
0.05	0	AG1	1	28.4333	0.868
0.05	0	ptsI	1	28.6833	1.118
0.05	0	clcB	1	28.6833	0.788
0.05	0	ycaM	1	28.6833	0.816
0.05	0	yadI	1	28.6833	1.109
0.05	0	AG1	1	28.6833	0.868
0.05	0	ptsI	1	28.9333	1.122
0.05	0	clcB	1	28.9333	0.801
0.05	0	ycaM	1	28.9333	0.82
0.05	0	yadI	1	28.9333	1.117
0.05	0	AG1	1	28.9333	0.875
0.05	0	ptsI	1	29.1833	1.123
0.05	0	clcB	1	29.1833	0.786
0.05	0	ycaM	1	29.1833	0.817
0.05	0	yadI	1	29.1833	1.117
0.05	0	AG1	1	29.1833	0.873
0.05	0	ptsI	1	29.4333	1.126
0.05	0	clcB	1	29.4333	0.798
0.05	0	ycaM	1	29.4333	0.818
0.05	0	yadI	1	29.4333	1.119
0.05	0	AG1	1	29.4333	0.876
0.05	0	ptsI	1	29.6833	1.134
0.05	0	clcB	1	29.6833	0.795

0.05	0	ycaM	1	29.6833	0.822
0.05	0	yadI	1	29.6833	1.124
0.05	0	AG1	1	29.6833	0.878
0.05	0	ptsI	1	29.9333	1.141
0.05	0	clcB	1	29.9333	0.798
0.05	0	ycaM	1	29.9333	0.825
0.05	0	yadI	1	29.9333	1.131
0.05	0	AG1	1	29.9333	0.88
0.05	0	ptsI	1	30.1833	1.147
0.05	0	clcB	1	30.1833	0.796
0.05	0	ycaM	1	30.1833	0.824
0.05	0	yadI	1	30.1833	1.133
0.05	0	AG1	1	30.1833	0.88
0.05	0	ptsI	1	30.4333	1.152
0.05	0	clcB	1	30.4333	0.805
0.05	0	ycaM	1	30.4333	0.829
0.05	0	yadI	1	30.4333	1.137
0.05	0	AG1	1	30.4333	0.883
0.05	0	ptsI	1	30.6833	1.161
0.05	0	clcB	1	30.6833	0.811
0.05	0	ycaM	1	30.6833	0.826
0.05	0	yadI	1	30.6833	1.146
0.05	0	AG1	1	30.6833	0.882
0.05	0	ptsI	1	30.9333	1.164
0.05	0	clcB	1	30.9333	0.815
0.05	0	ycaM	1	30.9333	0.833
0.05	0	yadI	1	30.9333	1.151
0.05	0	AG1	1	30.9333	0.889
0.05	0	ptsI	1	31.1833	1.172
0.05	0	clcB	1	31.1833	0.804
0.05	0	ycaM	1	31.1833	0.832
0.05	0	yadI	1	31.1833	1.152
0.05	0	AG1	1	31.1833	0.887
0.05	0	ptsI	1	31.4333	1.183
0.05	0	clcB	1	31.4333	0.804
0.05	0	ycaM	1	31.4333	0.843
0.05	0	yadI	1	31.4333	1.158
0.05	0	AG1	1	31.4333	0.892
0.05	0	ptsI	1	31.6833	1.191
0.05	0	clcB	1	31.6833	0.809
0.05	0	ycaM	1	31.6833	0.844
0.05	0	yadI	1	31.6833	1.16
0.05	0	AG1	1	31.6833	0.893
0.05	0	ptsI	1	31.9333	1.204
0.05	0	clcB	1	31.9333	0.807
0.05	0	ycaM	1	31.9333	0.847
0.05	0	yadI	1	31.9333	1.163
0.05	0	AG1	1	31.9333	0.896
0.05	0	ptsI	1	32.1833	1.207
0.05	0	clcB	1	32.1833	0.808
0.05	0	ycaM	1	32.1833	0.849
0.05	0	yadI	1	32.1833	1.164
0.05	0	AG1	1	32.1833	0.897

0.05	0	ptsI	1	32.4333	1.227
0.05	0	clcB	1	32.4333	0.805
0.05	0	ycaM	1	32.4333	0.849
0.05	0	yadI	1	32.4333	1.164
0.05	0	AG1	1	32.4333	0.897
0.05	0	ptsI	1	32.6833	1.223
0.05	0	clcB	1	32.6833	0.816
0.05	0	ycaM	1	32.6833	0.856
0.05	0	yadI	1	32.6833	1.17
0.05	0	AG1	1	32.6833	0.899
0.05	0	ptsI	1	32.9333	1.236
0.05	0	clcB	1	32.9333	0.824
0.05	0	ycaM	1	32.9333	0.858
0.05	0	yadI	1	32.9333	1.174
0.05	0	AG1	1	32.9333	0.901
0.05	0	ptsI	1	33.1833	1.245
0.05	0	clcB	1	33.1833	0.826
0.05	0	ycaM	1	33.1833	0.857
0.05	0	yadI	1	33.1833	1.179
0.05	0	AG1	1	33.1833	0.901
0.05	0	ptsI	1	33.4333	1.254
0.05	0	clcB	1	33.4333	0.838
0.05	0	ycaM	1	33.4333	0.863
0.05	0	yadI	1	33.4333	1.183
0.05	0	AG1	1	33.4333	0.907
0.05	0	ptsI	1	33.6833	1.263
0.05	0	clcB	1	33.6833	0.832
0.05	0	ycaM	1	33.6833	0.861
0.05	0	yadI	1	33.6833	1.187
0.05	0	AG1	1	33.6833	0.902
0.05	0	ptsI	1	33.9333	1.273
0.05	0	clcB	1	33.9333	0.846
0.05	0	ycaM	1	33.9333	0.872
0.05	0	yadI	1	33.9333	1.195
0.05	0	AG1	1	33.9333	0.911
0.05	0	ptsI	1	34.1833	1.282
0.05	0	clcB	1	34.1833	0.842
0.05	0	ycaM	1	34.1833	0.869
0.05	0	yadI	1	34.1833	1.2
0.05	0	AG1	1	34.1833	0.903
0.05	0	ptsI	1	34.4333	1.298
0.05	0	clcB	1	34.4333	0.842
0.05	0	ycaM	1	34.4333	0.873
0.05	0	yadI	1	34.4333	1.204
0.05	0	AG1	1	34.4333	0.912
0.05	0	ptsI	1	34.6833	1.302
0.05	0	clcB	1	34.6833	0.842
0.05	0	ycaM	1	34.6833	0.873
0.05	0	yadI	1	34.6833	1.211
0.05	0	AG1	1	34.6833	0.918
0.05	0	ptsI	1	34.9333	1.318
0.05	0	clcB	1	34.9333	0.859
0.05	0	ycaM	1	34.9333	0.874

0.05	0	yadI	1	34.9333	1.216
0.05	0	AG1	1	34.9333	0.918
0.05	0	ptsI	1	35.1833	1.33
0.05	0	clcB	1	35.1833	0.854
0.05	0	ycaM	1	35.1833	0.874
0.05	0	yadI	1	35.1833	1.221
0.05	0	AG1	1	35.1833	0.917
0.05	0	ptsI	1	35.4333	1.338
0.05	0	clcB	1	35.4333	0.861
0.05	0	ycaM	1	35.4333	0.873
0.05	0	yadI	1	35.4333	1.226
0.05	0	AG1	1	35.4333	0.924
0.05	0	ptsI	1	35.6833	1.347
0.05	0	clcB	1	35.6833	0.866
0.05	0	ycaM	1	35.6833	0.874
0.05	0	yadI	1	35.6833	1.233
0.05	0	AG1	1	35.6833	0.93
0.05	0	ptsI	1	35.9333	1.355
0.05	0	clcB	1	35.9333	0.862
0.05	0	ycaM	1	35.9333	0.869
0.05	0	yadI	1	35.9333	1.235
0.05	0	AG1	1	35.9333	0.924
0.05	0	ptsI	1	36.1833	1.358
0.05	0	clcB	1	36.1833	0.874
0.05	0	ycaM	1	36.1833	0.874
0.05	0	yadI	1	36.1833	1.242
0.05	0	AG1	1	36.1833	0.928
0.05	0	ptsI	1	36.4333	1.377
0.05	0	clcB	1	36.4333	0.873
0.05	0	ycaM	1	36.4333	0.872
0.05	0	yadI	1	36.4333	1.25
0.05	0	AG1	1	36.4333	0.932
0.05	0	ptsI	1	36.6833	1.375
0.05	0	clcB	1	36.6833	0.869
0.05	0	ycaM	1	36.6833	0.877
0.05	0	yadI	1	36.6833	1.253
0.05	0	AG1	1	36.6833	0.927
0.05	0	ptsI	2	0	0.209
0.05	0	clcB	2	0	0.224
0.05	0	ycaM	2	0	0.225
0.05	0	yadI	2	0	0.222
0.05	0	AG1	2	0	0.237
0.05	0	ptsI	2	0.25	0.21
0.05	0	clcB	2	0.25	0.218
0.05	0	ycaM	2	0.25	0.217
0.05	0	yadI	2	0.25	0.217
0.05	0	AG1	2	0.25	0.23
0.05	0	ptsI	2	0.5	0.211
0.05	0	clcB	2	0.5	0.218
0.05	0	ycaM	2	0.5	0.216
0.05	0	yadI	2	0.5	0.214
0.05	0	AG1	2	0.5	0.226
0.05	0	ptsI	2	0.75	0.212

0.05	0	clcB	2	0.75	0.219
0.05	0	ycaM	2	0.75	0.218
0.05	0	yadI	2	0.75	0.218
0.05	0	AG1	2	0.75	0.229
0.05	0	ptsI	2	1	0.216
0.05	0	clcB	2	1	0.22
0.05	0	ycaM	2	1	0.22
0.05	0	yadI	2	1	0.223
0.05	0	AG1	2	1	0.231
0.05	0	ptsI	2	1.25	0.217
0.05	0	clcB	2	1.25	0.223
0.05	0	ycaM	2	1.25	0.222
0.05	0	yadI	2	1.25	0.23
0.05	0	AG1	2	1.25	0.237
0.05	0	ptsI	2	1.5	0.22
0.05	0	clcB	2	1.5	0.225
0.05	0	ycaM	2	1.5	0.225
0.05	0	yadI	2	1.5	0.236
0.05	0	AG1	2	1.5	0.244
0.05	0	ptsI	2	1.75	0.224
0.05	0	clcB	2	1.75	0.228
0.05	0	ycaM	2	1.75	0.23
0.05	0	yadI	2	1.75	0.24
0.05	0	AG1	2	1.75	0.247
0.05	0	ptsI	2	2	0.231
0.05	0	clcB	2	2	0.234
0.05	0	ycaM	2	2	0.236
0.05	0	yadI	2	2	0.248
0.05	0	AG1	2	2	0.255
0.05	0	ptsI	2	2.25	0.24
0.05	0	clcB	2	2.25	0.241
0.05	0	ycaM	2	2.25	0.243
0.05	0	yadI	2	2.25	0.262
0.05	0	AG1	2	2.25	0.265
0.05	0	ptsI	2	2.5	0.248
0.05	0	clcB	2	2.5	0.247
0.05	0	ycaM	2	2.5	0.248
0.05	0	yadI	2	2.5	0.275
0.05	0	AG1	2	2.5	0.274
0.05	0	ptsI	2	2.75	0.254
0.05	0	clcB	2	2.75	0.252
0.05	0	ycaM	2	2.75	0.257
0.05	0	yadI	2	2.75	0.287
0.05	0	AG1	2	2.75	0.281
0.05	0	ptsI	2	3	0.265
0.05	0	clcB	2	3	0.258
0.05	0	ycaM	2	3	0.264
0.05	0	yadI	2	3	0.303
0.05	0	AG1	2	3	0.287
0.05	0	ptsI	2	3.25	0.271
0.05	0	clcB	2	3.25	0.27
0.05	0	ycaM	2	3.25	0.273
0.05	0	yadI	2	3.25	0.32

0.05	0	AG1	2	3.25	0.3
0.05	0	ptsI	2	3.5	0.292
0.05	0	clcB	2	3.5	0.283
0.05	0	ycaM	2	3.5	0.281
0.05	0	yadI	2	3.5	0.343
0.05	0	AG1	2	3.5	0.313
0.05	0	ptsI	2	3.75	0.294
0.05	0	clcB	2	3.75	0.282
0.05	0	ycaM	2	3.75	0.291
0.05	0	yadI	2	3.75	0.364
0.05	0	AG1	2	3.75	0.326
0.05	0	ptsI	2	4	0.302
0.05	0	clcB	2	4	0.291
0.05	0	ycaM	2	4	0.302
0.05	0	yadI	2	4	0.389
0.05	0	AG1	2	4	0.336
0.05	0	ptsI	2	4.38333	0.325
0.05	0	clcB	2	4.38333	0.307
0.05	0	ycaM	2	4.38333	0.32
0.05	0	yadI	2	4.38333	0.406
0.05	0	AG1	2	4.38333	0.356
0.05	0	ptsI	2	4.63333	0.315
0.05	0	clcB	2	4.63333	0.3
0.05	0	ycaM	2	4.63333	0.315
0.05	0	yadI	2	4.63333	0.412
0.05	0	AG1	2	4.63333	0.351
0.05	0	ptsI	2	4.88333	0.322
0.05	0	clcB	2	4.88333	0.305
0.05	0	ycaM	2	4.88333	0.323
0.05	0	yadI	2	4.88333	0.427
0.05	0	AG1	2	4.88333	0.361
0.05	0	ptsI	2	5.13333	0.329
0.05	0	clcB	2	5.13333	0.315
0.05	0	ycaM	2	5.13333	0.332
0.05	0	yadI	2	5.13333	0.442
0.05	0	AG1	2	5.13333	0.373
0.05	0	ptsI	2	5.38333	0.337
0.05	0	clcB	2	5.38333	0.323
0.05	0	ycaM	2	5.38333	0.341
0.05	0	yadI	2	5.38333	0.469
0.05	0	AG1	2	5.38333	0.383
0.05	0	ptsI	2	5.63333	0.345
0.05	0	clcB	2	5.63333	0.33
0.05	0	ycaM	2	5.63333	0.352
0.05	0	yadI	2	5.63333	0.47
0.05	0	AG1	2	5.63333	0.392
0.05	0	ptsI	2	5.88333	0.353
0.05	0	clcB	2	5.88333	0.336
0.05	0	ycaM	2	5.88333	0.363
0.05	0	yadI	2	5.88333	0.479
0.05	0	AG1	2	5.88333	0.401
0.05	0	ptsI	2	6.13333	0.361
0.05	0	clcB	2	6.13333	0.343

0.05	0	ycaM	2	6.13333	0.376
0.05	0	yadI	2	6.13333	0.494
0.05	0	AG1	2	6.13333	0.417
0.05	0	ptsI	2	6.38333	0.37
0.05	0	clcB	2	6.38333	0.352
0.05	0	ycaM	2	6.38333	0.388
0.05	0	yadI	2	6.38333	0.508
0.05	0	AG1	2	6.38333	0.43
0.05	0	ptsI	2	6.63333	0.379
0.05	0	clcB	2	6.63333	0.361
0.05	0	ycaM	2	6.63333	0.401
0.05	0	yadI	2	6.63333	0.523
0.05	0	AG1	2	6.63333	0.439
0.05	0	ptsI	2	6.88333	0.385
0.05	0	clcB	2	6.88333	0.368
0.05	0	ycaM	2	6.88333	0.41
0.05	0	yadI	2	6.88333	0.539
0.05	0	AG1	2	6.88333	0.454
0.05	0	ptsI	2	7.13333	0.391
0.05	0	clcB	2	7.13333	0.375
0.05	0	ycaM	2	7.13333	0.417
0.05	0	yadI	2	7.13333	0.546
0.05	0	AG1	2	7.13333	0.462
0.05	0	ptsI	2	7.38333	0.398
0.05	0	clcB	2	7.38333	0.384
0.05	0	ycaM	2	7.38333	0.429
0.05	0	yadI	2	7.38333	0.565
0.05	0	AG1	2	7.38333	0.476
0.05	0	ptsI	2	7.63333	0.406
0.05	0	clcB	2	7.63333	0.392
0.05	0	ycaM	2	7.63333	0.437
0.05	0	yadI	2	7.63333	0.576
0.05	0	AG1	2	7.63333	0.488
0.05	0	ptsI	2	7.88333	0.418
0.05	0	clcB	2	7.88333	0.4
0.05	0	ycaM	2	7.88333	0.446
0.05	0	yadI	2	7.88333	0.594
0.05	0	AG1	2	7.88333	0.499
0.05	0	ptsI	2	8.13333	0.428
0.05	0	clcB	2	8.13333	0.407
0.05	0	ycaM	2	8.13333	0.452
0.05	0	yadI	2	8.13333	0.606
0.05	0	AG1	2	8.13333	0.512
0.05	0	ptsI	2	8.38333	0.434
0.05	0	clcB	2	8.38333	0.419
0.05	0	ycaM	2	8.38333	0.456
0.05	0	yadI	2	8.38333	0.624
0.05	0	AG1	2	8.38333	0.523
0.05	0	ptsI	2	8.91667	0.496
0.05	0	clcB	2	8.91667	0.47
0.05	0	ycaM	2	8.91667	0.492
0.05	0	yadI	2	8.91667	0.669
0.05	0	AG1	2	8.91667	0.403

0.05	0	ptsI	2	9.16667	0.497
0.05	0	clcB	2	9.16667	0.471
0.05	0	ycaM	2	9.16667	0.489
0.05	0	yadI	2	9.16667	0.66
0.05	0	AG1	2	9.16667	0.404
0.05	0	ptsI	2	9.41667	0.501
0.05	0	clcB	2	9.41667	0.48
0.05	0	ycaM	2	9.41667	0.494
0.05	0	yadI	2	9.41667	0.667
0.05	0	AG1	2	9.41667	0.417
0.05	0	ptsI	2	9.66667	0.513
0.05	0	clcB	2	9.66667	0.488
0.05	0	ycaM	2	9.66667	0.503
0.05	0	yadI	2	9.66667	0.675
0.05	0	AG1	2	9.66667	0.428
0.05	0	ptsI	2	9.91667	0.517
0.05	0	clcB	2	9.91667	0.494
0.05	0	ycaM	2	9.91667	0.511
0.05	0	yadI	2	9.91667	0.685
0.05	0	AG1	2	9.91667	0.434
0.05	0	ptsI	2	10.1667	0.525
0.05	0	clcB	2	10.1667	0.503
0.05	0	ycaM	2	10.1667	0.517
0.05	0	yadI	2	10.1667	0.697
0.05	0	AG1	2	10.1667	0.434
0.05	0	ptsI	2	10.4167	0.528
0.05	0	clcB	2	10.4167	0.513
0.05	0	ycaM	2	10.4167	0.527
0.05	0	yadI	2	10.4167	0.712
0.05	0	AG1	2	10.4167	0.454
0.05	0	ptsI	2	10.6667	0.533
0.05	0	clcB	2	10.6667	0.52
0.05	0	ycaM	2	10.6667	0.536
0.05	0	yadI	2	10.6667	0.723
0.05	0	AG1	2	10.6667	0.458
0.05	0	ptsI	2	10.9167	0.561
0.05	0	clcB	2	10.9167	0.528
0.05	0	ycaM	2	10.9167	0.544
0.05	0	yadI	2	10.9167	0.731
0.05	0	AG1	2	10.9167	0.463
0.05	0	ptsI	2	11.1667	0.581
0.05	0	clcB	2	11.1667	0.541
0.05	0	ycaM	2	11.1667	0.555
0.05	0	yadI	2	11.1667	0.744
0.05	0	AG1	2	11.1667	0.466
0.05	0	ptsI	2	11.4167	0.575
0.05	0	clcB	2	11.4167	0.545
0.05	0	ycaM	2	11.4167	0.563
0.05	0	yadI	2	11.4167	0.753
0.05	0	AG1	2	11.4167	0.464
0.05	0	ptsI	2	11.6667	0.569
0.05	0	clcB	2	11.6667	0.56
0.05	0	ycaM	2	11.6667	0.574

0.05	0	yadI	2	11.6667	0.767
0.05	0	AG1	2	11.6667	0.485
0.05	0	ptsI	2	11.9167	0.548
0.05	0	clcB	2	11.9167	0.564
0.05	0	ycaM	2	11.9167	0.585
0.05	0	yadI	2	11.9167	0.774
0.05	0	AG1	2	11.9167	0.483
0.05	0	ptsI	2	12.1667	0.587
0.05	0	clcB	2	12.1667	0.58
0.05	0	ycaM	2	12.1667	0.595
0.05	0	yadI	2	12.1667	0.792
0.05	0	AG1	2	12.1667	0.496
0.05	0	ptsI	2	12.4167	0.568
0.05	0	clcB	2	12.4167	0.596
0.05	0	ycaM	2	12.4167	0.604
0.05	0	yadI	2	12.4167	0.792
0.05	0	AG1	2	12.4167	0.496
0.05	0	ptsI	2	12.6667	0.573
0.05	0	clcB	2	12.6667	0.587
0.05	0	ycaM	2	12.6667	0.612
0.05	0	yadI	2	12.6667	0.804
0.05	0	AG1	2	12.6667	0.5
0.05	0	ptsI	2	12.9167	0.585
0.05	0	clcB	2	12.9167	0.592
0.05	0	ycaM	2	12.9167	0.633
0.05	0	yadI	2	12.9167	0.818
0.05	0	AG1	2	12.9167	0.504
0.05	0	ptsI	2	13.1667	0.59
0.05	0	clcB	2	13.1667	0.578
0.05	0	ycaM	2	13.1667	0.641
0.05	0	yadI	2	13.1667	0.821
0.05	0	AG1	2	13.1667	0.509
0.05	0	ptsI	2	13.4167	0.587
0.05	0	clcB	2	13.4167	0.575
0.05	0	ycaM	2	13.4167	0.654
0.05	0	yadI	2	13.4167	0.837
0.05	0	AG1	2	13.4167	0.507
0.05	0	ptsI	2	13.6667	0.594
0.05	0	clcB	2	13.6667	0.583
0.05	0	ycaM	2	13.6667	0.678
0.05	0	yadI	2	13.6667	0.835
0.05	0	AG1	2	13.6667	0.539
0.05	0	ptsI	2	13.9167	0.604
0.05	0	clcB	2	13.9167	0.589
0.05	0	ycaM	2	13.9167	0.679
0.05	0	yadI	2	13.9167	0.841
0.05	0	AG1	2	13.9167	0.543
0.05	0	ptsI	2	14.1667	0.606
0.05	0	clcB	2	14.1667	0.584
0.05	0	ycaM	2	14.1667	0.685
0.05	0	yadI	2	14.1667	0.857
0.05	0	AG1	2	14.1667	0.534
0.05	0	ptsI	2	14.4167	0.624

0.05	0	clcB	2	14.4167	0.584
0.05	0	ycaM	2	14.4167	0.706
0.05	0	yadI	2	14.4167	0.862
0.05	0	AG1	2	14.4167	0.564
0.05	0	ptsI	2	14.6667	0.648
0.05	0	clcB	2	14.6667	0.592
0.05	0	ycaM	2	14.6667	0.693
0.05	0	yadI	2	14.6667	0.862
0.05	0	AG1	2	14.6667	0.586
0.05	0	ptsI	2	14.9167	0.67
0.05	0	clcB	2	14.9167	0.596
0.05	0	ycaM	2	14.9167	0.696
0.05	0	yadI	2	14.9167	0.865
0.05	0	AG1	2	14.9167	0.597
0.05	0	ptsI	2	15.1667	0.661
0.05	0	clcB	2	15.1667	0.605
0.05	0	ycaM	2	15.1667	0.69
0.05	0	yadI	2	15.1667	0.873
0.05	0	AG1	2	15.1667	0.614
0.05	0	ptsI	2	15.4167	0.678
0.05	0	clcB	2	15.4167	0.597
0.05	0	ycaM	2	15.4167	0.681
0.05	0	yadI	2	15.4167	0.878
0.05	0	AG1	2	15.4167	0.635
0.05	0	ptsI	2	15.6667	0.687
0.05	0	clcB	2	15.6667	0.618
0.05	0	ycaM	2	15.6667	0.691
0.05	0	yadI	2	15.6667	0.879
0.05	0	AG1	2	15.6667	0.654
0.05	0	ptsI	2	15.9167	0.712
0.05	0	clcB	2	15.9167	0.59
0.05	0	ycaM	2	15.9167	0.679
0.05	0	yadI	2	15.9167	0.889
0.05	0	AG1	2	15.9167	0.653
0.05	0	ptsI	2	16.1667	0.719
0.05	0	clcB	2	16.1667	0.62
0.05	0	ycaM	2	16.1667	0.676
0.05	0	yadI	2	16.1667	0.894
0.05	0	AG1	2	16.1667	0.688
0.05	0	ptsI	2	16.4167	0.744
0.05	0	clcB	2	16.4167	0.613
0.05	0	ycaM	2	16.4167	0.685
0.05	0	yadI	2	16.4167	0.896
0.05	0	AG1	2	16.4167	0.687
0.05	0	ptsI	2	16.6667	0.77
0.05	0	clcB	2	16.6667	0.634
0.05	0	ycaM	2	16.6667	0.679
0.05	0	yadI	2	16.6667	0.895
0.05	0	AG1	2	16.6667	0.673
0.05	0	ptsI	2	16.9167	0.788
0.05	0	clcB	2	16.9167	0.638
0.05	0	ycaM	2	16.9167	0.685
0.05	0	yadI	2	16.9167	0.902

0.05	0	AG1	2	16.9167	0.674
0.05	0	ptsI	2	17.1667	0.803
0.05	0	clcB	2	17.1667	0.629
0.05	0	ycaM	2	17.1667	0.681
0.05	0	yadI	2	17.1667	0.907
0.05	0	AG1	2	17.1667	0.692
0.05	0	ptsI	2	17.4167	0.811
0.05	0	clcB	2	17.4167	0.639
0.05	0	ycaM	2	17.4167	0.713
0.05	0	yadI	2	17.4167	0.906
0.05	0	AG1	2	17.4167	0.692
0.05	0	ptsI	2	17.6667	0.819
0.05	0	clcB	2	17.6667	0.62
0.05	0	ycaM	2	17.6667	0.714
0.05	0	yadI	2	17.6667	0.918
0.05	0	AG1	2	17.6667	0.699
0.05	0	ptsI	2	17.9167	0.825
0.05	0	clcB	2	17.9167	0.614
0.05	0	ycaM	2	17.9167	0.715
0.05	0	yadI	2	17.9167	0.91
0.05	0	AG1	2	17.9167	0.708
0.05	0	ptsI	2	18.1667	0.83
0.05	0	clcB	2	18.1667	0.62
0.05	0	ycaM	2	18.1667	0.706
0.05	0	yadI	2	18.1667	0.918
0.05	0	AG1	2	18.1667	0.718
0.05	0	ptsI	2	18.4167	0.842
0.05	0	clcB	2	18.4167	0.596
0.05	0	ycaM	2	18.4167	0.699
0.05	0	yadI	2	18.4167	0.92
0.05	0	AG1	2	18.4167	0.72
0.05	0	ptsI	2	18.6667	0.839
0.05	0	clcB	2	18.6667	0.6
0.05	0	ycaM	2	18.6667	0.693
0.05	0	yadI	2	18.6667	0.917
0.05	0	AG1	2	18.6667	0.724
0.05	0	ptsI	2	18.9167	0.84
0.05	0	clcB	2	18.9167	0.604
0.05	0	ycaM	2	18.9167	0.696
0.05	0	yadI	2	18.9167	0.92
0.05	0	AG1	2	18.9167	0.731
0.05	0	ptsI	2	19.1667	0.856
0.05	0	clcB	2	19.1667	0.611
0.05	0	ycaM	2	19.1667	0.703
0.05	0	yadI	2	19.1667	0.919
0.05	0	AG1	2	19.1667	0.737
0.05	0	ptsI	2	19.4167	0.857
0.05	0	clcB	2	19.4167	0.611
0.05	0	ycaM	2	19.4167	0.712
0.05	0	yadI	2	19.4167	0.917
0.05	0	AG1	2	19.4167	0.74
0.05	0	ptsI	2	19.6667	0.865
0.05	0	clcB	2	19.6667	0.613

0.05	0	ycaM	2	19.6667	0.711
0.05	0	yadI	2	19.6667	0.929
0.05	0	AG1	2	19.6667	0.748
0.05	0	ptsI	2	19.9167	0.877
0.05	0	clcB	2	19.9167	0.62
0.05	0	ycaM	2	19.9167	0.715
0.05	0	yadI	2	19.9167	0.924
0.05	0	AG1	2	19.9167	0.76
0.05	0	ptsI	2	20.1667	0.871
0.05	0	clcB	2	20.1667	0.616
0.05	0	ycaM	2	20.1667	0.712
0.05	0	yadI	2	20.1667	0.925
0.05	0	AG1	2	20.1667	0.753
0.05	0	ptsI	2	20.4167	0.876
0.05	0	clcB	2	20.4167	0.618
0.05	0	ycaM	2	20.4167	0.716
0.05	0	yadI	2	20.4167	0.926
0.05	0	AG1	2	20.4167	0.751
0.05	0	ptsI	2	20.6667	0.88
0.05	0	clcB	2	20.6667	0.618
0.05	0	ycaM	2	20.6667	0.716
0.05	0	yadI	2	20.6667	0.926
0.05	0	AG1	2	20.6667	0.754
0.05	0	ptsI	2	20.9167	0.88
0.05	0	clcB	2	20.9167	0.621
0.05	0	ycaM	2	20.9167	0.716
0.05	0	yadI	2	20.9167	0.932
0.05	0	AG1	2	20.9167	0.756
0.05	0	ptsI	2	21.1667	0.881
0.05	0	clcB	2	21.1667	0.623
0.05	0	ycaM	2	21.1667	0.721
0.05	0	yadI	2	21.1667	0.932
0.05	0	AG1	2	21.1667	0.755
0.05	0	ptsI	2	21.4167	0.872
0.05	0	clcB	2	21.4167	0.619
0.05	0	ycaM	2	21.4167	0.71
0.05	0	yadI	2	21.4167	0.943
0.05	0	AG1	2	21.4167	0.753
0.05	0	ptsI	2	21.6667	0.886
0.05	0	clcB	2	21.6667	0.624
0.05	0	ycaM	2	21.6667	0.722
0.05	0	yadI	2	21.6667	0.937
0.05	0	AG1	2	21.6667	0.757
0.05	0	ptsI	2	21.9167	0.899
0.05	0	clcB	2	21.9167	0.629
0.05	0	ycaM	2	21.9167	0.729
0.05	0	yadI	2	21.9167	0.937
0.05	0	AG1	2	21.9167	0.761
0.05	0	ptsI	2	22.1667	0.903
0.05	0	clcB	2	22.1667	0.629
0.05	0	ycaM	2	22.1667	0.726
0.05	0	yadI	2	22.1667	0.936
0.05	0	AG1	2	22.1667	0.76

0.05	0	ptsI	2	22.4167	0.91
0.05	0	clcB	2	22.4167	0.63
0.05	0	ycaM	2	22.4167	0.723
0.05	0	yadI	2	22.4167	0.941
0.05	0	AG1	2	22.4167	0.759
0.05	0	ptsI	2	22.6667	0.918
0.05	0	clcB	2	22.6667	0.633
0.05	0	ycaM	2	22.6667	0.725
0.05	0	yadI	2	22.6667	0.939
0.05	0	AG1	2	22.6667	0.759
0.05	0	ptsI	2	22.9167	0.915
0.05	0	clcB	2	22.9167	0.635
0.05	0	ycaM	2	22.9167	0.728
0.05	0	yadI	2	22.9167	0.943
0.05	0	AG1	2	22.9167	0.767
0.05	0	ptsI	2	23.1667	0.926
0.05	0	clcB	2	23.1667	0.64
0.05	0	ycaM	2	23.1667	0.725
0.05	0	yadI	2	23.1667	0.936
0.05	0	AG1	2	23.1667	0.758
0.05	0	ptsI	2	23.4167	0.927
0.05	0	clcB	2	23.4167	0.64
0.05	0	ycaM	2	23.4167	0.729
0.05	0	yadI	2	23.4167	0.942
0.05	0	AG1	2	23.4167	0.758
0.05	0	ptsI	2	23.6667	0.934
0.05	0	clcB	2	23.6667	0.644
0.05	0	ycaM	2	23.6667	0.729
0.05	0	yadI	2	23.6667	0.94
0.05	0	AG1	2	23.6667	0.76
0.05	0	ptsI	2	23.9167	0.946
0.05	0	clcB	2	23.9167	0.646
0.05	0	ycaM	2	23.9167	0.731
0.05	0	yadI	2	23.9167	0.943
0.05	0	AG1	2	23.9167	0.763
0.05	0	ptsI	2	24.1667	0.949
0.05	0	clcB	2	24.1667	0.644
0.05	0	ycaM	2	24.1667	0.73
0.05	0	yadI	2	24.1667	0.942
0.05	0	AG1	2	24.1667	0.762
0.05	0	ptsI	2	24.4167	0.952
0.05	0	clcB	2	24.4167	0.643
0.05	0	ycaM	2	24.4167	0.731
0.05	0	yadI	2	24.4167	0.945
0.05	0	AG1	2	24.4167	0.761
0.05	0	ptsI	2	24.6667	0.965
0.05	0	clcB	2	24.6667	0.649
0.05	0	ycaM	2	24.6667	0.732
0.05	0	yadI	2	24.6667	0.943
0.05	0	AG1	2	24.6667	0.764
0.05	0	ptsI	2	24.9167	0.977
0.05	0	clcB	2	24.9167	0.651
0.05	0	ycaM	2	24.9167	0.734

0.05	0	yadI	2	24.9167	0.953
0.05	0	AG1	2	24.9167	0.765
0.05	0	ptsI	2	25.1667	0.983
0.05	0	clcB	2	25.1667	0.648
0.05	0	ycaM	2	25.1667	0.735
0.05	0	yadI	2	25.1667	0.948
0.05	0	AG1	2	25.1667	0.763
0.05	0	ptsI	2	25.4167	0.996
0.05	0	clcB	2	25.4167	0.655
0.05	0	ycaM	2	25.4167	0.738
0.05	0	yadI	2	25.4167	0.944
0.05	0	AG1	2	25.4167	0.772
0.05	0	ptsI	2	25.6667	1.002
0.05	0	clcB	2	25.6667	0.652
0.05	0	ycaM	2	25.6667	0.736
0.05	0	yadI	2	25.6667	0.944
0.05	0	AG1	2	25.6667	0.766
0.05	0	ptsI	2	25.9167	1.015
0.05	0	clcB	2	25.9167	0.651
0.05	0	ycaM	2	25.9167	0.736
0.05	0	yadI	2	25.9167	0.954
0.05	0	AG1	2	25.9167	0.765
0.05	0	ptsI	2	26.1667	1.02
0.05	0	clcB	2	26.1667	0.656
0.05	0	ycaM	2	26.1667	0.733
0.05	0	yadI	2	26.1667	0.954
0.05	0	AG1	2	26.1667	0.768
0.05	0	ptsI	2	26.4167	1.038
0.05	0	clcB	2	26.4167	0.661
0.05	0	ycaM	2	26.4167	0.744
0.05	0	yadI	2	26.4167	0.952
0.05	0	AG1	2	26.4167	0.78
0.05	0	ptsI	3	0	0.254
0.05	0	clcB	3	0	0.153
0.05	0	ycaM	3	0	0.156
0.05	0	yadI	3	0	0.175
0.05	0	AG1	3	0	0.168
0.05	0	ptsI	3	0.25	0.149
0.05	0	clcB	3	0.25	0.152
0.05	0	ycaM	3	0.25	0.158
0.05	0	yadI	3	0.25	0.171
0.05	0	AG1	3	0.25	0.17
0.05	0	ptsI	3	0.5	0.145
0.05	0	clcB	3	0.5	0.152
0.05	0	ycaM	3	0.5	0.159
0.05	0	yadI	3	0.5	0.17
0.05	0	AG1	3	0.5	0.163
0.05	0	ptsI	3	0.75	0.146
0.05	0	clcB	3	0.75	0.152
0.05	0	ycaM	3	0.75	0.159
0.05	0	yadI	3	0.75	0.17
0.05	0	AG1	3	0.75	0.165
0.05	0	ptsI	3	1	0.147

0.05	0	clcB	3	1	0.153
0.05	0	ycaM	3	1	0.162
0.05	0	yadI	3	1	0.172
0.05	0	AG1	3	1	0.167
0.05	0	ptsI	3	1.25	0.149
0.05	0	clcB	3	1.25	0.153
0.05	0	ycaM	3	1.25	0.163
0.05	0	yadI	3	1.25	0.176
0.05	0	AG1	3	1.25	0.166
0.05	0	ptsI	3	1.5	0.15
0.05	0	clcB	3	1.5	0.156
0.05	0	ycaM	3	1.5	0.165
0.05	0	yadI	3	1.5	0.18
0.05	0	AG1	3	1.5	0.169
0.05	0	ptsI	3	1.75	0.151
0.05	0	clcB	3	1.75	0.156
0.05	0	ycaM	3	1.75	0.164
0.05	0	yadI	3	1.75	0.183
0.05	0	AG1	3	1.75	0.17
0.05	0	ptsI	3	2	0.154
0.05	0	clcB	3	2	0.158
0.05	0	ycaM	3	2	0.165
0.05	0	yadI	3	2	0.188
0.05	0	AG1	3	2	0.172
0.05	0	ptsI	3	2.25	0.157
0.05	0	clcB	3	2.25	0.16
0.05	0	ycaM	3	2.25	0.168
0.05	0	yadI	3	2.25	0.192
0.05	0	AG1	3	2.25	0.175
0.05	0	ptsI	3	2.5	0.159
0.05	0	clcB	3	2.5	0.162
0.05	0	ycaM	3	2.5	0.17
0.05	0	yadI	3	2.5	0.197
0.05	0	AG1	3	2.5	0.179
0.05	0	ptsI	3	2.75	0.164
0.05	0	clcB	3	2.75	0.166
0.05	0	ycaM	3	2.75	0.172
0.05	0	yadI	3	2.75	0.206
0.05	0	AG1	3	2.75	0.184
0.05	0	ptsI	3	3	0.167
0.05	0	clcB	3	3	0.167
0.05	0	ycaM	3	3	0.174
0.05	0	yadI	3	3	0.208
0.05	0	AG1	3	3	0.187
0.05	0	ptsI	3	3.25	0.171
0.05	0	clcB	3	3.25	0.172
0.05	0	ycaM	3	3.25	0.177
0.05	0	yadI	3	3.25	0.218
0.05	0	AG1	3	3.25	0.191
0.05	0	ptsI	3	3.5	0.175
0.05	0	clcB	3	3.5	0.176
0.05	0	ycaM	3	3.5	0.181
0.05	0	yadI	3	3.5	0.228

0.05	0	AG1	3	3.5	0.194
0.05	0	ptsI	3	3.75	0.18
0.05	0	clcB	3	3.75	0.181
0.05	0	ycaM	3	3.75	0.186
0.05	0	yadI	3	3.75	0.24
0.05	0	AG1	3	3.75	0.198
0.05	0	ptsI	3	4	0.184
0.05	0	clcB	3	4	0.184
0.05	0	ycaM	3	4	0.19
0.05	0	yadI	3	4	0.253
0.05	0	AG1	3	4	0.203
0.05	0	ptsI	3	4.41667	0.194
0.05	0	clcB	3	4.41667	0.204
0.05	0	ycaM	3	4.41667	0.201
0.05	0	yadI	3	4.41667	0.27
0.05	0	AG1	3	4.41667	0.212
0.05	0	ptsI	3	4.66667	0.198
0.05	0	clcB	3	4.66667	0.198
0.05	0	ycaM	3	4.66667	0.197
0.05	0	yadI	3	4.66667	0.266
0.05	0	AG1	3	4.66667	0.209
0.05	0	ptsI	3	4.91667	0.2
0.05	0	clcB	3	4.91667	0.205
0.05	0	ycaM	3	4.91667	0.199
0.05	0	yadI	3	4.91667	0.277
0.05	0	AG1	3	4.91667	0.211
0.05	0	ptsI	3	5.16667	0.205
0.05	0	clcB	3	5.16667	0.21
0.05	0	ycaM	3	5.16667	0.204
0.05	0	yadI	3	5.16667	0.295
0.05	0	AG1	3	5.16667	0.218
0.05	0	ptsI	3	5.41667	0.211
0.05	0	clcB	3	5.41667	0.22
0.05	0	ycaM	3	5.41667	0.211
0.05	0	yadI	3	5.41667	0.311
0.05	0	AG1	3	5.41667	0.228
0.05	0	ptsI	3	5.66667	0.218
0.05	0	clcB	3	5.66667	0.229
0.05	0	ycaM	3	5.66667	0.22
0.05	0	yadI	3	5.66667	0.323
0.05	0	AG1	3	5.66667	0.237
0.05	0	ptsI	3	5.91667	0.225
0.05	0	clcB	3	5.91667	0.243
0.05	0	ycaM	3	5.91667	0.228
0.05	0	yadI	3	5.91667	0.335
0.05	0	AG1	3	5.91667	0.246
0.05	0	ptsI	3	6.16667	0.236
0.05	0	clcB	3	6.16667	0.258
0.05	0	ycaM	3	6.16667	0.238
0.05	0	yadI	3	6.16667	0.352
0.05	0	AG1	3	6.16667	0.268
0.05	0	ptsI	3	6.41667	0.243
0.05	0	clcB	3	6.41667	0.272

0.05	0	ycaM	3	6.41667	0.25
0.05	0	yadI	3	6.41667	0.368
0.05	0	AG1	3	6.41667	0.273
0.05	0	ptsI	3	6.66667	0.255
0.05	0	clcB	3	6.66667	0.289
0.05	0	ycaM	3	6.66667	0.263
0.05	0	yadI	3	6.66667	0.383
0.05	0	AG1	3	6.66667	0.286
0.05	0	ptsI	3	6.91667	0.264
0.05	0	clcB	3	6.91667	0.291
0.05	0	ycaM	3	6.91667	0.276
0.05	0	yadI	3	6.91667	0.394
0.05	0	AG1	3	6.91667	0.293
0.05	0	ptsI	3	7.16667	0.274
0.05	0	clcB	3	7.16667	0.3
0.05	0	ycaM	3	7.16667	0.292
0.05	0	yadI	3	7.16667	0.406
0.05	0	AG1	3	7.16667	0.302
0.05	0	ptsI	3	7.41667	0.284
0.05	0	clcB	3	7.41667	0.309
0.05	0	ycaM	3	7.41667	0.3
0.05	0	yadI	3	7.41667	0.419
0.05	0	AG1	3	7.41667	0.313
0.05	0	ptsI	3	7.66667	0.291
0.05	0	clcB	3	7.66667	0.32
0.05	0	ycaM	3	7.66667	0.31
0.05	0	yadI	3	7.66667	0.43
0.05	0	AG1	3	7.66667	0.323
0.05	0	ptsI	3	7.91667	0.299
0.05	0	clcB	3	7.91667	0.324
0.05	0	ycaM	3	7.91667	0.317
0.05	0	yadI	3	7.91667	0.443
0.05	0	AG1	3	7.91667	0.331
0.05	0	ptsI	3	8.16667	0.306
0.05	0	clcB	3	8.16667	0.334
0.05	0	ycaM	3	8.16667	0.326
0.05	0	yadI	3	8.16667	0.453
0.05	0	AG1	3	8.16667	0.341
0.05	0	ptsI	3	8.41667	0.316
0.05	0	clcB	3	8.41667	0.344
0.05	0	ycaM	3	8.41667	0.336
0.05	0	yadI	3	8.41667	0.471
0.05	0	AG1	3	8.41667	0.354
0.05	0	ptsI	3	9.05	0.278
0.05	0	clcB	3	9.05	0.306
0.05	0	ycaM	3	9.05	0.302
0.05	0	yadI	3	9.05	0.451
0.05	0	AG1	3	9.05	0.317
0.05	0	ptsI	3	9.3	0.284
0.05	0	clcB	3	9.3	0.31
0.05	0	ycaM	3	9.3	0.306
0.05	0	yadI	3	9.3	0.455
0.05	0	AG1	3	9.3	0.315

0.05	0	ptsI	3	9.55	0.29
0.05	0	clcB	3	9.55	0.319
0.05	0	ycaM	3	9.55	0.313
0.05	0	yadI	3	9.55	0.464
0.05	0	AG1	3	9.55	0.323
0.05	0	ptsI	3	9.8	0.298
0.05	0	clcB	3	9.8	0.325
0.05	0	ycaM	3	9.8	0.321
0.05	0	yadI	3	9.8	0.476
0.05	0	AG1	3	9.8	0.331
0.05	0	ptsI	3	10.05	0.303
0.05	0	clcB	3	10.05	0.333
0.05	0	ycaM	3	10.05	0.328
0.05	0	yadI	3	10.05	0.489
0.05	0	AG1	3	10.05	0.339
0.05	0	ptsI	3	10.3	0.31
0.05	0	clcB	3	10.3	0.339
0.05	0	ycaM	3	10.3	0.338
0.05	0	yadI	3	10.3	0.5
0.05	0	AG1	3	10.3	0.35
0.05	0	ptsI	3	10.55	0.32
0.05	0	clcB	3	10.55	0.344
0.05	0	ycaM	3	10.55	0.348
0.05	0	yadI	3	10.55	0.521
0.05	0	AG1	3	10.55	0.358
0.05	0	ptsI	3	10.8	0.328
0.05	0	clcB	3	10.8	0.35
0.05	0	ycaM	3	10.8	0.356
0.05	0	yadI	3	10.8	0.534
0.05	0	AG1	3	10.8	0.366
0.05	0	ptsI	3	11.05	0.336
0.05	0	clcB	3	11.05	0.354
0.05	0	ycaM	3	11.05	0.365
0.05	0	yadI	3	11.05	0.545
0.05	0	AG1	3	11.05	0.375
0.05	0	ptsI	3	11.3	0.345
0.05	0	clcB	3	11.3	0.36
0.05	0	ycaM	3	11.3	0.373
0.05	0	yadI	3	11.3	0.555
0.05	0	AG1	3	11.3	0.381
0.05	0	ptsI	3	11.55	0.352
0.05	0	clcB	3	11.55	0.368
0.05	0	ycaM	3	11.55	0.375
0.05	0	yadI	3	11.55	0.572
0.05	0	AG1	3	11.55	0.388
0.05	0	ptsI	3	11.8	0.36
0.05	0	clcB	3	11.8	0.372
0.05	0	ycaM	3	11.8	0.377
0.05	0	yadI	3	11.8	0.586
0.05	0	AG1	3	11.8	0.4
0.05	0	ptsI	3	12.05	0.367
0.05	0	clcB	3	12.05	0.381
0.05	0	ycaM	3	12.05	0.381

0.05	0	yadI	3	12.05	0.602
0.05	0	AG1	3	12.05	0.409
0.05	0	ptsI	3	12.3	0.38
0.05	0	clcB	3	12.3	0.393
0.05	0	ycaM	3	12.3	0.386
0.05	0	yadI	3	12.3	0.617
0.05	0	AG1	3	12.3	0.421
0.05	0	ptsI	3	12.55	0.387
0.05	0	clcB	3	12.55	0.4
0.05	0	ycaM	3	12.55	0.389
0.05	0	yadI	3	12.55	0.631
0.05	0	AG1	3	12.55	0.435
0.05	0	ptsI	3	12.8	0.398
0.05	0	clcB	3	12.8	0.405
0.05	0	ycaM	3	12.8	0.399
0.05	0	yadI	3	12.8	0.648
0.05	0	AG1	3	12.8	0.448
0.05	0	ptsI	3	13.05	0.409
0.05	0	clcB	3	13.05	0.408
0.05	0	ycaM	3	13.05	0.4
0.05	0	yadI	3	13.05	0.641
0.05	0	AG1	3	13.05	0.454
0.05	0	ptsI	3	13.3	0.42
0.05	0	clcB	3	13.3	0.414
0.05	0	ycaM	3	13.3	0.408
0.05	0	yadI	3	13.3	0.658
0.05	0	AG1	3	13.3	0.466
0.05	0	ptsI	3	13.55	0.433
0.05	0	clcB	3	13.55	0.416
0.05	0	ycaM	3	13.55	0.415
0.05	0	yadI	3	13.55	0.674
0.05	0	AG1	3	13.55	0.478
0.05	0	ptsI	3	13.8	0.444
0.05	0	clcB	3	13.8	0.431
0.05	0	ycaM	3	13.8	0.419
0.05	0	yadI	3	13.8	0.699
0.05	0	AG1	3	13.8	0.497
0.05	0	ptsI	3	14.05	0.453
0.05	0	clcB	3	14.05	0.438
0.05	0	ycaM	3	14.05	0.431
0.05	0	yadI	3	14.05	0.71
0.05	0	AG1	3	14.05	0.5
0.05	0	ptsI	3	14.3	0.463
0.05	0	clcB	3	14.3	0.451
0.05	0	ycaM	3	14.3	0.444
0.05	0	yadI	3	14.3	0.717
0.05	0	AG1	3	14.3	0.514
0.05	0	ptsI	3	14.55	0.475
0.05	0	clcB	3	14.55	0.447
0.05	0	ycaM	3	14.55	0.45
0.05	0	yadI	3	14.55	0.732
0.05	0	AG1	3	14.55	0.518
0.05	0	ptsI	3	14.8	0.491

0.05	0	clcB	3	14.8	0.443
0.05	0	ycaM	3	14.8	0.459
0.05	0	yadI	3	14.8	0.752
0.05	0	AG1	3	14.8	0.53
0.05	0	ptsI	3	15.05	0.504
0.05	0	clcB	3	15.05	0.437
0.05	0	ycaM	3	15.05	0.465
0.05	0	yadI	3	15.05	0.762
0.05	0	AG1	3	15.05	0.538
0.05	0	ptsI	3	15.3	0.519
0.05	0	clcB	3	15.3	0.456
0.05	0	ycaM	3	15.3	0.474
0.05	0	yadI	3	15.3	0.772
0.05	0	AG1	3	15.3	0.553
0.05	0	ptsI	3	15.55	0.525
0.05	0	clcB	3	15.55	0.45
0.05	0	ycaM	3	15.55	0.474
0.05	0	yadI	3	15.55	0.78
0.05	0	AG1	3	15.55	0.555
0.05	0	ptsI	3	15.8	0.53
0.05	0	clcB	3	15.8	0.448
0.05	0	ycaM	3	15.8	0.48
0.05	0	yadI	3	15.8	0.791
0.05	0	AG1	3	15.8	0.571
0.05	0	ptsI	3	16.05	0.532
0.05	0	clcB	3	16.05	0.456
0.05	0	ycaM	3	16.05	0.49
0.05	0	yadI	3	16.05	0.792
0.05	0	AG1	3	16.05	0.575
0.05	0	ptsI	3	16.3	0.544
0.05	0	clcB	3	16.3	0.453
0.05	0	ycaM	3	16.3	0.493
0.05	0	yadI	3	16.3	0.801
0.05	0	AG1	3	16.3	0.584
0.05	0	ptsI	3	16.55	0.55
0.05	0	clcB	3	16.55	0.461
0.05	0	ycaM	3	16.55	0.501
0.05	0	yadI	3	16.55	0.812
0.05	0	AG1	3	16.55	0.608
0.05	0	ptsI	3	16.8	0.557
0.05	0	clcB	3	16.8	0.468
0.05	0	ycaM	3	16.8	0.506
0.05	0	yadI	3	16.8	0.82
0.05	0	AG1	3	16.8	0.642
0.05	0	ptsI	3	17.05	0.579
0.05	0	clcB	3	17.05	0.47
0.05	0	ycaM	3	17.05	0.51
0.05	0	yadI	3	17.05	0.825
0.05	0	AG1	3	17.05	0.653
0.05	0	ptsI	3	17.3	0.589
0.05	0	clcB	3	17.3	0.466
0.05	0	ycaM	3	17.3	0.517
0.05	0	yadI	3	17.3	0.834

0.05	0	AG1	3	17.3	0.66
0.05	0	ptsI	3	17.55	0.623
0.05	0	clcB	3	17.55	0.473
0.05	0	ycaM	3	17.55	0.52
0.05	0	yadI	3	17.55	0.842
0.05	0	AG1	3	17.55	0.686
0.05	0	ptsI	3	17.8	0.647
0.05	0	clcB	3	17.8	0.47
0.05	0	ycaM	3	17.8	0.526
0.05	0	yadI	3	17.8	0.855
0.05	0	AG1	3	17.8	0.699
0.05	0	ptsI	3	18.05	0.643
0.05	0	clcB	3	18.05	0.474
0.05	0	ycaM	3	18.05	0.533
0.05	0	yadI	3	18.05	0.861
0.05	0	AG1	3	18.05	0.73
0.05	0	ptsI	3	18.3	0.656
0.05	0	clcB	3	18.3	0.473
0.05	0	ycaM	3	18.3	0.536
0.05	0	yadI	3	18.3	0.868
0.05	0	AG1	3	18.3	0.738
0.05	0	ptsI	3	18.55	0.628
0.05	0	clcB	3	18.55	0.473
0.05	0	ycaM	3	18.55	0.54
0.05	0	yadI	3	18.55	0.879
0.05	0	AG1	3	18.55	0.742
0.05	0	ptsI	3	18.8	0.647
0.05	0	clcB	3	18.8	0.473
0.05	0	ycaM	3	18.8	0.549
0.05	0	yadI	3	18.8	0.884
0.05	0	AG1	3	18.8	0.758
0.05	0	ptsI	3	19.05	0.672
0.05	0	clcB	3	19.05	0.477
0.05	0	ycaM	3	19.05	0.554
0.05	0	yadI	3	19.05	0.891
0.05	0	AG1	3	19.05	0.778
0.05	0	ptsI	3	19.3	0.688
0.05	0	clcB	3	19.3	0.476
0.05	0	ycaM	3	19.3	0.564
0.05	0	yadI	3	19.3	0.903
0.05	0	AG1	3	19.3	0.789
0.05	0	ptsI	3	19.55	0.71
0.05	0	clcB	3	19.55	0.485
0.05	0	ycaM	3	19.55	0.57
0.05	0	yadI	3	19.55	0.907
0.05	0	AG1	3	19.55	0.807
0.05	0	ptsI	3	19.8	0.699
0.05	0	clcB	3	19.8	0.485
0.05	0	ycaM	3	19.8	0.571
0.05	0	yadI	3	19.8	0.912
0.05	0	AG1	3	19.8	0.808
0.05	0	ptsI	3	20.05	0.706
0.05	0	clcB	3	20.05	0.481

0.05	0	ycaM	3	20.05	0.576
0.05	0	yadI	3	20.05	0.922
0.05	0	AG1	3	20.05	0.826
0.05	0	ptsI	3	20.3	0.708
0.05	0	clcB	3	20.3	0.476
0.05	0	ycaM	3	20.3	0.578
0.05	0	yadI	3	20.3	0.925
0.05	0	AG1	3	20.3	0.825
0.05	0	ptsI	3	20.55	0.73
0.05	0	clcB	3	20.55	0.481
0.05	0	ycaM	3	20.55	0.583
0.05	0	yadI	3	20.55	0.937
0.05	0	AG1	3	20.55	0.844
0.05	0	ptsI	3	20.8	0.74
0.05	0	clcB	3	20.8	0.482
0.05	0	ycaM	3	20.8	0.589
0.05	0	yadI	3	20.8	0.946
0.05	0	AG1	3	20.8	0.852
0.05	0	ptsI	3	21.05	0.757
0.05	0	clcB	3	21.05	0.482
0.05	0	ycaM	3	21.05	0.594
0.05	0	yadI	3	21.05	0.954
0.05	0	AG1	3	21.05	0.859
0.05	0	ptsI	3	21.3	0.766
0.05	0	clcB	3	21.3	0.483
0.05	0	ycaM	3	21.3	0.595
0.05	0	yadI	3	21.3	0.956
0.05	0	AG1	3	21.3	0.863
0.05	0	ptsI	3	21.55	0.782
0.05	0	clcB	3	21.55	0.486
0.05	0	ycaM	3	21.55	0.603
0.05	0	yadI	3	21.55	0.964
0.05	0	AG1	3	21.55	0.876
0.05	0	ptsI	3	21.8	0.794
0.05	0	clcB	3	21.8	0.488
0.05	0	ycaM	3	21.8	0.605
0.05	0	yadI	3	21.8	0.968
0.05	0	AG1	3	21.8	0.89
0.05	0	ptsI	3	22.05	0.802
0.05	0	clcB	3	22.05	0.488
0.05	0	ycaM	3	22.05	0.609
0.05	0	yadI	3	22.05	0.977
0.05	0	AG1	3	22.05	0.891
0.05	0	ptsI	3	22.3	0.814
0.05	0	clcB	3	22.3	0.491
0.05	0	ycaM	3	22.3	0.613
0.05	0	yadI	3	22.3	0.978
0.05	0	AG1	3	22.3	0.899
0.05	0	ptsI	3	22.55	0.824
0.05	0	clcB	3	22.55	0.493
0.05	0	ycaM	3	22.55	0.615
0.05	0	yadI	3	22.55	0.981
0.05	0	AG1	3	22.55	0.903

0.05	0	ptsI	3	22.8	0.826
0.05	0	clcB	3	22.8	0.493
0.05	0	ycaM	3	22.8	0.614
0.05	0	yadI	3	22.8	0.985
0.05	0	AG1	3	22.8	0.904
0.05	0	ptsI	3	23.05	0.833
0.05	0	clcB	3	23.05	0.495
0.05	0	ycaM	3	23.05	0.619
0.05	0	yadI	3	23.05	0.988
0.05	0	AG1	3	23.05	0.909
0.05	0	ptsI	3	23.3	0.836
0.05	0	clcB	3	23.3	0.495
0.05	0	ycaM	3	23.3	0.62
0.05	0	yadI	3	23.3	0.993
0.05	0	AG1	3	23.3	0.916
0.05	0	ptsI	3	23.55	0.855
0.05	0	clcB	3	23.55	0.501
0.05	0	ycaM	3	23.55	0.625
0.05	0	yadI	3	23.55	1.005
0.05	0	AG1	3	23.55	0.926
0.05	0	ptsI	3	23.8	0.854
0.05	0	clcB	3	23.8	0.496
0.05	0	ycaM	3	23.8	0.623
0.05	0	yadI	3	23.8	1.004
0.05	0	AG1	3	23.8	0.925
0.05	0	ptsI	3	24.05	0.862
0.05	0	clcB	3	24.05	0.499
0.05	0	ycaM	3	24.05	0.622
0.05	0	yadI	3	24.05	1.012
0.05	0	AG1	3	24.05	0.931
0.05	0	ptsI	3	24.3	0.862
0.05	0	clcB	3	24.3	0.492
0.05	0	ycaM	3	24.3	0.62
0.05	0	yadI	3	24.3	1.012
0.05	0	AG1	3	24.3	0.926
0.05	0	ptsI	4	0	0.202
0.05	0	clcB	4	0	0.207
0.05	0	ycaM	4	0	0.199
0.05	0	yadI	4	0	0.227
0.05	0	AG1	4	0	0.204
0.05	0	ptsI	4	0.25	0.201
0.05	0	clcB	4	0.25	0.21
0.05	0	ycaM	4	0.25	0.2
0.05	0	yadI	4	0.25	0.217
0.05	0	AG1	4	0.25	0.2
0.05	0	ptsI	4	0.5	0.204
0.05	0	clcB	4	0.5	0.214
0.05	0	ycaM	4	0.5	0.2
0.05	0	yadI	4	0.5	0.222
0.05	0	AG1	4	0.5	0.202
0.05	0	ptsI	4	0.75	0.209
0.05	0	clcB	4	0.75	0.218
0.05	0	ycaM	4	0.75	0.205

0.05	0	yadI	4	0.75	0.222
0.05	0	AG1	4	0.75	0.205
0.05	0	ptsI	4	1	0.213
0.05	0	clcB	4	1	0.222
0.05	0	ycaM	4	1	0.21
0.05	0	yadI	4	1	0.223
0.05	0	AG1	4	1	0.208
0.05	0	ptsI	4	1.25	0.22
0.05	0	clcB	4	1.25	0.228
0.05	0	ycaM	4	1.25	0.22
0.05	0	yadI	4	1.25	0.229
0.05	0	AG1	4	1.25	0.216
0.05	0	ptsI	4	1.5	0.224
0.05	0	clcB	4	1.5	0.232
0.05	0	ycaM	4	1.5	0.224
0.05	0	yadI	4	1.5	0.234
0.05	0	AG1	4	1.5	0.222
0.05	0	ptsI	4	1.75	0.235
0.05	0	clcB	4	1.75	0.245
0.05	0	ycaM	4	1.75	0.236
0.05	0	yadI	4	1.75	0.243
0.05	0	AG1	4	1.75	0.231
0.05	0	ptsI	4	2	0.246
0.05	0	clcB	4	2	0.254
0.05	0	ycaM	4	2	0.248
0.05	0	yadI	4	2	0.252
0.05	0	AG1	4	2	0.245
0.05	0	ptsI	4	2.25	0.26
0.05	0	clcB	4	2.25	0.266
0.05	0	ycaM	4	2.25	0.261
0.05	0	yadI	4	2.25	0.265
0.05	0	AG1	4	2.25	0.259
0.05	0	ptsI	4	2.5	0.271
0.05	0	clcB	4	2.5	0.278
0.05	0	ycaM	4	2.5	0.27
0.05	0	yadI	4	2.5	0.276
0.05	0	AG1	4	2.5	0.272
0.05	0	ptsI	4	2.75	0.283
0.05	0	clcB	4	2.75	0.288
0.05	0	ycaM	4	2.75	0.276
0.05	0	yadI	4	2.75	0.289
0.05	0	AG1	4	2.75	0.284
0.05	0	ptsI	4	3	0.3
0.05	0	clcB	4	3	0.304
0.05	0	ycaM	4	3	0.296
0.05	0	yadI	4	3	0.31
0.05	0	AG1	4	3	0.302
0.05	0	ptsI	4	3.25	0.313
0.05	0	clcB	4	3.25	0.312
0.05	0	ycaM	4	3.25	0.306
0.05	0	yadI	4	3.25	0.324
0.05	0	AG1	4	3.25	0.319
0.05	0	ptsI	4	3.5	0.328

0.05	0	clcB	4	3.5	0.321
0.05	0	ycaM	4	3.5	0.325
0.05	0	yadI	4	3.5	0.345
0.05	0	AG1	4	3.5	0.337
0.05	0	ptsI	4	3.75	0.345
0.05	0	clcB	4	3.75	0.336
0.05	0	ycaM	4	3.75	0.333
0.05	0	yadI	4	3.75	0.361
0.05	0	AG1	4	3.75	0.351
0.05	0	ptsI	4	4	0.362
0.05	0	clcB	4	4	0.354
0.05	0	ycaM	4	4	0.356
0.05	0	yadI	4	4	0.39
0.05	0	AG1	4	4	0.375
0.05	0	ptsI	4	4.35	0.37
0.05	0	clcB	4	4.35	0.29
0.05	0	ycaM	4	4.35	0.397
0.05	0	yadI	4	4.35	0.341
0.05	0	AG1	4	4.35	0.384
0.05	0	ptsI	4	4.6	0.37
0.05	0	clcB	4	4.6	0.294
0.05	0	ycaM	4	4.6	0.381
0.05	0	yadI	4	4.6	0.315
0.05	0	AG1	4	4.6	0.365
0.05	0	ptsI	4	4.85	0.385
0.05	0	clcB	4	4.85	0.303
0.05	0	ycaM	4	4.85	0.393
0.05	0	yadI	4	4.85	0.32
0.05	0	AG1	4	4.85	0.374
0.05	0	ptsI	4	5.1	0.398
0.05	0	clcB	4	5.1	0.312
0.05	0	ycaM	4	5.1	0.409
0.05	0	yadI	4	5.1	0.332
0.05	0	AG1	4	5.1	0.385
0.05	0	ptsI	4	5.35	0.417
0.05	0	clcB	4	5.35	0.319
0.05	0	ycaM	4	5.35	0.427
0.05	0	yadI	4	5.35	0.345
0.05	0	AG1	4	5.35	0.399
0.05	0	ptsI	4	5.6	0.43
0.05	0	clcB	4	5.6	0.327
0.05	0	ycaM	4	5.6	0.445
0.05	0	yadI	4	5.6	0.356
0.05	0	AG1	4	5.6	0.408
0.05	0	ptsI	4	5.85	0.444
0.05	0	clcB	4	5.85	0.336
0.05	0	ycaM	4	5.85	0.452
0.05	0	yadI	4	5.85	0.369
0.05	0	AG1	4	5.85	0.422
0.05	0	ptsI	4	6.1	0.459
0.05	0	clcB	4	6.1	0.349
0.05	0	ycaM	4	6.1	0.465
0.05	0	yadI	4	6.1	0.384

0.05	0	AG1	4	6.1	0.435
0.05	0	ptsI	4	6.35	0.471
0.05	0	clcB	4	6.35	0.356
0.05	0	ycaM	4	6.35	0.473
0.05	0	yadI	4	6.35	0.395
0.05	0	AG1	4	6.35	0.444
0.05	0	ptsI	4	6.6	0.483
0.05	0	clcB	4	6.6	0.365
0.05	0	ycaM	4	6.6	0.482
0.05	0	yadI	4	6.6	0.409
0.05	0	AG1	4	6.6	0.455
0.05	0	ptsI	4	6.85	0.495
0.05	0	clcB	4	6.85	0.373
0.05	0	ycaM	4	6.85	0.493
0.05	0	yadI	4	6.85	0.418
0.05	0	AG1	4	6.85	0.468
0.05	0	ptsI	4	7.1	0.503
0.05	0	clcB	4	7.1	0.38
0.05	0	ycaM	4	7.1	0.506
0.05	0	yadI	4	7.1	0.429
0.05	0	AG1	4	7.1	0.482
0.05	0	ptsI	4	7.35	0.514
0.05	0	clcB	4	7.35	0.387
0.05	0	ycaM	4	7.35	0.517
0.05	0	yadI	4	7.35	0.435
0.05	0	AG1	4	7.35	0.497
0.05	0	ptsI	4	7.6	0.521
0.05	0	clcB	4	7.6	0.397
0.05	0	ycaM	4	7.6	0.524
0.05	0	yadI	4	7.6	0.443
0.05	0	AG1	4	7.6	0.506
0.05	0	ptsI	4	7.85	0.528
0.05	0	clcB	4	7.85	0.403
0.05	0	ycaM	4	7.85	0.528
0.05	0	yadI	4	7.85	0.448
0.05	0	AG1	4	7.85	0.516
0.05	0	ptsI	4	8.1	0.537
0.05	0	clcB	4	8.1	0.412
0.05	0	ycaM	4	8.1	0.536
0.05	0	yadI	4	8.1	0.465
0.05	0	AG1	4	8.1	0.529
0.05	0	ptsI	4	8.35	0.547
0.05	0	clcB	4	8.35	0.419
0.05	0	ycaM	4	8.35	0.54
0.05	0	yadI	4	8.35	0.476
0.05	0	AG1	4	8.35	0.541
0.05	0	ptsI	4	8.83333	0.39
0.05	0	clcB	4	8.83333	0.412
0.05	0	ycaM	4	8.83333	0.38
0.05	0	yadI	4	8.83333	0.504
0.05	0	AG1	4	8.83333	0.578
0.05	0	ptsI	4	9.08333	0.392
0.05	0	clcB	4	9.08333	0.409

0.05	0	ycaM	4	9.08333	0.379
0.05	0	yadI	4	9.08333	0.498
0.05	0	AG1	4	9.08333	0.573
0.05	0	ptsI	4	9.33333	0.402
0.05	0	clcB	4	9.33333	0.414
0.05	0	ycaM	4	9.33333	0.381
0.05	0	yadI	4	9.33333	0.507
0.05	0	AG1	4	9.33333	0.58
0.05	0	ptsI	4	9.58333	0.407
0.05	0	clcB	4	9.58333	0.42
0.05	0	ycaM	4	9.58333	0.382
0.05	0	yadI	4	9.58333	0.517
0.05	0	AG1	4	9.58333	0.594
0.05	0	ptsI	4	9.83333	0.417
0.05	0	clcB	4	9.83333	0.425
0.05	0	ycaM	4	9.83333	0.39
0.05	0	yadI	4	9.83333	0.523
0.05	0	AG1	4	9.83333	0.605
0.05	0	ptsI	4	10.0833	0.423
0.05	0	clcB	4	10.0833	0.43
0.05	0	ycaM	4	10.0833	0.393
0.05	0	yadI	4	10.0833	0.534
0.05	0	AG1	4	10.0833	0.614
0.05	0	ptsI	4	10.3333	0.43
0.05	0	clcB	4	10.3333	0.436
0.05	0	ycaM	4	10.3333	0.398
0.05	0	yadI	4	10.3333	0.539
0.05	0	AG1	4	10.3333	0.624
0.05	0	ptsI	4	10.5833	0.437
0.05	0	clcB	4	10.5833	0.44
0.05	0	ycaM	4	10.5833	0.401
0.05	0	yadI	4	10.5833	0.547
0.05	0	AG1	4	10.5833	0.63
0.05	0	ptsI	4	10.8333	0.445
0.05	0	clcB	4	10.8333	0.445
0.05	0	ycaM	4	10.8333	0.406
0.05	0	yadI	4	10.8333	0.551
0.05	0	AG1	4	10.8333	0.642
0.05	0	ptsI	4	11.0833	0.448
0.05	0	clcB	4	11.0833	0.449
0.05	0	ycaM	4	11.0833	0.407
0.05	0	yadI	4	11.0833	0.558
0.05	0	AG1	4	11.0833	0.649
0.05	0	ptsI	4	11.3333	0.46
0.05	0	clcB	4	11.3333	0.461
0.05	0	ycaM	4	11.3333	0.416
0.05	0	yadI	4	11.3333	0.566
0.05	0	AG1	4	11.3333	0.66
0.05	0	ptsI	4	11.5833	0.462
0.05	0	clcB	4	11.5833	0.458
0.05	0	ycaM	4	11.5833	0.419
0.05	0	yadI	4	11.5833	0.57
0.05	0	AG1	4	11.5833	0.663

0.05	0	ptsI	4	11.8333	0.466
0.05	0	clcB	4	11.8333	0.462
0.05	0	ycaM	4	11.8333	0.422
0.05	0	yadI	4	11.8333	0.575
0.05	0	AG1	4	11.8333	0.67
0.05	0	ptsI	4	12.0833	0.47
0.05	0	clcB	4	12.0833	0.469
0.05	0	ycaM	4	12.0833	0.427
0.05	0	yadI	4	12.0833	0.584
0.05	0	AG1	4	12.0833	0.679
0.05	0	ptsI	4	12.3333	0.48
0.05	0	clcB	4	12.3333	0.474
0.05	0	ycaM	4	12.3333	0.432
0.05	0	yadI	4	12.3333	0.586
0.05	0	AG1	4	12.3333	0.69
0.05	0	ptsI	4	12.5833	0.485
0.05	0	clcB	4	12.5833	0.478
0.05	0	ycaM	4	12.5833	0.437
0.05	0	yadI	4	12.5833	0.589
0.05	0	AG1	4	12.5833	0.693
0.05	0	ptsI	4	12.8333	0.484
0.05	0	clcB	4	12.8333	0.479
0.05	0	ycaM	4	12.8333	0.443
0.05	0	yadI	4	12.8333	0.595
0.05	0	AG1	4	12.8333	0.698
0.05	0	ptsI	4	13.0833	0.493
0.05	0	clcB	4	13.0833	0.487
0.05	0	ycaM	4	13.0833	0.446
0.05	0	yadI	4	13.0833	0.598
0.05	0	AG1	4	13.0833	0.705
0.05	0	ptsI	4	13.3333	0.5
0.05	0	clcB	4	13.3333	0.488
0.05	0	ycaM	4	13.3333	0.452
0.05	0	yadI	4	13.3333	0.603
0.05	0	AG1	4	13.3333	0.711
0.05	0	ptsI	4	13.5833	0.506
0.05	0	clcB	4	13.5833	0.493
0.05	0	ycaM	4	13.5833	0.455
0.05	0	yadI	4	13.5833	0.608
0.05	0	AG1	4	13.5833	0.716
0.05	0	ptsI	4	13.8333	0.508
0.05	0	clcB	4	13.8333	0.495
0.05	0	ycaM	4	13.8333	0.458
0.05	0	yadI	4	13.8333	0.612
0.05	0	AG1	4	13.8333	0.718
0.05	0	ptsI	4	14.0833	0.515
0.05	0	clcB	4	14.0833	0.497
0.05	0	ycaM	4	14.0833	0.462
0.05	0	yadI	4	14.0833	0.614
0.05	0	AG1	4	14.0833	0.726
0.05	0	ptsI	4	14.3333	0.522
0.05	0	clcB	4	14.3333	0.502
0.05	0	ycaM	4	14.3333	0.468

0.05	0	yadI	4	14.3333	0.621
0.05	0	AG1	4	14.3333	0.732
0.05	0	ptsI	4	14.5833	0.525
0.05	0	clcB	4	14.5833	0.501
0.05	0	ycaM	4	14.5833	0.469
0.05	0	yadI	4	14.5833	0.619
0.05	0	AG1	4	14.5833	0.732
0.05	0	ptsI	4	14.8333	0.532
0.05	0	clcB	4	14.8333	0.506
0.05	0	ycaM	4	14.8333	0.473
0.05	0	yadI	4	14.8333	0.624
0.05	0	AG1	4	14.8333	0.738
0.05	0	ptsI	4	15.0833	0.534
0.05	0	clcB	4	15.0833	0.506
0.05	0	ycaM	4	15.0833	0.474
0.05	0	yadI	4	15.0833	0.628
0.05	0	AG1	4	15.0833	0.74
0.05	0	ptsI	4	15.3333	0.541
0.05	0	clcB	4	15.3333	0.508
0.05	0	ycaM	4	15.3333	0.479
0.05	0	yadI	4	15.3333	0.631
0.05	0	AG1	4	15.3333	0.742
0.05	0	ptsI	4	15.5833	0.548
0.05	0	clcB	4	15.5833	0.512
0.05	0	ycaM	4	15.5833	0.482
0.05	0	yadI	4	15.5833	0.633
0.05	0	AG1	4	15.5833	0.747
0.05	0	ptsI	4	15.8333	0.545
0.05	0	clcB	4	15.8333	0.511
0.05	0	ycaM	4	15.8333	0.484
0.05	0	yadI	4	15.8333	0.634
0.05	0	AG1	4	15.8333	0.748
0.05	0	ptsI	4	16.0833	0.558
0.05	0	clcB	4	16.0833	0.516
0.05	0	ycaM	4	16.0833	0.487
0.05	0	yadI	4	16.0833	0.64
0.05	0	AG1	4	16.0833	0.752
0.05	0	ptsI	4	16.3333	0.552
0.05	0	clcB	4	16.3333	0.512
0.05	0	ycaM	4	16.3333	0.488
0.05	0	yadI	4	16.3333	0.644
0.05	0	AG1	4	16.3333	0.749
0.05	0	ptsI	4	16.5833	0.559
0.05	0	clcB	4	16.5833	0.516
0.05	0	ycaM	4	16.5833	0.493
0.05	0	yadI	4	16.5833	0.646
0.05	0	AG1	4	16.5833	0.755
0.05	0	ptsI	4	16.8333	0.566
0.05	0	clcB	4	16.8333	0.517
0.05	0	ycaM	4	16.8333	0.49
0.05	0	yadI	4	16.8333	0.647
0.05	0	AG1	4	16.8333	0.755
0.05	0	ptsI	4	17.0833	0.568

0.05	0	clcB	4	17.0833	0.519
0.05	0	ycaM	4	17.0833	0.491
0.05	0	yadI	4	17.0833	0.65
0.05	0	AG1	4	17.0833	0.755
0.05	0	ptsI	4	17.3333	0.574
0.05	0	clcB	4	17.3333	0.524
0.05	0	ycaM	4	17.3333	0.499
0.05	0	yadI	4	17.3333	0.657
0.05	0	AG1	4	17.3333	0.759
0.05	0	ptsI	4	17.5833	0.577
0.05	0	clcB	4	17.5833	0.522
0.05	0	ycaM	4	17.5833	0.495
0.05	0	yadI	4	17.5833	0.655
0.05	0	AG1	4	17.5833	0.757
0.05	0	ptsI	4	17.8333	0.58
0.05	0	clcB	4	17.8333	0.522
0.05	0	ycaM	4	17.8333	0.499
0.05	0	yadI	4	17.8333	0.655
0.05	0	AG1	4	17.8333	0.762
0.05	0	ptsI	4	18.0833	0.582
0.05	0	clcB	4	18.0833	0.523
0.05	0	ycaM	4	18.0833	0.5
0.05	0	yadI	4	18.0833	0.661
0.05	0	AG1	4	18.0833	0.762
0.05	0	ptsI	4	18.3333	0.583
0.05	0	clcB	4	18.3333	0.525
0.05	0	ycaM	4	18.3333	0.502
0.05	0	yadI	4	18.3333	0.662
0.05	0	AG1	4	18.3333	0.764
0.05	0	ptsI	4	18.5833	0.586
0.05	0	clcB	4	18.5833	0.525
0.05	0	ycaM	4	18.5833	0.501
0.05	0	yadI	4	18.5833	0.665
0.05	0	AG1	4	18.5833	0.766
0.05	0	ptsI	4	18.8333	0.589
0.05	0	clcB	4	18.8333	0.522
0.05	0	ycaM	4	18.8333	0.503
0.05	0	yadI	4	18.8333	0.663
0.05	0	AG1	4	18.8333	0.766
0.05	0	ptsI	4	19.0833	0.595
0.05	0	clcB	4	19.0833	0.529
0.05	0	ycaM	4	19.0833	0.506
0.05	0	yadI	4	19.0833	0.669
0.05	0	AG1	4	19.0833	0.769
0.05	0	ptsI	4	19.3333	0.596
0.05	0	clcB	4	19.3333	0.529
0.05	0	ycaM	4	19.3333	0.512
0.05	0	yadI	4	19.3333	0.675
0.05	0	AG1	4	19.3333	0.77
0.05	0	ptsI	4	19.5833	0.604
0.05	0	clcB	4	19.5833	0.532
0.05	0	ycaM	4	19.5833	0.511
0.05	0	yadI	4	19.5833	0.676

0.05	0	AG1	4	19.5833	0.773
0.05	0	ptsI	4	19.8333	0.602
0.05	0	clcB	4	19.8333	0.529
0.05	0	ycaM	4	19.8333	0.509
0.05	0	yadI	4	19.8333	0.672
0.05	0	AG1	4	19.8333	0.773
0.05	0	ptsI	4	20.0833	0.607
0.05	0	clcB	4	20.0833	0.529
0.05	0	ycaM	4	20.0833	0.513
0.05	0	yadI	4	20.0833	0.678
0.05	0	AG1	4	20.0833	0.768
0.05	0	ptsI	4	20.3333	0.606
0.05	0	clcB	4	20.3333	0.532
0.05	0	ycaM	4	20.3333	0.516
0.05	0	yadI	4	20.3333	0.676
0.05	0	AG1	4	20.3333	0.776
0.05	0	ptsI	4	20.5833	0.612
0.05	0	clcB	4	20.5833	0.53
0.05	0	ycaM	4	20.5833	0.516
0.05	0	yadI	4	20.5833	0.682
0.05	0	AG1	4	20.5833	0.778
0.05	0	ptsI	4	20.8333	0.613
0.05	0	clcB	4	20.8333	0.535
0.05	0	ycaM	4	20.8333	0.523
0.05	0	yadI	4	20.8333	0.681
0.05	0	AG1	4	20.8333	0.779
0.05	0	ptsI	4	21.0833	0.62
0.05	0	clcB	4	21.0833	0.534
0.05	0	ycaM	4	21.0833	0.522
0.05	0	yadI	4	21.0833	0.681
0.05	0	AG1	4	21.0833	0.781
0.05	0	ptsI	4	21.3333	0.62
0.05	0	clcB	4	21.3333	0.534
0.05	0	ycaM	4	21.3333	0.52
0.05	0	yadI	4	21.3333	0.681
0.05	0	AG1	4	21.3333	0.778
0.05	0	ptsI	4	21.5833	0.625
0.05	0	clcB	4	21.5833	0.534
0.05	0	ycaM	4	21.5833	0.525
0.05	0	yadI	4	21.5833	0.684
0.05	0	AG1	4	21.5833	0.78
0.05	0	ptsI	4	21.8333	0.621
0.05	0	clcB	4	21.8333	0.534
0.05	0	ycaM	4	21.8333	0.525
0.05	0	yadI	4	21.8333	0.687
0.05	0	AG1	4	21.8333	0.782
0.05	0	ptsI	4	22.0833	0.631
0.05	0	clcB	4	22.0833	0.536
0.05	0	ycaM	4	22.0833	0.526
0.05	0	yadI	4	22.0833	0.686
0.05	0	AG1	4	22.0833	0.781
0.05	0	ptsI	4	22.3333	0.637
0.05	0	clcB	4	22.3333	0.534

0.05	0	ycaM	4	22.3333	0.527
0.05	0	yadI	4	22.3333	0.686
0.05	0	AG1	4	22.3333	0.777
0.05	0	ptsI	4	22.5833	0.635
0.05	0	clcB	4	22.5833	0.533
0.05	0	ycaM	4	22.5833	0.525
0.05	0	yadI	4	22.5833	0.686
0.05	0	AG1	4	22.5833	0.779
0.05	0	ptsI	4	22.8333	0.638
0.05	0	clcB	4	22.8333	0.534
0.05	0	ycaM	4	22.8333	0.528
0.05	0	yadI	4	22.8333	0.686
0.05	0	AG1	4	22.8333	0.78
0.05	0	ptsI	4	23.0833	0.644
0.05	0	clcB	4	23.0833	0.536
0.05	0	ycaM	4	23.0833	0.531
0.05	0	yadI	4	23.0833	0.687
0.05	0	AG1	4	23.0833	0.781
0.05	0	ptsI	4	23.3333	0.643
0.05	0	clcB	4	23.3333	0.535
0.05	0	ycaM	4	23.3333	0.532
0.05	0	yadI	4	23.3333	0.688
0.05	0	AG1	4	23.3333	0.781
0.05	0	ptsI	4	23.5833	0.649
0.05	0	clcB	4	23.5833	0.535
0.05	0	ycaM	4	23.5833	0.532
0.05	0	yadI	4	23.5833	0.688
0.05	0	AG1	4	23.5833	0.781
0.05	0	ptsI	4	23.8333	0.655
0.05	0	clcB	4	23.8333	0.535
0.05	0	ycaM	4	23.8333	0.533
0.05	0	yadI	4	23.8333	0.689
0.05	0	AG1	4	23.8333	0.782
0.05	0	ptsI	4	24.0833	0.657
0.05	0	clcB	4	24.0833	0.535
0.05	0	ycaM	4	24.0833	0.534
0.05	0	yadI	4	24.0833	0.691
0.05	0	AG1	4	24.0833	0.78
0.05	0	ptsI	5	0	0.186
0.05	0	clcB	5	0	0.163
0.05	0	ycaM	5	0	0.176
0.05	0	yadI	5	0	0.17
0.05	0	AG1	5	0	0.19
0.05	0	ptsI	5	0.35	0.175
0.05	0	clcB	5	0.35	0.16
0.05	0	ycaM	5	0.35	0.167
0.05	0	yadI	5	0.35	0.166
0.05	0	AG1	5	0.35	0.187
0.05	0	ptsI	5	0.6	0.173
0.05	0	clcB	5	0.6	0.161
0.05	0	ycaM	5	0.6	0.168
0.05	0	yadI	5	0.6	0.166
0.05	0	AG1	5	0.6	0.187

0.05	0	ptsI	5	0.85	0.178
0.05	0	clcB	5	0.85	0.163
0.05	0	ycaM	5	0.85	0.19
0.05	0	yadI	5	0.85	0.167
0.05	0	AG1	5	0.85	0.188
0.05	0	ptsI	5	1.1	0.18
0.05	0	clcB	5	1.1	0.166
0.05	0	ycaM	5	1.1	0.228
0.05	0	yadI	5	1.1	0.168
0.05	0	AG1	5	1.1	0.188
0.05	0	ptsI	5	1.35	0.168
0.05	0	clcB	5	1.35	0.168
0.05	0	ycaM	5	1.35	0.172
0.05	0	yadI	5	1.35	0.172
0.05	0	AG1	5	1.35	0.192
0.05	0	ptsI	5	1.6	0.174
0.05	0	clcB	5	1.6	0.173
0.05	0	ycaM	5	1.6	0.181
0.05	0	yadI	5	1.6	0.174
0.05	0	AG1	5	1.6	0.196
0.05	0	ptsI	5	1.85	0.192
0.05	0	clcB	5	1.85	0.179
0.05	0	ycaM	5	1.85	0.182
0.05	0	yadI	5	1.85	0.179
0.05	0	AG1	5	1.85	0.201
0.05	0	ptsI	5	2.1	0.205
0.05	0	clcB	5	2.1	0.186
0.05	0	ycaM	5	2.1	0.184
0.05	0	yadI	5	2.1	0.183
0.05	0	AG1	5	2.1	0.207
0.05	0	ptsI	5	2.35	0.196
0.05	0	clcB	5	2.35	0.195
0.05	0	ycaM	5	2.35	0.19
0.05	0	yadI	5	2.35	0.19
0.05	0	AG1	5	2.35	0.216
0.05	0	ptsI	5	2.6	0.209
0.05	0	clcB	5	2.6	0.2
0.05	0	ycaM	5	2.6	0.201
0.05	0	yadI	5	2.6	0.195
0.05	0	AG1	5	2.6	0.212
0.05	0	ptsI	5	2.85	0.214
0.05	0	clcB	5	2.85	0.217
0.05	0	ycaM	5	2.85	0.208
0.05	0	yadI	5	2.85	0.205
0.05	0	AG1	5	2.85	0.228
0.05	0	ptsI	5	3.1	0.22
0.05	0	clcB	5	3.1	0.231
0.05	0	ycaM	5	3.1	0.218
0.05	0	yadI	5	3.1	0.215
0.05	0	AG1	5	3.1	0.238
0.05	0	ptsI	5	3.35	0.227
0.05	0	clcB	5	3.35	0.244
0.05	0	ycaM	5	3.35	0.245

0.05	0	yadI	5	3.35	0.23
0.05	0	AG1	5	3.35	0.255
0.05	0	ptsI	5	3.6	0.238
0.05	0	clcB	5	3.6	0.258
0.05	0	ycaM	5	3.6	0.246
0.05	0	yadI	5	3.6	0.246
0.05	0	AG1	5	3.6	0.277
0.05	0	ptsI	5	3.85	0.253
0.05	0	clcB	5	3.85	0.274
0.05	0	ycaM	5	3.85	0.252
0.05	0	yadI	5	3.85	0.263
0.05	0	AG1	5	3.85	0.299
0.05	0	ptsI	5	4.38333	0.249
0.05	0	clcB	5	4.38333	0.246
0.05	0	ycaM	5	4.38333	0.259
0.05	0	yadI	5	4.38333	0.273
0.05	0	AG1	5	4.38333	0.33
0.05	0	ptsI	5	4.63333	0.23
0.05	0	clcB	5	4.63333	0.248
0.05	0	ycaM	5	4.63333	0.261
0.05	0	yadI	5	4.63333	0.276
0.05	0	AG1	5	4.63333	0.328
0.05	0	ptsI	5	4.88333	0.233
0.05	0	clcB	5	4.88333	0.265
0.05	0	ycaM	5	4.88333	0.271
0.05	0	yadI	5	4.88333	0.287
0.05	0	AG1	5	4.88333	0.343
0.05	0	ptsI	5	5.13333	0.241
0.05	0	clcB	5	5.13333	0.278
0.05	0	ycaM	5	5.13333	0.28
0.05	0	yadI	5	5.13333	0.299
0.05	0	AG1	5	5.13333	0.352
0.05	0	ptsI	5	5.38333	0.248
0.05	0	clcB	5	5.38333	0.293
0.05	0	ycaM	5	5.38333	0.291
0.05	0	yadI	5	5.38333	0.314
0.05	0	AG1	5	5.38333	0.367
0.05	0	ptsI	5	5.63333	0.257
0.05	0	clcB	5	5.63333	0.304
0.05	0	ycaM	5	5.63333	0.304
0.05	0	yadI	5	5.63333	0.331
0.05	0	AG1	5	5.63333	0.38
0.05	0	ptsI	5	5.88333	0.267
0.05	0	clcB	5	5.88333	0.314
0.05	0	ycaM	5	5.88333	0.317
0.05	0	yadI	5	5.88333	0.346
0.05	0	AG1	5	5.88333	0.393
0.05	0	ptsI	5	6.13333	0.277
0.05	0	clcB	5	6.13333	0.326
0.05	0	ycaM	5	6.13333	0.329
0.05	0	yadI	5	6.13333	0.366
0.05	0	AG1	5	6.13333	0.408
0.05	0	ptsI	5	6.38333	0.288

0.05	0	clcB	5	6.38333	0.336
0.05	0	ycaM	5	6.38333	0.343
0.05	0	yadI	5	6.38333	0.386
0.05	0	AG1	5	6.38333	0.431
0.05	0	ptsI	5	6.63333	0.304
0.05	0	clcB	5	6.63333	0.348
0.05	0	ycaM	5	6.63333	0.356
0.05	0	yadI	5	6.63333	0.409
0.05	0	AG1	5	6.63333	0.45
0.05	0	ptsI	5	6.88333	0.317
0.05	0	clcB	5	6.88333	0.359
0.05	0	ycaM	5	6.88333	0.368
0.05	0	yadI	5	6.88333	0.429
0.05	0	AG1	5	6.88333	0.476
0.05	0	ptsI	5	7.13333	0.329
0.05	0	clcB	5	7.13333	0.371
0.05	0	ycaM	5	7.13333	0.382
0.05	0	yadI	5	7.13333	0.453
0.05	0	AG1	5	7.13333	0.497
0.05	0	ptsI	5	7.38333	0.348
0.05	0	clcB	5	7.38333	0.382
0.05	0	ycaM	5	7.38333	0.392
0.05	0	yadI	5	7.38333	0.478
0.05	0	AG1	5	7.38333	0.515
0.05	0	ptsI	5	7.63333	0.364
0.05	0	clcB	5	7.63333	0.395
0.05	0	ycaM	5	7.63333	0.403
0.05	0	yadI	5	7.63333	0.502
0.05	0	AG1	5	7.63333	0.537
0.05	0	ptsI	5	7.88333	0.373
0.05	0	clcB	5	7.88333	0.405
0.05	0	ycaM	5	7.88333	0.412
0.05	0	yadI	5	7.88333	0.515
0.05	0	AG1	5	7.88333	0.549
0.05	0	ptsI	5	8.13333	0.387
0.05	0	clcB	5	8.13333	0.417
0.05	0	ycaM	5	8.13333	0.423
0.05	0	yadI	5	8.13333	0.526
0.05	0	AG1	5	8.13333	0.568
0.05	0	ptsI	5	8.38333	0.395
0.05	0	clcB	5	8.38333	0.427
0.05	0	ycaM	5	8.38333	0.435
0.05	0	yadI	5	8.38333	0.539
0.05	0	AG1	5	8.38333	0.581
0.05	0	ptsI	5	8.88333	0.378
0.05	0	clcB	5	8.88333	0.354
0.05	0	ycaM	5	8.88333	0.362
0.05	0	yadI	5	8.88333	0.5
0.05	0	AG1	5	8.88333	0.609
0.05	0	ptsI	5	9.13333	0.379
0.05	0	clcB	5	9.13333	0.356
0.05	0	ycaM	5	9.13333	0.367
0.05	0	yadI	5	9.13333	0.502

0.05	0	AG1	5	9.13333	0.608
0.05	0	ptsI	5	9.38333	0.388
0.05	0	clcB	5	9.38333	0.366
0.05	0	ycaM	5	9.38333	0.373
0.05	0	yadI	5	9.38333	0.506
0.05	0	AG1	5	9.38333	0.614
0.05	0	ptsI	5	9.63333	0.405
0.05	0	clcB	5	9.63333	0.375
0.05	0	ycaM	5	9.63333	0.384
0.05	0	yadI	5	9.63333	0.522
0.05	0	AG1	5	9.63333	0.637
0.05	0	ptsI	5	9.88333	0.414
0.05	0	clcB	5	9.88333	0.385
0.05	0	ycaM	5	9.88333	0.395
0.05	0	yadI	5	9.88333	0.534
0.05	0	AG1	5	9.88333	0.652
0.05	0	ptsI	5	10.1333	0.421
0.05	0	clcB	5	10.1333	0.392
0.05	0	ycaM	5	10.1333	0.396
0.05	0	yadI	5	10.1333	0.538
0.05	0	AG1	5	10.1333	0.662
0.05	0	ptsI	5	10.3833	0.433
0.05	0	clcB	5	10.3833	0.398
0.05	0	ycaM	5	10.3833	0.403
0.05	0	yadI	5	10.3833	0.547
0.05	0	AG1	5	10.3833	0.676
0.05	0	ptsI	5	10.6333	0.44
0.05	0	clcB	5	10.6333	0.404
0.05	0	ycaM	5	10.6333	0.403
0.05	0	yadI	5	10.6333	0.556
0.05	0	AG1	5	10.6333	0.685
0.05	0	ptsI	5	10.8833	0.452
0.05	0	clcB	5	10.8833	0.413
0.05	0	ycaM	5	10.8833	0.408
0.05	0	yadI	5	10.8833	0.569
0.05	0	AG1	5	10.8833	0.699
0.05	0	ptsI	5	11.1333	0.458
0.05	0	clcB	5	11.1333	0.414
0.05	0	ycaM	5	11.1333	0.417
0.05	0	yadI	5	11.1333	0.576
0.05	0	AG1	5	11.1333	0.71
0.05	0	ptsI	5	11.3833	0.47
0.05	0	clcB	5	11.3833	0.42
0.05	0	ycaM	5	11.3833	0.415
0.05	0	yadI	5	11.3833	0.583
0.05	0	AG1	5	11.3833	0.722
0.05	0	ptsI	5	11.6333	0.478
0.05	0	clcB	5	11.6333	0.426
0.05	0	ycaM	5	11.6333	0.42
0.05	0	yadI	5	11.6333	0.593
0.05	0	AG1	5	11.6333	0.735
0.05	0	ptsI	5	11.8833	0.486
0.05	0	clcB	5	11.8833	0.431

0.05	0	ycaM	5	11.8833	0.422
0.05	0	yadI	5	11.8833	0.601
0.05	0	AG1	5	11.8833	0.743
0.05	0	ptsI	5	12.1333	0.501
0.05	0	clcB	5	12.1333	0.437
0.05	0	ycaM	5	12.1333	0.429
0.05	0	yadI	5	12.1333	0.611
0.05	0	AG1	5	12.1333	0.754
0.05	0	ptsI	5	12.3833	0.516
0.05	0	clcB	5	12.3833	0.438
0.05	0	ycaM	5	12.3833	0.429
0.05	0	yadI	5	12.3833	0.603
0.05	0	AG1	5	12.3833	0.747
0.05	0	ptsI	5	12.6333	0.514
0.05	0	clcB	5	12.6333	0.443
0.05	0	ycaM	5	12.6333	0.438
0.05	0	yadI	5	12.6333	0.621
0.05	0	AG1	5	12.6333	0.771
0.05	0	ptsI	5	12.8833	0.529
0.05	0	clcB	5	12.8833	0.451
0.05	0	ycaM	5	12.8833	0.448
0.05	0	yadI	5	12.8833	0.63
0.05	0	AG1	5	12.8833	0.783
0.05	0	ptsI	5	13.1333	0.533
0.05	0	clcB	5	13.1333	0.455
0.05	0	ycaM	5	13.1333	0.449
0.05	0	yadI	5	13.1333	0.634
0.05	0	AG1	5	13.1333	0.789
0.05	0	ptsI	5	13.3833	0.543
0.05	0	clcB	5	13.3833	0.457
0.05	0	ycaM	5	13.3833	0.447
0.05	0	yadI	5	13.3833	0.642
0.05	0	AG1	5	13.3833	0.795
0.05	0	ptsI	5	13.6333	0.554
0.05	0	clcB	5	13.6333	0.463
0.05	0	ycaM	5	13.6333	0.452
0.05	0	yadI	5	13.6333	0.648
0.05	0	AG1	5	13.6333	0.804
0.05	0	ptsI	5	13.8833	0.555
0.05	0	clcB	5	13.8833	0.466
0.05	0	ycaM	5	13.8833	0.452
0.05	0	yadI	5	13.8833	0.651
0.05	0	AG1	5	13.8833	0.81
0.05	0	ptsI	5	14.1333	0.567
0.05	0	clcB	5	14.1333	0.472
0.05	0	ycaM	5	14.1333	0.458
0.05	0	yadI	5	14.1333	0.659
0.05	0	AG1	5	14.1333	0.816
0.05	0	ptsI	5	14.3833	0.58
0.05	0	clcB	5	14.3833	0.479
0.05	0	ycaM	5	14.3833	0.462
0.05	0	yadI	5	14.3833	0.666
0.05	0	AG1	5	14.3833	0.824

0.05	0	ptsI	5	14.6333	0.584
0.05	0	clcB	5	14.6333	0.482
0.05	0	ycaM	5	14.6333	0.466
0.05	0	yadI	5	14.6333	0.671
0.05	0	AG1	5	14.6333	0.833
0.05	0	ptsI	5	14.8833	0.589
0.05	0	clcB	5	14.8833	0.485
0.05	0	ycaM	5	14.8833	0.469
0.05	0	yadI	5	14.8833	0.673
0.05	0	AG1	5	14.8833	0.835
0.05	0	ptsI	5	15.1333	0.602
0.05	0	clcB	5	15.1333	0.487
0.05	0	ycaM	5	15.1333	0.473
0.05	0	yadI	5	15.1333	0.678
0.05	0	AG1	5	15.1333	0.841
0.05	0	ptsI	5	15.3833	0.596
0.05	0	clcB	5	15.3833	0.492
0.05	0	ycaM	5	15.3833	0.479
0.05	0	yadI	5	15.3833	0.684
0.05	0	AG1	5	15.3833	0.849
0.05	0	ptsI	5	15.6333	0.599
0.05	0	clcB	5	15.6333	0.491
0.05	0	ycaM	5	15.6333	0.479
0.05	0	yadI	5	15.6333	0.685
0.05	0	AG1	5	15.6333	0.848
0.05	0	ptsI	5	15.8833	0.603
0.05	0	clcB	5	15.8833	0.493
0.05	0	ycaM	5	15.8833	0.482
0.05	0	yadI	5	15.8833	0.69
0.05	0	AG1	5	15.8833	0.854
0.05	0	ptsI	5	16.1333	0.601
0.05	0	clcB	5	16.1333	0.494
0.05	0	ycaM	5	16.1333	0.486
0.05	0	yadI	5	16.1333	0.69
0.05	0	AG1	5	16.1333	0.848
0.05	0	ptsI	5	16.3833	0.621
0.05	0	clcB	5	16.3833	0.496
0.05	0	ycaM	5	16.3833	0.483
0.05	0	yadI	5	16.3833	0.696
0.05	0	AG1	5	16.3833	0.861
0.05	0	ptsI	5	16.6333	0.627
0.05	0	clcB	5	16.6333	0.496
0.05	0	ycaM	5	16.6333	0.489
0.05	0	yadI	5	16.6333	0.697
0.05	0	AG1	5	16.6333	0.864
0.05	0	ptsI	5	16.8833	0.626
0.05	0	clcB	5	16.8833	0.504
0.05	0	ycaM	5	16.8833	0.494
0.05	0	yadI	5	16.8833	0.702
0.05	0	AG1	5	16.8833	0.872
0.05	0	ptsI	5	17.1333	0.639
0.05	0	clcB	5	17.1333	0.504
0.05	0	ycaM	5	17.1333	0.498

0.05	0	yadI	5	17.1333	0.706
0.05	0	AG1	5	17.1333	0.875
0.05	0	ptsI	5	17.3833	0.641
0.05	0	clcB	5	17.3833	0.499
0.05	0	ycaM	5	17.3833	0.495
0.05	0	yadI	5	17.3833	0.707
0.05	0	AG1	5	17.3833	0.877
0.05	0	ptsI	5	17.6333	0.644
0.05	0	clcB	5	17.6333	0.5
0.05	0	ycaM	5	17.6333	0.501
0.05	0	yadI	5	17.6333	0.708
0.05	0	AG1	5	17.6333	0.877
0.05	0	ptsI	5	17.8833	0.657
0.05	0	clcB	5	17.8833	0.506
0.05	0	ycaM	5	17.8833	0.507
0.05	0	yadI	5	17.8833	0.701
0.05	0	AG1	5	17.8833	0.869
0.05	0	ptsI	5	18.1333	0.65
0.05	0	clcB	5	18.1333	0.509
0.05	0	ycaM	5	18.1333	0.51
0.05	0	yadI	5	18.1333	0.714
0.05	0	AG1	5	18.1333	0.886
0.05	0	ptsI	5	18.3833	0.651
0.05	0	clcB	5	18.3833	0.509
0.05	0	ycaM	5	18.3833	0.51
0.05	0	yadI	5	18.3833	0.718
0.05	0	AG1	5	18.3833	0.886
0.05	0	ptsI	5	18.6333	0.659
0.05	0	clcB	5	18.6333	0.511
0.05	0	ycaM	5	18.6333	0.511
0.05	0	yadI	5	18.6333	0.717
0.05	0	AG1	5	18.6333	0.887
0.05	0	ptsI	5	18.8833	0.666
0.05	0	clcB	5	18.8833	0.509
0.05	0	ycaM	5	18.8833	0.518
0.05	0	yadI	5	18.8833	0.717
0.05	0	AG1	5	18.8833	0.89
0.05	0	ptsI	5	19.1333	0.676
0.05	0	clcB	5	19.1333	0.512
0.05	0	ycaM	5	19.1333	0.516
0.05	0	yadI	5	19.1333	0.722
0.05	0	AG1	5	19.1333	0.893
0.05	0	ptsI	5	19.3833	0.669
0.05	0	clcB	5	19.3833	0.517
0.05	0	ycaM	5	19.3833	0.524
0.05	0	yadI	5	19.3833	0.724
0.05	0	AG1	5	19.3833	0.895
0.05	0	ptsI	5	19.6333	0.678
0.05	0	clcB	5	19.6333	0.515
0.05	0	ycaM	5	19.6333	0.53
0.05	0	yadI	5	19.6333	0.726
0.05	0	AG1	5	19.6333	0.898
0.05	0	ptsI	5	19.8833	0.684

0.05	0	clcB	5	19.8833	0.516
0.05	0	ycaM	5	19.8833	0.518
0.05	0	yadI	5	19.8833	0.729
0.05	0	AG1	5	19.8833	0.9
0.05	0	ptsI	5	20.1333	0.686
0.05	0	clcB	5	20.1333	0.517
0.05	0	ycaM	5	20.1333	0.52
0.05	0	yadI	5	20.1333	0.731
0.05	0	AG1	5	20.1333	0.901
0.05	0	ptsI	5	20.3833	0.693
0.05	0	clcB	5	20.3833	0.517
0.05	0	ycaM	5	20.3833	0.525
0.05	0	yadI	5	20.3833	0.734
0.05	0	AG1	5	20.3833	0.903
0.05	0	ptsI	5	20.6333	0.699
0.05	0	clcB	5	20.6333	0.52
0.05	0	ycaM	5	20.6333	0.529
0.05	0	yadI	5	20.6333	0.721
0.05	0	AG1	5	20.6333	0.895
0.05	0	ptsI	5	20.8833	0.704
0.05	0	clcB	5	20.8833	0.518
0.05	0	ycaM	5	20.8833	0.524
0.05	0	yadI	5	20.8833	0.735
0.05	0	AG1	5	20.8833	0.905
0.05	0	ptsI	5	21.1333	0.709
0.05	0	clcB	5	21.1333	0.521
0.05	0	ycaM	5	21.1333	0.534
0.05	0	yadI	5	21.1333	0.735
0.05	0	AG1	5	21.1333	0.907
0.05	0	ptsI	5	21.3833	0.71
0.05	0	clcB	5	21.3833	0.521
0.05	0	ycaM	5	21.3833	0.536
0.05	0	yadI	5	21.3833	0.738
0.05	0	AG1	5	21.3833	0.907
0.05	0	ptsI	5	21.6333	0.714
0.05	0	clcB	5	21.6333	0.523
0.05	0	ycaM	5	21.6333	0.533
0.05	0	yadI	5	21.6333	0.738
0.05	0	AG1	5	21.6333	0.911
0.05	0	ptsI	5	21.8833	0.71
0.05	0	clcB	5	21.8833	0.524
0.05	0	ycaM	5	21.8833	0.536
0.05	0	yadI	5	21.8833	0.744
0.05	0	AG1	5	21.8833	0.911
0.05	0	ptsI	5	22.1333	0.73
0.05	0	clcB	5	22.1333	0.523
0.05	0	ycaM	5	22.1333	0.535
0.05	0	yadI	5	22.1333	0.73
0.05	0	AG1	5	22.1333	0.896
0.05	0	ptsI	5	22.3833	0.727
0.05	0	clcB	5	22.3833	0.522
0.05	0	ycaM	5	22.3833	0.542
0.05	0	yadI	5	22.3833	0.741

0.05	0	AG1	5	22.3833	0.91
0.05	0	ptsI	5	22.6333	0.728
0.05	0	clcB	5	22.6333	0.523
0.05	0	ycaM	5	22.6333	0.547
0.05	0	yadI	5	22.6333	0.745
0.05	0	AG1	5	22.6333	0.913
0.05	0	ptsI	5	22.8833	0.732
0.05	0	clcB	5	22.8833	0.522
0.05	0	ycaM	5	22.8833	0.543
0.05	0	yadI	5	22.8833	0.741
0.05	0	AG1	5	22.8833	0.913
0.05	0	ptsI	5	23.1333	0.736
0.05	0	clcB	5	23.1333	0.524
0.05	0	ycaM	5	23.1333	0.541
0.05	0	yadI	5	23.1333	0.746
0.05	0	AG1	5	23.1333	0.915
0.05	0	ptsI	5	23.3833	0.739
0.05	0	clcB	5	23.3833	0.524
0.05	0	ycaM	5	23.3833	0.546
0.05	0	yadI	5	23.3833	0.746
0.05	0	AG1	5	23.3833	0.917
0.05	0	ptsI	5	23.6333	0.742
0.05	0	clcB	5	23.6333	0.525
0.05	0	ycaM	5	23.6333	0.55
0.05	0	yadI	5	23.6333	0.748
0.05	0	AG1	5	23.6333	0.916
0.05	0	ptsI	5	23.8833	0.755
0.05	0	clcB	5	23.8833	0.526
0.05	0	ycaM	5	23.8833	0.549
0.05	0	yadI	5	23.8833	0.733
0.05	0	AG1	5	23.8833	0.902
0.05	0	ptsI	5	24.1333	0.751
0.05	0	clcB	5	24.1333	0.526
0.05	0	ycaM	5	24.1333	0.548
0.05	0	yadI	5	24.1333	0.748
0.05	0	AG1	5	24.1333	0.918
0.05	0	ptsI	5	24.3833	0.76
0.05	0	clcB	5	24.3833	0.527
0.05	0	ycaM	5	24.3833	0.552
0.05	0	yadI	5	24.3833	0.747
0.05	0	AG1	5	24.3833	0.915
0.1	0	ptsI	1	0	0.223
0.1	0	clcB	1	0	0.232
0.1	0	ycaM	1	0	0.239
0.1	0	yadI	1	0	0.252
0.1	0	AG1	1	0	0.236
0.1	0	ptsI	1	0.25	0.218
0.1	0	clcB	1	0.25	0.224
0.1	0	ycaM	1	0.25	0.231
0.1	0	yadI	1	0.25	0.242
0.1	0	AG1	1	0.25	0.228
0.1	0	ptsI	1	0.5	0.218
0.1	0	clcB	1	0.5	0.218

0.1	0	ycaM	1	0.5	0.226
0.1	0	yadI	1	0.5	0.24
0.1	0	AG1	1	0.5	0.224
0.1	0	ptsI	1	0.75	0.22
0.1	0	clcB	1	0.75	0.219
0.1	0	ycaM	1	0.75	0.219
0.1	0	yadI	1	0.75	0.239
0.1	0	AG1	1	0.75	0.221
0.1	0	ptsI	1	1	0.223
0.1	0	clcB	1	1	0.22
0.1	0	ycaM	1	1	0.221
0.1	0	yadI	1	1	0.242
0.1	0	AG1	1	1	0.227
0.1	0	ptsI	1	1.25	0.224
0.1	0	clcB	1	1.25	0.217
0.1	0	ycaM	1	1.25	0.223
0.1	0	yadI	1	1.25	0.242
0.1	0	AG1	1	1.25	0.226
0.1	0	ptsI	1	1.5	0.229
0.1	0	clcB	1	1.5	0.223
0.1	0	ycaM	1	1.5	0.242
0.1	0	yadI	1	1.5	0.256
0.1	0	AG1	1	1.5	0.229
0.1	0	ptsI	1	1.75	0.237
0.1	0	clcB	1	1.75	0.223
0.1	0	ycaM	1	1.75	0.23
0.1	0	yadI	1	1.75	0.255
0.1	0	AG1	1	1.75	0.232
0.1	0	ptsI	1	2	0.243
0.1	0	clcB	1	2	0.23
0.1	0	ycaM	1	2	0.238
0.1	0	yadI	1	2	0.262
0.1	0	AG1	1	2	0.238
0.1	0	ptsI	1	2.25	0.251
0.1	0	clcB	1	2.25	0.228
0.1	0	ycaM	1	2.25	0.243
0.1	0	yadI	1	2.25	0.268
0.1	0	AG1	1	2.25	0.239
0.1	0	ptsI	1	2.5	0.258
0.1	0	clcB	1	2.5	0.234
0.1	0	ycaM	1	2.5	0.25
0.1	0	yadI	1	2.5	0.278
0.1	0	AG1	1	2.5	0.245
0.1	0	ptsI	1	2.75	0.267
0.1	0	clcB	1	2.75	0.235
0.1	0	ycaM	1	2.75	0.256
0.1	0	yadI	1	2.75	0.286
0.1	0	AG1	1	2.75	0.248
0.1	0	ptsI	1	3	0.274
0.1	0	clcB	1	3	0.245
0.1	0	ycaM	1	3	0.264
0.1	0	yadI	1	3	0.298
0.1	0	AG1	1	3	0.254

0.1	0	ptsI	1	3.25	0.284
0.1	0	clcB	1	3.25	0.25
0.1	0	ycaM	1	3.25	0.273
0.1	0	yadI	1	3.25	0.314
0.1	0	AG1	1	3.25	0.26
0.1	0	ptsI	1	3.5	0.307
0.1	0	clcB	1	3.5	0.249
0.1	0	ycaM	1	3.5	0.297
0.1	0	yadI	1	3.5	0.331
0.1	0	AG1	1	3.5	0.264
0.1	0	ptsI	1	3.75	0.308
0.1	0	clcB	1	3.75	0.259
0.1	0	ycaM	1	3.75	0.3
0.1	0	yadI	1	3.75	0.35
0.1	0	AG1	1	3.75	0.269
0.1	0	ptsI	1	4	0.323
0.1	0	clcB	1	4	0.264
0.1	0	ycaM	1	4	0.313
0.1	0	yadI	1	4	0.382
0.1	0	AG1	1	4	0.283
0.1	0	ptsI	1	4.25	0.327
0.1	0	clcB	1	4.25	0.27
0.1	0	ycaM	1	4.25	0.317
0.1	0	yadI	1	4.25	0.406
0.1	0	AG1	1	4.25	0.287
0.1	0	ptsI	1	4.5	0.337
0.1	0	clcB	1	4.5	0.278
0.1	0	ycaM	1	4.5	0.327
0.1	0	yadI	1	4.5	0.414
0.1	0	AG1	1	4.5	0.3
0.1	0	ptsI	1	4.75	0.35
0.1	0	clcB	1	4.75	0.284
0.1	0	ycaM	1	4.75	0.338
0.1	0	yadI	1	4.75	0.423
0.1	0	AG1	1	4.75	0.306
0.1	0	ptsI	1	5	0.359
0.1	0	clcB	1	5	0.296
0.1	0	ycaM	1	5	0.359
0.1	0	yadI	1	5	0.446
0.1	0	AG1	1	5	0.314
0.1	0	ptsI	1	5.25	0.371
0.1	0	clcB	1	5.25	0.304
0.1	0	ycaM	1	5.25	0.366
0.1	0	yadI	1	5.25	0.462
0.1	0	AG1	1	5.25	0.32
0.1	0	ptsI	1	5.5	0.379
0.1	0	clcB	1	5.5	0.316
0.1	0	ycaM	1	5.5	0.368
0.1	0	yadI	1	5.5	0.47
0.1	0	AG1	1	5.5	0.337
0.1	0	ptsI	1	5.75	0.391
0.1	0	clcB	1	5.75	0.322
0.1	0	ycaM	1	5.75	0.376

0.1	0	yadI	1	5.75	0.483
0.1	0	AG1	1	5.75	0.348
0.1	0	ptsI	1	6	0.407
0.1	0	clcB	1	6	0.328
0.1	0	ycaM	1	6	0.387
0.1	0	yadI	1	6	0.485
0.1	0	AG1	1	6	0.353
0.1	0	ptsI	1	6.25	0.422
0.1	0	clcB	1	6.25	0.338
0.1	0	ycaM	1	6.25	0.393
0.1	0	yadI	1	6.25	0.514
0.1	0	AG1	1	6.25	0.366
0.1	0	ptsI	1	6.5	0.43
0.1	0	clcB	1	6.5	0.348
0.1	0	ycaM	1	6.5	0.401
0.1	0	yadI	1	6.5	0.516
0.1	0	AG1	1	6.5	0.378
0.1	0	ptsI	1	6.75	0.439
0.1	0	clcB	1	6.75	0.361
0.1	0	ycaM	1	6.75	0.409
0.1	0	yadI	1	6.75	0.534
0.1	0	AG1	1	6.75	0.395
0.1	0	ptsI	1	7	0.445
0.1	0	clcB	1	7	0.368
0.1	0	ycaM	1	7	0.412
0.1	0	yadI	1	7	0.553
0.1	0	AG1	1	7	0.406
0.1	0	ptsI	1	7.25	0.451
0.1	0	clcB	1	7.25	0.378
0.1	0	ycaM	1	7.25	0.417
0.1	0	yadI	1	7.25	0.564
0.1	0	AG1	1	7.25	0.42
0.1	0	ptsI	1	7.5	0.458
0.1	0	clcB	1	7.5	0.385
0.1	0	ycaM	1	7.5	0.42
0.1	0	yadI	1	7.5	0.57
0.1	0	AG1	1	7.5	0.429
0.1	0	ptsI	1	7.75	0.474
0.1	0	clcB	1	7.75	0.397
0.1	0	ycaM	1	7.75	0.432
0.1	0	yadI	1	7.75	0.585
0.1	0	AG1	1	7.75	0.438
0.1	0	ptsI	1	8	0.486
0.1	0	clcB	1	8	0.405
0.1	0	ycaM	1	8	0.442
0.1	0	yadI	1	8	0.6
0.1	0	AG1	1	8	0.452
0.1	0	ptsI	1	8.25	0.498
0.1	0	clcB	1	8.25	0.415
0.1	0	ycaM	1	8.25	0.449
0.1	0	yadI	1	8.25	0.613
0.1	0	AG1	1	8.25	0.458
0.1	0	ptsI	1	8.5	0.511

0.1	0	clcB	1	8.5	0.423
0.1	0	ycaM	1	8.5	0.462
0.1	0	yadI	1	8.5	0.624
0.1	0	AG1	1	8.5	0.474
0.1	0	ptsI	1	8.75	0.519
0.1	0	clcB	1	8.75	0.434
0.1	0	ycaM	1	8.75	0.469
0.1	0	yadI	1	8.75	0.631
0.1	0	AG1	1	8.75	0.484
0.1	0	ptsI	1	9	0.533
0.1	0	clcB	1	9	0.441
0.1	0	ycaM	1	9	0.481
0.1	0	yadI	1	9	0.647
0.1	0	AG1	1	9	0.495
0.1	0	ptsI	1	9.25	0.556
0.1	0	clcB	1	9.25	0.454
0.1	0	ycaM	1	9.25	0.492
0.1	0	yadI	1	9.25	0.651
0.1	0	AG1	1	9.25	0.514
0.1	0	ptsI	1	9.5	0.579
0.1	0	clcB	1	9.5	0.474
0.1	0	ycaM	1	9.5	0.509
0.1	0	yadI	1	9.5	0.659
0.1	0	AG1	1	9.5	0.531
0.1	0	ptsI	1	9.75	0.567
0.1	0	clcB	1	9.75	0.479
0.1	0	ycaM	1	9.75	0.536
0.1	0	yadI	1	9.75	0.681
0.1	0	AG1	1	9.75	0.545
0.1	0	ptsI	1	10	0.591
0.1	0	clcB	1	10	0.486
0.1	0	ycaM	1	10	0.527
0.1	0	yadI	1	10	0.67
0.1	0	AG1	1	10	0.55
0.1	0	ptsI	1	10.25	0.596
0.1	0	clcB	1	10.25	0.502
0.1	0	ycaM	1	10.25	0.543
0.1	0	yadI	1	10.25	0.692
0.1	0	AG1	1	10.25	0.565
0.1	0	ptsI	1	10.5	0.606
0.1	0	clcB	1	10.5	0.511
0.1	0	ycaM	1	10.5	0.573
0.1	0	yadI	1	10.5	0.707
0.1	0	AG1	1	10.5	0.573
0.1	0	ptsI	1	10.75	0.639
0.1	0	clcB	1	10.75	0.543
0.1	0	ycaM	1	10.75	0.558
0.1	0	yadI	1	10.75	0.715
0.1	0	AG1	1	10.75	0.583
0.1	0	ptsI	1	11	0.652
0.1	0	clcB	1	11	0.558
0.1	0	ycaM	1	11	0.603
0.1	0	yadI	1	11	0.713

0.1	0	AG1	1	11	0.607
0.1	0	ptsI	1	11.25	0.674
0.1	0	clcB	1	11.25	0.557
0.1	0	ycaM	1	11.25	0.614
0.1	0	yadI	1	11.25	0.729
0.1	0	AG1	1	11.25	0.626
0.1	0	ptsI	1	11.5	0.694
0.1	0	clcB	1	11.5	0.568
0.1	0	ycaM	1	11.5	0.608
0.1	0	yadI	1	11.5	0.734
0.1	0	AG1	1	11.5	0.634
0.1	0	ptsI	1	11.75	0.728
0.1	0	clcB	1	11.75	0.589
0.1	0	ycaM	1	11.75	0.628
0.1	0	yadI	1	11.75	0.754
0.1	0	AG1	1	11.75	0.671
0.1	0	ptsI	1	12	0.713
0.1	0	clcB	1	12	0.575
0.1	0	ycaM	1	12	0.631
0.1	0	yadI	1	12	0.778
0.1	0	AG1	1	12	0.678
0.1	0	ptsI	1	12.25	0.726
0.1	0	clcB	1	12.25	0.596
0.1	0	ycaM	1	12.25	0.639
0.1	0	yadI	1	12.25	0.776
0.1	0	AG1	1	12.25	0.668
0.1	0	ptsI	1	12.5	0.727
0.1	0	clcB	1	12.5	0.608
0.1	0	ycaM	1	12.5	0.648
0.1	0	yadI	1	12.5	0.795
0.1	0	AG1	1	12.5	0.696
0.1	0	ptsI	1	12.75	0.747
0.1	0	clcB	1	12.75	0.612
0.1	0	ycaM	1	12.75	0.668
0.1	0	yadI	1	12.75	0.812
0.1	0	AG1	1	12.75	0.715
0.1	0	ptsI	1	13	0.755
0.1	0	clcB	1	13	0.616
0.1	0	ycaM	1	13	0.668
0.1	0	yadI	1	13	0.823
0.1	0	AG1	1	13	0.732
0.1	0	ptsI	1	13.25	0.758
0.1	0	clcB	1	13.25	0.646
0.1	0	ycaM	1	13.25	0.666
0.1	0	yadI	1	13.25	0.835
0.1	0	AG1	1	13.25	0.724
0.1	0	ptsI	1	13.5	0.768
0.1	0	clcB	1	13.5	0.651
0.1	0	ycaM	1	13.5	0.698
0.1	0	yadI	1	13.5	0.866
0.1	0	AG1	1	13.5	0.748
0.1	0	ptsI	1	13.75	0.784
0.1	0	clcB	1	13.75	0.655

0.1	0	ycaM	1	13.75	0.688
0.1	0	yadI	1	13.75	0.856
0.1	0	AG1	1	13.75	0.744
0.1	0	ptsI	1	14	0.811
0.1	0	clcB	1	14	0.668
0.1	0	ycaM	1	14	0.727
0.1	0	yadI	1	14	0.883
0.1	0	AG1	1	14	0.779
0.1	0	ptsI	1	14.25	0.812
0.1	0	clcB	1	14.25	0.672
0.1	0	ycaM	1	14.25	0.728
0.1	0	yadI	1	14.25	0.89
0.1	0	AG1	1	14.25	0.773
0.1	0	ptsI	1	14.5	0.814
0.1	0	clcB	1	14.5	0.682
0.1	0	ycaM	1	14.5	0.69
0.1	0	yadI	1	14.5	0.898
0.1	0	AG1	1	14.5	0.778
0.1	0	ptsI	1	14.75	0.808
0.1	0	clcB	1	14.75	0.704
0.1	0	ycaM	1	14.75	0.704
0.1	0	yadI	1	14.75	0.885
0.1	0	AG1	1	14.75	0.786
0.1	0	ptsI	1	15	0.814
0.1	0	clcB	1	15	0.697
0.1	0	ycaM	1	15	0.716
0.1	0	yadI	1	15	0.896
0.1	0	AG1	1	15	0.791
0.1	0	ptsI	1	15.25	0.835
0.1	0	clcB	1	15.25	0.718
0.1	0	ycaM	1	15.25	0.713
0.1	0	yadI	1	15.25	0.91
0.1	0	AG1	1	15.25	0.826
0.1	0	ptsI	1	15.5	0.83
0.1	0	clcB	1	15.5	0.742
0.1	0	ycaM	1	15.5	0.722
0.1	0	yadI	1	15.5	0.921
0.1	0	AG1	1	15.5	0.799
0.1	0	ptsI	1	15.75	0.832
0.1	0	clcB	1	15.75	0.734
0.1	0	ycaM	1	15.75	0.727
0.1	0	yadI	1	15.75	0.927
0.1	0	AG1	1	15.75	0.812
0.1	0	ptsI	1	16	0.836
0.1	0	clcB	1	16	0.76
0.1	0	ycaM	1	16	0.735
0.1	0	yadI	1	16	0.931
0.1	0	AG1	1	16	0.816
0.1	0	ptsI	1	16.25	0.845
0.1	0	clcB	1	16.25	0.779
0.1	0	ycaM	1	16.25	0.743
0.1	0	yadI	1	16.25	0.937
0.1	0	AG1	1	16.25	0.833

0.1	0	ptsI	1	16.5	0.859
0.1	0	clcB	1	16.5	0.788
0.1	0	ycaM	1	16.5	0.748
0.1	0	yadI	1	16.5	0.933
0.1	0	AG1	1	16.5	0.838
0.1	0	ptsI	1	17.0667	0.912
0.1	0	clcB	1	17.0667	0.8
0.1	0	ycaM	1	17.0667	0.789
0.1	0	yadI	1	17.0667	1.005
0.1	0	AG1	1	17.0667	0.829
0.1	0	ptsI	1	17.3167	0.831
0.1	0	clcB	1	17.3167	0.748
0.1	0	ycaM	1	17.3167	0.736
0.1	0	yadI	1	17.3167	0.968
0.1	0	AG1	1	17.3167	0.777
0.1	0	ptsI	1	17.5667	0.837
0.1	0	clcB	1	17.5667	0.747
0.1	0	ycaM	1	17.5667	0.732
0.1	0	yadI	1	17.5667	0.959
0.1	0	AG1	1	17.5667	0.813
0.1	0	ptsI	1	17.8167	0.858
0.1	0	clcB	1	17.8167	0.789
0.1	0	ycaM	1	17.8167	0.75
0.1	0	yadI	1	17.8167	0.964
0.1	0	AG1	1	17.8167	0.84
0.1	0	ptsI	1	18.0667	0.851
0.1	0	clcB	1	18.0667	0.748
0.1	0	ycaM	1	18.0667	0.748
0.1	0	yadI	1	18.0667	0.968
0.1	0	AG1	1	18.0667	0.833
0.1	0	ptsI	1	18.3167	0.864
0.1	0	clcB	1	18.3167	0.762
0.1	0	ycaM	1	18.3167	0.751
0.1	0	yadI	1	18.3167	0.965
0.1	0	AG1	1	18.3167	0.835
0.1	0	ptsI	1	18.5667	0.873
0.1	0	clcB	1	18.5667	0.784
0.1	0	ycaM	1	18.5667	0.76
0.1	0	yadI	1	18.5667	0.97
0.1	0	AG1	1	18.5667	0.843
0.1	0	ptsI	1	18.8167	0.878
0.1	0	clcB	1	18.8167	0.782
0.1	0	ycaM	1	18.8167	0.759
0.1	0	yadI	1	18.8167	0.974
0.1	0	AG1	1	18.8167	0.848
0.1	0	ptsI	1	19.0667	0.887
0.1	0	clcB	1	19.0667	0.795
0.1	0	ycaM	1	19.0667	0.764
0.1	0	yadI	1	19.0667	0.981
0.1	0	AG1	1	19.0667	0.854
0.1	0	ptsI	1	19.3167	0.883
0.1	0	clcB	1	19.3167	0.799
0.1	0	ycaM	1	19.3167	0.766

0.1	0	yadI	1	19.3167	0.985
0.1	0	AG1	1	19.3167	0.853
0.1	0	ptsI	1	19.5667	0.898
0.1	0	clcB	1	19.5667	0.815
0.1	0	ycaM	1	19.5667	0.779
0.1	0	yadI	1	19.5667	0.985
0.1	0	AG1	1	19.5667	0.876
0.1	0	ptsI	1	19.8167	0.907
0.1	0	clcB	1	19.8167	0.811
0.1	0	ycaM	1	19.8167	0.781
0.1	0	yadI	1	19.8167	0.989
0.1	0	AG1	1	19.8167	0.87
0.1	0	ptsI	1	20.0667	0.922
0.1	0	clcB	1	20.0667	0.818
0.1	0	ycaM	1	20.0667	0.788
0.1	0	yadI	1	20.0667	0.989
0.1	0	AG1	1	20.0667	0.882
0.1	0	ptsI	1	20.3167	0.925
0.1	0	clcB	1	20.3167	0.819
0.1	0	ycaM	1	20.3167	0.788
0.1	0	yadI	1	20.3167	0.996
0.1	0	AG1	1	20.3167	0.882
0.1	0	ptsI	1	20.5667	0.924
0.1	0	clcB	1	20.5667	0.813
0.1	0	ycaM	1	20.5667	0.792
0.1	0	yadI	1	20.5667	0.999
0.1	0	AG1	1	20.5667	0.879
0.1	0	ptsI	1	20.8167	0.935
0.1	0	clcB	1	20.8167	0.821
0.1	0	ycaM	1	20.8167	0.802
0.1	0	yadI	1	20.8167	1.003
0.1	0	AG1	1	20.8167	0.887
0.1	0	ptsI	1	21.0667	0.951
0.1	0	clcB	1	21.0667	0.827
0.1	0	ycaM	1	21.0667	0.81
0.1	0	yadI	1	21.0667	1.007
0.1	0	AG1	1	21.0667	0.894
0.1	0	ptsI	1	21.3167	0.953
0.1	0	clcB	1	21.3167	0.836
0.1	0	ycaM	1	21.3167	0.813
0.1	0	yadI	1	21.3167	1.013
0.1	0	AG1	1	21.3167	0.904
0.1	0	ptsI	1	21.5667	0.957
0.1	0	clcB	1	21.5667	0.827
0.1	0	ycaM	1	21.5667	0.815
0.1	0	yadI	1	21.5667	1.01
0.1	0	AG1	1	21.5667	0.902
0.1	0	ptsI	1	21.8167	0.968
0.1	0	clcB	1	21.8167	0.835
0.1	0	ycaM	1	21.8167	0.823
0.1	0	yadI	1	21.8167	1.009
0.1	0	AG1	1	21.8167	0.909
0.1	0	ptsI	1	22.0667	0.99

0.1	0	clcB	1	22.0667	0.839
0.1	0	ycaM	1	22.0667	0.829
0.1	0	yadI	1	22.0667	1.013
0.1	0	AG1	1	22.0667	0.913
0.1	0	ptsI	1	22.9333	0.687
0.1	0	clcB	1	22.9333	0.549
0.1	0	ycaM	1	22.9333	0.639
0.1	0	yadI	1	22.9333	0.761
0.1	0	AG1	1	22.9333	0.84
0.1	0	ptsI	1	23.1833	0.702
0.1	0	clcB	1	23.1833	0.603
0.1	0	ycaM	1	23.1833	0.664
0.1	0	yadI	1	23.1833	0.747
0.1	0	AG1	1	23.1833	0.776
0.1	0	ptsI	1	23.4333	0.696
0.1	0	clcB	1	23.4333	0.547
0.1	0	ycaM	1	23.4333	0.677
0.1	0	yadI	1	23.4333	0.751
0.1	0	AG1	1	23.4333	0.787
0.1	0	ptsI	1	23.6833	0.713
0.1	0	clcB	1	23.6833	0.564
0.1	0	ycaM	1	23.6833	0.692
0.1	0	yadI	1	23.6833	0.768
0.1	0	AG1	1	23.6833	0.804
0.1	0	ptsI	1	23.9333	0.742
0.1	0	clcB	1	23.9333	0.56
0.1	0	ycaM	1	23.9333	0.704
0.1	0	yadI	1	23.9333	0.783
0.1	0	AG1	1	23.9333	0.803
0.1	0	ptsI	1	24.1833	0.766
0.1	0	clcB	1	24.1833	0.572
0.1	0	ycaM	1	24.1833	0.711
0.1	0	yadI	1	24.1833	0.802
0.1	0	AG1	1	24.1833	0.814
0.1	0	ptsI	1	24.4333	0.781
0.1	0	clcB	1	24.4333	0.578
0.1	0	ycaM	1	24.4333	0.727
0.1	0	yadI	1	24.4333	0.815
0.1	0	AG1	1	24.4333	0.823
0.1	0	ptsI	1	24.6833	0.788
0.1	0	clcB	1	24.6833	0.58
0.1	0	ycaM	1	24.6833	0.716
0.1	0	yadI	1	24.6833	0.825
0.1	0	AG1	1	24.6833	0.823
0.1	0	ptsI	1	24.9333	0.806
0.1	0	clcB	1	24.9333	0.596
0.1	0	ycaM	1	24.9333	0.722
0.1	0	yadI	1	24.9333	0.842
0.1	0	AG1	1	24.9333	0.829
0.1	0	ptsI	1	25.1833	0.822
0.1	0	clcB	1	25.1833	0.597
0.1	0	ycaM	1	25.1833	0.731
0.1	0	yadI	1	25.1833	0.845

0.1	0	AG1	1	25.1833	0.83
0.1	0	ptsI	1	25.4333	0.835
0.1	0	clcB	1	25.4333	0.608
0.1	0	ycaM	1	25.4333	0.738
0.1	0	yadI	1	25.4333	0.854
0.1	0	AG1	1	25.4333	0.839
0.1	0	ptsI	1	25.6833	0.842
0.1	0	clcB	1	25.6833	0.602
0.1	0	ycaM	1	25.6833	0.744
0.1	0	yadI	1	25.6833	0.862
0.1	0	AG1	1	25.6833	0.842
0.1	0	ptsI	1	25.9333	0.861
0.1	0	clcB	1	25.9333	0.609
0.1	0	ycaM	1	25.9333	0.75
0.1	0	yadI	1	25.9333	0.871
0.1	0	AG1	1	25.9333	0.847
0.1	0	ptsI	1	26.1833	0.864
0.1	0	clcB	1	26.1833	0.62
0.1	0	ycaM	1	26.1833	0.759
0.1	0	yadI	1	26.1833	0.882
0.1	0	AG1	1	26.1833	0.851
0.1	0	ptsI	1	26.4333	0.873
0.1	0	clcB	1	26.4333	0.615
0.1	0	ycaM	1	26.4333	0.761
0.1	0	yadI	1	26.4333	0.885
0.1	0	AG1	1	26.4333	0.843
0.1	0	ptsI	1	26.6833	0.882
0.1	0	clcB	1	26.6833	0.622
0.1	0	ycaM	1	26.6833	0.774
0.1	0	yadI	1	26.6833	0.892
0.1	0	AG1	1	26.6833	0.847
0.1	0	ptsI	1	26.9333	0.89
0.1	0	clcB	1	26.9333	0.624
0.1	0	ycaM	1	26.9333	0.782
0.1	0	yadI	1	26.9333	0.899
0.1	0	AG1	1	26.9333	0.852
0.1	0	ptsI	1	27.1833	0.904
0.1	0	clcB	1	27.1833	0.636
0.1	0	ycaM	1	27.1833	0.79
0.1	0	yadI	1	27.1833	0.906
0.1	0	AG1	1	27.1833	0.856
0.1	0	ptsI	1	27.4333	0.912
0.1	0	clcB	1	27.4333	0.638
0.1	0	ycaM	1	27.4333	0.798
0.1	0	yadI	1	27.4333	0.912
0.1	0	AG1	1	27.4333	0.851
0.1	0	ptsI	1	27.6833	0.922
0.1	0	clcB	1	27.6833	0.641
0.1	0	ycaM	1	27.6833	0.806
0.1	0	yadI	1	27.6833	0.92
0.1	0	AG1	1	27.6833	0.858
0.1	0	ptsI	1	27.9333	0.931
0.1	0	clcB	1	27.9333	0.642

0.1	0	ycaM	1	27.9333	0.817
0.1	0	yadI	1	27.9333	0.912
0.1	0	AG1	1	27.9333	0.856
0.1	0	ptsI	1	28.1833	0.938
0.1	0	clcB	1	28.1833	0.666
0.1	0	ycaM	1	28.1833	0.826
0.1	0	yadI	1	28.1833	0.93
0.1	0	AG1	1	28.1833	0.865
0.1	0	ptsI	1	28.4333	0.947
0.1	0	clcB	1	28.4333	0.659
0.1	0	ycaM	1	28.4333	0.835
0.1	0	yadI	1	28.4333	0.935
0.1	0	AG1	1	28.4333	0.865
0.1	0	ptsI	1	28.6833	0.955
0.1	0	clcB	1	28.6833	0.67
0.1	0	ycaM	1	28.6833	0.845
0.1	0	yadI	1	28.6833	0.94
0.1	0	AG1	1	28.6833	0.866
0.1	0	ptsI	1	28.9333	0.966
0.1	0	clcB	1	28.9333	0.685
0.1	0	ycaM	1	28.9333	0.856
0.1	0	yadI	1	28.9333	0.947
0.1	0	AG1	1	28.9333	0.872
0.1	0	ptsI	1	29.1833	0.969
0.1	0	clcB	1	29.1833	0.679
0.1	0	ycaM	1	29.1833	0.864
0.1	0	yadI	1	29.1833	0.948
0.1	0	AG1	1	29.1833	0.867
0.1	0	ptsI	1	29.4333	0.984
0.1	0	clcB	1	29.4333	0.695
0.1	0	ycaM	1	29.4333	0.876
0.1	0	yadI	1	29.4333	0.959
0.1	0	AG1	1	29.4333	0.873
0.1	0	ptsI	1	29.6833	0.996
0.1	0	clcB	1	29.6833	0.707
0.1	0	ycaM	1	29.6833	0.886
0.1	0	yadI	1	29.6833	0.966
0.1	0	AG1	1	29.6833	0.874
0.1	0	ptsI	1	29.9333	1.01
0.1	0	clcB	1	29.9333	0.71
0.1	0	ycaM	1	29.9333	0.894
0.1	0	yadI	1	29.9333	0.972
0.1	0	AG1	1	29.9333	0.876
0.1	0	ptsI	1	30.1833	1.02
0.1	0	clcB	1	30.1833	0.725
0.1	0	ycaM	1	30.1833	0.909
0.1	0	yadI	1	30.1833	0.98
0.1	0	AG1	1	30.1833	0.878
0.1	0	ptsI	1	30.4333	1.033
0.1	0	clcB	1	30.4333	0.741
0.1	0	ycaM	1	30.4333	0.918
0.1	0	yadI	1	30.4333	0.989
0.1	0	AG1	1	30.4333	0.879

0.1	0	ptsI	1	30.6833	1.036
0.1	0	clcB	1	30.6833	0.751
0.1	0	ycaM	1	30.6833	0.93
0.1	0	yadI	1	30.6833	0.991
0.1	0	AG1	1	30.6833	0.876
0.1	0	ptsI	1	30.9333	1.052
0.1	0	clcB	1	30.9333	0.768
0.1	0	ycaM	1	30.9333	0.942
0.1	0	yadI	1	30.9333	1.006
0.1	0	AG1	1	30.9333	0.879
0.1	0	ptsI	1	31.1833	1.062
0.1	0	clcB	1	31.1833	0.784
0.1	0	ycaM	1	31.1833	0.949
0.1	0	yadI	1	31.1833	1.01
0.1	0	AG1	1	31.1833	0.877
0.1	0	ptsI	1	31.4333	1.075
0.1	0	clcB	1	31.4333	0.804
0.1	0	ycaM	1	31.4333	0.97
0.1	0	yadI	1	31.4333	1.025
0.1	0	AG1	1	31.4333	0.884
0.1	0	ptsI	1	31.6833	1.084
0.1	0	clcB	1	31.6833	0.82
0.1	0	ycaM	1	31.6833	0.978
0.1	0	yadI	1	31.6833	1.029
0.1	0	AG1	1	31.6833	0.882
0.1	0	ptsI	1	31.9333	1.096
0.1	0	clcB	1	31.9333	0.834
0.1	0	ycaM	1	31.9333	0.992
0.1	0	yadI	1	31.9333	1.04
0.1	0	AG1	1	31.9333	0.885
0.1	0	ptsI	1	32.1833	1.106
0.1	0	clcB	1	32.1833	0.849
0.1	0	ycaM	1	32.1833	1
0.1	0	yadI	1	32.1833	1.05
0.1	0	AG1	1	32.1833	0.886
0.1	0	ptsI	1	32.4333	1.12
0.1	0	clcB	1	32.4333	0.859
0.1	0	ycaM	1	32.4333	1.014
0.1	0	yadI	1	32.4333	1.046
0.1	0	AG1	1	32.4333	0.89
0.1	0	ptsI	1	32.6833	1.123
0.1	0	clcB	1	32.6833	0.871
0.1	0	ycaM	1	32.6833	1.021
0.1	0	yadI	1	32.6833	1.065
0.1	0	AG1	1	32.6833	0.889
0.1	0	ptsI	1	32.9333	1.133
0.1	0	clcB	1	32.9333	0.886
0.1	0	ycaM	1	32.9333	1.037
0.1	0	yadI	1	32.9333	1.068
0.1	0	AG1	1	32.9333	0.887
0.1	0	ptsI	1	33.1833	1.148
0.1	0	clcB	1	33.1833	0.894
0.1	0	ycaM	1	33.1833	1.05

0.1	0	yadI	1	33.1833	1.072
0.1	0	AG1	1	33.1833	0.892
0.1	0	ptsI	1	33.4333	1.16
0.1	0	clcB	1	33.4333	0.906
0.1	0	ycaM	1	33.4333	1.056
0.1	0	yadI	1	33.4333	1.093
0.1	0	AG1	1	33.4333	0.892
0.1	0	ptsI	1	33.6833	1.163
0.1	0	clcB	1	33.6833	0.922
0.1	0	ycaM	1	33.6833	1.07
0.1	0	yadI	1	33.6833	1.092
0.1	0	AG1	1	33.6833	0.894
0.1	0	ptsI	1	33.9333	1.172
0.1	0	clcB	1	33.9333	0.932
0.1	0	ycaM	1	33.9333	1.079
0.1	0	yadI	1	33.9333	1.105
0.1	0	AG1	1	33.9333	0.897
0.1	0	ptsI	1	34.1833	1.182
0.1	0	clcB	1	34.1833	0.944
0.1	0	ycaM	1	34.1833	1.093
0.1	0	yadI	1	34.1833	1.119
0.1	0	AG1	1	34.1833	0.903
0.1	0	ptsI	1	34.4333	1.194
0.1	0	clcB	1	34.4333	0.947
0.1	0	ycaM	1	34.4333	1.098
0.1	0	yadI	1	34.4333	1.126
0.1	0	AG1	1	34.4333	0.903
0.1	0	ptsI	1	34.6833	1.205
0.1	0	clcB	1	34.6833	0.964
0.1	0	ycaM	1	34.6833	1.109
0.1	0	yadI	1	34.6833	1.14
0.1	0	AG1	1	34.6833	0.909
0.1	0	ptsI	1	34.9333	1.211
0.1	0	clcB	1	34.9333	0.974
0.1	0	ycaM	1	34.9333	1.116
0.1	0	yadI	1	34.9333	1.137
0.1	0	AG1	1	34.9333	0.905
0.1	0	ptsI	1	35.1833	1.222
0.1	0	clcB	1	35.1833	0.978
0.1	0	ycaM	1	35.1833	1.124
0.1	0	yadI	1	35.1833	1.15
0.1	0	AG1	1	35.1833	0.906
0.1	0	ptsI	1	35.4333	1.23
0.1	0	clcB	1	35.4333	0.991
0.1	0	ycaM	1	35.4333	1.133
0.1	0	yadI	1	35.4333	1.161
0.1	0	AG1	1	35.4333	0.908
0.1	0	ptsI	1	35.6833	1.241
0.1	0	clcB	1	35.6833	0.999
0.1	0	ycaM	1	35.6833	1.142
0.1	0	yadI	1	35.6833	1.174
0.1	0	AG1	1	35.6833	0.918
0.1	0	ptsI	1	35.9333	1.25

0.1	0	clcB	1	35.9333	1.01
0.1	0	ycaM	1	35.9333	1.152
0.1	0	yadI	1	35.9333	1.166
0.1	0	AG1	1	35.9333	0.917
0.1	0	ptsI	1	36.1833	1.255
0.1	0	clcB	1	36.1833	1.016
0.1	0	ycaM	1	36.1833	1.156
0.1	0	yadI	1	36.1833	1.187
0.1	0	AG1	1	36.1833	0.922
0.1	0	ptsI	1	36.4333	1.264
0.1	0	clcB	1	36.4333	1.032
0.1	0	ycaM	1	36.4333	1.161
0.1	0	yadI	1	36.4333	1.195
0.1	0	AG1	1	36.4333	0.921
0.1	0	ptsI	1	36.6833	1.273
0.1	0	clcB	1	36.6833	1.033
0.1	0	ycaM	1	36.6833	1.17
0.1	0	yadI	1	36.6833	1.201
0.1	0	AG1	1	36.6833	0.916
0.1	0	ptsI	2	0	0.222
0.1	0	clcB	2	0	0.206
0.1	0	ycaM	2	0	0.211
0.1	0	yadI	2	0	0.224
0.1	0	AG1	2	0	0.226
0.1	0	ptsI	2	0.25	0.216
0.1	0	clcB	2	0.25	0.199
0.1	0	ycaM	2	0.25	0.21
0.1	0	yadI	2	0.25	0.217
0.1	0	AG1	2	0.25	0.222
0.1	0	ptsI	2	0.5	0.213
0.1	0	clcB	2	0.5	0.2
0.1	0	ycaM	2	0.5	0.208
0.1	0	yadI	2	0.5	0.214
0.1	0	AG1	2	0.5	0.223
0.1	0	ptsI	2	0.75	0.217
0.1	0	clcB	2	0.75	0.201
0.1	0	ycaM	2	0.75	0.21
0.1	0	yadI	2	0.75	0.219
0.1	0	AG1	2	0.75	0.226
0.1	0	ptsI	2	1	0.22
0.1	0	clcB	2	1	0.202
0.1	0	ycaM	2	1	0.215
0.1	0	yadI	2	1	0.224
0.1	0	AG1	2	1	0.227
0.1	0	ptsI	2	1.25	0.22
0.1	0	clcB	2	1.25	0.204
0.1	0	ycaM	2	1.25	0.217
0.1	0	yadI	2	1.25	0.231
0.1	0	AG1	2	1.25	0.231
0.1	0	ptsI	2	1.5	0.224
0.1	0	clcB	2	1.5	0.206
0.1	0	ycaM	2	1.5	0.22
0.1	0	yadI	2	1.5	0.239

0.1	0	AG1	2	1.5	0.236
0.1	0	ptsI	2	1.75	0.228
0.1	0	clcB	2	1.75	0.208
0.1	0	ycaM	2	1.75	0.225
0.1	0	yadI	2	1.75	0.244
0.1	0	AG1	2	1.75	0.239
0.1	0	ptsI	2	2	0.233
0.1	0	clcB	2	2	0.21
0.1	0	ycaM	2	2	0.23
0.1	0	yadI	2	2	0.251
0.1	0	AG1	2	2	0.252
0.1	0	ptsI	2	2.25	0.242
0.1	0	clcB	2	2.25	0.212
0.1	0	ycaM	2	2.25	0.235
0.1	0	yadI	2	2.25	0.263
0.1	0	AG1	2	2.25	0.259
0.1	0	ptsI	2	2.5	0.247
0.1	0	clcB	2	2.5	0.217
0.1	0	ycaM	2	2.5	0.244
0.1	0	yadI	2	2.5	0.274
0.1	0	AG1	2	2.5	0.27
0.1	0	ptsI	2	2.75	0.256
0.1	0	clcB	2	2.75	0.222
0.1	0	ycaM	2	2.75	0.25
0.1	0	yadI	2	2.75	0.289
0.1	0	AG1	2	2.75	0.287
0.1	0	ptsI	2	3	0.267
0.1	0	clcB	2	3	0.23
0.1	0	ycaM	2	3	0.253
0.1	0	yadI	2	3	0.303
0.1	0	AG1	2	3	0.299
0.1	0	ptsI	2	3.25	0.273
0.1	0	clcB	2	3.25	0.232
0.1	0	ycaM	2	3.25	0.266
0.1	0	yadI	2	3.25	0.325
0.1	0	AG1	2	3.25	0.31
0.1	0	ptsI	2	3.5	0.282
0.1	0	clcB	2	3.5	0.236
0.1	0	ycaM	2	3.5	0.272
0.1	0	yadI	2	3.5	0.343
0.1	0	AG1	2	3.5	0.32
0.1	0	ptsI	2	3.75	0.289
0.1	0	clcB	2	3.75	0.241
0.1	0	ycaM	2	3.75	0.284
0.1	0	yadI	2	3.75	0.363
0.1	0	AG1	2	3.75	0.334
0.1	0	ptsI	2	4	0.293
0.1	0	clcB	2	4	0.246
0.1	0	ycaM	2	4	0.293
0.1	0	yadI	2	4	0.385
0.1	0	AG1	2	4	0.344
0.1	0	ptsI	2	4.38333	0.303
0.1	0	clcB	2	4.38333	0.259

0.1	0	ycaM	2	4.38333	0.307
0.1	0	yadI	2	4.38333	0.424
0.1	0	AG1	2	4.38333	0.382
0.1	0	ptsI	2	4.63333	0.296
0.1	0	clcB	2	4.63333	0.256
0.1	0	ycaM	2	4.63333	0.306
0.1	0	yadI	2	4.63333	0.415
0.1	0	AG1	2	4.63333	0.368
0.1	0	ptsI	2	4.88333	0.302
0.1	0	clcB	2	4.88333	0.26
0.1	0	ycaM	2	4.88333	0.314
0.1	0	yadI	2	4.88333	0.423
0.1	0	AG1	2	4.88333	0.378
0.1	0	ptsI	2	5.13333	0.309
0.1	0	clcB	2	5.13333	0.265
0.1	0	ycaM	2	5.13333	0.322
0.1	0	yadI	2	5.13333	0.436
0.1	0	AG1	2	5.13333	0.387
0.1	0	ptsI	2	5.38333	0.316
0.1	0	clcB	2	5.38333	0.274
0.1	0	ycaM	2	5.38333	0.332
0.1	0	yadI	2	5.38333	0.45
0.1	0	AG1	2	5.38333	0.398
0.1	0	ptsI	2	5.63333	0.321
0.1	0	clcB	2	5.63333	0.284
0.1	0	ycaM	2	5.63333	0.341
0.1	0	yadI	2	5.63333	0.46
0.1	0	AG1	2	5.63333	0.407
0.1	0	ptsI	2	5.88333	0.329
0.1	0	clcB	2	5.88333	0.292
0.1	0	ycaM	2	5.88333	0.354
0.1	0	yadI	2	5.88333	0.474
0.1	0	AG1	2	5.88333	0.419
0.1	0	ptsI	2	6.13333	0.334
0.1	0	clcB	2	6.13333	0.297
0.1	0	ycaM	2	6.13333	0.363
0.1	0	yadI	2	6.13333	0.486
0.1	0	AG1	2	6.13333	0.431
0.1	0	ptsI	2	6.38333	0.345
0.1	0	clcB	2	6.38333	0.302
0.1	0	ycaM	2	6.38333	0.374
0.1	0	yadI	2	6.38333	0.498
0.1	0	AG1	2	6.38333	0.445
0.1	0	ptsI	2	6.63333	0.351
0.1	0	clcB	2	6.63333	0.312
0.1	0	ycaM	2	6.63333	0.381
0.1	0	yadI	2	6.63333	0.509
0.1	0	AG1	2	6.63333	0.456
0.1	0	ptsI	2	6.88333	0.359
0.1	0	clcB	2	6.88333	0.319
0.1	0	ycaM	2	6.88333	0.391
0.1	0	yadI	2	6.88333	0.523
0.1	0	AG1	2	6.88333	0.47

0.1	0	ptsI	2	7.13333	0.365
0.1	0	clcB	2	7.13333	0.324
0.1	0	ycaM	2	7.13333	0.394
0.1	0	yadI	2	7.13333	0.529
0.1	0	AG1	2	7.13333	0.48
0.1	0	ptsI	2	7.38333	0.375
0.1	0	clcB	2	7.38333	0.331
0.1	0	ycaM	2	7.38333	0.407
0.1	0	yadI	2	7.38333	0.545
0.1	0	AG1	2	7.38333	0.493
0.1	0	ptsI	2	7.63333	0.379
0.1	0	clcB	2	7.63333	0.339
0.1	0	ycaM	2	7.63333	0.417
0.1	0	yadI	2	7.63333	0.561
0.1	0	AG1	2	7.63333	0.509
0.1	0	ptsI	2	7.88333	0.389
0.1	0	clcB	2	7.88333	0.345
0.1	0	ycaM	2	7.88333	0.426
0.1	0	yadI	2	7.88333	0.576
0.1	0	AG1	2	7.88333	0.521
0.1	0	ptsI	2	8.13333	0.396
0.1	0	clcB	2	8.13333	0.353
0.1	0	ycaM	2	8.13333	0.433
0.1	0	yadI	2	8.13333	0.582
0.1	0	AG1	2	8.13333	0.534
0.1	0	ptsI	2	8.38333	0.401
0.1	0	clcB	2	8.38333	0.361
0.1	0	ycaM	2	8.38333	0.439
0.1	0	yadI	2	8.38333	0.599
0.1	0	AG1	2	8.38333	0.548
0.1	0	ptsI	2	8.91667	0.338
0.1	0	clcB	2	8.91667	0.324
0.1	0	ycaM	2	8.91667	0.361
0.1	0	yadI	2	8.91667	0.568
0.1	0	AG1	2	8.91667	0.479
0.1	0	ptsI	2	9.16667	0.341
0.1	0	clcB	2	9.16667	0.325
0.1	0	ycaM	2	9.16667	0.364
0.1	0	yadI	2	9.16667	0.555
0.1	0	AG1	2	9.16667	0.475
0.1	0	ptsI	2	9.41667	0.346
0.1	0	clcB	2	9.41667	0.329
0.1	0	ycaM	2	9.41667	0.364
0.1	0	yadI	2	9.41667	0.564
0.1	0	AG1	2	9.41667	0.481
0.1	0	ptsI	2	9.66667	0.354
0.1	0	clcB	2	9.66667	0.339
0.1	0	ycaM	2	9.66667	0.37
0.1	0	yadI	2	9.66667	0.574
0.1	0	AG1	2	9.66667	0.496
0.1	0	ptsI	2	9.91667	0.358
0.1	0	clcB	2	9.91667	0.342
0.1	0	ycaM	2	9.91667	0.377

0.1	0	yadI	2	9.91667	0.582
0.1	0	AG1	2	9.91667	0.499
0.1	0	ptsI	2	10.1667	0.365
0.1	0	clcB	2	10.1667	0.348
0.1	0	ycaM	2	10.1667	0.382
0.1	0	yadI	2	10.1667	0.592
0.1	0	AG1	2	10.1667	0.502
0.1	0	ptsI	2	10.4167	0.368
0.1	0	clcB	2	10.4167	0.352
0.1	0	ycaM	2	10.4167	0.389
0.1	0	yadI	2	10.4167	0.601
0.1	0	AG1	2	10.4167	0.508
0.1	0	ptsI	2	10.6667	0.377
0.1	0	clcB	2	10.6667	0.358
0.1	0	ycaM	2	10.6667	0.396
0.1	0	yadI	2	10.6667	0.612
0.1	0	AG1	2	10.6667	0.508
0.1	0	ptsI	2	10.9167	0.384
0.1	0	clcB	2	10.9167	0.362
0.1	0	ycaM	2	10.9167	0.403
0.1	0	yadI	2	10.9167	0.62
0.1	0	AG1	2	10.9167	0.52
0.1	0	ptsI	2	11.1667	0.392
0.1	0	clcB	2	11.1667	0.367
0.1	0	ycaM	2	11.1667	0.409
0.1	0	yadI	2	11.1667	0.63
0.1	0	AG1	2	11.1667	0.544
0.1	0	ptsI	2	11.4167	0.399
0.1	0	clcB	2	11.4167	0.372
0.1	0	ycaM	2	11.4167	0.41
0.1	0	yadI	2	11.4167	0.635
0.1	0	AG1	2	11.4167	0.562
0.1	0	ptsI	2	11.6667	0.409
0.1	0	clcB	2	11.6667	0.381
0.1	0	ycaM	2	11.6667	0.424
0.1	0	yadI	2	11.6667	0.649
0.1	0	AG1	2	11.6667	0.603
0.1	0	ptsI	2	11.9167	0.411
0.1	0	clcB	2	11.9167	0.385
0.1	0	ycaM	2	11.9167	0.434
0.1	0	yadI	2	11.9167	0.658
0.1	0	AG1	2	11.9167	0.611
0.1	0	ptsI	2	12.1667	0.424
0.1	0	clcB	2	12.1667	0.392
0.1	0	ycaM	2	12.1667	0.435
0.1	0	yadI	2	12.1667	0.669
0.1	0	AG1	2	12.1667	0.642
0.1	0	ptsI	2	12.4167	0.458
0.1	0	clcB	2	12.4167	0.397
0.1	0	ycaM	2	12.4167	0.438
0.1	0	yadI	2	12.4167	0.674
0.1	0	AG1	2	12.4167	0.663
0.1	0	ptsI	2	12.6667	0.46

0.1	0	clcB	2	12.6667	0.404
0.1	0	ycaM	2	12.6667	0.431
0.1	0	yadI	2	12.6667	0.683
0.1	0	AG1	2	12.6667	0.681
0.1	0	ptsI	2	12.9167	0.447
0.1	0	clcB	2	12.9167	0.413
0.1	0	ycaM	2	12.9167	0.446
0.1	0	yadI	2	12.9167	0.697
0.1	0	AG1	2	12.9167	0.698
0.1	0	ptsI	2	13.1667	0.449
0.1	0	clcB	2	13.1667	0.414
0.1	0	ycaM	2	13.1667	0.452
0.1	0	yadI	2	13.1667	0.704
0.1	0	AG1	2	13.1667	0.698
0.1	0	ptsI	2	13.4167	0.458
0.1	0	clcB	2	13.4167	0.42
0.1	0	ycaM	2	13.4167	0.449
0.1	0	yadI	2	13.4167	0.714
0.1	0	AG1	2	13.4167	0.717
0.1	0	ptsI	2	13.6667	0.469
0.1	0	clcB	2	13.6667	0.43
0.1	0	ycaM	2	13.6667	0.46
0.1	0	yadI	2	13.6667	0.727
0.1	0	AG1	2	13.6667	0.758
0.1	0	ptsI	2	13.9167	0.477
0.1	0	clcB	2	13.9167	0.44
0.1	0	ycaM	2	13.9167	0.469
0.1	0	yadI	2	13.9167	0.735
0.1	0	AG1	2	13.9167	0.764
0.1	0	ptsI	2	14.1667	0.476
0.1	0	clcB	2	14.1667	0.439
0.1	0	ycaM	2	14.1667	0.465
0.1	0	yadI	2	14.1667	0.748
0.1	0	AG1	2	14.1667	0.764
0.1	0	ptsI	2	14.4167	0.48
0.1	0	clcB	2	14.4167	0.435
0.1	0	ycaM	2	14.4167	0.481
0.1	0	yadI	2	14.4167	0.764
0.1	0	AG1	2	14.4167	0.777
0.1	0	ptsI	2	14.6667	0.482
0.1	0	clcB	2	14.6667	0.441
0.1	0	ycaM	2	14.6667	0.484
0.1	0	yadI	2	14.6667	0.766
0.1	0	AG1	2	14.6667	0.78
0.1	0	ptsI	2	14.9167	0.487
0.1	0	clcB	2	14.9167	0.452
0.1	0	ycaM	2	14.9167	0.479
0.1	0	yadI	2	14.9167	0.779
0.1	0	AG1	2	14.9167	0.79
0.1	0	ptsI	2	15.1667	0.5
0.1	0	clcB	2	15.1667	0.461
0.1	0	ycaM	2	15.1667	0.499
0.1	0	yadI	2	15.1667	0.783

0.1	0	AG1	2	15.1667	0.807
0.1	0	ptsI	2	15.4167	0.508
0.1	0	clcB	2	15.4167	0.459
0.1	0	ycaM	2	15.4167	0.515
0.1	0	yadI	2	15.4167	0.803
0.1	0	AG1	2	15.4167	0.825
0.1	0	ptsI	2	15.6667	0.511
0.1	0	clcB	2	15.6667	0.471
0.1	0	ycaM	2	15.6667	0.514
0.1	0	yadI	2	15.6667	0.804
0.1	0	AG1	2	15.6667	0.829
0.1	0	ptsI	2	15.9167	0.524
0.1	0	clcB	2	15.9167	0.468
0.1	0	ycaM	2	15.9167	0.527
0.1	0	yadI	2	15.9167	0.813
0.1	0	AG1	2	15.9167	0.828
0.1	0	ptsI	2	16.1667	0.538
0.1	0	clcB	2	16.1667	0.476
0.1	0	ycaM	2	16.1667	0.551
0.1	0	yadI	2	16.1667	0.824
0.1	0	AG1	2	16.1667	0.836
0.1	0	ptsI	2	16.4167	0.562
0.1	0	clcB	2	16.4167	0.474
0.1	0	ycaM	2	16.4167	0.536
0.1	0	yadI	2	16.4167	0.829
0.1	0	AG1	2	16.4167	0.844
0.1	0	ptsI	2	16.6667	0.586
0.1	0	clcB	2	16.6667	0.495
0.1	0	ycaM	2	16.6667	0.552
0.1	0	yadI	2	16.6667	0.836
0.1	0	AG1	2	16.6667	0.851
0.1	0	ptsI	2	16.9167	0.602
0.1	0	clcB	2	16.9167	0.488
0.1	0	ycaM	2	16.9167	0.562
0.1	0	yadI	2	16.9167	0.843
0.1	0	AG1	2	16.9167	0.854
0.1	0	ptsI	2	17.1667	0.656
0.1	0	clcB	2	17.1667	0.489
0.1	0	ycaM	2	17.1667	0.549
0.1	0	yadI	2	17.1667	0.852
0.1	0	AG1	2	17.1667	0.854
0.1	0	ptsI	2	17.4167	0.691
0.1	0	clcB	2	17.4167	0.502
0.1	0	ycaM	2	17.4167	0.556
0.1	0	yadI	2	17.4167	0.856
0.1	0	AG1	2	17.4167	0.862
0.1	0	ptsI	2	17.6667	0.713
0.1	0	clcB	2	17.6667	0.5
0.1	0	ycaM	2	17.6667	0.549
0.1	0	yadI	2	17.6667	0.862
0.1	0	AG1	2	17.6667	0.862
0.1	0	ptsI	2	17.9167	0.713
0.1	0	clcB	2	17.9167	0.495

0.1	0	ycaM	2	17.9167	0.552
0.1	0	yadI	2	17.9167	0.872
0.1	0	AG1	2	17.9167	0.87
0.1	0	ptsI	2	18.1667	0.726
0.1	0	clcB	2	18.1667	0.504
0.1	0	ycaM	2	18.1667	0.555
0.1	0	yadI	2	18.1667	0.872
0.1	0	AG1	2	18.1667	0.881
0.1	0	ptsI	2	18.4167	0.742
0.1	0	clcB	2	18.4167	0.508
0.1	0	ycaM	2	18.4167	0.548
0.1	0	yadI	2	18.4167	0.88
0.1	0	AG1	2	18.4167	0.884
0.1	0	ptsI	2	18.6667	0.748
0.1	0	clcB	2	18.6667	0.495
0.1	0	ycaM	2	18.6667	0.556
0.1	0	yadI	2	18.6667	0.884
0.1	0	AG1	2	18.6667	0.893
0.1	0	ptsI	2	18.9167	0.773
0.1	0	clcB	2	18.9167	0.498
0.1	0	ycaM	2	18.9167	0.559
0.1	0	yadI	2	18.9167	0.887
0.1	0	AG1	2	18.9167	0.898
0.1	0	ptsI	2	19.1667	0.775
0.1	0	clcB	2	19.1667	0.499
0.1	0	ycaM	2	19.1667	0.566
0.1	0	yadI	2	19.1667	0.896
0.1	0	AG1	2	19.1667	0.908
0.1	0	ptsI	2	19.4167	0.776
0.1	0	clcB	2	19.4167	0.506
0.1	0	ycaM	2	19.4167	0.574
0.1	0	yadI	2	19.4167	0.9
0.1	0	AG1	2	19.4167	0.912
0.1	0	ptsI	2	19.6667	0.784
0.1	0	clcB	2	19.6667	0.502
0.1	0	ycaM	2	19.6667	0.58
0.1	0	yadI	2	19.6667	0.905
0.1	0	AG1	2	19.6667	0.911
0.1	0	ptsI	2	19.9167	0.801
0.1	0	clcB	2	19.9167	0.507
0.1	0	ycaM	2	19.9167	0.586
0.1	0	yadI	2	19.9167	0.908
0.1	0	AG1	2	19.9167	0.922
0.1	0	ptsI	2	20.1667	0.789
0.1	0	clcB	2	20.1667	0.5
0.1	0	ycaM	2	20.1667	0.587
0.1	0	yadI	2	20.1667	0.912
0.1	0	AG1	2	20.1667	0.914
0.1	0	ptsI	2	20.4167	0.793
0.1	0	clcB	2	20.4167	0.501
0.1	0	ycaM	2	20.4167	0.588
0.1	0	yadI	2	20.4167	0.92
0.1	0	AG1	2	20.4167	0.916

0.1	0	ptsI	2	20.6667	0.791
0.1	0	clcB	2	20.6667	0.507
0.1	0	ycaM	2	20.6667	0.594
0.1	0	yadI	2	20.6667	0.923
0.1	0	AG1	2	20.6667	0.922
0.1	0	ptsI	2	20.9167	0.799
0.1	0	clcB	2	20.9167	0.508
0.1	0	ycaM	2	20.9167	0.605
0.1	0	yadI	2	20.9167	0.93
0.1	0	AG1	2	20.9167	0.924
0.1	0	ptsI	2	21.1667	0.797
0.1	0	clcB	2	21.1667	0.509
0.1	0	ycaM	2	21.1667	0.61
0.1	0	yadI	2	21.1667	0.936
0.1	0	AG1	2	21.1667	0.925
0.1	0	ptsI	2	21.4167	0.798
0.1	0	clcB	2	21.4167	0.508
0.1	0	ycaM	2	21.4167	0.617
0.1	0	yadI	2	21.4167	0.932
0.1	0	AG1	2	21.4167	0.929
0.1	0	ptsI	2	21.6667	0.809
0.1	0	clcB	2	21.6667	0.51
0.1	0	ycaM	2	21.6667	0.623
0.1	0	yadI	2	21.6667	0.949
0.1	0	AG1	2	21.6667	0.941
0.1	0	ptsI	2	21.9167	0.821
0.1	0	clcB	2	21.9167	0.515
0.1	0	ycaM	2	21.9167	0.628
0.1	0	yadI	2	21.9167	0.953
0.1	0	AG1	2	21.9167	0.948
0.1	0	ptsI	2	22.1667	0.82
0.1	0	clcB	2	22.1667	0.512
0.1	0	ycaM	2	22.1667	0.633
0.1	0	yadI	2	22.1667	0.956
0.1	0	AG1	2	22.1667	0.946
0.1	0	ptsI	2	22.4167	0.828
0.1	0	clcB	2	22.4167	0.518
0.1	0	ycaM	2	22.4167	0.635
0.1	0	yadI	2	22.4167	0.962
0.1	0	AG1	2	22.4167	0.951
0.1	0	ptsI	2	22.6667	0.835
0.1	0	clcB	2	22.6667	0.519
0.1	0	ycaM	2	22.6667	0.643
0.1	0	yadI	2	22.6667	0.965
0.1	0	AG1	2	22.6667	0.96
0.1	0	ptsI	2	22.9167	0.844
0.1	0	clcB	2	22.9167	0.525
0.1	0	ycaM	2	22.9167	0.648
0.1	0	yadI	2	22.9167	0.972
0.1	0	AG1	2	22.9167	0.969
0.1	0	ptsI	2	23.1667	0.844
0.1	0	clcB	2	23.1667	0.517
0.1	0	ycaM	2	23.1667	0.645

0.1	0	yadI	2	23.1667	0.976
0.1	0	AG1	2	23.1667	0.962
0.1	0	ptsI	2	23.4167	0.851
0.1	0	clcB	2	23.4167	0.525
0.1	0	ycaM	2	23.4167	0.654
0.1	0	yadI	2	23.4167	0.978
0.1	0	AG1	2	23.4167	0.968
0.1	0	ptsI	2	23.6667	0.855
0.1	0	clcB	2	23.6667	0.53
0.1	0	ycaM	2	23.6667	0.657
0.1	0	yadI	2	23.6667	0.986
0.1	0	AG1	2	23.6667	0.971
0.1	0	ptsI	2	23.9167	0.86
0.1	0	clcB	2	23.9167	0.528
0.1	0	ycaM	2	23.9167	0.665
0.1	0	yadI	2	23.9167	0.99
0.1	0	AG1	2	23.9167	0.977
0.1	0	ptsI	2	24.1667	0.859
0.1	0	clcB	2	24.1667	0.526
0.1	0	ycaM	2	24.1667	0.667
0.1	0	yadI	2	24.1667	0.993
0.1	0	AG1	2	24.1667	0.976
0.1	0	ptsI	2	24.4167	0.863
0.1	0	clcB	2	24.4167	0.527
0.1	0	ycaM	2	24.4167	0.673
0.1	0	yadI	2	24.4167	1.003
0.1	0	AG1	2	24.4167	0.979
0.1	0	ptsI	2	24.6667	0.871
0.1	0	clcB	2	24.6667	0.535
0.1	0	ycaM	2	24.6667	0.676
0.1	0	yadI	2	24.6667	1.003
0.1	0	AG1	2	24.6667	0.989
0.1	0	ptsI	2	24.9167	0.884
0.1	0	clcB	2	24.9167	0.54
0.1	0	ycaM	2	24.9167	0.682
0.1	0	yadI	2	24.9167	1.01
0.1	0	AG1	2	24.9167	0.992
0.1	0	ptsI	2	25.1667	0.878
0.1	0	clcB	2	25.1667	0.531
0.1	0	ycaM	2	25.1667	0.685
0.1	0	yadI	2	25.1667	1.017
0.1	0	AG1	2	25.1667	0.992
0.1	0	ptsI	2	25.4167	0.895
0.1	0	clcB	2	25.4167	0.54
0.1	0	ycaM	2	25.4167	0.692
0.1	0	yadI	2	25.4167	1.019
0.1	0	AG1	2	25.4167	1.003
0.1	0	ptsI	2	25.6667	0.894
0.1	0	clcB	2	25.6667	0.542
0.1	0	ycaM	2	25.6667	0.694
0.1	0	yadI	2	25.6667	1.019
0.1	0	AG1	2	25.6667	1.005
0.1	0	ptsI	2	25.9167	0.899

0.1	0	clcB	2	25.9167	0.537
0.1	0	ycaM	2	25.9167	0.7
0.1	0	yadI	2	25.9167	1.032
0.1	0	AG1	2	25.9167	1.011
0.1	0	ptsI	2	26.1667	0.907
0.1	0	clcB	2	26.1667	0.542
0.1	0	ycaM	2	26.1667	0.706
0.1	0	yadI	2	26.1667	1.025
0.1	0	AG1	2	26.1667	1.013
0.1	0	ptsI	2	26.4167	0.922
0.1	0	clcB	2	26.4167	0.546
0.1	0	ycaM	2	26.4167	0.714
0.1	0	yadI	2	26.4167	1.036
0.1	0	AG1	2	26.4167	1.027
0.1	0	ptsI	3	0	0.147
0.1	0	clcB	3	0	0.161
0.1	0	ycaM	3	0	0.151
0.1	0	yadI	3	0	0.168
0.1	0	AG1	3	0	0.188
0.1	0	ptsI	3	0.25	0.146
0.1	0	clcB	3	0.25	0.154
0.1	0	ycaM	3	0.25	0.15
0.1	0	yadI	3	0.25	0.164
0.1	0	AG1	3	0.25	0.198
0.1	0	ptsI	3	0.5	0.148
0.1	0	clcB	3	0.5	0.152
0.1	0	ycaM	3	0.5	0.148
0.1	0	yadI	3	0.5	0.164
0.1	0	AG1	3	0.5	0.204
0.1	0	ptsI	3	0.75	0.148
0.1	0	clcB	3	0.75	0.152
0.1	0	ycaM	3	0.75	0.148
0.1	0	yadI	3	0.75	0.165
0.1	0	AG1	3	0.75	0.21
0.1	0	ptsI	3	1	0.149
0.1	0	clcB	3	1	0.154
0.1	0	ycaM	3	1	0.15
0.1	0	yadI	3	1	0.165
0.1	0	AG1	3	1	0.206
0.1	0	ptsI	3	1.25	0.148
0.1	0	clcB	3	1.25	0.154
0.1	0	ycaM	3	1.25	0.15
0.1	0	yadI	3	1.25	0.166
0.1	0	AG1	3	1.25	0.21
0.1	0	ptsI	3	1.5	0.15
0.1	0	clcB	3	1.5	0.156
0.1	0	ycaM	3	1.5	0.153
0.1	0	yadI	3	1.5	0.169
0.1	0	AG1	3	1.5	0.215
0.1	0	ptsI	3	1.75	0.15
0.1	0	clcB	3	1.75	0.158
0.1	0	ycaM	3	1.75	0.152
0.1	0	yadI	3	1.75	0.169

0.1	0	AG1	3	1.75	0.221
0.1	0	ptsI	3	2	0.152
0.1	0	clcB	3	2	0.162
0.1	0	ycaM	3	2	0.155
0.1	0	yadI	3	2	0.171
0.1	0	AG1	3	2	0.218
0.1	0	ptsI	3	2.25	0.154
0.1	0	clcB	3	2.25	0.166
0.1	0	ycaM	3	2.25	0.156
0.1	0	yadI	3	2.25	0.175
0.1	0	AG1	3	2.25	0.23
0.1	0	ptsI	3	2.5	0.156
0.1	0	clcB	3	2.5	0.167
0.1	0	ycaM	3	2.5	0.159
0.1	0	yadI	3	2.5	0.176
0.1	0	AG1	3	2.5	0.221
0.1	0	ptsI	3	2.75	0.159
0.1	0	clcB	3	2.75	0.173
0.1	0	ycaM	3	2.75	0.164
0.1	0	yadI	3	2.75	0.181
0.1	0	AG1	3	2.75	0.213
0.1	0	ptsI	3	3	0.16
0.1	0	clcB	3	3	0.173
0.1	0	ycaM	3	3	0.162
0.1	0	yadI	3	3	0.184
0.1	0	AG1	3	3	0.228
0.1	0	ptsI	3	3.25	0.162
0.1	0	clcB	3	3.25	0.175
0.1	0	ycaM	3	3.25	0.165
0.1	0	yadI	3	3.25	0.188
0.1	0	AG1	3	3.25	0.229
0.1	0	ptsI	3	3.5	0.166
0.1	0	clcB	3	3.5	0.181
0.1	0	ycaM	3	3.5	0.17
0.1	0	yadI	3	3.5	0.196
0.1	0	AG1	3	3.5	0.204
0.1	0	ptsI	3	3.75	0.17
0.1	0	clcB	3	3.75	0.186
0.1	0	ycaM	3	3.75	0.173
0.1	0	yadI	3	3.75	0.2
0.1	0	AG1	3	3.75	0.22
0.1	0	ptsI	3	4	0.172
0.1	0	clcB	3	4	0.19
0.1	0	ycaM	3	4	0.177
0.1	0	yadI	3	4	0.208
0.1	0	AG1	3	4	0.207
0.1	0	ptsI	3	4.41667	0.181
0.1	0	clcB	3	4.41667	0.198
0.1	0	ycaM	3	4.41667	0.194
0.1	0	yadI	3	4.41667	0.218
0.1	0	AG1	3	4.41667	0.193
0.1	0	ptsI	3	4.66667	0.18
0.1	0	clcB	3	4.66667	0.198

0.1	0	ycaM	3	4.66667	0.187
0.1	0	yadI	3	4.66667	0.205
0.1	0	AG1	3	4.66667	0.191
0.1	0	ptsI	3	4.91667	0.181
0.1	0	clcB	3	4.91667	0.199
0.1	0	ycaM	3	4.91667	0.188
0.1	0	yadI	3	4.91667	0.206
0.1	0	AG1	3	4.91667	0.194
0.1	0	ptsI	3	5.16667	0.185
0.1	0	clcB	3	5.16667	0.204
0.1	0	ycaM	3	5.16667	0.193
0.1	0	yadI	3	5.16667	0.213
0.1	0	AG1	3	5.16667	0.199
0.1	0	ptsI	3	5.41667	0.191
0.1	0	clcB	3	5.41667	0.21
0.1	0	ycaM	3	5.41667	0.201
0.1	0	yadI	3	5.41667	0.222
0.1	0	AG1	3	5.41667	0.205
0.1	0	ptsI	3	5.66667	0.196
0.1	0	clcB	3	5.66667	0.216
0.1	0	ycaM	3	5.66667	0.207
0.1	0	yadI	3	5.66667	0.235
0.1	0	AG1	3	5.66667	0.212
0.1	0	ptsI	3	5.91667	0.202
0.1	0	clcB	3	5.91667	0.223
0.1	0	ycaM	3	5.91667	0.216
0.1	0	yadI	3	5.91667	0.251
0.1	0	AG1	3	5.91667	0.22
0.1	0	ptsI	3	6.16667	0.209
0.1	0	clcB	3	6.16667	0.232
0.1	0	ycaM	3	6.16667	0.226
0.1	0	yadI	3	6.16667	0.266
0.1	0	AG1	3	6.16667	0.23
0.1	0	ptsI	3	6.41667	0.215
0.1	0	clcB	3	6.41667	0.243
0.1	0	ycaM	3	6.41667	0.238
0.1	0	yadI	3	6.41667	0.276
0.1	0	AG1	3	6.41667	0.239
0.1	0	ptsI	3	6.66667	0.225
0.1	0	clcB	3	6.66667	0.254
0.1	0	ycaM	3	6.66667	0.251
0.1	0	yadI	3	6.66667	0.285
0.1	0	AG1	3	6.66667	0.251
0.1	0	ptsI	3	6.91667	0.236
0.1	0	clcB	3	6.91667	0.266
0.1	0	ycaM	3	6.91667	0.264
0.1	0	yadI	3	6.91667	0.296
0.1	0	AG1	3	6.91667	0.256
0.1	0	ptsI	3	7.16667	0.246
0.1	0	clcB	3	7.16667	0.278
0.1	0	ycaM	3	7.16667	0.274
0.1	0	yadI	3	7.16667	0.304
0.1	0	AG1	3	7.16667	0.262

0.1	0	ptsI	3	7.41667	0.259
0.1	0	clcB	3	7.41667	0.291
0.1	0	ycaM	3	7.41667	0.281
0.1	0	yadI	3	7.41667	0.315
0.1	0	AG1	3	7.41667	0.271
0.1	0	ptsI	3	7.66667	0.268
0.1	0	clcB	3	7.66667	0.301
0.1	0	ycaM	3	7.66667	0.289
0.1	0	yadI	3	7.66667	0.322
0.1	0	AG1	3	7.66667	0.277
0.1	0	ptsI	3	7.91667	0.275
0.1	0	clcB	3	7.91667	0.308
0.1	0	ycaM	3	7.91667	0.297
0.1	0	yadI	3	7.91667	0.334
0.1	0	AG1	3	7.91667	0.283
0.1	0	ptsI	3	8.16667	0.283
0.1	0	clcB	3	8.16667	0.316
0.1	0	ycaM	3	8.16667	0.304
0.1	0	yadI	3	8.16667	0.343
0.1	0	AG1	3	8.16667	0.291
0.1	0	ptsI	3	8.41667	0.29
0.1	0	clcB	3	8.41667	0.324
0.1	0	ycaM	3	8.41667	0.313
0.1	0	yadI	3	8.41667	0.35
0.1	0	AG1	3	8.41667	0.297
0.1	0	ptsI	3	9.05	0.28
0.1	0	clcB	3	9.05	0.291
0.1	0	ycaM	3	9.05	0.295
0.1	0	yadI	3	9.05	0.402
0.1	0	AG1	3	9.05	0.335
0.1	0	ptsI	3	9.3	0.282
0.1	0	clcB	3	9.3	0.293
0.1	0	ycaM	3	9.3	0.299
0.1	0	yadI	3	9.3	0.406
0.1	0	AG1	3	9.3	0.336
0.1	0	ptsI	3	9.55	0.29
0.1	0	clcB	3	9.55	0.3
0.1	0	ycaM	3	9.55	0.305
0.1	0	yadI	3	9.55	0.415
0.1	0	AG1	3	9.55	0.345
0.1	0	ptsI	3	9.8	0.298
0.1	0	clcB	3	9.8	0.305
0.1	0	ycaM	3	9.8	0.312
0.1	0	yadI	3	9.8	0.423
0.1	0	AG1	3	9.8	0.354
0.1	0	ptsI	3	10.05	0.305
0.1	0	clcB	3	10.05	0.313
0.1	0	ycaM	3	10.05	0.32
0.1	0	yadI	3	10.05	0.433
0.1	0	AG1	3	10.05	0.365
0.1	0	ptsI	3	10.3	0.315
0.1	0	clcB	3	10.3	0.319
0.1	0	ycaM	3	10.3	0.328

0.1	0	yadI	3	10.3	0.444
0.1	0	AG1	3	10.3	0.376
0.1	0	ptsI	3	10.55	0.322
0.1	0	clcB	3	10.55	0.328
0.1	0	ycaM	3	10.55	0.336
0.1	0	yadI	3	10.55	0.454
0.1	0	AG1	3	10.55	0.383
0.1	0	ptsI	3	10.8	0.33
0.1	0	clcB	3	10.8	0.34
0.1	0	ycaM	3	10.8	0.348
0.1	0	yadI	3	10.8	0.462
0.1	0	AG1	3	10.8	0.391
0.1	0	ptsI	3	11.05	0.339
0.1	0	clcB	3	11.05	0.349
0.1	0	ycaM	3	11.05	0.352
0.1	0	yadI	3	11.05	0.468
0.1	0	AG1	3	11.05	0.398
0.1	0	ptsI	3	11.3	0.351
0.1	0	clcB	3	11.3	0.356
0.1	0	ycaM	3	11.3	0.359
0.1	0	yadI	3	11.3	0.48
0.1	0	AG1	3	11.3	0.408
0.1	0	ptsI	3	11.55	0.356
0.1	0	clcB	3	11.55	0.366
0.1	0	ycaM	3	11.55	0.361
0.1	0	yadI	3	11.55	0.488
0.1	0	AG1	3	11.55	0.415
0.1	0	ptsI	3	11.8	0.363
0.1	0	clcB	3	11.8	0.374
0.1	0	ycaM	3	11.8	0.366
0.1	0	yadI	3	11.8	0.491
0.1	0	AG1	3	11.8	0.423
0.1	0	ptsI	3	12.05	0.376
0.1	0	clcB	3	12.05	0.376
0.1	0	ycaM	3	12.05	0.368
0.1	0	yadI	3	12.05	0.503
0.1	0	AG1	3	12.05	0.432
0.1	0	ptsI	3	12.3	0.382
0.1	0	clcB	3	12.3	0.385
0.1	0	ycaM	3	12.3	0.374
0.1	0	yadI	3	12.3	0.506
0.1	0	AG1	3	12.3	0.444
0.1	0	ptsI	3	12.55	0.391
0.1	0	clcB	3	12.55	0.392
0.1	0	ycaM	3	12.55	0.378
0.1	0	yadI	3	12.55	0.514
0.1	0	AG1	3	12.55	0.454
0.1	0	ptsI	3	12.8	0.401
0.1	0	clcB	3	12.8	0.399
0.1	0	ycaM	3	12.8	0.383
0.1	0	yadI	3	12.8	0.522
0.1	0	AG1	3	12.8	0.472
0.1	0	ptsI	3	13.05	0.415

0.1	0	clcB	3	13.05	0.407
0.1	0	ycaM	3	13.05	0.388
0.1	0	yadI	3	13.05	0.532
0.1	0	AG1	3	13.05	0.482
0.1	0	ptsI	3	13.3	0.423
0.1	0	clcB	3	13.3	0.424
0.1	0	ycaM	3	13.3	0.397
0.1	0	yadI	3	13.3	0.532
0.1	0	AG1	3	13.3	0.492
0.1	0	ptsI	3	13.55	0.435
0.1	0	clcB	3	13.55	0.431
0.1	0	ycaM	3	13.55	0.403
0.1	0	yadI	3	13.55	0.536
0.1	0	AG1	3	13.55	0.508
0.1	0	ptsI	3	13.8	0.442
0.1	0	clcB	3	13.8	0.436
0.1	0	ycaM	3	13.8	0.414
0.1	0	yadI	3	13.8	0.539
0.1	0	AG1	3	13.8	0.522
0.1	0	ptsI	3	14.05	0.459
0.1	0	clcB	3	14.05	0.447
0.1	0	ycaM	3	14.05	0.417
0.1	0	yadI	3	14.05	0.548
0.1	0	AG1	3	14.05	0.533
0.1	0	ptsI	3	14.3	0.47
0.1	0	clcB	3	14.3	0.458
0.1	0	ycaM	3	14.3	0.422
0.1	0	yadI	3	14.3	0.552
0.1	0	AG1	3	14.3	0.542
0.1	0	ptsI	3	14.55	0.48
0.1	0	clcB	3	14.55	0.466
0.1	0	ycaM	3	14.55	0.426
0.1	0	yadI	3	14.55	0.552
0.1	0	AG1	3	14.55	0.546
0.1	0	ptsI	3	14.8	0.489
0.1	0	clcB	3	14.8	0.478
0.1	0	ycaM	3	14.8	0.435
0.1	0	yadI	3	14.8	0.552
0.1	0	AG1	3	14.8	0.564
0.1	0	ptsI	3	15.05	0.501
0.1	0	clcB	3	15.05	0.49
0.1	0	ycaM	3	15.05	0.442
0.1	0	yadI	3	15.05	0.555
0.1	0	AG1	3	15.05	0.576
0.1	0	ptsI	3	15.3	0.514
0.1	0	clcB	3	15.3	0.494
0.1	0	ycaM	3	15.3	0.444
0.1	0	yadI	3	15.3	0.556
0.1	0	AG1	3	15.3	0.582
0.1	0	ptsI	3	15.55	0.525
0.1	0	clcB	3	15.55	0.502
0.1	0	ycaM	3	15.55	0.448
0.1	0	yadI	3	15.55	0.555

0.1	0	AG1	3	15.55	0.591
0.1	0	ptsI	3	15.8	0.532
0.1	0	clcB	3	15.8	0.51
0.1	0	ycaM	3	15.8	0.451
0.1	0	yadI	3	15.8	0.558
0.1	0	AG1	3	15.8	0.594
0.1	0	ptsI	3	16.05	0.546
0.1	0	clcB	3	16.05	0.518
0.1	0	ycaM	3	16.05	0.456
0.1	0	yadI	3	16.05	0.565
0.1	0	AG1	3	16.05	0.607
0.1	0	ptsI	3	16.3	0.557
0.1	0	clcB	3	16.3	0.53
0.1	0	ycaM	3	16.3	0.457
0.1	0	yadI	3	16.3	0.563
0.1	0	AG1	3	16.3	0.643
0.1	0	ptsI	3	16.55	0.564
0.1	0	clcB	3	16.55	0.529
0.1	0	ycaM	3	16.55	0.465
0.1	0	yadI	3	16.55	0.564
0.1	0	AG1	3	16.55	0.637
0.1	0	ptsI	3	16.8	0.581
0.1	0	clcB	3	16.8	0.539
0.1	0	ycaM	3	16.8	0.47
0.1	0	yadI	3	16.8	0.559
0.1	0	AG1	3	16.8	0.638
0.1	0	ptsI	3	17.05	0.594
0.1	0	clcB	3	17.05	0.542
0.1	0	ycaM	3	17.05	0.47
0.1	0	yadI	3	17.05	0.567
0.1	0	AG1	3	17.05	0.629
0.1	0	ptsI	3	17.3	0.607
0.1	0	clcB	3	17.3	0.553
0.1	0	ycaM	3	17.3	0.478
0.1	0	yadI	3	17.3	0.569
0.1	0	AG1	3	17.3	0.623
0.1	0	ptsI	3	17.55	0.646
0.1	0	clcB	3	17.55	0.56
0.1	0	ycaM	3	17.55	0.483
0.1	0	yadI	3	17.55	0.566
0.1	0	AG1	3	17.55	0.625
0.1	0	ptsI	3	17.8	0.668
0.1	0	clcB	3	17.8	0.564
0.1	0	ycaM	3	17.8	0.485
0.1	0	yadI	3	17.8	0.562
0.1	0	AG1	3	17.8	0.622
0.1	0	ptsI	3	18.05	0.688
0.1	0	clcB	3	18.05	0.579
0.1	0	ycaM	3	18.05	0.488
0.1	0	yadI	3	18.05	0.566
0.1	0	AG1	3	18.05	0.639
0.1	0	ptsI	3	18.3	0.678
0.1	0	clcB	3	18.3	0.589

0.1	0	ycaM	3	18.3	0.496
0.1	0	yadI	3	18.3	0.564
0.1	0	AG1	3	18.3	0.647
0.1	0	ptsI	3	18.55	0.674
0.1	0	clcB	3	18.55	0.585
0.1	0	ycaM	3	18.55	0.5
0.1	0	yadI	3	18.55	0.572
0.1	0	AG1	3	18.55	0.645
0.1	0	ptsI	3	18.8	0.673
0.1	0	clcB	3	18.8	0.586
0.1	0	ycaM	3	18.8	0.505
0.1	0	yadI	3	18.8	0.569
0.1	0	AG1	3	18.8	0.644
0.1	0	ptsI	3	19.05	0.698
0.1	0	clcB	3	19.05	0.595
0.1	0	ycaM	3	19.05	0.51
0.1	0	yadI	3	19.05	0.566
0.1	0	AG1	3	19.05	0.642
0.1	0	ptsI	3	19.3	0.714
0.1	0	clcB	3	19.3	0.605
0.1	0	ycaM	3	19.3	0.513
0.1	0	yadI	3	19.3	0.57
0.1	0	AG1	3	19.3	0.648
0.1	0	ptsI	3	19.55	0.738
0.1	0	clcB	3	19.55	0.628
0.1	0	ycaM	3	19.55	0.518
0.1	0	yadI	3	19.55	0.57
0.1	0	AG1	3	19.55	0.663
0.1	0	ptsI	3	19.8	0.73
0.1	0	clcB	3	19.8	0.642
0.1	0	ycaM	3	19.8	0.52
0.1	0	yadI	3	19.8	0.569
0.1	0	AG1	3	19.8	0.649
0.1	0	ptsI	3	20.05	0.729
0.1	0	clcB	3	20.05	0.642
0.1	0	ycaM	3	20.05	0.525
0.1	0	yadI	3	20.05	0.572
0.1	0	AG1	3	20.05	0.648
0.1	0	ptsI	3	20.3	0.721
0.1	0	clcB	3	20.3	0.64
0.1	0	ycaM	3	20.3	0.528
0.1	0	yadI	3	20.3	0.572
0.1	0	AG1	3	20.3	0.639
0.1	0	ptsI	3	20.55	0.73
0.1	0	clcB	3	20.55	0.654
0.1	0	ycaM	3	20.55	0.536
0.1	0	yadI	3	20.55	0.573
0.1	0	AG1	3	20.55	0.639
0.1	0	ptsI	3	20.8	0.745
0.1	0	clcB	3	20.8	0.66
0.1	0	ycaM	3	20.8	0.541
0.1	0	yadI	3	20.8	0.57
0.1	0	AG1	3	20.8	0.634

0.1	0	ptsI	3	21.05	0.759
0.1	0	clcB	3	21.05	0.662
0.1	0	ycaM	3	21.05	0.544
0.1	0	yadI	3	21.05	0.578
0.1	0	AG1	3	21.05	0.637
0.1	0	ptsI	3	21.3	0.769
0.1	0	clcB	3	21.3	0.676
0.1	0	ycaM	3	21.3	0.548
0.1	0	yadI	3	21.3	0.57
0.1	0	AG1	3	21.3	0.635
0.1	0	ptsI	3	21.55	0.788
0.1	0	clcB	3	21.55	0.684
0.1	0	ycaM	3	21.55	0.555
0.1	0	yadI	3	21.55	0.571
0.1	0	AG1	3	21.55	0.637
0.1	0	ptsI	3	21.8	0.797
0.1	0	clcB	3	21.8	0.688
0.1	0	ycaM	3	21.8	0.556
0.1	0	yadI	3	21.8	0.567
0.1	0	AG1	3	21.8	0.64
0.1	0	ptsI	3	22.05	0.808
0.1	0	clcB	3	22.05	0.696
0.1	0	ycaM	3	22.05	0.562
0.1	0	yadI	3	22.05	0.569
0.1	0	AG1	3	22.05	0.643
0.1	0	ptsI	3	22.3	0.824
0.1	0	clcB	3	22.3	0.701
0.1	0	ycaM	3	22.3	0.563
0.1	0	yadI	3	22.3	0.577
0.1	0	AG1	3	22.3	0.644
0.1	0	ptsI	3	22.55	0.833
0.1	0	clcB	3	22.55	0.707
0.1	0	ycaM	3	22.55	0.564
0.1	0	yadI	3	22.55	0.575
0.1	0	AG1	3	22.55	0.648
0.1	0	ptsI	3	22.8	0.838
0.1	0	clcB	3	22.8	0.702
0.1	0	ycaM	3	22.8	0.565
0.1	0	yadI	3	22.8	0.566
0.1	0	AG1	3	22.8	0.649
0.1	0	ptsI	3	23.05	0.848
0.1	0	clcB	3	23.05	0.717
0.1	0	ycaM	3	23.05	0.566
0.1	0	yadI	3	23.05	0.567
0.1	0	AG1	3	23.05	0.65
0.1	0	ptsI	3	23.3	0.856
0.1	0	clcB	3	23.3	0.716
0.1	0	ycaM	3	23.3	0.567
0.1	0	yadI	3	23.3	0.563
0.1	0	AG1	3	23.3	0.651
0.1	0	ptsI	3	23.55	0.867
0.1	0	clcB	3	23.55	0.735
0.1	0	ycaM	3	23.55	0.573

0.1	0	yadI	3	23.55	0.564
0.1	0	AG1	3	23.55	0.657
0.1	0	ptsI	3	23.8	0.871
0.1	0	clcB	3	23.8	0.727
0.1	0	ycaM	3	23.8	0.572
0.1	0	yadI	3	23.8	0.562
0.1	0	AG1	3	23.8	0.652
0.1	0	ptsI	3	24.05	0.878
0.1	0	clcB	3	24.05	0.74
0.1	0	ycaM	3	24.05	0.573
0.1	0	yadI	3	24.05	0.558
0.1	0	AG1	3	24.05	0.657
0.1	0	ptsI	3	24.3	0.876
0.1	0	clcB	3	24.3	0.755
0.1	0	ycaM	3	24.3	0.57
0.1	0	yadI	3	24.3	0.559
0.1	0	AG1	3	24.3	0.658
0.1	0	ptsI	4	0	0.192
0.1	0	clcB	4	0	0.196
0.1	0	ycaM	4	0	0.204
0.1	0	yadI	4	0	0.225
0.1	0	AG1	4	0	0.214
0.1	0	ptsI	4	0.25	0.194
0.1	0	clcB	4	0.25	0.194
0.1	0	ycaM	4	0.25	0.196
0.1	0	yadI	4	0.25	0.223
0.1	0	AG1	4	0.25	0.214
0.1	0	ptsI	4	0.5	0.195
0.1	0	clcB	4	0.5	0.194
0.1	0	ycaM	4	0.5	0.199
0.1	0	yadI	4	0.5	0.225
0.1	0	AG1	4	0.5	0.217
0.1	0	ptsI	4	0.75	0.199
0.1	0	clcB	4	0.75	0.197
0.1	0	ycaM	4	0.75	0.202
0.1	0	yadI	4	0.75	0.23
0.1	0	AG1	4	0.75	0.22
0.1	0	ptsI	4	1	0.203
0.1	0	clcB	4	1	0.2
0.1	0	ycaM	4	1	0.206
0.1	0	yadI	4	1	0.232
0.1	0	AG1	4	1	0.223
0.1	0	ptsI	4	1.25	0.21
0.1	0	clcB	4	1.25	0.21
0.1	0	ycaM	4	1.25	0.212
0.1	0	yadI	4	1.25	0.238
0.1	0	AG1	4	1.25	0.23
0.1	0	ptsI	4	1.5	0.216
0.1	0	clcB	4	1.5	0.213
0.1	0	ycaM	4	1.5	0.212
0.1	0	yadI	4	1.5	0.243
0.1	0	AG1	4	1.5	0.233
0.1	0	ptsI	4	1.75	0.226

0.1	0	clcB	4	1.75	0.226
0.1	0	ycaM	4	1.75	0.224
0.1	0	yadI	4	1.75	0.252
0.1	0	AG1	4	1.75	0.242
0.1	0	ptsI	4	2	0.235
0.1	0	clcB	4	2	0.24
0.1	0	ycaM	4	2	0.233
0.1	0	yadI	4	2	0.266
0.1	0	AG1	4	2	0.252
0.1	0	ptsI	4	2.25	0.248
0.1	0	clcB	4	2.25	0.253
0.1	0	ycaM	4	2.25	0.246
0.1	0	yadI	4	2.25	0.278
0.1	0	AG1	4	2.25	0.267
0.1	0	ptsI	4	2.5	0.262
0.1	0	clcB	4	2.5	0.262
0.1	0	ycaM	4	2.5	0.253
0.1	0	yadI	4	2.5	0.29
0.1	0	AG1	4	2.5	0.274
0.1	0	ptsI	4	2.75	0.276
0.1	0	clcB	4	2.75	0.28
0.1	0	ycaM	4	2.75	0.266
0.1	0	yadI	4	2.75	0.301
0.1	0	AG1	4	2.75	0.282
0.1	0	ptsI	4	3	0.292
0.1	0	clcB	4	3	0.295
0.1	0	ycaM	4	3	0.284
0.1	0	yadI	4	3	0.321
0.1	0	AG1	4	3	0.301
0.1	0	ptsI	4	3.25	0.306
0.1	0	clcB	4	3.25	0.311
0.1	0	ycaM	4	3.25	0.298
0.1	0	yadI	4	3.25	0.341
0.1	0	AG1	4	3.25	0.316
0.1	0	ptsI	4	3.5	0.325
0.1	0	clcB	4	3.5	0.325
0.1	0	ycaM	4	3.5	0.316
0.1	0	yadI	4	3.5	0.362
0.1	0	AG1	4	3.5	0.334
0.1	0	ptsI	4	3.75	0.341
0.1	0	clcB	4	3.75	0.343
0.1	0	ycaM	4	3.75	0.325
0.1	0	yadI	4	3.75	0.382
0.1	0	AG1	4	3.75	0.346
0.1	0	ptsI	4	4	0.361
0.1	0	clcB	4	4	0.354
0.1	0	ycaM	4	4	0.343
0.1	0	yadI	4	4	0.403
0.1	0	AG1	4	4	0.369
0.1	0	ptsI	4	4.35	0.36
0.1	0	clcB	4	4.35	0.318
0.1	0	ycaM	4	4.35	0.373
0.1	0	yadI	4	4.35	0.412

0.1	0	AG1	4	4.35	0.41
0.1	0	ptsI	4	4.6	0.364
0.1	0	clcB	4	4.6	0.303
0.1	0	ycaM	4	4.6	0.34
0.1	0	yadI	4	4.6	0.386
0.1	0	AG1	4	4.6	0.392
0.1	0	ptsI	4	4.85	0.377
0.1	0	clcB	4	4.85	0.303
0.1	0	ycaM	4	4.85	0.345
0.1	0	yadI	4	4.85	0.397
0.1	0	AG1	4	4.85	0.401
0.1	0	ptsI	4	5.1	0.392
0.1	0	clcB	4	5.1	0.312
0.1	0	ycaM	4	5.1	0.356
0.1	0	yadI	4	5.1	0.41
0.1	0	AG1	4	5.1	0.417
0.1	0	ptsI	4	5.35	0.414
0.1	0	clcB	4	5.35	0.321
0.1	0	ycaM	4	5.35	0.365
0.1	0	yadI	4	5.35	0.426
0.1	0	AG1	4	5.35	0.431
0.1	0	ptsI	4	5.6	0.425
0.1	0	clcB	4	5.6	0.331
0.1	0	ycaM	4	5.6	0.376
0.1	0	yadI	4	5.6	0.443
0.1	0	AG1	4	5.6	0.441
0.1	0	ptsI	4	5.85	0.434
0.1	0	clcB	4	5.85	0.346
0.1	0	ycaM	4	5.85	0.385
0.1	0	yadI	4	5.85	0.456
0.1	0	AG1	4	5.85	0.451
0.1	0	ptsI	4	6.1	0.449
0.1	0	clcB	4	6.1	0.361
0.1	0	ycaM	4	6.1	0.396
0.1	0	yadI	4	6.1	0.473
0.1	0	AG1	4	6.1	0.466
0.1	0	ptsI	4	6.35	0.462
0.1	0	clcB	4	6.35	0.371
0.1	0	ycaM	4	6.35	0.405
0.1	0	yadI	4	6.35	0.496
0.1	0	AG1	4	6.35	0.486
0.1	0	ptsI	4	6.6	0.477
0.1	0	clcB	4	6.6	0.386
0.1	0	ycaM	4	6.6	0.414
0.1	0	yadI	4	6.6	0.512
0.1	0	AG1	4	6.6	0.492
0.1	0	ptsI	4	6.85	0.486
0.1	0	clcB	4	6.85	0.397
0.1	0	ycaM	4	6.85	0.425
0.1	0	yadI	4	6.85	0.528
0.1	0	AG1	4	6.85	0.507
0.1	0	ptsI	4	7.1	0.497
0.1	0	clcB	4	7.1	0.408

0.1	0	ycaM	4	7.1	0.432
0.1	0	yadI	4	7.1	0.541
0.1	0	AG1	4	7.1	0.525
0.1	0	ptsI	4	7.35	0.505
0.1	0	clcB	4	7.35	0.417
0.1	0	ycaM	4	7.35	0.44
0.1	0	yadI	4	7.35	0.549
0.1	0	AG1	4	7.35	0.538
0.1	0	ptsI	4	7.6	0.513
0.1	0	clcB	4	7.6	0.428
0.1	0	ycaM	4	7.6	0.451
0.1	0	yadI	4	7.6	0.56
0.1	0	AG1	4	7.6	0.544
0.1	0	ptsI	4	7.85	0.519
0.1	0	clcB	4	7.85	0.433
0.1	0	ycaM	4	7.85	0.457
0.1	0	yadI	4	7.85	0.57
0.1	0	AG1	4	7.85	0.558
0.1	0	ptsI	4	8.1	0.529
0.1	0	clcB	4	8.1	0.444
0.1	0	ycaM	4	8.1	0.467
0.1	0	yadI	4	8.1	0.584
0.1	0	AG1	4	8.1	0.568
0.1	0	ptsI	4	8.35	0.538
0.1	0	clcB	4	8.35	0.447
0.1	0	ycaM	4	8.35	0.472
0.1	0	yadI	4	8.35	0.593
0.1	0	AG1	4	8.35	0.581
0.1	0	ptsI	4	8.83333	0.378
0.1	0	clcB	4	8.83333	0.414
0.1	0	ycaM	4	8.83333	0.397
0.1	0	yadI	4	8.83333	0.499
0.1	0	AG1	4	8.83333	0.533
0.1	0	ptsI	4	9.08333	0.382
0.1	0	clcB	4	9.08333	0.41
0.1	0	ycaM	4	9.08333	0.398
0.1	0	yadI	4	9.08333	0.496
0.1	0	AG1	4	9.08333	0.539
0.1	0	ptsI	4	9.33333	0.389
0.1	0	clcB	4	9.33333	0.413
0.1	0	ycaM	4	9.33333	0.399
0.1	0	yadI	4	9.33333	0.503
0.1	0	AG1	4	9.33333	0.549
0.1	0	ptsI	4	9.58333	0.399
0.1	0	clcB	4	9.58333	0.422
0.1	0	ycaM	4	9.58333	0.4
0.1	0	yadI	4	9.58333	0.513
0.1	0	AG1	4	9.58333	0.562
0.1	0	ptsI	4	9.83333	0.407
0.1	0	clcB	4	9.83333	0.428
0.1	0	ycaM	4	9.83333	0.404
0.1	0	yadI	4	9.83333	0.522
0.1	0	AG1	4	9.83333	0.571

0.1	0	ptsI	4	10.0833	0.417
0.1	0	clcB	4	10.0833	0.435
0.1	0	ycaM	4	10.0833	0.405
0.1	0	yadI	4	10.0833	0.529
0.1	0	AG1	4	10.0833	0.576
0.1	0	ptsI	4	10.3333	0.422
0.1	0	clcB	4	10.3333	0.441
0.1	0	ycaM	4	10.3333	0.409
0.1	0	yadI	4	10.3333	0.536
0.1	0	AG1	4	10.3333	0.584
0.1	0	ptsI	4	10.5833	0.428
0.1	0	clcB	4	10.5833	0.449
0.1	0	ycaM	4	10.5833	0.415
0.1	0	yadI	4	10.5833	0.543
0.1	0	AG1	4	10.5833	0.592
0.1	0	ptsI	4	10.8333	0.432
0.1	0	clcB	4	10.8333	0.453
0.1	0	ycaM	4	10.8333	0.418
0.1	0	yadI	4	10.8333	0.548
0.1	0	AG1	4	10.8333	0.603
0.1	0	ptsI	4	11.0833	0.437
0.1	0	clcB	4	11.0833	0.456
0.1	0	ycaM	4	11.0833	0.421
0.1	0	yadI	4	11.0833	0.556
0.1	0	AG1	4	11.0833	0.61
0.1	0	ptsI	4	11.3333	0.445
0.1	0	clcB	4	11.3333	0.465
0.1	0	ycaM	4	11.3333	0.429
0.1	0	yadI	4	11.3333	0.563
0.1	0	AG1	4	11.3333	0.62
0.1	0	ptsI	4	11.5833	0.451
0.1	0	clcB	4	11.5833	0.466
0.1	0	ycaM	4	11.5833	0.431
0.1	0	yadI	4	11.5833	0.569
0.1	0	AG1	4	11.5833	0.624
0.1	0	ptsI	4	11.8333	0.458
0.1	0	clcB	4	11.8333	0.47
0.1	0	ycaM	4	11.8333	0.434
0.1	0	yadI	4	11.8333	0.577
0.1	0	AG1	4	11.8333	0.632
0.1	0	ptsI	4	12.0833	0.462
0.1	0	clcB	4	12.0833	0.473
0.1	0	ycaM	4	12.0833	0.438
0.1	0	yadI	4	12.0833	0.582
0.1	0	AG1	4	12.0833	0.636
0.1	0	ptsI	4	12.3333	0.472
0.1	0	clcB	4	12.3333	0.48
0.1	0	ycaM	4	12.3333	0.441
0.1	0	yadI	4	12.3333	0.584
0.1	0	AG1	4	12.3333	0.644
0.1	0	ptsI	4	12.5833	0.481
0.1	0	clcB	4	12.5833	0.483
0.1	0	ycaM	4	12.5833	0.443

0.1	0	yadI	4	12.5833	0.591
0.1	0	AG1	4	12.5833	0.648
0.1	0	ptsI	4	12.8333	0.484
0.1	0	clcB	4	12.8333	0.484
0.1	0	ycaM	4	12.8333	0.449
0.1	0	yadI	4	12.8333	0.598
0.1	0	AG1	4	12.8333	0.652
0.1	0	ptsI	4	13.0833	0.49
0.1	0	clcB	4	13.0833	0.49
0.1	0	ycaM	4	13.0833	0.452
0.1	0	yadI	4	13.0833	0.6
0.1	0	AG1	4	13.0833	0.659
0.1	0	ptsI	4	13.3333	0.499
0.1	0	clcB	4	13.3333	0.493
0.1	0	ycaM	4	13.3333	0.455
0.1	0	yadI	4	13.3333	0.604
0.1	0	AG1	4	13.3333	0.666
0.1	0	ptsI	4	13.5833	0.503
0.1	0	clcB	4	13.5833	0.494
0.1	0	ycaM	4	13.5833	0.459
0.1	0	yadI	4	13.5833	0.61
0.1	0	AG1	4	13.5833	0.672
0.1	0	ptsI	4	13.8333	0.505
0.1	0	clcB	4	13.8333	0.5
0.1	0	ycaM	4	13.8333	0.464
0.1	0	yadI	4	13.8333	0.615
0.1	0	AG1	4	13.8333	0.676
0.1	0	ptsI	4	14.0833	0.512
0.1	0	clcB	4	14.0833	0.502
0.1	0	ycaM	4	14.0833	0.467
0.1	0	yadI	4	14.0833	0.618
0.1	0	AG1	4	14.0833	0.683
0.1	0	ptsI	4	14.3333	0.518
0.1	0	clcB	4	14.3333	0.509
0.1	0	ycaM	4	14.3333	0.471
0.1	0	yadI	4	14.3333	0.626
0.1	0	AG1	4	14.3333	0.683
0.1	0	ptsI	4	14.5833	0.52
0.1	0	clcB	4	14.5833	0.506
0.1	0	ycaM	4	14.5833	0.471
0.1	0	yadI	4	14.5833	0.626
0.1	0	AG1	4	14.5833	0.686
0.1	0	ptsI	4	14.8333	0.526
0.1	0	clcB	4	14.8333	0.51
0.1	0	ycaM	4	14.8333	0.478
0.1	0	yadI	4	14.8333	0.629
0.1	0	AG1	4	14.8333	0.693
0.1	0	ptsI	4	15.0833	0.531
0.1	0	clcB	4	15.0833	0.51
0.1	0	ycaM	4	15.0833	0.479
0.1	0	yadI	4	15.0833	0.634
0.1	0	AG1	4	15.0833	0.694
0.1	0	ptsI	4	15.3333	0.534

0.1	0	clcB	4	15.3333	0.514
0.1	0	ycaM	4	15.3333	0.484
0.1	0	yadI	4	15.3333	0.637
0.1	0	AG1	4	15.3333	0.697
0.1	0	ptsI	4	15.5833	0.543
0.1	0	clcB	4	15.5833	0.516
0.1	0	ycaM	4	15.5833	0.487
0.1	0	yadI	4	15.5833	0.644
0.1	0	AG1	4	15.5833	0.701
0.1	0	ptsI	4	15.8333	0.54
0.1	0	clcB	4	15.8333	0.516
0.1	0	ycaM	4	15.8333	0.488
0.1	0	yadI	4	15.8333	0.648
0.1	0	AG1	4	15.8333	0.704
0.1	0	ptsI	4	16.0833	0.548
0.1	0	clcB	4	16.0833	0.521
0.1	0	ycaM	4	16.0833	0.492
0.1	0	yadI	4	16.0833	0.65
0.1	0	AG1	4	16.0833	0.707
0.1	0	ptsI	4	16.3333	0.546
0.1	0	clcB	4	16.3333	0.517
0.1	0	ycaM	4	16.3333	0.494
0.1	0	yadI	4	16.3333	0.651
0.1	0	AG1	4	16.3333	0.71
0.1	0	ptsI	4	16.5833	0.551
0.1	0	clcB	4	16.5833	0.516
0.1	0	ycaM	4	16.5833	0.496
0.1	0	yadI	4	16.5833	0.658
0.1	0	AG1	4	16.5833	0.714
0.1	0	ptsI	4	16.8333	0.559
0.1	0	clcB	4	16.8333	0.52
0.1	0	ycaM	4	16.8333	0.495
0.1	0	yadI	4	16.8333	0.658
0.1	0	AG1	4	16.8333	0.712
0.1	0	ptsI	4	17.0833	0.562
0.1	0	clcB	4	17.0833	0.519
0.1	0	ycaM	4	17.0833	0.493
0.1	0	yadI	4	17.0833	0.662
0.1	0	AG1	4	17.0833	0.715
0.1	0	ptsI	4	17.3333	0.559
0.1	0	clcB	4	17.3333	0.523
0.1	0	ycaM	4	17.3333	0.501
0.1	0	yadI	4	17.3333	0.67
0.1	0	AG1	4	17.3333	0.717
0.1	0	ptsI	4	17.5833	0.566
0.1	0	clcB	4	17.5833	0.523
0.1	0	ycaM	4	17.5833	0.501
0.1	0	yadI	4	17.5833	0.668
0.1	0	AG1	4	17.5833	0.722
0.1	0	ptsI	4	17.8333	0.58
0.1	0	clcB	4	17.8333	0.525
0.1	0	ycaM	4	17.8333	0.497
0.1	0	yadI	4	17.8333	0.668

0.1	0	AG1	4	17.8333	0.729
0.1	0	ptsI	4	18.0833	0.575
0.1	0	clcB	4	18.0833	0.525
0.1	0	ycaM	4	18.0833	0.504
0.1	0	yadI	4	18.0833	0.676
0.1	0	AG1	4	18.0833	0.726
0.1	0	ptsI	4	18.3333	0.573
0.1	0	clcB	4	18.3333	0.526
0.1	0	ycaM	4	18.3333	0.505
0.1	0	yadI	4	18.3333	0.68
0.1	0	AG1	4	18.3333	0.728
0.1	0	ptsI	4	18.5833	0.58
0.1	0	clcB	4	18.5833	0.525
0.1	0	ycaM	4	18.5833	0.506
0.1	0	yadI	4	18.5833	0.683
0.1	0	AG1	4	18.5833	0.732
0.1	0	ptsI	4	18.8333	0.59
0.1	0	clcB	4	18.8333	0.526
0.1	0	ycaM	4	18.8333	0.503
0.1	0	yadI	4	18.8333	0.684
0.1	0	AG1	4	18.8333	0.739
0.1	0	ptsI	4	19.0833	0.588
0.1	0	clcB	4	19.0833	0.528
0.1	0	ycaM	4	19.0833	0.511
0.1	0	yadI	4	19.0833	0.689
0.1	0	AG1	4	19.0833	0.738
0.1	0	ptsI	4	19.3333	0.585
0.1	0	clcB	4	19.3333	0.528
0.1	0	ycaM	4	19.3333	0.513
0.1	0	yadI	4	19.3333	0.694
0.1	0	AG1	4	19.3333	0.738
0.1	0	ptsI	4	19.5833	0.596
0.1	0	clcB	4	19.5833	0.532
0.1	0	ycaM	4	19.5833	0.515
0.1	0	yadI	4	19.5833	0.698
0.1	0	AG1	4	19.5833	0.741
0.1	0	ptsI	4	19.8333	0.595
0.1	0	clcB	4	19.8333	0.53
0.1	0	ycaM	4	19.8333	0.513
0.1	0	yadI	4	19.8333	0.697
0.1	0	AG1	4	19.8333	0.742
0.1	0	ptsI	4	20.0833	0.59
0.1	0	clcB	4	20.0833	0.534
0.1	0	ycaM	4	20.0833	0.521
0.1	0	yadI	4	20.0833	0.703
0.1	0	AG1	4	20.0833	0.714
0.1	0	ptsI	4	20.3333	0.597
0.1	0	clcB	4	20.3333	0.53
0.1	0	ycaM	4	20.3333	0.519
0.1	0	yadI	4	20.3333	0.703
0.1	0	AG1	4	20.3333	0.749
0.1	0	ptsI	4	20.5833	0.602
0.1	0	clcB	4	20.5833	0.53

0.1	0	ycaM	4	20.5833	0.515
0.1	0	yadI	4	20.5833	0.709
0.1	0	AG1	4	20.5833	0.747
0.1	0	ptsI	4	20.8333	0.601
0.1	0	clcB	4	20.8333	0.534
0.1	0	ycaM	4	20.8333	0.52
0.1	0	yadI	4	20.8333	0.71
0.1	0	AG1	4	20.8333	0.751
0.1	0	ptsI	4	21.0833	0.606
0.1	0	clcB	4	21.0833	0.532
0.1	0	ycaM	4	21.0833	0.52
0.1	0	yadI	4	21.0833	0.712
0.1	0	AG1	4	21.0833	0.753
0.1	0	ptsI	4	21.3333	0.605
0.1	0	clcB	4	21.3333	0.534
0.1	0	ycaM	4	21.3333	0.521
0.1	0	yadI	4	21.3333	0.714
0.1	0	AG1	4	21.3333	0.757
0.1	0	ptsI	4	21.5833	0.615
0.1	0	clcB	4	21.5833	0.534
0.1	0	ycaM	4	21.5833	0.523
0.1	0	yadI	4	21.5833	0.719
0.1	0	AG1	4	21.5833	0.758
0.1	0	ptsI	4	21.8333	0.609
0.1	0	clcB	4	21.8333	0.533
0.1	0	ycaM	4	21.8333	0.526
0.1	0	yadI	4	21.8333	0.722
0.1	0	AG1	4	21.8333	0.756
0.1	0	ptsI	4	22.0833	0.617
0.1	0	clcB	4	22.0833	0.536
0.1	0	ycaM	4	22.0833	0.525
0.1	0	yadI	4	22.0833	0.721
0.1	0	AG1	4	22.0833	0.759
0.1	0	ptsI	4	22.3333	0.628
0.1	0	clcB	4	22.3333	0.536
0.1	0	ycaM	4	22.3333	0.525
0.1	0	yadI	4	22.3333	0.724
0.1	0	AG1	4	22.3333	0.758
0.1	0	ptsI	4	22.5833	0.628
0.1	0	clcB	4	22.5833	0.532
0.1	0	ycaM	4	22.5833	0.521
0.1	0	yadI	4	22.5833	0.728
0.1	0	AG1	4	22.5833	0.759
0.1	0	ptsI	4	22.8333	0.63
0.1	0	clcB	4	22.8333	0.536
0.1	0	ycaM	4	22.8333	0.524
0.1	0	yadI	4	22.8333	0.729
0.1	0	AG1	4	22.8333	0.762
0.1	0	ptsI	4	23.0833	0.638
0.1	0	clcB	4	23.0833	0.538
0.1	0	ycaM	4	23.0833	0.528
0.1	0	yadI	4	23.0833	0.731
0.1	0	AG1	4	23.0833	0.767

0.1	0	ptsI	4	23.3333	0.632
0.1	0	clcB	4	23.3333	0.538
0.1	0	ycaM	4	23.3333	0.53
0.1	0	yadI	4	23.3333	0.734
0.1	0	AG1	4	23.3333	0.767
0.1	0	ptsI	4	23.5833	0.639
0.1	0	clcB	4	23.5833	0.536
0.1	0	ycaM	4	23.5833	0.527
0.1	0	yadI	4	23.5833	0.735
0.1	0	AG1	4	23.5833	0.766
0.1	0	ptsI	4	23.8333	0.645
0.1	0	clcB	4	23.8333	0.537
0.1	0	ycaM	4	23.8333	0.528
0.1	0	yadI	4	23.8333	0.738
0.1	0	AG1	4	23.8333	0.768
0.1	0	ptsI	4	24.0833	0.645
0.1	0	clcB	4	24.0833	0.538
0.1	0	ycaM	4	24.0833	0.529
0.1	0	yadI	4	24.0833	0.74
0.1	0	AG1	4	24.0833	0.773
0.1	0	ptsI	5	0	0.157
0.1	0	clcB	5	0	0.166
0.1	0	ycaM	5	0	0.173
0.1	0	yadI	5	0	0.192
0.1	0	AG1	5	0	0.166
0.1	0	ptsI	5	0.35	0.154
0.1	0	clcB	5	0.35	0.163
0.1	0	ycaM	5	0.35	0.173
0.1	0	yadI	5	0.35	0.182
0.1	0	AG1	5	0.35	0.164
0.1	0	ptsI	5	0.6	0.154
0.1	0	clcB	5	0.6	0.165
0.1	0	ycaM	5	0.6	0.168
0.1	0	yadI	5	0.6	0.182
0.1	0	AG1	5	0.6	0.164
0.1	0	ptsI	5	0.85	0.155
0.1	0	clcB	5	0.85	0.164
0.1	0	ycaM	5	0.85	0.168
0.1	0	yadI	5	0.85	0.184
0.1	0	AG1	5	0.85	0.166
0.1	0	ptsI	5	1.1	0.157
0.1	0	clcB	5	1.1	0.167
0.1	0	ycaM	5	1.1	0.171
0.1	0	yadI	5	1.1	0.189
0.1	0	AG1	5	1.1	0.167
0.1	0	ptsI	5	1.35	0.158
0.1	0	clcB	5	1.35	0.167
0.1	0	ycaM	5	1.35	0.175
0.1	0	yadI	5	1.35	0.189
0.1	0	AG1	5	1.35	0.169
0.1	0	ptsI	5	1.6	0.163
0.1	0	clcB	5	1.6	0.173
0.1	0	ycaM	5	1.6	0.178

0.1	0	yadI	5	1.6	0.196
0.1	0	AG1	5	1.6	0.175
0.1	0	ptsI	5	1.85	0.164
0.1	0	clcB	5	1.85	0.175
0.1	0	ycaM	5	1.85	0.182
0.1	0	yadI	5	1.85	0.201
0.1	0	AG1	5	1.85	0.178
0.1	0	ptsI	5	2.1	0.168
0.1	0	clcB	5	2.1	0.181
0.1	0	ycaM	5	2.1	0.186
0.1	0	yadI	5	2.1	0.208
0.1	0	AG1	5	2.1	0.186
0.1	0	ptsI	5	2.35	0.175
0.1	0	clcB	5	2.35	0.19
0.1	0	ycaM	5	2.35	0.191
0.1	0	yadI	5	2.35	0.219
0.1	0	AG1	5	2.35	0.194
0.1	0	ptsI	5	2.6	0.178
0.1	0	clcB	5	2.6	0.193
0.1	0	ycaM	5	2.6	0.196
0.1	0	yadI	5	2.6	0.224
0.1	0	AG1	5	2.6	0.193
0.1	0	ptsI	5	2.85	0.187
0.1	0	clcB	5	2.85	0.204
0.1	0	ycaM	5	2.85	0.201
0.1	0	yadI	5	2.85	0.238
0.1	0	AG1	5	2.85	0.209
0.1	0	ptsI	5	3.1	0.196
0.1	0	clcB	5	3.1	0.217
0.1	0	ycaM	5	3.1	0.21
0.1	0	yadI	5	3.1	0.252
0.1	0	AG1	5	3.1	0.22
0.1	0	ptsI	5	3.35	0.201
0.1	0	clcB	5	3.35	0.227
0.1	0	ycaM	5	3.35	0.218
0.1	0	yadI	5	3.35	0.268
0.1	0	AG1	5	3.35	0.237
0.1	0	ptsI	5	3.6	0.204
0.1	0	clcB	5	3.6	0.239
0.1	0	ycaM	5	3.6	0.23
0.1	0	yadI	5	3.6	0.287
0.1	0	AG1	5	3.6	0.256
0.1	0	ptsI	5	3.85	0.211
0.1	0	clcB	5	3.85	0.257
0.1	0	ycaM	5	3.85	0.244
0.1	0	yadI	5	3.85	0.308
0.1	0	AG1	5	3.85	0.276
0.1	0	ptsI	5	4.38333	0.21
0.1	0	clcB	5	4.38333	0.241
0.1	0	ycaM	5	4.38333	0.27
0.1	0	yadI	5	4.38333	0.323
0.1	0	AG1	5	4.38333	0.313
0.1	0	ptsI	5	4.63333	0.21

0.1	0	clcB	5	4.63333	0.244
0.1	0	ycaM	5	4.63333	0.268
0.1	0	yadI	5	4.63333	0.316
0.1	0	AG1	5	4.63333	0.302
0.1	0	ptsI	5	4.88333	0.214
0.1	0	clcB	5	4.88333	0.257
0.1	0	ycaM	5	4.88333	0.28
0.1	0	yadI	5	4.88333	0.328
0.1	0	AG1	5	4.88333	0.316
0.1	0	ptsI	5	5.13333	0.223
0.1	0	clcB	5	5.13333	0.269
0.1	0	ycaM	5	5.13333	0.291
0.1	0	yadI	5	5.13333	0.342
0.1	0	AG1	5	5.13333	0.326
0.1	0	ptsI	5	5.38333	0.23
0.1	0	clcB	5	5.38333	0.282
0.1	0	ycaM	5	5.38333	0.306
0.1	0	yadI	5	5.38333	0.354
0.1	0	AG1	5	5.38333	0.339
0.1	0	ptsI	5	5.63333	0.238
0.1	0	clcB	5	5.63333	0.295
0.1	0	ycaM	5	5.63333	0.316
0.1	0	yadI	5	5.63333	0.37
0.1	0	AG1	5	5.63333	0.351
0.1	0	ptsI	5	5.88333	0.248
0.1	0	clcB	5	5.88333	0.307
0.1	0	ycaM	5	5.88333	0.326
0.1	0	yadI	5	5.88333	0.382
0.1	0	AG1	5	5.88333	0.362
0.1	0	ptsI	5	6.13333	0.258
0.1	0	clcB	5	6.13333	0.322
0.1	0	ycaM	5	6.13333	0.335
0.1	0	yadI	5	6.13333	0.397
0.1	0	AG1	5	6.13333	0.376
0.1	0	ptsI	5	6.38333	0.268
0.1	0	clcB	5	6.38333	0.332
0.1	0	ycaM	5	6.38333	0.345
0.1	0	yadI	5	6.38333	0.414
0.1	0	AG1	5	6.38333	0.395
0.1	0	ptsI	5	6.63333	0.281
0.1	0	clcB	5	6.63333	0.345
0.1	0	ycaM	5	6.63333	0.355
0.1	0	yadI	5	6.63333	0.429
0.1	0	AG1	5	6.63333	0.416
0.1	0	ptsI	5	6.88333	0.292
0.1	0	clcB	5	6.88333	0.357
0.1	0	ycaM	5	6.88333	0.364
0.1	0	yadI	5	6.88333	0.454
0.1	0	AG1	5	6.88333	0.439
0.1	0	ptsI	5	7.13333	0.303
0.1	0	clcB	5	7.13333	0.367
0.1	0	ycaM	5	7.13333	0.372
0.1	0	yadI	5	7.13333	0.474

0.1	0	AG1	5	7.13333	0.459
0.1	0	ptsI	5	7.38333	0.316
0.1	0	clcB	5	7.38333	0.381
0.1	0	ycaM	5	7.38333	0.385
0.1	0	yadI	5	7.38333	0.492
0.1	0	AG1	5	7.38333	0.476
0.1	0	ptsI	5	7.63333	0.326
0.1	0	clcB	5	7.63333	0.392
0.1	0	ycaM	5	7.63333	0.396
0.1	0	yadI	5	7.63333	0.51
0.1	0	AG1	5	7.63333	0.5
0.1	0	ptsI	5	7.88333	0.336
0.1	0	clcB	5	7.88333	0.404
0.1	0	ycaM	5	7.88333	0.407
0.1	0	yadI	5	7.88333	0.529
0.1	0	AG1	5	7.88333	0.516
0.1	0	ptsI	5	8.13333	0.349
0.1	0	clcB	5	8.13333	0.414
0.1	0	ycaM	5	8.13333	0.415
0.1	0	yadI	5	8.13333	0.535
0.1	0	AG1	5	8.13333	0.532
0.1	0	ptsI	5	8.38333	0.363
0.1	0	clcB	5	8.38333	0.427
0.1	0	ycaM	5	8.38333	0.426
0.1	0	yadI	5	8.38333	0.554
0.1	0	AG1	5	8.38333	0.546
0.1	0	ptsI	5	8.88333	0.335
0.1	0	clcB	5	8.88333	0.362
0.1	0	ycaM	5	8.88333	0.356
0.1	0	yadI	5	8.88333	0.508
0.1	0	AG1	5	8.88333	0.599
0.1	0	ptsI	5	9.13333	0.339
0.1	0	clcB	5	9.13333	0.369
0.1	0	ycaM	5	9.13333	0.361
0.1	0	yadI	5	9.13333	0.507
0.1	0	AG1	5	9.13333	0.597
0.1	0	ptsI	5	9.38333	0.342
0.1	0	clcB	5	9.38333	0.378
0.1	0	ycaM	5	9.38333	0.365
0.1	0	yadI	5	9.38333	0.504
0.1	0	AG1	5	9.38333	0.616
0.1	0	ptsI	5	9.63333	0.357
0.1	0	clcB	5	9.63333	0.388
0.1	0	ycaM	5	9.63333	0.374
0.1	0	yadI	5	9.63333	0.52
0.1	0	AG1	5	9.63333	0.624
0.1	0	ptsI	5	9.88333	0.371
0.1	0	clcB	5	9.88333	0.4
0.1	0	ycaM	5	9.88333	0.384
0.1	0	yadI	5	9.88333	0.529
0.1	0	AG1	5	9.88333	0.638
0.1	0	ptsI	5	10.1333	0.378
0.1	0	clcB	5	10.1333	0.406

0.1	0	ycaM	5	10.1333	0.391
0.1	0	yadI	5	10.1333	0.536
0.1	0	AG1	5	10.1333	0.646
0.1	0	ptsI	5	10.3833	0.392
0.1	0	clcB	5	10.3833	0.416
0.1	0	ycaM	5	10.3833	0.397
0.1	0	yadI	5	10.3833	0.544
0.1	0	AG1	5	10.3833	0.66
0.1	0	ptsI	5	10.6333	0.397
0.1	0	clcB	5	10.6333	0.42
0.1	0	ycaM	5	10.6333	0.399
0.1	0	yadI	5	10.6333	0.551
0.1	0	AG1	5	10.6333	0.666
0.1	0	ptsI	5	10.8833	0.407
0.1	0	clcB	5	10.8833	0.426
0.1	0	ycaM	5	10.8833	0.405
0.1	0	yadI	5	10.8833	0.562
0.1	0	AG1	5	10.8833	0.678
0.1	0	ptsI	5	11.1333	0.412
0.1	0	clcB	5	11.1333	0.429
0.1	0	ycaM	5	11.1333	0.403
0.1	0	yadI	5	11.1333	0.564
0.1	0	AG1	5	11.1333	0.689
0.1	0	ptsI	5	11.3833	0.419
0.1	0	clcB	5	11.3833	0.433
0.1	0	ycaM	5	11.3833	0.406
0.1	0	yadI	5	11.3833	0.573
0.1	0	AG1	5	11.3833	0.7
0.1	0	ptsI	5	11.6333	0.427
0.1	0	clcB	5	11.6333	0.438
0.1	0	ycaM	5	11.6333	0.411
0.1	0	yadI	5	11.6333	0.58
0.1	0	AG1	5	11.6333	0.712
0.1	0	ptsI	5	11.8833	0.438
0.1	0	clcB	5	11.8833	0.445
0.1	0	ycaM	5	11.8833	0.416
0.1	0	yadI	5	11.8833	0.589
0.1	0	AG1	5	11.8833	0.719
0.1	0	ptsI	5	12.1333	0.448
0.1	0	clcB	5	12.1333	0.452
0.1	0	ycaM	5	12.1333	0.42
0.1	0	yadI	5	12.1333	0.598
0.1	0	AG1	5	12.1333	0.73
0.1	0	ptsI	5	12.3833	0.45
0.1	0	clcB	5	12.3833	0.453
0.1	0	ycaM	5	12.3833	0.422
0.1	0	yadI	5	12.3833	0.599
0.1	0	AG1	5	12.3833	0.733
0.1	0	ptsI	5	12.6333	0.46
0.1	0	clcB	5	12.6333	0.458
0.1	0	ycaM	5	12.6333	0.426
0.1	0	yadI	5	12.6333	0.607
0.1	0	AG1	5	12.6333	0.747

0.1	0	ptsI	5	12.8833	0.473
0.1	0	clcB	5	12.8833	0.469
0.1	0	ycaM	5	12.8833	0.431
0.1	0	yadI	5	12.8833	0.617
0.1	0	AG1	5	12.8833	0.756
0.1	0	ptsI	5	13.1333	0.478
0.1	0	clcB	5	13.1333	0.47
0.1	0	ycaM	5	13.1333	0.434
0.1	0	yadI	5	13.1333	0.62
0.1	0	AG1	5	13.1333	0.765
0.1	0	ptsI	5	13.3833	0.482
0.1	0	clcB	5	13.3833	0.473
0.1	0	ycaM	5	13.3833	0.436
0.1	0	yadI	5	13.3833	0.625
0.1	0	AG1	5	13.3833	0.771
0.1	0	ptsI	5	13.6333	0.491
0.1	0	clcB	5	13.6333	0.481
0.1	0	ycaM	5	13.6333	0.441
0.1	0	yadI	5	13.6333	0.632
0.1	0	AG1	5	13.6333	0.779
0.1	0	ptsI	5	13.8833	0.497
0.1	0	clcB	5	13.8833	0.481
0.1	0	ycaM	5	13.8833	0.443
0.1	0	yadI	5	13.8833	0.636
0.1	0	AG1	5	13.8833	0.783
0.1	0	ptsI	5	14.1333	0.51
0.1	0	clcB	5	14.1333	0.487
0.1	0	ycaM	5	14.1333	0.448
0.1	0	yadI	5	14.1333	0.643
0.1	0	AG1	5	14.1333	0.792
0.1	0	ptsI	5	14.3833	0.517
0.1	0	clcB	5	14.3833	0.492
0.1	0	ycaM	5	14.3833	0.455
0.1	0	yadI	5	14.3833	0.65
0.1	0	AG1	5	14.3833	0.8
0.1	0	ptsI	5	14.6333	0.521
0.1	0	clcB	5	14.6333	0.499
0.1	0	ycaM	5	14.6333	0.459
0.1	0	yadI	5	14.6333	0.651
0.1	0	AG1	5	14.6333	0.806
0.1	0	ptsI	5	14.8833	0.529
0.1	0	clcB	5	14.8833	0.498
0.1	0	ycaM	5	14.8833	0.462
0.1	0	yadI	5	14.8833	0.657
0.1	0	AG1	5	14.8833	0.811
0.1	0	ptsI	5	15.1333	0.537
0.1	0	clcB	5	15.1333	0.505
0.1	0	ycaM	5	15.1333	0.466
0.1	0	yadI	5	15.1333	0.663
0.1	0	AG1	5	15.1333	0.816
0.1	0	ptsI	5	15.3833	0.539
0.1	0	clcB	5	15.3833	0.507
0.1	0	ycaM	5	15.3833	0.469

0.1	0	yadI	5	15.3833	0.666
0.1	0	AG1	5	15.3833	0.819
0.1	0	ptsI	5	15.6333	0.545
0.1	0	clcB	5	15.6333	0.506
0.1	0	ycaM	5	15.6333	0.471
0.1	0	yadI	5	15.6333	0.67
0.1	0	AG1	5	15.6333	0.821
0.1	0	ptsI	5	15.8833	0.549
0.1	0	clcB	5	15.8833	0.509
0.1	0	ycaM	5	15.8833	0.473
0.1	0	yadI	5	15.8833	0.673
0.1	0	AG1	5	15.8833	0.826
0.1	0	ptsI	5	16.1333	0.554
0.1	0	clcB	5	16.1333	0.514
0.1	0	ycaM	5	16.1333	0.475
0.1	0	yadI	5	16.1333	0.67
0.1	0	AG1	5	16.1333	0.844
0.1	0	ptsI	5	16.3833	0.561
0.1	0	clcB	5	16.3833	0.512
0.1	0	ycaM	5	16.3833	0.478
0.1	0	yadI	5	16.3833	0.677
0.1	0	AG1	5	16.3833	0.834
0.1	0	ptsI	5	16.6333	0.562
0.1	0	clcB	5	16.6333	0.514
0.1	0	ycaM	5	16.6333	0.484
0.1	0	yadI	5	16.6333	0.683
0.1	0	AG1	5	16.6333	0.84
0.1	0	ptsI	5	16.8833	0.571
0.1	0	clcB	5	16.8833	0.519
0.1	0	ycaM	5	16.8833	0.49
0.1	0	yadI	5	16.8833	0.686
0.1	0	AG1	5	16.8833	0.843
0.1	0	ptsI	5	17.1333	0.578
0.1	0	clcB	5	17.1333	0.521
0.1	0	ycaM	5	17.1333	0.491
0.1	0	yadI	5	17.1333	0.692
0.1	0	AG1	5	17.1333	0.847
0.1	0	ptsI	5	17.3833	0.577
0.1	0	clcB	5	17.3833	0.518
0.1	0	ycaM	5	17.3833	0.492
0.1	0	yadI	5	17.3833	0.687
0.1	0	AG1	5	17.3833	0.848
0.1	0	ptsI	5	17.6333	0.584
0.1	0	clcB	5	17.6333	0.52
0.1	0	ycaM	5	17.6333	0.494
0.1	0	yadI	5	17.6333	0.69
0.1	0	AG1	5	17.6333	0.85
0.1	0	ptsI	5	17.8833	0.592
0.1	0	clcB	5	17.8833	0.523
0.1	0	ycaM	5	17.8833	0.499
0.1	0	yadI	5	17.8833	0.694
0.1	0	AG1	5	17.8833	0.853
0.1	0	ptsI	5	18.1333	0.591

0.1	0	clcB	5	18.1333	0.523
0.1	0	ycaM	5	18.1333	0.503
0.1	0	yadI	5	18.1333	0.698
0.1	0	AG1	5	18.1333	0.856
0.1	0	ptsI	5	18.3833	0.598
0.1	0	clcB	5	18.3833	0.527
0.1	0	ycaM	5	18.3833	0.504
0.1	0	yadI	5	18.3833	0.702
0.1	0	AG1	5	18.3833	0.858
0.1	0	ptsI	5	18.6333	0.603
0.1	0	clcB	5	18.6333	0.527
0.1	0	ycaM	5	18.6333	0.504
0.1	0	yadI	5	18.6333	0.702
0.1	0	AG1	5	18.6333	0.863
0.1	0	ptsI	5	18.8833	0.603
0.1	0	clcB	5	18.8833	0.527
0.1	0	ycaM	5	18.8833	0.507
0.1	0	yadI	5	18.8833	0.705
0.1	0	AG1	5	18.8833	0.864
0.1	0	ptsI	5	19.1333	0.611
0.1	0	clcB	5	19.1333	0.528
0.1	0	ycaM	5	19.1333	0.508
0.1	0	yadI	5	19.1333	0.706
0.1	0	AG1	5	19.1333	0.864
0.1	0	ptsI	5	19.3833	0.616
0.1	0	clcB	5	19.3833	0.532
0.1	0	ycaM	5	19.3833	0.51
0.1	0	yadI	5	19.3833	0.713
0.1	0	AG1	5	19.3833	0.869
0.1	0	ptsI	5	19.6333	0.616
0.1	0	clcB	5	19.6333	0.533
0.1	0	ycaM	5	19.6333	0.516
0.1	0	yadI	5	19.6333	0.713
0.1	0	AG1	5	19.6333	0.872
0.1	0	ptsI	5	19.8833	0.622
0.1	0	clcB	5	19.8833	0.533
0.1	0	ycaM	5	19.8833	0.515
0.1	0	yadI	5	19.8833	0.714
0.1	0	AG1	5	19.8833	0.872
0.1	0	ptsI	5	20.1333	0.625
0.1	0	clcB	5	20.1333	0.534
0.1	0	ycaM	5	20.1333	0.516
0.1	0	yadI	5	20.1333	0.714
0.1	0	AG1	5	20.1333	0.876
0.1	0	ptsI	5	20.3833	0.629
0.1	0	clcB	5	20.3833	0.535
0.1	0	ycaM	5	20.3833	0.517
0.1	0	yadI	5	20.3833	0.718
0.1	0	AG1	5	20.3833	0.876
0.1	0	ptsI	5	20.6333	0.628
0.1	0	clcB	5	20.6333	0.531
0.1	0	ycaM	5	20.6333	0.523
0.1	0	yadI	5	20.6333	0.717

0.1	0	AG1	5	20.6333	0.858
0.1	0	ptsI	5	20.8833	0.633
0.1	0	clcB	5	20.8833	0.538
0.1	0	ycaM	5	20.8833	0.521
0.1	0	yadI	5	20.8833	0.722
0.1	0	AG1	5	20.8833	0.879
0.1	0	ptsI	5	21.1333	0.634
0.1	0	clcB	5	21.1333	0.535
0.1	0	ycaM	5	21.1333	0.521
0.1	0	yadI	5	21.1333	0.723
0.1	0	AG1	5	21.1333	0.879
0.1	0	ptsI	5	21.3833	0.636
0.1	0	clcB	5	21.3833	0.539
0.1	0	ycaM	5	21.3833	0.522
0.1	0	yadI	5	21.3833	0.726
0.1	0	AG1	5	21.3833	0.882
0.1	0	ptsI	5	21.6333	0.639
0.1	0	clcB	5	21.6333	0.539
0.1	0	ycaM	5	21.6333	0.528
0.1	0	yadI	5	21.6333	0.725
0.1	0	AG1	5	21.6333	0.882
0.1	0	ptsI	5	21.8833	0.646
0.1	0	clcB	5	21.8833	0.54
0.1	0	ycaM	5	21.8833	0.531
0.1	0	yadI	5	21.8833	0.729
0.1	0	AG1	5	21.8833	0.884
0.1	0	ptsI	5	22.1333	0.64
0.1	0	clcB	5	22.1333	0.538
0.1	0	ycaM	5	22.1333	0.53
0.1	0	yadI	5	22.1333	0.724
0.1	0	AG1	5	22.1333	0.88
0.1	0	ptsI	5	22.3833	0.65
0.1	0	clcB	5	22.3833	0.539
0.1	0	ycaM	5	22.3833	0.529
0.1	0	yadI	5	22.3833	0.73
0.1	0	AG1	5	22.3833	0.883
0.1	0	ptsI	5	22.6333	0.643
0.1	0	clcB	5	22.6333	0.54
0.1	0	ycaM	5	22.6333	0.535
0.1	0	yadI	5	22.6333	0.732
0.1	0	AG1	5	22.6333	0.886
0.1	0	ptsI	5	22.8833	0.648
0.1	0	clcB	5	22.8833	0.538
0.1	0	ycaM	5	22.8833	0.533
0.1	0	yadI	5	22.8833	0.728
0.1	0	AG1	5	22.8833	0.884
0.1	0	ptsI	5	23.1333	0.651
0.1	0	clcB	5	23.1333	0.539
0.1	0	ycaM	5	23.1333	0.536
0.1	0	yadI	5	23.1333	0.732
0.1	0	AG1	5	23.1333	0.888
0.1	0	ptsI	5	23.3833	0.656
0.1	0	clcB	5	23.3833	0.542

0.1	0	ycaM	5	23.3833	0.534
0.1	0	yadI	5	23.3833	0.736
0.1	0	AG1	5	23.3833	0.887
0.1	0	ptsI	5	23.6333	0.659
0.1	0	clcB	5	23.6333	0.54
0.1	0	ycaM	5	23.6333	0.535
0.1	0	yadI	5	23.6333	0.736
0.1	0	AG1	5	23.6333	0.89
0.1	0	ptsI	5	23.8833	0.659
0.1	0	clcB	5	23.8833	0.54
0.1	0	ycaM	5	23.8833	0.536
0.1	0	yadI	5	23.8833	0.729
0.1	0	AG1	5	23.8833	0.884
0.1	0	ptsI	5	24.1333	0.664
0.1	0	clcB	5	24.1333	0.541
0.1	0	ycaM	5	24.1333	0.543
0.1	0	yadI	5	24.1333	0.739
0.1	0	AG1	5	24.1333	0.889
0.1	0	ptsI	5	24.3833	0.664
0.1	0	clcB	5	24.3833	0.541
0.1	0	ycaM	5	24.3833	0.539
0.1	0	yadI	5	24.3833	0.734
0.1	0	AG1	5	24.3833	0.889
0.2	0	ptsI	1	0	0.244
0.2	0	clcB	1	0	0.241
0.2	0	ycaM	1	0	0.241
0.2	0	yadI	1	0	0.281
0.2	0	AG1	1	0	0.283
0.2	0	ptsI	1	0.25	0.233
0.2	0	clcB	1	0.25	0.223
0.2	0	ycaM	1	0.25	0.224
0.2	0	yadI	1	0.25	0.267
0.2	0	AG1	1	0.25	0.27
0.2	0	ptsI	1	0.5	0.231
0.2	0	clcB	1	0.5	0.22
0.2	0	ycaM	1	0.5	0.217
0.2	0	yadI	1	0.5	0.263
0.2	0	AG1	1	0.5	0.265
0.2	0	ptsI	1	0.75	0.232
0.2	0	clcB	1	0.75	0.222
0.2	0	ycaM	1	0.75	0.217
0.2	0	yadI	1	0.75	0.261
0.2	0	AG1	1	0.75	0.264
0.2	0	ptsI	1	1	0.234
0.2	0	clcB	1	1	0.225
0.2	0	ycaM	1	1	0.217
0.2	0	yadI	1	1	0.262
0.2	0	AG1	1	1	0.266
0.2	0	ptsI	1	1.25	0.239
0.2	0	clcB	1	1.25	0.228
0.2	0	ycaM	1	1.25	0.218
0.2	0	yadI	1	1.25	0.267
0.2	0	AG1	1	1.25	0.264

0.2	0	ptsI	1	1.5	0.241
0.2	0	clcB	1	1.5	0.233
0.2	0	ycaM	1	1.5	0.219
0.2	0	yadI	1	1.5	0.27
0.2	0	AG1	1	1.5	0.267
0.2	0	ptsI	1	1.75	0.25
0.2	0	clcB	1	1.75	0.239
0.2	0	ycaM	1	1.75	0.222
0.2	0	yadI	1	1.75	0.28
0.2	0	AG1	1	1.75	0.268
0.2	0	ptsI	1	2	0.253
0.2	0	clcB	1	2	0.243
0.2	0	ycaM	1	2	0.226
0.2	0	yadI	1	2	0.284
0.2	0	AG1	1	2	0.274
0.2	0	ptsI	1	2.25	0.263
0.2	0	clcB	1	2.25	0.248
0.2	0	ycaM	1	2.25	0.234
0.2	0	yadI	1	2.25	0.293
0.2	0	AG1	1	2.25	0.278
0.2	0	ptsI	1	2.5	0.266
0.2	0	clcB	1	2.5	0.255
0.2	0	ycaM	1	2.5	0.236
0.2	0	yadI	1	2.5	0.299
0.2	0	AG1	1	2.5	0.284
0.2	0	ptsI	1	2.75	0.278
0.2	0	clcB	1	2.75	0.26
0.2	0	ycaM	1	2.75	0.246
0.2	0	yadI	1	2.75	0.312
0.2	0	AG1	1	2.75	0.288
0.2	0	ptsI	1	3	0.284
0.2	0	clcB	1	3	0.268
0.2	0	ycaM	1	3	0.248
0.2	0	yadI	1	3	0.32
0.2	0	AG1	1	3	0.294
0.2	0	ptsI	1	3.25	0.296
0.2	0	clcB	1	3.25	0.272
0.2	0	ycaM	1	3.25	0.259
0.2	0	yadI	1	3.25	0.334
0.2	0	AG1	1	3.25	0.304
0.2	0	ptsI	1	3.5	0.307
0.2	0	clcB	1	3.5	0.279
0.2	0	ycaM	1	3.5	0.268
0.2	0	yadI	1	3.5	0.354
0.2	0	AG1	1	3.5	0.31
0.2	0	ptsI	1	3.75	0.317
0.2	0	clcB	1	3.75	0.286
0.2	0	ycaM	1	3.75	0.275
0.2	0	yadI	1	3.75	0.372
0.2	0	AG1	1	3.75	0.32
0.2	0	ptsI	1	4	0.331
0.2	0	clcB	1	4	0.291
0.2	0	ycaM	1	4	0.289

0.2	0	yadI	1	4	0.387
0.2	0	AG1	1	4	0.319
0.2	0	ptsI	1	4.25	0.333
0.2	0	clcB	1	4.25	0.3
0.2	0	ycaM	1	4.25	0.296
0.2	0	yadI	1	4.25	0.385
0.2	0	AG1	1	4.25	0.338
0.2	0	ptsI	1	4.5	0.342
0.2	0	clcB	1	4.5	0.309
0.2	0	ycaM	1	4.5	0.304
0.2	0	yadI	1	4.5	0.408
0.2	0	AG1	1	4.5	0.353
0.2	0	ptsI	1	4.75	0.355
0.2	0	clcB	1	4.75	0.314
0.2	0	ycaM	1	4.75	0.318
0.2	0	yadI	1	4.75	0.408
0.2	0	AG1	1	4.75	0.362
0.2	0	ptsI	1	5	0.365
0.2	0	clcB	1	5	0.326
0.2	0	ycaM	1	5	0.327
0.2	0	yadI	1	5	0.431
0.2	0	AG1	1	5	0.37
0.2	0	ptsI	1	5.25	0.372
0.2	0	clcB	1	5.25	0.337
0.2	0	ycaM	1	5.25	0.34
0.2	0	yadI	1	5.25	0.448
0.2	0	AG1	1	5.25	0.378
0.2	0	ptsI	1	5.5	0.378
0.2	0	clcB	1	5.5	0.341
0.2	0	ycaM	1	5.5	0.348
0.2	0	yadI	1	5.5	0.456
0.2	0	AG1	1	5.5	0.388
0.2	0	ptsI	1	5.75	0.39
0.2	0	clcB	1	5.75	0.348
0.2	0	ycaM	1	5.75	0.359
0.2	0	yadI	1	5.75	0.47
0.2	0	AG1	1	5.75	0.397
0.2	0	ptsI	1	6	0.408
0.2	0	clcB	1	6	0.356
0.2	0	ycaM	1	6	0.37
0.2	0	yadI	1	6	0.486
0.2	0	AG1	1	6	0.414
0.2	0	ptsI	1	6.25	0.417
0.2	0	clcB	1	6.25	0.37
0.2	0	ycaM	1	6.25	0.379
0.2	0	yadI	1	6.25	0.494
0.2	0	AG1	1	6.25	0.424
0.2	0	ptsI	1	6.5	0.426
0.2	0	clcB	1	6.5	0.382
0.2	0	ycaM	1	6.5	0.389
0.2	0	yadI	1	6.5	0.51
0.2	0	AG1	1	6.5	0.433
0.2	0	ptsI	1	6.75	0.434

0.2	0	clcB	1	6.75	0.388
0.2	0	ycaM	1	6.75	0.397
0.2	0	yadI	1	6.75	0.519
0.2	0	AG1	1	6.75	0.443
0.2	0	ptsI	1	7	0.441
0.2	0	clcB	1	7	0.397
0.2	0	ycaM	1	7	0.406
0.2	0	yadI	1	7	0.53
0.2	0	AG1	1	7	0.454
0.2	0	ptsI	1	7.25	0.448
0.2	0	clcB	1	7.25	0.404
0.2	0	ycaM	1	7.25	0.408
0.2	0	yadI	1	7.25	0.546
0.2	0	AG1	1	7.25	0.464
0.2	0	ptsI	1	7.5	0.457
0.2	0	clcB	1	7.5	0.416
0.2	0	ycaM	1	7.5	0.414
0.2	0	yadI	1	7.5	0.554
0.2	0	AG1	1	7.5	0.468
0.2	0	ptsI	1	7.75	0.488
0.2	0	clcB	1	7.75	0.43
0.2	0	ycaM	1	7.75	0.422
0.2	0	yadI	1	7.75	0.568
0.2	0	AG1	1	7.75	0.479
0.2	0	ptsI	1	8	0.48
0.2	0	clcB	1	8	0.437
0.2	0	ycaM	1	8	0.427
0.2	0	yadI	1	8	0.574
0.2	0	AG1	1	8	0.486
0.2	0	ptsI	1	8.25	0.489
0.2	0	clcB	1	8.25	0.447
0.2	0	ycaM	1	8.25	0.436
0.2	0	yadI	1	8.25	0.587
0.2	0	AG1	1	8.25	0.494
0.2	0	ptsI	1	8.5	0.5
0.2	0	clcB	1	8.5	0.458
0.2	0	ycaM	1	8.5	0.45
0.2	0	yadI	1	8.5	0.596
0.2	0	AG1	1	8.5	0.503
0.2	0	ptsI	1	8.75	0.509
0.2	0	clcB	1	8.75	0.463
0.2	0	ycaM	1	8.75	0.464
0.2	0	yadI	1	8.75	0.605
0.2	0	AG1	1	8.75	0.514
0.2	0	ptsI	1	9	0.519
0.2	0	clcB	1	9	0.471
0.2	0	ycaM	1	9	0.483
0.2	0	yadI	1	9	0.619
0.2	0	AG1	1	9	0.523
0.2	0	ptsI	1	9.25	0.546
0.2	0	clcB	1	9.25	0.482
0.2	0	ycaM	1	9.25	0.488
0.2	0	yadI	1	9.25	0.62

0.2	0	AG1	1	9.25	0.533
0.2	0	ptsI	1	9.5	0.57
0.2	0	clcB	1	9.5	0.498
0.2	0	ycaM	1	9.5	0.504
0.2	0	yadI	1	9.5	0.634
0.2	0	AG1	1	9.5	0.548
0.2	0	ptsI	1	9.75	0.557
0.2	0	clcB	1	9.75	0.508
0.2	0	ycaM	1	9.75	0.519
0.2	0	yadI	1	9.75	0.666
0.2	0	AG1	1	9.75	0.565
0.2	0	ptsI	1	10	0.588
0.2	0	clcB	1	10	0.517
0.2	0	ycaM	1	10	0.531
0.2	0	yadI	1	10	0.66
0.2	0	AG1	1	10	0.569
0.2	0	ptsI	1	10.25	0.599
0.2	0	clcB	1	10.25	0.524
0.2	0	ycaM	1	10.25	0.552
0.2	0	yadI	1	10.25	0.671
0.2	0	AG1	1	10.25	0.58
0.2	0	ptsI	1	10.5	0.616
0.2	0	clcB	1	10.5	0.533
0.2	0	ycaM	1	10.5	0.572
0.2	0	yadI	1	10.5	0.668
0.2	0	AG1	1	10.5	0.584
0.2	0	ptsI	1	10.75	0.667
0.2	0	clcB	1	10.75	0.545
0.2	0	ycaM	1	10.75	0.567
0.2	0	yadI	1	10.75	0.683
0.2	0	AG1	1	10.75	0.586
0.2	0	ptsI	1	11	0.68
0.2	0	clcB	1	11	0.566
0.2	0	ycaM	1	11	0.594
0.2	0	yadI	1	11	0.664
0.2	0	AG1	1	11	0.596
0.2	0	ptsI	1	11.25	0.674
0.2	0	clcB	1	11.25	0.567
0.2	0	ycaM	1	11.25	0.616
0.2	0	yadI	1	11.25	0.685
0.2	0	AG1	1	11.25	0.602
0.2	0	ptsI	1	11.5	0.675
0.2	0	clcB	1	11.5	0.583
0.2	0	ycaM	1	11.5	0.628
0.2	0	yadI	1	11.5	0.693
0.2	0	AG1	1	11.5	0.618
0.2	0	ptsI	1	11.75	0.705
0.2	0	clcB	1	11.75	0.594
0.2	0	ycaM	1	11.75	0.643
0.2	0	yadI	1	11.75	0.704
0.2	0	AG1	1	11.75	0.621
0.2	0	ptsI	1	12	0.721
0.2	0	clcB	1	12	0.595

0.2	0	ycaM	1	12	0.633
0.2	0	yadI	1	12	0.708
0.2	0	AG1	1	12	0.63
0.2	0	ptsI	1	12.25	0.715
0.2	0	clcB	1	12.25	0.603
0.2	0	ycaM	1	12.25	0.645
0.2	0	yadI	1	12.25	0.679
0.2	0	AG1	1	12.25	0.637
0.2	0	ptsI	1	12.5	0.715
0.2	0	clcB	1	12.5	0.609
0.2	0	ycaM	1	12.5	0.629
0.2	0	yadI	1	12.5	0.703
0.2	0	AG1	1	12.5	0.656
0.2	0	ptsI	1	12.75	0.74
0.2	0	clcB	1	12.75	0.622
0.2	0	ycaM	1	12.75	0.629
0.2	0	yadI	1	12.75	0.708
0.2	0	AG1	1	12.75	0.67
0.2	0	ptsI	1	13	0.751
0.2	0	clcB	1	13	0.618
0.2	0	ycaM	1	13	0.646
0.2	0	yadI	1	13	0.724
0.2	0	AG1	1	13	0.683
0.2	0	ptsI	1	13.25	0.756
0.2	0	clcB	1	13.25	0.633
0.2	0	ycaM	1	13.25	0.682
0.2	0	yadI	1	13.25	0.724
0.2	0	AG1	1	13.25	0.689
0.2	0	ptsI	1	13.5	0.771
0.2	0	clcB	1	13.5	0.637
0.2	0	ycaM	1	13.5	0.69
0.2	0	yadI	1	13.5	0.738
0.2	0	AG1	1	13.5	0.716
0.2	0	ptsI	1	13.75	0.777
0.2	0	clcB	1	13.75	0.649
0.2	0	ycaM	1	13.75	0.688
0.2	0	yadI	1	13.75	0.745
0.2	0	AG1	1	13.75	0.725
0.2	0	ptsI	1	14	0.805
0.2	0	clcB	1	14	0.667
0.2	0	ycaM	1	14	0.707
0.2	0	yadI	1	14	0.753
0.2	0	AG1	1	14	0.753
0.2	0	ptsI	1	14.25	0.806
0.2	0	clcB	1	14.25	0.698
0.2	0	ycaM	1	14.25	0.712
0.2	0	yadI	1	14.25	0.757
0.2	0	AG1	1	14.25	0.759
0.2	0	ptsI	1	14.5	0.796
0.2	0	clcB	1	14.5	0.724
0.2	0	ycaM	1	14.5	0.713
0.2	0	yadI	1	14.5	0.761
0.2	0	AG1	1	14.5	0.767

0.2	0	ptsI	1	14.75	0.796
0.2	0	clcB	1	14.75	0.717
0.2	0	ycaM	1	14.75	0.724
0.2	0	yadI	1	14.75	0.731
0.2	0	AG1	1	14.75	0.771
0.2	0	ptsI	1	15	0.809
0.2	0	clcB	1	15	0.732
0.2	0	ycaM	1	15	0.73
0.2	0	yadI	1	15	0.739
0.2	0	AG1	1	15	0.794
0.2	0	ptsI	1	15.25	0.83
0.2	0	clcB	1	15.25	0.736
0.2	0	ycaM	1	15.25	0.741
0.2	0	yadI	1	15.25	0.752
0.2	0	AG1	1	15.25	0.81
0.2	0	ptsI	1	15.5	0.815
0.2	0	clcB	1	15.5	0.749
0.2	0	ycaM	1	15.5	0.743
0.2	0	yadI	1	15.5	0.737
0.2	0	AG1	1	15.5	0.807
0.2	0	ptsI	1	15.75	0.808
0.2	0	clcB	1	15.75	0.746
0.2	0	ycaM	1	15.75	0.746
0.2	0	yadI	1	15.75	0.733
0.2	0	AG1	1	15.75	0.804
0.2	0	ptsI	1	16	0.816
0.2	0	clcB	1	16	0.759
0.2	0	ycaM	1	16	0.752
0.2	0	yadI	1	16	0.729
0.2	0	AG1	1	16	0.802
0.2	0	ptsI	1	16.25	0.83
0.2	0	clcB	1	16.25	0.774
0.2	0	ycaM	1	16.25	0.763
0.2	0	yadI	1	16.25	0.74
0.2	0	AG1	1	16.25	0.824
0.2	0	ptsI	1	16.5	0.844
0.2	0	clcB	1	16.5	0.782
0.2	0	ycaM	1	16.5	0.77
0.2	0	yadI	1	16.5	0.747
0.2	0	AG1	1	16.5	0.843
0.2	0	ptsI	1	17.0667	0.867
0.2	0	clcB	1	17.0667	0.85
0.2	0	ycaM	1	17.0667	0.809
0.2	0	yadI	1	17.0667	0.784
0.2	0	AG1	1	17.0667	0.854
0.2	0	ptsI	1	17.3167	0.81
0.2	0	clcB	1	17.3167	0.772
0.2	0	ycaM	1	17.3167	0.774
0.2	0	yadI	1	17.3167	0.774
0.2	0	AG1	1	17.3167	0.824
0.2	0	ptsI	1	17.5667	0.816
0.2	0	clcB	1	17.5667	0.771
0.2	0	ycaM	1	17.5667	0.762

0.2	0	yadI	1	17.5667	0.772
0.2	0	AG1	1	17.5667	0.824
0.2	0	ptsI	1	17.8167	0.839
0.2	0	clcB	1	17.8167	0.799
0.2	0	ycaM	1	17.8167	0.766
0.2	0	yadI	1	17.8167	0.779
0.2	0	AG1	1	17.8167	0.866
0.2	0	ptsI	1	18.0667	0.842
0.2	0	clcB	1	18.0667	0.778
0.2	0	ycaM	1	18.0667	0.774
0.2	0	yadI	1	18.0667	0.78
0.2	0	AG1	1	18.0667	0.816
0.2	0	ptsI	1	18.3167	0.846
0.2	0	clcB	1	18.3167	0.78
0.2	0	ycaM	1	18.3167	0.773
0.2	0	yadI	1	18.3167	0.781
0.2	0	AG1	1	18.3167	0.778
0.2	0	ptsI	1	18.5667	0.861
0.2	0	clcB	1	18.5667	0.8
0.2	0	ycaM	1	18.5667	0.78
0.2	0	yadI	1	18.5667	0.802
0.2	0	AG1	1	18.5667	0.818
0.2	0	ptsI	1	18.8167	0.865
0.2	0	clcB	1	18.8167	0.808
0.2	0	ycaM	1	18.8167	0.785
0.2	0	yadI	1	18.8167	0.79
0.2	0	AG1	1	18.8167	0.826
0.2	0	ptsI	1	19.0667	0.872
0.2	0	clcB	1	19.0667	0.811
0.2	0	ycaM	1	19.0667	0.79
0.2	0	yadI	1	19.0667	0.798
0.2	0	AG1	1	19.0667	0.834
0.2	0	ptsI	1	19.3167	0.875
0.2	0	clcB	1	19.3167	0.818
0.2	0	ycaM	1	19.3167	0.789
0.2	0	yadI	1	19.3167	0.81
0.2	0	AG1	1	19.3167	0.843
0.2	0	ptsI	1	19.5667	0.884
0.2	0	clcB	1	19.5667	0.822
0.2	0	ycaM	1	19.5667	0.789
0.2	0	yadI	1	19.5667	0.824
0.2	0	AG1	1	19.5667	0.845
0.2	0	ptsI	1	19.8167	0.886
0.2	0	clcB	1	19.8167	0.821
0.2	0	ycaM	1	19.8167	0.797
0.2	0	yadI	1	19.8167	0.815
0.2	0	AG1	1	19.8167	0.848
0.2	0	ptsI	1	20.0667	0.897
0.2	0	clcB	1	20.0667	0.83
0.2	0	ycaM	1	20.0667	0.799
0.2	0	yadI	1	20.0667	0.823
0.2	0	AG1	1	20.0667	0.857
0.2	0	ptsI	1	20.3167	0.902

0.2	0	clcB	1	20.3167	0.83
0.2	0	ycaM	1	20.3167	0.804
0.2	0	yadI	1	20.3167	0.824
0.2	0	AG1	1	20.3167	0.858
0.2	0	ptsI	1	20.5667	0.898
0.2	0	clcB	1	20.5667	0.822
0.2	0	ycaM	1	20.5667	0.803
0.2	0	yadI	1	20.5667	0.836
0.2	0	AG1	1	20.5667	0.846
0.2	0	ptsI	1	20.8167	0.91
0.2	0	clcB	1	20.8167	0.828
0.2	0	ycaM	1	20.8167	0.811
0.2	0	yadI	1	20.8167	0.831
0.2	0	AG1	1	20.8167	0.833
0.2	0	ptsI	1	21.0667	0.926
0.2	0	clcB	1	21.0667	0.835
0.2	0	ycaM	1	21.0667	0.818
0.2	0	yadI	1	21.0667	0.841
0.2	0	AG1	1	21.0667	0.842
0.2	0	ptsI	1	21.3167	0.935
0.2	0	clcB	1	21.3167	0.84
0.2	0	ycaM	1	21.3167	0.82
0.2	0	yadI	1	21.3167	0.851
0.2	0	AG1	1	21.3167	0.838
0.2	0	ptsI	1	21.5667	0.933
0.2	0	clcB	1	21.5667	0.836
0.2	0	ycaM	1	21.5667	0.819
0.2	0	yadI	1	21.5667	0.857
0.2	0	AG1	1	21.5667	0.842
0.2	0	ptsI	1	21.8167	0.943
0.2	0	clcB	1	21.8167	0.835
0.2	0	ycaM	1	21.8167	0.826
0.2	0	yadI	1	21.8167	0.856
0.2	0	AG1	1	21.8167	0.832
0.2	0	ptsI	1	22.0667	0.963
0.2	0	clcB	1	22.0667	0.842
0.2	0	ycaM	1	22.0667	0.834
0.2	0	yadI	1	22.0667	0.859
0.2	0	AG1	1	22.0667	0.842
0.2	0	ptsI	1	22.9333	0.703
0.2	0	clcB	1	22.9333	0.912
0.2	0	ycaM	1	22.9333	0.704
0.2	0	yadI	1	22.9333	0.856
0.2	0	AG1	1	22.9333	0.844
0.2	0	ptsI	1	23.1833	0.745
0.2	0	clcB	1	23.1833	0.909
0.2	0	ycaM	1	23.1833	0.734
0.2	0	yadI	1	23.1833	0.874
0.2	0	AG1	1	23.1833	0.833
0.2	0	ptsI	1	23.4333	0.768
0.2	0	clcB	1	23.4333	0.899
0.2	0	ycaM	1	23.4333	0.767
0.2	0	yadI	1	23.4333	0.875

0.2	0	AG1	1	23.4333	0.845
0.2	0	ptsI	1	23.6833	0.793
0.2	0	clcB	1	23.6833	0.896
0.2	0	ycaM	1	23.6833	0.788
0.2	0	yadI	1	23.6833	0.88
0.2	0	AG1	1	23.6833	0.83
0.2	0	ptsI	1	23.9333	0.822
0.2	0	clcB	1	23.9333	0.892
0.2	0	ycaM	1	23.9333	0.793
0.2	0	yadI	1	23.9333	0.886
0.2	0	AG1	1	23.9333	0.851
0.2	0	ptsI	1	24.1833	0.841
0.2	0	clcB	1	24.1833	0.885
0.2	0	ycaM	1	24.1833	0.806
0.2	0	yadI	1	24.1833	0.897
0.2	0	AG1	1	24.1833	0.865
0.2	0	ptsI	1	24.4333	0.854
0.2	0	clcB	1	24.4333	0.896
0.2	0	ycaM	1	24.4333	0.807
0.2	0	yadI	1	24.4333	0.906
0.2	0	AG1	1	24.4333	0.859
0.2	0	ptsI	1	24.6833	0.861
0.2	0	clcB	1	24.6833	0.892
0.2	0	ycaM	1	24.6833	0.808
0.2	0	yadI	1	24.6833	0.909
0.2	0	AG1	1	24.6833	0.864
0.2	0	ptsI	1	24.9333	0.875
0.2	0	clcB	1	24.9333	0.895
0.2	0	ycaM	1	24.9333	0.814
0.2	0	yadI	1	24.9333	0.93
0.2	0	AG1	1	24.9333	0.861
0.2	0	ptsI	1	25.1833	0.886
0.2	0	clcB	1	25.1833	0.9
0.2	0	ycaM	1	25.1833	0.82
0.2	0	yadI	1	25.1833	0.926
0.2	0	AG1	1	25.1833	0.871
0.2	0	ptsI	1	25.4333	0.9
0.2	0	clcB	1	25.4333	0.899
0.2	0	ycaM	1	25.4333	0.832
0.2	0	yadI	1	25.4333	0.935
0.2	0	AG1	1	25.4333	0.887
0.2	0	ptsI	1	25.6833	0.907
0.2	0	clcB	1	25.6833	0.908
0.2	0	ycaM	1	25.6833	0.845
0.2	0	yadI	1	25.6833	0.939
0.2	0	AG1	1	25.6833	0.886
0.2	0	ptsI	1	25.9333	0.927
0.2	0	clcB	1	25.9333	0.908
0.2	0	ycaM	1	25.9333	0.854
0.2	0	yadI	1	25.9333	0.96
0.2	0	AG1	1	25.9333	0.894
0.2	0	ptsI	1	26.1833	0.924
0.2	0	clcB	1	26.1833	0.911

0.2	0	ycaM	1	26.1833	0.866
0.2	0	yadI	1	26.1833	0.955
0.2	0	AG1	1	26.1833	0.913
0.2	0	ptsI	1	26.4333	0.93
0.2	0	clcB	1	26.4333	0.909
0.2	0	ycaM	1	26.4333	0.871
0.2	0	yadI	1	26.4333	0.961
0.2	0	AG1	1	26.4333	0.906
0.2	0	ptsI	1	26.6833	0.939
0.2	0	clcB	1	26.6833	0.913
0.2	0	ycaM	1	26.6833	0.882
0.2	0	yadI	1	26.6833	0.962
0.2	0	AG1	1	26.6833	0.911
0.2	0	ptsI	1	26.9333	0.945
0.2	0	clcB	1	26.9333	0.911
0.2	0	ycaM	1	26.9333	0.889
0.2	0	yadI	1	26.9333	0.968
0.2	0	AG1	1	26.9333	0.917
0.2	0	ptsI	1	27.1833	0.946
0.2	0	clcB	1	27.1833	0.912
0.2	0	ycaM	1	27.1833	0.9
0.2	0	yadI	1	27.1833	0.979
0.2	0	AG1	1	27.1833	0.923
0.2	0	ptsI	1	27.4333	0.955
0.2	0	clcB	1	27.4333	0.915
0.2	0	ycaM	1	27.4333	0.906
0.2	0	yadI	1	27.4333	0.974
0.2	0	AG1	1	27.4333	0.916
0.2	0	ptsI	1	27.6833	0.965
0.2	0	clcB	1	27.6833	0.919
0.2	0	ycaM	1	27.6833	0.913
0.2	0	yadI	1	27.6833	0.982
0.2	0	AG1	1	27.6833	0.926
0.2	0	ptsI	1	27.9333	0.967
0.2	0	clcB	1	27.9333	0.909
0.2	0	ycaM	1	27.9333	0.924
0.2	0	yadI	1	27.9333	0.99
0.2	0	AG1	1	27.9333	0.916
0.2	0	ptsI	1	28.1833	0.972
0.2	0	clcB	1	28.1833	0.916
0.2	0	ycaM	1	28.1833	0.933
0.2	0	yadI	1	28.1833	0.994
0.2	0	AG1	1	28.1833	0.939
0.2	0	ptsI	1	28.4333	0.985
0.2	0	clcB	1	28.4333	0.926
0.2	0	ycaM	1	28.4333	0.938
0.2	0	yadI	1	28.4333	0.995
0.2	0	AG1	1	28.4333	0.937
0.2	0	ptsI	1	28.6833	0.992
0.2	0	clcB	1	28.6833	0.928
0.2	0	ycaM	1	28.6833	0.948
0.2	0	yadI	1	28.6833	1.002
0.2	0	AG1	1	28.6833	0.939

0.2	0	ptsI	1	28.9333	0.997
0.2	0	clcB	1	28.9333	0.933
0.2	0	ycaM	1	28.9333	0.954
0.2	0	yadI	1	28.9333	1.009
0.2	0	AG1	1	28.9333	0.948
0.2	0	ptsI	1	29.1833	1.005
0.2	0	clcB	1	29.1833	0.936
0.2	0	ycaM	1	29.1833	0.958
0.2	0	yadI	1	29.1833	1.003
0.2	0	AG1	1	29.1833	0.942
0.2	0	ptsI	1	29.4333	1.012
0.2	0	clcB	1	29.4333	0.94
0.2	0	ycaM	1	29.4333	0.964
0.2	0	yadI	1	29.4333	1.016
0.2	0	AG1	1	29.4333	0.95
0.2	0	ptsI	1	29.6833	1.019
0.2	0	clcB	1	29.6833	0.944
0.2	0	ycaM	1	29.6833	0.972
0.2	0	yadI	1	29.6833	1.018
0.2	0	AG1	1	29.6833	0.954
0.2	0	ptsI	1	29.9333	1.025
0.2	0	clcB	1	29.9333	0.945
0.2	0	ycaM	1	29.9333	0.966
0.2	0	yadI	1	29.9333	1.027
0.2	0	AG1	1	29.9333	0.961
0.2	0	ptsI	1	30.1833	1.034
0.2	0	clcB	1	30.1833	0.944
0.2	0	ycaM	1	30.1833	0.994
0.2	0	yadI	1	30.1833	1.03
0.2	0	AG1	1	30.1833	0.964
0.2	0	ptsI	1	30.4333	1.044
0.2	0	clcB	1	30.4333	0.953
0.2	0	ycaM	1	30.4333	0.984
0.2	0	yadI	1	30.4333	1.039
0.2	0	AG1	1	30.4333	0.968
0.2	0	ptsI	1	30.6833	1.051
0.2	0	clcB	1	30.6833	0.954
0.2	0	ycaM	1	30.6833	0.984
0.2	0	yadI	1	30.6833	1.033
0.2	0	AG1	1	30.6833	0.968
0.2	0	ptsI	1	30.9333	1.054
0.2	0	clcB	1	30.9333	0.955
0.2	0	ycaM	1	30.9333	1.003
0.2	0	yadI	1	30.9333	1.042
0.2	0	AG1	1	30.9333	0.972
0.2	0	ptsI	1	31.1833	1.061
0.2	0	clcB	1	31.1833	0.957
0.2	0	ycaM	1	31.1833	0.987
0.2	0	yadI	1	31.1833	1.044
0.2	0	AG1	1	31.1833	0.976
0.2	0	ptsI	1	31.4333	1.073
0.2	0	clcB	1	31.4333	0.96
0.2	0	ycaM	1	31.4333	1.008

0.2	0	yadI	1	31.4333	1.058
0.2	0	AG1	1	31.4333	0.989
0.2	0	ptsI	1	31.6833	1.08
0.2	0	clcB	1	31.6833	0.968
0.2	0	ycaM	1	31.6833	1.009
0.2	0	yadI	1	31.6833	1.065
0.2	0	AG1	1	31.6833	0.99
0.2	0	ptsI	1	31.9333	1.086
0.2	0	clcB	1	31.9333	0.976
0.2	0	ycaM	1	31.9333	1.007
0.2	0	yadI	1	31.9333	1.073
0.2	0	AG1	1	31.9333	0.998
0.2	0	ptsI	1	32.1833	1.093
0.2	0	clcB	1	32.1833	0.978
0.2	0	ycaM	1	32.1833	1.01
0.2	0	yadI	1	32.1833	1.083
0.2	0	AG1	1	32.1833	1
0.2	0	ptsI	1	32.4333	1.107
0.2	0	clcB	1	32.4333	0.973
0.2	0	ycaM	1	32.4333	1.011
0.2	0	yadI	1	32.4333	1.102
0.2	0	AG1	1	32.4333	0.988
0.2	0	ptsI	1	32.6833	1.109
0.2	0	clcB	1	32.6833	0.99
0.2	0	ycaM	1	32.6833	1.016
0.2	0	yadI	1	32.6833	1.096
0.2	0	AG1	1	32.6833	1.01
0.2	0	ptsI	1	32.9333	1.117
0.2	0	clcB	1	32.9333	1.005
0.2	0	ycaM	1	32.9333	1.017
0.2	0	yadI	1	32.9333	1.108
0.2	0	AG1	1	32.9333	1.016
0.2	0	ptsI	1	33.1833	1.129
0.2	0	clcB	1	33.1833	1.005
0.2	0	ycaM	1	33.1833	1.025
0.2	0	yadI	1	33.1833	1.12
0.2	0	AG1	1	33.1833	1.012
0.2	0	ptsI	1	33.4333	1.135
0.2	0	clcB	1	33.4333	1.022
0.2	0	ycaM	1	33.4333	1.03
0.2	0	yadI	1	33.4333	1.13
0.2	0	AG1	1	33.4333	1.035
0.2	0	ptsI	1	33.6833	1.148
0.2	0	clcB	1	33.6833	1.018
0.2	0	ycaM	1	33.6833	1.029
0.2	0	yadI	1	33.6833	1.146
0.2	0	AG1	1	33.6833	1.028
0.2	0	ptsI	1	33.9333	1.149
0.2	0	clcB	1	33.9333	1.038
0.2	0	ycaM	1	33.9333	1.028
0.2	0	yadI	1	33.9333	1.15
0.2	0	AG1	1	33.9333	1.04
0.2	0	ptsI	1	34.1833	1.156

0.2	0	clcB	1	34.1833	1.031
0.2	0	ycaM	1	34.1833	1.032
0.2	0	yadI	1	34.1833	1.144
0.2	0	AG1	1	34.1833	1.046
0.2	0	ptsI	1	34.4333	1.17
0.2	0	clcB	1	34.4333	1.045
0.2	0	ycaM	1	34.4333	1.034
0.2	0	yadI	1	34.4333	1.17
0.2	0	AG1	1	34.4333	1.059
0.2	0	ptsI	1	34.6833	1.185
0.2	0	clcB	1	34.6833	1.043
0.2	0	ycaM	1	34.6833	1.041
0.2	0	yadI	1	34.6833	1.182
0.2	0	AG1	1	34.6833	1.065
0.2	0	ptsI	1	34.9333	1.187
0.2	0	clcB	1	34.9333	1.042
0.2	0	ycaM	1	34.9333	1.04
0.2	0	yadI	1	34.9333	1.191
0.2	0	AG1	1	34.9333	1.071
0.2	0	ptsI	1	35.1833	1.2
0.2	0	clcB	1	35.1833	1.04
0.2	0	ycaM	1	35.1833	1.043
0.2	0	yadI	1	35.1833	1.2
0.2	0	AG1	1	35.1833	1.077
0.2	0	ptsI	1	35.4333	1.21
0.2	0	clcB	1	35.4333	1.052
0.2	0	ycaM	1	35.4333	1.042
0.2	0	yadI	1	35.4333	1.21
0.2	0	AG1	1	35.4333	1.091
0.2	0	ptsI	1	35.6833	1.223
0.2	0	clcB	1	35.6833	1.038
0.2	0	ycaM	1	35.6833	1.053
0.2	0	yadI	1	35.6833	1.219
0.2	0	AG1	1	35.6833	1.096
0.2	0	ptsI	1	35.9333	1.234
0.2	0	clcB	1	35.9333	1.034
0.2	0	ycaM	1	35.9333	1.047
0.2	0	yadI	1	35.9333	1.236
0.2	0	AG1	1	35.9333	1.098
0.2	0	ptsI	1	36.1833	1.238
0.2	0	clcB	1	36.1833	1.043
0.2	0	ycaM	1	36.1833	1.053
0.2	0	yadI	1	36.1833	1.235
0.2	0	AG1	1	36.1833	1.113
0.2	0	ptsI	1	36.4333	1.24
0.2	0	clcB	1	36.4333	1.049
0.2	0	ycaM	1	36.4333	1.056
0.2	0	yadI	1	36.4333	1.245
0.2	0	AG1	1	36.4333	1.122
0.2	0	ptsI	1	36.6833	1.256
0.2	0	clcB	1	36.6833	1.042
0.2	0	ycaM	1	36.6833	1.056
0.2	0	yadI	1	36.6833	1.254

0.2	0	AG1	1	36.6833	1.129
0.2	0	ptsI	2	0	0.213
0.2	0	clcB	2	0	0.206
0.2	0	ycaM	2	0	0.218
0.2	0	yadI	2	0	0.22
0.2	0	AG1	2	0	0.226
0.2	0	ptsI	2	0.25	0.213
0.2	0	clcB	2	0.25	0.201
0.2	0	ycaM	2	0.25	0.211
0.2	0	yadI	2	0.25	0.219
0.2	0	AG1	2	0.25	0.221
0.2	0	ptsI	2	0.5	0.214
0.2	0	clcB	2	0.5	0.201
0.2	0	ycaM	2	0.5	0.208
0.2	0	yadI	2	0.5	0.219
0.2	0	AG1	2	0.5	0.222
0.2	0	ptsI	2	0.75	0.217
0.2	0	clcB	2	0.75	0.201
0.2	0	ycaM	2	0.75	0.21
0.2	0	yadI	2	0.75	0.221
0.2	0	AG1	2	0.75	0.221
0.2	0	ptsI	2	1	0.221
0.2	0	clcB	2	1	0.202
0.2	0	ycaM	2	1	0.213
0.2	0	yadI	2	1	0.226
0.2	0	AG1	2	1	0.228
0.2	0	ptsI	2	1.25	0.224
0.2	0	clcB	2	1.25	0.204
0.2	0	ycaM	2	1.25	0.216
0.2	0	yadI	2	1.25	0.235
0.2	0	AG1	2	1.25	0.233
0.2	0	ptsI	2	1.5	0.228
0.2	0	clcB	2	1.5	0.208
0.2	0	ycaM	2	1.5	0.219
0.2	0	yadI	2	1.5	0.239
0.2	0	AG1	2	1.5	0.236
0.2	0	ptsI	2	1.75	0.235
0.2	0	clcB	2	1.75	0.211
0.2	0	ycaM	2	1.75	0.223
0.2	0	yadI	2	1.75	0.248
0.2	0	AG1	2	1.75	0.248
0.2	0	ptsI	2	2	0.241
0.2	0	clcB	2	2	0.214
0.2	0	ycaM	2	2	0.229
0.2	0	yadI	2	2	0.255
0.2	0	AG1	2	2	0.26
0.2	0	ptsI	2	2.25	0.25
0.2	0	clcB	2	2.25	0.216
0.2	0	ycaM	2	2.25	0.234
0.2	0	yadI	2	2.25	0.267
0.2	0	AG1	2	2.25	0.272
0.2	0	ptsI	2	2.5	0.262
0.2	0	clcB	2	2.5	0.223

0.2	0	ycaM	2	2.5	0.242
0.2	0	yadI	2	2.5	0.282
0.2	0	AG1	2	2.5	0.285
0.2	0	ptsI	2	2.75	0.272
0.2	0	clcB	2	2.75	0.227
0.2	0	ycaM	2	2.75	0.248
0.2	0	yadI	2	2.75	0.293
0.2	0	AG1	2	2.75	0.279
0.2	0	ptsI	2	3	0.28
0.2	0	clcB	2	3	0.234
0.2	0	ycaM	2	3	0.256
0.2	0	yadI	2	3	0.312
0.2	0	AG1	2	3	0.285
0.2	0	ptsI	2	3.25	0.298
0.2	0	clcB	2	3.25	0.238
0.2	0	ycaM	2	3.25	0.265
0.2	0	yadI	2	3.25	0.33
0.2	0	AG1	2	3.25	0.296
0.2	0	ptsI	2	3.5	0.305
0.2	0	clcB	2	3.5	0.24
0.2	0	ycaM	2	3.5	0.274
0.2	0	yadI	2	3.5	0.342
0.2	0	AG1	2	3.5	0.311
0.2	0	ptsI	2	3.75	0.316
0.2	0	clcB	2	3.75	0.247
0.2	0	ycaM	2	3.75	0.285
0.2	0	yadI	2	3.75	0.362
0.2	0	AG1	2	3.75	0.326
0.2	0	ptsI	2	4	0.329
0.2	0	clcB	2	4	0.254
0.2	0	ycaM	2	4	0.293
0.2	0	yadI	2	4	0.38
0.2	0	AG1	2	4	0.339
0.2	0	ptsI	2	4.38333	0.346
0.2	0	clcB	2	4.38333	0.312
0.2	0	ycaM	2	4.38333	0.315
0.2	0	yadI	2	4.38333	0.409
0.2	0	AG1	2	4.38333	0.304
0.2	0	ptsI	2	4.63333	0.341
0.2	0	clcB	2	4.63333	0.306
0.2	0	ycaM	2	4.63333	0.31
0.2	0	yadI	2	4.63333	0.406
0.2	0	AG1	2	4.63333	0.3
0.2	0	ptsI	2	4.88333	0.345
0.2	0	clcB	2	4.88333	0.308
0.2	0	ycaM	2	4.88333	0.319
0.2	0	yadI	2	4.88333	0.419
0.2	0	AG1	2	4.88333	0.308
0.2	0	ptsI	2	5.13333	0.355
0.2	0	clcB	2	5.13333	0.313
0.2	0	ycaM	2	5.13333	0.326
0.2	0	yadI	2	5.13333	0.429
0.2	0	AG1	2	5.13333	0.316

0.2	0	ptsI	2	5.38333	0.363
0.2	0	clcB	2	5.38333	0.32
0.2	0	ycaM	2	5.38333	0.336
0.2	0	yadI	2	5.38333	0.44
0.2	0	AG1	2	5.38333	0.322
0.2	0	ptsI	2	5.63333	0.375
0.2	0	clcB	2	5.63333	0.328
0.2	0	ycaM	2	5.63333	0.346
0.2	0	yadI	2	5.63333	0.453
0.2	0	AG1	2	5.63333	0.332
0.2	0	ptsI	2	5.88333	0.382
0.2	0	clcB	2	5.88333	0.339
0.2	0	ycaM	2	5.88333	0.359
0.2	0	yadI	2	5.88333	0.463
0.2	0	AG1	2	5.88333	0.34
0.2	0	ptsI	2	6.13333	0.391
0.2	0	clcB	2	6.13333	0.344
0.2	0	ycaM	2	6.13333	0.37
0.2	0	yadI	2	6.13333	0.471
0.2	0	AG1	2	6.13333	0.353
0.2	0	ptsI	2	6.38333	0.401
0.2	0	clcB	2	6.38333	0.352
0.2	0	ycaM	2	6.38333	0.381
0.2	0	yadI	2	6.38333	0.483
0.2	0	AG1	2	6.38333	0.364
0.2	0	ptsI	2	6.63333	0.408
0.2	0	clcB	2	6.63333	0.362
0.2	0	ycaM	2	6.63333	0.388
0.2	0	yadI	2	6.63333	0.492
0.2	0	AG1	2	6.63333	0.373
0.2	0	ptsI	2	6.88333	0.418
0.2	0	clcB	2	6.88333	0.367
0.2	0	ycaM	2	6.88333	0.396
0.2	0	yadI	2	6.88333	0.5
0.2	0	AG1	2	6.88333	0.383
0.2	0	ptsI	2	7.13333	0.427
0.2	0	clcB	2	7.13333	0.372
0.2	0	ycaM	2	7.13333	0.403
0.2	0	yadI	2	7.13333	0.512
0.2	0	AG1	2	7.13333	0.394
0.2	0	ptsI	2	7.38333	0.436
0.2	0	clcB	2	7.38333	0.382
0.2	0	ycaM	2	7.38333	0.412
0.2	0	yadI	2	7.38333	0.525
0.2	0	AG1	2	7.38333	0.4
0.2	0	ptsI	2	7.63333	0.444
0.2	0	clcB	2	7.63333	0.392
0.2	0	ycaM	2	7.63333	0.422
0.2	0	yadI	2	7.63333	0.535
0.2	0	AG1	2	7.63333	0.409
0.2	0	ptsI	2	7.88333	0.454
0.2	0	clcB	2	7.88333	0.402
0.2	0	ycaM	2	7.88333	0.428

0.2	0	yadI	2	7.88333	0.548
0.2	0	AG1	2	7.88333	0.417
0.2	0	ptsI	2	8.13333	0.464
0.2	0	clcB	2	8.13333	0.406
0.2	0	ycaM	2	8.13333	0.433
0.2	0	yadI	2	8.13333	0.557
0.2	0	AG1	2	8.13333	0.429
0.2	0	ptsI	2	8.38333	0.472
0.2	0	clcB	2	8.38333	0.42
0.2	0	ycaM	2	8.38333	0.438
0.2	0	yadI	2	8.38333	0.57
0.2	0	AG1	2	8.38333	0.436
0.2	0	ptsI	2	8.91667	0.543
0.2	0	clcB	2	8.91667	0.328
0.2	0	ycaM	2	8.91667	0.384
0.2	0	yadI	2	8.91667	0.464
0.2	0	AG1	2	8.91667	0.475
0.2	0	ptsI	2	9.16667	0.549
0.2	0	clcB	2	9.16667	0.33
0.2	0	ycaM	2	9.16667	0.389
0.2	0	yadI	2	9.16667	0.462
0.2	0	AG1	2	9.16667	0.475
0.2	0	ptsI	2	9.41667	0.55
0.2	0	clcB	2	9.41667	0.336
0.2	0	ycaM	2	9.41667	0.392
0.2	0	yadI	2	9.41667	0.469
0.2	0	AG1	2	9.41667	0.488
0.2	0	ptsI	2	9.66667	0.57
0.2	0	clcB	2	9.66667	0.341
0.2	0	ycaM	2	9.66667	0.398
0.2	0	yadI	2	9.66667	0.481
0.2	0	AG1	2	9.66667	0.504
0.2	0	ptsI	2	9.91667	0.565
0.2	0	clcB	2	9.91667	0.35
0.2	0	ycaM	2	9.91667	0.404
0.2	0	yadI	2	9.91667	0.49
0.2	0	AG1	2	9.91667	0.517
0.2	0	ptsI	2	10.1667	0.567
0.2	0	clcB	2	10.1667	0.355
0.2	0	ycaM	2	10.1667	0.406
0.2	0	yadI	2	10.1667	0.496
0.2	0	AG1	2	10.1667	0.524
0.2	0	ptsI	2	10.4167	0.603
0.2	0	clcB	2	10.4167	0.358
0.2	0	ycaM	2	10.4167	0.414
0.2	0	yadI	2	10.4167	0.505
0.2	0	AG1	2	10.4167	0.537
0.2	0	ptsI	2	10.6667	0.573
0.2	0	clcB	2	10.6667	0.369
0.2	0	ycaM	2	10.6667	0.423
0.2	0	yadI	2	10.6667	0.516
0.2	0	AG1	2	10.6667	0.544
0.2	0	ptsI	2	10.9167	0.58

0.2	0	clcB	2	10.9167	0.372
0.2	0	ycaM	2	10.9167	0.43
0.2	0	yadI	2	10.9167	0.523
0.2	0	AG1	2	10.9167	0.554
0.2	0	ptsI	2	11.1667	0.586
0.2	0	clcB	2	11.1667	0.378
0.2	0	ycaM	2	11.1667	0.438
0.2	0	yadI	2	11.1667	0.533
0.2	0	AG1	2	11.1667	0.568
0.2	0	ptsI	2	11.4167	0.584
0.2	0	clcB	2	11.4167	0.379
0.2	0	ycaM	2	11.4167	0.442
0.2	0	yadI	2	11.4167	0.537
0.2	0	AG1	2	11.4167	0.568
0.2	0	ptsI	2	11.6667	0.605
0.2	0	clcB	2	11.6667	0.386
0.2	0	ycaM	2	11.6667	0.46
0.2	0	yadI	2	11.6667	0.547
0.2	0	AG1	2	11.6667	0.598
0.2	0	ptsI	2	11.9167	0.581
0.2	0	clcB	2	11.9167	0.394
0.2	0	ycaM	2	11.9167	0.484
0.2	0	yadI	2	11.9167	0.552
0.2	0	AG1	2	11.9167	0.599
0.2	0	ptsI	2	12.1667	0.6
0.2	0	clcB	2	12.1667	0.4
0.2	0	ycaM	2	12.1667	0.472
0.2	0	yadI	2	12.1667	0.56
0.2	0	AG1	2	12.1667	0.625
0.2	0	ptsI	2	12.4167	0.585
0.2	0	clcB	2	12.4167	0.409
0.2	0	ycaM	2	12.4167	0.471
0.2	0	yadI	2	12.4167	0.566
0.2	0	AG1	2	12.4167	0.643
0.2	0	ptsI	2	12.6667	0.592
0.2	0	clcB	2	12.6667	0.419
0.2	0	ycaM	2	12.6667	0.474
0.2	0	yadI	2	12.6667	0.574
0.2	0	AG1	2	12.6667	0.639
0.2	0	ptsI	2	12.9167	0.603
0.2	0	clcB	2	12.9167	0.428
0.2	0	ycaM	2	12.9167	0.48
0.2	0	yadI	2	12.9167	0.584
0.2	0	AG1	2	12.9167	0.653
0.2	0	ptsI	2	13.1667	0.61
0.2	0	clcB	2	13.1667	0.434
0.2	0	ycaM	2	13.1667	0.501
0.2	0	yadI	2	13.1667	0.591
0.2	0	AG1	2	13.1667	0.664
0.2	0	ptsI	2	13.4167	0.611
0.2	0	clcB	2	13.4167	0.443
0.2	0	ycaM	2	13.4167	0.499
0.2	0	yadI	2	13.4167	0.598

0.2	0	AG1	2	13.4167	0.678
0.2	0	ptsI	2	13.6667	0.628
0.2	0	clcB	2	13.6667	0.448
0.2	0	ycaM	2	13.6667	0.518
0.2	0	yadI	2	13.6667	0.608
0.2	0	AG1	2	13.6667	0.726
0.2	0	ptsI	2	13.9167	0.64
0.2	0	clcB	2	13.9167	0.455
0.2	0	ycaM	2	13.9167	0.526
0.2	0	yadI	2	13.9167	0.619
0.2	0	AG1	2	13.9167	0.704
0.2	0	ptsI	2	14.1667	0.685
0.2	0	clcB	2	14.1667	0.461
0.2	0	ycaM	2	14.1667	0.536
0.2	0	yadI	2	14.1667	0.628
0.2	0	AG1	2	14.1667	0.707
0.2	0	ptsI	2	14.4167	0.689
0.2	0	clcB	2	14.4167	0.465
0.2	0	ycaM	2	14.4167	0.545
0.2	0	yadI	2	14.4167	0.642
0.2	0	AG1	2	14.4167	0.698
0.2	0	ptsI	2	14.6667	0.677
0.2	0	clcB	2	14.6667	0.47
0.2	0	ycaM	2	14.6667	0.558
0.2	0	yadI	2	14.6667	0.648
0.2	0	AG1	2	14.6667	0.711
0.2	0	ptsI	2	14.9167	0.702
0.2	0	clcB	2	14.9167	0.482
0.2	0	ycaM	2	14.9167	0.563
0.2	0	yadI	2	14.9167	0.661
0.2	0	AG1	2	14.9167	0.733
0.2	0	ptsI	2	15.1667	0.671
0.2	0	clcB	2	15.1667	0.493
0.2	0	ycaM	2	15.1667	0.566
0.2	0	yadI	2	15.1667	0.667
0.2	0	AG1	2	15.1667	0.74
0.2	0	ptsI	2	15.4167	0.688
0.2	0	clcB	2	15.4167	0.495
0.2	0	ycaM	2	15.4167	0.589
0.2	0	yadI	2	15.4167	0.688
0.2	0	AG1	2	15.4167	0.784
0.2	0	ptsI	2	15.6667	0.694
0.2	0	clcB	2	15.6667	0.503
0.2	0	ycaM	2	15.6667	0.589
0.2	0	yadI	2	15.6667	0.69
0.2	0	AG1	2	15.6667	0.787
0.2	0	ptsI	2	15.9167	0.723
0.2	0	clcB	2	15.9167	0.512
0.2	0	ycaM	2	15.9167	0.596
0.2	0	yadI	2	15.9167	0.7
0.2	0	AG1	2	15.9167	0.785
0.2	0	ptsI	2	16.1667	0.72
0.2	0	clcB	2	16.1667	0.522

0.2	0	ycaM	2	16.1667	0.581
0.2	0	yadI	2	16.1667	0.717
0.2	0	AG1	2	16.1667	0.798
0.2	0	ptsI	2	16.4167	0.742
0.2	0	clcB	2	16.4167	0.534
0.2	0	ycaM	2	16.4167	0.592
0.2	0	yadI	2	16.4167	0.722
0.2	0	AG1	2	16.4167	0.809
0.2	0	ptsI	2	16.6667	0.768
0.2	0	clcB	2	16.6667	0.544
0.2	0	ycaM	2	16.6667	0.603
0.2	0	yadI	2	16.6667	0.729
0.2	0	AG1	2	16.6667	0.807
0.2	0	ptsI	2	16.9167	0.781
0.2	0	clcB	2	16.9167	0.556
0.2	0	ycaM	2	16.9167	0.608
0.2	0	yadI	2	16.9167	0.735
0.2	0	AG1	2	16.9167	0.818
0.2	0	ptsI	2	17.1667	0.786
0.2	0	clcB	2	17.1667	0.537
0.2	0	ycaM	2	17.1667	0.609
0.2	0	yadI	2	17.1667	0.746
0.2	0	AG1	2	17.1667	0.82
0.2	0	ptsI	2	17.4167	0.809
0.2	0	clcB	2	17.4167	0.56
0.2	0	ycaM	2	17.4167	0.62
0.2	0	yadI	2	17.4167	0.748
0.2	0	AG1	2	17.4167	0.82
0.2	0	ptsI	2	17.6667	0.824
0.2	0	clcB	2	17.6667	0.559
0.2	0	ycaM	2	17.6667	0.617
0.2	0	yadI	2	17.6667	0.758
0.2	0	AG1	2	17.6667	0.818
0.2	0	ptsI	2	17.9167	0.827
0.2	0	clcB	2	17.9167	0.56
0.2	0	ycaM	2	17.9167	0.622
0.2	0	yadI	2	17.9167	0.761
0.2	0	AG1	2	17.9167	0.784
0.2	0	ptsI	2	18.1667	0.842
0.2	0	clcB	2	18.1667	0.571
0.2	0	ycaM	2	18.1667	0.624
0.2	0	yadI	2	18.1667	0.768
0.2	0	AG1	2	18.1667	0.802
0.2	0	ptsI	2	18.4167	0.844
0.2	0	clcB	2	18.4167	0.566
0.2	0	ycaM	2	18.4167	0.634
0.2	0	yadI	2	18.4167	0.77
0.2	0	AG1	2	18.4167	0.802
0.2	0	ptsI	2	18.6667	0.84
0.2	0	clcB	2	18.6667	0.569
0.2	0	ycaM	2	18.6667	0.647
0.2	0	yadI	2	18.6667	0.779
0.2	0	AG1	2	18.6667	0.793

0.2	0	ptsI	2	18.9167	0.837
0.2	0	clcB	2	18.9167	0.569
0.2	0	ycaM	2	18.9167	0.658
0.2	0	yadI	2	18.9167	0.78
0.2	0	AG1	2	18.9167	0.818
0.2	0	ptsI	2	19.1667	0.856
0.2	0	clcB	2	19.1667	0.578
0.2	0	ycaM	2	19.1667	0.654
0.2	0	yadI	2	19.1667	0.784
0.2	0	AG1	2	19.1667	0.827
0.2	0	ptsI	2	19.4167	0.86
0.2	0	clcB	2	19.4167	0.582
0.2	0	ycaM	2	19.4167	0.662
0.2	0	yadI	2	19.4167	0.79
0.2	0	AG1	2	19.4167	0.826
0.2	0	ptsI	2	19.6667	0.864
0.2	0	clcB	2	19.6667	0.585
0.2	0	ycaM	2	19.6667	0.674
0.2	0	yadI	2	19.6667	0.795
0.2	0	AG1	2	19.6667	0.827
0.2	0	ptsI	2	19.9167	0.872
0.2	0	clcB	2	19.9167	0.599
0.2	0	ycaM	2	19.9167	0.683
0.2	0	yadI	2	19.9167	0.799
0.2	0	AG1	2	19.9167	0.844
0.2	0	ptsI	2	20.1667	0.874
0.2	0	clcB	2	20.1667	0.602
0.2	0	ycaM	2	20.1667	0.684
0.2	0	yadI	2	20.1667	0.805
0.2	0	AG1	2	20.1667	0.837
0.2	0	ptsI	2	20.4167	0.873
0.2	0	clcB	2	20.4167	0.612
0.2	0	ycaM	2	20.4167	0.691
0.2	0	yadI	2	20.4167	0.812
0.2	0	AG1	2	20.4167	0.84
0.2	0	ptsI	2	20.6667	0.878
0.2	0	clcB	2	20.6667	0.616
0.2	0	ycaM	2	20.6667	0.701
0.2	0	yadI	2	20.6667	0.811
0.2	0	AG1	2	20.6667	0.835
0.2	0	ptsI	2	20.9167	0.884
0.2	0	clcB	2	20.9167	0.621
0.2	0	ycaM	2	20.9167	0.704
0.2	0	yadI	2	20.9167	0.814
0.2	0	AG1	2	20.9167	0.843
0.2	0	ptsI	2	21.1667	0.89
0.2	0	clcB	2	21.1667	0.622
0.2	0	ycaM	2	21.1667	0.705
0.2	0	yadI	2	21.1667	0.82
0.2	0	AG1	2	21.1667	0.84
0.2	0	ptsI	2	21.4167	0.874
0.2	0	clcB	2	21.4167	0.626
0.2	0	ycaM	2	21.4167	0.704

0.2	0	yadI	2	21.4167	0.821
0.2	0	AG1	2	21.4167	0.84
0.2	0	ptsI	2	21.6667	0.902
0.2	0	clcB	2	21.6667	0.636
0.2	0	ycaM	2	21.6667	0.717
0.2	0	yadI	2	21.6667	0.825
0.2	0	AG1	2	21.6667	0.839
0.2	0	ptsI	2	21.9167	0.91
0.2	0	clcB	2	21.9167	0.645
0.2	0	ycaM	2	21.9167	0.724
0.2	0	yadI	2	21.9167	0.83
0.2	0	AG1	2	21.9167	0.844
0.2	0	ptsI	2	22.1667	0.909
0.2	0	clcB	2	22.1667	0.649
0.2	0	ycaM	2	22.1667	0.726
0.2	0	yadI	2	22.1667	0.835
0.2	0	AG1	2	22.1667	0.844
0.2	0	ptsI	2	22.4167	0.916
0.2	0	clcB	2	22.4167	0.655
0.2	0	ycaM	2	22.4167	0.731
0.2	0	yadI	2	22.4167	0.836
0.2	0	AG1	2	22.4167	0.843
0.2	0	ptsI	2	22.6667	0.92
0.2	0	clcB	2	22.6667	0.664
0.2	0	ycaM	2	22.6667	0.732
0.2	0	yadI	2	22.6667	0.84
0.2	0	AG1	2	22.6667	0.849
0.2	0	ptsI	2	22.9167	0.93
0.2	0	clcB	2	22.9167	0.67
0.2	0	ycaM	2	22.9167	0.735
0.2	0	yadI	2	22.9167	0.843
0.2	0	AG1	2	22.9167	0.85
0.2	0	ptsI	2	23.1667	0.936
0.2	0	clcB	2	23.1667	0.673
0.2	0	ycaM	2	23.1667	0.739
0.2	0	yadI	2	23.1667	0.845
0.2	0	AG1	2	23.1667	0.851
0.2	0	ptsI	2	23.4167	0.939
0.2	0	clcB	2	23.4167	0.679
0.2	0	ycaM	2	23.4167	0.741
0.2	0	yadI	2	23.4167	0.85
0.2	0	AG1	2	23.4167	0.847
0.2	0	ptsI	2	23.6667	0.941
0.2	0	clcB	2	23.6667	0.686
0.2	0	ycaM	2	23.6667	0.755
0.2	0	yadI	2	23.6667	0.852
0.2	0	AG1	2	23.6667	0.852
0.2	0	ptsI	2	23.9167	0.955
0.2	0	clcB	2	23.9167	0.694
0.2	0	ycaM	2	23.9167	0.755
0.2	0	yadI	2	23.9167	0.851
0.2	0	AG1	2	23.9167	0.853
0.2	0	ptsI	2	24.1667	0.951

0.2	0	clcB	2	24.1667	0.696
0.2	0	ycaM	2	24.1667	0.758
0.2	0	yadI	2	24.1667	0.854
0.2	0	AG1	2	24.1667	0.846
0.2	0	ptsI	2	24.4167	0.963
0.2	0	clcB	2	24.4167	0.699
0.2	0	ycaM	2	24.4167	0.762
0.2	0	yadI	2	24.4167	0.858
0.2	0	AG1	2	24.4167	0.846
0.2	0	ptsI	2	24.6667	0.975
0.2	0	clcB	2	24.6667	0.706
0.2	0	ycaM	2	24.6667	0.765
0.2	0	yadI	2	24.6667	0.863
0.2	0	AG1	2	24.6667	0.852
0.2	0	ptsI	2	24.9167	0.977
0.2	0	clcB	2	24.9167	0.71
0.2	0	ycaM	2	24.9167	0.771
0.2	0	yadI	2	24.9167	0.864
0.2	0	AG1	2	24.9167	0.852
0.2	0	ptsI	2	25.1667	0.972
0.2	0	clcB	2	25.1667	0.716
0.2	0	ycaM	2	25.1667	0.774
0.2	0	yadI	2	25.1667	0.866
0.2	0	AG1	2	25.1667	0.848
0.2	0	ptsI	2	25.4167	0.983
0.2	0	clcB	2	25.4167	0.723
0.2	0	ycaM	2	25.4167	0.784
0.2	0	yadI	2	25.4167	0.868
0.2	0	AG1	2	25.4167	0.855
0.2	0	ptsI	2	25.6667	0.982
0.2	0	clcB	2	25.6667	0.724
0.2	0	ycaM	2	25.6667	0.783
0.2	0	yadI	2	25.6667	0.87
0.2	0	AG1	2	25.6667	0.848
0.2	0	ptsI	2	25.9167	0.988
0.2	0	clcB	2	25.9167	0.729
0.2	0	ycaM	2	25.9167	0.787
0.2	0	yadI	2	25.9167	0.873
0.2	0	AG1	2	25.9167	0.852
0.2	0	ptsI	2	26.1667	0.977
0.2	0	clcB	2	26.1667	0.727
0.2	0	ycaM	2	26.1667	0.793
0.2	0	yadI	2	26.1667	0.879
0.2	0	AG1	2	26.1667	0.849
0.2	0	ptsI	2	26.4167	1.003
0.2	0	clcB	2	26.4167	0.741
0.2	0	ycaM	2	26.4167	0.799
0.2	0	yadI	2	26.4167	0.877
0.2	0	AG1	2	26.4167	0.856
0.2	0	ptsI	3	0	0.154
0.2	0	clcB	3	0	0.163
0.2	0	ycaM	3	0	0.152
0.2	0	yadI	3	0	0.17

0.2	0	AG1	3	0	0.166
0.2	0	ptsI	3	0.25	0.153
0.2	0	clcB	3	0.25	0.214
0.2	0	ycaM	3	0.25	0.15
0.2	0	yadI	3	0.25	0.167
0.2	0	AG1	3	0.25	0.165
0.2	0	ptsI	3	0.5	0.151
0.2	0	clcB	3	0.5	0.154
0.2	0	ycaM	3	0.5	0.147
0.2	0	yadI	3	0.5	0.166
0.2	0	AG1	3	0.5	0.164
0.2	0	ptsI	3	0.75	0.151
0.2	0	clcB	3	0.75	0.156
0.2	0	ycaM	3	0.75	0.147
0.2	0	yadI	3	0.75	0.168
0.2	0	AG1	3	0.75	0.164
0.2	0	ptsI	3	1	0.152
0.2	0	clcB	3	1	0.162
0.2	0	ycaM	3	1	0.149
0.2	0	yadI	3	1	0.168
0.2	0	AG1	3	1	0.165
0.2	0	ptsI	3	1.25	0.152
0.2	0	clcB	3	1.25	0.154
0.2	0	ycaM	3	1.25	0.148
0.2	0	yadI	3	1.25	0.17
0.2	0	AG1	3	1.25	0.165
0.2	0	ptsI	3	1.5	0.153
0.2	0	clcB	3	1.5	0.154
0.2	0	ycaM	3	1.5	0.15
0.2	0	yadI	3	1.5	0.173
0.2	0	AG1	3	1.5	0.167
0.2	0	ptsI	3	1.75	0.153
0.2	0	clcB	3	1.75	0.154
0.2	0	ycaM	3	1.75	0.15
0.2	0	yadI	3	1.75	0.176
0.2	0	AG1	3	1.75	0.167
0.2	0	ptsI	3	2	0.155
0.2	0	clcB	3	2	0.156
0.2	0	ycaM	3	2	0.174
0.2	0	yadI	3	2	0.177
0.2	0	AG1	3	2	0.169
0.2	0	ptsI	3	2.25	0.156
0.2	0	clcB	3	2.25	0.157
0.2	0	ycaM	3	2.25	0.154
0.2	0	yadI	3	2.25	0.18
0.2	0	AG1	3	2.25	0.173
0.2	0	ptsI	3	2.5	0.158
0.2	0	clcB	3	2.5	0.159
0.2	0	ycaM	3	2.5	0.154
0.2	0	yadI	3	2.5	0.183
0.2	0	AG1	3	2.5	0.175
0.2	0	ptsI	3	2.75	0.16
0.2	0	clcB	3	2.75	0.16

0.2	0	ycaM	3	2.75	0.158
0.2	0	yadI	3	2.75	0.185
0.2	0	AG1	3	2.75	0.182
0.2	0	ptsI	3	3	0.162
0.2	0	clcB	3	3	0.163
0.2	0	ycaM	3	3	0.157
0.2	0	yadI	3	3	0.192
0.2	0	AG1	3	3	0.183
0.2	0	ptsI	3	3.25	0.164
0.2	0	clcB	3	3.25	0.162
0.2	0	ycaM	3	3.25	0.16
0.2	0	yadI	3	3.25	0.197
0.2	0	AG1	3	3.25	0.183
0.2	0	ptsI	3	3.5	0.168
0.2	0	clcB	3	3.5	0.164
0.2	0	ycaM	3	3.5	0.165
0.2	0	yadI	3	3.5	0.202
0.2	0	AG1	3	3.5	0.188
0.2	0	ptsI	3	3.75	0.172
0.2	0	clcB	3	3.75	0.167
0.2	0	ycaM	3	3.75	0.169
0.2	0	yadI	3	3.75	0.208
0.2	0	AG1	3	3.75	0.192
0.2	0	ptsI	3	4	0.176
0.2	0	clcB	3	4	0.171
0.2	0	ycaM	3	4	0.171
0.2	0	yadI	3	4	0.22
0.2	0	AG1	3	4	0.201
0.2	0	ptsI	3	4.41667	0.186
0.2	0	clcB	3	4.41667	0.203
0.2	0	ycaM	3	4.41667	0.192
0.2	0	yadI	3	4.41667	0.218
0.2	0	AG1	3	4.41667	0.228
0.2	0	ptsI	3	4.66667	0.183
0.2	0	clcB	3	4.66667	0.181
0.2	0	ycaM	3	4.66667	0.189
0.2	0	yadI	3	4.66667	0.212
0.2	0	AG1	3	4.66667	0.223
0.2	0	ptsI	3	4.91667	0.183
0.2	0	clcB	3	4.91667	0.188
0.2	0	ycaM	3	4.91667	0.189
0.2	0	yadI	3	4.91667	0.222
0.2	0	AG1	3	4.91667	0.226
0.2	0	ptsI	3	5.16667	0.188
0.2	0	clcB	3	5.16667	0.192
0.2	0	ycaM	3	5.16667	0.192
0.2	0	yadI	3	5.16667	0.234
0.2	0	AG1	3	5.16667	0.232
0.2	0	ptsI	3	5.41667	0.193
0.2	0	clcB	3	5.41667	0.199
0.2	0	ycaM	3	5.41667	0.199
0.2	0	yadI	3	5.41667	0.249
0.2	0	AG1	3	5.41667	0.242

0.2	0	ptsI	3	5.66667	0.199
0.2	0	clcB	3	5.66667	0.208
0.2	0	ycaM	3	5.66667	0.204
0.2	0	yadI	3	5.66667	0.265
0.2	0	AG1	3	5.66667	0.251
0.2	0	ptsI	3	5.91667	0.206
0.2	0	clcB	3	5.91667	0.216
0.2	0	ycaM	3	5.91667	0.212
0.2	0	yadI	3	5.91667	0.281
0.2	0	AG1	3	5.91667	0.265
0.2	0	ptsI	3	6.16667	0.214
0.2	0	clcB	3	6.16667	0.23
0.2	0	ycaM	3	6.16667	0.222
0.2	0	yadI	3	6.16667	0.302
0.2	0	AG1	3	6.16667	0.278
0.2	0	ptsI	3	6.41667	0.222
0.2	0	clcB	3	6.41667	0.247
0.2	0	ycaM	3	6.41667	0.232
0.2	0	yadI	3	6.41667	0.314
0.2	0	AG1	3	6.41667	0.294
0.2	0	ptsI	3	6.66667	0.232
0.2	0	clcB	3	6.66667	0.265
0.2	0	ycaM	3	6.66667	0.244
0.2	0	yadI	3	6.66667	0.327
0.2	0	AG1	3	6.66667	0.308
0.2	0	ptsI	3	6.91667	0.24
0.2	0	clcB	3	6.91667	0.276
0.2	0	ycaM	3	6.91667	0.258
0.2	0	yadI	3	6.91667	0.341
0.2	0	AG1	3	6.91667	0.324
0.2	0	ptsI	3	7.16667	0.252
0.2	0	clcB	3	7.16667	0.291
0.2	0	ycaM	3	7.16667	0.272
0.2	0	yadI	3	7.16667	0.358
0.2	0	AG1	3	7.16667	0.332
0.2	0	ptsI	3	7.41667	0.265
0.2	0	clcB	3	7.41667	0.299
0.2	0	ycaM	3	7.41667	0.28
0.2	0	yadI	3	7.41667	0.37
0.2	0	AG1	3	7.41667	0.344
0.2	0	ptsI	3	7.66667	0.274
0.2	0	clcB	3	7.66667	0.309
0.2	0	ycaM	3	7.66667	0.288
0.2	0	yadI	3	7.66667	0.381
0.2	0	AG1	3	7.66667	0.351
0.2	0	ptsI	3	7.91667	0.28
0.2	0	clcB	3	7.91667	0.316
0.2	0	ycaM	3	7.91667	0.295
0.2	0	yadI	3	7.91667	0.39
0.2	0	AG1	3	7.91667	0.364
0.2	0	ptsI	3	8.16667	0.29
0.2	0	clcB	3	8.16667	0.326
0.2	0	ycaM	3	8.16667	0.302

0.2	0	yadI	3	8.16667	0.401
0.2	0	AG1	3	8.16667	0.371
0.2	0	ptsI	3	8.41667	0.298
0.2	0	clcB	3	8.41667	0.335
0.2	0	ycaM	3	8.41667	0.313
0.2	0	yadI	3	8.41667	0.412
0.2	0	AG1	3	8.41667	0.381
0.2	0	ptsI	3	9.05	0.282
0.2	0	clcB	3	9.05	0.287
0.2	0	ycaM	3	9.05	0.291
0.2	0	yadI	3	9.05	0.37
0.2	0	AG1	3	9.05	0.432
0.2	0	ptsI	3	9.3	0.287
0.2	0	clcB	3	9.3	0.292
0.2	0	ycaM	3	9.3	0.297
0.2	0	yadI	3	9.3	0.373
0.2	0	AG1	3	9.3	0.427
0.2	0	ptsI	3	9.55	0.293
0.2	0	clcB	3	9.55	0.297
0.2	0	ycaM	3	9.55	0.303
0.2	0	yadI	3	9.55	0.382
0.2	0	AG1	3	9.55	0.438
0.2	0	ptsI	3	9.8	0.301
0.2	0	clcB	3	9.8	0.303
0.2	0	ycaM	3	9.8	0.311
0.2	0	yadI	3	9.8	0.392
0.2	0	AG1	3	9.8	0.45
0.2	0	ptsI	3	10.05	0.307
0.2	0	clcB	3	10.05	0.308
0.2	0	ycaM	3	10.05	0.316
0.2	0	yadI	3	10.05	0.4
0.2	0	AG1	3	10.05	0.46
0.2	0	ptsI	3	10.3	0.317
0.2	0	clcB	3	10.3	0.316
0.2	0	ycaM	3	10.3	0.325
0.2	0	yadI	3	10.3	0.41
0.2	0	AG1	3	10.3	0.472
0.2	0	ptsI	3	10.55	0.325
0.2	0	clcB	3	10.55	0.324
0.2	0	ycaM	3	10.55	0.335
0.2	0	yadI	3	10.55	0.422
0.2	0	AG1	3	10.55	0.48
0.2	0	ptsI	3	10.8	0.335
0.2	0	clcB	3	10.8	0.334
0.2	0	ycaM	3	10.8	0.342
0.2	0	yadI	3	10.8	0.434
0.2	0	AG1	3	10.8	0.492
0.2	0	ptsI	3	11.05	0.343
0.2	0	clcB	3	11.05	0.342
0.2	0	ycaM	3	11.05	0.365
0.2	0	yadI	3	11.05	0.444
0.2	0	AG1	3	11.05	0.501
0.2	0	ptsI	3	11.3	0.353

0.2	0	clcB	3	11.3	0.349
0.2	0	ycaM	3	11.3	0.357
0.2	0	yadI	3	11.3	0.454
0.2	0	AG1	3	11.3	0.514
0.2	0	ptsI	3	11.55	0.359
0.2	0	clcB	3	11.55	0.353
0.2	0	ycaM	3	11.55	0.361
0.2	0	yadI	3	11.55	0.465
0.2	0	AG1	3	11.55	0.525
0.2	0	ptsI	3	11.8	0.368
0.2	0	clcB	3	11.8	0.358
0.2	0	ycaM	3	11.8	0.363
0.2	0	yadI	3	11.8	0.476
0.2	0	AG1	3	11.8	0.538
0.2	0	ptsI	3	12.05	0.378
0.2	0	clcB	3	12.05	0.361
0.2	0	ycaM	3	12.05	0.366
0.2	0	yadI	3	12.05	0.484
0.2	0	AG1	3	12.05	0.549
0.2	0	ptsI	3	12.3	0.387
0.2	0	clcB	3	12.3	0.368
0.2	0	ycaM	3	12.3	0.374
0.2	0	yadI	3	12.3	0.5
0.2	0	AG1	3	12.3	0.57
0.2	0	ptsI	3	12.55	0.394
0.2	0	clcB	3	12.55	0.374
0.2	0	ycaM	3	12.55	0.373
0.2	0	yadI	3	12.55	0.509
0.2	0	AG1	3	12.55	0.585
0.2	0	ptsI	3	12.8	0.403
0.2	0	clcB	3	12.8	0.381
0.2	0	ycaM	3	12.8	0.381
0.2	0	yadI	3	12.8	0.515
0.2	0	AG1	3	12.8	0.606
0.2	0	ptsI	3	13.05	0.415
0.2	0	clcB	3	13.05	0.387
0.2	0	ycaM	3	13.05	0.386
0.2	0	yadI	3	13.05	0.524
0.2	0	AG1	3	13.05	0.621
0.2	0	ptsI	3	13.3	0.421
0.2	0	clcB	3	13.3	0.396
0.2	0	ycaM	3	13.3	0.391
0.2	0	yadI	3	13.3	0.535
0.2	0	AG1	3	13.3	0.635
0.2	0	ptsI	3	13.55	0.431
0.2	0	clcB	3	13.55	0.404
0.2	0	ycaM	3	13.55	0.399
0.2	0	yadI	3	13.55	0.548
0.2	0	AG1	3	13.55	0.65
0.2	0	ptsI	3	13.8	0.438
0.2	0	clcB	3	13.8	0.41
0.2	0	ycaM	3	13.8	0.407
0.2	0	yadI	3	13.8	0.556

0.2	0	AG1	3	13.8	0.661
0.2	0	ptsI	3	14.05	0.45
0.2	0	clcB	3	14.05	0.421
0.2	0	ycaM	3	14.05	0.413
0.2	0	yadI	3	14.05	0.562
0.2	0	AG1	3	14.05	0.672
0.2	0	ptsI	3	14.3	0.46
0.2	0	clcB	3	14.3	0.43
0.2	0	ycaM	3	14.3	0.419
0.2	0	yadI	3	14.3	0.57
0.2	0	AG1	3	14.3	0.681
0.2	0	ptsI	3	14.55	0.473
0.2	0	clcB	3	14.55	0.434
0.2	0	ycaM	3	14.55	0.423
0.2	0	yadI	3	14.55	0.576
0.2	0	AG1	3	14.55	0.681
0.2	0	ptsI	3	14.8	0.484
0.2	0	clcB	3	14.8	0.439
0.2	0	ycaM	3	14.8	0.433
0.2	0	yadI	3	14.8	0.583
0.2	0	AG1	3	14.8	0.692
0.2	0	ptsI	3	15.05	0.49
0.2	0	clcB	3	15.05	0.446
0.2	0	ycaM	3	15.05	0.439
0.2	0	yadI	3	15.05	0.589
0.2	0	AG1	3	15.05	0.699
0.2	0	ptsI	3	15.3	0.504
0.2	0	clcB	3	15.3	0.452
0.2	0	ycaM	3	15.3	0.443
0.2	0	yadI	3	15.3	0.602
0.2	0	AG1	3	15.3	0.709
0.2	0	ptsI	3	15.55	0.505
0.2	0	clcB	3	15.55	0.455
0.2	0	ycaM	3	15.55	0.446
0.2	0	yadI	3	15.55	0.604
0.2	0	AG1	3	15.55	0.711
0.2	0	ptsI	3	15.8	0.515
0.2	0	clcB	3	15.8	0.46
0.2	0	ycaM	3	15.8	0.451
0.2	0	yadI	3	15.8	0.614
0.2	0	AG1	3	15.8	0.722
0.2	0	ptsI	3	16.05	0.521
0.2	0	clcB	3	16.05	0.467
0.2	0	ycaM	3	16.05	0.458
0.2	0	yadI	3	16.05	0.618
0.2	0	AG1	3	16.05	0.731
0.2	0	ptsI	3	16.3	0.528
0.2	0	clcB	3	16.3	0.468
0.2	0	ycaM	3	16.3	0.458
0.2	0	yadI	3	16.3	0.623
0.2	0	AG1	3	16.3	0.744
0.2	0	ptsI	3	16.55	0.535
0.2	0	clcB	3	16.55	0.471

0.2	0	ycaM	3	16.55	0.465
0.2	0	yadI	3	16.55	0.629
0.2	0	AG1	3	16.55	0.752
0.2	0	ptsI	3	16.8	0.538
0.2	0	clcB	3	16.8	0.478
0.2	0	ycaM	3	16.8	0.474
0.2	0	yadI	3	16.8	0.633
0.2	0	AG1	3	16.8	0.757
0.2	0	ptsI	3	17.05	0.545
0.2	0	clcB	3	17.05	0.484
0.2	0	ycaM	3	17.05	0.472
0.2	0	yadI	3	17.05	0.64
0.2	0	AG1	3	17.05	0.776
0.2	0	ptsI	3	17.3	0.551
0.2	0	clcB	3	17.3	0.486
0.2	0	ycaM	3	17.3	0.48
0.2	0	yadI	3	17.3	0.649
0.2	0	AG1	3	17.3	0.793
0.2	0	ptsI	3	17.55	0.563
0.2	0	clcB	3	17.55	0.491
0.2	0	ycaM	3	17.55	0.48
0.2	0	yadI	3	17.55	0.654
0.2	0	AG1	3	17.55	0.806
0.2	0	ptsI	3	17.8	0.561
0.2	0	clcB	3	17.8	0.495
0.2	0	ycaM	3	17.8	0.488
0.2	0	yadI	3	17.8	0.66
0.2	0	AG1	3	17.8	0.798
0.2	0	ptsI	3	18.05	0.572
0.2	0	clcB	3	18.05	0.5
0.2	0	ycaM	3	18.05	0.492
0.2	0	yadI	3	18.05	0.664
0.2	0	AG1	3	18.05	0.806
0.2	0	ptsI	3	18.3	0.579
0.2	0	clcB	3	18.3	0.505
0.2	0	ycaM	3	18.3	0.497
0.2	0	yadI	3	18.3	0.674
0.2	0	AG1	3	18.3	0.806
0.2	0	ptsI	3	18.55	0.584
0.2	0	clcB	3	18.55	0.51
0.2	0	ycaM	3	18.55	0.502
0.2	0	yadI	3	18.55	0.68
0.2	0	AG1	3	18.55	0.811
0.2	0	ptsI	3	18.8	0.593
0.2	0	clcB	3	18.8	0.51
0.2	0	ycaM	3	18.8	0.504
0.2	0	yadI	3	18.8	0.68
0.2	0	AG1	3	18.8	0.814
0.2	0	ptsI	3	19.05	0.602
0.2	0	clcB	3	19.05	0.512
0.2	0	ycaM	3	19.05	0.51
0.2	0	yadI	3	19.05	0.686
0.2	0	AG1	3	19.05	0.826

0.2	0	ptsI	3	19.3	0.611
0.2	0	clcB	3	19.3	0.521
0.2	0	ycaM	3	19.3	0.514
0.2	0	yadI	3	19.3	0.69
0.2	0	AG1	3	19.3	0.833
0.2	0	ptsI	3	19.55	0.618
0.2	0	clcB	3	19.55	0.529
0.2	0	ycaM	3	19.55	0.518
0.2	0	yadI	3	19.55	0.693
0.2	0	AG1	3	19.55	0.851
0.2	0	ptsI	3	19.8	0.624
0.2	0	clcB	3	19.8	0.538
0.2	0	ycaM	3	19.8	0.521
0.2	0	yadI	3	19.8	0.698
0.2	0	AG1	3	19.8	0.856
0.2	0	ptsI	3	20.05	0.622
0.2	0	clcB	3	20.05	0.556
0.2	0	ycaM	3	20.05	0.523
0.2	0	yadI	3	20.05	0.704
0.2	0	AG1	3	20.05	0.838
0.2	0	ptsI	3	20.3	0.624
0.2	0	clcB	3	20.3	0.56
0.2	0	ycaM	3	20.3	0.528
0.2	0	yadI	3	20.3	0.707
0.2	0	AG1	3	20.3	0.819
0.2	0	ptsI	3	20.55	0.634
0.2	0	clcB	3	20.55	0.569
0.2	0	ycaM	3	20.55	0.535
0.2	0	yadI	3	20.55	0.709
0.2	0	AG1	3	20.55	0.815
0.2	0	ptsI	3	20.8	0.644
0.2	0	clcB	3	20.8	0.571
0.2	0	ycaM	3	20.8	0.535
0.2	0	yadI	3	20.8	0.718
0.2	0	AG1	3	20.8	0.823
0.2	0	ptsI	3	21.05	0.661
0.2	0	clcB	3	21.05	0.574
0.2	0	ycaM	3	21.05	0.537
0.2	0	yadI	3	21.05	0.72
0.2	0	AG1	3	21.05	0.824
0.2	0	ptsI	3	21.3	0.678
0.2	0	clcB	3	21.3	0.578
0.2	0	ycaM	3	21.3	0.539
0.2	0	yadI	3	21.3	0.722
0.2	0	AG1	3	21.3	0.829
0.2	0	ptsI	3	21.55	0.692
0.2	0	clcB	3	21.55	0.58
0.2	0	ycaM	3	21.55	0.547
0.2	0	yadI	3	21.55	0.722
0.2	0	AG1	3	21.55	0.83
0.2	0	ptsI	3	21.8	0.697
0.2	0	clcB	3	21.8	0.584
0.2	0	ycaM	3	21.8	0.552

0.2	0	yadI	3	21.8	0.727
0.2	0	AG1	3	21.8	0.832
0.2	0	ptsI	3	22.05	0.698
0.2	0	clcB	3	22.05	0.589
0.2	0	ycaM	3	22.05	0.555
0.2	0	yadI	3	22.05	0.732
0.2	0	AG1	3	22.05	0.832
0.2	0	ptsI	3	22.3	0.707
0.2	0	clcB	3	22.3	0.594
0.2	0	ycaM	3	22.3	0.561
0.2	0	yadI	3	22.3	0.734
0.2	0	AG1	3	22.3	0.834
0.2	0	ptsI	3	22.55	0.718
0.2	0	clcB	3	22.55	0.594
0.2	0	ycaM	3	22.55	0.585
0.2	0	yadI	3	22.55	0.737
0.2	0	AG1	3	22.55	0.834
0.2	0	ptsI	3	22.8	0.726
0.2	0	clcB	3	22.8	0.594
0.2	0	ycaM	3	22.8	0.565
0.2	0	yadI	3	22.8	0.74
0.2	0	AG1	3	22.8	0.839
0.2	0	ptsI	3	23.05	0.736
0.2	0	clcB	3	23.05	0.594
0.2	0	ycaM	3	23.05	0.568
0.2	0	yadI	3	23.05	0.743
0.2	0	AG1	3	23.05	0.835
0.2	0	ptsI	3	23.3	0.75
0.2	0	clcB	3	23.3	0.597
0.2	0	ycaM	3	23.3	0.57
0.2	0	yadI	3	23.3	0.746
0.2	0	AG1	3	23.3	0.838
0.2	0	ptsI	3	23.55	0.764
0.2	0	clcB	3	23.55	0.598
0.2	0	ycaM	3	23.55	0.573
0.2	0	yadI	3	23.55	0.748
0.2	0	AG1	3	23.55	0.839
0.2	0	ptsI	3	23.8	0.769
0.2	0	clcB	3	23.8	0.598
0.2	0	ycaM	3	23.8	0.575
0.2	0	yadI	3	23.8	0.748
0.2	0	AG1	3	23.8	0.841
0.2	0	ptsI	3	24.05	0.782
0.2	0	clcB	3	24.05	0.599
0.2	0	ycaM	3	24.05	0.574
0.2	0	yadI	3	24.05	0.754
0.2	0	AG1	3	24.05	0.842
0.2	0	ptsI	3	24.3	0.786
0.2	0	clcB	3	24.3	0.605
0.2	0	ycaM	3	24.3	0.576
0.2	0	yadI	3	24.3	0.756
0.2	0	AG1	3	24.3	0.84
0.2	0	ptsI	4	0	0.183

0.2	0	clcB	4	0	0.202
0.2	0	ycaM	4	0	0.197
0.2	0	yadI	4	0	0.232
0.2	0	AG1	4	0	0.204
0.2	0	ptsI	4	0.25	0.182
0.2	0	clcB	4	0.25	0.199
0.2	0	ycaM	4	0.25	0.191
0.2	0	yadI	4	0.25	0.221
0.2	0	AG1	4	0.25	0.202
0.2	0	ptsI	4	0.5	0.183
0.2	0	clcB	4	0.5	0.199
0.2	0	ycaM	4	0.5	0.191
0.2	0	yadI	4	0.5	0.221
0.2	0	AG1	4	0.5	0.203
0.2	0	ptsI	4	0.75	0.186
0.2	0	clcB	4	0.75	0.203
0.2	0	ycaM	4	0.75	0.194
0.2	0	yadI	4	0.75	0.224
0.2	0	AG1	4	0.75	0.206
0.2	0	ptsI	4	1	0.191
0.2	0	clcB	4	1	0.206
0.2	0	ycaM	4	1	0.198
0.2	0	yadI	4	1	0.228
0.2	0	AG1	4	1	0.212
0.2	0	ptsI	4	1.25	0.198
0.2	0	clcB	4	1.25	0.212
0.2	0	ycaM	4	1.25	0.203
0.2	0	yadI	4	1.25	0.232
0.2	0	AG1	4	1.25	0.221
0.2	0	ptsI	4	1.5	0.2
0.2	0	clcB	4	1.5	0.212
0.2	0	ycaM	4	1.5	0.207
0.2	0	yadI	4	1.5	0.243
0.2	0	AG1	4	1.5	0.217
0.2	0	ptsI	4	1.75	0.21
0.2	0	clcB	4	1.75	0.222
0.2	0	ycaM	4	1.75	0.216
0.2	0	yadI	4	1.75	0.255
0.2	0	AG1	4	1.75	0.23
0.2	0	ptsI	4	2	0.22
0.2	0	clcB	4	2	0.233
0.2	0	ycaM	4	2	0.226
0.2	0	yadI	4	2	0.269
0.2	0	AG1	4	2	0.247
0.2	0	ptsI	4	2.25	0.232
0.2	0	clcB	4	2.25	0.246
0.2	0	ycaM	4	2.25	0.237
0.2	0	yadI	4	2.25	0.284
0.2	0	AG1	4	2.25	0.269
0.2	0	ptsI	4	2.5	0.235
0.2	0	clcB	4	2.5	0.249
0.2	0	ycaM	4	2.5	0.249
0.2	0	yadI	4	2.5	0.305

0.2	0	AG1	4	2.5	0.26
0.2	0	ptsI	4	2.75	0.248
0.2	0	clcB	4	2.75	0.268
0.2	0	ycaM	4	2.75	0.263
0.2	0	yadI	4	2.75	0.322
0.2	0	AG1	4	2.75	0.274
0.2	0	ptsI	4	3	0.266
0.2	0	clcB	4	3	0.277
0.2	0	ycaM	4	3	0.273
0.2	0	yadI	4	3	0.342
0.2	0	AG1	4	3	0.294
0.2	0	ptsI	4	3.25	0.279
0.2	0	clcB	4	3.25	0.288
0.2	0	ycaM	4	3.25	0.289
0.2	0	yadI	4	3.25	0.363
0.2	0	AG1	4	3.25	0.312
0.2	0	ptsI	4	3.5	0.296
0.2	0	clcB	4	3.5	0.309
0.2	0	ycaM	4	3.5	0.306
0.2	0	yadI	4	3.5	0.385
0.2	0	AG1	4	3.5	0.338
0.2	0	ptsI	4	3.75	0.313
0.2	0	clcB	4	3.75	0.33
0.2	0	ycaM	4	3.75	0.323
0.2	0	yadI	4	3.75	0.406
0.2	0	AG1	4	3.75	0.353
0.2	0	ptsI	4	4	0.329
0.2	0	clcB	4	4	0.342
0.2	0	ycaM	4	4	0.335
0.2	0	yadI	4	4	0.439
0.2	0	AG1	4	4	0.38
0.2	0	ptsI	4	4.35	0.348
0.2	0	clcB	4	4.35	0.324
0.2	0	ycaM	4	4.35	0.388
0.2	0	yadI	4	4.35	0.394
0.2	0	AG1	4	4.35	0.326
0.2	0	ptsI	4	4.6	0.361
0.2	0	clcB	4	4.6	0.306
0.2	0	ycaM	4	4.6	0.36
0.2	0	yadI	4	4.6	0.375
0.2	0	AG1	4	4.6	0.347
0.2	0	ptsI	4	4.85	0.373
0.2	0	clcB	4	4.85	0.314
0.2	0	ycaM	4	4.85	0.37
0.2	0	yadI	4	4.85	0.385
0.2	0	AG1	4	4.85	0.361
0.2	0	ptsI	4	5.1	0.388
0.2	0	clcB	4	5.1	0.326
0.2	0	ycaM	4	5.1	0.386
0.2	0	yadI	4	5.1	0.398
0.2	0	AG1	4	5.1	0.376
0.2	0	ptsI	4	5.35	0.401
0.2	0	clcB	4	5.35	0.337

0.2	0	ycaM	4	5.35	0.403
0.2	0	yadI	4	5.35	0.413
0.2	0	AG1	4	5.35	0.392
0.2	0	ptsI	4	5.6	0.417
0.2	0	clcB	4	5.6	0.342
0.2	0	ycaM	4	5.6	0.417
0.2	0	yadI	4	5.6	0.428
0.2	0	AG1	4	5.6	0.405
0.2	0	ptsI	4	5.85	0.428
0.2	0	clcB	4	5.85	0.35
0.2	0	ycaM	4	5.85	0.427
0.2	0	yadI	4	5.85	0.441
0.2	0	AG1	4	5.85	0.424
0.2	0	ptsI	4	6.1	0.437
0.2	0	clcB	4	6.1	0.366
0.2	0	ycaM	4	6.1	0.442
0.2	0	yadI	4	6.1	0.456
0.2	0	AG1	4	6.1	0.442
0.2	0	ptsI	4	6.35	0.454
0.2	0	clcB	4	6.35	0.374
0.2	0	ycaM	4	6.35	0.458
0.2	0	yadI	4	6.35	0.47
0.2	0	AG1	4	6.35	0.453
0.2	0	ptsI	4	6.6	0.461
0.2	0	clcB	4	6.6	0.384
0.2	0	ycaM	4	6.6	0.463
0.2	0	yadI	4	6.6	0.484
0.2	0	AG1	4	6.6	0.468
0.2	0	ptsI	4	6.85	0.475
0.2	0	clcB	4	6.85	0.392
0.2	0	ycaM	4	6.85	0.474
0.2	0	yadI	4	6.85	0.496
0.2	0	AG1	4	6.85	0.48
0.2	0	ptsI	4	7.1	0.485
0.2	0	clcB	4	7.1	0.403
0.2	0	ycaM	4	7.1	0.483
0.2	0	yadI	4	7.1	0.506
0.2	0	AG1	4	7.1	0.495
0.2	0	ptsI	4	7.35	0.495
0.2	0	clcB	4	7.35	0.411
0.2	0	ycaM	4	7.35	0.493
0.2	0	yadI	4	7.35	0.514
0.2	0	AG1	4	7.35	0.503
0.2	0	ptsI	4	7.6	0.506
0.2	0	clcB	4	7.6	0.421
0.2	0	ycaM	4	7.6	0.5
0.2	0	yadI	4	7.6	0.523
0.2	0	AG1	4	7.6	0.515
0.2	0	ptsI	4	7.85	0.516
0.2	0	clcB	4	7.85	0.427
0.2	0	ycaM	4	7.85	0.501
0.2	0	yadI	4	7.85	0.531
0.2	0	AG1	4	7.85	0.52

0.2	0	ptsI	4	8.1	0.525
0.2	0	clcB	4	8.1	0.438
0.2	0	ycaM	4	8.1	0.505
0.2	0	yadI	4	8.1	0.543
0.2	0	AG1	4	8.1	0.532
0.2	0	ptsI	4	8.35	0.534
0.2	0	clcB	4	8.35	0.444
0.2	0	ycaM	4	8.35	0.511
0.2	0	yadI	4	8.35	0.552
0.2	0	AG1	4	8.35	0.54
0.2	0	ptsI	4	8.83333	0.383
0.2	0	clcB	4	8.83333	0.426
0.2	0	ycaM	4	8.83333	0.389
0.2	0	yadI	4	8.83333	0.514
0.2	0	AG1	4	8.83333	0.582
0.2	0	ptsI	4	9.08333	0.386
0.2	0	clcB	4	9.08333	0.422
0.2	0	ycaM	4	9.08333	0.389
0.2	0	yadI	4	9.08333	0.51
0.2	0	AG1	4	9.08333	0.578
0.2	0	ptsI	4	9.33333	0.393
0.2	0	clcB	4	9.33333	0.426
0.2	0	ycaM	4	9.33333	0.395
0.2	0	yadI	4	9.33333	0.52
0.2	0	AG1	4	9.33333	0.588
0.2	0	ptsI	4	9.58333	0.406
0.2	0	clcB	4	9.58333	0.434
0.2	0	ycaM	4	9.58333	0.402
0.2	0	yadI	4	9.58333	0.529
0.2	0	AG1	4	9.58333	0.6
0.2	0	ptsI	4	9.83333	0.412
0.2	0	clcB	4	9.83333	0.438
0.2	0	ycaM	4	9.83333	0.405
0.2	0	yadI	4	9.83333	0.536
0.2	0	AG1	4	9.83333	0.608
0.2	0	ptsI	4	10.0833	0.42
0.2	0	clcB	4	10.0833	0.446
0.2	0	ycaM	4	10.0833	0.412
0.2	0	yadI	4	10.0833	0.543
0.2	0	AG1	4	10.0833	0.62
0.2	0	ptsI	4	10.3333	0.426
0.2	0	clcB	4	10.3333	0.45
0.2	0	ycaM	4	10.3333	0.415
0.2	0	yadI	4	10.3333	0.551
0.2	0	AG1	4	10.3333	0.629
0.2	0	ptsI	4	10.5833	0.432
0.2	0	clcB	4	10.5833	0.459
0.2	0	ycaM	4	10.5833	0.42
0.2	0	yadI	4	10.5833	0.559
0.2	0	AG1	4	10.5833	0.638
0.2	0	ptsI	4	10.8333	0.436
0.2	0	clcB	4	10.8333	0.462
0.2	0	ycaM	4	10.8333	0.424

0.2	0	yadI	4	10.8333	0.565
0.2	0	AG1	4	10.8333	0.645
0.2	0	ptsI	4	11.0833	0.444
0.2	0	clcB	4	11.0833	0.466
0.2	0	ycaM	4	11.0833	0.43
0.2	0	yadI	4	11.0833	0.573
0.2	0	AG1	4	11.0833	0.657
0.2	0	ptsI	4	11.3333	0.454
0.2	0	clcB	4	11.3333	0.477
0.2	0	ycaM	4	11.3333	0.435
0.2	0	yadI	4	11.3333	0.578
0.2	0	AG1	4	11.3333	0.665
0.2	0	ptsI	4	11.5833	0.457
0.2	0	clcB	4	11.5833	0.478
0.2	0	ycaM	4	11.5833	0.438
0.2	0	yadI	4	11.5833	0.581
0.2	0	AG1	4	11.5833	0.672
0.2	0	ptsI	4	11.8333	0.466
0.2	0	clcB	4	11.8333	0.481
0.2	0	ycaM	4	11.8333	0.443
0.2	0	yadI	4	11.8333	0.588
0.2	0	AG1	4	11.8333	0.68
0.2	0	ptsI	4	12.0833	0.472
0.2	0	clcB	4	12.0833	0.488
0.2	0	ycaM	4	12.0833	0.446
0.2	0	yadI	4	12.0833	0.592
0.2	0	AG1	4	12.0833	0.686
0.2	0	ptsI	4	12.3333	0.477
0.2	0	clcB	4	12.3333	0.497
0.2	0	ycaM	4	12.3333	0.452
0.2	0	yadI	4	12.3333	0.597
0.2	0	AG1	4	12.3333	0.694
0.2	0	ptsI	4	12.5833	0.484
0.2	0	clcB	4	12.5833	0.5
0.2	0	ycaM	4	12.5833	0.455
0.2	0	yadI	4	12.5833	0.605
0.2	0	AG1	4	12.5833	0.701
0.2	0	ptsI	4	12.8333	0.488
0.2	0	clcB	4	12.8333	0.5
0.2	0	ycaM	4	12.8333	0.458
0.2	0	yadI	4	12.8333	0.609
0.2	0	AG1	4	12.8333	0.707
0.2	0	ptsI	4	13.0833	0.494
0.2	0	clcB	4	13.0833	0.509
0.2	0	ycaM	4	13.0833	0.464
0.2	0	yadI	4	13.0833	0.614
0.2	0	AG1	4	13.0833	0.713
0.2	0	ptsI	4	13.3333	0.499
0.2	0	clcB	4	13.3333	0.515
0.2	0	ycaM	4	13.3333	0.47
0.2	0	yadI	4	13.3333	0.616
0.2	0	AG1	4	13.3333	0.719
0.2	0	ptsI	4	13.5833	0.504

0.2	0	clcB	4	13.5833	0.516
0.2	0	ycaM	4	13.5833	0.473
0.2	0	yadI	4	13.5833	0.626
0.2	0	AG1	4	13.5833	0.721
0.2	0	ptsI	4	13.8333	0.509
0.2	0	clcB	4	13.8333	0.521
0.2	0	ycaM	4	13.8333	0.483
0.2	0	yadI	4	13.8333	0.623
0.2	0	AG1	4	13.8333	0.726
0.2	0	ptsI	4	14.0833	0.509
0.2	0	clcB	4	14.0833	0.524
0.2	0	ycaM	4	14.0833	0.485
0.2	0	yadI	4	14.0833	0.629
0.2	0	AG1	4	14.0833	0.73
0.2	0	ptsI	4	14.3333	0.518
0.2	0	clcB	4	14.3333	0.525
0.2	0	ycaM	4	14.3333	0.488
0.2	0	yadI	4	14.3333	0.632
0.2	0	AG1	4	14.3333	0.728
0.2	0	ptsI	4	14.5833	0.521
0.2	0	clcB	4	14.5833	0.527
0.2	0	ycaM	4	14.5833	0.491
0.2	0	yadI	4	14.5833	0.633
0.2	0	AG1	4	14.5833	0.737
0.2	0	ptsI	4	14.8333	0.524
0.2	0	clcB	4	14.8333	0.531
0.2	0	ycaM	4	14.8333	0.499
0.2	0	yadI	4	14.8333	0.638
0.2	0	AG1	4	14.8333	0.742
0.2	0	ptsI	4	15.0833	0.53
0.2	0	clcB	4	15.0833	0.531
0.2	0	ycaM	4	15.0833	0.5
0.2	0	yadI	4	15.0833	0.644
0.2	0	AG1	4	15.0833	0.746
0.2	0	ptsI	4	15.3333	0.536
0.2	0	clcB	4	15.3333	0.536
0.2	0	ycaM	4	15.3333	0.5
0.2	0	yadI	4	15.3333	0.644
0.2	0	AG1	4	15.3333	0.747
0.2	0	ptsI	4	15.5833	0.54
0.2	0	clcB	4	15.5833	0.541
0.2	0	ycaM	4	15.5833	0.503
0.2	0	yadI	4	15.5833	0.648
0.2	0	AG1	4	15.5833	0.75
0.2	0	ptsI	4	15.8333	0.542
0.2	0	clcB	4	15.8333	0.537
0.2	0	ycaM	4	15.8333	0.508
0.2	0	yadI	4	15.8333	0.655
0.2	0	AG1	4	15.8333	0.752
0.2	0	ptsI	4	16.0833	0.542
0.2	0	clcB	4	16.0833	0.546
0.2	0	ycaM	4	16.0833	0.511
0.2	0	yadI	4	16.0833	0.659

0.2	0	AG1	4	16.0833	0.757
0.2	0	ptsI	4	16.3333	0.546
0.2	0	clcB	4	16.3333	0.546
0.2	0	ycaM	4	16.3333	0.513
0.2	0	yadI	4	16.3333	0.656
0.2	0	AG1	4	16.3333	0.757
0.2	0	ptsI	4	16.5833	0.55
0.2	0	clcB	4	16.5833	0.545
0.2	0	ycaM	4	16.5833	0.518
0.2	0	yadI	4	16.5833	0.661
0.2	0	AG1	4	16.5833	0.757
0.2	0	ptsI	4	16.8333	0.552
0.2	0	clcB	4	16.8333	0.546
0.2	0	ycaM	4	16.8333	0.518
0.2	0	yadI	4	16.8333	0.663
0.2	0	AG1	4	16.8333	0.761
0.2	0	ptsI	4	17.0833	0.559
0.2	0	clcB	4	17.0833	0.551
0.2	0	ycaM	4	17.0833	0.52
0.2	0	yadI	4	17.0833	0.667
0.2	0	AG1	4	17.0833	0.76
0.2	0	ptsI	4	17.3333	0.563
0.2	0	clcB	4	17.3333	0.557
0.2	0	ycaM	4	17.3333	0.528
0.2	0	yadI	4	17.3333	0.668
0.2	0	AG1	4	17.3333	0.763
0.2	0	ptsI	4	17.5833	0.56
0.2	0	clcB	4	17.5833	0.551
0.2	0	ycaM	4	17.5833	0.524
0.2	0	yadI	4	17.5833	0.67
0.2	0	AG1	4	17.5833	0.762
0.2	0	ptsI	4	17.8333	0.577
0.2	0	clcB	4	17.8333	0.556
0.2	0	ycaM	4	17.8333	0.527
0.2	0	yadI	4	17.8333	0.671
0.2	0	AG1	4	17.8333	0.77
0.2	0	ptsI	4	18.0833	0.575
0.2	0	clcB	4	18.0833	0.554
0.2	0	ycaM	4	18.0833	0.53
0.2	0	yadI	4	18.0833	0.676
0.2	0	AG1	4	18.0833	0.764
0.2	0	ptsI	4	18.3333	0.57
0.2	0	clcB	4	18.3333	0.556
0.2	0	ycaM	4	18.3333	0.532
0.2	0	yadI	4	18.3333	0.675
0.2	0	AG1	4	18.3333	0.769
0.2	0	ptsI	4	18.5833	0.571
0.2	0	clcB	4	18.5833	0.558
0.2	0	ycaM	4	18.5833	0.532
0.2	0	yadI	4	18.5833	0.679
0.2	0	AG1	4	18.5833	0.77
0.2	0	ptsI	4	18.8333	0.585
0.2	0	clcB	4	18.8333	0.563

0.2	0	ycaM	4	18.8333	0.536
0.2	0	yadI	4	18.8333	0.677
0.2	0	AG1	4	18.8333	0.775
0.2	0	ptsI	4	19.0833	0.585
0.2	0	clcB	4	19.0833	0.566
0.2	0	ycaM	4	19.0833	0.539
0.2	0	yadI	4	19.0833	0.68
0.2	0	AG1	4	19.0833	0.777
0.2	0	ptsI	4	19.3333	0.587
0.2	0	clcB	4	19.3333	0.568
0.2	0	ycaM	4	19.3333	0.54
0.2	0	yadI	4	19.3333	0.683
0.2	0	AG1	4	19.3333	0.776
0.2	0	ptsI	4	19.5833	0.594
0.2	0	clcB	4	19.5833	0.57
0.2	0	ycaM	4	19.5833	0.543
0.2	0	yadI	4	19.5833	0.684
0.2	0	AG1	4	19.5833	0.776
0.2	0	ptsI	4	19.8333	0.594
0.2	0	clcB	4	19.8333	0.566
0.2	0	ycaM	4	19.8333	0.544
0.2	0	yadI	4	19.8333	0.68
0.2	0	AG1	4	19.8333	0.777
0.2	0	ptsI	4	20.0833	0.595
0.2	0	clcB	4	20.0833	0.573
0.2	0	ycaM	4	20.0833	0.548
0.2	0	yadI	4	20.0833	0.686
0.2	0	AG1	4	20.0833	0.787
0.2	0	ptsI	4	20.3333	0.6
0.2	0	clcB	4	20.3333	0.572
0.2	0	ycaM	4	20.3333	0.549
0.2	0	yadI	4	20.3333	0.686
0.2	0	AG1	4	20.3333	0.778
0.2	0	ptsI	4	20.5833	0.601
0.2	0	clcB	4	20.5833	0.572
0.2	0	ycaM	4	20.5833	0.552
0.2	0	yadI	4	20.5833	0.686
0.2	0	AG1	4	20.5833	0.778
0.2	0	ptsI	4	20.8333	0.604
0.2	0	clcB	4	20.8333	0.578
0.2	0	ycaM	4	20.8333	0.557
0.2	0	yadI	4	20.8333	0.687
0.2	0	AG1	4	20.8333	0.779
0.2	0	ptsI	4	21.0833	0.61
0.2	0	clcB	4	21.0833	0.576
0.2	0	ycaM	4	21.0833	0.557
0.2	0	yadI	4	21.0833	0.69
0.2	0	AG1	4	21.0833	0.781
0.2	0	ptsI	4	21.3333	0.605
0.2	0	clcB	4	21.3333	0.576
0.2	0	ycaM	4	21.3333	0.558
0.2	0	yadI	4	21.3333	0.687
0.2	0	AG1	4	21.3333	0.781

0.2	0	ptsI	4	21.5833	0.613
0.2	0	clcB	4	21.5833	0.579
0.2	0	ycaM	4	21.5833	0.564
0.2	0	yadI	4	21.5833	0.689
0.2	0	AG1	4	21.5833	0.784
0.2	0	ptsI	4	21.8333	0.613
0.2	0	clcB	4	21.8333	0.581
0.2	0	ycaM	4	21.8333	0.565
0.2	0	yadI	4	21.8333	0.691
0.2	0	AG1	4	21.8333	0.784
0.2	0	ptsI	4	22.0833	0.619
0.2	0	clcB	4	22.0833	0.582
0.2	0	ycaM	4	22.0833	0.565
0.2	0	yadI	4	22.0833	0.69
0.2	0	AG1	4	22.0833	0.784
0.2	0	ptsI	4	22.3333	0.623
0.2	0	clcB	4	22.3333	0.583
0.2	0	ycaM	4	22.3333	0.567
0.2	0	yadI	4	22.3333	0.695
0.2	0	AG1	4	22.3333	0.784
0.2	0	ptsI	4	22.5833	0.623
0.2	0	clcB	4	22.5833	0.581
0.2	0	ycaM	4	22.5833	0.57
0.2	0	yadI	4	22.5833	0.692
0.2	0	AG1	4	22.5833	0.781
0.2	0	ptsI	4	22.8333	0.628
0.2	0	clcB	4	22.8333	0.586
0.2	0	ycaM	4	22.8333	0.571
0.2	0	yadI	4	22.8333	0.695
0.2	0	AG1	4	22.8333	0.783
0.2	0	ptsI	4	23.0833	0.639
0.2	0	clcB	4	23.0833	0.588
0.2	0	ycaM	4	23.0833	0.574
0.2	0	yadI	4	23.0833	0.693
0.2	0	AG1	4	23.0833	0.786
0.2	0	ptsI	4	23.3333	0.638
0.2	0	clcB	4	23.3333	0.592
0.2	0	ycaM	4	23.3333	0.579
0.2	0	yadI	4	23.3333	0.698
0.2	0	AG1	4	23.3333	0.787
0.2	0	ptsI	4	23.5833	0.64
0.2	0	clcB	4	23.5833	0.59
0.2	0	ycaM	4	23.5833	0.583
0.2	0	yadI	4	23.5833	0.695
0.2	0	AG1	4	23.5833	0.787
0.2	0	ptsI	4	23.8333	0.645
0.2	0	clcB	4	23.8333	0.591
0.2	0	ycaM	4	23.8333	0.584
0.2	0	yadI	4	23.8333	0.697
0.2	0	AG1	4	23.8333	0.785
0.2	0	ptsI	4	24.0833	0.651
0.2	0	clcB	4	24.0833	0.594
0.2	0	ycaM	4	24.0833	0.584

0.2	0	yadI	4	24.0833	0.7
0.2	0	AG1	4	24.0833	0.784
0.2	0	ptsI	5	0	0.155
0.2	0	clcB	5	0	0.161
0.2	0	ycaM	5	0	0.175
0.2	0	yadI	5	0	0.166
0.2	0	AG1	5	0	0.162
0.2	0	ptsI	5	0.35	0.154
0.2	0	clcB	5	0.35	0.158
0.2	0	ycaM	5	0.35	0.17
0.2	0	yadI	5	0.35	0.161
0.2	0	AG1	5	0.35	0.157
0.2	0	ptsI	5	0.6	0.155
0.2	0	clcB	5	0.6	0.158
0.2	0	ycaM	5	0.6	0.169
0.2	0	yadI	5	0.6	0.161
0.2	0	AG1	5	0.6	0.159
0.2	0	ptsI	5	0.85	0.153
0.2	0	clcB	5	0.85	0.158
0.2	0	ycaM	5	0.85	0.17
0.2	0	yadI	5	0.85	0.161
0.2	0	AG1	5	0.85	0.16
0.2	0	ptsI	5	1.1	0.157
0.2	0	clcB	5	1.1	0.161
0.2	0	ycaM	5	1.1	0.172
0.2	0	yadI	5	1.1	0.162
0.2	0	AG1	5	1.1	0.162
0.2	0	ptsI	5	1.35	0.157
0.2	0	clcB	5	1.35	0.162
0.2	0	ycaM	5	1.35	0.174
0.2	0	yadI	5	1.35	0.164
0.2	0	AG1	5	1.35	0.163
0.2	0	ptsI	5	1.6	0.163
0.2	0	clcB	5	1.6	0.166
0.2	0	ycaM	5	1.6	0.177
0.2	0	yadI	5	1.6	0.166
0.2	0	AG1	5	1.6	0.169
0.2	0	ptsI	5	1.85	0.165
0.2	0	clcB	5	1.85	0.169
0.2	0	ycaM	5	1.85	0.179
0.2	0	yadI	5	1.85	0.169
0.2	0	AG1	5	1.85	0.174
0.2	0	ptsI	5	2.1	0.17
0.2	0	clcB	5	2.1	0.175
0.2	0	ycaM	5	2.1	0.184
0.2	0	yadI	5	2.1	0.174
0.2	0	AG1	5	2.1	0.182
0.2	0	ptsI	5	2.35	0.176
0.2	0	clcB	5	2.35	0.183
0.2	0	ycaM	5	2.35	0.188
0.2	0	yadI	5	2.35	0.179
0.2	0	AG1	5	2.35	0.191
0.2	0	ptsI	5	2.6	0.18

0.2	0	clcB	5	2.6	0.184
0.2	0	ycaM	5	2.6	0.19
0.2	0	yadI	5	2.6	0.187
0.2	0	AG1	5	2.6	0.191
0.2	0	ptsI	5	2.85	0.189
0.2	0	clcB	5	2.85	0.198
0.2	0	ycaM	5	2.85	0.204
0.2	0	yadI	5	2.85	0.194
0.2	0	AG1	5	2.85	0.21
0.2	0	ptsI	5	3.1	0.196
0.2	0	clcB	5	3.1	0.21
0.2	0	ycaM	5	3.1	0.207
0.2	0	yadI	5	3.1	0.202
0.2	0	AG1	5	3.1	0.221
0.2	0	ptsI	5	3.35	0.201
0.2	0	clcB	5	3.35	0.225
0.2	0	ycaM	5	3.35	0.217
0.2	0	yadI	5	3.35	0.213
0.2	0	AG1	5	3.35	0.244
0.2	0	ptsI	5	3.6	0.204
0.2	0	clcB	5	3.6	0.24
0.2	0	ycaM	5	3.6	0.228
0.2	0	yadI	5	3.6	0.224
0.2	0	AG1	5	3.6	0.263
0.2	0	ptsI	5	3.85	0.212
0.2	0	clcB	5	3.85	0.252
0.2	0	ycaM	5	3.85	0.243
0.2	0	yadI	5	3.85	0.237
0.2	0	AG1	5	3.85	0.278
0.2	0	ptsI	5	4.38333	0.209
0.2	0	clcB	5	4.38333	0.255
0.2	0	ycaM	5	4.38333	0.256
0.2	0	yadI	5	4.38333	0.263
0.2	0	AG1	5	4.38333	0.312
0.2	0	ptsI	5	4.63333	0.213
0.2	0	clcB	5	4.63333	0.268
0.2	0	ycaM	5	4.63333	0.258
0.2	0	yadI	5	4.63333	0.266
0.2	0	AG1	5	4.63333	0.283
0.2	0	ptsI	5	4.88333	0.218
0.2	0	clcB	5	4.88333	0.283
0.2	0	ycaM	5	4.88333	0.269
0.2	0	yadI	5	4.88333	0.277
0.2	0	AG1	5	4.88333	0.303
0.2	0	ptsI	5	5.13333	0.224
0.2	0	clcB	5	5.13333	0.296
0.2	0	ycaM	5	5.13333	0.281
0.2	0	yadI	5	5.13333	0.29
0.2	0	AG1	5	5.13333	0.313
0.2	0	ptsI	5	5.38333	0.233
0.2	0	clcB	5	5.38333	0.309
0.2	0	ycaM	5	5.38333	0.296
0.2	0	yadI	5	5.38333	0.304

0.2	0	AG1	5	5.38333	0.32
0.2	0	ptsI	5	5.63333	0.243
0.2	0	clcB	5	5.63333	0.33
0.2	0	ycaM	5	5.63333	0.307
0.2	0	yadI	5	5.63333	0.318
0.2	0	AG1	5	5.63333	0.338
0.2	0	ptsI	5	5.88333	0.254
0.2	0	clcB	5	5.88333	0.346
0.2	0	ycaM	5	5.88333	0.32
0.2	0	yadI	5	5.88333	0.333
0.2	0	AG1	5	5.88333	0.341
0.2	0	ptsI	5	6.13333	0.266
0.2	0	clcB	5	6.13333	0.36
0.2	0	ycaM	5	6.13333	0.33
0.2	0	yadI	5	6.13333	0.348
0.2	0	AG1	5	6.13333	0.36
0.2	0	ptsI	5	6.38333	0.276
0.2	0	clcB	5	6.38333	0.371
0.2	0	ycaM	5	6.38333	0.341
0.2	0	yadI	5	6.38333	0.364
0.2	0	AG1	5	6.38333	0.381
0.2	0	ptsI	5	6.63333	0.288
0.2	0	clcB	5	6.63333	0.385
0.2	0	ycaM	5	6.63333	0.349
0.2	0	yadI	5	6.63333	0.377
0.2	0	AG1	5	6.63333	0.4
0.2	0	ptsI	5	6.88333	0.299
0.2	0	clcB	5	6.88333	0.398
0.2	0	ycaM	5	6.88333	0.36
0.2	0	yadI	5	6.88333	0.392
0.2	0	AG1	5	6.88333	0.425
0.2	0	ptsI	5	7.13333	0.312
0.2	0	clcB	5	7.13333	0.413
0.2	0	ycaM	5	7.13333	0.369
0.2	0	yadI	5	7.13333	0.408
0.2	0	AG1	5	7.13333	0.443
0.2	0	ptsI	5	7.38333	0.324
0.2	0	clcB	5	7.38333	0.426
0.2	0	ycaM	5	7.38333	0.381
0.2	0	yadI	5	7.38333	0.421
0.2	0	AG1	5	7.38333	0.457
0.2	0	ptsI	5	7.63333	0.335
0.2	0	clcB	5	7.63333	0.438
0.2	0	ycaM	5	7.63333	0.391
0.2	0	yadI	5	7.63333	0.436
0.2	0	AG1	5	7.63333	0.472
0.2	0	ptsI	5	7.88333	0.346
0.2	0	clcB	5	7.88333	0.454
0.2	0	ycaM	5	7.88333	0.42
0.2	0	yadI	5	7.88333	0.45
0.2	0	AG1	5	7.88333	0.489
0.2	0	ptsI	5	8.13333	0.361
0.2	0	clcB	5	8.13333	0.465

0.2	0	ycaM	5	8.13333	0.422
0.2	0	yadI	5	8.13333	0.463
0.2	0	AG1	5	8.13333	0.502
0.2	0	ptsI	5	8.38333	0.375
0.2	0	clcB	5	8.38333	0.479
0.2	0	ycaM	5	8.38333	0.439
0.2	0	yadI	5	8.38333	0.475
0.2	0	AG1	5	8.38333	0.515
0.2	0	ptsI	5	8.88333	0.333
0.2	0	clcB	5	8.88333	0.349
0.2	0	ycaM	5	8.88333	0.349
0.2	0	yadI	5	8.88333	0.381
0.2	0	AG1	5	8.88333	0.536
0.2	0	ptsI	5	9.13333	0.337
0.2	0	clcB	5	9.13333	0.358
0.2	0	ycaM	5	9.13333	0.355
0.2	0	yadI	5	9.13333	0.382
0.2	0	AG1	5	9.13333	0.536
0.2	0	ptsI	5	9.38333	0.342
0.2	0	clcB	5	9.38333	0.368
0.2	0	ycaM	5	9.38333	0.363
0.2	0	yadI	5	9.38333	0.391
0.2	0	AG1	5	9.38333	0.546
0.2	0	ptsI	5	9.63333	0.357
0.2	0	clcB	5	9.63333	0.378
0.2	0	ycaM	5	9.63333	0.371
0.2	0	yadI	5	9.63333	0.401
0.2	0	AG1	5	9.63333	0.56
0.2	0	ptsI	5	9.88333	0.364
0.2	0	clcB	5	9.88333	0.387
0.2	0	ycaM	5	9.88333	0.378
0.2	0	yadI	5	9.88333	0.409
0.2	0	AG1	5	9.88333	0.575
0.2	0	ptsI	5	10.1333	0.373
0.2	0	clcB	5	10.1333	0.395
0.2	0	ycaM	5	10.1333	0.385
0.2	0	yadI	5	10.1333	0.416
0.2	0	AG1	5	10.1333	0.583
0.2	0	ptsI	5	10.3833	0.388
0.2	0	clcB	5	10.3833	0.404
0.2	0	ycaM	5	10.3833	0.392
0.2	0	yadI	5	10.3833	0.425
0.2	0	AG1	5	10.3833	0.595
0.2	0	ptsI	5	10.6333	0.391
0.2	0	clcB	5	10.6333	0.408
0.2	0	ycaM	5	10.6333	0.398
0.2	0	yadI	5	10.6333	0.429
0.2	0	AG1	5	10.6333	0.603
0.2	0	ptsI	5	10.8833	0.4
0.2	0	clcB	5	10.8833	0.414
0.2	0	ycaM	5	10.8833	0.401
0.2	0	yadI	5	10.8833	0.438
0.2	0	AG1	5	10.8833	0.613

0.2	0	ptsI	5	11.1333	0.408
0.2	0	clcB	5	11.1333	0.417
0.2	0	ycaM	5	11.1333	0.404
0.2	0	yadI	5	11.1333	0.44
0.2	0	AG1	5	11.1333	0.621
0.2	0	ptsI	5	11.3833	0.417
0.2	0	clcB	5	11.3833	0.422
0.2	0	ycaM	5	11.3833	0.405
0.2	0	yadI	5	11.3833	0.447
0.2	0	AG1	5	11.3833	0.635
0.2	0	ptsI	5	11.6333	0.424
0.2	0	clcB	5	11.6333	0.427
0.2	0	ycaM	5	11.6333	0.408
0.2	0	yadI	5	11.6333	0.454
0.2	0	AG1	5	11.6333	0.644
0.2	0	ptsI	5	11.8833	0.435
0.2	0	clcB	5	11.8833	0.432
0.2	0	ycaM	5	11.8833	0.411
0.2	0	yadI	5	11.8833	0.462
0.2	0	AG1	5	11.8833	0.652
0.2	0	ptsI	5	12.1333	0.446
0.2	0	clcB	5	12.1333	0.442
0.2	0	ycaM	5	12.1333	0.416
0.2	0	yadI	5	12.1333	0.469
0.2	0	AG1	5	12.1333	0.662
0.2	0	ptsI	5	12.3833	0.45
0.2	0	clcB	5	12.3833	0.445
0.2	0	ycaM	5	12.3833	0.414
0.2	0	yadI	5	12.3833	0.476
0.2	0	AG1	5	12.3833	0.665
0.2	0	ptsI	5	12.6333	0.457
0.2	0	clcB	5	12.6333	0.447
0.2	0	ycaM	5	12.6333	0.419
0.2	0	yadI	5	12.6333	0.479
0.2	0	AG1	5	12.6333	0.677
0.2	0	ptsI	5	12.8833	0.469
0.2	0	clcB	5	12.8833	0.457
0.2	0	ycaM	5	12.8833	0.427
0.2	0	yadI	5	12.8833	0.487
0.2	0	AG1	5	12.8833	0.687
0.2	0	ptsI	5	13.1333	0.473
0.2	0	clcB	5	13.1333	0.459
0.2	0	ycaM	5	13.1333	0.43
0.2	0	yadI	5	13.1333	0.491
0.2	0	AG1	5	13.1333	0.694
0.2	0	ptsI	5	13.3833	0.477
0.2	0	clcB	5	13.3833	0.463
0.2	0	ycaM	5	13.3833	0.432
0.2	0	yadI	5	13.3833	0.496
0.2	0	AG1	5	13.3833	0.698
0.2	0	ptsI	5	13.6333	0.488
0.2	0	clcB	5	13.6333	0.468
0.2	0	ycaM	5	13.6333	0.436

0.2	0	yadI	5	13.6333	0.503
0.2	0	AG1	5	13.6333	0.708
0.2	0	ptsI	5	13.8833	0.494
0.2	0	clcB	5	13.8833	0.471
0.2	0	ycaM	5	13.8833	0.44
0.2	0	yadI	5	13.8833	0.506
0.2	0	AG1	5	13.8833	0.714
0.2	0	ptsI	5	14.1333	0.503
0.2	0	clcB	5	14.1333	0.478
0.2	0	ycaM	5	14.1333	0.448
0.2	0	yadI	5	14.1333	0.51
0.2	0	AG1	5	14.1333	0.722
0.2	0	ptsI	5	14.3833	0.509
0.2	0	clcB	5	14.3833	0.486
0.2	0	ycaM	5	14.3833	0.45
0.2	0	yadI	5	14.3833	0.516
0.2	0	AG1	5	14.3833	0.729
0.2	0	ptsI	5	14.6333	0.517
0.2	0	clcB	5	14.6333	0.487
0.2	0	ycaM	5	14.6333	0.454
0.2	0	yadI	5	14.6333	0.519
0.2	0	AG1	5	14.6333	0.732
0.2	0	ptsI	5	14.8833	0.519
0.2	0	clcB	5	14.8833	0.488
0.2	0	ycaM	5	14.8833	0.456
0.2	0	yadI	5	14.8833	0.523
0.2	0	AG1	5	14.8833	0.737
0.2	0	ptsI	5	15.1333	0.524
0.2	0	clcB	5	15.1333	0.492
0.2	0	ycaM	5	15.1333	0.461
0.2	0	yadI	5	15.1333	0.528
0.2	0	AG1	5	15.1333	0.742
0.2	0	ptsI	5	15.3833	0.53
0.2	0	clcB	5	15.3833	0.495
0.2	0	ycaM	5	15.3833	0.464
0.2	0	yadI	5	15.3833	0.53
0.2	0	AG1	5	15.3833	0.745
0.2	0	ptsI	5	15.6333	0.531
0.2	0	clcB	5	15.6333	0.496
0.2	0	ycaM	5	15.6333	0.465
0.2	0	yadI	5	15.6333	0.535
0.2	0	AG1	5	15.6333	0.75
0.2	0	ptsI	5	15.8833	0.54
0.2	0	clcB	5	15.8833	0.499
0.2	0	ycaM	5	15.8833	0.47
0.2	0	yadI	5	15.8833	0.535
0.2	0	AG1	5	15.8833	0.754
0.2	0	ptsI	5	16.1333	0.545
0.2	0	clcB	5	16.1333	0.498
0.2	0	ycaM	5	16.1333	0.477
0.2	0	yadI	5	16.1333	0.543
0.2	0	AG1	5	16.1333	0.759
0.2	0	ptsI	5	16.3833	0.549

0.2	0	clcB	5	16.3833	0.501
0.2	0	ycaM	5	16.3833	0.475
0.2	0	yadI	5	16.3833	0.542
0.2	0	AG1	5	16.3833	0.761
0.2	0	ptsI	5	16.6333	0.553
0.2	0	clcB	5	16.6333	0.502
0.2	0	ycaM	5	16.6333	0.48
0.2	0	yadI	5	16.6333	0.544
0.2	0	AG1	5	16.6333	0.764
0.2	0	ptsI	5	16.8833	0.56
0.2	0	clcB	5	16.8833	0.508
0.2	0	ycaM	5	16.8833	0.484
0.2	0	yadI	5	16.8833	0.551
0.2	0	AG1	5	16.8833	0.771
0.2	0	ptsI	5	17.1333	0.566
0.2	0	clcB	5	17.1333	0.511
0.2	0	ycaM	5	17.1333	0.488
0.2	0	yadI	5	17.1333	0.556
0.2	0	AG1	5	17.1333	0.775
0.2	0	ptsI	5	17.3833	0.565
0.2	0	clcB	5	17.3833	0.508
0.2	0	ycaM	5	17.3833	0.489
0.2	0	yadI	5	17.3833	0.555
0.2	0	AG1	5	17.3833	0.776
0.2	0	ptsI	5	17.6333	0.571
0.2	0	clcB	5	17.6333	0.51
0.2	0	ycaM	5	17.6333	0.492
0.2	0	yadI	5	17.6333	0.558
0.2	0	AG1	5	17.6333	0.778
0.2	0	ptsI	5	17.8833	0.577
0.2	0	clcB	5	17.8833	0.517
0.2	0	ycaM	5	17.8833	0.492
0.2	0	yadI	5	17.8833	0.558
0.2	0	AG1	5	17.8833	0.78
0.2	0	ptsI	5	18.1333	0.58
0.2	0	clcB	5	18.1333	0.517
0.2	0	ycaM	5	18.1333	0.499
0.2	0	yadI	5	18.1333	0.564
0.2	0	AG1	5	18.1333	0.786
0.2	0	ptsI	5	18.3833	0.585
0.2	0	clcB	5	18.3833	0.517
0.2	0	ycaM	5	18.3833	0.499
0.2	0	yadI	5	18.3833	0.566
0.2	0	AG1	5	18.3833	0.789
0.2	0	ptsI	5	18.6333	0.588
0.2	0	clcB	5	18.6333	0.518
0.2	0	ycaM	5	18.6333	0.503
0.2	0	yadI	5	18.6333	0.568
0.2	0	AG1	5	18.6333	0.79
0.2	0	ptsI	5	18.8833	0.59
0.2	0	clcB	5	18.8833	0.517
0.2	0	ycaM	5	18.8833	0.507
0.2	0	yadI	5	18.8833	0.569

0.2	0	AG1	5	18.8833	0.793
0.2	0	ptsI	5	19.1333	0.595
0.2	0	clcB	5	19.1333	0.52
0.2	0	ycaM	5	19.1333	0.508
0.2	0	yadI	5	19.1333	0.572
0.2	0	AG1	5	19.1333	0.795
0.2	0	ptsI	5	19.3833	0.6
0.2	0	clcB	5	19.3833	0.523
0.2	0	ycaM	5	19.3833	0.509
0.2	0	yadI	5	19.3833	0.577
0.2	0	AG1	5	19.3833	0.799
0.2	0	ptsI	5	19.6333	0.602
0.2	0	clcB	5	19.6333	0.522
0.2	0	ycaM	5	19.6333	0.515
0.2	0	yadI	5	19.6333	0.582
0.2	0	AG1	5	19.6333	0.801
0.2	0	ptsI	5	19.8833	0.605
0.2	0	clcB	5	19.8833	0.525
0.2	0	ycaM	5	19.8833	0.513
0.2	0	yadI	5	19.8833	0.582
0.2	0	AG1	5	19.8833	0.801
0.2	0	ptsI	5	20.1333	0.608
0.2	0	clcB	5	20.1333	0.523
0.2	0	ycaM	5	20.1333	0.514
0.2	0	yadI	5	20.1333	0.584
0.2	0	AG1	5	20.1333	0.804
0.2	0	ptsI	5	20.3833	0.613
0.2	0	clcB	5	20.3833	0.525
0.2	0	ycaM	5	20.3833	0.515
0.2	0	yadI	5	20.3833	0.586
0.2	0	AG1	5	20.3833	0.807
0.2	0	ptsI	5	20.6333	0.614
0.2	0	clcB	5	20.6333	0.53
0.2	0	ycaM	5	20.6333	0.515
0.2	0	yadI	5	20.6333	0.584
0.2	0	AG1	5	20.6333	0.806
0.2	0	ptsI	5	20.8833	0.617
0.2	0	clcB	5	20.8833	0.526
0.2	0	ycaM	5	20.8833	0.518
0.2	0	yadI	5	20.8833	0.591
0.2	0	AG1	5	20.8833	0.812
0.2	0	ptsI	5	21.1333	0.619
0.2	0	clcB	5	21.1333	0.527
0.2	0	ycaM	5	21.1333	0.519
0.2	0	yadI	5	21.1333	0.591
0.2	0	AG1	5	21.1333	0.814
0.2	0	ptsI	5	21.3833	0.619
0.2	0	clcB	5	21.3833	0.527
0.2	0	ycaM	5	21.3833	0.522
0.2	0	yadI	5	21.3833	0.597
0.2	0	AG1	5	21.3833	0.814
0.2	0	ptsI	5	21.6333	0.622
0.2	0	clcB	5	21.6333	0.529

0.2	0	ycaM	5	21.6333	0.525
0.2	0	yadI	5	21.6333	0.597
0.2	0	AG1	5	21.6333	0.817
0.2	0	ptsI	5	21.8833	0.629
0.2	0	clcB	5	21.8833	0.53
0.2	0	ycaM	5	21.8833	0.527
0.2	0	yadI	5	21.8833	0.603
0.2	0	AG1	5	21.8833	0.818
0.2	0	ptsI	5	22.1333	0.627
0.2	0	clcB	5	22.1333	0.531
0.2	0	ycaM	5	22.1333	0.524
0.2	0	yadI	5	22.1333	0.601
0.2	0	AG1	5	22.1333	0.816
0.2	0	ptsI	5	22.3833	0.63
0.2	0	clcB	5	22.3833	0.528
0.2	0	ycaM	5	22.3833	0.529
0.2	0	yadI	5	22.3833	0.605
0.2	0	AG1	5	22.3833	0.819
0.2	0	ptsI	5	22.6333	0.629
0.2	0	clcB	5	22.6333	0.53
0.2	0	ycaM	5	22.6333	0.531
0.2	0	yadI	5	22.6333	0.609
0.2	0	AG1	5	22.6333	0.821
0.2	0	ptsI	5	22.8833	0.631
0.2	0	clcB	5	22.8833	0.53
0.2	0	ycaM	5	22.8833	0.53
0.2	0	yadI	5	22.8833	0.606
0.2	0	AG1	5	22.8833	0.821
0.2	0	ptsI	5	23.1333	0.637
0.2	0	clcB	5	23.1333	0.531
0.2	0	ycaM	5	23.1333	0.53
0.2	0	yadI	5	23.1333	0.61
0.2	0	AG1	5	23.1333	0.822
0.2	0	ptsI	5	23.3833	0.639
0.2	0	clcB	5	23.3833	0.53
0.2	0	ycaM	5	23.3833	0.53
0.2	0	yadI	5	23.3833	0.61
0.2	0	AG1	5	23.3833	0.821
0.2	0	ptsI	5	23.6333	0.638
0.2	0	clcB	5	23.6333	0.532
0.2	0	ycaM	5	23.6333	0.532
0.2	0	yadI	5	23.6333	0.614
0.2	0	AG1	5	23.6333	0.824
0.2	0	ptsI	5	23.8833	0.648
0.2	0	clcB	5	23.8833	0.534
0.2	0	ycaM	5	23.8833	0.53
0.2	0	yadI	5	23.8833	0.614
0.2	0	AG1	5	23.8833	0.822
0.2	0	ptsI	5	24.1333	0.648
0.2	0	clcB	5	24.1333	0.531
0.2	0	ycaM	5	24.1333	0.538
0.2	0	yadI	5	24.1333	0.622
0.2	0	AG1	5	24.1333	0.829

0.2	0	ptsI	5	24.3833	0.653
0.2	0	clcB	5	24.3833	0.531
0.2	0	ycaM	5	24.3833	0.533
0.2	0	yadI	5	24.3833	0.619
0.2	0	AG1	5	24.3833	0.825
0.4	0	ptsI	1	0	0.221
0.4	0	clcB	1	0	0.243
0.4	0	ycaM	1	0	0.227
0.4	0	yadI	1	0	0.272
0.4	0	AG1	1	0	0.237
0.4	0	ptsI	1	0.25	0.218
0.4	0	clcB	1	0.25	0.23
0.4	0	ycaM	1	0.25	0.232
0.4	0	yadI	1	0.25	0.259
0.4	0	AG1	1	0.25	0.223
0.4	0	ptsI	1	0.5	0.219
0.4	0	clcB	1	0.5	0.226
0.4	0	ycaM	1	0.5	0.23
0.4	0	yadI	1	0.5	0.255
0.4	0	AG1	1	0.5	0.222
0.4	0	ptsI	1	0.75	0.22
0.4	0	clcB	1	0.75	0.224
0.4	0	ycaM	1	0.75	0.227
0.4	0	yadI	1	0.75	0.255
0.4	0	AG1	1	0.75	0.222
0.4	0	ptsI	1	1	0.224
0.4	0	clcB	1	1	0.224
0.4	0	ycaM	1	1	0.229
0.4	0	yadI	1	1	0.257
0.4	0	AG1	1	1	0.225
0.4	0	ptsI	1	1.25	0.225
0.4	0	clcB	1	1.25	0.226
0.4	0	ycaM	1	1.25	0.23
0.4	0	yadI	1	1.25	0.258
0.4	0	AG1	1	1.25	0.226
0.4	0	ptsI	1	1.5	0.231
0.4	0	clcB	1	1.5	0.225
0.4	0	ycaM	1	1.5	0.235
0.4	0	yadI	1	1.5	0.261
0.4	0	AG1	1	1.5	0.228
0.4	0	ptsI	1	1.75	0.235
0.4	0	clcB	1	1.75	0.229
0.4	0	ycaM	1	1.75	0.238
0.4	0	yadI	1	1.75	0.266
0.4	0	AG1	1	1.75	0.232
0.4	0	ptsI	1	2	0.241
0.4	0	clcB	1	2	0.229
0.4	0	ycaM	1	2	0.247
0.4	0	yadI	1	2	0.272
0.4	0	AG1	1	2	0.239
0.4	0	ptsI	1	2.25	0.249
0.4	0	clcB	1	2.25	0.236
0.4	0	ycaM	1	2.25	0.253

0.4	0	yadI	1	2.25	0.28
0.4	0	AG1	1	2.25	0.241
0.4	0	ptsI	1	2.5	0.256
0.4	0	clcB	1	2.5	0.236
0.4	0	ycaM	1	2.5	0.263
0.4	0	yadI	1	2.5	0.286
0.4	0	AG1	1	2.5	0.253
0.4	0	ptsI	1	2.75	0.266
0.4	0	clcB	1	2.75	0.242
0.4	0	ycaM	1	2.75	0.271
0.4	0	yadI	1	2.75	0.296
0.4	0	AG1	1	2.75	0.253
0.4	0	ptsI	1	3	0.27
0.4	0	clcB	1	3	0.246
0.4	0	ycaM	1	3	0.28
0.4	0	yadI	1	3	0.307
0.4	0	AG1	1	3	0.265
0.4	0	ptsI	1	3.25	0.276
0.4	0	clcB	1	3.25	0.252
0.4	0	ycaM	1	3.25	0.291
0.4	0	yadI	1	3.25	0.327
0.4	0	AG1	1	3.25	0.279
0.4	0	ptsI	1	3.5	0.289
0.4	0	clcB	1	3.5	0.258
0.4	0	ycaM	1	3.5	0.301
0.4	0	yadI	1	3.5	0.342
0.4	0	AG1	1	3.5	0.278
0.4	0	ptsI	1	3.75	0.3
0.4	0	clcB	1	3.75	0.26
0.4	0	ycaM	1	3.75	0.317
0.4	0	yadI	1	3.75	0.357
0.4	0	AG1	1	3.75	0.278
0.4	0	ptsI	1	4	0.315
0.4	0	clcB	1	4	0.262
0.4	0	ycaM	1	4	0.326
0.4	0	yadI	1	4	0.39
0.4	0	AG1	1	4	0.296
0.4	0	ptsI	1	4.25	0.315
0.4	0	clcB	1	4.25	0.27
0.4	0	ycaM	1	4.25	0.338
0.4	0	yadI	1	4.25	0.394
0.4	0	AG1	1	4.25	0.302
0.4	0	ptsI	1	4.5	0.322
0.4	0	clcB	1	4.5	0.278
0.4	0	ycaM	1	4.5	0.348
0.4	0	yadI	1	4.5	0.412
0.4	0	AG1	1	4.5	0.314
0.4	0	ptsI	1	4.75	0.336
0.4	0	clcB	1	4.75	0.286
0.4	0	ycaM	1	4.75	0.376
0.4	0	yadI	1	4.75	0.412
0.4	0	AG1	1	4.75	0.32
0.4	0	ptsI	1	5	0.344

0.4	0	clcB	1	5	0.294
0.4	0	ycaM	1	5	0.371
0.4	0	yadI	1	5	0.433
0.4	0	AG1	1	5	0.334
0.4	0	ptsI	1	5.25	0.355
0.4	0	clcB	1	5.25	0.302
0.4	0	ycaM	1	5.25	0.378
0.4	0	yadI	1	5.25	0.452
0.4	0	AG1	1	5.25	0.345
0.4	0	ptsI	1	5.5	0.363
0.4	0	clcB	1	5.5	0.309
0.4	0	ycaM	1	5.5	0.386
0.4	0	yadI	1	5.5	0.459
0.4	0	AG1	1	5.5	0.358
0.4	0	ptsI	1	5.75	0.374
0.4	0	clcB	1	5.75	0.315
0.4	0	ycaM	1	5.75	0.393
0.4	0	yadI	1	5.75	0.474
0.4	0	AG1	1	5.75	0.369
0.4	0	ptsI	1	6	0.394
0.4	0	clcB	1	6	0.327
0.4	0	ycaM	1	6	0.402
0.4	0	yadI	1	6	0.475
0.4	0	AG1	1	6	0.378
0.4	0	ptsI	1	6.25	0.405
0.4	0	clcB	1	6.25	0.332
0.4	0	ycaM	1	6.25	0.412
0.4	0	yadI	1	6.25	0.486
0.4	0	AG1	1	6.25	0.388
0.4	0	ptsI	1	6.5	0.415
0.4	0	clcB	1	6.5	0.349
0.4	0	ycaM	1	6.5	0.418
0.4	0	yadI	1	6.5	0.497
0.4	0	AG1	1	6.5	0.402
0.4	0	ptsI	1	6.75	0.42
0.4	0	clcB	1	6.75	0.351
0.4	0	ycaM	1	6.75	0.42
0.4	0	yadI	1	6.75	0.514
0.4	0	AG1	1	6.75	0.419
0.4	0	ptsI	1	7	0.424
0.4	0	clcB	1	7	0.362
0.4	0	ycaM	1	7	0.423
0.4	0	yadI	1	7	0.529
0.4	0	AG1	1	7	0.431
0.4	0	ptsI	1	7.25	0.434
0.4	0	clcB	1	7.25	0.368
0.4	0	ycaM	1	7.25	0.43
0.4	0	yadI	1	7.25	0.549
0.4	0	AG1	1	7.25	0.441
0.4	0	ptsI	1	7.5	0.441
0.4	0	clcB	1	7.5	0.377
0.4	0	ycaM	1	7.5	0.437
0.4	0	yadI	1	7.5	0.559

0.4	0	AG1	1	7.5	0.448
0.4	0	ptsI	1	7.75	0.456
0.4	0	clcB	1	7.75	0.389
0.4	0	ycaM	1	7.75	0.447
0.4	0	yadI	1	7.75	0.566
0.4	0	AG1	1	7.75	0.456
0.4	0	ptsI	1	8	0.462
0.4	0	clcB	1	8	0.396
0.4	0	ycaM	1	8	0.457
0.4	0	yadI	1	8	0.583
0.4	0	AG1	1	8	0.468
0.4	0	ptsI	1	8.25	0.474
0.4	0	clcB	1	8.25	0.403
0.4	0	ycaM	1	8.25	0.466
0.4	0	yadI	1	8.25	0.601
0.4	0	AG1	1	8.25	0.477
0.4	0	ptsI	1	8.5	0.49
0.4	0	clcB	1	8.5	0.41
0.4	0	ycaM	1	8.5	0.477
0.4	0	yadI	1	8.5	0.604
0.4	0	AG1	1	8.5	0.492
0.4	0	ptsI	1	8.75	0.496
0.4	0	clcB	1	8.75	0.415
0.4	0	ycaM	1	8.75	0.487
0.4	0	yadI	1	8.75	0.615
0.4	0	AG1	1	8.75	0.504
0.4	0	ptsI	1	9	0.508
0.4	0	clcB	1	9	0.424
0.4	0	ycaM	1	9	0.498
0.4	0	yadI	1	9	0.621
0.4	0	AG1	1	9	0.517
0.4	0	ptsI	1	9.25	0.547
0.4	0	clcB	1	9.25	0.435
0.4	0	ycaM	1	9.25	0.509
0.4	0	yadI	1	9.25	0.631
0.4	0	AG1	1	9.25	0.528
0.4	0	ptsI	1	9.5	0.595
0.4	0	clcB	1	9.5	0.444
0.4	0	ycaM	1	9.5	0.527
0.4	0	yadI	1	9.5	0.638
0.4	0	AG1	1	9.5	0.554
0.4	0	ptsI	1	9.75	0.58
0.4	0	clcB	1	9.75	0.452
0.4	0	ycaM	1	9.75	0.525
0.4	0	yadI	1	9.75	0.663
0.4	0	AG1	1	9.75	0.572
0.4	0	ptsI	1	10	0.61
0.4	0	clcB	1	10	0.465
0.4	0	ycaM	1	10	0.54
0.4	0	yadI	1	10	0.656
0.4	0	AG1	1	10	0.566
0.4	0	ptsI	1	10.25	0.613
0.4	0	clcB	1	10.25	0.469

0.4	0	ycaM	1	10.25	0.548
0.4	0	yadI	1	10.25	0.673
0.4	0	AG1	1	10.25	0.577
0.4	0	ptsI	1	10.5	0.608
0.4	0	clcB	1	10.5	0.475
0.4	0	ycaM	1	10.5	0.566
0.4	0	yadI	1	10.5	0.681
0.4	0	AG1	1	10.5	0.588
0.4	0	ptsI	1	10.75	0.649
0.4	0	clcB	1	10.75	0.493
0.4	0	ycaM	1	10.75	0.563
0.4	0	yadI	1	10.75	0.681
0.4	0	AG1	1	10.75	0.584
0.4	0	ptsI	1	11	0.687
0.4	0	clcB	1	11	0.502
0.4	0	ycaM	1	11	0.579
0.4	0	yadI	1	11	0.681
0.4	0	AG1	1	11	0.631
0.4	0	ptsI	1	11.25	0.693
0.4	0	clcB	1	11.25	0.515
0.4	0	ycaM	1	11.25	0.595
0.4	0	yadI	1	11.25	0.7
0.4	0	AG1	1	11.25	0.636
0.4	0	ptsI	1	11.5	0.703
0.4	0	clcB	1	11.5	0.534
0.4	0	ycaM	1	11.5	0.575
0.4	0	yadI	1	11.5	0.702
0.4	0	AG1	1	11.5	0.663
0.4	0	ptsI	1	11.75	0.733
0.4	0	clcB	1	11.75	0.548
0.4	0	ycaM	1	11.75	0.601
0.4	0	yadI	1	11.75	0.714
0.4	0	AG1	1	11.75	0.71
0.4	0	ptsI	1	12	0.715
0.4	0	clcB	1	12	0.547
0.4	0	ycaM	1	12	0.615
0.4	0	yadI	1	12	0.727
0.4	0	AG1	1	12	0.723
0.4	0	ptsI	1	12.25	0.736
0.4	0	clcB	1	12.25	0.555
0.4	0	ycaM	1	12.25	0.604
0.4	0	yadI	1	12.25	0.721
0.4	0	AG1	1	12.25	0.733
0.4	0	ptsI	1	12.5	0.725
0.4	0	clcB	1	12.5	0.561
0.4	0	ycaM	1	12.5	0.629
0.4	0	yadI	1	12.5	0.728
0.4	0	AG1	1	12.5	0.746
0.4	0	ptsI	1	12.75	0.738
0.4	0	clcB	1	12.75	0.561
0.4	0	ycaM	1	12.75	0.65
0.4	0	yadI	1	12.75	0.732
0.4	0	AG1	1	12.75	0.745

0.4	0	ptsI	1	13	0.745
0.4	0	clcB	1	13	0.561
0.4	0	ycaM	1	13	0.646
0.4	0	yadI	1	13	0.739
0.4	0	AG1	1	13	0.754
0.4	0	ptsI	1	13.25	0.759
0.4	0	clcB	1	13.25	0.574
0.4	0	ycaM	1	13.25	0.614
0.4	0	yadI	1	13.25	0.731
0.4	0	AG1	1	13.25	0.759
0.4	0	ptsI	1	13.5	0.772
0.4	0	clcB	1	13.5	0.58
0.4	0	ycaM	1	13.5	0.617
0.4	0	yadI	1	13.5	0.756
0.4	0	AG1	1	13.5	0.756
0.4	0	ptsI	1	13.75	0.773
0.4	0	clcB	1	13.75	0.578
0.4	0	ycaM	1	13.75	0.617
0.4	0	yadI	1	13.75	0.76
0.4	0	AG1	1	13.75	0.762
0.4	0	ptsI	1	14	0.798
0.4	0	clcB	1	14	0.586
0.4	0	ycaM	1	14	0.64
0.4	0	yadI	1	14	0.77
0.4	0	AG1	1	14	0.747
0.4	0	ptsI	1	14.25	0.805
0.4	0	clcB	1	14.25	0.598
0.4	0	ycaM	1	14.25	0.674
0.4	0	yadI	1	14.25	0.778
0.4	0	AG1	1	14.25	0.764
0.4	0	ptsI	1	14.5	0.8
0.4	0	clcB	1	14.5	0.601
0.4	0	ycaM	1	14.5	0.681
0.4	0	yadI	1	14.5	0.775
0.4	0	AG1	1	14.5	0.777
0.4	0	ptsI	1	14.75	0.806
0.4	0	clcB	1	14.75	0.61
0.4	0	ycaM	1	14.75	0.647
0.4	0	yadI	1	14.75	0.785
0.4	0	AG1	1	14.75	0.788
0.4	0	ptsI	1	15	0.807
0.4	0	clcB	1	15	0.607
0.4	0	ycaM	1	15	0.637
0.4	0	yadI	1	15	0.79
0.4	0	AG1	1	15	0.796
0.4	0	ptsI	1	15.25	0.828
0.4	0	clcB	1	15.25	0.618
0.4	0	ycaM	1	15.25	0.681
0.4	0	yadI	1	15.25	0.798
0.4	0	AG1	1	15.25	0.81
0.4	0	ptsI	1	15.5	0.823
0.4	0	clcB	1	15.5	0.613
0.4	0	ycaM	1	15.5	0.69

0.4	0	yadI	1	15.5	0.805
0.4	0	AG1	1	15.5	0.799
0.4	0	ptsI	1	15.75	0.813
0.4	0	clcB	1	15.75	0.627
0.4	0	ycaM	1	15.75	0.7
0.4	0	yadI	1	15.75	0.804
0.4	0	AG1	1	15.75	0.811
0.4	0	ptsI	1	16	0.824
0.4	0	clcB	1	16	0.619
0.4	0	ycaM	1	16	0.697
0.4	0	yadI	1	16	0.805
0.4	0	AG1	1	16	0.809
0.4	0	ptsI	1	16.25	0.82
0.4	0	clcB	1	16.25	0.628
0.4	0	ycaM	1	16.25	0.722
0.4	0	yadI	1	16.25	0.813
0.4	0	AG1	1	16.25	0.833
0.4	0	ptsI	1	16.5	0.833
0.4	0	clcB	1	16.5	0.641
0.4	0	ycaM	1	16.5	0.733
0.4	0	yadI	1	16.5	0.808
0.4	0	AG1	1	16.5	0.833
0.4	0	ptsI	1	17.0667	0.861
0.4	0	clcB	1	17.0667	0.661
0.4	0	ycaM	1	17.0667	0.752
0.4	0	yadI	1	17.0667	0.864
0.4	0	AG1	1	17.0667	0.866
0.4	0	ptsI	1	17.3167	0.802
0.4	0	clcB	1	17.3167	0.624
0.4	0	ycaM	1	17.3167	0.712
0.4	0	yadI	1	17.3167	0.844
0.4	0	AG1	1	17.3167	0.837
0.4	0	ptsI	1	17.5667	0.806
0.4	0	clcB	1	17.5667	0.643
0.4	0	ycaM	1	17.5667	0.702
0.4	0	yadI	1	17.5667	0.841
0.4	0	AG1	1	17.5667	0.843
0.4	0	ptsI	1	17.8167	0.828
0.4	0	clcB	1	17.8167	0.687
0.4	0	ycaM	1	17.8167	0.716
0.4	0	yadI	1	17.8167	0.851
0.4	0	AG1	1	17.8167	0.808
0.4	0	ptsI	1	18.0667	0.834
0.4	0	clcB	1	18.0667	0.696
0.4	0	ycaM	1	18.0667	0.716
0.4	0	yadI	1	18.0667	0.851
0.4	0	AG1	1	18.0667	0.846
0.4	0	ptsI	1	18.3167	0.838
0.4	0	clcB	1	18.3167	0.676
0.4	0	ycaM	1	18.3167	0.713
0.4	0	yadI	1	18.3167	0.834
0.4	0	AG1	1	18.3167	0.853
0.4	0	ptsI	1	18.5667	0.847

0.4	0	clcB	1	18.5667	0.73
0.4	0	ycaM	1	18.5667	0.718
0.4	0	yadI	1	18.5667	0.844
0.4	0	AG1	1	18.5667	0.848
0.4	0	ptsI	1	18.8167	0.855
0.4	0	clcB	1	18.8167	0.714
0.4	0	ycaM	1	18.8167	0.716
0.4	0	yadI	1	18.8167	0.849
0.4	0	AG1	1	18.8167	0.856
0.4	0	ptsI	1	19.0667	0.862
0.4	0	clcB	1	19.0667	0.684
0.4	0	ycaM	1	19.0667	0.721
0.4	0	yadI	1	19.0667	0.857
0.4	0	AG1	1	19.0667	0.865
0.4	0	ptsI	1	19.3167	0.866
0.4	0	clcB	1	19.3167	0.707
0.4	0	ycaM	1	19.3167	0.723
0.4	0	yadI	1	19.3167	0.853
0.4	0	AG1	1	19.3167	0.859
0.4	0	ptsI	1	19.5667	0.879
0.4	0	clcB	1	19.5667	0.726
0.4	0	ycaM	1	19.5667	0.735
0.4	0	yadI	1	19.5667	0.868
0.4	0	AG1	1	19.5667	0.868
0.4	0	ptsI	1	19.8167	0.883
0.4	0	clcB	1	19.8167	0.721
0.4	0	ycaM	1	19.8167	0.734
0.4	0	yadI	1	19.8167	0.868
0.4	0	AG1	1	19.8167	0.855
0.4	0	ptsI	1	20.0667	0.895
0.4	0	clcB	1	20.0667	0.713
0.4	0	ycaM	1	20.0667	0.742
0.4	0	yadI	1	20.0667	0.872
0.4	0	AG1	1	20.0667	0.863
0.4	0	ptsI	1	20.3167	0.9
0.4	0	clcB	1	20.3167	0.719
0.4	0	ycaM	1	20.3167	0.742
0.4	0	yadI	1	20.3167	0.877
0.4	0	AG1	1	20.3167	0.86
0.4	0	ptsI	1	20.5667	0.902
0.4	0	clcB	1	20.5667	0.718
0.4	0	ycaM	1	20.5667	0.746
0.4	0	yadI	1	20.5667	0.88
0.4	0	AG1	1	20.5667	0.85
0.4	0	ptsI	1	20.8167	0.912
0.4	0	clcB	1	20.8167	0.725
0.4	0	ycaM	1	20.8167	0.749
0.4	0	yadI	1	20.8167	0.886
0.4	0	AG1	1	20.8167	0.837
0.4	0	ptsI	1	21.0667	0.928
0.4	0	clcB	1	21.0667	0.726
0.4	0	ycaM	1	21.0667	0.763
0.4	0	yadI	1	21.0667	0.894

0.4	0	AG1	1	21.0667	0.837
0.4	0	ptsI	1	21.3167	0.933
0.4	0	clcB	1	21.3167	0.725
0.4	0	ycaM	1	21.3167	0.763
0.4	0	yadI	1	21.3167	0.896
0.4	0	AG1	1	21.3167	0.842
0.4	0	ptsI	1	21.5667	0.943
0.4	0	clcB	1	21.5667	0.722
0.4	0	ycaM	1	21.5667	0.771
0.4	0	yadI	1	21.5667	0.896
0.4	0	AG1	1	21.5667	0.842
0.4	0	ptsI	1	21.8167	0.945
0.4	0	clcB	1	21.8167	0.723
0.4	0	ycaM	1	21.8167	0.775
0.4	0	yadI	1	21.8167	0.898
0.4	0	AG1	1	21.8167	0.844
0.4	0	ptsI	1	22.0667	0.963
0.4	0	clcB	1	22.0667	0.728
0.4	0	ycaM	1	22.0667	0.788
0.4	0	yadI	1	22.0667	0.906
0.4	0	AG1	1	22.0667	0.858
0.4	0	ptsI	1	22.9333	1.107
0.4	0	clcB	1	22.9333	0.601
0.4	0	ycaM	1	22.9333	0.534
0.4	0	yadI	1	22.9333	0.895
0.4	0	AG1	1	22.9333	0.798
0.4	0	ptsI	1	23.1833	1.093
0.4	0	clcB	1	23.1833	0.621
0.4	0	ycaM	1	23.1833	0.594
0.4	0	yadI	1	23.1833	0.86
0.4	0	AG1	1	23.1833	0.777
0.4	0	ptsI	1	23.4333	1.078
0.4	0	clcB	1	23.4333	0.603
0.4	0	ycaM	1	23.4333	0.616
0.4	0	yadI	1	23.4333	0.858
0.4	0	AG1	1	23.4333	0.815
0.4	0	ptsI	1	23.6833	1.082
0.4	0	clcB	1	23.6833	0.607
0.4	0	ycaM	1	23.6833	0.63
0.4	0	yadI	1	23.6833	0.855
0.4	0	AG1	1	23.6833	0.83
0.4	0	ptsI	1	23.9333	1.085
0.4	0	clcB	1	23.9333	0.604
0.4	0	ycaM	1	23.9333	0.648
0.4	0	yadI	1	23.9333	0.868
0.4	0	AG1	1	23.9333	0.832
0.4	0	ptsI	1	24.1833	1.087
0.4	0	clcB	1	24.1833	0.644
0.4	0	ycaM	1	24.1833	0.657
0.4	0	yadI	1	24.1833	0.881
0.4	0	AG1	1	24.1833	0.838
0.4	0	ptsI	1	24.4333	1.09
0.4	0	clcB	1	24.4333	0.657

0.4	0	ycaM	1	24.4333	0.669
0.4	0	yadI	1	24.4333	0.887
0.4	0	AG1	1	24.4333	0.829
0.4	0	ptsI	1	24.6833	1.087
0.4	0	clcB	1	24.6833	0.655
0.4	0	ycaM	1	24.6833	0.676
0.4	0	yadI	1	24.6833	0.894
0.4	0	AG1	1	24.6833	0.836
0.4	0	ptsI	1	24.9333	1.093
0.4	0	clcB	1	24.9333	0.654
0.4	0	ycaM	1	24.9333	0.686
0.4	0	yadI	1	24.9333	0.906
0.4	0	AG1	1	24.9333	0.845
0.4	0	ptsI	1	25.1833	1.097
0.4	0	clcB	1	25.1833	0.661
0.4	0	ycaM	1	25.1833	0.702
0.4	0	yadI	1	25.1833	0.915
0.4	0	AG1	1	25.1833	0.843
0.4	0	ptsI	1	25.4333	1.103
0.4	0	clcB	1	25.4333	0.665
0.4	0	ycaM	1	25.4333	0.708
0.4	0	yadI	1	25.4333	0.928
0.4	0	AG1	1	25.4333	0.859
0.4	0	ptsI	1	25.6833	1.109
0.4	0	clcB	1	25.6833	0.673
0.4	0	ycaM	1	25.6833	0.712
0.4	0	yadI	1	25.6833	0.933
0.4	0	AG1	1	25.6833	0.862
0.4	0	ptsI	1	25.9333	1.106
0.4	0	clcB	1	25.9333	0.698
0.4	0	ycaM	1	25.9333	0.722
0.4	0	yadI	1	25.9333	0.927
0.4	0	AG1	1	25.9333	0.872
0.4	0	ptsI	1	26.1833	1.112
0.4	0	clcB	1	26.1833	0.704
0.4	0	ycaM	1	26.1833	0.727
0.4	0	yadI	1	26.1833	0.949
0.4	0	AG1	1	26.1833	0.887
0.4	0	ptsI	1	26.4333	1.114
0.4	0	clcB	1	26.4333	0.703
0.4	0	ycaM	1	26.4333	0.725
0.4	0	yadI	1	26.4333	0.951
0.4	0	AG1	1	26.4333	0.876
0.4	0	ptsI	1	26.6833	1.122
0.4	0	clcB	1	26.6833	0.714
0.4	0	ycaM	1	26.6833	0.73
0.4	0	yadI	1	26.6833	0.954
0.4	0	AG1	1	26.6833	0.88
0.4	0	ptsI	1	26.9333	1.125
0.4	0	clcB	1	26.9333	0.719
0.4	0	ycaM	1	26.9333	0.735
0.4	0	yadI	1	26.9333	0.959
0.4	0	AG1	1	26.9333	0.885

0.4	0	ptsI	1	27.1833	1.128
0.4	0	clcB	1	27.1833	0.738
0.4	0	ycaM	1	27.1833	0.738
0.4	0	yadI	1	27.1833	0.96
0.4	0	AG1	1	27.1833	0.89
0.4	0	ptsI	1	27.4333	1.135
0.4	0	clcB	1	27.4333	0.731
0.4	0	ycaM	1	27.4333	0.73
0.4	0	yadI	1	27.4333	0.965
0.4	0	AG1	1	27.4333	0.882
0.4	0	ptsI	1	27.6833	1.138
0.4	0	clcB	1	27.6833	0.744
0.4	0	ycaM	1	27.6833	0.74
0.4	0	yadI	1	27.6833	0.971
0.4	0	AG1	1	27.6833	0.894
0.4	0	ptsI	1	27.9333	1.145
0.4	0	clcB	1	27.9333	0.741
0.4	0	ycaM	1	27.9333	0.733
0.4	0	yadI	1	27.9333	0.954
0.4	0	AG1	1	27.9333	0.884
0.4	0	ptsI	1	28.1833	1.151
0.4	0	clcB	1	28.1833	0.762
0.4	0	ycaM	1	28.1833	0.747
0.4	0	yadI	1	28.1833	0.977
0.4	0	AG1	1	28.1833	0.902
0.4	0	ptsI	1	28.4333	1.16
0.4	0	clcB	1	28.4333	0.754
0.4	0	ycaM	1	28.4333	0.75
0.4	0	yadI	1	28.4333	0.984
0.4	0	AG1	1	28.4333	0.898
0.4	0	ptsI	1	28.6833	1.164
0.4	0	clcB	1	28.6833	0.76
0.4	0	ycaM	1	28.6833	0.753
0.4	0	yadI	1	28.6833	0.987
0.4	0	AG1	1	28.6833	0.896
0.4	0	ptsI	1	28.9333	1.171
0.4	0	clcB	1	28.9333	0.768
0.4	0	ycaM	1	28.9333	0.764
0.4	0	yadI	1	28.9333	0.989
0.4	0	AG1	1	28.9333	0.904
0.4	0	ptsI	1	29.1833	1.174
0.4	0	clcB	1	29.1833	0.755
0.4	0	ycaM	1	29.1833	0.759
0.4	0	yadI	1	29.1833	0.998
0.4	0	AG1	1	29.1833	0.901
0.4	0	ptsI	1	29.4333	1.18
0.4	0	clcB	1	29.4333	0.768
0.4	0	ycaM	1	29.4333	0.771
0.4	0	yadI	1	29.4333	1
0.4	0	AG1	1	29.4333	0.908
0.4	0	ptsI	1	29.6833	1.187
0.4	0	clcB	1	29.6833	0.766
0.4	0	ycaM	1	29.6833	0.774

0.4	0	yadI	1	29.6833	1.009
0.4	0	AG1	1	29.6833	0.907
0.4	0	ptsI	1	29.9333	1.198
0.4	0	clcB	1	29.9333	0.784
0.4	0	ycaM	1	29.9333	0.775
0.4	0	yadI	1	29.9333	1.015
0.4	0	AG1	1	29.9333	0.911
0.4	0	ptsI	1	30.1833	1.202
0.4	0	clcB	1	30.1833	0.775
0.4	0	ycaM	1	30.1833	0.784
0.4	0	yadI	1	30.1833	1.018
0.4	0	AG1	1	30.1833	0.914
0.4	0	ptsI	1	30.4333	1.205
0.4	0	clcB	1	30.4333	0.773
0.4	0	ycaM	1	30.4333	0.794
0.4	0	yadI	1	30.4333	1.019
0.4	0	AG1	1	30.4333	0.917
0.4	0	ptsI	1	30.6833	1.212
0.4	0	clcB	1	30.6833	0.776
0.4	0	ycaM	1	30.6833	0.783
0.4	0	yadI	1	30.6833	1.035
0.4	0	AG1	1	30.6833	0.913
0.4	0	ptsI	1	30.9333	1.22
0.4	0	clcB	1	30.9333	0.776
0.4	0	ycaM	1	30.9333	0.79
0.4	0	yadI	1	30.9333	1.04
0.4	0	AG1	1	30.9333	0.915
0.4	0	ptsI	1	31.1833	1.232
0.4	0	clcB	1	31.1833	0.78
0.4	0	ycaM	1	31.1833	0.794
0.4	0	yadI	1	31.1833	1.048
0.4	0	AG1	1	31.1833	0.913
0.4	0	ptsI	1	31.4333	1.236
0.4	0	clcB	1	31.4333	0.78
0.4	0	ycaM	1	31.4333	0.81
0.4	0	yadI	1	31.4333	1.051
0.4	0	AG1	1	31.4333	0.921
0.4	0	ptsI	1	31.6833	1.241
0.4	0	clcB	1	31.6833	0.779
0.4	0	ycaM	1	31.6833	0.806
0.4	0	yadI	1	31.6833	1.053
0.4	0	AG1	1	31.6833	0.92
0.4	0	ptsI	1	31.9333	1.248
0.4	0	clcB	1	31.9333	0.779
0.4	0	ycaM	1	31.9333	0.808
0.4	0	yadI	1	31.9333	1.058
0.4	0	AG1	1	31.9333	0.921
0.4	0	ptsI	1	32.1833	1.254
0.4	0	clcB	1	32.1833	0.783
0.4	0	ycaM	1	32.1833	0.81
0.4	0	yadI	1	32.1833	1.062
0.4	0	AG1	1	32.1833	0.924
0.4	0	ptsI	1	32.4333	1.262

0.4	0	clcB	1	32.4333	0.786
0.4	0	ycaM	1	32.4333	0.804
0.4	0	yadI	1	32.4333	1.041
0.4	0	AG1	1	32.4333	0.916
0.4	0	ptsI	1	32.6833	1.266
0.4	0	clcB	1	32.6833	0.781
0.4	0	ycaM	1	32.6833	0.814
0.4	0	yadI	1	32.6833	1.072
0.4	0	AG1	1	32.6833	0.921
0.4	0	ptsI	1	32.9333	1.286
0.4	0	clcB	1	32.9333	0.787
0.4	0	ycaM	1	32.9333	0.813
0.4	0	yadI	1	32.9333	1.079
0.4	0	AG1	1	32.9333	0.92
0.4	0	ptsI	1	33.1833	1.288
0.4	0	clcB	1	33.1833	0.781
0.4	0	ycaM	1	33.1833	0.816
0.4	0	yadI	1	33.1833	1.066
0.4	0	AG1	1	33.1833	0.916
0.4	0	ptsI	1	33.4333	1.301
0.4	0	clcB	1	33.4333	0.786
0.4	0	ycaM	1	33.4333	0.832
0.4	0	yadI	1	33.4333	1.094
0.4	0	AG1	1	33.4333	0.926
0.4	0	ptsI	1	33.6833	1.302
0.4	0	clcB	1	33.6833	0.782
0.4	0	ycaM	1	33.6833	0.828
0.4	0	yadI	1	33.6833	1.084
0.4	0	AG1	1	33.6833	0.92
0.4	0	ptsI	1	33.9333	1.303
0.4	0	clcB	1	33.9333	0.787
0.4	0	ycaM	1	33.9333	0.829
0.4	0	yadI	1	33.9333	1.105
0.4	0	AG1	1	33.9333	0.927
0.4	0	ptsI	1	34.1833	1.313
0.4	0	clcB	1	34.1833	0.779
0.4	0	ycaM	1	34.1833	0.836
0.4	0	yadI	1	34.1833	1.117
0.4	0	AG1	1	34.1833	0.921
0.4	0	ptsI	1	34.4333	1.32
0.4	0	clcB	1	34.4333	0.793
0.4	0	ycaM	1	34.4333	0.835
0.4	0	yadI	1	34.4333	1.116
0.4	0	AG1	1	34.4333	0.93
0.4	0	ptsI	1	34.6833	1.332
0.4	0	clcB	1	34.6833	0.796
0.4	0	ycaM	1	34.6833	0.845
0.4	0	yadI	1	34.6833	1.129
0.4	0	AG1	1	34.6833	0.935
0.4	0	ptsI	1	34.9333	1.341
0.4	0	clcB	1	34.9333	0.805
0.4	0	ycaM	1	34.9333	0.842
0.4	0	yadI	1	34.9333	1.128

0.4	0	AG1	1	34.9333	0.935
0.4	0	ptsI	1	35.1833	1.349
0.4	0	clcB	1	35.1833	0.805
0.4	0	ycaM	1	35.1833	0.845
0.4	0	yadI	1	35.1833	1.135
0.4	0	AG1	1	35.1833	0.936
0.4	0	ptsI	1	35.4333	1.358
0.4	0	clcB	1	35.4333	0.815
0.4	0	ycaM	1	35.4333	0.846
0.4	0	yadI	1	35.4333	1.147
0.4	0	AG1	1	35.4333	0.936
0.4	0	ptsI	1	35.6833	1.363
0.4	0	clcB	1	35.6833	0.817
0.4	0	ycaM	1	35.6833	0.852
0.4	0	yadI	1	35.6833	1.152
0.4	0	AG1	1	35.6833	0.942
0.4	0	ptsI	1	35.9333	1.374
0.4	0	clcB	1	35.9333	0.824
0.4	0	ycaM	1	35.9333	0.85
0.4	0	yadI	1	35.9333	1.141
0.4	0	AG1	1	35.9333	0.939
0.4	0	ptsI	1	36.1833	1.372
0.4	0	clcB	1	36.1833	0.83
0.4	0	ycaM	1	36.1833	0.858
0.4	0	yadI	1	36.1833	1.167
0.4	0	AG1	1	36.1833	0.947
0.4	0	ptsI	1	36.4333	1.385
0.4	0	clcB	1	36.4333	0.845
0.4	0	ycaM	1	36.4333	0.86
0.4	0	yadI	1	36.4333	1.173
0.4	0	AG1	1	36.4333	0.95
0.4	0	ptsI	1	36.6833	1.394
0.4	0	clcB	1	36.6833	0.854
0.4	0	ycaM	1	36.6833	0.861
0.4	0	yadI	1	36.6833	1.184
0.4	0	AG1	1	36.6833	0.956
0.4	0	ptsI	2	0	0.212
0.4	0	clcB	2	0	0.205
0.4	0	ycaM	2	0	0.212
0.4	0	yadI	2	0	0.232
0.4	0	AG1	2	0	0.244
0.4	0	ptsI	2	0.25	0.214
0.4	0	clcB	2	0.25	0.203
0.4	0	ycaM	2	0.25	0.207
0.4	0	yadI	2	0.25	0.226
0.4	0	AG1	2	0.25	0.234
0.4	0	ptsI	2	0.5	0.212
0.4	0	clcB	2	0.5	0.201
0.4	0	ycaM	2	0.5	0.206
0.4	0	yadI	2	0.5	0.224
0.4	0	AG1	2	0.5	0.232
0.4	0	ptsI	2	0.75	0.215
0.4	0	clcB	2	0.75	0.203

0.4	0	ycaM	2	0.75	0.207
0.4	0	yadI	2	0.75	0.227
0.4	0	AG1	2	0.75	0.236
0.4	0	ptsI	2	1	0.215
0.4	0	clcB	2	1	0.207
0.4	0	ycaM	2	1	0.213
0.4	0	yadI	2	1	0.234
0.4	0	AG1	2	1	0.245
0.4	0	ptsI	2	1.25	0.22
0.4	0	clcB	2	1.25	0.205
0.4	0	ycaM	2	1.25	0.215
0.4	0	yadI	2	1.25	0.241
0.4	0	AG1	2	1.25	0.251
0.4	0	ptsI	2	1.5	0.223
0.4	0	clcB	2	1.5	0.208
0.4	0	ycaM	2	1.5	0.218
0.4	0	yadI	2	1.5	0.247
0.4	0	AG1	2	1.5	0.256
0.4	0	ptsI	2	1.75	0.226
0.4	0	clcB	2	1.75	0.211
0.4	0	ycaM	2	1.75	0.222
0.4	0	yadI	2	1.75	0.253
0.4	0	AG1	2	1.75	0.264
0.4	0	ptsI	2	2	0.23
0.4	0	clcB	2	2	0.215
0.4	0	ycaM	2	2	0.229
0.4	0	yadI	2	2	0.261
0.4	0	AG1	2	2	0.279
0.4	0	ptsI	2	2.25	0.241
0.4	0	clcB	2	2.25	0.219
0.4	0	ycaM	2	2.25	0.232
0.4	0	yadI	2	2.25	0.274
0.4	0	AG1	2	2.25	0.292
0.4	0	ptsI	2	2.5	0.248
0.4	0	clcB	2	2.5	0.226
0.4	0	ycaM	2	2.5	0.24
0.4	0	yadI	2	2.5	0.289
0.4	0	AG1	2	2.5	0.307
0.4	0	ptsI	2	2.75	0.252
0.4	0	clcB	2	2.75	0.231
0.4	0	ycaM	2	2.75	0.246
0.4	0	yadI	2	2.75	0.298
0.4	0	AG1	2	2.75	0.322
0.4	0	ptsI	2	3	0.258
0.4	0	clcB	2	3	0.238
0.4	0	ycaM	2	3	0.254
0.4	0	yadI	2	3	0.309
0.4	0	AG1	2	3	0.321
0.4	0	ptsI	2	3.25	0.27
0.4	0	clcB	2	3.25	0.242
0.4	0	ycaM	2	3.25	0.263
0.4	0	yadI	2	3.25	0.332
0.4	0	AG1	2	3.25	0.345

0.4	0	ptsI	2	3.5	0.277
0.4	0	clcB	2	3.5	0.248
0.4	0	ycaM	2	3.5	0.271
0.4	0	yadI	2	3.5	0.349
0.4	0	AG1	2	3.5	0.357
0.4	0	ptsI	2	3.75	0.285
0.4	0	clcB	2	3.75	0.253
0.4	0	ycaM	2	3.75	0.285
0.4	0	yadI	2	3.75	0.367
0.4	0	AG1	2	3.75	0.368
0.4	0	ptsI	2	4	0.292
0.4	0	clcB	2	4	0.259
0.4	0	ycaM	2	4	0.292
0.4	0	yadI	2	4	0.386
0.4	0	AG1	2	4	0.379
0.4	0	ptsI	2	4.38333	0.309
0.4	0	clcB	2	4.38333	0.269
0.4	0	ycaM	2	4.38333	0.306
0.4	0	yadI	2	4.38333	0.405
0.4	0	AG1	2	4.38333	0.44
0.4	0	ptsI	2	4.63333	0.303
0.4	0	clcB	2	4.63333	0.265
0.4	0	ycaM	2	4.63333	0.3
0.4	0	yadI	2	4.63333	0.402
0.4	0	AG1	2	4.63333	0.418
0.4	0	ptsI	2	4.88333	0.307
0.4	0	clcB	2	4.88333	0.269
0.4	0	ycaM	2	4.88333	0.307
0.4	0	yadI	2	4.88333	0.412
0.4	0	AG1	2	4.88333	0.426
0.4	0	ptsI	2	5.13333	0.315
0.4	0	clcB	2	5.13333	0.275
0.4	0	ycaM	2	5.13333	0.316
0.4	0	yadI	2	5.13333	0.423
0.4	0	AG1	2	5.13333	0.436
0.4	0	ptsI	2	5.38333	0.323
0.4	0	clcB	2	5.38333	0.283
0.4	0	ycaM	2	5.38333	0.342
0.4	0	yadI	2	5.38333	0.436
0.4	0	AG1	2	5.38333	0.443
0.4	0	ptsI	2	5.63333	0.332
0.4	0	clcB	2	5.63333	0.293
0.4	0	ycaM	2	5.63333	0.338
0.4	0	yadI	2	5.63333	0.446
0.4	0	AG1	2	5.63333	0.454
0.4	0	ptsI	2	5.88333	0.337
0.4	0	clcB	2	5.88333	0.301
0.4	0	ycaM	2	5.88333	0.35
0.4	0	yadI	2	5.88333	0.455
0.4	0	AG1	2	5.88333	0.465
0.4	0	ptsI	2	6.13333	0.345
0.4	0	clcB	2	6.13333	0.306
0.4	0	ycaM	2	6.13333	0.359

0.4	0	yadI	2	6.13333	0.465
0.4	0	AG1	2	6.13333	0.478
0.4	0	ptsI	2	6.38333	0.354
0.4	0	clcB	2	6.38333	0.313
0.4	0	ycaM	2	6.38333	0.372
0.4	0	yadI	2	6.38333	0.476
0.4	0	AG1	2	6.38333	0.49
0.4	0	ptsI	2	6.63333	0.361
0.4	0	clcB	2	6.63333	0.319
0.4	0	ycaM	2	6.63333	0.381
0.4	0	yadI	2	6.63333	0.487
0.4	0	AG1	2	6.63333	0.5
0.4	0	ptsI	2	6.88333	0.37
0.4	0	clcB	2	6.88333	0.329
0.4	0	ycaM	2	6.88333	0.391
0.4	0	yadI	2	6.88333	0.497
0.4	0	AG1	2	6.88333	0.51
0.4	0	ptsI	2	7.13333	0.378
0.4	0	clcB	2	7.13333	0.334
0.4	0	ycaM	2	7.13333	0.399
0.4	0	yadI	2	7.13333	0.506
0.4	0	AG1	2	7.13333	0.524
0.4	0	ptsI	2	7.38333	0.385
0.4	0	clcB	2	7.38333	0.343
0.4	0	ycaM	2	7.38333	0.407
0.4	0	yadI	2	7.38333	0.516
0.4	0	AG1	2	7.38333	0.532
0.4	0	ptsI	2	7.63333	0.393
0.4	0	clcB	2	7.63333	0.351
0.4	0	ycaM	2	7.63333	0.417
0.4	0	yadI	2	7.63333	0.527
0.4	0	AG1	2	7.63333	0.546
0.4	0	ptsI	2	7.88333	0.4
0.4	0	clcB	2	7.88333	0.36
0.4	0	ycaM	2	7.88333	0.423
0.4	0	yadI	2	7.88333	0.542
0.4	0	AG1	2	7.88333	0.56
0.4	0	ptsI	2	8.13333	0.409
0.4	0	clcB	2	8.13333	0.367
0.4	0	ycaM	2	8.13333	0.431
0.4	0	yadI	2	8.13333	0.55
0.4	0	AG1	2	8.13333	0.569
0.4	0	ptsI	2	8.38333	0.412
0.4	0	clcB	2	8.38333	0.376
0.4	0	ycaM	2	8.38333	0.436
0.4	0	yadI	2	8.38333	0.562
0.4	0	AG1	2	8.38333	0.582
0.4	0	ptsI	2	8.91667	0.354
0.4	0	clcB	2	8.91667	0.323
0.4	0	ycaM	2	8.91667	0.356
0.4	0	yadI	2	8.91667	0.652
0.4	0	AG1	2	8.91667	0.643
0.4	0	ptsI	2	9.16667	0.352

0.4	0	clcB	2	9.16667	0.332
0.4	0	ycaM	2	9.16667	0.356
0.4	0	yadI	2	9.16667	0.652
0.4	0	AG1	2	9.16667	0.636
0.4	0	ptsI	2	9.41667	0.356
0.4	0	clcB	2	9.41667	0.332
0.4	0	ycaM	2	9.41667	0.36
0.4	0	yadI	2	9.41667	0.659
0.4	0	AG1	2	9.41667	0.65
0.4	0	ptsI	2	9.66667	0.367
0.4	0	clcB	2	9.66667	0.338
0.4	0	ycaM	2	9.66667	0.365
0.4	0	yadI	2	9.66667	0.672
0.4	0	AG1	2	9.66667	0.66
0.4	0	ptsI	2	9.91667	0.372
0.4	0	clcB	2	9.91667	0.392
0.4	0	ycaM	2	9.91667	0.37
0.4	0	yadI	2	9.91667	0.685
0.4	0	AG1	2	9.91667	0.656
0.4	0	ptsI	2	10.1667	0.377
0.4	0	clcB	2	10.1667	0.346
0.4	0	ycaM	2	10.1667	0.374
0.4	0	yadI	2	10.1667	0.696
0.4	0	AG1	2	10.1667	0.648
0.4	0	ptsI	2	10.4167	0.385
0.4	0	clcB	2	10.4167	0.352
0.4	0	ycaM	2	10.4167	0.38
0.4	0	yadI	2	10.4167	0.708
0.4	0	AG1	2	10.4167	0.674
0.4	0	ptsI	2	10.6667	0.392
0.4	0	clcB	2	10.6667	0.358
0.4	0	ycaM	2	10.6667	0.391
0.4	0	yadI	2	10.6667	0.724
0.4	0	AG1	2	10.6667	0.65
0.4	0	ptsI	2	10.9167	0.399
0.4	0	clcB	2	10.9167	0.36
0.4	0	ycaM	2	10.9167	0.397
0.4	0	yadI	2	10.9167	0.733
0.4	0	AG1	2	10.9167	0.664
0.4	0	ptsI	2	11.1667	0.405
0.4	0	clcB	2	11.1667	0.366
0.4	0	ycaM	2	11.1667	0.406
0.4	0	yadI	2	11.1667	0.742
0.4	0	AG1	2	11.1667	0.672
0.4	0	ptsI	2	11.4167	0.425
0.4	0	clcB	2	11.4167	0.368
0.4	0	ycaM	2	11.4167	0.41
0.4	0	yadI	2	11.4167	0.752
0.4	0	AG1	2	11.4167	0.674
0.4	0	ptsI	2	11.6667	0.431
0.4	0	clcB	2	11.6667	0.378
0.4	0	ycaM	2	11.6667	0.416
0.4	0	yadI	2	11.6667	0.764

0.4	0	AG1	2	11.6667	0.696
0.4	0	ptsI	2	11.9167	0.46
0.4	0	clcB	2	11.9167	0.386
0.4	0	ycaM	2	11.9167	0.425
0.4	0	yadI	2	11.9167	0.773
0.4	0	AG1	2	11.9167	0.699
0.4	0	ptsI	2	12.1667	0.45
0.4	0	clcB	2	12.1667	0.397
0.4	0	ycaM	2	12.1667	0.421
0.4	0	yadI	2	12.1667	0.784
0.4	0	AG1	2	12.1667	0.718
0.4	0	ptsI	2	12.4167	0.452
0.4	0	clcB	2	12.4167	0.401
0.4	0	ycaM	2	12.4167	0.432
0.4	0	yadI	2	12.4167	0.791
0.4	0	AG1	2	12.4167	0.711
0.4	0	ptsI	2	12.6667	0.464
0.4	0	clcB	2	12.6667	0.407
0.4	0	ycaM	2	12.6667	0.429
0.4	0	yadI	2	12.6667	0.794
0.4	0	AG1	2	12.6667	0.719
0.4	0	ptsI	2	12.9167	0.477
0.4	0	clcB	2	12.9167	0.421
0.4	0	ycaM	2	12.9167	0.438
0.4	0	yadI	2	12.9167	0.806
0.4	0	AG1	2	12.9167	0.724
0.4	0	ptsI	2	13.1667	0.476
0.4	0	clcB	2	13.1667	0.417
0.4	0	ycaM	2	13.1667	0.455
0.4	0	yadI	2	13.1667	0.814
0.4	0	AG1	2	13.1667	0.733
0.4	0	ptsI	2	13.4167	0.478
0.4	0	clcB	2	13.4167	0.421
0.4	0	ycaM	2	13.4167	0.458
0.4	0	yadI	2	13.4167	0.821
0.4	0	AG1	2	13.4167	0.746
0.4	0	ptsI	2	13.6667	0.489
0.4	0	clcB	2	13.6667	0.427
0.4	0	ycaM	2	13.6667	0.468
0.4	0	yadI	2	13.6667	0.83
0.4	0	AG1	2	13.6667	0.756
0.4	0	ptsI	2	13.9167	0.524
0.4	0	clcB	2	13.9167	0.434
0.4	0	ycaM	2	13.9167	0.461
0.4	0	yadI	2	13.9167	0.839
0.4	0	AG1	2	13.9167	0.779
0.4	0	ptsI	2	14.1667	0.546
0.4	0	clcB	2	14.1667	0.432
0.4	0	ycaM	2	14.1667	0.468
0.4	0	yadI	2	14.1667	0.846
0.4	0	AG1	2	14.1667	0.792
0.4	0	ptsI	2	14.4167	0.571
0.4	0	clcB	2	14.4167	0.438

0.4	0	ycaM	2	14.4167	0.469
0.4	0	yadI	2	14.4167	0.843
0.4	0	AG1	2	14.4167	0.801
0.4	0	ptsI	2	14.6667	0.594
0.4	0	clcB	2	14.6667	0.44
0.4	0	ycaM	2	14.6667	0.467
0.4	0	yadI	2	14.6667	0.847
0.4	0	AG1	2	14.6667	0.797
0.4	0	ptsI	2	14.9167	0.62
0.4	0	clcB	2	14.9167	0.448
0.4	0	ycaM	2	14.9167	0.468
0.4	0	yadI	2	14.9167	0.85
0.4	0	AG1	2	14.9167	0.79
0.4	0	ptsI	2	15.1667	0.614
0.4	0	clcB	2	15.1667	0.46
0.4	0	ycaM	2	15.1667	0.472
0.4	0	yadI	2	15.1667	0.856
0.4	0	AG1	2	15.1667	0.777
0.4	0	ptsI	2	15.4167	0.673
0.4	0	clcB	2	15.4167	0.457
0.4	0	ycaM	2	15.4167	0.485
0.4	0	yadI	2	15.4167	0.865
0.4	0	AG1	2	15.4167	0.803
0.4	0	ptsI	2	15.6667	0.678
0.4	0	clcB	2	15.6667	0.465
0.4	0	ycaM	2	15.6667	0.482
0.4	0	yadI	2	15.6667	0.862
0.4	0	AG1	2	15.6667	0.802
0.4	0	ptsI	2	15.9167	0.68
0.4	0	clcB	2	15.9167	0.477
0.4	0	ycaM	2	15.9167	0.49
0.4	0	yadI	2	15.9167	0.868
0.4	0	AG1	2	15.9167	0.779
0.4	0	ptsI	2	16.1667	0.696
0.4	0	clcB	2	16.1667	0.47
0.4	0	ycaM	2	16.1667	0.499
0.4	0	yadI	2	16.1667	0.873
0.4	0	AG1	2	16.1667	0.78
0.4	0	ptsI	2	16.4167	0.718
0.4	0	clcB	2	16.4167	0.49
0.4	0	ycaM	2	16.4167	0.495
0.4	0	yadI	2	16.4167	0.877
0.4	0	AG1	2	16.4167	0.804
0.4	0	ptsI	2	16.6667	0.731
0.4	0	clcB	2	16.6667	0.492
0.4	0	ycaM	2	16.6667	0.508
0.4	0	yadI	2	16.6667	0.882
0.4	0	AG1	2	16.6667	0.806
0.4	0	ptsI	2	16.9167	0.747
0.4	0	clcB	2	16.9167	0.48
0.4	0	ycaM	2	16.9167	0.525
0.4	0	yadI	2	16.9167	0.882
0.4	0	AG1	2	16.9167	0.808

0.4	0	ptsI	2	17.1667	0.754
0.4	0	clcB	2	17.1667	0.482
0.4	0	ycaM	2	17.1667	0.526
0.4	0	yadI	2	17.1667	0.885
0.4	0	AG1	2	17.1667	0.802
0.4	0	ptsI	2	17.4167	0.759
0.4	0	clcB	2	17.4167	0.508
0.4	0	ycaM	2	17.4167	0.535
0.4	0	yadI	2	17.4167	0.887
0.4	0	AG1	2	17.4167	0.81
0.4	0	ptsI	2	17.6667	0.769
0.4	0	clcB	2	17.6667	0.507
0.4	0	ycaM	2	17.6667	0.531
0.4	0	yadI	2	17.6667	0.886
0.4	0	AG1	2	17.6667	0.818
0.4	0	ptsI	2	17.9167	0.773
0.4	0	clcB	2	17.9167	0.502
0.4	0	ycaM	2	17.9167	0.521
0.4	0	yadI	2	17.9167	0.89
0.4	0	AG1	2	17.9167	0.814
0.4	0	ptsI	2	18.1667	0.786
0.4	0	clcB	2	18.1667	0.501
0.4	0	ycaM	2	18.1667	0.528
0.4	0	yadI	2	18.1667	0.891
0.4	0	AG1	2	18.1667	0.824
0.4	0	ptsI	2	18.4167	0.792
0.4	0	clcB	2	18.4167	0.495
0.4	0	ycaM	2	18.4167	0.52
0.4	0	yadI	2	18.4167	0.893
0.4	0	AG1	2	18.4167	0.822
0.4	0	ptsI	2	18.6667	0.787
0.4	0	clcB	2	18.6667	0.498
0.4	0	ycaM	2	18.6667	0.526
0.4	0	yadI	2	18.6667	0.894
0.4	0	AG1	2	18.6667	0.821
0.4	0	ptsI	2	18.9167	0.813
0.4	0	clcB	2	18.9167	0.488
0.4	0	ycaM	2	18.9167	0.522
0.4	0	yadI	2	18.9167	0.875
0.4	0	AG1	2	18.9167	0.826
0.4	0	ptsI	2	19.1667	0.805
0.4	0	clcB	2	19.1667	0.49
0.4	0	ycaM	2	19.1667	0.522
0.4	0	yadI	2	19.1667	0.897
0.4	0	AG1	2	19.1667	0.816
0.4	0	ptsI	2	19.4167	0.81
0.4	0	clcB	2	19.4167	0.49
0.4	0	ycaM	2	19.4167	0.522
0.4	0	yadI	2	19.4167	0.896
0.4	0	AG1	2	19.4167	0.818
0.4	0	ptsI	2	19.6667	0.813
0.4	0	clcB	2	19.6667	0.488
0.4	0	ycaM	2	19.6667	0.524

0.4	0	yadI	2	19.6667	0.897
0.4	0	AG1	2	19.6667	0.817
0.4	0	ptsI	2	19.9167	0.826
0.4	0	clcB	2	19.9167	0.5
0.4	0	ycaM	2	19.9167	0.527
0.4	0	yadI	2	19.9167	0.896
0.4	0	AG1	2	19.9167	0.818
0.4	0	ptsI	2	20.1667	0.825
0.4	0	clcB	2	20.1667	0.495
0.4	0	ycaM	2	20.1667	0.529
0.4	0	yadI	2	20.1667	0.901
0.4	0	AG1	2	20.1667	0.815
0.4	0	ptsI	2	20.4167	0.83
0.4	0	clcB	2	20.4167	0.498
0.4	0	ycaM	2	20.4167	0.534
0.4	0	yadI	2	20.4167	0.903
0.4	0	AG1	2	20.4167	0.824
0.4	0	ptsI	2	20.6667	0.839
0.4	0	clcB	2	20.6667	0.494
0.4	0	ycaM	2	20.6667	0.535
0.4	0	yadI	2	20.6667	0.901
0.4	0	AG1	2	20.6667	0.82
0.4	0	ptsI	2	20.9167	0.843
0.4	0	clcB	2	20.9167	0.491
0.4	0	ycaM	2	20.9167	0.538
0.4	0	yadI	2	20.9167	0.905
0.4	0	AG1	2	20.9167	0.824
0.4	0	ptsI	2	21.1667	0.835
0.4	0	clcB	2	21.1667	0.492
0.4	0	ycaM	2	21.1667	0.539
0.4	0	yadI	2	21.1667	0.907
0.4	0	AG1	2	21.1667	0.823
0.4	0	ptsI	2	21.4167	0.841
0.4	0	clcB	2	21.4167	0.494
0.4	0	ycaM	2	21.4167	0.545
0.4	0	yadI	2	21.4167	0.891
0.4	0	AG1	2	21.4167	0.828
0.4	0	ptsI	2	21.6667	0.847
0.4	0	clcB	2	21.6667	0.498
0.4	0	ycaM	2	21.6667	0.545
0.4	0	yadI	2	21.6667	0.907
0.4	0	AG1	2	21.6667	0.83
0.4	0	ptsI	2	21.9167	0.852
0.4	0	clcB	2	21.9167	0.5
0.4	0	ycaM	2	21.9167	0.547
0.4	0	yadI	2	21.9167	0.909
0.4	0	AG1	2	21.9167	0.834
0.4	0	ptsI	2	22.1667	0.861
0.4	0	clcB	2	22.1667	0.502
0.4	0	ycaM	2	22.1667	0.549
0.4	0	yadI	2	22.1667	0.909
0.4	0	AG1	2	22.1667	0.835
0.4	0	ptsI	2	22.4167	0.859

0.4	0	clcB	2	22.4167	0.508
0.4	0	ycaM	2	22.4167	0.55
0.4	0	yadI	2	22.4167	0.912
0.4	0	AG1	2	22.4167	0.837
0.4	0	ptsI	2	22.6667	0.86
0.4	0	clcB	2	22.6667	0.507
0.4	0	ycaM	2	22.6667	0.554
0.4	0	yadI	2	22.6667	0.912
0.4	0	AG1	2	22.6667	0.841
0.4	0	ptsI	2	22.9167	0.86
0.4	0	clcB	2	22.9167	0.512
0.4	0	ycaM	2	22.9167	0.553
0.4	0	yadI	2	22.9167	0.917
0.4	0	AG1	2	22.9167	0.845
0.4	0	ptsI	2	23.1667	0.866
0.4	0	clcB	2	23.1667	0.511
0.4	0	ycaM	2	23.1667	0.554
0.4	0	yadI	2	23.1667	0.916
0.4	0	AG1	2	23.1667	0.846
0.4	0	ptsI	2	23.4167	0.868
0.4	0	clcB	2	23.4167	0.511
0.4	0	ycaM	2	23.4167	0.554
0.4	0	yadI	2	23.4167	0.916
0.4	0	AG1	2	23.4167	0.848
0.4	0	ptsI	2	23.6667	0.874
0.4	0	clcB	2	23.6667	0.514
0.4	0	ycaM	2	23.6667	0.561
0.4	0	yadI	2	23.6667	0.916
0.4	0	AG1	2	23.6667	0.852
0.4	0	ptsI	2	23.9167	0.879
0.4	0	clcB	2	23.9167	0.52
0.4	0	ycaM	2	23.9167	0.561
0.4	0	yadI	2	23.9167	0.919
0.4	0	AG1	2	23.9167	0.858
0.4	0	ptsI	2	24.1667	0.88
0.4	0	clcB	2	24.1667	0.518
0.4	0	ycaM	2	24.1667	0.56
0.4	0	yadI	2	24.1667	0.919
0.4	0	AG1	2	24.1667	0.854
0.4	0	ptsI	2	24.4167	0.884
0.4	0	clcB	2	24.4167	0.517
0.4	0	ycaM	2	24.4167	0.563
0.4	0	yadI	2	24.4167	0.92
0.4	0	AG1	2	24.4167	0.854
0.4	0	ptsI	2	24.6667	0.893
0.4	0	clcB	2	24.6667	0.52
0.4	0	ycaM	2	24.6667	0.564
0.4	0	yadI	2	24.6667	0.924
0.4	0	AG1	2	24.6667	0.849
0.4	0	ptsI	2	24.9167	0.896
0.4	0	clcB	2	24.9167	0.523
0.4	0	ycaM	2	24.9167	0.563
0.4	0	yadI	2	24.9167	0.922

0.4	0	AG1	2	24.9167	0.835
0.4	0	ptsI	2	25.1667	0.896
0.4	0	clcB	2	25.1667	0.521
0.4	0	ycaM	2	25.1667	0.565
0.4	0	yadI	2	25.1667	0.927
0.4	0	AG1	2	25.1667	0.825
0.4	0	ptsI	2	25.4167	0.906
0.4	0	clcB	2	25.4167	0.524
0.4	0	ycaM	2	25.4167	0.567
0.4	0	yadI	2	25.4167	0.925
0.4	0	AG1	2	25.4167	0.839
0.4	0	ptsI	2	25.6667	0.909
0.4	0	clcB	2	25.6667	0.527
0.4	0	ycaM	2	25.6667	0.565
0.4	0	yadI	2	25.6667	0.925
0.4	0	AG1	2	25.6667	0.826
0.4	0	ptsI	2	25.9167	0.914
0.4	0	clcB	2	25.9167	0.529
0.4	0	ycaM	2	25.9167	0.569
0.4	0	yadI	2	25.9167	0.927
0.4	0	AG1	2	25.9167	0.827
0.4	0	ptsI	2	26.1667	0.92
0.4	0	clcB	2	26.1667	0.527
0.4	0	ycaM	2	26.1667	0.571
0.4	0	yadI	2	26.1667	0.917
0.4	0	AG1	2	26.1667	0.84
0.4	0	ptsI	2	26.4167	0.933
0.4	0	clcB	2	26.4167	0.533
0.4	0	ycaM	2	26.4167	0.572
0.4	0	yadI	2	26.4167	0.931
0.4	0	AG1	2	26.4167	0.829
0.4	0	ptsI	3	0	0.149
0.4	0	clcB	3	0	0.157
0.4	0	ycaM	3	0	0.157
0.4	0	yadI	3	0	0.352
0.4	0	AG1	3	0	0.168
0.4	0	ptsI	3	0.25	0.147
0.4	0	clcB	3	0.25	0.158
0.4	0	ycaM	3	0.25	0.149
0.4	0	yadI	3	0.25	0.341
0.4	0	AG1	3	0.25	0.163
0.4	0	ptsI	3	0.5	0.147
0.4	0	clcB	3	0.5	0.158
0.4	0	ycaM	3	0.5	0.148
0.4	0	yadI	3	0.5	0.381
0.4	0	AG1	3	0.5	0.161
0.4	0	ptsI	3	0.75	0.149
0.4	0	clcB	3	0.75	0.158
0.4	0	ycaM	3	0.75	0.149
0.4	0	yadI	3	0.75	0.359
0.4	0	AG1	3	0.75	0.161
0.4	0	ptsI	3	1	0.15
0.4	0	clcB	3	1	0.157

0.4	0	ycaM	3	1	0.148
0.4	0	yadI	3	1	0.354
0.4	0	AG1	3	1	0.163
0.4	0	ptsI	3	1.25	0.15
0.4	0	clcB	3	1.25	0.157
0.4	0	ycaM	3	1.25	0.148
0.4	0	yadI	3	1.25	0.371
0.4	0	AG1	3	1.25	0.162
0.4	0	ptsI	3	1.5	0.152
0.4	0	clcB	3	1.5	0.158
0.4	0	ycaM	3	1.5	0.149
0.4	0	yadI	3	1.5	0.358
0.4	0	AG1	3	1.5	0.165
0.4	0	ptsI	3	1.75	0.153
0.4	0	clcB	3	1.75	0.158
0.4	0	ycaM	3	1.75	0.149
0.4	0	yadI	3	1.75	0.377
0.4	0	AG1	3	1.75	0.164
0.4	0	ptsI	3	2	0.155
0.4	0	clcB	3	2	0.159
0.4	0	ycaM	3	2	0.151
0.4	0	yadI	3	2	0.354
0.4	0	AG1	3	2	0.167
0.4	0	ptsI	3	2.25	0.159
0.4	0	clcB	3	2.25	0.16
0.4	0	ycaM	3	2.25	0.152
0.4	0	yadI	3	2.25	0.343
0.4	0	AG1	3	2.25	0.169
0.4	0	ptsI	3	2.5	0.162
0.4	0	clcB	3	2.5	0.161
0.4	0	ycaM	3	2.5	0.153
0.4	0	yadI	3	2.5	0.364
0.4	0	AG1	3	2.5	0.171
0.4	0	ptsI	3	2.75	0.166
0.4	0	clcB	3	2.75	0.162
0.4	0	ycaM	3	2.75	0.158
0.4	0	yadI	3	2.75	0.363
0.4	0	AG1	3	2.75	0.179
0.4	0	ptsI	3	3	0.168
0.4	0	clcB	3	3	0.162
0.4	0	ycaM	3	3	0.157
0.4	0	yadI	3	3	0.364
0.4	0	AG1	3	3	0.175
0.4	0	ptsI	3	3.25	0.171
0.4	0	clcB	3	3.25	0.164
0.4	0	ycaM	3	3.25	0.158
0.4	0	yadI	3	3.25	0.374
0.4	0	AG1	3	3.25	0.179
0.4	0	ptsI	3	3.5	0.175
0.4	0	clcB	3	3.5	0.165
0.4	0	ycaM	3	3.5	0.163
0.4	0	yadI	3	3.5	0.365
0.4	0	AG1	3	3.5	0.186

0.4	0	ptsI	3	3.75	0.18
0.4	0	clcB	3	3.75	0.166
0.4	0	ycaM	3	3.75	0.166
0.4	0	yadI	3	3.75	0.353
0.4	0	AG1	3	3.75	0.189
0.4	0	ptsI	3	4	0.182
0.4	0	clcB	3	4	0.169
0.4	0	ycaM	3	4	0.169
0.4	0	yadI	3	4	0.365
0.4	0	AG1	3	4	0.195
0.4	0	ptsI	3	4.41667	0.189
0.4	0	clcB	3	4.41667	0.171
0.4	0	ycaM	3	4.41667	0.188
0.4	0	yadI	3	4.41667	0.606
0.4	0	AG1	3	4.41667	0.214
0.4	0	ptsI	3	4.66667	0.186
0.4	0	clcB	3	4.66667	0.171
0.4	0	ycaM	3	4.66667	0.184
0.4	0	yadI	3	4.66667	0.526
0.4	0	AG1	3	4.66667	0.207
0.4	0	ptsI	3	4.91667	0.186
0.4	0	clcB	3	4.91667	0.17
0.4	0	ycaM	3	4.91667	0.184
0.4	0	yadI	3	4.91667	0.399
0.4	0	AG1	3	4.91667	0.211
0.4	0	ptsI	3	5.16667	0.189
0.4	0	clcB	3	5.16667	0.172
0.4	0	ycaM	3	5.16667	0.188
0.4	0	yadI	3	5.16667	0.422
0.4	0	AG1	3	5.16667	0.216
0.4	0	ptsI	3	5.41667	0.193
0.4	0	clcB	3	5.41667	0.174
0.4	0	ycaM	3	5.41667	0.193
0.4	0	yadI	3	5.41667	0.433
0.4	0	AG1	3	5.41667	0.227
0.4	0	ptsI	3	5.66667	0.198
0.4	0	clcB	3	5.66667	0.178
0.4	0	ycaM	3	5.66667	0.201
0.4	0	yadI	3	5.66667	0.45
0.4	0	AG1	3	5.66667	0.236
0.4	0	ptsI	3	5.91667	0.203
0.4	0	clcB	3	5.91667	0.18
0.4	0	ycaM	3	5.91667	0.208
0.4	0	yadI	3	5.91667	0.551
0.4	0	AG1	3	5.91667	0.248
0.4	0	ptsI	3	6.16667	0.209
0.4	0	clcB	3	6.16667	0.186
0.4	0	ycaM	3	6.16667	0.216
0.4	0	yadI	3	6.16667	0.538
0.4	0	AG1	3	6.16667	0.259
0.4	0	ptsI	3	6.41667	0.216
0.4	0	clcB	3	6.41667	0.191
0.4	0	ycaM	3	6.41667	0.227

0.4	0	yadI	3	6.41667	0.54
0.4	0	AG1	3	6.41667	0.272
0.4	0	ptsI	3	6.66667	0.223
0.4	0	clcB	3	6.66667	0.195
0.4	0	ycaM	3	6.66667	0.238
0.4	0	yadI	3	6.66667	0.545
0.4	0	AG1	3	6.66667	0.286
0.4	0	ptsI	3	6.91667	0.232
0.4	0	clcB	3	6.91667	0.202
0.4	0	ycaM	3	6.91667	0.252
0.4	0	yadI	3	6.91667	0.565
0.4	0	AG1	3	6.91667	0.299
0.4	0	ptsI	3	7.16667	0.241
0.4	0	clcB	3	7.16667	0.211
0.4	0	ycaM	3	7.16667	0.265
0.4	0	yadI	3	7.16667	0.547
0.4	0	AG1	3	7.16667	0.31
0.4	0	ptsI	3	7.41667	0.253
0.4	0	clcB	3	7.41667	0.218
0.4	0	ycaM	3	7.41667	0.277
0.4	0	yadI	3	7.41667	0.563
0.4	0	AG1	3	7.41667	0.317
0.4	0	ptsI	3	7.66667	0.263
0.4	0	clcB	3	7.66667	0.228
0.4	0	ycaM	3	7.66667	0.285
0.4	0	yadI	3	7.66667	0.558
0.4	0	AG1	3	7.66667	0.328
0.4	0	ptsI	3	7.91667	0.27
0.4	0	clcB	3	7.91667	0.236
0.4	0	ycaM	3	7.91667	0.292
0.4	0	yadI	3	7.91667	0.571
0.4	0	AG1	3	7.91667	0.334
0.4	0	ptsI	3	8.16667	0.279
0.4	0	clcB	3	8.16667	0.241
0.4	0	ycaM	3	8.16667	0.302
0.4	0	yadI	3	8.16667	0.572
0.4	0	AG1	3	8.16667	0.343
0.4	0	ptsI	3	8.41667	0.286
0.4	0	clcB	3	8.41667	0.247
0.4	0	ycaM	3	8.41667	0.31
0.4	0	yadI	3	8.41667	0.609
0.4	0	AG1	3	8.41667	0.353
0.4	0	ptsI	3	9.05	0.26
0.4	0	clcB	3	9.05	0.278
0.4	0	ycaM	3	9.05	0.593
0.4	0	yadI	3	9.05	1.3
0.4	0	AG1	3	9.05	0.373
0.4	0	ptsI	3	9.3	0.265
0.4	0	clcB	3	9.3	0.285
0.4	0	ycaM	3	9.3	0.527
0.4	0	yadI	3	9.3	1.255
0.4	0	AG1	3	9.3	0.372
0.4	0	ptsI	3	9.55	0.272

0.4	0	clcB	3	9.55	0.29
0.4	0	ycaM	3	9.55	0.488
0.4	0	yadI	3	9.55	1.237
0.4	0	AG1	3	9.55	0.381
0.4	0	ptsI	3	9.8	0.278
0.4	0	clcB	3	9.8	0.294
0.4	0	ycaM	3	9.8	0.475
0.4	0	yadI	3	9.8	1.234
0.4	0	AG1	3	9.8	0.393
0.4	0	ptsI	3	10.05	0.286
0.4	0	clcB	3	10.05	0.298
0.4	0	ycaM	3	10.05	0.472
0.4	0	yadI	3	10.05	1.248
0.4	0	AG1	3	10.05	0.404
0.4	0	ptsI	3	10.3	0.294
0.4	0	clcB	3	10.3	0.306
0.4	0	ycaM	3	10.3	0.47
0.4	0	yadI	3	10.3	1.249
0.4	0	AG1	3	10.3	0.413
0.4	0	ptsI	3	10.55	0.298
0.4	0	clcB	3	10.55	0.311
0.4	0	ycaM	3	10.55	0.455
0.4	0	yadI	3	10.55	1.262
0.4	0	AG1	3	10.55	0.421
0.4	0	ptsI	3	10.8	0.309
0.4	0	clcB	3	10.8	0.317
0.4	0	ycaM	3	10.8	0.444
0.4	0	yadI	3	10.8	1.26
0.4	0	AG1	3	10.8	0.429
0.4	0	ptsI	3	11.05	0.317
0.4	0	clcB	3	11.05	0.321
0.4	0	ycaM	3	11.05	0.427
0.4	0	yadI	3	11.05	1.334
0.4	0	AG1	3	11.05	0.441
0.4	0	ptsI	3	11.3	0.324
0.4	0	clcB	3	11.3	0.328
0.4	0	ycaM	3	11.3	0.424
0.4	0	yadI	3	11.3	1.311
0.4	0	AG1	3	11.3	0.45
0.4	0	ptsI	3	11.55	0.331
0.4	0	clcB	3	11.55	0.337
0.4	0	ycaM	3	11.55	0.42
0.4	0	yadI	3	11.55	1.323
0.4	0	AG1	3	11.55	0.46
0.4	0	ptsI	3	11.8	0.339
0.4	0	clcB	3	11.8	0.34
0.4	0	ycaM	3	11.8	0.412
0.4	0	yadI	3	11.8	1.451
0.4	0	AG1	3	11.8	0.471
0.4	0	ptsI	3	12.05	0.346
0.4	0	clcB	3	12.05	0.35
0.4	0	ycaM	3	12.05	0.403
0.4	0	yadI	3	12.05	1.368

0.4	0	AG1	3	12.05	0.48
0.4	0	ptsI	3	12.3	0.356
0.4	0	clcB	3	12.3	0.352
0.4	0	ycaM	3	12.3	0.404
0.4	0	yadI	3	12.3	1.4
0.4	0	AG1	3	12.3	0.496
0.4	0	ptsI	3	12.55	0.363
0.4	0	clcB	3	12.55	0.36
0.4	0	ycaM	3	12.55	0.405
0.4	0	yadI	3	12.55	1.416
0.4	0	AG1	3	12.55	0.509
0.4	0	ptsI	3	12.8	0.371
0.4	0	clcB	3	12.8	0.364
0.4	0	ycaM	3	12.8	0.41
0.4	0	yadI	3	12.8	1.489
0.4	0	AG1	3	12.8	0.524
0.4	0	ptsI	3	13.05	0.38
0.4	0	clcB	3	13.05	0.365
0.4	0	ycaM	3	13.05	0.407
0.4	0	yadI	3	13.05	1.456
0.4	0	AG1	3	13.05	0.534
0.4	0	ptsI	3	13.3	0.389
0.4	0	clcB	3	13.3	0.363
0.4	0	ycaM	3	13.3	0.411
0.4	0	yadI	3	13.3	1.5
0.4	0	AG1	3	13.3	0.55
0.4	0	ptsI	3	13.55	0.397
0.4	0	clcB	3	13.55	0.366
0.4	0	ycaM	3	13.55	0.422
0.4	0	yadI	3	13.55	1.532
0.4	0	AG1	3	13.55	0.567
0.4	0	ptsI	3	13.8	0.404
0.4	0	clcB	3	13.8	0.372
0.4	0	ycaM	3	13.8	0.431
0.4	0	yadI	3	13.8	1.599
0.4	0	AG1	3	13.8	0.579
0.4	0	ptsI	3	14.05	0.415
0.4	0	clcB	3	14.05	0.376
0.4	0	ycaM	3	14.05	0.435
0.4	0	yadI	3	14.05	1.574
0.4	0	AG1	3	14.05	0.582
0.4	0	ptsI	3	14.3	0.419
0.4	0	clcB	3	14.3	0.384
0.4	0	ycaM	3	14.3	0.437
0.4	0	yadI	3	14.3	1.563
0.4	0	AG1	3	14.3	0.589
0.4	0	ptsI	3	14.55	0.431
0.4	0	clcB	3	14.55	0.388
0.4	0	ycaM	3	14.55	0.442
0.4	0	yadI	3	14.55	1.564
0.4	0	AG1	3	14.55	0.592
0.4	0	ptsI	3	14.8	0.442
0.4	0	clcB	3	14.8	0.392

0.4	0	ycaM	3	14.8	0.448
0.4	0	yadI	3	14.8	1.655
0.4	0	AG1	3	14.8	0.604
0.4	0	ptsI	3	15.05	0.45
0.4	0	clcB	3	15.05	0.394
0.4	0	ycaM	3	15.05	0.459
0.4	0	yadI	3	15.05	1.698
0.4	0	AG1	3	15.05	0.612
0.4	0	ptsI	3	15.3	0.461
0.4	0	clcB	3	15.3	0.398
0.4	0	ycaM	3	15.3	0.464
0.4	0	yadI	3	15.3	1.682
0.4	0	AG1	3	15.3	0.622
0.4	0	ptsI	3	15.55	0.464
0.4	0	clcB	3	15.55	0.398
0.4	0	ycaM	3	15.55	0.469
0.4	0	yadI	3	15.55	1.716
0.4	0	AG1	3	15.55	0.628
0.4	0	ptsI	3	15.8	0.476
0.4	0	clcB	3	15.8	0.399
0.4	0	ycaM	3	15.8	0.478
0.4	0	yadI	3	15.8	1.725
0.4	0	AG1	3	15.8	0.63
0.4	0	ptsI	3	16.05	0.487
0.4	0	clcB	3	16.05	0.403
0.4	0	ycaM	3	16.05	0.483
0.4	0	yadI	3	16.05	1.704
0.4	0	AG1	3	16.05	0.643
0.4	0	ptsI	3	16.3	0.496
0.4	0	clcB	3	16.3	0.406
0.4	0	ycaM	3	16.3	0.488
0.4	0	yadI	3	16.3	1.645
0.4	0	AG1	3	16.3	0.644
0.4	0	ptsI	3	16.55	0.498
0.4	0	clcB	3	16.55	0.405
0.4	0	ycaM	3	16.55	0.496
0.4	0	yadI	3	16.55	1.734
0.4	0	AG1	3	16.55	0.642
0.4	0	ptsI	3	16.8	0.503
0.4	0	clcB	3	16.8	0.406
0.4	0	ycaM	3	16.8	0.502
0.4	0	yadI	3	16.8	1.686
0.4	0	AG1	3	16.8	0.655
0.4	0	ptsI	3	17.05	0.509
0.4	0	clcB	3	17.05	0.408
0.4	0	ycaM	3	17.05	0.507
0.4	0	yadI	3	17.05	1.694
0.4	0	AG1	3	17.05	0.662
0.4	0	ptsI	3	17.3	0.519
0.4	0	clcB	3	17.3	0.412
0.4	0	ycaM	3	17.3	0.511
0.4	0	yadI	3	17.3	1.691
0.4	0	AG1	3	17.3	0.659

0.4	0	ptsI	3	17.55	0.533
0.4	0	clcB	3	17.55	0.411
0.4	0	ycaM	3	17.55	0.513
0.4	0	yadI	3	17.55	1.672
0.4	0	AG1	3	17.55	0.66
0.4	0	ptsI	3	17.8	0.532
0.4	0	clcB	3	17.8	0.409
0.4	0	ycaM	3	17.8	0.519
0.4	0	yadI	3	17.8	1.672
0.4	0	AG1	3	17.8	0.666
0.4	0	ptsI	3	18.05	0.539
0.4	0	clcB	3	18.05	0.41
0.4	0	ycaM	3	18.05	0.524
0.4	0	yadI	3	18.05	1.653
0.4	0	AG1	3	18.05	0.67
0.4	0	ptsI	3	18.3	0.546
0.4	0	clcB	3	18.3	0.408
0.4	0	ycaM	3	18.3	0.526
0.4	0	yadI	3	18.3	1.627
0.4	0	AG1	3	18.3	0.664
0.4	0	ptsI	3	18.55	0.552
0.4	0	clcB	3	18.55	0.406
0.4	0	ycaM	3	18.55	0.533
0.4	0	yadI	3	18.55	1.601
0.4	0	AG1	3	18.55	0.675
0.4	0	ptsI	3	18.8	0.559
0.4	0	clcB	3	18.8	0.41
0.4	0	ycaM	3	18.8	0.538
0.4	0	yadI	3	18.8	1.589
0.4	0	AG1	3	18.8	0.686
0.4	0	ptsI	3	19.05	0.57
0.4	0	clcB	3	19.05	0.403
0.4	0	ycaM	3	19.05	0.544
0.4	0	yadI	3	19.05	1.426
0.4	0	AG1	3	19.05	0.691
0.4	0	ptsI	3	19.3	0.585
0.4	0	clcB	3	19.3	0.408
0.4	0	ycaM	3	19.3	0.55
0.4	0	yadI	3	19.3	1.356
0.4	0	AG1	3	19.3	0.693
0.4	0	ptsI	3	19.55	0.6
0.4	0	clcB	3	19.55	0.406
0.4	0	ycaM	3	19.55	0.551
0.4	0	yadI	3	19.55	1.359
0.4	0	AG1	3	19.55	0.694
0.4	0	ptsI	3	19.8	0.597
0.4	0	clcB	3	19.8	0.405
0.4	0	ycaM	3	19.8	0.558
0.4	0	yadI	3	19.8	1.346
0.4	0	AG1	3	19.8	0.694
0.4	0	ptsI	3	20.05	0.597
0.4	0	clcB	3	20.05	0.404
0.4	0	ycaM	3	20.05	0.56

0.4	0	yadI	3	20.05	1.339
0.4	0	AG1	3	20.05	0.692
0.4	0	ptsI	3	20.3	0.596
0.4	0	clcB	3	20.3	0.406
0.4	0	ycaM	3	20.3	0.566
0.4	0	yadI	3	20.3	1.215
0.4	0	AG1	3	20.3	0.694
0.4	0	ptsI	3	20.55	0.609
0.4	0	clcB	3	20.55	0.402
0.4	0	ycaM	3	20.55	0.573
0.4	0	yadI	3	20.55	1.223
0.4	0	AG1	3	20.55	0.699
0.4	0	ptsI	3	20.8	0.611
0.4	0	clcB	3	20.8	0.404
0.4	0	ycaM	3	20.8	0.574
0.4	0	yadI	3	20.8	1.135
0.4	0	AG1	3	20.8	0.701
0.4	0	ptsI	3	21.05	0.611
0.4	0	clcB	3	21.05	0.405
0.4	0	ycaM	3	21.05	0.578
0.4	0	yadI	3	21.05	1.101
0.4	0	AG1	3	21.05	0.687
0.4	0	ptsI	3	21.3	0.607
0.4	0	clcB	3	21.3	0.395
0.4	0	ycaM	3	21.3	0.581
0.4	0	yadI	3	21.3	1.103
0.4	0	AG1	3	21.3	0.686
0.4	0	ptsI	3	21.55	0.614
0.4	0	clcB	3	21.55	0.394
0.4	0	ycaM	3	21.55	0.586
0.4	0	yadI	3	21.55	1.116
0.4	0	AG1	3	21.55	0.689
0.4	0	ptsI	3	21.8	0.616
0.4	0	clcB	3	21.8	0.394
0.4	0	ycaM	3	21.8	0.588
0.4	0	yadI	3	21.8	1.102
0.4	0	AG1	3	21.8	0.693
0.4	0	ptsI	3	22.05	0.618
0.4	0	clcB	3	22.05	0.396
0.4	0	ycaM	3	22.05	0.592
0.4	0	yadI	3	22.05	1.044
0.4	0	AG1	3	22.05	0.695
0.4	0	ptsI	3	22.3	0.621
0.4	0	clcB	3	22.3	0.395
0.4	0	ycaM	3	22.3	0.596
0.4	0	yadI	3	22.3	1.04
0.4	0	AG1	3	22.3	0.697
0.4	0	ptsI	3	22.55	0.634
0.4	0	clcB	3	22.55	0.395
0.4	0	ycaM	3	22.55	0.6
0.4	0	yadI	3	22.55	1.002
0.4	0	AG1	3	22.55	0.698
0.4	0	ptsI	3	22.8	0.643

0.4	0	clcB	3	22.8	0.394
0.4	0	ycaM	3	22.8	0.6
0.4	0	yadI	3	22.8	1.01
0.4	0	AG1	3	22.8	0.691
0.4	0	ptsI	3	23.05	0.651
0.4	0	clcB	3	23.05	0.39
0.4	0	ycaM	3	23.05	0.603
0.4	0	yadI	3	23.05	1
0.4	0	AG1	3	23.05	0.694
0.4	0	ptsI	3	23.3	0.657
0.4	0	clcB	3	23.3	0.39
0.4	0	ycaM	3	23.3	0.608
0.4	0	yadI	3	23.3	0.982
0.4	0	AG1	3	23.3	0.692
0.4	0	ptsI	3	23.55	0.665
0.4	0	clcB	3	23.55	0.394
0.4	0	ycaM	3	23.55	0.606
0.4	0	yadI	3	23.55	0.91
0.4	0	AG1	3	23.55	0.695
0.4	0	ptsI	3	23.8	0.665
0.4	0	clcB	3	23.8	0.386
0.4	0	ycaM	3	23.8	0.614
0.4	0	yadI	3	23.8	0.891
0.4	0	AG1	3	23.8	0.693
0.4	0	ptsI	3	24.05	0.672
0.4	0	clcB	3	24.05	0.381
0.4	0	ycaM	3	24.05	0.615
0.4	0	yadI	3	24.05	0.885
0.4	0	AG1	3	24.05	0.692
0.4	0	ptsI	3	24.3	0.672
0.4	0	clcB	3	24.3	0.38
0.4	0	ycaM	3	24.3	0.622
0.4	0	yadI	3	24.3	0.763
0.4	0	AG1	3	24.3	0.704
0.4	0	ptsI	4	0	0.183
0.4	0	clcB	4	0	0.197
0.4	0	ycaM	4	0	0.223
0.4	0	yadI	4	0	0.233
0.4	0	AG1	4	0	0.22
0.4	0	ptsI	4	0.25	0.184
0.4	0	clcB	4	0.25	0.193
0.4	0	ycaM	4	0.25	0.216
0.4	0	yadI	4	0.25	0.227
0.4	0	AG1	4	0.25	0.214
0.4	0	ptsI	4	0.5	0.184
0.4	0	clcB	4	0.5	0.193
0.4	0	ycaM	4	0.5	0.216
0.4	0	yadI	4	0.5	0.227
0.4	0	AG1	4	0.5	0.217
0.4	0	ptsI	4	0.75	0.188
0.4	0	clcB	4	0.75	0.198
0.4	0	ycaM	4	0.75	0.217
0.4	0	yadI	4	0.75	0.228

0.4	0	AG1	4	0.75	0.22
0.4	0	ptsI	4	1	0.192
0.4	0	clcB	4	1	0.202
0.4	0	ycaM	4	1	0.219
0.4	0	yadI	4	1	0.231
0.4	0	AG1	4	1	0.222
0.4	0	ptsI	4	1.25	0.198
0.4	0	clcB	4	1.25	0.208
0.4	0	ycaM	4	1.25	0.225
0.4	0	yadI	4	1.25	0.235
0.4	0	AG1	4	1.25	0.227
0.4	0	ptsI	4	1.5	0.198
0.4	0	clcB	4	1.5	0.209
0.4	0	ycaM	4	1.5	0.23
0.4	0	yadI	4	1.5	0.24
0.4	0	AG1	4	1.5	0.231
0.4	0	ptsI	4	1.75	0.21
0.4	0	clcB	4	1.75	0.221
0.4	0	ycaM	4	1.75	0.238
0.4	0	yadI	4	1.75	0.25
0.4	0	AG1	4	1.75	0.239
0.4	0	ptsI	4	2	0.223
0.4	0	clcB	4	2	0.231
0.4	0	ycaM	4	2	0.247
0.4	0	yadI	4	2	0.26
0.4	0	AG1	4	2	0.248
0.4	0	ptsI	4	2.25	0.239
0.4	0	clcB	4	2.25	0.244
0.4	0	ycaM	4	2.25	0.259
0.4	0	yadI	4	2.25	0.271
0.4	0	AG1	4	2.25	0.262
0.4	0	ptsI	4	2.5	0.246
0.4	0	clcB	4	2.5	0.247
0.4	0	ycaM	4	2.5	0.268
0.4	0	yadI	4	2.5	0.282
0.4	0	AG1	4	2.5	0.267
0.4	0	ptsI	4	2.75	0.26
0.4	0	clcB	4	2.75	0.254
0.4	0	ycaM	4	2.75	0.28
0.4	0	yadI	4	2.75	0.293
0.4	0	AG1	4	2.75	0.278
0.4	0	ptsI	4	3	0.273
0.4	0	clcB	4	3	0.276
0.4	0	ycaM	4	3	0.292
0.4	0	yadI	4	3	0.311
0.4	0	AG1	4	3	0.29
0.4	0	ptsI	4	3.25	0.289
0.4	0	clcB	4	3.25	0.281
0.4	0	ycaM	4	3.25	0.306
0.4	0	yadI	4	3.25	0.329
0.4	0	AG1	4	3.25	0.301
0.4	0	ptsI	4	3.5	0.305
0.4	0	clcB	4	3.5	0.301

0.4	0	ycaM	4	3.5	0.32
0.4	0	yadI	4	3.5	0.356
0.4	0	AG1	4	3.5	0.32
0.4	0	ptsI	4	3.75	0.323
0.4	0	clcB	4	3.75	0.307
0.4	0	ycaM	4	3.75	0.335
0.4	0	yadI	4	3.75	0.374
0.4	0	AG1	4	3.75	0.34
0.4	0	ptsI	4	4	0.338
0.4	0	clcB	4	4	0.329
0.4	0	ycaM	4	4	0.346
0.4	0	yadI	4	4	0.391
0.4	0	AG1	4	4	0.352
0.4	0	ptsI	4	4.35	0.348
0.4	0	clcB	4	4.35	0.271
0.4	0	ycaM	4	4.35	0.392
0.4	0	yadI	4	4.35	0.402
0.4	0	AG1	4	4.35	0.346
0.4	0	ptsI	4	4.6	0.355
0.4	0	clcB	4	4.6	0.28
0.4	0	ycaM	4	4.6	0.367
0.4	0	yadI	4	4.6	0.384
0.4	0	AG1	4	4.6	0.35
0.4	0	ptsI	4	4.85	0.368
0.4	0	clcB	4	4.85	0.291
0.4	0	ycaM	4	4.85	0.378
0.4	0	yadI	4	4.85	0.396
0.4	0	AG1	4	4.85	0.362
0.4	0	ptsI	4	5.1	0.384
0.4	0	clcB	4	5.1	0.3
0.4	0	ycaM	4	5.1	0.39
0.4	0	yadI	4	5.1	0.411
0.4	0	AG1	4	5.1	0.374
0.4	0	ptsI	4	5.35	0.403
0.4	0	clcB	4	5.35	0.31
0.4	0	ycaM	4	5.35	0.406
0.4	0	yadI	4	5.35	0.426
0.4	0	AG1	4	5.35	0.391
0.4	0	ptsI	4	5.6	0.416
0.4	0	clcB	4	5.6	0.323
0.4	0	ycaM	4	5.6	0.411
0.4	0	yadI	4	5.6	0.448
0.4	0	AG1	4	5.6	0.403
0.4	0	ptsI	4	5.85	0.428
0.4	0	clcB	4	5.85	0.338
0.4	0	ycaM	4	5.85	0.419
0.4	0	yadI	4	5.85	0.454
0.4	0	AG1	4	5.85	0.418
0.4	0	ptsI	4	6.1	0.447
0.4	0	clcB	4	6.1	0.353
0.4	0	ycaM	4	6.1	0.428
0.4	0	yadI	4	6.1	0.47
0.4	0	AG1	4	6.1	0.431

0.4	0	ptsI	4	6.35	0.464
0.4	0	clcB	4	6.35	0.365
0.4	0	ycaM	4	6.35	0.437
0.4	0	yadI	4	6.35	0.485
0.4	0	AG1	4	6.35	0.439
0.4	0	ptsI	4	6.6	0.477
0.4	0	clcB	4	6.6	0.374
0.4	0	ycaM	4	6.6	0.446
0.4	0	yadI	4	6.6	0.499
0.4	0	AG1	4	6.6	0.452
0.4	0	ptsI	4	6.85	0.492
0.4	0	clcB	4	6.85	0.385
0.4	0	ycaM	4	6.85	0.451
0.4	0	yadI	4	6.85	0.512
0.4	0	AG1	4	6.85	0.463
0.4	0	ptsI	4	7.1	0.503
0.4	0	clcB	4	7.1	0.393
0.4	0	ycaM	4	7.1	0.459
0.4	0	yadI	4	7.1	0.522
0.4	0	AG1	4	7.1	0.477
0.4	0	ptsI	4	7.35	0.511
0.4	0	clcB	4	7.35	0.403
0.4	0	ycaM	4	7.35	0.465
0.4	0	yadI	4	7.35	0.529
0.4	0	AG1	4	7.35	0.486
0.4	0	ptsI	4	7.6	0.52
0.4	0	clcB	4	7.6	0.412
0.4	0	ycaM	4	7.6	0.469
0.4	0	yadI	4	7.6	0.536
0.4	0	AG1	4	7.6	0.496
0.4	0	ptsI	4	7.85	0.526
0.4	0	clcB	4	7.85	0.419
0.4	0	ycaM	4	7.85	0.471
0.4	0	yadI	4	7.85	0.54
0.4	0	AG1	4	7.85	0.505
0.4	0	ptsI	4	8.1	0.537
0.4	0	clcB	4	8.1	0.43
0.4	0	ycaM	4	8.1	0.477
0.4	0	yadI	4	8.1	0.554
0.4	0	AG1	4	8.1	0.515
0.4	0	ptsI	4	8.35	0.546
0.4	0	clcB	4	8.35	0.436
0.4	0	ycaM	4	8.35	0.48
0.4	0	yadI	4	8.35	0.564
0.4	0	AG1	4	8.35	0.52
0.4	0	ptsI	4	8.83333	0.38
0.4	0	clcB	4	8.83333	0.375
0.4	0	ycaM	4	8.83333	0.411
0.4	0	yadI	4	8.83333	0.512
0.4	0	AG1	4	8.83333	0.537
0.4	0	ptsI	4	9.08333	0.382
0.4	0	clcB	4	9.08333	0.375
0.4	0	ycaM	4	9.08333	0.412

0.4	0	yadI	4	9.08333	0.509
0.4	0	AG1	4	9.08333	0.531
0.4	0	ptsI	4	9.33333	0.393
0.4	0	clcB	4	9.33333	0.38
0.4	0	ycaM	4	9.33333	0.414
0.4	0	yadI	4	9.33333	0.512
0.4	0	AG1	4	9.33333	0.543
0.4	0	ptsI	4	9.58333	0.407
0.4	0	clcB	4	9.58333	0.385
0.4	0	ycaM	4	9.58333	0.42
0.4	0	yadI	4	9.58333	0.526
0.4	0	AG1	4	9.58333	0.557
0.4	0	ptsI	4	9.83333	0.414
0.4	0	clcB	4	9.83333	0.389
0.4	0	ycaM	4	9.83333	0.423
0.4	0	yadI	4	9.83333	0.532
0.4	0	AG1	4	9.83333	0.566
0.4	0	ptsI	4	10.0833	0.422
0.4	0	clcB	4	10.0833	0.394
0.4	0	ycaM	4	10.0833	0.428
0.4	0	yadI	4	10.0833	0.54
0.4	0	AG1	4	10.0833	0.572
0.4	0	ptsI	4	10.3333	0.428
0.4	0	clcB	4	10.3333	0.402
0.4	0	ycaM	4	10.3333	0.434
0.4	0	yadI	4	10.3333	0.546
0.4	0	AG1	4	10.3333	0.581
0.4	0	ptsI	4	10.5833	0.434
0.4	0	clcB	4	10.5833	0.406
0.4	0	ycaM	4	10.5833	0.439
0.4	0	yadI	4	10.5833	0.554
0.4	0	AG1	4	10.5833	0.589
0.4	0	ptsI	4	10.8333	0.442
0.4	0	clcB	4	10.8333	0.409
0.4	0	ycaM	4	10.8333	0.44
0.4	0	yadI	4	10.8333	0.558
0.4	0	AG1	4	10.8333	0.594
0.4	0	ptsI	4	11.0833	0.449
0.4	0	clcB	4	11.0833	0.413
0.4	0	ycaM	4	11.0833	0.446
0.4	0	yadI	4	11.0833	0.563
0.4	0	AG1	4	11.0833	0.602
0.4	0	ptsI	4	11.3333	0.458
0.4	0	clcB	4	11.3333	0.424
0.4	0	ycaM	4	11.3333	0.453
0.4	0	yadI	4	11.3333	0.57
0.4	0	AG1	4	11.3333	0.609
0.4	0	ptsI	4	11.5833	0.463
0.4	0	clcB	4	11.5833	0.425
0.4	0	ycaM	4	11.5833	0.458
0.4	0	yadI	4	11.5833	0.576
0.4	0	AG1	4	11.5833	0.615
0.4	0	ptsI	4	11.8333	0.471

0.4	0	clcB	4	11.8333	0.429
0.4	0	ycaM	4	11.8333	0.461
0.4	0	yadI	4	11.8333	0.58
0.4	0	AG1	4	11.8333	0.62
0.4	0	ptsI	4	12.0833	0.475
0.4	0	clcB	4	12.0833	0.433
0.4	0	ycaM	4	12.0833	0.467
0.4	0	yadI	4	12.0833	0.586
0.4	0	AG1	4	12.0833	0.625
0.4	0	ptsI	4	12.3333	0.483
0.4	0	clcB	4	12.3333	0.44
0.4	0	ycaM	4	12.3333	0.469
0.4	0	yadI	4	12.3333	0.59
0.4	0	AG1	4	12.3333	0.631
0.4	0	ptsI	4	12.5833	0.489
0.4	0	clcB	4	12.5833	0.445
0.4	0	ycaM	4	12.5833	0.475
0.4	0	yadI	4	12.5833	0.595
0.4	0	AG1	4	12.5833	0.64
0.4	0	ptsI	4	12.8333	0.492
0.4	0	clcB	4	12.8333	0.445
0.4	0	ycaM	4	12.8333	0.479
0.4	0	yadI	4	12.8333	0.599
0.4	0	AG1	4	12.8333	0.64
0.4	0	ptsI	4	13.0833	0.498
0.4	0	clcB	4	13.0833	0.449
0.4	0	ycaM	4	13.0833	0.482
0.4	0	yadI	4	13.0833	0.602
0.4	0	AG1	4	13.0833	0.649
0.4	0	ptsI	4	13.3333	0.507
0.4	0	clcB	4	13.3333	0.452
0.4	0	ycaM	4	13.3333	0.487
0.4	0	yadI	4	13.3333	0.606
0.4	0	AG1	4	13.3333	0.655
0.4	0	ptsI	4	13.5833	0.509
0.4	0	clcB	4	13.5833	0.454
0.4	0	ycaM	4	13.5833	0.491
0.4	0	yadI	4	13.5833	0.608
0.4	0	AG1	4	13.5833	0.658
0.4	0	ptsI	4	13.8333	0.515
0.4	0	clcB	4	13.8333	0.457
0.4	0	ycaM	4	13.8333	0.495
0.4	0	yadI	4	13.8333	0.611
0.4	0	AG1	4	13.8333	0.661
0.4	0	ptsI	4	14.0833	0.519
0.4	0	clcB	4	14.0833	0.456
0.4	0	ycaM	4	14.0833	0.496
0.4	0	yadI	4	14.0833	0.615
0.4	0	AG1	4	14.0833	0.667
0.4	0	ptsI	4	14.3333	0.527
0.4	0	clcB	4	14.3333	0.461
0.4	0	ycaM	4	14.3333	0.503
0.4	0	yadI	4	14.3333	0.618

0.4	0	AG1	4	14.3333	0.67
0.4	0	ptsI	4	14.5833	0.527
0.4	0	clcB	4	14.5833	0.462
0.4	0	ycaM	4	14.5833	0.501
0.4	0	yadI	4	14.5833	0.62
0.4	0	AG1	4	14.5833	0.672
0.4	0	ptsI	4	14.8333	0.532
0.4	0	clcB	4	14.8333	0.464
0.4	0	ycaM	4	14.8333	0.509
0.4	0	yadI	4	14.8333	0.623
0.4	0	AG1	4	14.8333	0.679
0.4	0	ptsI	4	15.0833	0.532
0.4	0	clcB	4	15.0833	0.465
0.4	0	ycaM	4	15.0833	0.512
0.4	0	yadI	4	15.0833	0.626
0.4	0	AG1	4	15.0833	0.682
0.4	0	ptsI	4	15.3333	0.541
0.4	0	clcB	4	15.3333	0.471
0.4	0	ycaM	4	15.3333	0.514
0.4	0	yadI	4	15.3333	0.631
0.4	0	AG1	4	15.3333	0.68
0.4	0	ptsI	4	15.5833	0.548
0.4	0	clcB	4	15.5833	0.472
0.4	0	ycaM	4	15.5833	0.516
0.4	0	yadI	4	15.5833	0.632
0.4	0	AG1	4	15.5833	0.685
0.4	0	ptsI	4	15.8333	0.546
0.4	0	clcB	4	15.8333	0.472
0.4	0	ycaM	4	15.8333	0.516
0.4	0	yadI	4	15.8333	0.636
0.4	0	AG1	4	15.8333	0.683
0.4	0	ptsI	4	16.0833	0.553
0.4	0	clcB	4	16.0833	0.476
0.4	0	ycaM	4	16.0833	0.519
0.4	0	yadI	4	16.0833	0.64
0.4	0	AG1	4	16.0833	0.69
0.4	0	ptsI	4	16.3333	0.555
0.4	0	clcB	4	16.3333	0.476
0.4	0	ycaM	4	16.3333	0.522
0.4	0	yadI	4	16.3333	0.64
0.4	0	AG1	4	16.3333	0.686
0.4	0	ptsI	4	16.5833	0.56
0.4	0	clcB	4	16.5833	0.475
0.4	0	ycaM	4	16.5833	0.523
0.4	0	yadI	4	16.5833	0.643
0.4	0	AG1	4	16.5833	0.69
0.4	0	ptsI	4	16.8333	0.561
0.4	0	clcB	4	16.8333	0.478
0.4	0	ycaM	4	16.8333	0.521
0.4	0	yadI	4	16.8333	0.642
0.4	0	AG1	4	16.8333	0.694
0.4	0	ptsI	4	17.0833	0.564
0.4	0	clcB	4	17.0833	0.483

0.4	0	ycaM	4	17.0833	0.527
0.4	0	yadI	4	17.0833	0.644
0.4	0	AG1	4	17.0833	0.696
0.4	0	ptsI	4	17.3333	0.568
0.4	0	clcB	4	17.3333	0.486
0.4	0	ycaM	4	17.3333	0.53
0.4	0	yadI	4	17.3333	0.649
0.4	0	AG1	4	17.3333	0.696
0.4	0	ptsI	4	17.5833	0.567
0.4	0	clcB	4	17.5833	0.483
0.4	0	ycaM	4	17.5833	0.528
0.4	0	yadI	4	17.5833	0.649
0.4	0	AG1	4	17.5833	0.701
0.4	0	ptsI	4	17.8333	0.573
0.4	0	clcB	4	17.8333	0.483
0.4	0	ycaM	4	17.8333	0.533
0.4	0	yadI	4	17.8333	0.647
0.4	0	AG1	4	17.8333	0.678
0.4	0	ptsI	4	18.0833	0.572
0.4	0	clcB	4	18.0833	0.486
0.4	0	ycaM	4	18.0833	0.535
0.4	0	yadI	4	18.0833	0.654
0.4	0	AG1	4	18.0833	0.7
0.4	0	ptsI	4	18.3333	0.577
0.4	0	clcB	4	18.3333	0.486
0.4	0	ycaM	4	18.3333	0.536
0.4	0	yadI	4	18.3333	0.659
0.4	0	AG1	4	18.3333	0.706
0.4	0	ptsI	4	18.5833	0.577
0.4	0	clcB	4	18.5833	0.487
0.4	0	ycaM	4	18.5833	0.536
0.4	0	yadI	4	18.5833	0.656
0.4	0	AG1	4	18.5833	0.705
0.4	0	ptsI	4	18.8333	0.586
0.4	0	clcB	4	18.8333	0.486
0.4	0	ycaM	4	18.8333	0.54
0.4	0	yadI	4	18.8333	0.654
0.4	0	AG1	4	18.8333	0.687
0.4	0	ptsI	4	19.0833	0.589
0.4	0	clcB	4	19.0833	0.492
0.4	0	ycaM	4	19.0833	0.54
0.4	0	yadI	4	19.0833	0.658
0.4	0	AG1	4	19.0833	0.712
0.4	0	ptsI	4	19.3333	0.59
0.4	0	clcB	4	19.3333	0.49
0.4	0	ycaM	4	19.3333	0.543
0.4	0	yadI	4	19.3333	0.66
0.4	0	AG1	4	19.3333	0.711
0.4	0	ptsI	4	19.5833	0.596
0.4	0	clcB	4	19.5833	0.497
0.4	0	ycaM	4	19.5833	0.545
0.4	0	yadI	4	19.5833	0.664
0.4	0	AG1	4	19.5833	0.712

0.4	0	ptsI	4	19.8333	0.597
0.4	0	clcB	4	19.8333	0.493
0.4	0	ycaM	4	19.8333	0.541
0.4	0	yadI	4	19.8333	0.663
0.4	0	AG1	4	19.8333	0.712
0.4	0	ptsI	4	20.0833	0.603
0.4	0	clcB	4	20.0833	0.498
0.4	0	ycaM	4	20.0833	0.549
0.4	0	yadI	4	20.0833	0.663
0.4	0	AG1	4	20.0833	0.72
0.4	0	ptsI	4	20.3333	0.602
0.4	0	clcB	4	20.3333	0.492
0.4	0	ycaM	4	20.3333	0.546
0.4	0	yadI	4	20.3333	0.665
0.4	0	AG1	4	20.3333	0.714
0.4	0	ptsI	4	20.5833	0.605
0.4	0	clcB	4	20.5833	0.493
0.4	0	ycaM	4	20.5833	0.546
0.4	0	yadI	4	20.5833	0.668
0.4	0	AG1	4	20.5833	0.717
0.4	0	ptsI	4	20.8333	0.608
0.4	0	clcB	4	20.8333	0.5
0.4	0	ycaM	4	20.8333	0.552
0.4	0	yadI	4	20.8333	0.67
0.4	0	AG1	4	20.8333	0.714
0.4	0	ptsI	4	21.0833	0.611
0.4	0	clcB	4	21.0833	0.498
0.4	0	ycaM	4	21.0833	0.547
0.4	0	yadI	4	21.0833	0.669
0.4	0	AG1	4	21.0833	0.716
0.4	0	ptsI	4	21.3333	0.61
0.4	0	clcB	4	21.3333	0.498
0.4	0	ycaM	4	21.3333	0.548
0.4	0	yadI	4	21.3333	0.671
0.4	0	AG1	4	21.3333	0.716
0.4	0	ptsI	4	21.5833	0.613
0.4	0	clcB	4	21.5833	0.497
0.4	0	ycaM	4	21.5833	0.551
0.4	0	yadI	4	21.5833	0.67
0.4	0	AG1	4	21.5833	0.718
0.4	0	ptsI	4	21.8333	0.618
0.4	0	clcB	4	21.8333	0.498
0.4	0	ycaM	4	21.8333	0.552
0.4	0	yadI	4	21.8333	0.675
0.4	0	AG1	4	21.8333	0.717
0.4	0	ptsI	4	22.0833	0.624
0.4	0	clcB	4	22.0833	0.5
0.4	0	ycaM	4	22.0833	0.554
0.4	0	yadI	4	22.0833	0.67
0.4	0	AG1	4	22.0833	0.716
0.4	0	ptsI	4	22.3333	0.626
0.4	0	clcB	4	22.3333	0.504
0.4	0	ycaM	4	22.3333	0.552

0.4	0	yadI	4	22.3333	0.674
0.4	0	AG1	4	22.3333	0.714
0.4	0	ptsI	4	22.5833	0.627
0.4	0	clcB	4	22.5833	0.5
0.4	0	ycaM	4	22.5833	0.55
0.4	0	yadI	4	22.5833	0.674
0.4	0	AG1	4	22.5833	0.718
0.4	0	ptsI	4	22.8333	0.631
0.4	0	clcB	4	22.8333	0.5
0.4	0	ycaM	4	22.8333	0.551
0.4	0	yadI	4	22.8333	0.676
0.4	0	AG1	4	22.8333	0.72
0.4	0	ptsI	4	23.0833	0.638
0.4	0	clcB	4	23.0833	0.501
0.4	0	ycaM	4	23.0833	0.552
0.4	0	yadI	4	23.0833	0.672
0.4	0	AG1	4	23.0833	0.72
0.4	0	ptsI	4	23.3333	0.64
0.4	0	clcB	4	23.3333	0.501
0.4	0	ycaM	4	23.3333	0.557
0.4	0	yadI	4	23.3333	0.679
0.4	0	AG1	4	23.3333	0.72
0.4	0	ptsI	4	23.5833	0.638
0.4	0	clcB	4	23.5833	0.502
0.4	0	ycaM	4	23.5833	0.557
0.4	0	yadI	4	23.5833	0.676
0.4	0	AG1	4	23.5833	0.719
0.4	0	ptsI	4	23.8333	0.65
0.4	0	clcB	4	23.8333	0.503
0.4	0	ycaM	4	23.8333	0.553
0.4	0	yadI	4	23.8333	0.678
0.4	0	AG1	4	23.8333	0.722
0.4	0	ptsI	4	24.0833	0.649
0.4	0	clcB	4	24.0833	0.503
0.4	0	ycaM	4	24.0833	0.553
0.4	0	yadI	4	24.0833	0.68
0.4	0	AG1	4	24.0833	0.718
0.4	0	ptsI	5	0	0.156
0.4	0	clcB	5	0	0.167
0.4	0	ycaM	5	0	0.16
0.4	0	yadI	5	0	0.171
0.4	0	AG1	5	0	0.166
0.4	0	ptsI	5	0.35	0.158
0.4	0	clcB	5	0.35	0.162
0.4	0	ycaM	5	0.35	0.154
0.4	0	yadI	5	0.35	0.166
0.4	0	AG1	5	0.35	0.162
0.4	0	ptsI	5	0.6	0.159
0.4	0	clcB	5	0.6	0.162
0.4	0	ycaM	5	0.6	0.153
0.4	0	yadI	5	0.6	0.163
0.4	0	AG1	5	0.6	0.163
0.4	0	ptsI	5	0.85	0.159

0.4	0	clcB	5	0.85	0.162
0.4	0	ycaM	5	0.85	0.152
0.4	0	yadI	5	0.85	0.164
0.4	0	AG1	5	0.85	0.164
0.4	0	ptsI	5	1.1	0.161
0.4	0	clcB	5	1.1	0.164
0.4	0	ycaM	5	1.1	0.154
0.4	0	yadI	5	1.1	0.167
0.4	0	AG1	5	1.1	0.165
0.4	0	ptsI	5	1.35	0.163
0.4	0	clcB	5	1.35	0.165
0.4	0	ycaM	5	1.35	0.156
0.4	0	yadI	5	1.35	0.167
0.4	0	AG1	5	1.35	0.168
0.4	0	ptsI	5	1.6	0.164
0.4	0	clcB	5	1.6	0.169
0.4	0	ycaM	5	1.6	0.158
0.4	0	yadI	5	1.6	0.172
0.4	0	AG1	5	1.6	0.172
0.4	0	ptsI	5	1.85	0.167
0.4	0	clcB	5	1.85	0.171
0.4	0	ycaM	5	1.85	0.161
0.4	0	yadI	5	1.85	0.175
0.4	0	AG1	5	1.85	0.176
0.4	0	ptsI	5	2.1	0.17
0.4	0	clcB	5	2.1	0.177
0.4	0	ycaM	5	2.1	0.164
0.4	0	yadI	5	2.1	0.183
0.4	0	AG1	5	2.1	0.184
0.4	0	ptsI	5	2.35	0.177
0.4	0	clcB	5	2.35	0.184
0.4	0	ycaM	5	2.35	0.169
0.4	0	yadI	5	2.35	0.19
0.4	0	AG1	5	2.35	0.192
0.4	0	ptsI	5	2.6	0.174
0.4	0	clcB	5	2.6	0.186
0.4	0	ycaM	5	2.6	0.176
0.4	0	yadI	5	2.6	0.192
0.4	0	AG1	5	2.6	0.189
0.4	0	ptsI	5	2.85	0.184
0.4	0	clcB	5	2.85	0.199
0.4	0	ycaM	5	2.85	0.182
0.4	0	yadI	5	2.85	0.205
0.4	0	AG1	5	2.85	0.205
0.4	0	ptsI	5	3.1	0.192
0.4	0	clcB	5	3.1	0.213
0.4	0	ycaM	5	3.1	0.19
0.4	0	yadI	5	3.1	0.218
0.4	0	AG1	5	3.1	0.217
0.4	0	ptsI	5	3.35	0.196
0.4	0	clcB	5	3.35	0.226
0.4	0	ycaM	5	3.35	0.201
0.4	0	yadI	5	3.35	0.234

0.4	0	AG1	5	3.35	0.236
0.4	0	ptsI	5	3.6	0.201
0.4	0	clcB	5	3.6	0.239
0.4	0	ycaM	5	3.6	0.21
0.4	0	yadI	5	3.6	0.25
0.4	0	AG1	5	3.6	0.252
0.4	0	ptsI	5	3.85	0.21
0.4	0	clcB	5	3.85	0.257
0.4	0	ycaM	5	3.85	0.223
0.4	0	yadI	5	3.85	0.266
0.4	0	AG1	5	3.85	0.269
0.4	0	ptsI	5	4.38333	0.209
0.4	0	clcB	5	4.38333	0.264
0.4	0	ycaM	5	4.38333	0.234
0.4	0	yadI	5	4.38333	0.284
0.4	0	AG1	5	4.38333	0.3
0.4	0	ptsI	5	4.63333	0.21
0.4	0	clcB	5	4.63333	0.258
0.4	0	ycaM	5	4.63333	0.236
0.4	0	yadI	5	4.63333	0.272
0.4	0	AG1	5	4.63333	0.286
0.4	0	ptsI	5	4.88333	0.216
0.4	0	clcB	5	4.88333	0.262
0.4	0	ycaM	5	4.88333	0.244
0.4	0	yadI	5	4.88333	0.288
0.4	0	AG1	5	4.88333	0.296
0.4	0	ptsI	5	5.13333	0.219
0.4	0	clcB	5	5.13333	0.27
0.4	0	ycaM	5	5.13333	0.256
0.4	0	yadI	5	5.13333	0.304
0.4	0	AG1	5	5.13333	0.308
0.4	0	ptsI	5	5.38333	0.226
0.4	0	clcB	5	5.38333	0.28
0.4	0	ycaM	5	5.38333	0.271
0.4	0	yadI	5	5.38333	0.318
0.4	0	AG1	5	5.38333	0.322
0.4	0	ptsI	5	5.63333	0.236
0.4	0	clcB	5	5.63333	0.294
0.4	0	ycaM	5	5.63333	0.284
0.4	0	yadI	5	5.63333	0.333
0.4	0	AG1	5	5.63333	0.332
0.4	0	ptsI	5	5.88333	0.243
0.4	0	clcB	5	5.88333	0.313
0.4	0	ycaM	5	5.88333	0.298
0.4	0	yadI	5	5.88333	0.347
0.4	0	AG1	5	5.88333	0.34
0.4	0	ptsI	5	6.13333	0.255
0.4	0	clcB	5	6.13333	0.329
0.4	0	ycaM	5	6.13333	0.312
0.4	0	yadI	5	6.13333	0.363
0.4	0	AG1	5	6.13333	0.353
0.4	0	ptsI	5	6.38333	0.265
0.4	0	clcB	5	6.38333	0.345

0.4	0	ycaM	5	6.38333	0.323
0.4	0	yadI	5	6.38333	0.376
0.4	0	AG1	5	6.38333	0.37
0.4	0	ptsI	5	6.63333	0.275
0.4	0	clcB	5	6.63333	0.355
0.4	0	ycaM	5	6.63333	0.336
0.4	0	yadI	5	6.63333	0.391
0.4	0	AG1	5	6.63333	0.388
0.4	0	ptsI	5	6.88333	0.287
0.4	0	clcB	5	6.88333	0.369
0.4	0	ycaM	5	6.88333	0.345
0.4	0	yadI	5	6.88333	0.408
0.4	0	AG1	5	6.88333	0.404
0.4	0	ptsI	5	7.13333	0.298
0.4	0	clcB	5	7.13333	0.377
0.4	0	ycaM	5	7.13333	0.358
0.4	0	yadI	5	7.13333	0.425
0.4	0	AG1	5	7.13333	0.417
0.4	0	ptsI	5	7.38333	0.31
0.4	0	clcB	5	7.38333	0.39
0.4	0	ycaM	5	7.38333	0.368
0.4	0	yadI	5	7.38333	0.438
0.4	0	AG1	5	7.38333	0.433
0.4	0	ptsI	5	7.63333	0.321
0.4	0	clcB	5	7.63333	0.402
0.4	0	ycaM	5	7.63333	0.382
0.4	0	yadI	5	7.63333	0.453
0.4	0	AG1	5	7.63333	0.45
0.4	0	ptsI	5	7.88333	0.331
0.4	0	clcB	5	7.88333	0.408
0.4	0	ycaM	5	7.88333	0.394
0.4	0	yadI	5	7.88333	0.466
0.4	0	AG1	5	7.88333	0.462
0.4	0	ptsI	5	8.13333	0.343
0.4	0	clcB	5	8.13333	0.42
0.4	0	ycaM	5	8.13333	0.404
0.4	0	yadI	5	8.13333	0.48
0.4	0	AG1	5	8.13333	0.474
0.4	0	ptsI	5	8.38333	0.356
0.4	0	clcB	5	8.38333	0.431
0.4	0	ycaM	5	8.38333	0.415
0.4	0	yadI	5	8.38333	0.493
0.4	0	AG1	5	8.38333	0.487
0.4	0	ptsI	5	8.88333	0.325
0.4	0	clcB	5	8.88333	0.352
0.4	0	ycaM	5	8.88333	0.337
0.4	0	yadI	5	8.88333	0.39
0.4	0	AG1	5	8.88333	0.478
0.4	0	ptsI	5	9.13333	0.333
0.4	0	clcB	5	9.13333	0.364
0.4	0	ycaM	5	9.13333	0.344
0.4	0	yadI	5	9.13333	0.39
0.4	0	AG1	5	9.13333	0.476

0.4	0	ptsI	5	9.38333	0.344
0.4	0	clcB	5	9.38333	0.376
0.4	0	ycaM	5	9.38333	0.355
0.4	0	yadI	5	9.38333	0.399
0.4	0	AG1	5	9.38333	0.483
0.4	0	ptsI	5	9.63333	0.353
0.4	0	clcB	5	9.63333	0.384
0.4	0	ycaM	5	9.63333	0.361
0.4	0	yadI	5	9.63333	0.412
0.4	0	AG1	5	9.63333	0.503
0.4	0	ptsI	5	9.88333	0.362
0.4	0	clcB	5	9.88333	0.392
0.4	0	ycaM	5	9.88333	0.37
0.4	0	yadI	5	9.88333	0.42
0.4	0	AG1	5	9.88333	0.513
0.4	0	ptsI	5	10.1333	0.372
0.4	0	clcB	5	10.1333	0.398
0.4	0	ycaM	5	10.1333	0.379
0.4	0	yadI	5	10.1333	0.428
0.4	0	AG1	5	10.1333	0.52
0.4	0	ptsI	5	10.3833	0.386
0.4	0	clcB	5	10.3833	0.408
0.4	0	ycaM	5	10.3833	0.386
0.4	0	yadI	5	10.3833	0.438
0.4	0	AG1	5	10.3833	0.531
0.4	0	ptsI	5	10.6333	0.394
0.4	0	clcB	5	10.6333	0.412
0.4	0	ycaM	5	10.6333	0.392
0.4	0	yadI	5	10.6333	0.441
0.4	0	AG1	5	10.6333	0.539
0.4	0	ptsI	5	10.8833	0.406
0.4	0	clcB	5	10.8833	0.422
0.4	0	ycaM	5	10.8833	0.398
0.4	0	yadI	5	10.8833	0.452
0.4	0	AG1	5	10.8833	0.548
0.4	0	ptsI	5	11.1333	0.409
0.4	0	clcB	5	11.1333	0.421
0.4	0	ycaM	5	11.1333	0.399
0.4	0	yadI	5	11.1333	0.454
0.4	0	AG1	5	11.1333	0.556
0.4	0	ptsI	5	11.3833	0.418
0.4	0	clcB	5	11.3833	0.426
0.4	0	ycaM	5	11.3833	0.402
0.4	0	yadI	5	11.3833	0.464
0.4	0	AG1	5	11.3833	0.565
0.4	0	ptsI	5	11.6333	0.428
0.4	0	clcB	5	11.6333	0.429
0.4	0	ycaM	5	11.6333	0.403
0.4	0	yadI	5	11.6333	0.47
0.4	0	AG1	5	11.6333	0.57
0.4	0	ptsI	5	11.8833	0.435
0.4	0	clcB	5	11.8833	0.437
0.4	0	ycaM	5	11.8833	0.407

0.4	0	yadI	5	11.8833	0.477
0.4	0	AG1	5	11.8833	0.581
0.4	0	ptsI	5	12.1333	0.447
0.4	0	clcB	5	12.1333	0.444
0.4	0	ycaM	5	12.1333	0.411
0.4	0	yadI	5	12.1333	0.486
0.4	0	AG1	5	12.1333	0.589
0.4	0	ptsI	5	12.3833	0.45
0.4	0	clcB	5	12.3833	0.443
0.4	0	ycaM	5	12.3833	0.412
0.4	0	yadI	5	12.3833	0.488
0.4	0	AG1	5	12.3833	0.594
0.4	0	ptsI	5	12.6333	0.458
0.4	0	clcB	5	12.6333	0.45
0.4	0	ycaM	5	12.6333	0.416
0.4	0	yadI	5	12.6333	0.496
0.4	0	AG1	5	12.6333	0.602
0.4	0	ptsI	5	12.8833	0.47
0.4	0	clcB	5	12.8833	0.459
0.4	0	ycaM	5	12.8833	0.424
0.4	0	yadI	5	12.8833	0.503
0.4	0	AG1	5	12.8833	0.611
0.4	0	ptsI	5	13.1333	0.478
0.4	0	clcB	5	13.1333	0.461
0.4	0	ycaM	5	13.1333	0.425
0.4	0	yadI	5	13.1333	0.506
0.4	0	AG1	5	13.1333	0.617
0.4	0	ptsI	5	13.3833	0.479
0.4	0	clcB	5	13.3833	0.467
0.4	0	ycaM	5	13.3833	0.428
0.4	0	yadI	5	13.3833	0.509
0.4	0	AG1	5	13.3833	0.622
0.4	0	ptsI	5	13.6333	0.491
0.4	0	clcB	5	13.6333	0.47
0.4	0	ycaM	5	13.6333	0.432
0.4	0	yadI	5	13.6333	0.517
0.4	0	AG1	5	13.6333	0.631
0.4	0	ptsI	5	13.8833	0.496
0.4	0	clcB	5	13.8833	0.473
0.4	0	ycaM	5	13.8833	0.435
0.4	0	yadI	5	13.8833	0.519
0.4	0	AG1	5	13.8833	0.636
0.4	0	ptsI	5	14.1333	0.506
0.4	0	clcB	5	14.1333	0.479
0.4	0	ycaM	5	14.1333	0.441
0.4	0	yadI	5	14.1333	0.526
0.4	0	AG1	5	14.1333	0.64
0.4	0	ptsI	5	14.3833	0.514
0.4	0	clcB	5	14.3833	0.484
0.4	0	ycaM	5	14.3833	0.444
0.4	0	yadI	5	14.3833	0.529
0.4	0	AG1	5	14.3833	0.646
0.4	0	ptsI	5	14.6333	0.519

0.4	0	clcB	5	14.6333	0.488
0.4	0	ycaM	5	14.6333	0.447
0.4	0	yadI	5	14.6333	0.533
0.4	0	AG1	5	14.6333	0.65
0.4	0	ptsI	5	14.8833	0.524
0.4	0	clcB	5	14.8833	0.49
0.4	0	ycaM	5	14.8833	0.45
0.4	0	yadI	5	14.8833	0.537
0.4	0	AG1	5	14.8833	0.655
0.4	0	ptsI	5	15.1333	0.532
0.4	0	clcB	5	15.1333	0.495
0.4	0	ycaM	5	15.1333	0.455
0.4	0	yadI	5	15.1333	0.543
0.4	0	AG1	5	15.1333	0.661
0.4	0	ptsI	5	15.3833	0.538
0.4	0	clcB	5	15.3833	0.496
0.4	0	ycaM	5	15.3833	0.457
0.4	0	yadI	5	15.3833	0.544
0.4	0	AG1	5	15.3833	0.664
0.4	0	ptsI	5	15.6333	0.543
0.4	0	clcB	5	15.6333	0.498
0.4	0	ycaM	5	15.6333	0.461
0.4	0	yadI	5	15.6333	0.547
0.4	0	AG1	5	15.6333	0.667
0.4	0	ptsI	5	15.8833	0.547
0.4	0	clcB	5	15.8833	0.5
0.4	0	ycaM	5	15.8833	0.464
0.4	0	yadI	5	15.8833	0.551
0.4	0	AG1	5	15.8833	0.67
0.4	0	ptsI	5	16.1333	0.553
0.4	0	clcB	5	16.1333	0.505
0.4	0	ycaM	5	16.1333	0.472
0.4	0	yadI	5	16.1333	0.559
0.4	0	AG1	5	16.1333	0.667
0.4	0	ptsI	5	16.3833	0.552
0.4	0	clcB	5	16.3833	0.503
0.4	0	ycaM	5	16.3833	0.469
0.4	0	yadI	5	16.3833	0.557
0.4	0	AG1	5	16.3833	0.678
0.4	0	ptsI	5	16.6333	0.557
0.4	0	clcB	5	16.6333	0.507
0.4	0	ycaM	5	16.6333	0.476
0.4	0	yadI	5	16.6333	0.562
0.4	0	AG1	5	16.6333	0.681
0.4	0	ptsI	5	16.8833	0.561
0.4	0	clcB	5	16.8833	0.511
0.4	0	ycaM	5	16.8833	0.478
0.4	0	yadI	5	16.8833	0.562
0.4	0	AG1	5	16.8833	0.683
0.4	0	ptsI	5	17.1333	0.567
0.4	0	clcB	5	17.1333	0.513
0.4	0	ycaM	5	17.1333	0.481
0.4	0	yadI	5	17.1333	0.565

0.4	0	AG1	5	17.1333	0.687
0.4	0	ptsI	5	17.3833	0.567
0.4	0	clcB	5	17.3833	0.512
0.4	0	ycaM	5	17.3833	0.484
0.4	0	yadI	5	17.3833	0.567
0.4	0	AG1	5	17.3833	0.689
0.4	0	ptsI	5	17.6333	0.573
0.4	0	clcB	5	17.6333	0.515
0.4	0	ycaM	5	17.6333	0.487
0.4	0	yadI	5	17.6333	0.568
0.4	0	AG1	5	17.6333	0.693
0.4	0	ptsI	5	17.8833	0.572
0.4	0	clcB	5	17.8833	0.514
0.4	0	ycaM	5	17.8833	0.489
0.4	0	yadI	5	17.8833	0.567
0.4	0	AG1	5	17.8833	0.692
0.4	0	ptsI	5	18.1333	0.58
0.4	0	clcB	5	18.1333	0.52
0.4	0	ycaM	5	18.1333	0.491
0.4	0	yadI	5	18.1333	0.576
0.4	0	AG1	5	18.1333	0.696
0.4	0	ptsI	5	18.3833	0.582
0.4	0	clcB	5	18.3833	0.52
0.4	0	ycaM	5	18.3833	0.494
0.4	0	yadI	5	18.3833	0.577
0.4	0	AG1	5	18.3833	0.702
0.4	0	ptsI	5	18.6333	0.586
0.4	0	clcB	5	18.6333	0.522
0.4	0	ycaM	5	18.6333	0.497
0.4	0	yadI	5	18.6333	0.577
0.4	0	AG1	5	18.6333	0.705
0.4	0	ptsI	5	18.8833	0.584
0.4	0	clcB	5	18.8833	0.521
0.4	0	ycaM	5	18.8833	0.499
0.4	0	yadI	5	18.8833	0.582
0.4	0	AG1	5	18.8833	0.704
0.4	0	ptsI	5	19.1333	0.588
0.4	0	clcB	5	19.1333	0.524
0.4	0	ycaM	5	19.1333	0.501
0.4	0	yadI	5	19.1333	0.58
0.4	0	AG1	5	19.1333	0.706
0.4	0	ptsI	5	19.3833	0.596
0.4	0	clcB	5	19.3833	0.526
0.4	0	ycaM	5	19.3833	0.503
0.4	0	yadI	5	19.3833	0.587
0.4	0	AG1	5	19.3833	0.707
0.4	0	ptsI	5	19.6333	0.595
0.4	0	clcB	5	19.6333	0.529
0.4	0	ycaM	5	19.6333	0.504
0.4	0	yadI	5	19.6333	0.591
0.4	0	AG1	5	19.6333	0.71
0.4	0	ptsI	5	19.8833	0.595
0.4	0	clcB	5	19.8833	0.527

0.4	0	ycaM	5	19.8833	0.507
0.4	0	yadI	5	19.8833	0.588
0.4	0	AG1	5	19.8833	0.71
0.4	0	ptsI	5	20.1333	0.597
0.4	0	clcB	5	20.1333	0.528
0.4	0	ycaM	5	20.1333	0.508
0.4	0	yadI	5	20.1333	0.591
0.4	0	AG1	5	20.1333	0.714
0.4	0	ptsI	5	20.3833	0.603
0.4	0	clcB	5	20.3833	0.53
0.4	0	ycaM	5	20.3833	0.509
0.4	0	yadI	5	20.3833	0.595
0.4	0	AG1	5	20.3833	0.714
0.4	0	ptsI	5	20.6333	0.604
0.4	0	clcB	5	20.6333	0.528
0.4	0	ycaM	5	20.6333	0.51
0.4	0	yadI	5	20.6333	0.592
0.4	0	AG1	5	20.6333	0.723
0.4	0	ptsI	5	20.8833	0.611
0.4	0	clcB	5	20.8833	0.528
0.4	0	ycaM	5	20.8833	0.513
0.4	0	yadI	5	20.8833	0.596
0.4	0	AG1	5	20.8833	0.716
0.4	0	ptsI	5	21.1333	0.611
0.4	0	clcB	5	21.1333	0.53
0.4	0	ycaM	5	21.1333	0.515
0.4	0	yadI	5	21.1333	0.595
0.4	0	AG1	5	21.1333	0.718
0.4	0	ptsI	5	21.3833	0.616
0.4	0	clcB	5	21.3833	0.531
0.4	0	ycaM	5	21.3833	0.516
0.4	0	yadI	5	21.3833	0.597
0.4	0	AG1	5	21.3833	0.719
0.4	0	ptsI	5	21.6333	0.619
0.4	0	clcB	5	21.6333	0.534
0.4	0	ycaM	5	21.6333	0.52
0.4	0	yadI	5	21.6333	0.607
0.4	0	AG1	5	21.6333	0.722
0.4	0	ptsI	5	21.8833	0.619
0.4	0	clcB	5	21.8833	0.535
0.4	0	ycaM	5	21.8833	0.519
0.4	0	yadI	5	21.8833	0.607
0.4	0	AG1	5	21.8833	0.721
0.4	0	ptsI	5	22.1333	0.619
0.4	0	clcB	5	22.1333	0.53
0.4	0	ycaM	5	22.1333	0.521
0.4	0	yadI	5	22.1333	0.602
0.4	0	AG1	5	22.1333	0.72
0.4	0	ptsI	5	22.3833	0.625
0.4	0	clcB	5	22.3833	0.533
0.4	0	ycaM	5	22.3833	0.523
0.4	0	yadI	5	22.3833	0.605
0.4	0	AG1	5	22.3833	0.722

0.4	0	ptsI	5	22.6333	0.626
0.4	0	clcB	5	22.6333	0.535
0.4	0	ycaM	5	22.6333	0.527
0.4	0	yadI	5	22.6333	0.615
0.4	0	AG1	5	22.6333	0.727
0.4	0	ptsI	5	22.8833	0.627
0.4	0	clcB	5	22.8833	0.535
0.4	0	ycaM	5	22.8833	0.526
0.4	0	yadI	5	22.8833	0.613
0.4	0	AG1	5	22.8833	0.724
0.4	0	ptsI	5	23.1333	0.631
0.4	0	clcB	5	23.1333	0.535
0.4	0	ycaM	5	23.1333	0.528
0.4	0	yadI	5	23.1333	0.611
0.4	0	AG1	5	23.1333	0.726
0.4	0	ptsI	5	23.3833	0.632
0.4	0	clcB	5	23.3833	0.535
0.4	0	ycaM	5	23.3833	0.525
0.4	0	yadI	5	23.3833	0.61
0.4	0	AG1	5	23.3833	0.725
0.4	0	ptsI	5	23.6333	0.633
0.4	0	clcB	5	23.6333	0.536
0.4	0	ycaM	5	23.6333	0.527
0.4	0	yadI	5	23.6333	0.607
0.4	0	AG1	5	23.6333	0.728
0.4	0	ptsI	5	23.8833	0.63
0.4	0	clcB	5	23.8833	0.531
0.4	0	ycaM	5	23.8833	0.528
0.4	0	yadI	5	23.8833	0.609
0.4	0	AG1	5	23.8833	0.723
0.4	0	ptsI	5	24.1333	0.64
0.4	0	clcB	5	24.1333	0.538
0.4	0	ycaM	5	24.1333	0.531
0.4	0	yadI	5	24.1333	0.618
0.4	0	AG1	5	24.1333	0.73
0.4	0	ptsI	5	24.3833	0.642
0.4	0	clcB	5	24.3833	0.535
0.4	0	ycaM	5	24.3833	0.53
0.4	0	yadI	5	24.3833	0.615
0.4	0	AG1	5	24.3833	0.731
0.8	0	ptsI	1	0	0.253
0.8	0	clcB	1	0	0.217
0.8	0	ycaM	1	0	0.233
0.8	0	yadI	1	0	0.247
0.8	0	AG1	1	0	0.231
0.8	0	ptsI	1	0.25	0.24
0.8	0	clcB	1	0.25	0.21
0.8	0	ycaM	1	0.25	0.219
0.8	0	yadI	1	0.25	0.237
0.8	0	AG1	1	0.25	0.223
0.8	0	ptsI	1	0.5	0.24
0.8	0	clcB	1	0.5	0.206
0.8	0	ycaM	1	0.5	0.213

0.8	0	yadI	1	0.5	0.233
0.8	0	AG1	1	0.5	0.221
0.8	0	ptsI	1	0.75	0.241
0.8	0	clcB	1	0.75	0.209
0.8	0	ycaM	1	0.75	0.213
0.8	0	yadI	1	0.75	0.232
0.8	0	AG1	1	0.75	0.221
0.8	0	ptsI	1	1	0.244
0.8	0	clcB	1	1	0.209
0.8	0	ycaM	1	1	0.214
0.8	0	yadI	1	1	0.235
0.8	0	AG1	1	1	0.224
0.8	0	ptsI	1	1.25	0.244
0.8	0	clcB	1	1.25	0.208
0.8	0	ycaM	1	1.25	0.213
0.8	0	yadI	1	1.25	0.239
0.8	0	AG1	1	1.25	0.226
0.8	0	ptsI	1	1.5	0.254
0.8	0	clcB	1	1.5	0.21
0.8	0	ycaM	1	1.5	0.217
0.8	0	yadI	1	1.5	0.242
0.8	0	AG1	1	1.5	0.228
0.8	0	ptsI	1	1.75	0.255
0.8	0	clcB	1	1.75	0.214
0.8	0	ycaM	1	1.75	0.221
0.8	0	yadI	1	1.75	0.249
0.8	0	AG1	1	1.75	0.231
0.8	0	ptsI	1	2	0.264
0.8	0	clcB	1	2	0.218
0.8	0	ycaM	1	2	0.224
0.8	0	yadI	1	2	0.254
0.8	0	AG1	1	2	0.236
0.8	0	ptsI	1	2.25	0.266
0.8	0	clcB	1	2.25	0.221
0.8	0	ycaM	1	2.25	0.23
0.8	0	yadI	1	2.25	0.266
0.8	0	AG1	1	2.25	0.242
0.8	0	ptsI	1	2.5	0.276
0.8	0	clcB	1	2.5	0.227
0.8	0	ycaM	1	2.5	0.234
0.8	0	yadI	1	2.5	0.272
0.8	0	AG1	1	2.5	0.249
0.8	0	ptsI	1	2.75	0.281
0.8	0	clcB	1	2.75	0.23
0.8	0	ycaM	1	2.75	0.243
0.8	0	yadI	1	2.75	0.292
0.8	0	AG1	1	2.75	0.255
0.8	0	ptsI	1	3	0.294
0.8	0	clcB	1	3	0.238
0.8	0	ycaM	1	3	0.248
0.8	0	yadI	1	3	0.3
0.8	0	AG1	1	3	0.26
0.8	0	ptsI	1	3.25	0.304

0.8	0	clcB	1	3.25	0.245
0.8	0	ycaM	1	3.25	0.256
0.8	0	yadI	1	3.25	0.319
0.8	0	AG1	1	3.25	0.267
0.8	0	ptsI	1	3.5	0.311
0.8	0	clcB	1	3.5	0.251
0.8	0	ycaM	1	3.5	0.264
0.8	0	yadI	1	3.5	0.338
0.8	0	AG1	1	3.5	0.276
0.8	0	ptsI	1	3.75	0.323
0.8	0	clcB	1	3.75	0.254
0.8	0	ycaM	1	3.75	0.271
0.8	0	yadI	1	3.75	0.366
0.8	0	AG1	1	3.75	0.274
0.8	0	ptsI	1	4	0.339
0.8	0	clcB	1	4	0.26
0.8	0	ycaM	1	4	0.285
0.8	0	yadI	1	4	0.373
0.8	0	AG1	1	4	0.283
0.8	0	ptsI	1	4.25	0.346
0.8	0	clcB	1	4.25	0.266
0.8	0	ycaM	1	4.25	0.294
0.8	0	yadI	1	4.25	0.386
0.8	0	AG1	1	4.25	0.293
0.8	0	ptsI	1	4.5	0.352
0.8	0	clcB	1	4.5	0.275
0.8	0	ycaM	1	4.5	0.301
0.8	0	yadI	1	4.5	0.404
0.8	0	AG1	1	4.5	0.308
0.8	0	ptsI	1	4.75	0.36
0.8	0	clcB	1	4.75	0.281
0.8	0	ycaM	1	4.75	0.306
0.8	0	yadI	1	4.75	0.4
0.8	0	AG1	1	4.75	0.313
0.8	0	ptsI	1	5	0.375
0.8	0	clcB	1	5	0.289
0.8	0	ycaM	1	5	0.315
0.8	0	yadI	1	5	0.436
0.8	0	AG1	1	5	0.325
0.8	0	ptsI	1	5.25	0.386
0.8	0	clcB	1	5.25	0.299
0.8	0	ycaM	1	5.25	0.326
0.8	0	yadI	1	5.25	0.445
0.8	0	AG1	1	5.25	0.332
0.8	0	ptsI	1	5.5	0.395
0.8	0	clcB	1	5.5	0.308
0.8	0	ycaM	1	5.5	0.333
0.8	0	yadI	1	5.5	0.456
0.8	0	AG1	1	5.5	0.345
0.8	0	ptsI	1	5.75	0.404
0.8	0	clcB	1	5.75	0.316
0.8	0	ycaM	1	5.75	0.356
0.8	0	yadI	1	5.75	0.47

0.8	0	AG1	1	5.75	0.356
0.8	0	ptsI	1	6	0.416
0.8	0	clcB	1	6	0.326
0.8	0	ycaM	1	6	0.346
0.8	0	yadI	1	6	0.475
0.8	0	AG1	1	6	0.364
0.8	0	ptsI	1	6.25	0.431
0.8	0	clcB	1	6.25	0.338
0.8	0	ycaM	1	6.25	0.359
0.8	0	yadI	1	6.25	0.488
0.8	0	AG1	1	6.25	0.37
0.8	0	ptsI	1	6.5	0.439
0.8	0	clcB	1	6.5	0.348
0.8	0	ycaM	1	6.5	0.371
0.8	0	yadI	1	6.5	0.498
0.8	0	AG1	1	6.5	0.384
0.8	0	ptsI	1	6.75	0.454
0.8	0	clcB	1	6.75	0.357
0.8	0	ycaM	1	6.75	0.381
0.8	0	yadI	1	6.75	0.523
0.8	0	AG1	1	6.75	0.4
0.8	0	ptsI	1	7	0.462
0.8	0	clcB	1	7	0.367
0.8	0	ycaM	1	7	0.391
0.8	0	yadI	1	7	0.539
0.8	0	AG1	1	7	0.414
0.8	0	ptsI	1	7.25	0.472
0.8	0	clcB	1	7.25	0.374
0.8	0	ycaM	1	7.25	0.4
0.8	0	yadI	1	7.25	0.55
0.8	0	AG1	1	7.25	0.422
0.8	0	ptsI	1	7.5	0.475
0.8	0	clcB	1	7.5	0.384
0.8	0	ycaM	1	7.5	0.41
0.8	0	yadI	1	7.5	0.571
0.8	0	AG1	1	7.5	0.431
0.8	0	ptsI	1	7.75	0.487
0.8	0	clcB	1	7.75	0.391
0.8	0	ycaM	1	7.75	0.417
0.8	0	yadI	1	7.75	0.582
0.8	0	AG1	1	7.75	0.441
0.8	0	ptsI	1	8	0.502
0.8	0	clcB	1	8	0.399
0.8	0	ycaM	1	8	0.424
0.8	0	yadI	1	8	0.591
0.8	0	AG1	1	8	0.451
0.8	0	ptsI	1	8.25	0.509
0.8	0	clcB	1	8.25	0.408
0.8	0	ycaM	1	8.25	0.433
0.8	0	yadI	1	8.25	0.607
0.8	0	AG1	1	8.25	0.461
0.8	0	ptsI	1	8.5	0.525
0.8	0	clcB	1	8.5	0.419

0.8	0	ycaM	1	8.5	0.437
0.8	0	yadI	1	8.5	0.63
0.8	0	AG1	1	8.5	0.472
0.8	0	ptsI	1	8.75	0.539
0.8	0	clcB	1	8.75	0.421
0.8	0	ycaM	1	8.75	0.442
0.8	0	yadI	1	8.75	0.628
0.8	0	AG1	1	8.75	0.479
0.8	0	ptsI	1	9	0.557
0.8	0	clcB	1	9	0.428
0.8	0	ycaM	1	9	0.446
0.8	0	yadI	1	9	0.644
0.8	0	AG1	1	9	0.492
0.8	0	ptsI	1	9.25	0.576
0.8	0	clcB	1	9.25	0.443
0.8	0	ycaM	1	9.25	0.454
0.8	0	yadI	1	9.25	0.635
0.8	0	AG1	1	9.25	0.501
0.8	0	ptsI	1	9.5	0.611
0.8	0	clcB	1	9.5	0.463
0.8	0	ycaM	1	9.5	0.466
0.8	0	yadI	1	9.5	0.65
0.8	0	AG1	1	9.5	0.524
0.8	0	ptsI	1	9.75	0.607
0.8	0	clcB	1	9.75	0.465
0.8	0	ycaM	1	9.75	0.477
0.8	0	yadI	1	9.75	0.678
0.8	0	AG1	1	9.75	0.541
0.8	0	ptsI	1	10	0.611
0.8	0	clcB	1	10	0.473
0.8	0	ycaM	1	10	0.478
0.8	0	yadI	1	10	0.665
0.8	0	AG1	1	10	0.54
0.8	0	ptsI	1	10.25	0.617
0.8	0	clcB	1	10.25	0.478
0.8	0	ycaM	1	10.25	0.495
0.8	0	yadI	1	10.25	0.688
0.8	0	AG1	1	10.25	0.559
0.8	0	ptsI	1	10.5	0.634
0.8	0	clcB	1	10.5	0.481
0.8	0	ycaM	1	10.5	0.505
0.8	0	yadI	1	10.5	0.708
0.8	0	AG1	1	10.5	0.571
0.8	0	ptsI	1	10.75	0.685
0.8	0	clcB	1	10.75	0.499
0.8	0	ycaM	1	10.75	0.506
0.8	0	yadI	1	10.75	0.694
0.8	0	AG1	1	10.75	0.568
0.8	0	ptsI	1	11	0.68
0.8	0	clcB	1	11	0.52
0.8	0	ycaM	1	11	0.511
0.8	0	yadI	1	11	0.708
0.8	0	AG1	1	11	0.611

0.8	0	ptsI	1	11.25	0.677
0.8	0	clcB	1	11.25	0.527
0.8	0	ycaM	1	11.25	0.524
0.8	0	yadI	1	11.25	0.714
0.8	0	AG1	1	11.25	0.617
0.8	0	ptsI	1	11.5	0.728
0.8	0	clcB	1	11.5	0.54
0.8	0	ycaM	1	11.5	0.526
0.8	0	yadI	1	11.5	0.725
0.8	0	AG1	1	11.5	0.63
0.8	0	ptsI	1	11.75	0.753
0.8	0	clcB	1	11.75	0.56
0.8	0	ycaM	1	11.75	0.539
0.8	0	yadI	1	11.75	0.753
0.8	0	AG1	1	11.75	0.667
0.8	0	ptsI	1	12	0.741
0.8	0	clcB	1	12	0.558
0.8	0	ycaM	1	12	0.549
0.8	0	yadI	1	12	0.762
0.8	0	AG1	1	12	0.675
0.8	0	ptsI	1	12.25	0.774
0.8	0	clcB	1	12.25	0.578
0.8	0	ycaM	1	12.25	0.562
0.8	0	yadI	1	12.25	0.769
0.8	0	AG1	1	12.25	0.686
0.8	0	ptsI	1	12.5	0.766
0.8	0	clcB	1	12.5	0.573
0.8	0	ycaM	1	12.5	0.572
0.8	0	yadI	1	12.5	0.77
0.8	0	AG1	1	12.5	0.684
0.8	0	ptsI	1	12.75	0.766
0.8	0	clcB	1	12.75	0.579
0.8	0	ycaM	1	12.75	0.57
0.8	0	yadI	1	12.75	0.777
0.8	0	AG1	1	12.75	0.713
0.8	0	ptsI	1	13	0.78
0.8	0	clcB	1	13	0.582
0.8	0	ycaM	1	13	0.566
0.8	0	yadI	1	13	0.779
0.8	0	AG1	1	13	0.727
0.8	0	ptsI	1	13.25	0.812
0.8	0	clcB	1	13.25	0.587
0.8	0	ycaM	1	13.25	0.584
0.8	0	yadI	1	13.25	0.799
0.8	0	AG1	1	13.25	0.738
0.8	0	ptsI	1	13.5	0.812
0.8	0	clcB	1	13.5	0.6
0.8	0	ycaM	1	13.5	0.591
0.8	0	yadI	1	13.5	0.803
0.8	0	AG1	1	13.5	0.737
0.8	0	ptsI	1	13.75	0.821
0.8	0	clcB	1	13.75	0.605
0.8	0	ycaM	1	13.75	0.598

0.8	0	yadI	1	13.75	0.786
0.8	0	AG1	1	13.75	0.738
0.8	0	ptsI	1	14	0.831
0.8	0	clcB	1	14	0.605
0.8	0	ycaM	1	14	0.597
0.8	0	yadI	1	14	0.804
0.8	0	AG1	1	14	0.735
0.8	0	ptsI	1	14.25	0.803
0.8	0	clcB	1	14.25	0.612
0.8	0	ycaM	1	14.25	0.61
0.8	0	yadI	1	14.25	0.801
0.8	0	AG1	1	14.25	0.744
0.8	0	ptsI	1	14.5	0.803
0.8	0	clcB	1	14.5	0.626
0.8	0	ycaM	1	14.5	0.62
0.8	0	yadI	1	14.5	0.802
0.8	0	AG1	1	14.5	0.772
0.8	0	ptsI	1	14.75	0.836
0.8	0	clcB	1	14.75	0.634
0.8	0	ycaM	1	14.75	0.634
0.8	0	yadI	1	14.75	0.83
0.8	0	AG1	1	14.75	0.779
0.8	0	ptsI	1	15	0.843
0.8	0	clcB	1	15	0.638
0.8	0	ycaM	1	15	0.628
0.8	0	yadI	1	15	0.826
0.8	0	AG1	1	15	0.797
0.8	0	ptsI	1	15.25	0.853
0.8	0	clcB	1	15.25	0.67
0.8	0	ycaM	1	15.25	0.638
0.8	0	yadI	1	15.25	0.827
0.8	0	AG1	1	15.25	0.812
0.8	0	ptsI	1	15.5	0.852
0.8	0	clcB	1	15.5	0.659
0.8	0	ycaM	1	15.5	0.652
0.8	0	yadI	1	15.5	0.82
0.8	0	AG1	1	15.5	0.804
0.8	0	ptsI	1	15.75	0.854
0.8	0	clcB	1	15.75	0.665
0.8	0	ycaM	1	15.75	0.658
0.8	0	yadI	1	15.75	0.841
0.8	0	AG1	1	15.75	0.808
0.8	0	ptsI	1	16	0.864
0.8	0	clcB	1	16	0.672
0.8	0	ycaM	1	16	0.664
0.8	0	yadI	1	16	0.83
0.8	0	AG1	1	16	0.817
0.8	0	ptsI	1	16.25	0.863
0.8	0	clcB	1	16.25	0.654
0.8	0	ycaM	1	16.25	0.684
0.8	0	yadI	1	16.25	0.826
0.8	0	AG1	1	16.25	0.829
0.8	0	ptsI	1	16.5	0.876

0.8	0	clcB	1	16.5	0.683
0.8	0	ycaM	1	16.5	0.685
0.8	0	yadI	1	16.5	0.831
0.8	0	AG1	1	16.5	0.845
0.8	0	ptsI	1	17.0667	0.869
0.8	0	clcB	1	17.0667	0.706
0.8	0	ycaM	1	17.0667	0.705
0.8	0	yadI	1	17.0667	0.882
0.8	0	AG1	1	17.0667	0.828
0.8	0	ptsI	1	17.3167	0.823
0.8	0	clcB	1	17.3167	0.673
0.8	0	ycaM	1	17.3167	0.684
0.8	0	yadI	1	17.3167	0.843
0.8	0	AG1	1	17.3167	0.803
0.8	0	ptsI	1	17.5667	0.836
0.8	0	clcB	1	17.5667	0.679
0.8	0	ycaM	1	17.5667	0.684
0.8	0	yadI	1	17.5667	0.83
0.8	0	AG1	1	17.5667	0.831
0.8	0	ptsI	1	17.8167	0.865
0.8	0	clcB	1	17.8167	0.744
0.8	0	ycaM	1	17.8167	0.717
0.8	0	yadI	1	17.8167	0.836
0.8	0	AG1	1	17.8167	0.816
0.8	0	ptsI	1	18.0667	0.863
0.8	0	clcB	1	18.0667	0.713
0.8	0	ycaM	1	18.0667	0.71
0.8	0	yadI	1	18.0667	0.842
0.8	0	AG1	1	18.0667	0.801
0.8	0	ptsI	1	18.3167	0.866
0.8	0	clcB	1	18.3167	0.72
0.8	0	ycaM	1	18.3167	0.714
0.8	0	yadI	1	18.3167	0.836
0.8	0	AG1	1	18.3167	0.822
0.8	0	ptsI	1	18.5667	0.879
0.8	0	clcB	1	18.5667	0.726
0.8	0	ycaM	1	18.5667	0.734
0.8	0	yadI	1	18.5667	0.85
0.8	0	AG1	1	18.5667	0.832
0.8	0	ptsI	1	18.8167	0.885
0.8	0	clcB	1	18.8167	0.73
0.8	0	ycaM	1	18.8167	0.731
0.8	0	yadI	1	18.8167	0.849
0.8	0	AG1	1	18.8167	0.826
0.8	0	ptsI	1	19.0667	0.895
0.8	0	clcB	1	19.0667	0.712
0.8	0	ycaM	1	19.0667	0.734
0.8	0	yadI	1	19.0667	0.863
0.8	0	AG1	1	19.0667	0.836
0.8	0	ptsI	1	19.3167	0.903
0.8	0	clcB	1	19.3167	0.707
0.8	0	ycaM	1	19.3167	0.738
0.8	0	yadI	1	19.3167	0.857

0.8	0	AG1	1	19.3167	0.829
0.8	0	ptsI	1	19.5667	0.91
0.8	0	clcB	1	19.5667	0.72
0.8	0	ycaM	1	19.5667	0.737
0.8	0	yadI	1	19.5667	0.864
0.8	0	AG1	1	19.5667	0.834
0.8	0	ptsI	1	19.8167	0.919
0.8	0	clcB	1	19.8167	0.713
0.8	0	ycaM	1	19.8167	0.741
0.8	0	yadI	1	19.8167	0.871
0.8	0	AG1	1	19.8167	0.842
0.8	0	ptsI	1	20.0667	0.931
0.8	0	clcB	1	20.0667	0.734
0.8	0	ycaM	1	20.0667	0.747
0.8	0	yadI	1	20.0667	0.876
0.8	0	AG1	1	20.0667	0.837
0.8	0	ptsI	1	20.3167	0.94
0.8	0	clcB	1	20.3167	0.74
0.8	0	ycaM	1	20.3167	0.749
0.8	0	yadI	1	20.3167	0.882
0.8	0	AG1	1	20.3167	0.838
0.8	0	ptsI	1	20.5667	0.944
0.8	0	clcB	1	20.5667	0.742
0.8	0	ycaM	1	20.5667	0.751
0.8	0	yadI	1	20.5667	0.89
0.8	0	AG1	1	20.5667	0.823
0.8	0	ptsI	1	20.8167	0.954
0.8	0	clcB	1	20.8167	0.75
0.8	0	ycaM	1	20.8167	0.744
0.8	0	yadI	1	20.8167	0.888
0.8	0	AG1	1	20.8167	0.814
0.8	0	ptsI	1	21.0667	0.972
0.8	0	clcB	1	21.0667	0.751
0.8	0	ycaM	1	21.0667	0.745
0.8	0	yadI	1	21.0667	0.891
0.8	0	AG1	1	21.0667	0.819
0.8	0	ptsI	1	21.3167	0.977
0.8	0	clcB	1	21.3167	0.751
0.8	0	ycaM	1	21.3167	0.752
0.8	0	yadI	1	21.3167	0.896
0.8	0	AG1	1	21.3167	0.822
0.8	0	ptsI	1	21.5667	0.987
0.8	0	clcB	1	21.5667	0.754
0.8	0	ycaM	1	21.5667	0.755
0.8	0	yadI	1	21.5667	0.904
0.8	0	AG1	1	21.5667	0.823
0.8	0	ptsI	1	21.8167	0.99
0.8	0	clcB	1	21.8167	0.757
0.8	0	ycaM	1	21.8167	0.753
0.8	0	yadI	1	21.8167	0.902
0.8	0	AG1	1	21.8167	0.821
0.8	0	ptsI	1	22.0667	1.003
0.8	0	clcB	1	22.0667	0.763

0.8	0	ycaM	1	22.0667	0.752
0.8	0	yadI	1	22.0667	0.908
0.8	0	AG1	1	22.0667	0.83
0.8	0	ptsI	1	22.9333	1.099
0.8	0	clcB	1	22.9333	0.65
0.8	0	ycaM	1	22.9333	0.684
0.8	0	yadI	1	22.9333	1.006
0.8	0	AG1	1	22.9333	0.726
0.8	0	ptsI	1	23.1833	1.088
0.8	0	clcB	1	23.1833	0.668
0.8	0	ycaM	1	23.1833	0.686
0.8	0	yadI	1	23.1833	0.983
0.8	0	AG1	1	23.1833	0.708
0.8	0	ptsI	1	23.4333	1.084
0.8	0	clcB	1	23.4333	0.697
0.8	0	ycaM	1	23.4333	0.624
0.8	0	yadI	1	23.4333	0.984
0.8	0	AG1	1	23.4333	0.731
0.8	0	ptsI	1	23.6833	1.086
0.8	0	clcB	1	23.6833	0.708
0.8	0	ycaM	1	23.6833	0.666
0.8	0	yadI	1	23.6833	0.98
0.8	0	AG1	1	23.6833	0.744
0.8	0	ptsI	1	23.9333	1.088
0.8	0	clcB	1	23.9333	0.715
0.8	0	ycaM	1	23.9333	0.676
0.8	0	yadI	1	23.9333	0.985
0.8	0	AG1	1	23.9333	0.765
0.8	0	ptsI	1	24.1833	1.089
0.8	0	clcB	1	24.1833	0.727
0.8	0	ycaM	1	24.1833	0.701
0.8	0	yadI	1	24.1833	0.993
0.8	0	AG1	1	24.1833	0.787
0.8	0	ptsI	1	24.4333	1.083
0.8	0	clcB	1	24.4333	0.73
0.8	0	ycaM	1	24.4333	0.728
0.8	0	yadI	1	24.4333	0.996
0.8	0	AG1	1	24.4333	0.81
0.8	0	ptsI	1	24.6833	1.085
0.8	0	clcB	1	24.6833	0.731
0.8	0	ycaM	1	24.6833	0.731
0.8	0	yadI	1	24.6833	1
0.8	0	AG1	1	24.6833	0.802
0.8	0	ptsI	1	24.9333	1.095
0.8	0	clcB	1	24.9333	0.741
0.8	0	ycaM	1	24.9333	0.738
0.8	0	yadI	1	24.9333	1.008
0.8	0	AG1	1	24.9333	0.808
0.8	0	ptsI	1	25.1833	1.094
0.8	0	clcB	1	25.1833	0.744
0.8	0	ycaM	1	25.1833	0.751
0.8	0	yadI	1	25.1833	1.005
0.8	0	AG1	1	25.1833	0.814

0.8	0	ptsI	1	25.4333	1.104
0.8	0	clcB	1	25.4333	0.748
0.8	0	ycaM	1	25.4333	0.77
0.8	0	yadI	1	25.4333	1.014
0.8	0	AG1	1	25.4333	0.823
0.8	0	ptsI	1	25.6833	1.103
0.8	0	clcB	1	25.6833	0.75
0.8	0	ycaM	1	25.6833	0.771
0.8	0	yadI	1	25.6833	1.017
0.8	0	AG1	1	25.6833	0.821
0.8	0	ptsI	1	25.9333	1.103
0.8	0	clcB	1	25.9333	0.766
0.8	0	ycaM	1	25.9333	0.779
0.8	0	yadI	1	25.9333	1.008
0.8	0	AG1	1	25.9333	0.83
0.8	0	ptsI	1	26.1833	1.112
0.8	0	clcB	1	26.1833	0.756
0.8	0	ycaM	1	26.1833	0.801
0.8	0	yadI	1	26.1833	1.019
0.8	0	AG1	1	26.1833	0.835
0.8	0	ptsI	1	26.4333	1.113
0.8	0	clcB	1	26.4333	0.76
0.8	0	ycaM	1	26.4333	0.786
0.8	0	yadI	1	26.4333	1.026
0.8	0	AG1	1	26.4333	0.83
0.8	0	ptsI	1	26.6833	1.121
0.8	0	clcB	1	26.6833	0.77
0.8	0	ycaM	1	26.6833	0.788
0.8	0	yadI	1	26.6833	1.025
0.8	0	AG1	1	26.6833	0.833
0.8	0	ptsI	1	26.9333	1.125
0.8	0	clcB	1	26.9333	0.774
0.8	0	ycaM	1	26.9333	0.798
0.8	0	yadI	1	26.9333	1.022
0.8	0	AG1	1	26.9333	0.839
0.8	0	ptsI	1	27.1833	1.127
0.8	0	clcB	1	27.1833	0.785
0.8	0	ycaM	1	27.1833	0.818
0.8	0	yadI	1	27.1833	1.023
0.8	0	AG1	1	27.1833	0.844
0.8	0	ptsI	1	27.4333	1.132
0.8	0	clcB	1	27.4333	0.78
0.8	0	ycaM	1	27.4333	0.794
0.8	0	yadI	1	27.4333	1.027
0.8	0	AG1	1	27.4333	0.836
0.8	0	ptsI	1	27.6833	1.138
0.8	0	clcB	1	27.6833	0.786
0.8	0	ycaM	1	27.6833	0.803
0.8	0	yadI	1	27.6833	1.03
0.8	0	AG1	1	27.6833	0.848
0.8	0	ptsI	1	27.9333	1.136
0.8	0	clcB	1	27.9333	0.778
0.8	0	ycaM	1	27.9333	0.801

0.8	0	yadI	1	27.9333	1.016
0.8	0	AG1	1	27.9333	0.848
0.8	0	ptsI	1	28.1833	1.137
0.8	0	clcB	1	28.1833	0.788
0.8	0	ycaM	1	28.1833	0.816
0.8	0	yadI	1	28.1833	1.028
0.8	0	AG1	1	28.1833	0.855
0.8	0	ptsI	1	28.4333	1.143
0.8	0	clcB	1	28.4333	0.785
0.8	0	ycaM	1	28.4333	0.814
0.8	0	yadI	1	28.4333	1.03
0.8	0	AG1	1	28.4333	0.855
0.8	0	ptsI	1	28.6833	1.148
0.8	0	clcB	1	28.6833	0.788
0.8	0	ycaM	1	28.6833	0.821
0.8	0	yadI	1	28.6833	1.03
0.8	0	AG1	1	28.6833	0.859
0.8	0	ptsI	1	28.9333	1.153
0.8	0	clcB	1	28.9333	0.787
0.8	0	ycaM	1	28.9333	0.831
0.8	0	yadI	1	28.9333	1.028
0.8	0	AG1	1	28.9333	0.863
0.8	0	ptsI	1	29.1833	1.149
0.8	0	clcB	1	29.1833	0.785
0.8	0	ycaM	1	29.1833	0.828
0.8	0	yadI	1	29.1833	1.03
0.8	0	AG1	1	29.1833	0.859
0.8	0	ptsI	1	29.4333	1.154
0.8	0	clcB	1	29.4333	0.783
0.8	0	ycaM	1	29.4333	0.84
0.8	0	yadI	1	29.4333	1.03
0.8	0	AG1	1	29.4333	0.865
0.8	0	ptsI	1	29.6833	1.158
0.8	0	clcB	1	29.6833	0.783
0.8	0	ycaM	1	29.6833	0.842
0.8	0	yadI	1	29.6833	1.035
0.8	0	AG1	1	29.6833	0.867
0.8	0	ptsI	1	29.9333	1.161
0.8	0	clcB	1	29.9333	0.785
0.8	0	ycaM	1	29.9333	0.856
0.8	0	yadI	1	29.9333	1.036
0.8	0	AG1	1	29.9333	0.873
0.8	0	ptsI	1	30.1833	1.172
0.8	0	clcB	1	30.1833	0.782
0.8	0	ycaM	1	30.1833	0.86
0.8	0	yadI	1	30.1833	1.038
0.8	0	AG1	1	30.1833	0.879
0.8	0	ptsI	1	30.4333	1.169
0.8	0	clcB	1	30.4333	0.783
0.8	0	ycaM	1	30.4333	0.867
0.8	0	yadI	1	30.4333	1.038
0.8	0	AG1	1	30.4333	0.881
0.8	0	ptsI	1	30.6833	1.174

0.8	0	clcB	1	30.6833	0.787
0.8	0	ycaM	1	30.6833	0.872
0.8	0	yadI	1	30.6833	1.047
0.8	0	AG1	1	30.6833	0.885
0.8	0	ptsI	1	30.9333	1.176
0.8	0	clcB	1	30.9333	0.784
0.8	0	ycaM	1	30.9333	0.884
0.8	0	yadI	1	30.9333	1.045
0.8	0	AG1	1	30.9333	0.891
0.8	0	ptsI	1	31.1833	1.19
0.8	0	clcB	1	31.1833	0.783
0.8	0	ycaM	1	31.1833	0.892
0.8	0	yadI	1	31.1833	1.048
0.8	0	AG1	1	31.1833	0.892
0.8	0	ptsI	1	31.4333	1.193
0.8	0	clcB	1	31.4333	0.787
0.8	0	ycaM	1	31.4333	0.905
0.8	0	yadI	1	31.4333	1.05
0.8	0	AG1	1	31.4333	0.901
0.8	0	ptsI	1	31.6833	1.2
0.8	0	clcB	1	31.6833	0.785
0.8	0	ycaM	1	31.6833	0.911
0.8	0	yadI	1	31.6833	1.049
0.8	0	AG1	1	31.6833	0.906
0.8	0	ptsI	1	31.9333	1.206
0.8	0	clcB	1	31.9333	0.786
0.8	0	ycaM	1	31.9333	0.923
0.8	0	yadI	1	31.9333	1.055
0.8	0	AG1	1	31.9333	0.908
0.8	0	ptsI	1	32.1833	1.205
0.8	0	clcB	1	32.1833	0.788
0.8	0	ycaM	1	32.1833	0.927
0.8	0	yadI	1	32.1833	1.052
0.8	0	AG1	1	32.1833	0.916
0.8	0	ptsI	1	32.4333	1.222
0.8	0	clcB	1	32.4333	0.8
0.8	0	ycaM	1	32.4333	0.934
0.8	0	yadI	1	32.4333	1.034
0.8	0	AG1	1	32.4333	0.92
0.8	0	ptsI	1	32.6833	1.226
0.8	0	clcB	1	32.6833	0.794
0.8	0	ycaM	1	32.6833	0.949
0.8	0	yadI	1	32.6833	1.055
0.8	0	AG1	1	32.6833	0.922
0.8	0	ptsI	1	32.9333	1.24
0.8	0	clcB	1	32.9333	0.794
0.8	0	ycaM	1	32.9333	0.955
0.8	0	yadI	1	32.9333	1.058
0.8	0	AG1	1	32.9333	0.926
0.8	0	ptsI	1	33.1833	1.255
0.8	0	clcB	1	33.1833	0.789
0.8	0	ycaM	1	33.1833	0.96
0.8	0	yadI	1	33.1833	1.049

0.8	0	AG1	1	33.1833	0.934
0.8	0	ptsI	1	33.4333	1.261
0.8	0	clcB	1	33.4333	0.796
0.8	0	ycaM	1	33.4333	0.977
0.8	0	yadI	1	33.4333	1.059
0.8	0	AG1	1	33.4333	0.943
0.8	0	ptsI	1	33.6833	1.272
0.8	0	clcB	1	33.6833	0.794
0.8	0	ycaM	1	33.6833	0.981
0.8	0	yadI	1	33.6833	1.049
0.8	0	AG1	1	33.6833	0.952
0.8	0	ptsI	1	33.9333	1.276
0.8	0	clcB	1	33.9333	0.805
0.8	0	ycaM	1	33.9333	0.991
0.8	0	yadI	1	33.9333	1.059
0.8	0	AG1	1	33.9333	0.952
0.8	0	ptsI	1	34.1833	1.287
0.8	0	clcB	1	34.1833	0.798
0.8	0	ycaM	1	34.1833	0.997
0.8	0	yadI	1	34.1833	1.072
0.8	0	AG1	1	34.1833	0.954
0.8	0	ptsI	1	34.4333	1.295
0.8	0	clcB	1	34.4333	0.817
0.8	0	ycaM	1	34.4333	1.009
0.8	0	yadI	1	34.4333	1.066
0.8	0	AG1	1	34.4333	0.967
0.8	0	ptsI	1	34.6833	1.298
0.8	0	clcB	1	34.6833	0.812
0.8	0	ycaM	1	34.6833	1.022
0.8	0	yadI	1	34.6833	1.07
0.8	0	AG1	1	34.6833	0.976
0.8	0	ptsI	1	34.9333	1.308
0.8	0	clcB	1	34.9333	0.826
0.8	0	ycaM	1	34.9333	1.026
0.8	0	yadI	1	34.9333	1.069
0.8	0	AG1	1	34.9333	0.976
0.8	0	ptsI	1	35.1833	1.313
0.8	0	clcB	1	35.1833	0.829
0.8	0	ycaM	1	35.1833	1.035
0.8	0	yadI	1	35.1833	1.072
0.8	0	AG1	1	35.1833	0.981
0.8	0	ptsI	1	35.4333	1.326
0.8	0	clcB	1	35.4333	0.84
0.8	0	ycaM	1	35.4333	1.042
0.8	0	yadI	1	35.4333	1.077
0.8	0	AG1	1	35.4333	0.99
0.8	0	ptsI	1	35.6833	1.329
0.8	0	clcB	1	35.6833	0.834
0.8	0	ycaM	1	35.6833	1.053
0.8	0	yadI	1	35.6833	1.08
0.8	0	AG1	1	35.6833	0.997
0.8	0	ptsI	1	35.9333	1.342
0.8	0	clcB	1	35.9333	0.831

0.8	0	ycaM	1	35.9333	1.057
0.8	0	yadI	1	35.9333	1.074
0.8	0	AG1	1	35.9333	1.008
0.8	0	ptsI	1	36.1833	1.339
0.8	0	clcB	1	36.1833	0.834
0.8	0	ycaM	1	36.1833	1.071
0.8	0	yadI	1	36.1833	1.085
0.8	0	AG1	1	36.1833	1.014
0.8	0	ptsI	1	36.4333	1.356
0.8	0	clcB	1	36.4333	0.835
0.8	0	ycaM	1	36.4333	1.083
0.8	0	yadI	1	36.4333	1.089
0.8	0	AG1	1	36.4333	1.022
0.8	0	ptsI	1	36.6833	1.366
0.8	0	clcB	1	36.6833	0.836
0.8	0	ycaM	1	36.6833	1.098
0.8	0	yadI	1	36.6833	1.091
0.8	0	AG1	1	36.6833	1.03
0.8	0	ptsI	2	0	0.206
0.8	0	clcB	2	0	0.202
0.8	0	ycaM	2	0	0.218
0.8	0	yadI	2	0	0.221
0.8	0	AG1	2	0	0.245
0.8	0	ptsI	2	0.25	0.208
0.8	0	clcB	2	0.25	0.2
0.8	0	ycaM	2	0.25	0.209
0.8	0	yadI	2	0.25	0.217
0.8	0	AG1	2	0.25	0.241
0.8	0	ptsI	2	0.5	0.213
0.8	0	clcB	2	0.5	0.2
0.8	0	ycaM	2	0.5	0.21
0.8	0	yadI	2	0.5	0.214
0.8	0	AG1	2	0.5	0.24
0.8	0	ptsI	2	0.75	0.214
0.8	0	clcB	2	0.75	0.201
0.8	0	ycaM	2	0.75	0.213
0.8	0	yadI	2	0.75	0.216
0.8	0	AG1	2	0.75	0.243
0.8	0	ptsI	2	1	0.218
0.8	0	clcB	2	1	0.203
0.8	0	ycaM	2	1	0.22
0.8	0	yadI	2	1	0.223
0.8	0	AG1	2	1	0.247
0.8	0	ptsI	2	1.25	0.222
0.8	0	clcB	2	1.25	0.206
0.8	0	ycaM	2	1.25	0.253
0.8	0	yadI	2	1.25	0.229
0.8	0	AG1	2	1.25	0.254
0.8	0	ptsI	2	1.5	0.225
0.8	0	clcB	2	1.5	0.206
0.8	0	ycaM	2	1.5	0.254
0.8	0	yadI	2	1.5	0.237
0.8	0	AG1	2	1.5	0.255

0.8	0	ptsI	2	1.75	0.232
0.8	0	clcB	2	1.75	0.208
0.8	0	ycaM	2	1.75	0.228
0.8	0	yadI	2	1.75	0.243
0.8	0	AG1	2	1.75	0.261
0.8	0	ptsI	2	2	0.238
0.8	0	clcB	2	2	0.212
0.8	0	ycaM	2	2	0.28
0.8	0	yadI	2	2	0.251
0.8	0	AG1	2	2	0.275
0.8	0	ptsI	2	2.25	0.249
0.8	0	clcB	2	2.25	0.215
0.8	0	ycaM	2	2.25	0.241
0.8	0	yadI	2	2.25	0.263
0.8	0	AG1	2	2.25	0.285
0.8	0	ptsI	2	2.5	0.26
0.8	0	clcB	2	2.5	0.22
0.8	0	ycaM	2	2.5	0.253
0.8	0	yadI	2	2.5	0.278
0.8	0	AG1	2	2.5	0.294
0.8	0	ptsI	2	2.75	0.266
0.8	0	clcB	2	2.75	0.226
0.8	0	ycaM	2	2.75	0.256
0.8	0	yadI	2	2.75	0.291
0.8	0	AG1	2	2.75	0.298
0.8	0	ptsI	2	3	0.272
0.8	0	clcB	2	3	0.231
0.8	0	ycaM	2	3	0.258
0.8	0	yadI	2	3	0.304
0.8	0	AG1	2	3	0.301
0.8	0	ptsI	2	3.25	0.281
0.8	0	clcB	2	3.25	0.236
0.8	0	ycaM	2	3.25	0.27
0.8	0	yadI	2	3.25	0.324
0.8	0	AG1	2	3.25	0.321
0.8	0	ptsI	2	3.5	0.285
0.8	0	clcB	2	3.5	0.242
0.8	0	ycaM	2	3.5	0.276
0.8	0	yadI	2	3.5	0.344
0.8	0	AG1	2	3.5	0.324
0.8	0	ptsI	2	3.75	0.294
0.8	0	clcB	2	3.75	0.246
0.8	0	ycaM	2	3.75	0.287
0.8	0	yadI	2	3.75	0.362
0.8	0	AG1	2	3.75	0.338
0.8	0	ptsI	2	4	0.306
0.8	0	clcB	2	4	0.255
0.8	0	ycaM	2	4	0.299
0.8	0	yadI	2	4	0.38
0.8	0	AG1	2	4	0.349
0.8	0	ptsI	2	4.38333	0.321
0.8	0	clcB	2	4.38333	0.261
0.8	0	ycaM	2	4.38333	0.32

0.8	0	yadI	2	4.38333	0.39
0.8	0	AG1	2	4.38333	0.302
0.8	0	ptsI	2	4.63333	0.315
0.8	0	clcB	2	4.63333	0.255
0.8	0	ycaM	2	4.63333	0.362
0.8	0	yadI	2	4.63333	0.386
0.8	0	AG1	2	4.63333	0.303
0.8	0	ptsI	2	4.88333	0.322
0.8	0	clcB	2	4.88333	0.261
0.8	0	ycaM	2	4.88333	0.317
0.8	0	yadI	2	4.88333	0.395
0.8	0	AG1	2	4.88333	0.314
0.8	0	ptsI	2	5.13333	0.327
0.8	0	clcB	2	5.13333	0.266
0.8	0	ycaM	2	5.13333	0.32
0.8	0	yadI	2	5.13333	0.407
0.8	0	AG1	2	5.13333	0.322
0.8	0	ptsI	2	5.38333	0.336
0.8	0	clcB	2	5.38333	0.274
0.8	0	ycaM	2	5.38333	0.33
0.8	0	yadI	2	5.38333	0.419
0.8	0	AG1	2	5.38333	0.329
0.8	0	ptsI	2	5.63333	0.345
0.8	0	clcB	2	5.63333	0.285
0.8	0	ycaM	2	5.63333	0.339
0.8	0	yadI	2	5.63333	0.428
0.8	0	AG1	2	5.63333	0.337
0.8	0	ptsI	2	5.88333	0.353
0.8	0	clcB	2	5.88333	0.291
0.8	0	ycaM	2	5.88333	0.349
0.8	0	yadI	2	5.88333	0.435
0.8	0	AG1	2	5.88333	0.348
0.8	0	ptsI	2	6.13333	0.362
0.8	0	clcB	2	6.13333	0.298
0.8	0	ycaM	2	6.13333	0.358
0.8	0	yadI	2	6.13333	0.448
0.8	0	AG1	2	6.13333	0.357
0.8	0	ptsI	2	6.38333	0.369
0.8	0	clcB	2	6.38333	0.306
0.8	0	ycaM	2	6.38333	0.377
0.8	0	yadI	2	6.38333	0.457
0.8	0	AG1	2	6.38333	0.364
0.8	0	ptsI	2	6.63333	0.379
0.8	0	clcB	2	6.63333	0.312
0.8	0	ycaM	2	6.63333	0.376
0.8	0	yadI	2	6.63333	0.466
0.8	0	AG1	2	6.63333	0.372
0.8	0	ptsI	2	6.88333	0.387
0.8	0	clcB	2	6.88333	0.32
0.8	0	ycaM	2	6.88333	0.392
0.8	0	yadI	2	6.88333	0.477
0.8	0	AG1	2	6.88333	0.379
0.8	0	ptsI	2	7.13333	0.394

0.8	0	clcB	2	7.13333	0.326
0.8	0	ycaM	2	7.13333	0.401
0.8	0	yadI	2	7.13333	0.485
0.8	0	AG1	2	7.13333	0.383
0.8	0	ptsI	2	7.38333	0.403
0.8	0	clcB	2	7.38333	0.332
0.8	0	ycaM	2	7.38333	0.402
0.8	0	yadI	2	7.38333	0.496
0.8	0	AG1	2	7.38333	0.391
0.8	0	ptsI	2	7.63333	0.412
0.8	0	clcB	2	7.63333	0.342
0.8	0	ycaM	2	7.63333	0.408
0.8	0	yadI	2	7.63333	0.508
0.8	0	AG1	2	7.63333	0.395
0.8	0	ptsI	2	7.88333	0.418
0.8	0	clcB	2	7.88333	0.35
0.8	0	ycaM	2	7.88333	0.415
0.8	0	yadI	2	7.88333	0.519
0.8	0	AG1	2	7.88333	0.405
0.8	0	ptsI	2	8.13333	0.428
0.8	0	clcB	2	8.13333	0.356
0.8	0	ycaM	2	8.13333	0.419
0.8	0	yadI	2	8.13333	0.526
0.8	0	AG1	2	8.13333	0.413
0.8	0	ptsI	2	8.38333	0.438
0.8	0	clcB	2	8.38333	0.366
0.8	0	ycaM	2	8.38333	0.422
0.8	0	yadI	2	8.38333	0.54
0.8	0	AG1	2	8.38333	0.422
0.8	0	ptsI	2	8.91667	0.353
0.8	0	clcB	2	8.91667	0.314
0.8	0	ycaM	2	8.91667	0.354
0.8	0	yadI	2	8.91667	0.61
0.8	0	AG1	2	8.91667	0.481
0.8	0	ptsI	2	9.16667	0.356
0.8	0	clcB	2	9.16667	0.319
0.8	0	ycaM	2	9.16667	0.358
0.8	0	yadI	2	9.16667	0.612
0.8	0	AG1	2	9.16667	0.484
0.8	0	ptsI	2	9.41667	0.364
0.8	0	clcB	2	9.41667	0.325
0.8	0	ycaM	2	9.41667	0.363
0.8	0	yadI	2	9.41667	0.625
0.8	0	AG1	2	9.41667	0.496
0.8	0	ptsI	2	9.66667	0.372
0.8	0	clcB	2	9.66667	0.333
0.8	0	ycaM	2	9.66667	0.37
0.8	0	yadI	2	9.66667	0.636
0.8	0	AG1	2	9.66667	0.508
0.8	0	ptsI	2	9.91667	0.38
0.8	0	clcB	2	9.91667	0.338
0.8	0	ycaM	2	9.91667	0.372
0.8	0	yadI	2	9.91667	0.646

0.8	0	AG1	2	9.91667	0.516
0.8	0	ptsI	2	10.1667	0.39
0.8	0	clcB	2	10.1667	0.342
0.8	0	ycaM	2	10.1667	0.379
0.8	0	yadI	2	10.1667	0.657
0.8	0	AG1	2	10.1667	0.524
0.8	0	ptsI	2	10.4167	0.4
0.8	0	clcB	2	10.4167	0.349
0.8	0	ycaM	2	10.4167	0.385
0.8	0	yadI	2	10.4167	0.67
0.8	0	AG1	2	10.4167	0.548
0.8	0	ptsI	2	10.6667	0.424
0.8	0	clcB	2	10.6667	0.354
0.8	0	ycaM	2	10.6667	0.392
0.8	0	yadI	2	10.6667	0.68
0.8	0	AG1	2	10.6667	0.541
0.8	0	ptsI	2	10.9167	0.454
0.8	0	clcB	2	10.9167	0.355
0.8	0	ycaM	2	10.9167	0.398
0.8	0	yadI	2	10.9167	0.69
0.8	0	AG1	2	10.9167	0.546
0.8	0	ptsI	2	11.1667	0.447
0.8	0	clcB	2	11.1667	0.36
0.8	0	ycaM	2	11.1667	0.408
0.8	0	yadI	2	11.1667	0.7
0.8	0	AG1	2	11.1667	0.556
0.8	0	ptsI	2	11.4167	0.468
0.8	0	clcB	2	11.4167	0.364
0.8	0	ycaM	2	11.4167	0.413
0.8	0	yadI	2	11.4167	0.708
0.8	0	AG1	2	11.4167	0.561
0.8	0	ptsI	2	11.6667	0.501
0.8	0	clcB	2	11.6667	0.372
0.8	0	ycaM	2	11.6667	0.417
0.8	0	yadI	2	11.6667	0.717
0.8	0	AG1	2	11.6667	0.593
0.8	0	ptsI	2	11.9167	0.505
0.8	0	clcB	2	11.9167	0.378
0.8	0	ycaM	2	11.9167	0.422
0.8	0	yadI	2	11.9167	0.723
0.8	0	AG1	2	11.9167	0.583
0.8	0	ptsI	2	12.1667	0.527
0.8	0	clcB	2	12.1667	0.384
0.8	0	ycaM	2	12.1667	0.44
0.8	0	yadI	2	12.1667	0.726
0.8	0	AG1	2	12.1667	0.6
0.8	0	ptsI	2	12.4167	0.52
0.8	0	clcB	2	12.4167	0.395
0.8	0	ycaM	2	12.4167	0.434
0.8	0	yadI	2	12.4167	0.731
0.8	0	AG1	2	12.4167	0.604
0.8	0	ptsI	2	12.6667	0.512
0.8	0	clcB	2	12.6667	0.399

0.8	0	ycaM	2	12.6667	0.463
0.8	0	yadI	2	12.6667	0.742
0.8	0	AG1	2	12.6667	0.595
0.8	0	ptsI	2	12.9167	0.52
0.8	0	clcB	2	12.9167	0.403
0.8	0	ycaM	2	12.9167	0.465
0.8	0	yadI	2	12.9167	0.755
0.8	0	AG1	2	12.9167	0.613
0.8	0	ptsI	2	13.1667	0.518
0.8	0	clcB	2	13.1667	0.404
0.8	0	ycaM	2	13.1667	0.47
0.8	0	yadI	2	13.1667	0.758
0.8	0	AG1	2	13.1667	0.613
0.8	0	ptsI	2	13.4167	0.537
0.8	0	clcB	2	13.4167	0.41
0.8	0	ycaM	2	13.4167	0.47
0.8	0	yadI	2	13.4167	0.767
0.8	0	AG1	2	13.4167	0.61
0.8	0	ptsI	2	13.6667	0.566
0.8	0	clcB	2	13.6667	0.42
0.8	0	ycaM	2	13.6667	0.504
0.8	0	yadI	2	13.6667	0.769
0.8	0	AG1	2	13.6667	0.611
0.8	0	ptsI	2	13.9167	0.597
0.8	0	clcB	2	13.9167	0.421
0.8	0	ycaM	2	13.9167	0.487
0.8	0	yadI	2	13.9167	0.772
0.8	0	AG1	2	13.9167	0.606
0.8	0	ptsI	2	14.1667	0.609
0.8	0	clcB	2	14.1667	0.422
0.8	0	ycaM	2	14.1667	0.483
0.8	0	yadI	2	14.1667	0.779
0.8	0	AG1	2	14.1667	0.624
0.8	0	ptsI	2	14.4167	0.625
0.8	0	clcB	2	14.4167	0.431
0.8	0	ycaM	2	14.4167	0.486
0.8	0	yadI	2	14.4167	0.783
0.8	0	AG1	2	14.4167	0.642
0.8	0	ptsI	2	14.6667	0.655
0.8	0	clcB	2	14.6667	0.432
0.8	0	ycaM	2	14.6667	0.502
0.8	0	yadI	2	14.6667	0.789
0.8	0	AG1	2	14.6667	0.654
0.8	0	ptsI	2	14.9167	0.648
0.8	0	clcB	2	14.9167	0.442
0.8	0	ycaM	2	14.9167	0.494
0.8	0	yadI	2	14.9167	0.797
0.8	0	AG1	2	14.9167	0.67
0.8	0	ptsI	2	15.1667	0.665
0.8	0	clcB	2	15.1667	0.44
0.8	0	ycaM	2	15.1667	0.512
0.8	0	yadI	2	15.1667	0.808
0.8	0	AG1	2	15.1667	0.656

0.8	0	ptsI	2	15.4167	0.669
0.8	0	clcB	2	15.4167	0.437
0.8	0	ycaM	2	15.4167	0.519
0.8	0	yadI	2	15.4167	0.807
0.8	0	AG1	2	15.4167	0.677
0.8	0	ptsI	2	15.6667	0.678
0.8	0	clcB	2	15.6667	0.471
0.8	0	ycaM	2	15.6667	0.525
0.8	0	yadI	2	15.6667	0.813
0.8	0	AG1	2	15.6667	0.7
0.8	0	ptsI	2	15.9167	0.676
0.8	0	clcB	2	15.9167	0.465
0.8	0	ycaM	2	15.9167	0.51
0.8	0	yadI	2	15.9167	0.815
0.8	0	AG1	2	15.9167	0.702
0.8	0	ptsI	2	16.1667	0.719
0.8	0	clcB	2	16.1667	0.453
0.8	0	ycaM	2	16.1667	0.528
0.8	0	yadI	2	16.1667	0.816
0.8	0	AG1	2	16.1667	0.702
0.8	0	ptsI	2	16.4167	0.738
0.8	0	clcB	2	16.4167	0.466
0.8	0	ycaM	2	16.4167	0.53
0.8	0	yadI	2	16.4167	0.821
0.8	0	AG1	2	16.4167	0.73
0.8	0	ptsI	2	16.6667	0.752
0.8	0	clcB	2	16.6667	0.478
0.8	0	ycaM	2	16.6667	0.538
0.8	0	yadI	2	16.6667	0.825
0.8	0	AG1	2	16.6667	0.739
0.8	0	ptsI	2	16.9167	0.742
0.8	0	clcB	2	16.9167	0.466
0.8	0	ycaM	2	16.9167	0.531
0.8	0	yadI	2	16.9167	0.826
0.8	0	AG1	2	16.9167	0.746
0.8	0	ptsI	2	17.1667	0.74
0.8	0	clcB	2	17.1667	0.467
0.8	0	ycaM	2	17.1667	0.545
0.8	0	yadI	2	17.1667	0.825
0.8	0	AG1	2	17.1667	0.747
0.8	0	ptsI	2	17.4167	0.754
0.8	0	clcB	2	17.4167	0.479
0.8	0	ycaM	2	17.4167	0.544
0.8	0	yadI	2	17.4167	0.83
0.8	0	AG1	2	17.4167	0.749
0.8	0	ptsI	2	17.6667	0.746
0.8	0	clcB	2	17.6667	0.473
0.8	0	ycaM	2	17.6667	0.544
0.8	0	yadI	2	17.6667	0.832
0.8	0	AG1	2	17.6667	0.756
0.8	0	ptsI	2	17.9167	0.767
0.8	0	clcB	2	17.9167	0.473
0.8	0	ycaM	2	17.9167	0.539

0.8	0	yadI	2	17.9167	0.834
0.8	0	AG1	2	17.9167	0.757
0.8	0	ptsI	2	18.1667	0.767
0.8	0	clcB	2	18.1667	0.474
0.8	0	ycaM	2	18.1667	0.532
0.8	0	yadI	2	18.1667	0.836
0.8	0	AG1	2	18.1667	0.767
0.8	0	ptsI	2	18.4167	0.778
0.8	0	clcB	2	18.4167	0.477
0.8	0	ycaM	2	18.4167	0.534
0.8	0	yadI	2	18.4167	0.838
0.8	0	AG1	2	18.4167	0.767
0.8	0	ptsI	2	18.6667	0.785
0.8	0	clcB	2	18.6667	0.476
0.8	0	ycaM	2	18.6667	0.532
0.8	0	yadI	2	18.6667	0.839
0.8	0	AG1	2	18.6667	0.764
0.8	0	ptsI	2	18.9167	0.786
0.8	0	clcB	2	18.9167	0.466
0.8	0	ycaM	2	18.9167	0.534
0.8	0	yadI	2	18.9167	0.839
0.8	0	AG1	2	18.9167	0.78
0.8	0	ptsI	2	19.1667	0.799
0.8	0	clcB	2	19.1667	0.473
0.8	0	ycaM	2	19.1667	0.541
0.8	0	yadI	2	19.1667	0.843
0.8	0	AG1	2	19.1667	0.766
0.8	0	ptsI	2	19.4167	0.798
0.8	0	clcB	2	19.4167	0.47
0.8	0	ycaM	2	19.4167	0.543
0.8	0	yadI	2	19.4167	0.844
0.8	0	AG1	2	19.4167	0.762
0.8	0	ptsI	2	19.6667	0.801
0.8	0	clcB	2	19.6667	0.463
0.8	0	ycaM	2	19.6667	0.55
0.8	0	yadI	2	19.6667	0.847
0.8	0	AG1	2	19.6667	0.767
0.8	0	ptsI	2	19.9167	0.804
0.8	0	clcB	2	19.9167	0.468
0.8	0	ycaM	2	19.9167	0.547
0.8	0	yadI	2	19.9167	0.848
0.8	0	AG1	2	19.9167	0.77
0.8	0	ptsI	2	20.1667	0.801
0.8	0	clcB	2	20.1667	0.469
0.8	0	ycaM	2	20.1667	0.549
0.8	0	yadI	2	20.1667	0.847
0.8	0	AG1	2	20.1667	0.757
0.8	0	ptsI	2	20.4167	0.809
0.8	0	clcB	2	20.4167	0.476
0.8	0	ycaM	2	20.4167	0.555
0.8	0	yadI	2	20.4167	0.85
0.8	0	AG1	2	20.4167	0.755
0.8	0	ptsI	2	20.6667	0.809

0.8	0	clcB	2	20.6667	0.468
0.8	0	ycaM	2	20.6667	0.56
0.8	0	yadI	2	20.6667	0.849
0.8	0	AG1	2	20.6667	0.747
0.8	0	ptsI	2	20.9167	0.815
0.8	0	clcB	2	20.9167	0.469
0.8	0	ycaM	2	20.9167	0.566
0.8	0	yadI	2	20.9167	0.851
0.8	0	AG1	2	20.9167	0.746
0.8	0	ptsI	2	21.1667	0.808
0.8	0	clcB	2	21.1667	0.472
0.8	0	ycaM	2	21.1667	0.572
0.8	0	yadI	2	21.1667	0.85
0.8	0	AG1	2	21.1667	0.749
0.8	0	ptsI	2	21.4167	0.813
0.8	0	clcB	2	21.4167	0.472
0.8	0	ycaM	2	21.4167	0.574
0.8	0	yadI	2	21.4167	0.858
0.8	0	AG1	2	21.4167	0.764
0.8	0	ptsI	2	21.6667	0.816
0.8	0	clcB	2	21.6667	0.475
0.8	0	ycaM	2	21.6667	0.578
0.8	0	yadI	2	21.6667	0.853
0.8	0	AG1	2	21.6667	0.753
0.8	0	ptsI	2	21.9167	0.821
0.8	0	clcB	2	21.9167	0.481
0.8	0	ycaM	2	21.9167	0.583
0.8	0	yadI	2	21.9167	0.855
0.8	0	AG1	2	21.9167	0.755
0.8	0	ptsI	2	22.1667	0.826
0.8	0	clcB	2	22.1667	0.48
0.8	0	ycaM	2	22.1667	0.586
0.8	0	yadI	2	22.1667	0.854
0.8	0	AG1	2	22.1667	0.758
0.8	0	ptsI	2	22.4167	0.826
0.8	0	clcB	2	22.4167	0.482
0.8	0	ycaM	2	22.4167	0.587
0.8	0	yadI	2	22.4167	0.854
0.8	0	AG1	2	22.4167	0.76
0.8	0	ptsI	2	22.6667	0.835
0.8	0	clcB	2	22.6667	0.485
0.8	0	ycaM	2	22.6667	0.59
0.8	0	yadI	2	22.6667	0.856
0.8	0	AG1	2	22.6667	0.759
0.8	0	ptsI	2	22.9167	0.844
0.8	0	clcB	2	22.9167	0.487
0.8	0	ycaM	2	22.9167	0.593
0.8	0	yadI	2	22.9167	0.862
0.8	0	AG1	2	22.9167	0.761
0.8	0	ptsI	2	23.1667	0.85
0.8	0	clcB	2	23.1667	0.489
0.8	0	ycaM	2	23.1667	0.595
0.8	0	yadI	2	23.1667	0.863

0.8	0	AG1	2	23.1667	0.764
0.8	0	ptsI	2	23.4167	0.852
0.8	0	clcB	2	23.4167	0.491
0.8	0	ycaM	2	23.4167	0.597
0.8	0	yadI	2	23.4167	0.863
0.8	0	AG1	2	23.4167	0.762
0.8	0	ptsI	2	23.6667	0.864
0.8	0	clcB	2	23.6667	0.494
0.8	0	ycaM	2	23.6667	0.6
0.8	0	yadI	2	23.6667	0.864
0.8	0	AG1	2	23.6667	0.768
0.8	0	ptsI	2	23.9167	0.869
0.8	0	clcB	2	23.9167	0.496
0.8	0	ycaM	2	23.9167	0.6
0.8	0	yadI	2	23.9167	0.866
0.8	0	AG1	2	23.9167	0.764
0.8	0	ptsI	2	24.1667	0.869
0.8	0	clcB	2	24.1667	0.497
0.8	0	ycaM	2	24.1667	0.599
0.8	0	yadI	2	24.1667	0.866
0.8	0	AG1	2	24.1667	0.764
0.8	0	ptsI	2	24.4167	0.872
0.8	0	clcB	2	24.4167	0.499
0.8	0	ycaM	2	24.4167	0.605
0.8	0	yadI	2	24.4167	0.868
0.8	0	AG1	2	24.4167	0.766
0.8	0	ptsI	2	24.6667	0.877
0.8	0	clcB	2	24.6667	0.502
0.8	0	ycaM	2	24.6667	0.607
0.8	0	yadI	2	24.6667	0.867
0.8	0	AG1	2	24.6667	0.77
0.8	0	ptsI	2	24.9167	0.882
0.8	0	clcB	2	24.9167	0.505
0.8	0	ycaM	2	24.9167	0.609
0.8	0	yadI	2	24.9167	0.87
0.8	0	AG1	2	24.9167	0.768
0.8	0	ptsI	2	25.1667	0.886
0.8	0	clcB	2	25.1667	0.506
0.8	0	ycaM	2	25.1667	0.61
0.8	0	yadI	2	25.1667	0.872
0.8	0	AG1	2	25.1667	0.768
0.8	0	ptsI	2	25.4167	0.892
0.8	0	clcB	2	25.4167	0.507
0.8	0	ycaM	2	25.4167	0.611
0.8	0	yadI	2	25.4167	0.873
0.8	0	AG1	2	25.4167	0.772
0.8	0	ptsI	2	25.6667	0.895
0.8	0	clcB	2	25.6667	0.509
0.8	0	ycaM	2	25.6667	0.614
0.8	0	yadI	2	25.6667	0.87
0.8	0	AG1	2	25.6667	0.771
0.8	0	ptsI	2	25.9167	0.896
0.8	0	clcB	2	25.9167	0.511

0.8	0	ycaM	2	25.9167	0.616
0.8	0	yadI	2	25.9167	0.876
0.8	0	AG1	2	25.9167	0.775
0.8	0	ptsI	2	26.1667	0.904
0.8	0	clcB	2	26.1667	0.516
0.8	0	ycaM	2	26.1667	0.617
0.8	0	yadI	2	26.1667	0.88
0.8	0	AG1	2	26.1667	0.784
0.8	0	ptsI	2	26.4167	0.911
0.8	0	clcB	2	26.4167	0.516
0.8	0	ycaM	2	26.4167	0.62
0.8	0	yadI	2	26.4167	0.878
0.8	0	AG1	2	26.4167	0.772
0.8	0	ptsI	3	0	0.153
0.8	0	clcB	3	0	0.155
0.8	0	ycaM	3	0	0.153
0.8	0	yadI	3	0	0.167
0.8	0	AG1	3	0	0.163
0.8	0	ptsI	3	0.25	0.152
0.8	0	clcB	3	0.25	0.152
0.8	0	ycaM	3	0.25	0.149
0.8	0	yadI	3	0.25	0.163
0.8	0	AG1	3	0.25	0.159
0.8	0	ptsI	3	0.5	0.151
0.8	0	clcB	3	0.5	0.152
0.8	0	ycaM	3	0.5	0.148
0.8	0	yadI	3	0.5	0.163
0.8	0	AG1	3	0.5	0.158
0.8	0	ptsI	3	0.75	0.152
0.8	0	clcB	3	0.75	0.153
0.8	0	ycaM	3	0.75	0.15
0.8	0	yadI	3	0.75	0.168
0.8	0	AG1	3	0.75	0.16
0.8	0	ptsI	3	1	0.153
0.8	0	clcB	3	1	0.154
0.8	0	ycaM	3	1	0.15
0.8	0	yadI	3	1	0.162
0.8	0	AG1	3	1	0.16
0.8	0	ptsI	3	1.25	0.154
0.8	0	clcB	3	1.25	0.154
0.8	0	ycaM	3	1.25	0.148
0.8	0	yadI	3	1.25	0.169
0.8	0	AG1	3	1.25	0.159
0.8	0	ptsI	3	1.5	0.154
0.8	0	clcB	3	1.5	0.156
0.8	0	ycaM	3	1.5	0.15
0.8	0	yadI	3	1.5	0.164
0.8	0	AG1	3	1.5	0.16
0.8	0	ptsI	3	1.75	0.155
0.8	0	clcB	3	1.75	0.156
0.8	0	ycaM	3	1.75	0.151
0.8	0	yadI	3	1.75	0.164
0.8	0	AG1	3	1.75	0.16

0.8	0	ptsI	3	2	0.157
0.8	0	clcB	3	2	0.157
0.8	0	ycaM	3	2	0.152
0.8	0	yadI	3	2	0.171
0.8	0	AG1	3	2	0.162
0.8	0	ptsI	3	2.25	0.159
0.8	0	clcB	3	2.25	0.16
0.8	0	ycaM	3	2.25	0.154
0.8	0	yadI	3	2.25	0.17
0.8	0	AG1	3	2.25	0.164
0.8	0	ptsI	3	2.5	0.161
0.8	0	clcB	3	2.5	0.162
0.8	0	ycaM	3	2.5	0.159
0.8	0	yadI	3	2.5	0.168
0.8	0	AG1	3	2.5	0.166
0.8	0	ptsI	3	2.75	0.163
0.8	0	clcB	3	2.75	0.164
0.8	0	ycaM	3	2.75	0.158
0.8	0	yadI	3	2.75	0.172
0.8	0	AG1	3	2.75	0.171
0.8	0	ptsI	3	3	0.164
0.8	0	clcB	3	3	0.165
0.8	0	ycaM	3	3	0.158
0.8	0	yadI	3	3	0.173
0.8	0	AG1	3	3	0.171
0.8	0	ptsI	3	3.25	0.167
0.8	0	clcB	3	3.25	0.169
0.8	0	ycaM	3	3.25	0.16
0.8	0	yadI	3	3.25	0.177
0.8	0	AG1	3	3.25	0.172
0.8	0	ptsI	3	3.5	0.17
0.8	0	clcB	3	3.5	0.171
0.8	0	ycaM	3	3.5	0.166
0.8	0	yadI	3	3.5	0.181
0.8	0	AG1	3	3.5	0.178
0.8	0	ptsI	3	3.75	0.174
0.8	0	clcB	3	3.75	0.175
0.8	0	ycaM	3	3.75	0.17
0.8	0	yadI	3	3.75	0.185
0.8	0	AG1	3	3.75	0.181
0.8	0	ptsI	3	4	0.182
0.8	0	clcB	3	4	0.178
0.8	0	ycaM	3	4	0.174
0.8	0	yadI	3	4	0.191
0.8	0	AG1	3	4	0.186
0.8	0	ptsI	3	4.41667	0.183
0.8	0	clcB	3	4.41667	0.194
0.8	0	ycaM	3	4.41667	0.194
0.8	0	yadI	3	4.41667	0.194
0.8	0	AG1	3	4.41667	0.201
0.8	0	ptsI	3	4.66667	0.183
0.8	0	clcB	3	4.66667	0.184
0.8	0	ycaM	3	4.66667	0.197

0.8	0	yadI	3	4.66667	0.186
0.8	0	AG1	3	4.66667	0.2
0.8	0	ptsI	3	4.91667	0.184
0.8	0	clcB	3	4.91667	0.18
0.8	0	ycaM	3	4.91667	0.193
0.8	0	yadI	3	4.91667	0.19
0.8	0	AG1	3	4.91667	0.199
0.8	0	ptsI	3	5.16667	0.188
0.8	0	clcB	3	5.16667	0.186
0.8	0	ycaM	3	5.16667	0.198
0.8	0	yadI	3	5.16667	0.2
0.8	0	AG1	3	5.16667	0.204
0.8	0	ptsI	3	5.41667	0.191
0.8	0	clcB	3	5.41667	0.19
0.8	0	ycaM	3	5.41667	0.205
0.8	0	yadI	3	5.41667	0.212
0.8	0	AG1	3	5.41667	0.212
0.8	0	ptsI	3	5.66667	0.197
0.8	0	clcB	3	5.66667	0.196
0.8	0	ycaM	3	5.66667	0.216
0.8	0	yadI	3	5.66667	0.226
0.8	0	AG1	3	5.66667	0.221
0.8	0	ptsI	3	5.91667	0.201
0.8	0	clcB	3	5.91667	0.222
0.8	0	ycaM	3	5.91667	0.227
0.8	0	yadI	3	5.91667	0.24
0.8	0	AG1	3	5.91667	0.231
0.8	0	ptsI	3	6.16667	0.208
0.8	0	clcB	3	6.16667	0.212
0.8	0	ycaM	3	6.16667	0.242
0.8	0	yadI	3	6.16667	0.258
0.8	0	AG1	3	6.16667	0.242
0.8	0	ptsI	3	6.41667	0.214
0.8	0	clcB	3	6.41667	0.22
0.8	0	ycaM	3	6.41667	0.257
0.8	0	yadI	3	6.41667	0.27
0.8	0	AG1	3	6.41667	0.252
0.8	0	ptsI	3	6.66667	0.222
0.8	0	clcB	3	6.66667	0.23
0.8	0	ycaM	3	6.66667	0.272
0.8	0	yadI	3	6.66667	0.282
0.8	0	AG1	3	6.66667	0.266
0.8	0	ptsI	3	6.91667	0.231
0.8	0	clcB	3	6.91667	0.254
0.8	0	ycaM	3	6.91667	0.281
0.8	0	yadI	3	6.91667	0.296
0.8	0	AG1	3	6.91667	0.277
0.8	0	ptsI	3	7.16667	0.242
0.8	0	clcB	3	7.16667	0.257
0.8	0	ycaM	3	7.16667	0.291
0.8	0	yadI	3	7.16667	0.318
0.8	0	AG1	3	7.16667	0.288
0.8	0	ptsI	3	7.41667	0.254

0.8	0	clcB	3	7.41667	0.265
0.8	0	ycaM	3	7.41667	0.301
0.8	0	yadI	3	7.41667	0.328
0.8	0	AG1	3	7.41667	0.294
0.8	0	ptsI	3	7.66667	0.263
0.8	0	clcB	3	7.66667	0.273
0.8	0	ycaM	3	7.66667	0.311
0.8	0	yadI	3	7.66667	0.339
0.8	0	AG1	3	7.66667	0.303
0.8	0	ptsI	3	7.91667	0.27
0.8	0	clcB	3	7.91667	0.28
0.8	0	ycaM	3	7.91667	0.322
0.8	0	yadI	3	7.91667	0.355
0.8	0	AG1	3	7.91667	0.312
0.8	0	ptsI	3	8.16667	0.28
0.8	0	clcB	3	8.16667	0.289
0.8	0	ycaM	3	8.16667	0.333
0.8	0	yadI	3	8.16667	0.382
0.8	0	AG1	3	8.16667	0.32
0.8	0	ptsI	3	8.41667	0.286
0.8	0	clcB	3	8.41667	0.298
0.8	0	ycaM	3	8.41667	0.354
0.8	0	yadI	3	8.41667	0.385
0.8	0	AG1	3	8.41667	0.328
0.8	0	ptsI	3	9.05	0.263
0.8	0	clcB	3	9.05	0.281
0.8	0	ycaM	3	9.05	0.307
0.8	0	yadI	3	9.05	0.452
0.8	0	AG1	3	9.05	0.358
0.8	0	ptsI	3	9.3	0.27
0.8	0	clcB	3	9.3	0.289
0.8	0	ycaM	3	9.3	0.31
0.8	0	yadI	3	9.3	0.448
0.8	0	AG1	3	9.3	0.356
0.8	0	ptsI	3	9.55	0.28
0.8	0	clcB	3	9.55	0.298
0.8	0	ycaM	3	9.55	0.321
0.8	0	yadI	3	9.55	0.462
0.8	0	AG1	3	9.55	0.368
0.8	0	ptsI	3	9.8	0.287
0.8	0	clcB	3	9.8	0.308
0.8	0	ycaM	3	9.8	0.33
0.8	0	yadI	3	9.8	0.47
0.8	0	AG1	3	9.8	0.38
0.8	0	ptsI	3	10.05	0.296
0.8	0	clcB	3	10.05	0.316
0.8	0	ycaM	3	10.05	0.342
0.8	0	yadI	3	10.05	0.485
0.8	0	AG1	3	10.05	0.392
0.8	0	ptsI	3	10.3	0.304
0.8	0	clcB	3	10.3	0.327
0.8	0	ycaM	3	10.3	0.356
0.8	0	yadI	3	10.3	0.497

0.8	0	AG1	3	10.3	0.398
0.8	0	ptsI	3	10.55	0.31
0.8	0	clcB	3	10.55	0.335
0.8	0	ycaM	3	10.55	0.363
0.8	0	yadI	3	10.55	0.503
0.8	0	AG1	3	10.55	0.406
0.8	0	ptsI	3	10.8	0.319
0.8	0	clcB	3	10.8	0.344
0.8	0	ycaM	3	10.8	0.371
0.8	0	yadI	3	10.8	0.516
0.8	0	AG1	3	10.8	0.414
0.8	0	ptsI	3	11.05	0.326
0.8	0	clcB	3	11.05	0.354
0.8	0	ycaM	3	11.05	0.38
0.8	0	yadI	3	11.05	0.534
0.8	0	AG1	3	11.05	0.427
0.8	0	ptsI	3	11.3	0.336
0.8	0	clcB	3	11.3	0.361
0.8	0	ycaM	3	11.3	0.38
0.8	0	yadI	3	11.3	0.542
0.8	0	AG1	3	11.3	0.435
0.8	0	ptsI	3	11.55	0.344
0.8	0	clcB	3	11.55	0.363
0.8	0	ycaM	3	11.55	0.39
0.8	0	yadI	3	11.55	0.556
0.8	0	AG1	3	11.55	0.445
0.8	0	ptsI	3	11.8	0.354
0.8	0	clcB	3	11.8	0.368
0.8	0	ycaM	3	11.8	0.402
0.8	0	yadI	3	11.8	0.564
0.8	0	AG1	3	11.8	0.453
0.8	0	ptsI	3	12.05	0.361
0.8	0	clcB	3	12.05	0.37
0.8	0	ycaM	3	12.05	0.407
0.8	0	yadI	3	12.05	0.569
0.8	0	AG1	3	12.05	0.463
0.8	0	ptsI	3	12.3	0.373
0.8	0	clcB	3	12.3	0.377
0.8	0	ycaM	3	12.3	0.415
0.8	0	yadI	3	12.3	0.589
0.8	0	AG1	3	12.3	0.478
0.8	0	ptsI	3	12.55	0.378
0.8	0	clcB	3	12.55	0.384
0.8	0	ycaM	3	12.55	0.423
0.8	0	yadI	3	12.55	0.597
0.8	0	AG1	3	12.55	0.491
0.8	0	ptsI	3	12.8	0.388
0.8	0	clcB	3	12.8	0.39
0.8	0	ycaM	3	12.8	0.432
0.8	0	yadI	3	12.8	0.609
0.8	0	AG1	3	12.8	0.502
0.8	0	ptsI	3	13.05	0.392
0.8	0	clcB	3	13.05	0.394

0.8	0	ycaM	3	13.05	0.43
0.8	0	yadI	3	13.05	0.612
0.8	0	AG1	3	13.05	0.512
0.8	0	ptsI	3	13.3	0.405
0.8	0	clcB	3	13.3	0.402
0.8	0	ycaM	3	13.3	0.438
0.8	0	yadI	3	13.3	0.633
0.8	0	AG1	3	13.3	0.529
0.8	0	ptsI	3	13.55	0.412
0.8	0	clcB	3	13.55	0.414
0.8	0	ycaM	3	13.55	0.445
0.8	0	yadI	3	13.55	0.627
0.8	0	AG1	3	13.55	0.544
0.8	0	ptsI	3	13.8	0.421
0.8	0	clcB	3	13.8	0.414
0.8	0	ycaM	3	13.8	0.452
0.8	0	yadI	3	13.8	0.647
0.8	0	AG1	3	13.8	0.555
0.8	0	ptsI	3	14.05	0.429
0.8	0	clcB	3	14.05	0.42
0.8	0	ycaM	3	14.05	0.463
0.8	0	yadI	3	14.05	0.644
0.8	0	AG1	3	14.05	0.568
0.8	0	ptsI	3	14.3	0.432
0.8	0	clcB	3	14.3	0.427
0.8	0	ycaM	3	14.3	0.47
0.8	0	yadI	3	14.3	0.651
0.8	0	AG1	3	14.3	0.575
0.8	0	ptsI	3	14.55	0.44
0.8	0	clcB	3	14.55	0.428
0.8	0	ycaM	3	14.55	0.474
0.8	0	yadI	3	14.55	0.665
0.8	0	AG1	3	14.55	0.579
0.8	0	ptsI	3	14.8	0.45
0.8	0	clcB	3	14.8	0.436
0.8	0	ycaM	3	14.8	0.486
0.8	0	yadI	3	14.8	0.672
0.8	0	AG1	3	14.8	0.583
0.8	0	ptsI	3	15.05	0.458
0.8	0	clcB	3	15.05	0.44
0.8	0	ycaM	3	15.05	0.488
0.8	0	yadI	3	15.05	0.679
0.8	0	AG1	3	15.05	0.615
0.8	0	ptsI	3	15.3	0.466
0.8	0	clcB	3	15.3	0.445
0.8	0	ycaM	3	15.3	0.499
0.8	0	yadI	3	15.3	0.698
0.8	0	AG1	3	15.3	0.632
0.8	0	ptsI	3	15.55	0.48
0.8	0	clcB	3	15.55	0.448
0.8	0	ycaM	3	15.55	0.507
0.8	0	yadI	3	15.55	0.694
0.8	0	AG1	3	15.55	0.639

0.8	0	ptsI	3	15.8	0.488
0.8	0	clcB	3	15.8	0.45
0.8	0	ycaM	3	15.8	0.524
0.8	0	yadI	3	15.8	0.703
0.8	0	AG1	3	15.8	0.646
0.8	0	ptsI	3	16.05	0.5
0.8	0	clcB	3	16.05	0.451
0.8	0	ycaM	3	16.05	0.528
0.8	0	yadI	3	16.05	0.71
0.8	0	AG1	3	16.05	0.646
0.8	0	ptsI	3	16.3	0.506
0.8	0	clcB	3	16.3	0.458
0.8	0	ycaM	3	16.3	0.531
0.8	0	yadI	3	16.3	0.692
0.8	0	AG1	3	16.3	0.638
0.8	0	ptsI	3	16.55	0.513
0.8	0	clcB	3	16.55	0.456
0.8	0	ycaM	3	16.55	0.542
0.8	0	yadI	3	16.55	0.72
0.8	0	AG1	3	16.55	0.661
0.8	0	ptsI	3	16.8	0.522
0.8	0	clcB	3	16.8	0.464
0.8	0	ycaM	3	16.8	0.547
0.8	0	yadI	3	16.8	0.75
0.8	0	AG1	3	16.8	0.678
0.8	0	ptsI	3	17.05	0.52
0.8	0	clcB	3	17.05	0.466
0.8	0	ycaM	3	17.05	0.549
0.8	0	yadI	3	17.05	0.738
0.8	0	AG1	3	17.05	0.675
0.8	0	ptsI	3	17.3	0.531
0.8	0	clcB	3	17.3	0.467
0.8	0	ycaM	3	17.3	0.552
0.8	0	yadI	3	17.3	0.742
0.8	0	AG1	3	17.3	0.682
0.8	0	ptsI	3	17.55	0.537
0.8	0	clcB	3	17.55	0.47
0.8	0	ycaM	3	17.55	0.568
0.8	0	yadI	3	17.55	0.749
0.8	0	AG1	3	17.55	0.678
0.8	0	ptsI	3	17.8	0.541
0.8	0	clcB	3	17.8	0.474
0.8	0	ycaM	3	17.8	0.577
0.8	0	yadI	3	17.8	0.755
0.8	0	AG1	3	17.8	0.701
0.8	0	ptsI	3	18.05	0.552
0.8	0	clcB	3	18.05	0.475
0.8	0	ycaM	3	18.05	0.574
0.8	0	yadI	3	18.05	0.763
0.8	0	AG1	3	18.05	0.693
0.8	0	ptsI	3	18.3	0.554
0.8	0	clcB	3	18.3	0.477
0.8	0	ycaM	3	18.3	0.592

0.8	0	yadI	3	18.3	0.765
0.8	0	AG1	3	18.3	0.708
0.8	0	ptsI	3	18.55	0.561
0.8	0	clcB	3	18.55	0.48
0.8	0	ycaM	3	18.55	0.584
0.8	0	yadI	3	18.55	0.772
0.8	0	AG1	3	18.55	0.721
0.8	0	ptsI	3	18.8	0.567
0.8	0	clcB	3	18.8	0.48
0.8	0	ycaM	3	18.8	0.579
0.8	0	yadI	3	18.8	0.781
0.8	0	AG1	3	18.8	0.71
0.8	0	ptsI	3	19.05	0.576
0.8	0	clcB	3	19.05	0.487
0.8	0	ycaM	3	19.05	0.579
0.8	0	yadI	3	19.05	0.785
0.8	0	AG1	3	19.05	0.715
0.8	0	ptsI	3	19.3	0.588
0.8	0	clcB	3	19.3	0.489
0.8	0	ycaM	3	19.3	0.574
0.8	0	yadI	3	19.3	0.796
0.8	0	AG1	3	19.3	0.716
0.8	0	ptsI	3	19.55	0.607
0.8	0	clcB	3	19.55	0.496
0.8	0	ycaM	3	19.55	0.572
0.8	0	yadI	3	19.55	0.804
0.8	0	AG1	3	19.55	0.715
0.8	0	ptsI	3	19.8	0.597
0.8	0	clcB	3	19.8	0.503
0.8	0	ycaM	3	19.8	0.575
0.8	0	yadI	3	19.8	0.805
0.8	0	AG1	3	19.8	0.734
0.8	0	ptsI	3	20.05	0.604
0.8	0	clcB	3	20.05	0.507
0.8	0	ycaM	3	20.05	0.58
0.8	0	yadI	3	20.05	0.818
0.8	0	AG1	3	20.05	0.747
0.8	0	ptsI	3	20.3	0.593
0.8	0	clcB	3	20.3	0.503
0.8	0	ycaM	3	20.3	0.571
0.8	0	yadI	3	20.3	0.857
0.8	0	AG1	3	20.3	0.75
0.8	0	ptsI	3	20.55	0.601
0.8	0	clcB	3	20.55	0.509
0.8	0	ycaM	3	20.55	0.584
0.8	0	yadI	3	20.55	0.828
0.8	0	AG1	3	20.55	0.757
0.8	0	ptsI	3	20.8	0.601
0.8	0	clcB	3	20.8	0.517
0.8	0	ycaM	3	20.8	0.594
0.8	0	yadI	3	20.8	0.834
0.8	0	AG1	3	20.8	0.76
0.8	0	ptsI	3	21.05	0.598

0.8	0	clcB	3	21.05	0.529
0.8	0	ycaM	3	21.05	0.601
0.8	0	yadI	3	21.05	0.843
0.8	0	AG1	3	21.05	0.769
0.8	0	ptsI	3	21.3	0.598
0.8	0	clcB	3	21.3	0.536
0.8	0	ycaM	3	21.3	0.604
0.8	0	yadI	3	21.3	0.848
0.8	0	AG1	3	21.3	0.774
0.8	0	ptsI	3	21.55	0.598
0.8	0	clcB	3	21.55	0.537
0.8	0	ycaM	3	21.55	0.606
0.8	0	yadI	3	21.55	0.852
0.8	0	AG1	3	21.55	0.772
0.8	0	ptsI	3	21.8	0.594
0.8	0	clcB	3	21.8	0.542
0.8	0	ycaM	3	21.8	0.611
0.8	0	yadI	3	21.8	0.854
0.8	0	AG1	3	21.8	0.774
0.8	0	ptsI	3	22.05	0.606
0.8	0	clcB	3	22.05	0.537
0.8	0	ycaM	3	22.05	0.611
0.8	0	yadI	3	22.05	0.863
0.8	0	AG1	3	22.05	0.784
0.8	0	ptsI	3	22.3	0.602
0.8	0	clcB	3	22.3	0.537
0.8	0	ycaM	3	22.3	0.618
0.8	0	yadI	3	22.3	0.87
0.8	0	AG1	3	22.3	0.786
0.8	0	ptsI	3	22.55	0.604
0.8	0	clcB	3	22.55	0.537
0.8	0	ycaM	3	22.55	0.625
0.8	0	yadI	3	22.55	0.876
0.8	0	AG1	3	22.55	0.79
0.8	0	ptsI	3	22.8	0.608
0.8	0	clcB	3	22.8	0.55
0.8	0	ycaM	3	22.8	0.636
0.8	0	yadI	3	22.8	0.881
0.8	0	AG1	3	22.8	0.79
0.8	0	ptsI	3	23.05	0.606
0.8	0	clcB	3	23.05	0.541
0.8	0	ycaM	3	23.05	0.636
0.8	0	yadI	3	23.05	0.883
0.8	0	AG1	3	23.05	0.802
0.8	0	ptsI	3	23.3	0.609
0.8	0	clcB	3	23.3	0.542
0.8	0	ycaM	3	23.3	0.642
0.8	0	yadI	3	23.3	0.925
0.8	0	AG1	3	23.3	0.807
0.8	0	ptsI	3	23.55	0.615
0.8	0	clcB	3	23.55	0.544
0.8	0	ycaM	3	23.55	0.646
0.8	0	yadI	3	23.55	0.92

0.8	0	AG1	3	23.55	0.81
0.8	0	ptsI	3	23.8	0.616
0.8	0	clcB	3	23.8	0.546
0.8	0	ycaM	3	23.8	0.649
0.8	0	yadI	3	23.8	0.948
0.8	0	AG1	3	23.8	0.82
0.8	0	ptsI	3	24.05	0.617
0.8	0	clcB	3	24.05	0.547
0.8	0	ycaM	3	24.05	0.656
0.8	0	yadI	3	24.05	0.915
0.8	0	AG1	3	24.05	0.825
0.8	0	ptsI	3	24.3	0.63
0.8	0	clcB	3	24.3	0.555
0.8	0	ycaM	3	24.3	0.666
0.8	0	yadI	3	24.3	0.946
0.8	0	AG1	3	24.3	0.832
0.8	0	ptsI	4	0	0.197
0.8	0	clcB	4	0	0.211
0.8	0	ycaM	4	0	0.218
0.8	0	yadI	4	0	0.235
0.8	0	AG1	4	0	0.203
0.8	0	ptsI	4	0.25	0.198
0.8	0	clcB	4	0.25	0.207
0.8	0	ycaM	4	0.25	0.212
0.8	0	yadI	4	0.25	0.236
0.8	0	AG1	4	0.25	0.201
0.8	0	ptsI	4	0.5	0.196
0.8	0	clcB	4	0.5	0.207
0.8	0	ycaM	4	0.5	0.213
0.8	0	yadI	4	0.5	0.236
0.8	0	AG1	4	0.5	0.202
0.8	0	ptsI	4	0.75	0.203
0.8	0	clcB	4	0.75	0.212
0.8	0	ycaM	4	0.75	0.216
0.8	0	yadI	4	0.75	0.239
0.8	0	AG1	4	0.75	0.207
0.8	0	ptsI	4	1	0.206
0.8	0	clcB	4	1	0.215
0.8	0	ycaM	4	1	0.219
0.8	0	yadI	4	1	0.24
0.8	0	AG1	4	1	0.207
0.8	0	ptsI	4	1.25	0.214
0.8	0	clcB	4	1.25	0.222
0.8	0	ycaM	4	1.25	0.226
0.8	0	yadI	4	1.25	0.243
0.8	0	AG1	4	1.25	0.213
0.8	0	ptsI	4	1.5	0.212
0.8	0	clcB	4	1.5	0.224
0.8	0	ycaM	4	1.5	0.228
0.8	0	yadI	4	1.5	0.251
0.8	0	AG1	4	1.5	0.214
0.8	0	ptsI	4	1.75	0.224
0.8	0	clcB	4	1.75	0.237

0.8	0	ycaM	4	1.75	0.234
0.8	0	yadI	4	1.75	0.259
0.8	0	AG1	4	1.75	0.223
0.8	0	ptsI	4	2	0.235
0.8	0	clcB	4	2	0.251
0.8	0	ycaM	4	2	0.249
0.8	0	yadI	4	2	0.269
0.8	0	AG1	4	2	0.234
0.8	0	ptsI	4	2.25	0.25
0.8	0	clcB	4	2.25	0.262
0.8	0	ycaM	4	2.25	0.259
0.8	0	yadI	4	2.25	0.281
0.8	0	AG1	4	2.25	0.244
0.8	0	ptsI	4	2.5	0.248
0.8	0	clcB	4	2.5	0.273
0.8	0	ycaM	4	2.5	0.266
0.8	0	yadI	4	2.5	0.292
0.8	0	AG1	4	2.5	0.252
0.8	0	ptsI	4	2.75	0.258
0.8	0	clcB	4	2.75	0.289
0.8	0	ycaM	4	2.75	0.28
0.8	0	yadI	4	2.75	0.311
0.8	0	AG1	4	2.75	0.261
0.8	0	ptsI	4	3	0.28
0.8	0	clcB	4	3	0.309
0.8	0	ycaM	4	3	0.285
0.8	0	yadI	4	3	0.325
0.8	0	AG1	4	3	0.274
0.8	0	ptsI	4	3.25	0.3
0.8	0	clcB	4	3.25	0.326
0.8	0	ycaM	4	3.25	0.299
0.8	0	yadI	4	3.25	0.341
0.8	0	AG1	4	3.25	0.287
0.8	0	ptsI	4	3.5	0.319
0.8	0	clcB	4	3.5	0.341
0.8	0	ycaM	4	3.5	0.314
0.8	0	yadI	4	3.5	0.361
0.8	0	AG1	4	3.5	0.306
0.8	0	ptsI	4	3.75	0.327
0.8	0	clcB	4	3.75	0.352
0.8	0	ycaM	4	3.75	0.333
0.8	0	yadI	4	3.75	0.381
0.8	0	AG1	4	3.75	0.322
0.8	0	ptsI	4	4	0.347
0.8	0	clcB	4	4	0.365
0.8	0	ycaM	4	4	0.345
0.8	0	yadI	4	4	0.394
0.8	0	AG1	4	4	0.338
0.8	0	ptsI	4	4.35	0.339
0.8	0	clcB	4	4.35	0.281
0.8	0	ycaM	4	4.35	0.387
0.8	0	yadI	4	4.35	0.417
0.8	0	AG1	4	4.35	0.305

0.8	0	ptsI	4	4.6	0.352
0.8	0	clcB	4	4.6	0.278
0.8	0	ycaM	4	4.6	0.372
0.8	0	yadI	4	4.6	0.4
0.8	0	AG1	4	4.6	0.287
0.8	0	ptsI	4	4.85	0.366
0.8	0	clcB	4	4.85	0.284
0.8	0	ycaM	4	4.85	0.384
0.8	0	yadI	4	4.85	0.412
0.8	0	AG1	4	4.85	0.294
0.8	0	ptsI	4	5.1	0.379
0.8	0	clcB	4	5.1	0.295
0.8	0	ycaM	4	5.1	0.397
0.8	0	yadI	4	5.1	0.423
0.8	0	AG1	4	5.1	0.304
0.8	0	ptsI	4	5.35	0.391
0.8	0	clcB	4	5.35	0.303
0.8	0	ycaM	4	5.35	0.411
0.8	0	yadI	4	5.35	0.438
0.8	0	AG1	4	5.35	0.314
0.8	0	ptsI	4	5.6	0.406
0.8	0	clcB	4	5.6	0.309
0.8	0	ycaM	4	5.6	0.419
0.8	0	yadI	4	5.6	0.45
0.8	0	AG1	4	5.6	0.323
0.8	0	ptsI	4	5.85	0.411
0.8	0	clcB	4	5.85	0.317
0.8	0	ycaM	4	5.85	0.427
0.8	0	yadI	4	5.85	0.468
0.8	0	AG1	4	5.85	0.334
0.8	0	ptsI	4	6.1	0.425
0.8	0	clcB	4	6.1	0.329
0.8	0	ycaM	4	6.1	0.434
0.8	0	yadI	4	6.1	0.484
0.8	0	AG1	4	6.1	0.344
0.8	0	ptsI	4	6.35	0.437
0.8	0	clcB	4	6.35	0.336
0.8	0	ycaM	4	6.35	0.447
0.8	0	yadI	4	6.35	0.498
0.8	0	AG1	4	6.35	0.354
0.8	0	ptsI	4	6.6	0.449
0.8	0	clcB	4	6.6	0.346
0.8	0	ycaM	4	6.6	0.456
0.8	0	yadI	4	6.6	0.512
0.8	0	AG1	4	6.6	0.363
0.8	0	ptsI	4	6.85	0.462
0.8	0	clcB	4	6.85	0.353
0.8	0	ycaM	4	6.85	0.467
0.8	0	yadI	4	6.85	0.524
0.8	0	AG1	4	6.85	0.372
0.8	0	ptsI	4	7.1	0.472
0.8	0	clcB	4	7.1	0.364
0.8	0	ycaM	4	7.1	0.476

0.8	0	yadI	4	7.1	0.531
0.8	0	AG1	4	7.1	0.381
0.8	0	ptsI	4	7.35	0.484
0.8	0	clcB	4	7.35	0.369
0.8	0	ycaM	4	7.35	0.478
0.8	0	yadI	4	7.35	0.542
0.8	0	AG1	4	7.35	0.39
0.8	0	ptsI	4	7.6	0.492
0.8	0	clcB	4	7.6	0.376
0.8	0	ycaM	4	7.6	0.484
0.8	0	yadI	4	7.6	0.549
0.8	0	AG1	4	7.6	0.401
0.8	0	ptsI	4	7.85	0.5
0.8	0	clcB	4	7.85	0.387
0.8	0	ycaM	4	7.85	0.487
0.8	0	yadI	4	7.85	0.559
0.8	0	AG1	4	7.85	0.406
0.8	0	ptsI	4	8.1	0.51
0.8	0	clcB	4	8.1	0.396
0.8	0	ycaM	4	8.1	0.49
0.8	0	yadI	4	8.1	0.566
0.8	0	AG1	4	8.1	0.415
0.8	0	ptsI	4	8.35	0.52
0.8	0	clcB	4	8.35	0.402
0.8	0	ycaM	4	8.35	0.492
0.8	0	yadI	4	8.35	0.574
0.8	0	AG1	4	8.35	0.421
0.8	0	ptsI	4	8.83333	0.416
0.8	0	clcB	4	8.83333	0.4
0.8	0	ycaM	4	8.83333	0.438
0.8	0	yadI	4	8.83333	0.553
0.8	0	AG1	4	8.83333	0.465
0.8	0	ptsI	4	9.08333	0.416
0.8	0	clcB	4	9.08333	0.397
0.8	0	ycaM	4	9.08333	0.436
0.8	0	yadI	4	9.08333	0.552
0.8	0	AG1	4	9.08333	0.462
0.8	0	ptsI	4	9.33333	0.427
0.8	0	clcB	4	9.33333	0.4
0.8	0	ycaM	4	9.33333	0.44
0.8	0	yadI	4	9.33333	0.555
0.8	0	AG1	4	9.33333	0.471
0.8	0	ptsI	4	9.58333	0.439
0.8	0	clcB	4	9.58333	0.401
0.8	0	ycaM	4	9.58333	0.45
0.8	0	yadI	4	9.58333	0.566
0.8	0	AG1	4	9.58333	0.483
0.8	0	ptsI	4	9.83333	0.447
0.8	0	clcB	4	9.83333	0.406
0.8	0	ycaM	4	9.83333	0.456
0.8	0	yadI	4	9.83333	0.577
0.8	0	AG1	4	9.83333	0.495
0.8	0	ptsI	4	10.0833	0.453

0.8	0	clcB	4	10.0833	0.412
0.8	0	ycaM	4	10.0833	0.462
0.8	0	yadI	4	10.0833	0.583
0.8	0	AG1	4	10.0833	0.502
0.8	0	ptsI	4	10.3333	0.459
0.8	0	clcB	4	10.3333	0.412
0.8	0	ycaM	4	10.3333	0.466
0.8	0	yadI	4	10.3333	0.59
0.8	0	AG1	4	10.3333	0.51
0.8	0	ptsI	4	10.5833	0.466
0.8	0	clcB	4	10.5833	0.418
0.8	0	ycaM	4	10.5833	0.473
0.8	0	yadI	4	10.5833	0.596
0.8	0	AG1	4	10.5833	0.519
0.8	0	ptsI	4	10.8333	0.473
0.8	0	clcB	4	10.8333	0.42
0.8	0	ycaM	4	10.8333	0.477
0.8	0	yadI	4	10.8333	0.602
0.8	0	AG1	4	10.8333	0.525
0.8	0	ptsI	4	11.0833	0.482
0.8	0	clcB	4	11.0833	0.423
0.8	0	ycaM	4	11.0833	0.481
0.8	0	yadI	4	11.0833	0.608
0.8	0	AG1	4	11.0833	0.526
0.8	0	ptsI	4	11.3333	0.489
0.8	0	clcB	4	11.3333	0.432
0.8	0	ycaM	4	11.3333	0.486
0.8	0	yadI	4	11.3333	0.615
0.8	0	AG1	4	11.3333	0.536
0.8	0	ptsI	4	11.5833	0.497
0.8	0	clcB	4	11.5833	0.433
0.8	0	ycaM	4	11.5833	0.489
0.8	0	yadI	4	11.5833	0.622
0.8	0	AG1	4	11.5833	0.539
0.8	0	ptsI	4	11.8333	0.505
0.8	0	clcB	4	11.8333	0.433
0.8	0	ycaM	4	11.8333	0.492
0.8	0	yadI	4	11.8333	0.625
0.8	0	AG1	4	11.8333	0.542
0.8	0	ptsI	4	12.0833	0.511
0.8	0	clcB	4	12.0833	0.438
0.8	0	ycaM	4	12.0833	0.498
0.8	0	yadI	4	12.0833	0.631
0.8	0	AG1	4	12.0833	0.549
0.8	0	ptsI	4	12.3333	0.516
0.8	0	clcB	4	12.3333	0.443
0.8	0	ycaM	4	12.3333	0.504
0.8	0	yadI	4	12.3333	0.639
0.8	0	AG1	4	12.3333	0.557
0.8	0	ptsI	4	12.5833	0.521
0.8	0	clcB	4	12.5833	0.444
0.8	0	ycaM	4	12.5833	0.508
0.8	0	yadI	4	12.5833	0.64

0.8	0	AG1	4	12.5833	0.561
0.8	0	ptsI	4	12.8333	0.528
0.8	0	clcB	4	12.8333	0.445
0.8	0	ycaM	4	12.8333	0.512
0.8	0	yadI	4	12.8333	0.643
0.8	0	AG1	4	12.8333	0.567
0.8	0	ptsI	4	13.0833	0.536
0.8	0	clcB	4	13.0833	0.448
0.8	0	ycaM	4	13.0833	0.516
0.8	0	yadI	4	13.0833	0.65
0.8	0	AG1	4	13.0833	0.572
0.8	0	ptsI	4	13.3333	0.541
0.8	0	clcB	4	13.3333	0.45
0.8	0	ycaM	4	13.3333	0.523
0.8	0	yadI	4	13.3333	0.652
0.8	0	AG1	4	13.3333	0.578
0.8	0	ptsI	4	13.5833	0.544
0.8	0	clcB	4	13.5833	0.455
0.8	0	ycaM	4	13.5833	0.524
0.8	0	yadI	4	13.5833	0.657
0.8	0	AG1	4	13.5833	0.582
0.8	0	ptsI	4	13.8333	0.552
0.8	0	clcB	4	13.8333	0.456
0.8	0	ycaM	4	13.8333	0.53
0.8	0	yadI	4	13.8333	0.657
0.8	0	AG1	4	13.8333	0.582
0.8	0	ptsI	4	14.0833	0.555
0.8	0	clcB	4	14.0833	0.457
0.8	0	ycaM	4	14.0833	0.533
0.8	0	yadI	4	14.0833	0.663
0.8	0	AG1	4	14.0833	0.587
0.8	0	ptsI	4	14.3333	0.56
0.8	0	clcB	4	14.3333	0.462
0.8	0	ycaM	4	14.3333	0.533
0.8	0	yadI	4	14.3333	0.663
0.8	0	AG1	4	14.3333	0.589
0.8	0	ptsI	4	14.5833	0.562
0.8	0	clcB	4	14.5833	0.46
0.8	0	ycaM	4	14.5833	0.535
0.8	0	yadI	4	14.5833	0.667
0.8	0	AG1	4	14.5833	0.592
0.8	0	ptsI	4	14.8333	0.567
0.8	0	clcB	4	14.8333	0.462
0.8	0	ycaM	4	14.8333	0.539
0.8	0	yadI	4	14.8333	0.667
0.8	0	AG1	4	14.8333	0.596
0.8	0	ptsI	4	15.0833	0.572
0.8	0	clcB	4	15.0833	0.464
0.8	0	ycaM	4	15.0833	0.539
0.8	0	yadI	4	15.0833	0.669
0.8	0	AG1	4	15.0833	0.597
0.8	0	ptsI	4	15.3333	0.575
0.8	0	clcB	4	15.3333	0.467

0.8	0	ycaM	4	15.3333	0.545
0.8	0	yadI	4	15.3333	0.67
0.8	0	AG1	4	15.3333	0.597
0.8	0	ptsI	4	15.5833	0.576
0.8	0	clcB	4	15.5833	0.47
0.8	0	ycaM	4	15.5833	0.544
0.8	0	yadI	4	15.5833	0.675
0.8	0	AG1	4	15.5833	0.6
0.8	0	ptsI	4	15.8333	0.583
0.8	0	clcB	4	15.8333	0.472
0.8	0	ycaM	4	15.8333	0.547
0.8	0	yadI	4	15.8333	0.678
0.8	0	AG1	4	15.8333	0.602
0.8	0	ptsI	4	16.0833	0.588
0.8	0	clcB	4	16.0833	0.475
0.8	0	ycaM	4	16.0833	0.55
0.8	0	yadI	4	16.0833	0.677
0.8	0	AG1	4	16.0833	0.605
0.8	0	ptsI	4	16.3333	0.589
0.8	0	clcB	4	16.3333	0.473
0.8	0	ycaM	4	16.3333	0.551
0.8	0	yadI	4	16.3333	0.682
0.8	0	AG1	4	16.3333	0.604
0.8	0	ptsI	4	16.5833	0.594
0.8	0	clcB	4	16.5833	0.473
0.8	0	ycaM	4	16.5833	0.553
0.8	0	yadI	4	16.5833	0.685
0.8	0	AG1	4	16.5833	0.608
0.8	0	ptsI	4	16.8333	0.596
0.8	0	clcB	4	16.8333	0.476
0.8	0	ycaM	4	16.8333	0.555
0.8	0	yadI	4	16.8333	0.686
0.8	0	AG1	4	16.8333	0.607
0.8	0	ptsI	4	17.0833	0.601
0.8	0	clcB	4	17.0833	0.478
0.8	0	ycaM	4	17.0833	0.558
0.8	0	yadI	4	17.0833	0.688
0.8	0	AG1	4	17.0833	0.606
0.8	0	ptsI	4	17.3333	0.603
0.8	0	clcB	4	17.3333	0.477
0.8	0	ycaM	4	17.3333	0.563
0.8	0	yadI	4	17.3333	0.694
0.8	0	AG1	4	17.3333	0.608
0.8	0	ptsI	4	17.5833	0.607
0.8	0	clcB	4	17.5833	0.481
0.8	0	ycaM	4	17.5833	0.559
0.8	0	yadI	4	17.5833	0.694
0.8	0	AG1	4	17.5833	0.611
0.8	0	ptsI	4	17.8333	0.609
0.8	0	clcB	4	17.8333	0.481
0.8	0	ycaM	4	17.8333	0.558
0.8	0	yadI	4	17.8333	0.693
0.8	0	AG1	4	17.8333	0.61

0.8	0	ptsI	4	18.0833	0.617
0.8	0	clcB	4	18.0833	0.481
0.8	0	ycaM	4	18.0833	0.563
0.8	0	yadI	4	18.0833	0.698
0.8	0	AG1	4	18.0833	0.612
0.8	0	ptsI	4	18.3333	0.62
0.8	0	clcB	4	18.3333	0.482
0.8	0	ycaM	4	18.3333	0.565
0.8	0	yadI	4	18.3333	0.701
0.8	0	AG1	4	18.3333	0.613
0.8	0	ptsI	4	18.5833	0.62
0.8	0	clcB	4	18.5833	0.482
0.8	0	ycaM	4	18.5833	0.566
0.8	0	yadI	4	18.5833	0.704
0.8	0	AG1	4	18.5833	0.613
0.8	0	ptsI	4	18.8333	0.624
0.8	0	clcB	4	18.8333	0.483
0.8	0	ycaM	4	18.8333	0.563
0.8	0	yadI	4	18.8333	0.703
0.8	0	AG1	4	18.8333	0.614
0.8	0	ptsI	4	19.0833	0.628
0.8	0	clcB	4	19.0833	0.486
0.8	0	ycaM	4	19.0833	0.568
0.8	0	yadI	4	19.0833	0.705
0.8	0	AG1	4	19.0833	0.619
0.8	0	ptsI	4	19.3333	0.628
0.8	0	clcB	4	19.3333	0.484
0.8	0	ycaM	4	19.3333	0.572
0.8	0	yadI	4	19.3333	0.709
0.8	0	AG1	4	19.3333	0.618
0.8	0	ptsI	4	19.5833	0.628
0.8	0	clcB	4	19.5833	0.489
0.8	0	ycaM	4	19.5833	0.57
0.8	0	yadI	4	19.5833	0.708
0.8	0	AG1	4	19.5833	0.616
0.8	0	ptsI	4	19.8333	0.632
0.8	0	clcB	4	19.8333	0.487
0.8	0	ycaM	4	19.8333	0.57
0.8	0	yadI	4	19.8333	0.706
0.8	0	AG1	4	19.8333	0.617
0.8	0	ptsI	4	20.0833	0.626
0.8	0	clcB	4	20.0833	0.489
0.8	0	ycaM	4	20.0833	0.572
0.8	0	yadI	4	20.0833	0.703
0.8	0	AG1	4	20.0833	0.626
0.8	0	ptsI	4	20.3333	0.633
0.8	0	clcB	4	20.3333	0.487
0.8	0	ycaM	4	20.3333	0.575
0.8	0	yadI	4	20.3333	0.71
0.8	0	AG1	4	20.3333	0.621
0.8	0	ptsI	4	20.5833	0.637
0.8	0	clcB	4	20.5833	0.49
0.8	0	ycaM	4	20.5833	0.574

0.8	0	yadI	4	20.5833	0.71
0.8	0	AG1	4	20.5833	0.624
0.8	0	ptsI	4	20.8333	0.64
0.8	0	clcB	4	20.8333	0.486
0.8	0	ycaM	4	20.8333	0.577
0.8	0	yadI	4	20.8333	0.711
0.8	0	AG1	4	20.8333	0.621
0.8	0	ptsI	4	21.0833	0.638
0.8	0	clcB	4	21.0833	0.492
0.8	0	ycaM	4	21.0833	0.578
0.8	0	yadI	4	21.0833	0.709
0.8	0	AG1	4	21.0833	0.621
0.8	0	ptsI	4	21.3333	0.64
0.8	0	clcB	4	21.3333	0.493
0.8	0	ycaM	4	21.3333	0.58
0.8	0	yadI	4	21.3333	0.708
0.8	0	AG1	4	21.3333	0.623
0.8	0	ptsI	4	21.5833	0.644
0.8	0	clcB	4	21.5833	0.496
0.8	0	ycaM	4	21.5833	0.577
0.8	0	yadI	4	21.5833	0.71
0.8	0	AG1	4	21.5833	0.623
0.8	0	ptsI	4	21.8333	0.644
0.8	0	clcB	4	21.8333	0.497
0.8	0	ycaM	4	21.8333	0.582
0.8	0	yadI	4	21.8333	0.713
0.8	0	AG1	4	21.8333	0.624
0.8	0	ptsI	4	22.0833	0.646
0.8	0	clcB	4	22.0833	0.498
0.8	0	ycaM	4	22.0833	0.581
0.8	0	yadI	4	22.0833	0.713
0.8	0	AG1	4	22.0833	0.622
0.8	0	ptsI	4	22.3333	0.65
0.8	0	clcB	4	22.3333	0.501
0.8	0	ycaM	4	22.3333	0.581
0.8	0	yadI	4	22.3333	0.711
0.8	0	AG1	4	22.3333	0.625
0.8	0	ptsI	4	22.5833	0.651
0.8	0	clcB	4	22.5833	0.501
0.8	0	ycaM	4	22.5833	0.582
0.8	0	yadI	4	22.5833	0.713
0.8	0	AG1	4	22.5833	0.624
0.8	0	ptsI	4	22.8333	0.65
0.8	0	clcB	4	22.8333	0.504
0.8	0	ycaM	4	22.8333	0.584
0.8	0	yadI	4	22.8333	0.709
0.8	0	AG1	4	22.8333	0.625
0.8	0	ptsI	4	23.0833	0.657
0.8	0	clcB	4	23.0833	0.508
0.8	0	ycaM	4	23.0833	0.581
0.8	0	yadI	4	23.0833	0.713
0.8	0	AG1	4	23.0833	0.624
0.8	0	ptsI	4	23.3333	0.655

0.8	0	clcB	4	23.3333	0.511
0.8	0	ycaM	4	23.3333	0.583
0.8	0	yadI	4	23.3333	0.712
0.8	0	AG1	4	23.3333	0.629
0.8	0	ptsI	4	23.5833	0.66
0.8	0	clcB	4	23.5833	0.509
0.8	0	ycaM	4	23.5833	0.583
0.8	0	yadI	4	23.5833	0.712
0.8	0	AG1	4	23.5833	0.626
0.8	0	ptsI	4	23.8333	0.668
0.8	0	clcB	4	23.8333	0.512
0.8	0	ycaM	4	23.8333	0.584
0.8	0	yadI	4	23.8333	0.712
0.8	0	AG1	4	23.8333	0.627
0.8	0	ptsI	4	24.0833	0.668
0.8	0	clcB	4	24.0833	0.514
0.8	0	ycaM	4	24.0833	0.582
0.8	0	yadI	4	24.0833	0.713
0.8	0	AG1	4	24.0833	0.629
0.8	0	ptsI	5	0	0.156
0.8	0	clcB	5	0	0.188
0.8	0	ycaM	5	0	0.163
0.8	0	yadI	5	0	0.166
0.8	0	AG1	5	0	0.167
0.8	0	ptsI	5	0.35	0.156
0.8	0	clcB	5	0.35	0.187
0.8	0	ycaM	5	0.35	0.156
0.8	0	yadI	5	0.35	0.164
0.8	0	AG1	5	0.35	0.163
0.8	0	ptsI	5	0.6	0.156
0.8	0	clcB	5	0.6	0.188
0.8	0	ycaM	5	0.6	0.156
0.8	0	yadI	5	0.6	0.164
0.8	0	AG1	5	0.6	0.163
0.8	0	ptsI	5	0.85	0.155
0.8	0	clcB	5	0.85	0.187
0.8	0	ycaM	5	0.85	0.156
0.8	0	yadI	5	0.85	0.165
0.8	0	AG1	5	0.85	0.163
0.8	0	ptsI	5	1.1	0.158
0.8	0	clcB	5	1.1	0.188
0.8	0	ycaM	5	1.1	0.158
0.8	0	yadI	5	1.1	0.167
0.8	0	AG1	5	1.1	0.166
0.8	0	ptsI	5	1.35	0.158
0.8	0	clcB	5	1.35	0.189
0.8	0	ycaM	5	1.35	0.16
0.8	0	yadI	5	1.35	0.166
0.8	0	AG1	5	1.35	0.167
0.8	0	ptsI	5	1.6	0.162
0.8	0	clcB	5	1.6	0.192
0.8	0	ycaM	5	1.6	0.163
0.8	0	yadI	5	1.6	0.172

0.8	0	AG1	5	1.6	0.17
0.8	0	ptsI	5	1.85	0.164
0.8	0	clcB	5	1.85	0.195
0.8	0	ycaM	5	1.85	0.167
0.8	0	yadI	5	1.85	0.175
0.8	0	AG1	5	1.85	0.174
0.8	0	ptsI	5	2.1	0.167
0.8	0	clcB	5	2.1	0.198
0.8	0	ycaM	5	2.1	0.172
0.8	0	yadI	5	2.1	0.179
0.8	0	AG1	5	2.1	0.181
0.8	0	ptsI	5	2.35	0.172
0.8	0	clcB	5	2.35	0.203
0.8	0	ycaM	5	2.35	0.178
0.8	0	yadI	5	2.35	0.184
0.8	0	AG1	5	2.35	0.188
0.8	0	ptsI	5	2.6	0.173
0.8	0	clcB	5	2.6	0.207
0.8	0	ycaM	5	2.6	0.182
0.8	0	yadI	5	2.6	0.186
0.8	0	AG1	5	2.6	0.187
0.8	0	ptsI	5	2.85	0.175
0.8	0	clcB	5	2.85	0.214
0.8	0	ycaM	5	2.85	0.191
0.8	0	yadI	5	2.85	0.196
0.8	0	AG1	5	2.85	0.2
0.8	0	ptsI	5	3.1	0.179
0.8	0	clcB	5	3.1	0.223
0.8	0	ycaM	5	3.1	0.202
0.8	0	yadI	5	3.1	0.206
0.8	0	AG1	5	3.1	0.212
0.8	0	ptsI	5	3.35	0.185
0.8	0	clcB	5	3.35	0.234
0.8	0	ycaM	5	3.35	0.214
0.8	0	yadI	5	3.35	0.218
0.8	0	AG1	5	3.35	0.229
0.8	0	ptsI	5	3.6	0.192
0.8	0	clcB	5	3.6	0.248
0.8	0	ycaM	5	3.6	0.226
0.8	0	yadI	5	3.6	0.23
0.8	0	AG1	5	3.6	0.246
0.8	0	ptsI	5	3.85	0.201
0.8	0	clcB	5	3.85	0.26
0.8	0	ycaM	5	3.85	0.237
0.8	0	yadI	5	3.85	0.244
0.8	0	AG1	5	3.85	0.263
0.8	0	ptsI	5	4.38333	0.212
0.8	0	clcB	5	4.38333	0.25
0.8	0	ycaM	5	4.38333	0.244
0.8	0	yadI	5	4.38333	0.258
0.8	0	AG1	5	4.38333	0.28
0.8	0	ptsI	5	4.63333	0.212
0.8	0	clcB	5	4.63333	0.24

0.8	0	ycaM	5	4.63333	0.247
0.8	0	yadI	5	4.63333	0.251
0.8	0	AG1	5	4.63333	0.28
0.8	0	ptsI	5	4.88333	0.217
0.8	0	clcB	5	4.88333	0.241
0.8	0	ycaM	5	4.88333	0.254
0.8	0	yadI	5	4.88333	0.266
0.8	0	AG1	5	4.88333	0.29
0.8	0	ptsI	5	5.13333	0.225
0.8	0	clcB	5	5.13333	0.248
0.8	0	ycaM	5	5.13333	0.266
0.8	0	yadI	5	5.13333	0.28
0.8	0	AG1	5	5.13333	0.3
0.8	0	ptsI	5	5.38333	0.233
0.8	0	clcB	5	5.38333	0.256
0.8	0	ycaM	5	5.38333	0.279
0.8	0	yadI	5	5.38333	0.295
0.8	0	AG1	5	5.38333	0.314
0.8	0	ptsI	5	5.63333	0.244
0.8	0	clcB	5	5.63333	0.264
0.8	0	ycaM	5	5.63333	0.29
0.8	0	yadI	5	5.63333	0.31
0.8	0	AG1	5	5.63333	0.326
0.8	0	ptsI	5	5.88333	0.25
0.8	0	clcB	5	5.88333	0.276
0.8	0	ycaM	5	5.88333	0.304
0.8	0	yadI	5	5.88333	0.324
0.8	0	AG1	5	5.88333	0.336
0.8	0	ptsI	5	6.13333	0.26
0.8	0	clcB	5	6.13333	0.287
0.8	0	ycaM	5	6.13333	0.315
0.8	0	yadI	5	6.13333	0.34
0.8	0	AG1	5	6.13333	0.346
0.8	0	ptsI	5	6.38333	0.268
0.8	0	clcB	5	6.38333	0.304
0.8	0	ycaM	5	6.38333	0.327
0.8	0	yadI	5	6.38333	0.355
0.8	0	AG1	5	6.38333	0.36
0.8	0	ptsI	5	6.63333	0.278
0.8	0	clcB	5	6.63333	0.32
0.8	0	ycaM	5	6.63333	0.338
0.8	0	yadI	5	6.63333	0.367
0.8	0	AG1	5	6.63333	0.374
0.8	0	ptsI	5	6.88333	0.288
0.8	0	clcB	5	6.88333	0.334
0.8	0	ycaM	5	6.88333	0.351
0.8	0	yadI	5	6.88333	0.38
0.8	0	AG1	5	6.88333	0.389
0.8	0	ptsI	5	7.13333	0.295
0.8	0	clcB	5	7.13333	0.349
0.8	0	ycaM	5	7.13333	0.364
0.8	0	yadI	5	7.13333	0.396
0.8	0	AG1	5	7.13333	0.405

0.8	0	ptsI	5	7.38333	0.304
0.8	0	clcB	5	7.38333	0.365
0.8	0	ycaM	5	7.38333	0.373
0.8	0	yadI	5	7.38333	0.415
0.8	0	AG1	5	7.38333	0.42
0.8	0	ptsI	5	7.63333	0.314
0.8	0	clcB	5	7.63333	0.382
0.8	0	ycaM	5	7.63333	0.385
0.8	0	yadI	5	7.63333	0.446
0.8	0	AG1	5	7.63333	0.43
0.8	0	ptsI	5	7.88333	0.325
0.8	0	clcB	5	7.88333	0.39
0.8	0	ycaM	5	7.88333	0.392
0.8	0	yadI	5	7.88333	0.461
0.8	0	AG1	5	7.88333	0.443
0.8	0	ptsI	5	8.13333	0.338
0.8	0	clcB	5	8.13333	0.4
0.8	0	ycaM	5	8.13333	0.404
0.8	0	yadI	5	8.13333	0.468
0.8	0	AG1	5	8.13333	0.455
0.8	0	ptsI	5	8.38333	0.347
0.8	0	clcB	5	8.38333	0.41
0.8	0	ycaM	5	8.38333	0.415
0.8	0	yadI	5	8.38333	0.478
0.8	0	AG1	5	8.38333	0.47
0.8	0	ptsI	5	8.88333	0.342
0.8	0	clcB	5	8.88333	0.362
0.8	0	ycaM	5	8.88333	0.34
0.8	0	yadI	5	8.88333	0.395
0.8	0	AG1	5	8.88333	0.525
0.8	0	ptsI	5	9.13333	0.345
0.8	0	clcB	5	9.13333	0.371
0.8	0	ycaM	5	9.13333	0.346
0.8	0	yadI	5	9.13333	0.394
0.8	0	AG1	5	9.13333	0.519
0.8	0	ptsI	5	9.38333	0.362
0.8	0	clcB	5	9.38333	0.38
0.8	0	ycaM	5	9.38333	0.359
0.8	0	yadI	5	9.38333	0.41
0.8	0	AG1	5	9.38333	0.534
0.8	0	ptsI	5	9.63333	0.366
0.8	0	clcB	5	9.63333	0.396
0.8	0	ycaM	5	9.63333	0.367
0.8	0	yadI	5	9.63333	0.416
0.8	0	AG1	5	9.63333	0.551
0.8	0	ptsI	5	9.88333	0.38
0.8	0	clcB	5	9.88333	0.407
0.8	0	ycaM	5	9.88333	0.376
0.8	0	yadI	5	9.88333	0.426
0.8	0	AG1	5	9.88333	0.564
0.8	0	ptsI	5	10.1333	0.39
0.8	0	clcB	5	10.1333	0.416
0.8	0	ycaM	5	10.1333	0.387

0.8	0	yadI	5	10.1333	0.432
0.8	0	AG1	5	10.1333	0.574
0.8	0	ptsI	5	10.3833	0.402
0.8	0	clcB	5	10.3833	0.425
0.8	0	ycaM	5	10.3833	0.394
0.8	0	yadI	5	10.3833	0.442
0.8	0	AG1	5	10.3833	0.585
0.8	0	ptsI	5	10.6333	0.412
0.8	0	clcB	5	10.6333	0.43
0.8	0	ycaM	5	10.6333	0.399
0.8	0	yadI	5	10.6333	0.446
0.8	0	AG1	5	10.6333	0.591
0.8	0	ptsI	5	10.8833	0.422
0.8	0	clcB	5	10.8833	0.433
0.8	0	ycaM	5	10.8833	0.404
0.8	0	yadI	5	10.8833	0.456
0.8	0	AG1	5	10.8833	0.602
0.8	0	ptsI	5	11.1333	0.429
0.8	0	clcB	5	11.1333	0.438
0.8	0	ycaM	5	11.1333	0.404
0.8	0	yadI	5	11.1333	0.458
0.8	0	AG1	5	11.1333	0.608
0.8	0	ptsI	5	11.3833	0.439
0.8	0	clcB	5	11.3833	0.441
0.8	0	ycaM	5	11.3833	0.408
0.8	0	yadI	5	11.3833	0.469
0.8	0	AG1	5	11.3833	0.615
0.8	0	ptsI	5	11.6333	0.447
0.8	0	clcB	5	11.6333	0.445
0.8	0	ycaM	5	11.6333	0.411
0.8	0	yadI	5	11.6333	0.474
0.8	0	AG1	5	11.6333	0.624
0.8	0	ptsI	5	11.8833	0.455
0.8	0	clcB	5	11.8833	0.45
0.8	0	ycaM	5	11.8833	0.414
0.8	0	yadI	5	11.8833	0.479
0.8	0	AG1	5	11.8833	0.634
0.8	0	ptsI	5	12.1333	0.465
0.8	0	clcB	5	12.1333	0.455
0.8	0	ycaM	5	12.1333	0.42
0.8	0	yadI	5	12.1333	0.486
0.8	0	AG1	5	12.1333	0.64
0.8	0	ptsI	5	12.3833	0.477
0.8	0	clcB	5	12.3833	0.457
0.8	0	ycaM	5	12.3833	0.419
0.8	0	yadI	5	12.3833	0.49
0.8	0	AG1	5	12.3833	0.65
0.8	0	ptsI	5	12.6333	0.478
0.8	0	clcB	5	12.6333	0.463
0.8	0	ycaM	5	12.6333	0.427
0.8	0	yadI	5	12.6333	0.496
0.8	0	AG1	5	12.6333	0.655
0.8	0	ptsI	5	12.8833	0.483

0.8	0	clcB	5	12.8833	0.468
0.8	0	ycaM	5	12.8833	0.429
0.8	0	yadI	5	12.8833	0.502
0.8	0	AG1	5	12.8833	0.663
0.8	0	ptsI	5	13.1333	0.491
0.8	0	clcB	5	13.1333	0.472
0.8	0	ycaM	5	13.1333	0.434
0.8	0	yadI	5	13.1333	0.507
0.8	0	AG1	5	13.1333	0.668
0.8	0	ptsI	5	13.3833	0.495
0.8	0	clcB	5	13.3833	0.476
0.8	0	ycaM	5	13.3833	0.436
0.8	0	yadI	5	13.3833	0.511
0.8	0	AG1	5	13.3833	0.674
0.8	0	ptsI	5	13.6333	0.5
0.8	0	clcB	5	13.6333	0.48
0.8	0	ycaM	5	13.6333	0.44
0.8	0	yadI	5	13.6333	0.516
0.8	0	AG1	5	13.6333	0.679
0.8	0	ptsI	5	13.8833	0.502
0.8	0	clcB	5	13.8833	0.484
0.8	0	ycaM	5	13.8833	0.441
0.8	0	yadI	5	13.8833	0.518
0.8	0	AG1	5	13.8833	0.684
0.8	0	ptsI	5	14.1333	0.507
0.8	0	clcB	5	14.1333	0.485
0.8	0	ycaM	5	14.1333	0.447
0.8	0	yadI	5	14.1333	0.527
0.8	0	AG1	5	14.1333	0.69
0.8	0	ptsI	5	14.3833	0.504
0.8	0	clcB	5	14.3833	0.486
0.8	0	ycaM	5	14.3833	0.446
0.8	0	yadI	5	14.3833	0.532
0.8	0	AG1	5	14.3833	0.691
0.8	0	ptsI	5	14.6333	0.505
0.8	0	clcB	5	14.6333	0.491
0.8	0	ycaM	5	14.6333	0.448
0.8	0	yadI	5	14.6333	0.528
0.8	0	AG1	5	14.6333	0.696
0.8	0	ptsI	5	14.8833	0.513
0.8	0	clcB	5	14.8833	0.495
0.8	0	ycaM	5	14.8833	0.452
0.8	0	yadI	5	14.8833	0.533
0.8	0	AG1	5	14.8833	0.705
0.8	0	ptsI	5	15.1333	0.515
0.8	0	clcB	5	15.1333	0.496
0.8	0	ycaM	5	15.1333	0.453
0.8	0	yadI	5	15.1333	0.539
0.8	0	AG1	5	15.1333	0.709
0.8	0	ptsI	5	15.3833	0.521
0.8	0	clcB	5	15.3833	0.499
0.8	0	ycaM	5	15.3833	0.459
0.8	0	yadI	5	15.3833	0.54

0.8	0	AG1	5	15.3833	0.711
0.8	0	ptsI	5	15.6333	0.521
0.8	0	clcB	5	15.6333	0.502
0.8	0	ycaM	5	15.6333	0.459
0.8	0	yadI	5	15.6333	0.541
0.8	0	AG1	5	15.6333	0.718
0.8	0	ptsI	5	15.8833	0.526
0.8	0	clcB	5	15.8833	0.503
0.8	0	ycaM	5	15.8833	0.461
0.8	0	yadI	5	15.8833	0.544
0.8	0	AG1	5	15.8833	0.72
0.8	0	ptsI	5	16.1333	0.536
0.8	0	clcB	5	16.1333	0.5
0.8	0	ycaM	5	16.1333	0.466
0.8	0	yadI	5	16.1333	0.553
0.8	0	AG1	5	16.1333	0.725
0.8	0	ptsI	5	16.3833	0.534
0.8	0	clcB	5	16.3833	0.508
0.8	0	ycaM	5	16.3833	0.466
0.8	0	yadI	5	16.3833	0.549
0.8	0	AG1	5	16.3833	0.725
0.8	0	ptsI	5	16.6333	0.533
0.8	0	clcB	5	16.6333	0.509
0.8	0	ycaM	5	16.6333	0.47
0.8	0	yadI	5	16.6333	0.551
0.8	0	AG1	5	16.6333	0.732
0.8	0	ptsI	5	16.8833	0.538
0.8	0	clcB	5	16.8833	0.51
0.8	0	ycaM	5	16.8833	0.47
0.8	0	yadI	5	16.8833	0.555
0.8	0	AG1	5	16.8833	0.733
0.8	0	ptsI	5	17.1333	0.541
0.8	0	clcB	5	17.1333	0.511
0.8	0	ycaM	5	17.1333	0.47
0.8	0	yadI	5	17.1333	0.551
0.8	0	AG1	5	17.1333	0.741
0.8	0	ptsI	5	17.3833	0.541
0.8	0	clcB	5	17.3833	0.513
0.8	0	ycaM	5	17.3833	0.475
0.8	0	yadI	5	17.3833	0.556
0.8	0	AG1	5	17.3833	0.742
0.8	0	ptsI	5	17.6333	0.543
0.8	0	clcB	5	17.6333	0.512
0.8	0	ycaM	5	17.6333	0.475
0.8	0	yadI	5	17.6333	0.556
0.8	0	AG1	5	17.6333	0.748
0.8	0	ptsI	5	17.8833	0.556
0.8	0	clcB	5	17.8833	0.514
0.8	0	ycaM	5	17.8833	0.477
0.8	0	yadI	5	17.8833	0.558
0.8	0	AG1	5	17.8833	0.75
0.8	0	ptsI	5	18.1333	0.55
0.8	0	clcB	5	18.1333	0.518

0.8	0	ycaM	5	18.1333	0.482
0.8	0	yadI	5	18.1333	0.564
0.8	0	AG1	5	18.1333	0.752
0.8	0	ptsI	5	18.3833	0.551
0.8	0	clcB	5	18.3833	0.518
0.8	0	ycaM	5	18.3833	0.482
0.8	0	yadI	5	18.3833	0.561
0.8	0	AG1	5	18.3833	0.752
0.8	0	ptsI	5	18.6333	0.549
0.8	0	clcB	5	18.6333	0.52
0.8	0	ycaM	5	18.6333	0.484
0.8	0	yadI	5	18.6333	0.564
0.8	0	AG1	5	18.6333	0.756
0.8	0	ptsI	5	18.8833	0.546
0.8	0	clcB	5	18.8833	0.521
0.8	0	ycaM	5	18.8833	0.487
0.8	0	yadI	5	18.8833	0.562
0.8	0	AG1	5	18.8833	0.759
0.8	0	ptsI	5	19.1333	0.554
0.8	0	clcB	5	19.1333	0.52
0.8	0	ycaM	5	19.1333	0.488
0.8	0	yadI	5	19.1333	0.561
0.8	0	AG1	5	19.1333	0.76
0.8	0	ptsI	5	19.3833	0.556
0.8	0	clcB	5	19.3833	0.526
0.8	0	ycaM	5	19.3833	0.49
0.8	0	yadI	5	19.3833	0.567
0.8	0	AG1	5	19.3833	0.763
0.8	0	ptsI	5	19.6333	0.547
0.8	0	clcB	5	19.6333	0.527
0.8	0	ycaM	5	19.6333	0.491
0.8	0	yadI	5	19.6333	0.568
0.8	0	AG1	5	19.6333	0.765
0.8	0	ptsI	5	19.8833	0.555
0.8	0	clcB	5	19.8833	0.524
0.8	0	ycaM	5	19.8833	0.494
0.8	0	yadI	5	19.8833	0.566
0.8	0	AG1	5	19.8833	0.765
0.8	0	ptsI	5	20.1333	0.555
0.8	0	clcB	5	20.1333	0.524
0.8	0	ycaM	5	20.1333	0.495
0.8	0	yadI	5	20.1333	0.567
0.8	0	AG1	5	20.1333	0.77
0.8	0	ptsI	5	20.3833	0.553
0.8	0	clcB	5	20.3833	0.525
0.8	0	ycaM	5	20.3833	0.495
0.8	0	yadI	5	20.3833	0.566
0.8	0	AG1	5	20.3833	0.771
0.8	0	ptsI	5	20.6333	0.556
0.8	0	clcB	5	20.6333	0.53
0.8	0	ycaM	5	20.6333	0.488
0.8	0	yadI	5	20.6333	0.569
0.8	0	AG1	5	20.6333	0.767

0.8	0	ptsI	5	20.8833	0.554
0.8	0	clcB	5	20.8833	0.532
0.8	0	ycaM	5	20.8833	0.499
0.8	0	yadI	5	20.8833	0.572
0.8	0	AG1	5	20.8833	0.775
0.8	0	ptsI	5	21.1333	0.556
0.8	0	clcB	5	21.1333	0.531
0.8	0	ycaM	5	21.1333	0.5
0.8	0	yadI	5	21.1333	0.571
0.8	0	AG1	5	21.1333	0.775
0.8	0	ptsI	5	21.3833	0.553
0.8	0	clcB	5	21.3833	0.531
0.8	0	ycaM	5	21.3833	0.5
0.8	0	yadI	5	21.3833	0.572
0.8	0	AG1	5	21.3833	0.775
0.8	0	ptsI	5	21.6333	0.552
0.8	0	clcB	5	21.6333	0.532
0.8	0	ycaM	5	21.6333	0.501
0.8	0	yadI	5	21.6333	0.573
0.8	0	AG1	5	21.6333	0.78
0.8	0	ptsI	5	21.8833	0.556
0.8	0	clcB	5	21.8833	0.532
0.8	0	ycaM	5	21.8833	0.503
0.8	0	yadI	5	21.8833	0.573
0.8	0	AG1	5	21.8833	0.78
0.8	0	ptsI	5	22.1333	0.563
0.8	0	clcB	5	22.1333	0.534
0.8	0	ycaM	5	22.1333	0.502
0.8	0	yadI	5	22.1333	0.577
0.8	0	AG1	5	22.1333	0.784
0.8	0	ptsI	5	22.3833	0.555
0.8	0	clcB	5	22.3833	0.535
0.8	0	ycaM	5	22.3833	0.503
0.8	0	yadI	5	22.3833	0.577
0.8	0	AG1	5	22.3833	0.782
0.8	0	ptsI	5	22.6333	0.555
0.8	0	clcB	5	22.6333	0.536
0.8	0	ycaM	5	22.6333	0.504
0.8	0	yadI	5	22.6333	0.576
0.8	0	AG1	5	22.6333	0.781
0.8	0	ptsI	5	22.8833	0.557
0.8	0	clcB	5	22.8833	0.534
0.8	0	ycaM	5	22.8833	0.503
0.8	0	yadI	5	22.8833	0.58
0.8	0	AG1	5	22.8833	0.783
0.8	0	ptsI	5	23.1333	0.558
0.8	0	clcB	5	23.1333	0.534
0.8	0	ycaM	5	23.1333	0.504
0.8	0	yadI	5	23.1333	0.583
0.8	0	AG1	5	23.1333	0.788
0.8	0	ptsI	5	23.3833	0.559
0.8	0	clcB	5	23.3833	0.535
0.8	0	ycaM	5	23.3833	0.504

0.8	0	yadI	5	23.3833	0.585
0.8	0	AG1	5	23.3833	0.784
0.8	0	ptsI	5	23.6333	0.563
0.8	0	clcB	5	23.6333	0.536
0.8	0	ycaM	5	23.6333	0.504
0.8	0	yadI	5	23.6333	0.582
0.8	0	AG1	5	23.6333	0.789
0.8	0	ptsI	5	23.8833	0.576
0.8	0	clcB	5	23.8833	0.533
0.8	0	ycaM	5	23.8833	0.502
0.8	0	yadI	5	23.8833	0.585
0.8	0	AG1	5	23.8833	0.787
0.8	0	ptsI	5	24.1333	0.567
0.8	0	clcB	5	24.1333	0.537
0.8	0	ycaM	5	24.1333	0.506
0.8	0	yadI	5	24.1333	0.587
0.8	0	AG1	5	24.1333	0.787
0.8	0	ptsI	5	24.3833	0.575
0.8	0	clcB	5	24.3833	0.536
0.8	0	ycaM	5	24.3833	0.506
0.8	0	yadI	5	24.3833	0.589
0.8	0	AG1	5	24.3833	0.79
1.6	0	ptsI	1	0	0.219
1.6	0	clcB	1	0	0.229
1.6	0	ycaM	1	0	0.234
1.6	0	yadI	1	0	0.269
1.6	0	AG1	1	0	0.243
1.6	0	ptsI	1	0.25	0.217
1.6	0	clcB	1	0.25	0.221
1.6	0	ycaM	1	0.25	0.227
1.6	0	yadI	1	0.25	0.26
1.6	0	AG1	1	0.25	0.228
1.6	0	ptsI	1	0.5	0.216
1.6	0	clcB	1	0.5	0.22
1.6	0	ycaM	1	0.5	0.222
1.6	0	yadI	1	0.5	0.257
1.6	0	AG1	1	0.5	0.228
1.6	0	ptsI	1	0.75	0.218
1.6	0	clcB	1	0.75	0.219
1.6	0	ycaM	1	0.75	0.22
1.6	0	yadI	1	0.75	0.256
1.6	0	AG1	1	0.75	0.228
1.6	0	ptsI	1	1	0.224
1.6	0	clcB	1	1	0.221
1.6	0	ycaM	1	1	0.22
1.6	0	yadI	1	1	0.26
1.6	0	AG1	1	1	0.229
1.6	0	ptsI	1	1.25	0.226
1.6	0	clcB	1	1.25	0.222
1.6	0	ycaM	1	1.25	0.221
1.6	0	yadI	1	1.25	0.263
1.6	0	AG1	1	1.25	0.231
1.6	0	ptsI	1	1.5	0.23

1.6	0	clcB	1	1.5	0.222
1.6	0	ycaM	1	1.5	0.222
1.6	0	yadI	1	1.5	0.264
1.6	0	AG1	1	1.5	0.232
1.6	0	ptsI	1	1.75	0.236
1.6	0	clcB	1	1.75	0.226
1.6	0	ycaM	1	1.75	0.231
1.6	0	yadI	1	1.75	0.275
1.6	0	AG1	1	1.75	0.236
1.6	0	ptsI	1	2	0.242
1.6	0	clcB	1	2	0.226
1.6	0	ycaM	1	2	0.234
1.6	0	yadI	1	2	0.274
1.6	0	AG1	1	2	0.242
1.6	0	ptsI	1	2.25	0.248
1.6	0	clcB	1	2.25	0.229
1.6	0	ycaM	1	2.25	0.242
1.6	0	yadI	1	2.25	0.289
1.6	0	AG1	1	2.25	0.25
1.6	0	ptsI	1	2.5	0.253
1.6	0	clcB	1	2.5	0.236
1.6	0	ycaM	1	2.5	0.245
1.6	0	yadI	1	2.5	0.294
1.6	0	AG1	1	2.5	0.254
1.6	0	ptsI	1	2.75	0.259
1.6	0	clcB	1	2.75	0.242
1.6	0	ycaM	1	2.75	0.255
1.6	0	yadI	1	2.75	0.309
1.6	0	AG1	1	2.75	0.26
1.6	0	ptsI	1	3	0.268
1.6	0	clcB	1	3	0.248
1.6	0	ycaM	1	3	0.26
1.6	0	yadI	1	3	0.317
1.6	0	AG1	1	3	0.268
1.6	0	ptsI	1	3.25	0.274
1.6	0	clcB	1	3.25	0.253
1.6	0	ycaM	1	3.25	0.266
1.6	0	yadI	1	3.25	0.329
1.6	0	AG1	1	3.25	0.279
1.6	0	ptsI	1	3.5	0.28
1.6	0	clcB	1	3.5	0.261
1.6	0	ycaM	1	3.5	0.273
1.6	0	yadI	1	3.5	0.348
1.6	0	AG1	1	3.5	0.291
1.6	0	ptsI	1	3.75	0.292
1.6	0	clcB	1	3.75	0.264
1.6	0	ycaM	1	3.75	0.281
1.6	0	yadI	1	3.75	0.362
1.6	0	AG1	1	3.75	0.292
1.6	0	ptsI	1	4	0.285
1.6	0	clcB	1	4	0.276
1.6	0	ycaM	1	4	0.287
1.6	0	yadI	1	4	0.391

1.6	0	AG1	1	4	0.314
1.6	0	ptsI	1	4.25	0.304
1.6	0	clcB	1	4.25	0.278
1.6	0	ycaM	1	4.25	0.298
1.6	0	yadI	1	4.25	0.397
1.6	0	AG1	1	4.25	0.314
1.6	0	ptsI	1	4.5	0.308
1.6	0	clcB	1	4.5	0.284
1.6	0	ycaM	1	4.5	0.304
1.6	0	yadI	1	4.5	0.413
1.6	0	AG1	1	4.5	0.322
1.6	0	ptsI	1	4.75	0.327
1.6	0	clcB	1	4.75	0.29
1.6	0	ycaM	1	4.75	0.31
1.6	0	yadI	1	4.75	0.417
1.6	0	AG1	1	4.75	0.331
1.6	0	ptsI	1	5	0.339
1.6	0	clcB	1	5	0.299
1.6	0	ycaM	1	5	0.32
1.6	0	yadI	1	5	0.432
1.6	0	AG1	1	5	0.338
1.6	0	ptsI	1	5.25	0.346
1.6	0	clcB	1	5.25	0.305
1.6	0	ycaM	1	5.25	0.329
1.6	0	yadI	1	5.25	0.445
1.6	0	AG1	1	5.25	0.348
1.6	0	ptsI	1	5.5	0.358
1.6	0	clcB	1	5.5	0.312
1.6	0	ycaM	1	5.5	0.34
1.6	0	yadI	1	5.5	0.457
1.6	0	AG1	1	5.5	0.357
1.6	0	ptsI	1	5.75	0.366
1.6	0	clcB	1	5.75	0.318
1.6	0	ycaM	1	5.75	0.35
1.6	0	yadI	1	5.75	0.465
1.6	0	AG1	1	5.75	0.366
1.6	0	ptsI	1	6	0.386
1.6	0	clcB	1	6	0.333
1.6	0	ycaM	1	6	0.357
1.6	0	yadI	1	6	0.469
1.6	0	AG1	1	6	0.382
1.6	0	ptsI	1	6.25	0.402
1.6	0	clcB	1	6.25	0.342
1.6	0	ycaM	1	6.25	0.365
1.6	0	yadI	1	6.25	0.48
1.6	0	AG1	1	6.25	0.397
1.6	0	ptsI	1	6.5	0.408
1.6	0	clcB	1	6.5	0.353
1.6	0	ycaM	1	6.5	0.375
1.6	0	yadI	1	6.5	0.488
1.6	0	AG1	1	6.5	0.408
1.6	0	ptsI	1	6.75	0.42
1.6	0	clcB	1	6.75	0.356

1.6	0	ycaM	1	6.75	0.386
1.6	0	yadI	1	6.75	0.505
1.6	0	AG1	1	6.75	0.419
1.6	0	ptsI	1	7	0.428
1.6	0	clcB	1	7	0.362
1.6	0	ycaM	1	7	0.399
1.6	0	yadI	1	7	0.518
1.6	0	AG1	1	7	0.427
1.6	0	ptsI	1	7.25	0.434
1.6	0	clcB	1	7.25	0.366
1.6	0	ycaM	1	7.25	0.4
1.6	0	yadI	1	7.25	0.532
1.6	0	AG1	1	7.25	0.434
1.6	0	ptsI	1	7.5	0.443
1.6	0	clcB	1	7.5	0.374
1.6	0	ycaM	1	7.5	0.407
1.6	0	yadI	1	7.5	0.541
1.6	0	AG1	1	7.5	0.442
1.6	0	ptsI	1	7.75	0.445
1.6	0	clcB	1	7.75	0.381
1.6	0	ycaM	1	7.75	0.41
1.6	0	yadI	1	7.75	0.557
1.6	0	AG1	1	7.75	0.452
1.6	0	ptsI	1	8	0.464
1.6	0	clcB	1	8	0.39
1.6	0	ycaM	1	8	0.412
1.6	0	yadI	1	8	0.56
1.6	0	AG1	1	8	0.46
1.6	0	ptsI	1	8.25	0.47
1.6	0	clcB	1	8.25	0.403
1.6	0	ycaM	1	8.25	0.416
1.6	0	yadI	1	8.25	0.568
1.6	0	AG1	1	8.25	0.474
1.6	0	ptsI	1	8.5	0.486
1.6	0	clcB	1	8.5	0.405
1.6	0	ycaM	1	8.5	0.424
1.6	0	yadI	1	8.5	0.578
1.6	0	AG1	1	8.5	0.484
1.6	0	ptsI	1	8.75	0.499
1.6	0	clcB	1	8.75	0.407
1.6	0	ycaM	1	8.75	0.428
1.6	0	yadI	1	8.75	0.588
1.6	0	AG1	1	8.75	0.495
1.6	0	ptsI	1	9	0.516
1.6	0	clcB	1	9	0.413
1.6	0	ycaM	1	9	0.432
1.6	0	yadI	1	9	0.591
1.6	0	AG1	1	9	0.507
1.6	0	ptsI	1	9.25	0.542
1.6	0	clcB	1	9.25	0.428
1.6	0	ycaM	1	9.25	0.444
1.6	0	yadI	1	9.25	0.597
1.6	0	AG1	1	9.25	0.524

1.6	0	ptsI	1	9.5	0.571
1.6	0	clcB	1	9.5	0.449
1.6	0	ycaM	1	9.5	0.455
1.6	0	yadI	1	9.5	0.605
1.6	0	AG1	1	9.5	0.547
1.6	0	ptsI	1	9.75	0.562
1.6	0	clcB	1	9.75	0.442
1.6	0	ycaM	1	9.75	0.455
1.6	0	yadI	1	9.75	0.62
1.6	0	AG1	1	9.75	0.556
1.6	0	ptsI	1	10	0.535
1.6	0	clcB	1	10	0.461
1.6	0	ycaM	1	10	0.467
1.6	0	yadI	1	10	0.617
1.6	0	AG1	1	10	0.561
1.6	0	ptsI	1	10.25	0.552
1.6	0	clcB	1	10.25	0.463
1.6	0	ycaM	1	10.25	0.471
1.6	0	yadI	1	10.25	0.632
1.6	0	AG1	1	10.25	0.572
1.6	0	ptsI	1	10.5	0.554
1.6	0	clcB	1	10.5	0.469
1.6	0	ycaM	1	10.5	0.487
1.6	0	yadI	1	10.5	0.645
1.6	0	AG1	1	10.5	0.57
1.6	0	ptsI	1	10.75	0.555
1.6	0	clcB	1	10.75	0.49
1.6	0	ycaM	1	10.75	0.491
1.6	0	yadI	1	10.75	0.644
1.6	0	AG1	1	10.75	0.592
1.6	0	ptsI	1	11	0.567
1.6	0	clcB	1	11	0.518
1.6	0	ycaM	1	11	0.497
1.6	0	yadI	1	11	0.648
1.6	0	AG1	1	11	0.61
1.6	0	ptsI	1	11.25	0.564
1.6	0	clcB	1	11.25	0.516
1.6	0	ycaM	1	11.25	0.515
1.6	0	yadI	1	11.25	0.673
1.6	0	AG1	1	11.25	0.601
1.6	0	ptsI	1	11.5	0.603
1.6	0	clcB	1	11.5	0.547
1.6	0	ycaM	1	11.5	0.514
1.6	0	yadI	1	11.5	0.69
1.6	0	AG1	1	11.5	0.624
1.6	0	ptsI	1	11.75	0.649
1.6	0	clcB	1	11.75	0.56
1.6	0	ycaM	1	11.75	0.52
1.6	0	yadI	1	11.75	0.688
1.6	0	AG1	1	11.75	0.65
1.6	0	ptsI	1	12	0.64
1.6	0	clcB	1	12	0.548
1.6	0	ycaM	1	12	0.526

1.6	0	yadI	1	12	0.7
1.6	0	AG1	1	12	0.651
1.6	0	ptsI	1	12.25	0.676
1.6	0	clcB	1	12.25	0.564
1.6	0	ycaM	1	12.25	0.533
1.6	0	yadI	1	12.25	0.706
1.6	0	AG1	1	12.25	0.683
1.6	0	ptsI	1	12.5	0.674
1.6	0	clcB	1	12.5	0.561
1.6	0	ycaM	1	12.5	0.532
1.6	0	yadI	1	12.5	0.699
1.6	0	AG1	1	12.5	0.673
1.6	0	ptsI	1	12.75	0.678
1.6	0	clcB	1	12.75	0.559
1.6	0	ycaM	1	12.75	0.528
1.6	0	yadI	1	12.75	0.694
1.6	0	AG1	1	12.75	0.666
1.6	0	ptsI	1	13	0.697
1.6	0	clcB	1	13	0.564
1.6	0	ycaM	1	13	0.538
1.6	0	yadI	1	13	0.701
1.6	0	AG1	1	13	0.66
1.6	0	ptsI	1	13.25	0.725
1.6	0	clcB	1	13.25	0.573
1.6	0	ycaM	1	13.25	0.554
1.6	0	yadI	1	13.25	0.65
1.6	0	AG1	1	13.25	0.677
1.6	0	ptsI	1	13.5	0.732
1.6	0	clcB	1	13.5	0.564
1.6	0	ycaM	1	13.5	0.553
1.6	0	yadI	1	13.5	0.662
1.6	0	AG1	1	13.5	0.698
1.6	0	ptsI	1	13.75	0.752
1.6	0	clcB	1	13.75	0.566
1.6	0	ycaM	1	13.75	0.553
1.6	0	yadI	1	13.75	0.658
1.6	0	AG1	1	13.75	0.687
1.6	0	ptsI	1	14	0.778
1.6	0	clcB	1	14	0.566
1.6	0	ycaM	1	14	0.561
1.6	0	yadI	1	14	0.659
1.6	0	AG1	1	14	0.684
1.6	0	ptsI	1	14.25	0.793
1.6	0	clcB	1	14.25	0.564
1.6	0	ycaM	1	14.25	0.569
1.6	0	yadI	1	14.25	0.659
1.6	0	AG1	1	14.25	0.679
1.6	0	ptsI	1	14.5	0.794
1.6	0	clcB	1	14.5	0.577
1.6	0	ycaM	1	14.5	0.572
1.6	0	yadI	1	14.5	0.666
1.6	0	AG1	1	14.5	0.669
1.6	0	ptsI	1	14.75	0.788

1.6	0	clcB	1	14.75	0.612
1.6	0	ycaM	1	14.75	0.592
1.6	0	yadI	1	14.75	0.669
1.6	0	AG1	1	14.75	0.685
1.6	0	ptsI	1	15	0.78
1.6	0	clcB	1	15	0.614
1.6	0	ycaM	1	15	0.586
1.6	0	yadI	1	15	0.662
1.6	0	AG1	1	15	0.712
1.6	0	ptsI	1	15.25	0.788
1.6	0	clcB	1	15.25	0.651
1.6	0	ycaM	1	15.25	0.616
1.6	0	yadI	1	15.25	0.672
1.6	0	AG1	1	15.25	0.713
1.6	0	ptsI	1	15.5	0.776
1.6	0	clcB	1	15.5	0.648
1.6	0	ycaM	1	15.5	0.624
1.6	0	yadI	1	15.5	0.678
1.6	0	AG1	1	15.5	0.694
1.6	0	ptsI	1	15.75	0.737
1.6	0	clcB	1	15.75	0.658
1.6	0	ycaM	1	15.75	0.638
1.6	0	yadI	1	15.75	0.691
1.6	0	AG1	1	15.75	0.716
1.6	0	ptsI	1	16	0.723
1.6	0	clcB	1	16	0.671
1.6	0	ycaM	1	16	0.654
1.6	0	yadI	1	16	0.689
1.6	0	AG1	1	16	0.714
1.6	0	ptsI	1	16.25	0.757
1.6	0	clcB	1	16.25	0.669
1.6	0	ycaM	1	16.25	0.666
1.6	0	yadI	1	16.25	0.689
1.6	0	AG1	1	16.25	0.721
1.6	0	ptsI	1	16.5	0.778
1.6	0	clcB	1	16.5	0.694
1.6	0	ycaM	1	16.5	0.671
1.6	0	yadI	1	16.5	0.68
1.6	0	AG1	1	16.5	0.736
1.6	0	ptsI	1	17.0667	0.768
1.6	0	clcB	1	17.0667	0.684
1.6	0	ycaM	1	17.0667	0.697
1.6	0	yadI	1	17.0667	0.759
1.6	0	AG1	1	17.0667	0.706
1.6	0	ptsI	1	17.3167	0.771
1.6	0	clcB	1	17.3167	0.669
1.6	0	ycaM	1	17.3167	0.678
1.6	0	yadI	1	17.3167	0.714
1.6	0	AG1	1	17.3167	0.696
1.6	0	ptsI	1	17.5667	0.796
1.6	0	clcB	1	17.5667	0.664
1.6	0	ycaM	1	17.5667	0.678
1.6	0	yadI	1	17.5667	0.711

1.6	0	AG1	1	17.5667	0.696
1.6	0	ptsI	1	17.8167	0.814
1.6	0	clcB	1	17.8167	0.721
1.6	0	ycaM	1	17.8167	0.69
1.6	0	yadI	1	17.8167	0.72
1.6	0	AG1	1	17.8167	0.698
1.6	0	ptsI	1	18.0667	0.827
1.6	0	clcB	1	18.0667	0.702
1.6	0	ycaM	1	18.0667	0.686
1.6	0	yadI	1	18.0667	0.721
1.6	0	AG1	1	18.0667	0.695
1.6	0	ptsI	1	18.3167	0.834
1.6	0	clcB	1	18.3167	0.705
1.6	0	ycaM	1	18.3167	0.69
1.6	0	yadI	1	18.3167	0.727
1.6	0	AG1	1	18.3167	0.732
1.6	0	ptsI	1	18.5667	0.847
1.6	0	clcB	1	18.5667	0.712
1.6	0	ycaM	1	18.5667	0.702
1.6	0	yadI	1	18.5667	0.73
1.6	0	AG1	1	18.5667	0.737
1.6	0	ptsI	1	18.8167	0.856
1.6	0	clcB	1	18.8167	0.712
1.6	0	ycaM	1	18.8167	0.703
1.6	0	yadI	1	18.8167	0.728
1.6	0	AG1	1	18.8167	0.733
1.6	0	ptsI	1	19.0667	0.862
1.6	0	clcB	1	19.0667	0.727
1.6	0	ycaM	1	19.0667	0.703
1.6	0	yadI	1	19.0667	0.742
1.6	0	AG1	1	19.0667	0.761
1.6	0	ptsI	1	19.3167	0.862
1.6	0	clcB	1	19.3167	0.734
1.6	0	ycaM	1	19.3167	0.706
1.6	0	yadI	1	19.3167	0.753
1.6	0	AG1	1	19.3167	0.76
1.6	0	ptsI	1	19.5667	0.874
1.6	0	clcB	1	19.5667	0.742
1.6	0	ycaM	1	19.5667	0.714
1.6	0	yadI	1	19.5667	0.748
1.6	0	AG1	1	19.5667	0.763
1.6	0	ptsI	1	19.8167	0.88
1.6	0	clcB	1	19.8167	0.74
1.6	0	ycaM	1	19.8167	0.713
1.6	0	yadI	1	19.8167	0.752
1.6	0	AG1	1	19.8167	0.773
1.6	0	ptsI	1	20.0667	0.893
1.6	0	clcB	1	20.0667	0.748
1.6	0	ycaM	1	20.0667	0.723
1.6	0	yadI	1	20.0667	0.758
1.6	0	AG1	1	20.0667	0.773
1.6	0	ptsI	1	20.3167	0.902
1.6	0	clcB	1	20.3167	0.754

1.6	0	ycaM	1	20.3167	0.723
1.6	0	yadI	1	20.3167	0.765
1.6	0	AG1	1	20.3167	0.769
1.6	0	ptsI	1	20.5667	0.905
1.6	0	clcB	1	20.5667	0.751
1.6	0	ycaM	1	20.5667	0.714
1.6	0	yadI	1	20.5667	0.782
1.6	0	AG1	1	20.5667	0.763
1.6	0	ptsI	1	20.8167	0.909
1.6	0	clcB	1	20.8167	0.752
1.6	0	ycaM	1	20.8167	0.716
1.6	0	yadI	1	20.8167	0.785
1.6	0	AG1	1	20.8167	0.759
1.6	0	ptsI	1	21.0667	0.928
1.6	0	clcB	1	21.0667	0.757
1.6	0	ycaM	1	21.0667	0.72
1.6	0	yadI	1	21.0667	0.784
1.6	0	AG1	1	21.0667	0.764
1.6	0	ptsI	1	21.3167	0.925
1.6	0	clcB	1	21.3167	0.76
1.6	0	ycaM	1	21.3167	0.726
1.6	0	yadI	1	21.3167	0.784
1.6	0	AG1	1	21.3167	0.761
1.6	0	ptsI	1	21.5667	0.936
1.6	0	clcB	1	21.5667	0.762
1.6	0	ycaM	1	21.5667	0.72
1.6	0	yadI	1	21.5667	0.806
1.6	0	AG1	1	21.5667	0.761
1.6	0	ptsI	1	21.8167	0.942
1.6	0	clcB	1	21.8167	0.76
1.6	0	ycaM	1	21.8167	0.721
1.6	0	yadI	1	21.8167	0.803
1.6	0	AG1	1	21.8167	0.749
1.6	0	ptsI	1	22.0667	0.952
1.6	0	clcB	1	22.0667	0.766
1.6	0	ycaM	1	22.0667	0.72
1.6	0	yadI	1	22.0667	0.806
1.6	0	AG1	1	22.0667	0.758
1.6	0	ptsI	1	22.9333	1.05
1.6	0	clcB	1	22.9333	0.736
1.6	0	ycaM	1	22.9333	0.675
1.6	0	yadI	1	22.9333	0.878
1.6	0	AG1	1	22.9333	0.71
1.6	0	ptsI	1	23.1833	1.054
1.6	0	clcB	1	23.1833	0.721
1.6	0	ycaM	1	23.1833	0.689
1.6	0	yadI	1	23.1833	0.851
1.6	0	AG1	1	23.1833	0.692
1.6	0	ptsI	1	23.4333	1.056
1.6	0	clcB	1	23.4333	0.737
1.6	0	ycaM	1	23.4333	0.714
1.6	0	yadI	1	23.4333	0.853
1.6	0	AG1	1	23.4333	0.701

1.6	0	ptsI	1	23.6833	1.065
1.6	0	clcB	1	23.6833	0.746
1.6	0	ycaM	1	23.6833	0.722
1.6	0	yadI	1	23.6833	0.857
1.6	0	AG1	1	23.6833	0.706
1.6	0	ptsI	1	23.9333	1.073
1.6	0	clcB	1	23.9333	0.746
1.6	0	ycaM	1	23.9333	0.731
1.6	0	yadI	1	23.9333	0.866
1.6	0	AG1	1	23.9333	0.717
1.6	0	ptsI	1	24.1833	1.076
1.6	0	clcB	1	24.1833	0.745
1.6	0	ycaM	1	24.1833	0.729
1.6	0	yadI	1	24.1833	0.879
1.6	0	AG1	1	24.1833	0.716
1.6	0	ptsI	1	24.4333	1.077
1.6	0	clcB	1	24.4333	0.744
1.6	0	ycaM	1	24.4333	0.741
1.6	0	yadI	1	24.4333	0.88
1.6	0	AG1	1	24.4333	0.725
1.6	0	ptsI	1	24.6833	1.082
1.6	0	clcB	1	24.6833	0.739
1.6	0	ycaM	1	24.6833	0.736
1.6	0	yadI	1	24.6833	0.878
1.6	0	AG1	1	24.6833	0.72
1.6	0	ptsI	1	24.9333	1.091
1.6	0	clcB	1	24.9333	0.744
1.6	0	ycaM	1	24.9333	0.733
1.6	0	yadI	1	24.9333	0.897
1.6	0	AG1	1	24.9333	0.717
1.6	0	ptsI	1	25.1833	1.099
1.6	0	clcB	1	25.1833	0.744
1.6	0	ycaM	1	25.1833	0.743
1.6	0	yadI	1	25.1833	0.896
1.6	0	AG1	1	25.1833	0.736
1.6	0	ptsI	1	25.4333	1.104
1.6	0	clcB	1	25.4333	0.744
1.6	0	ycaM	1	25.4333	0.757
1.6	0	yadI	1	25.4333	0.896
1.6	0	AG1	1	25.4333	0.751
1.6	0	ptsI	1	25.6833	1.111
1.6	0	clcB	1	25.6833	0.746
1.6	0	ycaM	1	25.6833	0.751
1.6	0	yadI	1	25.6833	0.904
1.6	0	AG1	1	25.6833	0.746
1.6	0	ptsI	1	25.9333	1.111
1.6	0	clcB	1	25.9333	0.755
1.6	0	ycaM	1	25.9333	0.762
1.6	0	yadI	1	25.9333	0.888
1.6	0	AG1	1	25.9333	0.762
1.6	0	ptsI	1	26.1833	1.115
1.6	0	clcB	1	26.1833	0.751
1.6	0	ycaM	1	26.1833	0.774

1.6	0	yadI	1	26.1833	0.906
1.6	0	AG1	1	26.1833	0.768
1.6	0	ptsI	1	26.4333	1.119
1.6	0	clcB	1	26.4333	0.75
1.6	0	ycaM	1	26.4333	0.774
1.6	0	yadI	1	26.4333	0.91
1.6	0	AG1	1	26.4333	0.762
1.6	0	ptsI	1	26.6833	1.126
1.6	0	clcB	1	26.6833	0.757
1.6	0	ycaM	1	26.6833	0.776
1.6	0	yadI	1	26.6833	0.915
1.6	0	AG1	1	26.6833	0.764
1.6	0	ptsI	1	26.9333	1.13
1.6	0	clcB	1	26.9333	0.759
1.6	0	ycaM	1	26.9333	0.78
1.6	0	yadI	1	26.9333	0.915
1.6	0	AG1	1	26.9333	0.772
1.6	0	ptsI	1	27.1833	1.13
1.6	0	clcB	1	27.1833	0.761
1.6	0	ycaM	1	27.1833	0.783
1.6	0	yadI	1	27.1833	0.916
1.6	0	AG1	1	27.1833	0.775
1.6	0	ptsI	1	27.4333	1.136
1.6	0	clcB	1	27.4333	0.768
1.6	0	ycaM	1	27.4333	0.775
1.6	0	yadI	1	27.4333	0.917
1.6	0	AG1	1	27.4333	0.769
1.6	0	ptsI	1	27.6833	1.141
1.6	0	clcB	1	27.6833	0.763
1.6	0	ycaM	1	27.6833	0.781
1.6	0	yadI	1	27.6833	0.92
1.6	0	AG1	1	27.6833	0.78
1.6	0	ptsI	1	27.9333	1.145
1.6	0	clcB	1	27.9333	0.756
1.6	0	ycaM	1	27.9333	0.782
1.6	0	yadI	1	27.9333	0.922
1.6	0	AG1	1	27.9333	0.778
1.6	0	ptsI	1	28.1833	1.142
1.6	0	clcB	1	28.1833	0.762
1.6	0	ycaM	1	28.1833	0.792
1.6	0	yadI	1	28.1833	0.927
1.6	0	AG1	1	28.1833	0.79
1.6	0	ptsI	1	28.4333	1.148
1.6	0	clcB	1	28.4333	0.762
1.6	0	ycaM	1	28.4333	0.793
1.6	0	yadI	1	28.4333	0.926
1.6	0	AG1	1	28.4333	0.79
1.6	0	ptsI	1	28.6833	1.156
1.6	0	clcB	1	28.6833	0.763
1.6	0	ycaM	1	28.6833	0.795
1.6	0	yadI	1	28.6833	0.932
1.6	0	AG1	1	28.6833	0.792
1.6	0	ptsI	1	28.9333	1.154

1.6	0	clcB	1	28.9333	0.76
1.6	0	ycaM	1	28.9333	0.8
1.6	0	yadI	1	28.9333	0.929
1.6	0	AG1	1	28.9333	0.796
1.6	0	ptsI	1	29.1833	1.153
1.6	0	clcB	1	29.1833	0.761
1.6	0	ycaM	1	29.1833	0.793
1.6	0	yadI	1	29.1833	0.936
1.6	0	AG1	1	29.1833	0.792
1.6	0	ptsI	1	29.4333	1.154
1.6	0	clcB	1	29.4333	0.762
1.6	0	ycaM	1	29.4333	0.803
1.6	0	yadI	1	29.4333	0.943
1.6	0	AG1	1	29.4333	0.797
1.6	0	ptsI	1	29.6833	1.156
1.6	0	clcB	1	29.6833	0.762
1.6	0	ycaM	1	29.6833	0.805
1.6	0	yadI	1	29.6833	0.949
1.6	0	AG1	1	29.6833	0.796
1.6	0	ptsI	1	29.9333	1.162
1.6	0	clcB	1	29.9333	0.762
1.6	0	ycaM	1	29.9333	0.81
1.6	0	yadI	1	29.9333	0.952
1.6	0	AG1	1	29.9333	0.798
1.6	0	ptsI	1	30.1833	1.173
1.6	0	clcB	1	30.1833	0.759
1.6	0	ycaM	1	30.1833	0.808
1.6	0	yadI	1	30.1833	0.957
1.6	0	AG1	1	30.1833	0.802
1.6	0	ptsI	1	30.4333	1.173
1.6	0	clcB	1	30.4333	0.764
1.6	0	ycaM	1	30.4333	0.816
1.6	0	yadI	1	30.4333	0.96
1.6	0	AG1	1	30.4333	0.805
1.6	0	ptsI	1	30.6833	1.185
1.6	0	clcB	1	30.6833	0.764
1.6	0	ycaM	1	30.6833	0.818
1.6	0	yadI	1	30.6833	0.974
1.6	0	AG1	1	30.6833	0.802
1.6	0	ptsI	1	30.9333	1.19
1.6	0	clcB	1	30.9333	0.766
1.6	0	ycaM	1	30.9333	0.816
1.6	0	yadI	1	30.9333	0.977
1.6	0	AG1	1	30.9333	0.809
1.6	0	ptsI	1	31.1833	1.198
1.6	0	clcB	1	31.1833	0.77
1.6	0	ycaM	1	31.1833	0.821
1.6	0	yadI	1	31.1833	0.984
1.6	0	AG1	1	31.1833	0.806
1.6	0	ptsI	1	31.4333	1.2
1.6	0	clcB	1	31.4333	0.774
1.6	0	ycaM	1	31.4333	0.824
1.6	0	yadI	1	31.4333	0.98

1.6	0	AG1	1	31.4333	0.812
1.6	0	ptsI	1	31.6833	1.209
1.6	0	clcB	1	31.6833	0.776
1.6	0	ycaM	1	31.6833	0.828
1.6	0	yadI	1	31.6833	0.986
1.6	0	AG1	1	31.6833	0.815
1.6	0	ptsI	1	31.9333	1.214
1.6	0	clcB	1	31.9333	0.776
1.6	0	ycaM	1	31.9333	0.828
1.6	0	yadI	1	31.9333	0.987
1.6	0	AG1	1	31.9333	0.812
1.6	0	ptsI	1	32.1833	1.22
1.6	0	clcB	1	32.1833	0.778
1.6	0	ycaM	1	32.1833	0.836
1.6	0	yadI	1	32.1833	0.994
1.6	0	AG1	1	32.1833	0.82
1.6	0	ptsI	1	32.4333	1.236
1.6	0	clcB	1	32.4333	0.786
1.6	0	ycaM	1	32.4333	0.836
1.6	0	yadI	1	32.4333	0.986
1.6	0	AG1	1	32.4333	0.821
1.6	0	ptsI	1	32.6833	1.235
1.6	0	clcB	1	32.6833	0.788
1.6	0	ycaM	1	32.6833	0.842
1.6	0	yadI	1	32.6833	1.012
1.6	0	AG1	1	32.6833	0.824
1.6	0	ptsI	1	32.9333	1.246
1.6	0	clcB	1	32.9333	0.791
1.6	0	ycaM	1	32.9333	0.847
1.6	0	yadI	1	32.9333	1.022
1.6	0	AG1	1	32.9333	0.823
1.6	0	ptsI	1	33.1833	1.263
1.6	0	clcB	1	33.1833	0.784
1.6	0	ycaM	1	33.1833	0.85
1.6	0	yadI	1	33.1833	1.019
1.6	0	AG1	1	33.1833	0.821
1.6	0	ptsI	1	33.4333	1.268
1.6	0	clcB	1	33.4333	0.794
1.6	0	ycaM	1	33.4333	0.85
1.6	0	yadI	1	33.4333	1.03
1.6	0	AG1	1	33.4333	0.828
1.6	0	ptsI	1	33.6833	1.28
1.6	0	clcB	1	33.6833	0.79
1.6	0	ycaM	1	33.6833	0.857
1.6	0	yadI	1	33.6833	1.03
1.6	0	AG1	1	33.6833	0.823
1.6	0	ptsI	1	33.9333	1.285
1.6	0	clcB	1	33.9333	0.806
1.6	0	ycaM	1	33.9333	0.859
1.6	0	yadI	1	33.9333	1.042
1.6	0	AG1	1	33.9333	0.827
1.6	0	ptsI	1	34.1833	1.292
1.6	0	clcB	1	34.1833	0.796

1.6	0	ycaM	1	34.1833	0.861
1.6	0	yadI	1	34.1833	1.069
1.6	0	AG1	1	34.1833	0.83
1.6	0	ptsI	1	34.4333	1.306
1.6	0	clcB	1	34.4333	0.813
1.6	0	ycaM	1	34.4333	0.864
1.6	0	yadI	1	34.4333	1.061
1.6	0	AG1	1	34.4333	0.831
1.6	0	ptsI	1	34.6833	1.312
1.6	0	clcB	1	34.6833	0.817
1.6	0	ycaM	1	34.6833	0.864
1.6	0	yadI	1	34.6833	1.068
1.6	0	AG1	1	34.6833	0.834
1.6	0	ptsI	1	34.9333	1.328
1.6	0	clcB	1	34.9333	0.822
1.6	0	ycaM	1	34.9333	0.862
1.6	0	yadI	1	34.9333	1.075
1.6	0	AG1	1	34.9333	0.838
1.6	0	ptsI	1	35.1833	1.334
1.6	0	clcB	1	35.1833	0.832
1.6	0	ycaM	1	35.1833	0.859
1.6	0	yadI	1	35.1833	1.082
1.6	0	AG1	1	35.1833	0.838
1.6	0	ptsI	1	35.4333	1.342
1.6	0	clcB	1	35.4333	0.836
1.6	0	ycaM	1	35.4333	0.86
1.6	0	yadI	1	35.4333	1.097
1.6	0	AG1	1	35.4333	0.84
1.6	0	ptsI	1	35.6833	1.355
1.6	0	clcB	1	35.6833	0.834
1.6	0	ycaM	1	35.6833	0.858
1.6	0	yadI	1	35.6833	1.102
1.6	0	AG1	1	35.6833	0.841
1.6	0	ptsI	1	35.9333	1.37
1.6	0	clcB	1	35.9333	0.83
1.6	0	ycaM	1	35.9333	0.853
1.6	0	yadI	1	35.9333	1.109
1.6	0	AG1	1	35.9333	0.843
1.6	0	ptsI	1	36.1833	1.357
1.6	0	clcB	1	36.1833	0.835
1.6	0	ycaM	1	36.1833	0.855
1.6	0	yadI	1	36.1833	1.118
1.6	0	AG1	1	36.1833	0.846
1.6	0	ptsI	1	36.4333	1.382
1.6	0	clcB	1	36.4333	0.838
1.6	0	ycaM	1	36.4333	0.854
1.6	0	yadI	1	36.4333	1.132
1.6	0	AG1	1	36.4333	0.851
1.6	0	ptsI	1	36.6833	1.39
1.6	0	clcB	1	36.6833	0.839
1.6	0	ycaM	1	36.6833	0.853
1.6	0	yadI	1	36.6833	1.136
1.6	0	AG1	1	36.6833	0.852

1.6	0	ptsI	2	0	0.22
1.6	0	clcB	2	0	0.204
1.6	0	ycaM	2	0	0.227
1.6	0	yadI	2	0	0.216
1.6	0	AG1	2	0	0.218
1.6	0	ptsI	2	0.25	0.217
1.6	0	clcB	2	0.25	0.2
1.6	0	ycaM	2	0.25	0.218
1.6	0	yadI	2	0.25	0.212
1.6	0	AG1	2	0.25	0.211
1.6	0	ptsI	2	0.5	0.222
1.6	0	clcB	2	0.5	0.205
1.6	0	ycaM	2	0.5	0.218
1.6	0	yadI	2	0.5	0.213
1.6	0	AG1	2	0.5	0.213
1.6	0	ptsI	2	0.75	0.224
1.6	0	clcB	2	0.75	0.201
1.6	0	ycaM	2	0.75	0.221
1.6	0	yadI	2	0.75	0.216
1.6	0	AG1	2	0.75	0.216
1.6	0	ptsI	2	1	0.228
1.6	0	clcB	2	1	0.205
1.6	0	ycaM	2	1	0.226
1.6	0	yadI	2	1	0.223
1.6	0	AG1	2	1	0.221
1.6	0	ptsI	2	1.25	0.229
1.6	0	clcB	2	1.25	0.204
1.6	0	ycaM	2	1.25	0.228
1.6	0	yadI	2	1.25	0.232
1.6	0	AG1	2	1.25	0.224
1.6	0	ptsI	2	1.5	0.231
1.6	0	clcB	2	1.5	0.209
1.6	0	ycaM	2	1.5	0.231
1.6	0	yadI	2	1.5	0.238
1.6	0	AG1	2	1.5	0.228
1.6	0	ptsI	2	1.75	0.238
1.6	0	clcB	2	1.75	0.21
1.6	0	ycaM	2	1.75	0.236
1.6	0	yadI	2	1.75	0.243
1.6	0	AG1	2	1.75	0.234
1.6	0	ptsI	2	2	0.244
1.6	0	clcB	2	2	0.212
1.6	0	ycaM	2	2	0.243
1.6	0	yadI	2	2	0.251
1.6	0	AG1	2	2	0.245
1.6	0	ptsI	2	2.25	0.252
1.6	0	clcB	2	2.25	0.221
1.6	0	ycaM	2	2.25	0.249
1.6	0	yadI	2	2.25	0.262
1.6	0	AG1	2	2.25	0.255
1.6	0	ptsI	2	2.5	0.263
1.6	0	clcB	2	2.5	0.222
1.6	0	ycaM	2	2.5	0.258

1.6	0	yadI	2	2.5	0.277
1.6	0	AG1	2	2.5	0.268
1.6	0	ptsI	2	2.75	0.266
1.6	0	clcB	2	2.75	0.23
1.6	0	ycaM	2	2.75	0.274
1.6	0	yadI	2	2.75	0.306
1.6	0	AG1	2	2.75	0.279
1.6	0	ptsI	2	3	0.274
1.6	0	clcB	2	3	0.237
1.6	0	ycaM	2	3	0.267
1.6	0	yadI	2	3	0.302
1.6	0	AG1	2	3	0.29
1.6	0	ptsI	2	3.25	0.286
1.6	0	clcB	2	3.25	0.243
1.6	0	ycaM	2	3.25	0.279
1.6	0	yadI	2	3.25	0.326
1.6	0	AG1	2	3.25	0.302
1.6	0	ptsI	2	3.5	0.294
1.6	0	clcB	2	3.5	0.251
1.6	0	ycaM	2	3.5	0.288
1.6	0	yadI	2	3.5	0.344
1.6	0	AG1	2	3.5	0.314
1.6	0	ptsI	2	3.75	0.306
1.6	0	clcB	2	3.75	0.257
1.6	0	ycaM	2	3.75	0.298
1.6	0	yadI	2	3.75	0.361
1.6	0	AG1	2	3.75	0.327
1.6	0	ptsI	2	4	0.316
1.6	0	clcB	2	4	0.261
1.6	0	ycaM	2	4	0.306
1.6	0	yadI	2	4	0.383
1.6	0	AG1	2	4	0.335
1.6	0	ptsI	2	4.38333	0.318
1.6	0	clcB	2	4.38333	0.267
1.6	0	ycaM	2	4.38333	0.342
1.6	0	yadI	2	4.38333	0.396
1.6	0	AG1	2	4.38333	0.298
1.6	0	ptsI	2	4.63333	0.316
1.6	0	clcB	2	4.63333	0.264
1.6	0	ycaM	2	4.63333	0.334
1.6	0	yadI	2	4.63333	0.394
1.6	0	AG1	2	4.63333	0.291
1.6	0	ptsI	2	4.88333	0.323
1.6	0	clcB	2	4.88333	0.266
1.6	0	ycaM	2	4.88333	0.343
1.6	0	yadI	2	4.88333	0.407
1.6	0	AG1	2	4.88333	0.297
1.6	0	ptsI	2	5.13333	0.334
1.6	0	clcB	2	5.13333	0.274
1.6	0	ycaM	2	5.13333	0.354
1.6	0	yadI	2	5.13333	0.418
1.6	0	AG1	2	5.13333	0.304
1.6	0	ptsI	2	5.38333	0.341

1.6	0	clcB	2	5.38333	0.281
1.6	0	ycaM	2	5.38333	0.36
1.6	0	yadI	2	5.38333	0.428
1.6	0	AG1	2	5.38333	0.309
1.6	0	ptsI	2	5.63333	0.348
1.6	0	clcB	2	5.63333	0.29
1.6	0	ycaM	2	5.63333	0.372
1.6	0	yadI	2	5.63333	0.438
1.6	0	AG1	2	5.63333	0.318
1.6	0	ptsI	2	5.88333	0.358
1.6	0	clcB	2	5.88333	0.297
1.6	0	ycaM	2	5.88333	0.381
1.6	0	yadI	2	5.88333	0.447
1.6	0	AG1	2	5.88333	0.329
1.6	0	ptsI	2	6.13333	0.364
1.6	0	clcB	2	6.13333	0.305
1.6	0	ycaM	2	6.13333	0.389
1.6	0	yadI	2	6.13333	0.458
1.6	0	AG1	2	6.13333	0.339
1.6	0	ptsI	2	6.38333	0.371
1.6	0	clcB	2	6.38333	0.31
1.6	0	ycaM	2	6.38333	0.401
1.6	0	yadI	2	6.38333	0.468
1.6	0	AG1	2	6.38333	0.349
1.6	0	ptsI	2	6.63333	0.378
1.6	0	clcB	2	6.63333	0.317
1.6	0	ycaM	2	6.63333	0.412
1.6	0	yadI	2	6.63333	0.478
1.6	0	AG1	2	6.63333	0.356
1.6	0	ptsI	2	6.88333	0.388
1.6	0	clcB	2	6.88333	0.324
1.6	0	ycaM	2	6.88333	0.418
1.6	0	yadI	2	6.88333	0.487
1.6	0	AG1	2	6.88333	0.364
1.6	0	ptsI	2	7.13333	0.397
1.6	0	clcB	2	7.13333	0.332
1.6	0	ycaM	2	7.13333	0.426
1.6	0	yadI	2	7.13333	0.498
1.6	0	AG1	2	7.13333	0.372
1.6	0	ptsI	2	7.38333	0.404
1.6	0	clcB	2	7.38333	0.34
1.6	0	ycaM	2	7.38333	0.435
1.6	0	yadI	2	7.38333	0.507
1.6	0	AG1	2	7.38333	0.382
1.6	0	ptsI	2	7.63333	0.412
1.6	0	clcB	2	7.63333	0.347
1.6	0	ycaM	2	7.63333	0.443
1.6	0	yadI	2	7.63333	0.519
1.6	0	AG1	2	7.63333	0.388
1.6	0	ptsI	2	7.88333	0.423
1.6	0	clcB	2	7.88333	0.354
1.6	0	ycaM	2	7.88333	0.451
1.6	0	yadI	2	7.88333	0.532

1.6	0	AG1	2	7.88333	0.395
1.6	0	ptsI	2	8.13333	0.429
1.6	0	clcB	2	8.13333	0.361
1.6	0	ycaM	2	8.13333	0.455
1.6	0	yadI	2	8.13333	0.541
1.6	0	AG1	2	8.13333	0.403
1.6	0	ptsI	2	8.38333	0.437
1.6	0	clcB	2	8.38333	0.372
1.6	0	ycaM	2	8.38333	0.463
1.6	0	yadI	2	8.38333	0.552
1.6	0	AG1	2	8.38333	0.409
1.6	0	ptsI	2	8.91667	0.358
1.6	0	clcB	2	8.91667	0.315
1.6	0	ycaM	2	8.91667	0.344
1.6	0	yadI	2	8.91667	0.617
1.6	0	AG1	2	8.91667	0.456
1.6	0	ptsI	2	9.16667	0.36
1.6	0	clcB	2	9.16667	0.322
1.6	0	ycaM	2	9.16667	0.36
1.6	0	yadI	2	9.16667	0.629
1.6	0	AG1	2	9.16667	0.46
1.6	0	ptsI	2	9.41667	0.368
1.6	0	clcB	2	9.41667	0.33
1.6	0	ycaM	2	9.41667	0.352
1.6	0	yadI	2	9.41667	0.64
1.6	0	AG1	2	9.41667	0.479
1.6	0	ptsI	2	9.66667	0.376
1.6	0	clcB	2	9.66667	0.337
1.6	0	ycaM	2	9.66667	0.352
1.6	0	yadI	2	9.66667	0.657
1.6	0	AG1	2	9.66667	0.494
1.6	0	ptsI	2	9.91667	0.38
1.6	0	clcB	2	9.91667	0.343
1.6	0	ycaM	2	9.91667	0.355
1.6	0	yadI	2	9.91667	0.666
1.6	0	AG1	2	9.91667	0.503
1.6	0	ptsI	2	10.1667	0.388
1.6	0	clcB	2	10.1667	0.351
1.6	0	ycaM	2	10.1667	0.36
1.6	0	yadI	2	10.1667	0.673
1.6	0	AG1	2	10.1667	0.506
1.6	0	ptsI	2	10.4167	0.396
1.6	0	clcB	2	10.4167	0.358
1.6	0	ycaM	2	10.4167	0.368
1.6	0	yadI	2	10.4167	0.687
1.6	0	AG1	2	10.4167	0.518
1.6	0	ptsI	2	10.6667	0.407
1.6	0	clcB	2	10.6667	0.365
1.6	0	ycaM	2	10.6667	0.372
1.6	0	yadI	2	10.6667	0.695
1.6	0	AG1	2	10.6667	0.523
1.6	0	ptsI	2	10.9167	0.41
1.6	0	clcB	2	10.9167	0.367

1.6	0	ycaM	2	10.9167	0.376
1.6	0	yadI	2	10.9167	0.694
1.6	0	AG1	2	10.9167	0.53
1.6	0	ptsI	2	11.1667	0.421
1.6	0	clcB	2	11.1667	0.372
1.6	0	ycaM	2	11.1667	0.378
1.6	0	yadI	2	11.1667	0.703
1.6	0	AG1	2	11.1667	0.538
1.6	0	ptsI	2	11.4167	0.439
1.6	0	clcB	2	11.4167	0.37
1.6	0	ycaM	2	11.4167	0.382
1.6	0	yadI	2	11.4167	0.711
1.6	0	AG1	2	11.4167	0.551
1.6	0	ptsI	2	11.6667	0.434
1.6	0	clcB	2	11.6667	0.37
1.6	0	ycaM	2	11.6667	0.382
1.6	0	yadI	2	11.6667	0.722
1.6	0	AG1	2	11.6667	0.54
1.6	0	ptsI	2	11.9167	0.444
1.6	0	clcB	2	11.9167	0.375
1.6	0	ycaM	2	11.9167	0.389
1.6	0	yadI	2	11.9167	0.73
1.6	0	AG1	2	11.9167	0.555
1.6	0	ptsI	2	12.1667	0.439
1.6	0	clcB	2	12.1667	0.378
1.6	0	ycaM	2	12.1667	0.39
1.6	0	yadI	2	12.1667	0.736
1.6	0	AG1	2	12.1667	0.546
1.6	0	ptsI	2	12.4167	0.446
1.6	0	clcB	2	12.4167	0.377
1.6	0	ycaM	2	12.4167	0.393
1.6	0	yadI	2	12.4167	0.742
1.6	0	AG1	2	12.4167	0.544
1.6	0	ptsI	2	12.6667	0.457
1.6	0	clcB	2	12.6667	0.381
1.6	0	ycaM	2	12.6667	0.394
1.6	0	yadI	2	12.6667	0.746
1.6	0	AG1	2	12.6667	0.549
1.6	0	ptsI	2	12.9167	0.455
1.6	0	clcB	2	12.9167	0.38
1.6	0	ycaM	2	12.9167	0.399
1.6	0	yadI	2	12.9167	0.756
1.6	0	AG1	2	12.9167	0.549
1.6	0	ptsI	2	13.1667	0.453
1.6	0	clcB	2	13.1667	0.383
1.6	0	ycaM	2	13.1667	0.399
1.6	0	yadI	2	13.1667	0.761
1.6	0	AG1	2	13.1667	0.543
1.6	0	ptsI	2	13.4167	0.458
1.6	0	clcB	2	13.4167	0.387
1.6	0	ycaM	2	13.4167	0.404
1.6	0	yadI	2	13.4167	0.765
1.6	0	AG1	2	13.4167	0.542

1.6	0	ptsI	2	13.6667	0.468
1.6	0	clcB	2	13.6667	0.39
1.6	0	ycaM	2	13.6667	0.402
1.6	0	yadI	2	13.6667	0.773
1.6	0	AG1	2	13.6667	0.546
1.6	0	ptsI	2	13.9167	0.469
1.6	0	clcB	2	13.9167	0.399
1.6	0	ycaM	2	13.9167	0.404
1.6	0	yadI	2	13.9167	0.775
1.6	0	AG1	2	13.9167	0.548
1.6	0	ptsI	2	14.1667	0.48
1.6	0	clcB	2	14.1667	0.4
1.6	0	ycaM	2	14.1667	0.408
1.6	0	yadI	2	14.1667	0.781
1.6	0	AG1	2	14.1667	0.55
1.6	0	ptsI	2	14.4167	0.481
1.6	0	clcB	2	14.4167	0.401
1.6	0	ycaM	2	14.4167	0.401
1.6	0	yadI	2	14.4167	0.786
1.6	0	AG1	2	14.4167	0.555
1.6	0	ptsI	2	14.6667	0.483
1.6	0	clcB	2	14.6667	0.408
1.6	0	ycaM	2	14.6667	0.413
1.6	0	yadI	2	14.6667	0.789
1.6	0	AG1	2	14.6667	0.556
1.6	0	ptsI	2	14.9167	0.496
1.6	0	clcB	2	14.9167	0.409
1.6	0	ycaM	2	14.9167	0.416
1.6	0	yadI	2	14.9167	0.794
1.6	0	AG1	2	14.9167	0.561
1.6	0	ptsI	2	15.1667	0.506
1.6	0	clcB	2	15.1667	0.416
1.6	0	ycaM	2	15.1667	0.419
1.6	0	yadI	2	15.1667	0.806
1.6	0	AG1	2	15.1667	0.578
1.6	0	ptsI	2	15.4167	0.517
1.6	0	clcB	2	15.4167	0.418
1.6	0	ycaM	2	15.4167	0.411
1.6	0	yadI	2	15.4167	0.806
1.6	0	AG1	2	15.4167	0.564
1.6	0	ptsI	2	15.6667	0.53
1.6	0	clcB	2	15.6667	0.421
1.6	0	ycaM	2	15.6667	0.421
1.6	0	yadI	2	15.6667	0.804
1.6	0	AG1	2	15.6667	0.572
1.6	0	ptsI	2	15.9167	0.537
1.6	0	clcB	2	15.9167	0.427
1.6	0	ycaM	2	15.9167	0.435
1.6	0	yadI	2	15.9167	0.808
1.6	0	AG1	2	15.9167	0.568
1.6	0	ptsI	2	16.1667	0.533
1.6	0	clcB	2	16.1667	0.432
1.6	0	ycaM	2	16.1667	0.428

1.6	0	yadI	2	16.1667	0.81
1.6	0	AG1	2	16.1667	0.568
1.6	0	ptsI	2	16.4167	0.541
1.6	0	clcB	2	16.4167	0.438
1.6	0	ycaM	2	16.4167	0.431
1.6	0	yadI	2	16.4167	0.815
1.6	0	AG1	2	16.4167	0.566
1.6	0	ptsI	2	16.6667	0.542
1.6	0	clcB	2	16.6667	0.449
1.6	0	ycaM	2	16.6667	0.436
1.6	0	yadI	2	16.6667	0.818
1.6	0	AG1	2	16.6667	0.553
1.6	0	ptsI	2	16.9167	0.566
1.6	0	clcB	2	16.9167	0.451
1.6	0	ycaM	2	16.9167	0.429
1.6	0	yadI	2	16.9167	0.821
1.6	0	AG1	2	16.9167	0.559
1.6	0	ptsI	2	17.1667	0.573
1.6	0	clcB	2	17.1667	0.467
1.6	0	ycaM	2	17.1667	0.436
1.6	0	yadI	2	17.1667	0.824
1.6	0	AG1	2	17.1667	0.591
1.6	0	ptsI	2	17.4167	0.56
1.6	0	clcB	2	17.4167	0.466
1.6	0	ycaM	2	17.4167	0.433
1.6	0	yadI	2	17.4167	0.823
1.6	0	AG1	2	17.4167	0.586
1.6	0	ptsI	2	17.6667	0.563
1.6	0	clcB	2	17.6667	0.474
1.6	0	ycaM	2	17.6667	0.443
1.6	0	yadI	2	17.6667	0.822
1.6	0	AG1	2	17.6667	0.572
1.6	0	ptsI	2	17.9167	0.554
1.6	0	clcB	2	17.9167	0.473
1.6	0	ycaM	2	17.9167	0.439
1.6	0	yadI	2	17.9167	0.824
1.6	0	AG1	2	17.9167	0.579
1.6	0	ptsI	2	18.1667	0.571
1.6	0	clcB	2	18.1667	0.47
1.6	0	ycaM	2	18.1667	0.443
1.6	0	yadI	2	18.1667	0.822
1.6	0	AG1	2	18.1667	0.582
1.6	0	ptsI	2	18.4167	0.567
1.6	0	clcB	2	18.4167	0.471
1.6	0	ycaM	2	18.4167	0.442
1.6	0	yadI	2	18.4167	0.824
1.6	0	AG1	2	18.4167	0.57
1.6	0	ptsI	2	18.6667	0.568
1.6	0	clcB	2	18.6667	0.484
1.6	0	ycaM	2	18.6667	0.453
1.6	0	yadI	2	18.6667	0.825
1.6	0	AG1	2	18.6667	0.566
1.6	0	ptsI	2	18.9167	0.555

1.6	0	clcB	2	18.9167	0.472
1.6	0	ycaM	2	18.9167	0.449
1.6	0	yadI	2	18.9167	0.811
1.6	0	AG1	2	18.9167	0.561
1.6	0	ptsI	2	19.1667	0.567
1.6	0	clcB	2	19.1667	0.477
1.6	0	ycaM	2	19.1667	0.449
1.6	0	yadI	2	19.1667	0.822
1.6	0	AG1	2	19.1667	0.561
1.6	0	ptsI	2	19.4167	0.572
1.6	0	clcB	2	19.4167	0.468
1.6	0	ycaM	2	19.4167	0.458
1.6	0	yadI	2	19.4167	0.825
1.6	0	AG1	2	19.4167	0.566
1.6	0	ptsI	2	19.6667	0.57
1.6	0	clcB	2	19.6667	0.476
1.6	0	ycaM	2	19.6667	0.45
1.6	0	yadI	2	19.6667	0.83
1.6	0	AG1	2	19.6667	0.562
1.6	0	ptsI	2	19.9167	0.572
1.6	0	clcB	2	19.9167	0.455
1.6	0	ycaM	2	19.9167	0.461
1.6	0	yadI	2	19.9167	0.827
1.6	0	AG1	2	19.9167	0.577
1.6	0	ptsI	2	20.1667	0.581
1.6	0	clcB	2	20.1667	0.468
1.6	0	ycaM	2	20.1667	0.461
1.6	0	yadI	2	20.1667	0.827
1.6	0	AG1	2	20.1667	0.575
1.6	0	ptsI	2	20.4167	0.583
1.6	0	clcB	2	20.4167	0.466
1.6	0	ycaM	2	20.4167	0.456
1.6	0	yadI	2	20.4167	0.83
1.6	0	AG1	2	20.4167	0.577
1.6	0	ptsI	2	20.6667	0.584
1.6	0	clcB	2	20.6667	0.468
1.6	0	ycaM	2	20.6667	0.462
1.6	0	yadI	2	20.6667	0.832
1.6	0	AG1	2	20.6667	0.571
1.6	0	ptsI	2	20.9167	0.595
1.6	0	clcB	2	20.9167	0.482
1.6	0	ycaM	2	20.9167	0.458
1.6	0	yadI	2	20.9167	0.833
1.6	0	AG1	2	20.9167	0.571
1.6	0	ptsI	2	21.1667	0.597
1.6	0	clcB	2	21.1667	0.464
1.6	0	ycaM	2	21.1667	0.453
1.6	0	yadI	2	21.1667	0.829
1.6	0	AG1	2	21.1667	0.57
1.6	0	ptsI	2	21.4167	0.602
1.6	0	clcB	2	21.4167	0.463
1.6	0	ycaM	2	21.4167	0.454
1.6	0	yadI	2	21.4167	0.826

1.6	0	AG1	2	21.4167	0.574
1.6	0	ptsI	2	21.6667	0.608
1.6	0	clcB	2	21.6667	0.465
1.6	0	ycaM	2	21.6667	0.453
1.6	0	yadI	2	21.6667	0.828
1.6	0	AG1	2	21.6667	0.565
1.6	0	ptsI	2	21.9167	0.619
1.6	0	clcB	2	21.9167	0.466
1.6	0	ycaM	2	21.9167	0.453
1.6	0	yadI	2	21.9167	0.827
1.6	0	AG1	2	21.9167	0.572
1.6	0	ptsI	2	22.1667	0.618
1.6	0	clcB	2	22.1667	0.46
1.6	0	ycaM	2	22.1667	0.453
1.6	0	yadI	2	22.1667	0.829
1.6	0	AG1	2	22.1667	0.573
1.6	0	ptsI	2	22.4167	0.622
1.6	0	clcB	2	22.4167	0.443
1.6	0	ycaM	2	22.4167	0.452
1.6	0	yadI	2	22.4167	0.829
1.6	0	AG1	2	22.4167	0.564
1.6	0	ptsI	2	22.6667	0.626
1.6	0	clcB	2	22.6667	0.452
1.6	0	ycaM	2	22.6667	0.45
1.6	0	yadI	2	22.6667	0.829
1.6	0	AG1	2	22.6667	0.565
1.6	0	ptsI	2	22.9167	0.634
1.6	0	clcB	2	22.9167	0.439
1.6	0	ycaM	2	22.9167	0.452
1.6	0	yadI	2	22.9167	0.833
1.6	0	AG1	2	22.9167	0.565
1.6	0	ptsI	2	23.1667	0.639
1.6	0	clcB	2	23.1667	0.436
1.6	0	ycaM	2	23.1667	0.452
1.6	0	yadI	2	23.1667	0.831
1.6	0	AG1	2	23.1667	0.567
1.6	0	ptsI	2	23.4167	0.644
1.6	0	clcB	2	23.4167	0.439
1.6	0	ycaM	2	23.4167	0.451
1.6	0	yadI	2	23.4167	0.832
1.6	0	AG1	2	23.4167	0.566
1.6	0	ptsI	2	23.6667	0.649
1.6	0	clcB	2	23.6667	0.444
1.6	0	ycaM	2	23.6667	0.454
1.6	0	yadI	2	23.6667	0.832
1.6	0	AG1	2	23.6667	0.566
1.6	0	ptsI	2	23.9167	0.654
1.6	0	clcB	2	23.9167	0.443
1.6	0	ycaM	2	23.9167	0.451
1.6	0	yadI	2	23.9167	0.833
1.6	0	AG1	2	23.9167	0.563
1.6	0	ptsI	2	24.1667	0.663
1.6	0	clcB	2	24.1667	0.448

1.6	0	ycaM	2	24.1667	0.452
1.6	0	yadI	2	24.1667	0.83
1.6	0	AG1	2	24.1667	0.561
1.6	0	ptsI	2	24.4167	0.675
1.6	0	clcB	2	24.4167	0.451
1.6	0	ycaM	2	24.4167	0.455
1.6	0	yadI	2	24.4167	0.837
1.6	0	AG1	2	24.4167	0.563
1.6	0	ptsI	2	24.6667	0.68
1.6	0	clcB	2	24.6667	0.455
1.6	0	ycaM	2	24.6667	0.453
1.6	0	yadI	2	24.6667	0.833
1.6	0	AG1	2	24.6667	0.565
1.6	0	ptsI	2	24.9167	0.69
1.6	0	clcB	2	24.9167	0.453
1.6	0	ycaM	2	24.9167	0.457
1.6	0	yadI	2	24.9167	0.837
1.6	0	AG1	2	24.9167	0.568
1.6	0	ptsI	2	25.1667	0.697
1.6	0	clcB	2	25.1667	0.454
1.6	0	ycaM	2	25.1667	0.456
1.6	0	yadI	2	25.1667	0.834
1.6	0	AG1	2	25.1667	0.569
1.6	0	ptsI	2	25.4167	0.701
1.6	0	clcB	2	25.4167	0.459
1.6	0	ycaM	2	25.4167	0.458
1.6	0	yadI	2	25.4167	0.833
1.6	0	AG1	2	25.4167	0.574
1.6	0	ptsI	2	25.6667	0.708
1.6	0	clcB	2	25.6667	0.456
1.6	0	ycaM	2	25.6667	0.457
1.6	0	yadI	2	25.6667	0.831
1.6	0	AG1	2	25.6667	0.577
1.6	0	ptsI	2	25.9167	0.713
1.6	0	clcB	2	25.9167	0.459
1.6	0	ycaM	2	25.9167	0.458
1.6	0	yadI	2	25.9167	0.833
1.6	0	AG1	2	25.9167	0.578
1.6	0	ptsI	2	26.1667	0.724
1.6	0	clcB	2	26.1667	0.462
1.6	0	ycaM	2	26.1667	0.462
1.6	0	yadI	2	26.1667	0.83
1.6	0	AG1	2	26.1667	0.589
1.6	0	ptsI	2	26.4167	0.732
1.6	0	clcB	2	26.4167	0.462
1.6	0	ycaM	2	26.4167	0.463
1.6	0	yadI	2	26.4167	0.835
1.6	0	AG1	2	26.4167	0.588
1.6	0	ptsI	3	0	0.15
1.6	0	clcB	3	0	0.155
1.6	0	ycaM	3	0	0.154
1.6	0	yadI	3	0	0.166
1.6	0	AG1	3	0	0.167

1.6	0	ptsI	3	0.25	0.147
1.6	0	clcB	3	0.25	0.152
1.6	0	ycaM	3	0.25	0.15
1.6	0	yadI	3	0.25	0.163
1.6	0	AG1	3	0.25	0.167
1.6	0	ptsI	3	0.5	0.148
1.6	0	clcB	3	0.5	0.151
1.6	0	ycaM	3	0.5	0.148
1.6	0	yadI	3	0.5	0.162
1.6	0	AG1	3	0.5	0.161
1.6	0	ptsI	3	0.75	0.149
1.6	0	clcB	3	0.75	0.151
1.6	0	ycaM	3	0.75	0.148
1.6	0	yadI	3	0.75	0.163
1.6	0	AG1	3	0.75	0.16
1.6	0	ptsI	3	1	0.162
1.6	0	clcB	3	1	0.152
1.6	0	ycaM	3	1	0.15
1.6	0	yadI	3	1	0.163
1.6	0	AG1	3	1	0.162
1.6	0	ptsI	3	1.25	0.15
1.6	0	clcB	3	1.25	0.152
1.6	0	ycaM	3	1.25	0.15
1.6	0	yadI	3	1.25	0.163
1.6	0	AG1	3	1.25	0.164
1.6	0	ptsI	3	1.5	0.151
1.6	0	clcB	3	1.5	0.153
1.6	0	ycaM	3	1.5	0.151
1.6	0	yadI	3	1.5	0.164
1.6	0	AG1	3	1.5	0.166
1.6	0	ptsI	3	1.75	0.152
1.6	0	clcB	3	1.75	0.153
1.6	0	ycaM	3	1.75	0.151
1.6	0	yadI	3	1.75	0.164
1.6	0	AG1	3	1.75	0.165
1.6	0	ptsI	3	2	0.153
1.6	0	clcB	3	2	0.156
1.6	0	ycaM	3	2	0.153
1.6	0	yadI	3	2	0.165
1.6	0	AG1	3	2	0.167
1.6	0	ptsI	3	2.25	0.155
1.6	0	clcB	3	2.25	0.157
1.6	0	ycaM	3	2.25	0.154
1.6	0	yadI	3	2.25	0.169
1.6	0	AG1	3	2.25	0.169
1.6	0	ptsI	3	2.5	0.156
1.6	0	clcB	3	2.5	0.16
1.6	0	ycaM	3	2.5	0.157
1.6	0	yadI	3	2.5	0.169
1.6	0	AG1	3	2.5	0.17
1.6	0	ptsI	3	2.75	0.159
1.6	0	clcB	3	2.75	0.163
1.6	0	ycaM	3	2.75	0.161

1.6	0	yadI	3	2.75	0.177
1.6	0	AG1	3	2.75	0.176
1.6	0	ptsI	3	3	0.16
1.6	0	clcB	3	3	0.164
1.6	0	ycaM	3	3	0.159
1.6	0	yadI	3	3	0.178
1.6	0	AG1	3	3	0.176
1.6	0	ptsI	3	3.25	0.162
1.6	0	clcB	3	3.25	0.165
1.6	0	ycaM	3	3.25	0.161
1.6	0	yadI	3	3.25	0.18
1.6	0	AG1	3	3.25	0.178
1.6	0	ptsI	3	3.5	0.166
1.6	0	clcB	3	3.5	0.168
1.6	0	ycaM	3	3.5	0.166
1.6	0	yadI	3	3.5	0.187
1.6	0	AG1	3	3.5	0.182
1.6	0	ptsI	3	3.75	0.167
1.6	0	clcB	3	3.75	0.172
1.6	0	ycaM	3	3.75	0.17
1.6	0	yadI	3	3.75	0.195
1.6	0	AG1	3	3.75	0.186
1.6	0	ptsI	3	4	0.171
1.6	0	clcB	3	4	0.174
1.6	0	ycaM	3	4	0.173
1.6	0	yadI	3	4	0.205
1.6	0	AG1	3	4	0.19
1.6	0	ptsI	3	4.41667	0.192
1.6	0	clcB	3	4.41667	0.164
1.6	0	ycaM	3	4.41667	0.184
1.6	0	yadI	3	4.41667	0.201
1.6	0	AG1	3	4.41667	0.204
1.6	0	ptsI	3	4.66667	0.2
1.6	0	clcB	3	4.66667	0.164
1.6	0	ycaM	3	4.66667	0.18
1.6	0	yadI	3	4.66667	0.201
1.6	0	AG1	3	4.66667	0.193
1.6	0	ptsI	3	4.91667	0.201
1.6	0	clcB	3	4.91667	0.163
1.6	0	ycaM	3	4.91667	0.184
1.6	0	yadI	3	4.91667	0.206
1.6	0	AG1	3	4.91667	0.196
1.6	0	ptsI	3	5.16667	0.204
1.6	0	clcB	3	5.16667	0.164
1.6	0	ycaM	3	5.16667	0.189
1.6	0	yadI	3	5.16667	0.217
1.6	0	AG1	3	5.16667	0.2
1.6	0	ptsI	3	5.41667	0.206
1.6	0	clcB	3	5.41667	0.168
1.6	0	ycaM	3	5.41667	0.194
1.6	0	yadI	3	5.41667	0.227
1.6	0	AG1	3	5.41667	0.208
1.6	0	ptsI	3	5.66667	0.211

1.6	0	clcB	3	5.66667	0.171
1.6	0	ycaM	3	5.66667	0.202
1.6	0	yadI	3	5.66667	0.24
1.6	0	AG1	3	5.66667	0.214
1.6	0	ptsI	3	5.91667	0.215
1.6	0	clcB	3	5.91667	0.175
1.6	0	ycaM	3	5.91667	0.209
1.6	0	yadI	3	5.91667	0.254
1.6	0	AG1	3	5.91667	0.223
1.6	0	ptsI	3	6.16667	0.219
1.6	0	clcB	3	6.16667	0.181
1.6	0	ycaM	3	6.16667	0.219
1.6	0	yadI	3	6.16667	0.267
1.6	0	AG1	3	6.16667	0.232
1.6	0	ptsI	3	6.41667	0.227
1.6	0	clcB	3	6.41667	0.188
1.6	0	ycaM	3	6.41667	0.231
1.6	0	yadI	3	6.41667	0.277
1.6	0	AG1	3	6.41667	0.242
1.6	0	ptsI	3	6.66667	0.237
1.6	0	clcB	3	6.66667	0.192
1.6	0	ycaM	3	6.66667	0.243
1.6	0	yadI	3	6.66667	0.289
1.6	0	AG1	3	6.66667	0.255
1.6	0	ptsI	3	6.91667	0.246
1.6	0	clcB	3	6.91667	0.198
1.6	0	ycaM	3	6.91667	0.257
1.6	0	yadI	3	6.91667	0.3
1.6	0	AG1	3	6.91667	0.265
1.6	0	ptsI	3	7.16667	0.256
1.6	0	clcB	3	7.16667	0.206
1.6	0	ycaM	3	7.16667	0.27
1.6	0	yadI	3	7.16667	0.313
1.6	0	AG1	3	7.16667	0.274
1.6	0	ptsI	3	7.41667	0.268
1.6	0	clcB	3	7.41667	0.214
1.6	0	ycaM	3	7.41667	0.278
1.6	0	yadI	3	7.41667	0.324
1.6	0	AG1	3	7.41667	0.279
1.6	0	ptsI	3	7.66667	0.275
1.6	0	clcB	3	7.66667	0.224
1.6	0	ycaM	3	7.66667	0.286
1.6	0	yadI	3	7.66667	0.337
1.6	0	AG1	3	7.66667	0.288
1.6	0	ptsI	3	7.91667	0.285
1.6	0	clcB	3	7.91667	0.235
1.6	0	ycaM	3	7.91667	0.294
1.6	0	yadI	3	7.91667	0.348
1.6	0	AG1	3	7.91667	0.295
1.6	0	ptsI	3	8.16667	0.291
1.6	0	clcB	3	8.16667	0.245
1.6	0	ycaM	3	8.16667	0.303
1.6	0	yadI	3	8.16667	0.358

1.6	0	AG1	3	8.16667	0.303
1.6	0	ptsI	3	8.41667	0.3
1.6	0	clcB	3	8.41667	0.252
1.6	0	ycaM	3	8.41667	0.311
1.6	0	yadI	3	8.41667	0.367
1.6	0	AG1	3	8.41667	0.31
1.6	0	ptsI	3	9.05	0.261
1.6	0	clcB	3	9.05	0.247
1.6	0	ycaM	3	9.05	0.303
1.6	0	yadI	3	9.05	0.401
1.6	0	AG1	3	9.05	0.342
1.6	0	ptsI	3	9.3	0.272
1.6	0	clcB	3	9.3	0.255
1.6	0	ycaM	3	9.3	0.312
1.6	0	yadI	3	9.3	0.414
1.6	0	AG1	3	9.3	0.341
1.6	0	ptsI	3	9.55	0.284
1.6	0	clcB	3	9.55	0.261
1.6	0	ycaM	3	9.55	0.322
1.6	0	yadI	3	9.55	0.423
1.6	0	AG1	3	9.55	0.355
1.6	0	ptsI	3	9.8	0.291
1.6	0	clcB	3	9.8	0.268
1.6	0	ycaM	3	9.8	0.329
1.6	0	yadI	3	9.8	0.434
1.6	0	AG1	3	9.8	0.367
1.6	0	ptsI	3	10.05	0.302
1.6	0	clcB	3	10.05	0.273
1.6	0	ycaM	3	10.05	0.338
1.6	0	yadI	3	10.05	0.442
1.6	0	AG1	3	10.05	0.376
1.6	0	ptsI	3	10.3	0.311
1.6	0	clcB	3	10.3	0.28
1.6	0	ycaM	3	10.3	0.345
1.6	0	yadI	3	10.3	0.451
1.6	0	AG1	3	10.3	0.387
1.6	0	ptsI	3	10.55	0.321
1.6	0	clcB	3	10.55	0.288
1.6	0	ycaM	3	10.55	0.353
1.6	0	yadI	3	10.55	0.458
1.6	0	AG1	3	10.55	0.397
1.6	0	ptsI	3	10.8	0.328
1.6	0	clcB	3	10.8	0.294
1.6	0	ycaM	3	10.8	0.36
1.6	0	yadI	3	10.8	0.468
1.6	0	AG1	3	10.8	0.404
1.6	0	ptsI	3	11.05	0.339
1.6	0	clcB	3	11.05	0.301
1.6	0	ycaM	3	11.05	0.371
1.6	0	yadI	3	11.05	0.474
1.6	0	AG1	3	11.05	0.414
1.6	0	ptsI	3	11.3	0.346
1.6	0	clcB	3	11.3	0.292

1.6	0	ycaM	3	11.3	0.369
1.6	0	yadI	3	11.3	0.478
1.6	0	AG1	3	11.3	0.414
1.6	0	ptsI	3	11.55	0.356
1.6	0	clcB	3	11.55	0.3
1.6	0	ycaM	3	11.55	0.369
1.6	0	yadI	3	11.55	0.487
1.6	0	AG1	3	11.55	0.419
1.6	0	ptsI	3	11.8	0.356
1.6	0	clcB	3	11.8	0.288
1.6	0	ycaM	3	11.8	0.378
1.6	0	yadI	3	11.8	0.495
1.6	0	AG1	3	11.8	0.429
1.6	0	ptsI	3	12.05	0.349
1.6	0	clcB	3	12.05	0.304
1.6	0	ycaM	3	12.05	0.375
1.6	0	yadI	3	12.05	0.501
1.6	0	AG1	3	12.05	0.429
1.6	0	ptsI	3	12.3	0.358
1.6	0	clcB	3	12.3	0.308
1.6	0	ycaM	3	12.3	0.376
1.6	0	yadI	3	12.3	0.506
1.6	0	AG1	3	12.3	0.434
1.6	0	ptsI	3	12.55	0.361
1.6	0	clcB	3	12.55	0.292
1.6	0	ycaM	3	12.55	0.377
1.6	0	yadI	3	12.55	0.51
1.6	0	AG1	3	12.55	0.448
1.6	0	ptsI	3	12.8	0.366
1.6	0	clcB	3	12.8	0.33
1.6	0	ycaM	3	12.8	0.376
1.6	0	yadI	3	12.8	0.515
1.6	0	AG1	3	12.8	0.449
1.6	0	ptsI	3	13.05	0.389
1.6	0	clcB	3	13.05	0.317
1.6	0	ycaM	3	13.05	0.388
1.6	0	yadI	3	13.05	0.514
1.6	0	AG1	3	13.05	0.452
1.6	0	ptsI	3	13.3	0.382
1.6	0	clcB	3	13.3	0.323
1.6	0	ycaM	3	13.3	0.392
1.6	0	yadI	3	13.3	0.516
1.6	0	AG1	3	13.3	0.457
1.6	0	ptsI	3	13.55	0.382
1.6	0	clcB	3	13.55	0.321
1.6	0	ycaM	3	13.55	0.393
1.6	0	yadI	3	13.55	0.521
1.6	0	AG1	3	13.55	0.463
1.6	0	ptsI	3	13.8	0.404
1.6	0	clcB	3	13.8	0.312
1.6	0	ycaM	3	13.8	0.398
1.6	0	yadI	3	13.8	0.523
1.6	0	AG1	3	13.8	0.47

1.6	0	ptsI	3	14.05	0.405
1.6	0	clcB	3	14.05	0.315
1.6	0	ycaM	3	14.05	0.4
1.6	0	yadI	3	14.05	0.53
1.6	0	AG1	3	14.05	0.482
1.6	0	ptsI	3	14.3	0.418
1.6	0	clcB	3	14.3	0.326
1.6	0	ycaM	3	14.3	0.41
1.6	0	yadI	3	14.3	0.534
1.6	0	AG1	3	14.3	0.485
1.6	0	ptsI	3	14.55	0.419
1.6	0	clcB	3	14.55	0.33
1.6	0	ycaM	3	14.55	0.416
1.6	0	yadI	3	14.55	0.539
1.6	0	AG1	3	14.55	0.496
1.6	0	ptsI	3	14.8	0.424
1.6	0	clcB	3	14.8	0.502
1.6	0	ycaM	3	14.8	0.422
1.6	0	yadI	3	14.8	0.543
1.6	0	AG1	3	14.8	0.5
1.6	0	ptsI	3	15.05	0.428
1.6	0	clcB	3	15.05	0.337
1.6	0	ycaM	3	15.05	0.427
1.6	0	yadI	3	15.05	0.548
1.6	0	AG1	3	15.05	0.504
1.6	0	ptsI	3	15.3	0.443
1.6	0	clcB	3	15.3	0.353
1.6	0	ycaM	3	15.3	0.434
1.6	0	yadI	3	15.3	0.551
1.6	0	AG1	3	15.3	0.513
1.6	0	ptsI	3	15.55	0.446
1.6	0	clcB	3	15.55	0.357
1.6	0	ycaM	3	15.55	0.439
1.6	0	yadI	3	15.55	0.554
1.6	0	AG1	3	15.55	0.519
1.6	0	ptsI	3	15.8	0.454
1.6	0	clcB	3	15.8	0.359
1.6	0	ycaM	3	15.8	0.446
1.6	0	yadI	3	15.8	0.557
1.6	0	AG1	3	15.8	0.529
1.6	0	ptsI	3	16.05	0.466
1.6	0	clcB	3	16.05	0.353
1.6	0	ycaM	3	16.05	0.452
1.6	0	yadI	3	16.05	0.563
1.6	0	AG1	3	16.05	0.539
1.6	0	ptsI	3	16.3	0.471
1.6	0	clcB	3	16.3	0.356
1.6	0	ycaM	3	16.3	0.456
1.6	0	yadI	3	16.3	0.557
1.6	0	AG1	3	16.3	0.553
1.6	0	ptsI	3	16.55	0.475
1.6	0	clcB	3	16.55	0.365
1.6	0	ycaM	3	16.55	0.466

1.6	0	yadI	3	16.55	0.568
1.6	0	AG1	3	16.55	0.556
1.6	0	ptsI	3	16.8	0.477
1.6	0	clcB	3	16.8	0.374
1.6	0	ycaM	3	16.8	0.472
1.6	0	yadI	3	16.8	0.569
1.6	0	AG1	3	16.8	0.571
1.6	0	ptsI	3	17.05	0.486
1.6	0	clcB	3	17.05	0.378
1.6	0	ycaM	3	17.05	0.476
1.6	0	yadI	3	17.05	0.571
1.6	0	AG1	3	17.05	0.581
1.6	0	ptsI	3	17.3	0.487
1.6	0	clcB	3	17.3	0.384
1.6	0	ycaM	3	17.3	0.482
1.6	0	yadI	3	17.3	0.571
1.6	0	AG1	3	17.3	0.593
1.6	0	ptsI	3	17.55	0.49
1.6	0	clcB	3	17.55	0.388
1.6	0	ycaM	3	17.55	0.484
1.6	0	yadI	3	17.55	0.578
1.6	0	AG1	3	17.55	0.589
1.6	0	ptsI	3	17.8	0.492
1.6	0	clcB	3	17.8	0.391
1.6	0	ycaM	3	17.8	0.495
1.6	0	yadI	3	17.8	0.58
1.6	0	AG1	3	17.8	0.609
1.6	0	ptsI	3	18.05	0.496
1.6	0	clcB	3	18.05	0.403
1.6	0	ycaM	3	18.05	0.499
1.6	0	yadI	3	18.05	0.579
1.6	0	AG1	3	18.05	0.608
1.6	0	ptsI	3	18.3	0.5
1.6	0	clcB	3	18.3	0.404
1.6	0	ycaM	3	18.3	0.502
1.6	0	yadI	3	18.3	0.581
1.6	0	AG1	3	18.3	0.597
1.6	0	ptsI	3	18.55	0.505
1.6	0	clcB	3	18.55	0.409
1.6	0	ycaM	3	18.55	0.504
1.6	0	yadI	3	18.55	0.587
1.6	0	AG1	3	18.55	0.598
1.6	0	ptsI	3	18.8	0.517
1.6	0	clcB	3	18.8	0.41
1.6	0	ycaM	3	18.8	0.51
1.6	0	yadI	3	18.8	0.589
1.6	0	AG1	3	18.8	0.611
1.6	0	ptsI	3	19.05	0.519
1.6	0	clcB	3	19.05	0.414
1.6	0	ycaM	3	19.05	0.514
1.6	0	yadI	3	19.05	0.588
1.6	0	AG1	3	19.05	0.604
1.6	0	ptsI	3	19.3	0.531

1.6	0	clcB	3	19.3	0.411
1.6	0	ycaM	3	19.3	0.518
1.6	0	yadI	3	19.3	0.59
1.6	0	AG1	3	19.3	0.59
1.6	0	ptsI	3	19.55	0.542
1.6	0	clcB	3	19.55	0.421
1.6	0	ycaM	3	19.55	0.516
1.6	0	yadI	3	19.55	0.592
1.6	0	AG1	3	19.55	0.609
1.6	0	ptsI	3	19.8	0.557
1.6	0	clcB	3	19.8	0.425
1.6	0	ycaM	3	19.8	0.516
1.6	0	yadI	3	19.8	0.593
1.6	0	AG1	3	19.8	0.602
1.6	0	ptsI	3	20.05	0.565
1.6	0	clcB	3	20.05	0.414
1.6	0	ycaM	3	20.05	0.52
1.6	0	yadI	3	20.05	0.591
1.6	0	AG1	3	20.05	0.598
1.6	0	ptsI	3	20.3	0.563
1.6	0	clcB	3	20.3	0.424
1.6	0	ycaM	3	20.3	0.526
1.6	0	yadI	3	20.3	0.591
1.6	0	AG1	3	20.3	0.58
1.6	0	ptsI	3	20.55	0.564
1.6	0	clcB	3	20.55	0.427
1.6	0	ycaM	3	20.55	0.532
1.6	0	yadI	3	20.55	0.594
1.6	0	AG1	3	20.55	0.581
1.6	0	ptsI	3	20.8	0.571
1.6	0	clcB	3	20.8	0.424
1.6	0	ycaM	3	20.8	0.533
1.6	0	yadI	3	20.8	0.594
1.6	0	AG1	3	20.8	0.569
1.6	0	ptsI	3	21.05	0.57
1.6	0	clcB	3	21.05	0.434
1.6	0	ycaM	3	21.05	0.543
1.6	0	yadI	3	21.05	0.596
1.6	0	AG1	3	21.05	0.561
1.6	0	ptsI	3	21.3	0.584
1.6	0	clcB	3	21.3	0.433
1.6	0	ycaM	3	21.3	0.538
1.6	0	yadI	3	21.3	0.594
1.6	0	AG1	3	21.3	0.555
1.6	0	ptsI	3	21.55	0.576
1.6	0	clcB	3	21.55	0.44
1.6	0	ycaM	3	21.55	0.546
1.6	0	yadI	3	21.55	0.6
1.6	0	AG1	3	21.55	0.565
1.6	0	ptsI	3	21.8	0.58
1.6	0	clcB	3	21.8	0.444
1.6	0	ycaM	3	21.8	0.551
1.6	0	yadI	3	21.8	0.6

1.6	0	AG1	3	21.8	0.565
1.6	0	ptsI	3	22.05	0.574
1.6	0	clcB	3	22.05	0.45
1.6	0	ycaM	3	22.05	0.541
1.6	0	yadI	3	22.05	0.6
1.6	0	AG1	3	22.05	0.564
1.6	0	ptsI	3	22.3	0.576
1.6	0	clcB	3	22.3	0.448
1.6	0	ycaM	3	22.3	0.555
1.6	0	yadI	3	22.3	0.601
1.6	0	AG1	3	22.3	0.552
1.6	0	ptsI	3	22.55	0.58
1.6	0	clcB	3	22.55	0.461
1.6	0	ycaM	3	22.55	0.556
1.6	0	yadI	3	22.55	0.603
1.6	0	AG1	3	22.55	0.56
1.6	0	ptsI	3	22.8	0.572
1.6	0	clcB	3	22.8	0.471
1.6	0	ycaM	3	22.8	0.555
1.6	0	yadI	3	22.8	0.602
1.6	0	AG1	3	22.8	0.56
1.6	0	ptsI	3	23.05	0.57
1.6	0	clcB	3	23.05	0.464
1.6	0	ycaM	3	23.05	0.564
1.6	0	yadI	3	23.05	0.6
1.6	0	AG1	3	23.05	0.576
1.6	0	ptsI	3	23.3	0.574
1.6	0	clcB	3	23.3	0.461
1.6	0	ycaM	3	23.3	0.57
1.6	0	yadI	3	23.3	0.602
1.6	0	AG1	3	23.3	0.576
1.6	0	ptsI	3	23.55	0.564
1.6	0	clcB	3	23.55	0.455
1.6	0	ycaM	3	23.55	0.58
1.6	0	yadI	3	23.55	0.601
1.6	0	AG1	3	23.55	0.559
1.6	0	ptsI	3	23.8	0.575
1.6	0	clcB	3	23.8	0.457
1.6	0	ycaM	3	23.8	0.591
1.6	0	yadI	3	23.8	0.602
1.6	0	AG1	3	23.8	0.569
1.6	0	ptsI	3	24.05	0.585
1.6	0	clcB	3	24.05	0.461
1.6	0	ycaM	3	24.05	0.588
1.6	0	yadI	3	24.05	0.601
1.6	0	AG1	3	24.05	0.592
1.6	0	ptsI	3	24.3	0.587
1.6	0	clcB	3	24.3	0.467
1.6	0	ycaM	3	24.3	0.59
1.6	0	yadI	3	24.3	0.598
1.6	0	AG1	3	24.3	0.584
1.6	0	ptsI	4	0	0.192
1.6	0	clcB	4	0	0.2

1.6	0	ycaM	4	0	0.278
1.6	0	yadI	4	0	0.248
1.6	0	AG1	4	0	0.233
1.6	0	ptsI	4	0.25	0.19
1.6	0	clcB	4	0.25	0.197
1.6	0	ycaM	4	0.25	0.271
1.6	0	yadI	4	0.25	0.247
1.6	0	AG1	4	0.25	0.229
1.6	0	ptsI	4	0.5	0.192
1.6	0	clcB	4	0.5	0.197
1.6	0	ycaM	4	0.5	0.271
1.6	0	yadI	4	0.5	0.257
1.6	0	AG1	4	0.5	0.232
1.6	0	ptsI	4	0.75	0.195
1.6	0	clcB	4	0.75	0.2
1.6	0	ycaM	4	0.75	0.274
1.6	0	yadI	4	0.75	0.255
1.6	0	AG1	4	0.75	0.236
1.6	0	ptsI	4	1	0.201
1.6	0	clcB	4	1	0.201
1.6	0	ycaM	4	1	0.276
1.6	0	yadI	4	1	0.252
1.6	0	AG1	4	1	0.241
1.6	0	ptsI	4	1.25	0.206
1.6	0	clcB	4	1.25	0.209
1.6	0	ycaM	4	1.25	0.28
1.6	0	yadI	4	1.25	0.258
1.6	0	AG1	4	1.25	0.248
1.6	0	ptsI	4	1.5	0.205
1.6	0	clcB	4	1.5	0.208
1.6	0	ycaM	4	1.5	0.285
1.6	0	yadI	4	1.5	0.264
1.6	0	AG1	4	1.5	0.243
1.6	0	ptsI	4	1.75	0.215
1.6	0	clcB	4	1.75	0.219
1.6	0	ycaM	4	1.75	0.292
1.6	0	yadI	4	1.75	0.272
1.6	0	AG1	4	1.75	0.259
1.6	0	ptsI	4	2	0.227
1.6	0	clcB	4	2	0.232
1.6	0	ycaM	4	2	0.301
1.6	0	yadI	4	2	0.283
1.6	0	AG1	4	2	0.272
1.6	0	ptsI	4	2.25	0.243
1.6	0	clcB	4	2.25	0.242
1.6	0	ycaM	4	2.25	0.312
1.6	0	yadI	4	2.25	0.292
1.6	0	AG1	4	2.25	0.289
1.6	0	ptsI	4	2.5	0.24
1.6	0	clcB	4	2.5	0.247
1.6	0	ycaM	4	2.5	0.321
1.6	0	yadI	4	2.5	0.301
1.6	0	AG1	4	2.5	0.292

1.6	0	ptsI	4	2.75	0.25
1.6	0	clcB	4	2.75	0.26
1.6	0	ycaM	4	2.75	0.327
1.6	0	yadI	4	2.75	0.312
1.6	0	AG1	4	2.75	0.304
1.6	0	ptsI	4	3	0.264
1.6	0	clcB	4	3	0.275
1.6	0	ycaM	4	3	0.342
1.6	0	yadI	4	3	0.329
1.6	0	AG1	4	3	0.32
1.6	0	ptsI	4	3.25	0.277
1.6	0	clcB	4	3.25	0.284
1.6	0	ycaM	4	3.25	0.354
1.6	0	yadI	4	3.25	0.349
1.6	0	AG1	4	3.25	0.337
1.6	0	ptsI	4	3.5	0.297
1.6	0	clcB	4	3.5	0.3
1.6	0	ycaM	4	3.5	0.362
1.6	0	yadI	4	3.5	0.366
1.6	0	AG1	4	3.5	0.356
1.6	0	ptsI	4	3.75	0.314
1.6	0	clcB	4	3.75	0.314
1.6	0	ycaM	4	3.75	0.368
1.6	0	yadI	4	3.75	0.385
1.6	0	AG1	4	3.75	0.368
1.6	0	ptsI	4	4	0.331
1.6	0	clcB	4	4	0.33
1.6	0	ycaM	4	4	0.388
1.6	0	yadI	4	4	0.405
1.6	0	AG1	4	4	0.39
1.6	0	ptsI	4	4.35	0.334
1.6	0	clcB	4	4.35	0.27
1.6	0	ycaM	4	4.35	0.392
1.6	0	yadI	4	4.35	0.39
1.6	0	AG1	4	4.35	0.298
1.6	0	ptsI	4	4.6	0.343
1.6	0	clcB	4	4.6	0.273
1.6	0	ycaM	4	4.6	0.386
1.6	0	yadI	4	4.6	0.394
1.6	0	AG1	4	4.6	0.307
1.6	0	ptsI	4	4.85	0.347
1.6	0	clcB	4	4.85	0.28
1.6	0	ycaM	4	4.85	0.398
1.6	0	yadI	4	4.85	0.408
1.6	0	AG1	4	4.85	0.316
1.6	0	ptsI	4	5.1	0.353
1.6	0	clcB	4	5.1	0.288
1.6	0	ycaM	4	5.1	0.41
1.6	0	yadI	4	5.1	0.422
1.6	0	AG1	4	5.1	0.325
1.6	0	ptsI	4	5.35	0.37
1.6	0	clcB	4	5.35	0.298
1.6	0	ycaM	4	5.35	0.42

1.6	0	yadI	4	5.35	0.435
1.6	0	AG1	4	5.35	0.337
1.6	0	ptsI	4	5.6	0.377
1.6	0	clcB	4	5.6	0.307
1.6	0	ycaM	4	5.6	0.424
1.6	0	yadI	4	5.6	0.448
1.6	0	AG1	4	5.6	0.346
1.6	0	ptsI	4	5.85	0.382
1.6	0	clcB	4	5.85	0.317
1.6	0	ycaM	4	5.85	0.431
1.6	0	yadI	4	5.85	0.464
1.6	0	AG1	4	5.85	0.352
1.6	0	ptsI	4	6.1	0.396
1.6	0	clcB	4	6.1	0.327
1.6	0	ycaM	4	6.1	0.443
1.6	0	yadI	4	6.1	0.477
1.6	0	AG1	4	6.1	0.361
1.6	0	ptsI	4	6.35	0.407
1.6	0	clcB	4	6.35	0.338
1.6	0	ycaM	4	6.35	0.452
1.6	0	yadI	4	6.35	0.491
1.6	0	AG1	4	6.35	0.369
1.6	0	ptsI	4	6.6	0.419
1.6	0	clcB	4	6.6	0.347
1.6	0	ycaM	4	6.6	0.461
1.6	0	yadI	4	6.6	0.501
1.6	0	AG1	4	6.6	0.378
1.6	0	ptsI	4	6.85	0.43
1.6	0	clcB	4	6.85	0.352
1.6	0	ycaM	4	6.85	0.468
1.6	0	yadI	4	6.85	0.512
1.6	0	AG1	4	6.85	0.386
1.6	0	ptsI	4	7.1	0.438
1.6	0	clcB	4	7.1	0.361
1.6	0	ycaM	4	7.1	0.478
1.6	0	yadI	4	7.1	0.522
1.6	0	AG1	4	7.1	0.396
1.6	0	ptsI	4	7.35	0.446
1.6	0	clcB	4	7.35	0.369
1.6	0	ycaM	4	7.35	0.482
1.6	0	yadI	4	7.35	0.532
1.6	0	AG1	4	7.35	0.402
1.6	0	ptsI	4	7.6	0.455
1.6	0	clcB	4	7.6	0.377
1.6	0	ycaM	4	7.6	0.485
1.6	0	yadI	4	7.6	0.541
1.6	0	AG1	4	7.6	0.406
1.6	0	ptsI	4	7.85	0.462
1.6	0	clcB	4	7.85	0.383
1.6	0	ycaM	4	7.85	0.486
1.6	0	yadI	4	7.85	0.545
1.6	0	AG1	4	7.85	0.417
1.6	0	ptsI	4	8.1	0.473

1.6	0	clcB	4	8.1	0.391
1.6	0	ycaM	4	8.1	0.493
1.6	0	yadI	4	8.1	0.559
1.6	0	AG1	4	8.1	0.426
1.6	0	ptsI	4	8.35	0.483
1.6	0	clcB	4	8.35	0.396
1.6	0	ycaM	4	8.35	0.495
1.6	0	yadI	4	8.35	0.566
1.6	0	AG1	4	8.35	0.433
1.6	0	ptsI	4	8.83333	0.403
1.6	0	clcB	4	8.83333	0.388
1.6	0	ycaM	4	8.83333	0.434
1.6	0	yadI	4	8.83333	0.543
1.6	0	AG1	4	8.83333	0.425
1.6	0	ptsI	4	9.08333	0.406
1.6	0	clcB	4	9.08333	0.389
1.6	0	ycaM	4	9.08333	0.429
1.6	0	yadI	4	9.08333	0.533
1.6	0	AG1	4	9.08333	0.427
1.6	0	ptsI	4	9.33333	0.416
1.6	0	clcB	4	9.33333	0.399
1.6	0	ycaM	4	9.33333	0.431
1.6	0	yadI	4	9.33333	0.534
1.6	0	AG1	4	9.33333	0.437
1.6	0	ptsI	4	9.58333	0.428
1.6	0	clcB	4	9.58333	0.404
1.6	0	ycaM	4	9.58333	0.434
1.6	0	yadI	4	9.58333	0.546
1.6	0	AG1	4	9.58333	0.446
1.6	0	ptsI	4	9.83333	0.434
1.6	0	clcB	4	9.83333	0.409
1.6	0	ycaM	4	9.83333	0.442
1.6	0	yadI	4	9.83333	0.553
1.6	0	AG1	4	9.83333	0.455
1.6	0	ptsI	4	10.0833	0.439
1.6	0	clcB	4	10.0833	0.412
1.6	0	ycaM	4	10.0833	0.447
1.6	0	yadI	4	10.0833	0.559
1.6	0	AG1	4	10.0833	0.461
1.6	0	ptsI	4	10.3333	0.445
1.6	0	clcB	4	10.3333	0.414
1.6	0	ycaM	4	10.3333	0.45
1.6	0	yadI	4	10.3333	0.563
1.6	0	AG1	4	10.3333	0.465
1.6	0	ptsI	4	10.5833	0.449
1.6	0	clcB	4	10.5833	0.415
1.6	0	ycaM	4	10.5833	0.452
1.6	0	yadI	4	10.5833	0.57
1.6	0	AG1	4	10.5833	0.472
1.6	0	ptsI	4	10.8333	0.454
1.6	0	clcB	4	10.8333	0.422
1.6	0	ycaM	4	10.8333	0.456
1.6	0	yadI	4	10.8333	0.572

1.6	0	AG1	4	10.8333	0.477
1.6	0	ptsI	4	11.0833	0.457
1.6	0	clcB	4	11.0833	0.424
1.6	0	ycaM	4	11.0833	0.459
1.6	0	yadI	4	11.0833	0.578
1.6	0	AG1	4	11.0833	0.477
1.6	0	ptsI	4	11.3333	0.462
1.6	0	clcB	4	11.3333	0.426
1.6	0	ycaM	4	11.3333	0.464
1.6	0	yadI	4	11.3333	0.584
1.6	0	AG1	4	11.3333	0.482
1.6	0	ptsI	4	11.5833	0.467
1.6	0	clcB	4	11.5833	0.428
1.6	0	ycaM	4	11.5833	0.466
1.6	0	yadI	4	11.5833	0.589
1.6	0	AG1	4	11.5833	0.481
1.6	0	ptsI	4	11.8333	0.47
1.6	0	clcB	4	11.8333	0.43
1.6	0	ycaM	4	11.8333	0.468
1.6	0	yadI	4	11.8333	0.591
1.6	0	AG1	4	11.8333	0.483
1.6	0	ptsI	4	12.0833	0.473
1.6	0	clcB	4	12.0833	0.432
1.6	0	ycaM	4	12.0833	0.473
1.6	0	yadI	4	12.0833	0.597
1.6	0	AG1	4	12.0833	0.489
1.6	0	ptsI	4	12.3333	0.474
1.6	0	clcB	4	12.3333	0.436
1.6	0	ycaM	4	12.3333	0.474
1.6	0	yadI	4	12.3333	0.603
1.6	0	AG1	4	12.3333	0.492
1.6	0	ptsI	4	12.5833	0.478
1.6	0	clcB	4	12.5833	0.432
1.6	0	ycaM	4	12.5833	0.475
1.6	0	yadI	4	12.5833	0.604
1.6	0	AG1	4	12.5833	0.487
1.6	0	ptsI	4	12.8333	0.482
1.6	0	clcB	4	12.8333	0.439
1.6	0	ycaM	4	12.8333	0.48
1.6	0	yadI	4	12.8333	0.609
1.6	0	AG1	4	12.8333	0.494
1.6	0	ptsI	4	13.0833	0.483
1.6	0	clcB	4	13.0833	0.437
1.6	0	ycaM	4	13.0833	0.485
1.6	0	yadI	4	13.0833	0.611
1.6	0	AG1	4	13.0833	0.499
1.6	0	ptsI	4	13.3333	0.487
1.6	0	clcB	4	13.3333	0.44
1.6	0	ycaM	4	13.3333	0.486
1.6	0	yadI	4	13.3333	0.613
1.6	0	AG1	4	13.3333	0.499
1.6	0	ptsI	4	13.5833	0.485
1.6	0	clcB	4	13.5833	0.44

1.6	0	ycaM	4	13.5833	0.487
1.6	0	yadI	4	13.5833	0.615
1.6	0	AG1	4	13.5833	0.5
1.6	0	ptsI	4	13.8333	0.487
1.6	0	clcB	4	13.8333	0.442
1.6	0	ycaM	4	13.8333	0.492
1.6	0	yadI	4	13.8333	0.618
1.6	0	AG1	4	13.8333	0.505
1.6	0	ptsI	4	14.0833	0.49
1.6	0	clcB	4	14.0833	0.444
1.6	0	ycaM	4	14.0833	0.49
1.6	0	yadI	4	14.0833	0.619
1.6	0	AG1	4	14.0833	0.503
1.6	0	ptsI	4	14.3333	0.489
1.6	0	clcB	4	14.3333	0.447
1.6	0	ycaM	4	14.3333	0.492
1.6	0	yadI	4	14.3333	0.624
1.6	0	AG1	4	14.3333	0.506
1.6	0	ptsI	4	14.5833	0.493
1.6	0	clcB	4	14.5833	0.45
1.6	0	ycaM	4	14.5833	0.495
1.6	0	yadI	4	14.5833	0.626
1.6	0	AG1	4	14.5833	0.505
1.6	0	ptsI	4	14.8333	0.499
1.6	0	clcB	4	14.8333	0.451
1.6	0	ycaM	4	14.8333	0.5
1.6	0	yadI	4	14.8333	0.624
1.6	0	AG1	4	14.8333	0.508
1.6	0	ptsI	4	15.0833	0.5
1.6	0	clcB	4	15.0833	0.452
1.6	0	ycaM	4	15.0833	0.499
1.6	0	yadI	4	15.0833	0.624
1.6	0	AG1	4	15.0833	0.508
1.6	0	ptsI	4	15.3333	0.499
1.6	0	clcB	4	15.3333	0.451
1.6	0	ycaM	4	15.3333	0.498
1.6	0	yadI	4	15.3333	0.625
1.6	0	AG1	4	15.3333	0.509
1.6	0	ptsI	4	15.5833	0.503
1.6	0	clcB	4	15.5833	0.454
1.6	0	ycaM	4	15.5833	0.5
1.6	0	yadI	4	15.5833	0.629
1.6	0	AG1	4	15.5833	0.509
1.6	0	ptsI	4	15.8333	0.509
1.6	0	clcB	4	15.8333	0.459
1.6	0	ycaM	4	15.8333	0.503
1.6	0	yadI	4	15.8333	0.632
1.6	0	AG1	4	15.8333	0.514
1.6	0	ptsI	4	16.0833	0.511
1.6	0	clcB	4	16.0833	0.454
1.6	0	ycaM	4	16.0833	0.504
1.6	0	yadI	4	16.0833	0.63
1.6	0	AG1	4	16.0833	0.513

1.6	0	ptsI	4	16.3333	0.514
1.6	0	clcB	4	16.3333	0.46
1.6	0	ycaM	4	16.3333	0.508
1.6	0	yadI	4	16.3333	0.634
1.6	0	AG1	4	16.3333	0.516
1.6	0	ptsI	4	16.5833	0.513
1.6	0	clcB	4	16.5833	0.458
1.6	0	ycaM	4	16.5833	0.507
1.6	0	yadI	4	16.5833	0.634
1.6	0	AG1	4	16.5833	0.515
1.6	0	ptsI	4	16.8333	0.517
1.6	0	clcB	4	16.8333	0.463
1.6	0	ycaM	4	16.8333	0.511
1.6	0	yadI	4	16.8333	0.635
1.6	0	AG1	4	16.8333	0.516
1.6	0	ptsI	4	17.0833	0.518
1.6	0	clcB	4	17.0833	0.465
1.6	0	ycaM	4	17.0833	0.513
1.6	0	yadI	4	17.0833	0.639
1.6	0	AG1	4	17.0833	0.517
1.6	0	ptsI	4	17.3333	0.517
1.6	0	clcB	4	17.3333	0.464
1.6	0	ycaM	4	17.3333	0.516
1.6	0	yadI	4	17.3333	0.641
1.6	0	AG1	4	17.3333	0.52
1.6	0	ptsI	4	17.5833	0.523
1.6	0	clcB	4	17.5833	0.468
1.6	0	ycaM	4	17.5833	0.516
1.6	0	yadI	4	17.5833	0.645
1.6	0	AG1	4	17.5833	0.522
1.6	0	ptsI	4	17.8333	0.521
1.6	0	clcB	4	17.8333	0.468
1.6	0	ycaM	4	17.8333	0.513
1.6	0	yadI	4	17.8333	0.642
1.6	0	AG1	4	17.8333	0.522
1.6	0	ptsI	4	18.0833	0.522
1.6	0	clcB	4	18.0833	0.47
1.6	0	ycaM	4	18.0833	0.518
1.6	0	yadI	4	18.0833	0.646
1.6	0	AG1	4	18.0833	0.524
1.6	0	ptsI	4	18.3333	0.519
1.6	0	clcB	4	18.3333	0.472
1.6	0	ycaM	4	18.3333	0.519
1.6	0	yadI	4	18.3333	0.644
1.6	0	AG1	4	18.3333	0.523
1.6	0	ptsI	4	18.5833	0.518
1.6	0	clcB	4	18.5833	0.472
1.6	0	ycaM	4	18.5833	0.52
1.6	0	yadI	4	18.5833	0.644
1.6	0	AG1	4	18.5833	0.526
1.6	0	ptsI	4	18.8333	0.518
1.6	0	clcB	4	18.8333	0.474
1.6	0	ycaM	4	18.8333	0.517

1.6	0	yadI	4	18.8333	0.644
1.6	0	AG1	4	18.8333	0.525
1.6	0	ptsI	4	19.0833	0.523
1.6	0	clcB	4	19.0833	0.474
1.6	0	ycaM	4	19.0833	0.522
1.6	0	yadI	4	19.0833	0.643
1.6	0	AG1	4	19.0833	0.526
1.6	0	ptsI	4	19.3333	0.523
1.6	0	clcB	4	19.3333	0.477
1.6	0	ycaM	4	19.3333	0.523
1.6	0	yadI	4	19.3333	0.647
1.6	0	AG1	4	19.3333	0.528
1.6	0	ptsI	4	19.5833	0.524
1.6	0	clcB	4	19.5833	0.479
1.6	0	ycaM	4	19.5833	0.526
1.6	0	yadI	4	19.5833	0.65
1.6	0	AG1	4	19.5833	0.532
1.6	0	ptsI	4	19.8333	0.527
1.6	0	clcB	4	19.8333	0.481
1.6	0	ycaM	4	19.8333	0.523
1.6	0	yadI	4	19.8333	0.649
1.6	0	AG1	4	19.8333	0.532
1.6	0	ptsI	4	20.0833	0.53
1.6	0	clcB	4	20.0833	0.478
1.6	0	ycaM	4	20.0833	0.524
1.6	0	yadI	4	20.0833	0.65
1.6	0	AG1	4	20.0833	0.532
1.6	0	ptsI	4	20.3333	0.532
1.6	0	clcB	4	20.3333	0.481
1.6	0	ycaM	4	20.3333	0.528
1.6	0	yadI	4	20.3333	0.647
1.6	0	AG1	4	20.3333	0.531
1.6	0	ptsI	4	20.5833	0.536
1.6	0	clcB	4	20.5833	0.485
1.6	0	ycaM	4	20.5833	0.53
1.6	0	yadI	4	20.5833	0.654
1.6	0	AG1	4	20.5833	0.538
1.6	0	ptsI	4	20.8333	0.539
1.6	0	clcB	4	20.8333	0.486
1.6	0	ycaM	4	20.8333	0.531
1.6	0	yadI	4	20.8333	0.649
1.6	0	AG1	4	20.8333	0.537
1.6	0	ptsI	4	21.0833	0.541
1.6	0	clcB	4	21.0833	0.485
1.6	0	ycaM	4	21.0833	0.53
1.6	0	yadI	4	21.0833	0.651
1.6	0	AG1	4	21.0833	0.533
1.6	0	ptsI	4	21.3333	0.542
1.6	0	clcB	4	21.3333	0.488
1.6	0	ycaM	4	21.3333	0.532
1.6	0	yadI	4	21.3333	0.656
1.6	0	AG1	4	21.3333	0.535
1.6	0	ptsI	4	21.5833	0.546

1.6	0	clcB	4	21.5833	0.49
1.6	0	ycaM	4	21.5833	0.534
1.6	0	yadI	4	21.5833	0.652
1.6	0	AG1	4	21.5833	0.539
1.6	0	ptsI	4	21.8333	0.55
1.6	0	clcB	4	21.8333	0.49
1.6	0	ycaM	4	21.8333	0.534
1.6	0	yadI	4	21.8333	0.655
1.6	0	AG1	4	21.8333	0.542
1.6	0	ptsI	4	22.0833	0.551
1.6	0	clcB	4	22.0833	0.49
1.6	0	ycaM	4	22.0833	0.532
1.6	0	yadI	4	22.0833	0.652
1.6	0	AG1	4	22.0833	0.54
1.6	0	ptsI	4	22.3333	0.554
1.6	0	clcB	4	22.3333	0.493
1.6	0	ycaM	4	22.3333	0.535
1.6	0	yadI	4	22.3333	0.656
1.6	0	AG1	4	22.3333	0.545
1.6	0	ptsI	4	22.5833	0.559
1.6	0	clcB	4	22.5833	0.493
1.6	0	ycaM	4	22.5833	0.536
1.6	0	yadI	4	22.5833	0.658
1.6	0	AG1	4	22.5833	0.543
1.6	0	ptsI	4	22.8333	0.559
1.6	0	clcB	4	22.8333	0.494
1.6	0	ycaM	4	22.8333	0.538
1.6	0	yadI	4	22.8333	0.655
1.6	0	AG1	4	22.8333	0.545
1.6	0	ptsI	4	23.0833	0.563
1.6	0	clcB	4	23.0833	0.495
1.6	0	ycaM	4	23.0833	0.538
1.6	0	yadI	4	23.0833	0.658
1.6	0	AG1	4	23.0833	0.545
1.6	0	ptsI	4	23.3333	0.57
1.6	0	clcB	4	23.3333	0.495
1.6	0	ycaM	4	23.3333	0.538
1.6	0	yadI	4	23.3333	0.657
1.6	0	AG1	4	23.3333	0.546
1.6	0	ptsI	4	23.5833	0.572
1.6	0	clcB	4	23.5833	0.499
1.6	0	ycaM	4	23.5833	0.539
1.6	0	yadI	4	23.5833	0.658
1.6	0	AG1	4	23.5833	0.545
1.6	0	ptsI	4	23.8333	0.574
1.6	0	clcB	4	23.8333	0.499
1.6	0	ycaM	4	23.8333	0.538
1.6	0	yadI	4	23.8333	0.657
1.6	0	AG1	4	23.8333	0.546
1.6	0	ptsI	4	24.0833	0.577
1.6	0	clcB	4	24.0833	0.5
1.6	0	ycaM	4	24.0833	0.538
1.6	0	yadI	4	24.0833	0.659

1.6	0	AG1	4	24.0833	0.546
1.6	0	ptsI	5	0	0.159
1.6	0	clcB	5	0	0.163
1.6	0	ycaM	5	0	0.168
1.6	0	yadI	5	0	0.161
1.6	0	AG1	5	0	0.161
1.6	0	ptsI	5	0.35	0.157
1.6	0	clcB	5	0.35	0.159
1.6	0	ycaM	5	0.35	0.162
1.6	0	yadI	5	0.35	0.158
1.6	0	AG1	5	0.35	0.16
1.6	0	ptsI	5	0.6	0.158
1.6	0	clcB	5	0.6	0.161
1.6	0	ycaM	5	0.6	0.161
1.6	0	yadI	5	0.6	0.16
1.6	0	AG1	5	0.6	0.161
1.6	0	ptsI	5	0.85	0.158
1.6	0	clcB	5	0.85	0.16
1.6	0	ycaM	5	0.85	0.161
1.6	0	yadI	5	0.85	0.16
1.6	0	AG1	5	0.85	0.16
1.6	0	ptsI	5	1.1	0.16
1.6	0	clcB	5	1.1	0.16
1.6	0	ycaM	5	1.1	0.163
1.6	0	yadI	5	1.1	0.161
1.6	0	AG1	5	1.1	0.161
1.6	0	ptsI	5	1.35	0.162
1.6	0	clcB	5	1.35	0.162
1.6	0	ycaM	5	1.35	0.164
1.6	0	yadI	5	1.35	0.162
1.6	0	AG1	5	1.35	0.162
1.6	0	ptsI	5	1.6	0.164
1.6	0	clcB	5	1.6	0.164
1.6	0	ycaM	5	1.6	0.167
1.6	0	yadI	5	1.6	0.165
1.6	0	AG1	5	1.6	0.167
1.6	0	ptsI	5	1.85	0.174
1.6	0	clcB	5	1.85	0.169
1.6	0	ycaM	5	1.85	0.171
1.6	0	yadI	5	1.85	0.168
1.6	0	AG1	5	1.85	0.171
1.6	0	ptsI	5	2.1	0.168
1.6	0	clcB	5	2.1	0.172
1.6	0	ycaM	5	2.1	0.176
1.6	0	yadI	5	2.1	0.173
1.6	0	AG1	5	2.1	0.17
1.6	0	ptsI	5	2.35	0.17
1.6	0	clcB	5	2.35	0.176
1.6	0	ycaM	5	2.35	0.18
1.6	0	yadI	5	2.35	0.178
1.6	0	AG1	5	2.35	0.174
1.6	0	ptsI	5	2.6	0.172
1.6	0	clcB	5	2.6	0.178

1.6	0	ycaM	5	2.6	0.183
1.6	0	yadI	5	2.6	0.185
1.6	0	AG1	5	2.6	0.184
1.6	0	ptsI	5	2.85	0.176
1.6	0	clcB	5	2.85	0.186
1.6	0	ycaM	5	2.85	0.189
1.6	0	yadI	5	2.85	0.19
1.6	0	AG1	5	2.85	0.192
1.6	0	ptsI	5	3.1	0.184
1.6	0	clcB	5	3.1	0.198
1.6	0	ycaM	5	3.1	0.198
1.6	0	yadI	5	3.1	0.197
1.6	0	AG1	5	3.1	0.206
1.6	0	ptsI	5	3.35	0.186
1.6	0	clcB	5	3.35	0.207
1.6	0	ycaM	5	3.35	0.207
1.6	0	yadI	5	3.35	0.209
1.6	0	AG1	5	3.35	0.204
1.6	0	ptsI	5	3.6	0.193
1.6	0	clcB	5	3.6	0.214
1.6	0	ycaM	5	3.6	0.219
1.6	0	yadI	5	3.6	0.223
1.6	0	AG1	5	3.6	0.211
1.6	0	ptsI	5	3.85	0.206
1.6	0	clcB	5	3.85	0.225
1.6	0	ycaM	5	3.85	0.228
1.6	0	yadI	5	3.85	0.236
1.6	0	AG1	5	3.85	0.217
1.6	0	ptsI	5	4.38333	0.202
1.6	0	clcB	5	4.38333	0.254
1.6	0	ycaM	5	4.38333	0.236
1.6	0	yadI	5	4.38333	0.24
1.6	0	AG1	5	4.38333	0.232
1.6	0	ptsI	5	4.63333	0.198
1.6	0	clcB	5	4.63333	0.27
1.6	0	ycaM	5	4.63333	0.24
1.6	0	yadI	5	4.63333	0.231
1.6	0	AG1	5	4.63333	0.226
1.6	0	ptsI	5	4.88333	0.204
1.6	0	clcB	5	4.88333	0.282
1.6	0	ycaM	5	4.88333	0.251
1.6	0	yadI	5	4.88333	0.245
1.6	0	AG1	5	4.88333	0.229
1.6	0	ptsI	5	5.13333	0.211
1.6	0	clcB	5	5.13333	0.295
1.6	0	ycaM	5	5.13333	0.257
1.6	0	yadI	5	5.13333	0.258
1.6	0	AG1	5	5.13333	0.237
1.6	0	ptsI	5	5.38333	0.218
1.6	0	clcB	5	5.38333	0.312
1.6	0	ycaM	5	5.38333	0.267
1.6	0	yadI	5	5.38333	0.273
1.6	0	AG1	5	5.38333	0.242

1.6	0	ptsI	5	5.63333	0.226
1.6	0	clcB	5	5.63333	0.324
1.6	0	ycaM	5	5.63333	0.277
1.6	0	yadI	5	5.63333	0.288
1.6	0	AG1	5	5.63333	0.249
1.6	0	ptsI	5	5.88333	0.232
1.6	0	clcB	5	5.88333	0.339
1.6	0	ycaM	5	5.88333	0.288
1.6	0	yadI	5	5.88333	0.301
1.6	0	AG1	5	5.88333	0.259
1.6	0	ptsI	5	6.13333	0.241
1.6	0	clcB	5	6.13333	0.348
1.6	0	ycaM	5	6.13333	0.3
1.6	0	yadI	5	6.13333	0.316
1.6	0	AG1	5	6.13333	0.273
1.6	0	ptsI	5	6.38333	0.252
1.6	0	clcB	5	6.38333	0.36
1.6	0	ycaM	5	6.38333	0.31
1.6	0	yadI	5	6.38333	0.326
1.6	0	AG1	5	6.38333	0.278
1.6	0	ptsI	5	6.63333	0.261
1.6	0	clcB	5	6.63333	0.369
1.6	0	ycaM	5	6.63333	0.322
1.6	0	yadI	5	6.63333	0.335
1.6	0	AG1	5	6.63333	0.282
1.6	0	ptsI	5	6.88333	0.271
1.6	0	clcB	5	6.88333	0.379
1.6	0	ycaM	5	6.88333	0.332
1.6	0	yadI	5	6.88333	0.348
1.6	0	AG1	5	6.88333	0.288
1.6	0	ptsI	5	7.13333	0.281
1.6	0	clcB	5	7.13333	0.391
1.6	0	ycaM	5	7.13333	0.343
1.6	0	yadI	5	7.13333	0.364
1.6	0	AG1	5	7.13333	0.299
1.6	0	ptsI	5	7.38333	0.295
1.6	0	clcB	5	7.38333	0.402
1.6	0	ycaM	5	7.38333	0.356
1.6	0	yadI	5	7.38333	0.378
1.6	0	AG1	5	7.38333	0.312
1.6	0	ptsI	5	7.63333	0.307
1.6	0	clcB	5	7.63333	0.418
1.6	0	ycaM	5	7.63333	0.366
1.6	0	yadI	5	7.63333	0.39
1.6	0	AG1	5	7.63333	0.322
1.6	0	ptsI	5	7.88333	0.318
1.6	0	clcB	5	7.88333	0.43
1.6	0	ycaM	5	7.88333	0.375
1.6	0	yadI	5	7.88333	0.401
1.6	0	AG1	5	7.88333	0.326
1.6	0	ptsI	5	8.13333	0.329
1.6	0	clcB	5	8.13333	0.443
1.6	0	ycaM	5	8.13333	0.385

1.6	0	yadI	5	8.13333	0.414
1.6	0	AG1	5	8.13333	0.34
1.6	0	ptsI	5	8.38333	0.342
1.6	0	clcB	5	8.38333	0.458
1.6	0	ycaM	5	8.38333	0.395
1.6	0	yadI	5	8.38333	0.424
1.6	0	AG1	5	8.38333	0.35
1.6	0	ptsI	5	8.88333	0.32
1.6	0	clcB	5	8.88333	0.367
1.6	0	ycaM	5	8.88333	0.353
1.6	0	yadI	5	8.88333	0.427
1.6	0	AG1	5	8.88333	0.384
1.6	0	ptsI	5	9.13333	0.332
1.6	0	clcB	5	9.13333	0.373
1.6	0	ycaM	5	9.13333	0.358
1.6	0	yadI	5	9.13333	0.42
1.6	0	AG1	5	9.13333	0.389
1.6	0	ptsI	5	9.38333	0.345
1.6	0	clcB	5	9.38333	0.383
1.6	0	ycaM	5	9.38333	0.368
1.6	0	yadI	5	9.38333	0.428
1.6	0	AG1	5	9.38333	0.399
1.6	0	ptsI	5	9.63333	0.36
1.6	0	clcB	5	9.63333	0.398
1.6	0	ycaM	5	9.63333	0.379
1.6	0	yadI	5	9.63333	0.437
1.6	0	AG1	5	9.63333	0.418
1.6	0	ptsI	5	9.88333	0.369
1.6	0	clcB	5	9.88333	0.408
1.6	0	ycaM	5	9.88333	0.389
1.6	0	yadI	5	9.88333	0.443
1.6	0	AG1	5	9.88333	0.43
1.6	0	ptsI	5	10.1333	0.379
1.6	0	clcB	5	10.1333	0.414
1.6	0	ycaM	5	10.1333	0.394
1.6	0	yadI	5	10.1333	0.448
1.6	0	AG1	5	10.1333	0.435
1.6	0	ptsI	5	10.3833	0.39
1.6	0	clcB	5	10.3833	0.42
1.6	0	ycaM	5	10.3833	0.401
1.6	0	yadI	5	10.3833	0.458
1.6	0	AG1	5	10.3833	0.446
1.6	0	ptsI	5	10.6333	0.395
1.6	0	clcB	5	10.6333	0.424
1.6	0	ycaM	5	10.6333	0.401
1.6	0	yadI	5	10.6333	0.459
1.6	0	AG1	5	10.6333	0.452
1.6	0	ptsI	5	10.8833	0.402
1.6	0	clcB	5	10.8833	0.428
1.6	0	ycaM	5	10.8833	0.407
1.6	0	yadI	5	10.8833	0.466
1.6	0	AG1	5	10.8833	0.458
1.6	0	ptsI	5	11.1333	0.407

1.6	0	clcB	5	11.1333	0.431
1.6	0	ycaM	5	11.1333	0.406
1.6	0	yadI	5	11.1333	0.471
1.6	0	AG1	5	11.1333	0.459
1.6	0	ptsI	5	11.3833	0.413
1.6	0	clcB	5	11.3833	0.431
1.6	0	ycaM	5	11.3833	0.409
1.6	0	yadI	5	11.3833	0.475
1.6	0	AG1	5	11.3833	0.463
1.6	0	ptsI	5	11.6333	0.417
1.6	0	clcB	5	11.6333	0.433
1.6	0	ycaM	5	11.6333	0.412
1.6	0	yadI	5	11.6333	0.48
1.6	0	AG1	5	11.6333	0.468
1.6	0	ptsI	5	11.8833	0.424
1.6	0	clcB	5	11.8833	0.44
1.6	0	ycaM	5	11.8833	0.412
1.6	0	yadI	5	11.8833	0.484
1.6	0	AG1	5	11.8833	0.471
1.6	0	ptsI	5	12.1333	0.43
1.6	0	clcB	5	12.1333	0.442
1.6	0	ycaM	5	12.1333	0.416
1.6	0	yadI	5	12.1333	0.489
1.6	0	AG1	5	12.1333	0.474
1.6	0	ptsI	5	12.3833	0.432
1.6	0	clcB	5	12.3833	0.445
1.6	0	ycaM	5	12.3833	0.416
1.6	0	yadI	5	12.3833	0.492
1.6	0	AG1	5	12.3833	0.478
1.6	0	ptsI	5	12.6333	0.44
1.6	0	clcB	5	12.6333	0.448
1.6	0	ycaM	5	12.6333	0.42
1.6	0	yadI	5	12.6333	0.5
1.6	0	AG1	5	12.6333	0.483
1.6	0	ptsI	5	12.8833	0.441
1.6	0	clcB	5	12.8833	0.452
1.6	0	ycaM	5	12.8833	0.42
1.6	0	yadI	5	12.8833	0.504
1.6	0	AG1	5	12.8833	0.487
1.6	0	ptsI	5	13.1333	0.444
1.6	0	clcB	5	13.1333	0.454
1.6	0	ycaM	5	13.1333	0.424
1.6	0	yadI	5	13.1333	0.506
1.6	0	AG1	5	13.1333	0.491
1.6	0	ptsI	5	13.3833	0.447
1.6	0	clcB	5	13.3833	0.457
1.6	0	ycaM	5	13.3833	0.427
1.6	0	yadI	5	13.3833	0.514
1.6	0	AG1	5	13.3833	0.497
1.6	0	ptsI	5	13.6333	0.451
1.6	0	clcB	5	13.6333	0.462
1.6	0	ycaM	5	13.6333	0.43
1.6	0	yadI	5	13.6333	0.517

1.6	0	AG1	5	13.6333	0.5
1.6	0	ptsI	5	13.8833	0.456
1.6	0	clcB	5	13.8833	0.461
1.6	0	ycaM	5	13.8833	0.433
1.6	0	yadI	5	13.8833	0.518
1.6	0	AG1	5	13.8833	0.506
1.6	0	ptsI	5	14.1333	0.46
1.6	0	clcB	5	14.1333	0.467
1.6	0	ycaM	5	14.1333	0.436
1.6	0	yadI	5	14.1333	0.525
1.6	0	AG1	5	14.1333	0.509
1.6	0	ptsI	5	14.3833	0.464
1.6	0	clcB	5	14.3833	0.464
1.6	0	ycaM	5	14.3833	0.435
1.6	0	yadI	5	14.3833	0.527
1.6	0	AG1	5	14.3833	0.509
1.6	0	ptsI	5	14.6333	0.468
1.6	0	clcB	5	14.6333	0.468
1.6	0	ycaM	5	14.6333	0.439
1.6	0	yadI	5	14.6333	0.528
1.6	0	AG1	5	14.6333	0.517
1.6	0	ptsI	5	14.8833	0.468
1.6	0	clcB	5	14.8833	0.472
1.6	0	ycaM	5	14.8833	0.443
1.6	0	yadI	5	14.8833	0.534
1.6	0	AG1	5	14.8833	0.518
1.6	0	ptsI	5	15.1333	0.475
1.6	0	clcB	5	15.1333	0.473
1.6	0	ycaM	5	15.1333	0.444
1.6	0	yadI	5	15.1333	0.536
1.6	0	AG1	5	15.1333	0.524
1.6	0	ptsI	5	15.3833	0.482
1.6	0	clcB	5	15.3833	0.476
1.6	0	ycaM	5	15.3833	0.447
1.6	0	yadI	5	15.3833	0.537
1.6	0	AG1	5	15.3833	0.531
1.6	0	ptsI	5	15.6333	0.479
1.6	0	clcB	5	15.6333	0.479
1.6	0	ycaM	5	15.6333	0.45
1.6	0	yadI	5	15.6333	0.538
1.6	0	AG1	5	15.6333	0.532
1.6	0	ptsI	5	15.8833	0.486
1.6	0	clcB	5	15.8833	0.479
1.6	0	ycaM	5	15.8833	0.452
1.6	0	yadI	5	15.8833	0.545
1.6	0	AG1	5	15.8833	0.531
1.6	0	ptsI	5	16.1333	0.49
1.6	0	clcB	5	16.1333	0.48
1.6	0	ycaM	5	16.1333	0.453
1.6	0	yadI	5	16.1333	0.554
1.6	0	AG1	5	16.1333	0.521
1.6	0	ptsI	5	16.3833	0.495
1.6	0	clcB	5	16.3833	0.484

1.6	0	ycaM	5	16.3833	0.46
1.6	0	yadI	5	16.3833	0.558
1.6	0	AG1	5	16.3833	0.537
1.6	0	ptsI	5	16.6333	0.501
1.6	0	clcB	5	16.6333	0.485
1.6	0	ycaM	5	16.6333	0.462
1.6	0	yadI	5	16.6333	0.561
1.6	0	AG1	5	16.6333	0.539
1.6	0	ptsI	5	16.8833	0.501
1.6	0	clcB	5	16.8833	0.487
1.6	0	ycaM	5	16.8833	0.462
1.6	0	yadI	5	16.8833	0.562
1.6	0	AG1	5	16.8833	0.541
1.6	0	ptsI	5	17.1333	0.5
1.6	0	clcB	5	17.1333	0.489
1.6	0	ycaM	5	17.1333	0.461
1.6	0	yadI	5	17.1333	0.562
1.6	0	AG1	5	17.1333	0.544
1.6	0	ptsI	5	17.3833	0.507
1.6	0	clcB	5	17.3833	0.49
1.6	0	ycaM	5	17.3833	0.462
1.6	0	yadI	5	17.3833	0.57
1.6	0	AG1	5	17.3833	0.547
1.6	0	ptsI	5	17.6333	0.511
1.6	0	clcB	5	17.6333	0.493
1.6	0	ycaM	5	17.6333	0.466
1.6	0	yadI	5	17.6333	0.573
1.6	0	AG1	5	17.6333	0.551
1.6	0	ptsI	5	17.8833	0.512
1.6	0	clcB	5	17.8833	0.494
1.6	0	ycaM	5	17.8833	0.47
1.6	0	yadI	5	17.8833	0.577
1.6	0	AG1	5	17.8833	0.561
1.6	0	ptsI	5	18.1333	0.514
1.6	0	clcB	5	18.1333	0.492
1.6	0	ycaM	5	18.1333	0.467
1.6	0	yadI	5	18.1333	0.58
1.6	0	AG1	5	18.1333	0.573
1.6	0	ptsI	5	18.3833	0.517
1.6	0	clcB	5	18.3833	0.495
1.6	0	ycaM	5	18.3833	0.469
1.6	0	yadI	5	18.3833	0.584
1.6	0	AG1	5	18.3833	0.583
1.6	0	ptsI	5	18.6333	0.516
1.6	0	clcB	5	18.6333	0.493
1.6	0	ycaM	5	18.6333	0.47
1.6	0	yadI	5	18.6333	0.588
1.6	0	AG1	5	18.6333	0.589
1.6	0	ptsI	5	18.8833	0.521
1.6	0	clcB	5	18.8833	0.498
1.6	0	ycaM	5	18.8833	0.474
1.6	0	yadI	5	18.8833	0.592
1.6	0	AG1	5	18.8833	0.596

1.6	0	ptsI	5	19.1333	0.521
1.6	0	clcB	5	19.1333	0.495
1.6	0	ycaM	5	19.1333	0.472
1.6	0	yadI	5	19.1333	0.588
1.6	0	AG1	5	19.1333	0.603
1.6	0	ptsI	5	19.3833	0.523
1.6	0	clcB	5	19.3833	0.499
1.6	0	ycaM	5	19.3833	0.472
1.6	0	yadI	5	19.3833	0.59
1.6	0	AG1	5	19.3833	0.603
1.6	0	ptsI	5	19.6333	0.524
1.6	0	clcB	5	19.6333	0.498
1.6	0	ycaM	5	19.6333	0.474
1.6	0	yadI	5	19.6333	0.596
1.6	0	AG1	5	19.6333	0.604
1.6	0	ptsI	5	19.8833	0.529
1.6	0	clcB	5	19.8833	0.496
1.6	0	ycaM	5	19.8833	0.474
1.6	0	yadI	5	19.8833	0.591
1.6	0	AG1	5	19.8833	0.604
1.6	0	ptsI	5	20.1333	0.532
1.6	0	clcB	5	20.1333	0.501
1.6	0	ycaM	5	20.1333	0.474
1.6	0	yadI	5	20.1333	0.594
1.6	0	AG1	5	20.1333	0.607
1.6	0	ptsI	5	20.3833	0.533
1.6	0	clcB	5	20.3833	0.497
1.6	0	ycaM	5	20.3833	0.476
1.6	0	yadI	5	20.3833	0.594
1.6	0	AG1	5	20.3833	0.608
1.6	0	ptsI	5	20.6333	0.536
1.6	0	clcB	5	20.6333	0.502
1.6	0	ycaM	5	20.6333	0.477
1.6	0	yadI	5	20.6333	0.594
1.6	0	AG1	5	20.6333	0.619
1.6	0	ptsI	5	20.8833	0.536
1.6	0	clcB	5	20.8833	0.503
1.6	0	ycaM	5	20.8833	0.479
1.6	0	yadI	5	20.8833	0.597
1.6	0	AG1	5	20.8833	0.606
1.6	0	ptsI	5	21.1333	0.542
1.6	0	clcB	5	21.1333	0.499
1.6	0	ycaM	5	21.1333	0.481
1.6	0	yadI	5	21.1333	0.597
1.6	0	AG1	5	21.1333	0.606
1.6	0	ptsI	5	21.3833	0.539
1.6	0	clcB	5	21.3833	0.502
1.6	0	ycaM	5	21.3833	0.479
1.6	0	yadI	5	21.3833	0.592
1.6	0	AG1	5	21.3833	0.605
1.6	0	ptsI	5	21.6333	0.543
1.6	0	clcB	5	21.6333	0.502
1.6	0	ycaM	5	21.6333	0.481

1.6	0	yadI	5	21.6333	0.597
1.6	0	AG1	5	21.6333	0.607
1.6	0	ptsI	5	21.8833	0.542
1.6	0	clcB	5	21.8833	0.501
1.6	0	ycaM	5	21.8833	0.482
1.6	0	yadI	5	21.8833	0.594
1.6	0	AG1	5	21.8833	0.61
1.6	0	ptsI	5	22.1333	0.55
1.6	0	clcB	5	22.1333	0.503
1.6	0	ycaM	5	22.1333	0.483
1.6	0	yadI	5	22.1333	0.594
1.6	0	AG1	5	22.1333	0.608
1.6	0	ptsI	5	22.3833	0.55
1.6	0	clcB	5	22.3833	0.501
1.6	0	ycaM	5	22.3833	0.481
1.6	0	yadI	5	22.3833	0.596
1.6	0	AG1	5	22.3833	0.61
1.6	0	ptsI	5	22.6333	0.556
1.6	0	clcB	5	22.6333	0.504
1.6	0	ycaM	5	22.6333	0.484
1.6	0	yadI	5	22.6333	0.594
1.6	0	AG1	5	22.6333	0.613
1.6	0	ptsI	5	22.8833	0.556
1.6	0	clcB	5	22.8833	0.504
1.6	0	ycaM	5	22.8833	0.486
1.6	0	yadI	5	22.8833	0.597
1.6	0	AG1	5	22.8833	0.615
1.6	0	ptsI	5	23.1333	0.558
1.6	0	clcB	5	23.1333	0.504
1.6	0	ycaM	5	23.1333	0.488
1.6	0	yadI	5	23.1333	0.596
1.6	0	AG1	5	23.1333	0.614
1.6	0	ptsI	5	23.3833	0.559
1.6	0	clcB	5	23.3833	0.505
1.6	0	ycaM	5	23.3833	0.487
1.6	0	yadI	5	23.3833	0.598
1.6	0	AG1	5	23.3833	0.612
1.6	0	ptsI	5	23.6333	0.559
1.6	0	clcB	5	23.6333	0.506
1.6	0	ycaM	5	23.6333	0.487
1.6	0	yadI	5	23.6333	0.594
1.6	0	AG1	5	23.6333	0.612
1.6	0	ptsI	5	23.8833	0.563
1.6	0	clcB	5	23.8833	0.506
1.6	0	ycaM	5	23.8833	0.486
1.6	0	yadI	5	23.8833	0.595
1.6	0	AG1	5	23.8833	0.609
1.6	0	ptsI	5	24.1333	0.565
1.6	0	clcB	5	24.1333	0.505
1.6	0	ycaM	5	24.1333	0.488
1.6	0	yadI	5	24.1333	0.594
1.6	0	AG1	5	24.1333	0.614
1.6	0	ptsI	5	24.3833	0.566

1.6	0	clcB	5	24.3833	0.506
1.6	0	ycaM	5	24.3833	0.492
1.6	0	yadI	5	24.3833	0.596
1.6	0	AG1	5	24.3833	0.613
3.2	0	ptsI	1	0	0.219
3.2	0	clcB	1	0	0.22
3.2	0	ycaM	1	0	0.223
3.2	0	yadI	1	0	0.264
3.2	0	AG1	1	0	0.246
3.2	0	ptsI	1	0.25	0.221
3.2	0	clcB	1	0.25	0.215
3.2	0	ycaM	1	0.25	0.215
3.2	0	yadI	1	0.25	0.26
3.2	0	AG1	1	0.25	0.233
3.2	0	ptsI	1	0.5	0.224
3.2	0	clcB	1	0.5	0.214
3.2	0	ycaM	1	0.5	0.214
3.2	0	yadI	1	0.5	0.257
3.2	0	AG1	1	0.5	0.233
3.2	0	ptsI	1	0.75	0.222
3.2	0	clcB	1	0.75	0.214
3.2	0	ycaM	1	0.75	0.21
3.2	0	yadI	1	0.75	0.255
3.2	0	AG1	1	0.75	0.232
3.2	0	ptsI	1	1	0.225
3.2	0	clcB	1	1	0.216
3.2	0	ycaM	1	1	0.212
3.2	0	yadI	1	1	0.259
3.2	0	AG1	1	1	0.234
3.2	0	ptsI	1	1.25	0.229
3.2	0	clcB	1	1.25	0.216
3.2	0	ycaM	1	1.25	0.212
3.2	0	yadI	1	1.25	0.256
3.2	0	AG1	1	1.25	0.231
3.2	0	ptsI	1	1.5	0.235
3.2	0	clcB	1	1.5	0.217
3.2	0	ycaM	1	1.5	0.214
3.2	0	yadI	1	1.5	0.262
3.2	0	AG1	1	1.5	0.235
3.2	0	ptsI	1	1.75	0.242
3.2	0	clcB	1	1.75	0.217
3.2	0	ycaM	1	1.75	0.22
3.2	0	yadI	1	1.75	0.266
3.2	0	AG1	1	1.75	0.236
3.2	0	ptsI	1	2	0.245
3.2	0	clcB	1	2	0.22
3.2	0	ycaM	1	2	0.223
3.2	0	yadI	1	2	0.274
3.2	0	AG1	1	2	0.238
3.2	0	ptsI	1	2.25	0.255
3.2	0	clcB	1	2.25	0.224
3.2	0	ycaM	1	2.25	0.231
3.2	0	yadI	1	2.25	0.285

3.2	0	AG1	1	2.25	0.245
3.2	0	ptsI	1	2.5	0.259
3.2	0	clcB	1	2.5	0.228
3.2	0	ycaM	1	2.5	0.236
3.2	0	yadI	1	2.5	0.292
3.2	0	AG1	1	2.5	0.25
3.2	0	ptsI	1	2.75	0.269
3.2	0	clcB	1	2.75	0.235
3.2	0	ycaM	1	2.75	0.244
3.2	0	yadI	1	2.75	0.301
3.2	0	AG1	1	2.75	0.256
3.2	0	ptsI	1	3	0.28
3.2	0	clcB	1	3	0.241
3.2	0	ycaM	1	3	0.252
3.2	0	yadI	1	3	0.313
3.2	0	AG1	1	3	0.262
3.2	0	ptsI	1	3.25	0.29
3.2	0	clcB	1	3.25	0.247
3.2	0	ycaM	1	3.25	0.258
3.2	0	yadI	1	3.25	0.33
3.2	0	AG1	1	3.25	0.273
3.2	0	ptsI	1	3.5	0.296
3.2	0	clcB	1	3.5	0.251
3.2	0	ycaM	1	3.5	0.266
3.2	0	yadI	1	3.5	0.343
3.2	0	AG1	1	3.5	0.276
3.2	0	ptsI	1	3.75	0.307
3.2	0	clcB	1	3.75	0.253
3.2	0	ycaM	1	3.75	0.276
3.2	0	yadI	1	3.75	0.353
3.2	0	AG1	1	3.75	0.274
3.2	0	ptsI	1	4	0.313
3.2	0	clcB	1	4	0.267
3.2	0	ycaM	1	4	0.288
3.2	0	yadI	1	4	0.386
3.2	0	AG1	1	4	0.286
3.2	0	ptsI	1	4.25	0.318
3.2	0	clcB	1	4.25	0.266
3.2	0	ycaM	1	4.25	0.298
3.2	0	yadI	1	4.25	0.389
3.2	0	AG1	1	4.25	0.295
3.2	0	ptsI	1	4.5	0.323
3.2	0	clcB	1	4.5	0.271
3.2	0	ycaM	1	4.5	0.304
3.2	0	yadI	1	4.5	0.412
3.2	0	AG1	1	4.5	0.308
3.2	0	ptsI	1	4.75	0.332
3.2	0	clcB	1	4.75	0.273
3.2	0	ycaM	1	4.75	0.305
3.2	0	yadI	1	4.75	0.396
3.2	0	AG1	1	4.75	0.308
3.2	0	ptsI	1	5	0.34
3.2	0	clcB	1	5	0.286

3.2	0	ycaM	1	5	0.318
3.2	0	yadI	1	5	0.43
3.2	0	AG1	1	5	0.317
3.2	0	ptsI	1	5.25	0.351
3.2	0	clcB	1	5.25	0.292
3.2	0	ycaM	1	5.25	0.329
3.2	0	yadI	1	5.25	0.448
3.2	0	AG1	1	5.25	0.329
3.2	0	ptsI	1	5.5	0.356
3.2	0	clcB	1	5.5	0.301
3.2	0	ycaM	1	5.5	0.34
3.2	0	yadI	1	5.5	0.464
3.2	0	AG1	1	5.5	0.34
3.2	0	ptsI	1	5.75	0.362
3.2	0	clcB	1	5.75	0.307
3.2	0	ycaM	1	5.75	0.351
3.2	0	yadI	1	5.75	0.481
3.2	0	AG1	1	5.75	0.354
3.2	0	ptsI	1	6	0.378
3.2	0	clcB	1	6	0.313
3.2	0	ycaM	1	6	0.351
3.2	0	yadI	1	6	0.476
3.2	0	AG1	1	6	0.357
3.2	0	ptsI	1	6.25	0.394
3.2	0	clcB	1	6.25	0.322
3.2	0	ycaM	1	6.25	0.362
3.2	0	yadI	1	6.25	0.495
3.2	0	AG1	1	6.25	0.364
3.2	0	ptsI	1	6.5	0.402
3.2	0	clcB	1	6.5	0.327
3.2	0	ycaM	1	6.5	0.372
3.2	0	yadI	1	6.5	0.493
3.2	0	AG1	1	6.5	0.368
3.2	0	ptsI	1	6.75	0.414
3.2	0	clcB	1	6.75	0.344
3.2	0	ycaM	1	6.75	0.393
3.2	0	yadI	1	6.75	0.525
3.2	0	AG1	1	6.75	0.391
3.2	0	ptsI	1	7	0.417
3.2	0	clcB	1	7	0.35
3.2	0	ycaM	1	7	0.403
3.2	0	yadI	1	7	0.544
3.2	0	AG1	1	7	0.411
3.2	0	ptsI	1	7.25	0.422
3.2	0	clcB	1	7.25	0.356
3.2	0	ycaM	1	7.25	0.411
3.2	0	yadI	1	7.25	0.558
3.2	0	AG1	1	7.25	0.419
3.2	0	ptsI	1	7.5	0.429
3.2	0	clcB	1	7.5	0.366
3.2	0	ycaM	1	7.5	0.417
3.2	0	yadI	1	7.5	0.573
3.2	0	AG1	1	7.5	0.43

3.2	0	ptsI	1	7.75	0.439
3.2	0	clcB	1	7.75	0.374
3.2	0	ycaM	1	7.75	0.425
3.2	0	yadI	1	7.75	0.582
3.2	0	AG1	1	7.75	0.44
3.2	0	ptsI	1	8	0.451
3.2	0	clcB	1	8	0.382
3.2	0	ycaM	1	8	0.434
3.2	0	yadI	1	8	0.599
3.2	0	AG1	1	8	0.454
3.2	0	ptsI	1	8.25	0.459
3.2	0	clcB	1	8.25	0.391
3.2	0	ycaM	1	8.25	0.438
3.2	0	yadI	1	8.25	0.611
3.2	0	AG1	1	8.25	0.465
3.2	0	ptsI	1	8.5	0.469
3.2	0	clcB	1	8.5	0.396
3.2	0	ycaM	1	8.5	0.44
3.2	0	yadI	1	8.5	0.627
3.2	0	AG1	1	8.5	0.479
3.2	0	ptsI	1	8.75	0.481
3.2	0	clcB	1	8.75	0.403
3.2	0	ycaM	1	8.75	0.446
3.2	0	yadI	1	8.75	0.632
3.2	0	AG1	1	8.75	0.494
3.2	0	ptsI	1	9	0.493
3.2	0	clcB	1	9	0.416
3.2	0	ycaM	1	9	0.452
3.2	0	yadI	1	9	0.648
3.2	0	AG1	1	9	0.505
3.2	0	ptsI	1	9.25	0.516
3.2	0	clcB	1	9.25	0.424
3.2	0	ycaM	1	9.25	0.466
3.2	0	yadI	1	9.25	0.648
3.2	0	AG1	1	9.25	0.521
3.2	0	ptsI	1	9.5	0.552
3.2	0	clcB	1	9.5	0.448
3.2	0	ycaM	1	9.5	0.485
3.2	0	yadI	1	9.5	0.667
3.2	0	AG1	1	9.5	0.547
3.2	0	ptsI	1	9.75	0.545
3.2	0	clcB	1	9.75	0.45
3.2	0	ycaM	1	9.75	0.494
3.2	0	yadI	1	9.75	0.694
3.2	0	AG1	1	9.75	0.567
3.2	0	ptsI	1	10	0.504
3.2	0	clcB	1	10	0.46
3.2	0	ycaM	1	10	0.504
3.2	0	yadI	1	10	0.678
3.2	0	AG1	1	10	0.562
3.2	0	ptsI	1	10.25	0.526
3.2	0	clcB	1	10.25	0.466
3.2	0	ycaM	1	10.25	0.515

3.2	0	yadI	1	10.25	0.696
3.2	0	AG1	1	10.25	0.576
3.2	0	ptsI	1	10.5	0.526
3.2	0	clcB	1	10.5	0.465
3.2	0	ycaM	1	10.5	0.523
3.2	0	yadI	1	10.5	0.718
3.2	0	AG1	1	10.5	0.576
3.2	0	ptsI	1	10.75	0.515
3.2	0	clcB	1	10.75	0.488
3.2	0	ycaM	1	10.75	0.538
3.2	0	yadI	1	10.75	0.704
3.2	0	AG1	1	10.75	0.585
3.2	0	ptsI	1	11	0.519
3.2	0	clcB	1	11	0.505
3.2	0	ycaM	1	11	0.557
3.2	0	yadI	1	11	0.728
3.2	0	AG1	1	11	0.643
3.2	0	ptsI	1	11.25	0.55
3.2	0	clcB	1	11.25	0.517
3.2	0	ycaM	1	11.25	0.568
3.2	0	yadI	1	11.25	0.749
3.2	0	AG1	1	11.25	0.645
3.2	0	ptsI	1	11.5	0.564
3.2	0	clcB	1	11.5	0.533
3.2	0	ycaM	1	11.5	0.577
3.2	0	yadI	1	11.5	0.73
3.2	0	AG1	1	11.5	0.675
3.2	0	ptsI	1	11.75	0.585
3.2	0	clcB	1	11.75	0.555
3.2	0	ycaM	1	11.75	0.586
3.2	0	yadI	1	11.75	0.657
3.2	0	AG1	1	11.75	0.692
3.2	0	ptsI	1	12	0.609
3.2	0	clcB	1	12	0.554
3.2	0	ycaM	1	12	0.58
3.2	0	yadI	1	12	0.677
3.2	0	AG1	1	12	0.682
3.2	0	ptsI	1	12.25	0.639
3.2	0	clcB	1	12.25	0.565
3.2	0	ycaM	1	12.25	0.601
3.2	0	yadI	1	12.25	0.609
3.2	0	AG1	1	12.25	0.696
3.2	0	ptsI	1	12.5	0.633
3.2	0	clcB	1	12.5	0.573
3.2	0	ycaM	1	12.5	0.6
3.2	0	yadI	1	12.5	0.643
3.2	0	AG1	1	12.5	0.706
3.2	0	ptsI	1	12.75	0.654
3.2	0	clcB	1	12.75	0.564
3.2	0	ycaM	1	12.75	0.594
3.2	0	yadI	1	12.75	0.655
3.2	0	AG1	1	12.75	0.704
3.2	0	ptsI	1	13	0.665

3.2	0	clcB	1	13	0.568
3.2	0	ycaM	1	13	0.602
3.2	0	yadI	1	13	0.655
3.2	0	AG1	1	13	0.715
3.2	0	ptsI	1	13.25	0.721
3.2	0	clcB	1	13.25	0.576
3.2	0	ycaM	1	13.25	0.62
3.2	0	yadI	1	13.25	0.639
3.2	0	AG1	1	13.25	0.748
3.2	0	ptsI	1	13.5	0.724
3.2	0	clcB	1	13.5	0.574
3.2	0	ycaM	1	13.5	0.617
3.2	0	yadI	1	13.5	0.65
3.2	0	AG1	1	13.5	0.744
3.2	0	ptsI	1	13.75	0.757
3.2	0	clcB	1	13.75	0.578
3.2	0	ycaM	1	13.75	0.626
3.2	0	yadI	1	13.75	0.66
3.2	0	AG1	1	13.75	0.763
3.2	0	ptsI	1	14	0.782
3.2	0	clcB	1	14	0.579
3.2	0	ycaM	1	14	0.618
3.2	0	yadI	1	14	0.665
3.2	0	AG1	1	14	0.776
3.2	0	ptsI	1	14.25	0.798
3.2	0	clcB	1	14.25	0.583
3.2	0	ycaM	1	14.25	0.615
3.2	0	yadI	1	14.25	0.668
3.2	0	AG1	1	14.25	0.779
3.2	0	ptsI	1	14.5	0.807
3.2	0	clcB	1	14.5	0.581
3.2	0	ycaM	1	14.5	0.641
3.2	0	yadI	1	14.5	0.67
3.2	0	AG1	1	14.5	0.792
3.2	0	ptsI	1	14.75	0.844
3.2	0	clcB	1	14.75	0.596
3.2	0	ycaM	1	14.75	0.683
3.2	0	yadI	1	14.75	0.667
3.2	0	AG1	1	14.75	0.818
3.2	0	ptsI	1	15	0.824
3.2	0	clcB	1	15	0.599
3.2	0	ycaM	1	15	0.663
3.2	0	yadI	1	15	0.695
3.2	0	AG1	1	15	0.814
3.2	0	ptsI	1	15.25	0.826
3.2	0	clcB	1	15.25	0.606
3.2	0	ycaM	1	15.25	0.677
3.2	0	yadI	1	15.25	0.706
3.2	0	AG1	1	15.25	0.828
3.2	0	ptsI	1	15.5	0.814
3.2	0	clcB	1	15.5	0.629
3.2	0	ycaM	1	15.5	0.674
3.2	0	yadI	1	15.5	0.701

3.2	0	AG1	1	15.5	0.825
3.2	0	ptsI	1	15.75	0.74
3.2	0	clcB	1	15.75	0.654
3.2	0	ycaM	1	15.75	0.678
3.2	0	yadI	1	15.75	0.691
3.2	0	AG1	1	15.75	0.813
3.2	0	ptsI	1	16	0.707
3.2	0	clcB	1	16	0.678
3.2	0	ycaM	1	16	0.695
3.2	0	yadI	1	16	0.683
3.2	0	AG1	1	16	0.812
3.2	0	ptsI	1	16.25	0.722
3.2	0	clcB	1	16.25	0.672
3.2	0	ycaM	1	16.25	0.688
3.2	0	yadI	1	16.25	0.709
3.2	0	AG1	1	16.25	0.807
3.2	0	ptsI	1	16.5	0.751
3.2	0	clcB	1	16.5	0.675
3.2	0	ycaM	1	16.5	0.695
3.2	0	yadI	1	16.5	0.716
3.2	0	AG1	1	16.5	0.802
3.2	0	ptsI	1	17.0667	0.684
3.2	0	clcB	1	17.0667	0.677
3.2	0	ycaM	1	17.0667	0.734
3.2	0	yadI	1	17.0667	0.725
3.2	0	AG1	1	17.0667	0.84
3.2	0	ptsI	1	17.3167	0.722
3.2	0	clcB	1	17.3167	0.675
3.2	0	ycaM	1	17.3167	0.715
3.2	0	yadI	1	17.3167	0.716
3.2	0	AG1	1	17.3167	0.812
3.2	0	ptsI	1	17.5667	0.763
3.2	0	clcB	1	17.5667	0.678
3.2	0	ycaM	1	17.5667	0.711
3.2	0	yadI	1	17.5667	0.724
3.2	0	AG1	1	17.5667	0.796
3.2	0	ptsI	1	17.8167	0.778
3.2	0	clcB	1	17.8167	0.677
3.2	0	ycaM	1	17.8167	0.717
3.2	0	yadI	1	17.8167	0.726
3.2	0	AG1	1	17.8167	0.802
3.2	0	ptsI	1	18.0667	0.801
3.2	0	clcB	1	18.0667	0.678
3.2	0	ycaM	1	18.0667	0.715
3.2	0	yadI	1	18.0667	0.729
3.2	0	AG1	1	18.0667	0.802
3.2	0	ptsI	1	18.3167	0.795
3.2	0	clcB	1	18.3167	0.679
3.2	0	ycaM	1	18.3167	0.721
3.2	0	yadI	1	18.3167	0.721
3.2	0	AG1	1	18.3167	0.812
3.2	0	ptsI	1	18.5667	0.813
3.2	0	clcB	1	18.5667	0.69

3.2	0	ycaM	1	18.5667	0.729
3.2	0	yadI	1	18.5667	0.741
3.2	0	AG1	1	18.5667	0.811
3.2	0	ptsI	1	18.8167	0.825
3.2	0	clcB	1	18.8167	0.692
3.2	0	ycaM	1	18.8167	0.734
3.2	0	yadI	1	18.8167	0.737
3.2	0	AG1	1	18.8167	0.822
3.2	0	ptsI	1	19.0667	0.836
3.2	0	clcB	1	19.0667	0.695
3.2	0	ycaM	1	19.0667	0.747
3.2	0	yadI	1	19.0667	0.744
3.2	0	AG1	1	19.0667	0.831
3.2	0	ptsI	1	19.3167	0.84
3.2	0	clcB	1	19.3167	0.706
3.2	0	ycaM	1	19.3167	0.751
3.2	0	yadI	1	19.3167	0.747
3.2	0	AG1	1	19.3167	0.836
3.2	0	ptsI	1	19.5667	0.845
3.2	0	clcB	1	19.5667	0.71
3.2	0	ycaM	1	19.5667	0.756
3.2	0	yadI	1	19.5667	0.747
3.2	0	AG1	1	19.5667	0.842
3.2	0	ptsI	1	19.8167	0.846
3.2	0	clcB	1	19.8167	0.715
3.2	0	ycaM	1	19.8167	0.759
3.2	0	yadI	1	19.8167	0.744
3.2	0	AG1	1	19.8167	0.849
3.2	0	ptsI	1	20.0667	0.871
3.2	0	clcB	1	20.0667	0.723
3.2	0	ycaM	1	20.0667	0.768
3.2	0	yadI	1	20.0667	0.745
3.2	0	AG1	1	20.0667	0.856
3.2	0	ptsI	1	20.3167	0.881
3.2	0	clcB	1	20.3167	0.725
3.2	0	ycaM	1	20.3167	0.768
3.2	0	yadI	1	20.3167	0.744
3.2	0	AG1	1	20.3167	0.855
3.2	0	ptsI	1	20.5667	0.894
3.2	0	clcB	1	20.5667	0.734
3.2	0	ycaM	1	20.5667	0.78
3.2	0	yadI	1	20.5667	0.765
3.2	0	AG1	1	20.5667	0.861
3.2	0	ptsI	1	20.8167	0.906
3.2	0	clcB	1	20.8167	0.739
3.2	0	ycaM	1	20.8167	0.783
3.2	0	yadI	1	20.8167	0.773
3.2	0	AG1	1	20.8167	0.865
3.2	0	ptsI	1	21.0667	0.911
3.2	0	clcB	1	21.0667	0.748
3.2	0	ycaM	1	21.0667	0.79
3.2	0	yadI	1	21.0667	0.771
3.2	0	AG1	1	21.0667	0.879

3.2	0	ptsI	1	21.3167	0.918
3.2	0	clcB	1	21.3167	0.751
3.2	0	ycaM	1	21.3167	0.794
3.2	0	yadI	1	21.3167	0.769
3.2	0	AG1	1	21.3167	0.883
3.2	0	ptsI	1	21.5667	0.921
3.2	0	clcB	1	21.5667	0.754
3.2	0	ycaM	1	21.5667	0.797
3.2	0	yadI	1	21.5667	0.782
3.2	0	AG1	1	21.5667	0.887
3.2	0	ptsI	1	21.8167	0.926
3.2	0	clcB	1	21.8167	0.76
3.2	0	ycaM	1	21.8167	0.796
3.2	0	yadI	1	21.8167	0.773
3.2	0	AG1	1	21.8167	0.887
3.2	0	ptsI	1	22.0667	0.95
3.2	0	clcB	1	22.0667	0.766
3.2	0	ycaM	1	22.0667	0.796
3.2	0	yadI	1	22.0667	0.77
3.2	0	AG1	1	22.0667	0.894
3.2	0	ptsI	1	22.9333	0.946
3.2	0	clcB	1	22.9333	0.738
3.2	0	ycaM	1	22.9333	0.895
3.2	0	yadI	1	22.9333	0.879
3.2	0	AG1	1	22.9333	0.931
3.2	0	ptsI	1	23.1833	0.96
3.2	0	clcB	1	23.1833	0.716
3.2	0	ycaM	1	23.1833	0.863
3.2	0	yadI	1	23.1833	0.866
3.2	0	AG1	1	23.1833	0.867
3.2	0	ptsI	1	23.4333	0.952
3.2	0	clcB	1	23.4333	0.715
3.2	0	ycaM	1	23.4333	0.839
3.2	0	yadI	1	23.4333	0.878
3.2	0	AG1	1	23.4333	0.854
3.2	0	ptsI	1	23.6833	0.977
3.2	0	clcB	1	23.6833	0.718
3.2	0	ycaM	1	23.6833	0.824
3.2	0	yadI	1	23.6833	0.895
3.2	0	AG1	1	23.6833	0.86
3.2	0	ptsI	1	23.9333	0.986
3.2	0	clcB	1	23.9333	0.719
3.2	0	ycaM	1	23.9333	0.827
3.2	0	yadI	1	23.9333	0.899
3.2	0	AG1	1	23.9333	0.866
3.2	0	ptsI	1	24.1833	0.99
3.2	0	clcB	1	24.1833	0.715
3.2	0	ycaM	1	24.1833	0.828
3.2	0	yadI	1	24.1833	0.897
3.2	0	AG1	1	24.1833	0.876
3.2	0	ptsI	1	24.4333	0.958
3.2	0	clcB	1	24.4333	0.714
3.2	0	ycaM	1	24.4333	0.831

3.2	0	yadI	1	24.4333	0.894
3.2	0	AG1	1	24.4333	0.886
3.2	0	ptsI	1	24.6833	0.999
3.2	0	clcB	1	24.6833	0.715
3.2	0	ycaM	1	24.6833	0.827
3.2	0	yadI	1	24.6833	0.89
3.2	0	AG1	1	24.6833	0.886
3.2	0	ptsI	1	24.9333	0.968
3.2	0	clcB	1	24.9333	0.72
3.2	0	ycaM	1	24.9333	0.831
3.2	0	yadI	1	24.9333	0.896
3.2	0	AG1	1	24.9333	0.887
3.2	0	ptsI	1	25.1833	0.997
3.2	0	clcB	1	25.1833	0.723
3.2	0	ycaM	1	25.1833	0.836
3.2	0	yadI	1	25.1833	0.901
3.2	0	AG1	1	25.1833	0.889
3.2	0	ptsI	1	25.4333	0.995
3.2	0	clcB	1	25.4333	0.735
3.2	0	ycaM	1	25.4333	0.835
3.2	0	yadI	1	25.4333	0.895
3.2	0	AG1	1	25.4333	0.888
3.2	0	ptsI	1	25.6833	1.003
3.2	0	clcB	1	25.6833	0.73
3.2	0	ycaM	1	25.6833	0.836
3.2	0	yadI	1	25.6833	0.899
3.2	0	AG1	1	25.6833	0.895
3.2	0	ptsI	1	25.9333	1.013
3.2	0	clcB	1	25.9333	0.72
3.2	0	ycaM	1	25.9333	0.838
3.2	0	yadI	1	25.9333	0.893
3.2	0	AG1	1	25.9333	0.889
3.2	0	ptsI	1	26.1833	0.999
3.2	0	clcB	1	26.1833	0.737
3.2	0	ycaM	1	26.1833	0.85
3.2	0	yadI	1	26.1833	0.898
3.2	0	AG1	1	26.1833	0.897
3.2	0	ptsI	1	26.4333	1.02
3.2	0	clcB	1	26.4333	0.747
3.2	0	ycaM	1	26.4333	0.851
3.2	0	yadI	1	26.4333	0.905
3.2	0	AG1	1	26.4333	0.899
3.2	0	ptsI	1	26.6833	1.016
3.2	0	clcB	1	26.6833	0.741
3.2	0	ycaM	1	26.6833	0.857
3.2	0	yadI	1	26.6833	0.908
3.2	0	AG1	1	26.6833	0.898
3.2	0	ptsI	1	26.9333	1.041
3.2	0	clcB	1	26.9333	0.737
3.2	0	ycaM	1	26.9333	0.862
3.2	0	yadI	1	26.9333	0.908
3.2	0	AG1	1	26.9333	0.897
3.2	0	ptsI	1	27.1833	1.046

3.2	0	clcB	1	27.1833	0.741
3.2	0	ycaM	1	27.1833	0.868
3.2	0	yadI	1	27.1833	0.91
3.2	0	AG1	1	27.1833	0.898
3.2	0	ptsI	1	27.4333	1.054
3.2	0	clcB	1	27.4333	0.744
3.2	0	ycaM	1	27.4333	0.876
3.2	0	yadI	1	27.4333	0.929
3.2	0	AG1	1	27.4333	0.901
3.2	0	ptsI	1	27.6833	1.058
3.2	0	clcB	1	27.6833	0.747
3.2	0	ycaM	1	27.6833	0.881
3.2	0	yadI	1	27.6833	0.932
3.2	0	AG1	1	27.6833	0.901
3.2	0	ptsI	1	27.9333	1.058
3.2	0	clcB	1	27.9333	0.76
3.2	0	ycaM	1	27.9333	0.886
3.2	0	yadI	1	27.9333	0.94
3.2	0	AG1	1	27.9333	0.905
3.2	0	ptsI	1	28.1833	1.077
3.2	0	clcB	1	28.1833	0.749
3.2	0	ycaM	1	28.1833	0.893
3.2	0	yadI	1	28.1833	0.937
3.2	0	AG1	1	28.1833	0.899
3.2	0	ptsI	1	28.4333	1.089
3.2	0	clcB	1	28.4333	0.755
3.2	0	ycaM	1	28.4333	0.9
3.2	0	yadI	1	28.4333	0.946
3.2	0	AG1	1	28.4333	0.907
3.2	0	ptsI	1	28.6833	1.099
3.2	0	clcB	1	28.6833	0.757
3.2	0	ycaM	1	28.6833	0.909
3.2	0	yadI	1	28.6833	0.954
3.2	0	AG1	1	28.6833	0.906
3.2	0	ptsI	1	28.9333	1.104
3.2	0	clcB	1	28.9333	0.765
3.2	0	ycaM	1	28.9333	0.917
3.2	0	yadI	1	28.9333	0.956
3.2	0	AG1	1	28.9333	0.911
3.2	0	ptsI	1	29.1833	1.111
3.2	0	clcB	1	29.1833	0.768
3.2	0	ycaM	1	29.1833	0.928
3.2	0	yadI	1	29.1833	0.965
3.2	0	AG1	1	29.1833	0.911
3.2	0	ptsI	1	29.4333	1.111
3.2	0	clcB	1	29.4333	0.774
3.2	0	ycaM	1	29.4333	0.936
3.2	0	yadI	1	29.4333	0.968
3.2	0	AG1	1	29.4333	0.913
3.2	0	ptsI	1	29.6833	1.123
3.2	0	clcB	1	29.6833	0.779
3.2	0	ycaM	1	29.6833	0.944
3.2	0	yadI	1	29.6833	0.971

3.2	0	AG1	1	29.6833	0.917
3.2	0	ptsI	1	29.9333	1.13
3.2	0	clcB	1	29.9333	0.784
3.2	0	ycaM	1	29.9333	0.952
3.2	0	yadI	1	29.9333	0.984
3.2	0	AG1	1	29.9333	0.92
3.2	0	ptsI	1	30.1833	1.148
3.2	0	clcB	1	30.1833	0.79
3.2	0	ycaM	1	30.1833	0.963
3.2	0	yadI	1	30.1833	0.992
3.2	0	AG1	1	30.1833	0.927
3.2	0	ptsI	1	30.4333	1.164
3.2	0	clcB	1	30.4333	0.798
3.2	0	ycaM	1	30.4333	0.97
3.2	0	yadI	1	30.4333	0.995
3.2	0	AG1	1	30.4333	0.929
3.2	0	ptsI	1	30.6833	1.19
3.2	0	clcB	1	30.6833	0.803
3.2	0	ycaM	1	30.6833	0.979
3.2	0	yadI	1	30.6833	1.01
3.2	0	AG1	1	30.6833	0.936
3.2	0	ptsI	1	30.9333	1.178
3.2	0	clcB	1	30.9333	0.808
3.2	0	ycaM	1	30.9333	0.988
3.2	0	yadI	1	30.9333	1.012
3.2	0	AG1	1	30.9333	0.938
3.2	0	ptsI	1	31.1833	1.216
3.2	0	clcB	1	31.1833	0.813
3.2	0	ycaM	1	31.1833	0.997
3.2	0	yadI	1	31.1833	1.017
3.2	0	AG1	1	31.1833	0.942
3.2	0	ptsI	1	31.4333	1.211
3.2	0	clcB	1	31.4333	0.821
3.2	0	ycaM	1	31.4333	1.004
3.2	0	yadI	1	31.4333	1.022
3.2	0	AG1	1	31.4333	0.951
3.2	0	ptsI	1	31.6833	1.224
3.2	0	clcB	1	31.6833	0.828
3.2	0	ycaM	1	31.6833	1.012
3.2	0	yadI	1	31.6833	1.028
3.2	0	AG1	1	31.6833	0.952
3.2	0	ptsI	1	31.9333	1.217
3.2	0	clcB	1	31.9333	0.834
3.2	0	ycaM	1	31.9333	1.022
3.2	0	yadI	1	31.9333	1.033
3.2	0	AG1	1	31.9333	0.961
3.2	0	ptsI	1	32.1833	1.244
3.2	0	clcB	1	32.1833	0.839
3.2	0	ycaM	1	32.1833	1.026
3.2	0	yadI	1	32.1833	1.039
3.2	0	AG1	1	32.1833	0.968
3.2	0	ptsI	1	32.4333	1.273
3.2	0	clcB	1	32.4333	0.855

3.2	0	ycaM	1	32.4333	1.033
3.2	0	yadI	1	32.4333	1.051
3.2	0	AG1	1	32.4333	0.97
3.2	0	ptsI	1	32.6833	1.263
3.2	0	clcB	1	32.6833	0.848
3.2	0	ycaM	1	32.6833	1.044
3.2	0	yadI	1	32.6833	1.053
3.2	0	AG1	1	32.6833	0.976
3.2	0	ptsI	1	32.9333	1.289
3.2	0	clcB	1	32.9333	0.857
3.2	0	ycaM	1	32.9333	1.052
3.2	0	yadI	1	32.9333	1.064
3.2	0	AG1	1	32.9333	0.984
3.2	0	ptsI	1	33.1833	1.277
3.2	0	clcB	1	33.1833	0.871
3.2	0	ycaM	1	33.1833	1.06
3.2	0	yadI	1	33.1833	1.072
3.2	0	AG1	1	33.1833	0.993
3.2	0	ptsI	1	33.4333	1.282
3.2	0	clcB	1	33.4333	0.867
3.2	0	ycaM	1	33.4333	1.067
3.2	0	yadI	1	33.4333	1.074
3.2	0	AG1	1	33.4333	0.998
3.2	0	ptsI	1	33.6833	1.297
3.2	0	clcB	1	33.6833	0.882
3.2	0	ycaM	1	33.6833	1.08
3.2	0	yadI	1	33.6833	1.086
3.2	0	AG1	1	33.6833	1.007
3.2	0	ptsI	1	33.9333	1.313
3.2	0	clcB	1	33.9333	0.879
3.2	0	ycaM	1	33.9333	1.086
3.2	0	yadI	1	33.9333	1.091
3.2	0	AG1	1	33.9333	1.01
3.2	0	ptsI	1	34.1833	1.3
3.2	0	clcB	1	34.1833	0.892
3.2	0	ycaM	1	34.1833	1.098
3.2	0	yadI	1	34.1833	1.105
3.2	0	AG1	1	34.1833	1.016
3.2	0	ptsI	1	34.4333	1.33
3.2	0	clcB	1	34.4333	0.889
3.2	0	ycaM	1	34.4333	1.109
3.2	0	yadI	1	34.4333	1.117
3.2	0	AG1	1	34.4333	1.022
3.2	0	ptsI	1	34.6833	1.346
3.2	0	clcB	1	34.6833	0.893
3.2	0	ycaM	1	34.6833	1.116
3.2	0	yadI	1	34.6833	1.121
3.2	0	AG1	1	34.6833	1.031
3.2	0	ptsI	1	34.9333	1.343
3.2	0	clcB	1	34.9333	0.9
3.2	0	ycaM	1	34.9333	1.126
3.2	0	yadI	1	34.9333	1.131
3.2	0	AG1	1	34.9333	1.038

3.2	0	ptsI	1	35.1833	1.346
3.2	0	clcB	1	35.1833	0.903
3.2	0	ycaM	1	35.1833	1.132
3.2	0	yadI	1	35.1833	1.138
3.2	0	AG1	1	35.1833	1.043
3.2	0	ptsI	1	35.4333	1.352
3.2	0	clcB	1	35.4333	0.91
3.2	0	ycaM	1	35.4333	1.144
3.2	0	yadI	1	35.4333	1.147
3.2	0	AG1	1	35.4333	1.05
3.2	0	ptsI	1	35.6833	1.368
3.2	0	clcB	1	35.6833	0.914
3.2	0	ycaM	1	35.6833	1.149
3.2	0	yadI	1	35.6833	1.155
3.2	0	AG1	1	35.6833	1.055
3.2	0	ptsI	1	35.9333	1.368
3.2	0	clcB	1	35.9333	0.929
3.2	0	ycaM	1	35.9333	1.151
3.2	0	yadI	1	35.9333	1.169
3.2	0	AG1	1	35.9333	1.066
3.2	0	ptsI	1	36.1833	1.398
3.2	0	clcB	1	36.1833	0.921
3.2	0	ycaM	1	36.1833	1.152
3.2	0	yadI	1	36.1833	1.172
3.2	0	AG1	1	36.1833	1.075
3.2	0	ptsI	1	36.4333	1.388
3.2	0	clcB	1	36.4333	0.928
3.2	0	ycaM	1	36.4333	1.157
3.2	0	yadI	1	36.4333	1.185
3.2	0	AG1	1	36.4333	1.084
3.2	0	ptsI	1	36.6833	1.396
3.2	0	clcB	1	36.6833	0.934
3.2	0	ycaM	1	36.6833	1.163
3.2	0	yadI	1	36.6833	1.191
3.2	0	AG1	1	36.6833	1.088
3.2	0	ptsI	2	0	0.211
3.2	0	clcB	2	0	0.214
3.2	0	ycaM	2	0	0.216
3.2	0	yadI	2	0	0.215
3.2	0	AG1	2	0	0.238
3.2	0	ptsI	2	0.25	0.216
3.2	0	clcB	2	0.25	0.204
3.2	0	ycaM	2	0.25	0.213
3.2	0	yadI	2	0.25	0.212
3.2	0	AG1	2	0.25	0.235
3.2	0	ptsI	2	0.5	0.22
3.2	0	clcB	2	0.5	0.205
3.2	0	ycaM	2	0.5	0.215
3.2	0	yadI	2	0.5	0.212
3.2	0	AG1	2	0.5	0.238
3.2	0	ptsI	2	0.75	0.226
3.2	0	clcB	2	0.75	0.211
3.2	0	ycaM	2	0.75	0.215

3.2	0	yadI	2	0.75	0.216
3.2	0	AG1	2	0.75	0.24
3.2	0	ptsI	2	1	0.228
3.2	0	clcB	2	1	0.21
3.2	0	ycaM	2	1	0.22
3.2	0	yadI	2	1	0.224
3.2	0	AG1	2	1	0.247
3.2	0	ptsI	2	1.25	0.23
3.2	0	clcB	2	1.25	0.21
3.2	0	ycaM	2	1.25	0.222
3.2	0	yadI	2	1.25	0.231
3.2	0	AG1	2	1.25	0.25
3.2	0	ptsI	2	1.5	0.236
3.2	0	clcB	2	1.5	0.209
3.2	0	ycaM	2	1.5	0.227
3.2	0	yadI	2	1.5	0.236
3.2	0	AG1	2	1.5	0.255
3.2	0	ptsI	2	1.75	0.24
3.2	0	clcB	2	1.75	0.212
3.2	0	ycaM	2	1.75	0.233
3.2	0	yadI	2	1.75	0.243
3.2	0	AG1	2	1.75	0.264
3.2	0	ptsI	2	2	0.247
3.2	0	clcB	2	2	0.215
3.2	0	ycaM	2	2	0.24
3.2	0	yadI	2	2	0.252
3.2	0	AG1	2	2	0.277
3.2	0	ptsI	2	2.25	0.256
3.2	0	clcB	2	2.25	0.219
3.2	0	ycaM	2	2.25	0.244
3.2	0	yadI	2	2.25	0.263
3.2	0	AG1	2	2.25	0.289
3.2	0	ptsI	2	2.5	0.268
3.2	0	clcB	2	2.5	0.223
3.2	0	ycaM	2	2.5	0.252
3.2	0	yadI	2	2.5	0.278
3.2	0	AG1	2	2.5	0.302
3.2	0	ptsI	2	2.75	0.272
3.2	0	clcB	2	2.75	0.228
3.2	0	ycaM	2	2.75	0.257
3.2	0	yadI	2	2.75	0.289
3.2	0	AG1	2	2.75	0.294
3.2	0	ptsI	2	3	0.27
3.2	0	clcB	2	3	0.238
3.2	0	ycaM	2	3	0.261
3.2	0	yadI	2	3	0.304
3.2	0	AG1	2	3	0.294
3.2	0	ptsI	2	3.25	0.286
3.2	0	clcB	2	3.25	0.24
3.2	0	ycaM	2	3.25	0.272
3.2	0	yadI	2	3.25	0.326
3.2	0	AG1	2	3.25	0.311
3.2	0	ptsI	2	3.5	0.294

3.2	0	clcB	2	3.5	0.245
3.2	0	ycaM	2	3.5	0.277
3.2	0	yadI	2	3.5	0.342
3.2	0	AG1	2	3.5	0.318
3.2	0	ptsI	2	3.75	0.303
3.2	0	clcB	2	3.75	0.252
3.2	0	ycaM	2	3.75	0.287
3.2	0	yadI	2	3.75	0.363
3.2	0	AG1	2	3.75	0.329
3.2	0	ptsI	2	4	0.314
3.2	0	clcB	2	4	0.259
3.2	0	ycaM	2	4	0.292
3.2	0	yadI	2	4	0.381
3.2	0	AG1	2	4	0.339
3.2	0	ptsI	2	4.38333	0.325
3.2	0	clcB	2	4.38333	0.263
3.2	0	ycaM	2	4.38333	0.305
3.2	0	yadI	2	4.38333	0.383
3.2	0	AG1	2	4.38333	0.275
3.2	0	ptsI	2	4.63333	0.317
3.2	0	clcB	2	4.63333	0.255
3.2	0	ycaM	2	4.63333	0.289
3.2	0	yadI	2	4.63333	0.388
3.2	0	AG1	2	4.63333	0.283
3.2	0	ptsI	2	4.88333	0.324
3.2	0	clcB	2	4.88333	0.259
3.2	0	ycaM	2	4.88333	0.292
3.2	0	yadI	2	4.88333	0.402
3.2	0	AG1	2	4.88333	0.295
3.2	0	ptsI	2	5.13333	0.331
3.2	0	clcB	2	5.13333	0.266
3.2	0	ycaM	2	5.13333	0.298
3.2	0	yadI	2	5.13333	0.414
3.2	0	AG1	2	5.13333	0.303
3.2	0	ptsI	2	5.38333	0.339
3.2	0	clcB	2	5.38333	0.274
3.2	0	ycaM	2	5.38333	0.303
3.2	0	yadI	2	5.38333	0.426
3.2	0	AG1	2	5.38333	0.313
3.2	0	ptsI	2	5.63333	0.347
3.2	0	clcB	2	5.63333	0.28
3.2	0	ycaM	2	5.63333	0.314
3.2	0	yadI	2	5.63333	0.436
3.2	0	AG1	2	5.63333	0.321
3.2	0	ptsI	2	5.88333	0.354
3.2	0	clcB	2	5.88333	0.286
3.2	0	ycaM	2	5.88333	0.32
3.2	0	yadI	2	5.88333	0.443
3.2	0	AG1	2	5.88333	0.331
3.2	0	ptsI	2	6.13333	0.361
3.2	0	clcB	2	6.13333	0.294
3.2	0	ycaM	2	6.13333	0.332
3.2	0	yadI	2	6.13333	0.453

3.2	0	AG1	2	6.13333	0.339
3.2	0	ptsI	2	6.38333	0.372
3.2	0	clcB	2	6.38333	0.301
3.2	0	ycaM	2	6.38333	0.34
3.2	0	yadI	2	6.38333	0.467
3.2	0	AG1	2	6.38333	0.349
3.2	0	ptsI	2	6.63333	0.38
3.2	0	clcB	2	6.63333	0.307
3.2	0	ycaM	2	6.63333	0.348
3.2	0	yadI	2	6.63333	0.477
3.2	0	AG1	2	6.63333	0.357
3.2	0	ptsI	2	6.88333	0.388
3.2	0	clcB	2	6.88333	0.314
3.2	0	ycaM	2	6.88333	0.356
3.2	0	yadI	2	6.88333	0.487
3.2	0	AG1	2	6.88333	0.365
3.2	0	ptsI	2	7.13333	0.393
3.2	0	clcB	2	7.13333	0.322
3.2	0	ycaM	2	7.13333	0.364
3.2	0	yadI	2	7.13333	0.498
3.2	0	AG1	2	7.13333	0.375
3.2	0	ptsI	2	7.38333	0.402
3.2	0	clcB	2	7.38333	0.327
3.2	0	ycaM	2	7.38333	0.372
3.2	0	yadI	2	7.38333	0.512
3.2	0	AG1	2	7.38333	0.382
3.2	0	ptsI	2	7.63333	0.408
3.2	0	clcB	2	7.63333	0.336
3.2	0	ycaM	2	7.63333	0.379
3.2	0	yadI	2	7.63333	0.523
3.2	0	AG1	2	7.63333	0.388
3.2	0	ptsI	2	7.88333	0.419
3.2	0	clcB	2	7.88333	0.343
3.2	0	ycaM	2	7.88333	0.384
3.2	0	yadI	2	7.88333	0.534
3.2	0	AG1	2	7.88333	0.397
3.2	0	ptsI	2	8.13333	0.43
3.2	0	clcB	2	8.13333	0.351
3.2	0	ycaM	2	8.13333	0.388
3.2	0	yadI	2	8.13333	0.55
3.2	0	AG1	2	8.13333	0.404
3.2	0	ptsI	2	8.38333	0.437
3.2	0	clcB	2	8.38333	0.36
3.2	0	ycaM	2	8.38333	0.391
3.2	0	yadI	2	8.38333	0.561
3.2	0	AG1	2	8.38333	0.414
3.2	0	ptsI	2	8.91667	0.368
3.2	0	clcB	2	8.91667	0.319
3.2	0	ycaM	2	8.91667	0.388
3.2	0	yadI	2	8.91667	0.554
3.2	0	AG1	2	8.91667	0.475
3.2	0	ptsI	2	9.16667	0.378
3.2	0	clcB	2	9.16667	0.333

3.2	0	ycaM	2	9.16667	0.391
3.2	0	yadI	2	9.16667	0.574
3.2	0	AG1	2	9.16667	0.484
3.2	0	ptsI	2	9.41667	0.381
3.2	0	clcB	2	9.41667	0.34
3.2	0	ycaM	2	9.41667	0.393
3.2	0	yadI	2	9.41667	0.58
3.2	0	AG1	2	9.41667	0.484
3.2	0	ptsI	2	9.66667	0.389
3.2	0	clcB	2	9.66667	0.345
3.2	0	ycaM	2	9.66667	0.399
3.2	0	yadI	2	9.66667	0.586
3.2	0	AG1	2	9.66667	0.491
3.2	0	ptsI	2	9.91667	0.393
3.2	0	clcB	2	9.91667	0.352
3.2	0	ycaM	2	9.91667	0.397
3.2	0	yadI	2	9.91667	0.588
3.2	0	AG1	2	9.91667	0.498
3.2	0	ptsI	2	10.1667	0.402
3.2	0	clcB	2	10.1667	0.356
3.2	0	ycaM	2	10.1667	0.402
3.2	0	yadI	2	10.1667	0.592
3.2	0	AG1	2	10.1667	0.52
3.2	0	ptsI	2	10.4167	0.402
3.2	0	clcB	2	10.4167	0.36
3.2	0	ycaM	2	10.4167	0.418
3.2	0	yadI	2	10.4167	0.605
3.2	0	AG1	2	10.4167	0.526
3.2	0	ptsI	2	10.6667	0.409
3.2	0	clcB	2	10.6667	0.366
3.2	0	ycaM	2	10.6667	0.419
3.2	0	yadI	2	10.6667	0.61
3.2	0	AG1	2	10.6667	0.534
3.2	0	ptsI	2	10.9167	0.41
3.2	0	clcB	2	10.9167	0.37
3.2	0	ycaM	2	10.9167	0.426
3.2	0	yadI	2	10.9167	0.612
3.2	0	AG1	2	10.9167	0.54
3.2	0	ptsI	2	11.1667	0.409
3.2	0	clcB	2	11.1667	0.372
3.2	0	ycaM	2	11.1667	0.431
3.2	0	yadI	2	11.1667	0.619
3.2	0	AG1	2	11.1667	0.537
3.2	0	ptsI	2	11.4167	0.419
3.2	0	clcB	2	11.4167	0.377
3.2	0	ycaM	2	11.4167	0.429
3.2	0	yadI	2	11.4167	0.627
3.2	0	AG1	2	11.4167	0.542
3.2	0	ptsI	2	11.6667	0.431
3.2	0	clcB	2	11.6667	0.374
3.2	0	ycaM	2	11.6667	0.439
3.2	0	yadI	2	11.6667	0.634
3.2	0	AG1	2	11.6667	0.543

3.2	0	ptsI	2	11.9167	0.424
3.2	0	clcB	2	11.9167	0.38
3.2	0	ycaM	2	11.9167	0.447
3.2	0	yadI	2	11.9167	0.635
3.2	0	AG1	2	11.9167	0.543
3.2	0	ptsI	2	12.1667	0.434
3.2	0	clcB	2	12.1667	0.385
3.2	0	ycaM	2	12.1667	0.448
3.2	0	yadI	2	12.1667	0.645
3.2	0	AG1	2	12.1667	0.548
3.2	0	ptsI	2	12.4167	0.429
3.2	0	clcB	2	12.4167	0.385
3.2	0	ycaM	2	12.4167	0.445
3.2	0	yadI	2	12.4167	0.652
3.2	0	AG1	2	12.4167	0.553
3.2	0	ptsI	2	12.6667	0.432
3.2	0	clcB	2	12.6667	0.396
3.2	0	ycaM	2	12.6667	0.456
3.2	0	yadI	2	12.6667	0.656
3.2	0	AG1	2	12.6667	0.556
3.2	0	ptsI	2	12.9167	0.43
3.2	0	clcB	2	12.9167	0.396
3.2	0	ycaM	2	12.9167	0.458
3.2	0	yadI	2	12.9167	0.66
3.2	0	AG1	2	12.9167	0.562
3.2	0	ptsI	2	13.1667	0.429
3.2	0	clcB	2	13.1667	0.397
3.2	0	ycaM	2	13.1667	0.467
3.2	0	yadI	2	13.1667	0.667
3.2	0	AG1	2	13.1667	0.563
3.2	0	ptsI	2	13.4167	0.432
3.2	0	clcB	2	13.4167	0.401
3.2	0	ycaM	2	13.4167	0.469
3.2	0	yadI	2	13.4167	0.672
3.2	0	AG1	2	13.4167	0.564
3.2	0	ptsI	2	13.6667	0.436
3.2	0	clcB	2	13.6667	0.402
3.2	0	ycaM	2	13.6667	0.471
3.2	0	yadI	2	13.6667	0.68
3.2	0	AG1	2	13.6667	0.565
3.2	0	ptsI	2	13.9167	0.439
3.2	0	clcB	2	13.9167	0.408
3.2	0	ycaM	2	13.9167	0.476
3.2	0	yadI	2	13.9167	0.683
3.2	0	AG1	2	13.9167	0.57
3.2	0	ptsI	2	14.1667	0.437
3.2	0	clcB	2	14.1667	0.413
3.2	0	ycaM	2	14.1667	0.48
3.2	0	yadI	2	14.1667	0.691
3.2	0	AG1	2	14.1667	0.573
3.2	0	ptsI	2	14.4167	0.443
3.2	0	clcB	2	14.4167	0.416
3.2	0	ycaM	2	14.4167	0.478

3.2	0	yadI	2	14.4167	0.694
3.2	0	AG1	2	14.4167	0.582
3.2	0	ptsI	2	14.6667	0.446
3.2	0	clcB	2	14.6667	0.423
3.2	0	ycaM	2	14.6667	0.485
3.2	0	yadI	2	14.6667	0.704
3.2	0	AG1	2	14.6667	0.58
3.2	0	ptsI	2	14.9167	0.446
3.2	0	clcB	2	14.9167	0.426
3.2	0	ycaM	2	14.9167	0.484
3.2	0	yadI	2	14.9167	0.709
3.2	0	AG1	2	14.9167	0.582
3.2	0	ptsI	2	15.1667	0.45
3.2	0	clcB	2	15.1667	0.431
3.2	0	ycaM	2	15.1667	0.483
3.2	0	yadI	2	15.1667	0.701
3.2	0	AG1	2	15.1667	0.595
3.2	0	ptsI	2	15.4167	0.454
3.2	0	clcB	2	15.4167	0.427
3.2	0	ycaM	2	15.4167	0.488
3.2	0	yadI	2	15.4167	0.721
3.2	0	AG1	2	15.4167	0.591
3.2	0	ptsI	2	15.6667	0.454
3.2	0	clcB	2	15.6667	0.441
3.2	0	ycaM	2	15.6667	0.492
3.2	0	yadI	2	15.6667	0.724
3.2	0	AG1	2	15.6667	0.594
3.2	0	ptsI	2	15.9167	0.461
3.2	0	clcB	2	15.9167	0.444
3.2	0	ycaM	2	15.9167	0.493
3.2	0	yadI	2	15.9167	0.73
3.2	0	AG1	2	15.9167	0.59
3.2	0	ptsI	2	16.1667	0.465
3.2	0	clcB	2	16.1667	0.444
3.2	0	ycaM	2	16.1667	0.494
3.2	0	yadI	2	16.1667	0.734
3.2	0	AG1	2	16.1667	0.597
3.2	0	ptsI	2	16.4167	0.462
3.2	0	clcB	2	16.4167	0.452
3.2	0	ycaM	2	16.4167	0.497
3.2	0	yadI	2	16.4167	0.737
3.2	0	AG1	2	16.4167	0.604
3.2	0	ptsI	2	16.6667	0.467
3.2	0	clcB	2	16.6667	0.453
3.2	0	ycaM	2	16.6667	0.499
3.2	0	yadI	2	16.6667	0.746
3.2	0	AG1	2	16.6667	0.6
3.2	0	ptsI	2	16.9167	0.47
3.2	0	clcB	2	16.9167	0.455
3.2	0	ycaM	2	16.9167	0.502
3.2	0	yadI	2	16.9167	0.746
3.2	0	AG1	2	16.9167	0.603
3.2	0	ptsI	2	17.1667	0.478

3.2	0	clcB	2	17.1667	0.457
3.2	0	ycaM	2	17.1667	0.505
3.2	0	yadI	2	17.1667	0.749
3.2	0	AG1	2	17.1667	0.605
3.2	0	ptsI	2	17.4167	0.48
3.2	0	clcB	2	17.4167	0.464
3.2	0	ycaM	2	17.4167	0.507
3.2	0	yadI	2	17.4167	0.751
3.2	0	AG1	2	17.4167	0.609
3.2	0	ptsI	2	17.6667	0.484
3.2	0	clcB	2	17.6667	0.466
3.2	0	ycaM	2	17.6667	0.51
3.2	0	yadI	2	17.6667	0.757
3.2	0	AG1	2	17.6667	0.612
3.2	0	ptsI	2	17.9167	0.49
3.2	0	clcB	2	17.9167	0.475
3.2	0	ycaM	2	17.9167	0.51
3.2	0	yadI	2	17.9167	0.762
3.2	0	AG1	2	17.9167	0.618
3.2	0	ptsI	2	18.1667	0.494
3.2	0	clcB	2	18.1667	0.479
3.2	0	ycaM	2	18.1667	0.514
3.2	0	yadI	2	18.1667	0.767
3.2	0	AG1	2	18.1667	0.618
3.2	0	ptsI	2	18.4167	0.496
3.2	0	clcB	2	18.4167	0.474
3.2	0	ycaM	2	18.4167	0.514
3.2	0	yadI	2	18.4167	0.766
3.2	0	AG1	2	18.4167	0.619
3.2	0	ptsI	2	18.6667	0.501
3.2	0	clcB	2	18.6667	0.487
3.2	0	ycaM	2	18.6667	0.517
3.2	0	yadI	2	18.6667	0.769
3.2	0	AG1	2	18.6667	0.622
3.2	0	ptsI	2	18.9167	0.504
3.2	0	clcB	2	18.9167	0.487
3.2	0	ycaM	2	18.9167	0.517
3.2	0	yadI	2	18.9167	0.76
3.2	0	AG1	2	18.9167	0.608
3.2	0	ptsI	2	19.1667	0.509
3.2	0	clcB	2	19.1667	0.493
3.2	0	ycaM	2	19.1667	0.524
3.2	0	yadI	2	19.1667	0.772
3.2	0	AG1	2	19.1667	0.626
3.2	0	ptsI	2	19.4167	0.512
3.2	0	clcB	2	19.4167	0.496
3.2	0	ycaM	2	19.4167	0.526
3.2	0	yadI	2	19.4167	0.778
3.2	0	AG1	2	19.4167	0.628
3.2	0	ptsI	2	19.6667	0.518
3.2	0	clcB	2	19.6667	0.502
3.2	0	ycaM	2	19.6667	0.529
3.2	0	yadI	2	19.6667	0.776

3.2	0	AG1	2	19.6667	0.63
3.2	0	ptsI	2	19.9167	0.519
3.2	0	clcB	2	19.9167	0.509
3.2	0	ycaM	2	19.9167	0.531
3.2	0	yadI	2	19.9167	0.779
3.2	0	AG1	2	19.9167	0.63
3.2	0	ptsI	2	20.1667	0.526
3.2	0	clcB	2	20.1667	0.504
3.2	0	ycaM	2	20.1667	0.534
3.2	0	yadI	2	20.1667	0.784
3.2	0	AG1	2	20.1667	0.634
3.2	0	ptsI	2	20.4167	0.532
3.2	0	clcB	2	20.4167	0.517
3.2	0	ycaM	2	20.4167	0.533
3.2	0	yadI	2	20.4167	0.784
3.2	0	AG1	2	20.4167	0.634
3.2	0	ptsI	2	20.6667	0.535
3.2	0	clcB	2	20.6667	0.523
3.2	0	ycaM	2	20.6667	0.538
3.2	0	yadI	2	20.6667	0.785
3.2	0	AG1	2	20.6667	0.637
3.2	0	ptsI	2	20.9167	0.539
3.2	0	clcB	2	20.9167	0.525
3.2	0	ycaM	2	20.9167	0.539
3.2	0	yadI	2	20.9167	0.785
3.2	0	AG1	2	20.9167	0.641
3.2	0	ptsI	2	21.1667	0.542
3.2	0	clcB	2	21.1667	0.532
3.2	0	ycaM	2	21.1667	0.54
3.2	0	yadI	2	21.1667	0.785
3.2	0	AG1	2	21.1667	0.641
3.2	0	ptsI	2	21.4167	0.549
3.2	0	clcB	2	21.4167	0.536
3.2	0	ycaM	2	21.4167	0.538
3.2	0	yadI	2	21.4167	0.763
3.2	0	AG1	2	21.4167	0.64
3.2	0	ptsI	2	21.6667	0.553
3.2	0	clcB	2	21.6667	0.542
3.2	0	ycaM	2	21.6667	0.543
3.2	0	yadI	2	21.6667	0.79
3.2	0	AG1	2	21.6667	0.646
3.2	0	ptsI	2	21.9167	0.56
3.2	0	clcB	2	21.9167	0.549
3.2	0	ycaM	2	21.9167	0.546
3.2	0	yadI	2	21.9167	0.79
3.2	0	AG1	2	21.9167	0.651
3.2	0	ptsI	2	22.1667	0.563
3.2	0	clcB	2	22.1667	0.552
3.2	0	ycaM	2	22.1667	0.546
3.2	0	yadI	2	22.1667	0.791
3.2	0	AG1	2	22.1667	0.652
3.2	0	ptsI	2	22.4167	0.571
3.2	0	clcB	2	22.4167	0.556

3.2	0	ycaM	2	22.4167	0.548
3.2	0	yadI	2	22.4167	0.791
3.2	0	AG1	2	22.4167	0.65
3.2	0	ptsI	2	22.6667	0.574
3.2	0	clcB	2	22.6667	0.56
3.2	0	ycaM	2	22.6667	0.549
3.2	0	yadI	2	22.6667	0.794
3.2	0	AG1	2	22.6667	0.65
3.2	0	ptsI	2	22.9167	0.577
3.2	0	clcB	2	22.9167	0.559
3.2	0	ycaM	2	22.9167	0.548
3.2	0	yadI	2	22.9167	0.793
3.2	0	AG1	2	22.9167	0.652
3.2	0	ptsI	2	23.1667	0.58
3.2	0	clcB	2	23.1667	0.568
3.2	0	ycaM	2	23.1667	0.549
3.2	0	yadI	2	23.1667	0.792
3.2	0	AG1	2	23.1667	0.655
3.2	0	ptsI	2	23.4167	0.587
3.2	0	clcB	2	23.4167	0.575
3.2	0	ycaM	2	23.4167	0.548
3.2	0	yadI	2	23.4167	0.792
3.2	0	AG1	2	23.4167	0.656
3.2	0	ptsI	2	23.6667	0.587
3.2	0	clcB	2	23.6667	0.575
3.2	0	ycaM	2	23.6667	0.545
3.2	0	yadI	2	23.6667	0.791
3.2	0	AG1	2	23.6667	0.66
3.2	0	ptsI	2	23.9167	0.594
3.2	0	clcB	2	23.9167	0.586
3.2	0	ycaM	2	23.9167	0.548
3.2	0	yadI	2	23.9167	0.794
3.2	0	AG1	2	23.9167	0.659
3.2	0	ptsI	2	24.1667	0.592
3.2	0	clcB	2	24.1667	0.587
3.2	0	ycaM	2	24.1667	0.547
3.2	0	yadI	2	24.1667	0.794
3.2	0	AG1	2	24.1667	0.662
3.2	0	ptsI	2	24.4167	0.602
3.2	0	clcB	2	24.4167	0.59
3.2	0	ycaM	2	24.4167	0.544
3.2	0	yadI	2	24.4167	0.794
3.2	0	AG1	2	24.4167	0.666
3.2	0	ptsI	2	24.6667	0.605
3.2	0	clcB	2	24.6667	0.597
3.2	0	ycaM	2	24.6667	0.545
3.2	0	yadI	2	24.6667	0.795
3.2	0	AG1	2	24.6667	0.668
3.2	0	ptsI	2	24.9167	0.608
3.2	0	clcB	2	24.9167	0.599
3.2	0	ycaM	2	24.9167	0.547
3.2	0	yadI	2	24.9167	0.794
3.2	0	AG1	2	24.9167	0.667

3.2	0	ptsI	2	25.1667	0.611
3.2	0	clcB	2	25.1667	0.607
3.2	0	ycaM	2	25.1667	0.547
3.2	0	yadI	2	25.1667	0.792
3.2	0	AG1	2	25.1667	0.67
3.2	0	ptsI	2	25.4167	0.616
3.2	0	clcB	2	25.4167	0.614
3.2	0	ycaM	2	25.4167	0.546
3.2	0	yadI	2	25.4167	0.793
3.2	0	AG1	2	25.4167	0.676
3.2	0	ptsI	2	25.6667	0.619
3.2	0	clcB	2	25.6667	0.618
3.2	0	ycaM	2	25.6667	0.547
3.2	0	yadI	2	25.6667	0.79
3.2	0	AG1	2	25.6667	0.675
3.2	0	ptsI	2	25.9167	0.626
3.2	0	clcB	2	25.9167	0.63
3.2	0	ycaM	2	25.9167	0.546
3.2	0	yadI	2	25.9167	0.79
3.2	0	AG1	2	25.9167	0.678
3.2	0	ptsI	2	26.1667	0.626
3.2	0	clcB	2	26.1667	0.637
3.2	0	ycaM	2	26.1667	0.544
3.2	0	yadI	2	26.1667	0.768
3.2	0	AG1	2	26.1667	0.674
3.2	0	ptsI	2	26.4167	0.631
3.2	0	clcB	2	26.4167	0.642
3.2	0	ycaM	2	26.4167	0.546
3.2	0	yadI	2	26.4167	0.792
3.2	0	AG1	2	26.4167	0.684
3.2	0	ptsI	3	0	0.164
3.2	0	clcB	3	0	0.156
3.2	0	ycaM	3	0	0.161
3.2	0	yadI	3	0	0.16
3.2	0	AG1	3	0	0.18
3.2	0	ptsI	3	0.25	0.162
3.2	0	clcB	3	0.25	0.153
3.2	0	ycaM	3	0.25	0.16
3.2	0	yadI	3	0.25	0.158
3.2	0	AG1	3	0.25	0.178
3.2	0	ptsI	3	0.5	0.163
3.2	0	clcB	3	0.5	0.152
3.2	0	ycaM	3	0.5	0.16
3.2	0	yadI	3	0.5	0.158
3.2	0	AG1	3	0.5	0.178
3.2	0	ptsI	3	0.75	0.164
3.2	0	clcB	3	0.75	0.154
3.2	0	ycaM	3	0.75	0.159
3.2	0	yadI	3	0.75	0.159
3.2	0	AG1	3	0.75	0.177
3.2	0	ptsI	3	1	0.164
3.2	0	clcB	3	1	0.154
3.2	0	ycaM	3	1	0.16

3.2	0	yadI	3	1	0.162
3.2	0	AG1	3	1	0.18
3.2	0	ptsI	3	1.25	0.164
3.2	0	clcB	3	1.25	0.156
3.2	0	ycaM	3	1.25	0.162
3.2	0	yadI	3	1.25	0.163
3.2	0	AG1	3	1.25	0.181
3.2	0	ptsI	3	1.5	0.166
3.2	0	clcB	3	1.5	0.157
3.2	0	ycaM	3	1.5	0.163
3.2	0	yadI	3	1.5	0.164
3.2	0	AG1	3	1.5	0.181
3.2	0	ptsI	3	1.75	0.165
3.2	0	clcB	3	1.75	0.158
3.2	0	ycaM	3	1.75	0.162
3.2	0	yadI	3	1.75	0.163
3.2	0	AG1	3	1.75	0.179
3.2	0	ptsI	3	2	0.168
3.2	0	clcB	3	2	0.16
3.2	0	ycaM	3	2	0.165
3.2	0	yadI	3	2	0.167
3.2	0	AG1	3	2	0.183
3.2	0	ptsI	3	2.25	0.172
3.2	0	clcB	3	2.25	0.161
3.2	0	ycaM	3	2.25	0.167
3.2	0	yadI	3	2.25	0.169
3.2	0	AG1	3	2.25	0.187
3.2	0	ptsI	3	2.5	0.174
3.2	0	clcB	3	2.5	0.163
3.2	0	ycaM	3	2.5	0.168
3.2	0	yadI	3	2.5	0.172
3.2	0	AG1	3	2.5	0.189
3.2	0	ptsI	3	2.75	0.176
3.2	0	clcB	3	2.75	0.162
3.2	0	ycaM	3	2.75	0.175
3.2	0	yadI	3	2.75	0.178
3.2	0	AG1	3	2.75	0.197
3.2	0	ptsI	3	3	0.177
3.2	0	clcB	3	3	0.164
3.2	0	ycaM	3	3	0.174
3.2	0	yadI	3	3	0.178
3.2	0	AG1	3	3	0.194
3.2	0	ptsI	3	3.25	0.178
3.2	0	clcB	3	3.25	0.168
3.2	0	ycaM	3	3.25	0.177
3.2	0	yadI	3	3.25	0.182
3.2	0	AG1	3	3.25	0.195
3.2	0	ptsI	3	3.5	0.182
3.2	0	clcB	3	3.5	0.17
3.2	0	ycaM	3	3.5	0.18
3.2	0	yadI	3	3.5	0.192
3.2	0	AG1	3	3.5	0.201
3.2	0	ptsI	3	3.75	0.186

3.2	0	clcB	3	3.75	0.171
3.2	0	ycaM	3	3.75	0.185
3.2	0	yadI	3	3.75	0.202
3.2	0	AG1	3	3.75	0.208
3.2	0	ptsI	3	4	0.188
3.2	0	clcB	3	4	0.174
3.2	0	ycaM	3	4	0.19
3.2	0	yadI	3	4	0.214
3.2	0	AG1	3	4	0.215
3.2	0	ptsI	3	4.41667	0.195
3.2	0	clcB	3	4.41667	0.17
3.2	0	ycaM	3	4.41667	0.195
3.2	0	yadI	3	4.41667	0.193
3.2	0	AG1	3	4.41667	0.222
3.2	0	ptsI	3	4.66667	0.189
3.2	0	clcB	3	4.66667	0.17
3.2	0	ycaM	3	4.66667	0.193
3.2	0	yadI	3	4.66667	0.193
3.2	0	AG1	3	4.66667	0.217
3.2	0	ptsI	3	4.91667	0.189
3.2	0	clcB	3	4.91667	0.17
3.2	0	ycaM	3	4.91667	0.195
3.2	0	yadI	3	4.91667	0.203
3.2	0	AG1	3	4.91667	0.228
3.2	0	ptsI	3	5.16667	0.193
3.2	0	clcB	3	5.16667	0.174
3.2	0	ycaM	3	5.16667	0.2
3.2	0	yadI	3	5.16667	0.213
3.2	0	AG1	3	5.16667	0.236
3.2	0	ptsI	3	5.41667	0.198
3.2	0	clcB	3	5.41667	0.176
3.2	0	ycaM	3	5.41667	0.207
3.2	0	yadI	3	5.41667	0.227
3.2	0	AG1	3	5.41667	0.246
3.2	0	ptsI	3	5.66667	0.204
3.2	0	clcB	3	5.66667	0.18
3.2	0	ycaM	3	5.66667	0.215
3.2	0	yadI	3	5.66667	0.24
3.2	0	AG1	3	5.66667	0.259
3.2	0	ptsI	3	5.91667	0.21
3.2	0	clcB	3	5.91667	0.185
3.2	0	ycaM	3	5.91667	0.226
3.2	0	yadI	3	5.91667	0.255
3.2	0	AG1	3	5.91667	0.27
3.2	0	ptsI	3	6.16667	0.217
3.2	0	clcB	3	6.16667	0.19
3.2	0	ycaM	3	6.16667	0.236
3.2	0	yadI	3	6.16667	0.266
3.2	0	AG1	3	6.16667	0.281
3.2	0	ptsI	3	6.41667	0.224
3.2	0	clcB	3	6.41667	0.195
3.2	0	ycaM	3	6.41667	0.25
3.2	0	yadI	3	6.41667	0.278

3.2	0	AG1	3	6.41667	0.288
3.2	0	ptsI	3	6.66667	0.234
3.2	0	clcB	3	6.66667	0.202
3.2	0	ycaM	3	6.66667	0.262
3.2	0	yadI	3	6.66667	0.292
3.2	0	AG1	3	6.66667	0.298
3.2	0	ptsI	3	6.91667	0.243
3.2	0	clcB	3	6.91667	0.208
3.2	0	ycaM	3	6.91667	0.272
3.2	0	yadI	3	6.91667	0.302
3.2	0	AG1	3	6.91667	0.307
3.2	0	ptsI	3	7.16667	0.253
3.2	0	clcB	3	7.16667	0.217
3.2	0	ycaM	3	7.16667	0.281
3.2	0	yadI	3	7.16667	0.316
3.2	0	AG1	3	7.16667	0.315
3.2	0	ptsI	3	7.41667	0.262
3.2	0	clcB	3	7.41667	0.226
3.2	0	ycaM	3	7.41667	0.288
3.2	0	yadI	3	7.41667	0.329
3.2	0	AG1	3	7.41667	0.325
3.2	0	ptsI	3	7.66667	0.271
3.2	0	clcB	3	7.66667	0.235
3.2	0	ycaM	3	7.66667	0.297
3.2	0	yadI	3	7.66667	0.341
3.2	0	AG1	3	7.66667	0.332
3.2	0	ptsI	3	7.91667	0.278
3.2	0	clcB	3	7.91667	0.244
3.2	0	ycaM	3	7.91667	0.305
3.2	0	yadI	3	7.91667	0.352
3.2	0	AG1	3	7.91667	0.34
3.2	0	ptsI	3	8.16667	0.286
3.2	0	clcB	3	8.16667	0.251
3.2	0	ycaM	3	8.16667	0.314
3.2	0	yadI	3	8.16667	0.361
3.2	0	AG1	3	8.16667	0.35
3.2	0	ptsI	3	8.41667	0.293
3.2	0	clcB	3	8.41667	0.259
3.2	0	ycaM	3	8.41667	0.325
3.2	0	yadI	3	8.41667	0.372
3.2	0	AG1	3	8.41667	0.36
3.2	0	ptsI	3	9.05	0.283
3.2	0	clcB	3	9.05	0.266
3.2	0	ycaM	3	9.05	0.313
3.2	0	yadI	3	9.05	0.396
3.2	0	AG1	3	9.05	0.374
3.2	0	ptsI	3	9.3	0.286
3.2	0	clcB	3	9.3	0.265
3.2	0	ycaM	3	9.3	0.315
3.2	0	yadI	3	9.3	0.415
3.2	0	AG1	3	9.3	0.383
3.2	0	ptsI	3	9.55	0.286
3.2	0	clcB	3	9.55	0.263

3.2	0	ycaM	3	9.55	0.311
3.2	0	yadI	3	9.55	0.423
3.2	0	AG1	3	9.55	0.389
3.2	0	ptsI	3	9.8	0.285
3.2	0	clcB	3	9.8	0.26
3.2	0	ycaM	3	9.8	0.309
3.2	0	yadI	3	9.8	0.43
3.2	0	AG1	3	9.8	0.389
3.2	0	ptsI	3	10.05	0.279
3.2	0	clcB	3	10.05	0.253
3.2	0	ycaM	3	10.05	0.304
3.2	0	yadI	3	10.05	0.434
3.2	0	AG1	3	10.05	0.391
3.2	0	ptsI	3	10.3	0.278
3.2	0	clcB	3	10.3	0.251
3.2	0	ycaM	3	10.3	0.304
3.2	0	yadI	3	10.3	0.438
3.2	0	AG1	3	10.3	0.393
3.2	0	ptsI	3	10.55	0.269
3.2	0	clcB	3	10.55	0.248
3.2	0	ycaM	3	10.55	0.306
3.2	0	yadI	3	10.55	0.447
3.2	0	AG1	3	10.55	0.393
3.2	0	ptsI	3	10.8	0.264
3.2	0	clcB	3	10.8	0.242
3.2	0	ycaM	3	10.8	0.303
3.2	0	yadI	3	10.8	0.449
3.2	0	AG1	3	10.8	0.4
3.2	0	ptsI	3	11.05	0.261
3.2	0	clcB	3	11.05	0.242
3.2	0	ycaM	3	11.05	0.3
3.2	0	yadI	3	11.05	0.456
3.2	0	AG1	3	11.05	0.402
3.2	0	ptsI	3	11.3	0.259
3.2	0	clcB	3	11.3	0.237
3.2	0	ycaM	3	11.3	0.298
3.2	0	yadI	3	11.3	0.457
3.2	0	AG1	3	11.3	0.402
3.2	0	ptsI	3	11.55	0.254
3.2	0	clcB	3	11.55	0.237
3.2	0	ycaM	3	11.55	0.298
3.2	0	yadI	3	11.55	0.465
3.2	0	AG1	3	11.55	0.398
3.2	0	ptsI	3	11.8	0.253
3.2	0	clcB	3	11.8	0.234
3.2	0	ycaM	3	11.8	0.297
3.2	0	yadI	3	11.8	0.474
3.2	0	AG1	3	11.8	0.403
3.2	0	ptsI	3	12.05	0.249
3.2	0	clcB	3	12.05	0.234
3.2	0	ycaM	3	12.05	0.298
3.2	0	yadI	3	12.05	0.476
3.2	0	AG1	3	12.05	0.4

3.2	0	ptsI	3	12.3	0.247
3.2	0	clcB	3	12.3	0.23
3.2	0	ycaM	3	12.3	0.294
3.2	0	yadI	3	12.3	0.48
3.2	0	AG1	3	12.3	0.401
3.2	0	ptsI	3	12.55	0.243
3.2	0	clcB	3	12.55	0.229
3.2	0	ycaM	3	12.55	0.294
3.2	0	yadI	3	12.55	0.481
3.2	0	AG1	3	12.55	0.408
3.2	0	ptsI	3	12.8	0.243
3.2	0	clcB	3	12.8	0.226
3.2	0	ycaM	3	12.8	0.29
3.2	0	yadI	3	12.8	0.486
3.2	0	AG1	3	12.8	0.409
3.2	0	ptsI	3	13.05	0.242
3.2	0	clcB	3	13.05	0.226
3.2	0	ycaM	3	13.05	0.291
3.2	0	yadI	3	13.05	0.496
3.2	0	AG1	3	13.05	0.412
3.2	0	ptsI	3	13.3	0.241
3.2	0	clcB	3	13.3	0.224
3.2	0	ycaM	3	13.3	0.289
3.2	0	yadI	3	13.3	0.5
3.2	0	AG1	3	13.3	0.414
3.2	0	ptsI	3	13.55	0.238
3.2	0	clcB	3	13.55	0.223
3.2	0	ycaM	3	13.55	0.292
3.2	0	yadI	3	13.55	0.503
3.2	0	AG1	3	13.55	0.412
3.2	0	ptsI	3	13.8	0.239
3.2	0	clcB	3	13.8	0.221
3.2	0	ycaM	3	13.8	0.287
3.2	0	yadI	3	13.8	0.509
3.2	0	AG1	3	13.8	0.414
3.2	0	ptsI	3	14.05	0.237
3.2	0	clcB	3	14.05	0.221
3.2	0	ycaM	3	14.05	0.287
3.2	0	yadI	3	14.05	0.514
3.2	0	AG1	3	14.05	0.412
3.2	0	ptsI	3	14.3	0.237
3.2	0	clcB	3	14.3	0.222
3.2	0	ycaM	3	14.3	0.288
3.2	0	yadI	3	14.3	0.52
3.2	0	AG1	3	14.3	0.421
3.2	0	ptsI	3	14.55	0.235
3.2	0	clcB	3	14.55	0.221
3.2	0	ycaM	3	14.55	0.287
3.2	0	yadI	3	14.55	0.525
3.2	0	AG1	3	14.55	0.427
3.2	0	ptsI	3	14.8	0.235
3.2	0	clcB	3	14.8	0.22
3.2	0	ycaM	3	14.8	0.286

3.2	0	yadI	3	14.8	0.536
3.2	0	AG1	3	14.8	0.431
3.2	0	ptsI	3	15.05	0.234
3.2	0	clcB	3	15.05	0.22
3.2	0	ycaM	3	15.05	0.284
3.2	0	yadI	3	15.05	0.538
3.2	0	AG1	3	15.05	0.434
3.2	0	ptsI	3	15.3	0.234
3.2	0	clcB	3	15.3	0.22
3.2	0	ycaM	3	15.3	0.285
3.2	0	yadI	3	15.3	0.545
3.2	0	AG1	3	15.3	0.437
3.2	0	ptsI	3	15.55	0.232
3.2	0	clcB	3	15.55	0.221
3.2	0	ycaM	3	15.55	0.286
3.2	0	yadI	3	15.55	0.54
3.2	0	AG1	3	15.55	0.44
3.2	0	ptsI	3	15.8	0.232
3.2	0	clcB	3	15.8	0.235
3.2	0	ycaM	3	15.8	0.284
3.2	0	yadI	3	15.8	0.545
3.2	0	AG1	3	15.8	0.441
3.2	0	ptsI	3	16.05	0.232
3.2	0	clcB	3	16.05	0.218
3.2	0	ycaM	3	16.05	0.282
3.2	0	yadI	3	16.05	0.555
3.2	0	AG1	3	16.05	0.446
3.2	0	ptsI	3	16.3	0.229
3.2	0	clcB	3	16.3	0.215
3.2	0	ycaM	3	16.3	0.282
3.2	0	yadI	3	16.3	0.552
3.2	0	AG1	3	16.3	0.446
3.2	0	ptsI	3	16.55	0.23
3.2	0	clcB	3	16.55	0.216
3.2	0	ycaM	3	16.55	0.281
3.2	0	yadI	3	16.55	0.557
3.2	0	AG1	3	16.55	0.452
3.2	0	ptsI	3	16.8	0.229
3.2	0	clcB	3	16.8	0.215
3.2	0	ycaM	3	16.8	0.281
3.2	0	yadI	3	16.8	0.556
3.2	0	AG1	3	16.8	0.456
3.2	0	ptsI	3	17.05	0.229
3.2	0	clcB	3	17.05	0.217
3.2	0	ycaM	3	17.05	0.281
3.2	0	yadI	3	17.05	0.564
3.2	0	AG1	3	17.05	0.462
3.2	0	ptsI	3	17.3	0.23
3.2	0	clcB	3	17.3	0.219
3.2	0	ycaM	3	17.3	0.281
3.2	0	yadI	3	17.3	0.568
3.2	0	AG1	3	17.3	0.466
3.2	0	ptsI	3	17.55	0.229

3.2	0	clcB	3	17.55	0.218
3.2	0	ycaM	3	17.55	0.282
3.2	0	yadI	3	17.55	0.572
3.2	0	AG1	3	17.55	0.466
3.2	0	ptsI	3	17.8	0.229
3.2	0	clcB	3	17.8	0.218
3.2	0	ycaM	3	17.8	0.282
3.2	0	yadI	3	17.8	0.578
3.2	0	AG1	3	17.8	0.474
3.2	0	ptsI	3	18.05	0.229
3.2	0	clcB	3	18.05	0.219
3.2	0	ycaM	3	18.05	0.284
3.2	0	yadI	3	18.05	0.588
3.2	0	AG1	3	18.05	0.48
3.2	0	ptsI	3	18.3	0.23
3.2	0	clcB	3	18.3	0.219
3.2	0	ycaM	3	18.3	0.283
3.2	0	yadI	3	18.3	0.593
3.2	0	AG1	3	18.3	0.485
3.2	0	ptsI	3	18.55	0.229
3.2	0	clcB	3	18.55	0.22
3.2	0	ycaM	3	18.55	0.286
3.2	0	yadI	3	18.55	0.598
3.2	0	AG1	3	18.55	0.487
3.2	0	ptsI	3	18.8	0.23
3.2	0	clcB	3	18.8	0.221
3.2	0	ycaM	3	18.8	0.285
3.2	0	yadI	3	18.8	0.601
3.2	0	AG1	3	18.8	0.488
3.2	0	ptsI	3	19.05	0.231
3.2	0	clcB	3	19.05	0.22
3.2	0	ycaM	3	19.05	0.286
3.2	0	yadI	3	19.05	0.612
3.2	0	AG1	3	19.05	0.494
3.2	0	ptsI	3	19.3	0.232
3.2	0	clcB	3	19.3	0.223
3.2	0	ycaM	3	19.3	0.287
3.2	0	yadI	3	19.3	0.615
3.2	0	AG1	3	19.3	0.496
3.2	0	ptsI	3	19.55	0.231
3.2	0	clcB	3	19.55	0.225
3.2	0	ycaM	3	19.55	0.29
3.2	0	yadI	3	19.55	0.617
3.2	0	AG1	3	19.55	0.499
3.2	0	ptsI	3	19.8	0.232
3.2	0	clcB	3	19.8	0.228
3.2	0	ycaM	3	19.8	0.29
3.2	0	yadI	3	19.8	0.622
3.2	0	AG1	3	19.8	0.503
3.2	0	ptsI	3	20.05	0.234
3.2	0	clcB	3	20.05	0.232
3.2	0	ycaM	3	20.05	0.293
3.2	0	yadI	3	20.05	0.628

3.2	0	AG1	3	20.05	0.51
3.2	0	ptsI	3	20.3	0.235
3.2	0	clcB	3	20.3	0.239
3.2	0	ycaM	3	20.3	0.294
3.2	0	yadI	3	20.3	0.634
3.2	0	AG1	3	20.3	0.511
3.2	0	ptsI	3	20.55	0.236
3.2	0	clcB	3	20.55	0.243
3.2	0	ycaM	3	20.55	0.296
3.2	0	yadI	3	20.55	0.639
3.2	0	AG1	3	20.55	0.514
3.2	0	ptsI	3	20.8	0.237
3.2	0	clcB	3	20.8	0.249
3.2	0	ycaM	3	20.8	0.299
3.2	0	yadI	3	20.8	0.642
3.2	0	AG1	3	20.8	0.52
3.2	0	ptsI	3	21.05	0.238
3.2	0	clcB	3	21.05	0.253
3.2	0	ycaM	3	21.05	0.302
3.2	0	yadI	3	21.05	0.643
3.2	0	AG1	3	21.05	0.52
3.2	0	ptsI	3	21.3	0.239
3.2	0	clcB	3	21.3	0.259
3.2	0	ycaM	3	21.3	0.303
3.2	0	yadI	3	21.3	0.646
3.2	0	AG1	3	21.3	0.525
3.2	0	ptsI	3	21.55	0.24
3.2	0	clcB	3	21.55	0.268
3.2	0	ycaM	3	21.55	0.326
3.2	0	yadI	3	21.55	0.652
3.2	0	AG1	3	21.55	0.529
3.2	0	ptsI	3	21.8	0.242
3.2	0	clcB	3	21.8	0.275
3.2	0	ycaM	3	21.8	0.319
3.2	0	yadI	3	21.8	0.651
3.2	0	AG1	3	21.8	0.533
3.2	0	ptsI	3	22.05	0.242
3.2	0	clcB	3	22.05	0.282
3.2	0	ycaM	3	22.05	0.32
3.2	0	yadI	3	22.05	0.652
3.2	0	AG1	3	22.05	0.534
3.2	0	ptsI	3	22.3	0.244
3.2	0	clcB	3	22.3	0.289
3.2	0	ycaM	3	22.3	0.322
3.2	0	yadI	3	22.3	0.659
3.2	0	AG1	3	22.3	0.537
3.2	0	ptsI	3	22.55	0.244
3.2	0	clcB	3	22.55	0.294
3.2	0	ycaM	3	22.55	0.324
3.2	0	yadI	3	22.55	0.666
3.2	0	AG1	3	22.55	0.543
3.2	0	ptsI	3	22.8	0.247
3.2	0	clcB	3	22.8	0.301

3.2	0	ycaM	3	22.8	0.326
3.2	0	yadI	3	22.8	0.671
3.2	0	AG1	3	22.8	0.546
3.2	0	ptsI	3	23.05	0.249
3.2	0	clcB	3	23.05	0.308
3.2	0	ycaM	3	23.05	0.326
3.2	0	yadI	3	23.05	0.666
3.2	0	AG1	3	23.05	0.551
3.2	0	ptsI	3	23.3	0.249
3.2	0	clcB	3	23.3	0.312
3.2	0	ycaM	3	23.3	0.328
3.2	0	yadI	3	23.3	0.672
3.2	0	AG1	3	23.3	0.554
3.2	0	ptsI	3	23.55	0.251
3.2	0	clcB	3	23.55	0.316
3.2	0	ycaM	3	23.55	0.328
3.2	0	yadI	3	23.55	0.682
3.2	0	AG1	3	23.55	0.558
3.2	0	ptsI	3	23.8	0.253
3.2	0	clcB	3	23.8	0.32
3.2	0	ycaM	3	23.8	0.331
3.2	0	yadI	3	23.8	0.687
3.2	0	AG1	3	23.8	0.562
3.2	0	ptsI	3	24.05	0.255
3.2	0	clcB	3	24.05	0.322
3.2	0	ycaM	3	24.05	0.333
3.2	0	yadI	3	24.05	0.687
3.2	0	AG1	3	24.05	0.57
3.2	0	ptsI	3	24.3	0.254
3.2	0	clcB	3	24.3	0.32
3.2	0	ycaM	3	24.3	0.344
3.2	0	yadI	3	24.3	0.695
3.2	0	AG1	3	24.3	0.569
3.2	0	ptsI	4	0	0.176
3.2	0	clcB	4	0	0.193
3.2	0	ycaM	4	0	0.197
3.2	0	yadI	4	0	0.215
3.2	0	AG1	4	0	0.238
3.2	0	ptsI	4	0.25	0.181
3.2	0	clcB	4	0.25	0.192
3.2	0	ycaM	4	0.25	0.197
3.2	0	yadI	4	0.25	0.216
3.2	0	AG1	4	0.25	0.239
3.2	0	ptsI	4	0.5	0.182
3.2	0	clcB	4	0.5	0.195
3.2	0	ycaM	4	0.5	0.199
3.2	0	yadI	4	0.5	0.219
3.2	0	AG1	4	0.5	0.24
3.2	0	ptsI	4	0.75	0.188
3.2	0	clcB	4	0.75	0.199
3.2	0	ycaM	4	0.75	0.201
3.2	0	yadI	4	0.75	0.224
3.2	0	AG1	4	0.75	0.244

3.2	0	ptsI	4	1	0.19
3.2	0	clcB	4	1	0.203
3.2	0	ycaM	4	1	0.203
3.2	0	yadI	4	1	0.224
3.2	0	AG1	4	1	0.246
3.2	0	ptsI	4	1.25	0.2
3.2	0	clcB	4	1.25	0.209
3.2	0	ycaM	4	1.25	0.207
3.2	0	yadI	4	1.25	0.23
3.2	0	AG1	4	1.25	0.255
3.2	0	ptsI	4	1.5	0.201
3.2	0	clcB	4	1.5	0.205
3.2	0	ycaM	4	1.5	0.209
3.2	0	yadI	4	1.5	0.229
3.2	0	AG1	4	1.5	0.254
3.2	0	ptsI	4	1.75	0.213
3.2	0	clcB	4	1.75	0.215
3.2	0	ycaM	4	1.75	0.217
3.2	0	yadI	4	1.75	0.239
3.2	0	AG1	4	1.75	0.263
3.2	0	ptsI	4	2	0.223
3.2	0	clcB	4	2	0.229
3.2	0	ycaM	4	2	0.226
3.2	0	yadI	4	2	0.247
3.2	0	AG1	4	2	0.272
3.2	0	ptsI	4	2.25	0.234
3.2	0	clcB	4	2.25	0.244
3.2	0	ycaM	4	2.25	0.236
3.2	0	yadI	4	2.25	0.263
3.2	0	AG1	4	2.25	0.288
3.2	0	ptsI	4	2.5	0.247
3.2	0	clcB	4	2.5	0.24
3.2	0	ycaM	4	2.5	0.24
3.2	0	yadI	4	2.5	0.269
3.2	0	AG1	4	2.5	0.292
3.2	0	ptsI	4	2.75	0.262
3.2	0	clcB	4	2.75	0.251
3.2	0	ycaM	4	2.75	0.25
3.2	0	yadI	4	2.75	0.282
3.2	0	AG1	4	2.75	0.302
3.2	0	ptsI	4	3	0.28
3.2	0	clcB	4	3	0.272
3.2	0	ycaM	4	3	0.264
3.2	0	yadI	4	3	0.301
3.2	0	AG1	4	3	0.321
3.2	0	ptsI	4	3.25	0.297
3.2	0	clcB	4	3.25	0.287
3.2	0	ycaM	4	3.25	0.273
3.2	0	yadI	4	3.25	0.319
3.2	0	AG1	4	3.25	0.337
3.2	0	ptsI	4	3.5	0.306
3.2	0	clcB	4	3.5	0.309
3.2	0	ycaM	4	3.5	0.289

3.2	0	yadI	4	3.5	0.342
3.2	0	AG1	4	3.5	0.352
3.2	0	ptsI	4	3.75	0.322
3.2	0	clcB	4	3.75	0.32
3.2	0	ycaM	4	3.75	0.299
3.2	0	yadI	4	3.75	0.358
3.2	0	AG1	4	3.75	0.369
3.2	0	ptsI	4	4	0.338
3.2	0	clcB	4	4	0.341
3.2	0	ycaM	4	4	0.317
3.2	0	yadI	4	4	0.379
3.2	0	AG1	4	4	0.392
3.2	0	ptsI	4	4.35	0.324
3.2	0	clcB	4	4.35	0.262
3.2	0	ycaM	4	4.35	0.345
3.2	0	yadI	4	4.35	0.362
3.2	0	AG1	4	4.35	0.28
3.2	0	ptsI	4	4.6	0.362
3.2	0	clcB	4	4.6	0.268
3.2	0	ycaM	4	4.6	0.348
3.2	0	yadI	4	4.6	0.375
3.2	0	AG1	4	4.6	0.289
3.2	0	ptsI	4	4.85	0.379
3.2	0	clcB	4	4.85	0.276
3.2	0	ycaM	4	4.85	0.358
3.2	0	yadI	4	4.85	0.39
3.2	0	AG1	4	4.85	0.3
3.2	0	ptsI	4	5.1	0.393
3.2	0	clcB	4	5.1	0.282
3.2	0	ycaM	4	5.1	0.373
3.2	0	yadI	4	5.1	0.405
3.2	0	AG1	4	5.1	0.311
3.2	0	ptsI	4	5.35	0.407
3.2	0	clcB	4	5.35	0.29
3.2	0	ycaM	4	5.35	0.389
3.2	0	yadI	4	5.35	0.42
3.2	0	AG1	4	5.35	0.322
3.2	0	ptsI	4	5.6	0.429
3.2	0	clcB	4	5.6	0.298
3.2	0	ycaM	4	5.6	0.401
3.2	0	yadI	4	5.6	0.433
3.2	0	AG1	4	5.6	0.329
3.2	0	ptsI	4	5.85	0.438
3.2	0	clcB	4	5.85	0.307
3.2	0	ycaM	4	5.85	0.411
3.2	0	yadI	4	5.85	0.449
3.2	0	AG1	4	5.85	0.335
3.2	0	ptsI	4	6.1	0.453
3.2	0	clcB	4	6.1	0.317
3.2	0	ycaM	4	6.1	0.422
3.2	0	yadI	4	6.1	0.466
3.2	0	AG1	4	6.1	0.345
3.2	0	ptsI	4	6.35	0.466

3.2	0	clcB	4	6.35	0.326
3.2	0	ycaM	4	6.35	0.435
3.2	0	yadI	4	6.35	0.48
3.2	0	AG1	4	6.35	0.356
3.2	0	ptsI	4	6.6	0.475
3.2	0	clcB	4	6.6	0.337
3.2	0	ycaM	4	6.6	0.445
3.2	0	yadI	4	6.6	0.492
3.2	0	AG1	4	6.6	0.364
3.2	0	ptsI	4	6.85	0.487
3.2	0	clcB	4	6.85	0.347
3.2	0	ycaM	4	6.85	0.455
3.2	0	yadI	4	6.85	0.506
3.2	0	AG1	4	6.85	0.375
3.2	0	ptsI	4	7.1	0.497
3.2	0	clcB	4	7.1	0.354
3.2	0	ycaM	4	7.1	0.465
3.2	0	yadI	4	7.1	0.517
3.2	0	AG1	4	7.1	0.384
3.2	0	ptsI	4	7.35	0.507
3.2	0	clcB	4	7.35	0.363
3.2	0	ycaM	4	7.35	0.473
3.2	0	yadI	4	7.35	0.525
3.2	0	AG1	4	7.35	0.393
3.2	0	ptsI	4	7.6	0.514
3.2	0	clcB	4	7.6	0.372
3.2	0	ycaM	4	7.6	0.48
3.2	0	yadI	4	7.6	0.536
3.2	0	AG1	4	7.6	0.4
3.2	0	ptsI	4	7.85	0.524
3.2	0	clcB	4	7.85	0.383
3.2	0	ycaM	4	7.85	0.486
3.2	0	yadI	4	7.85	0.544
3.2	0	AG1	4	7.85	0.409
3.2	0	ptsI	4	8.1	0.532
3.2	0	clcB	4	8.1	0.394
3.2	0	ycaM	4	8.1	0.492
3.2	0	yadI	4	8.1	0.556
3.2	0	AG1	4	8.1	0.416
3.2	0	ptsI	4	8.35	0.541
3.2	0	clcB	4	8.35	0.401
3.2	0	ycaM	4	8.35	0.494
3.2	0	yadI	4	8.35	0.564
3.2	0	AG1	4	8.35	0.425
3.2	0	ptsI	4	8.83333	0.391
3.2	0	clcB	4	8.83333	0.399
3.2	0	ycaM	4	8.83333	0.398
3.2	0	yadI	4	8.83333	0.564
3.2	0	AG1	4	8.83333	0.467
3.2	0	ptsI	4	9.08333	0.414
3.2	0	clcB	4	9.08333	0.417
3.2	0	ycaM	4	9.08333	0.407
3.2	0	yadI	4	9.08333	0.568

3.2	0	AG1	4	9.08333	0.49
3.2	0	ptsI	4	9.33333	0.434
3.2	0	clcB	4	9.33333	0.437
3.2	0	ycaM	4	9.33333	0.42
3.2	0	yadI	4	9.33333	0.582
3.2	0	AG1	4	9.33333	0.509
3.2	0	ptsI	4	9.58333	0.437
3.2	0	clcB	4	9.58333	0.439
3.2	0	ycaM	4	9.58333	0.427
3.2	0	yadI	4	9.58333	0.597
3.2	0	AG1	4	9.58333	0.514
3.2	0	ptsI	4	9.83333	0.443
3.2	0	clcB	4	9.83333	0.434
3.2	0	ycaM	4	9.83333	0.426
3.2	0	yadI	4	9.83333	0.601
3.2	0	AG1	4	9.83333	0.517
3.2	0	ptsI	4	10.0833	0.443
3.2	0	clcB	4	10.0833	0.44
3.2	0	ycaM	4	10.0833	0.43
3.2	0	yadI	4	10.0833	0.605
3.2	0	AG1	4	10.0833	0.521
3.2	0	ptsI	4	10.3333	0.441
3.2	0	clcB	4	10.3333	0.437
3.2	0	ycaM	4	10.3333	0.433
3.2	0	yadI	4	10.3333	0.613
3.2	0	AG1	4	10.3333	0.529
3.2	0	ptsI	4	10.5833	0.447
3.2	0	clcB	4	10.5833	0.443
3.2	0	ycaM	4	10.5833	0.438
3.2	0	yadI	4	10.5833	0.615
3.2	0	AG1	4	10.5833	0.534
3.2	0	ptsI	4	10.8333	0.445
3.2	0	clcB	4	10.8333	0.442
3.2	0	ycaM	4	10.8333	0.438
3.2	0	yadI	4	10.8333	0.622
3.2	0	AG1	4	10.8333	0.532
3.2	0	ptsI	4	11.0833	0.456
3.2	0	clcB	4	11.0833	0.444
3.2	0	ycaM	4	11.0833	0.442
3.2	0	yadI	4	11.0833	0.626
3.2	0	AG1	4	11.0833	0.535
3.2	0	ptsI	4	11.3333	0.461
3.2	0	clcB	4	11.3333	0.443
3.2	0	ycaM	4	11.3333	0.449
3.2	0	yadI	4	11.3333	0.63
3.2	0	AG1	4	11.3333	0.538
3.2	0	ptsI	4	11.5833	0.464
3.2	0	clcB	4	11.5833	0.45
3.2	0	ycaM	4	11.5833	0.451
3.2	0	yadI	4	11.5833	0.636
3.2	0	AG1	4	11.5833	0.543
3.2	0	ptsI	4	11.8333	0.471
3.2	0	clcB	4	11.8333	0.454

3.2	0	ycaM	4	11.8333	0.459
3.2	0	yadI	4	11.8333	0.645
3.2	0	AG1	4	11.8333	0.544
3.2	0	ptsI	4	12.0833	0.474
3.2	0	clcB	4	12.0833	0.458
3.2	0	ycaM	4	12.0833	0.462
3.2	0	yadI	4	12.0833	0.646
3.2	0	AG1	4	12.0833	0.553
3.2	0	ptsI	4	12.3333	0.481
3.2	0	clcB	4	12.3333	0.456
3.2	0	ycaM	4	12.3333	0.47
3.2	0	yadI	4	12.3333	0.656
3.2	0	AG1	4	12.3333	0.555
3.2	0	ptsI	4	12.5833	0.48
3.2	0	clcB	4	12.5833	0.462
3.2	0	ycaM	4	12.5833	0.467
3.2	0	yadI	4	12.5833	0.658
3.2	0	AG1	4	12.5833	0.561
3.2	0	ptsI	4	12.8333	0.488
3.2	0	clcB	4	12.8333	0.463
3.2	0	ycaM	4	12.8333	0.475
3.2	0	yadI	4	12.8333	0.663
3.2	0	AG1	4	12.8333	0.564
3.2	0	ptsI	4	13.0833	0.499
3.2	0	clcB	4	13.0833	0.465
3.2	0	ycaM	4	13.0833	0.48
3.2	0	yadI	4	13.0833	0.668
3.2	0	AG1	4	13.0833	0.568
3.2	0	ptsI	4	13.3333	0.504
3.2	0	clcB	4	13.3333	0.47
3.2	0	ycaM	4	13.3333	0.484
3.2	0	yadI	4	13.3333	0.672
3.2	0	AG1	4	13.3333	0.575
3.2	0	ptsI	4	13.5833	0.512
3.2	0	clcB	4	13.5833	0.473
3.2	0	ycaM	4	13.5833	0.487
3.2	0	yadI	4	13.5833	0.675
3.2	0	AG1	4	13.5833	0.573
3.2	0	ptsI	4	13.8333	0.518
3.2	0	clcB	4	13.8333	0.475
3.2	0	ycaM	4	13.8333	0.493
3.2	0	yadI	4	13.8333	0.682
3.2	0	AG1	4	13.8333	0.584
3.2	0	ptsI	4	14.0833	0.519
3.2	0	clcB	4	14.0833	0.483
3.2	0	ycaM	4	14.0833	0.495
3.2	0	yadI	4	14.0833	0.687
3.2	0	AG1	4	14.0833	0.586
3.2	0	ptsI	4	14.3333	0.52
3.2	0	clcB	4	14.3333	0.486
3.2	0	ycaM	4	14.3333	0.498
3.2	0	yadI	4	14.3333	0.689
3.2	0	AG1	4	14.3333	0.589

3.2	0	ptsI	4	14.5833	0.528
3.2	0	clcB	4	14.5833	0.488
3.2	0	ycaM	4	14.5833	0.508
3.2	0	yadI	4	14.5833	0.698
3.2	0	AG1	4	14.5833	0.592
3.2	0	ptsI	4	14.8333	0.531
3.2	0	clcB	4	14.8333	0.487
3.2	0	ycaM	4	14.8333	0.511
3.2	0	yadI	4	14.8333	0.696
3.2	0	AG1	4	14.8333	0.597
3.2	0	ptsI	4	15.0833	0.537
3.2	0	clcB	4	15.0833	0.491
3.2	0	ycaM	4	15.0833	0.507
3.2	0	yadI	4	15.0833	0.698
3.2	0	AG1	4	15.0833	0.599
3.2	0	ptsI	4	15.3333	0.546
3.2	0	clcB	4	15.3333	0.494
3.2	0	ycaM	4	15.3333	0.514
3.2	0	yadI	4	15.3333	0.706
3.2	0	AG1	4	15.3333	0.606
3.2	0	ptsI	4	15.5833	0.545
3.2	0	clcB	4	15.5833	0.496
3.2	0	ycaM	4	15.5833	0.521
3.2	0	yadI	4	15.5833	0.707
3.2	0	AG1	4	15.5833	0.608
3.2	0	ptsI	4	15.8333	0.552
3.2	0	clcB	4	15.8333	0.499
3.2	0	ycaM	4	15.8333	0.521
3.2	0	yadI	4	15.8333	0.714
3.2	0	AG1	4	15.8333	0.614
3.2	0	ptsI	4	16.0833	0.559
3.2	0	clcB	4	16.0833	0.505
3.2	0	ycaM	4	16.0833	0.524
3.2	0	yadI	4	16.0833	0.716
3.2	0	AG1	4	16.0833	0.614
3.2	0	ptsI	4	16.3333	0.561
3.2	0	clcB	4	16.3333	0.506
3.2	0	ycaM	4	16.3333	0.529
3.2	0	yadI	4	16.3333	0.722
3.2	0	AG1	4	16.3333	0.621
3.2	0	ptsI	4	16.5833	0.56
3.2	0	clcB	4	16.5833	0.506
3.2	0	ycaM	4	16.5833	0.53
3.2	0	yadI	4	16.5833	0.725
3.2	0	AG1	4	16.5833	0.623
3.2	0	ptsI	4	16.8333	0.573
3.2	0	clcB	4	16.8333	0.512
3.2	0	ycaM	4	16.8333	0.534
3.2	0	yadI	4	16.8333	0.727
3.2	0	AG1	4	16.8333	0.627
3.2	0	ptsI	4	17.0833	0.578
3.2	0	clcB	4	17.0833	0.517
3.2	0	ycaM	4	17.0833	0.537

3.2	0	yadI	4	17.0833	0.728
3.2	0	AG1	4	17.0833	0.629
3.2	0	ptsI	4	17.3333	0.583
3.2	0	clcB	4	17.3333	0.52
3.2	0	ycaM	4	17.3333	0.54
3.2	0	yadI	4	17.3333	0.732
3.2	0	AG1	4	17.3333	0.63
3.2	0	ptsI	4	17.5833	0.592
3.2	0	clcB	4	17.5833	0.519
3.2	0	ycaM	4	17.5833	0.544
3.2	0	yadI	4	17.5833	0.733
3.2	0	AG1	4	17.5833	0.636
3.2	0	ptsI	4	17.8333	0.598
3.2	0	clcB	4	17.8333	0.522
3.2	0	ycaM	4	17.8333	0.548
3.2	0	yadI	4	17.8333	0.719
3.2	0	AG1	4	17.8333	0.64
3.2	0	ptsI	4	18.0833	0.599
3.2	0	clcB	4	18.0833	0.525
3.2	0	ycaM	4	18.0833	0.551
3.2	0	yadI	4	18.0833	0.738
3.2	0	AG1	4	18.0833	0.64
3.2	0	ptsI	4	18.3333	0.608
3.2	0	clcB	4	18.3333	0.527
3.2	0	ycaM	4	18.3333	0.554
3.2	0	yadI	4	18.3333	0.743
3.2	0	AG1	4	18.3333	0.643
3.2	0	ptsI	4	18.5833	0.609
3.2	0	clcB	4	18.5833	0.527
3.2	0	ycaM	4	18.5833	0.555
3.2	0	yadI	4	18.5833	0.744
3.2	0	AG1	4	18.5833	0.645
3.2	0	ptsI	4	18.8333	0.613
3.2	0	clcB	4	18.8333	0.528
3.2	0	ycaM	4	18.8333	0.56
3.2	0	yadI	4	18.8333	0.73
3.2	0	AG1	4	18.8333	0.648
3.2	0	ptsI	4	19.0833	0.62
3.2	0	clcB	4	19.0833	0.533
3.2	0	ycaM	4	19.0833	0.56
3.2	0	yadI	4	19.0833	0.75
3.2	0	AG1	4	19.0833	0.65
3.2	0	ptsI	4	19.3333	0.621
3.2	0	clcB	4	19.3333	0.534
3.2	0	ycaM	4	19.3333	0.564
3.2	0	yadI	4	19.3333	0.754
3.2	0	AG1	4	19.3333	0.652
3.2	0	ptsI	4	19.5833	0.624
3.2	0	clcB	4	19.5833	0.538
3.2	0	ycaM	4	19.5833	0.568
3.2	0	yadI	4	19.5833	0.756
3.2	0	AG1	4	19.5833	0.656
3.2	0	ptsI	4	19.8333	0.629

3.2	0	clcB	4	19.8333	0.535
3.2	0	ycaM	4	19.8333	0.569
3.2	0	yadI	4	19.8333	0.758
3.2	0	AG1	4	19.8333	0.662
3.2	0	ptsI	4	20.0833	0.644
3.2	0	clcB	4	20.0833	0.541
3.2	0	ycaM	4	20.0833	0.573
3.2	0	yadI	4	20.0833	0.75
3.2	0	AG1	4	20.0833	0.663
3.2	0	ptsI	4	20.3333	0.638
3.2	0	clcB	4	20.3333	0.54
3.2	0	ycaM	4	20.3333	0.574
3.2	0	yadI	4	20.3333	0.767
3.2	0	AG1	4	20.3333	0.663
3.2	0	ptsI	4	20.5833	0.642
3.2	0	clcB	4	20.5833	0.546
3.2	0	ycaM	4	20.5833	0.576
3.2	0	yadI	4	20.5833	0.769
3.2	0	AG1	4	20.5833	0.669
3.2	0	ptsI	4	20.8333	0.648
3.2	0	clcB	4	20.8333	0.548
3.2	0	ycaM	4	20.8333	0.58
3.2	0	yadI	4	20.8333	0.771
3.2	0	AG1	4	20.8333	0.669
3.2	0	ptsI	4	21.0833	0.653
3.2	0	clcB	4	21.0833	0.547
3.2	0	ycaM	4	21.0833	0.578
3.2	0	yadI	4	21.0833	0.774
3.2	0	AG1	4	21.0833	0.668
3.2	0	ptsI	4	21.3333	0.654
3.2	0	clcB	4	21.3333	0.548
3.2	0	ycaM	4	21.3333	0.584
3.2	0	yadI	4	21.3333	0.777
3.2	0	AG1	4	21.3333	0.672
3.2	0	ptsI	4	21.5833	0.658
3.2	0	clcB	4	21.5833	0.552
3.2	0	ycaM	4	21.5833	0.582
3.2	0	yadI	4	21.5833	0.775
3.2	0	AG1	4	21.5833	0.671
3.2	0	ptsI	4	21.8333	0.658
3.2	0	clcB	4	21.8333	0.556
3.2	0	ycaM	4	21.8333	0.586
3.2	0	yadI	4	21.8333	0.782
3.2	0	AG1	4	21.8333	0.676
3.2	0	ptsI	4	22.0833	0.666
3.2	0	clcB	4	22.0833	0.557
3.2	0	ycaM	4	22.0833	0.587
3.2	0	yadI	4	22.0833	0.784
3.2	0	AG1	4	22.0833	0.679
3.2	0	ptsI	4	22.3333	0.666
3.2	0	clcB	4	22.3333	0.56
3.2	0	ycaM	4	22.3333	0.59
3.2	0	yadI	4	22.3333	0.786

3.2	0	AG1	4	22.3333	0.683
3.2	0	ptsI	4	22.5833	0.665
3.2	0	clcB	4	22.5833	0.561
3.2	0	ycaM	4	22.5833	0.592
3.2	0	yadI	4	22.5833	0.786
3.2	0	AG1	4	22.5833	0.684
3.2	0	ptsI	4	22.8333	0.672
3.2	0	clcB	4	22.8333	0.561
3.2	0	ycaM	4	22.8333	0.593
3.2	0	yadI	4	22.8333	0.789
3.2	0	AG1	4	22.8333	0.686
3.2	0	ptsI	4	23.0833	0.675
3.2	0	clcB	4	23.0833	0.561
3.2	0	ycaM	4	23.0833	0.593
3.2	0	yadI	4	23.0833	0.789
3.2	0	AG1	4	23.0833	0.683
3.2	0	ptsI	4	23.3333	0.679
3.2	0	clcB	4	23.3333	0.565
3.2	0	ycaM	4	23.3333	0.596
3.2	0	yadI	4	23.3333	0.803
3.2	0	AG1	4	23.3333	0.688
3.2	0	ptsI	4	23.5833	0.683
3.2	0	clcB	4	23.5833	0.566
3.2	0	ycaM	4	23.5833	0.6
3.2	0	yadI	4	23.5833	0.796
3.2	0	AG1	4	23.5833	0.692
3.2	0	ptsI	4	23.8333	0.684
3.2	0	clcB	4	23.8333	0.571
3.2	0	ycaM	4	23.8333	0.596
3.2	0	yadI	4	23.8333	0.793
3.2	0	AG1	4	23.8333	0.693
3.2	0	ptsI	4	24.0833	0.689
3.2	0	clcB	4	24.0833	0.57
3.2	0	ycaM	4	24.0833	0.601
3.2	0	yadI	4	24.0833	0.799
3.2	0	AG1	4	24.0833	0.695
3.2	0	ptsI	5	0	0.163
3.2	0	clcB	5	0	0.163
3.2	0	ycaM	5	0	0.165
3.2	0	yadI	5	0	0.154
3.2	0	AG1	5	0	0.156
3.2	0	ptsI	5	0.35	0.161
3.2	0	clcB	5	0.35	0.162
3.2	0	ycaM	5	0.35	0.163
3.2	0	yadI	5	0.35	0.157
3.2	0	AG1	5	0.35	0.156
3.2	0	ptsI	5	0.6	0.165
3.2	0	clcB	5	0.6	0.163
3.2	0	ycaM	5	0.6	0.164
3.2	0	yadI	5	0.6	0.158
3.2	0	AG1	5	0.6	0.156
3.2	0	ptsI	5	0.85	0.164
3.2	0	clcB	5	0.85	0.163

3.2	0	ycaM	5	0.85	0.162
3.2	0	yadI	5	0.85	0.157
3.2	0	AG1	5	0.85	0.156
3.2	0	ptsI	5	1.1	0.164
3.2	0	clcB	5	1.1	0.163
3.2	0	ycaM	5	1.1	0.165
3.2	0	yadI	5	1.1	0.158
3.2	0	AG1	5	1.1	0.157
3.2	0	ptsI	5	1.35	0.168
3.2	0	clcB	5	1.35	0.165
3.2	0	ycaM	5	1.35	0.166
3.2	0	yadI	5	1.35	0.161
3.2	0	AG1	5	1.35	0.158
3.2	0	ptsI	5	1.6	0.166
3.2	0	clcB	5	1.6	0.169
3.2	0	ycaM	5	1.6	0.17
3.2	0	yadI	5	1.6	0.163
3.2	0	AG1	5	1.6	0.161
3.2	0	ptsI	5	1.85	0.171
3.2	0	clcB	5	1.85	0.172
3.2	0	ycaM	5	1.85	0.173
3.2	0	yadI	5	1.85	0.167
3.2	0	AG1	5	1.85	0.165
3.2	0	ptsI	5	2.1	0.172
3.2	0	clcB	5	2.1	0.177
3.2	0	ycaM	5	2.1	0.175
3.2	0	yadI	5	2.1	0.173
3.2	0	AG1	5	2.1	0.172
3.2	0	ptsI	5	2.35	0.173
3.2	0	clcB	5	2.35	0.183
3.2	0	ycaM	5	2.35	0.18
3.2	0	yadI	5	2.35	0.178
3.2	0	AG1	5	2.35	0.177
3.2	0	ptsI	5	2.6	0.179
3.2	0	clcB	5	2.6	0.189
3.2	0	ycaM	5	2.6	0.183
3.2	0	yadI	5	2.6	0.179
3.2	0	AG1	5	2.6	0.174
3.2	0	ptsI	5	2.85	0.185
3.2	0	clcB	5	2.85	0.199
3.2	0	ycaM	5	2.85	0.19
3.2	0	yadI	5	2.85	0.189
3.2	0	AG1	5	2.85	0.189
3.2	0	ptsI	5	3.1	0.188
3.2	0	clcB	5	3.1	0.21
3.2	0	ycaM	5	3.1	0.199
3.2	0	yadI	5	3.1	0.199
3.2	0	AG1	5	3.1	0.198
3.2	0	ptsI	5	3.35	0.193
3.2	0	clcB	5	3.35	0.222
3.2	0	ycaM	5	3.35	0.209
3.2	0	yadI	5	3.35	0.211
3.2	0	AG1	5	3.35	0.213

3.2	0	ptsI	5	3.6	0.197
3.2	0	clcB	5	3.6	0.229
3.2	0	ycaM	5	3.6	0.216
3.2	0	yadI	5	3.6	0.224
3.2	0	AG1	5	3.6	0.227
3.2	0	ptsI	5	3.85	0.205
3.2	0	clcB	5	3.85	0.24
3.2	0	ycaM	5	3.85	0.226
3.2	0	yadI	5	3.85	0.238
3.2	0	AG1	5	3.85	0.244
3.2	0	ptsI	5	4.38333	0.202
3.2	0	clcB	5	4.38333	0.22
3.2	0	ycaM	5	4.38333	0.235
3.2	0	yadI	5	4.38333	0.198
3.2	0	AG1	5	4.38333	0.201
3.2	0	ptsI	5	4.63333	0.2
3.2	0	clcB	5	4.63333	0.213
3.2	0	ycaM	5	4.63333	0.237
3.2	0	yadI	5	4.63333	0.201
3.2	0	AG1	5	4.63333	0.208
3.2	0	ptsI	5	4.88333	0.207
3.2	0	clcB	5	4.88333	0.22
3.2	0	ycaM	5	4.88333	0.249
3.2	0	yadI	5	4.88333	0.209
3.2	0	AG1	5	4.88333	0.223
3.2	0	ptsI	5	5.13333	0.214
3.2	0	clcB	5	5.13333	0.227
3.2	0	ycaM	5	5.13333	0.26
3.2	0	yadI	5	5.13333	0.219
3.2	0	AG1	5	5.13333	0.236
3.2	0	ptsI	5	5.38333	0.221
3.2	0	clcB	5	5.38333	0.236
3.2	0	ycaM	5	5.38333	0.268
3.2	0	yadI	5	5.38333	0.229
3.2	0	AG1	5	5.38333	0.249
3.2	0	ptsI	5	5.63333	0.226
3.2	0	clcB	5	5.63333	0.246
3.2	0	ycaM	5	5.63333	0.277
3.2	0	yadI	5	5.63333	0.241
3.2	0	AG1	5	5.63333	0.264
3.2	0	ptsI	5	5.88333	0.233
3.2	0	clcB	5	5.88333	0.255
3.2	0	ycaM	5	5.88333	0.29
3.2	0	yadI	5	5.88333	0.249
3.2	0	AG1	5	5.88333	0.276
3.2	0	ptsI	5	6.13333	0.244
3.2	0	clcB	5	6.13333	0.271
3.2	0	ycaM	5	6.13333	0.3
3.2	0	yadI	5	6.13333	0.261
3.2	0	AG1	5	6.13333	0.294
3.2	0	ptsI	5	6.38333	0.252
3.2	0	clcB	5	6.38333	0.287
3.2	0	ycaM	5	6.38333	0.308

3.2	0	yadI	5	6.38333	0.276
3.2	0	AG1	5	6.38333	0.314
3.2	0	ptsI	5	6.63333	0.258
3.2	0	clcB	5	6.63333	0.306
3.2	0	ycaM	5	6.63333	0.322
3.2	0	yadI	5	6.63333	0.292
3.2	0	AG1	5	6.63333	0.337
3.2	0	ptsI	5	6.88333	0.268
3.2	0	clcB	5	6.88333	0.322
3.2	0	ycaM	5	6.88333	0.332
3.2	0	yadI	5	6.88333	0.31
3.2	0	AG1	5	6.88333	0.353
3.2	0	ptsI	5	7.13333	0.276
3.2	0	clcB	5	7.13333	0.341
3.2	0	ycaM	5	7.13333	0.344
3.2	0	yadI	5	7.13333	0.325
3.2	0	AG1	5	7.13333	0.376
3.2	0	ptsI	5	7.38333	0.284
3.2	0	clcB	5	7.38333	0.36
3.2	0	ycaM	5	7.38333	0.357
3.2	0	yadI	5	7.38333	0.343
3.2	0	AG1	5	7.38333	0.396
3.2	0	ptsI	5	7.63333	0.293
3.2	0	clcB	5	7.63333	0.383
3.2	0	ycaM	5	7.63333	0.371
3.2	0	yadI	5	7.63333	0.367
3.2	0	AG1	5	7.63333	0.413
3.2	0	ptsI	5	7.88333	0.302
3.2	0	clcB	5	7.88333	0.396
3.2	0	ycaM	5	7.88333	0.38
3.2	0	yadI	5	7.88333	0.389
3.2	0	AG1	5	7.88333	0.435
3.2	0	ptsI	5	8.13333	0.312
3.2	0	clcB	5	8.13333	0.407
3.2	0	ycaM	5	8.13333	0.388
3.2	0	yadI	5	8.13333	0.402
3.2	0	AG1	5	8.13333	0.445
3.2	0	ptsI	5	8.38333	0.318
3.2	0	clcB	5	8.38333	0.425
3.2	0	ycaM	5	8.38333	0.401
3.2	0	yadI	5	8.38333	0.42
3.2	0	AG1	5	8.38333	0.458
3.2	0	ptsI	5	8.88333	0.324
3.2	0	clcB	5	8.88333	0.346
3.2	0	ycaM	5	8.88333	0.338
3.2	0	yadI	5	8.88333	0.39
3.2	0	AG1	5	8.88333	0.455
3.2	0	ptsI	5	9.13333	0.343
3.2	0	clcB	5	9.13333	0.372
3.2	0	ycaM	5	9.13333	0.361
3.2	0	yadI	5	9.13333	0.408
3.2	0	AG1	5	9.13333	0.463
3.2	0	ptsI	5	9.38333	0.35

3.2	0	clcB	5	9.38333	0.389
3.2	0	ycaM	5	9.38333	0.373
3.2	0	yadI	5	9.38333	0.424
3.2	0	AG1	5	9.38333	0.482
3.2	0	ptsI	5	9.63333	0.357
3.2	0	clcB	5	9.63333	0.388
3.2	0	ycaM	5	9.63333	0.379
3.2	0	yadI	5	9.63333	0.431
3.2	0	AG1	5	9.63333	0.487
3.2	0	ptsI	5	9.88333	0.361
3.2	0	clcB	5	9.88333	0.389
3.2	0	ycaM	5	9.88333	0.386
3.2	0	yadI	5	9.88333	0.438
3.2	0	AG1	5	9.88333	0.494
3.2	0	ptsI	5	10.1333	0.361
3.2	0	clcB	5	10.1333	0.393
3.2	0	ycaM	5	10.1333	0.381
3.2	0	yadI	5	10.1333	0.434
3.2	0	AG1	5	10.1333	0.503
3.2	0	ptsI	5	10.3833	0.372
3.2	0	clcB	5	10.3833	0.4
3.2	0	ycaM	5	10.3833	0.389
3.2	0	yadI	5	10.3833	0.444
3.2	0	AG1	5	10.3833	0.51
3.2	0	ptsI	5	10.6333	0.372
3.2	0	clcB	5	10.6333	0.399
3.2	0	ycaM	5	10.6333	0.388
3.2	0	yadI	5	10.6333	0.444
3.2	0	AG1	5	10.6333	0.512
3.2	0	ptsI	5	10.8833	0.378
3.2	0	clcB	5	10.8833	0.407
3.2	0	ycaM	5	10.8833	0.39
3.2	0	yadI	5	10.8833	0.451
3.2	0	AG1	5	10.8833	0.525
3.2	0	ptsI	5	11.1333	0.379
3.2	0	clcB	5	11.1333	0.408
3.2	0	ycaM	5	11.1333	0.391
3.2	0	yadI	5	11.1333	0.451
3.2	0	AG1	5	11.1333	0.53
3.2	0	ptsI	5	11.3833	0.386
3.2	0	clcB	5	11.3833	0.414
3.2	0	ycaM	5	11.3833	0.396
3.2	0	yadI	5	11.3833	0.461
3.2	0	AG1	5	11.3833	0.537
3.2	0	ptsI	5	11.6333	0.388
3.2	0	clcB	5	11.6333	0.418
3.2	0	ycaM	5	11.6333	0.396
3.2	0	yadI	5	11.6333	0.461
3.2	0	AG1	5	11.6333	0.543
3.2	0	ptsI	5	11.8833	0.395
3.2	0	clcB	5	11.8833	0.418
3.2	0	ycaM	5	11.8833	0.396
3.2	0	yadI	5	11.8833	0.466

3.2	0	AG1	5	11.8833	0.549
3.2	0	ptsI	5	12.1333	0.398
3.2	0	clcB	5	12.1333	0.423
3.2	0	ycaM	5	12.1333	0.402
3.2	0	yadI	5	12.1333	0.472
3.2	0	AG1	5	12.1333	0.56
3.2	0	ptsI	5	12.3833	0.401
3.2	0	clcB	5	12.3833	0.426
3.2	0	ycaM	5	12.3833	0.405
3.2	0	yadI	5	12.3833	0.472
3.2	0	AG1	5	12.3833	0.539
3.2	0	ptsI	5	12.6333	0.408
3.2	0	clcB	5	12.6333	0.43
3.2	0	ycaM	5	12.6333	0.41
3.2	0	yadI	5	12.6333	0.479
3.2	0	AG1	5	12.6333	0.57
3.2	0	ptsI	5	12.8833	0.412
3.2	0	clcB	5	12.8833	0.435
3.2	0	ycaM	5	12.8833	0.412
3.2	0	yadI	5	12.8833	0.486
3.2	0	AG1	5	12.8833	0.578
3.2	0	ptsI	5	13.1333	0.41
3.2	0	clcB	5	13.1333	0.44
3.2	0	ycaM	5	13.1333	0.416
3.2	0	yadI	5	13.1333	0.484
3.2	0	AG1	5	13.1333	0.585
3.2	0	ptsI	5	13.3833	0.414
3.2	0	clcB	5	13.3833	0.443
3.2	0	ycaM	5	13.3833	0.422
3.2	0	yadI	5	13.3833	0.493
3.2	0	AG1	5	13.3833	0.591
3.2	0	ptsI	5	13.6333	0.421
3.2	0	clcB	5	13.6333	0.443
3.2	0	ycaM	5	13.6333	0.427
3.2	0	yadI	5	13.6333	0.498
3.2	0	AG1	5	13.6333	0.596
3.2	0	ptsI	5	13.8833	0.422
3.2	0	clcB	5	13.8833	0.446
3.2	0	ycaM	5	13.8833	0.43
3.2	0	yadI	5	13.8833	0.503
3.2	0	AG1	5	13.8833	0.599
3.2	0	ptsI	5	14.1333	0.424
3.2	0	clcB	5	14.1333	0.454
3.2	0	ycaM	5	14.1333	0.431
3.2	0	yadI	5	14.1333	0.507
3.2	0	AG1	5	14.1333	0.607
3.2	0	ptsI	5	14.3833	0.437
3.2	0	clcB	5	14.3833	0.454
3.2	0	ycaM	5	14.3833	0.438
3.2	0	yadI	5	14.3833	0.511
3.2	0	AG1	5	14.3833	0.615
3.2	0	ptsI	5	14.6333	0.438
3.2	0	clcB	5	14.6333	0.463

3.2	0	ycaM	5	14.6333	0.445
3.2	0	yadI	5	14.6333	0.512
3.2	0	AG1	5	14.6333	0.618
3.2	0	ptsI	5	14.8833	0.437
3.2	0	clcB	5	14.8833	0.463
3.2	0	ycaM	5	14.8833	0.442
3.2	0	yadI	5	14.8833	0.52
3.2	0	AG1	5	14.8833	0.622
3.2	0	ptsI	5	15.1333	0.444
3.2	0	clcB	5	15.1333	0.47
3.2	0	ycaM	5	15.1333	0.452
3.2	0	yadI	5	15.1333	0.523
3.2	0	AG1	5	15.1333	0.632
3.2	0	ptsI	5	15.3833	0.449
3.2	0	clcB	5	15.3833	0.472
3.2	0	ycaM	5	15.3833	0.453
3.2	0	yadI	5	15.3833	0.531
3.2	0	AG1	5	15.3833	0.638
3.2	0	ptsI	5	15.6333	0.45
3.2	0	clcB	5	15.6333	0.476
3.2	0	ycaM	5	15.6333	0.457
3.2	0	yadI	5	15.6333	0.53
3.2	0	AG1	5	15.6333	0.638
3.2	0	ptsI	5	15.8833	0.448
3.2	0	clcB	5	15.8833	0.479
3.2	0	ycaM	5	15.8833	0.458
3.2	0	yadI	5	15.8833	0.539
3.2	0	AG1	5	15.8833	0.646
3.2	0	ptsI	5	16.1333	0.458
3.2	0	clcB	5	16.1333	0.483
3.2	0	ycaM	5	16.1333	0.463
3.2	0	yadI	5	16.1333	0.541
3.2	0	AG1	5	16.1333	0.652
3.2	0	ptsI	5	16.3833	0.459
3.2	0	clcB	5	16.3833	0.485
3.2	0	ycaM	5	16.3833	0.464
3.2	0	yadI	5	16.3833	0.547
3.2	0	AG1	5	16.3833	0.658
3.2	0	ptsI	5	16.6333	0.464
3.2	0	clcB	5	16.6333	0.488
3.2	0	ycaM	5	16.6333	0.472
3.2	0	yadI	5	16.6333	0.555
3.2	0	AG1	5	16.6333	0.665
3.2	0	ptsI	5	16.8833	0.47
3.2	0	clcB	5	16.8833	0.491
3.2	0	ycaM	5	16.8833	0.478
3.2	0	yadI	5	16.8833	0.558
3.2	0	AG1	5	16.8833	0.667
3.2	0	ptsI	5	17.1333	0.468
3.2	0	clcB	5	17.1333	0.493
3.2	0	ycaM	5	17.1333	0.476
3.2	0	yadI	5	17.1333	0.558
3.2	0	AG1	5	17.1333	0.67

3.2	0	ptsI	5	17.3833	0.48
3.2	0	clcB	5	17.3833	0.497
3.2	0	ycaM	5	17.3833	0.483
3.2	0	yadI	5	17.3833	0.56
3.2	0	AG1	5	17.3833	0.68
3.2	0	ptsI	5	17.6333	0.479
3.2	0	clcB	5	17.6333	0.5
3.2	0	ycaM	5	17.6333	0.483
3.2	0	yadI	5	17.6333	0.567
3.2	0	AG1	5	17.6333	0.682
3.2	0	ptsI	5	17.8833	0.478
3.2	0	clcB	5	17.8833	0.505
3.2	0	ycaM	5	17.8833	0.487
3.2	0	yadI	5	17.8833	0.566
3.2	0	AG1	5	17.8833	0.648
3.2	0	ptsI	5	18.1333	0.485
3.2	0	clcB	5	18.1333	0.503
3.2	0	ycaM	5	18.1333	0.491
3.2	0	yadI	5	18.1333	0.572
3.2	0	AG1	5	18.1333	0.691
3.2	0	ptsI	5	18.3833	0.487
3.2	0	clcB	5	18.3833	0.509
3.2	0	ycaM	5	18.3833	0.497
3.2	0	yadI	5	18.3833	0.577
3.2	0	AG1	5	18.3833	0.69
3.2	0	ptsI	5	18.6333	0.494
3.2	0	clcB	5	18.6333	0.506
3.2	0	ycaM	5	18.6333	0.5
3.2	0	yadI	5	18.6333	0.583
3.2	0	AG1	5	18.6333	0.698
3.2	0	ptsI	5	18.8833	0.496
3.2	0	clcB	5	18.8833	0.512
3.2	0	ycaM	5	18.8833	0.506
3.2	0	yadI	5	18.8833	0.59
3.2	0	AG1	5	18.8833	0.704
3.2	0	ptsI	5	19.1333	0.494
3.2	0	clcB	5	19.1333	0.513
3.2	0	ycaM	5	19.1333	0.505
3.2	0	yadI	5	19.1333	0.591
3.2	0	AG1	5	19.1333	0.705
3.2	0	ptsI	5	19.3833	0.508
3.2	0	clcB	5	19.3833	0.512
3.2	0	ycaM	5	19.3833	0.502
3.2	0	yadI	5	19.3833	0.601
3.2	0	AG1	5	19.3833	0.712
3.2	0	ptsI	5	19.6333	0.507
3.2	0	clcB	5	19.6333	0.519
3.2	0	ycaM	5	19.6333	0.513
3.2	0	yadI	5	19.6333	0.603
3.2	0	AG1	5	19.6333	0.717
3.2	0	ptsI	5	19.8833	0.511
3.2	0	clcB	5	19.8833	0.519
3.2	0	ycaM	5	19.8833	0.516

3.2	0	yadI	5	19.8833	0.603
3.2	0	AG1	5	19.8833	0.718
3.2	0	ptsI	5	20.1333	0.517
3.2	0	clcB	5	20.1333	0.521
3.2	0	ycaM	5	20.1333	0.517
3.2	0	yadI	5	20.1333	0.611
3.2	0	AG1	5	20.1333	0.723
3.2	0	ptsI	5	20.3833	0.52
3.2	0	clcB	5	20.3833	0.521
3.2	0	ycaM	5	20.3833	0.52
3.2	0	yadI	5	20.3833	0.617
3.2	0	AG1	5	20.3833	0.726
3.2	0	ptsI	5	20.6333	0.521
3.2	0	clcB	5	20.6333	0.522
3.2	0	ycaM	5	20.6333	0.524
3.2	0	yadI	5	20.6333	0.625
3.2	0	AG1	5	20.6333	0.69
3.2	0	ptsI	5	20.8833	0.524
3.2	0	clcB	5	20.8833	0.532
3.2	0	ycaM	5	20.8833	0.529
3.2	0	yadI	5	20.8833	0.624
3.2	0	AG1	5	20.8833	0.733
3.2	0	ptsI	5	21.1333	0.531
3.2	0	clcB	5	21.1333	0.531
3.2	0	ycaM	5	21.1333	0.529
3.2	0	yadI	5	21.1333	0.629
3.2	0	AG1	5	21.1333	0.738
3.2	0	ptsI	5	21.3833	0.529
3.2	0	clcB	5	21.3833	0.533
3.2	0	ycaM	5	21.3833	0.533
3.2	0	yadI	5	21.3833	0.629
3.2	0	AG1	5	21.3833	0.743
3.2	0	ptsI	5	21.6333	0.534
3.2	0	clcB	5	21.6333	0.537
3.2	0	ycaM	5	21.6333	0.538
3.2	0	yadI	5	21.6333	0.634
3.2	0	AG1	5	21.6333	0.746
3.2	0	ptsI	5	21.8833	0.535
3.2	0	clcB	5	21.8833	0.539
3.2	0	ycaM	5	21.8833	0.539
3.2	0	yadI	5	21.8833	0.639
3.2	0	AG1	5	21.8833	0.746
3.2	0	ptsI	5	22.1333	0.537
3.2	0	clcB	5	22.1333	0.545
3.2	0	ycaM	5	22.1333	0.539
3.2	0	yadI	5	22.1333	0.643
3.2	0	AG1	5	22.1333	0.718
3.2	0	ptsI	5	22.3833	0.539
3.2	0	clcB	5	22.3833	0.543
3.2	0	ycaM	5	22.3833	0.543
3.2	0	yadI	5	22.3833	0.647
3.2	0	AG1	5	22.3833	0.764
3.2	0	ptsI	5	22.6333	0.548

3.2	0	clcB	5	22.6333	0.546
3.2	0	ycaM	5	22.6333	0.546
3.2	0	yadI	5	22.6333	0.655
3.2	0	AG1	5	22.6333	0.765
3.2	0	ptsI	5	22.8833	0.553
3.2	0	clcB	5	22.8833	0.55
3.2	0	ycaM	5	22.8833	0.55
3.2	0	yadI	5	22.8833	0.658
3.2	0	AG1	5	22.8833	0.768
3.2	0	ptsI	5	23.1333	0.557
3.2	0	clcB	5	23.1333	0.556
3.2	0	ycaM	5	23.1333	0.549
3.2	0	yadI	5	23.1333	0.666
3.2	0	AG1	5	23.1333	0.775
3.2	0	ptsI	5	23.3833	0.558
3.2	0	clcB	5	23.3833	0.552
3.2	0	ycaM	5	23.3833	0.554
3.2	0	yadI	5	23.3833	0.666
3.2	0	AG1	5	23.3833	0.773
3.2	0	ptsI	5	23.6333	0.568
3.2	0	clcB	5	23.6333	0.556
3.2	0	ycaM	5	23.6333	0.554
3.2	0	yadI	5	23.6333	0.669
3.2	0	AG1	5	23.6333	0.772
3.2	0	ptsI	5	23.8833	0.575
3.2	0	clcB	5	23.8833	0.558
3.2	0	ycaM	5	23.8833	0.554
3.2	0	yadI	5	23.8833	0.668
3.2	0	AG1	5	23.8833	0.734
3.2	0	ptsI	5	24.1333	0.582
3.2	0	clcB	5	24.1333	0.561
3.2	0	ycaM	5	24.1333	0.56
3.2	0	yadI	5	24.1333	0.677
3.2	0	AG1	5	24.1333	0.777
3.2	0	ptsI	5	24.3833	0.586
3.2	0	clcB	5	24.3833	0.566
3.2	0	ycaM	5	24.3833	0.563
3.2	0	yadI	5	24.3833	0.68
3.2	0	AG1	5	24.3833	0.778
0	0.05	ptsI	2	0	0.206
0	0.05	clcB	2	0	0.204
0	0.05	ycaM	2	0	0.214
0	0.05	yadI	2	0	0.216
0	0.05	AG1	2	0	0.233
0	0.05	ptsI	2	0.25	0.204
0	0.05	clcB	2	0.25	0.204
0	0.05	ycaM	2	0.25	0.211
0	0.05	yadI	2	0.25	0.21
0	0.05	AG1	2	0.25	0.223
0	0.05	ptsI	2	0.5	0.203
0	0.05	clcB	2	0.5	0.203
0	0.05	ycaM	2	0.5	0.209
0	0.05	yadI	2	0.5	0.209

0	0.05	AG1	2	0.5	0.221
0	0.05	ptsI	2	0.75	0.207
0	0.05	clcB	2	0.75	0.205
0	0.05	ycaM	2	0.75	0.21
0	0.05	yadI	2	0.75	0.211
0	0.05	AG1	2	0.75	0.221
0	0.05	ptsI	2	1	0.206
0	0.05	clcB	2	1	0.207
0	0.05	ycaM	2	1	0.214
0	0.05	yadI	2	1	0.216
0	0.05	AG1	2	1	0.227
0	0.05	ptsI	2	1.25	0.209
0	0.05	clcB	2	1.25	0.208
0	0.05	ycaM	2	1.25	0.218
0	0.05	yadI	2	1.25	0.224
0	0.05	AG1	2	1.25	0.231
0	0.05	ptsI	2	1.5	0.21
0	0.05	clcB	2	1.5	0.21
0	0.05	ycaM	2	1.5	0.22
0	0.05	yadI	2	1.5	0.228
0	0.05	AG1	2	1.5	0.235
0	0.05	ptsI	2	1.75	0.216
0	0.05	clcB	2	1.75	0.212
0	0.05	ycaM	2	1.75	0.224
0	0.05	yadI	2	1.75	0.236
0	0.05	AG1	2	1.75	0.239
0	0.05	ptsI	2	2	0.217
0	0.05	clcB	2	2	0.214
0	0.05	ycaM	2	2	0.232
0	0.05	yadI	2	2	0.243
0	0.05	AG1	2	2	0.246
0	0.05	ptsI	2	2.25	0.223
0	0.05	clcB	2	2.25	0.217
0	0.05	ycaM	2	2.25	0.239
0	0.05	yadI	2	2.25	0.251
0	0.05	AG1	2	2.25	0.251
0	0.05	ptsI	2	2.5	0.231
0	0.05	clcB	2	2.5	0.223
0	0.05	ycaM	2	2.5	0.247
0	0.05	yadI	2	2.5	0.266
0	0.05	AG1	2	2.5	0.259
0	0.05	ptsI	2	2.75	0.235
0	0.05	clcB	2	2.75	0.232
0	0.05	ycaM	2	2.75	0.259
0	0.05	yadI	2	2.75	0.277
0	0.05	AG1	2	2.75	0.268
0	0.05	ptsI	2	3	0.241
0	0.05	clcB	2	3	0.233
0	0.05	ycaM	2	3	0.26
0	0.05	yadI	2	3	0.286
0	0.05	AG1	2	3	0.265
0	0.05	ptsI	2	3.25	0.247
0	0.05	clcB	2	3.25	0.241

0	0.05	ycaM	2	3.25	0.276
0	0.05	yadI	2	3.25	0.302
0	0.05	AG1	2	3.25	0.281
0	0.05	ptsI	2	3.5	0.257
0	0.05	clcB	2	3.5	0.243
0	0.05	ycaM	2	3.5	0.284
0	0.05	yadI	2	3.5	0.312
0	0.05	AG1	2	3.5	0.289
0	0.05	ptsI	2	3.75	0.263
0	0.05	clcB	2	3.75	0.247
0	0.05	ycaM	2	3.75	0.294
0	0.05	yadI	2	3.75	0.326
0	0.05	AG1	2	3.75	0.3
0	0.05	ptsI	2	4	0.271
0	0.05	clcB	2	4	0.253
0	0.05	ycaM	2	4	0.306
0	0.05	yadI	2	4	0.343
0	0.05	AG1	2	4	0.311
0	0.05	ptsI	2	4.38333	0.282
0	0.05	clcB	2	4.38333	0.27
0	0.05	ycaM	2	4.38333	0.297
0	0.05	yadI	2	4.38333	0.377
0	0.05	AG1	2	4.38333	0.34
0	0.05	ptsI	2	4.63333	0.28
0	0.05	clcB	2	4.63333	0.265
0	0.05	ycaM	2	4.63333	0.299
0	0.05	yadI	2	4.63333	0.386
0	0.05	AG1	2	4.63333	0.339
0	0.05	ptsI	2	4.88333	0.288
0	0.05	clcB	2	4.88333	0.27
0	0.05	ycaM	2	4.88333	0.313
0	0.05	yadI	2	4.88333	0.401
0	0.05	AG1	2	4.88333	0.349
0	0.05	ptsI	2	5.13333	0.293
0	0.05	clcB	2	5.13333	0.276
0	0.05	ycaM	2	5.13333	0.316
0	0.05	yadI	2	5.13333	0.417
0	0.05	AG1	2	5.13333	0.357
0	0.05	ptsI	2	5.38333	0.299
0	0.05	clcB	2	5.38333	0.284
0	0.05	ycaM	2	5.38333	0.322
0	0.05	yadI	2	5.38333	0.434
0	0.05	AG1	2	5.38333	0.368
0	0.05	ptsI	2	5.63333	0.307
0	0.05	clcB	2	5.63333	0.29
0	0.05	ycaM	2	5.63333	0.324
0	0.05	yadI	2	5.63333	0.45
0	0.05	AG1	2	5.63333	0.378
0	0.05	ptsI	2	5.88333	0.314
0	0.05	clcB	2	5.88333	0.296
0	0.05	ycaM	2	5.88333	0.326
0	0.05	yadI	2	5.88333	0.466
0	0.05	AG1	2	5.88333	0.39

0	0.05	ptsI	2	6.13333	0.317
0	0.05	clcB	2	6.13333	0.305
0	0.05	ycaM	2	6.13333	0.37
0	0.05	yadI	2	6.13333	0.459
0	0.05	AG1	2	6.13333	0.4
0	0.05	ptsI	2	6.38333	0.326
0	0.05	clcB	2	6.38333	0.309
0	0.05	ycaM	2	6.38333	0.327
0	0.05	yadI	2	6.38333	0.47
0	0.05	AG1	2	6.38333	0.41
0	0.05	ptsI	2	6.63333	0.334
0	0.05	clcB	2	6.63333	0.315
0	0.05	ycaM	2	6.63333	0.331
0	0.05	yadI	2	6.63333	0.482
0	0.05	AG1	2	6.63333	0.424
0	0.05	ptsI	2	6.88333	0.344
0	0.05	clcB	2	6.88333	0.324
0	0.05	ycaM	2	6.88333	0.37
0	0.05	yadI	2	6.88333	0.492
0	0.05	AG1	2	6.88333	0.438
0	0.05	ptsI	2	7.13333	0.348
0	0.05	clcB	2	7.13333	0.322
0	0.05	ycaM	2	7.13333	0.335
0	0.05	yadI	2	7.13333	0.514
0	0.05	AG1	2	7.13333	0.438
0	0.05	ptsI	2	7.38333	0.357
0	0.05	clcB	2	7.38333	0.331
0	0.05	ycaM	2	7.38333	0.339
0	0.05	yadI	2	7.38333	0.506
0	0.05	AG1	2	7.38333	0.45
0	0.05	ptsI	2	7.63333	0.362
0	0.05	clcB	2	7.63333	0.34
0	0.05	ycaM	2	7.63333	0.347
0	0.05	yadI	2	7.63333	0.518
0	0.05	AG1	2	7.63333	0.464
0	0.05	ptsI	2	7.88333	0.374
0	0.05	clcB	2	7.88333	0.346
0	0.05	ycaM	2	7.88333	0.344
0	0.05	yadI	2	7.88333	0.528
0	0.05	AG1	2	7.88333	0.47
0	0.05	ptsI	2	8.13333	0.383
0	0.05	clcB	2	8.13333	0.349
0	0.05	ycaM	2	8.13333	0.344
0	0.05	yadI	2	8.13333	0.536
0	0.05	AG1	2	8.13333	0.476
0	0.05	ptsI	2	8.38333	0.39
0	0.05	clcB	2	8.38333	0.355
0	0.05	ycaM	2	8.38333	0.348
0	0.05	yadI	2	8.38333	0.55
0	0.05	AG1	2	8.38333	0.491
0	0.05	ptsI	2	8.91667	0.424
0	0.05	clcB	2	8.91667	0.358
0	0.05	ycaM	2	8.91667	0.374

0	0.05	yadI	2	8.91667	0.578
0	0.05	AG1	2	8.91667	0.558
0	0.05	ptsI	2	9.16667	0.425
0	0.05	clcB	2	9.16667	0.362
0	0.05	ycaM	2	9.16667	0.351
0	0.05	yadI	2	9.16667	0.59
0	0.05	AG1	2	9.16667	0.573
0	0.05	ptsI	2	9.41667	0.432
0	0.05	clcB	2	9.41667	0.371
0	0.05	ycaM	2	9.41667	0.387
0	0.05	yadI	2	9.41667	0.606
0	0.05	AG1	2	9.41667	0.59
0	0.05	ptsI	2	9.66667	0.445
0	0.05	clcB	2	9.66667	0.373
0	0.05	ycaM	2	9.66667	0.355
0	0.05	yadI	2	9.66667	0.614
0	0.05	AG1	2	9.66667	0.599
0	0.05	ptsI	2	9.91667	0.454
0	0.05	clcB	2	9.91667	0.382
0	0.05	ycaM	2	9.91667	0.357
0	0.05	yadI	2	9.91667	0.641
0	0.05	AG1	2	9.91667	0.613
0	0.05	ptsI	2	10.1667	0.463
0	0.05	clcB	2	10.1667	0.384
0	0.05	ycaM	2	10.1667	0.409
0	0.05	yadI	2	10.1667	0.645
0	0.05	AG1	2	10.1667	0.621
0	0.05	ptsI	2	10.4167	0.476
0	0.05	clcB	2	10.4167	0.388
0	0.05	ycaM	2	10.4167	0.358
0	0.05	yadI	2	10.4167	0.654
0	0.05	AG1	2	10.4167	0.626
0	0.05	ptsI	2	10.6667	0.484
0	0.05	clcB	2	10.6667	0.399
0	0.05	ycaM	2	10.6667	0.362
0	0.05	yadI	2	10.6667	0.673
0	0.05	AG1	2	10.6667	0.638
0	0.05	ptsI	2	10.9167	0.494
0	0.05	clcB	2	10.9167	0.405
0	0.05	ycaM	2	10.9167	0.364
0	0.05	yadI	2	10.9167	0.678
0	0.05	AG1	2	10.9167	0.649
0	0.05	ptsI	2	11.1667	0.502
0	0.05	clcB	2	11.1667	0.401
0	0.05	ycaM	2	11.1667	0.361
0	0.05	yadI	2	11.1667	0.693
0	0.05	AG1	2	11.1667	0.659
0	0.05	ptsI	2	11.4167	0.512
0	0.05	clcB	2	11.4167	0.406
0	0.05	ycaM	2	11.4167	0.363
0	0.05	yadI	2	11.4167	0.7
0	0.05	AG1	2	11.4167	0.657
0	0.05	ptsI	2	11.6667	0.532

0	0.05	clcB	2	11.6667	0.406
0	0.05	ycaM	2	11.6667	0.363
0	0.05	yadI	2	11.6667	0.706
0	0.05	AG1	2	11.6667	0.673
0	0.05	ptsI	2	11.9167	0.53
0	0.05	clcB	2	11.9167	0.417
0	0.05	ycaM	2	11.9167	0.367
0	0.05	yadI	2	11.9167	0.723
0	0.05	AG1	2	11.9167	0.671
0	0.05	ptsI	2	12.1667	0.548
0	0.05	clcB	2	12.1667	0.417
0	0.05	ycaM	2	12.1667	0.366
0	0.05	yadI	2	12.1667	0.723
0	0.05	AG1	2	12.1667	0.688
0	0.05	ptsI	2	12.4167	0.548
0	0.05	clcB	2	12.4167	0.414
0	0.05	ycaM	2	12.4167	0.368
0	0.05	yadI	2	12.4167	0.732
0	0.05	AG1	2	12.4167	0.688
0	0.05	ptsI	2	12.6667	0.54
0	0.05	clcB	2	12.6667	0.411
0	0.05	ycaM	2	12.6667	0.362
0	0.05	yadI	2	12.6667	0.74
0	0.05	AG1	2	12.6667	0.689
0	0.05	ptsI	2	12.9167	0.548
0	0.05	clcB	2	12.9167	0.412
0	0.05	ycaM	2	12.9167	0.367
0	0.05	yadI	2	12.9167	0.754
0	0.05	AG1	2	12.9167	0.692
0	0.05	ptsI	2	13.1667	0.558
0	0.05	clcB	2	13.1667	0.417
0	0.05	ycaM	2	13.1667	0.367
0	0.05	yadI	2	13.1667	0.772
0	0.05	AG1	2	13.1667	0.692
0	0.05	ptsI	2	13.4167	0.557
0	0.05	clcB	2	13.4167	0.412
0	0.05	ycaM	2	13.4167	0.367
0	0.05	yadI	2	13.4167	0.784
0	0.05	AG1	2	13.4167	0.695
0	0.05	ptsI	2	13.6667	0.576
0	0.05	clcB	2	13.6667	0.412
0	0.05	ycaM	2	13.6667	0.369
0	0.05	yadI	2	13.6667	0.791
0	0.05	AG1	2	13.6667	0.735
0	0.05	ptsI	2	13.9167	0.577
0	0.05	clcB	2	13.9167	0.414
0	0.05	ycaM	2	13.9167	0.37
0	0.05	yadI	2	13.9167	0.797
0	0.05	AG1	2	13.9167	0.709
0	0.05	ptsI	2	14.1667	0.587
0	0.05	clcB	2	14.1667	0.416
0	0.05	ycaM	2	14.1667	0.375
0	0.05	yadI	2	14.1667	0.82

0	0.05	AG1	2	14.1667	0.727
0	0.05	ptsI	2	14.4167	0.583
0	0.05	clcB	2	14.4167	0.414
0	0.05	ycaM	2	14.4167	0.368
0	0.05	yadI	2	14.4167	0.826
0	0.05	AG1	2	14.4167	0.71
0	0.05	ptsI	2	14.6667	0.587
0	0.05	clcB	2	14.6667	0.418
0	0.05	ycaM	2	14.6667	0.365
0	0.05	yadI	2	14.6667	0.821
0	0.05	AG1	2	14.6667	0.701
0	0.05	ptsI	2	14.9167	0.589
0	0.05	clcB	2	14.9167	0.419
0	0.05	ycaM	2	14.9167	0.363
0	0.05	yadI	2	14.9167	0.839
0	0.05	AG1	2	14.9167	0.702
0	0.05	ptsI	2	15.1667	0.611
0	0.05	clcB	2	15.1667	0.425
0	0.05	ycaM	2	15.1667	0.374
0	0.05	yadI	2	15.1667	0.853
0	0.05	AG1	2	15.1667	0.705
0	0.05	ptsI	2	15.4167	0.614
0	0.05	clcB	2	15.4167	0.425
0	0.05	ycaM	2	15.4167	0.368
0	0.05	yadI	2	15.4167	0.854
0	0.05	AG1	2	15.4167	0.697
0	0.05	ptsI	2	15.6667	0.603
0	0.05	clcB	2	15.6667	0.427
0	0.05	ycaM	2	15.6667	0.363
0	0.05	yadI	2	15.6667	0.856
0	0.05	AG1	2	15.6667	0.7
0	0.05	ptsI	2	15.9167	0.612
0	0.05	clcB	2	15.9167	0.432
0	0.05	ycaM	2	15.9167	0.366
0	0.05	yadI	2	15.9167	0.865
0	0.05	AG1	2	15.9167	0.717
0	0.05	ptsI	2	16.1667	0.632
0	0.05	clcB	2	16.1667	0.433
0	0.05	ycaM	2	16.1667	0.372
0	0.05	yadI	2	16.1667	0.885
0	0.05	AG1	2	16.1667	0.735
0	0.05	ptsI	2	16.4167	0.625
0	0.05	clcB	2	16.4167	0.437
0	0.05	ycaM	2	16.4167	0.369
0	0.05	yadI	2	16.4167	0.889
0	0.05	AG1	2	16.4167	0.73
0	0.05	ptsI	2	16.6667	0.625
0	0.05	clcB	2	16.6667	0.436
0	0.05	ycaM	2	16.6667	0.371
0	0.05	yadI	2	16.6667	0.893
0	0.05	AG1	2	16.6667	0.703
0	0.05	ptsI	2	16.9167	0.64
0	0.05	clcB	2	16.9167	0.441

0	0.05	ycaM	2	16.9167	0.374
0	0.05	yadI	2	16.9167	0.901
0	0.05	AG1	2	16.9167	0.712
0	0.05	ptsI	2	17.1667	0.649
0	0.05	clcB	2	17.1667	0.445
0	0.05	ycaM	2	17.1667	0.38
0	0.05	yadI	2	17.1667	0.925
0	0.05	AG1	2	17.1667	0.726
0	0.05	ptsI	2	17.4167	0.657
0	0.05	clcB	2	17.4167	0.446
0	0.05	ycaM	2	17.4167	0.386
0	0.05	yadI	2	17.4167	0.933
0	0.05	AG1	2	17.4167	0.733
0	0.05	ptsI	2	17.6667	0.656
0	0.05	clcB	2	17.6667	0.445
0	0.05	ycaM	2	17.6667	0.385
0	0.05	yadI	2	17.6667	0.933
0	0.05	AG1	2	17.6667	0.73
0	0.05	ptsI	2	17.9167	0.65
0	0.05	clcB	2	17.9167	0.45
0	0.05	ycaM	2	17.9167	0.393
0	0.05	yadI	2	17.9167	0.947
0	0.05	AG1	2	17.9167	0.734
0	0.05	ptsI	2	18.1667	0.669
0	0.05	clcB	2	18.1667	0.447
0	0.05	ycaM	2	18.1667	0.388
0	0.05	yadI	2	18.1667	0.942
0	0.05	AG1	2	18.1667	0.757
0	0.05	ptsI	2	18.4167	0.682
0	0.05	clcB	2	18.4167	0.452
0	0.05	ycaM	2	18.4167	0.398
0	0.05	yadI	2	18.4167	0.959
0	0.05	AG1	2	18.4167	0.763
0	0.05	ptsI	2	18.6667	0.665
0	0.05	clcB	2	18.6667	0.458
0	0.05	ycaM	2	18.6667	0.396
0	0.05	yadI	2	18.6667	0.962
0	0.05	AG1	2	18.6667	0.765
0	0.05	ptsI	2	18.9167	0.633
0	0.05	clcB	2	18.9167	0.45
0	0.05	ycaM	2	18.9167	0.392
0	0.05	yadI	2	18.9167	0.956
0	0.05	AG1	2	18.9167	0.773
0	0.05	ptsI	2	19.1667	0.68
0	0.05	clcB	2	19.1667	0.459
0	0.05	ycaM	2	19.1667	0.402
0	0.05	yadI	2	19.1667	0.969
0	0.05	AG1	2	19.1667	0.792
0	0.05	ptsI	2	19.4167	0.663
0	0.05	clcB	2	19.4167	0.459
0	0.05	ycaM	2	19.4167	0.4
0	0.05	yadI	2	19.4167	0.972
0	0.05	AG1	2	19.4167	0.787

0	0.05	ptsI	2	19.6667	0.668
0	0.05	clcB	2	19.6667	0.465
0	0.05	ycaM	2	19.6667	0.406
0	0.05	yadI	2	19.6667	0.983
0	0.05	AG1	2	19.6667	0.795
0	0.05	ptsI	2	19.9167	0.696
0	0.05	clcB	2	19.9167	0.466
0	0.05	ycaM	2	19.9167	0.404
0	0.05	yadI	2	19.9167	0.992
0	0.05	AG1	2	19.9167	0.822
0	0.05	ptsI	2	20.1667	0.665
0	0.05	clcB	2	20.1667	0.469
0	0.05	ycaM	2	20.1667	0.412
0	0.05	yadI	2	20.1667	0.994
0	0.05	AG1	2	20.1667	0.81
0	0.05	ptsI	2	20.4167	0.679
0	0.05	clcB	2	20.4167	0.469
0	0.05	ycaM	2	20.4167	0.406
0	0.05	yadI	2	20.4167	0.996
0	0.05	AG1	2	20.4167	0.819
0	0.05	ptsI	2	20.6667	0.666
0	0.05	clcB	2	20.6667	0.473
0	0.05	ycaM	2	20.6667	0.416
0	0.05	yadI	2	20.6667	0.995
0	0.05	AG1	2	20.6667	0.816
0	0.05	ptsI	2	20.9167	0.675
0	0.05	clcB	2	20.9167	0.477
0	0.05	ycaM	2	20.9167	0.416
0	0.05	yadI	2	20.9167	1.004
0	0.05	AG1	2	20.9167	0.822
0	0.05	ptsI	2	21.1667	0.676
0	0.05	clcB	2	21.1667	0.478
0	0.05	ycaM	2	21.1667	0.425
0	0.05	yadI	2	21.1667	1.018
0	0.05	AG1	2	21.1667	0.829
0	0.05	ptsI	2	21.4167	0.662
0	0.05	clcB	2	21.4167	0.472
0	0.05	ycaM	2	21.4167	0.429
0	0.05	yadI	2	21.4167	1.021
0	0.05	AG1	2	21.4167	0.834
0	0.05	ptsI	2	21.6667	0.687
0	0.05	clcB	2	21.6667	0.482
0	0.05	ycaM	2	21.6667	0.433
0	0.05	yadI	2	21.6667	1.033
0	0.05	AG1	2	21.6667	0.842
0	0.05	ptsI	2	21.9167	0.698
0	0.05	clcB	2	21.9167	0.481
0	0.05	ycaM	2	21.9167	0.436
0	0.05	yadI	2	21.9167	1.038
0	0.05	AG1	2	21.9167	0.845
0	0.05	ptsI	2	22.1667	0.692
0	0.05	clcB	2	22.1667	0.485
0	0.05	ycaM	2	22.1667	0.442

0	0.05	yadI	2	22.1667	1.043
0	0.05	AG1	2	22.1667	0.845
0	0.05	ptsI	2	22.4167	0.684
0	0.05	clcB	2	22.4167	0.484
0	0.05	ycaM	2	22.4167	0.445
0	0.05	yadI	2	22.4167	1.046
0	0.05	AG1	2	22.4167	0.852
0	0.05	ptsI	2	22.6667	0.683
0	0.05	clcB	2	22.6667	0.485
0	0.05	ycaM	2	22.6667	0.446
0	0.05	yadI	2	22.6667	1.052
0	0.05	AG1	2	22.6667	0.857
0	0.05	ptsI	2	22.9167	0.689
0	0.05	clcB	2	22.9167	0.486
0	0.05	ycaM	2	22.9167	0.45
0	0.05	yadI	2	22.9167	1.059
0	0.05	AG1	2	22.9167	0.867
0	0.05	ptsI	2	23.1667	0.68
0	0.05	clcB	2	23.1667	0.492
0	0.05	ycaM	2	23.1667	0.44
0	0.05	yadI	2	23.1667	1.054
0	0.05	AG1	2	23.1667	0.868
0	0.05	ptsI	2	23.4167	0.685
0	0.05	clcB	2	23.4167	0.485
0	0.05	ycaM	2	23.4167	0.45
0	0.05	yadI	2	23.4167	1.066
0	0.05	AG1	2	23.4167	0.868
0	0.05	ptsI	2	23.6667	0.691
0	0.05	clcB	2	23.6667	0.491
0	0.05	ycaM	2	23.6667	0.45
0	0.05	yadI	2	23.6667	1.069
0	0.05	AG1	2	23.6667	0.874
0	0.05	ptsI	2	23.9167	0.691
0	0.05	clcB	2	23.9167	0.49
0	0.05	ycaM	2	23.9167	0.457
0	0.05	yadI	2	23.9167	1.078
0	0.05	AG1	2	23.9167	0.883
0	0.05	ptsI	2	24.1667	0.685
0	0.05	clcB	2	24.1667	0.486
0	0.05	ycaM	2	24.1667	0.458
0	0.05	yadI	2	24.1667	1.079
0	0.05	AG1	2	24.1667	0.881
0	0.05	ptsI	2	24.4167	0.688
0	0.05	clcB	2	24.4167	0.488
0	0.05	ycaM	2	24.4167	0.462
0	0.05	yadI	2	24.4167	1.084
0	0.05	AG1	2	24.4167	0.888
0	0.05	ptsI	2	24.6667	0.685
0	0.05	clcB	2	24.6667	0.493
0	0.05	ycaM	2	24.6667	0.46
0	0.05	yadI	2	24.6667	1.084
0	0.05	AG1	2	24.6667	0.897
0	0.05	ptsI	2	24.9167	0.685

0	0.05	clcB	2	24.9167	0.493
0	0.05	ycaM	2	24.9167	0.464
0	0.05	yadI	2	24.9167	1.09
0	0.05	AG1	2	24.9167	0.906
0	0.05	ptsI	2	25.1667	0.688
0	0.05	clcB	2	25.1667	0.497
0	0.05	ycaM	2	25.1667	0.468
0	0.05	yadI	2	25.1667	1.103
0	0.05	AG1	2	25.1667	0.908
0	0.05	ptsI	2	25.4167	0.684
0	0.05	clcB	2	25.4167	0.493
0	0.05	ycaM	2	25.4167	0.469
0	0.05	yadI	2	25.4167	1.102
0	0.05	AG1	2	25.4167	0.92
0	0.05	ptsI	2	25.6667	0.682
0	0.05	clcB	2	25.6667	0.495
0	0.05	ycaM	2	25.6667	0.474
0	0.05	yadI	2	25.6667	1.104
0	0.05	AG1	2	25.6667	0.922
0	0.05	ptsI	2	25.9167	0.69
0	0.05	clcB	2	25.9167	0.499
0	0.05	ycaM	2	25.9167	0.481
0	0.05	yadI	2	25.9167	1.118
0	0.05	AG1	2	25.9167	0.932
0	0.05	ptsI	2	26.1667	0.665
0	0.05	clcB	2	26.1667	0.494
0	0.05	ycaM	2	26.1667	0.474
0	0.05	yadI	2	26.1667	1.116
0	0.05	AG1	2	26.1667	0.939
0	0.05	ptsI	2	26.4167	0.685
0	0.05	clcB	2	26.4167	0.499
0	0.05	ycaM	2	26.4167	0.483
0	0.05	yadI	2	26.4167	1.116
0	0.05	AG1	2	26.4167	0.953
0	0.05	ptsI	3	0	0.16
0	0.05	clcB	3	0	0.162
0	0.05	ycaM	3	0	0.156
0	0.05	yadI	3	0	0.159
0	0.05	AG1	3	0	0.159
0	0.05	ptsI	3	0.25	0.156
0	0.05	clcB	3	0.25	0.161
0	0.05	ycaM	3	0.25	0.153
0	0.05	yadI	3	0.25	0.167
0	0.05	AG1	3	0.25	0.158
0	0.05	ptsI	3	0.5	0.156
0	0.05	clcB	3	0.5	0.16
0	0.05	ycaM	3	0.5	0.153
0	0.05	yadI	3	0.5	0.158
0	0.05	AG1	3	0.5	0.157
0	0.05	ptsI	3	0.75	0.156
0	0.05	clcB	3	0.75	0.161
0	0.05	ycaM	3	0.75	0.152
0	0.05	yadI	3	0.75	0.159

0	0.05	AG1	3	0.75	0.157
0	0.05	ptsI	3	1	0.156
0	0.05	clcB	3	1	0.161
0	0.05	ycaM	3	1	0.154
0	0.05	yadI	3	1	0.158
0	0.05	AG1	3	1	0.158
0	0.05	ptsI	3	1.25	0.157
0	0.05	clcB	3	1.25	0.162
0	0.05	ycaM	3	1.25	0.153
0	0.05	yadI	3	1.25	0.16
0	0.05	AG1	3	1.25	0.159
0	0.05	ptsI	3	1.5	0.158
0	0.05	clcB	3	1.5	0.166
0	0.05	ycaM	3	1.5	0.155
0	0.05	yadI	3	1.5	0.163
0	0.05	AG1	3	1.5	0.161
0	0.05	ptsI	3	1.75	0.159
0	0.05	clcB	3	1.75	0.163
0	0.05	ycaM	3	1.75	0.156
0	0.05	yadI	3	1.75	0.163
0	0.05	AG1	3	1.75	0.162
0	0.05	ptsI	3	2	0.161
0	0.05	clcB	3	2	0.162
0	0.05	ycaM	3	2	0.157
0	0.05	yadI	3	2	0.164
0	0.05	AG1	3	2	0.164
0	0.05	ptsI	3	2.25	0.162
0	0.05	clcB	3	2.25	0.164
0	0.05	ycaM	3	2.25	0.158
0	0.05	yadI	3	2.25	0.166
0	0.05	AG1	3	2.25	0.165
0	0.05	ptsI	3	2.5	0.162
0	0.05	clcB	3	2.5	0.166
0	0.05	ycaM	3	2.5	0.159
0	0.05	yadI	3	2.5	0.169
0	0.05	AG1	3	2.5	0.167
0	0.05	ptsI	3	2.75	0.165
0	0.05	clcB	3	2.75	0.168
0	0.05	ycaM	3	2.75	0.161
0	0.05	yadI	3	2.75	0.171
0	0.05	AG1	3	2.75	0.168
0	0.05	ptsI	3	3	0.165
0	0.05	clcB	3	3	0.168
0	0.05	ycaM	3	3	0.163
0	0.05	yadI	3	3	0.175
0	0.05	AG1	3	3	0.17
0	0.05	ptsI	3	3.25	0.168
0	0.05	clcB	3	3.25	0.171
0	0.05	ycaM	3	3.25	0.165
0	0.05	yadI	3	3.25	0.178
0	0.05	AG1	3	3.25	0.172
0	0.05	ptsI	3	3.5	0.171
0	0.05	clcB	3	3.5	0.171

0	0.05	ycaM	3	3.5	0.168
0	0.05	yadI	3	3.5	0.182
0	0.05	AG1	3	3.5	0.174
0	0.05	ptsI	3	3.75	0.173
0	0.05	clcB	3	3.75	0.175
0	0.05	ycaM	3	3.75	0.171
0	0.05	yadI	3	3.75	0.186
0	0.05	AG1	3	3.75	0.182
0	0.05	ptsI	3	4	0.177
0	0.05	clcB	3	4	0.178
0	0.05	ycaM	3	4	0.174
0	0.05	yadI	3	4	0.194
0	0.05	AG1	3	4	0.18
0	0.05	ptsI	3	4.41667	0.197
0	0.05	clcB	3	4.41667	0.196
0	0.05	ycaM	3	4.41667	0.183
0	0.05	yadI	3	4.41667	0.218
0	0.05	AG1	3	4.41667	0.208
0	0.05	ptsI	3	4.66667	0.197
0	0.05	clcB	3	4.66667	0.193
0	0.05	ycaM	3	4.66667	0.184
0	0.05	yadI	3	4.66667	0.226
0	0.05	AG1	3	4.66667	0.201
0	0.05	ptsI	3	4.91667	0.198
0	0.05	clcB	3	4.91667	0.197
0	0.05	ycaM	3	4.91667	0.19
0	0.05	yadI	3	4.91667	0.237
0	0.05	AG1	3	4.91667	0.208
0	0.05	ptsI	3	5.16667	0.203
0	0.05	clcB	3	5.16667	0.2
0	0.05	ycaM	3	5.16667	0.195
0	0.05	yadI	3	5.16667	0.252
0	0.05	AG1	3	5.16667	0.214
0	0.05	ptsI	3	5.41667	0.209
0	0.05	clcB	3	5.41667	0.207
0	0.05	ycaM	3	5.41667	0.201
0	0.05	yadI	3	5.41667	0.268
0	0.05	AG1	3	5.41667	0.221
0	0.05	ptsI	3	5.66667	0.212
0	0.05	clcB	3	5.66667	0.232
0	0.05	ycaM	3	5.66667	0.21
0	0.05	yadI	3	5.66667	0.287
0	0.05	AG1	3	5.66667	0.23
0	0.05	ptsI	3	5.91667	0.221
0	0.05	clcB	3	5.91667	0.22
0	0.05	ycaM	3	5.91667	0.218
0	0.05	yadI	3	5.91667	0.304
0	0.05	AG1	3	5.91667	0.241
0	0.05	ptsI	3	6.16667	0.228
0	0.05	clcB	3	6.16667	0.23
0	0.05	ycaM	3	6.16667	0.226
0	0.05	yadI	3	6.16667	0.318
0	0.05	AG1	3	6.16667	0.25

0	0.05	ptsI	3	6.41667	0.235
0	0.05	clcB	3	6.41667	0.236
0	0.05	ycaM	3	6.41667	0.233
0	0.05	yadI	3	6.41667	0.325
0	0.05	AG1	3	6.41667	0.264
0	0.05	ptsI	3	6.66667	0.246
0	0.05	clcB	3	6.66667	0.247
0	0.05	ycaM	3	6.66667	0.243
0	0.05	yadI	3	6.66667	0.338
0	0.05	AG1	3	6.66667	0.276
0	0.05	ptsI	3	6.91667	0.258
0	0.05	clcB	3	6.91667	0.261
0	0.05	ycaM	3	6.91667	0.252
0	0.05	yadI	3	6.91667	0.347
0	0.05	AG1	3	6.91667	0.29
0	0.05	ptsI	3	7.16667	0.263
0	0.05	clcB	3	7.16667	0.268
0	0.05	ycaM	3	7.16667	0.253
0	0.05	yadI	3	7.16667	0.357
0	0.05	AG1	3	7.16667	0.298
0	0.05	ptsI	3	7.41667	0.275
0	0.05	clcB	3	7.41667	0.278
0	0.05	ycaM	3	7.41667	0.259
0	0.05	yadI	3	7.41667	0.372
0	0.05	AG1	3	7.41667	0.308
0	0.05	ptsI	3	7.66667	0.28
0	0.05	clcB	3	7.66667	0.292
0	0.05	ycaM	3	7.66667	0.261
0	0.05	yadI	3	7.66667	0.385
0	0.05	AG1	3	7.66667	0.317
0	0.05	ptsI	3	7.91667	0.287
0	0.05	clcB	3	7.91667	0.29
0	0.05	ycaM	3	7.91667	0.264
0	0.05	yadI	3	7.91667	0.393
0	0.05	AG1	3	7.91667	0.323
0	0.05	ptsI	3	8.16667	0.296
0	0.05	clcB	3	8.16667	0.296
0	0.05	ycaM	3	8.16667	0.266
0	0.05	yadI	3	8.16667	0.403
0	0.05	AG1	3	8.16667	0.332
0	0.05	ptsI	3	8.41667	0.306
0	0.05	clcB	3	8.41667	0.305
0	0.05	ycaM	3	8.41667	0.269
0	0.05	yadI	3	8.41667	0.418
0	0.05	AG1	3	8.41667	0.343
0	0.05	ptsI	3	9.05	0.29
0	0.05	clcB	3	9.05	0.39
0	0.05	ycaM	3	9.05	0.295
0	0.05	yadI	3	9.05	0.433
0	0.05	AG1	3	9.05	0.374
0	0.05	ptsI	3	9.3	0.292
0	0.05	clcB	3	9.3	0.283
0	0.05	ycaM	3	9.3	0.29

0	0.05	yadI	3	9.3	0.442
0	0.05	AG1	3	9.3	0.378
0	0.05	ptsI	3	9.55	0.3
0	0.05	clcB	3	9.55	0.294
0	0.05	ycaM	3	9.55	0.296
0	0.05	yadI	3	9.55	0.456
0	0.05	AG1	3	9.55	0.388
0	0.05	ptsI	3	9.8	0.309
0	0.05	clcB	3	9.8	0.299
0	0.05	ycaM	3	9.8	0.298
0	0.05	yadI	3	9.8	0.472
0	0.05	AG1	3	9.8	0.396
0	0.05	ptsI	3	10.05	0.318
0	0.05	clcB	3	10.05	0.307
0	0.05	ycaM	3	10.05	0.304
0	0.05	yadI	3	10.05	0.486
0	0.05	AG1	3	10.05	0.409
0	0.05	ptsI	3	10.3	0.326
0	0.05	clcB	3	10.3	0.314
0	0.05	ycaM	3	10.3	0.306
0	0.05	yadI	3	10.3	0.498
0	0.05	AG1	3	10.3	0.422
0	0.05	ptsI	3	10.55	0.343
0	0.05	clcB	3	10.55	0.32
0	0.05	ycaM	3	10.55	0.311
0	0.05	yadI	3	10.55	0.516
0	0.05	AG1	3	10.55	0.435
0	0.05	ptsI	3	10.8	0.345
0	0.05	clcB	3	10.8	0.316
0	0.05	ycaM	3	10.8	0.309
0	0.05	yadI	3	10.8	0.522
0	0.05	AG1	3	10.8	0.442
0	0.05	ptsI	3	11.05	0.358
0	0.05	clcB	3	11.05	0.328
0	0.05	ycaM	3	11.05	0.315
0	0.05	yadI	3	11.05	0.538
0	0.05	AG1	3	11.05	0.458
0	0.05	ptsI	3	11.3	0.362
0	0.05	clcB	3	11.3	0.321
0	0.05	ycaM	3	11.3	0.31
0	0.05	yadI	3	11.3	0.54
0	0.05	AG1	3	11.3	0.462
0	0.05	ptsI	3	11.55	0.373
0	0.05	clcB	3	11.55	0.328
0	0.05	ycaM	3	11.55	0.312
0	0.05	yadI	3	11.55	0.557
0	0.05	AG1	3	11.55	0.476
0	0.05	ptsI	3	11.8	0.391
0	0.05	clcB	3	11.8	0.343
0	0.05	ycaM	3	11.8	0.322
0	0.05	yadI	3	11.8	0.579
0	0.05	AG1	3	11.8	0.494
0	0.05	ptsI	3	12.05	0.392

0	0.05	clcB	3	12.05	0.333
0	0.05	ycaM	3	12.05	0.315
0	0.05	yadI	3	12.05	0.578
0	0.05	AG1	3	12.05	0.495
0	0.05	ptsI	3	12.3	0.406
0	0.05	clcB	3	12.3	0.392
0	0.05	ycaM	3	12.3	0.325
0	0.05	yadI	3	12.3	0.596
0	0.05	AG1	3	12.3	0.512
0	0.05	ptsI	3	12.55	0.424
0	0.05	clcB	3	12.55	0.342
0	0.05	ycaM	3	12.55	0.326
0	0.05	yadI	3	12.55	0.62
0	0.05	AG1	3	12.55	0.532
0	0.05	ptsI	3	12.8	0.437
0	0.05	clcB	3	12.8	0.337
0	0.05	ycaM	3	12.8	0.328
0	0.05	yadI	3	12.8	0.635
0	0.05	AG1	3	12.8	0.548
0	0.05	ptsI	3	13.05	0.428
0	0.05	clcB	3	13.05	0.331
0	0.05	ycaM	3	13.05	0.316
0	0.05	yadI	3	13.05	0.627
0	0.05	AG1	3	13.05	0.549
0	0.05	ptsI	3	13.3	0.443
0	0.05	clcB	3	13.3	0.341
0	0.05	ycaM	3	13.3	0.326
0	0.05	yadI	3	13.3	0.645
0	0.05	AG1	3	13.3	0.574
0	0.05	ptsI	3	13.55	0.455
0	0.05	clcB	3	13.55	0.336
0	0.05	ycaM	3	13.55	0.322
0	0.05	yadI	3	13.55	0.657
0	0.05	AG1	3	13.55	0.586
0	0.05	ptsI	3	13.8	0.472
0	0.05	clcB	3	13.8	0.34
0	0.05	ycaM	3	13.8	0.325
0	0.05	yadI	3	13.8	0.67
0	0.05	AG1	3	13.8	0.604
0	0.05	ptsI	3	14.05	0.473
0	0.05	clcB	3	14.05	0.334
0	0.05	ycaM	3	14.05	0.326
0	0.05	yadI	3	14.05	0.676
0	0.05	AG1	3	14.05	0.616
0	0.05	ptsI	3	14.3	0.481
0	0.05	clcB	3	14.3	0.336
0	0.05	ycaM	3	14.3	0.319
0	0.05	yadI	3	14.3	0.682
0	0.05	AG1	3	14.3	0.626
0	0.05	ptsI	3	14.55	0.491
0	0.05	clcB	3	14.55	0.338
0	0.05	ycaM	3	14.55	0.317
0	0.05	yadI	3	14.55	0.693

0	0.05	AG1	3	14.55	0.637
0	0.05	ptsI	3	14.8	0.512
0	0.05	clcB	3	14.8	0.34
0	0.05	ycaM	3	14.8	0.333
0	0.05	yadI	3	14.8	0.721
0	0.05	AG1	3	14.8	0.656
0	0.05	ptsI	3	15.05	0.53
0	0.05	clcB	3	15.05	0.336
0	0.05	ycaM	3	15.05	0.334
0	0.05	yadI	3	15.05	0.734
0	0.05	AG1	3	15.05	0.657
0	0.05	ptsI	3	15.3	0.527
0	0.05	clcB	3	15.3	0.332
0	0.05	ycaM	3	15.3	0.325
0	0.05	yadI	3	15.3	0.737
0	0.05	AG1	3	15.3	0.668
0	0.05	ptsI	3	15.55	0.532
0	0.05	clcB	3	15.55	0.336
0	0.05	ycaM	3	15.55	0.328
0	0.05	yadI	3	15.55	0.745
0	0.05	AG1	3	15.55	0.684
0	0.05	ptsI	3	15.8	0.549
0	0.05	clcB	3	15.8	0.34
0	0.05	ycaM	3	15.8	0.327
0	0.05	yadI	3	15.8	0.766
0	0.05	AG1	3	15.8	0.693
0	0.05	ptsI	3	16.05	0.54
0	0.05	clcB	3	16.05	0.328
0	0.05	ycaM	3	16.05	0.322
0	0.05	yadI	3	16.05	0.756
0	0.05	AG1	3	16.05	0.686
0	0.05	ptsI	3	16.3	0.553
0	0.05	clcB	3	16.3	0.329
0	0.05	ycaM	3	16.3	0.318
0	0.05	yadI	3	16.3	0.766
0	0.05	AG1	3	16.3	0.706
0	0.05	ptsI	3	16.55	0.569
0	0.05	clcB	3	16.55	0.332
0	0.05	ycaM	3	16.55	0.322
0	0.05	yadI	3	16.55	0.786
0	0.05	AG1	3	16.55	0.707
0	0.05	ptsI	3	16.8	0.576
0	0.05	clcB	3	16.8	0.338
0	0.05	ycaM	3	16.8	0.32
0	0.05	yadI	3	16.8	0.807
0	0.05	AG1	3	16.8	0.734
0	0.05	ptsI	3	17.05	0.574
0	0.05	clcB	3	17.05	0.329
0	0.05	ycaM	3	17.05	0.316
0	0.05	yadI	3	17.05	0.809
0	0.05	AG1	3	17.05	0.72
0	0.05	ptsI	3	17.3	0.581
0	0.05	clcB	3	17.3	0.327

0	0.05	ycaM	3	17.3	0.323
0	0.05	yadI	3	17.3	0.822
0	0.05	AG1	3	17.3	0.724
0	0.05	ptsI	3	17.55	0.587
0	0.05	clcB	3	17.55	0.333
0	0.05	ycaM	3	17.55	0.318
0	0.05	yadI	3	17.55	0.828
0	0.05	AG1	3	17.55	0.734
0	0.05	ptsI	3	17.8	0.599
0	0.05	clcB	3	17.8	0.332
0	0.05	ycaM	3	17.8	0.321
0	0.05	yadI	3	17.8	0.838
0	0.05	AG1	3	17.8	0.76
0	0.05	ptsI	3	18.05	0.599
0	0.05	clcB	3	18.05	0.334
0	0.05	ycaM	3	18.05	0.316
0	0.05	yadI	3	18.05	0.837
0	0.05	AG1	3	18.05	0.748
0	0.05	ptsI	3	18.3	0.595
0	0.05	clcB	3	18.3	0.34
0	0.05	ycaM	3	18.3	0.318
0	0.05	yadI	3	18.3	0.856
0	0.05	AG1	3	18.3	0.755
0	0.05	ptsI	3	18.55	0.598
0	0.05	clcB	3	18.55	0.336
0	0.05	ycaM	3	18.55	0.318
0	0.05	yadI	3	18.55	0.86
0	0.05	AG1	3	18.55	0.755
0	0.05	ptsI	3	18.8	0.602
0	0.05	clcB	3	18.8	0.33
0	0.05	ycaM	3	18.8	0.319
0	0.05	yadI	3	18.8	0.866
0	0.05	AG1	3	18.8	0.774
0	0.05	ptsI	3	19.05	0.615
0	0.05	clcB	3	19.05	0.333
0	0.05	ycaM	3	19.05	0.32
0	0.05	yadI	3	19.05	0.881
0	0.05	AG1	3	19.05	0.805
0	0.05	ptsI	3	19.3	0.613
0	0.05	clcB	3	19.3	0.334
0	0.05	ycaM	3	19.3	0.323
0	0.05	yadI	3	19.3	0.885
0	0.05	AG1	3	19.3	0.802
0	0.05	ptsI	3	19.55	0.625
0	0.05	clcB	3	19.55	0.335
0	0.05	ycaM	3	19.55	0.321
0	0.05	yadI	3	19.55	0.903
0	0.05	AG1	3	19.55	0.822
0	0.05	ptsI	3	19.8	0.607
0	0.05	clcB	3	19.8	0.333
0	0.05	ycaM	3	19.8	0.32
0	0.05	yadI	3	19.8	0.89
0	0.05	AG1	3	19.8	0.83

0	0.05	ptsI	3	20.05	0.611
0	0.05	clcB	3	20.05	0.332
0	0.05	ycaM	3	20.05	0.325
0	0.05	yadI	3	20.05	0.9
0	0.05	AG1	3	20.05	0.839
0	0.05	ptsI	3	20.3	0.611
0	0.05	clcB	3	20.3	0.336
0	0.05	ycaM	3	20.3	0.323
0	0.05	yadI	3	20.3	0.917
0	0.05	AG1	3	20.3	0.839
0	0.05	ptsI	3	20.55	0.615
0	0.05	clcB	3	20.55	0.334
0	0.05	ycaM	3	20.55	0.327
0	0.05	yadI	3	20.55	0.915
0	0.05	AG1	3	20.55	0.849
0	0.05	ptsI	3	20.8	0.629
0	0.05	clcB	3	20.8	0.335
0	0.05	ycaM	3	20.8	0.329
0	0.05	yadI	3	20.8	0.947
0	0.05	AG1	3	20.8	0.857
0	0.05	ptsI	3	21.05	0.62
0	0.05	clcB	3	21.05	0.333
0	0.05	ycaM	3	21.05	0.332
0	0.05	yadI	3	21.05	0.933
0	0.05	AG1	3	21.05	0.865
0	0.05	ptsI	3	21.3	0.617
0	0.05	clcB	3	21.3	0.334
0	0.05	ycaM	3	21.3	0.334
0	0.05	yadI	3	21.3	0.942
0	0.05	AG1	3	21.3	0.879
0	0.05	ptsI	3	21.55	0.63
0	0.05	clcB	3	21.55	0.338
0	0.05	ycaM	3	21.55	0.334
0	0.05	yadI	3	21.55	0.947
0	0.05	AG1	3	21.55	0.888
0	0.05	ptsI	3	21.8	0.628
0	0.05	clcB	3	21.8	0.339
0	0.05	ycaM	3	21.8	0.333
0	0.05	yadI	3	21.8	0.959
0	0.05	AG1	3	21.8	0.896
0	0.05	ptsI	3	22.05	0.622
0	0.05	clcB	3	22.05	0.336
0	0.05	ycaM	3	22.05	0.336
0	0.05	yadI	3	22.05	0.964
0	0.05	AG1	3	22.05	0.894
0	0.05	ptsI	3	22.3	0.625
0	0.05	clcB	3	22.3	0.341
0	0.05	ycaM	3	22.3	0.341
0	0.05	yadI	3	22.3	0.971
0	0.05	AG1	3	22.3	0.902
0	0.05	ptsI	3	22.55	0.621
0	0.05	clcB	3	22.55	0.339
0	0.05	ycaM	3	22.55	0.342

0	0.05	yadI	3	22.55	0.976
0	0.05	AG1	3	22.55	0.913
0	0.05	ptsI	3	22.8	0.618
0	0.05	clcB	3	22.8	0.339
0	0.05	ycaM	3	22.8	0.344
0	0.05	yadI	3	22.8	0.986
0	0.05	AG1	3	22.8	0.916
0	0.05	ptsI	3	23.05	0.625
0	0.05	clcB	3	23.05	0.339
0	0.05	ycaM	3	23.05	0.349
0	0.05	yadI	3	23.05	0.997
0	0.05	AG1	3	23.05	0.919
0	0.05	ptsI	3	23.3	0.62
0	0.05	clcB	3	23.3	0.338
0	0.05	ycaM	3	23.3	0.354
0	0.05	yadI	3	23.3	1.001
0	0.05	AG1	3	23.3	0.924
0	0.05	ptsI	3	23.55	0.632
0	0.05	clcB	3	23.55	0.34
0	0.05	ycaM	3	23.55	0.369
0	0.05	yadI	3	23.55	1.018
0	0.05	AG1	3	23.55	0.937
0	0.05	ptsI	3	23.8	0.625
0	0.05	clcB	3	23.8	0.345
0	0.05	ycaM	3	23.8	0.366
0	0.05	yadI	3	23.8	1.024
0	0.05	AG1	3	23.8	0.937
0	0.05	ptsI	3	24.05	0.623
0	0.05	clcB	3	24.05	0.343
0	0.05	ycaM	3	24.05	0.369
0	0.05	yadI	3	24.05	1.034
0	0.05	AG1	3	24.05	0.943
0	0.05	ptsI	3	24.3	0.616
0	0.05	clcB	3	24.3	0.341
0	0.05	ycaM	3	24.3	0.373
0	0.05	yadI	3	24.3	1.028
0	0.05	AG1	3	24.3	0.935
0	0.05	ptsI	4	0	0.194
0	0.05	clcB	4	0	0.2
0	0.05	ycaM	4	0	0.224
0	0.05	yadI	4	0	0.21
0	0.05	AG1	4	0	0.197
0	0.05	ptsI	4	0.25	0.194
0	0.05	clcB	4	0.25	0.205
0	0.05	ycaM	4	0.25	0.214
0	0.05	yadI	4	0.25	0.205
0	0.05	AG1	4	0.25	0.196
0	0.05	ptsI	4	0.5	0.194
0	0.05	clcB	4	0.5	0.209
0	0.05	ycaM	4	0.5	0.212
0	0.05	yadI	4	0.5	0.206
0	0.05	AG1	4	0.5	0.198
0	0.05	ptsI	4	0.75	0.197

0	0.05	clcB	4	0.75	0.213
0	0.05	ycaM	4	0.75	0.219
0	0.05	yadI	4	0.75	0.209
0	0.05	AG1	4	0.75	0.202
0	0.05	ptsI	4	1	0.201
0	0.05	clcB	4	1	0.219
0	0.05	ycaM	4	1	0.223
0	0.05	yadI	4	1	0.212
0	0.05	AG1	4	1	0.207
0	0.05	ptsI	4	1.25	0.205
0	0.05	clcB	4	1.25	0.223
0	0.05	ycaM	4	1.25	0.227
0	0.05	yadI	4	1.25	0.218
0	0.05	AG1	4	1.25	0.211
0	0.05	ptsI	4	1.5	0.21
0	0.05	clcB	4	1.5	0.227
0	0.05	ycaM	4	1.5	0.235
0	0.05	yadI	4	1.5	0.224
0	0.05	AG1	4	1.5	0.215
0	0.05	ptsI	4	1.75	0.218
0	0.05	clcB	4	1.75	0.236
0	0.05	ycaM	4	1.75	0.242
0	0.05	yadI	4	1.75	0.232
0	0.05	AG1	4	1.75	0.222
0	0.05	ptsI	4	2	0.223
0	0.05	clcB	4	2	0.246
0	0.05	ycaM	4	2	0.252
0	0.05	yadI	4	2	0.239
0	0.05	AG1	4	2	0.234
0	0.05	ptsI	4	2.25	0.23
0	0.05	clcB	4	2.25	0.255
0	0.05	ycaM	4	2.25	0.26
0	0.05	yadI	4	2.25	0.248
0	0.05	AG1	4	2.25	0.238
0	0.05	ptsI	4	2.5	0.24
0	0.05	clcB	4	2.5	0.258
0	0.05	ycaM	4	2.5	0.269
0	0.05	yadI	4	2.5	0.257
0	0.05	AG1	4	2.5	0.248
0	0.05	ptsI	4	2.75	0.248
0	0.05	clcB	4	2.75	0.264
0	0.05	ycaM	4	2.75	0.28
0	0.05	yadI	4	2.75	0.264
0	0.05	AG1	4	2.75	0.259
0	0.05	ptsI	4	3	0.256
0	0.05	clcB	4	3	0.276
0	0.05	ycaM	4	3	0.29
0	0.05	yadI	4	3	0.274
0	0.05	AG1	4	3	0.272
0	0.05	ptsI	4	3.25	0.265
0	0.05	clcB	4	3.25	0.286
0	0.05	ycaM	4	3.25	0.301
0	0.05	yadI	4	3.25	0.285

0	0.05	AG1	4	3.25	0.281
0	0.05	ptsI	4	3.5	0.274
0	0.05	clcB	4	3.5	0.298
0	0.05	ycaM	4	3.5	0.313
0	0.05	yadI	4	3.5	0.298
0	0.05	AG1	4	3.5	0.292
0	0.05	ptsI	4	3.75	0.282
0	0.05	clcB	4	3.75	0.305
0	0.05	ycaM	4	3.75	0.321
0	0.05	yadI	4	3.75	0.305
0	0.05	AG1	4	3.75	0.299
0	0.05	ptsI	4	4	0.291
0	0.05	clcB	4	4	0.32
0	0.05	ycaM	4	4	0.34
0	0.05	yadI	4	4	0.321
0	0.05	AG1	4	4	0.314
0	0.05	ptsI	4	4.35	0.287
0	0.05	clcB	4	4.35	0.311
0	0.05	ycaM	4	4.35	0.343
0	0.05	yadI	4	4.35	0.316
0	0.05	AG1	4	4.35	0.293
0	0.05	ptsI	4	4.6	0.291
0	0.05	clcB	4	4.6	0.315
0	0.05	ycaM	4	4.6	0.336
0	0.05	yadI	4	4.6	0.323
0	0.05	AG1	4	4.6	0.307
0	0.05	ptsI	4	4.85	0.3
0	0.05	clcB	4	4.85	0.32
0	0.05	ycaM	4	4.85	0.332
0	0.05	yadI	4	4.85	0.338
0	0.05	AG1	4	4.85	0.316
0	0.05	ptsI	4	5.1	0.308
0	0.05	clcB	4	5.1	0.322
0	0.05	ycaM	4	5.1	0.333
0	0.05	yadI	4	5.1	0.35
0	0.05	AG1	4	5.1	0.328
0	0.05	ptsI	4	5.35	0.319
0	0.05	clcB	4	5.35	0.324
0	0.05	ycaM	4	5.35	0.334
0	0.05	yadI	4	5.35	0.363
0	0.05	AG1	4	5.35	0.338
0	0.05	ptsI	4	5.6	0.331
0	0.05	clcB	4	5.6	0.324
0	0.05	ycaM	4	5.6	0.33
0	0.05	yadI	4	5.6	0.374
0	0.05	AG1	4	5.6	0.349
0	0.05	ptsI	4	5.85	0.348
0	0.05	clcB	4	5.85	0.325
0	0.05	ycaM	4	5.85	0.33
0	0.05	yadI	4	5.85	0.387
0	0.05	AG1	4	5.85	0.359
0	0.05	ptsI	4	6.1	0.363
0	0.05	clcB	4	6.1	0.328

0	0.05	ycaM	4	6.1	0.332
0	0.05	yadI	4	6.1	0.398
0	0.05	AG1	4	6.1	0.368
0	0.05	ptsI	4	6.35	0.372
0	0.05	clcB	4	6.35	0.329
0	0.05	ycaM	4	6.35	0.331
0	0.05	yadI	4	6.35	0.411
0	0.05	AG1	4	6.35	0.377
0	0.05	ptsI	4	6.6	0.385
0	0.05	clcB	4	6.6	0.329
0	0.05	ycaM	4	6.6	0.328
0	0.05	yadI	4	6.6	0.417
0	0.05	AG1	4	6.6	0.384
0	0.05	ptsI	4	6.85	0.398
0	0.05	clcB	4	6.85	0.33
0	0.05	ycaM	4	6.85	0.328
0	0.05	yadI	4	6.85	0.424
0	0.05	AG1	4	6.85	0.397
0	0.05	ptsI	4	7.1	0.405
0	0.05	clcB	4	7.1	0.329
0	0.05	ycaM	4	7.1	0.328
0	0.05	yadI	4	7.1	0.431
0	0.05	AG1	4	7.1	0.406
0	0.05	ptsI	4	7.35	0.415
0	0.05	clcB	4	7.35	0.333
0	0.05	ycaM	4	7.35	0.33
0	0.05	yadI	4	7.35	0.439
0	0.05	AG1	4	7.35	0.416
0	0.05	ptsI	4	7.6	0.421
0	0.05	clcB	4	7.6	0.337
0	0.05	ycaM	4	7.6	0.334
0	0.05	yadI	4	7.6	0.446
0	0.05	AG1	4	7.6	0.423
0	0.05	ptsI	4	7.85	0.427
0	0.05	clcB	4	7.85	0.338
0	0.05	ycaM	4	7.85	0.339
0	0.05	yadI	4	7.85	0.452
0	0.05	AG1	4	7.85	0.43
0	0.05	ptsI	4	8.1	0.44
0	0.05	clcB	4	8.1	0.34
0	0.05	ycaM	4	8.1	0.342
0	0.05	yadI	4	8.1	0.461
0	0.05	AG1	4	8.1	0.44
0	0.05	ptsI	4	8.35	0.449
0	0.05	clcB	4	8.35	0.343
0	0.05	ycaM	4	8.35	0.345
0	0.05	yadI	4	8.35	0.466
0	0.05	AG1	4	8.35	0.448
0	0.05	ptsI	4	8.83333	0.427
0	0.05	clcB	4	8.83333	0.316
0	0.05	ycaM	4	8.83333	0.311
0	0.05	yadI	4	8.83333	0.51
0	0.05	AG1	4	8.83333	0.488

0	0.05	ptsI	4	9.08333	0.43
0	0.05	clcB	4	9.08333	0.314
0	0.05	ycaM	4	9.08333	0.316
0	0.05	yadI	4	9.08333	0.513
0	0.05	AG1	4	9.08333	0.49
0	0.05	ptsI	4	9.33333	0.438
0	0.05	clcB	4	9.33333	0.316
0	0.05	ycaM	4	9.33333	0.316
0	0.05	yadI	4	9.33333	0.518
0	0.05	AG1	4	9.33333	0.503
0	0.05	ptsI	4	9.58333	0.45
0	0.05	clcB	4	9.58333	0.327
0	0.05	ycaM	4	9.58333	0.321
0	0.05	yadI	4	9.58333	0.529
0	0.05	AG1	4	9.58333	0.513
0	0.05	ptsI	4	9.83333	0.459
0	0.05	clcB	4	9.83333	0.318
0	0.05	ycaM	4	9.83333	0.33
0	0.05	yadI	4	9.83333	0.544
0	0.05	AG1	4	9.83333	0.52
0	0.05	ptsI	4	10.0833	0.47
0	0.05	clcB	4	10.0833	0.32
0	0.05	ycaM	4	10.0833	0.328
0	0.05	yadI	4	10.0833	0.551
0	0.05	AG1	4	10.0833	0.526
0	0.05	ptsI	4	10.3333	0.481
0	0.05	clcB	4	10.3333	0.326
0	0.05	ycaM	4	10.3333	0.328
0	0.05	yadI	4	10.3333	0.554
0	0.05	AG1	4	10.3333	0.536
0	0.05	ptsI	4	10.5833	0.491
0	0.05	clcB	4	10.5833	0.324
0	0.05	ycaM	4	10.5833	0.331
0	0.05	yadI	4	10.5833	0.566
0	0.05	AG1	4	10.5833	0.544
0	0.05	ptsI	4	10.8333	0.493
0	0.05	clcB	4	10.8333	0.341
0	0.05	ycaM	4	10.8333	0.331
0	0.05	yadI	4	10.8333	0.57
0	0.05	AG1	4	10.8333	0.552
0	0.05	ptsI	4	11.0833	0.503
0	0.05	clcB	4	11.0833	0.329
0	0.05	ycaM	4	11.0833	0.328
0	0.05	yadI	4	11.0833	0.579
0	0.05	AG1	4	11.0833	0.559
0	0.05	ptsI	4	11.3333	0.513
0	0.05	clcB	4	11.3333	0.336
0	0.05	ycaM	4	11.3333	0.335
0	0.05	yadI	4	11.3333	0.586
0	0.05	AG1	4	11.3333	0.567
0	0.05	ptsI	4	11.5833	0.52
0	0.05	clcB	4	11.5833	0.327
0	0.05	ycaM	4	11.5833	0.335

0	0.05	yadI	4	11.5833	0.594
0	0.05	AG1	4	11.5833	0.575
0	0.05	ptsI	4	11.8333	0.528
0	0.05	clcB	4	11.8333	0.336
0	0.05	ycaM	4	11.8333	0.329
0	0.05	yadI	4	11.8333	0.598
0	0.05	AG1	4	11.8333	0.575
0	0.05	ptsI	4	12.0833	0.538
0	0.05	clcB	4	12.0833	0.322
0	0.05	ycaM	4	12.0833	0.329
0	0.05	yadI	4	12.0833	0.606
0	0.05	AG1	4	12.0833	0.582
0	0.05	ptsI	4	12.3333	0.544
0	0.05	clcB	4	12.3333	0.341
0	0.05	ycaM	4	12.3333	0.328
0	0.05	yadI	4	12.3333	0.608
0	0.05	AG1	4	12.3333	0.586
0	0.05	ptsI	4	12.5833	0.55
0	0.05	clcB	4	12.5833	0.338
0	0.05	ycaM	4	12.5833	0.336
0	0.05	yadI	4	12.5833	0.616
0	0.05	AG1	4	12.5833	0.593
0	0.05	ptsI	4	12.8333	0.557
0	0.05	clcB	4	12.8333	0.336
0	0.05	ycaM	4	12.8333	0.333
0	0.05	yadI	4	12.8333	0.621
0	0.05	AG1	4	12.8333	0.597
0	0.05	ptsI	4	13.0833	0.559
0	0.05	clcB	4	13.0833	0.342
0	0.05	ycaM	4	13.0833	0.334
0	0.05	yadI	4	13.0833	0.623
0	0.05	AG1	4	13.0833	0.6
0	0.05	ptsI	4	13.3333	0.571
0	0.05	clcB	4	13.3333	0.341
0	0.05	ycaM	4	13.3333	0.354
0	0.05	yadI	4	13.3333	0.629
0	0.05	AG1	4	13.3333	0.607
0	0.05	ptsI	4	13.5833	0.574
0	0.05	clcB	4	13.5833	0.34
0	0.05	ycaM	4	13.5833	0.354
0	0.05	yadI	4	13.5833	0.635
0	0.05	AG1	4	13.5833	0.61
0	0.05	ptsI	4	13.8333	0.583
0	0.05	clcB	4	13.8333	0.34
0	0.05	ycaM	4	13.8333	0.334
0	0.05	yadI	4	13.8333	0.636
0	0.05	AG1	4	13.8333	0.614
0	0.05	ptsI	4	14.0833	0.589
0	0.05	clcB	4	14.0833	0.338
0	0.05	ycaM	4	14.0833	0.33
0	0.05	yadI	4	14.0833	0.643
0	0.05	AG1	4	14.0833	0.616
0	0.05	ptsI	4	14.3333	0.597

0	0.05	clcB	4	14.3333	0.334
0	0.05	ycaM	4	14.3333	0.33
0	0.05	yadI	4	14.3333	0.651
0	0.05	AG1	4	14.3333	0.626
0	0.05	ptsI	4	14.5833	0.595
0	0.05	clcB	4	14.5833	0.342
0	0.05	ycaM	4	14.5833	0.332
0	0.05	yadI	4	14.5833	0.649
0	0.05	AG1	4	14.5833	0.62
0	0.05	ptsI	4	14.8333	0.604
0	0.05	clcB	4	14.8333	0.338
0	0.05	ycaM	4	14.8333	0.331
0	0.05	yadI	4	14.8333	0.657
0	0.05	AG1	4	14.8333	0.629
0	0.05	ptsI	4	15.0833	0.606
0	0.05	clcB	4	15.0833	0.34
0	0.05	ycaM	4	15.0833	0.33
0	0.05	yadI	4	15.0833	0.66
0	0.05	AG1	4	15.0833	0.628
0	0.05	ptsI	4	15.3333	0.622
0	0.05	clcB	4	15.3333	0.345
0	0.05	ycaM	4	15.3333	0.332
0	0.05	yadI	4	15.3333	0.667
0	0.05	AG1	4	15.3333	0.635
0	0.05	ptsI	4	15.5833	0.622
0	0.05	clcB	4	15.5833	0.341
0	0.05	ycaM	4	15.5833	0.335
0	0.05	yadI	4	15.5833	0.671
0	0.05	AG1	4	15.5833	0.636
0	0.05	ptsI	4	15.8333	0.629
0	0.05	clcB	4	15.8333	0.342
0	0.05	ycaM	4	15.8333	0.345
0	0.05	yadI	4	15.8333	0.678
0	0.05	AG1	4	15.8333	0.639
0	0.05	ptsI	4	16.0833	0.632
0	0.05	clcB	4	16.0833	0.342
0	0.05	ycaM	4	16.0833	0.35
0	0.05	yadI	4	16.0833	0.679
0	0.05	AG1	4	16.0833	0.641
0	0.05	ptsI	4	16.3333	0.635
0	0.05	clcB	4	16.3333	0.345
0	0.05	ycaM	4	16.3333	0.339
0	0.05	yadI	4	16.3333	0.682
0	0.05	AG1	4	16.3333	0.642
0	0.05	ptsI	4	16.5833	0.642
0	0.05	clcB	4	16.5833	0.349
0	0.05	ycaM	4	16.5833	0.341
0	0.05	yadI	4	16.5833	0.686
0	0.05	AG1	4	16.5833	0.646
0	0.05	ptsI	4	16.8333	0.637
0	0.05	clcB	4	16.8333	0.347
0	0.05	ycaM	4	16.8333	0.339
0	0.05	yadI	4	16.8333	0.687

0	0.05	AG1	4	16.8333	0.648
0	0.05	ptsI	4	17.0833	0.639
0	0.05	clcB	4	17.0833	0.349
0	0.05	ycaM	4	17.0833	0.344
0	0.05	yadI	4	17.0833	0.692
0	0.05	AG1	4	17.0833	0.65
0	0.05	ptsI	4	17.3333	0.65
0	0.05	clcB	4	17.3333	0.378
0	0.05	ycaM	4	17.3333	0.348
0	0.05	yadI	4	17.3333	0.698
0	0.05	AG1	4	17.3333	0.657
0	0.05	ptsI	4	17.5833	0.642
0	0.05	clcB	4	17.5833	0.362
0	0.05	ycaM	4	17.5833	0.348
0	0.05	yadI	4	17.5833	0.694
0	0.05	AG1	4	17.5833	0.651
0	0.05	ptsI	4	17.8333	0.64
0	0.05	clcB	4	17.8333	0.347
0	0.05	ycaM	4	17.8333	0.349
0	0.05	yadI	4	17.8333	0.697
0	0.05	AG1	4	17.8333	0.645
0	0.05	ptsI	4	18.0833	0.644
0	0.05	clcB	4	18.0833	0.351
0	0.05	ycaM	4	18.0833	0.36
0	0.05	yadI	4	18.0833	0.707
0	0.05	AG1	4	18.0833	0.659
0	0.05	ptsI	4	18.3333	0.649
0	0.05	clcB	4	18.3333	0.363
0	0.05	ycaM	4	18.3333	0.362
0	0.05	yadI	4	18.3333	0.707
0	0.05	AG1	4	18.3333	0.657
0	0.05	ptsI	4	18.5833	0.645
0	0.05	clcB	4	18.5833	0.352
0	0.05	ycaM	4	18.5833	0.365
0	0.05	yadI	4	18.5833	0.71
0	0.05	AG1	4	18.5833	0.663
0	0.05	ptsI	4	18.8333	0.645
0	0.05	clcB	4	18.8333	0.351
0	0.05	ycaM	4	18.8333	0.362
0	0.05	yadI	4	18.8333	0.714
0	0.05	AG1	4	18.8333	0.659
0	0.05	ptsI	4	19.0833	0.644
0	0.05	clcB	4	19.0833	0.354
0	0.05	ycaM	4	19.0833	0.366
0	0.05	yadI	4	19.0833	0.717
0	0.05	AG1	4	19.0833	0.667
0	0.05	ptsI	4	19.3333	0.658
0	0.05	clcB	4	19.3333	0.383
0	0.05	ycaM	4	19.3333	0.382
0	0.05	yadI	4	19.3333	0.724
0	0.05	AG1	4	19.3333	0.674
0	0.05	ptsI	4	19.5833	0.651
0	0.05	clcB	4	19.5833	0.351

0	0.05	ycaM	4	19.5833	0.375
0	0.05	yadI	4	19.5833	0.723
0	0.05	AG1	4	19.5833	0.67
0	0.05	ptsI	4	19.8333	0.652
0	0.05	clcB	4	19.8333	0.344
0	0.05	ycaM	4	19.8333	0.38
0	0.05	yadI	4	19.8333	0.717
0	0.05	AG1	4	19.8333	0.668
0	0.05	ptsI	4	20.0833	0.657
0	0.05	clcB	4	20.0833	0.378
0	0.05	ycaM	4	20.0833	0.394
0	0.05	yadI	4	20.0833	0.727
0	0.05	AG1	4	20.0833	0.678
0	0.05	ptsI	4	20.3333	0.654
0	0.05	clcB	4	20.3333	0.4
0	0.05	ycaM	4	20.3333	0.401
0	0.05	yadI	4	20.3333	0.722
0	0.05	AG1	4	20.3333	0.675
0	0.05	ptsI	4	20.5833	0.653
0	0.05	clcB	4	20.5833	0.378
0	0.05	ycaM	4	20.5833	0.395
0	0.05	yadI	4	20.5833	0.74
0	0.05	AG1	4	20.5833	0.681
0	0.05	ptsI	4	20.8333	0.657
0	0.05	clcB	4	20.8333	0.381
0	0.05	ycaM	4	20.8333	0.404
0	0.05	yadI	4	20.8333	0.73
0	0.05	AG1	4	20.8333	0.681
0	0.05	ptsI	4	21.0833	0.66
0	0.05	clcB	4	21.0833	0.396
0	0.05	ycaM	4	21.0833	0.405
0	0.05	yadI	4	21.0833	0.73
0	0.05	AG1	4	21.0833	0.68
0	0.05	ptsI	4	21.3333	0.656
0	0.05	clcB	4	21.3333	0.371
0	0.05	ycaM	4	21.3333	0.424
0	0.05	yadI	4	21.3333	0.732
0	0.05	AG1	4	21.3333	0.679
0	0.05	ptsI	4	21.5833	0.662
0	0.05	clcB	4	21.5833	0.365
0	0.05	ycaM	4	21.5833	0.42
0	0.05	yadI	4	21.5833	0.734
0	0.05	AG1	4	21.5833	0.68
0	0.05	ptsI	4	21.8333	0.659
0	0.05	clcB	4	21.8333	0.388
0	0.05	ycaM	4	21.8333	0.433
0	0.05	yadI	4	21.8333	0.744
0	0.05	AG1	4	21.8333	0.688
0	0.05	ptsI	4	22.0833	0.664
0	0.05	clcB	4	22.0833	0.392
0	0.05	ycaM	4	22.0833	0.43
0	0.05	yadI	4	22.0833	0.738
0	0.05	AG1	4	22.0833	0.686

0	0.05	ptsI	4	22.3333	0.663
0	0.05	clcB	4	22.3333	0.358
0	0.05	ycaM	4	22.3333	0.426
0	0.05	yadI	4	22.3333	0.74
0	0.05	AG1	4	22.3333	0.682
0	0.05	ptsI	4	22.5833	0.666
0	0.05	clcB	4	22.5833	0.354
0	0.05	ycaM	4	22.5833	0.43
0	0.05	yadI	4	22.5833	0.742
0	0.05	AG1	4	22.5833	0.688
0	0.05	ptsI	4	22.8333	0.655
0	0.05	clcB	4	22.8333	0.376
0	0.05	ycaM	4	22.8333	0.434
0	0.05	yadI	4	22.8333	0.743
0	0.05	AG1	4	22.8333	0.686
0	0.05	ptsI	4	23.0833	0.668
0	0.05	clcB	4	23.0833	0.37
0	0.05	ycaM	4	23.0833	0.43
0	0.05	yadI	4	23.0833	0.744
0	0.05	AG1	4	23.0833	0.688
0	0.05	ptsI	4	23.3333	0.673
0	0.05	clcB	4	23.3333	0.379
0	0.05	ycaM	4	23.3333	0.432
0	0.05	yadI	4	23.3333	0.75
0	0.05	AG1	4	23.3333	0.689
0	0.05	ptsI	4	23.5833	0.66
0	0.05	clcB	4	23.5833	0.389
0	0.05	ycaM	4	23.5833	0.433
0	0.05	yadI	4	23.5833	0.75
0	0.05	AG1	4	23.5833	0.69
0	0.05	ptsI	4	23.8333	0.665
0	0.05	clcB	4	23.8333	0.389
0	0.05	ycaM	4	23.8333	0.429
0	0.05	yadI	4	23.8333	0.752
0	0.05	AG1	4	23.8333	0.693
0	0.05	ptsI	4	24.0833	0.665
0	0.05	clcB	4	24.0833	0.394
0	0.05	ycaM	4	24.0833	0.424
0	0.05	yadI	4	24.0833	0.75
0	0.05	AG1	4	24.0833	0.691
0	0.05	ptsI	5	0	0.159
0	0.05	clcB	5	0	0.161
0	0.05	ycaM	5	0	0.16
0	0.05	yadI	5	0	0.188
0	0.05	AG1	5	0	0.16
0	0.05	ptsI	5	0.35	0.153
0	0.05	clcB	5	0.35	0.158
0	0.05	ycaM	5	0.35	0.157
0	0.05	yadI	5	0.35	0.16
0	0.05	AG1	5	0.35	0.16
0	0.05	ptsI	5	0.6	0.153
0	0.05	clcB	5	0.6	0.159
0	0.05	ycaM	5	0.6	0.157

0	0.05	yadI	5	0.6	0.163
0	0.05	AG1	5	0.6	0.159
0	0.05	ptsI	5	0.85	0.153
0	0.05	clcB	5	0.85	0.158
0	0.05	ycaM	5	0.85	0.157
0	0.05	yadI	5	0.85	0.168
0	0.05	AG1	5	0.85	0.159
0	0.05	ptsI	5	1.1	0.154
0	0.05	clcB	5	1.1	0.16
0	0.05	ycaM	5	1.1	0.158
0	0.05	yadI	5	1.1	0.166
0	0.05	AG1	5	1.1	0.161
0	0.05	ptsI	5	1.35	0.157
0	0.05	clcB	5	1.35	0.162
0	0.05	ycaM	5	1.35	0.163
0	0.05	yadI	5	1.35	0.187
0	0.05	AG1	5	1.35	0.164
0	0.05	ptsI	5	1.6	0.159
0	0.05	clcB	5	1.6	0.166
0	0.05	ycaM	5	1.6	0.165
0	0.05	yadI	5	1.6	0.174
0	0.05	AG1	5	1.6	0.166
0	0.05	ptsI	5	1.85	0.164
0	0.05	clcB	5	1.85	0.169
0	0.05	ycaM	5	1.85	0.168
0	0.05	yadI	5	1.85	0.178
0	0.05	AG1	5	1.85	0.17
0	0.05	ptsI	5	2.1	0.164
0	0.05	clcB	5	2.1	0.172
0	0.05	ycaM	5	2.1	0.171
0	0.05	yadI	5	2.1	0.183
0	0.05	AG1	5	2.1	0.172
0	0.05	ptsI	5	2.35	0.168
0	0.05	clcB	5	2.35	0.179
0	0.05	ycaM	5	2.35	0.175
0	0.05	yadI	5	2.35	0.188
0	0.05	AG1	5	2.35	0.176
0	0.05	ptsI	5	2.6	0.173
0	0.05	clcB	5	2.6	0.178
0	0.05	ycaM	5	2.6	0.181
0	0.05	yadI	5	2.6	0.198
0	0.05	AG1	5	2.6	0.181
0	0.05	ptsI	5	2.85	0.177
0	0.05	clcB	5	2.85	0.186
0	0.05	ycaM	5	2.85	0.187
0	0.05	yadI	5	2.85	0.202
0	0.05	AG1	5	2.85	0.186
0	0.05	ptsI	5	3.1	0.181
0	0.05	clcB	5	3.1	0.195
0	0.05	ycaM	5	3.1	0.192
0	0.05	yadI	5	3.1	0.211
0	0.05	AG1	5	3.1	0.192
0	0.05	ptsI	5	3.35	0.188

0	0.05	clcB	5	3.35	0.207
0	0.05	ycaM	5	3.35	0.203
0	0.05	yadI	5	3.35	0.223
0	0.05	AG1	5	3.35	0.198
0	0.05	ptsI	5	3.6	0.2
0	0.05	clcB	5	3.6	0.22
0	0.05	ycaM	5	3.6	0.212
0	0.05	yadI	5	3.6	0.229
0	0.05	AG1	5	3.6	0.207
0	0.05	ptsI	5	3.85	0.209
0	0.05	clcB	5	3.85	0.228
0	0.05	ycaM	5	3.85	0.221
0	0.05	yadI	5	3.85	0.239
0	0.05	AG1	5	3.85	0.215
0	0.05	ptsI	5	4.38333	0.254
0	0.05	clcB	5	4.38333	0.255
0	0.05	ycaM	5	4.38333	0.262
0	0.05	yadI	5	4.38333	0.319
0	0.05	AG1	5	4.38333	0.249
0	0.05	ptsI	5	4.63333	0.233
0	0.05	clcB	5	4.63333	0.252
0	0.05	ycaM	5	4.63333	0.266
0	0.05	yadI	5	4.63333	0.314
0	0.05	AG1	5	4.63333	0.266
0	0.05	ptsI	5	4.88333	0.234
0	0.05	clcB	5	4.88333	0.27
0	0.05	ycaM	5	4.88333	0.274
0	0.05	yadI	5	4.88333	0.332
0	0.05	AG1	5	4.88333	0.287
0	0.05	ptsI	5	5.13333	0.244
0	0.05	clcB	5	5.13333	0.281
0	0.05	ycaM	5	5.13333	0.278
0	0.05	yadI	5	5.13333	0.353
0	0.05	AG1	5	5.13333	0.302
0	0.05	ptsI	5	5.38333	0.254
0	0.05	clcB	5	5.38333	0.292
0	0.05	ycaM	5	5.38333	0.281
0	0.05	yadI	5	5.38333	0.377
0	0.05	AG1	5	5.38333	0.321
0	0.05	ptsI	5	5.63333	0.262
0	0.05	clcB	5	5.63333	0.303
0	0.05	ycaM	5	5.63333	0.286
0	0.05	yadI	5	5.63333	0.391
0	0.05	AG1	5	5.63333	0.335
0	0.05	ptsI	5	5.88333	0.271
0	0.05	clcB	5	5.88333	0.313
0	0.05	ycaM	5	5.88333	0.286
0	0.05	yadI	5	5.88333	0.407
0	0.05	AG1	5	5.88333	0.341
0	0.05	ptsI	5	6.13333	0.282
0	0.05	clcB	5	6.13333	0.319
0	0.05	ycaM	5	6.13333	0.289
0	0.05	yadI	5	6.13333	0.418

0	0.05	AG1	5	6.13333	0.356
0	0.05	ptsI	5	6.38333	0.295
0	0.05	clcB	5	6.38333	0.328
0	0.05	ycaM	5	6.38333	0.293
0	0.05	yadI	5	6.38333	0.436
0	0.05	AG1	5	6.38333	0.371
0	0.05	ptsI	5	6.63333	0.309
0	0.05	clcB	5	6.63333	0.332
0	0.05	ycaM	5	6.63333	0.297
0	0.05	yadI	5	6.63333	0.45
0	0.05	AG1	5	6.63333	0.384
0	0.05	ptsI	5	6.88333	0.323
0	0.05	clcB	5	6.88333	0.339
0	0.05	ycaM	5	6.88333	0.299
0	0.05	yadI	5	6.88333	0.466
0	0.05	AG1	5	6.88333	0.398
0	0.05	ptsI	5	7.13333	0.331
0	0.05	clcB	5	7.13333	0.345
0	0.05	ycaM	5	7.13333	0.3
0	0.05	yadI	5	7.13333	0.476
0	0.05	AG1	5	7.13333	0.412
0	0.05	ptsI	5	7.38333	0.343
0	0.05	clcB	5	7.38333	0.353
0	0.05	ycaM	5	7.38333	0.303
0	0.05	yadI	5	7.38333	0.49
0	0.05	AG1	5	7.38333	0.423
0	0.05	ptsI	5	7.63333	0.352
0	0.05	clcB	5	7.63333	0.359
0	0.05	ycaM	5	7.63333	0.306
0	0.05	yadI	5	7.63333	0.499
0	0.05	AG1	5	7.63333	0.437
0	0.05	ptsI	5	7.88333	0.366
0	0.05	clcB	5	7.88333	0.366
0	0.05	ycaM	5	7.88333	0.312
0	0.05	yadI	5	7.88333	0.512
0	0.05	AG1	5	7.88333	0.451
0	0.05	ptsI	5	8.13333	0.378
0	0.05	clcB	5	8.13333	0.369
0	0.05	ycaM	5	8.13333	0.312
0	0.05	yadI	5	8.13333	0.517
0	0.05	AG1	5	8.13333	0.466
0	0.05	ptsI	5	8.38333	0.396
0	0.05	clcB	5	8.38333	0.375
0	0.05	ycaM	5	8.38333	0.313
0	0.05	yadI	5	8.38333	0.525
0	0.05	AG1	5	8.38333	0.48
0	0.05	ptsI	5	8.88333	0.337
0	0.05	clcB	5	8.88333	0.294
0	0.05	ycaM	5	8.88333	0.27
0	0.05	yadI	5	8.88333	0.531
0	0.05	AG1	5	8.88333	0.386
0	0.05	ptsI	5	9.13333	0.34
0	0.05	clcB	5	9.13333	0.288

0	0.05	ycaM	5	9.13333	0.28
0	0.05	yadI	5	9.13333	0.533
0	0.05	AG1	5	9.13333	0.386
0	0.05	ptsI	5	9.38333	0.35
0	0.05	clcB	5	9.38333	0.294
0	0.05	ycaM	5	9.38333	0.283
0	0.05	yadI	5	9.38333	0.556
0	0.05	AG1	5	9.38333	0.402
0	0.05	ptsI	5	9.63333	0.362
0	0.05	clcB	5	9.63333	0.295
0	0.05	ycaM	5	9.63333	0.282
0	0.05	yadI	5	9.63333	0.564
0	0.05	AG1	5	9.63333	0.406
0	0.05	ptsI	5	9.88333	0.374
0	0.05	clcB	5	9.88333	0.296
0	0.05	ycaM	5	9.88333	0.283
0	0.05	yadI	5	9.88333	0.576
0	0.05	AG1	5	9.88333	0.42
0	0.05	ptsI	5	10.1333	0.386
0	0.05	clcB	5	10.1333	0.304
0	0.05	ycaM	5	10.1333	0.286
0	0.05	yadI	5	10.1333	0.581
0	0.05	AG1	5	10.1333	0.431
0	0.05	ptsI	5	10.3833	0.395
0	0.05	clcB	5	10.3833	0.305
0	0.05	ycaM	5	10.3833	0.282
0	0.05	yadI	5	10.3833	0.59
0	0.05	AG1	5	10.3833	0.441
0	0.05	ptsI	5	10.6333	0.403
0	0.05	clcB	5	10.6333	0.304
0	0.05	ycaM	5	10.6333	0.281
0	0.05	yadI	5	10.6333	0.598
0	0.05	AG1	5	10.6333	0.449
0	0.05	ptsI	5	10.8833	0.419
0	0.05	clcB	5	10.8833	0.307
0	0.05	ycaM	5	10.8833	0.281
0	0.05	yadI	5	10.8833	0.602
0	0.05	AG1	5	10.8833	0.458
0	0.05	ptsI	5	11.1333	0.424
0	0.05	clcB	5	11.1333	0.302
0	0.05	ycaM	5	11.1333	0.281
0	0.05	yadI	5	11.1333	0.611
0	0.05	AG1	5	11.1333	0.466
0	0.05	ptsI	5	11.3833	0.438
0	0.05	clcB	5	11.3833	0.31
0	0.05	ycaM	5	11.3833	0.283
0	0.05	yadI	5	11.3833	0.618
0	0.05	AG1	5	11.3833	0.473
0	0.05	ptsI	5	11.6333	0.448
0	0.05	clcB	5	11.6333	0.313
0	0.05	ycaM	5	11.6333	0.285
0	0.05	yadI	5	11.6333	0.625
0	0.05	AG1	5	11.6333	0.483

0	0.05	ptsI	5	11.8833	0.455
0	0.05	clcB	5	11.8833	0.311
0	0.05	ycaM	5	11.8833	0.285
0	0.05	yadI	5	11.8833	0.631
0	0.05	AG1	5	11.8833	0.489
0	0.05	ptsI	5	12.1333	0.47
0	0.05	clcB	5	12.1333	0.308
0	0.05	ycaM	5	12.1333	0.287
0	0.05	yadI	5	12.1333	0.638
0	0.05	AG1	5	12.1333	0.497
0	0.05	ptsI	5	12.3833	0.471
0	0.05	clcB	5	12.3833	0.299
0	0.05	ycaM	5	12.3833	0.288
0	0.05	yadI	5	12.3833	0.631
0	0.05	AG1	5	12.3833	0.5
0	0.05	ptsI	5	12.6333	0.486
0	0.05	clcB	5	12.6333	0.312
0	0.05	ycaM	5	12.6333	0.292
0	0.05	yadI	5	12.6333	0.648
0	0.05	AG1	5	12.6333	0.512
0	0.05	ptsI	5	12.8833	0.503
0	0.05	clcB	5	12.8833	0.305
0	0.05	ycaM	5	12.8833	0.294
0	0.05	yadI	5	12.8833	0.66
0	0.05	AG1	5	12.8833	0.517
0	0.05	ptsI	5	13.1333	0.506
0	0.05	clcB	5	13.1333	0.316
0	0.05	ycaM	5	13.1333	0.295
0	0.05	yadI	5	13.1333	0.662
0	0.05	AG1	5	13.1333	0.524
0	0.05	ptsI	5	13.3833	0.513
0	0.05	clcB	5	13.3833	0.312
0	0.05	ycaM	5	13.3833	0.291
0	0.05	yadI	5	13.3833	0.663
0	0.05	AG1	5	13.3833	0.528
0	0.05	ptsI	5	13.6333	0.523
0	0.05	clcB	5	13.6333	0.305
0	0.05	ycaM	5	13.6333	0.292
0	0.05	yadI	5	13.6333	0.671
0	0.05	AG1	5	13.6333	0.534
0	0.05	ptsI	5	13.8833	0.528
0	0.05	clcB	5	13.8833	0.303
0	0.05	ycaM	5	13.8833	0.295
0	0.05	yadI	5	13.8833	0.679
0	0.05	AG1	5	13.8833	0.54
0	0.05	ptsI	5	14.1333	0.537
0	0.05	clcB	5	14.1333	0.305
0	0.05	ycaM	5	14.1333	0.298
0	0.05	yadI	5	14.1333	0.682
0	0.05	AG1	5	14.1333	0.541
0	0.05	ptsI	5	14.3833	0.55
0	0.05	clcB	5	14.3833	0.304
0	0.05	ycaM	5	14.3833	0.3

0	0.05	yadI	5	14.3833	0.691
0	0.05	AG1	5	14.3833	0.548
0	0.05	ptsI	5	14.6333	0.563
0	0.05	clcB	5	14.6333	0.312
0	0.05	ycaM	5	14.6333	0.304
0	0.05	yadI	5	14.6333	0.693
0	0.05	AG1	5	14.6333	0.554
0	0.05	ptsI	5	14.8833	0.567
0	0.05	clcB	5	14.8833	0.312
0	0.05	ycaM	5	14.8833	0.3
0	0.05	yadI	5	14.8833	0.691
0	0.05	AG1	5	14.8833	0.556
0	0.05	ptsI	5	15.1333	0.578
0	0.05	clcB	5	15.1333	0.307
0	0.05	ycaM	5	15.1333	0.301
0	0.05	yadI	5	15.1333	0.696
0	0.05	AG1	5	15.1333	0.56
0	0.05	ptsI	5	15.3833	0.585
0	0.05	clcB	5	15.3833	0.31
0	0.05	ycaM	5	15.3833	0.303
0	0.05	yadI	5	15.3833	0.698
0	0.05	AG1	5	15.3833	0.563
0	0.05	ptsI	5	15.6333	0.592
0	0.05	clcB	5	15.6333	0.308
0	0.05	ycaM	5	15.6333	0.305
0	0.05	yadI	5	15.6333	0.702
0	0.05	AG1	5	15.6333	0.568
0	0.05	ptsI	5	15.8833	0.598
0	0.05	clcB	5	15.8833	0.307
0	0.05	ycaM	5	15.8833	0.306
0	0.05	yadI	5	15.8833	0.706
0	0.05	AG1	5	15.8833	0.57
0	0.05	ptsI	5	16.1333	0.597
0	0.05	clcB	5	16.1333	0.317
0	0.05	ycaM	5	16.1333	0.303
0	0.05	yadI	5	16.1333	0.713
0	0.05	AG1	5	16.1333	0.584
0	0.05	ptsI	5	16.3833	0.6
0	0.05	clcB	5	16.3833	0.309
0	0.05	ycaM	5	16.3833	0.3
0	0.05	yadI	5	16.3833	0.71
0	0.05	AG1	5	16.3833	0.574
0	0.05	ptsI	5	16.6333	0.607
0	0.05	clcB	5	16.6333	0.308
0	0.05	ycaM	5	16.6333	0.305
0	0.05	yadI	5	16.6333	0.715
0	0.05	AG1	5	16.6333	0.576
0	0.05	ptsI	5	16.8833	0.625
0	0.05	clcB	5	16.8833	0.312
0	0.05	ycaM	5	16.8833	0.304
0	0.05	yadI	5	16.8833	0.722
0	0.05	AG1	5	16.8833	0.583
0	0.05	ptsI	5	17.1333	0.624

0	0.05	clcB	5	17.1333	0.312
0	0.05	ycaM	5	17.1333	0.306
0	0.05	yadI	5	17.1333	0.72
0	0.05	AG1	5	17.1333	0.583
0	0.05	ptsI	5	17.3833	0.625
0	0.05	clcB	5	17.3833	0.308
0	0.05	ycaM	5	17.3833	0.306
0	0.05	yadI	5	17.3833	0.718
0	0.05	AG1	5	17.3833	0.583
0	0.05	ptsI	5	17.6333	0.629
0	0.05	clcB	5	17.6333	0.309
0	0.05	ycaM	5	17.6333	0.308
0	0.05	yadI	5	17.6333	0.719
0	0.05	AG1	5	17.6333	0.586
0	0.05	ptsI	5	17.8833	0.635
0	0.05	clcB	5	17.8833	0.31
0	0.05	ycaM	5	17.8833	0.302
0	0.05	yadI	5	17.8833	0.709
0	0.05	AG1	5	17.8833	0.586
0	0.05	ptsI	5	18.1333	0.65
0	0.05	clcB	5	18.1333	0.31
0	0.05	ycaM	5	18.1333	0.31
0	0.05	yadI	5	18.1333	0.727
0	0.05	AG1	5	18.1333	0.596
0	0.05	ptsI	5	18.3833	0.641
0	0.05	clcB	5	18.3833	0.313
0	0.05	ycaM	5	18.3833	0.309
0	0.05	yadI	5	18.3833	0.726
0	0.05	AG1	5	18.3833	0.594
0	0.05	ptsI	5	18.6333	0.644
0	0.05	clcB	5	18.6333	0.315
0	0.05	ycaM	5	18.6333	0.307
0	0.05	yadI	5	18.6333	0.733
0	0.05	AG1	5	18.6333	0.598
0	0.05	ptsI	5	18.8833	0.653
0	0.05	clcB	5	18.8833	0.307
0	0.05	ycaM	5	18.8833	0.313
0	0.05	yadI	5	18.8833	0.732
0	0.05	AG1	5	18.8833	0.597
0	0.05	ptsI	5	19.1333	0.651
0	0.05	clcB	5	19.1333	0.317
0	0.05	ycaM	5	19.1333	0.31
0	0.05	yadI	5	19.1333	0.743
0	0.05	AG1	5	19.1333	0.604
0	0.05	ptsI	5	19.3833	0.669
0	0.05	clcB	5	19.3833	0.316
0	0.05	ycaM	5	19.3833	0.318
0	0.05	yadI	5	19.3833	0.737
0	0.05	AG1	5	19.3833	0.607
0	0.05	ptsI	5	19.6333	0.668
0	0.05	clcB	5	19.6333	0.309
0	0.05	ycaM	5	19.6333	0.319
0	0.05	yadI	5	19.6333	0.736

0	0.05	AG1	5	19.6333	0.606
0	0.05	ptsI	5	19.8833	0.661
0	0.05	clcB	5	19.8833	0.323
0	0.05	ycaM	5	19.8833	0.319
0	0.05	yadI	5	19.8833	0.74
0	0.05	AG1	5	19.8833	0.608
0	0.05	ptsI	5	20.1333	0.661
0	0.05	clcB	5	20.1333	0.326
0	0.05	ycaM	5	20.1333	0.324
0	0.05	yadI	5	20.1333	0.744
0	0.05	AG1	5	20.1333	0.615
0	0.05	ptsI	5	20.3833	0.66
0	0.05	clcB	5	20.3833	0.327
0	0.05	ycaM	5	20.3833	0.327
0	0.05	yadI	5	20.3833	0.741
0	0.05	AG1	5	20.3833	0.616
0	0.05	ptsI	5	20.6333	0.665
0	0.05	clcB	5	20.6333	0.33
0	0.05	ycaM	5	20.6333	0.327
0	0.05	yadI	5	20.6333	0.727
0	0.05	AG1	5	20.6333	0.613
0	0.05	ptsI	5	20.8833	0.675
0	0.05	clcB	5	20.8833	0.328
0	0.05	ycaM	5	20.8833	0.338
0	0.05	yadI	5	20.8833	0.744
0	0.05	AG1	5	20.8833	0.618
0	0.05	ptsI	5	21.1333	0.668
0	0.05	clcB	5	21.1333	0.332
0	0.05	ycaM	5	21.1333	0.336
0	0.05	yadI	5	21.1333	0.751
0	0.05	AG1	5	21.1333	0.621
0	0.05	ptsI	5	21.3833	0.677
0	0.05	clcB	5	21.3833	0.335
0	0.05	ycaM	5	21.3833	0.343
0	0.05	yadI	5	21.3833	0.748
0	0.05	AG1	5	21.3833	0.618
0	0.05	ptsI	5	21.6333	0.671
0	0.05	clcB	5	21.6333	0.325
0	0.05	ycaM	5	21.6333	0.348
0	0.05	yadI	5	21.6333	0.749
0	0.05	AG1	5	21.6333	0.624
0	0.05	ptsI	5	21.8833	0.68
0	0.05	clcB	5	21.8833	0.341
0	0.05	ycaM	5	21.8833	0.347
0	0.05	yadI	5	21.8833	0.75
0	0.05	AG1	5	21.8833	0.629
0	0.05	ptsI	5	22.1333	0.668
0	0.05	clcB	5	22.1333	0.337
0	0.05	ycaM	5	22.1333	0.34
0	0.05	yadI	5	22.1333	0.731
0	0.05	AG1	5	22.1333	0.615
0	0.05	ptsI	5	22.3833	0.674
0	0.05	clcB	5	22.3833	0.34

0	0.05	ycaM	5	22.3833	0.353
0	0.05	yadI	5	22.3833	0.75
0	0.05	AG1	5	22.3833	0.622
0	0.05	ptsI	5	22.6333	0.664
0	0.05	clcB	5	22.6333	0.332
0	0.05	ycaM	5	22.6333	0.361
0	0.05	yadI	5	22.6333	0.753
0	0.05	AG1	5	22.6333	0.63
0	0.05	ptsI	5	22.8833	0.671
0	0.05	clcB	5	22.8833	0.344
0	0.05	ycaM	5	22.8833	0.366
0	0.05	yadI	5	22.8833	0.746
0	0.05	AG1	5	22.8833	0.631
0	0.05	ptsI	5	23.1333	0.659
0	0.05	clcB	5	23.1333	0.336
0	0.05	ycaM	5	23.1333	0.367
0	0.05	yadI	5	23.1333	0.754
0	0.05	AG1	5	23.1333	0.635
0	0.05	ptsI	5	23.3833	0.682
0	0.05	clcB	5	23.3833	0.361
0	0.05	ycaM	5	23.3833	0.364
0	0.05	yadI	5	23.3833	0.751
0	0.05	AG1	5	23.3833	0.63
0	0.05	ptsI	5	23.6333	0.677
0	0.05	clcB	5	23.6333	0.367
0	0.05	ycaM	5	23.6333	0.37
0	0.05	yadI	5	23.6333	0.751
0	0.05	AG1	5	23.6333	0.632
0	0.05	ptsI	5	23.8833	0.684
0	0.05	clcB	5	23.8833	0.373
0	0.05	ycaM	5	23.8833	0.363
0	0.05	yadI	5	23.8833	0.732
0	0.05	AG1	5	23.8833	0.627
0	0.05	ptsI	5	24.1333	0.667
0	0.05	clcB	5	24.1333	0.364
0	0.05	ycaM	5	24.1333	0.391
0	0.05	yadI	5	24.1333	0.752
0	0.05	AG1	5	24.1333	0.637
0	0.05	ptsI	5	24.3833	0.676
0	0.05	clcB	5	24.3833	0.373
0	0.05	ycaM	5	24.3833	0.372
0	0.05	yadI	5	24.3833	0.75
0	0.05	AG1	5	24.3833	0.63
0.05	0.05	ptsI	2	0	0.222
0.05	0.05	clcB	2	0	0.207
0.05	0.05	ycaM	2	0	0.214
0.05	0.05	yadI	2	0	0.213
0.05	0.05	AG1	2	0	0.225
0.05	0.05	ptsI	2	0.25	0.219
0.05	0.05	clcB	2	0.25	0.201
0.05	0.05	ycaM	2	0.25	0.209
0.05	0.05	yadI	2	0.25	0.206
0.05	0.05	AG1	2	0.25	0.216

0.05	0.05	ptsI	2	0.5	0.219
0.05	0.05	clcB	2	0.5	0.2
0.05	0.05	ycaM	2	0.5	0.208
0.05	0.05	yadI	2	0.5	0.205
0.05	0.05	AG1	2	0.5	0.214
0.05	0.05	ptsI	2	0.75	0.222
0.05	0.05	clcB	2	0.75	0.202
0.05	0.05	ycaM	2	0.75	0.213
0.05	0.05	yadI	2	0.75	0.209
0.05	0.05	AG1	2	0.75	0.216
0.05	0.05	ptsI	2	1	0.222
0.05	0.05	clcB	2	1	0.199
0.05	0.05	ycaM	2	1	0.216
0.05	0.05	yadI	2	1	0.213
0.05	0.05	AG1	2	1	0.223
0.05	0.05	ptsI	2	1.25	0.225
0.05	0.05	clcB	2	1.25	0.202
0.05	0.05	ycaM	2	1.25	0.22
0.05	0.05	yadI	2	1.25	0.221
0.05	0.05	AG1	2	1.25	0.226
0.05	0.05	ptsI	2	1.5	0.225
0.05	0.05	clcB	2	1.5	0.204
0.05	0.05	ycaM	2	1.5	0.223
0.05	0.05	yadI	2	1.5	0.225
0.05	0.05	AG1	2	1.5	0.227
0.05	0.05	ptsI	2	1.75	0.23
0.05	0.05	clcB	2	1.75	0.204
0.05	0.05	ycaM	2	1.75	0.228
0.05	0.05	yadI	2	1.75	0.232
0.05	0.05	AG1	2	1.75	0.232
0.05	0.05	ptsI	2	2	0.234
0.05	0.05	clcB	2	2	0.206
0.05	0.05	ycaM	2	2	0.234
0.05	0.05	yadI	2	2	0.237
0.05	0.05	AG1	2	2	0.242
0.05	0.05	ptsI	2	2.25	0.24
0.05	0.05	clcB	2	2.25	0.209
0.05	0.05	ycaM	2	2.25	0.238
0.05	0.05	yadI	2	2.25	0.245
0.05	0.05	AG1	2	2.25	0.249
0.05	0.05	ptsI	2	2.5	0.247
0.05	0.05	clcB	2	2.5	0.214
0.05	0.05	ycaM	2	2.5	0.248
0.05	0.05	yadI	2	2.5	0.263
0.05	0.05	AG1	2	2.5	0.261
0.05	0.05	ptsI	2	2.75	0.251
0.05	0.05	clcB	2	2.75	0.215
0.05	0.05	ycaM	2	2.75	0.253
0.05	0.05	yadI	2	2.75	0.269
0.05	0.05	AG1	2	2.75	0.267
0.05	0.05	ptsI	2	3	0.257
0.05	0.05	clcB	2	3	0.217
0.05	0.05	ycaM	2	3	0.256

0.05	0.05	yadI	2	3	0.278
0.05	0.05	AG1	2	3	0.265
0.05	0.05	ptsI	2	3.25	0.267
0.05	0.05	clcB	2	3.25	0.225
0.05	0.05	ycaM	2	3.25	0.266
0.05	0.05	yadI	2	3.25	0.292
0.05	0.05	AG1	2	3.25	0.282
0.05	0.05	ptsI	2	3.5	0.271
0.05	0.05	clcB	2	3.5	0.23
0.05	0.05	ycaM	2	3.5	0.273
0.05	0.05	yadI	2	3.5	0.304
0.05	0.05	AG1	2	3.5	0.287
0.05	0.05	ptsI	2	3.75	0.279
0.05	0.05	clcB	2	3.75	0.231
0.05	0.05	ycaM	2	3.75	0.282
0.05	0.05	yadI	2	3.75	0.315
0.05	0.05	AG1	2	3.75	0.3
0.05	0.05	ptsI	2	4	0.286
0.05	0.05	clcB	2	4	0.252
0.05	0.05	ycaM	2	4	0.293
0.05	0.05	yadI	2	4	0.328
0.05	0.05	AG1	2	4	0.315
0.05	0.05	ptsI	2	4.38333	0.292
0.05	0.05	clcB	2	4.38333	0.254
0.05	0.05	ycaM	2	4.38333	0.294
0.05	0.05	yadI	2	4.38333	0.38
0.05	0.05	AG1	2	4.38333	0.287
0.05	0.05	ptsI	2	4.63333	0.287
0.05	0.05	clcB	2	4.63333	0.248
0.05	0.05	ycaM	2	4.63333	0.29
0.05	0.05	yadI	2	4.63333	0.389
0.05	0.05	AG1	2	4.63333	0.289
0.05	0.05	ptsI	2	4.88333	0.285
0.05	0.05	clcB	2	4.88333	0.251
0.05	0.05	ycaM	2	4.88333	0.299
0.05	0.05	yadI	2	4.88333	0.404
0.05	0.05	AG1	2	4.88333	0.299
0.05	0.05	ptsI	2	5.13333	0.287
0.05	0.05	clcB	2	5.13333	0.255
0.05	0.05	ycaM	2	5.13333	0.298
0.05	0.05	yadI	2	5.13333	0.417
0.05	0.05	AG1	2	5.13333	0.306
0.05	0.05	ptsI	2	5.38333	0.29
0.05	0.05	clcB	2	5.38333	0.261
0.05	0.05	ycaM	2	5.38333	0.3
0.05	0.05	yadI	2	5.38333	0.434
0.05	0.05	AG1	2	5.38333	0.318
0.05	0.05	ptsI	2	5.63333	0.296
0.05	0.05	clcB	2	5.63333	0.269
0.05	0.05	ycaM	2	5.63333	0.301
0.05	0.05	yadI	2	5.63333	0.444
0.05	0.05	AG1	2	5.63333	0.324
0.05	0.05	ptsI	2	5.88333	0.301

0.05	0.05	clcB	2	5.88333	0.276
0.05	0.05	ycaM	2	5.88333	0.303
0.05	0.05	yadI	2	5.88333	0.455
0.05	0.05	AG1	2	5.88333	0.333
0.05	0.05	ptsI	2	6.13333	0.305
0.05	0.05	clcB	2	6.13333	0.283
0.05	0.05	ycaM	2	6.13333	0.302
0.05	0.05	yadI	2	6.13333	0.465
0.05	0.05	AG1	2	6.13333	0.341
0.05	0.05	ptsI	2	6.38333	0.316
0.05	0.05	clcB	2	6.38333	0.286
0.05	0.05	ycaM	2	6.38333	0.305
0.05	0.05	yadI	2	6.38333	0.47
0.05	0.05	AG1	2	6.38333	0.348
0.05	0.05	ptsI	2	6.63333	0.319
0.05	0.05	clcB	2	6.63333	0.294
0.05	0.05	ycaM	2	6.63333	0.308
0.05	0.05	yadI	2	6.63333	0.48
0.05	0.05	AG1	2	6.63333	0.358
0.05	0.05	ptsI	2	6.88333	0.328
0.05	0.05	clcB	2	6.88333	0.299
0.05	0.05	ycaM	2	6.88333	0.311
0.05	0.05	yadI	2	6.88333	0.492
0.05	0.05	AG1	2	6.88333	0.369
0.05	0.05	ptsI	2	7.13333	0.332
0.05	0.05	clcB	2	7.13333	0.301
0.05	0.05	ycaM	2	7.13333	0.307
0.05	0.05	yadI	2	7.13333	0.494
0.05	0.05	AG1	2	7.13333	0.374
0.05	0.05	ptsI	2	7.38333	0.342
0.05	0.05	clcB	2	7.38333	0.308
0.05	0.05	ycaM	2	7.38333	0.308
0.05	0.05	yadI	2	7.38333	0.503
0.05	0.05	AG1	2	7.38333	0.382
0.05	0.05	ptsI	2	7.63333	0.347
0.05	0.05	clcB	2	7.63333	0.311
0.05	0.05	ycaM	2	7.63333	0.31
0.05	0.05	yadI	2	7.63333	0.513
0.05	0.05	AG1	2	7.63333	0.393
0.05	0.05	ptsI	2	7.88333	0.354
0.05	0.05	clcB	2	7.88333	0.315
0.05	0.05	ycaM	2	7.88333	0.311
0.05	0.05	yadI	2	7.88333	0.529
0.05	0.05	AG1	2	7.88333	0.399
0.05	0.05	ptsI	2	8.13333	0.362
0.05	0.05	clcB	2	8.13333	0.317
0.05	0.05	ycaM	2	8.13333	0.311
0.05	0.05	yadI	2	8.13333	0.528
0.05	0.05	AG1	2	8.13333	0.41
0.05	0.05	ptsI	2	8.38333	0.372
0.05	0.05	clcB	2	8.38333	0.322
0.05	0.05	ycaM	2	8.38333	0.311
0.05	0.05	yadI	2	8.38333	0.538

0.05	0.05	AG1	2	8.38333	0.417
0.05	0.05	ptsI	2	8.91667	0.369
0.05	0.05	clcB	2	8.91667	0.293
0.05	0.05	ycaM	2	8.91667	0.276
0.05	0.05	yadI	2	8.91667	0.563
0.05	0.05	AG1	2	8.91667	0.46
0.05	0.05	ptsI	2	9.16667	0.374
0.05	0.05	clcB	2	9.16667	0.286
0.05	0.05	ycaM	2	9.16667	0.275
0.05	0.05	yadI	2	9.16667	0.574
0.05	0.05	AG1	2	9.16667	0.466
0.05	0.05	ptsI	2	9.41667	0.381
0.05	0.05	clcB	2	9.41667	0.298
0.05	0.05	ycaM	2	9.41667	0.279
0.05	0.05	yadI	2	9.41667	0.59
0.05	0.05	AG1	2	9.41667	0.48
0.05	0.05	ptsI	2	9.66667	0.389
0.05	0.05	clcB	2	9.66667	0.303
0.05	0.05	ycaM	2	9.66667	0.283
0.05	0.05	yadI	2	9.66667	0.605
0.05	0.05	AG1	2	9.66667	0.494
0.05	0.05	ptsI	2	9.91667	0.394
0.05	0.05	clcB	2	9.91667	0.309
0.05	0.05	ycaM	2	9.91667	0.282
0.05	0.05	yadI	2	9.91667	0.619
0.05	0.05	AG1	2	9.91667	0.51
0.05	0.05	ptsI	2	10.1667	0.401
0.05	0.05	clcB	2	10.1667	0.314
0.05	0.05	ycaM	2	10.1667	0.285
0.05	0.05	yadI	2	10.1667	0.631
0.05	0.05	AG1	2	10.1667	0.517
0.05	0.05	ptsI	2	10.4167	0.411
0.05	0.05	clcB	2	10.4167	0.315
0.05	0.05	ycaM	2	10.4167	0.286
0.05	0.05	yadI	2	10.4167	0.644
0.05	0.05	AG1	2	10.4167	0.529
0.05	0.05	ptsI	2	10.6667	0.414
0.05	0.05	clcB	2	10.6667	0.324
0.05	0.05	ycaM	2	10.6667	0.288
0.05	0.05	yadI	2	10.6667	0.656
0.05	0.05	AG1	2	10.6667	0.54
0.05	0.05	ptsI	2	10.9167	0.423
0.05	0.05	clcB	2	10.9167	0.325
0.05	0.05	ycaM	2	10.9167	0.286
0.05	0.05	yadI	2	10.9167	0.664
0.05	0.05	AG1	2	10.9167	0.546
0.05	0.05	ptsI	2	11.1667	0.433
0.05	0.05	clcB	2	11.1667	0.346
0.05	0.05	ycaM	2	11.1667	0.29
0.05	0.05	yadI	2	11.1667	0.673
0.05	0.05	AG1	2	11.1667	0.564
0.05	0.05	ptsI	2	11.4167	0.439
0.05	0.05	clcB	2	11.4167	0.334

0.05	0.05	ycaM	2	11.4167	0.286
0.05	0.05	yadI	2	11.4167	0.682
0.05	0.05	AG1	2	11.4167	0.588
0.05	0.05	ptsI	2	11.6667	0.446
0.05	0.05	clcB	2	11.6667	0.327
0.05	0.05	ycaM	2	11.6667	0.287
0.05	0.05	yadI	2	11.6667	0.693
0.05	0.05	AG1	2	11.6667	0.622
0.05	0.05	ptsI	2	11.9167	0.452
0.05	0.05	clcB	2	11.9167	0.336
0.05	0.05	ycaM	2	11.9167	0.29
0.05	0.05	yadI	2	11.9167	0.699
0.05	0.05	AG1	2	11.9167	0.617
0.05	0.05	ptsI	2	12.1667	0.459
0.05	0.05	clcB	2	12.1667	0.33
0.05	0.05	ycaM	2	12.1667	0.289
0.05	0.05	yadI	2	12.1667	0.71
0.05	0.05	AG1	2	12.1667	0.656
0.05	0.05	ptsI	2	12.4167	0.47
0.05	0.05	clcB	2	12.4167	0.33
0.05	0.05	ycaM	2	12.4167	0.286
0.05	0.05	yadI	2	12.4167	0.72
0.05	0.05	AG1	2	12.4167	0.664
0.05	0.05	ptsI	2	12.6667	0.471
0.05	0.05	clcB	2	12.6667	0.329
0.05	0.05	ycaM	2	12.6667	0.286
0.05	0.05	yadI	2	12.6667	0.723
0.05	0.05	AG1	2	12.6667	0.672
0.05	0.05	ptsI	2	12.9167	0.482
0.05	0.05	clcB	2	12.9167	0.333
0.05	0.05	ycaM	2	12.9167	0.29
0.05	0.05	yadI	2	12.9167	0.738
0.05	0.05	AG1	2	12.9167	0.662
0.05	0.05	ptsI	2	13.1667	0.481
0.05	0.05	clcB	2	13.1667	0.334
0.05	0.05	ycaM	2	13.1667	0.291
0.05	0.05	yadI	2	13.1667	0.749
0.05	0.05	AG1	2	13.1667	0.662
0.05	0.05	ptsI	2	13.4167	0.485
0.05	0.05	clcB	2	13.4167	0.332
0.05	0.05	ycaM	2	13.4167	0.289
0.05	0.05	yadI	2	13.4167	0.752
0.05	0.05	AG1	2	13.4167	0.659
0.05	0.05	ptsI	2	13.6667	0.491
0.05	0.05	clcB	2	13.6667	0.338
0.05	0.05	ycaM	2	13.6667	0.289
0.05	0.05	yadI	2	13.6667	0.762
0.05	0.05	AG1	2	13.6667	0.684
0.05	0.05	ptsI	2	13.9167	0.493
0.05	0.05	clcB	2	13.9167	0.331
0.05	0.05	ycaM	2	13.9167	0.289
0.05	0.05	yadI	2	13.9167	0.77
0.05	0.05	AG1	2	13.9167	0.663

0.05	0.05	ptsI	2	14.1667	0.502
0.05	0.05	clcB	2	14.1667	0.339
0.05	0.05	ycaM	2	14.1667	0.29
0.05	0.05	yadI	2	14.1667	0.776
0.05	0.05	AG1	2	14.1667	0.705
0.05	0.05	ptsI	2	14.4167	0.504
0.05	0.05	clcB	2	14.4167	0.332
0.05	0.05	ycaM	2	14.4167	0.286
0.05	0.05	yadI	2	14.4167	0.78
0.05	0.05	AG1	2	14.4167	0.674
0.05	0.05	ptsI	2	14.6667	0.502
0.05	0.05	clcB	2	14.6667	0.334
0.05	0.05	ycaM	2	14.6667	0.284
0.05	0.05	yadI	2	14.6667	0.782
0.05	0.05	AG1	2	14.6667	0.684
0.05	0.05	ptsI	2	14.9167	0.511
0.05	0.05	clcB	2	14.9167	0.338
0.05	0.05	ycaM	2	14.9167	0.285
0.05	0.05	yadI	2	14.9167	0.788
0.05	0.05	AG1	2	14.9167	0.692
0.05	0.05	ptsI	2	15.1667	0.515
0.05	0.05	clcB	2	15.1667	0.333
0.05	0.05	ycaM	2	15.1667	0.286
0.05	0.05	yadI	2	15.1667	0.801
0.05	0.05	AG1	2	15.1667	0.709
0.05	0.05	ptsI	2	15.4167	0.525
0.05	0.05	clcB	2	15.4167	0.332
0.05	0.05	ycaM	2	15.4167	0.282
0.05	0.05	yadI	2	15.4167	0.803
0.05	0.05	AG1	2	15.4167	0.701
0.05	0.05	ptsI	2	15.6667	0.521
0.05	0.05	clcB	2	15.6667	0.334
0.05	0.05	ycaM	2	15.6667	0.282
0.05	0.05	yadI	2	15.6667	0.799
0.05	0.05	AG1	2	15.6667	0.702
0.05	0.05	ptsI	2	15.9167	0.519
0.05	0.05	clcB	2	15.9167	0.334
0.05	0.05	ycaM	2	15.9167	0.281
0.05	0.05	yadI	2	15.9167	0.801
0.05	0.05	AG1	2	15.9167	0.708
0.05	0.05	ptsI	2	16.1667	0.529
0.05	0.05	clcB	2	16.1667	0.346
0.05	0.05	ycaM	2	16.1667	0.281
0.05	0.05	yadI	2	16.1667	0.821
0.05	0.05	AG1	2	16.1667	0.727
0.05	0.05	ptsI	2	16.4167	0.526
0.05	0.05	clcB	2	16.4167	0.332
0.05	0.05	ycaM	2	16.4167	0.284
0.05	0.05	yadI	2	16.4167	0.818
0.05	0.05	AG1	2	16.4167	0.719
0.05	0.05	ptsI	2	16.6667	0.523
0.05	0.05	clcB	2	16.6667	0.332
0.05	0.05	ycaM	2	16.6667	0.288

0.05	0.05	yadI	2	16.6667	0.822
0.05	0.05	AG1	2	16.6667	0.717
0.05	0.05	ptsI	2	16.9167	0.521
0.05	0.05	clcB	2	16.9167	0.338
0.05	0.05	ycaM	2	16.9167	0.287
0.05	0.05	yadI	2	16.9167	0.826
0.05	0.05	AG1	2	16.9167	0.732
0.05	0.05	ptsI	2	17.1667	0.53
0.05	0.05	clcB	2	17.1667	0.338
0.05	0.05	ycaM	2	17.1667	0.287
0.05	0.05	yadI	2	17.1667	0.839
0.05	0.05	AG1	2	17.1667	0.754
0.05	0.05	ptsI	2	17.4167	0.533
0.05	0.05	clcB	2	17.4167	0.337
0.05	0.05	ycaM	2	17.4167	0.289
0.05	0.05	yadI	2	17.4167	0.843
0.05	0.05	AG1	2	17.4167	0.764
0.05	0.05	ptsI	2	17.6667	0.537
0.05	0.05	clcB	2	17.6667	0.335
0.05	0.05	ycaM	2	17.6667	0.289
0.05	0.05	yadI	2	17.6667	0.845
0.05	0.05	AG1	2	17.6667	0.774
0.05	0.05	ptsI	2	17.9167	0.53
0.05	0.05	clcB	2	17.9167	0.346
0.05	0.05	ycaM	2	17.9167	0.293
0.05	0.05	yadI	2	17.9167	0.844
0.05	0.05	AG1	2	17.9167	0.763
0.05	0.05	ptsI	2	18.1667	0.54
0.05	0.05	clcB	2	18.1667	0.329
0.05	0.05	ycaM	2	18.1667	0.29
0.05	0.05	yadI	2	18.1667	0.848
0.05	0.05	AG1	2	18.1667	0.775
0.05	0.05	ptsI	2	18.4167	0.542
0.05	0.05	clcB	2	18.4167	0.333
0.05	0.05	ycaM	2	18.4167	0.294
0.05	0.05	yadI	2	18.4167	0.856
0.05	0.05	AG1	2	18.4167	0.775
0.05	0.05	ptsI	2	18.6667	0.535
0.05	0.05	clcB	2	18.6667	0.336
0.05	0.05	ycaM	2	18.6667	0.291
0.05	0.05	yadI	2	18.6667	0.853
0.05	0.05	AG1	2	18.6667	0.776
0.05	0.05	ptsI	2	18.9167	0.521
0.05	0.05	clcB	2	18.9167	0.33
0.05	0.05	ycaM	2	18.9167	0.294
0.05	0.05	yadI	2	18.9167	0.844
0.05	0.05	AG1	2	18.9167	0.778
0.05	0.05	ptsI	2	19.1667	0.542
0.05	0.05	clcB	2	19.1667	0.327
0.05	0.05	ycaM	2	19.1667	0.292
0.05	0.05	yadI	2	19.1667	0.857
0.05	0.05	AG1	2	19.1667	0.794
0.05	0.05	ptsI	2	19.4167	0.534

0.05	0.05	clcB	2	19.4167	0.332
0.05	0.05	ycaM	2	19.4167	0.298
0.05	0.05	yadI	2	19.4167	0.848
0.05	0.05	AG1	2	19.4167	0.793
0.05	0.05	ptsI	2	19.6667	0.538
0.05	0.05	clcB	2	19.6667	0.33
0.05	0.05	ycaM	2	19.6667	0.298
0.05	0.05	yadI	2	19.6667	0.863
0.05	0.05	AG1	2	19.6667	0.796
0.05	0.05	ptsI	2	19.9167	0.539
0.05	0.05	clcB	2	19.9167	0.328
0.05	0.05	ycaM	2	19.9167	0.3
0.05	0.05	yadI	2	19.9167	0.864
0.05	0.05	AG1	2	19.9167	0.819
0.05	0.05	ptsI	2	20.1667	0.539
0.05	0.05	clcB	2	20.1667	0.329
0.05	0.05	ycaM	2	20.1667	0.304
0.05	0.05	yadI	2	20.1667	0.865
0.05	0.05	AG1	2	20.1667	0.806
0.05	0.05	ptsI	2	20.4167	0.541
0.05	0.05	clcB	2	20.4167	0.329
0.05	0.05	ycaM	2	20.4167	0.304
0.05	0.05	yadI	2	20.4167	0.864
0.05	0.05	AG1	2	20.4167	0.811
0.05	0.05	ptsI	2	20.6667	0.536
0.05	0.05	clcB	2	20.6667	0.328
0.05	0.05	ycaM	2	20.6667	0.307
0.05	0.05	yadI	2	20.6667	0.861
0.05	0.05	AG1	2	20.6667	0.811
0.05	0.05	ptsI	2	20.9167	0.536
0.05	0.05	clcB	2	20.9167	0.327
0.05	0.05	ycaM	2	20.9167	0.309
0.05	0.05	yadI	2	20.9167	0.864
0.05	0.05	AG1	2	20.9167	0.813
0.05	0.05	ptsI	2	21.1667	0.534
0.05	0.05	clcB	2	21.1667	0.327
0.05	0.05	ycaM	2	21.1667	0.312
0.05	0.05	yadI	2	21.1667	0.877
0.05	0.05	AG1	2	21.1667	0.819
0.05	0.05	ptsI	2	21.4167	0.527
0.05	0.05	clcB	2	21.4167	0.325
0.05	0.05	ycaM	2	21.4167	0.316
0.05	0.05	yadI	2	21.4167	0.87
0.05	0.05	AG1	2	21.4167	0.832
0.05	0.05	ptsI	2	21.6667	0.538
0.05	0.05	clcB	2	21.6667	0.329
0.05	0.05	ycaM	2	21.6667	0.32
0.05	0.05	yadI	2	21.6667	0.874
0.05	0.05	AG1	2	21.6667	0.835
0.05	0.05	ptsI	2	21.9167	0.538
0.05	0.05	clcB	2	21.9167	0.329
0.05	0.05	ycaM	2	21.9167	0.32
0.05	0.05	yadI	2	21.9167	0.882

0.05	0.05	AG1	2	21.9167	0.838
0.05	0.05	ptsI	2	22.1667	0.539
0.05	0.05	clcB	2	22.1667	0.327
0.05	0.05	ycaM	2	22.1667	0.326
0.05	0.05	yadI	2	22.1667	0.882
0.05	0.05	AG1	2	22.1667	0.84
0.05	0.05	ptsI	2	22.4167	0.535
0.05	0.05	clcB	2	22.4167	0.329
0.05	0.05	ycaM	2	22.4167	0.325
0.05	0.05	yadI	2	22.4167	0.884
0.05	0.05	AG1	2	22.4167	0.847
0.05	0.05	ptsI	2	22.6667	0.534
0.05	0.05	clcB	2	22.6667	0.327
0.05	0.05	ycaM	2	22.6667	0.33
0.05	0.05	yadI	2	22.6667	0.887
0.05	0.05	AG1	2	22.6667	0.85
0.05	0.05	ptsI	2	22.9167	0.543
0.05	0.05	clcB	2	22.9167	0.33
0.05	0.05	ycaM	2	22.9167	0.333
0.05	0.05	yadI	2	22.9167	0.885
0.05	0.05	AG1	2	22.9167	0.859
0.05	0.05	ptsI	2	23.1667	0.537
0.05	0.05	clcB	2	23.1667	0.329
0.05	0.05	ycaM	2	23.1667	0.332
0.05	0.05	yadI	2	23.1667	0.882
0.05	0.05	AG1	2	23.1667	0.861
0.05	0.05	ptsI	2	23.4167	0.537
0.05	0.05	clcB	2	23.4167	0.329
0.05	0.05	ycaM	2	23.4167	0.336
0.05	0.05	yadI	2	23.4167	0.891
0.05	0.05	AG1	2	23.4167	0.865
0.05	0.05	ptsI	2	23.6667	0.537
0.05	0.05	clcB	2	23.6667	0.33
0.05	0.05	ycaM	2	23.6667	0.34
0.05	0.05	yadI	2	23.6667	0.89
0.05	0.05	AG1	2	23.6667	0.87
0.05	0.05	ptsI	2	23.9167	0.539
0.05	0.05	clcB	2	23.9167	0.332
0.05	0.05	ycaM	2	23.9167	0.337
0.05	0.05	yadI	2	23.9167	0.897
0.05	0.05	AG1	2	23.9167	0.878
0.05	0.05	ptsI	2	24.1667	0.538
0.05	0.05	clcB	2	24.1667	0.333
0.05	0.05	ycaM	2	24.1667	0.337
0.05	0.05	yadI	2	24.1667	0.89
0.05	0.05	AG1	2	24.1667	0.882
0.05	0.05	ptsI	2	24.4167	0.54
0.05	0.05	clcB	2	24.4167	0.333
0.05	0.05	ycaM	2	24.4167	0.344
0.05	0.05	yadI	2	24.4167	0.89
0.05	0.05	AG1	2	24.4167	0.89
0.05	0.05	ptsI	2	24.6667	0.543
0.05	0.05	clcB	2	24.6667	0.332

0.05	0.05	ycaM	2	24.6667	0.343
0.05	0.05	yadI	2	24.6667	0.889
0.05	0.05	AG1	2	24.6667	0.898
0.05	0.05	ptsI	2	24.9167	0.541
0.05	0.05	clcB	2	24.9167	0.334
0.05	0.05	ycaM	2	24.9167	0.344
0.05	0.05	yadI	2	24.9167	0.89
0.05	0.05	AG1	2	24.9167	0.91
0.05	0.05	ptsI	2	25.1667	0.542
0.05	0.05	clcB	2	25.1667	0.335
0.05	0.05	ycaM	2	25.1667	0.347
0.05	0.05	yadI	2	25.1667	0.9
0.05	0.05	AG1	2	25.1667	0.913
0.05	0.05	ptsI	2	25.4167	0.546
0.05	0.05	clcB	2	25.4167	0.338
0.05	0.05	ycaM	2	25.4167	0.348
0.05	0.05	yadI	2	25.4167	0.896
0.05	0.05	AG1	2	25.4167	0.924
0.05	0.05	ptsI	2	25.6667	0.54
0.05	0.05	clcB	2	25.6667	0.335
0.05	0.05	ycaM	2	25.6667	0.348
0.05	0.05	yadI	2	25.6667	0.898
0.05	0.05	AG1	2	25.6667	0.93
0.05	0.05	ptsI	2	25.9167	0.542
0.05	0.05	clcB	2	25.9167	0.339
0.05	0.05	ycaM	2	25.9167	0.355
0.05	0.05	yadI	2	25.9167	0.905
0.05	0.05	AG1	2	25.9167	0.94
0.05	0.05	ptsI	2	26.1667	0.538
0.05	0.05	clcB	2	26.1667	0.335
0.05	0.05	ycaM	2	26.1667	0.356
0.05	0.05	yadI	2	26.1667	0.891
0.05	0.05	AG1	2	26.1667	0.954
0.05	0.05	ptsI	2	26.4167	0.54
0.05	0.05	clcB	2	26.4167	0.336
0.05	0.05	ycaM	2	26.4167	0.357
0.05	0.05	yadI	2	26.4167	0.9
0.05	0.05	AG1	2	26.4167	0.963
0.05	0.05	ptsI	3	0	0.155
0.05	0.05	clcB	3	0	0.164
0.05	0.05	ycaM	3	0	0.162
0.05	0.05	yadI	3	0	0.162
0.05	0.05	AG1	3	0	0.163
0.05	0.05	ptsI	3	0.25	0.152
0.05	0.05	clcB	3	0.25	0.161
0.05	0.05	ycaM	3	0.25	0.158
0.05	0.05	yadI	3	0.25	0.159
0.05	0.05	AG1	3	0.25	0.159
0.05	0.05	ptsI	3	0.5	0.151
0.05	0.05	clcB	3	0.5	0.16
0.05	0.05	ycaM	3	0.5	0.156
0.05	0.05	yadI	3	0.5	0.158
0.05	0.05	AG1	3	0.5	0.158

0.05	0.05	ptsI	3	0.75	0.152
0.05	0.05	clcB	3	0.75	0.16
0.05	0.05	ycaM	3	0.75	0.156
0.05	0.05	yadI	3	0.75	0.16
0.05	0.05	AG1	3	0.75	0.159
0.05	0.05	ptsI	3	1	0.152
0.05	0.05	clcB	3	1	0.16
0.05	0.05	ycaM	3	1	0.157
0.05	0.05	yadI	3	1	0.161
0.05	0.05	AG1	3	1	0.159
0.05	0.05	ptsI	3	1.25	0.153
0.05	0.05	clcB	3	1.25	0.161
0.05	0.05	ycaM	3	1.25	0.157
0.05	0.05	yadI	3	1.25	0.161
0.05	0.05	AG1	3	1.25	0.161
0.05	0.05	ptsI	3	1.5	0.166
0.05	0.05	clcB	3	1.5	0.16
0.05	0.05	ycaM	3	1.5	0.158
0.05	0.05	yadI	3	1.5	0.163
0.05	0.05	AG1	3	1.5	0.162
0.05	0.05	ptsI	3	1.75	0.155
0.05	0.05	clcB	3	1.75	0.161
0.05	0.05	ycaM	3	1.75	0.16
0.05	0.05	yadI	3	1.75	0.164
0.05	0.05	AG1	3	1.75	0.165
0.05	0.05	ptsI	3	2	0.158
0.05	0.05	clcB	3	2	0.161
0.05	0.05	ycaM	3	2	0.161
0.05	0.05	yadI	3	2	0.166
0.05	0.05	AG1	3	2	0.164
0.05	0.05	ptsI	3	2.25	0.159
0.05	0.05	clcB	3	2.25	0.162
0.05	0.05	ycaM	3	2.25	0.163
0.05	0.05	yadI	3	2.25	0.168
0.05	0.05	AG1	3	2.25	0.168
0.05	0.05	ptsI	3	2.5	0.16
0.05	0.05	clcB	3	2.5	0.164
0.05	0.05	ycaM	3	2.5	0.163
0.05	0.05	yadI	3	2.5	0.172
0.05	0.05	AG1	3	2.5	0.17
0.05	0.05	ptsI	3	2.75	0.164
0.05	0.05	clcB	3	2.75	0.167
0.05	0.05	ycaM	3	2.75	0.168
0.05	0.05	yadI	3	2.75	0.174
0.05	0.05	AG1	3	2.75	0.173
0.05	0.05	ptsI	3	3	0.165
0.05	0.05	clcB	3	3	0.167
0.05	0.05	ycaM	3	3	0.167
0.05	0.05	yadI	3	3	0.18
0.05	0.05	AG1	3	3	0.176
0.05	0.05	ptsI	3	3.25	0.167
0.05	0.05	clcB	3	3.25	0.169
0.05	0.05	ycaM	3	3.25	0.17

0.05	0.05	yadI	3	3.25	0.186
0.05	0.05	AG1	3	3.25	0.179
0.05	0.05	ptsI	3	3.5	0.17
0.05	0.05	clcB	3	3.5	0.172
0.05	0.05	ycaM	3	3.5	0.174
0.05	0.05	yadI	3	3.5	0.19
0.05	0.05	AG1	3	3.5	0.186
0.05	0.05	ptsI	3	3.75	0.174
0.05	0.05	clcB	3	3.75	0.174
0.05	0.05	ycaM	3	3.75	0.175
0.05	0.05	yadI	3	3.75	0.197
0.05	0.05	AG1	3	3.75	0.185
0.05	0.05	ptsI	3	4	0.176
0.05	0.05	clcB	3	4	0.177
0.05	0.05	ycaM	3	4	0.178
0.05	0.05	yadI	3	4	0.204
0.05	0.05	AG1	3	4	0.191
0.05	0.05	ptsI	3	4.41667	0.195
0.05	0.05	clcB	3	4.41667	0.202
0.05	0.05	ycaM	3	4.41667	0.206
0.05	0.05	yadI	3	4.41667	0.221
0.05	0.05	AG1	3	4.41667	0.197
0.05	0.05	ptsI	3	4.66667	0.186
0.05	0.05	clcB	3	4.66667	0.2
0.05	0.05	ycaM	3	4.66667	0.194
0.05	0.05	yadI	3	4.66667	0.218
0.05	0.05	AG1	3	4.66667	0.194
0.05	0.05	ptsI	3	4.91667	0.189
0.05	0.05	clcB	3	4.91667	0.198
0.05	0.05	ycaM	3	4.91667	0.202
0.05	0.05	yadI	3	4.91667	0.232
0.05	0.05	AG1	3	4.91667	0.194
0.05	0.05	ptsI	3	5.16667	0.192
0.05	0.05	clcB	3	5.16667	0.203
0.05	0.05	ycaM	3	5.16667	0.207
0.05	0.05	yadI	3	5.16667	0.244
0.05	0.05	AG1	3	5.16667	0.2
0.05	0.05	ptsI	3	5.41667	0.197
0.05	0.05	clcB	3	5.41667	0.212
0.05	0.05	ycaM	3	5.41667	0.215
0.05	0.05	yadI	3	5.41667	0.26
0.05	0.05	AG1	3	5.41667	0.206
0.05	0.05	ptsI	3	5.66667	0.201
0.05	0.05	clcB	3	5.66667	0.219
0.05	0.05	ycaM	3	5.66667	0.221
0.05	0.05	yadI	3	5.66667	0.277
0.05	0.05	AG1	3	5.66667	0.214
0.05	0.05	ptsI	3	5.91667	0.206
0.05	0.05	clcB	3	5.91667	0.227
0.05	0.05	ycaM	3	5.91667	0.23
0.05	0.05	yadI	3	5.91667	0.297
0.05	0.05	AG1	3	5.91667	0.229
0.05	0.05	ptsI	3	6.16667	0.212

0.05	0.05	clcB	3	6.16667	0.237
0.05	0.05	ycaM	3	6.16667	0.238
0.05	0.05	yadI	3	6.16667	0.316
0.05	0.05	AG1	3	6.16667	0.24
0.05	0.05	ptsI	3	6.41667	0.218
0.05	0.05	clcB	3	6.41667	0.245
0.05	0.05	ycaM	3	6.41667	0.245
0.05	0.05	yadI	3	6.41667	0.326
0.05	0.05	AG1	3	6.41667	0.251
0.05	0.05	ptsI	3	6.66667	0.226
0.05	0.05	clcB	3	6.66667	0.256
0.05	0.05	ycaM	3	6.66667	0.255
0.05	0.05	yadI	3	6.66667	0.336
0.05	0.05	AG1	3	6.66667	0.266
0.05	0.05	ptsI	3	6.91667	0.234
0.05	0.05	clcB	3	6.91667	0.266
0.05	0.05	ycaM	3	6.91667	0.262
0.05	0.05	yadI	3	6.91667	0.348
0.05	0.05	AG1	3	6.91667	0.281
0.05	0.05	ptsI	3	7.16667	0.242
0.05	0.05	clcB	3	7.16667	0.276
0.05	0.05	ycaM	3	7.16667	0.264
0.05	0.05	yadI	3	7.16667	0.36
0.05	0.05	AG1	3	7.16667	0.29
0.05	0.05	ptsI	3	7.41667	0.254
0.05	0.05	clcB	3	7.41667	0.283
0.05	0.05	ycaM	3	7.41667	0.266
0.05	0.05	yadI	3	7.41667	0.374
0.05	0.05	AG1	3	7.41667	0.298
0.05	0.05	ptsI	3	7.66667	0.264
0.05	0.05	clcB	3	7.66667	0.289
0.05	0.05	ycaM	3	7.66667	0.27
0.05	0.05	yadI	3	7.66667	0.389
0.05	0.05	AG1	3	7.66667	0.306
0.05	0.05	ptsI	3	7.91667	0.272
0.05	0.05	clcB	3	7.91667	0.293
0.05	0.05	ycaM	3	7.91667	0.271
0.05	0.05	yadI	3	7.91667	0.398
0.05	0.05	AG1	3	7.91667	0.316
0.05	0.05	ptsI	3	8.16667	0.278
0.05	0.05	clcB	3	8.16667	0.299
0.05	0.05	ycaM	3	8.16667	0.272
0.05	0.05	yadI	3	8.16667	0.41
0.05	0.05	AG1	3	8.16667	0.325
0.05	0.05	ptsI	3	8.41667	0.287
0.05	0.05	clcB	3	8.41667	0.306
0.05	0.05	ycaM	3	8.41667	0.275
0.05	0.05	yadI	3	8.41667	0.418
0.05	0.05	AG1	3	8.41667	0.334
0.05	0.05	ptsI	3	9.05	0.279
0.05	0.05	clcB	3	9.05	0.27
0.05	0.05	ycaM	3	9.05	0.252
0.05	0.05	yadI	3	9.05	0.453

0.05	0.05	AG1	3	9.05	0.363
0.05	0.05	ptsI	3	9.3	0.279
0.05	0.05	clcB	3	9.3	0.269
0.05	0.05	ycaM	3	9.3	0.251
0.05	0.05	yadI	3	9.3	0.463
0.05	0.05	AG1	3	9.3	0.368
0.05	0.05	ptsI	3	9.55	0.286
0.05	0.05	clcB	3	9.55	0.278
0.05	0.05	ycaM	3	9.55	0.255
0.05	0.05	yadI	3	9.55	0.476
0.05	0.05	AG1	3	9.55	0.377
0.05	0.05	ptsI	3	9.8	0.293
0.05	0.05	clcB	3	9.8	0.282
0.05	0.05	ycaM	3	9.8	0.256
0.05	0.05	yadI	3	9.8	0.492
0.05	0.05	AG1	3	9.8	0.387
0.05	0.05	ptsI	3	10.05	0.302
0.05	0.05	clcB	3	10.05	0.288
0.05	0.05	ycaM	3	10.05	0.262
0.05	0.05	yadI	3	10.05	0.511
0.05	0.05	AG1	3	10.05	0.4
0.05	0.05	ptsI	3	10.3	0.31
0.05	0.05	clcB	3	10.3	0.295
0.05	0.05	ycaM	3	10.3	0.263
0.05	0.05	yadI	3	10.3	0.524
0.05	0.05	AG1	3	10.3	0.411
0.05	0.05	ptsI	3	10.55	0.319
0.05	0.05	clcB	3	10.55	0.296
0.05	0.05	ycaM	3	10.55	0.263
0.05	0.05	yadI	3	10.55	0.536
0.05	0.05	AG1	3	10.55	0.424
0.05	0.05	ptsI	3	10.8	0.327
0.05	0.05	clcB	3	10.8	0.297
0.05	0.05	ycaM	3	10.8	0.265
0.05	0.05	yadI	3	10.8	0.548
0.05	0.05	AG1	3	10.8	0.431
0.05	0.05	ptsI	3	11.05	0.338
0.05	0.05	clcB	3	11.05	0.308
0.05	0.05	ycaM	3	11.05	0.267
0.05	0.05	yadI	3	11.05	0.562
0.05	0.05	AG1	3	11.05	0.445
0.05	0.05	ptsI	3	11.3	0.345
0.05	0.05	clcB	3	11.3	0.3
0.05	0.05	ycaM	3	11.3	0.264
0.05	0.05	yadI	3	11.3	0.571
0.05	0.05	AG1	3	11.3	0.451
0.05	0.05	ptsI	3	11.55	0.356
0.05	0.05	clcB	3	11.55	0.301
0.05	0.05	ycaM	3	11.55	0.265
0.05	0.05	yadI	3	11.55	0.582
0.05	0.05	AG1	3	11.55	0.463
0.05	0.05	ptsI	3	11.8	0.366
0.05	0.05	clcB	3	11.8	0.311

0.05	0.05	ycaM	3	11.8	0.269
0.05	0.05	yadI	3	11.8	0.599
0.05	0.05	AG1	3	11.8	0.479
0.05	0.05	ptsI	3	12.05	0.371
0.05	0.05	clcB	3	12.05	0.299
0.05	0.05	ycaM	3	12.05	0.264
0.05	0.05	yadI	3	12.05	0.605
0.05	0.05	AG1	3	12.05	0.487
0.05	0.05	ptsI	3	12.3	0.386
0.05	0.05	clcB	3	12.3	0.31
0.05	0.05	ycaM	3	12.3	0.268
0.05	0.05	yadI	3	12.3	0.621
0.05	0.05	AG1	3	12.3	0.5
0.05	0.05	ptsI	3	12.55	0.396
0.05	0.05	clcB	3	12.55	0.304
0.05	0.05	ycaM	3	12.55	0.269
0.05	0.05	yadI	3	12.55	0.638
0.05	0.05	AG1	3	12.55	0.519
0.05	0.05	ptsI	3	12.8	0.408
0.05	0.05	clcB	3	12.8	0.298
0.05	0.05	ycaM	3	12.8	0.268
0.05	0.05	yadI	3	12.8	0.655
0.05	0.05	AG1	3	12.8	0.539
0.05	0.05	ptsI	3	13.05	0.41
0.05	0.05	clcB	3	13.05	0.296
0.05	0.05	ycaM	3	13.05	0.264
0.05	0.05	yadI	3	13.05	0.654
0.05	0.05	AG1	3	13.05	0.546
0.05	0.05	ptsI	3	13.3	0.417
0.05	0.05	clcB	3	13.3	0.305
0.05	0.05	ycaM	3	13.3	0.267
0.05	0.05	yadI	3	13.3	0.671
0.05	0.05	AG1	3	13.3	0.562
0.05	0.05	ptsI	3	13.55	0.43
0.05	0.05	clcB	3	13.55	0.301
0.05	0.05	ycaM	3	13.55	0.266
0.05	0.05	yadI	3	13.55	0.682
0.05	0.05	AG1	3	13.55	0.574
0.05	0.05	ptsI	3	13.8	0.438
0.05	0.05	clcB	3	13.8	0.296
0.05	0.05	ycaM	3	13.8	0.266
0.05	0.05	yadI	3	13.8	0.695
0.05	0.05	AG1	3	13.8	0.592
0.05	0.05	ptsI	3	14.05	0.446
0.05	0.05	clcB	3	14.05	0.304
0.05	0.05	ycaM	3	14.05	0.267
0.05	0.05	yadI	3	14.05	0.695
0.05	0.05	AG1	3	14.05	0.598
0.05	0.05	ptsI	3	14.3	0.45
0.05	0.05	clcB	3	14.3	0.303
0.05	0.05	ycaM	3	14.3	0.266
0.05	0.05	yadI	3	14.3	0.704
0.05	0.05	AG1	3	14.3	0.607

0.05	0.05	ptsI	3	14.55	0.459
0.05	0.05	clcB	3	14.55	0.296
0.05	0.05	ycaM	3	14.55	0.261
0.05	0.05	yadI	3	14.55	0.711
0.05	0.05	AG1	3	14.55	0.618
0.05	0.05	ptsI	3	14.8	0.471
0.05	0.05	clcB	3	14.8	0.296
0.05	0.05	ycaM	3	14.8	0.264
0.05	0.05	yadI	3	14.8	0.733
0.05	0.05	AG1	3	14.8	0.632
0.05	0.05	ptsI	3	15.05	0.486
0.05	0.05	clcB	3	15.05	0.297
0.05	0.05	ycaM	3	15.05	0.265
0.05	0.05	yadI	3	15.05	0.752
0.05	0.05	AG1	3	15.05	0.65
0.05	0.05	ptsI	3	15.3	0.486
0.05	0.05	clcB	3	15.3	0.297
0.05	0.05	ycaM	3	15.3	0.261
0.05	0.05	yadI	3	15.3	0.745
0.05	0.05	AG1	3	15.3	0.653
0.05	0.05	ptsI	3	15.55	0.49
0.05	0.05	clcB	3	15.55	0.294
0.05	0.05	ycaM	3	15.55	0.261
0.05	0.05	yadI	3	15.55	0.757
0.05	0.05	AG1	3	15.55	0.665
0.05	0.05	ptsI	3	15.8	0.499
0.05	0.05	clcB	3	15.8	0.295
0.05	0.05	ycaM	3	15.8	0.261
0.05	0.05	yadI	3	15.8	0.761
0.05	0.05	AG1	3	15.8	0.685
0.05	0.05	ptsI	3	16.05	0.501
0.05	0.05	clcB	3	16.05	0.292
0.05	0.05	ycaM	3	16.05	0.258
0.05	0.05	yadI	3	16.05	0.764
0.05	0.05	AG1	3	16.05	0.696
0.05	0.05	ptsI	3	16.3	0.51
0.05	0.05	clcB	3	16.3	0.295
0.05	0.05	ycaM	3	16.3	0.258
0.05	0.05	yadI	3	16.3	0.758
0.05	0.05	AG1	3	16.3	0.725
0.05	0.05	ptsI	3	16.55	0.517
0.05	0.05	clcB	3	16.55	0.296
0.05	0.05	ycaM	3	16.55	0.259
0.05	0.05	yadI	3	16.55	0.783
0.05	0.05	AG1	3	16.55	0.718
0.05	0.05	ptsI	3	16.8	0.526
0.05	0.05	clcB	3	16.8	0.294
0.05	0.05	ycaM	3	16.8	0.257
0.05	0.05	yadI	3	16.8	0.789
0.05	0.05	AG1	3	16.8	0.749
0.05	0.05	ptsI	3	17.05	0.531
0.05	0.05	clcB	3	17.05	0.294
0.05	0.05	ycaM	3	17.05	0.257

0.05	0.05	yadI	3	17.05	0.789
0.05	0.05	AG1	3	17.05	0.752
0.05	0.05	ptsI	3	17.3	0.53
0.05	0.05	clcB	3	17.3	0.293
0.05	0.05	ycaM	3	17.3	0.258
0.05	0.05	yadI	3	17.3	0.795
0.05	0.05	AG1	3	17.3	0.748
0.05	0.05	ptsI	3	17.55	0.538
0.05	0.05	clcB	3	17.55	0.296
0.05	0.05	ycaM	3	17.55	0.259
0.05	0.05	yadI	3	17.55	0.796
0.05	0.05	AG1	3	17.55	0.756
0.05	0.05	ptsI	3	17.8	0.545
0.05	0.05	clcB	3	17.8	0.293
0.05	0.05	ycaM	3	17.8	0.259
0.05	0.05	yadI	3	17.8	0.806
0.05	0.05	AG1	3	17.8	0.771
0.05	0.05	ptsI	3	18.05	0.545
0.05	0.05	clcB	3	18.05	0.295
0.05	0.05	ycaM	3	18.05	0.259
0.05	0.05	yadI	3	18.05	0.803
0.05	0.05	AG1	3	18.05	0.778
0.05	0.05	ptsI	3	18.3	0.545
0.05	0.05	clcB	3	18.3	0.294
0.05	0.05	ycaM	3	18.3	0.259
0.05	0.05	yadI	3	18.3	0.813
0.05	0.05	AG1	3	18.3	0.777
0.05	0.05	ptsI	3	18.55	0.544
0.05	0.05	clcB	3	18.55	0.296
0.05	0.05	ycaM	3	18.55	0.26
0.05	0.05	yadI	3	18.55	0.818
0.05	0.05	AG1	3	18.55	0.757
0.05	0.05	ptsI	3	18.8	0.546
0.05	0.05	clcB	3	18.8	0.296
0.05	0.05	ycaM	3	18.8	0.264
0.05	0.05	yadI	3	18.8	0.817
0.05	0.05	AG1	3	18.8	0.747
0.05	0.05	ptsI	3	19.05	0.549
0.05	0.05	clcB	3	19.05	0.298
0.05	0.05	ycaM	3	19.05	0.263
0.05	0.05	yadI	3	19.05	0.829
0.05	0.05	AG1	3	19.05	0.759
0.05	0.05	ptsI	3	19.3	0.552
0.05	0.05	clcB	3	19.3	0.297
0.05	0.05	ycaM	3	19.3	0.266
0.05	0.05	yadI	3	19.3	0.832
0.05	0.05	AG1	3	19.3	0.774
0.05	0.05	ptsI	3	19.55	0.56
0.05	0.05	clcB	3	19.55	0.298
0.05	0.05	ycaM	3	19.55	0.264
0.05	0.05	yadI	3	19.55	0.828
0.05	0.05	AG1	3	19.55	0.772
0.05	0.05	ptsI	3	19.8	0.55

0.05	0.05	clcB	3	19.8	0.296
0.05	0.05	ycaM	3	19.8	0.266
0.05	0.05	yadI	3	19.8	0.824
0.05	0.05	AG1	3	19.8	0.769
0.05	0.05	ptsI	3	20.05	0.554
0.05	0.05	clcB	3	20.05	0.296
0.05	0.05	ycaM	3	20.05	0.267
0.05	0.05	yadI	3	20.05	0.833
0.05	0.05	AG1	3	20.05	0.757
0.05	0.05	ptsI	3	20.3	0.556
0.05	0.05	clcB	3	20.3	0.295
0.05	0.05	ycaM	3	20.3	0.269
0.05	0.05	yadI	3	20.3	0.832
0.05	0.05	AG1	3	20.3	0.758
0.05	0.05	ptsI	3	20.55	0.553
0.05	0.05	clcB	3	20.55	0.296
0.05	0.05	ycaM	3	20.55	0.271
0.05	0.05	yadI	3	20.55	0.831
0.05	0.05	AG1	3	20.55	0.767
0.05	0.05	ptsI	3	20.8	0.557
0.05	0.05	clcB	3	20.8	0.296
0.05	0.05	ycaM	3	20.8	0.272
0.05	0.05	yadI	3	20.8	0.839
0.05	0.05	AG1	3	20.8	0.781
0.05	0.05	ptsI	3	21.05	0.554
0.05	0.05	clcB	3	21.05	0.296
0.05	0.05	ycaM	3	21.05	0.275
0.05	0.05	yadI	3	21.05	0.837
0.05	0.05	AG1	3	21.05	0.782
0.05	0.05	ptsI	3	21.3	0.558
0.05	0.05	clcB	3	21.3	0.296
0.05	0.05	ycaM	3	21.3	0.277
0.05	0.05	yadI	3	21.3	0.84
0.05	0.05	AG1	3	21.3	0.793
0.05	0.05	ptsI	3	21.55	0.557
0.05	0.05	clcB	3	21.55	0.297
0.05	0.05	ycaM	3	21.55	0.278
0.05	0.05	yadI	3	21.55	0.842
0.05	0.05	AG1	3	21.55	0.802
0.05	0.05	ptsI	3	21.8	0.559
0.05	0.05	clcB	3	21.8	0.299
0.05	0.05	ycaM	3	21.8	0.279
0.05	0.05	yadI	3	21.8	0.844
0.05	0.05	AG1	3	21.8	0.809
0.05	0.05	ptsI	3	22.05	0.557
0.05	0.05	clcB	3	22.05	0.297
0.05	0.05	ycaM	3	22.05	0.28
0.05	0.05	yadI	3	22.05	0.846
0.05	0.05	AG1	3	22.05	0.811
0.05	0.05	ptsI	3	22.3	0.557
0.05	0.05	clcB	3	22.3	0.299
0.05	0.05	ycaM	3	22.3	0.285
0.05	0.05	yadI	3	22.3	0.843

0.05	0.05	AG1	3	22.3	0.822
0.05	0.05	ptsI	3	22.55	0.559
0.05	0.05	clcB	3	22.55	0.301
0.05	0.05	ycaM	3	22.55	0.288
0.05	0.05	yadI	3	22.55	0.851
0.05	0.05	AG1	3	22.55	0.828
0.05	0.05	ptsI	3	22.8	0.558
0.05	0.05	clcB	3	22.8	0.3
0.05	0.05	ycaM	3	22.8	0.29
0.05	0.05	yadI	3	22.8	0.848
0.05	0.05	AG1	3	22.8	0.836
0.05	0.05	ptsI	3	23.05	0.558
0.05	0.05	clcB	3	23.05	0.301
0.05	0.05	ycaM	3	23.05	0.292
0.05	0.05	yadI	3	23.05	0.855
0.05	0.05	AG1	3	23.05	0.841
0.05	0.05	ptsI	3	23.3	0.562
0.05	0.05	clcB	3	23.3	0.302
0.05	0.05	ycaM	3	23.3	0.294
0.05	0.05	yadI	3	23.3	0.854
0.05	0.05	AG1	3	23.3	0.848
0.05	0.05	ptsI	3	23.55	0.567
0.05	0.05	clcB	3	23.55	0.306
0.05	0.05	ycaM	3	23.55	0.304
0.05	0.05	yadI	3	23.55	0.862
0.05	0.05	AG1	3	23.55	0.865
0.05	0.05	ptsI	3	23.8	0.565
0.05	0.05	clcB	3	23.8	0.31
0.05	0.05	ycaM	3	23.8	0.306
0.05	0.05	yadI	3	23.8	0.86
0.05	0.05	AG1	3	23.8	0.866
0.05	0.05	ptsI	3	24.05	0.569
0.05	0.05	clcB	3	24.05	0.308
0.05	0.05	ycaM	3	24.05	0.306
0.05	0.05	yadI	3	24.05	0.861
0.05	0.05	AG1	3	24.05	0.87
0.05	0.05	ptsI	3	24.3	0.562
0.05	0.05	clcB	3	24.3	0.305
0.05	0.05	ycaM	3	24.3	0.305
0.05	0.05	yadI	3	24.3	0.851
0.05	0.05	AG1	3	24.3	0.881
0.05	0.05	ptsI	4	0	0.207
0.05	0.05	clcB	4	0	0.198
0.05	0.05	ycaM	4	0	0.194
0.05	0.05	yadI	4	0	0.254
0.05	0.05	AG1	4	0	0.196
0.05	0.05	ptsI	4	0.25	0.204
0.05	0.05	clcB	4	0.25	0.196
0.05	0.05	ycaM	4	0.25	0.19
0.05	0.05	yadI	4	0.25	0.257
0.05	0.05	AG1	4	0.25	0.194
0.05	0.05	ptsI	4	0.5	0.204
0.05	0.05	clcB	4	0.5	0.197

0.05	0.05	ycaM	4	0.5	0.19
0.05	0.05	yadI	4	0.5	0.255
0.05	0.05	AG1	4	0.5	0.195
0.05	0.05	ptsI	4	0.75	0.202
0.05	0.05	clcB	4	0.75	0.199
0.05	0.05	ycaM	4	0.75	0.193
0.05	0.05	yadI	4	0.75	0.259
0.05	0.05	AG1	4	0.75	0.197
0.05	0.05	ptsI	4	1	0.205
0.05	0.05	clcB	4	1	0.202
0.05	0.05	ycaM	4	1	0.194
0.05	0.05	yadI	4	1	0.262
0.05	0.05	AG1	4	1	0.2
0.05	0.05	ptsI	4	1.25	0.209
0.05	0.05	clcB	4	1.25	0.206
0.05	0.05	ycaM	4	1.25	0.201
0.05	0.05	yadI	4	1.25	0.265
0.05	0.05	AG1	4	1.25	0.205
0.05	0.05	ptsI	4	1.5	0.213
0.05	0.05	clcB	4	1.5	0.212
0.05	0.05	ycaM	4	1.5	0.204
0.05	0.05	yadI	4	1.5	0.272
0.05	0.05	AG1	4	1.5	0.213
0.05	0.05	ptsI	4	1.75	0.221
0.05	0.05	clcB	4	1.75	0.218
0.05	0.05	ycaM	4	1.75	0.211
0.05	0.05	yadI	4	1.75	0.277
0.05	0.05	AG1	4	1.75	0.218
0.05	0.05	ptsI	4	2	0.226
0.05	0.05	clcB	4	2	0.226
0.05	0.05	ycaM	4	2	0.219
0.05	0.05	yadI	4	2	0.287
0.05	0.05	AG1	4	2	0.226
0.05	0.05	ptsI	4	2.25	0.236
0.05	0.05	clcB	4	2.25	0.234
0.05	0.05	ycaM	4	2.25	0.228
0.05	0.05	yadI	4	2.25	0.298
0.05	0.05	AG1	4	2.25	0.235
0.05	0.05	ptsI	4	2.5	0.243
0.05	0.05	clcB	4	2.5	0.241
0.05	0.05	ycaM	4	2.5	0.236
0.05	0.05	yadI	4	2.5	0.311
0.05	0.05	AG1	4	2.5	0.244
0.05	0.05	ptsI	4	2.75	0.249
0.05	0.05	clcB	4	2.75	0.247
0.05	0.05	ycaM	4	2.75	0.245
0.05	0.05	yadI	4	2.75	0.33
0.05	0.05	AG1	4	2.75	0.252
0.05	0.05	ptsI	4	3	0.26
0.05	0.05	clcB	4	3	0.259
0.05	0.05	ycaM	4	3	0.254
0.05	0.05	yadI	4	3	0.324
0.05	0.05	AG1	4	3	0.261

0.05	0.05	ptsI	4	3.25	0.265
0.05	0.05	clcB	4	3.25	0.27
0.05	0.05	ycaM	4	3.25	0.263
0.05	0.05	yadI	4	3.25	0.334
0.05	0.05	AG1	4	3.25	0.272
0.05	0.05	ptsI	4	3.5	0.277
0.05	0.05	clcB	4	3.5	0.278
0.05	0.05	ycaM	4	3.5	0.276
0.05	0.05	yadI	4	3.5	0.347
0.05	0.05	AG1	4	3.5	0.281
0.05	0.05	ptsI	4	3.75	0.285
0.05	0.05	clcB	4	3.75	0.288
0.05	0.05	ycaM	4	3.75	0.283
0.05	0.05	yadI	4	3.75	0.372
0.05	0.05	AG1	4	3.75	0.29
0.05	0.05	ptsI	4	4	0.296
0.05	0.05	clcB	4	4	0.301
0.05	0.05	ycaM	4	4	0.295
0.05	0.05	yadI	4	4	0.37
0.05	0.05	AG1	4	4	0.301
0.05	0.05	ptsI	4	4.35	0.307
0.05	0.05	clcB	4	4.35	0.312
0.05	0.05	ycaM	4	4.35	0.39
0.05	0.05	yadI	4	4.35	0.338
0.05	0.05	AG1	4	4.35	0.329
0.05	0.05	ptsI	4	4.6	0.3
0.05	0.05	clcB	4	4.6	0.302
0.05	0.05	ycaM	4	4.6	0.344
0.05	0.05	yadI	4	4.6	0.342
0.05	0.05	AG1	4	4.6	0.342
0.05	0.05	ptsI	4	4.85	0.31
0.05	0.05	clcB	4	4.85	0.307
0.05	0.05	ycaM	4	4.85	0.344
0.05	0.05	yadI	4	4.85	0.355
0.05	0.05	AG1	4	4.85	0.356
0.05	0.05	ptsI	4	5.1	0.321
0.05	0.05	clcB	4	5.1	0.309
0.05	0.05	ycaM	4	5.1	0.345
0.05	0.05	yadI	4	5.1	0.366
0.05	0.05	AG1	4	5.1	0.369
0.05	0.05	ptsI	4	5.35	0.333
0.05	0.05	clcB	4	5.35	0.312
0.05	0.05	ycaM	4	5.35	0.347
0.05	0.05	yadI	4	5.35	0.378
0.05	0.05	AG1	4	5.35	0.385
0.05	0.05	ptsI	4	5.6	0.347
0.05	0.05	clcB	4	5.6	0.311
0.05	0.05	ycaM	4	5.6	0.346
0.05	0.05	yadI	4	5.6	0.396
0.05	0.05	AG1	4	5.6	0.394
0.05	0.05	ptsI	4	5.85	0.36
0.05	0.05	clcB	4	5.85	0.315
0.05	0.05	ycaM	4	5.85	0.426

0.05	0.05	yadI	4	5.85	0.406
0.05	0.05	AG1	4	5.85	0.405
0.05	0.05	ptsI	4	6.1	0.375
0.05	0.05	clcB	4	6.1	0.322
0.05	0.05	ycaM	4	6.1	0.362
0.05	0.05	yadI	4	6.1	0.416
0.05	0.05	AG1	4	6.1	0.415
0.05	0.05	ptsI	4	6.35	0.384
0.05	0.05	clcB	4	6.35	0.325
0.05	0.05	ycaM	4	6.35	0.365
0.05	0.05	yadI	4	6.35	0.428
0.05	0.05	AG1	4	6.35	0.425
0.05	0.05	ptsI	4	6.6	0.398
0.05	0.05	clcB	4	6.6	0.326
0.05	0.05	ycaM	4	6.6	0.373
0.05	0.05	yadI	4	6.6	0.428
0.05	0.05	AG1	4	6.6	0.436
0.05	0.05	ptsI	4	6.85	0.405
0.05	0.05	clcB	4	6.85	0.329
0.05	0.05	ycaM	4	6.85	0.453
0.05	0.05	yadI	4	6.85	0.432
0.05	0.05	AG1	4	6.85	0.447
0.05	0.05	ptsI	4	7.1	0.413
0.05	0.05	clcB	4	7.1	0.331
0.05	0.05	ycaM	4	7.1	0.447
0.05	0.05	yadI	4	7.1	0.438
0.05	0.05	AG1	4	7.1	0.461
0.05	0.05	ptsI	4	7.35	0.42
0.05	0.05	clcB	4	7.35	0.332
0.05	0.05	ycaM	4	7.35	0.508
0.05	0.05	yadI	4	7.35	0.445
0.05	0.05	AG1	4	7.35	0.472
0.05	0.05	ptsI	4	7.6	0.426
0.05	0.05	clcB	4	7.6	0.335
0.05	0.05	ycaM	4	7.6	0.521
0.05	0.05	yadI	4	7.6	0.451
0.05	0.05	AG1	4	7.6	0.478
0.05	0.05	ptsI	4	7.85	0.435
0.05	0.05	clcB	4	7.85	0.336
0.05	0.05	ycaM	4	7.85	0.363
0.05	0.05	yadI	4	7.85	0.459
0.05	0.05	AG1	4	7.85	0.487
0.05	0.05	ptsI	4	8.1	0.443
0.05	0.05	clcB	4	8.1	0.337
0.05	0.05	ycaM	4	8.1	0.366
0.05	0.05	yadI	4	8.1	0.46
0.05	0.05	AG1	4	8.1	0.497
0.05	0.05	ptsI	4	8.35	0.452
0.05	0.05	clcB	4	8.35	0.342
0.05	0.05	ycaM	4	8.35	0.371
0.05	0.05	yadI	4	8.35	0.467
0.05	0.05	AG1	4	8.35	0.507
0.05	0.05	ptsI	4	8.83333	0.438

0.05	0.05	clcB	4	8.83333	0.306
0.05	0.05	ycaM	4	8.83333	0.284
0.05	0.05	yadI	4	8.83333	0.521
0.05	0.05	AG1	4	8.83333	0.536
0.05	0.05	ptsI	4	9.08333	0.437
0.05	0.05	clcB	4	9.08333	0.299
0.05	0.05	ycaM	4	9.08333	0.279
0.05	0.05	yadI	4	9.08333	0.522
0.05	0.05	AG1	4	9.08333	0.541
0.05	0.05	ptsI	4	9.33333	0.445
0.05	0.05	clcB	4	9.33333	0.297
0.05	0.05	ycaM	4	9.33333	0.276
0.05	0.05	yadI	4	9.33333	0.529
0.05	0.05	AG1	4	9.33333	0.554
0.05	0.05	ptsI	4	9.58333	0.457
0.05	0.05	clcB	4	9.58333	0.303
0.05	0.05	ycaM	4	9.58333	0.279
0.05	0.05	yadI	4	9.58333	0.534
0.05	0.05	AG1	4	9.58333	0.565
0.05	0.05	ptsI	4	9.83333	0.471
0.05	0.05	clcB	4	9.83333	0.3
0.05	0.05	ycaM	4	9.83333	0.279
0.05	0.05	yadI	4	9.83333	0.536
0.05	0.05	AG1	4	9.83333	0.577
0.05	0.05	ptsI	4	10.0833	0.478
0.05	0.05	clcB	4	10.0833	0.301
0.05	0.05	ycaM	4	10.0833	0.279
0.05	0.05	yadI	4	10.0833	0.541
0.05	0.05	AG1	4	10.0833	0.585
0.05	0.05	ptsI	4	10.3333	0.488
0.05	0.05	clcB	4	10.3333	0.3
0.05	0.05	ycaM	4	10.3333	0.278
0.05	0.05	yadI	4	10.3333	0.548
0.05	0.05	AG1	4	10.3333	0.596
0.05	0.05	ptsI	4	10.5833	0.494
0.05	0.05	clcB	4	10.5833	0.3
0.05	0.05	ycaM	4	10.5833	0.281
0.05	0.05	yadI	4	10.5833	0.55
0.05	0.05	AG1	4	10.5833	0.604
0.05	0.05	ptsI	4	10.8333	0.503
0.05	0.05	clcB	4	10.8333	0.3
0.05	0.05	ycaM	4	10.8333	0.283
0.05	0.05	yadI	4	10.8333	0.561
0.05	0.05	AG1	4	10.8333	0.613
0.05	0.05	ptsI	4	11.0833	0.51
0.05	0.05	clcB	4	11.0833	0.301
0.05	0.05	ycaM	4	11.0833	0.275
0.05	0.05	yadI	4	11.0833	0.561
0.05	0.05	AG1	4	11.0833	0.62
0.05	0.05	ptsI	4	11.3333	0.52
0.05	0.05	clcB	4	11.3333	0.302
0.05	0.05	ycaM	4	11.3333	0.283
0.05	0.05	yadI	4	11.3333	0.559

0.05	0.05	AG1	4	11.3333	0.627
0.05	0.05	ptsI	4	11.5833	0.529
0.05	0.05	clcB	4	11.5833	0.304
0.05	0.05	ycaM	4	11.5833	0.28
0.05	0.05	yadI	4	11.5833	0.565
0.05	0.05	AG1	4	11.5833	0.632
0.05	0.05	ptsI	4	11.8333	0.534
0.05	0.05	clcB	4	11.8333	0.31
0.05	0.05	ycaM	4	11.8333	0.279
0.05	0.05	yadI	4	11.8333	0.567
0.05	0.05	AG1	4	11.8333	0.639
0.05	0.05	ptsI	4	12.0833	0.545
0.05	0.05	clcB	4	12.0833	0.31
0.05	0.05	ycaM	4	12.0833	0.278
0.05	0.05	yadI	4	12.0833	0.566
0.05	0.05	AG1	4	12.0833	0.642
0.05	0.05	ptsI	4	12.3333	0.553
0.05	0.05	clcB	4	12.3333	0.307
0.05	0.05	ycaM	4	12.3333	0.279
0.05	0.05	yadI	4	12.3333	0.572
0.05	0.05	AG1	4	12.3333	0.647
0.05	0.05	ptsI	4	12.5833	0.56
0.05	0.05	clcB	4	12.5833	0.315
0.05	0.05	ycaM	4	12.5833	0.285
0.05	0.05	yadI	4	12.5833	0.572
0.05	0.05	AG1	4	12.5833	0.655
0.05	0.05	ptsI	4	12.8333	0.566
0.05	0.05	clcB	4	12.8333	0.304
0.05	0.05	ycaM	4	12.8333	0.284
0.05	0.05	yadI	4	12.8333	0.574
0.05	0.05	AG1	4	12.8333	0.66
0.05	0.05	ptsI	4	13.0833	0.571
0.05	0.05	clcB	4	13.0833	0.31
0.05	0.05	ycaM	4	13.0833	0.283
0.05	0.05	yadI	4	13.0833	0.571
0.05	0.05	AG1	4	13.0833	0.664
0.05	0.05	ptsI	4	13.3333	0.579
0.05	0.05	clcB	4	13.3333	0.315
0.05	0.05	ycaM	4	13.3333	0.286
0.05	0.05	yadI	4	13.3333	0.572
0.05	0.05	AG1	4	13.3333	0.674
0.05	0.05	ptsI	4	13.5833	0.586
0.05	0.05	clcB	4	13.5833	0.31
0.05	0.05	ycaM	4	13.5833	0.284
0.05	0.05	yadI	4	13.5833	0.573
0.05	0.05	AG1	4	13.5833	0.676
0.05	0.05	ptsI	4	13.8333	0.592
0.05	0.05	clcB	4	13.8333	0.327
0.05	0.05	ycaM	4	13.8333	0.285
0.05	0.05	yadI	4	13.8333	0.571
0.05	0.05	AG1	4	13.8333	0.681
0.05	0.05	ptsI	4	14.0833	0.599
0.05	0.05	clcB	4	14.0833	0.317

0.05	0.05	ycaM	4	14.0833	0.286
0.05	0.05	yadI	4	14.0833	0.577
0.05	0.05	AG1	4	14.0833	0.684
0.05	0.05	ptsI	4	14.3333	0.604
0.05	0.05	clcB	4	14.3333	0.315
0.05	0.05	ycaM	4	14.3333	0.288
0.05	0.05	yadI	4	14.3333	0.573
0.05	0.05	AG1	4	14.3333	0.692
0.05	0.05	ptsI	4	14.5833	0.61
0.05	0.05	clcB	4	14.5833	0.315
0.05	0.05	ycaM	4	14.5833	0.288
0.05	0.05	yadI	4	14.5833	0.579
0.05	0.05	AG1	4	14.5833	0.693
0.05	0.05	ptsI	4	14.8333	0.618
0.05	0.05	clcB	4	14.8333	0.313
0.05	0.05	ycaM	4	14.8333	0.286
0.05	0.05	yadI	4	14.8333	0.578
0.05	0.05	AG1	4	14.8333	0.699
0.05	0.05	ptsI	4	15.0833	0.619
0.05	0.05	clcB	4	15.0833	0.315
0.05	0.05	ycaM	4	15.0833	0.287
0.05	0.05	yadI	4	15.0833	0.58
0.05	0.05	AG1	4	15.0833	0.7
0.05	0.05	ptsI	4	15.3333	0.625
0.05	0.05	clcB	4	15.3333	0.316
0.05	0.05	ycaM	4	15.3333	0.292
0.05	0.05	yadI	4	15.3333	0.568
0.05	0.05	AG1	4	15.3333	0.705
0.05	0.05	ptsI	4	15.5833	0.629
0.05	0.05	clcB	4	15.5833	0.316
0.05	0.05	ycaM	4	15.5833	0.292
0.05	0.05	yadI	4	15.5833	0.572
0.05	0.05	AG1	4	15.5833	0.707
0.05	0.05	ptsI	4	15.8333	0.63
0.05	0.05	clcB	4	15.8333	0.318
0.05	0.05	ycaM	4	15.8333	0.292
0.05	0.05	yadI	4	15.8333	0.573
0.05	0.05	AG1	4	15.8333	0.71
0.05	0.05	ptsI	4	16.0833	0.635
0.05	0.05	clcB	4	16.0833	0.324
0.05	0.05	ycaM	4	16.0833	0.291
0.05	0.05	yadI	4	16.0833	0.576
0.05	0.05	AG1	4	16.0833	0.712
0.05	0.05	ptsI	4	16.3333	0.634
0.05	0.05	clcB	4	16.3333	0.322
0.05	0.05	ycaM	4	16.3333	0.295
0.05	0.05	yadI	4	16.3333	0.57
0.05	0.05	AG1	4	16.3333	0.716
0.05	0.05	ptsI	4	16.5833	0.637
0.05	0.05	clcB	4	16.5833	0.327
0.05	0.05	ycaM	4	16.5833	0.299
0.05	0.05	yadI	4	16.5833	0.575
0.05	0.05	AG1	4	16.5833	0.719

0.05	0.05	ptsI	4	16.8333	0.637
0.05	0.05	clcB	4	16.8333	0.328
0.05	0.05	ycaM	4	16.8333	0.296
0.05	0.05	yadI	4	16.8333	0.576
0.05	0.05	AG1	4	16.8333	0.718
0.05	0.05	ptsI	4	17.0833	0.637
0.05	0.05	clcB	4	17.0833	0.327
0.05	0.05	ycaM	4	17.0833	0.299
0.05	0.05	yadI	4	17.0833	0.568
0.05	0.05	AG1	4	17.0833	0.724
0.05	0.05	ptsI	4	17.3333	0.643
0.05	0.05	clcB	4	17.3333	0.332
0.05	0.05	ycaM	4	17.3333	0.302
0.05	0.05	yadI	4	17.3333	0.566
0.05	0.05	AG1	4	17.3333	0.727
0.05	0.05	ptsI	4	17.5833	0.642
0.05	0.05	clcB	4	17.5833	0.337
0.05	0.05	ycaM	4	17.5833	0.305
0.05	0.05	yadI	4	17.5833	0.572
0.05	0.05	AG1	4	17.5833	0.727
0.05	0.05	ptsI	4	17.8333	0.638
0.05	0.05	clcB	4	17.8333	0.339
0.05	0.05	ycaM	4	17.8333	0.299
0.05	0.05	yadI	4	17.8333	0.577
0.05	0.05	AG1	4	17.8333	0.734
0.05	0.05	ptsI	4	18.0833	0.642
0.05	0.05	clcB	4	18.0833	0.328
0.05	0.05	ycaM	4	18.0833	0.306
0.05	0.05	yadI	4	18.0833	0.571
0.05	0.05	AG1	4	18.0833	0.738
0.05	0.05	ptsI	4	18.3333	0.644
0.05	0.05	clcB	4	18.3333	0.328
0.05	0.05	ycaM	4	18.3333	0.312
0.05	0.05	yadI	4	18.3333	0.572
0.05	0.05	AG1	4	18.3333	0.734
0.05	0.05	ptsI	4	18.5833	0.645
0.05	0.05	clcB	4	18.5833	0.329
0.05	0.05	ycaM	4	18.5833	0.313
0.05	0.05	yadI	4	18.5833	0.569
0.05	0.05	AG1	4	18.5833	0.74
0.05	0.05	ptsI	4	18.8333	0.644
0.05	0.05	clcB	4	18.8333	0.329
0.05	0.05	ycaM	4	18.8333	0.311
0.05	0.05	yadI	4	18.8333	0.57
0.05	0.05	AG1	4	18.8333	0.753
0.05	0.05	ptsI	4	19.0833	0.649
0.05	0.05	clcB	4	19.0833	0.338
0.05	0.05	ycaM	4	19.0833	0.31
0.05	0.05	yadI	4	19.0833	0.567
0.05	0.05	AG1	4	19.0833	0.749
0.05	0.05	ptsI	4	19.3333	0.649
0.05	0.05	clcB	4	19.3333	0.319
0.05	0.05	ycaM	4	19.3333	0.329

0.05	0.05	yadI	4	19.3333	0.557
0.05	0.05	AG1	4	19.3333	0.756
0.05	0.05	ptsI	4	19.5833	0.65
0.05	0.05	clcB	4	19.5833	0.337
0.05	0.05	ycaM	4	19.5833	0.313
0.05	0.05	yadI	4	19.5833	0.555
0.05	0.05	AG1	4	19.5833	0.755
0.05	0.05	ptsI	4	19.8333	0.65
0.05	0.05	clcB	4	19.8333	0.33
0.05	0.05	ycaM	4	19.8333	0.315
0.05	0.05	yadI	4	19.8333	0.564
0.05	0.05	AG1	4	19.8333	0.754
0.05	0.05	ptsI	4	20.0833	0.65
0.05	0.05	clcB	4	20.0833	0.328
0.05	0.05	ycaM	4	20.0833	0.324
0.05	0.05	yadI	4	20.0833	0.565
0.05	0.05	AG1	4	20.0833	0.765
0.05	0.05	ptsI	4	20.3333	0.649
0.05	0.05	clcB	4	20.3333	0.317
0.05	0.05	ycaM	4	20.3333	0.341
0.05	0.05	yadI	4	20.3333	0.555
0.05	0.05	AG1	4	20.3333	0.757
0.05	0.05	ptsI	4	20.5833	0.648
0.05	0.05	clcB	4	20.5833	0.317
0.05	0.05	ycaM	4	20.5833	0.328
0.05	0.05	yadI	4	20.5833	0.56
0.05	0.05	AG1	4	20.5833	0.767
0.05	0.05	ptsI	4	20.8333	0.652
0.05	0.05	clcB	4	20.8333	0.322
0.05	0.05	ycaM	4	20.8333	0.36
0.05	0.05	yadI	4	20.8333	0.552
0.05	0.05	AG1	4	20.8333	0.765
0.05	0.05	ptsI	4	21.0833	0.648
0.05	0.05	clcB	4	21.0833	0.323
0.05	0.05	ycaM	4	21.0833	0.336
0.05	0.05	yadI	4	21.0833	0.55
0.05	0.05	AG1	4	21.0833	0.767
0.05	0.05	ptsI	4	21.3333	0.653
0.05	0.05	clcB	4	21.3333	0.324
0.05	0.05	ycaM	4	21.3333	0.34
0.05	0.05	yadI	4	21.3333	0.548
0.05	0.05	AG1	4	21.3333	0.769
0.05	0.05	ptsI	4	21.5833	0.655
0.05	0.05	clcB	4	21.5833	0.335
0.05	0.05	ycaM	4	21.5833	0.33
0.05	0.05	yadI	4	21.5833	0.549
0.05	0.05	AG1	4	21.5833	0.774
0.05	0.05	ptsI	4	21.8333	0.648
0.05	0.05	clcB	4	21.8333	0.331
0.05	0.05	ycaM	4	21.8333	0.344
0.05	0.05	yadI	4	21.8333	0.539
0.05	0.05	AG1	4	21.8333	0.777
0.05	0.05	ptsI	4	22.0833	0.652

0.05	0.05	clcB	4	22.0833	0.33
0.05	0.05	ycaM	4	22.0833	0.335
0.05	0.05	yadI	4	22.0833	0.547
0.05	0.05	AG1	4	22.0833	0.776
0.05	0.05	ptsI	4	22.3333	0.653
0.05	0.05	clcB	4	22.3333	0.331
0.05	0.05	ycaM	4	22.3333	0.324
0.05	0.05	yadI	4	22.3333	0.543
0.05	0.05	AG1	4	22.3333	0.78
0.05	0.05	ptsI	4	22.5833	0.647
0.05	0.05	clcB	4	22.5833	0.334
0.05	0.05	ycaM	4	22.5833	0.324
0.05	0.05	yadI	4	22.5833	0.54
0.05	0.05	AG1	4	22.5833	0.783
0.05	0.05	ptsI	4	22.8333	0.64
0.05	0.05	clcB	4	22.8333	0.335
0.05	0.05	ycaM	4	22.8333	0.328
0.05	0.05	yadI	4	22.8333	0.539
0.05	0.05	AG1	4	22.8333	0.784
0.05	0.05	ptsI	4	23.0833	0.652
0.05	0.05	clcB	4	23.0833	0.344
0.05	0.05	ycaM	4	23.0833	0.326
0.05	0.05	yadI	4	23.0833	0.542
0.05	0.05	AG1	4	23.0833	0.789
0.05	0.05	ptsI	4	23.3333	0.65
0.05	0.05	clcB	4	23.3333	0.343
0.05	0.05	ycaM	4	23.3333	0.331
0.05	0.05	yadI	4	23.3333	0.539
0.05	0.05	AG1	4	23.3333	0.789
0.05	0.05	ptsI	4	23.5833	0.64
0.05	0.05	clcB	4	23.5833	0.336
0.05	0.05	ycaM	4	23.5833	0.332
0.05	0.05	yadI	4	23.5833	0.542
0.05	0.05	AG1	4	23.5833	0.79
0.05	0.05	ptsI	4	23.8333	0.638
0.05	0.05	clcB	4	23.8333	0.345
0.05	0.05	ycaM	4	23.8333	0.333
0.05	0.05	yadI	4	23.8333	0.543
0.05	0.05	AG1	4	23.8333	0.794
0.05	0.05	ptsI	4	24.0833	0.642
0.05	0.05	clcB	4	24.0833	0.348
0.05	0.05	ycaM	4	24.0833	0.334
0.05	0.05	yadI	4	24.0833	0.541
0.05	0.05	AG1	4	24.0833	0.794
0.05	0.05	ptsI	5	0	0.161
0.05	0.05	clcB	5	0	0.157
0.05	0.05	ycaM	5	0	0.165
0.05	0.05	yadI	5	0	0.17
0.05	0.05	AG1	5	0	0.163
0.05	0.05	ptsI	5	0.35	0.158
0.05	0.05	clcB	5	0.35	0.154
0.05	0.05	ycaM	5	0.35	0.159
0.05	0.05	yadI	5	0.35	0.167

0.05	0.05	AG1	5	0.35	0.158
0.05	0.05	ptsI	5	0.6	0.158
0.05	0.05	clcB	5	0.6	0.156
0.05	0.05	ycaM	5	0.6	0.159
0.05	0.05	yadI	5	0.6	0.167
0.05	0.05	AG1	5	0.6	0.157
0.05	0.05	ptsI	5	0.85	0.157
0.05	0.05	clcB	5	0.85	0.156
0.05	0.05	ycaM	5	0.85	0.159
0.05	0.05	yadI	5	0.85	0.167
0.05	0.05	AG1	5	0.85	0.159
0.05	0.05	ptsI	5	1.1	0.159
0.05	0.05	clcB	5	1.1	0.158
0.05	0.05	ycaM	5	1.1	0.162
0.05	0.05	yadI	5	1.1	0.168
0.05	0.05	AG1	5	1.1	0.162
0.05	0.05	ptsI	5	1.35	0.161
0.05	0.05	clcB	5	1.35	0.16
0.05	0.05	ycaM	5	1.35	0.163
0.05	0.05	yadI	5	1.35	0.17
0.05	0.05	AG1	5	1.35	0.163
0.05	0.05	ptsI	5	1.6	0.163
0.05	0.05	clcB	5	1.6	0.16
0.05	0.05	ycaM	5	1.6	0.165
0.05	0.05	yadI	5	1.6	0.173
0.05	0.05	AG1	5	1.6	0.167
0.05	0.05	ptsI	5	1.85	0.166
0.05	0.05	clcB	5	1.85	0.164
0.05	0.05	ycaM	5	1.85	0.17
0.05	0.05	yadI	5	1.85	0.176
0.05	0.05	AG1	5	1.85	0.169
0.05	0.05	ptsI	5	2.1	0.17
0.05	0.05	clcB	5	2.1	0.167
0.05	0.05	ycaM	5	2.1	0.175
0.05	0.05	yadI	5	2.1	0.181
0.05	0.05	AG1	5	2.1	0.174
0.05	0.05	ptsI	5	2.35	0.175
0.05	0.05	clcB	5	2.35	0.172
0.05	0.05	ycaM	5	2.35	0.18
0.05	0.05	yadI	5	2.35	0.185
0.05	0.05	AG1	5	2.35	0.176
0.05	0.05	ptsI	5	2.6	0.175
0.05	0.05	clcB	5	2.6	0.174
0.05	0.05	ycaM	5	2.6	0.184
0.05	0.05	yadI	5	2.6	0.187
0.05	0.05	AG1	5	2.6	0.182
0.05	0.05	ptsI	5	2.85	0.183
0.05	0.05	clcB	5	2.85	0.181
0.05	0.05	ycaM	5	2.85	0.192
0.05	0.05	yadI	5	2.85	0.196
0.05	0.05	AG1	5	2.85	0.186
0.05	0.05	ptsI	5	3.1	0.187
0.05	0.05	clcB	5	3.1	0.189

0.05	0.05	ycaM	5	3.1	0.198
0.05	0.05	yadI	5	3.1	0.203
0.05	0.05	AG1	5	3.1	0.192
0.05	0.05	ptsI	5	3.35	0.196
0.05	0.05	clcB	5	3.35	0.198
0.05	0.05	ycaM	5	3.35	0.209
0.05	0.05	yadI	5	3.35	0.212
0.05	0.05	AG1	5	3.35	0.199
0.05	0.05	ptsI	5	3.6	0.205
0.05	0.05	clcB	5	3.6	0.209
0.05	0.05	ycaM	5	3.6	0.22
0.05	0.05	yadI	5	3.6	0.224
0.05	0.05	AG1	5	3.6	0.206
0.05	0.05	ptsI	5	3.85	0.221
0.05	0.05	clcB	5	3.85	0.219
0.05	0.05	ycaM	5	3.85	0.232
0.05	0.05	yadI	5	3.85	0.233
0.05	0.05	AG1	5	3.85	0.214
0.05	0.05	ptsI	5	4.38333	0.233
0.05	0.05	clcB	5	4.38333	0.267
0.05	0.05	ycaM	5	4.38333	0.262
0.05	0.05	yadI	5	4.38333	0.278
0.05	0.05	AG1	5	4.38333	0.252
0.05	0.05	ptsI	5	4.63333	0.239
0.05	0.05	clcB	5	4.63333	0.261
0.05	0.05	ycaM	5	4.63333	0.254
0.05	0.05	yadI	5	4.63333	0.279
0.05	0.05	AG1	5	4.63333	0.263
0.05	0.05	ptsI	5	4.88333	0.249
0.05	0.05	clcB	5	4.88333	0.284
0.05	0.05	ycaM	5	4.88333	0.268
0.05	0.05	yadI	5	4.88333	0.296
0.05	0.05	AG1	5	4.88333	0.284
0.05	0.05	ptsI	5	5.13333	0.256
0.05	0.05	clcB	5	5.13333	0.297
0.05	0.05	ycaM	5	5.13333	0.273
0.05	0.05	yadI	5	5.13333	0.312
0.05	0.05	AG1	5	5.13333	0.299
0.05	0.05	ptsI	5	5.38333	0.269
0.05	0.05	clcB	5	5.38333	0.306
0.05	0.05	ycaM	5	5.38333	0.277
0.05	0.05	yadI	5	5.38333	0.329
0.05	0.05	AG1	5	5.38333	0.315
0.05	0.05	ptsI	5	5.63333	0.28
0.05	0.05	clcB	5	5.63333	0.313
0.05	0.05	ycaM	5	5.63333	0.284
0.05	0.05	yadI	5	5.63333	0.342
0.05	0.05	AG1	5	5.63333	0.329
0.05	0.05	ptsI	5	5.88333	0.291
0.05	0.05	clcB	5	5.88333	0.321
0.05	0.05	ycaM	5	5.88333	0.288
0.05	0.05	yadI	5	5.88333	0.357
0.05	0.05	AG1	5	5.88333	0.34

0.05	0.05	ptsI	5	6.13333	0.303
0.05	0.05	clcB	5	6.13333	0.33
0.05	0.05	ycaM	5	6.13333	0.292
0.05	0.05	yadI	5	6.13333	0.37
0.05	0.05	AG1	5	6.13333	0.35
0.05	0.05	ptsI	5	6.38333	0.316
0.05	0.05	clcB	5	6.38333	0.334
0.05	0.05	ycaM	5	6.38333	0.298
0.05	0.05	yadI	5	6.38333	0.384
0.05	0.05	AG1	5	6.38333	0.366
0.05	0.05	ptsI	5	6.63333	0.326
0.05	0.05	clcB	5	6.63333	0.34
0.05	0.05	ycaM	5	6.63333	0.3
0.05	0.05	yadI	5	6.63333	0.398
0.05	0.05	AG1	5	6.63333	0.381
0.05	0.05	ptsI	5	6.88333	0.339
0.05	0.05	clcB	5	6.88333	0.343
0.05	0.05	ycaM	5	6.88333	0.302
0.05	0.05	yadI	5	6.88333	0.413
0.05	0.05	AG1	5	6.88333	0.394
0.05	0.05	ptsI	5	7.13333	0.352
0.05	0.05	clcB	5	7.13333	0.348
0.05	0.05	ycaM	5	7.13333	0.306
0.05	0.05	yadI	5	7.13333	0.428
0.05	0.05	AG1	5	7.13333	0.409
0.05	0.05	ptsI	5	7.38333	0.362
0.05	0.05	clcB	5	7.38333	0.351
0.05	0.05	ycaM	5	7.38333	0.309
0.05	0.05	yadI	5	7.38333	0.443
0.05	0.05	AG1	5	7.38333	0.425
0.05	0.05	ptsI	5	7.63333	0.374
0.05	0.05	clcB	5	7.63333	0.354
0.05	0.05	ycaM	5	7.63333	0.31
0.05	0.05	yadI	5	7.63333	0.456
0.05	0.05	AG1	5	7.63333	0.438
0.05	0.05	ptsI	5	7.88333	0.384
0.05	0.05	clcB	5	7.88333	0.358
0.05	0.05	ycaM	5	7.88333	0.313
0.05	0.05	yadI	5	7.88333	0.468
0.05	0.05	AG1	5	7.88333	0.454
0.05	0.05	ptsI	5	8.13333	0.398
0.05	0.05	clcB	5	8.13333	0.361
0.05	0.05	ycaM	5	8.13333	0.317
0.05	0.05	yadI	5	8.13333	0.477
0.05	0.05	AG1	5	8.13333	0.468
0.05	0.05	ptsI	5	8.38333	0.409
0.05	0.05	clcB	5	8.38333	0.364
0.05	0.05	ycaM	5	8.38333	0.316
0.05	0.05	yadI	5	8.38333	0.486
0.05	0.05	AG1	5	8.38333	0.484
0.05	0.05	ptsI	5	8.88333	0.34
0.05	0.05	clcB	5	8.88333	0.299
0.05	0.05	ycaM	5	8.88333	0.312

0.05	0.05	yadI	5	8.88333	0.442
0.05	0.05	AG1	5	8.88333	0.468
0.05	0.05	ptsI	5	9.13333	0.349
0.05	0.05	clcB	5	9.13333	0.294
0.05	0.05	ycaM	5	9.13333	0.308
0.05	0.05	yadI	5	9.13333	0.438
0.05	0.05	AG1	5	9.13333	0.475
0.05	0.05	ptsI	5	9.38333	0.361
0.05	0.05	clcB	5	9.38333	0.298
0.05	0.05	ycaM	5	9.38333	0.309
0.05	0.05	yadI	5	9.38333	0.446
0.05	0.05	AG1	5	9.38333	0.463
0.05	0.05	ptsI	5	9.63333	0.372
0.05	0.05	clcB	5	9.63333	0.307
0.05	0.05	ycaM	5	9.63333	0.311
0.05	0.05	yadI	5	9.63333	0.46
0.05	0.05	AG1	5	9.63333	0.501
0.05	0.05	ptsI	5	9.88333	0.378
0.05	0.05	clcB	5	9.88333	0.305
0.05	0.05	ycaM	5	9.88333	0.311
0.05	0.05	yadI	5	9.88333	0.47
0.05	0.05	AG1	5	9.88333	0.511
0.05	0.05	ptsI	5	10.1333	0.389
0.05	0.05	clcB	5	10.1333	0.308
0.05	0.05	ycaM	5	10.1333	0.309
0.05	0.05	yadI	5	10.1333	0.478
0.05	0.05	AG1	5	10.1333	0.518
0.05	0.05	ptsI	5	10.3833	0.398
0.05	0.05	clcB	5	10.3833	0.313
0.05	0.05	ycaM	5	10.3833	0.31
0.05	0.05	yadI	5	10.3833	0.487
0.05	0.05	AG1	5	10.3833	0.53
0.05	0.05	ptsI	5	10.6333	0.406
0.05	0.05	clcB	5	10.6333	0.311
0.05	0.05	ycaM	5	10.6333	0.312
0.05	0.05	yadI	5	10.6333	0.497
0.05	0.05	AG1	5	10.6333	0.545
0.05	0.05	ptsI	5	10.8833	0.418
0.05	0.05	clcB	5	10.8833	0.316
0.05	0.05	ycaM	5	10.8833	0.312
0.05	0.05	yadI	5	10.8833	0.504
0.05	0.05	AG1	5	10.8833	0.556
0.05	0.05	ptsI	5	11.1333	0.425
0.05	0.05	clcB	5	11.1333	0.31
0.05	0.05	ycaM	5	11.1333	0.31
0.05	0.05	yadI	5	11.1333	0.51
0.05	0.05	AG1	5	11.1333	0.566
0.05	0.05	ptsI	5	11.3833	0.435
0.05	0.05	clcB	5	11.3833	0.31
0.05	0.05	ycaM	5	11.3833	0.309
0.05	0.05	yadI	5	11.3833	0.518
0.05	0.05	AG1	5	11.3833	0.577
0.05	0.05	ptsI	5	11.6333	0.443

0.05	0.05	clcB	5	11.6333	0.309
0.05	0.05	ycaM	5	11.6333	0.31
0.05	0.05	yadI	5	11.6333	0.526
0.05	0.05	AG1	5	11.6333	0.589
0.05	0.05	ptsI	5	11.8833	0.451
0.05	0.05	clcB	5	11.8833	0.315
0.05	0.05	ycaM	5	11.8833	0.312
0.05	0.05	yadI	5	11.8833	0.532
0.05	0.05	AG1	5	11.8833	0.601
0.05	0.05	ptsI	5	12.1333	0.458
0.05	0.05	clcB	5	12.1333	0.312
0.05	0.05	ycaM	5	12.1333	0.313
0.05	0.05	yadI	5	12.1333	0.54
0.05	0.05	AG1	5	12.1333	0.612
0.05	0.05	ptsI	5	12.3833	0.465
0.05	0.05	clcB	5	12.3833	0.311
0.05	0.05	ycaM	5	12.3833	0.311
0.05	0.05	yadI	5	12.3833	0.539
0.05	0.05	AG1	5	12.3833	0.612
0.05	0.05	ptsI	5	12.6333	0.473
0.05	0.05	clcB	5	12.6333	0.312
0.05	0.05	ycaM	5	12.6333	0.314
0.05	0.05	yadI	5	12.6333	0.558
0.05	0.05	AG1	5	12.6333	0.631
0.05	0.05	ptsI	5	12.8833	0.484
0.05	0.05	clcB	5	12.8833	0.311
0.05	0.05	ycaM	5	12.8833	0.316
0.05	0.05	yadI	5	12.8833	0.561
0.05	0.05	AG1	5	12.8833	0.642
0.05	0.05	ptsI	5	13.1333	0.493
0.05	0.05	clcB	5	13.1333	0.314
0.05	0.05	ycaM	5	13.1333	0.315
0.05	0.05	yadI	5	13.1333	0.566
0.05	0.05	AG1	5	13.1333	0.648
0.05	0.05	ptsI	5	13.3833	0.496
0.05	0.05	clcB	5	13.3833	0.31
0.05	0.05	ycaM	5	13.3833	0.314
0.05	0.05	yadI	5	13.3833	0.572
0.05	0.05	AG1	5	13.3833	0.655
0.05	0.05	ptsI	5	13.6333	0.508
0.05	0.05	clcB	5	13.6333	0.316
0.05	0.05	ycaM	5	13.6333	0.317
0.05	0.05	yadI	5	13.6333	0.577
0.05	0.05	AG1	5	13.6333	0.66
0.05	0.05	ptsI	5	13.8833	0.512
0.05	0.05	clcB	5	13.8833	0.318
0.05	0.05	ycaM	5	13.8833	0.318
0.05	0.05	yadI	5	13.8833	0.585
0.05	0.05	AG1	5	13.8833	0.672
0.05	0.05	ptsI	5	14.1333	0.522
0.05	0.05	clcB	5	14.1333	0.314
0.05	0.05	ycaM	5	14.1333	0.317
0.05	0.05	yadI	5	14.1333	0.589

0.05	0.05	AG1	5	14.1333	0.676
0.05	0.05	ptsI	5	14.3833	0.53
0.05	0.05	clcB	5	14.3833	0.309
0.05	0.05	ycaM	5	14.3833	0.32
0.05	0.05	yadI	5	14.3833	0.596
0.05	0.05	AG1	5	14.3833	0.682
0.05	0.05	ptsI	5	14.6333	0.542
0.05	0.05	clcB	5	14.6333	0.312
0.05	0.05	ycaM	5	14.6333	0.32
0.05	0.05	yadI	5	14.6333	0.602
0.05	0.05	AG1	5	14.6333	0.687
0.05	0.05	ptsI	5	14.8833	0.543
0.05	0.05	clcB	5	14.8833	0.314
0.05	0.05	ycaM	5	14.8833	0.32
0.05	0.05	yadI	5	14.8833	0.603
0.05	0.05	AG1	5	14.8833	0.692
0.05	0.05	ptsI	5	15.1333	0.55
0.05	0.05	clcB	5	15.1333	0.313
0.05	0.05	ycaM	5	15.1333	0.322
0.05	0.05	yadI	5	15.1333	0.607
0.05	0.05	AG1	5	15.1333	0.697
0.05	0.05	ptsI	5	15.3833	0.557
0.05	0.05	clcB	5	15.3833	0.312
0.05	0.05	ycaM	5	15.3833	0.322
0.05	0.05	yadI	5	15.3833	0.61
0.05	0.05	AG1	5	15.3833	0.703
0.05	0.05	ptsI	5	15.6333	0.561
0.05	0.05	clcB	5	15.6333	0.306
0.05	0.05	ycaM	5	15.6333	0.322
0.05	0.05	yadI	5	15.6333	0.614
0.05	0.05	AG1	5	15.6333	0.709
0.05	0.05	ptsI	5	15.8833	0.567
0.05	0.05	clcB	5	15.8833	0.31
0.05	0.05	ycaM	5	15.8833	0.323
0.05	0.05	yadI	5	15.8833	0.618
0.05	0.05	AG1	5	15.8833	0.713
0.05	0.05	ptsI	5	16.1333	0.575
0.05	0.05	clcB	5	16.1333	0.315
0.05	0.05	ycaM	5	16.1333	0.322
0.05	0.05	yadI	5	16.1333	0.621
0.05	0.05	AG1	5	16.1333	0.685
0.05	0.05	ptsI	5	16.3833	0.578
0.05	0.05	clcB	5	16.3833	0.312
0.05	0.05	ycaM	5	16.3833	0.323
0.05	0.05	yadI	5	16.3833	0.628
0.05	0.05	AG1	5	16.3833	0.723
0.05	0.05	ptsI	5	16.6333	0.583
0.05	0.05	clcB	5	16.6333	0.313
0.05	0.05	ycaM	5	16.6333	0.325
0.05	0.05	yadI	5	16.6333	0.629
0.05	0.05	AG1	5	16.6333	0.725
0.05	0.05	ptsI	5	16.8833	0.591
0.05	0.05	clcB	5	16.8833	0.319

0.05	0.05	ycaM	5	16.8833	0.326
0.05	0.05	yadI	5	16.8833	0.634
0.05	0.05	AG1	5	16.8833	0.729
0.05	0.05	ptsI	5	17.1333	0.594
0.05	0.05	clcB	5	17.1333	0.317
0.05	0.05	ycaM	5	17.1333	0.325
0.05	0.05	yadI	5	17.1333	0.637
0.05	0.05	AG1	5	17.1333	0.738
0.05	0.05	ptsI	5	17.3833	0.595
0.05	0.05	clcB	5	17.3833	0.318
0.05	0.05	ycaM	5	17.3833	0.327
0.05	0.05	yadI	5	17.3833	0.64
0.05	0.05	AG1	5	17.3833	0.736
0.05	0.05	ptsI	5	17.6333	0.601
0.05	0.05	clcB	5	17.6333	0.324
0.05	0.05	ycaM	5	17.6333	0.325
0.05	0.05	yadI	5	17.6333	0.642
0.05	0.05	AG1	5	17.6333	0.742
0.05	0.05	ptsI	5	17.8833	0.606
0.05	0.05	clcB	5	17.8833	0.325
0.05	0.05	ycaM	5	17.8833	0.328
0.05	0.05	yadI	5	17.8833	0.634
0.05	0.05	AG1	5	17.8833	0.734
0.05	0.05	ptsI	5	18.1333	0.612
0.05	0.05	clcB	5	18.1333	0.332
0.05	0.05	ycaM	5	18.1333	0.335
0.05	0.05	yadI	5	18.1333	0.646
0.05	0.05	AG1	5	18.1333	0.751
0.05	0.05	ptsI	5	18.3833	0.618
0.05	0.05	clcB	5	18.3833	0.333
0.05	0.05	ycaM	5	18.3833	0.332
0.05	0.05	yadI	5	18.3833	0.653
0.05	0.05	AG1	5	18.3833	0.753
0.05	0.05	ptsI	5	18.6333	0.622
0.05	0.05	clcB	5	18.6333	0.333
0.05	0.05	ycaM	5	18.6333	0.34
0.05	0.05	yadI	5	18.6333	0.653
0.05	0.05	AG1	5	18.6333	0.756
0.05	0.05	ptsI	5	18.8833	0.625
0.05	0.05	clcB	5	18.8833	0.334
0.05	0.05	ycaM	5	18.8833	0.343
0.05	0.05	yadI	5	18.8833	0.655
0.05	0.05	AG1	5	18.8833	0.76
0.05	0.05	ptsI	5	19.1333	0.63
0.05	0.05	clcB	5	19.1333	0.338
0.05	0.05	ycaM	5	19.1333	0.343
0.05	0.05	yadI	5	19.1333	0.664
0.05	0.05	AG1	5	19.1333	0.768
0.05	0.05	ptsI	5	19.3833	0.626
0.05	0.05	clcB	5	19.3833	0.34
0.05	0.05	ycaM	5	19.3833	0.34
0.05	0.05	yadI	5	19.3833	0.661
0.05	0.05	AG1	5	19.3833	0.77

0.05	0.05	ptsI	5	19.6333	0.632
0.05	0.05	clcB	5	19.6333	0.341
0.05	0.05	ycaM	5	19.6333	0.351
0.05	0.05	yadI	5	19.6333	0.661
0.05	0.05	AG1	5	19.6333	0.768
0.05	0.05	ptsI	5	19.8833	0.633
0.05	0.05	clcB	5	19.8833	0.342
0.05	0.05	ycaM	5	19.8833	0.352
0.05	0.05	yadI	5	19.8833	0.666
0.05	0.05	AG1	5	19.8833	0.774
0.05	0.05	ptsI	5	20.1333	0.638
0.05	0.05	clcB	5	20.1333	0.345
0.05	0.05	ycaM	5	20.1333	0.358
0.05	0.05	yadI	5	20.1333	0.663
0.05	0.05	AG1	5	20.1333	0.774
0.05	0.05	ptsI	5	20.3833	0.639
0.05	0.05	clcB	5	20.3833	0.345
0.05	0.05	ycaM	5	20.3833	0.358
0.05	0.05	yadI	5	20.3833	0.67
0.05	0.05	AG1	5	20.3833	0.776
0.05	0.05	ptsI	5	20.6333	0.642
0.05	0.05	clcB	5	20.6333	0.345
0.05	0.05	ycaM	5	20.6333	0.356
0.05	0.05	yadI	5	20.6333	0.662
0.05	0.05	AG1	5	20.6333	0.812
0.05	0.05	ptsI	5	20.8833	0.643
0.05	0.05	clcB	5	20.8833	0.348
0.05	0.05	ycaM	5	20.8833	0.36
0.05	0.05	yadI	5	20.8833	0.67
0.05	0.05	AG1	5	20.8833	0.778
0.05	0.05	ptsI	5	21.1333	0.644
0.05	0.05	clcB	5	21.1333	0.358
0.05	0.05	ycaM	5	21.1333	0.366
0.05	0.05	yadI	5	21.1333	0.676
0.05	0.05	AG1	5	21.1333	0.784
0.05	0.05	ptsI	5	21.3833	0.64
0.05	0.05	clcB	5	21.3833	0.362
0.05	0.05	ycaM	5	21.3833	0.366
0.05	0.05	yadI	5	21.3833	0.671
0.05	0.05	AG1	5	21.3833	0.783
0.05	0.05	ptsI	5	21.6333	0.636
0.05	0.05	clcB	5	21.6333	0.367
0.05	0.05	ycaM	5	21.6333	0.379
0.05	0.05	yadI	5	21.6333	0.672
0.05	0.05	AG1	5	21.6333	0.785
0.05	0.05	ptsI	5	21.8833	0.638
0.05	0.05	clcB	5	21.8833	0.386
0.05	0.05	ycaM	5	21.8833	0.382
0.05	0.05	yadI	5	21.8833	0.674
0.05	0.05	AG1	5	21.8833	0.789
0.05	0.05	ptsI	5	22.1333	0.634
0.05	0.05	clcB	5	22.1333	0.379
0.05	0.05	ycaM	5	22.1333	0.381

0.05	0.05	yadI	5	22.1333	0.665
0.05	0.05	AG1	5	22.1333	0.778
0.05	0.05	ptsI	5	22.3833	0.635
0.05	0.05	clcB	5	22.3833	0.383
0.05	0.05	ycaM	5	22.3833	0.388
0.05	0.05	yadI	5	22.3833	0.674
0.05	0.05	AG1	5	22.3833	0.792
0.05	0.05	ptsI	5	22.6333	0.638
0.05	0.05	clcB	5	22.6333	0.376
0.05	0.05	ycaM	5	22.6333	0.393
0.05	0.05	yadI	5	22.6333	0.676
0.05	0.05	AG1	5	22.6333	0.798
0.05	0.05	ptsI	5	22.8833	0.641
0.05	0.05	clcB	5	22.8833	0.377
0.05	0.05	ycaM	5	22.8833	0.399
0.05	0.05	yadI	5	22.8833	0.676
0.05	0.05	AG1	5	22.8833	0.795
0.05	0.05	ptsI	5	23.1333	0.646
0.05	0.05	clcB	5	23.1333	0.381
0.05	0.05	ycaM	5	23.1333	0.404
0.05	0.05	yadI	5	23.1333	0.679
0.05	0.05	AG1	5	23.1333	0.802
0.05	0.05	ptsI	5	23.3833	0.643
0.05	0.05	clcB	5	23.3833	0.379
0.05	0.05	ycaM	5	23.3833	0.396
0.05	0.05	yadI	5	23.3833	0.679
0.05	0.05	AG1	5	23.3833	0.8
0.05	0.05	ptsI	5	23.6333	0.647
0.05	0.05	clcB	5	23.6333	0.379
0.05	0.05	ycaM	5	23.6333	0.404
0.05	0.05	yadI	5	23.6333	0.679
0.05	0.05	AG1	5	23.6333	0.804
0.05	0.05	ptsI	5	23.8833	0.643
0.05	0.05	clcB	5	23.8833	0.388
0.05	0.05	ycaM	5	23.8833	0.406
0.05	0.05	yadI	5	23.8833	0.672
0.05	0.05	AG1	5	23.8833	0.79
0.05	0.05	ptsI	5	24.1333	0.642
0.05	0.05	clcB	5	24.1333	0.39
0.05	0.05	ycaM	5	24.1333	0.424
0.05	0.05	yadI	5	24.1333	0.68
0.05	0.05	AG1	5	24.1333	0.806
0.05	0.05	ptsI	5	24.3833	0.647
0.05	0.05	clcB	5	24.3833	0.398
0.05	0.05	ycaM	5	24.3833	0.421
0.05	0.05	yadI	5	24.3833	0.685
0.05	0.05	AG1	5	24.3833	0.811
0.1	0.05	ptsI	2	0	0.216
0.1	0.05	clcB	2	0	0.226
0.1	0.05	ycaM	2	0	0.218
0.1	0.05	yadI	2	0	0.222
0.1	0.05	AG1	2	0	0.224
0.1	0.05	ptsI	2	0.25	0.212

0.1	0.05	clcB	2	0.25	0.221
0.1	0.05	ycaM	2	0.25	0.213
0.1	0.05	yadI	2	0.25	0.215
0.1	0.05	AG1	2	0.25	0.22
0.1	0.05	ptsI	2	0.5	0.209
0.1	0.05	clcB	2	0.5	0.215
0.1	0.05	ycaM	2	0.5	0.213
0.1	0.05	yadI	2	0.5	0.214
0.1	0.05	AG1	2	0.5	0.217
0.1	0.05	ptsI	2	0.75	0.211
0.1	0.05	clcB	2	0.75	0.217
0.1	0.05	ycaM	2	0.75	0.216
0.1	0.05	yadI	2	0.75	0.218
0.1	0.05	AG1	2	0.75	0.218
0.1	0.05	ptsI	2	1	0.213
0.1	0.05	clcB	2	1	0.219
0.1	0.05	ycaM	2	1	0.215
0.1	0.05	yadI	2	1	0.225
0.1	0.05	AG1	2	1	0.225
0.1	0.05	ptsI	2	1.25	0.214
0.1	0.05	clcB	2	1.25	0.217
0.1	0.05	ycaM	2	1.25	0.218
0.1	0.05	yadI	2	1.25	0.232
0.1	0.05	AG1	2	1.25	0.227
0.1	0.05	ptsI	2	1.5	0.215
0.1	0.05	clcB	2	1.5	0.219
0.1	0.05	ycaM	2	1.5	0.221
0.1	0.05	yadI	2	1.5	0.24
0.1	0.05	AG1	2	1.5	0.233
0.1	0.05	ptsI	2	1.75	0.219
0.1	0.05	clcB	2	1.75	0.22
0.1	0.05	ycaM	2	1.75	0.226
0.1	0.05	yadI	2	1.75	0.246
0.1	0.05	AG1	2	1.75	0.236
0.1	0.05	ptsI	2	2	0.221
0.1	0.05	clcB	2	2	0.222
0.1	0.05	ycaM	2	2	0.232
0.1	0.05	yadI	2	2	0.253
0.1	0.05	AG1	2	2	0.246
0.1	0.05	ptsI	2	2.25	0.226
0.1	0.05	clcB	2	2.25	0.227
0.1	0.05	ycaM	2	2.25	0.24
0.1	0.05	yadI	2	2.25	0.264
0.1	0.05	AG1	2	2.25	0.256
0.1	0.05	ptsI	2	2.5	0.231
0.1	0.05	clcB	2	2.5	0.232
0.1	0.05	ycaM	2	2.5	0.246
0.1	0.05	yadI	2	2.5	0.278
0.1	0.05	AG1	2	2.5	0.268
0.1	0.05	ptsI	2	2.75	0.237
0.1	0.05	clcB	2	2.75	0.236
0.1	0.05	ycaM	2	2.75	0.247
0.1	0.05	yadI	2	2.75	0.287

0.1	0.05	AG1	2	2.75	0.277
0.1	0.05	ptsI	2	3	0.243
0.1	0.05	clcB	2	3	0.236
0.1	0.05	ycaM	2	3	0.252
0.1	0.05	yadI	2	3	0.294
0.1	0.05	AG1	2	3	0.28
0.1	0.05	ptsI	2	3.25	0.248
0.1	0.05	clcB	2	3.25	0.243
0.1	0.05	ycaM	2	3.25	0.259
0.1	0.05	yadI	2	3.25	0.311
0.1	0.05	AG1	2	3.25	0.297
0.1	0.05	ptsI	2	3.5	0.254
0.1	0.05	clcB	2	3.5	0.245
0.1	0.05	ycaM	2	3.5	0.264
0.1	0.05	yadI	2	3.5	0.32
0.1	0.05	AG1	2	3.5	0.303
0.1	0.05	ptsI	2	3.75	0.26
0.1	0.05	clcB	2	3.75	0.25
0.1	0.05	ycaM	2	3.75	0.274
0.1	0.05	yadI	2	3.75	0.332
0.1	0.05	AG1	2	3.75	0.317
0.1	0.05	ptsI	2	4	0.265
0.1	0.05	clcB	2	4	0.254
0.1	0.05	ycaM	2	4	0.286
0.1	0.05	yadI	2	4	0.347
0.1	0.05	AG1	2	4	0.329
0.1	0.05	ptsI	2	4.38333	0.306
0.1	0.05	clcB	2	4.38333	0.244
0.1	0.05	ycaM	2	4.38333	0.299
0.1	0.05	yadI	2	4.38333	0.372
0.1	0.05	AG1	2	4.38333	0.306
0.1	0.05	ptsI	2	4.63333	0.298
0.1	0.05	clcB	2	4.63333	0.241
0.1	0.05	ycaM	2	4.63333	0.296
0.1	0.05	yadI	2	4.63333	0.378
0.1	0.05	AG1	2	4.63333	0.303
0.1	0.05	ptsI	2	4.88333	0.304
0.1	0.05	clcB	2	4.88333	0.244
0.1	0.05	ycaM	2	4.88333	0.302
0.1	0.05	yadI	2	4.88333	0.39
0.1	0.05	AG1	2	4.88333	0.312
0.1	0.05	ptsI	2	5.13333	0.311
0.1	0.05	clcB	2	5.13333	0.247
0.1	0.05	ycaM	2	5.13333	0.304
0.1	0.05	yadI	2	5.13333	0.406
0.1	0.05	AG1	2	5.13333	0.32
0.1	0.05	ptsI	2	5.38333	0.319
0.1	0.05	clcB	2	5.38333	0.259
0.1	0.05	ycaM	2	5.38333	0.306
0.1	0.05	yadI	2	5.38333	0.417
0.1	0.05	AG1	2	5.38333	0.329
0.1	0.05	ptsI	2	5.63333	0.325
0.1	0.05	clcB	2	5.63333	0.266

0.1	0.05	ycaM	2	5.63333	0.309
0.1	0.05	yadI	2	5.63333	0.428
0.1	0.05	AG1	2	5.63333	0.339
0.1	0.05	ptsI	2	5.88333	0.331
0.1	0.05	clcB	2	5.88333	0.274
0.1	0.05	ycaM	2	5.88333	0.309
0.1	0.05	yadI	2	5.88333	0.437
0.1	0.05	AG1	2	5.88333	0.348
0.1	0.05	ptsI	2	6.13333	0.338
0.1	0.05	clcB	2	6.13333	0.281
0.1	0.05	ycaM	2	6.13333	0.31
0.1	0.05	yadI	2	6.13333	0.445
0.1	0.05	AG1	2	6.13333	0.358
0.1	0.05	ptsI	2	6.38333	0.345
0.1	0.05	clcB	2	6.38333	0.287
0.1	0.05	ycaM	2	6.38333	0.311
0.1	0.05	yadI	2	6.38333	0.458
0.1	0.05	AG1	2	6.38333	0.366
0.1	0.05	ptsI	2	6.63333	0.354
0.1	0.05	clcB	2	6.63333	0.293
0.1	0.05	ycaM	2	6.63333	0.313
0.1	0.05	yadI	2	6.63333	0.466
0.1	0.05	AG1	2	6.63333	0.378
0.1	0.05	ptsI	2	6.88333	0.362
0.1	0.05	clcB	2	6.88333	0.302
0.1	0.05	ycaM	2	6.88333	0.315
0.1	0.05	yadI	2	6.88333	0.484
0.1	0.05	AG1	2	6.88333	0.387
0.1	0.05	ptsI	2	7.13333	0.369
0.1	0.05	clcB	2	7.13333	0.303
0.1	0.05	ycaM	2	7.13333	0.313
0.1	0.05	yadI	2	7.13333	0.487
0.1	0.05	AG1	2	7.13333	0.398
0.1	0.05	ptsI	2	7.38333	0.377
0.1	0.05	clcB	2	7.38333	0.309
0.1	0.05	ycaM	2	7.38333	0.313
0.1	0.05	yadI	2	7.38333	0.498
0.1	0.05	AG1	2	7.38333	0.409
0.1	0.05	ptsI	2	7.63333	0.386
0.1	0.05	clcB	2	7.63333	0.317
0.1	0.05	ycaM	2	7.63333	0.314
0.1	0.05	yadI	2	7.63333	0.507
0.1	0.05	AG1	2	7.63333	0.417
0.1	0.05	ptsI	2	7.88333	0.393
0.1	0.05	clcB	2	7.88333	0.322
0.1	0.05	ycaM	2	7.88333	0.314
0.1	0.05	yadI	2	7.88333	0.524
0.1	0.05	AG1	2	7.88333	0.427
0.1	0.05	ptsI	2	8.13333	0.401
0.1	0.05	clcB	2	8.13333	0.321
0.1	0.05	ycaM	2	8.13333	0.315
0.1	0.05	yadI	2	8.13333	0.53
0.1	0.05	AG1	2	8.13333	0.434

0.1	0.05	ptsI	2	8.38333	0.409
0.1	0.05	clcB	2	8.38333	0.329
0.1	0.05	ycaM	2	8.38333	0.315
0.1	0.05	yadI	2	8.38333	0.546
0.1	0.05	AG1	2	8.38333	0.446
0.1	0.05	ptsI	2	8.91667	0.357
0.1	0.05	clcB	2	8.91667	0.302
0.1	0.05	ycaM	2	8.91667	0.275
0.1	0.05	yadI	2	8.91667	0.481
0.1	0.05	AG1	2	8.91667	0.472
0.1	0.05	ptsI	2	9.16667	0.356
0.1	0.05	clcB	2	9.16667	0.298
0.1	0.05	ycaM	2	9.16667	0.274
0.1	0.05	yadI	2	9.16667	0.483
0.1	0.05	AG1	2	9.16667	0.484
0.1	0.05	ptsI	2	9.41667	0.365
0.1	0.05	clcB	2	9.41667	0.304
0.1	0.05	ycaM	2	9.41667	0.275
0.1	0.05	yadI	2	9.41667	0.495
0.1	0.05	AG1	2	9.41667	0.505
0.1	0.05	ptsI	2	9.66667	0.369
0.1	0.05	clcB	2	9.66667	0.31
0.1	0.05	ycaM	2	9.66667	0.274
0.1	0.05	yadI	2	9.66667	0.505
0.1	0.05	AG1	2	9.66667	0.516
0.1	0.05	ptsI	2	9.91667	0.378
0.1	0.05	clcB	2	9.91667	0.315
0.1	0.05	ycaM	2	9.91667	0.279
0.1	0.05	yadI	2	9.91667	0.516
0.1	0.05	AG1	2	9.91667	0.528
0.1	0.05	ptsI	2	10.1667	0.385
0.1	0.05	clcB	2	10.1667	0.32
0.1	0.05	ycaM	2	10.1667	0.28
0.1	0.05	yadI	2	10.1667	0.523
0.1	0.05	AG1	2	10.1667	0.53
0.1	0.05	ptsI	2	10.4167	0.395
0.1	0.05	clcB	2	10.4167	0.321
0.1	0.05	ycaM	2	10.4167	0.28
0.1	0.05	yadI	2	10.4167	0.534
0.1	0.05	AG1	2	10.4167	0.543
0.1	0.05	ptsI	2	10.6667	0.402
0.1	0.05	clcB	2	10.6667	0.327
0.1	0.05	ycaM	2	10.6667	0.281
0.1	0.05	yadI	2	10.6667	0.543
0.1	0.05	AG1	2	10.6667	0.556
0.1	0.05	ptsI	2	10.9167	0.413
0.1	0.05	clcB	2	10.9167	0.328
0.1	0.05	ycaM	2	10.9167	0.282
0.1	0.05	yadI	2	10.9167	0.55
0.1	0.05	AG1	2	10.9167	0.574
0.1	0.05	ptsI	2	11.1667	0.423
0.1	0.05	clcB	2	11.1667	0.334
0.1	0.05	ycaM	2	11.1667	0.281

0.1	0.05	yadI	2	11.1667	0.564
0.1	0.05	AG1	2	11.1667	0.59
0.1	0.05	ptsI	2	11.4167	0.432
0.1	0.05	clcB	2	11.4167	0.335
0.1	0.05	ycaM	2	11.4167	0.283
0.1	0.05	yadI	2	11.4167	0.565
0.1	0.05	AG1	2	11.4167	0.597
0.1	0.05	ptsI	2	11.6667	0.439
0.1	0.05	clcB	2	11.6667	0.337
0.1	0.05	ycaM	2	11.6667	0.282
0.1	0.05	yadI	2	11.6667	0.572
0.1	0.05	AG1	2	11.6667	0.626
0.1	0.05	ptsI	2	11.9167	0.446
0.1	0.05	clcB	2	11.9167	0.337
0.1	0.05	ycaM	2	11.9167	0.282
0.1	0.05	yadI	2	11.9167	0.579
0.1	0.05	AG1	2	11.9167	0.626
0.1	0.05	ptsI	2	12.1667	0.458
0.1	0.05	clcB	2	12.1667	0.331
0.1	0.05	ycaM	2	12.1667	0.278
0.1	0.05	yadI	2	12.1667	0.588
0.1	0.05	AG1	2	12.1667	0.648
0.1	0.05	ptsI	2	12.4167	0.466
0.1	0.05	clcB	2	12.4167	0.33
0.1	0.05	ycaM	2	12.4167	0.276
0.1	0.05	yadI	2	12.4167	0.59
0.1	0.05	AG1	2	12.4167	0.662
0.1	0.05	ptsI	2	12.6667	0.465
0.1	0.05	clcB	2	12.6667	0.33
0.1	0.05	ycaM	2	12.6667	0.274
0.1	0.05	yadI	2	12.6667	0.599
0.1	0.05	AG1	2	12.6667	0.671
0.1	0.05	ptsI	2	12.9167	0.477
0.1	0.05	clcB	2	12.9167	0.325
0.1	0.05	ycaM	2	12.9167	0.277
0.1	0.05	yadI	2	12.9167	0.609
0.1	0.05	AG1	2	12.9167	0.677
0.1	0.05	ptsI	2	13.1667	0.485
0.1	0.05	clcB	2	13.1667	0.327
0.1	0.05	ycaM	2	13.1667	0.279
0.1	0.05	yadI	2	13.1667	0.616
0.1	0.05	AG1	2	13.1667	0.684
0.1	0.05	ptsI	2	13.4167	0.486
0.1	0.05	clcB	2	13.4167	0.327
0.1	0.05	ycaM	2	13.4167	0.278
0.1	0.05	yadI	2	13.4167	0.618
0.1	0.05	AG1	2	13.4167	0.682
0.1	0.05	ptsI	2	13.6667	0.494
0.1	0.05	clcB	2	13.6667	0.328
0.1	0.05	ycaM	2	13.6667	0.278
0.1	0.05	yadI	2	13.6667	0.628
0.1	0.05	AG1	2	13.6667	0.707
0.1	0.05	ptsI	2	13.9167	0.5

0.1	0.05	clcB	2	13.9167	0.325
0.1	0.05	ycaM	2	13.9167	0.282
0.1	0.05	yadI	2	13.9167	0.637
0.1	0.05	AG1	2	13.9167	0.704
0.1	0.05	ptsI	2	14.1667	0.503
0.1	0.05	clcB	2	14.1667	0.321
0.1	0.05	ycaM	2	14.1667	0.281
0.1	0.05	yadI	2	14.1667	0.636
0.1	0.05	AG1	2	14.1667	0.714
0.1	0.05	ptsI	2	14.4167	0.506
0.1	0.05	clcB	2	14.4167	0.325
0.1	0.05	ycaM	2	14.4167	0.279
0.1	0.05	yadI	2	14.4167	0.645
0.1	0.05	AG1	2	14.4167	0.711
0.1	0.05	ptsI	2	14.6667	0.504
0.1	0.05	clcB	2	14.6667	0.327
0.1	0.05	ycaM	2	14.6667	0.278
0.1	0.05	yadI	2	14.6667	0.648
0.1	0.05	AG1	2	14.6667	0.7
0.1	0.05	ptsI	2	14.9167	0.512
0.1	0.05	clcB	2	14.9167	0.328
0.1	0.05	ycaM	2	14.9167	0.275
0.1	0.05	yadI	2	14.9167	0.66
0.1	0.05	AG1	2	14.9167	0.704
0.1	0.05	ptsI	2	15.1667	0.529
0.1	0.05	clcB	2	15.1667	0.328
0.1	0.05	ycaM	2	15.1667	0.279
0.1	0.05	yadI	2	15.1667	0.663
0.1	0.05	AG1	2	15.1667	0.703
0.1	0.05	ptsI	2	15.4167	0.529
0.1	0.05	clcB	2	15.4167	0.327
0.1	0.05	ycaM	2	15.4167	0.277
0.1	0.05	yadI	2	15.4167	0.67
0.1	0.05	AG1	2	15.4167	0.704
0.1	0.05	ptsI	2	15.6667	0.517
0.1	0.05	clcB	2	15.6667	0.329
0.1	0.05	ycaM	2	15.6667	0.272
0.1	0.05	yadI	2	15.6667	0.672
0.1	0.05	AG1	2	15.6667	0.706
0.1	0.05	ptsI	2	15.9167	0.516
0.1	0.05	clcB	2	15.9167	0.33
0.1	0.05	ycaM	2	15.9167	0.274
0.1	0.05	yadI	2	15.9167	0.678
0.1	0.05	AG1	2	15.9167	0.716
0.1	0.05	ptsI	2	16.1667	0.528
0.1	0.05	clcB	2	16.1667	0.33
0.1	0.05	ycaM	2	16.1667	0.273
0.1	0.05	yadI	2	16.1667	0.682
0.1	0.05	AG1	2	16.1667	0.727
0.1	0.05	ptsI	2	16.4167	0.527
0.1	0.05	clcB	2	16.4167	0.33
0.1	0.05	ycaM	2	16.4167	0.275
0.1	0.05	yadI	2	16.4167	0.69

0.1	0.05	AG1	2	16.4167	0.72
0.1	0.05	ptsI	2	16.6667	0.528
0.1	0.05	clcB	2	16.6667	0.328
0.1	0.05	ycaM	2	16.6667	0.274
0.1	0.05	yadI	2	16.6667	0.694
0.1	0.05	AG1	2	16.6667	0.729
0.1	0.05	ptsI	2	16.9167	0.53
0.1	0.05	clcB	2	16.9167	0.33
0.1	0.05	ycaM	2	16.9167	0.275
0.1	0.05	yadI	2	16.9167	0.699
0.1	0.05	AG1	2	16.9167	0.741
0.1	0.05	ptsI	2	17.1667	0.539
0.1	0.05	clcB	2	17.1667	0.331
0.1	0.05	ycaM	2	17.1667	0.274
0.1	0.05	yadI	2	17.1667	0.708
0.1	0.05	AG1	2	17.1667	0.731
0.1	0.05	ptsI	2	17.4167	0.542
0.1	0.05	clcB	2	17.4167	0.328
0.1	0.05	ycaM	2	17.4167	0.275
0.1	0.05	yadI	2	17.4167	0.711
0.1	0.05	AG1	2	17.4167	0.739
0.1	0.05	ptsI	2	17.6667	0.552
0.1	0.05	clcB	2	17.6667	0.332
0.1	0.05	ycaM	2	17.6667	0.276
0.1	0.05	yadI	2	17.6667	0.717
0.1	0.05	AG1	2	17.6667	0.726
0.1	0.05	ptsI	2	17.9167	0.545
0.1	0.05	clcB	2	17.9167	0.331
0.1	0.05	ycaM	2	17.9167	0.273
0.1	0.05	yadI	2	17.9167	0.722
0.1	0.05	AG1	2	17.9167	0.727
0.1	0.05	ptsI	2	18.1667	0.55
0.1	0.05	clcB	2	18.1667	0.33
0.1	0.05	ycaM	2	18.1667	0.277
0.1	0.05	yadI	2	18.1667	0.718
0.1	0.05	AG1	2	18.1667	0.74
0.1	0.05	ptsI	2	18.4167	0.556
0.1	0.05	clcB	2	18.4167	0.333
0.1	0.05	ycaM	2	18.4167	0.278
0.1	0.05	yadI	2	18.4167	0.728
0.1	0.05	AG1	2	18.4167	0.737
0.1	0.05	ptsI	2	18.6667	0.547
0.1	0.05	clcB	2	18.6667	0.333
0.1	0.05	ycaM	2	18.6667	0.278
0.1	0.05	yadI	2	18.6667	0.729
0.1	0.05	AG1	2	18.6667	0.743
0.1	0.05	ptsI	2	18.9167	0.536
0.1	0.05	clcB	2	18.9167	0.33
0.1	0.05	ycaM	2	18.9167	0.278
0.1	0.05	yadI	2	18.9167	0.727
0.1	0.05	AG1	2	18.9167	0.744
0.1	0.05	ptsI	2	19.1667	0.556
0.1	0.05	clcB	2	19.1667	0.331

0.1	0.05	ycaM	2	19.1667	0.28
0.1	0.05	yadI	2	19.1667	0.727
0.1	0.05	AG1	2	19.1667	0.76
0.1	0.05	ptsI	2	19.4167	0.553
0.1	0.05	clcB	2	19.4167	0.333
0.1	0.05	ycaM	2	19.4167	0.28
0.1	0.05	yadI	2	19.4167	0.729
0.1	0.05	AG1	2	19.4167	0.76
0.1	0.05	ptsI	2	19.6667	0.556
0.1	0.05	clcB	2	19.6667	0.332
0.1	0.05	ycaM	2	19.6667	0.281
0.1	0.05	yadI	2	19.6667	0.74
0.1	0.05	AG1	2	19.6667	0.764
0.1	0.05	ptsI	2	19.9167	0.559
0.1	0.05	clcB	2	19.9167	0.329
0.1	0.05	ycaM	2	19.9167	0.283
0.1	0.05	yadI	2	19.9167	0.736
0.1	0.05	AG1	2	19.9167	0.784
0.1	0.05	ptsI	2	20.1667	0.557
0.1	0.05	clcB	2	20.1667	0.334
0.1	0.05	ycaM	2	20.1667	0.286
0.1	0.05	yadI	2	20.1667	0.749
0.1	0.05	AG1	2	20.1667	0.777
0.1	0.05	ptsI	2	20.4167	0.56
0.1	0.05	clcB	2	20.4167	0.333
0.1	0.05	ycaM	2	20.4167	0.287
0.1	0.05	yadI	2	20.4167	0.75
0.1	0.05	AG1	2	20.4167	0.788
0.1	0.05	ptsI	2	20.6667	0.556
0.1	0.05	clcB	2	20.6667	0.334
0.1	0.05	ycaM	2	20.6667	0.291
0.1	0.05	yadI	2	20.6667	0.749
0.1	0.05	AG1	2	20.6667	0.786
0.1	0.05	ptsI	2	20.9167	0.564
0.1	0.05	clcB	2	20.9167	0.332
0.1	0.05	ycaM	2	20.9167	0.294
0.1	0.05	yadI	2	20.9167	0.752
0.1	0.05	AG1	2	20.9167	0.792
0.1	0.05	ptsI	2	21.1667	0.572
0.1	0.05	clcB	2	21.1667	0.332
0.1	0.05	ycaM	2	21.1667	0.297
0.1	0.05	yadI	2	21.1667	0.762
0.1	0.05	AG1	2	21.1667	0.801
0.1	0.05	ptsI	2	21.4167	0.571
0.1	0.05	clcB	2	21.4167	0.33
0.1	0.05	ycaM	2	21.4167	0.297
0.1	0.05	yadI	2	21.4167	0.761
0.1	0.05	AG1	2	21.4167	0.809
0.1	0.05	ptsI	2	21.6667	0.581
0.1	0.05	clcB	2	21.6667	0.333
0.1	0.05	ycaM	2	21.6667	0.299
0.1	0.05	yadI	2	21.6667	0.768
0.1	0.05	AG1	2	21.6667	0.81

0.1	0.05	ptsI	2	21.9167	0.589
0.1	0.05	clcB	2	21.9167	0.333
0.1	0.05	ycaM	2	21.9167	0.299
0.1	0.05	yadI	2	21.9167	0.771
0.1	0.05	AG1	2	21.9167	0.818
0.1	0.05	ptsI	2	22.1667	0.593
0.1	0.05	clcB	2	22.1667	0.333
0.1	0.05	ycaM	2	22.1667	0.302
0.1	0.05	yadI	2	22.1667	0.768
0.1	0.05	AG1	2	22.1667	0.823
0.1	0.05	ptsI	2	22.4167	0.588
0.1	0.05	clcB	2	22.4167	0.332
0.1	0.05	ycaM	2	22.4167	0.306
0.1	0.05	yadI	2	22.4167	0.773
0.1	0.05	AG1	2	22.4167	0.828
0.1	0.05	ptsI	2	22.6667	0.59
0.1	0.05	clcB	2	22.6667	0.332
0.1	0.05	ycaM	2	22.6667	0.306
0.1	0.05	yadI	2	22.6667	0.776
0.1	0.05	AG1	2	22.6667	0.834
0.1	0.05	ptsI	2	22.9167	0.596
0.1	0.05	clcB	2	22.9167	0.333
0.1	0.05	ycaM	2	22.9167	0.31
0.1	0.05	yadI	2	22.9167	0.779
0.1	0.05	AG1	2	22.9167	0.84
0.1	0.05	ptsI	2	23.1667	0.596
0.1	0.05	clcB	2	23.1667	0.333
0.1	0.05	ycaM	2	23.1667	0.308
0.1	0.05	yadI	2	23.1667	0.777
0.1	0.05	AG1	2	23.1667	0.843
0.1	0.05	ptsI	2	23.4167	0.593
0.1	0.05	clcB	2	23.4167	0.334
0.1	0.05	ycaM	2	23.4167	0.31
0.1	0.05	yadI	2	23.4167	0.784
0.1	0.05	AG1	2	23.4167	0.848
0.1	0.05	ptsI	2	23.6667	0.598
0.1	0.05	clcB	2	23.6667	0.335
0.1	0.05	ycaM	2	23.6667	0.312
0.1	0.05	yadI	2	23.6667	0.785
0.1	0.05	AG1	2	23.6667	0.857
0.1	0.05	ptsI	2	23.9167	0.6
0.1	0.05	clcB	2	23.9167	0.335
0.1	0.05	ycaM	2	23.9167	0.314
0.1	0.05	yadI	2	23.9167	0.79
0.1	0.05	AG1	2	23.9167	0.865
0.1	0.05	ptsI	2	24.1667	0.596
0.1	0.05	clcB	2	24.1667	0.338
0.1	0.05	ycaM	2	24.1667	0.317
0.1	0.05	yadI	2	24.1667	0.794
0.1	0.05	AG1	2	24.1667	0.866
0.1	0.05	ptsI	2	24.4167	0.597
0.1	0.05	clcB	2	24.4167	0.34
0.1	0.05	ycaM	2	24.4167	0.321

0.1	0.05	yadI	2	24.4167	0.794
0.1	0.05	AG1	2	24.4167	0.873
0.1	0.05	ptsI	2	24.6667	0.597
0.1	0.05	clcB	2	24.6667	0.341
0.1	0.05	ycaM	2	24.6667	0.324
0.1	0.05	yadI	2	24.6667	0.797
0.1	0.05	AG1	2	24.6667	0.883
0.1	0.05	ptsI	2	24.9167	0.599
0.1	0.05	clcB	2	24.9167	0.342
0.1	0.05	ycaM	2	24.9167	0.323
0.1	0.05	yadI	2	24.9167	0.802
0.1	0.05	AG1	2	24.9167	0.893
0.1	0.05	ptsI	2	25.1667	0.599
0.1	0.05	clcB	2	25.1667	0.344
0.1	0.05	ycaM	2	25.1667	0.331
0.1	0.05	yadI	2	25.1667	0.809
0.1	0.05	AG1	2	25.1667	0.897
0.1	0.05	ptsI	2	25.4167	0.6
0.1	0.05	clcB	2	25.4167	0.347
0.1	0.05	ycaM	2	25.4167	0.336
0.1	0.05	yadI	2	25.4167	0.811
0.1	0.05	AG1	2	25.4167	0.906
0.1	0.05	ptsI	2	25.6667	0.597
0.1	0.05	clcB	2	25.6667	0.345
0.1	0.05	ycaM	2	25.6667	0.336
0.1	0.05	yadI	2	25.6667	0.811
0.1	0.05	AG1	2	25.6667	0.912
0.1	0.05	ptsI	2	25.9167	0.598
0.1	0.05	clcB	2	25.9167	0.346
0.1	0.05	ycaM	2	25.9167	0.345
0.1	0.05	yadI	2	25.9167	0.817
0.1	0.05	AG1	2	25.9167	0.923
0.1	0.05	ptsI	2	26.1667	0.594
0.1	0.05	clcB	2	26.1667	0.345
0.1	0.05	ycaM	2	26.1667	0.344
0.1	0.05	yadI	2	26.1667	0.819
0.1	0.05	AG1	2	26.1667	0.934
0.1	0.05	ptsI	2	26.4167	0.598
0.1	0.05	clcB	2	26.4167	0.347
0.1	0.05	ycaM	2	26.4167	0.353
0.1	0.05	yadI	2	26.4167	0.822
0.1	0.05	AG1	2	26.4167	0.946
0.1	0.05	ptsI	3	0	0.176
0.1	0.05	clcB	3	0	0.161
0.1	0.05	ycaM	3	0	0.162
0.1	0.05	yadI	3	0	0.176
0.1	0.05	AG1	3	0	0.351
0.1	0.05	ptsI	3	0.25	0.172
0.1	0.05	clcB	3	0.25	0.156
0.1	0.05	ycaM	3	0.25	0.159
0.1	0.05	yadI	3	0.25	0.174
0.1	0.05	AG1	3	0.25	0.359
0.1	0.05	ptsI	3	0.5	0.169

0.1	0.05	clcB	3	0.5	0.154
0.1	0.05	ycaM	3	0.5	0.158
0.1	0.05	yadI	3	0.5	0.174
0.1	0.05	AG1	3	0.5	0.358
0.1	0.05	ptsI	3	0.75	0.17
0.1	0.05	clcB	3	0.75	0.154
0.1	0.05	ycaM	3	0.75	0.158
0.1	0.05	yadI	3	0.75	0.173
0.1	0.05	AG1	3	0.75	0.361
0.1	0.05	ptsI	3	1	0.17
0.1	0.05	clcB	3	1	0.153
0.1	0.05	ycaM	3	1	0.157
0.1	0.05	yadI	3	1	0.174
0.1	0.05	AG1	3	1	0.368
0.1	0.05	ptsI	3	1.25	0.17
0.1	0.05	clcB	3	1.25	0.154
0.1	0.05	ycaM	3	1.25	0.159
0.1	0.05	yadI	3	1.25	0.174
0.1	0.05	AG1	3	1.25	0.366
0.1	0.05	ptsI	3	1.5	0.17
0.1	0.05	clcB	3	1.5	0.153
0.1	0.05	ycaM	3	1.5	0.158
0.1	0.05	yadI	3	1.5	0.176
0.1	0.05	AG1	3	1.5	0.359
0.1	0.05	ptsI	3	1.75	0.172
0.1	0.05	clcB	3	1.75	0.154
0.1	0.05	ycaM	3	1.75	0.158
0.1	0.05	yadI	3	1.75	0.19
0.1	0.05	AG1	3	1.75	0.364
0.1	0.05	ptsI	3	2	0.174
0.1	0.05	clcB	3	2	0.16
0.1	0.05	ycaM	3	2	0.161
0.1	0.05	yadI	3	2	0.176
0.1	0.05	AG1	3	2	0.356
0.1	0.05	ptsI	3	2.25	0.174
0.1	0.05	clcB	3	2.25	0.155
0.1	0.05	ycaM	3	2.25	0.161
0.1	0.05	yadI	3	2.25	0.178
0.1	0.05	AG1	3	2.25	0.357
0.1	0.05	ptsI	3	2.5	0.177
0.1	0.05	clcB	3	2.5	0.156
0.1	0.05	ycaM	3	2.5	0.162
0.1	0.05	yadI	3	2.5	0.178
0.1	0.05	AG1	3	2.5	0.347
0.1	0.05	ptsI	3	2.75	0.178
0.1	0.05	clcB	3	2.75	0.158
0.1	0.05	ycaM	3	2.75	0.165
0.1	0.05	yadI	3	2.75	0.179
0.1	0.05	AG1	3	2.75	0.329
0.1	0.05	ptsI	3	3	0.18
0.1	0.05	clcB	3	3	0.159
0.1	0.05	ycaM	3	3	0.166
0.1	0.05	yadI	3	3	0.183

0.1	0.05	AG1	3	3	0.297
0.1	0.05	ptsI	3	3.25	0.182
0.1	0.05	clcB	3	3.25	0.16
0.1	0.05	ycaM	3	3.25	0.167
0.1	0.05	yadI	3	3.25	0.186
0.1	0.05	AG1	3	3.25	0.269
0.1	0.05	ptsI	3	3.5	0.184
0.1	0.05	clcB	3	3.5	0.161
0.1	0.05	ycaM	3	3.5	0.172
0.1	0.05	yadI	3	3.5	0.189
0.1	0.05	AG1	3	3.5	0.249
0.1	0.05	ptsI	3	3.75	0.186
0.1	0.05	clcB	3	3.75	0.164
0.1	0.05	ycaM	3	3.75	0.174
0.1	0.05	yadI	3	3.75	0.191
0.1	0.05	AG1	3	3.75	0.236
0.1	0.05	ptsI	3	4	0.189
0.1	0.05	clcB	3	4	0.166
0.1	0.05	ycaM	3	4	0.176
0.1	0.05	yadI	3	4	0.197
0.1	0.05	AG1	3	4	0.227
0.1	0.05	ptsI	3	4.41667	0.195
0.1	0.05	clcB	3	4.41667	0.192
0.1	0.05	ycaM	3	4.41667	0.193
0.1	0.05	yadI	3	4.41667	0.216
0.1	0.05	AG1	3	4.41667	0.204
0.1	0.05	ptsI	3	4.66667	0.185
0.1	0.05	clcB	3	4.66667	0.186
0.1	0.05	ycaM	3	4.66667	0.193
0.1	0.05	yadI	3	4.66667	0.22
0.1	0.05	AG1	3	4.66667	0.191
0.1	0.05	ptsI	3	4.91667	0.188
0.1	0.05	clcB	3	4.91667	0.188
0.1	0.05	ycaM	3	4.91667	0.193
0.1	0.05	yadI	3	4.91667	0.232
0.1	0.05	AG1	3	4.91667	0.199
0.1	0.05	ptsI	3	5.16667	0.191
0.1	0.05	clcB	3	5.16667	0.193
0.1	0.05	ycaM	3	5.16667	0.198
0.1	0.05	yadI	3	5.16667	0.246
0.1	0.05	AG1	3	5.16667	0.198
0.1	0.05	ptsI	3	5.41667	0.195
0.1	0.05	clcB	3	5.41667	0.198
0.1	0.05	ycaM	3	5.41667	0.204
0.1	0.05	yadI	3	5.41667	0.264
0.1	0.05	AG1	3	5.41667	0.198
0.1	0.05	ptsI	3	5.66667	0.2
0.1	0.05	clcB	3	5.66667	0.205
0.1	0.05	ycaM	3	5.66667	0.212
0.1	0.05	yadI	3	5.66667	0.284
0.1	0.05	AG1	3	5.66667	0.199
0.1	0.05	ptsI	3	5.91667	0.205
0.1	0.05	clcB	3	5.91667	0.213

0.1	0.05	ycaM	3	5.91667	0.218
0.1	0.05	yadI	3	5.91667	0.286
0.1	0.05	AG1	3	5.91667	0.204
0.1	0.05	ptsI	3	6.16667	0.212
0.1	0.05	clcB	3	6.16667	0.22
0.1	0.05	ycaM	3	6.16667	0.228
0.1	0.05	yadI	3	6.16667	0.316
0.1	0.05	AG1	3	6.16667	0.211
0.1	0.05	ptsI	3	6.41667	0.217
0.1	0.05	clcB	3	6.41667	0.229
0.1	0.05	ycaM	3	6.41667	0.24
0.1	0.05	yadI	3	6.41667	0.307
0.1	0.05	AG1	3	6.41667	0.219
0.1	0.05	ptsI	3	6.66667	0.226
0.1	0.05	clcB	3	6.66667	0.239
0.1	0.05	ycaM	3	6.66667	0.249
0.1	0.05	yadI	3	6.66667	0.317
0.1	0.05	AG1	3	6.66667	0.23
0.1	0.05	ptsI	3	6.91667	0.237
0.1	0.05	clcB	3	6.91667	0.25
0.1	0.05	ycaM	3	6.91667	0.259
0.1	0.05	yadI	3	6.91667	0.33
0.1	0.05	AG1	3	6.91667	0.239
0.1	0.05	ptsI	3	7.16667	0.246
0.1	0.05	clcB	3	7.16667	0.26
0.1	0.05	ycaM	3	7.16667	0.265
0.1	0.05	yadI	3	7.16667	0.342
0.1	0.05	AG1	3	7.16667	0.251
0.1	0.05	ptsI	3	7.41667	0.259
0.1	0.05	clcB	3	7.41667	0.267
0.1	0.05	ycaM	3	7.41667	0.269
0.1	0.05	yadI	3	7.41667	0.358
0.1	0.05	AG1	3	7.41667	0.258
0.1	0.05	ptsI	3	7.66667	0.269
0.1	0.05	clcB	3	7.66667	0.276
0.1	0.05	ycaM	3	7.66667	0.274
0.1	0.05	yadI	3	7.66667	0.358
0.1	0.05	AG1	3	7.66667	0.266
0.1	0.05	ptsI	3	7.91667	0.276
0.1	0.05	clcB	3	7.91667	0.281
0.1	0.05	ycaM	3	7.91667	0.275
0.1	0.05	yadI	3	7.91667	0.366
0.1	0.05	AG1	3	7.91667	0.273
0.1	0.05	ptsI	3	8.16667	0.284
0.1	0.05	clcB	3	8.16667	0.286
0.1	0.05	ycaM	3	8.16667	0.274
0.1	0.05	yadI	3	8.16667	0.379
0.1	0.05	AG1	3	8.16667	0.28
0.1	0.05	ptsI	3	8.41667	0.294
0.1	0.05	clcB	3	8.41667	0.294
0.1	0.05	ycaM	3	8.41667	0.275
0.1	0.05	yadI	3	8.41667	0.388
0.1	0.05	AG1	3	8.41667	0.293

0.1	0.05	ptsI	3	9.05	0.283
0.1	0.05	clcB	3	9.05	0.28
0.1	0.05	ycaM	3	9.05	0.292
0.1	0.05	yadI	3	9.05	0.412
0.1	0.05	AG1	3	9.05	0.299
0.1	0.05	ptsI	3	9.3	0.288
0.1	0.05	clcB	3	9.3	0.282
0.1	0.05	ycaM	3	9.3	0.287
0.1	0.05	yadI	3	9.3	0.421
0.1	0.05	AG1	3	9.3	0.313
0.1	0.05	ptsI	3	9.55	0.294
0.1	0.05	clcB	3	9.55	0.286
0.1	0.05	ycaM	3	9.55	0.286
0.1	0.05	yadI	3	9.55	0.435
0.1	0.05	AG1	3	9.55	0.325
0.1	0.05	ptsI	3	9.8	0.302
0.1	0.05	clcB	3	9.8	0.295
0.1	0.05	ycaM	3	9.8	0.289
0.1	0.05	yadI	3	9.8	0.448
0.1	0.05	AG1	3	9.8	0.331
0.1	0.05	ptsI	3	10.05	0.309
0.1	0.05	clcB	3	10.05	0.298
0.1	0.05	ycaM	3	10.05	0.29
0.1	0.05	yadI	3	10.05	0.461
0.1	0.05	AG1	3	10.05	0.342
0.1	0.05	ptsI	3	10.3	0.32
0.1	0.05	clcB	3	10.3	0.3
0.1	0.05	ycaM	3	10.3	0.293
0.1	0.05	yadI	3	10.3	0.472
0.1	0.05	AG1	3	10.3	0.349
0.1	0.05	ptsI	3	10.55	0.328
0.1	0.05	clcB	3	10.55	0.305
0.1	0.05	ycaM	3	10.55	0.292
0.1	0.05	yadI	3	10.55	0.486
0.1	0.05	AG1	3	10.55	0.356
0.1	0.05	ptsI	3	10.8	0.332
0.1	0.05	clcB	3	10.8	0.306
0.1	0.05	ycaM	3	10.8	0.292
0.1	0.05	yadI	3	10.8	0.495
0.1	0.05	AG1	3	10.8	0.361
0.1	0.05	ptsI	3	11.05	0.347
0.1	0.05	clcB	3	11.05	0.311
0.1	0.05	ycaM	3	11.05	0.295
0.1	0.05	yadI	3	11.05	0.506
0.1	0.05	AG1	3	11.05	0.37
0.1	0.05	ptsI	3	11.3	0.353
0.1	0.05	clcB	3	11.3	0.316
0.1	0.05	ycaM	3	11.3	0.294
0.1	0.05	yadI	3	11.3	0.513
0.1	0.05	AG1	3	11.3	0.376
0.1	0.05	ptsI	3	11.55	0.363
0.1	0.05	clcB	3	11.55	0.312
0.1	0.05	ycaM	3	11.55	0.292

0.1	0.05	yadI	3	11.55	0.525
0.1	0.05	AG1	3	11.55	0.383
0.1	0.05	ptsI	3	11.8	0.373
0.1	0.05	clcB	3	11.8	0.31
0.1	0.05	ycaM	3	11.8	0.293
0.1	0.05	yadI	3	11.8	0.54
0.1	0.05	AG1	3	11.8	0.396
0.1	0.05	ptsI	3	12.05	0.38
0.1	0.05	clcB	3	12.05	0.317
0.1	0.05	ycaM	3	12.05	0.294
0.1	0.05	yadI	3	12.05	0.547
0.1	0.05	AG1	3	12.05	0.397
0.1	0.05	ptsI	3	12.3	0.394
0.1	0.05	clcB	3	12.3	0.314
0.1	0.05	ycaM	3	12.3	0.291
0.1	0.05	yadI	3	12.3	0.558
0.1	0.05	AG1	3	12.3	0.406
0.1	0.05	ptsI	3	12.55	0.403
0.1	0.05	clcB	3	12.55	0.307
0.1	0.05	ycaM	3	12.55	0.294
0.1	0.05	yadI	3	12.55	0.569
0.1	0.05	AG1	3	12.55	0.42
0.1	0.05	ptsI	3	12.8	0.414
0.1	0.05	clcB	3	12.8	0.305
0.1	0.05	ycaM	3	12.8	0.29
0.1	0.05	yadI	3	12.8	0.582
0.1	0.05	AG1	3	12.8	0.43
0.1	0.05	ptsI	3	13.05	0.418
0.1	0.05	clcB	3	13.05	0.302
0.1	0.05	ycaM	3	13.05	0.289
0.1	0.05	yadI	3	13.05	0.586
0.1	0.05	AG1	3	13.05	0.434
0.1	0.05	ptsI	3	13.3	0.427
0.1	0.05	clcB	3	13.3	0.317
0.1	0.05	ycaM	3	13.3	0.287
0.1	0.05	yadI	3	13.3	0.597
0.1	0.05	AG1	3	13.3	0.447
0.1	0.05	ptsI	3	13.55	0.44
0.1	0.05	clcB	3	13.55	0.321
0.1	0.05	ycaM	3	13.55	0.288
0.1	0.05	yadI	3	13.55	0.606
0.1	0.05	AG1	3	13.55	0.46
0.1	0.05	ptsI	3	13.8	0.451
0.1	0.05	clcB	3	13.8	0.304
0.1	0.05	ycaM	3	13.8	0.284
0.1	0.05	yadI	3	13.8	0.617
0.1	0.05	AG1	3	13.8	0.474
0.1	0.05	ptsI	3	14.05	0.459
0.1	0.05	clcB	3	14.05	0.31
0.1	0.05	ycaM	3	14.05	0.286
0.1	0.05	yadI	3	14.05	0.625
0.1	0.05	AG1	3	14.05	0.484
0.1	0.05	ptsI	3	14.3	0.464

0.1	0.05	clcB	3	14.3	0.318
0.1	0.05	ycaM	3	14.3	0.284
0.1	0.05	yadI	3	14.3	0.63
0.1	0.05	AG1	3	14.3	0.489
0.1	0.05	ptsI	3	14.55	0.472
0.1	0.05	clcB	3	14.55	0.31
0.1	0.05	ycaM	3	14.55	0.286
0.1	0.05	yadI	3	14.55	0.639
0.1	0.05	AG1	3	14.55	0.501
0.1	0.05	ptsI	3	14.8	0.486
0.1	0.05	clcB	3	14.8	0.304
0.1	0.05	ycaM	3	14.8	0.285
0.1	0.05	yadI	3	14.8	0.652
0.1	0.05	AG1	3	14.8	0.522
0.1	0.05	ptsI	3	15.05	0.497
0.1	0.05	clcB	3	15.05	0.308
0.1	0.05	ycaM	3	15.05	0.286
0.1	0.05	yadI	3	15.05	0.67
0.1	0.05	AG1	3	15.05	0.542
0.1	0.05	ptsI	3	15.3	0.501
0.1	0.05	clcB	3	15.3	0.309
0.1	0.05	ycaM	3	15.3	0.28
0.1	0.05	yadI	3	15.3	0.67
0.1	0.05	AG1	3	15.3	0.542
0.1	0.05	ptsI	3	15.55	0.505
0.1	0.05	clcB	3	15.55	0.307
0.1	0.05	ycaM	3	15.55	0.284
0.1	0.05	yadI	3	15.55	0.675
0.1	0.05	AG1	3	15.55	0.551
0.1	0.05	ptsI	3	15.8	0.511
0.1	0.05	clcB	3	15.8	0.305
0.1	0.05	ycaM	3	15.8	0.279
0.1	0.05	yadI	3	15.8	0.685
0.1	0.05	AG1	3	15.8	0.564
0.1	0.05	ptsI	3	16.05	0.514
0.1	0.05	clcB	3	16.05	0.305
0.1	0.05	ycaM	3	16.05	0.278
0.1	0.05	yadI	3	16.05	0.681
0.1	0.05	AG1	3	16.05	0.567
0.1	0.05	ptsI	3	16.3	0.521
0.1	0.05	clcB	3	16.3	0.308
0.1	0.05	ycaM	3	16.3	0.275
0.1	0.05	yadI	3	16.3	0.689
0.1	0.05	AG1	3	16.3	0.591
0.1	0.05	ptsI	3	16.55	0.527
0.1	0.05	clcB	3	16.55	0.305
0.1	0.05	ycaM	3	16.55	0.278
0.1	0.05	yadI	3	16.55	0.703
0.1	0.05	AG1	3	16.55	0.598
0.1	0.05	ptsI	3	16.8	0.536
0.1	0.05	clcB	3	16.8	0.306
0.1	0.05	ycaM	3	16.8	0.278
0.1	0.05	yadI	3	16.8	0.71

0.1	0.05	AG1	3	16.8	0.62
0.1	0.05	ptsI	3	17.05	0.536
0.1	0.05	clcB	3	17.05	0.305
0.1	0.05	ycaM	3	17.05	0.278
0.1	0.05	yadI	3	17.05	0.714
0.1	0.05	AG1	3	17.05	0.633
0.1	0.05	ptsI	3	17.3	0.542
0.1	0.05	clcB	3	17.3	0.307
0.1	0.05	ycaM	3	17.3	0.277
0.1	0.05	yadI	3	17.3	0.715
0.1	0.05	AG1	3	17.3	0.64
0.1	0.05	ptsI	3	17.55	0.547
0.1	0.05	clcB	3	17.55	0.305
0.1	0.05	ycaM	3	17.55	0.276
0.1	0.05	yadI	3	17.55	0.718
0.1	0.05	AG1	3	17.55	0.653
0.1	0.05	ptsI	3	17.8	0.552
0.1	0.05	clcB	3	17.8	0.306
0.1	0.05	ycaM	3	17.8	0.276
0.1	0.05	yadI	3	17.8	0.729
0.1	0.05	AG1	3	17.8	0.678
0.1	0.05	ptsI	3	18.05	0.55
0.1	0.05	clcB	3	18.05	0.305
0.1	0.05	ycaM	3	18.05	0.278
0.1	0.05	yadI	3	18.05	0.736
0.1	0.05	AG1	3	18.05	0.684
0.1	0.05	ptsI	3	18.3	0.554
0.1	0.05	clcB	3	18.3	0.305
0.1	0.05	ycaM	3	18.3	0.278
0.1	0.05	yadI	3	18.3	0.742
0.1	0.05	AG1	3	18.3	0.689
0.1	0.05	ptsI	3	18.55	0.558
0.1	0.05	clcB	3	18.55	0.305
0.1	0.05	ycaM	3	18.55	0.281
0.1	0.05	yadI	3	18.55	0.748
0.1	0.05	AG1	3	18.55	0.678
0.1	0.05	ptsI	3	18.8	0.561
0.1	0.05	clcB	3	18.8	0.302
0.1	0.05	ycaM	3	18.8	0.281
0.1	0.05	yadI	3	18.8	0.754
0.1	0.05	AG1	3	18.8	0.673
0.1	0.05	ptsI	3	19.05	0.569
0.1	0.05	clcB	3	19.05	0.302
0.1	0.05	ycaM	3	19.05	0.28
0.1	0.05	yadI	3	19.05	0.76
0.1	0.05	AG1	3	19.05	0.677
0.1	0.05	ptsI	3	19.3	0.57
0.1	0.05	clcB	3	19.3	0.304
0.1	0.05	ycaM	3	19.3	0.281
0.1	0.05	yadI	3	19.3	0.762
0.1	0.05	AG1	3	19.3	0.683
0.1	0.05	ptsI	3	19.55	0.565
0.1	0.05	clcB	3	19.55	0.3

0.1	0.05	ycaM	3	19.55	0.281
0.1	0.05	yadI	3	19.55	0.764
0.1	0.05	AG1	3	19.55	0.677
0.1	0.05	ptsI	3	19.8	0.568
0.1	0.05	clcB	3	19.8	0.302
0.1	0.05	ycaM	3	19.8	0.283
0.1	0.05	yadI	3	19.8	0.765
0.1	0.05	AG1	3	19.8	0.68
0.1	0.05	ptsI	3	20.05	0.572
0.1	0.05	clcB	3	20.05	0.301
0.1	0.05	ycaM	3	20.05	0.284
0.1	0.05	yadI	3	20.05	0.774
0.1	0.05	AG1	3	20.05	0.675
0.1	0.05	ptsI	3	20.3	0.573
0.1	0.05	clcB	3	20.3	0.302
0.1	0.05	ycaM	3	20.3	0.284
0.1	0.05	yadI	3	20.3	0.77
0.1	0.05	AG1	3	20.3	0.667
0.1	0.05	ptsI	3	20.55	0.573
0.1	0.05	clcB	3	20.55	0.304
0.1	0.05	ycaM	3	20.55	0.285
0.1	0.05	yadI	3	20.55	0.774
0.1	0.05	AG1	3	20.55	0.68
0.1	0.05	ptsI	3	20.8	0.582
0.1	0.05	clcB	3	20.8	0.303
0.1	0.05	ycaM	3	20.8	0.286
0.1	0.05	yadI	3	20.8	0.783
0.1	0.05	AG1	3	20.8	0.686
0.1	0.05	ptsI	3	21.05	0.578
0.1	0.05	clcB	3	21.05	0.302
0.1	0.05	ycaM	3	21.05	0.29
0.1	0.05	yadI	3	21.05	0.779
0.1	0.05	AG1	3	21.05	0.69
0.1	0.05	ptsI	3	21.3	0.581
0.1	0.05	clcB	3	21.3	0.302
0.1	0.05	ycaM	3	21.3	0.291
0.1	0.05	yadI	3	21.3	0.778
0.1	0.05	AG1	3	21.3	0.696
0.1	0.05	ptsI	3	21.55	0.582
0.1	0.05	clcB	3	21.55	0.304
0.1	0.05	ycaM	3	21.55	0.295
0.1	0.05	yadI	3	21.55	0.787
0.1	0.05	AG1	3	21.55	0.709
0.1	0.05	ptsI	3	21.8	0.585
0.1	0.05	clcB	3	21.8	0.305
0.1	0.05	ycaM	3	21.8	0.299
0.1	0.05	yadI	3	21.8	0.787
0.1	0.05	AG1	3	21.8	0.714
0.1	0.05	ptsI	3	22.05	0.588
0.1	0.05	clcB	3	22.05	0.304
0.1	0.05	ycaM	3	22.05	0.3
0.1	0.05	yadI	3	22.05	0.792
0.1	0.05	AG1	3	22.05	0.722

0.1	0.05	ptsI	3	22.3	0.591
0.1	0.05	clcB	3	22.3	0.307
0.1	0.05	ycaM	3	22.3	0.306
0.1	0.05	yadI	3	22.3	0.792
0.1	0.05	AG1	3	22.3	0.729
0.1	0.05	ptsI	3	22.55	0.59
0.1	0.05	clcB	3	22.55	0.309
0.1	0.05	ycaM	3	22.55	0.311
0.1	0.05	yadI	3	22.55	0.797
0.1	0.05	AG1	3	22.55	0.738
0.1	0.05	ptsI	3	22.8	0.59
0.1	0.05	clcB	3	22.8	0.308
0.1	0.05	ycaM	3	22.8	0.315
0.1	0.05	yadI	3	22.8	0.796
0.1	0.05	AG1	3	22.8	0.741
0.1	0.05	ptsI	3	23.05	0.59
0.1	0.05	clcB	3	23.05	0.31
0.1	0.05	ycaM	3	23.05	0.321
0.1	0.05	yadI	3	23.05	0.801
0.1	0.05	AG1	3	23.05	0.752
0.1	0.05	ptsI	3	23.3	0.594
0.1	0.05	clcB	3	23.3	0.31
0.1	0.05	ycaM	3	23.3	0.327
0.1	0.05	yadI	3	23.3	0.802
0.1	0.05	AG1	3	23.3	0.757
0.1	0.05	ptsI	3	23.55	0.594
0.1	0.05	clcB	3	23.55	0.309
0.1	0.05	ycaM	3	23.55	0.33
0.1	0.05	yadI	3	23.55	0.81
0.1	0.05	AG1	3	23.55	0.768
0.1	0.05	ptsI	3	23.8	0.593
0.1	0.05	clcB	3	23.8	0.311
0.1	0.05	ycaM	3	23.8	0.336
0.1	0.05	yadI	3	23.8	0.807
0.1	0.05	AG1	3	23.8	0.773
0.1	0.05	ptsI	3	24.05	0.593
0.1	0.05	clcB	3	24.05	0.311
0.1	0.05	ycaM	3	24.05	0.34
0.1	0.05	yadI	3	24.05	0.814
0.1	0.05	AG1	3	24.05	0.779
0.1	0.05	ptsI	3	24.3	0.593
0.1	0.05	clcB	3	24.3	0.312
0.1	0.05	ycaM	3	24.3	0.345
0.1	0.05	yadI	3	24.3	0.813
0.1	0.05	AG1	3	24.3	0.791
0.1	0.05	ptsI	4	0	0.192
0.1	0.05	clcB	4	0	0.212
0.1	0.05	ycaM	4	0	0.224
0.1	0.05	yadI	4	0	0.232
0.1	0.05	AG1	4	0	0.198
0.1	0.05	ptsI	4	0.25	0.191
0.1	0.05	clcB	4	0.25	0.208
0.1	0.05	ycaM	4	0.25	0.218

0.1	0.05	yadI	4	0.25	0.222
0.1	0.05	AG1	4	0.25	0.196
0.1	0.05	ptsI	4	0.5	0.187
0.1	0.05	clcB	4	0.5	0.208
0.1	0.05	ycaM	4	0.5	0.218
0.1	0.05	yadI	4	0.5	0.225
0.1	0.05	AG1	4	0.5	0.198
0.1	0.05	ptsI	4	0.75	0.19
0.1	0.05	clcB	4	0.75	0.214
0.1	0.05	ycaM	4	0.75	0.22
0.1	0.05	yadI	4	0.75	0.228
0.1	0.05	AG1	4	0.75	0.199
0.1	0.05	ptsI	4	1	0.193
0.1	0.05	clcB	4	1	0.217
0.1	0.05	ycaM	4	1	0.222
0.1	0.05	yadI	4	1	0.228
0.1	0.05	AG1	4	1	0.201
0.1	0.05	ptsI	4	1.25	0.197
0.1	0.05	clcB	4	1.25	0.223
0.1	0.05	ycaM	4	1.25	0.228
0.1	0.05	yadI	4	1.25	0.231
0.1	0.05	AG1	4	1.25	0.205
0.1	0.05	ptsI	4	1.5	0.201
0.1	0.05	clcB	4	1.5	0.224
0.1	0.05	ycaM	4	1.5	0.233
0.1	0.05	yadI	4	1.5	0.235
0.1	0.05	AG1	4	1.5	0.212
0.1	0.05	ptsI	4	1.75	0.208
0.1	0.05	clcB	4	1.75	0.229
0.1	0.05	ycaM	4	1.75	0.237
0.1	0.05	yadI	4	1.75	0.242
0.1	0.05	AG1	4	1.75	0.22
0.1	0.05	ptsI	4	2	0.215
0.1	0.05	clcB	4	2	0.24
0.1	0.05	ycaM	4	2	0.248
0.1	0.05	yadI	4	2	0.249
0.1	0.05	AG1	4	2	0.228
0.1	0.05	ptsI	4	2.25	0.224
0.1	0.05	clcB	4	2.25	0.249
0.1	0.05	ycaM	4	2.25	0.253
0.1	0.05	yadI	4	2.25	0.258
0.1	0.05	AG1	4	2.25	0.239
0.1	0.05	ptsI	4	2.5	0.229
0.1	0.05	clcB	4	2.5	0.254
0.1	0.05	ycaM	4	2.5	0.263
0.1	0.05	yadI	4	2.5	0.265
0.1	0.05	AG1	4	2.5	0.247
0.1	0.05	ptsI	4	2.75	0.24
0.1	0.05	clcB	4	2.75	0.26
0.1	0.05	ycaM	4	2.75	0.272
0.1	0.05	yadI	4	2.75	0.269
0.1	0.05	AG1	4	2.75	0.256
0.1	0.05	ptsI	4	3	0.247

0.1	0.05	clcB	4	3	0.268
0.1	0.05	ycaM	4	3	0.276
0.1	0.05	yadI	4	3	0.282
0.1	0.05	AG1	4	3	0.27
0.1	0.05	ptsI	4	3.25	0.258
0.1	0.05	clcB	4	3.25	0.278
0.1	0.05	ycaM	4	3.25	0.288
0.1	0.05	yadI	4	3.25	0.293
0.1	0.05	AG1	4	3.25	0.28
0.1	0.05	ptsI	4	3.5	0.266
0.1	0.05	clcB	4	3.5	0.293
0.1	0.05	ycaM	4	3.5	0.298
0.1	0.05	yadI	4	3.5	0.302
0.1	0.05	AG1	4	3.5	0.296
0.1	0.05	ptsI	4	3.75	0.275
0.1	0.05	clcB	4	3.75	0.298
0.1	0.05	ycaM	4	3.75	0.31
0.1	0.05	yadI	4	3.75	0.308
0.1	0.05	AG1	4	3.75	0.304
0.1	0.05	ptsI	4	4	0.292
0.1	0.05	clcB	4	4	0.312
0.1	0.05	ycaM	4	4	0.322
0.1	0.05	yadI	4	4	0.327
0.1	0.05	AG1	4	4	0.318
0.1	0.05	ptsI	4	4.35	0.307
0.1	0.05	clcB	4	4.35	0.312
0.1	0.05	ycaM	4	4.35	0.408
0.1	0.05	yadI	4	4.35	0.308
0.1	0.05	AG1	4	4.35	0.318
0.1	0.05	ptsI	4	4.6	0.289
0.1	0.05	clcB	4	4.6	0.297
0.1	0.05	ycaM	4	4.6	0.371
0.1	0.05	yadI	4	4.6	0.32
0.1	0.05	AG1	4	4.6	0.334
0.1	0.05	ptsI	4	4.85	0.297
0.1	0.05	clcB	4	4.85	0.304
0.1	0.05	ycaM	4	4.85	0.37
0.1	0.05	yadI	4	4.85	0.332
0.1	0.05	AG1	4	4.85	0.344
0.1	0.05	ptsI	4	5.1	0.305
0.1	0.05	clcB	4	5.1	0.308
0.1	0.05	ycaM	4	5.1	0.37
0.1	0.05	yadI	4	5.1	0.346
0.1	0.05	AG1	4	5.1	0.355
0.1	0.05	ptsI	4	5.35	0.315
0.1	0.05	clcB	4	5.35	0.309
0.1	0.05	ycaM	4	5.35	0.375
0.1	0.05	yadI	4	5.35	0.359
0.1	0.05	AG1	4	5.35	0.372
0.1	0.05	ptsI	4	5.6	0.325
0.1	0.05	clcB	4	5.6	0.308
0.1	0.05	ycaM	4	5.6	0.376
0.1	0.05	yadI	4	5.6	0.37

0.1	0.05	AG1	4	5.6	0.383
0.1	0.05	ptsI	4	5.85	0.335
0.1	0.05	clcB	4	5.85	0.31
0.1	0.05	ycaM	4	5.85	0.376
0.1	0.05	yadI	4	5.85	0.381
0.1	0.05	AG1	4	5.85	0.392
0.1	0.05	ptsI	4	6.1	0.349
0.1	0.05	clcB	4	6.1	0.315
0.1	0.05	ycaM	4	6.1	0.377
0.1	0.05	yadI	4	6.1	0.394
0.1	0.05	AG1	4	6.1	0.408
0.1	0.05	ptsI	4	6.35	0.365
0.1	0.05	clcB	4	6.35	0.32
0.1	0.05	ycaM	4	6.35	0.38
0.1	0.05	yadI	4	6.35	0.406
0.1	0.05	AG1	4	6.35	0.417
0.1	0.05	ptsI	4	6.6	0.376
0.1	0.05	clcB	4	6.6	0.319
0.1	0.05	ycaM	4	6.6	0.376
0.1	0.05	yadI	4	6.6	0.41
0.1	0.05	AG1	4	6.6	0.428
0.1	0.05	ptsI	4	6.85	0.388
0.1	0.05	clcB	4	6.85	0.322
0.1	0.05	ycaM	4	6.85	0.38
0.1	0.05	yadI	4	6.85	0.418
0.1	0.05	AG1	4	6.85	0.44
0.1	0.05	ptsI	4	7.1	0.397
0.1	0.05	clcB	4	7.1	0.322
0.1	0.05	ycaM	4	7.1	0.375
0.1	0.05	yadI	4	7.1	0.423
0.1	0.05	AG1	4	7.1	0.452
0.1	0.05	ptsI	4	7.35	0.406
0.1	0.05	clcB	4	7.35	0.326
0.1	0.05	ycaM	4	7.35	0.392
0.1	0.05	yadI	4	7.35	0.43
0.1	0.05	AG1	4	7.35	0.464
0.1	0.05	ptsI	4	7.6	0.413
0.1	0.05	clcB	4	7.6	0.326
0.1	0.05	ycaM	4	7.6	0.392
0.1	0.05	yadI	4	7.6	0.436
0.1	0.05	AG1	4	7.6	0.472
0.1	0.05	ptsI	4	7.85	0.418
0.1	0.05	clcB	4	7.85	0.327
0.1	0.05	ycaM	4	7.85	0.369
0.1	0.05	yadI	4	7.85	0.441
0.1	0.05	AG1	4	7.85	0.478
0.1	0.05	ptsI	4	8.1	0.43
0.1	0.05	clcB	4	8.1	0.326
0.1	0.05	ycaM	4	8.1	0.378
0.1	0.05	yadI	4	8.1	0.45
0.1	0.05	AG1	4	8.1	0.49
0.1	0.05	ptsI	4	8.35	0.439
0.1	0.05	clcB	4	8.35	0.332

0.1	0.05	ycaM	4	8.35	0.389
0.1	0.05	yadI	4	8.35	0.455
0.1	0.05	AG1	4	8.35	0.5
0.1	0.05	ptsI	4	8.83333	0.416
0.1	0.05	clcB	4	8.83333	0.293
0.1	0.05	ycaM	4	8.83333	0.286
0.1	0.05	yadI	4	8.83333	0.484
0.1	0.05	AG1	4	8.83333	0.529
0.1	0.05	ptsI	4	9.08333	0.415
0.1	0.05	clcB	4	9.08333	0.288
0.1	0.05	ycaM	4	9.08333	0.283
0.1	0.05	yadI	4	9.08333	0.482
0.1	0.05	AG1	4	9.08333	0.534
0.1	0.05	ptsI	4	9.33333	0.422
0.1	0.05	clcB	4	9.33333	0.285
0.1	0.05	ycaM	4	9.33333	0.279
0.1	0.05	yadI	4	9.33333	0.489
0.1	0.05	AG1	4	9.33333	0.549
0.1	0.05	ptsI	4	9.58333	0.434
0.1	0.05	clcB	4	9.58333	0.287
0.1	0.05	ycaM	4	9.58333	0.28
0.1	0.05	yadI	4	9.58333	0.501
0.1	0.05	AG1	4	9.58333	0.562
0.1	0.05	ptsI	4	9.83333	0.444
0.1	0.05	clcB	4	9.83333	0.286
0.1	0.05	ycaM	4	9.83333	0.28
0.1	0.05	yadI	4	9.83333	0.508
0.1	0.05	AG1	4	9.83333	0.571
0.1	0.05	ptsI	4	10.0833	0.451
0.1	0.05	clcB	4	10.0833	0.286
0.1	0.05	ycaM	4	10.0833	0.282
0.1	0.05	yadI	4	10.0833	0.514
0.1	0.05	AG1	4	10.0833	0.581
0.1	0.05	ptsI	4	10.3333	0.461
0.1	0.05	clcB	4	10.3333	0.285
0.1	0.05	ycaM	4	10.3333	0.28
0.1	0.05	yadI	4	10.3333	0.522
0.1	0.05	AG1	4	10.3333	0.592
0.1	0.05	ptsI	4	10.5833	0.471
0.1	0.05	clcB	4	10.5833	0.286
0.1	0.05	ycaM	4	10.5833	0.284
0.1	0.05	yadI	4	10.5833	0.532
0.1	0.05	AG1	4	10.5833	0.598
0.1	0.05	ptsI	4	10.8333	0.48
0.1	0.05	clcB	4	10.8333	0.288
0.1	0.05	ycaM	4	10.8333	0.282
0.1	0.05	yadI	4	10.8333	0.535
0.1	0.05	AG1	4	10.8333	0.607
0.1	0.05	ptsI	4	11.0833	0.488
0.1	0.05	clcB	4	11.0833	0.279
0.1	0.05	ycaM	4	11.0833	0.282
0.1	0.05	yadI	4	11.0833	0.541
0.1	0.05	AG1	4	11.0833	0.613

0.1	0.05	ptsI	4	11.3333	0.499
0.1	0.05	clcB	4	11.3333	0.292
0.1	0.05	ycaM	4	11.3333	0.287
0.1	0.05	yadI	4	11.3333	0.547
0.1	0.05	AG1	4	11.3333	0.619
0.1	0.05	ptsI	4	11.5833	0.504
0.1	0.05	clcB	4	11.5833	0.285
0.1	0.05	ycaM	4	11.5833	0.286
0.1	0.05	yadI	4	11.5833	0.555
0.1	0.05	AG1	4	11.5833	0.628
0.1	0.05	ptsI	4	11.8333	0.51
0.1	0.05	clcB	4	11.8333	0.29
0.1	0.05	ycaM	4	11.8333	0.29
0.1	0.05	yadI	4	11.8333	0.558
0.1	0.05	AG1	4	11.8333	0.632
0.1	0.05	ptsI	4	12.0833	0.519
0.1	0.05	clcB	4	12.0833	0.291
0.1	0.05	ycaM	4	12.0833	0.29
0.1	0.05	yadI	4	12.0833	0.564
0.1	0.05	AG1	4	12.0833	0.637
0.1	0.05	ptsI	4	12.3333	0.528
0.1	0.05	clcB	4	12.3333	0.292
0.1	0.05	ycaM	4	12.3333	0.289
0.1	0.05	yadI	4	12.3333	0.569
0.1	0.05	AG1	4	12.3333	0.646
0.1	0.05	ptsI	4	12.5833	0.535
0.1	0.05	clcB	4	12.5833	0.3
0.1	0.05	ycaM	4	12.5833	0.289
0.1	0.05	yadI	4	12.5833	0.574
0.1	0.05	AG1	4	12.5833	0.653
0.1	0.05	ptsI	4	12.8333	0.54
0.1	0.05	clcB	4	12.8333	0.299
0.1	0.05	ycaM	4	12.8333	0.289
0.1	0.05	yadI	4	12.8333	0.58
0.1	0.05	AG1	4	12.8333	0.656
0.1	0.05	ptsI	4	13.0833	0.545
0.1	0.05	clcB	4	13.0833	0.299
0.1	0.05	ycaM	4	13.0833	0.291
0.1	0.05	yadI	4	13.0833	0.586
0.1	0.05	AG1	4	13.0833	0.663
0.1	0.05	ptsI	4	13.3333	0.553
0.1	0.05	clcB	4	13.3333	0.304
0.1	0.05	ycaM	4	13.3333	0.291
0.1	0.05	yadI	4	13.3333	0.588
0.1	0.05	AG1	4	13.3333	0.667
0.1	0.05	ptsI	4	13.5833	0.558
0.1	0.05	clcB	4	13.5833	0.301
0.1	0.05	ycaM	4	13.5833	0.302
0.1	0.05	yadI	4	13.5833	0.592
0.1	0.05	AG1	4	13.5833	0.672
0.1	0.05	ptsI	4	13.8333	0.565
0.1	0.05	clcB	4	13.8333	0.304
0.1	0.05	ycaM	4	13.8333	0.292

0.1	0.05	yadI	4	13.8333	0.595
0.1	0.05	AG1	4	13.8333	0.675
0.1	0.05	ptsI	4	14.0833	0.571
0.1	0.05	clcB	4	14.0833	0.303
0.1	0.05	ycaM	4	14.0833	0.304
0.1	0.05	yadI	4	14.0833	0.602
0.1	0.05	AG1	4	14.0833	0.679
0.1	0.05	ptsI	4	14.3333	0.577
0.1	0.05	clcB	4	14.3333	0.305
0.1	0.05	ycaM	4	14.3333	0.304
0.1	0.05	yadI	4	14.3333	0.603
0.1	0.05	AG1	4	14.3333	0.68
0.1	0.05	ptsI	4	14.5833	0.582
0.1	0.05	clcB	4	14.5833	0.307
0.1	0.05	ycaM	4	14.5833	0.288
0.1	0.05	yadI	4	14.5833	0.609
0.1	0.05	AG1	4	14.5833	0.686
0.1	0.05	ptsI	4	14.8333	0.588
0.1	0.05	clcB	4	14.8333	0.31
0.1	0.05	ycaM	4	14.8333	0.322
0.1	0.05	yadI	4	14.8333	0.612
0.1	0.05	AG1	4	14.8333	0.693
0.1	0.05	ptsI	4	15.0833	0.59
0.1	0.05	clcB	4	15.0833	0.308
0.1	0.05	ycaM	4	15.0833	0.319
0.1	0.05	yadI	4	15.0833	0.617
0.1	0.05	AG1	4	15.0833	0.691
0.1	0.05	ptsI	4	15.3333	0.596
0.1	0.05	clcB	4	15.3333	0.31
0.1	0.05	ycaM	4	15.3333	0.324
0.1	0.05	yadI	4	15.3333	0.616
0.1	0.05	AG1	4	15.3333	0.696
0.1	0.05	ptsI	4	15.5833	0.602
0.1	0.05	clcB	4	15.5833	0.314
0.1	0.05	ycaM	4	15.5833	0.326
0.1	0.05	yadI	4	15.5833	0.623
0.1	0.05	AG1	4	15.5833	0.698
0.1	0.05	ptsI	4	15.8333	0.606
0.1	0.05	clcB	4	15.8333	0.313
0.1	0.05	ycaM	4	15.8333	0.326
0.1	0.05	yadI	4	15.8333	0.628
0.1	0.05	AG1	4	15.8333	0.702
0.1	0.05	ptsI	4	16.0833	0.611
0.1	0.05	clcB	4	16.0833	0.316
0.1	0.05	ycaM	4	16.0833	0.328
0.1	0.05	yadI	4	16.0833	0.629
0.1	0.05	AG1	4	16.0833	0.705
0.1	0.05	ptsI	4	16.3333	0.616
0.1	0.05	clcB	4	16.3333	0.316
0.1	0.05	ycaM	4	16.3333	0.331
0.1	0.05	yadI	4	16.3333	0.631
0.1	0.05	AG1	4	16.3333	0.708
0.1	0.05	ptsI	4	16.5833	0.619

0.1	0.05	clcB	4	16.5833	0.319
0.1	0.05	ycaM	4	16.5833	0.331
0.1	0.05	yadI	4	16.5833	0.635
0.1	0.05	AG1	4	16.5833	0.711
0.1	0.05	ptsI	4	16.8333	0.623
0.1	0.05	clcB	4	16.8333	0.323
0.1	0.05	ycaM	4	16.8333	0.335
0.1	0.05	yadI	4	16.8333	0.641
0.1	0.05	AG1	4	16.8333	0.717
0.1	0.05	ptsI	4	17.0833	0.619
0.1	0.05	clcB	4	17.0833	0.325
0.1	0.05	ycaM	4	17.0833	0.339
0.1	0.05	yadI	4	17.0833	0.64
0.1	0.05	AG1	4	17.0833	0.719
0.1	0.05	ptsI	4	17.3333	0.625
0.1	0.05	clcB	4	17.3333	0.338
0.1	0.05	ycaM	4	17.3333	0.341
0.1	0.05	yadI	4	17.3333	0.642
0.1	0.05	AG1	4	17.3333	0.719
0.1	0.05	ptsI	4	17.5833	0.625
0.1	0.05	clcB	4	17.5833	0.339
0.1	0.05	ycaM	4	17.5833	0.344
0.1	0.05	yadI	4	17.5833	0.647
0.1	0.05	AG1	4	17.5833	0.722
0.1	0.05	ptsI	4	17.8333	0.628
0.1	0.05	clcB	4	17.8333	0.339
0.1	0.05	ycaM	4	17.8333	0.352
0.1	0.05	yadI	4	17.8333	0.644
0.1	0.05	AG1	4	17.8333	0.716
0.1	0.05	ptsI	4	18.0833	0.629
0.1	0.05	clcB	4	18.0833	0.339
0.1	0.05	ycaM	4	18.0833	0.351
0.1	0.05	yadI	4	18.0833	0.649
0.1	0.05	AG1	4	18.0833	0.727
0.1	0.05	ptsI	4	18.3333	0.63
0.1	0.05	clcB	4	18.3333	0.331
0.1	0.05	ycaM	4	18.3333	0.354
0.1	0.05	yadI	4	18.3333	0.654
0.1	0.05	AG1	4	18.3333	0.729
0.1	0.05	ptsI	4	18.5833	0.63
0.1	0.05	clcB	4	18.5833	0.33
0.1	0.05	ycaM	4	18.5833	0.361
0.1	0.05	yadI	4	18.5833	0.656
0.1	0.05	AG1	4	18.5833	0.735
0.1	0.05	ptsI	4	18.8333	0.634
0.1	0.05	clcB	4	18.8333	0.322
0.1	0.05	ycaM	4	18.8333	0.37
0.1	0.05	yadI	4	18.8333	0.657
0.1	0.05	AG1	4	18.8333	0.731
0.1	0.05	ptsI	4	19.0833	0.633
0.1	0.05	clcB	4	19.0833	0.331
0.1	0.05	ycaM	4	19.0833	0.366
0.1	0.05	yadI	4	19.0833	0.664

0.1	0.05	AG1	4	19.0833	0.743
0.1	0.05	ptsI	4	19.3333	0.629
0.1	0.05	clcB	4	19.3333	0.318
0.1	0.05	ycaM	4	19.3333	0.371
0.1	0.05	yadI	4	19.3333	0.66
0.1	0.05	AG1	4	19.3333	0.752
0.1	0.05	ptsI	4	19.5833	0.628
0.1	0.05	clcB	4	19.5833	0.334
0.1	0.05	ycaM	4	19.5833	0.373
0.1	0.05	yadI	4	19.5833	0.66
0.1	0.05	AG1	4	19.5833	0.76
0.1	0.05	ptsI	4	19.8333	0.628
0.1	0.05	clcB	4	19.8333	0.323
0.1	0.05	ycaM	4	19.8333	0.378
0.1	0.05	yadI	4	19.8333	0.662
0.1	0.05	AG1	4	19.8333	0.765
0.1	0.05	ptsI	4	20.0833	0.631
0.1	0.05	clcB	4	20.0833	0.327
0.1	0.05	ycaM	4	20.0833	0.387
0.1	0.05	yadI	4	20.0833	0.665
0.1	0.05	AG1	4	20.0833	0.765
0.1	0.05	ptsI	4	20.3333	0.633
0.1	0.05	clcB	4	20.3333	0.32
0.1	0.05	ycaM	4	20.3333	0.387
0.1	0.05	yadI	4	20.3333	0.667
0.1	0.05	AG1	4	20.3333	0.769
0.1	0.05	ptsI	4	20.5833	0.627
0.1	0.05	clcB	4	20.5833	0.326
0.1	0.05	ycaM	4	20.5833	0.393
0.1	0.05	yadI	4	20.5833	0.668
0.1	0.05	AG1	4	20.5833	0.775
0.1	0.05	ptsI	4	20.8333	0.637
0.1	0.05	clcB	4	20.8333	0.327
0.1	0.05	ycaM	4	20.8333	0.397
0.1	0.05	yadI	4	20.8333	0.669
0.1	0.05	AG1	4	20.8333	0.774
0.1	0.05	ptsI	4	21.0833	0.634
0.1	0.05	clcB	4	21.0833	0.327
0.1	0.05	ycaM	4	21.0833	0.396
0.1	0.05	yadI	4	21.0833	0.669
0.1	0.05	AG1	4	21.0833	0.778
0.1	0.05	ptsI	4	21.3333	0.631
0.1	0.05	clcB	4	21.3333	0.326
0.1	0.05	ycaM	4	21.3333	0.399
0.1	0.05	yadI	4	21.3333	0.67
0.1	0.05	AG1	4	21.3333	0.778
0.1	0.05	ptsI	4	21.5833	0.636
0.1	0.05	clcB	4	21.5833	0.33
0.1	0.05	ycaM	4	21.5833	0.401
0.1	0.05	yadI	4	21.5833	0.671
0.1	0.05	AG1	4	21.5833	0.781
0.1	0.05	ptsI	4	21.8333	0.638
0.1	0.05	clcB	4	21.8333	0.325

0.1	0.05	ycaM	4	21.8333	0.401
0.1	0.05	yadI	4	21.8333	0.671
0.1	0.05	AG1	4	21.8333	0.781
0.1	0.05	ptsI	4	22.0833	0.634
0.1	0.05	clcB	4	22.0833	0.325
0.1	0.05	ycaM	4	22.0833	0.403
0.1	0.05	yadI	4	22.0833	0.671
0.1	0.05	AG1	4	22.0833	0.784
0.1	0.05	ptsI	4	22.3333	0.637
0.1	0.05	clcB	4	22.3333	0.331
0.1	0.05	ycaM	4	22.3333	0.397
0.1	0.05	yadI	4	22.3333	0.672
0.1	0.05	AG1	4	22.3333	0.786
0.1	0.05	ptsI	4	22.5833	0.637
0.1	0.05	clcB	4	22.5833	0.327
0.1	0.05	ycaM	4	22.5833	0.399
0.1	0.05	yadI	4	22.5833	0.674
0.1	0.05	AG1	4	22.5833	0.788
0.1	0.05	ptsI	4	22.8333	0.634
0.1	0.05	clcB	4	22.8333	0.326
0.1	0.05	ycaM	4	22.8333	0.4
0.1	0.05	yadI	4	22.8333	0.674
0.1	0.05	AG1	4	22.8333	0.792
0.1	0.05	ptsI	4	23.0833	0.633
0.1	0.05	clcB	4	23.0833	0.334
0.1	0.05	ycaM	4	23.0833	0.401
0.1	0.05	yadI	4	23.0833	0.674
0.1	0.05	AG1	4	23.0833	0.793
0.1	0.05	ptsI	4	23.3333	0.636
0.1	0.05	clcB	4	23.3333	0.33
0.1	0.05	ycaM	4	23.3333	0.406
0.1	0.05	yadI	4	23.3333	0.677
0.1	0.05	AG1	4	23.3333	0.796
0.1	0.05	ptsI	4	23.5833	0.634
0.1	0.05	clcB	4	23.5833	0.328
0.1	0.05	ycaM	4	23.5833	0.407
0.1	0.05	yadI	4	23.5833	0.678
0.1	0.05	AG1	4	23.5833	0.798
0.1	0.05	ptsI	4	23.8333	0.63
0.1	0.05	clcB	4	23.8333	0.336
0.1	0.05	ycaM	4	23.8333	0.408
0.1	0.05	yadI	4	23.8333	0.678
0.1	0.05	AG1	4	23.8333	0.801
0.1	0.05	ptsI	4	24.0833	0.635
0.1	0.05	clcB	4	24.0833	0.338
0.1	0.05	ycaM	4	24.0833	0.41
0.1	0.05	yadI	4	24.0833	0.679
0.1	0.05	AG1	4	24.0833	0.8
0.1	0.05	ptsI	5	0	0.18
0.1	0.05	clcB	5	0	0.172
0.1	0.05	ycaM	5	0	0.176
0.1	0.05	yadI	5	0	0.18
0.1	0.05	AG1	5	0	0.174

0.1	0.05	ptsI	5	0.35	0.176
0.1	0.05	clcB	5	0.35	0.166
0.1	0.05	ycaM	5	0.35	0.167
0.1	0.05	yadI	5	0.35	0.173
0.1	0.05	AG1	5	0.35	0.17
0.1	0.05	ptsI	5	0.6	0.176
0.1	0.05	clcB	5	0.6	0.166
0.1	0.05	ycaM	5	0.6	0.168
0.1	0.05	yadI	5	0.6	0.173
0.1	0.05	AG1	5	0.6	0.171
0.1	0.05	ptsI	5	0.85	0.175
0.1	0.05	clcB	5	0.85	0.166
0.1	0.05	ycaM	5	0.85	0.168
0.1	0.05	yadI	5	0.85	0.176
0.1	0.05	AG1	5	0.85	0.171
0.1	0.05	ptsI	5	1.1	0.178
0.1	0.05	clcB	5	1.1	0.166
0.1	0.05	ycaM	5	1.1	0.17
0.1	0.05	yadI	5	1.1	0.176
0.1	0.05	AG1	5	1.1	0.172
0.1	0.05	ptsI	5	1.35	0.179
0.1	0.05	clcB	5	1.35	0.167
0.1	0.05	ycaM	5	1.35	0.172
0.1	0.05	yadI	5	1.35	0.18
0.1	0.05	AG1	5	1.35	0.175
0.1	0.05	ptsI	5	1.6	0.182
0.1	0.05	clcB	5	1.6	0.168
0.1	0.05	ycaM	5	1.6	0.174
0.1	0.05	yadI	5	1.6	0.18
0.1	0.05	AG1	5	1.6	0.177
0.1	0.05	ptsI	5	1.85	0.185
0.1	0.05	clcB	5	1.85	0.171
0.1	0.05	ycaM	5	1.85	0.178
0.1	0.05	yadI	5	1.85	0.184
0.1	0.05	AG1	5	1.85	0.18
0.1	0.05	ptsI	5	2.1	0.191
0.1	0.05	clcB	5	2.1	0.181
0.1	0.05	ycaM	5	2.1	0.182
0.1	0.05	yadI	5	2.1	0.187
0.1	0.05	AG1	5	2.1	0.185
0.1	0.05	ptsI	5	2.35	0.198
0.1	0.05	clcB	5	2.35	0.188
0.1	0.05	ycaM	5	2.35	0.187
0.1	0.05	yadI	5	2.35	0.192
0.1	0.05	AG1	5	2.35	0.186
0.1	0.05	ptsI	5	2.6	0.194
0.1	0.05	clcB	5	2.6	0.183
0.1	0.05	ycaM	5	2.6	0.19
0.1	0.05	yadI	5	2.6	0.192
0.1	0.05	AG1	5	2.6	0.183
0.1	0.05	ptsI	5	2.85	0.203
0.1	0.05	clcB	5	2.85	0.193
0.1	0.05	ycaM	5	2.85	0.196

0.1	0.05	yadI	5	2.85	0.203
0.1	0.05	AG1	5	2.85	0.194
0.1	0.05	ptsI	5	3.1	0.209
0.1	0.05	clcB	5	3.1	0.2
0.1	0.05	ycaM	5	3.1	0.2
0.1	0.05	yadI	5	3.1	0.211
0.1	0.05	AG1	5	3.1	0.2
0.1	0.05	ptsI	5	3.35	0.22
0.1	0.05	clcB	5	3.35	0.216
0.1	0.05	ycaM	5	3.35	0.209
0.1	0.05	yadI	5	3.35	0.22
0.1	0.05	AG1	5	3.35	0.205
0.1	0.05	ptsI	5	3.6	0.233
0.1	0.05	clcB	5	3.6	0.234
0.1	0.05	ycaM	5	3.6	0.219
0.1	0.05	yadI	5	3.6	0.228
0.1	0.05	AG1	5	3.6	0.214
0.1	0.05	ptsI	5	3.85	0.241
0.1	0.05	clcB	5	3.85	0.248
0.1	0.05	ycaM	5	3.85	0.227
0.1	0.05	yadI	5	3.85	0.239
0.1	0.05	AG1	5	3.85	0.225
0.1	0.05	ptsI	5	4.38333	0.263
0.1	0.05	clcB	5	4.38333	0.286
0.1	0.05	ycaM	5	4.38333	0.246
0.1	0.05	yadI	5	4.38333	0.267
0.1	0.05	AG1	5	4.38333	0.257
0.1	0.05	ptsI	5	4.63333	0.264
0.1	0.05	clcB	5	4.63333	0.292
0.1	0.05	ycaM	5	4.63333	0.257
0.1	0.05	yadI	5	4.63333	0.269
0.1	0.05	AG1	5	4.63333	0.257
0.1	0.05	ptsI	5	4.88333	0.271
0.1	0.05	clcB	5	4.88333	0.311
0.1	0.05	ycaM	5	4.88333	0.27
0.1	0.05	yadI	5	4.88333	0.288
0.1	0.05	AG1	5	4.88333	0.268
0.1	0.05	ptsI	5	5.13333	0.279
0.1	0.05	clcB	5	5.13333	0.324
0.1	0.05	ycaM	5	5.13333	0.273
0.1	0.05	yadI	5	5.13333	0.3
0.1	0.05	AG1	5	5.13333	0.281
0.1	0.05	ptsI	5	5.38333	0.289
0.1	0.05	clcB	5	5.38333	0.336
0.1	0.05	ycaM	5	5.38333	0.275
0.1	0.05	yadI	5	5.38333	0.313
0.1	0.05	AG1	5	5.38333	0.297
0.1	0.05	ptsI	5	5.63333	0.302
0.1	0.05	clcB	5	5.63333	0.348
0.1	0.05	ycaM	5	5.63333	0.282
0.1	0.05	yadI	5	5.63333	0.326
0.1	0.05	AG1	5	5.63333	0.312
0.1	0.05	ptsI	5	5.88333	0.314

0.1	0.05	clcB	5	5.88333	0.355
0.1	0.05	ycaM	5	5.88333	0.286
0.1	0.05	yadI	5	5.88333	0.336
0.1	0.05	AG1	5	5.88333	0.324
0.1	0.05	ptsI	5	6.13333	0.328
0.1	0.05	clcB	5	6.13333	0.364
0.1	0.05	ycaM	5	6.13333	0.286
0.1	0.05	yadI	5	6.13333	0.351
0.1	0.05	AG1	5	6.13333	0.338
0.1	0.05	ptsI	5	6.38333	0.342
0.1	0.05	clcB	5	6.38333	0.368
0.1	0.05	ycaM	5	6.38333	0.289
0.1	0.05	yadI	5	6.38333	0.364
0.1	0.05	AG1	5	6.38333	0.351
0.1	0.05	ptsI	5	6.63333	0.361
0.1	0.05	clcB	5	6.63333	0.373
0.1	0.05	ycaM	5	6.63333	0.29
0.1	0.05	yadI	5	6.63333	0.377
0.1	0.05	AG1	5	6.63333	0.365
0.1	0.05	ptsI	5	6.88333	0.376
0.1	0.05	clcB	5	6.88333	0.381
0.1	0.05	ycaM	5	6.88333	0.291
0.1	0.05	yadI	5	6.88333	0.39
0.1	0.05	AG1	5	6.88333	0.378
0.1	0.05	ptsI	5	7.13333	0.392
0.1	0.05	clcB	5	7.13333	0.384
0.1	0.05	ycaM	5	7.13333	0.294
0.1	0.05	yadI	5	7.13333	0.406
0.1	0.05	AG1	5	7.13333	0.392
0.1	0.05	ptsI	5	7.38333	0.405
0.1	0.05	clcB	5	7.38333	0.385
0.1	0.05	ycaM	5	7.38333	0.297
0.1	0.05	yadI	5	7.38333	0.42
0.1	0.05	AG1	5	7.38333	0.404
0.1	0.05	ptsI	5	7.63333	0.414
0.1	0.05	clcB	5	7.63333	0.388
0.1	0.05	ycaM	5	7.63333	0.298
0.1	0.05	yadI	5	7.63333	0.429
0.1	0.05	AG1	5	7.63333	0.415
0.1	0.05	ptsI	5	7.88333	0.432
0.1	0.05	clcB	5	7.88333	0.393
0.1	0.05	ycaM	5	7.88333	0.297
0.1	0.05	yadI	5	7.88333	0.443
0.1	0.05	AG1	5	7.88333	0.428
0.1	0.05	ptsI	5	8.13333	0.452
0.1	0.05	clcB	5	8.13333	0.392
0.1	0.05	ycaM	5	8.13333	0.301
0.1	0.05	yadI	5	8.13333	0.452
0.1	0.05	AG1	5	8.13333	0.444
0.1	0.05	ptsI	5	8.38333	0.465
0.1	0.05	clcB	5	8.38333	0.397
0.1	0.05	ycaM	5	8.38333	0.299
0.1	0.05	yadI	5	8.38333	0.46

0.1	0.05	AG1	5	8.38333	0.455
0.1	0.05	ptsI	5	8.88333	0.355
0.1	0.05	clcB	5	8.88333	0.296
0.1	0.05	ycaM	5	8.88333	0.266
0.1	0.05	yadI	5	8.88333	0.459
0.1	0.05	AG1	5	8.88333	0.503
0.1	0.05	ptsI	5	9.13333	0.365
0.1	0.05	clcB	5	9.13333	0.295
0.1	0.05	ycaM	5	9.13333	0.265
0.1	0.05	yadI	5	9.13333	0.457
0.1	0.05	AG1	5	9.13333	0.506
0.1	0.05	ptsI	5	9.38333	0.372
0.1	0.05	clcB	5	9.38333	0.298
0.1	0.05	ycaM	5	9.38333	0.263
0.1	0.05	yadI	5	9.38333	0.472
0.1	0.05	AG1	5	9.38333	0.526
0.1	0.05	ptsI	5	9.63333	0.384
0.1	0.05	clcB	5	9.63333	0.301
0.1	0.05	ycaM	5	9.63333	0.266
0.1	0.05	yadI	5	9.63333	0.482
0.1	0.05	AG1	5	9.63333	0.54
0.1	0.05	ptsI	5	9.88333	0.392
0.1	0.05	clcB	5	9.88333	0.301
0.1	0.05	ycaM	5	9.88333	0.266
0.1	0.05	yadI	5	9.88333	0.491
0.1	0.05	AG1	5	9.88333	0.554
0.1	0.05	ptsI	5	10.1333	0.402
0.1	0.05	clcB	5	10.1333	0.304
0.1	0.05	ycaM	5	10.1333	0.264
0.1	0.05	yadI	5	10.1333	0.499
0.1	0.05	AG1	5	10.1333	0.563
0.1	0.05	ptsI	5	10.3833	0.414
0.1	0.05	clcB	5	10.3833	0.305
0.1	0.05	ycaM	5	10.3833	0.27
0.1	0.05	yadI	5	10.3833	0.509
0.1	0.05	AG1	5	10.3833	0.578
0.1	0.05	ptsI	5	10.6333	0.42
0.1	0.05	clcB	5	10.6333	0.304
0.1	0.05	ycaM	5	10.6333	0.268
0.1	0.05	yadI	5	10.6333	0.515
0.1	0.05	AG1	5	10.6333	0.588
0.1	0.05	ptsI	5	10.8833	0.433
0.1	0.05	clcB	5	10.8833	0.309
0.1	0.05	ycaM	5	10.8833	0.269
0.1	0.05	yadI	5	10.8833	0.523
0.1	0.05	AG1	5	10.8833	0.599
0.1	0.05	ptsI	5	11.1333	0.441
0.1	0.05	clcB	5	11.1333	0.306
0.1	0.05	ycaM	5	11.1333	0.267
0.1	0.05	yadI	5	11.1333	0.526
0.1	0.05	AG1	5	11.1333	0.609
0.1	0.05	ptsI	5	11.3833	0.45
0.1	0.05	clcB	5	11.3833	0.306

0.1	0.05	ycaM	5	11.3833	0.268
0.1	0.05	yadI	5	11.3833	0.534
0.1	0.05	AG1	5	11.3833	0.618
0.1	0.05	ptsI	5	11.6333	0.461
0.1	0.05	clcB	5	11.6333	0.305
0.1	0.05	ycaM	5	11.6333	0.268
0.1	0.05	yadI	5	11.6333	0.544
0.1	0.05	AG1	5	11.6333	0.632
0.1	0.05	ptsI	5	11.8833	0.466
0.1	0.05	clcB	5	11.8833	0.304
0.1	0.05	ycaM	5	11.8833	0.268
0.1	0.05	yadI	5	11.8833	0.549
0.1	0.05	AG1	5	11.8833	0.64
0.1	0.05	ptsI	5	12.1333	0.478
0.1	0.05	clcB	5	12.1333	0.303
0.1	0.05	ycaM	5	12.1333	0.271
0.1	0.05	yadI	5	12.1333	0.556
0.1	0.05	AG1	5	12.1333	0.652
0.1	0.05	ptsI	5	12.3833	0.481
0.1	0.05	clcB	5	12.3833	0.298
0.1	0.05	ycaM	5	12.3833	0.268
0.1	0.05	yadI	5	12.3833	0.558
0.1	0.05	AG1	5	12.3833	0.658
0.1	0.05	ptsI	5	12.6333	0.496
0.1	0.05	clcB	5	12.6333	0.309
0.1	0.05	ycaM	5	12.6333	0.271
0.1	0.05	yadI	5	12.6333	0.568
0.1	0.05	AG1	5	12.6333	0.671
0.1	0.05	ptsI	5	12.8833	0.503
0.1	0.05	clcB	5	12.8833	0.316
0.1	0.05	ycaM	5	12.8833	0.273
0.1	0.05	yadI	5	12.8833	0.575
0.1	0.05	AG1	5	12.8833	0.681
0.1	0.05	ptsI	5	13.1333	0.512
0.1	0.05	clcB	5	13.1333	0.317
0.1	0.05	ycaM	5	13.1333	0.275
0.1	0.05	yadI	5	13.1333	0.581
0.1	0.05	AG1	5	13.1333	0.69
0.1	0.05	ptsI	5	13.3833	0.521
0.1	0.05	clcB	5	13.3833	0.312
0.1	0.05	ycaM	5	13.3833	0.273
0.1	0.05	yadI	5	13.3833	0.584
0.1	0.05	AG1	5	13.3833	0.696
0.1	0.05	ptsI	5	13.6333	0.527
0.1	0.05	clcB	5	13.6333	0.316
0.1	0.05	ycaM	5	13.6333	0.275
0.1	0.05	yadI	5	13.6333	0.592
0.1	0.05	AG1	5	13.6333	0.707
0.1	0.05	ptsI	5	13.8833	0.533
0.1	0.05	clcB	5	13.8833	0.318
0.1	0.05	ycaM	5	13.8833	0.278
0.1	0.05	yadI	5	13.8833	0.595
0.1	0.05	AG1	5	13.8833	0.715

0.1	0.05	ptsI	5	14.1333	0.543
0.1	0.05	clcB	5	14.1333	0.319
0.1	0.05	ycaM	5	14.1333	0.28
0.1	0.05	yadI	5	14.1333	0.601
0.1	0.05	AG1	5	14.1333	0.717
0.1	0.05	ptsI	5	14.3833	0.552
0.1	0.05	clcB	5	14.3833	0.317
0.1	0.05	ycaM	5	14.3833	0.278
0.1	0.05	yadI	5	14.3833	0.605
0.1	0.05	AG1	5	14.3833	0.724
0.1	0.05	ptsI	5	14.6333	0.558
0.1	0.05	clcB	5	14.6333	0.32
0.1	0.05	ycaM	5	14.6333	0.279
0.1	0.05	yadI	5	14.6333	0.609
0.1	0.05	AG1	5	14.6333	0.73
0.1	0.05	ptsI	5	14.8833	0.567
0.1	0.05	clcB	5	14.8833	0.316
0.1	0.05	ycaM	5	14.8833	0.28
0.1	0.05	yadI	5	14.8833	0.612
0.1	0.05	AG1	5	14.8833	0.738
0.1	0.05	ptsI	5	15.1333	0.574
0.1	0.05	clcB	5	15.1333	0.317
0.1	0.05	ycaM	5	15.1333	0.282
0.1	0.05	yadI	5	15.1333	0.616
0.1	0.05	AG1	5	15.1333	0.742
0.1	0.05	ptsI	5	15.3833	0.581
0.1	0.05	clcB	5	15.3833	0.318
0.1	0.05	ycaM	5	15.3833	0.281
0.1	0.05	yadI	5	15.3833	0.62
0.1	0.05	AG1	5	15.3833	0.745
0.1	0.05	ptsI	5	15.6333	0.584
0.1	0.05	clcB	5	15.6333	0.313
0.1	0.05	ycaM	5	15.6333	0.282
0.1	0.05	yadI	5	15.6333	0.626
0.1	0.05	AG1	5	15.6333	0.753
0.1	0.05	ptsI	5	15.8833	0.591
0.1	0.05	clcB	5	15.8833	0.314
0.1	0.05	ycaM	5	15.8833	0.283
0.1	0.05	yadI	5	15.8833	0.629
0.1	0.05	AG1	5	15.8833	0.755
0.1	0.05	ptsI	5	16.1333	0.6
0.1	0.05	clcB	5	16.1333	0.317
0.1	0.05	ycaM	5	16.1333	0.287
0.1	0.05	yadI	5	16.1333	0.639
0.1	0.05	AG1	5	16.1333	0.765
0.1	0.05	ptsI	5	16.3833	0.602
0.1	0.05	clcB	5	16.3833	0.324
0.1	0.05	ycaM	5	16.3833	0.284
0.1	0.05	yadI	5	16.3833	0.634
0.1	0.05	AG1	5	16.3833	0.766
0.1	0.05	ptsI	5	16.6333	0.609
0.1	0.05	clcB	5	16.6333	0.324
0.1	0.05	ycaM	5	16.6333	0.288

0.1	0.05	yadI	5	16.6333	0.637
0.1	0.05	AG1	5	16.6333	0.768
0.1	0.05	ptsI	5	16.8833	0.614
0.1	0.05	clcB	5	16.8833	0.331
0.1	0.05	ycaM	5	16.8833	0.289
0.1	0.05	yadI	5	16.8833	0.64
0.1	0.05	AG1	5	16.8833	0.775
0.1	0.05	ptsI	5	17.1333	0.624
0.1	0.05	clcB	5	17.1333	0.327
0.1	0.05	ycaM	5	17.1333	0.291
0.1	0.05	yadI	5	17.1333	0.647
0.1	0.05	AG1	5	17.1333	0.784
0.1	0.05	ptsI	5	17.3833	0.627
0.1	0.05	clcB	5	17.3833	0.331
0.1	0.05	ycaM	5	17.3833	0.294
0.1	0.05	yadI	5	17.3833	0.645
0.1	0.05	AG1	5	17.3833	0.786
0.1	0.05	ptsI	5	17.6333	0.629
0.1	0.05	clcB	5	17.6333	0.331
0.1	0.05	ycaM	5	17.6333	0.297
0.1	0.05	yadI	5	17.6333	0.647
0.1	0.05	AG1	5	17.6333	0.79
0.1	0.05	ptsI	5	17.8833	0.634
0.1	0.05	clcB	5	17.8833	0.334
0.1	0.05	ycaM	5	17.8833	0.292
0.1	0.05	yadI	5	17.8833	0.648
0.1	0.05	AG1	5	17.8833	0.789
0.1	0.05	ptsI	5	18.1333	0.643
0.1	0.05	clcB	5	18.1333	0.344
0.1	0.05	ycaM	5	18.1333	0.298
0.1	0.05	yadI	5	18.1333	0.653
0.1	0.05	AG1	5	18.1333	0.794
0.1	0.05	ptsI	5	18.3833	0.648
0.1	0.05	clcB	5	18.3833	0.348
0.1	0.05	ycaM	5	18.3833	0.301
0.1	0.05	yadI	5	18.3833	0.658
0.1	0.05	AG1	5	18.3833	0.802
0.1	0.05	ptsI	5	18.6333	0.65
0.1	0.05	clcB	5	18.6333	0.351
0.1	0.05	ycaM	5	18.6333	0.303
0.1	0.05	yadI	5	18.6333	0.659
0.1	0.05	AG1	5	18.6333	0.805
0.1	0.05	ptsI	5	18.8833	0.656
0.1	0.05	clcB	5	18.8833	0.355
0.1	0.05	ycaM	5	18.8833	0.307
0.1	0.05	yadI	5	18.8833	0.662
0.1	0.05	AG1	5	18.8833	0.807
0.1	0.05	ptsI	5	19.1333	0.658
0.1	0.05	clcB	5	19.1333	0.362
0.1	0.05	ycaM	5	19.1333	0.31
0.1	0.05	yadI	5	19.1333	0.67
0.1	0.05	AG1	5	19.1333	0.813
0.1	0.05	ptsI	5	19.3833	0.66

0.1	0.05	clcB	5	19.3833	0.366
0.1	0.05	ycaM	5	19.3833	0.313
0.1	0.05	yadI	5	19.3833	0.67
0.1	0.05	AG1	5	19.3833	0.817
0.1	0.05	ptsI	5	19.6333	0.667
0.1	0.05	clcB	5	19.6333	0.366
0.1	0.05	ycaM	5	19.6333	0.318
0.1	0.05	yadI	5	19.6333	0.667
0.1	0.05	AG1	5	19.6333	0.817
0.1	0.05	ptsI	5	19.8833	0.669
0.1	0.05	clcB	5	19.8833	0.371
0.1	0.05	ycaM	5	19.8833	0.326
0.1	0.05	yadI	5	19.8833	0.673
0.1	0.05	AG1	5	19.8833	0.82
0.1	0.05	ptsI	5	20.1333	0.668
0.1	0.05	clcB	5	20.1333	0.377
0.1	0.05	ycaM	5	20.1333	0.329
0.1	0.05	yadI	5	20.1333	0.674
0.1	0.05	AG1	5	20.1333	0.827
0.1	0.05	ptsI	5	20.3833	0.667
0.1	0.05	clcB	5	20.3833	0.38
0.1	0.05	ycaM	5	20.3833	0.336
0.1	0.05	yadI	5	20.3833	0.677
0.1	0.05	AG1	5	20.3833	0.828
0.1	0.05	ptsI	5	20.6333	0.663
0.1	0.05	clcB	5	20.6333	0.376
0.1	0.05	ycaM	5	20.6333	0.333
0.1	0.05	yadI	5	20.6333	0.67
0.1	0.05	AG1	5	20.6333	0.816
0.1	0.05	ptsI	5	20.8833	0.669
0.1	0.05	clcB	5	20.8833	0.387
0.1	0.05	ycaM	5	20.8833	0.344
0.1	0.05	yadI	5	20.8833	0.682
0.1	0.05	AG1	5	20.8833	0.835
0.1	0.05	ptsI	5	21.1333	0.667
0.1	0.05	clcB	5	21.1333	0.383
0.1	0.05	ycaM	5	21.1333	0.355
0.1	0.05	yadI	5	21.1333	0.684
0.1	0.05	AG1	5	21.1333	0.837
0.1	0.05	ptsI	5	21.3833	0.665
0.1	0.05	clcB	5	21.3833	0.383
0.1	0.05	ycaM	5	21.3833	0.358
0.1	0.05	yadI	5	21.3833	0.687
0.1	0.05	AG1	5	21.3833	0.834
0.1	0.05	ptsI	5	21.6333	0.668
0.1	0.05	clcB	5	21.6333	0.382
0.1	0.05	ycaM	5	21.6333	0.366
0.1	0.05	yadI	5	21.6333	0.684
0.1	0.05	AG1	5	21.6333	0.833
0.1	0.05	ptsI	5	21.8833	0.667
0.1	0.05	clcB	5	21.8833	0.387
0.1	0.05	ycaM	5	21.8833	0.373
0.1	0.05	yadI	5	21.8833	0.688

0.1	0.05	AG1	5	21.8833	0.836
0.1	0.05	ptsI	5	22.1333	0.665
0.1	0.05	clcB	5	22.1333	0.384
0.1	0.05	ycaM	5	22.1333	0.378
0.1	0.05	yadI	5	22.1333	0.684
0.1	0.05	AG1	5	22.1333	0.836
0.1	0.05	ptsI	5	22.3833	0.662
0.1	0.05	clcB	5	22.3833	0.387
0.1	0.05	ycaM	5	22.3833	0.396
0.1	0.05	yadI	5	22.3833	0.691
0.1	0.05	AG1	5	22.3833	0.848
0.1	0.05	ptsI	5	22.6333	0.664
0.1	0.05	clcB	5	22.6333	0.381
0.1	0.05	ycaM	5	22.6333	0.395
0.1	0.05	yadI	5	22.6333	0.69
0.1	0.05	AG1	5	22.6333	0.842
0.1	0.05	ptsI	5	22.8833	0.661
0.1	0.05	clcB	5	22.8833	0.379
0.1	0.05	ycaM	5	22.8833	0.405
0.1	0.05	yadI	5	22.8833	0.69
0.1	0.05	AG1	5	22.8833	0.847
0.1	0.05	ptsI	5	23.1333	0.664
0.1	0.05	clcB	5	23.1333	0.376
0.1	0.05	ycaM	5	23.1333	0.41
0.1	0.05	yadI	5	23.1333	0.688
0.1	0.05	AG1	5	23.1333	0.845
0.1	0.05	ptsI	5	23.3833	0.666
0.1	0.05	clcB	5	23.3833	0.377
0.1	0.05	ycaM	5	23.3833	0.417
0.1	0.05	yadI	5	23.3833	0.694
0.1	0.05	AG1	5	23.3833	0.852
0.1	0.05	ptsI	5	23.6333	0.664
0.1	0.05	clcB	5	23.6333	0.37
0.1	0.05	ycaM	5	23.6333	0.418
0.1	0.05	yadI	5	23.6333	0.698
0.1	0.05	AG1	5	23.6333	0.858
0.1	0.05	ptsI	5	23.8833	0.657
0.1	0.05	clcB	5	23.8833	0.369
0.1	0.05	ycaM	5	23.8833	0.416
0.1	0.05	yadI	5	23.8833	0.693
0.1	0.05	AG1	5	23.8833	0.851
0.1	0.05	ptsI	5	24.1333	0.658
0.1	0.05	clcB	5	24.1333	0.362
0.1	0.05	ycaM	5	24.1333	0.422
0.1	0.05	yadI	5	24.1333	0.698
0.1	0.05	AG1	5	24.1333	0.859
0.1	0.05	ptsI	5	24.3833	0.654
0.1	0.05	clcB	5	24.3833	0.362
0.1	0.05	ycaM	5	24.3833	0.43
0.1	0.05	yadI	5	24.3833	0.698
0.1	0.05	AG1	5	24.3833	0.862
0.2	0.05	ptsI	2	0	0.216
0.2	0.05	clcB	2	0	0.229

0.2	0.05	ycaM	2	0	0.218
0.2	0.05	yadI	2	0	0.222
0.2	0.05	AG1	2	0	0.234
0.2	0.05	ptsI	2	0.25	0.218
0.2	0.05	clcB	2	0.25	0.226
0.2	0.05	ycaM	2	0.25	0.213
0.2	0.05	yadI	2	0.25	0.218
0.2	0.05	AG1	2	0.25	0.226
0.2	0.05	ptsI	2	0.5	0.213
0.2	0.05	clcB	2	0.5	0.214
0.2	0.05	ycaM	2	0.5	0.212
0.2	0.05	yadI	2	0.5	0.216
0.2	0.05	AG1	2	0.5	0.221
0.2	0.05	ptsI	2	0.75	0.214
0.2	0.05	clcB	2	0.75	0.214
0.2	0.05	ycaM	2	0.75	0.213
0.2	0.05	yadI	2	0.75	0.218
0.2	0.05	AG1	2	0.75	0.225
0.2	0.05	ptsI	2	1	0.218
0.2	0.05	clcB	2	1	0.218
0.2	0.05	ycaM	2	1	0.216
0.2	0.05	yadI	2	1	0.224
0.2	0.05	AG1	2	1	0.227
0.2	0.05	ptsI	2	1.25	0.22
0.2	0.05	clcB	2	1.25	0.219
0.2	0.05	ycaM	2	1.25	0.217
0.2	0.05	yadI	2	1.25	0.229
0.2	0.05	AG1	2	1.25	0.229
0.2	0.05	ptsI	2	1.5	0.22
0.2	0.05	clcB	2	1.5	0.222
0.2	0.05	ycaM	2	1.5	0.219
0.2	0.05	yadI	2	1.5	0.236
0.2	0.05	AG1	2	1.5	0.23
0.2	0.05	ptsI	2	1.75	0.224
0.2	0.05	clcB	2	1.75	0.226
0.2	0.05	ycaM	2	1.75	0.223
0.2	0.05	yadI	2	1.75	0.241
0.2	0.05	AG1	2	1.75	0.239
0.2	0.05	ptsI	2	2	0.226
0.2	0.05	clcB	2	2	0.229
0.2	0.05	ycaM	2	2	0.23
0.2	0.05	yadI	2	2	0.249
0.2	0.05	AG1	2	2	0.244
0.2	0.05	ptsI	2	2.25	0.235
0.2	0.05	clcB	2	2.25	0.236
0.2	0.05	ycaM	2	2.25	0.234
0.2	0.05	yadI	2	2.25	0.259
0.2	0.05	AG1	2	2.25	0.252
0.2	0.05	ptsI	2	2.5	0.242
0.2	0.05	clcB	2	2.5	0.243
0.2	0.05	ycaM	2	2.5	0.241
0.2	0.05	yadI	2	2.5	0.27
0.2	0.05	AG1	2	2.5	0.26

0.2	0.05	ptsI	2	2.75	0.243
0.2	0.05	clcB	2	2.75	0.251
0.2	0.05	ycaM	2	2.75	0.244
0.2	0.05	yadI	2	2.75	0.281
0.2	0.05	AG1	2	2.75	0.258
0.2	0.05	ptsI	2	3	0.248
0.2	0.05	clcB	2	3	0.249
0.2	0.05	ycaM	2	3	0.246
0.2	0.05	yadI	2	3	0.287
0.2	0.05	AG1	2	3	0.258
0.2	0.05	ptsI	2	3.25	0.255
0.2	0.05	clcB	2	3.25	0.265
0.2	0.05	ycaM	2	3.25	0.254
0.2	0.05	yadI	2	3.25	0.305
0.2	0.05	AG1	2	3.25	0.269
0.2	0.05	ptsI	2	3.5	0.264
0.2	0.05	clcB	2	3.5	0.271
0.2	0.05	ycaM	2	3.5	0.258
0.2	0.05	yadI	2	3.5	0.318
0.2	0.05	AG1	2	3.5	0.281
0.2	0.05	ptsI	2	3.75	0.27
0.2	0.05	clcB	2	3.75	0.274
0.2	0.05	ycaM	2	3.75	0.268
0.2	0.05	yadI	2	3.75	0.332
0.2	0.05	AG1	2	3.75	0.289
0.2	0.05	ptsI	2	4	0.28
0.2	0.05	clcB	2	4	0.282
0.2	0.05	ycaM	2	4	0.279
0.2	0.05	yadI	2	4	0.344
0.2	0.05	AG1	2	4	0.3
0.2	0.05	ptsI	2	4.38333	0.318
0.2	0.05	clcB	2	4.38333	0.262
0.2	0.05	ycaM	2	4.38333	0.288
0.2	0.05	yadI	2	4.38333	0.32
0.2	0.05	AG1	2	4.38333	0.294
0.2	0.05	ptsI	2	4.63333	0.311
0.2	0.05	clcB	2	4.63333	0.252
0.2	0.05	ycaM	2	4.63333	0.285
0.2	0.05	yadI	2	4.63333	0.33
0.2	0.05	AG1	2	4.63333	0.293
0.2	0.05	ptsI	2	4.88333	0.316
0.2	0.05	clcB	2	4.88333	0.252
0.2	0.05	ycaM	2	4.88333	0.289
0.2	0.05	yadI	2	4.88333	0.34
0.2	0.05	AG1	2	4.88333	0.304
0.2	0.05	ptsI	2	5.13333	0.324
0.2	0.05	clcB	2	5.13333	0.255
0.2	0.05	ycaM	2	5.13333	0.293
0.2	0.05	yadI	2	5.13333	0.357
0.2	0.05	AG1	2	5.13333	0.311
0.2	0.05	ptsI	2	5.38333	0.334
0.2	0.05	clcB	2	5.38333	0.259
0.2	0.05	ycaM	2	5.38333	0.295

0.2	0.05	yadI	2	5.38333	0.368
0.2	0.05	AG1	2	5.38333	0.323
0.2	0.05	ptsI	2	5.63333	0.342
0.2	0.05	clcB	2	5.63333	0.269
0.2	0.05	ycaM	2	5.63333	0.297
0.2	0.05	yadI	2	5.63333	0.382
0.2	0.05	AG1	2	5.63333	0.332
0.2	0.05	ptsI	2	5.88333	0.35
0.2	0.05	clcB	2	5.88333	0.274
0.2	0.05	ycaM	2	5.88333	0.298
0.2	0.05	yadI	2	5.88333	0.387
0.2	0.05	AG1	2	5.88333	0.34
0.2	0.05	ptsI	2	6.13333	0.362
0.2	0.05	clcB	2	6.13333	0.277
0.2	0.05	ycaM	2	6.13333	0.299
0.2	0.05	yadI	2	6.13333	0.396
0.2	0.05	AG1	2	6.13333	0.35
0.2	0.05	ptsI	2	6.38333	0.371
0.2	0.05	clcB	2	6.38333	0.282
0.2	0.05	ycaM	2	6.38333	0.302
0.2	0.05	yadI	2	6.38333	0.404
0.2	0.05	AG1	2	6.38333	0.356
0.2	0.05	ptsI	2	6.63333	0.381
0.2	0.05	clcB	2	6.63333	0.287
0.2	0.05	ycaM	2	6.63333	0.301
0.2	0.05	yadI	2	6.63333	0.414
0.2	0.05	AG1	2	6.63333	0.366
0.2	0.05	ptsI	2	6.88333	0.394
0.2	0.05	clcB	2	6.88333	0.293
0.2	0.05	ycaM	2	6.88333	0.303
0.2	0.05	yadI	2	6.88333	0.424
0.2	0.05	AG1	2	6.88333	0.376
0.2	0.05	ptsI	2	7.13333	0.406
0.2	0.05	clcB	2	7.13333	0.294
0.2	0.05	ycaM	2	7.13333	0.304
0.2	0.05	yadI	2	7.13333	0.431
0.2	0.05	AG1	2	7.13333	0.385
0.2	0.05	ptsI	2	7.38333	0.415
0.2	0.05	clcB	2	7.38333	0.303
0.2	0.05	ycaM	2	7.38333	0.305
0.2	0.05	yadI	2	7.38333	0.441
0.2	0.05	AG1	2	7.38333	0.393
0.2	0.05	ptsI	2	7.63333	0.422
0.2	0.05	clcB	2	7.63333	0.304
0.2	0.05	ycaM	2	7.63333	0.303
0.2	0.05	yadI	2	7.63333	0.443
0.2	0.05	AG1	2	7.63333	0.399
0.2	0.05	ptsI	2	7.88333	0.435
0.2	0.05	clcB	2	7.88333	0.306
0.2	0.05	ycaM	2	7.88333	0.305
0.2	0.05	yadI	2	7.88333	0.456
0.2	0.05	AG1	2	7.88333	0.408
0.2	0.05	ptsI	2	8.13333	0.448

0.2	0.05	clcB	2	8.13333	0.309
0.2	0.05	ycaM	2	8.13333	0.306
0.2	0.05	yadI	2	8.13333	0.462
0.2	0.05	AG1	2	8.13333	0.418
0.2	0.05	ptsI	2	8.38333	0.455
0.2	0.05	clcB	2	8.38333	0.318
0.2	0.05	ycaM	2	8.38333	0.306
0.2	0.05	yadI	2	8.38333	0.472
0.2	0.05	AG1	2	8.38333	0.426
0.2	0.05	ptsI	2	8.91667	0.425
0.2	0.05	clcB	2	8.91667	0.328
0.2	0.05	ycaM	2	8.91667	0.286
0.2	0.05	yadI	2	8.91667	0.503
0.2	0.05	AG1	2	8.91667	0.463
0.2	0.05	ptsI	2	9.16667	0.424
0.2	0.05	clcB	2	9.16667	0.327
0.2	0.05	ycaM	2	9.16667	0.287
0.2	0.05	yadI	2	9.16667	0.512
0.2	0.05	AG1	2	9.16667	0.476
0.2	0.05	ptsI	2	9.41667	0.432
0.2	0.05	clcB	2	9.41667	0.332
0.2	0.05	ycaM	2	9.41667	0.286
0.2	0.05	yadI	2	9.41667	0.524
0.2	0.05	AG1	2	9.41667	0.492
0.2	0.05	ptsI	2	9.66667	0.445
0.2	0.05	clcB	2	9.66667	0.338
0.2	0.05	ycaM	2	9.66667	0.289
0.2	0.05	yadI	2	9.66667	0.538
0.2	0.05	AG1	2	9.66667	0.51
0.2	0.05	ptsI	2	9.91667	0.456
0.2	0.05	clcB	2	9.91667	0.342
0.2	0.05	ycaM	2	9.91667	0.29
0.2	0.05	yadI	2	9.91667	0.549
0.2	0.05	AG1	2	9.91667	0.528
0.2	0.05	ptsI	2	10.1667	0.47
0.2	0.05	clcB	2	10.1667	0.342
0.2	0.05	ycaM	2	10.1667	0.29
0.2	0.05	yadI	2	10.1667	0.555
0.2	0.05	AG1	2	10.1667	0.536
0.2	0.05	ptsI	2	10.4167	0.487
0.2	0.05	clcB	2	10.4167	0.349
0.2	0.05	ycaM	2	10.4167	0.294
0.2	0.05	yadI	2	10.4167	0.567
0.2	0.05	AG1	2	10.4167	0.559
0.2	0.05	ptsI	2	10.6667	0.502
0.2	0.05	clcB	2	10.6667	0.353
0.2	0.05	ycaM	2	10.6667	0.295
0.2	0.05	yadI	2	10.6667	0.574
0.2	0.05	AG1	2	10.6667	0.571
0.2	0.05	ptsI	2	10.9167	0.518
0.2	0.05	clcB	2	10.9167	0.361
0.2	0.05	ycaM	2	10.9167	0.294
0.2	0.05	yadI	2	10.9167	0.585

0.2	0.05	AG1	2	10.9167	0.585
0.2	0.05	ptsI	2	11.1667	0.539
0.2	0.05	clcB	2	11.1667	0.352
0.2	0.05	ycaM	2	11.1667	0.296
0.2	0.05	yadI	2	11.1667	0.592
0.2	0.05	AG1	2	11.1667	0.597
0.2	0.05	ptsI	2	11.4167	0.551
0.2	0.05	clcB	2	11.4167	0.352
0.2	0.05	ycaM	2	11.4167	0.296
0.2	0.05	yadI	2	11.4167	0.6
0.2	0.05	AG1	2	11.4167	0.596
0.2	0.05	ptsI	2	11.6667	0.564
0.2	0.05	clcB	2	11.6667	0.353
0.2	0.05	ycaM	2	11.6667	0.296
0.2	0.05	yadI	2	11.6667	0.613
0.2	0.05	AG1	2	11.6667	0.628
0.2	0.05	ptsI	2	11.9167	0.578
0.2	0.05	clcB	2	11.9167	0.351
0.2	0.05	ycaM	2	11.9167	0.296
0.2	0.05	yadI	2	11.9167	0.614
0.2	0.05	AG1	2	11.9167	0.615
0.2	0.05	ptsI	2	12.1667	0.598
0.2	0.05	clcB	2	12.1667	0.352
0.2	0.05	ycaM	2	12.1667	0.296
0.2	0.05	yadI	2	12.1667	0.625
0.2	0.05	AG1	2	12.1667	0.647
0.2	0.05	ptsI	2	12.4167	0.6
0.2	0.05	clcB	2	12.4167	0.347
0.2	0.05	ycaM	2	12.4167	0.296
0.2	0.05	yadI	2	12.4167	0.631
0.2	0.05	AG1	2	12.4167	0.637
0.2	0.05	ptsI	2	12.6667	0.609
0.2	0.05	clcB	2	12.6667	0.347
0.2	0.05	ycaM	2	12.6667	0.298
0.2	0.05	yadI	2	12.6667	0.635
0.2	0.05	AG1	2	12.6667	0.644
0.2	0.05	ptsI	2	12.9167	0.629
0.2	0.05	clcB	2	12.9167	0.338
0.2	0.05	ycaM	2	12.9167	0.297
0.2	0.05	yadI	2	12.9167	0.644
0.2	0.05	AG1	2	12.9167	0.638
0.2	0.05	ptsI	2	13.1667	0.637
0.2	0.05	clcB	2	13.1667	0.343
0.2	0.05	ycaM	2	13.1667	0.297
0.2	0.05	yadI	2	13.1667	0.65
0.2	0.05	AG1	2	13.1667	0.645
0.2	0.05	ptsI	2	13.4167	0.646
0.2	0.05	clcB	2	13.4167	0.336
0.2	0.05	ycaM	2	13.4167	0.293
0.2	0.05	yadI	2	13.4167	0.656
0.2	0.05	AG1	2	13.4167	0.638
0.2	0.05	ptsI	2	13.6667	0.668
0.2	0.05	clcB	2	13.6667	0.341

0.2	0.05	ycaM	2	13.6667	0.293
0.2	0.05	yadI	2	13.6667	0.669
0.2	0.05	AG1	2	13.6667	0.663
0.2	0.05	ptsI	2	13.9167	0.674
0.2	0.05	clcB	2	13.9167	0.35
0.2	0.05	ycaM	2	13.9167	0.294
0.2	0.05	yadI	2	13.9167	0.672
0.2	0.05	AG1	2	13.9167	0.666
0.2	0.05	ptsI	2	14.1667	0.676
0.2	0.05	clcB	2	14.1667	0.34
0.2	0.05	ycaM	2	14.1667	0.296
0.2	0.05	yadI	2	14.1667	0.677
0.2	0.05	AG1	2	14.1667	0.669
0.2	0.05	ptsI	2	14.4167	0.69
0.2	0.05	clcB	2	14.4167	0.343
0.2	0.05	ycaM	2	14.4167	0.295
0.2	0.05	yadI	2	14.4167	0.681
0.2	0.05	AG1	2	14.4167	0.655
0.2	0.05	ptsI	2	14.6667	0.69
0.2	0.05	clcB	2	14.6667	0.342
0.2	0.05	ycaM	2	14.6667	0.294
0.2	0.05	yadI	2	14.6667	0.69
0.2	0.05	AG1	2	14.6667	0.659
0.2	0.05	ptsI	2	14.9167	0.708
0.2	0.05	clcB	2	14.9167	0.345
0.2	0.05	ycaM	2	14.9167	0.294
0.2	0.05	yadI	2	14.9167	0.703
0.2	0.05	AG1	2	14.9167	0.68
0.2	0.05	ptsI	2	15.1667	0.709
0.2	0.05	clcB	2	15.1667	0.353
0.2	0.05	ycaM	2	15.1667	0.296
0.2	0.05	yadI	2	15.1667	0.712
0.2	0.05	AG1	2	15.1667	0.703
0.2	0.05	ptsI	2	15.4167	0.722
0.2	0.05	clcB	2	15.4167	0.358
0.2	0.05	ycaM	2	15.4167	0.292
0.2	0.05	yadI	2	15.4167	0.713
0.2	0.05	AG1	2	15.4167	0.696
0.2	0.05	ptsI	2	15.6667	0.719
0.2	0.05	clcB	2	15.6667	0.35
0.2	0.05	ycaM	2	15.6667	0.29
0.2	0.05	yadI	2	15.6667	0.716
0.2	0.05	AG1	2	15.6667	0.704
0.2	0.05	ptsI	2	15.9167	0.719
0.2	0.05	clcB	2	15.9167	0.355
0.2	0.05	ycaM	2	15.9167	0.29
0.2	0.05	yadI	2	15.9167	0.719
0.2	0.05	AG1	2	15.9167	0.727
0.2	0.05	ptsI	2	16.1667	0.739
0.2	0.05	clcB	2	16.1667	0.352
0.2	0.05	ycaM	2	16.1667	0.29
0.2	0.05	yadI	2	16.1667	0.731
0.2	0.05	AG1	2	16.1667	0.733

0.2	0.05	ptsI	2	16.4167	0.731
0.2	0.05	clcB	2	16.4167	0.353
0.2	0.05	ycaM	2	16.4167	0.29
0.2	0.05	yadI	2	16.4167	0.733
0.2	0.05	AG1	2	16.4167	0.752
0.2	0.05	ptsI	2	16.6667	0.732
0.2	0.05	clcB	2	16.6667	0.353
0.2	0.05	ycaM	2	16.6667	0.291
0.2	0.05	yadI	2	16.6667	0.738
0.2	0.05	AG1	2	16.6667	0.748
0.2	0.05	ptsI	2	16.9167	0.742
0.2	0.05	clcB	2	16.9167	0.357
0.2	0.05	ycaM	2	16.9167	0.29
0.2	0.05	yadI	2	16.9167	0.739
0.2	0.05	AG1	2	16.9167	0.747
0.2	0.05	ptsI	2	17.1667	0.748
0.2	0.05	clcB	2	17.1667	0.354
0.2	0.05	ycaM	2	17.1667	0.291
0.2	0.05	yadI	2	17.1667	0.756
0.2	0.05	AG1	2	17.1667	0.734
0.2	0.05	ptsI	2	17.4167	0.755
0.2	0.05	clcB	2	17.4167	0.354
0.2	0.05	ycaM	2	17.4167	0.291
0.2	0.05	yadI	2	17.4167	0.757
0.2	0.05	AG1	2	17.4167	0.753
0.2	0.05	ptsI	2	17.6667	0.758
0.2	0.05	clcB	2	17.6667	0.356
0.2	0.05	ycaM	2	17.6667	0.291
0.2	0.05	yadI	2	17.6667	0.763
0.2	0.05	AG1	2	17.6667	0.768
0.2	0.05	ptsI	2	17.9167	0.755
0.2	0.05	clcB	2	17.9167	0.354
0.2	0.05	ycaM	2	17.9167	0.292
0.2	0.05	yadI	2	17.9167	0.764
0.2	0.05	AG1	2	17.9167	0.753
0.2	0.05	ptsI	2	18.1667	0.765
0.2	0.05	clcB	2	18.1667	0.355
0.2	0.05	ycaM	2	18.1667	0.291
0.2	0.05	yadI	2	18.1667	0.761
0.2	0.05	AG1	2	18.1667	0.781
0.2	0.05	ptsI	2	18.4167	0.772
0.2	0.05	clcB	2	18.4167	0.354
0.2	0.05	ycaM	2	18.4167	0.293
0.2	0.05	yadI	2	18.4167	0.766
0.2	0.05	AG1	2	18.4167	0.776
0.2	0.05	ptsI	2	18.6667	0.758
0.2	0.05	clcB	2	18.6667	0.354
0.2	0.05	ycaM	2	18.6667	0.293
0.2	0.05	yadI	2	18.6667	0.767
0.2	0.05	AG1	2	18.6667	0.782
0.2	0.05	ptsI	2	18.9167	0.744
0.2	0.05	clcB	2	18.9167	0.355
0.2	0.05	ycaM	2	18.9167	0.289

0.2	0.05	yadI	2	18.9167	0.769
0.2	0.05	AG1	2	18.9167	0.791
0.2	0.05	ptsI	2	19.1667	0.77
0.2	0.05	clcB	2	19.1667	0.355
0.2	0.05	ycaM	2	19.1667	0.294
0.2	0.05	yadI	2	19.1667	0.767
0.2	0.05	AG1	2	19.1667	0.802
0.2	0.05	ptsI	2	19.4167	0.772
0.2	0.05	clcB	2	19.4167	0.356
0.2	0.05	ycaM	2	19.4167	0.294
0.2	0.05	yadI	2	19.4167	0.773
0.2	0.05	AG1	2	19.4167	0.801
0.2	0.05	ptsI	2	19.6667	0.776
0.2	0.05	clcB	2	19.6667	0.356
0.2	0.05	ycaM	2	19.6667	0.295
0.2	0.05	yadI	2	19.6667	0.785
0.2	0.05	AG1	2	19.6667	0.804
0.2	0.05	ptsI	2	19.9167	0.782
0.2	0.05	clcB	2	19.9167	0.353
0.2	0.05	ycaM	2	19.9167	0.297
0.2	0.05	yadI	2	19.9167	0.778
0.2	0.05	AG1	2	19.9167	0.819
0.2	0.05	ptsI	2	20.1667	0.775
0.2	0.05	clcB	2	20.1667	0.356
0.2	0.05	ycaM	2	20.1667	0.297
0.2	0.05	yadI	2	20.1667	0.79
0.2	0.05	AG1	2	20.1667	0.816
0.2	0.05	ptsI	2	20.4167	0.782
0.2	0.05	clcB	2	20.4167	0.353
0.2	0.05	ycaM	2	20.4167	0.297
0.2	0.05	yadI	2	20.4167	0.787
0.2	0.05	AG1	2	20.4167	0.825
0.2	0.05	ptsI	2	20.6667	0.774
0.2	0.05	clcB	2	20.6667	0.354
0.2	0.05	ycaM	2	20.6667	0.3
0.2	0.05	yadI	2	20.6667	0.786
0.2	0.05	AG1	2	20.6667	0.824
0.2	0.05	ptsI	2	20.9167	0.782
0.2	0.05	clcB	2	20.9167	0.354
0.2	0.05	ycaM	2	20.9167	0.302
0.2	0.05	yadI	2	20.9167	0.797
0.2	0.05	AG1	2	20.9167	0.832
0.2	0.05	ptsI	2	21.1667	0.784
0.2	0.05	clcB	2	21.1667	0.351
0.2	0.05	ycaM	2	21.1667	0.304
0.2	0.05	yadI	2	21.1667	0.799
0.2	0.05	AG1	2	21.1667	0.837
0.2	0.05	ptsI	2	21.4167	0.768
0.2	0.05	clcB	2	21.4167	0.353
0.2	0.05	ycaM	2	21.4167	0.304
0.2	0.05	yadI	2	21.4167	0.8
0.2	0.05	AG1	2	21.4167	0.84
0.2	0.05	ptsI	2	21.6667	0.773

0.2	0.05	clcB	2	21.6667	0.352
0.2	0.05	ycaM	2	21.6667	0.307
0.2	0.05	yadI	2	21.6667	0.798
0.2	0.05	AG1	2	21.6667	0.849
0.2	0.05	ptsI	2	21.9167	0.766
0.2	0.05	clcB	2	21.9167	0.352
0.2	0.05	ycaM	2	21.9167	0.308
0.2	0.05	yadI	2	21.9167	0.806
0.2	0.05	AG1	2	21.9167	0.851
0.2	0.05	ptsI	2	22.1667	0.769
0.2	0.05	clcB	2	22.1667	0.355
0.2	0.05	ycaM	2	22.1667	0.31
0.2	0.05	yadI	2	22.1667	0.801
0.2	0.05	AG1	2	22.1667	0.858
0.2	0.05	ptsI	2	22.4167	0.776
0.2	0.05	clcB	2	22.4167	0.355
0.2	0.05	ycaM	2	22.4167	0.313
0.2	0.05	yadI	2	22.4167	0.803
0.2	0.05	AG1	2	22.4167	0.862
0.2	0.05	ptsI	2	22.6667	0.766
0.2	0.05	clcB	2	22.6667	0.354
0.2	0.05	ycaM	2	22.6667	0.314
0.2	0.05	yadI	2	22.6667	0.81
0.2	0.05	AG1	2	22.6667	0.868
0.2	0.05	ptsI	2	22.9167	0.778
0.2	0.05	clcB	2	22.9167	0.352
0.2	0.05	ycaM	2	22.9167	0.316
0.2	0.05	yadI	2	22.9167	0.809
0.2	0.05	AG1	2	22.9167	0.878
0.2	0.05	ptsI	2	23.1667	0.767
0.2	0.05	clcB	2	23.1667	0.352
0.2	0.05	ycaM	2	23.1667	0.318
0.2	0.05	yadI	2	23.1667	0.809
0.2	0.05	AG1	2	23.1667	0.882
0.2	0.05	ptsI	2	23.4167	0.773
0.2	0.05	clcB	2	23.4167	0.352
0.2	0.05	ycaM	2	23.4167	0.321
0.2	0.05	yadI	2	23.4167	0.813
0.2	0.05	AG1	2	23.4167	0.883
0.2	0.05	ptsI	2	23.6667	0.775
0.2	0.05	clcB	2	23.6667	0.351
0.2	0.05	ycaM	2	23.6667	0.322
0.2	0.05	yadI	2	23.6667	0.821
0.2	0.05	AG1	2	23.6667	0.889
0.2	0.05	ptsI	2	23.9167	0.773
0.2	0.05	clcB	2	23.9167	0.352
0.2	0.05	ycaM	2	23.9167	0.327
0.2	0.05	yadI	2	23.9167	0.822
0.2	0.05	AG1	2	23.9167	0.899
0.2	0.05	ptsI	2	24.1667	0.77
0.2	0.05	clcB	2	24.1667	0.352
0.2	0.05	ycaM	2	24.1667	0.327
0.2	0.05	yadI	2	24.1667	0.824

0.2	0.05	AG1	2	24.1667	0.907
0.2	0.05	ptsI	2	24.4167	0.764
0.2	0.05	clcB	2	24.4167	0.353
0.2	0.05	ycaM	2	24.4167	0.329
0.2	0.05	yadI	2	24.4167	0.825
0.2	0.05	AG1	2	24.4167	0.912
0.2	0.05	ptsI	2	24.6667	0.763
0.2	0.05	clcB	2	24.6667	0.355
0.2	0.05	ycaM	2	24.6667	0.332
0.2	0.05	yadI	2	24.6667	0.826
0.2	0.05	AG1	2	24.6667	0.917
0.2	0.05	ptsI	2	24.9167	0.764
0.2	0.05	clcB	2	24.9167	0.354
0.2	0.05	ycaM	2	24.9167	0.333
0.2	0.05	yadI	2	24.9167	0.829
0.2	0.05	AG1	2	24.9167	0.925
0.2	0.05	ptsI	2	25.1667	0.764
0.2	0.05	clcB	2	25.1667	0.355
0.2	0.05	ycaM	2	25.1667	0.339
0.2	0.05	yadI	2	25.1667	0.837
0.2	0.05	AG1	2	25.1667	0.932
0.2	0.05	ptsI	2	25.4167	0.762
0.2	0.05	clcB	2	25.4167	0.36
0.2	0.05	ycaM	2	25.4167	0.343
0.2	0.05	yadI	2	25.4167	0.833
0.2	0.05	AG1	2	25.4167	0.941
0.2	0.05	ptsI	2	25.6667	0.756
0.2	0.05	clcB	2	25.6667	0.359
0.2	0.05	ycaM	2	25.6667	0.343
0.2	0.05	yadI	2	25.6667	0.84
0.2	0.05	AG1	2	25.6667	0.95
0.2	0.05	ptsI	2	25.9167	0.759
0.2	0.05	clcB	2	25.9167	0.361
0.2	0.05	ycaM	2	25.9167	0.348
0.2	0.05	yadI	2	25.9167	0.845
0.2	0.05	AG1	2	25.9167	0.959
0.2	0.05	ptsI	2	26.1667	0.734
0.2	0.05	clcB	2	26.1667	0.357
0.2	0.05	ycaM	2	26.1667	0.347
0.2	0.05	yadI	2	26.1667	0.846
0.2	0.05	AG1	2	26.1667	0.972
0.2	0.05	ptsI	2	26.4167	0.752
0.2	0.05	clcB	2	26.4167	0.362
0.2	0.05	ycaM	2	26.4167	0.35
0.2	0.05	yadI	2	26.4167	0.844
0.2	0.05	AG1	2	26.4167	0.984
0.2	0.05	ptsI	3	0	0.161
0.2	0.05	clcB	3	0	0.163
0.2	0.05	ycaM	3	0	0.155
0.2	0.05	yadI	3	0	0.168
0.2	0.05	AG1	3	0	0.163
0.2	0.05	ptsI	3	0.25	0.153
0.2	0.05	clcB	3	0.25	0.161

0.2	0.05	ycaM	3	0.25	0.152
0.2	0.05	yadI	3	0.25	0.164
0.2	0.05	AG1	3	0.25	0.159
0.2	0.05	ptsI	3	0.5	0.152
0.2	0.05	clcB	3	0.5	0.159
0.2	0.05	ycaM	3	0.5	0.151
0.2	0.05	yadI	3	0.5	0.164
0.2	0.05	AG1	3	0.5	0.156
0.2	0.05	ptsI	3	0.75	0.152
0.2	0.05	clcB	3	0.75	0.159
0.2	0.05	ycaM	3	0.75	0.151
0.2	0.05	yadI	3	0.75	0.164
0.2	0.05	AG1	3	0.75	0.156
0.2	0.05	ptsI	3	1	0.152
0.2	0.05	clcB	3	1	0.16
0.2	0.05	ycaM	3	1	0.152
0.2	0.05	yadI	3	1	0.164
0.2	0.05	AG1	3	1	0.158
0.2	0.05	ptsI	3	1.25	0.152
0.2	0.05	clcB	3	1.25	0.16
0.2	0.05	ycaM	3	1.25	0.152
0.2	0.05	yadI	3	1.25	0.164
0.2	0.05	AG1	3	1.25	0.157
0.2	0.05	ptsI	3	1.5	0.154
0.2	0.05	clcB	3	1.5	0.161
0.2	0.05	ycaM	3	1.5	0.153
0.2	0.05	yadI	3	1.5	0.167
0.2	0.05	AG1	3	1.5	0.158
0.2	0.05	ptsI	3	1.75	0.154
0.2	0.05	clcB	3	1.75	0.161
0.2	0.05	ycaM	3	1.75	0.153
0.2	0.05	yadI	3	1.75	0.167
0.2	0.05	AG1	3	1.75	0.158
0.2	0.05	ptsI	3	2	0.156
0.2	0.05	clcB	3	2	0.161
0.2	0.05	ycaM	3	2	0.154
0.2	0.05	yadI	3	2	0.168
0.2	0.05	AG1	3	2	0.159
0.2	0.05	ptsI	3	2.25	0.158
0.2	0.05	clcB	3	2.25	0.164
0.2	0.05	ycaM	3	2.25	0.156
0.2	0.05	yadI	3	2.25	0.17
0.2	0.05	AG1	3	2.25	0.159
0.2	0.05	ptsI	3	2.5	0.16
0.2	0.05	clcB	3	2.5	0.164
0.2	0.05	ycaM	3	2.5	0.156
0.2	0.05	yadI	3	2.5	0.172
0.2	0.05	AG1	3	2.5	0.162
0.2	0.05	ptsI	3	2.75	0.164
0.2	0.05	clcB	3	2.75	0.167
0.2	0.05	ycaM	3	2.75	0.159
0.2	0.05	yadI	3	2.75	0.177
0.2	0.05	AG1	3	2.75	0.165

0.2	0.05	ptsI	3	3	0.164
0.2	0.05	clcB	3	3	0.167
0.2	0.05	ycaM	3	3	0.16
0.2	0.05	yadI	3	3	0.181
0.2	0.05	AG1	3	3	0.164
0.2	0.05	ptsI	3	3.25	0.166
0.2	0.05	clcB	3	3.25	0.168
0.2	0.05	ycaM	3	3.25	0.162
0.2	0.05	yadI	3	3.25	0.185
0.2	0.05	AG1	3	3.25	0.167
0.2	0.05	ptsI	3	3.5	0.17
0.2	0.05	clcB	3	3.5	0.172
0.2	0.05	ycaM	3	3.5	0.164
0.2	0.05	yadI	3	3.5	0.19
0.2	0.05	AG1	3	3.5	0.17
0.2	0.05	ptsI	3	3.75	0.172
0.2	0.05	clcB	3	3.75	0.173
0.2	0.05	ycaM	3	3.75	0.167
0.2	0.05	yadI	3	3.75	0.194
0.2	0.05	AG1	3	3.75	0.172
0.2	0.05	ptsI	3	4	0.175
0.2	0.05	clcB	3	4	0.176
0.2	0.05	ycaM	3	4	0.169
0.2	0.05	yadI	3	4	0.199
0.2	0.05	AG1	3	4	0.173
0.2	0.05	ptsI	3	4.41667	0.208
0.2	0.05	clcB	3	4.41667	0.223
0.2	0.05	ycaM	3	4.41667	0.183
0.2	0.05	yadI	3	4.41667	0.235
0.2	0.05	AG1	3	4.41667	0.187
0.2	0.05	ptsI	3	4.66667	0.193
0.2	0.05	clcB	3	4.66667	0.182
0.2	0.05	ycaM	3	4.66667	0.177
0.2	0.05	yadI	3	4.66667	0.236
0.2	0.05	AG1	3	4.66667	0.196
0.2	0.05	ptsI	3	4.91667	0.191
0.2	0.05	clcB	3	4.91667	0.183
0.2	0.05	ycaM	3	4.91667	0.18
0.2	0.05	yadI	3	4.91667	0.249
0.2	0.05	AG1	3	4.91667	0.206
0.2	0.05	ptsI	3	5.16667	0.196
0.2	0.05	clcB	3	5.16667	0.186
0.2	0.05	ycaM	3	5.16667	0.184
0.2	0.05	yadI	3	5.16667	0.264
0.2	0.05	AG1	3	5.16667	0.212
0.2	0.05	ptsI	3	5.41667	0.2
0.2	0.05	clcB	3	5.41667	0.189
0.2	0.05	ycaM	3	5.41667	0.19
0.2	0.05	yadI	3	5.41667	0.282
0.2	0.05	AG1	3	5.41667	0.214
0.2	0.05	ptsI	3	5.66667	0.205
0.2	0.05	clcB	3	5.66667	0.195
0.2	0.05	ycaM	3	5.66667	0.196

0.2	0.05	yadI	3	5.66667	0.301
0.2	0.05	AG1	3	5.66667	0.221
0.2	0.05	ptsI	3	5.91667	0.212
0.2	0.05	clcB	3	5.91667	0.201
0.2	0.05	ycaM	3	5.91667	0.203
0.2	0.05	yadI	3	5.91667	0.321
0.2	0.05	AG1	3	5.91667	0.222
0.2	0.05	ptsI	3	6.16667	0.218
0.2	0.05	clcB	3	6.16667	0.21
0.2	0.05	ycaM	3	6.16667	0.212
0.2	0.05	yadI	3	6.16667	0.336
0.2	0.05	AG1	3	6.16667	0.23
0.2	0.05	ptsI	3	6.41667	0.228
0.2	0.05	clcB	3	6.41667	0.217
0.2	0.05	ycaM	3	6.41667	0.219
0.2	0.05	yadI	3	6.41667	0.345
0.2	0.05	AG1	3	6.41667	0.239
0.2	0.05	ptsI	3	6.66667	0.238
0.2	0.05	clcB	3	6.66667	0.227
0.2	0.05	ycaM	3	6.66667	0.227
0.2	0.05	yadI	3	6.66667	0.357
0.2	0.05	AG1	3	6.66667	0.251
0.2	0.05	ptsI	3	6.91667	0.249
0.2	0.05	clcB	3	6.91667	0.236
0.2	0.05	ycaM	3	6.91667	0.235
0.2	0.05	yadI	3	6.91667	0.368
0.2	0.05	AG1	3	6.91667	0.262
0.2	0.05	ptsI	3	7.16667	0.258
0.2	0.05	clcB	3	7.16667	0.247
0.2	0.05	ycaM	3	7.16667	0.241
0.2	0.05	yadI	3	7.16667	0.381
0.2	0.05	AG1	3	7.16667	0.271
0.2	0.05	ptsI	3	7.41667	0.268
0.2	0.05	clcB	3	7.41667	0.258
0.2	0.05	ycaM	3	7.41667	0.245
0.2	0.05	yadI	3	7.41667	0.393
0.2	0.05	AG1	3	7.41667	0.279
0.2	0.05	ptsI	3	7.66667	0.278
0.2	0.05	clcB	3	7.66667	0.267
0.2	0.05	ycaM	3	7.66667	0.249
0.2	0.05	yadI	3	7.66667	0.405
0.2	0.05	AG1	3	7.66667	0.287
0.2	0.05	ptsI	3	7.91667	0.287
0.2	0.05	clcB	3	7.91667	0.272
0.2	0.05	ycaM	3	7.91667	0.249
0.2	0.05	yadI	3	7.91667	0.416
0.2	0.05	AG1	3	7.91667	0.295
0.2	0.05	ptsI	3	8.16667	0.3
0.2	0.05	clcB	3	8.16667	0.28
0.2	0.05	ycaM	3	8.16667	0.252
0.2	0.05	yadI	3	8.16667	0.426
0.2	0.05	AG1	3	8.16667	0.302
0.2	0.05	ptsI	3	8.41667	0.31

0.2	0.05	clcB	3	8.41667	0.285
0.2	0.05	ycaM	3	8.41667	0.254
0.2	0.05	yadI	3	8.41667	0.437
0.2	0.05	AG1	3	8.41667	0.31
0.2	0.05	ptsI	3	9.05	0.29
0.2	0.05	clcB	3	9.05	0.273
0.2	0.05	ycaM	3	9.05	0.252
0.2	0.05	yadI	3	9.05	0.443
0.2	0.05	AG1	3	9.05	0.372
0.2	0.05	ptsI	3	9.3	0.294
0.2	0.05	clcB	3	9.3	0.275
0.2	0.05	ycaM	3	9.3	0.249
0.2	0.05	yadI	3	9.3	0.45
0.2	0.05	AG1	3	9.3	0.382
0.2	0.05	ptsI	3	9.55	0.302
0.2	0.05	clcB	3	9.55	0.282
0.2	0.05	ycaM	3	9.55	0.252
0.2	0.05	yadI	3	9.55	0.467
0.2	0.05	AG1	3	9.55	0.394
0.2	0.05	ptsI	3	9.8	0.31
0.2	0.05	clcB	3	9.8	0.289
0.2	0.05	ycaM	3	9.8	0.254
0.2	0.05	yadI	3	9.8	0.48
0.2	0.05	AG1	3	9.8	0.406
0.2	0.05	ptsI	3	10.05	0.32
0.2	0.05	clcB	3	10.05	0.296
0.2	0.05	ycaM	3	10.05	0.256
0.2	0.05	yadI	3	10.05	0.494
0.2	0.05	AG1	3	10.05	0.418
0.2	0.05	ptsI	3	10.3	0.325
0.2	0.05	clcB	3	10.3	0.299
0.2	0.05	ycaM	3	10.3	0.256
0.2	0.05	yadI	3	10.3	0.507
0.2	0.05	AG1	3	10.3	0.429
0.2	0.05	ptsI	3	10.55	0.336
0.2	0.05	clcB	3	10.55	0.304
0.2	0.05	ycaM	3	10.55	0.257
0.2	0.05	yadI	3	10.55	0.518
0.2	0.05	AG1	3	10.55	0.439
0.2	0.05	ptsI	3	10.8	0.343
0.2	0.05	clcB	3	10.8	0.301
0.2	0.05	ycaM	3	10.8	0.259
0.2	0.05	yadI	3	10.8	0.53
0.2	0.05	AG1	3	10.8	0.446
0.2	0.05	ptsI	3	11.05	0.355
0.2	0.05	clcB	3	11.05	0.309
0.2	0.05	ycaM	3	11.05	0.262
0.2	0.05	yadI	3	11.05	0.544
0.2	0.05	AG1	3	11.05	0.458
0.2	0.05	ptsI	3	11.3	0.362
0.2	0.05	clcB	3	11.3	0.307
0.2	0.05	ycaM	3	11.3	0.263
0.2	0.05	yadI	3	11.3	0.553

0.2	0.05	AG1	3	11.3	0.468
0.2	0.05	ptsI	3	11.55	0.373
0.2	0.05	clcB	3	11.55	0.308
0.2	0.05	ycaM	3	11.55	0.262
0.2	0.05	yadI	3	11.55	0.566
0.2	0.05	AG1	3	11.55	0.479
0.2	0.05	ptsI	3	11.8	0.383
0.2	0.05	clcB	3	11.8	0.317
0.2	0.05	ycaM	3	11.8	0.266
0.2	0.05	yadI	3	11.8	0.578
0.2	0.05	AG1	3	11.8	0.493
0.2	0.05	ptsI	3	12.05	0.394
0.2	0.05	clcB	3	12.05	0.31
0.2	0.05	ycaM	3	12.05	0.261
0.2	0.05	yadI	3	12.05	0.589
0.2	0.05	AG1	3	12.05	0.504
0.2	0.05	ptsI	3	12.3	0.405
0.2	0.05	clcB	3	12.3	0.315
0.2	0.05	ycaM	3	12.3	0.27
0.2	0.05	yadI	3	12.3	0.602
0.2	0.05	AG1	3	12.3	0.519
0.2	0.05	ptsI	3	12.55	0.413
0.2	0.05	clcB	3	12.55	0.313
0.2	0.05	ycaM	3	12.55	0.268
0.2	0.05	yadI	3	12.55	0.615
0.2	0.05	AG1	3	12.55	0.538
0.2	0.05	ptsI	3	12.8	0.423
0.2	0.05	clcB	3	12.8	0.316
0.2	0.05	ycaM	3	12.8	0.269
0.2	0.05	yadI	3	12.8	0.626
0.2	0.05	AG1	3	12.8	0.557
0.2	0.05	ptsI	3	13.05	0.43
0.2	0.05	clcB	3	13.05	0.302
0.2	0.05	ycaM	3	13.05	0.264
0.2	0.05	yadI	3	13.05	0.633
0.2	0.05	AG1	3	13.05	0.565
0.2	0.05	ptsI	3	13.3	0.438
0.2	0.05	clcB	3	13.3	0.301
0.2	0.05	ycaM	3	13.3	0.266
0.2	0.05	yadI	3	13.3	0.644
0.2	0.05	AG1	3	13.3	0.583
0.2	0.05	ptsI	3	13.55	0.45
0.2	0.05	clcB	3	13.55	0.309
0.2	0.05	ycaM	3	13.55	0.268
0.2	0.05	yadI	3	13.55	0.659
0.2	0.05	AG1	3	13.55	0.601
0.2	0.05	ptsI	3	13.8	0.461
0.2	0.05	clcB	3	13.8	0.304
0.2	0.05	ycaM	3	13.8	0.269
0.2	0.05	yadI	3	13.8	0.665
0.2	0.05	AG1	3	13.8	0.611
0.2	0.05	ptsI	3	14.05	0.465
0.2	0.05	clcB	3	14.05	0.316

0.2	0.05	ycaM	3	14.05	0.27
0.2	0.05	yadI	3	14.05	0.672
0.2	0.05	AG1	3	14.05	0.62
0.2	0.05	ptsI	3	14.3	0.469
0.2	0.05	clcB	3	14.3	0.299
0.2	0.05	ycaM	3	14.3	0.265
0.2	0.05	yadI	3	14.3	0.679
0.2	0.05	AG1	3	14.3	0.63
0.2	0.05	ptsI	3	14.55	0.473
0.2	0.05	clcB	3	14.55	0.298
0.2	0.05	ycaM	3	14.55	0.262
0.2	0.05	yadI	3	14.55	0.688
0.2	0.05	AG1	3	14.55	0.642
0.2	0.05	ptsI	3	14.8	0.489
0.2	0.05	clcB	3	14.8	0.295
0.2	0.05	ycaM	3	14.8	0.265
0.2	0.05	yadI	3	14.8	0.703
0.2	0.05	AG1	3	14.8	0.656
0.2	0.05	ptsI	3	15.05	0.503
0.2	0.05	clcB	3	15.05	0.295
0.2	0.05	ycaM	3	15.05	0.266
0.2	0.05	yadI	3	15.05	0.717
0.2	0.05	AG1	3	15.05	0.663
0.2	0.05	ptsI	3	15.3	0.501
0.2	0.05	clcB	3	15.3	0.296
0.2	0.05	ycaM	3	15.3	0.262
0.2	0.05	yadI	3	15.3	0.717
0.2	0.05	AG1	3	15.3	0.682
0.2	0.05	ptsI	3	15.55	0.506
0.2	0.05	clcB	3	15.55	0.298
0.2	0.05	ycaM	3	15.55	0.261
0.2	0.05	yadI	3	15.55	0.72
0.2	0.05	AG1	3	15.55	0.686
0.2	0.05	ptsI	3	15.8	0.512
0.2	0.05	clcB	3	15.8	0.298
0.2	0.05	ycaM	3	15.8	0.258
0.2	0.05	yadI	3	15.8	0.73
0.2	0.05	AG1	3	15.8	0.697
0.2	0.05	ptsI	3	16.05	0.519
0.2	0.05	clcB	3	16.05	0.298
0.2	0.05	ycaM	3	16.05	0.257
0.2	0.05	yadI	3	16.05	0.733
0.2	0.05	AG1	3	16.05	0.693
0.2	0.05	ptsI	3	16.3	0.522
0.2	0.05	clcB	3	16.3	0.302
0.2	0.05	ycaM	3	16.3	0.255
0.2	0.05	yadI	3	16.3	0.735
0.2	0.05	AG1	3	16.3	0.696
0.2	0.05	ptsI	3	16.55	0.534
0.2	0.05	clcB	3	16.55	0.306
0.2	0.05	ycaM	3	16.55	0.259
0.2	0.05	yadI	3	16.55	0.748
0.2	0.05	AG1	3	16.55	0.711

0.2	0.05	ptsI	3	16.8	0.542
0.2	0.05	clcB	3	16.8	0.303
0.2	0.05	ycaM	3	16.8	0.256
0.2	0.05	yadI	3	16.8	0.759
0.2	0.05	AG1	3	16.8	0.734
0.2	0.05	ptsI	3	17.05	0.544
0.2	0.05	clcB	3	17.05	0.305
0.2	0.05	ycaM	3	17.05	0.256
0.2	0.05	yadI	3	17.05	0.762
0.2	0.05	AG1	3	17.05	0.727
0.2	0.05	ptsI	3	17.3	0.544
0.2	0.05	clcB	3	17.3	0.304
0.2	0.05	ycaM	3	17.3	0.257
0.2	0.05	yadI	3	17.3	0.77
0.2	0.05	AG1	3	17.3	0.733
0.2	0.05	ptsI	3	17.55	0.554
0.2	0.05	clcB	3	17.55	0.303
0.2	0.05	ycaM	3	17.55	0.256
0.2	0.05	yadI	3	17.55	0.785
0.2	0.05	AG1	3	17.55	0.743
0.2	0.05	ptsI	3	17.8	0.559
0.2	0.05	clcB	3	17.8	0.302
0.2	0.05	ycaM	3	17.8	0.259
0.2	0.05	yadI	3	17.8	0.79
0.2	0.05	AG1	3	17.8	0.753
0.2	0.05	ptsI	3	18.05	0.56
0.2	0.05	clcB	3	18.05	0.3
0.2	0.05	ycaM	3	18.05	0.26
0.2	0.05	yadI	3	18.05	0.791
0.2	0.05	AG1	3	18.05	0.77
0.2	0.05	ptsI	3	18.3	0.562
0.2	0.05	clcB	3	18.3	0.3
0.2	0.05	ycaM	3	18.3	0.26
0.2	0.05	yadI	3	18.3	0.796
0.2	0.05	AG1	3	18.3	0.766
0.2	0.05	ptsI	3	18.55	0.563
0.2	0.05	clcB	3	18.55	0.3
0.2	0.05	ycaM	3	18.55	0.262
0.2	0.05	yadI	3	18.55	0.798
0.2	0.05	AG1	3	18.55	0.757
0.2	0.05	ptsI	3	18.8	0.565
0.2	0.05	clcB	3	18.8	0.298
0.2	0.05	ycaM	3	18.8	0.263
0.2	0.05	yadI	3	18.8	0.795
0.2	0.05	AG1	3	18.8	0.76
0.2	0.05	ptsI	3	19.05	0.566
0.2	0.05	clcB	3	19.05	0.297
0.2	0.05	ycaM	3	19.05	0.264
0.2	0.05	yadI	3	19.05	0.802
0.2	0.05	AG1	3	19.05	0.774
0.2	0.05	ptsI	3	19.3	0.567
0.2	0.05	clcB	3	19.3	0.298
0.2	0.05	ycaM	3	19.3	0.264

0.2	0.05	yadI	3	19.3	0.803
0.2	0.05	AG1	3	19.3	0.77
0.2	0.05	ptsI	3	19.55	0.566
0.2	0.05	clcB	3	19.55	0.299
0.2	0.05	ycaM	3	19.55	0.265
0.2	0.05	yadI	3	19.55	0.81
0.2	0.05	AG1	3	19.55	0.778
0.2	0.05	ptsI	3	19.8	0.568
0.2	0.05	clcB	3	19.8	0.299
0.2	0.05	ycaM	3	19.8	0.266
0.2	0.05	yadI	3	19.8	0.806
0.2	0.05	AG1	3	19.8	0.768
0.2	0.05	ptsI	3	20.05	0.569
0.2	0.05	clcB	3	20.05	0.299
0.2	0.05	ycaM	3	20.05	0.268
0.2	0.05	yadI	3	20.05	0.814
0.2	0.05	AG1	3	20.05	0.76
0.2	0.05	ptsI	3	20.3	0.568
0.2	0.05	clcB	3	20.3	0.298
0.2	0.05	ycaM	3	20.3	0.268
0.2	0.05	yadI	3	20.3	0.812
0.2	0.05	AG1	3	20.3	0.754
0.2	0.05	ptsI	3	20.55	0.563
0.2	0.05	clcB	3	20.55	0.299
0.2	0.05	ycaM	3	20.55	0.269
0.2	0.05	yadI	3	20.55	0.81
0.2	0.05	AG1	3	20.55	0.768
0.2	0.05	ptsI	3	20.8	0.566
0.2	0.05	clcB	3	20.8	0.298
0.2	0.05	ycaM	3	20.8	0.27
0.2	0.05	yadI	3	20.8	0.817
0.2	0.05	AG1	3	20.8	0.777
0.2	0.05	ptsI	3	21.05	0.563
0.2	0.05	clcB	3	21.05	0.298
0.2	0.05	ycaM	3	21.05	0.271
0.2	0.05	yadI	3	21.05	0.813
0.2	0.05	AG1	3	21.05	0.779
0.2	0.05	ptsI	3	21.3	0.561
0.2	0.05	clcB	3	21.3	0.298
0.2	0.05	ycaM	3	21.3	0.274
0.2	0.05	yadI	3	21.3	0.818
0.2	0.05	AG1	3	21.3	0.784
0.2	0.05	ptsI	3	21.55	0.561
0.2	0.05	clcB	3	21.55	0.3
0.2	0.05	ycaM	3	21.55	0.276
0.2	0.05	yadI	3	21.55	0.817
0.2	0.05	AG1	3	21.55	0.796
0.2	0.05	ptsI	3	21.8	0.561
0.2	0.05	clcB	3	21.8	0.299
0.2	0.05	ycaM	3	21.8	0.278
0.2	0.05	yadI	3	21.8	0.819
0.2	0.05	AG1	3	21.8	0.804
0.2	0.05	ptsI	3	22.05	0.562

0.2	0.05	clcB	3	22.05	0.298
0.2	0.05	ycaM	3	22.05	0.278
0.2	0.05	yadI	3	22.05	0.822
0.2	0.05	AG1	3	22.05	0.806
0.2	0.05	ptsI	3	22.3	0.558
0.2	0.05	clcB	3	22.3	0.298
0.2	0.05	ycaM	3	22.3	0.283
0.2	0.05	yadI	3	22.3	0.822
0.2	0.05	AG1	3	22.3	0.814
0.2	0.05	ptsI	3	22.55	0.562
0.2	0.05	clcB	3	22.55	0.302
0.2	0.05	ycaM	3	22.55	0.284
0.2	0.05	yadI	3	22.55	0.828
0.2	0.05	AG1	3	22.55	0.819
0.2	0.05	ptsI	3	22.8	0.564
0.2	0.05	clcB	3	22.8	0.302
0.2	0.05	ycaM	3	22.8	0.286
0.2	0.05	yadI	3	22.8	0.831
0.2	0.05	AG1	3	22.8	0.829
0.2	0.05	ptsI	3	23.05	0.566
0.2	0.05	clcB	3	23.05	0.302
0.2	0.05	ycaM	3	23.05	0.291
0.2	0.05	yadI	3	23.05	0.833
0.2	0.05	AG1	3	23.05	0.834
0.2	0.05	ptsI	3	23.3	0.565
0.2	0.05	clcB	3	23.3	0.301
0.2	0.05	ycaM	3	23.3	0.293
0.2	0.05	yadI	3	23.3	0.829
0.2	0.05	AG1	3	23.3	0.839
0.2	0.05	ptsI	3	23.55	0.569
0.2	0.05	clcB	3	23.55	0.306
0.2	0.05	ycaM	3	23.55	0.297
0.2	0.05	yadI	3	23.55	0.834
0.2	0.05	AG1	3	23.55	0.851
0.2	0.05	ptsI	3	23.8	0.564
0.2	0.05	clcB	3	23.8	0.307
0.2	0.05	ycaM	3	23.8	0.3
0.2	0.05	yadI	3	23.8	0.829
0.2	0.05	AG1	3	23.8	0.859
0.2	0.05	ptsI	3	24.05	0.566
0.2	0.05	clcB	3	24.05	0.309
0.2	0.05	ycaM	3	24.05	0.305
0.2	0.05	yadI	3	24.05	0.829
0.2	0.05	AG1	3	24.05	0.87
0.2	0.05	ptsI	3	24.3	0.566
0.2	0.05	clcB	3	24.3	0.308
0.2	0.05	ycaM	3	24.3	0.307
0.2	0.05	yadI	3	24.3	0.824
0.2	0.05	AG1	3	24.3	0.874
0.2	0.05	ptsI	4	0	0.212
0.2	0.05	clcB	4	0	0.205
0.2	0.05	ycaM	4	0	0.204
0.2	0.05	yadI	4	0	0.215

0.2	0.05	AG1	4	0	0.205
0.2	0.05	ptsI	4	0.25	0.205
0.2	0.05	clcB	4	0.25	0.198
0.2	0.05	ycaM	4	0.25	0.199
0.2	0.05	yadI	4	0.25	0.217
0.2	0.05	AG1	4	0.25	0.204
0.2	0.05	ptsI	4	0.5	0.203
0.2	0.05	clcB	4	0.5	0.197
0.2	0.05	ycaM	4	0.5	0.2
0.2	0.05	yadI	4	0.5	0.217
0.2	0.05	AG1	4	0.5	0.204
0.2	0.05	ptsI	4	0.75	0.205
0.2	0.05	clcB	4	0.75	0.2
0.2	0.05	ycaM	4	0.75	0.2
0.2	0.05	yadI	4	0.75	0.217
0.2	0.05	AG1	4	0.75	0.206
0.2	0.05	ptsI	4	1	0.207
0.2	0.05	clcB	4	1	0.204
0.2	0.05	ycaM	4	1	0.204
0.2	0.05	yadI	4	1	0.218
0.2	0.05	AG1	4	1	0.21
0.2	0.05	ptsI	4	1.25	0.212
0.2	0.05	clcB	4	1.25	0.209
0.2	0.05	ycaM	4	1.25	0.208
0.2	0.05	yadI	4	1.25	0.224
0.2	0.05	AG1	4	1.25	0.214
0.2	0.05	ptsI	4	1.5	0.221
0.2	0.05	clcB	4	1.5	0.212
0.2	0.05	ycaM	4	1.5	0.214
0.2	0.05	yadI	4	1.5	0.226
0.2	0.05	AG1	4	1.5	0.213
0.2	0.05	ptsI	4	1.75	0.225
0.2	0.05	clcB	4	1.75	0.218
0.2	0.05	ycaM	4	1.75	0.221
0.2	0.05	yadI	4	1.75	0.233
0.2	0.05	AG1	4	1.75	0.221
0.2	0.05	ptsI	4	2	0.236
0.2	0.05	clcB	4	2	0.229
0.2	0.05	ycaM	4	2	0.23
0.2	0.05	yadI	4	2	0.242
0.2	0.05	AG1	4	2	0.228
0.2	0.05	ptsI	4	2.25	0.245
0.2	0.05	clcB	4	2.25	0.236
0.2	0.05	ycaM	4	2.25	0.238
0.2	0.05	yadI	4	2.25	0.254
0.2	0.05	AG1	4	2.25	0.238
0.2	0.05	ptsI	4	2.5	0.254
0.2	0.05	clcB	4	2.5	0.24
0.2	0.05	ycaM	4	2.5	0.244
0.2	0.05	yadI	4	2.5	0.257
0.2	0.05	AG1	4	2.5	0.241
0.2	0.05	ptsI	4	2.75	0.264
0.2	0.05	clcB	4	2.75	0.252

0.2	0.05	ycaM	4	2.75	0.253
0.2	0.05	yadI	4	2.75	0.267
0.2	0.05	AG1	4	2.75	0.25
0.2	0.05	ptsI	4	3	0.272
0.2	0.05	clcB	4	3	0.257
0.2	0.05	ycaM	4	3	0.26
0.2	0.05	yadI	4	3	0.276
0.2	0.05	AG1	4	3	0.26
0.2	0.05	ptsI	4	3.25	0.282
0.2	0.05	clcB	4	3.25	0.268
0.2	0.05	ycaM	4	3.25	0.271
0.2	0.05	yadI	4	3.25	0.285
0.2	0.05	AG1	4	3.25	0.268
0.2	0.05	ptsI	4	3.5	0.298
0.2	0.05	clcB	4	3.5	0.283
0.2	0.05	ycaM	4	3.5	0.279
0.2	0.05	yadI	4	3.5	0.296
0.2	0.05	AG1	4	3.5	0.282
0.2	0.05	ptsI	4	3.75	0.31
0.2	0.05	clcB	4	3.75	0.296
0.2	0.05	ycaM	4	3.75	0.289
0.2	0.05	yadI	4	3.75	0.304
0.2	0.05	AG1	4	3.75	0.289
0.2	0.05	ptsI	4	4	0.328
0.2	0.05	clcB	4	4	0.309
0.2	0.05	ycaM	4	4	0.297
0.2	0.05	yadI	4	4	0.318
0.2	0.05	AG1	4	4	0.307
0.2	0.05	ptsI	4	4.35	0.331
0.2	0.05	clcB	4	4.35	0.406
0.2	0.05	ycaM	4	4.35	0.378
0.2	0.05	yadI	4	4.35	0.323
0.2	0.05	AG1	4	4.35	0.339
0.2	0.05	ptsI	4	4.6	0.31
0.2	0.05	clcB	4	4.6	0.388
0.2	0.05	ycaM	4	4.6	0.358
0.2	0.05	yadI	4	4.6	0.33
0.2	0.05	AG1	4	4.6	0.35
0.2	0.05	ptsI	4	4.85	0.314
0.2	0.05	clcB	4	4.85	0.39
0.2	0.05	ycaM	4	4.85	0.354
0.2	0.05	yadI	4	4.85	0.346
0.2	0.05	AG1	4	4.85	0.363
0.2	0.05	ptsI	4	5.1	0.323
0.2	0.05	clcB	4	5.1	0.392
0.2	0.05	ycaM	4	5.1	0.357
0.2	0.05	yadI	4	5.1	0.359
0.2	0.05	AG1	4	5.1	0.373
0.2	0.05	ptsI	4	5.35	0.338
0.2	0.05	clcB	4	5.35	0.396
0.2	0.05	ycaM	4	5.35	0.36
0.2	0.05	yadI	4	5.35	0.374
0.2	0.05	AG1	4	5.35	0.39

0.2	0.05	ptsI	4	5.6	0.347
0.2	0.05	clcB	4	5.6	0.392
0.2	0.05	ycaM	4	5.6	0.36
0.2	0.05	yadI	4	5.6	0.384
0.2	0.05	AG1	4	5.6	0.408
0.2	0.05	ptsI	4	5.85	0.358
0.2	0.05	clcB	4	5.85	0.394
0.2	0.05	ycaM	4	5.85	0.359
0.2	0.05	yadI	4	5.85	0.395
0.2	0.05	AG1	4	5.85	0.413
0.2	0.05	ptsI	4	6.1	0.376
0.2	0.05	clcB	4	6.1	0.399
0.2	0.05	ycaM	4	6.1	0.359
0.2	0.05	yadI	4	6.1	0.408
0.2	0.05	AG1	4	6.1	0.423
0.2	0.05	ptsI	4	6.35	0.39
0.2	0.05	clcB	4	6.35	0.402
0.2	0.05	ycaM	4	6.35	0.361
0.2	0.05	yadI	4	6.35	0.421
0.2	0.05	AG1	4	6.35	0.435
0.2	0.05	ptsI	4	6.6	0.404
0.2	0.05	clcB	4	6.6	0.406
0.2	0.05	ycaM	4	6.6	0.36
0.2	0.05	yadI	4	6.6	0.426
0.2	0.05	AG1	4	6.6	0.447
0.2	0.05	ptsI	4	6.85	0.414
0.2	0.05	clcB	4	6.85	0.407
0.2	0.05	ycaM	4	6.85	0.364
0.2	0.05	yadI	4	6.85	0.435
0.2	0.05	AG1	4	6.85	0.46
0.2	0.05	ptsI	4	7.1	0.42
0.2	0.05	clcB	4	7.1	0.408
0.2	0.05	ycaM	4	7.1	0.364
0.2	0.05	yadI	4	7.1	0.444
0.2	0.05	AG1	4	7.1	0.468
0.2	0.05	ptsI	4	7.35	0.431
0.2	0.05	clcB	4	7.35	0.412
0.2	0.05	ycaM	4	7.35	0.373
0.2	0.05	yadI	4	7.35	0.453
0.2	0.05	AG1	4	7.35	0.481
0.2	0.05	ptsI	4	7.6	0.441
0.2	0.05	clcB	4	7.6	0.414
0.2	0.05	ycaM	4	7.6	0.38
0.2	0.05	yadI	4	7.6	0.463
0.2	0.05	AG1	4	7.6	0.49
0.2	0.05	ptsI	4	7.85	0.446
0.2	0.05	clcB	4	7.85	0.407
0.2	0.05	ycaM	4	7.85	0.356
0.2	0.05	yadI	4	7.85	0.47
0.2	0.05	AG1	4	7.85	0.497
0.2	0.05	ptsI	4	8.1	0.458
0.2	0.05	clcB	4	8.1	0.412
0.2	0.05	ycaM	4	8.1	0.364

0.2	0.05	yadI	4	8.1	0.479
0.2	0.05	AG1	4	8.1	0.503
0.2	0.05	ptsI	4	8.35	0.468
0.2	0.05	clcB	4	8.35	0.416
0.2	0.05	ycaM	4	8.35	0.376
0.2	0.05	yadI	4	8.35	0.488
0.2	0.05	AG1	4	8.35	0.516
0.2	0.05	ptsI	4	8.83333	0.431
0.2	0.05	clcB	4	8.83333	0.306
0.2	0.05	ycaM	4	8.83333	0.282
0.2	0.05	yadI	4	8.83333	0.512
0.2	0.05	AG1	4	8.83333	0.56
0.2	0.05	ptsI	4	9.08333	0.436
0.2	0.05	clcB	4	9.08333	0.307
0.2	0.05	ycaM	4	9.08333	0.286
0.2	0.05	yadI	4	9.08333	0.508
0.2	0.05	AG1	4	9.08333	0.563
0.2	0.05	ptsI	4	9.33333	0.443
0.2	0.05	clcB	4	9.33333	0.303
0.2	0.05	ycaM	4	9.33333	0.28
0.2	0.05	yadI	4	9.33333	0.518
0.2	0.05	AG1	4	9.33333	0.579
0.2	0.05	ptsI	4	9.58333	0.451
0.2	0.05	clcB	4	9.58333	0.302
0.2	0.05	ycaM	4	9.58333	0.284
0.2	0.05	yadI	4	9.58333	0.529
0.2	0.05	AG1	4	9.58333	0.592
0.2	0.05	ptsI	4	9.83333	0.463
0.2	0.05	clcB	4	9.83333	0.302
0.2	0.05	ycaM	4	9.83333	0.282
0.2	0.05	yadI	4	9.83333	0.539
0.2	0.05	AG1	4	9.83333	0.601
0.2	0.05	ptsI	4	10.0833	0.471
0.2	0.05	clcB	4	10.0833	0.301
0.2	0.05	ycaM	4	10.0833	0.282
0.2	0.05	yadI	4	10.0833	0.543
0.2	0.05	AG1	4	10.0833	0.607
0.2	0.05	ptsI	4	10.3333	0.481
0.2	0.05	clcB	4	10.3333	0.304
0.2	0.05	ycaM	4	10.3333	0.282
0.2	0.05	yadI	4	10.3333	0.551
0.2	0.05	AG1	4	10.3333	0.619
0.2	0.05	ptsI	4	10.5833	0.492
0.2	0.05	clcB	4	10.5833	0.302
0.2	0.05	ycaM	4	10.5833	0.284
0.2	0.05	yadI	4	10.5833	0.56
0.2	0.05	AG1	4	10.5833	0.626
0.2	0.05	ptsI	4	10.8333	0.501
0.2	0.05	clcB	4	10.8333	0.297
0.2	0.05	ycaM	4	10.8333	0.285
0.2	0.05	yadI	4	10.8333	0.566
0.2	0.05	AG1	4	10.8333	0.636
0.2	0.05	ptsI	4	11.0833	0.508

0.2	0.05	clcB	4	11.0833	0.299
0.2	0.05	ycaM	4	11.0833	0.281
0.2	0.05	yadI	4	11.0833	0.57
0.2	0.05	AG1	4	11.0833	0.641
0.2	0.05	ptsI	4	11.3333	0.518
0.2	0.05	clcB	4	11.3333	0.303
0.2	0.05	ycaM	4	11.3333	0.288
0.2	0.05	yadI	4	11.3333	0.58
0.2	0.05	AG1	4	11.3333	0.651
0.2	0.05	ptsI	4	11.5833	0.524
0.2	0.05	clcB	4	11.5833	0.298
0.2	0.05	ycaM	4	11.5833	0.281
0.2	0.05	yadI	4	11.5833	0.587
0.2	0.05	AG1	4	11.5833	0.656
0.2	0.05	ptsI	4	11.8333	0.534
0.2	0.05	clcB	4	11.8333	0.292
0.2	0.05	ycaM	4	11.8333	0.29
0.2	0.05	yadI	4	11.8333	0.589
0.2	0.05	AG1	4	11.8333	0.658
0.2	0.05	ptsI	4	12.0833	0.539
0.2	0.05	clcB	4	12.0833	0.299
0.2	0.05	ycaM	4	12.0833	0.288
0.2	0.05	yadI	4	12.0833	0.595
0.2	0.05	AG1	4	12.0833	0.665
0.2	0.05	ptsI	4	12.3333	0.549
0.2	0.05	clcB	4	12.3333	0.304
0.2	0.05	ycaM	4	12.3333	0.286
0.2	0.05	yadI	4	12.3333	0.601
0.2	0.05	AG1	4	12.3333	0.672
0.2	0.05	ptsI	4	12.5833	0.555
0.2	0.05	clcB	4	12.5833	0.307
0.2	0.05	ycaM	4	12.5833	0.287
0.2	0.05	yadI	4	12.5833	0.605
0.2	0.05	AG1	4	12.5833	0.678
0.2	0.05	ptsI	4	12.8333	0.56
0.2	0.05	clcB	4	12.8333	0.299
0.2	0.05	ycaM	4	12.8333	0.286
0.2	0.05	yadI	4	12.8333	0.612
0.2	0.05	AG1	4	12.8333	0.683
0.2	0.05	ptsI	4	13.0833	0.569
0.2	0.05	clcB	4	13.0833	0.303
0.2	0.05	ycaM	4	13.0833	0.291
0.2	0.05	yadI	4	13.0833	0.616
0.2	0.05	AG1	4	13.0833	0.69
0.2	0.05	ptsI	4	13.3333	0.579
0.2	0.05	clcB	4	13.3333	0.302
0.2	0.05	ycaM	4	13.3333	0.29
0.2	0.05	yadI	4	13.3333	0.619
0.2	0.05	AG1	4	13.3333	0.696
0.2	0.05	ptsI	4	13.5833	0.585
0.2	0.05	clcB	4	13.5833	0.306
0.2	0.05	ycaM	4	13.5833	0.284
0.2	0.05	yadI	4	13.5833	0.624

0.2	0.05	AG1	4	13.5833	0.699
0.2	0.05	ptsI	4	13.8333	0.592
0.2	0.05	clcB	4	13.8333	0.311
0.2	0.05	ycaM	4	13.8333	0.29
0.2	0.05	yadI	4	13.8333	0.627
0.2	0.05	AG1	4	13.8333	0.7
0.2	0.05	ptsI	4	14.0833	0.6
0.2	0.05	clcB	4	14.0833	0.311
0.2	0.05	ycaM	4	14.0833	0.29
0.2	0.05	yadI	4	14.0833	0.634
0.2	0.05	AG1	4	14.0833	0.704
0.2	0.05	ptsI	4	14.3333	0.606
0.2	0.05	clcB	4	14.3333	0.309
0.2	0.05	ycaM	4	14.3333	0.292
0.2	0.05	yadI	4	14.3333	0.632
0.2	0.05	AG1	4	14.3333	0.708
0.2	0.05	ptsI	4	14.5833	0.609
0.2	0.05	clcB	4	14.5833	0.311
0.2	0.05	ycaM	4	14.5833	0.302
0.2	0.05	yadI	4	14.5833	0.638
0.2	0.05	AG1	4	14.5833	0.711
0.2	0.05	ptsI	4	14.8333	0.614
0.2	0.05	clcB	4	14.8333	0.311
0.2	0.05	ycaM	4	14.8333	0.292
0.2	0.05	yadI	4	14.8333	0.644
0.2	0.05	AG1	4	14.8333	0.718
0.2	0.05	ptsI	4	15.0833	0.622
0.2	0.05	clcB	4	15.0833	0.308
0.2	0.05	ycaM	4	15.0833	0.292
0.2	0.05	yadI	4	15.0833	0.645
0.2	0.05	AG1	4	15.0833	0.72
0.2	0.05	ptsI	4	15.3333	0.626
0.2	0.05	clcB	4	15.3333	0.312
0.2	0.05	ycaM	4	15.3333	0.295
0.2	0.05	yadI	4	15.3333	0.647
0.2	0.05	AG1	4	15.3333	0.722
0.2	0.05	ptsI	4	15.5833	0.632
0.2	0.05	clcB	4	15.5833	0.313
0.2	0.05	ycaM	4	15.5833	0.298
0.2	0.05	yadI	4	15.5833	0.65
0.2	0.05	AG1	4	15.5833	0.727
0.2	0.05	ptsI	4	15.8333	0.632
0.2	0.05	clcB	4	15.8333	0.316
0.2	0.05	ycaM	4	15.8333	0.299
0.2	0.05	yadI	4	15.8333	0.658
0.2	0.05	AG1	4	15.8333	0.73
0.2	0.05	ptsI	4	16.0833	0.638
0.2	0.05	clcB	4	16.0833	0.314
0.2	0.05	ycaM	4	16.0833	0.299
0.2	0.05	yadI	4	16.0833	0.658
0.2	0.05	AG1	4	16.0833	0.734
0.2	0.05	ptsI	4	16.3333	0.636
0.2	0.05	clcB	4	16.3333	0.315

0.2	0.05	ycaM	4	16.3333	0.301
0.2	0.05	yadI	4	16.3333	0.66
0.2	0.05	AG1	4	16.3333	0.737
0.2	0.05	ptsI	4	16.5833	0.637
0.2	0.05	clcB	4	16.5833	0.314
0.2	0.05	ycaM	4	16.5833	0.301
0.2	0.05	yadI	4	16.5833	0.665
0.2	0.05	AG1	4	16.5833	0.744
0.2	0.05	ptsI	4	16.8333	0.645
0.2	0.05	clcB	4	16.8333	0.316
0.2	0.05	ycaM	4	16.8333	0.298
0.2	0.05	yadI	4	16.8333	0.67
0.2	0.05	AG1	4	16.8333	0.746
0.2	0.05	ptsI	4	17.0833	0.644
0.2	0.05	clcB	4	17.0833	0.317
0.2	0.05	ycaM	4	17.0833	0.303
0.2	0.05	yadI	4	17.0833	0.67
0.2	0.05	AG1	4	17.0833	0.746
0.2	0.05	ptsI	4	17.3333	0.644
0.2	0.05	clcB	4	17.3333	0.322
0.2	0.05	ycaM	4	17.3333	0.3
0.2	0.05	yadI	4	17.3333	0.672
0.2	0.05	AG1	4	17.3333	0.746
0.2	0.05	ptsI	4	17.5833	0.649
0.2	0.05	clcB	4	17.5833	0.319
0.2	0.05	ycaM	4	17.5833	0.305
0.2	0.05	yadI	4	17.5833	0.677
0.2	0.05	AG1	4	17.5833	0.752
0.2	0.05	ptsI	4	17.8333	0.645
0.2	0.05	clcB	4	17.8333	0.313
0.2	0.05	ycaM	4	17.8333	0.307
0.2	0.05	yadI	4	17.8333	0.679
0.2	0.05	AG1	4	17.8333	0.759
0.2	0.05	ptsI	4	18.0833	0.648
0.2	0.05	clcB	4	18.0833	0.314
0.2	0.05	ycaM	4	18.0833	0.305
0.2	0.05	yadI	4	18.0833	0.679
0.2	0.05	AG1	4	18.0833	0.758
0.2	0.05	ptsI	4	18.3333	0.653
0.2	0.05	clcB	4	18.3333	0.31
0.2	0.05	ycaM	4	18.3333	0.311
0.2	0.05	yadI	4	18.3333	0.683
0.2	0.05	AG1	4	18.3333	0.759
0.2	0.05	ptsI	4	18.5833	0.652
0.2	0.05	clcB	4	18.5833	0.309
0.2	0.05	ycaM	4	18.5833	0.308
0.2	0.05	yadI	4	18.5833	0.682
0.2	0.05	AG1	4	18.5833	0.76
0.2	0.05	ptsI	4	18.8333	0.652
0.2	0.05	clcB	4	18.8333	0.303
0.2	0.05	ycaM	4	18.8333	0.317
0.2	0.05	yadI	4	18.8333	0.686
0.2	0.05	AG1	4	18.8333	0.767

0.2	0.05	ptsI	4	19.0833	0.653
0.2	0.05	clcB	4	19.0833	0.304
0.2	0.05	ycaM	4	19.0833	0.315
0.2	0.05	yadI	4	19.0833	0.69
0.2	0.05	AG1	4	19.0833	0.765
0.2	0.05	ptsI	4	19.3333	0.65
0.2	0.05	clcB	4	19.3333	0.306
0.2	0.05	ycaM	4	19.3333	0.32
0.2	0.05	yadI	4	19.3333	0.685
0.2	0.05	AG1	4	19.3333	0.763
0.2	0.05	ptsI	4	19.5833	0.654
0.2	0.05	clcB	4	19.5833	0.307
0.2	0.05	ycaM	4	19.5833	0.322
0.2	0.05	yadI	4	19.5833	0.687
0.2	0.05	AG1	4	19.5833	0.764
0.2	0.05	ptsI	4	19.8333	0.654
0.2	0.05	clcB	4	19.8333	0.305
0.2	0.05	ycaM	4	19.8333	0.323
0.2	0.05	yadI	4	19.8333	0.686
0.2	0.05	AG1	4	19.8333	0.767
0.2	0.05	ptsI	4	20.0833	0.66
0.2	0.05	clcB	4	20.0833	0.304
0.2	0.05	ycaM	4	20.0833	0.326
0.2	0.05	yadI	4	20.0833	0.688
0.2	0.05	AG1	4	20.0833	0.772
0.2	0.05	ptsI	4	20.3333	0.654
0.2	0.05	clcB	4	20.3333	0.302
0.2	0.05	ycaM	4	20.3333	0.325
0.2	0.05	yadI	4	20.3333	0.69
0.2	0.05	AG1	4	20.3333	0.769
0.2	0.05	ptsI	4	20.5833	0.653
0.2	0.05	clcB	4	20.5833	0.3
0.2	0.05	ycaM	4	20.5833	0.328
0.2	0.05	yadI	4	20.5833	0.693
0.2	0.05	AG1	4	20.5833	0.778
0.2	0.05	ptsI	4	20.8333	0.662
0.2	0.05	clcB	4	20.8333	0.303
0.2	0.05	ycaM	4	20.8333	0.333
0.2	0.05	yadI	4	20.8333	0.692
0.2	0.05	AG1	4	20.8333	0.774
0.2	0.05	ptsI	4	21.0833	0.659
0.2	0.05	clcB	4	21.0833	0.301
0.2	0.05	ycaM	4	21.0833	0.332
0.2	0.05	yadI	4	21.0833	0.694
0.2	0.05	AG1	4	21.0833	0.777
0.2	0.05	ptsI	4	21.3333	0.664
0.2	0.05	clcB	4	21.3333	0.303
0.2	0.05	ycaM	4	21.3333	0.33
0.2	0.05	yadI	4	21.3333	0.692
0.2	0.05	AG1	4	21.3333	0.779
0.2	0.05	ptsI	4	21.5833	0.659
0.2	0.05	clcB	4	21.5833	0.304
0.2	0.05	ycaM	4	21.5833	0.328

0.2	0.05	yadI	4	21.5833	0.693
0.2	0.05	AG1	4	21.5833	0.782
0.2	0.05	ptsI	4	21.8333	0.66
0.2	0.05	clcB	4	21.8333	0.307
0.2	0.05	ycaM	4	21.8333	0.333
0.2	0.05	yadI	4	21.8333	0.694
0.2	0.05	AG1	4	21.8333	0.782
0.2	0.05	ptsI	4	22.0833	0.662
0.2	0.05	clcB	4	22.0833	0.308
0.2	0.05	ycaM	4	22.0833	0.33
0.2	0.05	yadI	4	22.0833	0.694
0.2	0.05	AG1	4	22.0833	0.786
0.2	0.05	ptsI	4	22.3333	0.657
0.2	0.05	clcB	4	22.3333	0.307
0.2	0.05	ycaM	4	22.3333	0.328
0.2	0.05	yadI	4	22.3333	0.695
0.2	0.05	AG1	4	22.3333	0.787
0.2	0.05	ptsI	4	22.5833	0.659
0.2	0.05	clcB	4	22.5833	0.304
0.2	0.05	ycaM	4	22.5833	0.328
0.2	0.05	yadI	4	22.5833	0.695
0.2	0.05	AG1	4	22.5833	0.789
0.2	0.05	ptsI	4	22.8333	0.663
0.2	0.05	clcB	4	22.8333	0.308
0.2	0.05	ycaM	4	22.8333	0.332
0.2	0.05	yadI	4	22.8333	0.699
0.2	0.05	AG1	4	22.8333	0.789
0.2	0.05	ptsI	4	23.0833	0.658
0.2	0.05	clcB	4	23.0833	0.312
0.2	0.05	ycaM	4	23.0833	0.329
0.2	0.05	yadI	4	23.0833	0.696
0.2	0.05	AG1	4	23.0833	0.792
0.2	0.05	ptsI	4	23.3333	0.663
0.2	0.05	clcB	4	23.3333	0.316
0.2	0.05	ycaM	4	23.3333	0.335
0.2	0.05	yadI	4	23.3333	0.7
0.2	0.05	AG1	4	23.3333	0.794
0.2	0.05	ptsI	4	23.5833	0.662
0.2	0.05	clcB	4	23.5833	0.315
0.2	0.05	ycaM	4	23.5833	0.334
0.2	0.05	yadI	4	23.5833	0.702
0.2	0.05	AG1	4	23.5833	0.795
0.2	0.05	ptsI	4	23.8333	0.659
0.2	0.05	clcB	4	23.8333	0.316
0.2	0.05	ycaM	4	23.8333	0.334
0.2	0.05	yadI	4	23.8333	0.701
0.2	0.05	AG1	4	23.8333	0.801
0.2	0.05	ptsI	4	24.0833	0.661
0.2	0.05	clcB	4	24.0833	0.318
0.2	0.05	ycaM	4	24.0833	0.34
0.2	0.05	yadI	4	24.0833	0.702
0.2	0.05	AG1	4	24.0833	0.801
0.2	0.05	ptsI	5	0	0.167

0.2	0.05	clcB	5	0	0.21
0.2	0.05	ycaM	5	0	0.256
0.2	0.05	yadI	5	0	0.186
0.2	0.05	AG1	5	0	0.169
0.2	0.05	ptsI	5	0.35	0.159
0.2	0.05	clcB	5	0.35	0.206
0.2	0.05	ycaM	5	0.35	0.257
0.2	0.05	yadI	5	0.35	0.182
0.2	0.05	AG1	5	0.35	0.167
0.2	0.05	ptsI	5	0.6	0.161
0.2	0.05	clcB	5	0.6	0.205
0.2	0.05	ycaM	5	0.6	0.26
0.2	0.05	yadI	5	0.6	0.183
0.2	0.05	AG1	5	0.6	0.167
0.2	0.05	ptsI	5	0.85	0.161
0.2	0.05	clcB	5	0.85	0.207
0.2	0.05	ycaM	5	0.85	0.265
0.2	0.05	yadI	5	0.85	0.183
0.2	0.05	AG1	5	0.85	0.168
0.2	0.05	ptsI	5	1.1	0.161
0.2	0.05	clcB	5	1.1	0.205
0.2	0.05	ycaM	5	1.1	0.267
0.2	0.05	yadI	5	1.1	0.186
0.2	0.05	AG1	5	1.1	0.169
0.2	0.05	ptsI	5	1.35	0.162
0.2	0.05	clcB	5	1.35	0.207
0.2	0.05	ycaM	5	1.35	0.267
0.2	0.05	yadI	5	1.35	0.186
0.2	0.05	AG1	5	1.35	0.171
0.2	0.05	ptsI	5	1.6	0.164
0.2	0.05	clcB	5	1.6	0.209
0.2	0.05	ycaM	5	1.6	0.275
0.2	0.05	yadI	5	1.6	0.189
0.2	0.05	AG1	5	1.6	0.172
0.2	0.05	ptsI	5	1.85	0.168
0.2	0.05	clcB	5	1.85	0.211
0.2	0.05	ycaM	5	1.85	0.284
0.2	0.05	yadI	5	1.85	0.191
0.2	0.05	AG1	5	1.85	0.175
0.2	0.05	ptsI	5	2.1	0.174
0.2	0.05	clcB	5	2.1	0.215
0.2	0.05	ycaM	5	2.1	0.29
0.2	0.05	yadI	5	2.1	0.195
0.2	0.05	AG1	5	2.1	0.179
0.2	0.05	ptsI	5	2.35	0.177
0.2	0.05	clcB	5	2.35	0.221
0.2	0.05	ycaM	5	2.35	0.299
0.2	0.05	yadI	5	2.35	0.2
0.2	0.05	AG1	5	2.35	0.182
0.2	0.05	ptsI	5	2.6	0.174
0.2	0.05	clcB	5	2.6	0.225
0.2	0.05	ycaM	5	2.6	0.263
0.2	0.05	yadI	5	2.6	0.207

0.2	0.05	AG1	5	2.6	0.187
0.2	0.05	ptsI	5	2.85	0.185
0.2	0.05	clcB	5	2.85	0.23
0.2	0.05	ycaM	5	2.85	0.316
0.2	0.05	yadI	5	2.85	0.208
0.2	0.05	AG1	5	2.85	0.19
0.2	0.05	ptsI	5	3.1	0.19
0.2	0.05	clcB	5	3.1	0.235
0.2	0.05	ycaM	5	3.1	0.321
0.2	0.05	yadI	5	3.1	0.215
0.2	0.05	AG1	5	3.1	0.193
0.2	0.05	ptsI	5	3.35	0.204
0.2	0.05	clcB	5	3.35	0.244
0.2	0.05	ycaM	5	3.35	0.335
0.2	0.05	yadI	5	3.35	0.223
0.2	0.05	AG1	5	3.35	0.202
0.2	0.05	ptsI	5	3.6	0.211
0.2	0.05	clcB	5	3.6	0.256
0.2	0.05	ycaM	5	3.6	0.347
0.2	0.05	yadI	5	3.6	0.23
0.2	0.05	AG1	5	3.6	0.21
0.2	0.05	ptsI	5	3.85	0.22
0.2	0.05	clcB	5	3.85	0.268
0.2	0.05	ycaM	5	3.85	0.353
0.2	0.05	yadI	5	3.85	0.241
0.2	0.05	AG1	5	3.85	0.221
0.2	0.05	ptsI	5	4.38333	0.232
0.2	0.05	clcB	5	4.38333	0.283
0.2	0.05	ycaM	5	4.38333	0.227
0.2	0.05	yadI	5	4.38333	0.282
0.2	0.05	AG1	5	4.38333	0.251
0.2	0.05	ptsI	5	4.63333	0.23
0.2	0.05	clcB	5	4.63333	0.273
0.2	0.05	ycaM	5	4.63333	0.23
0.2	0.05	yadI	5	4.63333	0.288
0.2	0.05	AG1	5	4.63333	0.261
0.2	0.05	ptsI	5	4.88333	0.239
0.2	0.05	clcB	5	4.88333	0.285
0.2	0.05	ycaM	5	4.88333	0.24
0.2	0.05	yadI	5	4.88333	0.305
0.2	0.05	AG1	5	4.88333	0.272
0.2	0.05	ptsI	5	5.13333	0.246
0.2	0.05	clcB	5	5.13333	0.296
0.2	0.05	ycaM	5	5.13333	0.245
0.2	0.05	yadI	5	5.13333	0.319
0.2	0.05	AG1	5	5.13333	0.287
0.2	0.05	ptsI	5	5.38333	0.256
0.2	0.05	clcB	5	5.38333	0.307
0.2	0.05	ycaM	5	5.38333	0.249
0.2	0.05	yadI	5	5.38333	0.334
0.2	0.05	AG1	5	5.38333	0.304
0.2	0.05	ptsI	5	5.63333	0.266
0.2	0.05	clcB	5	5.63333	0.317

0.2	0.05	ycaM	5	5.63333	0.249
0.2	0.05	yadI	5	5.63333	0.348
0.2	0.05	AG1	5	5.63333	0.317
0.2	0.05	ptsI	5	5.88333	0.276
0.2	0.05	clcB	5	5.88333	0.328
0.2	0.05	ycaM	5	5.88333	0.252
0.2	0.05	yadI	5	5.88333	0.363
0.2	0.05	AG1	5	5.88333	0.328
0.2	0.05	ptsI	5	6.13333	0.287
0.2	0.05	clcB	5	6.13333	0.338
0.2	0.05	ycaM	5	6.13333	0.256
0.2	0.05	yadI	5	6.13333	0.375
0.2	0.05	AG1	5	6.13333	0.344
0.2	0.05	ptsI	5	6.38333	0.299
0.2	0.05	clcB	5	6.38333	0.344
0.2	0.05	ycaM	5	6.38333	0.256
0.2	0.05	yadI	5	6.38333	0.386
0.2	0.05	AG1	5	6.38333	0.357
0.2	0.05	ptsI	5	6.63333	0.308
0.2	0.05	clcB	5	6.63333	0.351
0.2	0.05	ycaM	5	6.63333	0.258
0.2	0.05	yadI	5	6.63333	0.4
0.2	0.05	AG1	5	6.63333	0.371
0.2	0.05	ptsI	5	6.88333	0.32
0.2	0.05	clcB	5	6.88333	0.356
0.2	0.05	ycaM	5	6.88333	0.261
0.2	0.05	yadI	5	6.88333	0.414
0.2	0.05	AG1	5	6.88333	0.385
0.2	0.05	ptsI	5	7.13333	0.336
0.2	0.05	clcB	5	7.13333	0.363
0.2	0.05	ycaM	5	7.13333	0.262
0.2	0.05	yadI	5	7.13333	0.427
0.2	0.05	AG1	5	7.13333	0.399
0.2	0.05	ptsI	5	7.38333	0.348
0.2	0.05	clcB	5	7.38333	0.366
0.2	0.05	ycaM	5	7.38333	0.265
0.2	0.05	yadI	5	7.38333	0.44
0.2	0.05	AG1	5	7.38333	0.411
0.2	0.05	ptsI	5	7.63333	0.362
0.2	0.05	clcB	5	7.63333	0.369
0.2	0.05	ycaM	5	7.63333	0.268
0.2	0.05	yadI	5	7.63333	0.45
0.2	0.05	AG1	5	7.63333	0.422
0.2	0.05	ptsI	5	7.88333	0.372
0.2	0.05	clcB	5	7.88333	0.372
0.2	0.05	ycaM	5	7.88333	0.269
0.2	0.05	yadI	5	7.88333	0.462
0.2	0.05	AG1	5	7.88333	0.432
0.2	0.05	ptsI	5	8.13333	0.388
0.2	0.05	clcB	5	8.13333	0.38
0.2	0.05	ycaM	5	8.13333	0.273
0.2	0.05	yadI	5	8.13333	0.471
0.2	0.05	AG1	5	8.13333	0.448

0.2	0.05	ptsI	5	8.38333	0.405
0.2	0.05	clcB	5	8.38333	0.384
0.2	0.05	ycaM	5	8.38333	0.271
0.2	0.05	yadI	5	8.38333	0.476
0.2	0.05	AG1	5	8.38333	0.46
0.2	0.05	ptsI	5	8.88333	0.35
0.2	0.05	clcB	5	8.88333	0.296
0.2	0.05	ycaM	5	8.88333	0.272
0.2	0.05	yadI	5	8.88333	0.48
0.2	0.05	AG1	5	8.88333	0.499
0.2	0.05	ptsI	5	9.13333	0.356
0.2	0.05	clcB	5	9.13333	0.294
0.2	0.05	ycaM	5	9.13333	0.268
0.2	0.05	yadI	5	9.13333	0.482
0.2	0.05	AG1	5	9.13333	0.503
0.2	0.05	ptsI	5	9.38333	0.37
0.2	0.05	clcB	5	9.38333	0.296
0.2	0.05	ycaM	5	9.38333	0.267
0.2	0.05	yadI	5	9.38333	0.493
0.2	0.05	AG1	5	9.38333	0.525
0.2	0.05	ptsI	5	9.63333	0.375
0.2	0.05	clcB	5	9.63333	0.298
0.2	0.05	ycaM	5	9.63333	0.266
0.2	0.05	yadI	5	9.63333	0.504
0.2	0.05	AG1	5	9.63333	0.536
0.2	0.05	ptsI	5	9.88333	0.385
0.2	0.05	clcB	5	9.88333	0.3
0.2	0.05	ycaM	5	9.88333	0.266
0.2	0.05	yadI	5	9.88333	0.513
0.2	0.05	AG1	5	9.88333	0.55
0.2	0.05	ptsI	5	10.1333	0.393
0.2	0.05	clcB	5	10.1333	0.303
0.2	0.05	ycaM	5	10.1333	0.267
0.2	0.05	yadI	5	10.1333	0.521
0.2	0.05	AG1	5	10.1333	0.562
0.2	0.05	ptsI	5	10.3833	0.404
0.2	0.05	clcB	5	10.3833	0.306
0.2	0.05	ycaM	5	10.3833	0.268
0.2	0.05	yadI	5	10.3833	0.534
0.2	0.05	AG1	5	10.3833	0.575
0.2	0.05	ptsI	5	10.6333	0.411
0.2	0.05	clcB	5	10.6333	0.304
0.2	0.05	ycaM	5	10.6333	0.266
0.2	0.05	yadI	5	10.6333	0.536
0.2	0.05	AG1	5	10.6333	0.585
0.2	0.05	ptsI	5	10.8833	0.421
0.2	0.05	clcB	5	10.8833	0.307
0.2	0.05	ycaM	5	10.8833	0.27
0.2	0.05	yadI	5	10.8833	0.546
0.2	0.05	AG1	5	10.8833	0.597
0.2	0.05	ptsI	5	11.1333	0.428
0.2	0.05	clcB	5	11.1333	0.304
0.2	0.05	ycaM	5	11.1333	0.268

0.2	0.05	yadI	5	11.1333	0.552
0.2	0.05	AG1	5	11.1333	0.609
0.2	0.05	ptsI	5	11.3833	0.436
0.2	0.05	clcB	5	11.3833	0.302
0.2	0.05	ycaM	5	11.3833	0.269
0.2	0.05	yadI	5	11.3833	0.559
0.2	0.05	AG1	5	11.3833	0.62
0.2	0.05	ptsI	5	11.6333	0.447
0.2	0.05	clcB	5	11.6333	0.306
0.2	0.05	ycaM	5	11.6333	0.269
0.2	0.05	yadI	5	11.6333	0.568
0.2	0.05	AG1	5	11.6333	0.63
0.2	0.05	ptsI	5	11.8833	0.454
0.2	0.05	clcB	5	11.8833	0.306
0.2	0.05	ycaM	5	11.8833	0.269
0.2	0.05	yadI	5	11.8833	0.576
0.2	0.05	AG1	5	11.8833	0.64
0.2	0.05	ptsI	5	12.1333	0.466
0.2	0.05	clcB	5	12.1333	0.303
0.2	0.05	ycaM	5	12.1333	0.271
0.2	0.05	yadI	5	12.1333	0.583
0.2	0.05	AG1	5	12.1333	0.653
0.2	0.05	ptsI	5	12.3833	0.467
0.2	0.05	clcB	5	12.3833	0.295
0.2	0.05	ycaM	5	12.3833	0.275
0.2	0.05	yadI	5	12.3833	0.596
0.2	0.05	AG1	5	12.3833	0.661
0.2	0.05	ptsI	5	12.6333	0.479
0.2	0.05	clcB	5	12.6333	0.301
0.2	0.05	ycaM	5	12.6333	0.272
0.2	0.05	yadI	5	12.6333	0.594
0.2	0.05	AG1	5	12.6333	0.672
0.2	0.05	ptsI	5	12.8833	0.491
0.2	0.05	clcB	5	12.8833	0.309
0.2	0.05	ycaM	5	12.8833	0.276
0.2	0.05	yadI	5	12.8833	0.601
0.2	0.05	AG1	5	12.8833	0.679
0.2	0.05	ptsI	5	13.1333	0.497
0.2	0.05	clcB	5	13.1333	0.311
0.2	0.05	ycaM	5	13.1333	0.276
0.2	0.05	yadI	5	13.1333	0.607
0.2	0.05	AG1	5	13.1333	0.692
0.2	0.05	ptsI	5	13.3833	0.505
0.2	0.05	clcB	5	13.3833	0.307
0.2	0.05	ycaM	5	13.3833	0.274
0.2	0.05	yadI	5	13.3833	0.612
0.2	0.05	AG1	5	13.3833	0.699
0.2	0.05	ptsI	5	13.6333	0.513
0.2	0.05	clcB	5	13.6333	0.31
0.2	0.05	ycaM	5	13.6333	0.277
0.2	0.05	yadI	5	13.6333	0.618
0.2	0.05	AG1	5	13.6333	0.707
0.2	0.05	ptsI	5	13.8833	0.518

0.2	0.05	clcB	5	13.8833	0.307
0.2	0.05	ycaM	5	13.8833	0.277
0.2	0.05	yadI	5	13.8833	0.618
0.2	0.05	AG1	5	13.8833	0.712
0.2	0.05	ptsI	5	14.1333	0.525
0.2	0.05	clcB	5	14.1333	0.319
0.2	0.05	ycaM	5	14.1333	0.279
0.2	0.05	yadI	5	14.1333	0.625
0.2	0.05	AG1	5	14.1333	0.72
0.2	0.05	ptsI	5	14.3833	0.536
0.2	0.05	clcB	5	14.3833	0.316
0.2	0.05	ycaM	5	14.3833	0.282
0.2	0.05	yadI	5	14.3833	0.63
0.2	0.05	AG1	5	14.3833	0.725
0.2	0.05	ptsI	5	14.6333	0.545
0.2	0.05	clcB	5	14.6333	0.313
0.2	0.05	ycaM	5	14.6333	0.283
0.2	0.05	yadI	5	14.6333	0.635
0.2	0.05	AG1	5	14.6333	0.729
0.2	0.05	ptsI	5	14.8833	0.549
0.2	0.05	clcB	5	14.8833	0.316
0.2	0.05	ycaM	5	14.8833	0.283
0.2	0.05	yadI	5	14.8833	0.641
0.2	0.05	AG1	5	14.8833	0.739
0.2	0.05	ptsI	5	15.1333	0.555
0.2	0.05	clcB	5	15.1333	0.317
0.2	0.05	ycaM	5	15.1333	0.284
0.2	0.05	yadI	5	15.1333	0.646
0.2	0.05	AG1	5	15.1333	0.742
0.2	0.05	ptsI	5	15.3833	0.56
0.2	0.05	clcB	5	15.3833	0.315
0.2	0.05	ycaM	5	15.3833	0.286
0.2	0.05	yadI	5	15.3833	0.647
0.2	0.05	AG1	5	15.3833	0.749
0.2	0.05	ptsI	5	15.6333	0.568
0.2	0.05	clcB	5	15.6333	0.317
0.2	0.05	ycaM	5	15.6333	0.286
0.2	0.05	yadI	5	15.6333	0.654
0.2	0.05	AG1	5	15.6333	0.755
0.2	0.05	ptsI	5	15.8833	0.574
0.2	0.05	clcB	5	15.8833	0.312
0.2	0.05	ycaM	5	15.8833	0.285
0.2	0.05	yadI	5	15.8833	0.658
0.2	0.05	AG1	5	15.8833	0.76
0.2	0.05	ptsI	5	16.1333	0.585
0.2	0.05	clcB	5	16.1333	0.321
0.2	0.05	ycaM	5	16.1333	0.289
0.2	0.05	yadI	5	16.1333	0.662
0.2	0.05	AG1	5	16.1333	0.772
0.2	0.05	ptsI	5	16.3833	0.586
0.2	0.05	clcB	5	16.3833	0.325
0.2	0.05	ycaM	5	16.3833	0.289
0.2	0.05	yadI	5	16.3833	0.659

0.2	0.05	AG1	5	16.3833	0.77
0.2	0.05	ptsI	5	16.6333	0.592
0.2	0.05	clcB	5	16.6333	0.326
0.2	0.05	ycaM	5	16.6333	0.291
0.2	0.05	yadI	5	16.6333	0.665
0.2	0.05	AG1	5	16.6333	0.776
0.2	0.05	ptsI	5	16.8833	0.595
0.2	0.05	clcB	5	16.8833	0.329
0.2	0.05	ycaM	5	16.8833	0.292
0.2	0.05	yadI	5	16.8833	0.668
0.2	0.05	AG1	5	16.8833	0.777
0.2	0.05	ptsI	5	17.1333	0.602
0.2	0.05	clcB	5	17.1333	0.326
0.2	0.05	ycaM	5	17.1333	0.293
0.2	0.05	yadI	5	17.1333	0.674
0.2	0.05	AG1	5	17.1333	0.785
0.2	0.05	ptsI	5	17.3833	0.604
0.2	0.05	clcB	5	17.3833	0.331
0.2	0.05	ycaM	5	17.3833	0.294
0.2	0.05	yadI	5	17.3833	0.675
0.2	0.05	AG1	5	17.3833	0.791
0.2	0.05	ptsI	5	17.6333	0.61
0.2	0.05	clcB	5	17.6333	0.341
0.2	0.05	ycaM	5	17.6333	0.294
0.2	0.05	yadI	5	17.6333	0.678
0.2	0.05	AG1	5	17.6333	0.793
0.2	0.05	ptsI	5	17.8833	0.612
0.2	0.05	clcB	5	17.8833	0.34
0.2	0.05	ycaM	5	17.8833	0.299
0.2	0.05	yadI	5	17.8833	0.689
0.2	0.05	AG1	5	17.8833	0.796
0.2	0.05	ptsI	5	18.1333	0.621
0.2	0.05	clcB	5	18.1333	0.349
0.2	0.05	ycaM	5	18.1333	0.299
0.2	0.05	yadI	5	18.1333	0.68
0.2	0.05	AG1	5	18.1333	0.798
0.2	0.05	ptsI	5	18.3833	0.625
0.2	0.05	clcB	5	18.3833	0.351
0.2	0.05	ycaM	5	18.3833	0.298
0.2	0.05	yadI	5	18.3833	0.688
0.2	0.05	AG1	5	18.3833	0.804
0.2	0.05	ptsI	5	18.6333	0.626
0.2	0.05	clcB	5	18.6333	0.354
0.2	0.05	ycaM	5	18.6333	0.302
0.2	0.05	yadI	5	18.6333	0.689
0.2	0.05	AG1	5	18.6333	0.806
0.2	0.05	ptsI	5	18.8833	0.634
0.2	0.05	clcB	5	18.8833	0.359
0.2	0.05	ycaM	5	18.8833	0.304
0.2	0.05	yadI	5	18.8833	0.69
0.2	0.05	AG1	5	18.8833	0.814
0.2	0.05	ptsI	5	19.1333	0.637
0.2	0.05	clcB	5	19.1333	0.362

0.2	0.05	ycaM	5	19.1333	0.306
0.2	0.05	yadI	5	19.1333	0.694
0.2	0.05	AG1	5	19.1333	0.816
0.2	0.05	ptsI	5	19.3833	0.637
0.2	0.05	clcB	5	19.3833	0.366
0.2	0.05	ycaM	5	19.3833	0.303
0.2	0.05	yadI	5	19.3833	0.699
0.2	0.05	AG1	5	19.3833	0.818
0.2	0.05	ptsI	5	19.6333	0.639
0.2	0.05	clcB	5	19.6333	0.37
0.2	0.05	ycaM	5	19.6333	0.309
0.2	0.05	yadI	5	19.6333	0.702
0.2	0.05	AG1	5	19.6333	0.821
0.2	0.05	ptsI	5	19.8833	0.643
0.2	0.05	clcB	5	19.8833	0.375
0.2	0.05	ycaM	5	19.8833	0.31
0.2	0.05	yadI	5	19.8833	0.701
0.2	0.05	AG1	5	19.8833	0.826
0.2	0.05	ptsI	5	20.1333	0.642
0.2	0.05	clcB	5	20.1333	0.378
0.2	0.05	ycaM	5	20.1333	0.315
0.2	0.05	yadI	5	20.1333	0.704
0.2	0.05	AG1	5	20.1333	0.829
0.2	0.05	ptsI	5	20.3833	0.645
0.2	0.05	clcB	5	20.3833	0.376
0.2	0.05	ycaM	5	20.3833	0.314
0.2	0.05	yadI	5	20.3833	0.706
0.2	0.05	AG1	5	20.3833	0.832
0.2	0.05	ptsI	5	20.6333	0.638
0.2	0.05	clcB	5	20.6333	0.372
0.2	0.05	ycaM	5	20.6333	0.315
0.2	0.05	yadI	5	20.6333	0.714
0.2	0.05	AG1	5	20.6333	0.828
0.2	0.05	ptsI	5	20.8833	0.648
0.2	0.05	clcB	5	20.8833	0.387
0.2	0.05	ycaM	5	20.8833	0.317
0.2	0.05	yadI	5	20.8833	0.712
0.2	0.05	AG1	5	20.8833	0.836
0.2	0.05	ptsI	5	21.1333	0.65
0.2	0.05	clcB	5	21.1333	0.385
0.2	0.05	ycaM	5	21.1333	0.32
0.2	0.05	yadI	5	21.1333	0.712
0.2	0.05	AG1	5	21.1333	0.838
0.2	0.05	ptsI	5	21.3833	0.65
0.2	0.05	clcB	5	21.3833	0.394
0.2	0.05	ycaM	5	21.3833	0.322
0.2	0.05	yadI	5	21.3833	0.713
0.2	0.05	AG1	5	21.3833	0.839
0.2	0.05	ptsI	5	21.6333	0.652
0.2	0.05	clcB	5	21.6333	0.379
0.2	0.05	ycaM	5	21.6333	0.325
0.2	0.05	yadI	5	21.6333	0.716
0.2	0.05	AG1	5	21.6333	0.839

0.2	0.05	ptsI	5	21.8833	0.651
0.2	0.05	clcB	5	21.8833	0.398
0.2	0.05	ycaM	5	21.8833	0.328
0.2	0.05	yadI	5	21.8833	0.715
0.2	0.05	AG1	5	21.8833	0.839
0.2	0.05	ptsI	5	22.1333	0.648
0.2	0.05	clcB	5	22.1333	0.375
0.2	0.05	ycaM	5	22.1333	0.33
0.2	0.05	yadI	5	22.1333	0.726
0.2	0.05	AG1	5	22.1333	0.84
0.2	0.05	ptsI	5	22.3833	0.638
0.2	0.05	clcB	5	22.3833	0.383
0.2	0.05	ycaM	5	22.3833	0.329
0.2	0.05	yadI	5	22.3833	0.724
0.2	0.05	AG1	5	22.3833	0.845
0.2	0.05	ptsI	5	22.6333	0.638
0.2	0.05	clcB	5	22.6333	0.377
0.2	0.05	ycaM	5	22.6333	0.334
0.2	0.05	yadI	5	22.6333	0.718
0.2	0.05	AG1	5	22.6333	0.846
0.2	0.05	ptsI	5	22.8833	0.642
0.2	0.05	clcB	5	22.8833	0.379
0.2	0.05	ycaM	5	22.8833	0.337
0.2	0.05	yadI	5	22.8833	0.721
0.2	0.05	AG1	5	22.8833	0.848
0.2	0.05	ptsI	5	23.1333	0.647
0.2	0.05	clcB	5	23.1333	0.366
0.2	0.05	ycaM	5	23.1333	0.338
0.2	0.05	yadI	5	23.1333	0.719
0.2	0.05	AG1	5	23.1333	0.85
0.2	0.05	ptsI	5	23.3833	0.653
0.2	0.05	clcB	5	23.3833	0.373
0.2	0.05	ycaM	5	23.3833	0.341
0.2	0.05	yadI	5	23.3833	0.724
0.2	0.05	AG1	5	23.3833	0.852
0.2	0.05	ptsI	5	23.6333	0.651
0.2	0.05	clcB	5	23.6333	0.369
0.2	0.05	ycaM	5	23.6333	0.342
0.2	0.05	yadI	5	23.6333	0.727
0.2	0.05	AG1	5	23.6333	0.855
0.2	0.05	ptsI	5	23.8833	0.651
0.2	0.05	clcB	5	23.8833	0.356
0.2	0.05	ycaM	5	23.8833	0.34
0.2	0.05	yadI	5	23.8833	0.732
0.2	0.05	AG1	5	23.8833	0.851
0.2	0.05	ptsI	5	24.1333	0.65
0.2	0.05	clcB	5	24.1333	0.371
0.2	0.05	ycaM	5	24.1333	0.345
0.2	0.05	yadI	5	24.1333	0.723
0.2	0.05	AG1	5	24.1333	0.858
0.2	0.05	ptsI	5	24.3833	0.656
0.2	0.05	clcB	5	24.3833	0.365
0.2	0.05	ycaM	5	24.3833	0.345

0.2	0.05	yadI	5	24.3833	0.728
0.2	0.05	AG1	5	24.3833	0.856
0.4	0.05	ptsI	2	0	0.211
0.4	0.05	clcB	2	0	0.228
0.4	0.05	ycaM	2	0	0.217
0.4	0.05	yadI	2	0	0.221
0.4	0.05	AG1	2	0	0.227
0.4	0.05	ptsI	2	0.25	0.211
0.4	0.05	clcB	2	0.25	0.22
0.4	0.05	ycaM	2	0.25	0.212
0.4	0.05	yadI	2	0.25	0.215
0.4	0.05	AG1	2	0.25	0.22
0.4	0.05	ptsI	2	0.5	0.21
0.4	0.05	clcB	2	0.5	0.218
0.4	0.05	ycaM	2	0.5	0.211
0.4	0.05	yadI	2	0.5	0.214
0.4	0.05	AG1	2	0.5	0.221
0.4	0.05	ptsI	2	0.75	0.214
0.4	0.05	clcB	2	0.75	0.218
0.4	0.05	ycaM	2	0.75	0.212
0.4	0.05	yadI	2	0.75	0.216
0.4	0.05	AG1	2	0.75	0.221
0.4	0.05	ptsI	2	1	0.217
0.4	0.05	clcB	2	1	0.22
0.4	0.05	ycaM	2	1	0.214
0.4	0.05	yadI	2	1	0.223
0.4	0.05	AG1	2	1	0.223
0.4	0.05	ptsI	2	1.25	0.219
0.4	0.05	clcB	2	1.25	0.222
0.4	0.05	ycaM	2	1.25	0.214
0.4	0.05	yadI	2	1.25	0.227
0.4	0.05	AG1	2	1.25	0.227
0.4	0.05	ptsI	2	1.5	0.224
0.4	0.05	clcB	2	1.5	0.229
0.4	0.05	ycaM	2	1.5	0.214
0.4	0.05	yadI	2	1.5	0.233
0.4	0.05	AG1	2	1.5	0.229
0.4	0.05	ptsI	2	1.75	0.23
0.4	0.05	clcB	2	1.75	0.23
0.4	0.05	ycaM	2	1.75	0.217
0.4	0.05	yadI	2	1.75	0.239
0.4	0.05	AG1	2	1.75	0.234
0.4	0.05	ptsI	2	2	0.24
0.4	0.05	clcB	2	2	0.232
0.4	0.05	ycaM	2	2	0.225
0.4	0.05	yadI	2	2	0.247
0.4	0.05	AG1	2	2	0.24
0.4	0.05	ptsI	2	2.25	0.249
0.4	0.05	clcB	2	2.25	0.235
0.4	0.05	ycaM	2	2.25	0.227
0.4	0.05	yadI	2	2.25	0.256
0.4	0.05	AG1	2	2.25	0.248
0.4	0.05	ptsI	2	2.5	0.26

0.4	0.05	clcB	2	2.5	0.24
0.4	0.05	ycaM	2	2.5	0.235
0.4	0.05	yadI	2	2.5	0.266
0.4	0.05	AG1	2	2.5	0.259
0.4	0.05	ptsI	2	2.75	0.256
0.4	0.05	clcB	2	2.75	0.244
0.4	0.05	ycaM	2	2.75	0.236
0.4	0.05	yadI	2	2.75	0.278
0.4	0.05	AG1	2	2.75	0.253
0.4	0.05	ptsI	2	3	0.262
0.4	0.05	clcB	2	3	0.25
0.4	0.05	ycaM	2	3	0.24
0.4	0.05	yadI	2	3	0.284
0.4	0.05	AG1	2	3	0.256
0.4	0.05	ptsI	2	3.25	0.286
0.4	0.05	clcB	2	3.25	0.256
0.4	0.05	ycaM	2	3.25	0.249
0.4	0.05	yadI	2	3.25	0.3
0.4	0.05	AG1	2	3.25	0.269
0.4	0.05	ptsI	2	3.5	0.297
0.4	0.05	clcB	2	3.5	0.261
0.4	0.05	ycaM	2	3.5	0.255
0.4	0.05	yadI	2	3.5	0.311
0.4	0.05	AG1	2	3.5	0.278
0.4	0.05	ptsI	2	3.75	0.311
0.4	0.05	clcB	2	3.75	0.269
0.4	0.05	ycaM	2	3.75	0.264
0.4	0.05	yadI	2	3.75	0.325
0.4	0.05	AG1	2	3.75	0.287
0.4	0.05	ptsI	2	4	0.326
0.4	0.05	clcB	2	4	0.274
0.4	0.05	ycaM	2	4	0.275
0.4	0.05	yadI	2	4	0.339
0.4	0.05	AG1	2	4	0.298
0.4	0.05	ptsI	2	4.38333	0.299
0.4	0.05	clcB	2	4.38333	0.247
0.4	0.05	ycaM	2	4.38333	0.292
0.4	0.05	yadI	2	4.38333	0.331
0.4	0.05	AG1	2	4.38333	0.288
0.4	0.05	ptsI	2	4.63333	0.299
0.4	0.05	clcB	2	4.63333	0.245
0.4	0.05	ycaM	2	4.63333	0.29
0.4	0.05	yadI	2	4.63333	0.336
0.4	0.05	AG1	2	4.63333	0.286
0.4	0.05	ptsI	2	4.88333	0.307
0.4	0.05	clcB	2	4.88333	0.247
0.4	0.05	ycaM	2	4.88333	0.29
0.4	0.05	yadI	2	4.88333	0.35
0.4	0.05	AG1	2	4.88333	0.298
0.4	0.05	ptsI	2	5.13333	0.317
0.4	0.05	clcB	2	5.13333	0.252
0.4	0.05	ycaM	2	5.13333	0.294
0.4	0.05	yadI	2	5.13333	0.365

0.4	0.05	AG1	2	5.13333	0.304
0.4	0.05	ptsI	2	5.38333	0.326
0.4	0.05	clcB	2	5.38333	0.258
0.4	0.05	ycaM	2	5.38333	0.293
0.4	0.05	yadI	2	5.38333	0.375
0.4	0.05	AG1	2	5.38333	0.313
0.4	0.05	ptsI	2	5.63333	0.336
0.4	0.05	clcB	2	5.63333	0.268
0.4	0.05	ycaM	2	5.63333	0.295
0.4	0.05	yadI	2	5.63333	0.387
0.4	0.05	AG1	2	5.63333	0.324
0.4	0.05	ptsI	2	5.88333	0.349
0.4	0.05	clcB	2	5.88333	0.275
0.4	0.05	ycaM	2	5.88333	0.294
0.4	0.05	yadI	2	5.88333	0.393
0.4	0.05	AG1	2	5.88333	0.334
0.4	0.05	ptsI	2	6.13333	0.362
0.4	0.05	clcB	2	6.13333	0.28
0.4	0.05	ycaM	2	6.13333	0.297
0.4	0.05	yadI	2	6.13333	0.402
0.4	0.05	AG1	2	6.13333	0.34
0.4	0.05	ptsI	2	6.38333	0.382
0.4	0.05	clcB	2	6.38333	0.286
0.4	0.05	ycaM	2	6.38333	0.299
0.4	0.05	yadI	2	6.38333	0.411
0.4	0.05	AG1	2	6.38333	0.351
0.4	0.05	ptsI	2	6.63333	0.396
0.4	0.05	clcB	2	6.63333	0.291
0.4	0.05	ycaM	2	6.63333	0.298
0.4	0.05	yadI	2	6.63333	0.421
0.4	0.05	AG1	2	6.63333	0.358
0.4	0.05	ptsI	2	6.88333	0.411
0.4	0.05	clcB	2	6.88333	0.298
0.4	0.05	ycaM	2	6.88333	0.299
0.4	0.05	yadI	2	6.88333	0.428
0.4	0.05	AG1	2	6.88333	0.371
0.4	0.05	ptsI	2	7.13333	0.425
0.4	0.05	clcB	2	7.13333	0.302
0.4	0.05	ycaM	2	7.13333	0.299
0.4	0.05	yadI	2	7.13333	0.434
0.4	0.05	AG1	2	7.13333	0.397
0.4	0.05	ptsI	2	7.38333	0.436
0.4	0.05	clcB	2	7.38333	0.308
0.4	0.05	ycaM	2	7.38333	0.301
0.4	0.05	yadI	2	7.38333	0.443
0.4	0.05	AG1	2	7.38333	0.389
0.4	0.05	ptsI	2	7.63333	0.434
0.4	0.05	clcB	2	7.63333	0.312
0.4	0.05	ycaM	2	7.63333	0.298
0.4	0.05	yadI	2	7.63333	0.448
0.4	0.05	AG1	2	7.63333	0.392
0.4	0.05	ptsI	2	7.88333	0.452
0.4	0.05	clcB	2	7.88333	0.317

0.4	0.05	ycaM	2	7.88333	0.299
0.4	0.05	yadI	2	7.88333	0.468
0.4	0.05	AG1	2	7.88333	0.402
0.4	0.05	ptsI	2	8.13333	0.466
0.4	0.05	clcB	2	8.13333	0.318
0.4	0.05	ycaM	2	8.13333	0.3
0.4	0.05	yadI	2	8.13333	0.462
0.4	0.05	AG1	2	8.13333	0.412
0.4	0.05	ptsI	2	8.38333	0.473
0.4	0.05	clcB	2	8.38333	0.33
0.4	0.05	ycaM	2	8.38333	0.3
0.4	0.05	yadI	2	8.38333	0.471
0.4	0.05	AG1	2	8.38333	0.42
0.4	0.05	ptsI	2	8.91667	0.376
0.4	0.05	clcB	2	8.91667	0.342
0.4	0.05	ycaM	2	8.91667	0.324
0.4	0.05	yadI	2	8.91667	0.52
0.4	0.05	AG1	2	8.91667	0.466
0.4	0.05	ptsI	2	9.16667	0.376
0.4	0.05	clcB	2	9.16667	0.341
0.4	0.05	ycaM	2	9.16667	0.322
0.4	0.05	yadI	2	9.16667	0.524
0.4	0.05	AG1	2	9.16667	0.474
0.4	0.05	ptsI	2	9.41667	0.384
0.4	0.05	clcB	2	9.41667	0.342
0.4	0.05	ycaM	2	9.41667	0.321
0.4	0.05	yadI	2	9.41667	0.543
0.4	0.05	AG1	2	9.41667	0.494
0.4	0.05	ptsI	2	9.66667	0.394
0.4	0.05	clcB	2	9.66667	0.345
0.4	0.05	ycaM	2	9.66667	0.322
0.4	0.05	yadI	2	9.66667	0.546
0.4	0.05	AG1	2	9.66667	0.511
0.4	0.05	ptsI	2	9.91667	0.403
0.4	0.05	clcB	2	9.91667	0.35
0.4	0.05	ycaM	2	9.91667	0.322
0.4	0.05	yadI	2	9.91667	0.548
0.4	0.05	AG1	2	9.91667	0.523
0.4	0.05	ptsI	2	10.1667	0.407
0.4	0.05	clcB	2	10.1667	0.353
0.4	0.05	ycaM	2	10.1667	0.321
0.4	0.05	yadI	2	10.1667	0.568
0.4	0.05	AG1	2	10.1667	0.533
0.4	0.05	ptsI	2	10.4167	0.418
0.4	0.05	clcB	2	10.4167	0.36
0.4	0.05	ycaM	2	10.4167	0.326
0.4	0.05	yadI	2	10.4167	0.578
0.4	0.05	AG1	2	10.4167	0.545
0.4	0.05	ptsI	2	10.6667	0.43
0.4	0.05	clcB	2	10.6667	0.362
0.4	0.05	ycaM	2	10.6667	0.325
0.4	0.05	yadI	2	10.6667	0.578
0.4	0.05	AG1	2	10.6667	0.572

0.4	0.05	ptsI	2	10.9167	0.441
0.4	0.05	clcB	2	10.9167	0.361
0.4	0.05	ycaM	2	10.9167	0.324
0.4	0.05	yadI	2	10.9167	0.584
0.4	0.05	AG1	2	10.9167	0.587
0.4	0.05	ptsI	2	11.1667	0.448
0.4	0.05	clcB	2	11.1667	0.36
0.4	0.05	ycaM	2	11.1667	0.324
0.4	0.05	yadI	2	11.1667	0.594
0.4	0.05	AG1	2	11.1667	0.598
0.4	0.05	ptsI	2	11.4167	0.46
0.4	0.05	clcB	2	11.4167	0.363
0.4	0.05	ycaM	2	11.4167	0.324
0.4	0.05	yadI	2	11.4167	0.595
0.4	0.05	AG1	2	11.4167	0.606
0.4	0.05	ptsI	2	11.6667	0.473
0.4	0.05	clcB	2	11.6667	0.363
0.4	0.05	ycaM	2	11.6667	0.325
0.4	0.05	yadI	2	11.6667	0.606
0.4	0.05	AG1	2	11.6667	0.632
0.4	0.05	ptsI	2	11.9167	0.479
0.4	0.05	clcB	2	11.9167	0.356
0.4	0.05	ycaM	2	11.9167	0.324
0.4	0.05	yadI	2	11.9167	0.61
0.4	0.05	AG1	2	11.9167	0.632
0.4	0.05	ptsI	2	12.1667	0.492
0.4	0.05	clcB	2	12.1667	0.359
0.4	0.05	ycaM	2	12.1667	0.323
0.4	0.05	yadI	2	12.1667	0.619
0.4	0.05	AG1	2	12.1667	0.656
0.4	0.05	ptsI	2	12.4167	0.5
0.4	0.05	clcB	2	12.4167	0.356
0.4	0.05	ycaM	2	12.4167	0.325
0.4	0.05	yadI	2	12.4167	0.626
0.4	0.05	AG1	2	12.4167	0.674
0.4	0.05	ptsI	2	12.6667	0.502
0.4	0.05	clcB	2	12.6667	0.345
0.4	0.05	ycaM	2	12.6667	0.322
0.4	0.05	yadI	2	12.6667	0.63
0.4	0.05	AG1	2	12.6667	0.669
0.4	0.05	ptsI	2	12.9167	0.523
0.4	0.05	clcB	2	12.9167	0.344
0.4	0.05	ycaM	2	12.9167	0.325
0.4	0.05	yadI	2	12.9167	0.639
0.4	0.05	AG1	2	12.9167	0.669
0.4	0.05	ptsI	2	13.1667	0.541
0.4	0.05	clcB	2	13.1667	0.342
0.4	0.05	ycaM	2	13.1667	0.322
0.4	0.05	yadI	2	13.1667	0.648
0.4	0.05	AG1	2	13.1667	0.672
0.4	0.05	ptsI	2	13.4167	0.555
0.4	0.05	clcB	2	13.4167	0.343
0.4	0.05	ycaM	2	13.4167	0.319

0.4	0.05	yadI	2	13.4167	0.663
0.4	0.05	AG1	2	13.4167	0.649
0.4	0.05	ptsI	2	13.6667	0.57
0.4	0.05	clcB	2	13.6667	0.346
0.4	0.05	ycaM	2	13.6667	0.319
0.4	0.05	yadI	2	13.6667	0.662
0.4	0.05	AG1	2	13.6667	0.68
0.4	0.05	ptsI	2	13.9167	0.575
0.4	0.05	clcB	2	13.9167	0.348
0.4	0.05	ycaM	2	13.9167	0.32
0.4	0.05	yadI	2	13.9167	0.67
0.4	0.05	AG1	2	13.9167	0.706
0.4	0.05	ptsI	2	14.1667	0.577
0.4	0.05	clcB	2	14.1667	0.349
0.4	0.05	ycaM	2	14.1667	0.319
0.4	0.05	yadI	2	14.1667	0.664
0.4	0.05	AG1	2	14.1667	0.695
0.4	0.05	ptsI	2	14.4167	0.59
0.4	0.05	clcB	2	14.4167	0.345
0.4	0.05	ycaM	2	14.4167	0.318
0.4	0.05	yadI	2	14.4167	0.68
0.4	0.05	AG1	2	14.4167	0.671
0.4	0.05	ptsI	2	14.6667	0.596
0.4	0.05	clcB	2	14.6667	0.34
0.4	0.05	ycaM	2	14.6667	0.317
0.4	0.05	yadI	2	14.6667	0.697
0.4	0.05	AG1	2	14.6667	0.679
0.4	0.05	ptsI	2	14.9167	0.608
0.4	0.05	clcB	2	14.9167	0.346
0.4	0.05	ycaM	2	14.9167	0.319
0.4	0.05	yadI	2	14.9167	0.711
0.4	0.05	AG1	2	14.9167	0.687
0.4	0.05	ptsI	2	15.1667	0.617
0.4	0.05	clcB	2	15.1667	0.349
0.4	0.05	ycaM	2	15.1667	0.319
0.4	0.05	yadI	2	15.1667	0.694
0.4	0.05	AG1	2	15.1667	0.682
0.4	0.05	ptsI	2	15.4167	0.635
0.4	0.05	clcB	2	15.4167	0.346
0.4	0.05	ycaM	2	15.4167	0.315
0.4	0.05	yadI	2	15.4167	0.709
0.4	0.05	AG1	2	15.4167	0.712
0.4	0.05	ptsI	2	15.6667	0.617
0.4	0.05	clcB	2	15.6667	0.344
0.4	0.05	ycaM	2	15.6667	0.312
0.4	0.05	yadI	2	15.6667	0.702
0.4	0.05	AG1	2	15.6667	0.693
0.4	0.05	ptsI	2	15.9167	0.63
0.4	0.05	clcB	2	15.9167	0.344
0.4	0.05	ycaM	2	15.9167	0.315
0.4	0.05	yadI	2	15.9167	0.706
0.4	0.05	AG1	2	15.9167	0.717
0.4	0.05	ptsI	2	16.1667	0.641

0.4	0.05	clcB	2	16.1667	0.341
0.4	0.05	ycaM	2	16.1667	0.315
0.4	0.05	yadI	2	16.1667	0.738
0.4	0.05	AG1	2	16.1667	0.729
0.4	0.05	ptsI	2	16.4167	0.643
0.4	0.05	clcB	2	16.4167	0.351
0.4	0.05	ycaM	2	16.4167	0.316
0.4	0.05	yadI	2	16.4167	0.714
0.4	0.05	AG1	2	16.4167	0.743
0.4	0.05	ptsI	2	16.6667	0.645
0.4	0.05	clcB	2	16.6667	0.351
0.4	0.05	ycaM	2	16.6667	0.313
0.4	0.05	yadI	2	16.6667	0.718
0.4	0.05	AG1	2	16.6667	0.751
0.4	0.05	ptsI	2	16.9167	0.65
0.4	0.05	clcB	2	16.9167	0.35
0.4	0.05	ycaM	2	16.9167	0.314
0.4	0.05	yadI	2	16.9167	0.735
0.4	0.05	AG1	2	16.9167	0.766
0.4	0.05	ptsI	2	17.1667	0.664
0.4	0.05	clcB	2	17.1667	0.35
0.4	0.05	ycaM	2	17.1667	0.315
0.4	0.05	yadI	2	17.1667	0.731
0.4	0.05	AG1	2	17.1667	0.767
0.4	0.05	ptsI	2	17.4167	0.669
0.4	0.05	clcB	2	17.4167	0.342
0.4	0.05	ycaM	2	17.4167	0.314
0.4	0.05	yadI	2	17.4167	0.748
0.4	0.05	AG1	2	17.4167	0.775
0.4	0.05	ptsI	2	17.6667	0.676
0.4	0.05	clcB	2	17.6667	0.344
0.4	0.05	ycaM	2	17.6667	0.314
0.4	0.05	yadI	2	17.6667	0.75
0.4	0.05	AG1	2	17.6667	0.771
0.4	0.05	ptsI	2	17.9167	0.673
0.4	0.05	clcB	2	17.9167	0.343
0.4	0.05	ycaM	2	17.9167	0.314
0.4	0.05	yadI	2	17.9167	0.746
0.4	0.05	AG1	2	17.9167	0.764
0.4	0.05	ptsI	2	18.1667	0.679
0.4	0.05	clcB	2	18.1667	0.345
0.4	0.05	ycaM	2	18.1667	0.314
0.4	0.05	yadI	2	18.1667	0.753
0.4	0.05	AG1	2	18.1667	0.766
0.4	0.05	ptsI	2	18.4167	0.685
0.4	0.05	clcB	2	18.4167	0.344
0.4	0.05	ycaM	2	18.4167	0.317
0.4	0.05	yadI	2	18.4167	0.744
0.4	0.05	AG1	2	18.4167	0.774
0.4	0.05	ptsI	2	18.6667	0.687
0.4	0.05	clcB	2	18.6667	0.343
0.4	0.05	ycaM	2	18.6667	0.315
0.4	0.05	yadI	2	18.6667	0.744

0.4	0.05	AG1	2	18.6667	0.774
0.4	0.05	ptsI	2	18.9167	0.688
0.4	0.05	clcB	2	18.9167	0.341
0.4	0.05	ycaM	2	18.9167	0.309
0.4	0.05	yadI	2	18.9167	0.73
0.4	0.05	AG1	2	18.9167	0.772
0.4	0.05	ptsI	2	19.1667	0.684
0.4	0.05	clcB	2	19.1667	0.344
0.4	0.05	ycaM	2	19.1667	0.314
0.4	0.05	yadI	2	19.1667	0.748
0.4	0.05	AG1	2	19.1667	0.778
0.4	0.05	ptsI	2	19.4167	0.69
0.4	0.05	clcB	2	19.4167	0.345
0.4	0.05	ycaM	2	19.4167	0.315
0.4	0.05	yadI	2	19.4167	0.753
0.4	0.05	AG1	2	19.4167	0.783
0.4	0.05	ptsI	2	19.6667	0.693
0.4	0.05	clcB	2	19.6667	0.342
0.4	0.05	ycaM	2	19.6667	0.318
0.4	0.05	yadI	2	19.6667	0.756
0.4	0.05	AG1	2	19.6667	0.783
0.4	0.05	ptsI	2	19.9167	0.693
0.4	0.05	clcB	2	19.9167	0.343
0.4	0.05	ycaM	2	19.9167	0.32
0.4	0.05	yadI	2	19.9167	0.756
0.4	0.05	AG1	2	19.9167	0.79
0.4	0.05	ptsI	2	20.1667	0.691
0.4	0.05	clcB	2	20.1667	0.344
0.4	0.05	ycaM	2	20.1667	0.321
0.4	0.05	yadI	2	20.1667	0.766
0.4	0.05	AG1	2	20.1667	0.793
0.4	0.05	ptsI	2	20.4167	0.693
0.4	0.05	clcB	2	20.4167	0.343
0.4	0.05	ycaM	2	20.4167	0.322
0.4	0.05	yadI	2	20.4167	0.762
0.4	0.05	AG1	2	20.4167	0.8
0.4	0.05	ptsI	2	20.6667	0.693
0.4	0.05	clcB	2	20.6667	0.344
0.4	0.05	ycaM	2	20.6667	0.323
0.4	0.05	yadI	2	20.6667	0.776
0.4	0.05	AG1	2	20.6667	0.799
0.4	0.05	ptsI	2	20.9167	0.698
0.4	0.05	clcB	2	20.9167	0.344
0.4	0.05	ycaM	2	20.9167	0.324
0.4	0.05	yadI	2	20.9167	0.768
0.4	0.05	AG1	2	20.9167	0.806
0.4	0.05	ptsI	2	21.1667	0.702
0.4	0.05	clcB	2	21.1667	0.346
0.4	0.05	ycaM	2	21.1667	0.331
0.4	0.05	yadI	2	21.1667	0.788
0.4	0.05	AG1	2	21.1667	0.818
0.4	0.05	ptsI	2	21.4167	0.698
0.4	0.05	clcB	2	21.4167	0.344

0.4	0.05	ycaM	2	21.4167	0.326
0.4	0.05	yadI	2	21.4167	0.783
0.4	0.05	AG1	2	21.4167	0.817
0.4	0.05	ptsI	2	21.6667	0.693
0.4	0.05	clcB	2	21.6667	0.347
0.4	0.05	ycaM	2	21.6667	0.333
0.4	0.05	yadI	2	21.6667	0.789
0.4	0.05	AG1	2	21.6667	0.833
0.4	0.05	ptsI	2	21.9167	0.69
0.4	0.05	clcB	2	21.9167	0.347
0.4	0.05	ycaM	2	21.9167	0.335
0.4	0.05	yadI	2	21.9167	0.791
0.4	0.05	AG1	2	21.9167	0.836
0.4	0.05	ptsI	2	22.1667	0.701
0.4	0.05	clcB	2	22.1667	0.347
0.4	0.05	ycaM	2	22.1667	0.339
0.4	0.05	yadI	2	22.1667	0.797
0.4	0.05	AG1	2	22.1667	0.834
0.4	0.05	ptsI	2	22.4167	0.706
0.4	0.05	clcB	2	22.4167	0.346
0.4	0.05	ycaM	2	22.4167	0.344
0.4	0.05	yadI	2	22.4167	0.8
0.4	0.05	AG1	2	22.4167	0.843
0.4	0.05	ptsI	2	22.6667	0.7
0.4	0.05	clcB	2	22.6667	0.345
0.4	0.05	ycaM	2	22.6667	0.347
0.4	0.05	yadI	2	22.6667	0.798
0.4	0.05	AG1	2	22.6667	0.839
0.4	0.05	ptsI	2	22.9167	0.707
0.4	0.05	clcB	2	22.9167	0.349
0.4	0.05	ycaM	2	22.9167	0.347
0.4	0.05	yadI	2	22.9167	0.803
0.4	0.05	AG1	2	22.9167	0.862
0.4	0.05	ptsI	2	23.1667	0.699
0.4	0.05	clcB	2	23.1667	0.348
0.4	0.05	ycaM	2	23.1667	0.35
0.4	0.05	yadI	2	23.1667	0.806
0.4	0.05	AG1	2	23.1667	0.851
0.4	0.05	ptsI	2	23.4167	0.705
0.4	0.05	clcB	2	23.4167	0.352
0.4	0.05	ycaM	2	23.4167	0.352
0.4	0.05	yadI	2	23.4167	0.812
0.4	0.05	AG1	2	23.4167	0.857
0.4	0.05	ptsI	2	23.6667	0.704
0.4	0.05	clcB	2	23.6667	0.348
0.4	0.05	ycaM	2	23.6667	0.353
0.4	0.05	yadI	2	23.6667	0.811
0.4	0.05	AG1	2	23.6667	0.862
0.4	0.05	ptsI	2	23.9167	0.702
0.4	0.05	clcB	2	23.9167	0.347
0.4	0.05	ycaM	2	23.9167	0.357
0.4	0.05	yadI	2	23.9167	0.81
0.4	0.05	AG1	2	23.9167	0.868

0.4	0.05	ptsI	2	24.1667	0.702
0.4	0.05	clcB	2	24.1667	0.352
0.4	0.05	ycaM	2	24.1667	0.36
0.4	0.05	yadI	2	24.1667	0.814
0.4	0.05	AG1	2	24.1667	0.877
0.4	0.05	ptsI	2	24.4167	0.7
0.4	0.05	clcB	2	24.4167	0.353
0.4	0.05	ycaM	2	24.4167	0.361
0.4	0.05	yadI	2	24.4167	0.821
0.4	0.05	AG1	2	24.4167	0.884
0.4	0.05	ptsI	2	24.6667	0.701
0.4	0.05	clcB	2	24.6667	0.352
0.4	0.05	ycaM	2	24.6667	0.36
0.4	0.05	yadI	2	24.6667	0.818
0.4	0.05	AG1	2	24.6667	0.892
0.4	0.05	ptsI	2	24.9167	0.706
0.4	0.05	clcB	2	24.9167	0.352
0.4	0.05	ycaM	2	24.9167	0.36
0.4	0.05	yadI	2	24.9167	0.82
0.4	0.05	AG1	2	24.9167	0.9
0.4	0.05	ptsI	2	25.1667	0.703
0.4	0.05	clcB	2	25.1667	0.354
0.4	0.05	ycaM	2	25.1667	0.364
0.4	0.05	yadI	2	25.1667	0.825
0.4	0.05	AG1	2	25.1667	0.908
0.4	0.05	ptsI	2	25.4167	0.698
0.4	0.05	clcB	2	25.4167	0.357
0.4	0.05	ycaM	2	25.4167	0.366
0.4	0.05	yadI	2	25.4167	0.828
0.4	0.05	AG1	2	25.4167	0.914
0.4	0.05	ptsI	2	25.6667	0.698
0.4	0.05	clcB	2	25.6667	0.358
0.4	0.05	ycaM	2	25.6667	0.367
0.4	0.05	yadI	2	25.6667	0.835
0.4	0.05	AG1	2	25.6667	0.925
0.4	0.05	ptsI	2	25.9167	0.698
0.4	0.05	clcB	2	25.9167	0.364
0.4	0.05	ycaM	2	25.9167	0.374
0.4	0.05	yadI	2	25.9167	0.83
0.4	0.05	AG1	2	25.9167	0.935
0.4	0.05	ptsI	2	26.1667	0.688
0.4	0.05	clcB	2	26.1667	0.362
0.4	0.05	ycaM	2	26.1667	0.372
0.4	0.05	yadI	2	26.1667	0.827
0.4	0.05	AG1	2	26.1667	0.936
0.4	0.05	ptsI	2	26.4167	0.697
0.4	0.05	clcB	2	26.4167	0.362
0.4	0.05	ycaM	2	26.4167	0.379
0.4	0.05	yadI	2	26.4167	0.831
0.4	0.05	AG1	2	26.4167	0.957
0.4	0.05	ptsI	3	0	0.166
0.4	0.05	clcB	3	0	0.16
0.4	0.05	ycaM	3	0	0.158

0.4	0.05	yadI	3	0	0.158
0.4	0.05	AG1	3	0	0.159
0.4	0.05	ptsI	3	0.25	0.161
0.4	0.05	clcB	3	0.25	0.159
0.4	0.05	ycaM	3	0.25	0.154
0.4	0.05	yadI	3	0.25	0.155
0.4	0.05	AG1	3	0.25	0.155
0.4	0.05	ptsI	3	0.5	0.16
0.4	0.05	clcB	3	0.5	0.158
0.4	0.05	ycaM	3	0.5	0.153
0.4	0.05	yadI	3	0.5	0.153
0.4	0.05	AG1	3	0.5	0.155
0.4	0.05	ptsI	3	0.75	0.16
0.4	0.05	clcB	3	0.75	0.159
0.4	0.05	ycaM	3	0.75	0.152
0.4	0.05	yadI	3	0.75	0.169
0.4	0.05	AG1	3	0.75	0.155
0.4	0.05	ptsI	3	1	0.162
0.4	0.05	clcB	3	1	0.16
0.4	0.05	ycaM	3	1	0.152
0.4	0.05	yadI	3	1	0.153
0.4	0.05	AG1	3	1	0.155
0.4	0.05	ptsI	3	1.25	0.162
0.4	0.05	clcB	3	1.25	0.16
0.4	0.05	ycaM	3	1.25	0.152
0.4	0.05	yadI	3	1.25	0.153
0.4	0.05	AG1	3	1.25	0.154
0.4	0.05	ptsI	3	1.5	0.163
0.4	0.05	clcB	3	1.5	0.16
0.4	0.05	ycaM	3	1.5	0.153
0.4	0.05	yadI	3	1.5	0.154
0.4	0.05	AG1	3	1.5	0.155
0.4	0.05	ptsI	3	1.75	0.166
0.4	0.05	clcB	3	1.75	0.162
0.4	0.05	ycaM	3	1.75	0.152
0.4	0.05	yadI	3	1.75	0.155
0.4	0.05	AG1	3	1.75	0.155
0.4	0.05	ptsI	3	2	0.167
0.4	0.05	clcB	3	2	0.165
0.4	0.05	ycaM	3	2	0.154
0.4	0.05	yadI	3	2	0.157
0.4	0.05	AG1	3	2	0.157
0.4	0.05	ptsI	3	2.25	0.168
0.4	0.05	clcB	3	2.25	0.165
0.4	0.05	ycaM	3	2.25	0.155
0.4	0.05	yadI	3	2.25	0.158
0.4	0.05	AG1	3	2.25	0.158
0.4	0.05	ptsI	3	2.5	0.17
0.4	0.05	clcB	3	2.5	0.17
0.4	0.05	ycaM	3	2.5	0.157
0.4	0.05	yadI	3	2.5	0.16
0.4	0.05	AG1	3	2.5	0.16
0.4	0.05	ptsI	3	2.75	0.17

0.4	0.05	clcB	3	2.75	0.174
0.4	0.05	ycaM	3	2.75	0.159
0.4	0.05	yadI	3	2.75	0.163
0.4	0.05	AG1	3	2.75	0.161
0.4	0.05	ptsI	3	3	0.171
0.4	0.05	clcB	3	3	0.175
0.4	0.05	ycaM	3	3	0.159
0.4	0.05	yadI	3	3	0.166
0.4	0.05	AG1	3	3	0.163
0.4	0.05	ptsI	3	3.25	0.176
0.4	0.05	clcB	3	3.25	0.175
0.4	0.05	ycaM	3	3.25	0.161
0.4	0.05	yadI	3	3.25	0.168
0.4	0.05	AG1	3	3.25	0.164
0.4	0.05	ptsI	3	3.5	0.18
0.4	0.05	clcB	3	3.5	0.18
0.4	0.05	ycaM	3	3.5	0.164
0.4	0.05	yadI	3	3.5	0.173
0.4	0.05	AG1	3	3.5	0.166
0.4	0.05	ptsI	3	3.75	0.182
0.4	0.05	clcB	3	3.75	0.183
0.4	0.05	ycaM	3	3.75	0.166
0.4	0.05	yadI	3	3.75	0.178
0.4	0.05	AG1	3	3.75	0.168
0.4	0.05	ptsI	3	4	0.184
0.4	0.05	clcB	3	4	0.186
0.4	0.05	ycaM	3	4	0.168
0.4	0.05	yadI	3	4	0.183
0.4	0.05	AG1	3	4	0.17
0.4	0.05	ptsI	3	4.41667	0.206
0.4	0.05	clcB	3	4.41667	0.21
0.4	0.05	ycaM	3	4.41667	0.172
0.4	0.05	yadI	3	4.41667	0.214
0.4	0.05	AG1	3	4.41667	0.192
0.4	0.05	ptsI	3	4.66667	0.195
0.4	0.05	clcB	3	4.66667	0.186
0.4	0.05	ycaM	3	4.66667	0.17
0.4	0.05	yadI	3	4.66667	0.22
0.4	0.05	AG1	3	4.66667	0.192
0.4	0.05	ptsI	3	4.91667	0.192
0.4	0.05	clcB	3	4.91667	0.191
0.4	0.05	ycaM	3	4.91667	0.171
0.4	0.05	yadI	3	4.91667	0.24
0.4	0.05	AG1	3	4.91667	0.197
0.4	0.05	ptsI	3	5.16667	0.195
0.4	0.05	clcB	3	5.16667	0.196
0.4	0.05	ycaM	3	5.16667	0.175
0.4	0.05	yadI	3	5.16667	0.254
0.4	0.05	AG1	3	5.16667	0.202
0.4	0.05	ptsI	3	5.41667	0.2
0.4	0.05	clcB	3	5.41667	0.2
0.4	0.05	ycaM	3	5.41667	0.181
0.4	0.05	yadI	3	5.41667	0.262

0.4	0.05	AG1	3	5.41667	0.206
0.4	0.05	ptsI	3	5.66667	0.205
0.4	0.05	clcB	3	5.66667	0.208
0.4	0.05	ycaM	3	5.66667	0.187
0.4	0.05	yadI	3	5.66667	0.276
0.4	0.05	AG1	3	5.66667	0.212
0.4	0.05	ptsI	3	5.91667	0.208
0.4	0.05	clcB	3	5.91667	0.213
0.4	0.05	ycaM	3	5.91667	0.193
0.4	0.05	yadI	3	5.91667	0.282
0.4	0.05	AG1	3	5.91667	0.214
0.4	0.05	ptsI	3	6.16667	0.216
0.4	0.05	clcB	3	6.16667	0.219
0.4	0.05	ycaM	3	6.16667	0.201
0.4	0.05	yadI	3	6.16667	0.29
0.4	0.05	AG1	3	6.16667	0.221
0.4	0.05	ptsI	3	6.41667	0.223
0.4	0.05	clcB	3	6.41667	0.227
0.4	0.05	ycaM	3	6.41667	0.21
0.4	0.05	yadI	3	6.41667	0.299
0.4	0.05	AG1	3	6.41667	0.23
0.4	0.05	ptsI	3	6.66667	0.233
0.4	0.05	clcB	3	6.66667	0.235
0.4	0.05	ycaM	3	6.66667	0.219
0.4	0.05	yadI	3	6.66667	0.307
0.4	0.05	AG1	3	6.66667	0.24
0.4	0.05	ptsI	3	6.91667	0.243
0.4	0.05	clcB	3	6.91667	0.242
0.4	0.05	ycaM	3	6.91667	0.226
0.4	0.05	yadI	3	6.91667	0.315
0.4	0.05	AG1	3	6.91667	0.253
0.4	0.05	ptsI	3	7.16667	0.254
0.4	0.05	clcB	3	7.16667	0.254
0.4	0.05	ycaM	3	7.16667	0.233
0.4	0.05	yadI	3	7.16667	0.325
0.4	0.05	AG1	3	7.16667	0.26
0.4	0.05	ptsI	3	7.41667	0.263
0.4	0.05	clcB	3	7.41667	0.266
0.4	0.05	ycaM	3	7.41667	0.236
0.4	0.05	yadI	3	7.41667	0.336
0.4	0.05	AG1	3	7.41667	0.269
0.4	0.05	ptsI	3	7.66667	0.272
0.4	0.05	clcB	3	7.66667	0.274
0.4	0.05	ycaM	3	7.66667	0.24
0.4	0.05	yadI	3	7.66667	0.347
0.4	0.05	AG1	3	7.66667	0.274
0.4	0.05	ptsI	3	7.91667	0.278
0.4	0.05	clcB	3	7.91667	0.28
0.4	0.05	ycaM	3	7.91667	0.241
0.4	0.05	yadI	3	7.91667	0.357
0.4	0.05	AG1	3	7.91667	0.28
0.4	0.05	ptsI	3	8.16667	0.289
0.4	0.05	clcB	3	8.16667	0.286

0.4	0.05	ycaM	3	8.16667	0.244
0.4	0.05	yadI	3	8.16667	0.364
0.4	0.05	AG1	3	8.16667	0.288
0.4	0.05	ptsI	3	8.41667	0.298
0.4	0.05	clcB	3	8.41667	0.292
0.4	0.05	ycaM	3	8.41667	0.246
0.4	0.05	yadI	3	8.41667	0.373
0.4	0.05	AG1	3	8.41667	0.296
0.4	0.05	ptsI	3	9.05	0.29
0.4	0.05	clcB	3	9.05	0.281
0.4	0.05	ycaM	3	9.05	0.257
0.4	0.05	yadI	3	9.05	0.466
0.4	0.05	AG1	3	9.05	0.358
0.4	0.05	ptsI	3	9.3	0.296
0.4	0.05	clcB	3	9.3	0.281
0.4	0.05	ycaM	3	9.3	0.252
0.4	0.05	yadI	3	9.3	0.473
0.4	0.05	AG1	3	9.3	0.372
0.4	0.05	ptsI	3	9.55	0.305
0.4	0.05	clcB	3	9.55	0.289
0.4	0.05	ycaM	3	9.55	0.257
0.4	0.05	yadI	3	9.55	0.49
0.4	0.05	AG1	3	9.55	0.386
0.4	0.05	ptsI	3	9.8	0.31
0.4	0.05	clcB	3	9.8	0.29
0.4	0.05	ycaM	3	9.8	0.255
0.4	0.05	yadI	3	9.8	0.505
0.4	0.05	AG1	3	9.8	0.396
0.4	0.05	ptsI	3	10.05	0.321
0.4	0.05	clcB	3	10.05	0.295
0.4	0.05	ycaM	3	10.05	0.256
0.4	0.05	yadI	3	10.05	0.519
0.4	0.05	AG1	3	10.05	0.406
0.4	0.05	ptsI	3	10.3	0.326
0.4	0.05	clcB	3	10.3	0.297
0.4	0.05	ycaM	3	10.3	0.26
0.4	0.05	yadI	3	10.3	0.531
0.4	0.05	AG1	3	10.3	0.415
0.4	0.05	ptsI	3	10.55	0.337
0.4	0.05	clcB	3	10.55	0.303
0.4	0.05	ycaM	3	10.55	0.258
0.4	0.05	yadI	3	10.55	0.539
0.4	0.05	AG1	3	10.55	0.422
0.4	0.05	ptsI	3	10.8	0.343
0.4	0.05	clcB	3	10.8	0.301
0.4	0.05	ycaM	3	10.8	0.26
0.4	0.05	yadI	3	10.8	0.553
0.4	0.05	AG1	3	10.8	0.431
0.4	0.05	ptsI	3	11.05	0.356
0.4	0.05	clcB	3	11.05	0.306
0.4	0.05	ycaM	3	11.05	0.262
0.4	0.05	yadI	3	11.05	0.566
0.4	0.05	AG1	3	11.05	0.444

0.4	0.05	ptsI	3	11.3	0.364
0.4	0.05	clcB	3	11.3	0.308
0.4	0.05	ycaM	3	11.3	0.262
0.4	0.05	yadI	3	11.3	0.572
0.4	0.05	AG1	3	11.3	0.45
0.4	0.05	ptsI	3	11.55	0.374
0.4	0.05	clcB	3	11.55	0.313
0.4	0.05	ycaM	3	11.55	0.264
0.4	0.05	yadI	3	11.55	0.583
0.4	0.05	AG1	3	11.55	0.461
0.4	0.05	ptsI	3	11.8	0.384
0.4	0.05	clcB	3	11.8	0.305
0.4	0.05	ycaM	3	11.8	0.264
0.4	0.05	yadI	3	11.8	0.599
0.4	0.05	AG1	3	11.8	0.472
0.4	0.05	ptsI	3	12.05	0.391
0.4	0.05	clcB	3	12.05	0.299
0.4	0.05	ycaM	3	12.05	0.265
0.4	0.05	yadI	3	12.05	0.604
0.4	0.05	AG1	3	12.05	0.481
0.4	0.05	ptsI	3	12.3	0.4
0.4	0.05	clcB	3	12.3	0.305
0.4	0.05	ycaM	3	12.3	0.263
0.4	0.05	yadI	3	12.3	0.621
0.4	0.05	AG1	3	12.3	0.496
0.4	0.05	ptsI	3	12.55	0.406
0.4	0.05	clcB	3	12.55	0.302
0.4	0.05	ycaM	3	12.55	0.262
0.4	0.05	yadI	3	12.55	0.634
0.4	0.05	AG1	3	12.55	0.509
0.4	0.05	ptsI	3	12.8	0.414
0.4	0.05	clcB	3	12.8	0.305
0.4	0.05	ycaM	3	12.8	0.262
0.4	0.05	yadI	3	12.8	0.643
0.4	0.05	AG1	3	12.8	0.52
0.4	0.05	ptsI	3	13.05	0.42
0.4	0.05	clcB	3	13.05	0.296
0.4	0.05	ycaM	3	13.05	0.259
0.4	0.05	yadI	3	13.05	0.641
0.4	0.05	AG1	3	13.05	0.53
0.4	0.05	ptsI	3	13.3	0.43
0.4	0.05	clcB	3	13.3	0.298
0.4	0.05	ycaM	3	13.3	0.26
0.4	0.05	yadI	3	13.3	0.649
0.4	0.05	AG1	3	13.3	0.547
0.4	0.05	ptsI	3	13.55	0.439
0.4	0.05	clcB	3	13.55	0.292
0.4	0.05	ycaM	3	13.55	0.258
0.4	0.05	yadI	3	13.55	0.668
0.4	0.05	AG1	3	13.55	0.562
0.4	0.05	ptsI	3	13.8	0.443
0.4	0.05	clcB	3	13.8	0.29
0.4	0.05	ycaM	3	13.8	0.264

0.4	0.05	yadI	3	13.8	0.664
0.4	0.05	AG1	3	13.8	0.573
0.4	0.05	ptsI	3	14.05	0.45
0.4	0.05	clcB	3	14.05	0.29
0.4	0.05	ycaM	3	14.05	0.259
0.4	0.05	yadI	3	14.05	0.678
0.4	0.05	AG1	3	14.05	0.588
0.4	0.05	ptsI	3	14.3	0.453
0.4	0.05	clcB	3	14.3	0.286
0.4	0.05	ycaM	3	14.3	0.255
0.4	0.05	yadI	3	14.3	0.686
0.4	0.05	AG1	3	14.3	0.59
0.4	0.05	ptsI	3	14.55	0.462
0.4	0.05	clcB	3	14.55	0.285
0.4	0.05	ycaM	3	14.55	0.254
0.4	0.05	yadI	3	14.55	0.682
0.4	0.05	AG1	3	14.55	0.604
0.4	0.05	ptsI	3	14.8	0.471
0.4	0.05	clcB	3	14.8	0.286
0.4	0.05	ycaM	3	14.8	0.254
0.4	0.05	yadI	3	14.8	0.691
0.4	0.05	AG1	3	14.8	0.621
0.4	0.05	ptsI	3	15.05	0.476
0.4	0.05	clcB	3	15.05	0.288
0.4	0.05	ycaM	3	15.05	0.256
0.4	0.05	yadI	3	15.05	0.712
0.4	0.05	AG1	3	15.05	0.632
0.4	0.05	ptsI	3	15.3	0.479
0.4	0.05	clcB	3	15.3	0.288
0.4	0.05	ycaM	3	15.3	0.257
0.4	0.05	yadI	3	15.3	0.703
0.4	0.05	AG1	3	15.3	0.632
0.4	0.05	ptsI	3	15.55	0.484
0.4	0.05	clcB	3	15.55	0.285
0.4	0.05	ycaM	3	15.55	0.257
0.4	0.05	yadI	3	15.55	0.72
0.4	0.05	AG1	3	15.55	0.648
0.4	0.05	ptsI	3	15.8	0.49
0.4	0.05	clcB	3	15.8	0.284
0.4	0.05	ycaM	3	15.8	0.252
0.4	0.05	yadI	3	15.8	0.722
0.4	0.05	AG1	3	15.8	0.648
0.4	0.05	ptsI	3	16.05	0.49
0.4	0.05	clcB	3	16.05	0.284
0.4	0.05	ycaM	3	16.05	0.256
0.4	0.05	yadI	3	16.05	0.718
0.4	0.05	AG1	3	16.05	0.655
0.4	0.05	ptsI	3	16.3	0.494
0.4	0.05	clcB	3	16.3	0.284
0.4	0.05	ycaM	3	16.3	0.256
0.4	0.05	yadI	3	16.3	0.722
0.4	0.05	AG1	3	16.3	0.662
0.4	0.05	ptsI	3	16.55	0.501

0.4	0.05	clcB	3	16.55	0.286
0.4	0.05	ycaM	3	16.55	0.26
0.4	0.05	yadI	3	16.55	0.741
0.4	0.05	AG1	3	16.55	0.68
0.4	0.05	ptsI	3	16.8	0.503
0.4	0.05	clcB	3	16.8	0.286
0.4	0.05	ycaM	3	16.8	0.261
0.4	0.05	yadI	3	16.8	0.753
0.4	0.05	AG1	3	16.8	0.703
0.4	0.05	ptsI	3	17.05	0.509
0.4	0.05	clcB	3	17.05	0.284
0.4	0.05	ycaM	3	17.05	0.262
0.4	0.05	yadI	3	17.05	0.748
0.4	0.05	AG1	3	17.05	0.694
0.4	0.05	ptsI	3	17.3	0.517
0.4	0.05	clcB	3	17.3	0.282
0.4	0.05	ycaM	3	17.3	0.26
0.4	0.05	yadI	3	17.3	0.759
0.4	0.05	AG1	3	17.3	0.708
0.4	0.05	ptsI	3	17.55	0.52
0.4	0.05	clcB	3	17.55	0.281
0.4	0.05	ycaM	3	17.55	0.258
0.4	0.05	yadI	3	17.55	0.75
0.4	0.05	AG1	3	17.55	0.716
0.4	0.05	ptsI	3	17.8	0.524
0.4	0.05	clcB	3	17.8	0.28
0.4	0.05	ycaM	3	17.8	0.258
0.4	0.05	yadI	3	17.8	0.759
0.4	0.05	AG1	3	17.8	0.733
0.4	0.05	ptsI	3	18.05	0.525
0.4	0.05	clcB	3	18.05	0.283
0.4	0.05	ycaM	3	18.05	0.261
0.4	0.05	yadI	3	18.05	0.761
0.4	0.05	AG1	3	18.05	0.739
0.4	0.05	ptsI	3	18.3	0.531
0.4	0.05	clcB	3	18.3	0.283
0.4	0.05	ycaM	3	18.3	0.261
0.4	0.05	yadI	3	18.3	0.766
0.4	0.05	AG1	3	18.3	0.745
0.4	0.05	ptsI	3	18.55	0.538
0.4	0.05	clcB	3	18.55	0.283
0.4	0.05	ycaM	3	18.55	0.264
0.4	0.05	yadI	3	18.55	0.779
0.4	0.05	AG1	3	18.55	0.728
0.4	0.05	ptsI	3	18.8	0.541
0.4	0.05	clcB	3	18.8	0.282
0.4	0.05	ycaM	3	18.8	0.263
0.4	0.05	yadI	3	18.8	0.776
0.4	0.05	AG1	3	18.8	0.725
0.4	0.05	ptsI	3	19.05	0.543
0.4	0.05	clcB	3	19.05	0.284
0.4	0.05	ycaM	3	19.05	0.265
0.4	0.05	yadI	3	19.05	0.783

0.4	0.05	AG1	3	19.05	0.739
0.4	0.05	ptsI	3	19.3	0.543
0.4	0.05	clcB	3	19.3	0.284
0.4	0.05	ycaM	3	19.3	0.266
0.4	0.05	yadI	3	19.3	0.782
0.4	0.05	AG1	3	19.3	0.752
0.4	0.05	ptsI	3	19.55	0.549
0.4	0.05	clcB	3	19.55	0.283
0.4	0.05	ycaM	3	19.55	0.267
0.4	0.05	yadI	3	19.55	0.791
0.4	0.05	AG1	3	19.55	0.756
0.4	0.05	ptsI	3	19.8	0.548
0.4	0.05	clcB	3	19.8	0.284
0.4	0.05	ycaM	3	19.8	0.268
0.4	0.05	yadI	3	19.8	0.792
0.4	0.05	AG1	3	19.8	0.747
0.4	0.05	ptsI	3	20.05	0.549
0.4	0.05	clcB	3	20.05	0.285
0.4	0.05	ycaM	3	20.05	0.269
0.4	0.05	yadI	3	20.05	0.796
0.4	0.05	AG1	3	20.05	0.754
0.4	0.05	ptsI	3	20.3	0.553
0.4	0.05	clcB	3	20.3	0.287
0.4	0.05	ycaM	3	20.3	0.271
0.4	0.05	yadI	3	20.3	0.801
0.4	0.05	AG1	3	20.3	0.747
0.4	0.05	ptsI	3	20.55	0.55
0.4	0.05	clcB	3	20.55	0.287
0.4	0.05	ycaM	3	20.55	0.276
0.4	0.05	yadI	3	20.55	0.795
0.4	0.05	AG1	3	20.55	0.75
0.4	0.05	ptsI	3	20.8	0.552
0.4	0.05	clcB	3	20.8	0.291
0.4	0.05	ycaM	3	20.8	0.277
0.4	0.05	yadI	3	20.8	0.805
0.4	0.05	AG1	3	20.8	0.758
0.4	0.05	ptsI	3	21.05	0.555
0.4	0.05	clcB	3	21.05	0.291
0.4	0.05	ycaM	3	21.05	0.279
0.4	0.05	yadI	3	21.05	0.803
0.4	0.05	AG1	3	21.05	0.757
0.4	0.05	ptsI	3	21.3	0.552
0.4	0.05	clcB	3	21.3	0.291
0.4	0.05	ycaM	3	21.3	0.281
0.4	0.05	yadI	3	21.3	0.805
0.4	0.05	AG1	3	21.3	0.77
0.4	0.05	ptsI	3	21.55	0.55
0.4	0.05	clcB	3	21.55	0.292
0.4	0.05	ycaM	3	21.55	0.283
0.4	0.05	yadI	3	21.55	0.815
0.4	0.05	AG1	3	21.55	0.77
0.4	0.05	ptsI	3	21.8	0.549
0.4	0.05	clcB	3	21.8	0.295

0.4	0.05	ycaM	3	21.8	0.285
0.4	0.05	yadI	3	21.8	0.813
0.4	0.05	AG1	3	21.8	0.78
0.4	0.05	ptsI	3	22.05	0.545
0.4	0.05	clcB	3	22.05	0.296
0.4	0.05	ycaM	3	22.05	0.286
0.4	0.05	yadI	3	22.05	0.813
0.4	0.05	AG1	3	22.05	0.79
0.4	0.05	ptsI	3	22.3	0.543
0.4	0.05	clcB	3	22.3	0.296
0.4	0.05	ycaM	3	22.3	0.288
0.4	0.05	yadI	3	22.3	0.817
0.4	0.05	AG1	3	22.3	0.791
0.4	0.05	ptsI	3	22.55	0.539
0.4	0.05	clcB	3	22.55	0.301
0.4	0.05	ycaM	3	22.55	0.294
0.4	0.05	yadI	3	22.55	0.816
0.4	0.05	AG1	3	22.55	0.797
0.4	0.05	ptsI	3	22.8	0.537
0.4	0.05	clcB	3	22.8	0.299
0.4	0.05	ycaM	3	22.8	0.296
0.4	0.05	yadI	3	22.8	0.817
0.4	0.05	AG1	3	22.8	0.809
0.4	0.05	ptsI	3	23.05	0.539
0.4	0.05	clcB	3	23.05	0.301
0.4	0.05	ycaM	3	23.05	0.299
0.4	0.05	yadI	3	23.05	0.818
0.4	0.05	AG1	3	23.05	0.812
0.4	0.05	ptsI	3	23.3	0.535
0.4	0.05	clcB	3	23.3	0.302
0.4	0.05	ycaM	3	23.3	0.3
0.4	0.05	yadI	3	23.3	0.821
0.4	0.05	AG1	3	23.3	0.821
0.4	0.05	ptsI	3	23.55	0.535
0.4	0.05	clcB	3	23.55	0.303
0.4	0.05	ycaM	3	23.55	0.305
0.4	0.05	yadI	3	23.55	0.835
0.4	0.05	AG1	3	23.55	0.83
0.4	0.05	ptsI	3	23.8	0.538
0.4	0.05	clcB	3	23.8	0.303
0.4	0.05	ycaM	3	23.8	0.307
0.4	0.05	yadI	3	23.8	0.838
0.4	0.05	AG1	3	23.8	0.834
0.4	0.05	ptsI	3	24.05	0.537
0.4	0.05	clcB	3	24.05	0.306
0.4	0.05	ycaM	3	24.05	0.313
0.4	0.05	yadI	3	24.05	0.836
0.4	0.05	AG1	3	24.05	0.843
0.4	0.05	ptsI	3	24.3	0.535
0.4	0.05	clcB	3	24.3	0.308
0.4	0.05	ycaM	3	24.3	0.316
0.4	0.05	yadI	3	24.3	0.837
0.4	0.05	AG1	3	24.3	0.842

0.4	0.05	ptsI	4	0	0.186
0.4	0.05	clcB	4	0	0.203
0.4	0.05	ycaM	4	0	0.353
0.4	0.05	yadI	4	0	0.208
0.4	0.05	AG1	4	0	0.2
0.4	0.05	ptsI	4	0.25	0.186
0.4	0.05	clcB	4	0.25	0.2
0.4	0.05	ycaM	4	0.25	0.346
0.4	0.05	yadI	4	0.25	0.206
0.4	0.05	AG1	4	0.25	0.196
0.4	0.05	ptsI	4	0.5	0.186
0.4	0.05	clcB	4	0.5	0.201
0.4	0.05	ycaM	4	0.5	0.338
0.4	0.05	yadI	4	0.5	0.207
0.4	0.05	AG1	4	0.5	0.196
0.4	0.05	ptsI	4	0.75	0.184
0.4	0.05	clcB	4	0.75	0.204
0.4	0.05	ycaM	4	0.75	0.338
0.4	0.05	yadI	4	0.75	0.212
0.4	0.05	AG1	4	0.75	0.2
0.4	0.05	ptsI	4	1	0.188
0.4	0.05	clcB	4	1	0.206
0.4	0.05	ycaM	4	1	0.336
0.4	0.05	yadI	4	1	0.213
0.4	0.05	AG1	4	1	0.202
0.4	0.05	ptsI	4	1.25	0.194
0.4	0.05	clcB	4	1.25	0.21
0.4	0.05	ycaM	4	1.25	0.344
0.4	0.05	yadI	4	1.25	0.218
0.4	0.05	AG1	4	1.25	0.205
0.4	0.05	ptsI	4	1.5	0.193
0.4	0.05	clcB	4	1.5	0.208
0.4	0.05	ycaM	4	1.5	0.358
0.4	0.05	yadI	4	1.5	0.219
0.4	0.05	AG1	4	1.5	0.205
0.4	0.05	ptsI	4	1.75	0.203
0.4	0.05	clcB	4	1.75	0.218
0.4	0.05	ycaM	4	1.75	0.368
0.4	0.05	yadI	4	1.75	0.225
0.4	0.05	AG1	4	1.75	0.213
0.4	0.05	ptsI	4	2	0.211
0.4	0.05	clcB	4	2	0.226
0.4	0.05	ycaM	4	2	0.376
0.4	0.05	yadI	4	2	0.231
0.4	0.05	AG1	4	2	0.222
0.4	0.05	ptsI	4	2.25	0.22
0.4	0.05	clcB	4	2.25	0.233
0.4	0.05	ycaM	4	2.25	0.378
0.4	0.05	yadI	4	2.25	0.241
0.4	0.05	AG1	4	2.25	0.23
0.4	0.05	ptsI	4	2.5	0.224
0.4	0.05	clcB	4	2.5	0.238
0.4	0.05	ycaM	4	2.5	0.386

0.4	0.05	yadI	4	2.5	0.246
0.4	0.05	AG1	4	2.5	0.233
0.4	0.05	ptsI	4	2.75	0.229
0.4	0.05	clcB	4	2.75	0.248
0.4	0.05	ycaM	4	2.75	0.462
0.4	0.05	yadI	4	2.75	0.252
0.4	0.05	AG1	4	2.75	0.241
0.4	0.05	ptsI	4	3	0.242
0.4	0.05	clcB	4	3	0.256
0.4	0.05	ycaM	4	3	0.403
0.4	0.05	yadI	4	3	0.262
0.4	0.05	AG1	4	3	0.249
0.4	0.05	ptsI	4	3.25	0.251
0.4	0.05	clcB	4	3.25	0.268
0.4	0.05	ycaM	4	3.25	0.418
0.4	0.05	yadI	4	3.25	0.269
0.4	0.05	AG1	4	3.25	0.256
0.4	0.05	ptsI	4	3.5	0.264
0.4	0.05	clcB	4	3.5	0.281
0.4	0.05	ycaM	4	3.5	0.426
0.4	0.05	yadI	4	3.5	0.282
0.4	0.05	AG1	4	3.5	0.268
0.4	0.05	ptsI	4	3.75	0.273
0.4	0.05	clcB	4	3.75	0.29
0.4	0.05	ycaM	4	3.75	0.507
0.4	0.05	yadI	4	3.75	0.29
0.4	0.05	AG1	4	3.75	0.276
0.4	0.05	ptsI	4	4	0.292
0.4	0.05	clcB	4	4	0.301
0.4	0.05	ycaM	4	4	0.444
0.4	0.05	yadI	4	4	0.301
0.4	0.05	AG1	4	4	0.288
0.4	0.05	ptsI	4	4.35	0.34
0.4	0.05	clcB	4	4.35	0.366
0.4	0.05	ycaM	4	4.35	0.474
0.4	0.05	yadI	4	4.35	0.362
0.4	0.05	AG1	4	4.35	0.277
0.4	0.05	ptsI	4	4.6	0.307
0.4	0.05	clcB	4	4.6	0.344
0.4	0.05	ycaM	4	4.6	0.43
0.4	0.05	yadI	4	4.6	0.359
0.4	0.05	AG1	4	4.6	0.287
0.4	0.05	ptsI	4	4.85	0.304
0.4	0.05	clcB	4	4.85	0.35
0.4	0.05	ycaM	4	4.85	0.424
0.4	0.05	yadI	4	4.85	0.376
0.4	0.05	AG1	4	4.85	0.301
0.4	0.05	ptsI	4	5.1	0.309
0.4	0.05	clcB	4	5.1	0.352
0.4	0.05	ycaM	4	5.1	0.428
0.4	0.05	yadI	4	5.1	0.389
0.4	0.05	AG1	4	5.1	0.312
0.4	0.05	ptsI	4	5.35	0.319

0.4	0.05	clcB	4	5.35	0.356
0.4	0.05	ycaM	4	5.35	0.431
0.4	0.05	yadI	4	5.35	0.406
0.4	0.05	AG1	4	5.35	0.322
0.4	0.05	ptsI	4	5.6	0.321
0.4	0.05	clcB	4	5.6	0.353
0.4	0.05	ycaM	4	5.6	0.429
0.4	0.05	yadI	4	5.6	0.414
0.4	0.05	AG1	4	5.6	0.331
0.4	0.05	ptsI	4	5.85	0.328
0.4	0.05	clcB	4	5.85	0.354
0.4	0.05	ycaM	4	5.85	0.437
0.4	0.05	yadI	4	5.85	0.427
0.4	0.05	AG1	4	5.85	0.335
0.4	0.05	ptsI	4	6.1	0.34
0.4	0.05	clcB	4	6.1	0.361
0.4	0.05	ycaM	4	6.1	0.441
0.4	0.05	yadI	4	6.1	0.44
0.4	0.05	AG1	4	6.1	0.346
0.4	0.05	ptsI	4	6.35	0.352
0.4	0.05	clcB	4	6.35	0.364
0.4	0.05	ycaM	4	6.35	0.444
0.4	0.05	yadI	4	6.35	0.45
0.4	0.05	AG1	4	6.35	0.354
0.4	0.05	ptsI	4	6.6	0.363
0.4	0.05	clcB	4	6.6	0.366
0.4	0.05	ycaM	4	6.6	0.447
0.4	0.05	yadI	4	6.6	0.457
0.4	0.05	AG1	4	6.6	0.364
0.4	0.05	ptsI	4	6.85	0.376
0.4	0.05	clcB	4	6.85	0.367
0.4	0.05	ycaM	4	6.85	0.448
0.4	0.05	yadI	4	6.85	0.465
0.4	0.05	AG1	4	6.85	0.374
0.4	0.05	ptsI	4	7.1	0.384
0.4	0.05	clcB	4	7.1	0.369
0.4	0.05	ycaM	4	7.1	0.448
0.4	0.05	yadI	4	7.1	0.472
0.4	0.05	AG1	4	7.1	0.383
0.4	0.05	ptsI	4	7.35	0.393
0.4	0.05	clcB	4	7.35	0.37
0.4	0.05	ycaM	4	7.35	0.45
0.4	0.05	yadI	4	7.35	0.48
0.4	0.05	AG1	4	7.35	0.393
0.4	0.05	ptsI	4	7.6	0.401
0.4	0.05	clcB	4	7.6	0.371
0.4	0.05	ycaM	4	7.6	0.448
0.4	0.05	yadI	4	7.6	0.488
0.4	0.05	AG1	4	7.6	0.401
0.4	0.05	ptsI	4	7.85	0.403
0.4	0.05	clcB	4	7.85	0.371
0.4	0.05	ycaM	4	7.85	0.446
0.4	0.05	yadI	4	7.85	0.49

0.4	0.05	AG1	4	7.85	0.408
0.4	0.05	ptsI	4	8.1	0.413
0.4	0.05	clcB	4	8.1	0.374
0.4	0.05	ycaM	4	8.1	0.453
0.4	0.05	yadI	4	8.1	0.496
0.4	0.05	AG1	4	8.1	0.416
0.4	0.05	ptsI	4	8.35	0.424
0.4	0.05	clcB	4	8.35	0.379
0.4	0.05	ycaM	4	8.35	0.455
0.4	0.05	yadI	4	8.35	0.503
0.4	0.05	AG1	4	8.35	0.427
0.4	0.05	ptsI	4	8.83333	0.422
0.4	0.05	clcB	4	8.83333	0.312
0.4	0.05	ycaM	4	8.83333	0.417
0.4	0.05	yadI	4	8.83333	0.498
0.4	0.05	AG1	4	8.83333	0.452
0.4	0.05	ptsI	4	9.08333	0.422
0.4	0.05	clcB	4	9.08333	0.303
0.4	0.05	ycaM	4	9.08333	0.383
0.4	0.05	yadI	4	9.08333	0.496
0.4	0.05	AG1	4	9.08333	0.454
0.4	0.05	ptsI	4	9.33333	0.431
0.4	0.05	clcB	4	9.33333	0.3
0.4	0.05	ycaM	4	9.33333	0.364
0.4	0.05	yadI	4	9.33333	0.503
0.4	0.05	AG1	4	9.33333	0.47
0.4	0.05	ptsI	4	9.58333	0.441
0.4	0.05	clcB	4	9.58333	0.302
0.4	0.05	ycaM	4	9.58333	0.351
0.4	0.05	yadI	4	9.58333	0.514
0.4	0.05	AG1	4	9.58333	0.48
0.4	0.05	ptsI	4	9.83333	0.45
0.4	0.05	clcB	4	9.83333	0.302
0.4	0.05	ycaM	4	9.83333	0.338
0.4	0.05	yadI	4	9.83333	0.525
0.4	0.05	AG1	4	9.83333	0.487
0.4	0.05	ptsI	4	10.0833	0.457
0.4	0.05	clcB	4	10.0833	0.301
0.4	0.05	ycaM	4	10.0833	0.325
0.4	0.05	yadI	4	10.0833	0.53
0.4	0.05	AG1	4	10.0833	0.493
0.4	0.05	ptsI	4	10.3333	0.468
0.4	0.05	clcB	4	10.3333	0.304
0.4	0.05	ycaM	4	10.3333	0.314
0.4	0.05	yadI	4	10.3333	0.537
0.4	0.05	AG1	4	10.3333	0.502
0.4	0.05	ptsI	4	10.5833	0.475
0.4	0.05	clcB	4	10.5833	0.302
0.4	0.05	ycaM	4	10.5833	0.3
0.4	0.05	yadI	4	10.5833	0.545
0.4	0.05	AG1	4	10.5833	0.508
0.4	0.05	ptsI	4	10.8333	0.482
0.4	0.05	clcB	4	10.8333	0.302

0.4	0.05	ycaM	4	10.8333	0.297
0.4	0.05	yadI	4	10.8333	0.55
0.4	0.05	AG1	4	10.8333	0.513
0.4	0.05	ptsI	4	11.0833	0.487
0.4	0.05	clcB	4	11.0833	0.302
0.4	0.05	ycaM	4	11.0833	0.293
0.4	0.05	yadI	4	11.0833	0.558
0.4	0.05	AG1	4	11.0833	0.518
0.4	0.05	ptsI	4	11.3333	0.497
0.4	0.05	clcB	4	11.3333	0.305
0.4	0.05	ycaM	4	11.3333	0.298
0.4	0.05	yadI	4	11.3333	0.562
0.4	0.05	AG1	4	11.3333	0.524
0.4	0.05	ptsI	4	11.5833	0.504
0.4	0.05	clcB	4	11.5833	0.3
0.4	0.05	ycaM	4	11.5833	0.293
0.4	0.05	yadI	4	11.5833	0.569
0.4	0.05	AG1	4	11.5833	0.528
0.4	0.05	ptsI	4	11.8333	0.507
0.4	0.05	clcB	4	11.8333	0.304
0.4	0.05	ycaM	4	11.8333	0.29
0.4	0.05	yadI	4	11.8333	0.572
0.4	0.05	AG1	4	11.8333	0.53
0.4	0.05	ptsI	4	12.0833	0.517
0.4	0.05	clcB	4	12.0833	0.313
0.4	0.05	ycaM	4	12.0833	0.29
0.4	0.05	yadI	4	12.0833	0.575
0.4	0.05	AG1	4	12.0833	0.536
0.4	0.05	ptsI	4	12.3333	0.523
0.4	0.05	clcB	4	12.3333	0.312
0.4	0.05	ycaM	4	12.3333	0.299
0.4	0.05	yadI	4	12.3333	0.581
0.4	0.05	AG1	4	12.3333	0.54
0.4	0.05	ptsI	4	12.5833	0.533
0.4	0.05	clcB	4	12.5833	0.319
0.4	0.05	ycaM	4	12.5833	0.295
0.4	0.05	yadI	4	12.5833	0.586
0.4	0.05	AG1	4	12.5833	0.544
0.4	0.05	ptsI	4	12.8333	0.54
0.4	0.05	clcB	4	12.8333	0.316
0.4	0.05	ycaM	4	12.8333	0.296
0.4	0.05	yadI	4	12.8333	0.592
0.4	0.05	AG1	4	12.8333	0.547
0.4	0.05	ptsI	4	13.0833	0.549
0.4	0.05	clcB	4	13.0833	0.315
0.4	0.05	ycaM	4	13.0833	0.298
0.4	0.05	yadI	4	13.0833	0.596
0.4	0.05	AG1	4	13.0833	0.555
0.4	0.05	ptsI	4	13.3333	0.556
0.4	0.05	clcB	4	13.3333	0.326
0.4	0.05	ycaM	4	13.3333	0.3
0.4	0.05	yadI	4	13.3333	0.598
0.4	0.05	AG1	4	13.3333	0.561

0.4	0.05	ptsI	4	13.5833	0.562
0.4	0.05	clcB	4	13.5833	0.326
0.4	0.05	ycaM	4	13.5833	0.3
0.4	0.05	yadI	4	13.5833	0.604
0.4	0.05	AG1	4	13.5833	0.564
0.4	0.05	ptsI	4	13.8333	0.566
0.4	0.05	clcB	4	13.8333	0.328
0.4	0.05	ycaM	4	13.8333	0.303
0.4	0.05	yadI	4	13.8333	0.607
0.4	0.05	AG1	4	13.8333	0.569
0.4	0.05	ptsI	4	14.0833	0.569
0.4	0.05	clcB	4	14.0833	0.326
0.4	0.05	ycaM	4	14.0833	0.303
0.4	0.05	yadI	4	14.0833	0.614
0.4	0.05	AG1	4	14.0833	0.573
0.4	0.05	ptsI	4	14.3333	0.577
0.4	0.05	clcB	4	14.3333	0.32
0.4	0.05	ycaM	4	14.3333	0.302
0.4	0.05	yadI	4	14.3333	0.611
0.4	0.05	AG1	4	14.3333	0.577
0.4	0.05	ptsI	4	14.5833	0.58
0.4	0.05	clcB	4	14.5833	0.325
0.4	0.05	ycaM	4	14.5833	0.307
0.4	0.05	yadI	4	14.5833	0.617
0.4	0.05	AG1	4	14.5833	0.579
0.4	0.05	ptsI	4	14.8333	0.587
0.4	0.05	clcB	4	14.8333	0.324
0.4	0.05	ycaM	4	14.8333	0.304
0.4	0.05	yadI	4	14.8333	0.62
0.4	0.05	AG1	4	14.8333	0.582
0.4	0.05	ptsI	4	15.0833	0.59
0.4	0.05	clcB	4	15.0833	0.323
0.4	0.05	ycaM	4	15.0833	0.302
0.4	0.05	yadI	4	15.0833	0.624
0.4	0.05	AG1	4	15.0833	0.587
0.4	0.05	ptsI	4	15.3333	0.595
0.4	0.05	clcB	4	15.3333	0.327
0.4	0.05	ycaM	4	15.3333	0.305
0.4	0.05	yadI	4	15.3333	0.624
0.4	0.05	AG1	4	15.3333	0.588
0.4	0.05	ptsI	4	15.5833	0.602
0.4	0.05	clcB	4	15.5833	0.327
0.4	0.05	ycaM	4	15.5833	0.308
0.4	0.05	yadI	4	15.5833	0.627
0.4	0.05	AG1	4	15.5833	0.592
0.4	0.05	ptsI	4	15.8333	0.604
0.4	0.05	clcB	4	15.8333	0.327
0.4	0.05	ycaM	4	15.8333	0.306
0.4	0.05	yadI	4	15.8333	0.633
0.4	0.05	AG1	4	15.8333	0.594
0.4	0.05	ptsI	4	16.0833	0.609
0.4	0.05	clcB	4	16.0833	0.328
0.4	0.05	ycaM	4	16.0833	0.324

0.4	0.05	yadI	4	16.0833	0.635
0.4	0.05	AG1	4	16.0833	0.6
0.4	0.05	ptsI	4	16.3333	0.61
0.4	0.05	clcB	4	16.3333	0.332
0.4	0.05	ycaM	4	16.3333	0.33
0.4	0.05	yadI	4	16.3333	0.636
0.4	0.05	AG1	4	16.3333	0.602
0.4	0.05	ptsI	4	16.5833	0.612
0.4	0.05	clcB	4	16.5833	0.331
0.4	0.05	ycaM	4	16.5833	0.333
0.4	0.05	yadI	4	16.5833	0.64
0.4	0.05	AG1	4	16.5833	0.606
0.4	0.05	ptsI	4	16.8333	0.615
0.4	0.05	clcB	4	16.8333	0.33
0.4	0.05	ycaM	4	16.8333	0.323
0.4	0.05	yadI	4	16.8333	0.645
0.4	0.05	AG1	4	16.8333	0.608
0.4	0.05	ptsI	4	17.0833	0.614
0.4	0.05	clcB	4	17.0833	0.333
0.4	0.05	ycaM	4	17.0833	0.323
0.4	0.05	yadI	4	17.0833	0.644
0.4	0.05	AG1	4	17.0833	0.607
0.4	0.05	ptsI	4	17.3333	0.617
0.4	0.05	clcB	4	17.3333	0.34
0.4	0.05	ycaM	4	17.3333	0.327
0.4	0.05	yadI	4	17.3333	0.648
0.4	0.05	AG1	4	17.3333	0.612
0.4	0.05	ptsI	4	17.5833	0.62
0.4	0.05	clcB	4	17.5833	0.342
0.4	0.05	ycaM	4	17.5833	0.327
0.4	0.05	yadI	4	17.5833	0.649
0.4	0.05	AG1	4	17.5833	0.617
0.4	0.05	ptsI	4	17.8333	0.619
0.4	0.05	clcB	4	17.8333	0.335
0.4	0.05	ycaM	4	17.8333	0.325
0.4	0.05	yadI	4	17.8333	0.648
0.4	0.05	AG1	4	17.8333	0.62
0.4	0.05	ptsI	4	18.0833	0.619
0.4	0.05	clcB	4	18.0833	0.342
0.4	0.05	ycaM	4	18.0833	0.333
0.4	0.05	yadI	4	18.0833	0.654
0.4	0.05	AG1	4	18.0833	0.62
0.4	0.05	ptsI	4	18.3333	0.622
0.4	0.05	clcB	4	18.3333	0.337
0.4	0.05	ycaM	4	18.3333	0.338
0.4	0.05	yadI	4	18.3333	0.66
0.4	0.05	AG1	4	18.3333	0.623
0.4	0.05	ptsI	4	18.5833	0.62
0.4	0.05	clcB	4	18.5833	0.331
0.4	0.05	ycaM	4	18.5833	0.341
0.4	0.05	yadI	4	18.5833	0.66
0.4	0.05	AG1	4	18.5833	0.624
0.4	0.05	ptsI	4	18.8333	0.62

0.4	0.05	clcB	4	18.8333	0.323
0.4	0.05	ycaM	4	18.8333	0.336
0.4	0.05	yadI	4	18.8333	0.66
0.4	0.05	AG1	4	18.8333	0.628
0.4	0.05	ptsI	4	19.0833	0.624
0.4	0.05	clcB	4	19.0833	0.326
0.4	0.05	ycaM	4	19.0833	0.348
0.4	0.05	yadI	4	19.0833	0.664
0.4	0.05	AG1	4	19.0833	0.629
0.4	0.05	ptsI	4	19.3333	0.622
0.4	0.05	clcB	4	19.3333	0.324
0.4	0.05	ycaM	4	19.3333	0.345
0.4	0.05	yadI	4	19.3333	0.66
0.4	0.05	AG1	4	19.3333	0.628
0.4	0.05	ptsI	4	19.5833	0.624
0.4	0.05	clcB	4	19.5833	0.321
0.4	0.05	ycaM	4	19.5833	0.347
0.4	0.05	yadI	4	19.5833	0.662
0.4	0.05	AG1	4	19.5833	0.628
0.4	0.05	ptsI	4	19.8333	0.624
0.4	0.05	clcB	4	19.8333	0.318
0.4	0.05	ycaM	4	19.8333	0.349
0.4	0.05	yadI	4	19.8333	0.665
0.4	0.05	AG1	4	19.8333	0.634
0.4	0.05	ptsI	4	20.0833	0.635
0.4	0.05	clcB	4	20.0833	0.32
0.4	0.05	ycaM	4	20.0833	0.363
0.4	0.05	yadI	4	20.0833	0.664
0.4	0.05	AG1	4	20.0833	0.636
0.4	0.05	ptsI	4	20.3333	0.63
0.4	0.05	clcB	4	20.3333	0.316
0.4	0.05	ycaM	4	20.3333	0.349
0.4	0.05	yadI	4	20.3333	0.664
0.4	0.05	AG1	4	20.3333	0.633
0.4	0.05	ptsI	4	20.5833	0.628
0.4	0.05	clcB	4	20.5833	0.314
0.4	0.05	ycaM	4	20.5833	0.352
0.4	0.05	yadI	4	20.5833	0.671
0.4	0.05	AG1	4	20.5833	0.641
0.4	0.05	ptsI	4	20.8333	0.628
0.4	0.05	clcB	4	20.8333	0.318
0.4	0.05	ycaM	4	20.8333	0.356
0.4	0.05	yadI	4	20.8333	0.67
0.4	0.05	AG1	4	20.8333	0.638
0.4	0.05	ptsI	4	21.0833	0.627
0.4	0.05	clcB	4	21.0833	0.316
0.4	0.05	ycaM	4	21.0833	0.357
0.4	0.05	yadI	4	21.0833	0.67
0.4	0.05	AG1	4	21.0833	0.642
0.4	0.05	ptsI	4	21.3333	0.629
0.4	0.05	clcB	4	21.3333	0.32
0.4	0.05	ycaM	4	21.3333	0.357
0.4	0.05	yadI	4	21.3333	0.67

0.4	0.05	AG1	4	21.3333	0.643
0.4	0.05	ptsI	4	21.5833	0.628
0.4	0.05	clcB	4	21.5833	0.318
0.4	0.05	ycaM	4	21.5833	0.357
0.4	0.05	yadI	4	21.5833	0.673
0.4	0.05	AG1	4	21.5833	0.645
0.4	0.05	ptsI	4	21.8333	0.63
0.4	0.05	clcB	4	21.8333	0.319
0.4	0.05	ycaM	4	21.8333	0.363
0.4	0.05	yadI	4	21.8333	0.672
0.4	0.05	AG1	4	21.8333	0.644
0.4	0.05	ptsI	4	22.0833	0.624
0.4	0.05	clcB	4	22.0833	0.319
0.4	0.05	ycaM	4	22.0833	0.36
0.4	0.05	yadI	4	22.0833	0.674
0.4	0.05	AG1	4	22.0833	0.648
0.4	0.05	ptsI	4	22.3333	0.627
0.4	0.05	clcB	4	22.3333	0.32
0.4	0.05	ycaM	4	22.3333	0.359
0.4	0.05	yadI	4	22.3333	0.674
0.4	0.05	AG1	4	22.3333	0.648
0.4	0.05	ptsI	4	22.5833	0.628
0.4	0.05	clcB	4	22.5833	0.319
0.4	0.05	ycaM	4	22.5833	0.361
0.4	0.05	yadI	4	22.5833	0.68
0.4	0.05	AG1	4	22.5833	0.651
0.4	0.05	ptsI	4	22.8333	0.625
0.4	0.05	clcB	4	22.8333	0.321
0.4	0.05	ycaM	4	22.8333	0.36
0.4	0.05	yadI	4	22.8333	0.68
0.4	0.05	AG1	4	22.8333	0.655
0.4	0.05	ptsI	4	23.0833	0.625
0.4	0.05	clcB	4	23.0833	0.324
0.4	0.05	ycaM	4	23.0833	0.363
0.4	0.05	yadI	4	23.0833	0.68
0.4	0.05	AG1	4	23.0833	0.653
0.4	0.05	ptsI	4	23.3333	0.627
0.4	0.05	clcB	4	23.3333	0.327
0.4	0.05	ycaM	4	23.3333	0.366
0.4	0.05	yadI	4	23.3333	0.683
0.4	0.05	AG1	4	23.3333	0.657
0.4	0.05	ptsI	4	23.5833	0.63
0.4	0.05	clcB	4	23.5833	0.327
0.4	0.05	ycaM	4	23.5833	0.367
0.4	0.05	yadI	4	23.5833	0.684
0.4	0.05	AG1	4	23.5833	0.658
0.4	0.05	ptsI	4	23.8333	0.626
0.4	0.05	clcB	4	23.8333	0.33
0.4	0.05	ycaM	4	23.8333	0.37
0.4	0.05	yadI	4	23.8333	0.682
0.4	0.05	AG1	4	23.8333	0.658
0.4	0.05	ptsI	4	24.0833	0.628
0.4	0.05	clcB	4	24.0833	0.332

0.4	0.05	ycaM	4	24.0833	0.371
0.4	0.05	yadI	4	24.0833	0.682
0.4	0.05	AG1	4	24.0833	0.661
0.4	0.05	ptsI	5	0	0.165
0.4	0.05	clcB	5	0	0.168
0.4	0.05	ycaM	5	0	0.177
0.4	0.05	yadI	5	0	0.17
0.4	0.05	AG1	5	0	0.161
0.4	0.05	ptsI	5	0.35	0.162
0.4	0.05	clcB	5	0.35	0.164
0.4	0.05	ycaM	5	0.35	0.171
0.4	0.05	yadI	5	0.35	0.163
0.4	0.05	AG1	5	0.35	0.158
0.4	0.05	ptsI	5	0.6	0.161
0.4	0.05	clcB	5	0.6	0.164
0.4	0.05	ycaM	5	0.6	0.17
0.4	0.05	yadI	5	0.6	0.164
0.4	0.05	AG1	5	0.6	0.159
0.4	0.05	ptsI	5	0.85	0.16
0.4	0.05	clcB	5	0.85	0.163
0.4	0.05	ycaM	5	0.85	0.169
0.4	0.05	yadI	5	0.85	0.163
0.4	0.05	AG1	5	0.85	0.156
0.4	0.05	ptsI	5	1.1	0.162
0.4	0.05	clcB	5	1.1	0.165
0.4	0.05	ycaM	5	1.1	0.171
0.4	0.05	yadI	5	1.1	0.165
0.4	0.05	AG1	5	1.1	0.159
0.4	0.05	ptsI	5	1.35	0.163
0.4	0.05	clcB	5	1.35	0.166
0.4	0.05	ycaM	5	1.35	0.172
0.4	0.05	yadI	5	1.35	0.167
0.4	0.05	AG1	5	1.35	0.16
0.4	0.05	ptsI	5	1.6	0.164
0.4	0.05	clcB	5	1.6	0.168
0.4	0.05	ycaM	5	1.6	0.174
0.4	0.05	yadI	5	1.6	0.169
0.4	0.05	AG1	5	1.6	0.162
0.4	0.05	ptsI	5	1.85	0.167
0.4	0.05	clcB	5	1.85	0.171
0.4	0.05	ycaM	5	1.85	0.176
0.4	0.05	yadI	5	1.85	0.171
0.4	0.05	AG1	5	1.85	0.164
0.4	0.05	ptsI	5	2.1	0.17
0.4	0.05	clcB	5	2.1	0.176
0.4	0.05	ycaM	5	2.1	0.179
0.4	0.05	yadI	5	2.1	0.175
0.4	0.05	AG1	5	2.1	0.169
0.4	0.05	ptsI	5	2.35	0.173
0.4	0.05	clcB	5	2.35	0.18
0.4	0.05	ycaM	5	2.35	0.184
0.4	0.05	yadI	5	2.35	0.18
0.4	0.05	AG1	5	2.35	0.172

0.4	0.05	ptsI	5	2.6	0.175
0.4	0.05	clcB	5	2.6	0.179
0.4	0.05	ycaM	5	2.6	0.183
0.4	0.05	yadI	5	2.6	0.18
0.4	0.05	AG1	5	2.6	0.173
0.4	0.05	ptsI	5	2.85	0.181
0.4	0.05	clcB	5	2.85	0.189
0.4	0.05	ycaM	5	2.85	0.191
0.4	0.05	yadI	5	2.85	0.188
0.4	0.05	AG1	5	2.85	0.178
0.4	0.05	ptsI	5	3.1	0.186
0.4	0.05	clcB	5	3.1	0.195
0.4	0.05	ycaM	5	3.1	0.198
0.4	0.05	yadI	5	3.1	0.194
0.4	0.05	AG1	5	3.1	0.183
0.4	0.05	ptsI	5	3.35	0.195
0.4	0.05	clcB	5	3.35	0.205
0.4	0.05	ycaM	5	3.35	0.217
0.4	0.05	yadI	5	3.35	0.203
0.4	0.05	AG1	5	3.35	0.192
0.4	0.05	ptsI	5	3.6	0.206
0.4	0.05	clcB	5	3.6	0.218
0.4	0.05	ycaM	5	3.6	0.225
0.4	0.05	yadI	5	3.6	0.212
0.4	0.05	AG1	5	3.6	0.197
0.4	0.05	ptsI	5	3.85	0.22
0.4	0.05	clcB	5	3.85	0.229
0.4	0.05	ycaM	5	3.85	0.234
0.4	0.05	yadI	5	3.85	0.222
0.4	0.05	AG1	5	3.85	0.207
0.4	0.05	ptsI	5	4.38333	0.231
0.4	0.05	clcB	5	4.38333	0.311
0.4	0.05	ycaM	5	4.38333	0.236
0.4	0.05	yadI	5	4.38333	0.262
0.4	0.05	AG1	5	4.38333	0.246
0.4	0.05	ptsI	5	4.63333	0.22
0.4	0.05	clcB	5	4.63333	0.262
0.4	0.05	ycaM	5	4.63333	0.225
0.4	0.05	yadI	5	4.63333	0.26
0.4	0.05	AG1	5	4.63333	0.242
0.4	0.05	ptsI	5	4.88333	0.23
0.4	0.05	clcB	5	4.88333	0.274
0.4	0.05	ycaM	5	4.88333	0.235
0.4	0.05	yadI	5	4.88333	0.276
0.4	0.05	AG1	5	4.88333	0.257
0.4	0.05	ptsI	5	5.13333	0.238
0.4	0.05	clcB	5	5.13333	0.286
0.4	0.05	ycaM	5	5.13333	0.239
0.4	0.05	yadI	5	5.13333	0.29
0.4	0.05	AG1	5	5.13333	0.268
0.4	0.05	ptsI	5	5.38333	0.249
0.4	0.05	clcB	5	5.38333	0.295
0.4	0.05	ycaM	5	5.38333	0.242

0.4	0.05	yadI	5	5.38333	0.307
0.4	0.05	AG1	5	5.38333	0.282
0.4	0.05	ptsI	5	5.63333	0.26
0.4	0.05	clcB	5	5.63333	0.304
0.4	0.05	ycaM	5	5.63333	0.243
0.4	0.05	yadI	5	5.63333	0.319
0.4	0.05	AG1	5	5.63333	0.296
0.4	0.05	ptsI	5	5.88333	0.27
0.4	0.05	clcB	5	5.88333	0.312
0.4	0.05	ycaM	5	5.88333	0.245
0.4	0.05	yadI	5	5.88333	0.331
0.4	0.05	AG1	5	5.88333	0.307
0.4	0.05	ptsI	5	6.13333	0.284
0.4	0.05	clcB	5	6.13333	0.322
0.4	0.05	ycaM	5	6.13333	0.247
0.4	0.05	yadI	5	6.13333	0.343
0.4	0.05	AG1	5	6.13333	0.32
0.4	0.05	ptsI	5	6.38333	0.297
0.4	0.05	clcB	5	6.38333	0.328
0.4	0.05	ycaM	5	6.38333	0.248
0.4	0.05	yadI	5	6.38333	0.356
0.4	0.05	AG1	5	6.38333	0.333
0.4	0.05	ptsI	5	6.63333	0.31
0.4	0.05	clcB	5	6.63333	0.332
0.4	0.05	ycaM	5	6.63333	0.25
0.4	0.05	yadI	5	6.63333	0.368
0.4	0.05	AG1	5	6.63333	0.346
0.4	0.05	ptsI	5	6.88333	0.324
0.4	0.05	clcB	5	6.88333	0.34
0.4	0.05	ycaM	5	6.88333	0.251
0.4	0.05	yadI	5	6.88333	0.383
0.4	0.05	AG1	5	6.88333	0.357
0.4	0.05	ptsI	5	7.13333	0.34
0.4	0.05	clcB	5	7.13333	0.346
0.4	0.05	ycaM	5	7.13333	0.251
0.4	0.05	yadI	5	7.13333	0.397
0.4	0.05	AG1	5	7.13333	0.369
0.4	0.05	ptsI	5	7.38333	0.356
0.4	0.05	clcB	5	7.38333	0.351
0.4	0.05	ycaM	5	7.38333	0.255
0.4	0.05	yadI	5	7.38333	0.411
0.4	0.05	AG1	5	7.38333	0.382
0.4	0.05	ptsI	5	7.63333	0.372
0.4	0.05	clcB	5	7.63333	0.354
0.4	0.05	ycaM	5	7.63333	0.256
0.4	0.05	yadI	5	7.63333	0.422
0.4	0.05	AG1	5	7.63333	0.392
0.4	0.05	ptsI	5	7.88333	0.387
0.4	0.05	clcB	5	7.88333	0.358
0.4	0.05	ycaM	5	7.88333	0.261
0.4	0.05	yadI	5	7.88333	0.434
0.4	0.05	AG1	5	7.88333	0.4
0.4	0.05	ptsI	5	8.13333	0.403

0.4	0.05	clcB	5	8.13333	0.362
0.4	0.05	ycaM	5	8.13333	0.265
0.4	0.05	yadI	5	8.13333	0.443
0.4	0.05	AG1	5	8.13333	0.413
0.4	0.05	ptsI	5	8.38333	0.421
0.4	0.05	clcB	5	8.38333	0.366
0.4	0.05	ycaM	5	8.38333	0.267
0.4	0.05	yadI	5	8.38333	0.452
0.4	0.05	AG1	5	8.38333	0.425
0.4	0.05	ptsI	5	8.88333	0.339
0.4	0.05	clcB	5	8.88333	0.29
0.4	0.05	ycaM	5	8.88333	0.262
0.4	0.05	yadI	5	8.88333	0.437
0.4	0.05	AG1	5	8.88333	0.467
0.4	0.05	ptsI	5	9.13333	0.343
0.4	0.05	clcB	5	9.13333	0.288
0.4	0.05	ycaM	5	9.13333	0.26
0.4	0.05	yadI	5	9.13333	0.436
0.4	0.05	AG1	5	9.13333	0.472
0.4	0.05	ptsI	5	9.38333	0.356
0.4	0.05	clcB	5	9.38333	0.288
0.4	0.05	ycaM	5	9.38333	0.257
0.4	0.05	yadI	5	9.38333	0.444
0.4	0.05	AG1	5	9.38333	0.488
0.4	0.05	ptsI	5	9.63333	0.366
0.4	0.05	clcB	5	9.63333	0.29
0.4	0.05	ycaM	5	9.63333	0.257
0.4	0.05	yadI	5	9.63333	0.455
0.4	0.05	AG1	5	9.63333	0.506
0.4	0.05	ptsI	5	9.88333	0.375
0.4	0.05	clcB	5	9.88333	0.29
0.4	0.05	ycaM	5	9.88333	0.257
0.4	0.05	yadI	5	9.88333	0.463
0.4	0.05	AG1	5	9.88333	0.516
0.4	0.05	ptsI	5	10.1333	0.386
0.4	0.05	clcB	5	10.1333	0.295
0.4	0.05	ycaM	5	10.1333	0.259
0.4	0.05	yadI	5	10.1333	0.472
0.4	0.05	AG1	5	10.1333	0.522
0.4	0.05	ptsI	5	10.3833	0.396
0.4	0.05	clcB	5	10.3833	0.294
0.4	0.05	ycaM	5	10.3833	0.259
0.4	0.05	yadI	5	10.3833	0.48
0.4	0.05	AG1	5	10.3833	0.536
0.4	0.05	ptsI	5	10.6333	0.406
0.4	0.05	clcB	5	10.6333	0.294
0.4	0.05	ycaM	5	10.6333	0.259
0.4	0.05	yadI	5	10.6333	0.485
0.4	0.05	AG1	5	10.6333	0.543
0.4	0.05	ptsI	5	10.8833	0.416
0.4	0.05	clcB	5	10.8833	0.296
0.4	0.05	ycaM	5	10.8833	0.258
0.4	0.05	yadI	5	10.8833	0.492

0.4	0.05	AG1	5	10.8833	0.557
0.4	0.05	ptsI	5	11.1333	0.427
0.4	0.05	clcB	5	11.1333	0.295
0.4	0.05	ycaM	5	11.1333	0.258
0.4	0.05	yadI	5	11.1333	0.498
0.4	0.05	AG1	5	11.1333	0.566
0.4	0.05	ptsI	5	11.3833	0.434
0.4	0.05	clcB	5	11.3833	0.296
0.4	0.05	ycaM	5	11.3833	0.259
0.4	0.05	yadI	5	11.3833	0.507
0.4	0.05	AG1	5	11.3833	0.578
0.4	0.05	ptsI	5	11.6333	0.441
0.4	0.05	clcB	5	11.6333	0.296
0.4	0.05	ycaM	5	11.6333	0.259
0.4	0.05	yadI	5	11.6333	0.514
0.4	0.05	AG1	5	11.6333	0.586
0.4	0.05	ptsI	5	11.8833	0.449
0.4	0.05	clcB	5	11.8833	0.295
0.4	0.05	ycaM	5	11.8833	0.26
0.4	0.05	yadI	5	11.8833	0.521
0.4	0.05	AG1	5	11.8833	0.596
0.4	0.05	ptsI	5	12.1333	0.46
0.4	0.05	clcB	5	12.1333	0.294
0.4	0.05	ycaM	5	12.1333	0.26
0.4	0.05	yadI	5	12.1333	0.527
0.4	0.05	AG1	5	12.1333	0.605
0.4	0.05	ptsI	5	12.3833	0.463
0.4	0.05	clcB	5	12.3833	0.296
0.4	0.05	ycaM	5	12.3833	0.256
0.4	0.05	yadI	5	12.3833	0.523
0.4	0.05	AG1	5	12.3833	0.602
0.4	0.05	ptsI	5	12.6333	0.474
0.4	0.05	clcB	5	12.6333	0.294
0.4	0.05	ycaM	5	12.6333	0.257
0.4	0.05	yadI	5	12.6333	0.54
0.4	0.05	AG1	5	12.6333	0.623
0.4	0.05	ptsI	5	12.8833	0.483
0.4	0.05	clcB	5	12.8833	0.296
0.4	0.05	ycaM	5	12.8833	0.262
0.4	0.05	yadI	5	12.8833	0.546
0.4	0.05	AG1	5	12.8833	0.632
0.4	0.05	ptsI	5	13.1333	0.491
0.4	0.05	clcB	5	13.1333	0.293
0.4	0.05	ycaM	5	13.1333	0.263
0.4	0.05	yadI	5	13.1333	0.549
0.4	0.05	AG1	5	13.1333	0.64
0.4	0.05	ptsI	5	13.3833	0.499
0.4	0.05	clcB	5	13.3833	0.292
0.4	0.05	ycaM	5	13.3833	0.26
0.4	0.05	yadI	5	13.3833	0.552
0.4	0.05	AG1	5	13.3833	0.645
0.4	0.05	ptsI	5	13.6333	0.506
0.4	0.05	clcB	5	13.6333	0.292

0.4	0.05	ycaM	5	13.6333	0.262
0.4	0.05	yadI	5	13.6333	0.557
0.4	0.05	AG1	5	13.6333	0.652
0.4	0.05	ptsI	5	13.8833	0.511
0.4	0.05	clcB	5	13.8833	0.294
0.4	0.05	ycaM	5	13.8833	0.262
0.4	0.05	yadI	5	13.8833	0.563
0.4	0.05	AG1	5	13.8833	0.656
0.4	0.05	ptsI	5	14.1333	0.52
0.4	0.05	clcB	5	14.1333	0.296
0.4	0.05	ycaM	5	14.1333	0.261
0.4	0.05	yadI	5	14.1333	0.568
0.4	0.05	AG1	5	14.1333	0.663
0.4	0.05	ptsI	5	14.3833	0.523
0.4	0.05	clcB	5	14.3833	0.296
0.4	0.05	ycaM	5	14.3833	0.264
0.4	0.05	yadI	5	14.3833	0.57
0.4	0.05	AG1	5	14.3833	0.669
0.4	0.05	ptsI	5	14.6333	0.53
0.4	0.05	clcB	5	14.6333	0.297
0.4	0.05	ycaM	5	14.6333	0.267
0.4	0.05	yadI	5	14.6333	0.575
0.4	0.05	AG1	5	14.6333	0.674
0.4	0.05	ptsI	5	14.8833	0.544
0.4	0.05	clcB	5	14.8833	0.297
0.4	0.05	ycaM	5	14.8833	0.265
0.4	0.05	yadI	5	14.8833	0.579
0.4	0.05	AG1	5	14.8833	0.681
0.4	0.05	ptsI	5	15.1333	0.547
0.4	0.05	clcB	5	15.1333	0.297
0.4	0.05	ycaM	5	15.1333	0.268
0.4	0.05	yadI	5	15.1333	0.581
0.4	0.05	AG1	5	15.1333	0.684
0.4	0.05	ptsI	5	15.3833	0.551
0.4	0.05	clcB	5	15.3833	0.299
0.4	0.05	ycaM	5	15.3833	0.267
0.4	0.05	yadI	5	15.3833	0.586
0.4	0.05	AG1	5	15.3833	0.69
0.4	0.05	ptsI	5	15.6333	0.562
0.4	0.05	clcB	5	15.6333	0.298
0.4	0.05	ycaM	5	15.6333	0.268
0.4	0.05	yadI	5	15.6333	0.592
0.4	0.05	AG1	5	15.6333	0.697
0.4	0.05	ptsI	5	15.8833	0.567
0.4	0.05	clcB	5	15.8833	0.298
0.4	0.05	ycaM	5	15.8833	0.268
0.4	0.05	yadI	5	15.8833	0.596
0.4	0.05	AG1	5	15.8833	0.702
0.4	0.05	ptsI	5	16.1333	0.576
0.4	0.05	clcB	5	16.1333	0.303
0.4	0.05	ycaM	5	16.1333	0.272
0.4	0.05	yadI	5	16.1333	0.593
0.4	0.05	AG1	5	16.1333	0.709

0.4	0.05	ptsI	5	16.3833	0.577
0.4	0.05	clcB	5	16.3833	0.299
0.4	0.05	ycaM	5	16.3833	0.269
0.4	0.05	yadI	5	16.3833	0.595
0.4	0.05	AG1	5	16.3833	0.708
0.4	0.05	ptsI	5	16.6333	0.582
0.4	0.05	clcB	5	16.6333	0.298
0.4	0.05	ycaM	5	16.6333	0.273
0.4	0.05	yadI	5	16.6333	0.605
0.4	0.05	AG1	5	16.6333	0.713
0.4	0.05	ptsI	5	16.8833	0.589
0.4	0.05	clcB	5	16.8833	0.303
0.4	0.05	ycaM	5	16.8833	0.274
0.4	0.05	yadI	5	16.8833	0.602
0.4	0.05	AG1	5	16.8833	0.72
0.4	0.05	ptsI	5	17.1333	0.597
0.4	0.05	clcB	5	17.1333	0.301
0.4	0.05	ycaM	5	17.1333	0.276
0.4	0.05	yadI	5	17.1333	0.604
0.4	0.05	AG1	5	17.1333	0.728
0.4	0.05	ptsI	5	17.3833	0.599
0.4	0.05	clcB	5	17.3833	0.303
0.4	0.05	ycaM	5	17.3833	0.275
0.4	0.05	yadI	5	17.3833	0.608
0.4	0.05	AG1	5	17.3833	0.729
0.4	0.05	ptsI	5	17.6333	0.603
0.4	0.05	clcB	5	17.6333	0.304
0.4	0.05	ycaM	5	17.6333	0.276
0.4	0.05	yadI	5	17.6333	0.61
0.4	0.05	AG1	5	17.6333	0.738
0.4	0.05	ptsI	5	17.8833	0.607
0.4	0.05	clcB	5	17.8833	0.304
0.4	0.05	ycaM	5	17.8833	0.278
0.4	0.05	yadI	5	17.8833	0.605
0.4	0.05	AG1	5	17.8833	0.723
0.4	0.05	ptsI	5	18.1333	0.613
0.4	0.05	clcB	5	18.1333	0.309
0.4	0.05	ycaM	5	18.1333	0.282
0.4	0.05	yadI	5	18.1333	0.613
0.4	0.05	AG1	5	18.1333	0.739
0.4	0.05	ptsI	5	18.3833	0.618
0.4	0.05	clcB	5	18.3833	0.312
0.4	0.05	ycaM	5	18.3833	0.282
0.4	0.05	yadI	5	18.3833	0.615
0.4	0.05	AG1	5	18.3833	0.748
0.4	0.05	ptsI	5	18.6333	0.621
0.4	0.05	clcB	5	18.6333	0.314
0.4	0.05	ycaM	5	18.6333	0.283
0.4	0.05	yadI	5	18.6333	0.618
0.4	0.05	AG1	5	18.6333	0.746
0.4	0.05	ptsI	5	18.8833	0.623
0.4	0.05	clcB	5	18.8833	0.317
0.4	0.05	ycaM	5	18.8833	0.287

0.4	0.05	yadI	5	18.8833	0.621
0.4	0.05	AG1	5	18.8833	0.748
0.4	0.05	ptsI	5	19.1333	0.626
0.4	0.05	clcB	5	19.1333	0.316
0.4	0.05	ycaM	5	19.1333	0.285
0.4	0.05	yadI	5	19.1333	0.622
0.4	0.05	AG1	5	19.1333	0.754
0.4	0.05	ptsI	5	19.3833	0.634
0.4	0.05	clcB	5	19.3833	0.314
0.4	0.05	ycaM	5	19.3833	0.291
0.4	0.05	yadI	5	19.3833	0.625
0.4	0.05	AG1	5	19.3833	0.755
0.4	0.05	ptsI	5	19.6333	0.636
0.4	0.05	clcB	5	19.6333	0.318
0.4	0.05	ycaM	5	19.6333	0.294
0.4	0.05	yadI	5	19.6333	0.627
0.4	0.05	AG1	5	19.6333	0.758
0.4	0.05	ptsI	5	19.8833	0.634
0.4	0.05	clcB	5	19.8833	0.319
0.4	0.05	ycaM	5	19.8833	0.292
0.4	0.05	yadI	5	19.8833	0.627
0.4	0.05	AG1	5	19.8833	0.764
0.4	0.05	ptsI	5	20.1333	0.635
0.4	0.05	clcB	5	20.1333	0.323
0.4	0.05	ycaM	5	20.1333	0.293
0.4	0.05	yadI	5	20.1333	0.627
0.4	0.05	AG1	5	20.1333	0.768
0.4	0.05	ptsI	5	20.3833	0.636
0.4	0.05	clcB	5	20.3833	0.33
0.4	0.05	ycaM	5	20.3833	0.295
0.4	0.05	yadI	5	20.3833	0.629
0.4	0.05	AG1	5	20.3833	0.769
0.4	0.05	ptsI	5	20.6333	0.633
0.4	0.05	clcB	5	20.6333	0.33
0.4	0.05	ycaM	5	20.6333	0.292
0.4	0.05	yadI	5	20.6333	0.621
0.4	0.05	AG1	5	20.6333	0.757
0.4	0.05	ptsI	5	20.8833	0.637
0.4	0.05	clcB	5	20.8833	0.334
0.4	0.05	ycaM	5	20.8833	0.3
0.4	0.05	yadI	5	20.8833	0.637
0.4	0.05	AG1	5	20.8833	0.775
0.4	0.05	ptsI	5	21.1333	0.639
0.4	0.05	clcB	5	21.1333	0.335
0.4	0.05	ycaM	5	21.1333	0.3
0.4	0.05	yadI	5	21.1333	0.638
0.4	0.05	AG1	5	21.1333	0.774
0.4	0.05	ptsI	5	21.3833	0.639
0.4	0.05	clcB	5	21.3833	0.338
0.4	0.05	ycaM	5	21.3833	0.306
0.4	0.05	yadI	5	21.3833	0.64
0.4	0.05	AG1	5	21.3833	0.774
0.4	0.05	ptsI	5	21.6333	0.64

0.4	0.05	clcB	5	21.6333	0.344
0.4	0.05	ycaM	5	21.6333	0.31
0.4	0.05	yadI	5	21.6333	0.64
0.4	0.05	AG1	5	21.6333	0.773
0.4	0.05	ptsI	5	21.8833	0.642
0.4	0.05	clcB	5	21.8833	0.343
0.4	0.05	ycaM	5	21.8833	0.312
0.4	0.05	yadI	5	21.8833	0.639
0.4	0.05	AG1	5	21.8833	0.775
0.4	0.05	ptsI	5	22.1333	0.636
0.4	0.05	clcB	5	22.1333	0.34
0.4	0.05	ycaM	5	22.1333	0.313
0.4	0.05	yadI	5	22.1333	0.628
0.4	0.05	AG1	5	22.1333	0.768
0.4	0.05	ptsI	5	22.3833	0.642
0.4	0.05	clcB	5	22.3833	0.337
0.4	0.05	ycaM	5	22.3833	0.319
0.4	0.05	yadI	5	22.3833	0.646
0.4	0.05	AG1	5	22.3833	0.79
0.4	0.05	ptsI	5	22.6333	0.644
0.4	0.05	clcB	5	22.6333	0.334
0.4	0.05	ycaM	5	22.6333	0.324
0.4	0.05	yadI	5	22.6333	0.648
0.4	0.05	AG1	5	22.6333	0.788
0.4	0.05	ptsI	5	22.8833	0.641
0.4	0.05	clcB	5	22.8833	0.329
0.4	0.05	ycaM	5	22.8833	0.326
0.4	0.05	yadI	5	22.8833	0.647
0.4	0.05	AG1	5	22.8833	0.784
0.4	0.05	ptsI	5	23.1333	0.639
0.4	0.05	clcB	5	23.1333	0.334
0.4	0.05	ycaM	5	23.1333	0.328
0.4	0.05	yadI	5	23.1333	0.649
0.4	0.05	AG1	5	23.1333	0.788
0.4	0.05	ptsI	5	23.3833	0.641
0.4	0.05	clcB	5	23.3833	0.325
0.4	0.05	ycaM	5	23.3833	0.331
0.4	0.05	yadI	5	23.3833	0.649
0.4	0.05	AG1	5	23.3833	0.791
0.4	0.05	ptsI	5	23.6333	0.641
0.4	0.05	clcB	5	23.6333	0.323
0.4	0.05	ycaM	5	23.6333	0.333
0.4	0.05	yadI	5	23.6333	0.646
0.4	0.05	AG1	5	23.6333	0.794
0.4	0.05	ptsI	5	23.8833	0.638
0.4	0.05	clcB	5	23.8833	0.316
0.4	0.05	ycaM	5	23.8833	0.335
0.4	0.05	yadI	5	23.8833	0.641
0.4	0.05	AG1	5	23.8833	0.782
0.4	0.05	ptsI	5	24.1333	0.647
0.4	0.05	clcB	5	24.1333	0.318
0.4	0.05	ycaM	5	24.1333	0.341
0.4	0.05	yadI	5	24.1333	0.648

0.4	0.05	AG1	5	24.1333	0.796
0.4	0.05	ptsI	5	24.3833	0.647
0.4	0.05	clcB	5	24.3833	0.314
0.4	0.05	ycaM	5	24.3833	0.341
0.4	0.05	yadI	5	24.3833	0.653
0.4	0.05	AG1	5	24.3833	0.799
0.8	0.05	ptsI	2	0	0.221
0.8	0.05	clcB	2	0	0.224
0.8	0.05	ycaM	2	0	0.217
0.8	0.05	yadI	2	0	0.223
0.8	0.05	AG1	2	0	0.232
0.8	0.05	ptsI	2	0.25	0.215
0.8	0.05	clcB	2	0.25	0.218
0.8	0.05	ycaM	2	0.25	0.213
0.8	0.05	yadI	2	0.25	0.217
0.8	0.05	AG1	2	0.25	0.224
0.8	0.05	ptsI	2	0.5	0.215
0.8	0.05	clcB	2	0.5	0.219
0.8	0.05	ycaM	2	0.5	0.212
0.8	0.05	yadI	2	0.5	0.215
0.8	0.05	AG1	2	0.5	0.223
0.8	0.05	ptsI	2	0.75	0.216
0.8	0.05	clcB	2	0.75	0.22
0.8	0.05	ycaM	2	0.75	0.212
0.8	0.05	yadI	2	0.75	0.218
0.8	0.05	AG1	2	0.75	0.224
0.8	0.05	ptsI	2	1	0.219
0.8	0.05	clcB	2	1	0.22
0.8	0.05	ycaM	2	1	0.214
0.8	0.05	yadI	2	1	0.222
0.8	0.05	AG1	2	1	0.229
0.8	0.05	ptsI	2	1.25	0.22
0.8	0.05	clcB	2	1.25	0.222
0.8	0.05	ycaM	2	1.25	0.212
0.8	0.05	yadI	2	1.25	0.229
0.8	0.05	AG1	2	1.25	0.23
0.8	0.05	ptsI	2	1.5	0.222
0.8	0.05	clcB	2	1.5	0.221
0.8	0.05	ycaM	2	1.5	0.214
0.8	0.05	yadI	2	1.5	0.234
0.8	0.05	AG1	2	1.5	0.234
0.8	0.05	ptsI	2	1.75	0.227
0.8	0.05	clcB	2	1.75	0.224
0.8	0.05	ycaM	2	1.75	0.217
0.8	0.05	yadI	2	1.75	0.239
0.8	0.05	AG1	2	1.75	0.237
0.8	0.05	ptsI	2	2	0.233
0.8	0.05	clcB	2	2	0.228
0.8	0.05	ycaM	2	2	0.223
0.8	0.05	yadI	2	2	0.244
0.8	0.05	AG1	2	2	0.243
0.8	0.05	ptsI	2	2.25	0.239
0.8	0.05	clcB	2	2.25	0.234

0.8	0.05	ycaM	2	2.25	0.228
0.8	0.05	yadI	2	2.25	0.256
0.8	0.05	AG1	2	2.25	0.254
0.8	0.05	ptsI	2	2.5	0.247
0.8	0.05	clcB	2	2.5	0.24
0.8	0.05	ycaM	2	2.5	0.234
0.8	0.05	yadI	2	2.5	0.265
0.8	0.05	AG1	2	2.5	0.259
0.8	0.05	ptsI	2	2.75	0.246
0.8	0.05	clcB	2	2.75	0.246
0.8	0.05	ycaM	2	2.75	0.233
0.8	0.05	yadI	2	2.75	0.274
0.8	0.05	AG1	2	2.75	0.256
0.8	0.05	ptsI	2	3	0.251
0.8	0.05	clcB	2	3	0.246
0.8	0.05	ycaM	2	3	0.235
0.8	0.05	yadI	2	3	0.281
0.8	0.05	AG1	2	3	0.259
0.8	0.05	ptsI	2	3.25	0.257
0.8	0.05	clcB	2	3.25	0.248
0.8	0.05	ycaM	2	3.25	0.244
0.8	0.05	yadI	2	3.25	0.292
0.8	0.05	AG1	2	3.25	0.271
0.8	0.05	ptsI	2	3.5	0.263
0.8	0.05	clcB	2	3.5	0.257
0.8	0.05	ycaM	2	3.5	0.249
0.8	0.05	yadI	2	3.5	0.3
0.8	0.05	AG1	2	3.5	0.28
0.8	0.05	ptsI	2	3.75	0.273
0.8	0.05	clcB	2	3.75	0.265
0.8	0.05	ycaM	2	3.75	0.259
0.8	0.05	yadI	2	3.75	0.312
0.8	0.05	AG1	2	3.75	0.289
0.8	0.05	ptsI	2	4	0.282
0.8	0.05	clcB	2	4	0.275
0.8	0.05	ycaM	2	4	0.267
0.8	0.05	yadI	2	4	0.323
0.8	0.05	AG1	2	4	0.299
0.8	0.05	ptsI	2	4.38333	0.291
0.8	0.05	clcB	2	4.38333	0.32
0.8	0.05	ycaM	2	4.38333	0.284
0.8	0.05	yadI	2	4.38333	0.333
0.8	0.05	AG1	2	4.38333	0.314
0.8	0.05	ptsI	2	4.63333	0.287
0.8	0.05	clcB	2	4.63333	0.316
0.8	0.05	ycaM	2	4.63333	0.284
0.8	0.05	yadI	2	4.63333	0.34
0.8	0.05	AG1	2	4.63333	0.307
0.8	0.05	ptsI	2	4.88333	0.292
0.8	0.05	clcB	2	4.88333	0.324
0.8	0.05	ycaM	2	4.88333	0.288
0.8	0.05	yadI	2	4.88333	0.356
0.8	0.05	AG1	2	4.88333	0.317

0.8	0.05	ptsI	2	5.13333	0.299
0.8	0.05	clcB	2	5.13333	0.334
0.8	0.05	ycaM	2	5.13333	0.291
0.8	0.05	yadI	2	5.13333	0.371
0.8	0.05	AG1	2	5.13333	0.325
0.8	0.05	ptsI	2	5.38333	0.305
0.8	0.05	clcB	2	5.38333	0.34
0.8	0.05	ycaM	2	5.38333	0.293
0.8	0.05	yadI	2	5.38333	0.38
0.8	0.05	AG1	2	5.38333	0.335
0.8	0.05	ptsI	2	5.63333	0.312
0.8	0.05	clcB	2	5.63333	0.346
0.8	0.05	ycaM	2	5.63333	0.294
0.8	0.05	yadI	2	5.63333	0.39
0.8	0.05	AG1	2	5.63333	0.343
0.8	0.05	ptsI	2	5.88333	0.319
0.8	0.05	clcB	2	5.88333	0.353
0.8	0.05	ycaM	2	5.88333	0.296
0.8	0.05	yadI	2	5.88333	0.398
0.8	0.05	AG1	2	5.88333	0.352
0.8	0.05	ptsI	2	6.13333	0.326
0.8	0.05	clcB	2	6.13333	0.359
0.8	0.05	ycaM	2	6.13333	0.299
0.8	0.05	yadI	2	6.13333	0.404
0.8	0.05	AG1	2	6.13333	0.359
0.8	0.05	ptsI	2	6.38333	0.334
0.8	0.05	clcB	2	6.38333	0.366
0.8	0.05	ycaM	2	6.38333	0.299
0.8	0.05	yadI	2	6.38333	0.412
0.8	0.05	AG1	2	6.38333	0.368
0.8	0.05	ptsI	2	6.63333	0.343
0.8	0.05	clcB	2	6.63333	0.37
0.8	0.05	ycaM	2	6.63333	0.3
0.8	0.05	yadI	2	6.63333	0.42
0.8	0.05	AG1	2	6.63333	0.377
0.8	0.05	ptsI	2	6.88333	0.35
0.8	0.05	clcB	2	6.88333	0.375
0.8	0.05	ycaM	2	6.88333	0.302
0.8	0.05	yadI	2	6.88333	0.427
0.8	0.05	AG1	2	6.88333	0.386
0.8	0.05	ptsI	2	7.13333	0.36
0.8	0.05	clcB	2	7.13333	0.381
0.8	0.05	ycaM	2	7.13333	0.302
0.8	0.05	yadI	2	7.13333	0.435
0.8	0.05	AG1	2	7.13333	0.394
0.8	0.05	ptsI	2	7.38333	0.368
0.8	0.05	clcB	2	7.38333	0.386
0.8	0.05	ycaM	2	7.38333	0.302
0.8	0.05	yadI	2	7.38333	0.441
0.8	0.05	AG1	2	7.38333	0.399
0.8	0.05	ptsI	2	7.63333	0.375
0.8	0.05	clcB	2	7.63333	0.392
0.8	0.05	ycaM	2	7.63333	0.303

0.8	0.05	yadI	2	7.63333	0.445
0.8	0.05	AG1	2	7.63333	0.405
0.8	0.05	ptsI	2	7.88333	0.382
0.8	0.05	clcB	2	7.88333	0.395
0.8	0.05	ycaM	2	7.88333	0.305
0.8	0.05	yadI	2	7.88333	0.453
0.8	0.05	AG1	2	7.88333	0.411
0.8	0.05	ptsI	2	8.13333	0.389
0.8	0.05	clcB	2	8.13333	0.396
0.8	0.05	ycaM	2	8.13333	0.306
0.8	0.05	yadI	2	8.13333	0.463
0.8	0.05	AG1	2	8.13333	0.419
0.8	0.05	ptsI	2	8.38333	0.397
0.8	0.05	clcB	2	8.38333	0.405
0.8	0.05	ycaM	2	8.38333	0.304
0.8	0.05	yadI	2	8.38333	0.471
0.8	0.05	AG1	2	8.38333	0.427
0.8	0.05	ptsI	2	8.91667	0.36
0.8	0.05	clcB	2	8.91667	0.309
0.8	0.05	ycaM	2	8.91667	0.274
0.8	0.05	yadI	2	8.91667	0.505
0.8	0.05	AG1	2	8.91667	0.438
0.8	0.05	ptsI	2	9.16667	0.361
0.8	0.05	clcB	2	9.16667	0.304
0.8	0.05	ycaM	2	9.16667	0.272
0.8	0.05	yadI	2	9.16667	0.511
0.8	0.05	AG1	2	9.16667	0.443
0.8	0.05	ptsI	2	9.41667	0.367
0.8	0.05	clcB	2	9.41667	0.309
0.8	0.05	ycaM	2	9.41667	0.273
0.8	0.05	yadI	2	9.41667	0.523
0.8	0.05	AG1	2	9.41667	0.46
0.8	0.05	ptsI	2	9.66667	0.374
0.8	0.05	clcB	2	9.66667	0.314
0.8	0.05	ycaM	2	9.66667	0.276
0.8	0.05	yadI	2	9.66667	0.535
0.8	0.05	AG1	2	9.66667	0.474
0.8	0.05	ptsI	2	9.91667	0.38
0.8	0.05	clcB	2	9.91667	0.311
0.8	0.05	ycaM	2	9.91667	0.277
0.8	0.05	yadI	2	9.91667	0.543
0.8	0.05	AG1	2	9.91667	0.476
0.8	0.05	ptsI	2	10.1667	0.385
0.8	0.05	clcB	2	10.1667	0.319
0.8	0.05	ycaM	2	10.1667	0.278
0.8	0.05	yadI	2	10.1667	0.552
0.8	0.05	AG1	2	10.1667	0.48
0.8	0.05	ptsI	2	10.4167	0.392
0.8	0.05	clcB	2	10.4167	0.319
0.8	0.05	ycaM	2	10.4167	0.28
0.8	0.05	yadI	2	10.4167	0.562
0.8	0.05	AG1	2	10.4167	0.488
0.8	0.05	ptsI	2	10.6667	0.401

0.8	0.05	clcB	2	10.6667	0.328
0.8	0.05	ycaM	2	10.6667	0.28
0.8	0.05	yadI	2	10.6667	0.57
0.8	0.05	AG1	2	10.6667	0.5
0.8	0.05	ptsI	2	10.9167	0.406
0.8	0.05	clcB	2	10.9167	0.328
0.8	0.05	ycaM	2	10.9167	0.282
0.8	0.05	yadI	2	10.9167	0.579
0.8	0.05	AG1	2	10.9167	0.512
0.8	0.05	ptsI	2	11.1667	0.416
0.8	0.05	clcB	2	11.1667	0.32
0.8	0.05	ycaM	2	11.1667	0.284
0.8	0.05	yadI	2	11.1667	0.587
0.8	0.05	AG1	2	11.1667	0.52
0.8	0.05	ptsI	2	11.4167	0.422
0.8	0.05	clcB	2	11.4167	0.324
0.8	0.05	ycaM	2	11.4167	0.283
0.8	0.05	yadI	2	11.4167	0.593
0.8	0.05	AG1	2	11.4167	0.52
0.8	0.05	ptsI	2	11.6667	0.432
0.8	0.05	clcB	2	11.6667	0.315
0.8	0.05	ycaM	2	11.6667	0.285
0.8	0.05	yadI	2	11.6667	0.598
0.8	0.05	AG1	2	11.6667	0.537
0.8	0.05	ptsI	2	11.9167	0.437
0.8	0.05	clcB	2	11.9167	0.317
0.8	0.05	ycaM	2	11.9167	0.285
0.8	0.05	yadI	2	11.9167	0.6
0.8	0.05	AG1	2	11.9167	0.533
0.8	0.05	ptsI	2	12.1667	0.447
0.8	0.05	clcB	2	12.1667	0.314
0.8	0.05	ycaM	2	12.1667	0.285
0.8	0.05	yadI	2	12.1667	0.61
0.8	0.05	AG1	2	12.1667	0.552
0.8	0.05	ptsI	2	12.4167	0.454
0.8	0.05	clcB	2	12.4167	0.316
0.8	0.05	ycaM	2	12.4167	0.284
0.8	0.05	yadI	2	12.4167	0.618
0.8	0.05	AG1	2	12.4167	0.563
0.8	0.05	ptsI	2	12.6667	0.46
0.8	0.05	clcB	2	12.6667	0.319
0.8	0.05	ycaM	2	12.6667	0.281
0.8	0.05	yadI	2	12.6667	0.624
0.8	0.05	AG1	2	12.6667	0.564
0.8	0.05	ptsI	2	12.9167	0.469
0.8	0.05	clcB	2	12.9167	0.313
0.8	0.05	ycaM	2	12.9167	0.287
0.8	0.05	yadI	2	12.9167	0.635
0.8	0.05	AG1	2	12.9167	0.574
0.8	0.05	ptsI	2	13.1667	0.478
0.8	0.05	clcB	2	13.1667	0.316
0.8	0.05	ycaM	2	13.1667	0.282
0.8	0.05	yadI	2	13.1667	0.636

0.8	0.05	AG1	2	13.1667	0.575
0.8	0.05	ptsI	2	13.4167	0.482
0.8	0.05	clcB	2	13.4167	0.317
0.8	0.05	ycaM	2	13.4167	0.281
0.8	0.05	yadI	2	13.4167	0.645
0.8	0.05	AG1	2	13.4167	0.572
0.8	0.05	ptsI	2	13.6667	0.493
0.8	0.05	clcB	2	13.6667	0.311
0.8	0.05	ycaM	2	13.6667	0.281
0.8	0.05	yadI	2	13.6667	0.645
0.8	0.05	AG1	2	13.6667	0.602
0.8	0.05	ptsI	2	13.9167	0.496
0.8	0.05	clcB	2	13.9167	0.312
0.8	0.05	ycaM	2	13.9167	0.279
0.8	0.05	yadI	2	13.9167	0.644
0.8	0.05	AG1	2	13.9167	0.596
0.8	0.05	ptsI	2	14.1667	0.502
0.8	0.05	clcB	2	14.1667	0.313
0.8	0.05	ycaM	2	14.1667	0.282
0.8	0.05	yadI	2	14.1667	0.648
0.8	0.05	AG1	2	14.1667	0.587
0.8	0.05	ptsI	2	14.4167	0.51
0.8	0.05	clcB	2	14.4167	0.32
0.8	0.05	ycaM	2	14.4167	0.283
0.8	0.05	yadI	2	14.4167	0.652
0.8	0.05	AG1	2	14.4167	0.592
0.8	0.05	ptsI	2	14.6667	0.517
0.8	0.05	clcB	2	14.6667	0.323
0.8	0.05	ycaM	2	14.6667	0.281
0.8	0.05	yadI	2	14.6667	0.659
0.8	0.05	AG1	2	14.6667	0.604
0.8	0.05	ptsI	2	14.9167	0.518
0.8	0.05	clcB	2	14.9167	0.325
0.8	0.05	ycaM	2	14.9167	0.282
0.8	0.05	yadI	2	14.9167	0.663
0.8	0.05	AG1	2	14.9167	0.61
0.8	0.05	ptsI	2	15.1667	0.53
0.8	0.05	clcB	2	15.1667	0.33
0.8	0.05	ycaM	2	15.1667	0.281
0.8	0.05	yadI	2	15.1667	0.666
0.8	0.05	AG1	2	15.1667	0.608
0.8	0.05	ptsI	2	15.4167	0.534
0.8	0.05	clcB	2	15.4167	0.323
0.8	0.05	ycaM	2	15.4167	0.278
0.8	0.05	yadI	2	15.4167	0.667
0.8	0.05	AG1	2	15.4167	0.634
0.8	0.05	ptsI	2	15.6667	0.525
0.8	0.05	clcB	2	15.6667	0.33
0.8	0.05	ycaM	2	15.6667	0.276
0.8	0.05	yadI	2	15.6667	0.671
0.8	0.05	AG1	2	15.6667	0.626
0.8	0.05	ptsI	2	15.9167	0.53
0.8	0.05	clcB	2	15.9167	0.326

0.8	0.05	ycaM	2	15.9167	0.278
0.8	0.05	yadI	2	15.9167	0.676
0.8	0.05	AG1	2	15.9167	0.638
0.8	0.05	ptsI	2	16.1667	0.536
0.8	0.05	clcB	2	16.1667	0.334
0.8	0.05	ycaM	2	16.1667	0.277
0.8	0.05	yadI	2	16.1667	0.68
0.8	0.05	AG1	2	16.1667	0.653
0.8	0.05	ptsI	2	16.4167	0.54
0.8	0.05	clcB	2	16.4167	0.33
0.8	0.05	ycaM	2	16.4167	0.277
0.8	0.05	yadI	2	16.4167	0.682
0.8	0.05	AG1	2	16.4167	0.655
0.8	0.05	ptsI	2	16.6667	0.541
0.8	0.05	clcB	2	16.6667	0.327
0.8	0.05	ycaM	2	16.6667	0.278
0.8	0.05	yadI	2	16.6667	0.683
0.8	0.05	AG1	2	16.6667	0.646
0.8	0.05	ptsI	2	16.9167	0.551
0.8	0.05	clcB	2	16.9167	0.324
0.8	0.05	ycaM	2	16.9167	0.28
0.8	0.05	yadI	2	16.9167	0.689
0.8	0.05	AG1	2	16.9167	0.658
0.8	0.05	ptsI	2	17.1667	0.558
0.8	0.05	clcB	2	17.1667	0.326
0.8	0.05	ycaM	2	17.1667	0.279
0.8	0.05	yadI	2	17.1667	0.69
0.8	0.05	AG1	2	17.1667	0.66
0.8	0.05	ptsI	2	17.4167	0.563
0.8	0.05	clcB	2	17.4167	0.331
0.8	0.05	ycaM	2	17.4167	0.278
0.8	0.05	yadI	2	17.4167	0.696
0.8	0.05	AG1	2	17.4167	0.662
0.8	0.05	ptsI	2	17.6667	0.561
0.8	0.05	clcB	2	17.6667	0.328
0.8	0.05	ycaM	2	17.6667	0.28
0.8	0.05	yadI	2	17.6667	0.698
0.8	0.05	AG1	2	17.6667	0.664
0.8	0.05	ptsI	2	17.9167	0.564
0.8	0.05	clcB	2	17.9167	0.326
0.8	0.05	ycaM	2	17.9167	0.28
0.8	0.05	yadI	2	17.9167	0.7
0.8	0.05	AG1	2	17.9167	0.687
0.8	0.05	ptsI	2	18.1667	0.564
0.8	0.05	clcB	2	18.1667	0.325
0.8	0.05	ycaM	2	18.1667	0.282
0.8	0.05	yadI	2	18.1667	0.708
0.8	0.05	AG1	2	18.1667	0.701
0.8	0.05	ptsI	2	18.4167	0.567
0.8	0.05	clcB	2	18.4167	0.326
0.8	0.05	ycaM	2	18.4167	0.282
0.8	0.05	yadI	2	18.4167	0.707
0.8	0.05	AG1	2	18.4167	0.72

0.8	0.05	ptsI	2	18.6667	0.57
0.8	0.05	clcB	2	18.6667	0.328
0.8	0.05	ycaM	2	18.6667	0.285
0.8	0.05	yadI	2	18.6667	0.71
0.8	0.05	AG1	2	18.6667	0.716
0.8	0.05	ptsI	2	18.9167	0.579
0.8	0.05	clcB	2	18.9167	0.326
0.8	0.05	ycaM	2	18.9167	0.284
0.8	0.05	yadI	2	18.9167	0.708
0.8	0.05	AG1	2	18.9167	0.715
0.8	0.05	ptsI	2	19.1667	0.571
0.8	0.05	clcB	2	19.1667	0.328
0.8	0.05	ycaM	2	19.1667	0.29
0.8	0.05	yadI	2	19.1667	0.717
0.8	0.05	AG1	2	19.1667	0.735
0.8	0.05	ptsI	2	19.4167	0.577
0.8	0.05	clcB	2	19.4167	0.329
0.8	0.05	ycaM	2	19.4167	0.29
0.8	0.05	yadI	2	19.4167	0.721
0.8	0.05	AG1	2	19.4167	0.734
0.8	0.05	ptsI	2	19.6667	0.582
0.8	0.05	clcB	2	19.6667	0.331
0.8	0.05	ycaM	2	19.6667	0.292
0.8	0.05	yadI	2	19.6667	0.723
0.8	0.05	AG1	2	19.6667	0.728
0.8	0.05	ptsI	2	19.9167	0.588
0.8	0.05	clcB	2	19.9167	0.329
0.8	0.05	ycaM	2	19.9167	0.293
0.8	0.05	yadI	2	19.9167	0.725
0.8	0.05	AG1	2	19.9167	0.727
0.8	0.05	ptsI	2	20.1667	0.587
0.8	0.05	clcB	2	20.1667	0.328
0.8	0.05	ycaM	2	20.1667	0.293
0.8	0.05	yadI	2	20.1667	0.727
0.8	0.05	AG1	2	20.1667	0.726
0.8	0.05	ptsI	2	20.4167	0.589
0.8	0.05	clcB	2	20.4167	0.333
0.8	0.05	ycaM	2	20.4167	0.295
0.8	0.05	yadI	2	20.4167	0.73
0.8	0.05	AG1	2	20.4167	0.732
0.8	0.05	ptsI	2	20.6667	0.592
0.8	0.05	clcB	2	20.6667	0.326
0.8	0.05	ycaM	2	20.6667	0.297
0.8	0.05	yadI	2	20.6667	0.729
0.8	0.05	AG1	2	20.6667	0.732
0.8	0.05	ptsI	2	20.9167	0.593
0.8	0.05	clcB	2	20.9167	0.326
0.8	0.05	ycaM	2	20.9167	0.3
0.8	0.05	yadI	2	20.9167	0.735
0.8	0.05	AG1	2	20.9167	0.736
0.8	0.05	ptsI	2	21.1667	0.593
0.8	0.05	clcB	2	21.1667	0.326
0.8	0.05	ycaM	2	21.1667	0.3

0.8	0.05	yadI	2	21.1667	0.735
0.8	0.05	AG1	2	21.1667	0.736
0.8	0.05	ptsI	2	21.4167	0.598
0.8	0.05	clcB	2	21.4167	0.329
0.8	0.05	ycaM	2	21.4167	0.299
0.8	0.05	yadI	2	21.4167	0.735
0.8	0.05	AG1	2	21.4167	0.748
0.8	0.05	ptsI	2	21.6667	0.599
0.8	0.05	clcB	2	21.6667	0.328
0.8	0.05	ycaM	2	21.6667	0.299
0.8	0.05	yadI	2	21.6667	0.737
0.8	0.05	AG1	2	21.6667	0.758
0.8	0.05	ptsI	2	21.9167	0.599
0.8	0.05	clcB	2	21.9167	0.329
0.8	0.05	ycaM	2	21.9167	0.301
0.8	0.05	yadI	2	21.9167	0.74
0.8	0.05	AG1	2	21.9167	0.758
0.8	0.05	ptsI	2	22.1667	0.602
0.8	0.05	clcB	2	22.1667	0.328
0.8	0.05	ycaM	2	22.1667	0.304
0.8	0.05	yadI	2	22.1667	0.745
0.8	0.05	AG1	2	22.1667	0.761
0.8	0.05	ptsI	2	22.4167	0.596
0.8	0.05	clcB	2	22.4167	0.331
0.8	0.05	ycaM	2	22.4167	0.305
0.8	0.05	yadI	2	22.4167	0.747
0.8	0.05	AG1	2	22.4167	0.77
0.8	0.05	ptsI	2	22.6667	0.598
0.8	0.05	clcB	2	22.6667	0.332
0.8	0.05	ycaM	2	22.6667	0.307
0.8	0.05	yadI	2	22.6667	0.746
0.8	0.05	AG1	2	22.6667	0.768
0.8	0.05	ptsI	2	22.9167	0.604
0.8	0.05	clcB	2	22.9167	0.331
0.8	0.05	ycaM	2	22.9167	0.307
0.8	0.05	yadI	2	22.9167	0.746
0.8	0.05	AG1	2	22.9167	0.792
0.8	0.05	ptsI	2	23.1667	0.603
0.8	0.05	clcB	2	23.1667	0.331
0.8	0.05	ycaM	2	23.1667	0.309
0.8	0.05	yadI	2	23.1667	0.752
0.8	0.05	AG1	2	23.1667	0.779
0.8	0.05	ptsI	2	23.4167	0.599
0.8	0.05	clcB	2	23.4167	0.331
0.8	0.05	ycaM	2	23.4167	0.312
0.8	0.05	yadI	2	23.4167	0.757
0.8	0.05	AG1	2	23.4167	0.783
0.8	0.05	ptsI	2	23.6667	0.601
0.8	0.05	clcB	2	23.6667	0.333
0.8	0.05	ycaM	2	23.6667	0.314
0.8	0.05	yadI	2	23.6667	0.76
0.8	0.05	AG1	2	23.6667	0.787
0.8	0.05	ptsI	2	23.9167	0.605

0.8	0.05	clcB	2	23.9167	0.331
0.8	0.05	ycaM	2	23.9167	0.313
0.8	0.05	yadI	2	23.9167	0.76
0.8	0.05	AG1	2	23.9167	0.793
0.8	0.05	ptsI	2	24.1667	0.603
0.8	0.05	clcB	2	24.1667	0.328
0.8	0.05	ycaM	2	24.1667	0.314
0.8	0.05	yadI	2	24.1667	0.759
0.8	0.05	AG1	2	24.1667	0.799
0.8	0.05	ptsI	2	24.4167	0.6
0.8	0.05	clcB	2	24.4167	0.327
0.8	0.05	ycaM	2	24.4167	0.314
0.8	0.05	yadI	2	24.4167	0.763
0.8	0.05	AG1	2	24.4167	0.805
0.8	0.05	ptsI	2	24.6667	0.599
0.8	0.05	clcB	2	24.6667	0.327
0.8	0.05	ycaM	2	24.6667	0.315
0.8	0.05	yadI	2	24.6667	0.763
0.8	0.05	AG1	2	24.6667	0.811
0.8	0.05	ptsI	2	24.9167	0.598
0.8	0.05	clcB	2	24.9167	0.324
0.8	0.05	ycaM	2	24.9167	0.317
0.8	0.05	yadI	2	24.9167	0.77
0.8	0.05	AG1	2	24.9167	0.82
0.8	0.05	ptsI	2	25.1667	0.6
0.8	0.05	clcB	2	25.1667	0.325
0.8	0.05	ycaM	2	25.1667	0.318
0.8	0.05	yadI	2	25.1667	0.769
0.8	0.05	AG1	2	25.1667	0.829
0.8	0.05	ptsI	2	25.4167	0.595
0.8	0.05	clcB	2	25.4167	0.326
0.8	0.05	ycaM	2	25.4167	0.32
0.8	0.05	yadI	2	25.4167	0.772
0.8	0.05	AG1	2	25.4167	0.832
0.8	0.05	ptsI	2	25.6667	0.594
0.8	0.05	clcB	2	25.6667	0.326
0.8	0.05	ycaM	2	25.6667	0.323
0.8	0.05	yadI	2	25.6667	0.773
0.8	0.05	AG1	2	25.6667	0.839
0.8	0.05	ptsI	2	25.9167	0.593
0.8	0.05	clcB	2	25.9167	0.327
0.8	0.05	ycaM	2	25.9167	0.326
0.8	0.05	yadI	2	25.9167	0.776
0.8	0.05	AG1	2	25.9167	0.856
0.8	0.05	ptsI	2	26.1667	0.591
0.8	0.05	clcB	2	26.1667	0.328
0.8	0.05	ycaM	2	26.1667	0.328
0.8	0.05	yadI	2	26.1667	0.778
0.8	0.05	AG1	2	26.1667	0.865
0.8	0.05	ptsI	2	26.4167	0.59
0.8	0.05	clcB	2	26.4167	0.326
0.8	0.05	ycaM	2	26.4167	0.329
0.8	0.05	yadI	2	26.4167	0.779

0.8	0.05	AG1	2	26.4167	0.88
0.8	0.05	ptsI	3	0	0.158
0.8	0.05	clcB	3	0	0.155
0.8	0.05	ycaM	3	0	0.154
0.8	0.05	yadI	3	0	0.167
0.8	0.05	AG1	3	0	0.163
0.8	0.05	ptsI	3	0.25	0.157
0.8	0.05	clcB	3	0.25	0.153
0.8	0.05	ycaM	3	0.25	0.153
0.8	0.05	yadI	3	0.25	0.158
0.8	0.05	AG1	3	0.25	0.162
0.8	0.05	ptsI	3	0.5	0.156
0.8	0.05	clcB	3	0.5	0.153
0.8	0.05	ycaM	3	0.5	0.152
0.8	0.05	yadI	3	0.5	0.156
0.8	0.05	AG1	3	0.5	0.159
0.8	0.05	ptsI	3	0.75	0.156
0.8	0.05	clcB	3	0.75	0.152
0.8	0.05	ycaM	3	0.75	0.152
0.8	0.05	yadI	3	0.75	0.156
0.8	0.05	AG1	3	0.75	0.16
0.8	0.05	ptsI	3	1	0.157
0.8	0.05	clcB	3	1	0.166
0.8	0.05	ycaM	3	1	0.153
0.8	0.05	yadI	3	1	0.156
0.8	0.05	AG1	3	1	0.16
0.8	0.05	ptsI	3	1.25	0.157
0.8	0.05	clcB	3	1.25	0.154
0.8	0.05	ycaM	3	1.25	0.153
0.8	0.05	yadI	3	1.25	0.156
0.8	0.05	AG1	3	1.25	0.16
0.8	0.05	ptsI	3	1.5	0.158
0.8	0.05	clcB	3	1.5	0.154
0.8	0.05	ycaM	3	1.5	0.154
0.8	0.05	yadI	3	1.5	0.156
0.8	0.05	AG1	3	1.5	0.16
0.8	0.05	ptsI	3	1.75	0.158
0.8	0.05	clcB	3	1.75	0.154
0.8	0.05	ycaM	3	1.75	0.154
0.8	0.05	yadI	3	1.75	0.157
0.8	0.05	AG1	3	1.75	0.161
0.8	0.05	ptsI	3	2	0.159
0.8	0.05	clcB	3	2	0.158
0.8	0.05	ycaM	3	2	0.155
0.8	0.05	yadI	3	2	0.158
0.8	0.05	AG1	3	2	0.161
0.8	0.05	ptsI	3	2.25	0.16
0.8	0.05	clcB	3	2.25	0.157
0.8	0.05	ycaM	3	2.25	0.157
0.8	0.05	yadI	3	2.25	0.161
0.8	0.05	AG1	3	2.25	0.162
0.8	0.05	ptsI	3	2.5	0.162
0.8	0.05	clcB	3	2.5	0.16

0.8	0.05	ycaM	3	2.5	0.157
0.8	0.05	yadI	3	2.5	0.162
0.8	0.05	AG1	3	2.5	0.164
0.8	0.05	ptsI	3	2.75	0.165
0.8	0.05	clcB	3	2.75	0.161
0.8	0.05	ycaM	3	2.75	0.16
0.8	0.05	yadI	3	2.75	0.165
0.8	0.05	AG1	3	2.75	0.166
0.8	0.05	ptsI	3	3	0.166
0.8	0.05	clcB	3	3	0.162
0.8	0.05	ycaM	3	3	0.16
0.8	0.05	yadI	3	3	0.166
0.8	0.05	AG1	3	3	0.167
0.8	0.05	ptsI	3	3.25	0.168
0.8	0.05	clcB	3	3.25	0.164
0.8	0.05	ycaM	3	3.25	0.162
0.8	0.05	yadI	3	3.25	0.169
0.8	0.05	AG1	3	3.25	0.168
0.8	0.05	ptsI	3	3.5	0.172
0.8	0.05	clcB	3	3.5	0.165
0.8	0.05	ycaM	3	3.5	0.165
0.8	0.05	yadI	3	3.5	0.173
0.8	0.05	AG1	3	3.5	0.17
0.8	0.05	ptsI	3	3.75	0.174
0.8	0.05	clcB	3	3.75	0.169
0.8	0.05	ycaM	3	3.75	0.167
0.8	0.05	yadI	3	3.75	0.177
0.8	0.05	AG1	3	3.75	0.171
0.8	0.05	ptsI	3	4	0.178
0.8	0.05	clcB	3	4	0.171
0.8	0.05	ycaM	3	4	0.17
0.8	0.05	yadI	3	4	0.182
0.8	0.05	AG1	3	4	0.174
0.8	0.05	ptsI	3	4.41667	0.192
0.8	0.05	clcB	3	4.41667	0.219
0.8	0.05	ycaM	3	4.41667	0.172
0.8	0.05	yadI	3	4.41667	0.22
0.8	0.05	AG1	3	4.41667	0.188
0.8	0.05	ptsI	3	4.66667	0.171
0.8	0.05	clcB	3	4.66667	0.182
0.8	0.05	ycaM	3	4.66667	0.17
0.8	0.05	yadI	3	4.66667	0.221
0.8	0.05	AG1	3	4.66667	0.188
0.8	0.05	ptsI	3	4.91667	0.172
0.8	0.05	clcB	3	4.91667	0.183
0.8	0.05	ycaM	3	4.91667	0.171
0.8	0.05	yadI	3	4.91667	0.228
0.8	0.05	AG1	3	4.91667	0.192
0.8	0.05	ptsI	3	5.16667	0.175
0.8	0.05	clcB	3	5.16667	0.186
0.8	0.05	ycaM	3	5.16667	0.175
0.8	0.05	yadI	3	5.16667	0.24
0.8	0.05	AG1	3	5.16667	0.197

0.8	0.05	ptsI	3	5.41667	0.178
0.8	0.05	clcB	3	5.41667	0.189
0.8	0.05	ycaM	3	5.41667	0.181
0.8	0.05	yadI	3	5.41667	0.254
0.8	0.05	AG1	3	5.41667	0.204
0.8	0.05	ptsI	3	5.66667	0.182
0.8	0.05	clcB	3	5.66667	0.195
0.8	0.05	ycaM	3	5.66667	0.187
0.8	0.05	yadI	3	5.66667	0.272
0.8	0.05	AG1	3	5.66667	0.212
0.8	0.05	ptsI	3	5.91667	0.186
0.8	0.05	clcB	3	5.91667	0.197
0.8	0.05	ycaM	3	5.91667	0.194
0.8	0.05	yadI	3	5.91667	0.274
0.8	0.05	AG1	3	5.91667	0.214
0.8	0.05	ptsI	3	6.16667	0.192
0.8	0.05	clcB	3	6.16667	0.204
0.8	0.05	ycaM	3	6.16667	0.201
0.8	0.05	yadI	3	6.16667	0.292
0.8	0.05	AG1	3	6.16667	0.222
0.8	0.05	ptsI	3	6.41667	0.198
0.8	0.05	clcB	3	6.41667	0.212
0.8	0.05	ycaM	3	6.41667	0.209
0.8	0.05	yadI	3	6.41667	0.301
0.8	0.05	AG1	3	6.41667	0.231
0.8	0.05	ptsI	3	6.66667	0.206
0.8	0.05	clcB	3	6.66667	0.22
0.8	0.05	ycaM	3	6.66667	0.219
0.8	0.05	yadI	3	6.66667	0.309
0.8	0.05	AG1	3	6.66667	0.242
0.8	0.05	ptsI	3	6.91667	0.214
0.8	0.05	clcB	3	6.91667	0.229
0.8	0.05	ycaM	3	6.91667	0.227
0.8	0.05	yadI	3	6.91667	0.317
0.8	0.05	AG1	3	6.91667	0.25
0.8	0.05	ptsI	3	7.16667	0.224
0.8	0.05	clcB	3	7.16667	0.238
0.8	0.05	ycaM	3	7.16667	0.233
0.8	0.05	yadI	3	7.16667	0.328
0.8	0.05	AG1	3	7.16667	0.259
0.8	0.05	ptsI	3	7.41667	0.233
0.8	0.05	clcB	3	7.41667	0.25
0.8	0.05	ycaM	3	7.41667	0.236
0.8	0.05	yadI	3	7.41667	0.339
0.8	0.05	AG1	3	7.41667	0.267
0.8	0.05	ptsI	3	7.66667	0.242
0.8	0.05	clcB	3	7.66667	0.26
0.8	0.05	ycaM	3	7.66667	0.238
0.8	0.05	yadI	3	7.66667	0.352
0.8	0.05	AG1	3	7.66667	0.272
0.8	0.05	ptsI	3	7.91667	0.25
0.8	0.05	clcB	3	7.91667	0.262
0.8	0.05	ycaM	3	7.91667	0.24

0.8	0.05	yadI	3	7.91667	0.362
0.8	0.05	AG1	3	7.91667	0.281
0.8	0.05	ptsI	3	8.16667	0.258
0.8	0.05	clcB	3	8.16667	0.266
0.8	0.05	ycaM	3	8.16667	0.245
0.8	0.05	yadI	3	8.16667	0.371
0.8	0.05	AG1	3	8.16667	0.289
0.8	0.05	ptsI	3	8.41667	0.267
0.8	0.05	clcB	3	8.41667	0.27
0.8	0.05	ycaM	3	8.41667	0.245
0.8	0.05	yadI	3	8.41667	0.38
0.8	0.05	AG1	3	8.41667	0.296
0.8	0.05	ptsI	3	9.05	0.285
0.8	0.05	clcB	3	9.05	0.28
0.8	0.05	ycaM	3	9.05	0.233
0.8	0.05	yadI	3	9.05	0.368
0.8	0.05	AG1	3	9.05	0.353
0.8	0.05	ptsI	3	9.3	0.288
0.8	0.05	clcB	3	9.3	0.282
0.8	0.05	ycaM	3	9.3	0.229
0.8	0.05	yadI	3	9.3	0.374
0.8	0.05	AG1	3	9.3	0.362
0.8	0.05	ptsI	3	9.55	0.298
0.8	0.05	clcB	3	9.55	0.292
0.8	0.05	ycaM	3	9.55	0.232
0.8	0.05	yadI	3	9.55	0.387
0.8	0.05	AG1	3	9.55	0.377
0.8	0.05	ptsI	3	9.8	0.306
0.8	0.05	clcB	3	9.8	0.296
0.8	0.05	ycaM	3	9.8	0.232
0.8	0.05	yadI	3	9.8	0.401
0.8	0.05	AG1	3	9.8	0.387
0.8	0.05	ptsI	3	10.05	0.313
0.8	0.05	clcB	3	10.05	0.302
0.8	0.05	ycaM	3	10.05	0.234
0.8	0.05	yadI	3	10.05	0.413
0.8	0.05	AG1	3	10.05	0.396
0.8	0.05	ptsI	3	10.3	0.321
0.8	0.05	clcB	3	10.3	0.307
0.8	0.05	ycaM	3	10.3	0.236
0.8	0.05	yadI	3	10.3	0.422
0.8	0.05	AG1	3	10.3	0.404
0.8	0.05	ptsI	3	10.55	0.33
0.8	0.05	clcB	3	10.55	0.312
0.8	0.05	ycaM	3	10.55	0.235
0.8	0.05	yadI	3	10.55	0.431
0.8	0.05	AG1	3	10.55	0.415
0.8	0.05	ptsI	3	10.8	0.34
0.8	0.05	clcB	3	10.8	0.309
0.8	0.05	ycaM	3	10.8	0.238
0.8	0.05	yadI	3	10.8	0.438
0.8	0.05	AG1	3	10.8	0.423
0.8	0.05	ptsI	3	11.05	0.352

0.8	0.05	clcB	3	11.05	0.316
0.8	0.05	ycaM	3	11.05	0.24
0.8	0.05	yadI	3	11.05	0.45
0.8	0.05	AG1	3	11.05	0.435
0.8	0.05	ptsI	3	11.3	0.359
0.8	0.05	clcB	3	11.3	0.318
0.8	0.05	ycaM	3	11.3	0.24
0.8	0.05	yadI	3	11.3	0.456
0.8	0.05	AG1	3	11.3	0.444
0.8	0.05	ptsI	3	11.55	0.373
0.8	0.05	clcB	3	11.55	0.318
0.8	0.05	ycaM	3	11.55	0.242
0.8	0.05	yadI	3	11.55	0.47
0.8	0.05	AG1	3	11.55	0.45
0.8	0.05	ptsI	3	11.8	0.38
0.8	0.05	clcB	3	11.8	0.314
0.8	0.05	ycaM	3	11.8	0.241
0.8	0.05	yadI	3	11.8	0.479
0.8	0.05	AG1	3	11.8	0.46
0.8	0.05	ptsI	3	12.05	0.386
0.8	0.05	clcB	3	12.05	0.311
0.8	0.05	ycaM	3	12.05	0.236
0.8	0.05	yadI	3	12.05	0.479
0.8	0.05	AG1	3	12.05	0.467
0.8	0.05	ptsI	3	12.3	0.398
0.8	0.05	clcB	3	12.3	0.305
0.8	0.05	ycaM	3	12.3	0.24
0.8	0.05	yadI	3	12.3	0.494
0.8	0.05	AG1	3	12.3	0.476
0.8	0.05	ptsI	3	12.55	0.408
0.8	0.05	clcB	3	12.55	0.302
0.8	0.05	ycaM	3	12.55	0.238
0.8	0.05	yadI	3	12.55	0.501
0.8	0.05	AG1	3	12.55	0.489
0.8	0.05	ptsI	3	12.8	0.417
0.8	0.05	clcB	3	12.8	0.301
0.8	0.05	ycaM	3	12.8	0.239
0.8	0.05	yadI	3	12.8	0.504
0.8	0.05	AG1	3	12.8	0.499
0.8	0.05	ptsI	3	13.05	0.417
0.8	0.05	clcB	3	13.05	0.296
0.8	0.05	ycaM	3	13.05	0.237
0.8	0.05	yadI	3	13.05	0.505
0.8	0.05	AG1	3	13.05	0.504
0.8	0.05	ptsI	3	13.3	0.429
0.8	0.05	clcB	3	13.3	0.292
0.8	0.05	ycaM	3	13.3	0.236
0.8	0.05	yadI	3	13.3	0.513
0.8	0.05	AG1	3	13.3	0.524
0.8	0.05	ptsI	3	13.55	0.439
0.8	0.05	clcB	3	13.55	0.293
0.8	0.05	ycaM	3	13.55	0.237
0.8	0.05	yadI	3	13.55	0.52

0.8	0.05	AG1	3	13.55	0.534
0.8	0.05	ptsI	3	13.8	0.446
0.8	0.05	clcB	3	13.8	0.284
0.8	0.05	ycaM	3	13.8	0.236
0.8	0.05	yadI	3	13.8	0.526
0.8	0.05	AG1	3	13.8	0.542
0.8	0.05	ptsI	3	14.05	0.454
0.8	0.05	clcB	3	14.05	0.284
0.8	0.05	ycaM	3	14.05	0.236
0.8	0.05	yadI	3	14.05	0.532
0.8	0.05	AG1	3	14.05	0.549
0.8	0.05	ptsI	3	14.3	0.454
0.8	0.05	clcB	3	14.3	0.284
0.8	0.05	ycaM	3	14.3	0.238
0.8	0.05	yadI	3	14.3	0.535
0.8	0.05	AG1	3	14.3	0.552
0.8	0.05	ptsI	3	14.55	0.456
0.8	0.05	clcB	3	14.55	0.286
0.8	0.05	ycaM	3	14.55	0.235
0.8	0.05	yadI	3	14.55	0.538
0.8	0.05	AG1	3	14.55	0.558
0.8	0.05	ptsI	3	14.8	0.465
0.8	0.05	clcB	3	14.8	0.289
0.8	0.05	ycaM	3	14.8	0.235
0.8	0.05	yadI	3	14.8	0.541
0.8	0.05	AG1	3	14.8	0.573
0.8	0.05	ptsI	3	15.05	0.473
0.8	0.05	clcB	3	15.05	0.288
0.8	0.05	ycaM	3	15.05	0.233
0.8	0.05	yadI	3	15.05	0.546
0.8	0.05	AG1	3	15.05	0.586
0.8	0.05	ptsI	3	15.3	0.48
0.8	0.05	clcB	3	15.3	0.293
0.8	0.05	ycaM	3	15.3	0.235
0.8	0.05	yadI	3	15.3	0.55
0.8	0.05	AG1	3	15.3	0.582
0.8	0.05	ptsI	3	15.55	0.489
0.8	0.05	clcB	3	15.55	0.284
0.8	0.05	ycaM	3	15.55	0.234
0.8	0.05	yadI	3	15.55	0.555
0.8	0.05	AG1	3	15.55	0.596
0.8	0.05	ptsI	3	15.8	0.498
0.8	0.05	clcB	3	15.8	0.284
0.8	0.05	ycaM	3	15.8	0.234
0.8	0.05	yadI	3	15.8	0.561
0.8	0.05	AG1	3	15.8	0.61
0.8	0.05	ptsI	3	16.05	0.498
0.8	0.05	clcB	3	16.05	0.286
0.8	0.05	ycaM	3	16.05	0.234
0.8	0.05	yadI	3	16.05	0.562
0.8	0.05	AG1	3	16.05	0.618
0.8	0.05	ptsI	3	16.3	0.501
0.8	0.05	clcB	3	16.3	0.284

0.8	0.05	ycaM	3	16.3	0.233
0.8	0.05	yadI	3	16.3	0.564
0.8	0.05	AG1	3	16.3	0.615
0.8	0.05	ptsI	3	16.55	0.508
0.8	0.05	clcB	3	16.55	0.281
0.8	0.05	ycaM	3	16.55	0.233
0.8	0.05	yadI	3	16.55	0.569
0.8	0.05	AG1	3	16.55	0.631
0.8	0.05	ptsI	3	16.8	0.515
0.8	0.05	clcB	3	16.8	0.28
0.8	0.05	ycaM	3	16.8	0.232
0.8	0.05	yadI	3	16.8	0.577
0.8	0.05	AG1	3	16.8	0.642
0.8	0.05	ptsI	3	17.05	0.511
0.8	0.05	clcB	3	17.05	0.281
0.8	0.05	ycaM	3	17.05	0.232
0.8	0.05	yadI	3	17.05	0.583
0.8	0.05	AG1	3	17.05	0.658
0.8	0.05	ptsI	3	17.3	0.513
0.8	0.05	clcB	3	17.3	0.283
0.8	0.05	ycaM	3	17.3	0.234
0.8	0.05	yadI	3	17.3	0.585
0.8	0.05	AG1	3	17.3	0.663
0.8	0.05	ptsI	3	17.55	0.519
0.8	0.05	clcB	3	17.55	0.281
0.8	0.05	ycaM	3	17.55	0.234
0.8	0.05	yadI	3	17.55	0.591
0.8	0.05	AG1	3	17.55	0.66
0.8	0.05	ptsI	3	17.8	0.518
0.8	0.05	clcB	3	17.8	0.282
0.8	0.05	ycaM	3	17.8	0.234
0.8	0.05	yadI	3	17.8	0.595
0.8	0.05	AG1	3	17.8	0.667
0.8	0.05	ptsI	3	18.05	0.526
0.8	0.05	clcB	3	18.05	0.283
0.8	0.05	ycaM	3	18.05	0.236
0.8	0.05	yadI	3	18.05	0.598
0.8	0.05	AG1	3	18.05	0.686
0.8	0.05	ptsI	3	18.3	0.524
0.8	0.05	clcB	3	18.3	0.282
0.8	0.05	ycaM	3	18.3	0.235
0.8	0.05	yadI	3	18.3	0.601
0.8	0.05	AG1	3	18.3	0.682
0.8	0.05	ptsI	3	18.55	0.528
0.8	0.05	clcB	3	18.55	0.283
0.8	0.05	ycaM	3	18.55	0.237
0.8	0.05	yadI	3	18.55	0.603
0.8	0.05	AG1	3	18.55	0.673
0.8	0.05	ptsI	3	18.8	0.533
0.8	0.05	clcB	3	18.8	0.281
0.8	0.05	ycaM	3	18.8	0.237
0.8	0.05	yadI	3	18.8	0.606
0.8	0.05	AG1	3	18.8	0.675

0.8	0.05	ptsI	3	19.05	0.539
0.8	0.05	clcB	3	19.05	0.282
0.8	0.05	ycaM	3	19.05	0.237
0.8	0.05	yadI	3	19.05	0.611
0.8	0.05	AG1	3	19.05	0.661
0.8	0.05	ptsI	3	19.3	0.54
0.8	0.05	clcB	3	19.3	0.283
0.8	0.05	ycaM	3	19.3	0.239
0.8	0.05	yadI	3	19.3	0.614
0.8	0.05	AG1	3	19.3	0.665
0.8	0.05	ptsI	3	19.55	0.546
0.8	0.05	clcB	3	19.55	0.281
0.8	0.05	ycaM	3	19.55	0.24
0.8	0.05	yadI	3	19.55	0.615
0.8	0.05	AG1	3	19.55	0.665
0.8	0.05	ptsI	3	19.8	0.546
0.8	0.05	clcB	3	19.8	0.282
0.8	0.05	ycaM	3	19.8	0.24
0.8	0.05	yadI	3	19.8	0.619
0.8	0.05	AG1	3	19.8	0.681
0.8	0.05	ptsI	3	20.05	0.546
0.8	0.05	clcB	3	20.05	0.28
0.8	0.05	ycaM	3	20.05	0.243
0.8	0.05	yadI	3	20.05	0.622
0.8	0.05	AG1	3	20.05	0.693
0.8	0.05	ptsI	3	20.3	0.55
0.8	0.05	clcB	3	20.3	0.283
0.8	0.05	ycaM	3	20.3	0.246
0.8	0.05	yadI	3	20.3	0.624
0.8	0.05	AG1	3	20.3	0.701
0.8	0.05	ptsI	3	20.55	0.548
0.8	0.05	clcB	3	20.55	0.283
0.8	0.05	ycaM	3	20.55	0.246
0.8	0.05	yadI	3	20.55	0.628
0.8	0.05	AG1	3	20.55	0.709
0.8	0.05	ptsI	3	20.8	0.545
0.8	0.05	clcB	3	20.8	0.285
0.8	0.05	ycaM	3	20.8	0.248
0.8	0.05	yadI	3	20.8	0.63
0.8	0.05	AG1	3	20.8	0.713
0.8	0.05	ptsI	3	21.05	0.546
0.8	0.05	clcB	3	21.05	0.285
0.8	0.05	ycaM	3	21.05	0.248
0.8	0.05	yadI	3	21.05	0.632
0.8	0.05	AG1	3	21.05	0.714
0.8	0.05	ptsI	3	21.3	0.545
0.8	0.05	clcB	3	21.3	0.284
0.8	0.05	ycaM	3	21.3	0.249
0.8	0.05	yadI	3	21.3	0.633
0.8	0.05	AG1	3	21.3	0.717
0.8	0.05	ptsI	3	21.55	0.545
0.8	0.05	clcB	3	21.55	0.284
0.8	0.05	ycaM	3	21.55	0.251

0.8	0.05	yadI	3	21.55	0.634
0.8	0.05	AG1	3	21.55	0.725
0.8	0.05	ptsI	3	21.8	0.542
0.8	0.05	clcB	3	21.8	0.286
0.8	0.05	ycaM	3	21.8	0.253
0.8	0.05	yadI	3	21.8	0.64
0.8	0.05	AG1	3	21.8	0.735
0.8	0.05	ptsI	3	22.05	0.544
0.8	0.05	clcB	3	22.05	0.286
0.8	0.05	ycaM	3	22.05	0.254
0.8	0.05	yadI	3	22.05	0.639
0.8	0.05	AG1	3	22.05	0.744
0.8	0.05	ptsI	3	22.3	0.548
0.8	0.05	clcB	3	22.3	0.286
0.8	0.05	ycaM	3	22.3	0.257
0.8	0.05	yadI	3	22.3	0.639
0.8	0.05	AG1	3	22.3	0.751
0.8	0.05	ptsI	3	22.55	0.55
0.8	0.05	clcB	3	22.55	0.286
0.8	0.05	ycaM	3	22.55	0.259
0.8	0.05	yadI	3	22.55	0.647
0.8	0.05	AG1	3	22.55	0.755
0.8	0.05	ptsI	3	22.8	0.544
0.8	0.05	clcB	3	22.8	0.288
0.8	0.05	ycaM	3	22.8	0.259
0.8	0.05	yadI	3	22.8	0.65
0.8	0.05	AG1	3	22.8	0.766
0.8	0.05	ptsI	3	23.05	0.541
0.8	0.05	clcB	3	23.05	0.291
0.8	0.05	ycaM	3	23.05	0.263
0.8	0.05	yadI	3	23.05	0.651
0.8	0.05	AG1	3	23.05	0.774
0.8	0.05	ptsI	3	23.3	0.541
0.8	0.05	clcB	3	23.3	0.292
0.8	0.05	ycaM	3	23.3	0.266
0.8	0.05	yadI	3	23.3	0.655
0.8	0.05	AG1	3	23.3	0.78
0.8	0.05	ptsI	3	23.55	0.548
0.8	0.05	clcB	3	23.55	0.292
0.8	0.05	ycaM	3	23.55	0.268
0.8	0.05	yadI	3	23.55	0.654
0.8	0.05	AG1	3	23.55	0.791
0.8	0.05	ptsI	3	23.8	0.543
0.8	0.05	clcB	3	23.8	0.295
0.8	0.05	ycaM	3	23.8	0.272
0.8	0.05	yadI	3	23.8	0.656
0.8	0.05	AG1	3	23.8	0.797
0.8	0.05	ptsI	3	24.05	0.546
0.8	0.05	clcB	3	24.05	0.296
0.8	0.05	ycaM	3	24.05	0.273
0.8	0.05	yadI	3	24.05	0.658
0.8	0.05	AG1	3	24.05	0.802
0.8	0.05	ptsI	3	24.3	0.548

0.8	0.05	clcB	3	24.3	0.293
0.8	0.05	ycaM	3	24.3	0.276
0.8	0.05	yadI	3	24.3	0.658
0.8	0.05	AG1	3	24.3	0.813
0.8	0.05	ptsI	4	0	0.203
0.8	0.05	clcB	4	0	0.196
0.8	0.05	ycaM	4	0	0.212
0.8	0.05	yadI	4	0	0.217
0.8	0.05	AG1	4	0	0.208
0.8	0.05	ptsI	4	0.25	0.2
0.8	0.05	clcB	4	0.25	0.192
0.8	0.05	ycaM	4	0.25	0.203
0.8	0.05	yadI	4	0.25	0.211
0.8	0.05	AG1	4	0.25	0.204
0.8	0.05	ptsI	4	0.5	0.202
0.8	0.05	clcB	4	0.5	0.193
0.8	0.05	ycaM	4	0.5	0.204
0.8	0.05	yadI	4	0.5	0.213
0.8	0.05	AG1	4	0.5	0.206
0.8	0.05	ptsI	4	0.75	0.203
0.8	0.05	clcB	4	0.75	0.194
0.8	0.05	ycaM	4	0.75	0.207
0.8	0.05	yadI	4	0.75	0.214
0.8	0.05	AG1	4	0.75	0.208
0.8	0.05	ptsI	4	1	0.205
0.8	0.05	clcB	4	1	0.199
0.8	0.05	ycaM	4	1	0.211
0.8	0.05	yadI	4	1	0.211
0.8	0.05	AG1	4	1	0.212
0.8	0.05	ptsI	4	1.25	0.21
0.8	0.05	clcB	4	1.25	0.205
0.8	0.05	ycaM	4	1.25	0.218
0.8	0.05	yadI	4	1.25	0.218
0.8	0.05	AG1	4	1.25	0.216
0.8	0.05	ptsI	4	1.5	0.211
0.8	0.05	clcB	4	1.5	0.202
0.8	0.05	ycaM	4	1.5	0.213
0.8	0.05	yadI	4	1.5	0.221
0.8	0.05	AG1	4	1.5	0.214
0.8	0.05	ptsI	4	1.75	0.22
0.8	0.05	clcB	4	1.75	0.207
0.8	0.05	ycaM	4	1.75	0.222
0.8	0.05	yadI	4	1.75	0.227
0.8	0.05	AG1	4	1.75	0.223
0.8	0.05	ptsI	4	2	0.23
0.8	0.05	clcB	4	2	0.216
0.8	0.05	ycaM	4	2	0.229
0.8	0.05	yadI	4	2	0.234
0.8	0.05	AG1	4	2	0.23
0.8	0.05	ptsI	4	2.25	0.238
0.8	0.05	clcB	4	2.25	0.224
0.8	0.05	ycaM	4	2.25	0.241
0.8	0.05	yadI	4	2.25	0.243

0.8	0.05	AG1	4	2.25	0.238
0.8	0.05	ptsI	4	2.5	0.242
0.8	0.05	clcB	4	2.5	0.229
0.8	0.05	ycaM	4	2.5	0.24
0.8	0.05	yadI	4	2.5	0.25
0.8	0.05	AG1	4	2.5	0.24
0.8	0.05	ptsI	4	2.75	0.244
0.8	0.05	clcB	4	2.75	0.235
0.8	0.05	ycaM	4	2.75	0.248
0.8	0.05	yadI	4	2.75	0.256
0.8	0.05	AG1	4	2.75	0.249
0.8	0.05	ptsI	4	3	0.261
0.8	0.05	clcB	4	3	0.246
0.8	0.05	ycaM	4	3	0.257
0.8	0.05	yadI	4	3	0.264
0.8	0.05	AG1	4	3	0.257
0.8	0.05	ptsI	4	3.25	0.27
0.8	0.05	clcB	4	3.25	0.255
0.8	0.05	ycaM	4	3.25	0.267
0.8	0.05	yadI	4	3.25	0.274
0.8	0.05	AG1	4	3.25	0.267
0.8	0.05	ptsI	4	3.5	0.286
0.8	0.05	clcB	4	3.5	0.264
0.8	0.05	ycaM	4	3.5	0.277
0.8	0.05	yadI	4	3.5	0.284
0.8	0.05	AG1	4	3.5	0.279
0.8	0.05	ptsI	4	3.75	0.292
0.8	0.05	clcB	4	3.75	0.275
0.8	0.05	ycaM	4	3.75	0.284
0.8	0.05	yadI	4	3.75	0.296
0.8	0.05	AG1	4	3.75	0.287
0.8	0.05	ptsI	4	4	0.321
0.8	0.05	clcB	4	4	0.291
0.8	0.05	ycaM	4	4	0.3
0.8	0.05	yadI	4	4	0.309
0.8	0.05	AG1	4	4	0.298
0.8	0.05	ptsI	4	4.35	0.313
0.8	0.05	clcB	4	4.35	0.359
0.8	0.05	ycaM	4	4.35	0.383
0.8	0.05	yadI	4	4.35	0.36
0.8	0.05	AG1	4	4.35	0.295
0.8	0.05	ptsI	4	4.6	0.312
0.8	0.05	clcB	4	4.6	0.341
0.8	0.05	ycaM	4	4.6	0.351
0.8	0.05	yadI	4	4.6	0.365
0.8	0.05	AG1	4	4.6	0.303
0.8	0.05	ptsI	4	4.85	0.322
0.8	0.05	clcB	4	4.85	0.346
0.8	0.05	ycaM	4	4.85	0.343
0.8	0.05	yadI	4	4.85	0.385
0.8	0.05	AG1	4	4.85	0.316
0.8	0.05	ptsI	4	5.1	0.332
0.8	0.05	clcB	4	5.1	0.349

0.8	0.05	ycaM	4	5.1	0.344
0.8	0.05	yadI	4	5.1	0.4
0.8	0.05	AG1	4	5.1	0.327
0.8	0.05	ptsI	4	5.35	0.347
0.8	0.05	clcB	4	5.35	0.351
0.8	0.05	ycaM	4	5.35	0.347
0.8	0.05	yadI	4	5.35	0.418
0.8	0.05	AG1	4	5.35	0.338
0.8	0.05	ptsI	4	5.6	0.359
0.8	0.05	clcB	4	5.6	0.346
0.8	0.05	ycaM	4	5.6	0.345
0.8	0.05	yadI	4	5.6	0.427
0.8	0.05	AG1	4	5.6	0.344
0.8	0.05	ptsI	4	5.85	0.368
0.8	0.05	clcB	4	5.85	0.344
0.8	0.05	ycaM	4	5.85	0.341
0.8	0.05	yadI	4	5.85	0.441
0.8	0.05	AG1	4	5.85	0.349
0.8	0.05	ptsI	4	6.1	0.383
0.8	0.05	clcB	4	6.1	0.35
0.8	0.05	ycaM	4	6.1	0.342
0.8	0.05	yadI	4	6.1	0.457
0.8	0.05	AG1	4	6.1	0.36
0.8	0.05	ptsI	4	6.35	0.396
0.8	0.05	clcB	4	6.35	0.354
0.8	0.05	ycaM	4	6.35	0.344
0.8	0.05	yadI	4	6.35	0.466
0.8	0.05	AG1	4	6.35	0.37
0.8	0.05	ptsI	4	6.6	0.408
0.8	0.05	clcB	4	6.6	0.358
0.8	0.05	ycaM	4	6.6	0.346
0.8	0.05	yadI	4	6.6	0.472
0.8	0.05	AG1	4	6.6	0.38
0.8	0.05	ptsI	4	6.85	0.417
0.8	0.05	clcB	4	6.85	0.361
0.8	0.05	ycaM	4	6.85	0.348
0.8	0.05	yadI	4	6.85	0.482
0.8	0.05	AG1	4	6.85	0.388
0.8	0.05	ptsI	4	7.1	0.427
0.8	0.05	clcB	4	7.1	0.362
0.8	0.05	ycaM	4	7.1	0.349
0.8	0.05	yadI	4	7.1	0.489
0.8	0.05	AG1	4	7.1	0.401
0.8	0.05	ptsI	4	7.35	0.435
0.8	0.05	clcB	4	7.35	0.365
0.8	0.05	ycaM	4	7.35	0.356
0.8	0.05	yadI	4	7.35	0.497
0.8	0.05	AG1	4	7.35	0.408
0.8	0.05	ptsI	4	7.6	0.443
0.8	0.05	clcB	4	7.6	0.367
0.8	0.05	ycaM	4	7.6	0.369
0.8	0.05	yadI	4	7.6	0.507
0.8	0.05	AG1	4	7.6	0.415

0.8	0.05	ptsI	4	7.85	0.446
0.8	0.05	clcB	4	7.85	0.364
0.8	0.05	ycaM	4	7.85	0.347
0.8	0.05	yadI	4	7.85	0.508
0.8	0.05	AG1	4	7.85	0.419
0.8	0.05	ptsI	4	8.1	0.454
0.8	0.05	clcB	4	8.1	0.37
0.8	0.05	ycaM	4	8.1	0.35
0.8	0.05	yadI	4	8.1	0.514
0.8	0.05	AG1	4	8.1	0.428
0.8	0.05	ptsI	4	8.35	0.463
0.8	0.05	clcB	4	8.35	0.372
0.8	0.05	ycaM	4	8.35	0.352
0.8	0.05	yadI	4	8.35	0.522
0.8	0.05	AG1	4	8.35	0.439
0.8	0.05	ptsI	4	8.83333	0.436
0.8	0.05	clcB	4	8.83333	0.32
0.8	0.05	ycaM	4	8.83333	0.31
0.8	0.05	yadI	4	8.83333	0.513
0.8	0.05	AG1	4	8.83333	0.457
0.8	0.05	ptsI	4	9.08333	0.438
0.8	0.05	clcB	4	9.08333	0.314
0.8	0.05	ycaM	4	9.08333	0.306
0.8	0.05	yadI	4	9.08333	0.507
0.8	0.05	AG1	4	9.08333	0.456
0.8	0.05	ptsI	4	9.33333	0.446
0.8	0.05	clcB	4	9.33333	0.311
0.8	0.05	ycaM	4	9.33333	0.301
0.8	0.05	yadI	4	9.33333	0.515
0.8	0.05	AG1	4	9.33333	0.468
0.8	0.05	ptsI	4	9.58333	0.456
0.8	0.05	clcB	4	9.58333	0.313
0.8	0.05	ycaM	4	9.58333	0.3
0.8	0.05	yadI	4	9.58333	0.528
0.8	0.05	AG1	4	9.58333	0.479
0.8	0.05	ptsI	4	9.83333	0.465
0.8	0.05	clcB	4	9.83333	0.312
0.8	0.05	ycaM	4	9.83333	0.297
0.8	0.05	yadI	4	9.83333	0.537
0.8	0.05	AG1	4	9.83333	0.488
0.8	0.05	ptsI	4	10.0833	0.474
0.8	0.05	clcB	4	10.0833	0.31
0.8	0.05	ycaM	4	10.0833	0.301
0.8	0.05	yadI	4	10.0833	0.545
0.8	0.05	AG1	4	10.0833	0.495
0.8	0.05	ptsI	4	10.3333	0.481
0.8	0.05	clcB	4	10.3333	0.311
0.8	0.05	ycaM	4	10.3333	0.301
0.8	0.05	yadI	4	10.3333	0.55
0.8	0.05	AG1	4	10.3333	0.499
0.8	0.05	ptsI	4	10.5833	0.491
0.8	0.05	clcB	4	10.5833	0.313
0.8	0.05	ycaM	4	10.5833	0.302

0.8	0.05	yadI	4	10.5833	0.555
0.8	0.05	AG1	4	10.5833	0.506
0.8	0.05	ptsI	4	10.8333	0.498
0.8	0.05	clcB	4	10.8333	0.312
0.8	0.05	ycaM	4	10.8333	0.301
0.8	0.05	yadI	4	10.8333	0.563
0.8	0.05	AG1	4	10.8333	0.513
0.8	0.05	ptsI	4	11.0833	0.504
0.8	0.05	clcB	4	11.0833	0.314
0.8	0.05	ycaM	4	11.0833	0.295
0.8	0.05	yadI	4	11.0833	0.565
0.8	0.05	AG1	4	11.0833	0.519
0.8	0.05	ptsI	4	11.3333	0.511
0.8	0.05	clcB	4	11.3333	0.311
0.8	0.05	ycaM	4	11.3333	0.297
0.8	0.05	yadI	4	11.3333	0.571
0.8	0.05	AG1	4	11.3333	0.525
0.8	0.05	ptsI	4	11.5833	0.52
0.8	0.05	clcB	4	11.5833	0.314
0.8	0.05	ycaM	4	11.5833	0.297
0.8	0.05	yadI	4	11.5833	0.579
0.8	0.05	AG1	4	11.5833	0.528
0.8	0.05	ptsI	4	11.8333	0.526
0.8	0.05	clcB	4	11.8333	0.308
0.8	0.05	ycaM	4	11.8333	0.296
0.8	0.05	yadI	4	11.8333	0.583
0.8	0.05	AG1	4	11.8333	0.532
0.8	0.05	ptsI	4	12.0833	0.531
0.8	0.05	clcB	4	12.0833	0.315
0.8	0.05	ycaM	4	12.0833	0.295
0.8	0.05	yadI	4	12.0833	0.589
0.8	0.05	AG1	4	12.0833	0.539
0.8	0.05	ptsI	4	12.3333	0.54
0.8	0.05	clcB	4	12.3333	0.316
0.8	0.05	ycaM	4	12.3333	0.302
0.8	0.05	yadI	4	12.3333	0.594
0.8	0.05	AG1	4	12.3333	0.543
0.8	0.05	ptsI	4	12.5833	0.548
0.8	0.05	clcB	4	12.5833	0.318
0.8	0.05	ycaM	4	12.5833	0.304
0.8	0.05	yadI	4	12.5833	0.592
0.8	0.05	AG1	4	12.5833	0.548
0.8	0.05	ptsI	4	12.8333	0.551
0.8	0.05	clcB	4	12.8333	0.318
0.8	0.05	ycaM	4	12.8333	0.308
0.8	0.05	yadI	4	12.8333	0.601
0.8	0.05	AG1	4	12.8333	0.553
0.8	0.05	ptsI	4	13.0833	0.557
0.8	0.05	clcB	4	13.0833	0.321
0.8	0.05	ycaM	4	13.0833	0.304
0.8	0.05	yadI	4	13.0833	0.605
0.8	0.05	AG1	4	13.0833	0.559
0.8	0.05	ptsI	4	13.3333	0.562

0.8	0.05	clcB	4	13.3333	0.324
0.8	0.05	ycaM	4	13.3333	0.304
0.8	0.05	yadI	4	13.3333	0.608
0.8	0.05	AG1	4	13.3333	0.564
0.8	0.05	ptsI	4	13.5833	0.567
0.8	0.05	clcB	4	13.5833	0.323
0.8	0.05	ycaM	4	13.5833	0.314
0.8	0.05	yadI	4	13.5833	0.61
0.8	0.05	AG1	4	13.5833	0.568
0.8	0.05	ptsI	4	13.8333	0.568
0.8	0.05	clcB	4	13.8333	0.329
0.8	0.05	ycaM	4	13.8333	0.312
0.8	0.05	yadI	4	13.8333	0.612
0.8	0.05	AG1	4	13.8333	0.573
0.8	0.05	ptsI	4	14.0833	0.575
0.8	0.05	clcB	4	14.0833	0.329
0.8	0.05	ycaM	4	14.0833	0.312
0.8	0.05	yadI	4	14.0833	0.62
0.8	0.05	AG1	4	14.0833	0.579
0.8	0.05	ptsI	4	14.3333	0.579
0.8	0.05	clcB	4	14.3333	0.331
0.8	0.05	ycaM	4	14.3333	0.316
0.8	0.05	yadI	4	14.3333	0.618
0.8	0.05	AG1	4	14.3333	0.58
0.8	0.05	ptsI	4	14.5833	0.583
0.8	0.05	clcB	4	14.5833	0.332
0.8	0.05	ycaM	4	14.5833	0.315
0.8	0.05	yadI	4	14.5833	0.622
0.8	0.05	AG1	4	14.5833	0.588
0.8	0.05	ptsI	4	14.8333	0.586
0.8	0.05	clcB	4	14.8333	0.332
0.8	0.05	ycaM	4	14.8333	0.317
0.8	0.05	yadI	4	14.8333	0.621
0.8	0.05	AG1	4	14.8333	0.591
0.8	0.05	ptsI	4	15.0833	0.588
0.8	0.05	clcB	4	15.0833	0.332
0.8	0.05	ycaM	4	15.0833	0.32
0.8	0.05	yadI	4	15.0833	0.622
0.8	0.05	AG1	4	15.0833	0.594
0.8	0.05	ptsI	4	15.3333	0.592
0.8	0.05	clcB	4	15.3333	0.338
0.8	0.05	ycaM	4	15.3333	0.32
0.8	0.05	yadI	4	15.3333	0.624
0.8	0.05	AG1	4	15.3333	0.595
0.8	0.05	ptsI	4	15.5833	0.595
0.8	0.05	clcB	4	15.5833	0.339
0.8	0.05	ycaM	4	15.5833	0.322
0.8	0.05	yadI	4	15.5833	0.627
0.8	0.05	AG1	4	15.5833	0.599
0.8	0.05	ptsI	4	15.8333	0.599
0.8	0.05	clcB	4	15.8333	0.339
0.8	0.05	ycaM	4	15.8333	0.324
0.8	0.05	yadI	4	15.8333	0.629

0.8	0.05	AG1	4	15.8333	0.604
0.8	0.05	ptsI	4	16.0833	0.599
0.8	0.05	clcB	4	16.0833	0.339
0.8	0.05	ycaM	4	16.0833	0.32
0.8	0.05	yadI	4	16.0833	0.631
0.8	0.05	AG1	4	16.0833	0.603
0.8	0.05	ptsI	4	16.3333	0.6
0.8	0.05	clcB	4	16.3333	0.34
0.8	0.05	ycaM	4	16.3333	0.325
0.8	0.05	yadI	4	16.3333	0.634
0.8	0.05	AG1	4	16.3333	0.608
0.8	0.05	ptsI	4	16.5833	0.603
0.8	0.05	clcB	4	16.5833	0.34
0.8	0.05	ycaM	4	16.5833	0.328
0.8	0.05	yadI	4	16.5833	0.635
0.8	0.05	AG1	4	16.5833	0.613
0.8	0.05	ptsI	4	16.8333	0.601
0.8	0.05	clcB	4	16.8333	0.337
0.8	0.05	ycaM	4	16.8333	0.326
0.8	0.05	yadI	4	16.8333	0.639
0.8	0.05	AG1	4	16.8333	0.61
0.8	0.05	ptsI	4	17.0833	0.602
0.8	0.05	clcB	4	17.0833	0.337
0.8	0.05	ycaM	4	17.0833	0.325
0.8	0.05	yadI	4	17.0833	0.641
0.8	0.05	AG1	4	17.0833	0.612
0.8	0.05	ptsI	4	17.3333	0.606
0.8	0.05	clcB	4	17.3333	0.343
0.8	0.05	ycaM	4	17.3333	0.327
0.8	0.05	yadI	4	17.3333	0.644
0.8	0.05	AG1	4	17.3333	0.616
0.8	0.05	ptsI	4	17.5833	0.604
0.8	0.05	clcB	4	17.5833	0.34
0.8	0.05	ycaM	4	17.5833	0.325
0.8	0.05	yadI	4	17.5833	0.647
0.8	0.05	AG1	4	17.5833	0.62
0.8	0.05	ptsI	4	17.8333	0.599
0.8	0.05	clcB	4	17.8333	0.34
0.8	0.05	ycaM	4	17.8333	0.326
0.8	0.05	yadI	4	17.8333	0.647
0.8	0.05	AG1	4	17.8333	0.616
0.8	0.05	ptsI	4	18.0833	0.605
0.8	0.05	clcB	4	18.0833	0.34
0.8	0.05	ycaM	4	18.0833	0.325
0.8	0.05	yadI	4	18.0833	0.648
0.8	0.05	AG1	4	18.0833	0.622
0.8	0.05	ptsI	4	18.3333	0.605
0.8	0.05	clcB	4	18.3333	0.338
0.8	0.05	ycaM	4	18.3333	0.328
0.8	0.05	yadI	4	18.3333	0.65
0.8	0.05	AG1	4	18.3333	0.625
0.8	0.05	ptsI	4	18.5833	0.605
0.8	0.05	clcB	4	18.5833	0.342

0.8	0.05	ycaM	4	18.5833	0.328
0.8	0.05	yadI	4	18.5833	0.652
0.8	0.05	AG1	4	18.5833	0.628
0.8	0.05	ptsI	4	18.8333	0.599
0.8	0.05	clcB	4	18.8333	0.339
0.8	0.05	ycaM	4	18.8333	0.325
0.8	0.05	yadI	4	18.8333	0.651
0.8	0.05	AG1	4	18.8333	0.625
0.8	0.05	ptsI	4	19.0833	0.603
0.8	0.05	clcB	4	19.0833	0.343
0.8	0.05	ycaM	4	19.0833	0.324
0.8	0.05	yadI	4	19.0833	0.654
0.8	0.05	AG1	4	19.0833	0.632
0.8	0.05	ptsI	4	19.3333	0.601
0.8	0.05	clcB	4	19.3333	0.342
0.8	0.05	ycaM	4	19.3333	0.329
0.8	0.05	yadI	4	19.3333	0.654
0.8	0.05	AG1	4	19.3333	0.631
0.8	0.05	ptsI	4	19.5833	0.603
0.8	0.05	clcB	4	19.5833	0.342
0.8	0.05	ycaM	4	19.5833	0.331
0.8	0.05	yadI	4	19.5833	0.658
0.8	0.05	AG1	4	19.5833	0.635
0.8	0.05	ptsI	4	19.8333	0.604
0.8	0.05	clcB	4	19.8333	0.335
0.8	0.05	ycaM	4	19.8333	0.332
0.8	0.05	yadI	4	19.8333	0.654
0.8	0.05	AG1	4	19.8333	0.641
0.8	0.05	ptsI	4	20.0833	0.605
0.8	0.05	clcB	4	20.0833	0.342
0.8	0.05	ycaM	4	20.0833	0.337
0.8	0.05	yadI	4	20.0833	0.657
0.8	0.05	AG1	4	20.0833	0.642
0.8	0.05	ptsI	4	20.3333	0.6
0.8	0.05	clcB	4	20.3333	0.333
0.8	0.05	ycaM	4	20.3333	0.338
0.8	0.05	yadI	4	20.3333	0.654
0.8	0.05	AG1	4	20.3333	0.641
0.8	0.05	ptsI	4	20.5833	0.603
0.8	0.05	clcB	4	20.5833	0.335
0.8	0.05	ycaM	4	20.5833	0.339
0.8	0.05	yadI	4	20.5833	0.659
0.8	0.05	AG1	4	20.5833	0.649
0.8	0.05	ptsI	4	20.8333	0.6
0.8	0.05	clcB	4	20.8333	0.336
0.8	0.05	ycaM	4	20.8333	0.343
0.8	0.05	yadI	4	20.8333	0.655
0.8	0.05	AG1	4	20.8333	0.647
0.8	0.05	ptsI	4	21.0833	0.6
0.8	0.05	clcB	4	21.0833	0.336
0.8	0.05	ycaM	4	21.0833	0.34
0.8	0.05	yadI	4	21.0833	0.659
0.8	0.05	AG1	4	21.0833	0.654

0.8	0.05	ptsI	4	21.3333	0.596
0.8	0.05	clcB	4	21.3333	0.34
0.8	0.05	ycaM	4	21.3333	0.342
0.8	0.05	yadI	4	21.3333	0.656
0.8	0.05	AG1	4	21.3333	0.652
0.8	0.05	ptsI	4	21.5833	0.595
0.8	0.05	clcB	4	21.5833	0.338
0.8	0.05	ycaM	4	21.5833	0.344
0.8	0.05	yadI	4	21.5833	0.66
0.8	0.05	AG1	4	21.5833	0.656
0.8	0.05	ptsI	4	21.8333	0.602
0.8	0.05	clcB	4	21.8333	0.335
0.8	0.05	ycaM	4	21.8333	0.345
0.8	0.05	yadI	4	21.8333	0.662
0.8	0.05	AG1	4	21.8333	0.655
0.8	0.05	ptsI	4	22.0833	0.592
0.8	0.05	clcB	4	22.0833	0.333
0.8	0.05	ycaM	4	22.0833	0.346
0.8	0.05	yadI	4	22.0833	0.66
0.8	0.05	AG1	4	22.0833	0.654
0.8	0.05	ptsI	4	22.3333	0.597
0.8	0.05	clcB	4	22.3333	0.331
0.8	0.05	ycaM	4	22.3333	0.345
0.8	0.05	yadI	4	22.3333	0.662
0.8	0.05	AG1	4	22.3333	0.654
0.8	0.05	ptsI	4	22.5833	0.597
0.8	0.05	clcB	4	22.5833	0.33
0.8	0.05	ycaM	4	22.5833	0.348
0.8	0.05	yadI	4	22.5833	0.667
0.8	0.05	AG1	4	22.5833	0.658
0.8	0.05	ptsI	4	22.8333	0.595
0.8	0.05	clcB	4	22.8333	0.331
0.8	0.05	ycaM	4	22.8333	0.351
0.8	0.05	yadI	4	22.8333	0.666
0.8	0.05	AG1	4	22.8333	0.662
0.8	0.05	ptsI	4	23.0833	0.593
0.8	0.05	clcB	4	23.0833	0.335
0.8	0.05	ycaM	4	23.0833	0.35
0.8	0.05	yadI	4	23.0833	0.668
0.8	0.05	AG1	4	23.0833	0.661
0.8	0.05	ptsI	4	23.3333	0.602
0.8	0.05	clcB	4	23.3333	0.337
0.8	0.05	ycaM	4	23.3333	0.355
0.8	0.05	yadI	4	23.3333	0.668
0.8	0.05	AG1	4	23.3333	0.664
0.8	0.05	ptsI	4	23.5833	0.603
0.8	0.05	clcB	4	23.5833	0.338
0.8	0.05	ycaM	4	23.5833	0.354
0.8	0.05	yadI	4	23.5833	0.669
0.8	0.05	AG1	4	23.5833	0.667
0.8	0.05	ptsI	4	23.8333	0.6
0.8	0.05	clcB	4	23.8333	0.335
0.8	0.05	ycaM	4	23.8333	0.354

0.8	0.05	yadI	4	23.8333	0.668
0.8	0.05	AG1	4	23.8333	0.668
0.8	0.05	ptsI	4	24.0833	0.594
0.8	0.05	clcB	4	24.0833	0.339
0.8	0.05	ycaM	4	24.0833	0.354
0.8	0.05	yadI	4	24.0833	0.665
0.8	0.05	AG1	4	24.0833	0.669
0.8	0.05	ptsI	5	0	0.164
0.8	0.05	clcB	5	0	0.171
0.8	0.05	ycaM	5	0	0.163
0.8	0.05	yadI	5	0	0.17
0.8	0.05	AG1	5	0	0.162
0.8	0.05	ptsI	5	0.35	0.157
0.8	0.05	clcB	5	0.35	0.167
0.8	0.05	ycaM	5	0.35	0.156
0.8	0.05	yadI	5	0.35	0.166
0.8	0.05	AG1	5	0.35	0.161
0.8	0.05	ptsI	5	0.6	0.157
0.8	0.05	clcB	5	0.6	0.168
0.8	0.05	ycaM	5	0.6	0.156
0.8	0.05	yadI	5	0.6	0.168
0.8	0.05	AG1	5	0.6	0.161
0.8	0.05	ptsI	5	0.85	0.155
0.8	0.05	clcB	5	0.85	0.165
0.8	0.05	ycaM	5	0.85	0.154
0.8	0.05	yadI	5	0.85	0.167
0.8	0.05	AG1	5	0.85	0.162
0.8	0.05	ptsI	5	1.1	0.156
0.8	0.05	clcB	5	1.1	0.17
0.8	0.05	ycaM	5	1.1	0.157
0.8	0.05	yadI	5	1.1	0.168
0.8	0.05	AG1	5	1.1	0.165
0.8	0.05	ptsI	5	1.35	0.157
0.8	0.05	clcB	5	1.35	0.17
0.8	0.05	ycaM	5	1.35	0.156
0.8	0.05	yadI	5	1.35	0.17
0.8	0.05	AG1	5	1.35	0.164
0.8	0.05	ptsI	5	1.6	0.16
0.8	0.05	clcB	5	1.6	0.172
0.8	0.05	ycaM	5	1.6	0.158
0.8	0.05	yadI	5	1.6	0.173
0.8	0.05	AG1	5	1.6	0.166
0.8	0.05	ptsI	5	1.85	0.162
0.8	0.05	clcB	5	1.85	0.176
0.8	0.05	ycaM	5	1.85	0.162
0.8	0.05	yadI	5	1.85	0.178
0.8	0.05	AG1	5	1.85	0.168
0.8	0.05	ptsI	5	2.1	0.166
0.8	0.05	clcB	5	2.1	0.182
0.8	0.05	ycaM	5	2.1	0.167
0.8	0.05	yadI	5	2.1	0.18
0.8	0.05	AG1	5	2.1	0.173
0.8	0.05	ptsI	5	2.35	0.169

0.8	0.05	clcB	5	2.35	0.187
0.8	0.05	ycaM	5	2.35	0.171
0.8	0.05	yadI	5	2.35	0.187
0.8	0.05	AG1	5	2.35	0.177
0.8	0.05	ptsI	5	2.6	0.165
0.8	0.05	clcB	5	2.6	0.186
0.8	0.05	ycaM	5	2.6	0.176
0.8	0.05	yadI	5	2.6	0.194
0.8	0.05	AG1	5	2.6	0.175
0.8	0.05	ptsI	5	2.85	0.176
0.8	0.05	clcB	5	2.85	0.196
0.8	0.05	ycaM	5	2.85	0.18
0.8	0.05	yadI	5	2.85	0.199
0.8	0.05	AG1	5	2.85	0.182
0.8	0.05	ptsI	5	3.1	0.181
0.8	0.05	clcB	5	3.1	0.206
0.8	0.05	ycaM	5	3.1	0.186
0.8	0.05	yadI	5	3.1	0.204
0.8	0.05	AG1	5	3.1	0.189
0.8	0.05	ptsI	5	3.35	0.191
0.8	0.05	clcB	5	3.35	0.217
0.8	0.05	ycaM	5	3.35	0.196
0.8	0.05	yadI	5	3.35	0.213
0.8	0.05	AG1	5	3.35	0.195
0.8	0.05	ptsI	5	3.6	0.199
0.8	0.05	clcB	5	3.6	0.231
0.8	0.05	ycaM	5	3.6	0.209
0.8	0.05	yadI	5	3.6	0.221
0.8	0.05	AG1	5	3.6	0.203
0.8	0.05	ptsI	5	3.85	0.209
0.8	0.05	clcB	5	3.85	0.246
0.8	0.05	ycaM	5	3.85	0.222
0.8	0.05	yadI	5	3.85	0.229
0.8	0.05	AG1	5	3.85	0.21
0.8	0.05	ptsI	5	4.38333	0.214
0.8	0.05	clcB	5	4.38333	0.317
0.8	0.05	ycaM	5	4.38333	0.224
0.8	0.05	yadI	5	4.38333	0.283
0.8	0.05	AG1	5	4.38333	0.248
0.8	0.05	ptsI	5	4.63333	0.212
0.8	0.05	clcB	5	4.63333	0.271
0.8	0.05	ycaM	5	4.63333	0.216
0.8	0.05	yadI	5	4.63333	0.281
0.8	0.05	AG1	5	4.63333	0.24
0.8	0.05	ptsI	5	4.88333	0.22
0.8	0.05	clcB	5	4.88333	0.286
0.8	0.05	ycaM	5	4.88333	0.226
0.8	0.05	yadI	5	4.88333	0.299
0.8	0.05	AG1	5	4.88333	0.255
0.8	0.05	ptsI	5	5.13333	0.229
0.8	0.05	clcB	5	5.13333	0.295
0.8	0.05	ycaM	5	5.13333	0.231
0.8	0.05	yadI	5	5.13333	0.313

0.8	0.05	AG1	5	5.13333	0.264
0.8	0.05	ptsI	5	5.38333	0.239
0.8	0.05	clcB	5	5.38333	0.302
0.8	0.05	ycaM	5	5.38333	0.232
0.8	0.05	yadI	5	5.38333	0.333
0.8	0.05	AG1	5	5.38333	0.28
0.8	0.05	ptsI	5	5.63333	0.248
0.8	0.05	clcB	5	5.63333	0.311
0.8	0.05	ycaM	5	5.63333	0.236
0.8	0.05	yadI	5	5.63333	0.348
0.8	0.05	AG1	5	5.63333	0.295
0.8	0.05	ptsI	5	5.88333	0.258
0.8	0.05	clcB	5	5.88333	0.315
0.8	0.05	ycaM	5	5.88333	0.236
0.8	0.05	yadI	5	5.88333	0.361
0.8	0.05	AG1	5	5.88333	0.304
0.8	0.05	ptsI	5	6.13333	0.271
0.8	0.05	clcB	5	6.13333	0.33
0.8	0.05	ycaM	5	6.13333	0.241
0.8	0.05	yadI	5	6.13333	0.374
0.8	0.05	AG1	5	6.13333	0.317
0.8	0.05	ptsI	5	6.38333	0.286
0.8	0.05	clcB	5	6.38333	0.342
0.8	0.05	ycaM	5	6.38333	0.242
0.8	0.05	yadI	5	6.38333	0.388
0.8	0.05	AG1	5	6.38333	0.329
0.8	0.05	ptsI	5	6.63333	0.297
0.8	0.05	clcB	5	6.63333	0.351
0.8	0.05	ycaM	5	6.63333	0.245
0.8	0.05	yadI	5	6.63333	0.4
0.8	0.05	AG1	5	6.63333	0.342
0.8	0.05	ptsI	5	6.88333	0.31
0.8	0.05	clcB	5	6.88333	0.359
0.8	0.05	ycaM	5	6.88333	0.248
0.8	0.05	yadI	5	6.88333	0.413
0.8	0.05	AG1	5	6.88333	0.354
0.8	0.05	ptsI	5	7.13333	0.324
0.8	0.05	clcB	5	7.13333	0.368
0.8	0.05	ycaM	5	7.13333	0.25
0.8	0.05	yadI	5	7.13333	0.425
0.8	0.05	AG1	5	7.13333	0.363
0.8	0.05	ptsI	5	7.38333	0.337
0.8	0.05	clcB	5	7.38333	0.373
0.8	0.05	ycaM	5	7.38333	0.251
0.8	0.05	yadI	5	7.38333	0.438
0.8	0.05	AG1	5	7.38333	0.377
0.8	0.05	ptsI	5	7.63333	0.352
0.8	0.05	clcB	5	7.63333	0.38
0.8	0.05	ycaM	5	7.63333	0.253
0.8	0.05	yadI	5	7.63333	0.448
0.8	0.05	AG1	5	7.63333	0.388
0.8	0.05	ptsI	5	7.88333	0.369
0.8	0.05	clcB	5	7.88333	0.391

0.8	0.05	ycaM	5	7.88333	0.257
0.8	0.05	yadI	5	7.88333	0.457
0.8	0.05	AG1	5	7.88333	0.399
0.8	0.05	ptsI	5	8.13333	0.382
0.8	0.05	clcB	5	8.13333	0.392
0.8	0.05	ycaM	5	8.13333	0.261
0.8	0.05	yadI	5	8.13333	0.468
0.8	0.05	AG1	5	8.13333	0.412
0.8	0.05	ptsI	5	8.38333	0.4
0.8	0.05	clcB	5	8.38333	0.398
0.8	0.05	ycaM	5	8.38333	0.262
0.8	0.05	yadI	5	8.38333	0.473
0.8	0.05	AG1	5	8.38333	0.423
0.8	0.05	ptsI	5	8.88333	0.353
0.8	0.05	clcB	5	8.88333	0.291
0.8	0.05	ycaM	5	8.88333	0.25
0.8	0.05	yadI	5	8.88333	0.495
0.8	0.05	AG1	5	8.88333	0.468
0.8	0.05	ptsI	5	9.13333	0.357
0.8	0.05	clcB	5	9.13333	0.288
0.8	0.05	ycaM	5	9.13333	0.245
0.8	0.05	yadI	5	9.13333	0.486
0.8	0.05	AG1	5	9.13333	0.475
0.8	0.05	ptsI	5	9.38333	0.372
0.8	0.05	clcB	5	9.38333	0.29
0.8	0.05	ycaM	5	9.38333	0.243
0.8	0.05	yadI	5	9.38333	0.495
0.8	0.05	AG1	5	9.38333	0.498
0.8	0.05	ptsI	5	9.63333	0.383
0.8	0.05	clcB	5	9.63333	0.29
0.8	0.05	ycaM	5	9.63333	0.246
0.8	0.05	yadI	5	9.63333	0.507
0.8	0.05	AG1	5	9.63333	0.511
0.8	0.05	ptsI	5	9.88333	0.392
0.8	0.05	clcB	5	9.88333	0.292
0.8	0.05	ycaM	5	9.88333	0.247
0.8	0.05	yadI	5	9.88333	0.52
0.8	0.05	AG1	5	9.88333	0.523
0.8	0.05	ptsI	5	10.1333	0.403
0.8	0.05	clcB	5	10.1333	0.292
0.8	0.05	ycaM	5	10.1333	0.247
0.8	0.05	yadI	5	10.1333	0.53
0.8	0.05	AG1	5	10.1333	0.533
0.8	0.05	ptsI	5	10.3833	0.412
0.8	0.05	clcB	5	10.3833	0.294
0.8	0.05	ycaM	5	10.3833	0.248
0.8	0.05	yadI	5	10.3833	0.537
0.8	0.05	AG1	5	10.3833	0.545
0.8	0.05	ptsI	5	10.6333	0.42
0.8	0.05	clcB	5	10.6333	0.294
0.8	0.05	ycaM	5	10.6333	0.248
0.8	0.05	yadI	5	10.6333	0.544
0.8	0.05	AG1	5	10.6333	0.552

0.8	0.05	ptsI	5	10.8833	0.429
0.8	0.05	clcB	5	10.8833	0.296
0.8	0.05	ycaM	5	10.8833	0.249
0.8	0.05	yadI	5	10.8833	0.552
0.8	0.05	AG1	5	10.8833	0.562
0.8	0.05	ptsI	5	11.1333	0.436
0.8	0.05	clcB	5	11.1333	0.294
0.8	0.05	ycaM	5	11.1333	0.247
0.8	0.05	yadI	5	11.1333	0.557
0.8	0.05	AG1	5	11.1333	0.569
0.8	0.05	ptsI	5	11.3833	0.445
0.8	0.05	clcB	5	11.3833	0.298
0.8	0.05	ycaM	5	11.3833	0.251
0.8	0.05	yadI	5	11.3833	0.564
0.8	0.05	AG1	5	11.3833	0.577
0.8	0.05	ptsI	5	11.6333	0.453
0.8	0.05	clcB	5	11.6333	0.297
0.8	0.05	ycaM	5	11.6333	0.252
0.8	0.05	yadI	5	11.6333	0.571
0.8	0.05	AG1	5	11.6333	0.585
0.8	0.05	ptsI	5	11.8833	0.46
0.8	0.05	clcB	5	11.8833	0.296
0.8	0.05	ycaM	5	11.8833	0.25
0.8	0.05	yadI	5	11.8833	0.578
0.8	0.05	AG1	5	11.8833	0.595
0.8	0.05	ptsI	5	12.1333	0.468
0.8	0.05	clcB	5	12.1333	0.299
0.8	0.05	ycaM	5	12.1333	0.252
0.8	0.05	yadI	5	12.1333	0.58
0.8	0.05	AG1	5	12.1333	0.605
0.8	0.05	ptsI	5	12.3833	0.475
0.8	0.05	clcB	5	12.3833	0.295
0.8	0.05	ycaM	5	12.3833	0.25
0.8	0.05	yadI	5	12.3833	0.57
0.8	0.05	AG1	5	12.3833	0.613
0.8	0.05	ptsI	5	12.6333	0.481
0.8	0.05	clcB	5	12.6333	0.299
0.8	0.05	ycaM	5	12.6333	0.251
0.8	0.05	yadI	5	12.6333	0.594
0.8	0.05	AG1	5	12.6333	0.624
0.8	0.05	ptsI	5	12.8833	0.487
0.8	0.05	clcB	5	12.8833	0.297
0.8	0.05	ycaM	5	12.8833	0.251
0.8	0.05	yadI	5	12.8833	0.597
0.8	0.05	AG1	5	12.8833	0.627
0.8	0.05	ptsI	5	13.1333	0.492
0.8	0.05	clcB	5	13.1333	0.294
0.8	0.05	ycaM	5	13.1333	0.255
0.8	0.05	yadI	5	13.1333	0.603
0.8	0.05	AG1	5	13.1333	0.637
0.8	0.05	ptsI	5	13.3833	0.5
0.8	0.05	clcB	5	13.3833	0.296
0.8	0.05	ycaM	5	13.3833	0.255

0.8	0.05	yadI	5	13.3833	0.606
0.8	0.05	AG1	5	13.3833	0.638
0.8	0.05	ptsI	5	13.6333	0.509
0.8	0.05	clcB	5	13.6333	0.293
0.8	0.05	ycaM	5	13.6333	0.256
0.8	0.05	yadI	5	13.6333	0.612
0.8	0.05	AG1	5	13.6333	0.647
0.8	0.05	ptsI	5	13.8833	0.511
0.8	0.05	clcB	5	13.8833	0.294
0.8	0.05	ycaM	5	13.8833	0.256
0.8	0.05	yadI	5	13.8833	0.617
0.8	0.05	AG1	5	13.8833	0.655
0.8	0.05	ptsI	5	14.1333	0.517
0.8	0.05	clcB	5	14.1333	0.293
0.8	0.05	ycaM	5	14.1333	0.257
0.8	0.05	yadI	5	14.1333	0.623
0.8	0.05	AG1	5	14.1333	0.664
0.8	0.05	ptsI	5	14.3833	0.523
0.8	0.05	clcB	5	14.3833	0.296
0.8	0.05	ycaM	5	14.3833	0.259
0.8	0.05	yadI	5	14.3833	0.621
0.8	0.05	AG1	5	14.3833	0.666
0.8	0.05	ptsI	5	14.6333	0.526
0.8	0.05	clcB	5	14.6333	0.292
0.8	0.05	ycaM	5	14.6333	0.257
0.8	0.05	yadI	5	14.6333	0.622
0.8	0.05	AG1	5	14.6333	0.675
0.8	0.05	ptsI	5	14.8833	0.535
0.8	0.05	clcB	5	14.8833	0.292
0.8	0.05	ycaM	5	14.8833	0.259
0.8	0.05	yadI	5	14.8833	0.628
0.8	0.05	AG1	5	14.8833	0.682
0.8	0.05	ptsI	5	15.1333	0.535
0.8	0.05	clcB	5	15.1333	0.294
0.8	0.05	ycaM	5	15.1333	0.258
0.8	0.05	yadI	5	15.1333	0.627
0.8	0.05	AG1	5	15.1333	0.686
0.8	0.05	ptsI	5	15.3833	0.542
0.8	0.05	clcB	5	15.3833	0.293
0.8	0.05	ycaM	5	15.3833	0.259
0.8	0.05	yadI	5	15.3833	0.629
0.8	0.05	AG1	5	15.3833	0.689
0.8	0.05	ptsI	5	15.6333	0.546
0.8	0.05	clcB	5	15.6333	0.295
0.8	0.05	ycaM	5	15.6333	0.259
0.8	0.05	yadI	5	15.6333	0.635
0.8	0.05	AG1	5	15.6333	0.698
0.8	0.05	ptsI	5	15.8833	0.551
0.8	0.05	clcB	5	15.8833	0.296
0.8	0.05	ycaM	5	15.8833	0.26
0.8	0.05	yadI	5	15.8833	0.635
0.8	0.05	AG1	5	15.8833	0.696
0.8	0.05	ptsI	5	16.1333	0.56

0.8	0.05	clcB	5	16.1333	0.299
0.8	0.05	ycaM	5	16.1333	0.261
0.8	0.05	yadI	5	16.1333	0.638
0.8	0.05	AG1	5	16.1333	0.71
0.8	0.05	ptsI	5	16.3833	0.559
0.8	0.05	clcB	5	16.3833	0.296
0.8	0.05	ycaM	5	16.3833	0.261
0.8	0.05	yadI	5	16.3833	0.642
0.8	0.05	AG1	5	16.3833	0.704
0.8	0.05	ptsI	5	16.6333	0.564
0.8	0.05	clcB	5	16.6333	0.3
0.8	0.05	ycaM	5	16.6333	0.262
0.8	0.05	yadI	5	16.6333	0.643
0.8	0.05	AG1	5	16.6333	0.71
0.8	0.05	ptsI	5	16.8833	0.567
0.8	0.05	clcB	5	16.8833	0.297
0.8	0.05	ycaM	5	16.8833	0.262
0.8	0.05	yadI	5	16.8833	0.647
0.8	0.05	AG1	5	16.8833	0.714
0.8	0.05	ptsI	5	17.1333	0.572
0.8	0.05	clcB	5	17.1333	0.298
0.8	0.05	ycaM	5	17.1333	0.261
0.8	0.05	yadI	5	17.1333	0.643
0.8	0.05	AG1	5	17.1333	0.72
0.8	0.05	ptsI	5	17.3833	0.577
0.8	0.05	clcB	5	17.3833	0.301
0.8	0.05	ycaM	5	17.3833	0.265
0.8	0.05	yadI	5	17.3833	0.647
0.8	0.05	AG1	5	17.3833	0.723
0.8	0.05	ptsI	5	17.6333	0.58
0.8	0.05	clcB	5	17.6333	0.302
0.8	0.05	ycaM	5	17.6333	0.266
0.8	0.05	yadI	5	17.6333	0.648
0.8	0.05	AG1	5	17.6333	0.729
0.8	0.05	ptsI	5	17.8833	0.58
0.8	0.05	clcB	5	17.8833	0.3
0.8	0.05	ycaM	5	17.8833	0.266
0.8	0.05	yadI	5	17.8833	0.631
0.8	0.05	AG1	5	17.8833	0.727
0.8	0.05	ptsI	5	18.1333	0.587
0.8	0.05	clcB	5	18.1333	0.305
0.8	0.05	ycaM	5	18.1333	0.268
0.8	0.05	yadI	5	18.1333	0.654
0.8	0.05	AG1	5	18.1333	0.733
0.8	0.05	ptsI	5	18.3833	0.588
0.8	0.05	clcB	5	18.3833	0.306
0.8	0.05	ycaM	5	18.3833	0.268
0.8	0.05	yadI	5	18.3833	0.654
0.8	0.05	AG1	5	18.3833	0.735
0.8	0.05	ptsI	5	18.6333	0.588
0.8	0.05	clcB	5	18.6333	0.304
0.8	0.05	ycaM	5	18.6333	0.27
0.8	0.05	yadI	5	18.6333	0.658

0.8	0.05	AG1	5	18.6333	0.736
0.8	0.05	ptsI	5	18.8833	0.593
0.8	0.05	clcB	5	18.8833	0.306
0.8	0.05	ycaM	5	18.8833	0.272
0.8	0.05	yadI	5	18.8833	0.66
0.8	0.05	AG1	5	18.8833	0.741
0.8	0.05	ptsI	5	19.1333	0.598
0.8	0.05	clcB	5	19.1333	0.309
0.8	0.05	ycaM	5	19.1333	0.272
0.8	0.05	yadI	5	19.1333	0.66
0.8	0.05	AG1	5	19.1333	0.745
0.8	0.05	ptsI	5	19.3833	0.601
0.8	0.05	clcB	5	19.3833	0.312
0.8	0.05	ycaM	5	19.3833	0.274
0.8	0.05	yadI	5	19.3833	0.665
0.8	0.05	AG1	5	19.3833	0.751
0.8	0.05	ptsI	5	19.6333	0.604
0.8	0.05	clcB	5	19.6333	0.316
0.8	0.05	ycaM	5	19.6333	0.276
0.8	0.05	yadI	5	19.6333	0.662
0.8	0.05	AG1	5	19.6333	0.752
0.8	0.05	ptsI	5	19.8833	0.601
0.8	0.05	clcB	5	19.8833	0.317
0.8	0.05	ycaM	5	19.8833	0.277
0.8	0.05	yadI	5	19.8833	0.66
0.8	0.05	AG1	5	19.8833	0.756
0.8	0.05	ptsI	5	20.1333	0.603
0.8	0.05	clcB	5	20.1333	0.32
0.8	0.05	ycaM	5	20.1333	0.278
0.8	0.05	yadI	5	20.1333	0.664
0.8	0.05	AG1	5	20.1333	0.761
0.8	0.05	ptsI	5	20.3833	0.604
0.8	0.05	clcB	5	20.3833	0.324
0.8	0.05	ycaM	5	20.3833	0.28
0.8	0.05	yadI	5	20.3833	0.666
0.8	0.05	AG1	5	20.3833	0.761
0.8	0.05	ptsI	5	20.6333	0.607
0.8	0.05	clcB	5	20.6333	0.321
0.8	0.05	ycaM	5	20.6333	0.281
0.8	0.05	yadI	5	20.6333	0.644
0.8	0.05	AG1	5	20.6333	0.756
0.8	0.05	ptsI	5	20.8833	0.606
0.8	0.05	clcB	5	20.8833	0.323
0.8	0.05	ycaM	5	20.8833	0.284
0.8	0.05	yadI	5	20.8833	0.67
0.8	0.05	AG1	5	20.8833	0.77
0.8	0.05	ptsI	5	21.1333	0.607
0.8	0.05	clcB	5	21.1333	0.324
0.8	0.05	ycaM	5	21.1333	0.286
0.8	0.05	yadI	5	21.1333	0.672
0.8	0.05	AG1	5	21.1333	0.772
0.8	0.05	ptsI	5	21.3833	0.609
0.8	0.05	clcB	5	21.3833	0.319

0.8	0.05	ycaM	5	21.3833	0.29
0.8	0.05	yadI	5	21.3833	0.671
0.8	0.05	AG1	5	21.3833	0.772
0.8	0.05	ptsI	5	21.6333	0.611
0.8	0.05	clcB	5	21.6333	0.324
0.8	0.05	ycaM	5	21.6333	0.292
0.8	0.05	yadI	5	21.6333	0.67
0.8	0.05	AG1	5	21.6333	0.775
0.8	0.05	ptsI	5	21.8833	0.607
0.8	0.05	clcB	5	21.8833	0.324
0.8	0.05	ycaM	5	21.8833	0.297
0.8	0.05	yadI	5	21.8833	0.668
0.8	0.05	AG1	5	21.8833	0.772
0.8	0.05	ptsI	5	22.1333	0.61
0.8	0.05	clcB	5	22.1333	0.323
0.8	0.05	ycaM	5	22.1333	0.3
0.8	0.05	yadI	5	22.1333	0.648
0.8	0.05	AG1	5	22.1333	0.771
0.8	0.05	ptsI	5	22.3833	0.609
0.8	0.05	clcB	5	22.3833	0.324
0.8	0.05	ycaM	5	22.3833	0.301
0.8	0.05	yadI	5	22.3833	0.672
0.8	0.05	AG1	5	22.3833	0.778
0.8	0.05	ptsI	5	22.6333	0.609
0.8	0.05	clcB	5	22.6333	0.321
0.8	0.05	ycaM	5	22.6333	0.306
0.8	0.05	yadI	5	22.6333	0.674
0.8	0.05	AG1	5	22.6333	0.781
0.8	0.05	ptsI	5	22.8833	0.61
0.8	0.05	clcB	5	22.8833	0.319
0.8	0.05	ycaM	5	22.8833	0.308
0.8	0.05	yadI	5	22.8833	0.672
0.8	0.05	AG1	5	22.8833	0.777
0.8	0.05	ptsI	5	23.1333	0.611
0.8	0.05	clcB	5	23.1333	0.32
0.8	0.05	ycaM	5	23.1333	0.311
0.8	0.05	yadI	5	23.1333	0.676
0.8	0.05	AG1	5	23.1333	0.784
0.8	0.05	ptsI	5	23.3833	0.612
0.8	0.05	clcB	5	23.3833	0.323
0.8	0.05	ycaM	5	23.3833	0.314
0.8	0.05	yadI	5	23.3833	0.674
0.8	0.05	AG1	5	23.3833	0.784
0.8	0.05	ptsI	5	23.6333	0.612
0.8	0.05	clcB	5	23.6333	0.324
0.8	0.05	ycaM	5	23.6333	0.317
0.8	0.05	yadI	5	23.6333	0.676
0.8	0.05	AG1	5	23.6333	0.785
0.8	0.05	ptsI	5	23.8833	0.617
0.8	0.05	clcB	5	23.8833	0.321
0.8	0.05	ycaM	5	23.8833	0.323
0.8	0.05	yadI	5	23.8833	0.654
0.8	0.05	AG1	5	23.8833	0.781

0.8	0.05	ptsI	5	24.1333	0.612
0.8	0.05	clcB	5	24.1333	0.324
0.8	0.05	ycaM	5	24.1333	0.325
0.8	0.05	yadI	5	24.1333	0.675
0.8	0.05	AG1	5	24.1333	0.79
0.8	0.05	ptsI	5	24.3833	0.611
0.8	0.05	clcB	5	24.3833	0.319
0.8	0.05	ycaM	5	24.3833	0.328
0.8	0.05	yadI	5	24.3833	0.674
0.8	0.05	AG1	5	24.3833	0.79
1.6	0.05	ptsI	2	0	0.22
1.6	0.05	clcB	2	0	0.201
1.6	0.05	ycaM	2	0	0.21
1.6	0.05	yadI	2	0	0.222
1.6	0.05	AG1	2	0	0.233
1.6	0.05	ptsI	2	0.25	0.218
1.6	0.05	clcB	2	0.25	0.2
1.6	0.05	ycaM	2	0.25	0.204
1.6	0.05	yadI	2	0.25	0.218
1.6	0.05	AG1	2	0.25	0.225
1.6	0.05	ptsI	2	0.5	0.216
1.6	0.05	clcB	2	0.5	0.199
1.6	0.05	ycaM	2	0.5	0.206
1.6	0.05	yadI	2	0.5	0.219
1.6	0.05	AG1	2	0.5	0.228
1.6	0.05	ptsI	2	0.75	0.218
1.6	0.05	clcB	2	0.75	0.2
1.6	0.05	ycaM	2	0.75	0.207
1.6	0.05	yadI	2	0.75	0.223
1.6	0.05	AG1	2	0.75	0.229
1.6	0.05	ptsI	2	1	0.219
1.6	0.05	clcB	2	1	0.201
1.6	0.05	ycaM	2	1	0.21
1.6	0.05	yadI	2	1	0.23
1.6	0.05	AG1	2	1	0.232
1.6	0.05	ptsI	2	1.25	0.222
1.6	0.05	clcB	2	1.25	0.202
1.6	0.05	ycaM	2	1.25	0.21
1.6	0.05	yadI	2	1.25	0.234
1.6	0.05	AG1	2	1.25	0.233
1.6	0.05	ptsI	2	1.5	0.222
1.6	0.05	clcB	2	1.5	0.207
1.6	0.05	ycaM	2	1.5	0.212
1.6	0.05	yadI	2	1.5	0.24
1.6	0.05	AG1	2	1.5	0.235
1.6	0.05	ptsI	2	1.75	0.227
1.6	0.05	clcB	2	1.75	0.206
1.6	0.05	ycaM	2	1.75	0.216
1.6	0.05	yadI	2	1.75	0.246
1.6	0.05	AG1	2	1.75	0.24
1.6	0.05	ptsI	2	2	0.231
1.6	0.05	clcB	2	2	0.21
1.6	0.05	ycaM	2	2	0.224

1.6	0.05	yadI	2	2	0.253
1.6	0.05	AG1	2	2	0.247
1.6	0.05	ptsI	2	2.25	0.238
1.6	0.05	clcB	2	2.25	0.214
1.6	0.05	ycaM	2	2.25	0.23
1.6	0.05	yadI	2	2.25	0.263
1.6	0.05	AG1	2	2.25	0.254
1.6	0.05	ptsI	2	2.5	0.245
1.6	0.05	clcB	2	2.5	0.218
1.6	0.05	ycaM	2	2.5	0.239
1.6	0.05	yadI	2	2.5	0.277
1.6	0.05	AG1	2	2.5	0.262
1.6	0.05	ptsI	2	2.75	0.244
1.6	0.05	clcB	2	2.75	0.218
1.6	0.05	ycaM	2	2.75	0.238
1.6	0.05	yadI	2	2.75	0.283
1.6	0.05	AG1	2	2.75	0.259
1.6	0.05	ptsI	2	3	0.249
1.6	0.05	clcB	2	3	0.22
1.6	0.05	ycaM	2	3	0.239
1.6	0.05	yadI	2	3	0.29
1.6	0.05	AG1	2	3	0.259
1.6	0.05	ptsI	2	3.25	0.255
1.6	0.05	clcB	2	3.25	0.229
1.6	0.05	ycaM	2	3.25	0.249
1.6	0.05	yadI	2	3.25	0.308
1.6	0.05	AG1	2	3.25	0.268
1.6	0.05	ptsI	2	3.5	0.26
1.6	0.05	clcB	2	3.5	0.232
1.6	0.05	ycaM	2	3.5	0.256
1.6	0.05	yadI	2	3.5	0.319
1.6	0.05	AG1	2	3.5	0.273
1.6	0.05	ptsI	2	3.75	0.269
1.6	0.05	clcB	2	3.75	0.238
1.6	0.05	ycaM	2	3.75	0.265
1.6	0.05	yadI	2	3.75	0.332
1.6	0.05	AG1	2	3.75	0.285
1.6	0.05	ptsI	2	4	0.277
1.6	0.05	clcB	2	4	0.252
1.6	0.05	ycaM	2	4	0.279
1.6	0.05	yadI	2	4	0.348
1.6	0.05	AG1	2	4	0.295
1.6	0.05	ptsI	2	4.38333	0.301
1.6	0.05	clcB	2	4.38333	0.274
1.6	0.05	ycaM	2	4.38333	0.288
1.6	0.05	yadI	2	4.38333	0.333
1.6	0.05	AG1	2	4.38333	0.312
1.6	0.05	ptsI	2	4.63333	0.301
1.6	0.05	clcB	2	4.63333	0.267
1.6	0.05	ycaM	2	4.63333	0.287
1.6	0.05	yadI	2	4.63333	0.336
1.6	0.05	AG1	2	4.63333	0.307
1.6	0.05	ptsI	2	4.88333	0.305

1.6	0.05	clcB	2	4.88333	0.272
1.6	0.05	ycaM	2	4.88333	0.292
1.6	0.05	yadI	2	4.88333	0.35
1.6	0.05	AG1	2	4.88333	0.317
1.6	0.05	ptsI	2	5.13333	0.313
1.6	0.05	clcB	2	5.13333	0.278
1.6	0.05	ycaM	2	5.13333	0.296
1.6	0.05	yadI	2	5.13333	0.365
1.6	0.05	AG1	2	5.13333	0.327
1.6	0.05	ptsI	2	5.38333	0.32
1.6	0.05	clcB	2	5.38333	0.284
1.6	0.05	ycaM	2	5.38333	0.296
1.6	0.05	yadI	2	5.38333	0.375
1.6	0.05	AG1	2	5.38333	0.338
1.6	0.05	ptsI	2	5.63333	0.327
1.6	0.05	clcB	2	5.63333	0.292
1.6	0.05	ycaM	2	5.63333	0.298
1.6	0.05	yadI	2	5.63333	0.386
1.6	0.05	AG1	2	5.63333	0.346
1.6	0.05	ptsI	2	5.88333	0.335
1.6	0.05	clcB	2	5.88333	0.299
1.6	0.05	ycaM	2	5.88333	0.3
1.6	0.05	yadI	2	5.88333	0.393
1.6	0.05	AG1	2	5.88333	0.355
1.6	0.05	ptsI	2	6.13333	0.344
1.6	0.05	clcB	2	6.13333	0.304
1.6	0.05	ycaM	2	6.13333	0.302
1.6	0.05	yadI	2	6.13333	0.401
1.6	0.05	AG1	2	6.13333	0.365
1.6	0.05	ptsI	2	6.38333	0.353
1.6	0.05	clcB	2	6.38333	0.312
1.6	0.05	ycaM	2	6.38333	0.302
1.6	0.05	yadI	2	6.38333	0.41
1.6	0.05	AG1	2	6.38333	0.374
1.6	0.05	ptsI	2	6.63333	0.361
1.6	0.05	clcB	2	6.63333	0.317
1.6	0.05	ycaM	2	6.63333	0.304
1.6	0.05	yadI	2	6.63333	0.413
1.6	0.05	AG1	2	6.63333	0.382
1.6	0.05	ptsI	2	6.88333	0.37
1.6	0.05	clcB	2	6.88333	0.323
1.6	0.05	ycaM	2	6.88333	0.304
1.6	0.05	yadI	2	6.88333	0.423
1.6	0.05	AG1	2	6.88333	0.391
1.6	0.05	ptsI	2	7.13333	0.38
1.6	0.05	clcB	2	7.13333	0.329
1.6	0.05	ycaM	2	7.13333	0.307
1.6	0.05	yadI	2	7.13333	0.434
1.6	0.05	AG1	2	7.13333	0.401
1.6	0.05	ptsI	2	7.38333	0.392
1.6	0.05	clcB	2	7.38333	0.336
1.6	0.05	ycaM	2	7.38333	0.309
1.6	0.05	yadI	2	7.38333	0.441

1.6	0.05	AG1	2	7.38333	0.411
1.6	0.05	ptsI	2	7.63333	0.396
1.6	0.05	clcB	2	7.63333	0.341
1.6	0.05	ycaM	2	7.63333	0.308
1.6	0.05	yadI	2	7.63333	0.439
1.6	0.05	AG1	2	7.63333	0.418
1.6	0.05	ptsI	2	7.88333	0.405
1.6	0.05	clcB	2	7.88333	0.347
1.6	0.05	ycaM	2	7.88333	0.309
1.6	0.05	yadI	2	7.88333	0.448
1.6	0.05	AG1	2	7.88333	0.424
1.6	0.05	ptsI	2	8.13333	0.417
1.6	0.05	clcB	2	8.13333	0.349
1.6	0.05	ycaM	2	8.13333	0.312
1.6	0.05	yadI	2	8.13333	0.456
1.6	0.05	AG1	2	8.13333	0.433
1.6	0.05	ptsI	2	8.38333	0.424
1.6	0.05	clcB	2	8.38333	0.355
1.6	0.05	ycaM	2	8.38333	0.31
1.6	0.05	yadI	2	8.38333	0.463
1.6	0.05	AG1	2	8.38333	0.441
1.6	0.05	ptsI	2	8.91667	0.348
1.6	0.05	clcB	2	8.91667	0.295
1.6	0.05	ycaM	2	8.91667	0.282
1.6	0.05	yadI	2	8.91667	0.503
1.6	0.05	AG1	2	8.91667	0.482
1.6	0.05	ptsI	2	9.16667	0.349
1.6	0.05	clcB	2	9.16667	0.297
1.6	0.05	ycaM	2	9.16667	0.279
1.6	0.05	yadI	2	9.16667	0.512
1.6	0.05	AG1	2	9.16667	0.498
1.6	0.05	ptsI	2	9.41667	0.356
1.6	0.05	clcB	2	9.41667	0.304
1.6	0.05	ycaM	2	9.41667	0.281
1.6	0.05	yadI	2	9.41667	0.525
1.6	0.05	AG1	2	9.41667	0.515
1.6	0.05	ptsI	2	9.66667	0.364
1.6	0.05	clcB	2	9.66667	0.309
1.6	0.05	ycaM	2	9.66667	0.281
1.6	0.05	yadI	2	9.66667	0.537
1.6	0.05	AG1	2	9.66667	0.532
1.6	0.05	ptsI	2	9.91667	0.372
1.6	0.05	clcB	2	9.91667	0.312
1.6	0.05	ycaM	2	9.91667	0.281
1.6	0.05	yadI	2	9.91667	0.546
1.6	0.05	AG1	2	9.91667	0.541
1.6	0.05	ptsI	2	10.1667	0.379
1.6	0.05	clcB	2	10.1667	0.312
1.6	0.05	ycaM	2	10.1667	0.283
1.6	0.05	yadI	2	10.1667	0.548
1.6	0.05	AG1	2	10.1667	0.548
1.6	0.05	ptsI	2	10.4167	0.387
1.6	0.05	clcB	2	10.4167	0.317

1.6	0.05	ycaM	2	10.4167	0.281
1.6	0.05	yadI	2	10.4167	0.563
1.6	0.05	AG1	2	10.4167	0.563
1.6	0.05	ptsI	2	10.6667	0.392
1.6	0.05	clcB	2	10.6667	0.315
1.6	0.05	ycaM	2	10.6667	0.285
1.6	0.05	yadI	2	10.6667	0.563
1.6	0.05	AG1	2	10.6667	0.57
1.6	0.05	ptsI	2	10.9167	0.4
1.6	0.05	clcB	2	10.9167	0.319
1.6	0.05	ycaM	2	10.9167	0.285
1.6	0.05	yadI	2	10.9167	0.559
1.6	0.05	AG1	2	10.9167	0.57
1.6	0.05	ptsI	2	11.1667	0.404
1.6	0.05	clcB	2	11.1667	0.317
1.6	0.05	ycaM	2	11.1667	0.282
1.6	0.05	yadI	2	11.1667	0.564
1.6	0.05	AG1	2	11.1667	0.573
1.6	0.05	ptsI	2	11.4167	0.409
1.6	0.05	clcB	2	11.4167	0.316
1.6	0.05	ycaM	2	11.4167	0.283
1.6	0.05	yadI	2	11.4167	0.566
1.6	0.05	AG1	2	11.4167	0.574
1.6	0.05	ptsI	2	11.6667	0.413
1.6	0.05	clcB	2	11.6667	0.309
1.6	0.05	ycaM	2	11.6667	0.282
1.6	0.05	yadI	2	11.6667	0.571
1.6	0.05	AG1	2	11.6667	0.586
1.6	0.05	ptsI	2	11.9167	0.417
1.6	0.05	clcB	2	11.9167	0.309
1.6	0.05	ycaM	2	11.9167	0.28
1.6	0.05	yadI	2	11.9167	0.573
1.6	0.05	AG1	2	11.9167	0.592
1.6	0.05	ptsI	2	12.1667	0.421
1.6	0.05	clcB	2	12.1667	0.31
1.6	0.05	ycaM	2	12.1667	0.279
1.6	0.05	yadI	2	12.1667	0.579
1.6	0.05	AG1	2	12.1667	0.588
1.6	0.05	ptsI	2	12.4167	0.431
1.6	0.05	clcB	2	12.4167	0.309
1.6	0.05	ycaM	2	12.4167	0.278
1.6	0.05	yadI	2	12.4167	0.582
1.6	0.05	AG1	2	12.4167	0.6
1.6	0.05	ptsI	2	12.6667	0.436
1.6	0.05	clcB	2	12.6667	0.304
1.6	0.05	ycaM	2	12.6667	0.274
1.6	0.05	yadI	2	12.6667	0.576
1.6	0.05	AG1	2	12.6667	0.601
1.6	0.05	ptsI	2	12.9167	0.446
1.6	0.05	clcB	2	12.9167	0.3
1.6	0.05	ycaM	2	12.9167	0.274
1.6	0.05	yadI	2	12.9167	0.577
1.6	0.05	AG1	2	12.9167	0.595

1.6	0.05	ptsI	2	13.1667	0.45
1.6	0.05	clcB	2	13.1667	0.302
1.6	0.05	ycaM	2	13.1667	0.273
1.6	0.05	yadI	2	13.1667	0.584
1.6	0.05	AG1	2	13.1667	0.606
1.6	0.05	ptsI	2	13.4167	0.451
1.6	0.05	clcB	2	13.4167	0.3
1.6	0.05	ycaM	2	13.4167	0.274
1.6	0.05	yadI	2	13.4167	0.585
1.6	0.05	AG1	2	13.4167	0.629
1.6	0.05	ptsI	2	13.6667	0.453
1.6	0.05	clcB	2	13.6667	0.301
1.6	0.05	ycaM	2	13.6667	0.272
1.6	0.05	yadI	2	13.6667	0.587
1.6	0.05	AG1	2	13.6667	0.626
1.6	0.05	ptsI	2	13.9167	0.454
1.6	0.05	clcB	2	13.9167	0.299
1.6	0.05	ycaM	2	13.9167	0.27
1.6	0.05	yadI	2	13.9167	0.59
1.6	0.05	AG1	2	13.9167	0.662
1.6	0.05	ptsI	2	14.1667	0.459
1.6	0.05	clcB	2	14.1667	0.298
1.6	0.05	ycaM	2	14.1667	0.27
1.6	0.05	yadI	2	14.1667	0.592
1.6	0.05	AG1	2	14.1667	0.649
1.6	0.05	ptsI	2	14.4167	0.46
1.6	0.05	clcB	2	14.4167	0.295
1.6	0.05	ycaM	2	14.4167	0.272
1.6	0.05	yadI	2	14.4167	0.597
1.6	0.05	AG1	2	14.4167	0.661
1.6	0.05	ptsI	2	14.6667	0.459
1.6	0.05	clcB	2	14.6667	0.294
1.6	0.05	ycaM	2	14.6667	0.273
1.6	0.05	yadI	2	14.6667	0.598
1.6	0.05	AG1	2	14.6667	0.687
1.6	0.05	ptsI	2	14.9167	0.463
1.6	0.05	clcB	2	14.9167	0.292
1.6	0.05	ycaM	2	14.9167	0.271
1.6	0.05	yadI	2	14.9167	0.598
1.6	0.05	AG1	2	14.9167	0.688
1.6	0.05	ptsI	2	15.1667	0.469
1.6	0.05	clcB	2	15.1667	0.289
1.6	0.05	ycaM	2	15.1667	0.272
1.6	0.05	yadI	2	15.1667	0.607
1.6	0.05	AG1	2	15.1667	0.73
1.6	0.05	ptsI	2	15.4167	0.47
1.6	0.05	clcB	2	15.4167	0.29
1.6	0.05	ycaM	2	15.4167	0.271
1.6	0.05	yadI	2	15.4167	0.604
1.6	0.05	AG1	2	15.4167	0.702
1.6	0.05	ptsI	2	15.6667	0.476
1.6	0.05	clcB	2	15.6667	0.289
1.6	0.05	ycaM	2	15.6667	0.27

1.6	0.05	yadI	2	15.6667	0.606
1.6	0.05	AG1	2	15.6667	0.725
1.6	0.05	ptsI	2	15.9167	0.478
1.6	0.05	clcB	2	15.9167	0.288
1.6	0.05	ycaM	2	15.9167	0.272
1.6	0.05	yadI	2	15.9167	0.607
1.6	0.05	AG1	2	15.9167	0.73
1.6	0.05	ptsI	2	16.1667	0.476
1.6	0.05	clcB	2	16.1667	0.288
1.6	0.05	ycaM	2	16.1667	0.273
1.6	0.05	yadI	2	16.1667	0.613
1.6	0.05	AG1	2	16.1667	0.703
1.6	0.05	ptsI	2	16.4167	0.48
1.6	0.05	clcB	2	16.4167	0.287
1.6	0.05	ycaM	2	16.4167	0.269
1.6	0.05	yadI	2	16.4167	0.614
1.6	0.05	AG1	2	16.4167	0.713
1.6	0.05	ptsI	2	16.6667	0.48
1.6	0.05	clcB	2	16.6667	0.287
1.6	0.05	ycaM	2	16.6667	0.271
1.6	0.05	yadI	2	16.6667	0.616
1.6	0.05	AG1	2	16.6667	0.726
1.6	0.05	ptsI	2	16.9167	0.482
1.6	0.05	clcB	2	16.9167	0.286
1.6	0.05	ycaM	2	16.9167	0.272
1.6	0.05	yadI	2	16.9167	0.621
1.6	0.05	AG1	2	16.9167	0.744
1.6	0.05	ptsI	2	17.1667	0.487
1.6	0.05	clcB	2	17.1667	0.287
1.6	0.05	ycaM	2	17.1667	0.27
1.6	0.05	yadI	2	17.1667	0.622
1.6	0.05	AG1	2	17.1667	0.753
1.6	0.05	ptsI	2	17.4167	0.487
1.6	0.05	clcB	2	17.4167	0.288
1.6	0.05	ycaM	2	17.4167	0.271
1.6	0.05	yadI	2	17.4167	0.625
1.6	0.05	AG1	2	17.4167	0.739
1.6	0.05	ptsI	2	17.6667	0.489
1.6	0.05	clcB	2	17.6667	0.287
1.6	0.05	ycaM	2	17.6667	0.271
1.6	0.05	yadI	2	17.6667	0.625
1.6	0.05	AG1	2	17.6667	0.731
1.6	0.05	ptsI	2	17.9167	0.487
1.6	0.05	clcB	2	17.9167	0.287
1.6	0.05	ycaM	2	17.9167	0.272
1.6	0.05	yadI	2	17.9167	0.627
1.6	0.05	AG1	2	17.9167	0.728
1.6	0.05	ptsI	2	18.1667	0.493
1.6	0.05	clcB	2	18.1667	0.286
1.6	0.05	ycaM	2	18.1667	0.272
1.6	0.05	yadI	2	18.1667	0.63
1.6	0.05	AG1	2	18.1667	0.739
1.6	0.05	ptsI	2	18.4167	0.496

1.6	0.05	clcB	2	18.4167	0.286
1.6	0.05	ycaM	2	18.4167	0.274
1.6	0.05	yadI	2	18.4167	0.63
1.6	0.05	AG1	2	18.4167	0.756
1.6	0.05	ptsI	2	18.6667	0.496
1.6	0.05	clcB	2	18.6667	0.285
1.6	0.05	ycaM	2	18.6667	0.275
1.6	0.05	yadI	2	18.6667	0.633
1.6	0.05	AG1	2	18.6667	0.744
1.6	0.05	ptsI	2	18.9167	0.494
1.6	0.05	clcB	2	18.9167	0.286
1.6	0.05	ycaM	2	18.9167	0.277
1.6	0.05	yadI	2	18.9167	0.626
1.6	0.05	AG1	2	18.9167	0.753
1.6	0.05	ptsI	2	19.1667	0.498
1.6	0.05	clcB	2	19.1667	0.285
1.6	0.05	ycaM	2	19.1667	0.278
1.6	0.05	yadI	2	19.1667	0.637
1.6	0.05	AG1	2	19.1667	0.759
1.6	0.05	ptsI	2	19.4167	0.501
1.6	0.05	clcB	2	19.4167	0.286
1.6	0.05	ycaM	2	19.4167	0.279
1.6	0.05	yadI	2	19.4167	0.639
1.6	0.05	AG1	2	19.4167	0.751
1.6	0.05	ptsI	2	19.6667	0.503
1.6	0.05	clcB	2	19.6667	0.286
1.6	0.05	ycaM	2	19.6667	0.281
1.6	0.05	yadI	2	19.6667	0.641
1.6	0.05	AG1	2	19.6667	0.754
1.6	0.05	ptsI	2	19.9167	0.503
1.6	0.05	clcB	2	19.9167	0.286
1.6	0.05	ycaM	2	19.9167	0.282
1.6	0.05	yadI	2	19.9167	0.642
1.6	0.05	AG1	2	19.9167	0.749
1.6	0.05	ptsI	2	20.1667	0.504
1.6	0.05	clcB	2	20.1667	0.287
1.6	0.05	ycaM	2	20.1667	0.284
1.6	0.05	yadI	2	20.1667	0.645
1.6	0.05	AG1	2	20.1667	0.75
1.6	0.05	ptsI	2	20.4167	0.504
1.6	0.05	clcB	2	20.4167	0.287
1.6	0.05	ycaM	2	20.4167	0.286
1.6	0.05	yadI	2	20.4167	0.648
1.6	0.05	AG1	2	20.4167	0.754
1.6	0.05	ptsI	2	20.6667	0.507
1.6	0.05	clcB	2	20.6667	0.287
1.6	0.05	ycaM	2	20.6667	0.288
1.6	0.05	yadI	2	20.6667	0.649
1.6	0.05	AG1	2	20.6667	0.753
1.6	0.05	ptsI	2	20.9167	0.509
1.6	0.05	clcB	2	20.9167	0.289
1.6	0.05	ycaM	2	20.9167	0.29
1.6	0.05	yadI	2	20.9167	0.651

1.6	0.05	AG1	2	20.9167	0.759
1.6	0.05	ptsI	2	21.1667	0.508
1.6	0.05	clcB	2	21.1667	0.29
1.6	0.05	ycaM	2	21.1667	0.291
1.6	0.05	yadI	2	21.1667	0.65
1.6	0.05	AG1	2	21.1667	0.762
1.6	0.05	ptsI	2	21.4167	0.509
1.6	0.05	clcB	2	21.4167	0.29
1.6	0.05	ycaM	2	21.4167	0.293
1.6	0.05	yadI	2	21.4167	0.647
1.6	0.05	AG1	2	21.4167	0.782
1.6	0.05	ptsI	2	21.6667	0.508
1.6	0.05	clcB	2	21.6667	0.291
1.6	0.05	ycaM	2	21.6667	0.295
1.6	0.05	yadI	2	21.6667	0.656
1.6	0.05	AG1	2	21.6667	0.774
1.6	0.05	ptsI	2	21.9167	0.511
1.6	0.05	clcB	2	21.9167	0.292
1.6	0.05	ycaM	2	21.9167	0.297
1.6	0.05	yadI	2	21.9167	0.655
1.6	0.05	AG1	2	21.9167	0.78
1.6	0.05	ptsI	2	22.1667	0.51
1.6	0.05	clcB	2	22.1667	0.293
1.6	0.05	ycaM	2	22.1667	0.298
1.6	0.05	yadI	2	22.1667	0.655
1.6	0.05	AG1	2	22.1667	0.782
1.6	0.05	ptsI	2	22.4167	0.509
1.6	0.05	clcB	2	22.4167	0.296
1.6	0.05	ycaM	2	22.4167	0.3
1.6	0.05	yadI	2	22.4167	0.653
1.6	0.05	AG1	2	22.4167	0.785
1.6	0.05	ptsI	2	22.6667	0.507
1.6	0.05	clcB	2	22.6667	0.296
1.6	0.05	ycaM	2	22.6667	0.302
1.6	0.05	yadI	2	22.6667	0.656
1.6	0.05	AG1	2	22.6667	0.792
1.6	0.05	ptsI	2	22.9167	0.513
1.6	0.05	clcB	2	22.9167	0.295
1.6	0.05	ycaM	2	22.9167	0.302
1.6	0.05	yadI	2	22.9167	0.656
1.6	0.05	AG1	2	22.9167	0.798
1.6	0.05	ptsI	2	23.1667	0.51
1.6	0.05	clcB	2	23.1667	0.295
1.6	0.05	ycaM	2	23.1667	0.305
1.6	0.05	yadI	2	23.1667	0.659
1.6	0.05	AG1	2	23.1667	0.802
1.6	0.05	ptsI	2	23.4167	0.511
1.6	0.05	clcB	2	23.4167	0.297
1.6	0.05	ycaM	2	23.4167	0.307
1.6	0.05	yadI	2	23.4167	0.66
1.6	0.05	AG1	2	23.4167	0.811
1.6	0.05	ptsI	2	23.6667	0.511
1.6	0.05	clcB	2	23.6667	0.298

1.6	0.05	ycaM	2	23.6667	0.311
1.6	0.05	yadI	2	23.6667	0.659
1.6	0.05	AG1	2	23.6667	0.816
1.6	0.05	ptsI	2	23.9167	0.509
1.6	0.05	clcB	2	23.9167	0.3
1.6	0.05	ycaM	2	23.9167	0.313
1.6	0.05	yadI	2	23.9167	0.662
1.6	0.05	AG1	2	23.9167	0.821
1.6	0.05	ptsI	2	24.1667	0.511
1.6	0.05	clcB	2	24.1667	0.302
1.6	0.05	ycaM	2	24.1667	0.316
1.6	0.05	yadI	2	24.1667	0.662
1.6	0.05	AG1	2	24.1667	0.83
1.6	0.05	ptsI	2	24.4167	0.511
1.6	0.05	clcB	2	24.4167	0.302
1.6	0.05	ycaM	2	24.4167	0.32
1.6	0.05	yadI	2	24.4167	0.667
1.6	0.05	AG1	2	24.4167	0.839
1.6	0.05	ptsI	2	24.6667	0.511
1.6	0.05	clcB	2	24.6667	0.305
1.6	0.05	ycaM	2	24.6667	0.323
1.6	0.05	yadI	2	24.6667	0.665
1.6	0.05	AG1	2	24.6667	0.842
1.6	0.05	ptsI	2	24.9167	0.509
1.6	0.05	clcB	2	24.9167	0.307
1.6	0.05	ycaM	2	24.9167	0.327
1.6	0.05	yadI	2	24.9167	0.666
1.6	0.05	AG1	2	24.9167	0.853
1.6	0.05	ptsI	2	25.1667	0.512
1.6	0.05	clcB	2	25.1667	0.309
1.6	0.05	ycaM	2	25.1667	0.326
1.6	0.05	yadI	2	25.1667	0.667
1.6	0.05	AG1	2	25.1667	0.86
1.6	0.05	ptsI	2	25.4167	0.511
1.6	0.05	clcB	2	25.4167	0.311
1.6	0.05	ycaM	2	25.4167	0.331
1.6	0.05	yadI	2	25.4167	0.67
1.6	0.05	AG1	2	25.4167	0.871
1.6	0.05	ptsI	2	25.6667	0.512
1.6	0.05	clcB	2	25.6667	0.313
1.6	0.05	ycaM	2	25.6667	0.333
1.6	0.05	yadI	2	25.6667	0.67
1.6	0.05	AG1	2	25.6667	0.877
1.6	0.05	ptsI	2	25.9167	0.514
1.6	0.05	clcB	2	25.9167	0.315
1.6	0.05	ycaM	2	25.9167	0.337
1.6	0.05	yadI	2	25.9167	0.669
1.6	0.05	AG1	2	25.9167	0.885
1.6	0.05	ptsI	2	26.1667	0.516
1.6	0.05	clcB	2	26.1667	0.318
1.6	0.05	ycaM	2	26.1667	0.339
1.6	0.05	yadI	2	26.1667	0.672
1.6	0.05	AG1	2	26.1667	0.904

1.6	0.05	ptsI	2	26.4167	0.514
1.6	0.05	clcB	2	26.4167	0.318
1.6	0.05	ycaM	2	26.4167	0.34
1.6	0.05	yadI	2	26.4167	0.674
1.6	0.05	AG1	2	26.4167	0.906
1.6	0.05	ptsI	3	0	0.161
1.6	0.05	clcB	3	0	0.162
1.6	0.05	ycaM	3	0	0.157
1.6	0.05	yadI	3	0	0.158
1.6	0.05	AG1	3	0	0.16
1.6	0.05	ptsI	3	0.25	0.157
1.6	0.05	clcB	3	0.25	0.158
1.6	0.05	ycaM	3	0.25	0.154
1.6	0.05	yadI	3	0.25	0.156
1.6	0.05	AG1	3	0.25	0.157
1.6	0.05	ptsI	3	0.5	0.158
1.6	0.05	clcB	3	0.5	0.158
1.6	0.05	ycaM	3	0.5	0.151
1.6	0.05	yadI	3	0.5	0.154
1.6	0.05	AG1	3	0.5	0.155
1.6	0.05	ptsI	3	0.75	0.157
1.6	0.05	clcB	3	0.75	0.157
1.6	0.05	ycaM	3	0.75	0.152
1.6	0.05	yadI	3	0.75	0.153
1.6	0.05	AG1	3	0.75	0.156
1.6	0.05	ptsI	3	1	0.159
1.6	0.05	clcB	3	1	0.172
1.6	0.05	ycaM	3	1	0.158
1.6	0.05	yadI	3	1	0.155
1.6	0.05	AG1	3	1	0.156
1.6	0.05	ptsI	3	1.25	0.158
1.6	0.05	clcB	3	1.25	0.157
1.6	0.05	ycaM	3	1.25	0.151
1.6	0.05	yadI	3	1.25	0.155
1.6	0.05	AG1	3	1.25	0.156
1.6	0.05	ptsI	3	1.5	0.159
1.6	0.05	clcB	3	1.5	0.157
1.6	0.05	ycaM	3	1.5	0.152
1.6	0.05	yadI	3	1.5	0.154
1.6	0.05	AG1	3	1.5	0.156
1.6	0.05	ptsI	3	1.75	0.161
1.6	0.05	clcB	3	1.75	0.156
1.6	0.05	ycaM	3	1.75	0.152
1.6	0.05	yadI	3	1.75	0.155
1.6	0.05	AG1	3	1.75	0.157
1.6	0.05	ptsI	3	2	0.161
1.6	0.05	clcB	3	2	0.189
1.6	0.05	ycaM	3	2	0.154
1.6	0.05	yadI	3	2	0.157
1.6	0.05	AG1	3	2	0.157
1.6	0.05	ptsI	3	2.25	0.162
1.6	0.05	clcB	3	2.25	0.176
1.6	0.05	ycaM	3	2.25	0.156

1.6	0.05	yadI	3	2.25	0.157
1.6	0.05	AG1	3	2.25	0.159
1.6	0.05	ptsI	3	2.5	0.163
1.6	0.05	clcB	3	2.5	0.172
1.6	0.05	ycaM	3	2.5	0.157
1.6	0.05	yadI	3	2.5	0.159
1.6	0.05	AG1	3	2.5	0.16
1.6	0.05	ptsI	3	2.75	0.166
1.6	0.05	clcB	3	2.75	0.191
1.6	0.05	ycaM	3	2.75	0.16
1.6	0.05	yadI	3	2.75	0.162
1.6	0.05	AG1	3	2.75	0.164
1.6	0.05	ptsI	3	3	0.165
1.6	0.05	clcB	3	3	0.163
1.6	0.05	ycaM	3	3	0.16
1.6	0.05	yadI	3	3	0.163
1.6	0.05	AG1	3	3	0.163
1.6	0.05	ptsI	3	3.25	0.167
1.6	0.05	clcB	3	3.25	0.165
1.6	0.05	ycaM	3	3.25	0.162
1.6	0.05	yadI	3	3.25	0.165
1.6	0.05	AG1	3	3.25	0.163
1.6	0.05	ptsI	3	3.5	0.17
1.6	0.05	clcB	3	3.5	0.168
1.6	0.05	ycaM	3	3.5	0.166
1.6	0.05	yadI	3	3.5	0.17
1.6	0.05	AG1	3	3.5	0.165
1.6	0.05	ptsI	3	3.75	0.172
1.6	0.05	clcB	3	3.75	0.169
1.6	0.05	ycaM	3	3.75	0.17
1.6	0.05	yadI	3	3.75	0.173
1.6	0.05	AG1	3	3.75	0.168
1.6	0.05	ptsI	3	4	0.174
1.6	0.05	clcB	3	4	0.171
1.6	0.05	ycaM	3	4	0.172
1.6	0.05	yadI	3	4	0.178
1.6	0.05	AG1	3	4	0.171
1.6	0.05	ptsI	3	4.41667	0.174
1.6	0.05	clcB	3	4.41667	0.214
1.6	0.05	ycaM	3	4.41667	0.179
1.6	0.05	yadI	3	4.41667	0.193
1.6	0.05	AG1	3	4.41667	0.189
1.6	0.05	ptsI	3	4.66667	0.167
1.6	0.05	clcB	3	4.66667	0.187
1.6	0.05	ycaM	3	4.66667	0.171
1.6	0.05	yadI	3	4.66667	0.193
1.6	0.05	AG1	3	4.66667	0.19
1.6	0.05	ptsI	3	4.91667	0.168
1.6	0.05	clcB	3	4.91667	0.192
1.6	0.05	ycaM	3	4.91667	0.179
1.6	0.05	yadI	3	4.91667	0.2
1.6	0.05	AG1	3	4.91667	0.196
1.6	0.05	ptsI	3	5.16667	0.169

1.6	0.05	clcB	3	5.16667	0.197
1.6	0.05	ycaM	3	5.16667	0.184
1.6	0.05	yadI	3	5.16667	0.209
1.6	0.05	AG1	3	5.16667	0.202
1.6	0.05	ptsI	3	5.41667	0.173
1.6	0.05	clcB	3	5.41667	0.202
1.6	0.05	ycaM	3	5.41667	0.188
1.6	0.05	yadI	3	5.41667	0.219
1.6	0.05	AG1	3	5.41667	0.207
1.6	0.05	ptsI	3	5.66667	0.177
1.6	0.05	clcB	3	5.66667	0.208
1.6	0.05	ycaM	3	5.66667	0.196
1.6	0.05	yadI	3	5.66667	0.232
1.6	0.05	AG1	3	5.66667	0.214
1.6	0.05	ptsI	3	5.91667	0.181
1.6	0.05	clcB	3	5.91667	0.209
1.6	0.05	ycaM	3	5.91667	0.202
1.6	0.05	yadI	3	5.91667	0.238
1.6	0.05	AG1	3	5.91667	0.214
1.6	0.05	ptsI	3	6.16667	0.187
1.6	0.05	clcB	3	6.16667	0.216
1.6	0.05	ycaM	3	6.16667	0.212
1.6	0.05	yadI	3	6.16667	0.253
1.6	0.05	AG1	3	6.16667	0.222
1.6	0.05	ptsI	3	6.41667	0.193
1.6	0.05	clcB	3	6.41667	0.225
1.6	0.05	ycaM	3	6.41667	0.219
1.6	0.05	yadI	3	6.41667	0.264
1.6	0.05	AG1	3	6.41667	0.23
1.6	0.05	ptsI	3	6.66667	0.2
1.6	0.05	clcB	3	6.66667	0.233
1.6	0.05	ycaM	3	6.66667	0.228
1.6	0.05	yadI	3	6.66667	0.272
1.6	0.05	AG1	3	6.66667	0.241
1.6	0.05	ptsI	3	6.91667	0.207
1.6	0.05	clcB	3	6.91667	0.244
1.6	0.05	ycaM	3	6.91667	0.235
1.6	0.05	yadI	3	6.91667	0.282
1.6	0.05	AG1	3	6.91667	0.25
1.6	0.05	ptsI	3	7.16667	0.217
1.6	0.05	clcB	3	7.16667	0.255
1.6	0.05	ycaM	3	7.16667	0.239
1.6	0.05	yadI	3	7.16667	0.292
1.6	0.05	AG1	3	7.16667	0.258
1.6	0.05	ptsI	3	7.41667	0.225
1.6	0.05	clcB	3	7.41667	0.267
1.6	0.05	ycaM	3	7.41667	0.243
1.6	0.05	yadI	3	7.41667	0.301
1.6	0.05	AG1	3	7.41667	0.265
1.6	0.05	ptsI	3	7.66667	0.234
1.6	0.05	clcB	3	7.66667	0.275
1.6	0.05	ycaM	3	7.66667	0.244
1.6	0.05	yadI	3	7.66667	0.312

1.6	0.05	AG1	3	7.66667	0.272
1.6	0.05	ptsI	3	7.91667	0.242
1.6	0.05	clcB	3	7.91667	0.281
1.6	0.05	ycaM	3	7.91667	0.246
1.6	0.05	yadI	3	7.91667	0.321
1.6	0.05	AG1	3	7.91667	0.281
1.6	0.05	ptsI	3	8.16667	0.252
1.6	0.05	clcB	3	8.16667	0.288
1.6	0.05	ycaM	3	8.16667	0.248
1.6	0.05	yadI	3	8.16667	0.33
1.6	0.05	AG1	3	8.16667	0.286
1.6	0.05	ptsI	3	8.41667	0.259
1.6	0.05	clcB	3	8.41667	0.294
1.6	0.05	ycaM	3	8.41667	0.25
1.6	0.05	yadI	3	8.41667	0.339
1.6	0.05	AG1	3	8.41667	0.295
1.6	0.05	ptsI	3	9.05	0.262
1.6	0.05	clcB	3	9.05	0.268
1.6	0.05	ycaM	3	9.05	0.241
1.6	0.05	yadI	3	9.05	0.376
1.6	0.05	AG1	3	9.05	0.314
1.6	0.05	ptsI	3	9.3	0.27
1.6	0.05	clcB	3	9.3	0.271
1.6	0.05	ycaM	3	9.3	0.236
1.6	0.05	yadI	3	9.3	0.384
1.6	0.05	AG1	3	9.3	0.324
1.6	0.05	ptsI	3	9.55	0.281
1.6	0.05	clcB	3	9.55	0.275
1.6	0.05	ycaM	3	9.55	0.236
1.6	0.05	yadI	3	9.55	0.399
1.6	0.05	AG1	3	9.55	0.342
1.6	0.05	ptsI	3	9.8	0.292
1.6	0.05	clcB	3	9.8	0.279
1.6	0.05	ycaM	3	9.8	0.233
1.6	0.05	yadI	3	9.8	0.411
1.6	0.05	AG1	3	9.8	0.35
1.6	0.05	ptsI	3	10.05	0.299
1.6	0.05	clcB	3	10.05	0.282
1.6	0.05	ycaM	3	10.05	0.233
1.6	0.05	yadI	3	10.05	0.42
1.6	0.05	AG1	3	10.05	0.359
1.6	0.05	ptsI	3	10.3	0.307
1.6	0.05	clcB	3	10.3	0.286
1.6	0.05	ycaM	3	10.3	0.233
1.6	0.05	yadI	3	10.3	0.425
1.6	0.05	AG1	3	10.3	0.366
1.6	0.05	ptsI	3	10.55	0.317
1.6	0.05	clcB	3	10.55	0.283
1.6	0.05	ycaM	3	10.55	0.235
1.6	0.05	yadI	3	10.55	0.43
1.6	0.05	AG1	3	10.55	0.373
1.6	0.05	ptsI	3	10.8	0.323
1.6	0.05	clcB	3	10.8	0.282

1.6	0.05	ycaM	3	10.8	0.234
1.6	0.05	yadI	3	10.8	0.437
1.6	0.05	AG1	3	10.8	0.382
1.6	0.05	ptsI	3	11.05	0.331
1.6	0.05	clcB	3	11.05	0.288
1.6	0.05	ycaM	3	11.05	0.234
1.6	0.05	yadI	3	11.05	0.444
1.6	0.05	AG1	3	11.05	0.387
1.6	0.05	ptsI	3	11.3	0.325
1.6	0.05	clcB	3	11.3	0.281
1.6	0.05	ycaM	3	11.3	0.232
1.6	0.05	yadI	3	11.3	0.452
1.6	0.05	AG1	3	11.3	0.396
1.6	0.05	ptsI	3	11.55	0.33
1.6	0.05	clcB	3	11.55	0.282
1.6	0.05	ycaM	3	11.55	0.232
1.6	0.05	yadI	3	11.55	0.457
1.6	0.05	AG1	3	11.55	0.398
1.6	0.05	ptsI	3	11.8	0.335
1.6	0.05	clcB	3	11.8	0.286
1.6	0.05	ycaM	3	11.8	0.23
1.6	0.05	yadI	3	11.8	0.46
1.6	0.05	AG1	3	11.8	0.399
1.6	0.05	ptsI	3	12.05	0.336
1.6	0.05	clcB	3	12.05	0.278
1.6	0.05	ycaM	3	12.05	0.226
1.6	0.05	yadI	3	12.05	0.46
1.6	0.05	AG1	3	12.05	0.401
1.6	0.05	ptsI	3	12.3	0.343
1.6	0.05	clcB	3	12.3	0.279
1.6	0.05	ycaM	3	12.3	0.225
1.6	0.05	yadI	3	12.3	0.465
1.6	0.05	AG1	3	12.3	0.409
1.6	0.05	ptsI	3	12.55	0.351
1.6	0.05	clcB	3	12.55	0.28
1.6	0.05	ycaM	3	12.55	0.227
1.6	0.05	yadI	3	12.55	0.472
1.6	0.05	AG1	3	12.55	0.408
1.6	0.05	ptsI	3	12.8	0.356
1.6	0.05	clcB	3	12.8	0.281
1.6	0.05	ycaM	3	12.8	0.226
1.6	0.05	yadI	3	12.8	0.474
1.6	0.05	AG1	3	12.8	0.411
1.6	0.05	ptsI	3	13.05	0.36
1.6	0.05	clcB	3	13.05	0.278
1.6	0.05	ycaM	3	13.05	0.226
1.6	0.05	yadI	3	13.05	0.472
1.6	0.05	AG1	3	13.05	0.411
1.6	0.05	ptsI	3	13.3	0.368
1.6	0.05	clcB	3	13.3	0.276
1.6	0.05	ycaM	3	13.3	0.223
1.6	0.05	yadI	3	13.3	0.476
1.6	0.05	AG1	3	13.3	0.42

1.6	0.05	ptsI	3	13.55	0.367
1.6	0.05	clcB	3	13.55	0.276
1.6	0.05	ycaM	3	13.55	0.221
1.6	0.05	yadI	3	13.55	0.48
1.6	0.05	AG1	3	13.55	0.425
1.6	0.05	ptsI	3	13.8	0.378
1.6	0.05	clcB	3	13.8	0.274
1.6	0.05	ycaM	3	13.8	0.224
1.6	0.05	yadI	3	13.8	0.484
1.6	0.05	AG1	3	13.8	0.427
1.6	0.05	ptsI	3	14.05	0.382
1.6	0.05	clcB	3	14.05	0.276
1.6	0.05	ycaM	3	14.05	0.222
1.6	0.05	yadI	3	14.05	0.487
1.6	0.05	AG1	3	14.05	0.436
1.6	0.05	ptsI	3	14.3	0.388
1.6	0.05	clcB	3	14.3	0.275
1.6	0.05	ycaM	3	14.3	0.221
1.6	0.05	yadI	3	14.3	0.492
1.6	0.05	AG1	3	14.3	0.44
1.6	0.05	ptsI	3	14.55	0.39
1.6	0.05	clcB	3	14.55	0.274
1.6	0.05	ycaM	3	14.55	0.219
1.6	0.05	yadI	3	14.55	0.49
1.6	0.05	AG1	3	14.55	0.445
1.6	0.05	ptsI	3	14.8	0.394
1.6	0.05	clcB	3	14.8	0.272
1.6	0.05	ycaM	3	14.8	0.22
1.6	0.05	yadI	3	14.8	0.494
1.6	0.05	AG1	3	14.8	0.45
1.6	0.05	ptsI	3	15.05	0.402
1.6	0.05	clcB	3	15.05	0.272
1.6	0.05	ycaM	3	15.05	0.219
1.6	0.05	yadI	3	15.05	0.497
1.6	0.05	AG1	3	15.05	0.454
1.6	0.05	ptsI	3	15.3	0.405
1.6	0.05	clcB	3	15.3	0.272
1.6	0.05	ycaM	3	15.3	0.218
1.6	0.05	yadI	3	15.3	0.497
1.6	0.05	AG1	3	15.3	0.46
1.6	0.05	ptsI	3	15.55	0.414
1.6	0.05	clcB	3	15.55	0.272
1.6	0.05	ycaM	3	15.55	0.217
1.6	0.05	yadI	3	15.55	0.501
1.6	0.05	AG1	3	15.55	0.467
1.6	0.05	ptsI	3	15.8	0.418
1.6	0.05	clcB	3	15.8	0.271
1.6	0.05	ycaM	3	15.8	0.217
1.6	0.05	yadI	3	15.8	0.498
1.6	0.05	AG1	3	15.8	0.471
1.6	0.05	ptsI	3	16.05	0.427
1.6	0.05	clcB	3	16.05	0.271
1.6	0.05	ycaM	3	16.05	0.215

1.6	0.05	yadI	3	16.05	0.5
1.6	0.05	AG1	3	16.05	0.475
1.6	0.05	ptsI	3	16.3	0.43
1.6	0.05	clcB	3	16.3	0.269
1.6	0.05	ycaM	3	16.3	0.214
1.6	0.05	yadI	3	16.3	0.498
1.6	0.05	AG1	3	16.3	0.48
1.6	0.05	ptsI	3	16.55	0.432
1.6	0.05	clcB	3	16.55	0.27
1.6	0.05	ycaM	3	16.55	0.216
1.6	0.05	yadI	3	16.55	0.497
1.6	0.05	AG1	3	16.55	0.485
1.6	0.05	ptsI	3	16.8	0.438
1.6	0.05	clcB	3	16.8	0.269
1.6	0.05	ycaM	3	16.8	0.214
1.6	0.05	yadI	3	16.8	0.496
1.6	0.05	AG1	3	16.8	0.491
1.6	0.05	ptsI	3	17.05	0.438
1.6	0.05	clcB	3	17.05	0.268
1.6	0.05	ycaM	3	17.05	0.214
1.6	0.05	yadI	3	17.05	0.501
1.6	0.05	AG1	3	17.05	0.495
1.6	0.05	ptsI	3	17.3	0.444
1.6	0.05	clcB	3	17.3	0.265
1.6	0.05	ycaM	3	17.3	0.214
1.6	0.05	yadI	3	17.3	0.502
1.6	0.05	AG1	3	17.3	0.502
1.6	0.05	ptsI	3	17.55	0.446
1.6	0.05	clcB	3	17.55	0.266
1.6	0.05	ycaM	3	17.55	0.214
1.6	0.05	yadI	3	17.55	0.504
1.6	0.05	AG1	3	17.55	0.512
1.6	0.05	ptsI	3	17.8	0.45
1.6	0.05	clcB	3	17.8	0.266
1.6	0.05	ycaM	3	17.8	0.215
1.6	0.05	yadI	3	17.8	0.505
1.6	0.05	AG1	3	17.8	0.522
1.6	0.05	ptsI	3	18.05	0.45
1.6	0.05	clcB	3	18.05	0.266
1.6	0.05	ycaM	3	18.05	0.214
1.6	0.05	yadI	3	18.05	0.506
1.6	0.05	AG1	3	18.05	0.534
1.6	0.05	ptsI	3	18.3	0.454
1.6	0.05	clcB	3	18.3	0.267
1.6	0.05	ycaM	3	18.3	0.213
1.6	0.05	yadI	3	18.3	0.508
1.6	0.05	AG1	3	18.3	0.554
1.6	0.05	ptsI	3	18.55	0.458
1.6	0.05	clcB	3	18.55	0.267
1.6	0.05	ycaM	3	18.55	0.214
1.6	0.05	yadI	3	18.55	0.511
1.6	0.05	AG1	3	18.55	0.551
1.6	0.05	ptsI	3	18.8	0.461

1.6	0.05	clcB	3	18.8	0.266
1.6	0.05	ycaM	3	18.8	0.212
1.6	0.05	yadI	3	18.8	0.512
1.6	0.05	AG1	3	18.8	0.578
1.6	0.05	ptsI	3	19.05	0.462
1.6	0.05	clcB	3	19.05	0.266
1.6	0.05	ycaM	3	19.05	0.213
1.6	0.05	yadI	3	19.05	0.514
1.6	0.05	AG1	3	19.05	0.574
1.6	0.05	ptsI	3	19.3	0.459
1.6	0.05	clcB	3	19.3	0.265
1.6	0.05	ycaM	3	19.3	0.213
1.6	0.05	yadI	3	19.3	0.516
1.6	0.05	AG1	3	19.3	0.61
1.6	0.05	ptsI	3	19.55	0.467
1.6	0.05	clcB	3	19.55	0.265
1.6	0.05	ycaM	3	19.55	0.212
1.6	0.05	yadI	3	19.55	0.518
1.6	0.05	AG1	3	19.55	0.644
1.6	0.05	ptsI	3	19.8	0.469
1.6	0.05	clcB	3	19.8	0.265
1.6	0.05	ycaM	3	19.8	0.212
1.6	0.05	yadI	3	19.8	0.517
1.6	0.05	AG1	3	19.8	0.675
1.6	0.05	ptsI	3	20.05	0.472
1.6	0.05	clcB	3	20.05	0.265
1.6	0.05	ycaM	3	20.05	0.213
1.6	0.05	yadI	3	20.05	0.523
1.6	0.05	AG1	3	20.05	0.662
1.6	0.05	ptsI	3	20.3	0.474
1.6	0.05	clcB	3	20.3	0.264
1.6	0.05	ycaM	3	20.3	0.212
1.6	0.05	yadI	3	20.3	0.524
1.6	0.05	AG1	3	20.3	0.676
1.6	0.05	ptsI	3	20.55	0.477
1.6	0.05	clcB	3	20.55	0.264
1.6	0.05	ycaM	3	20.55	0.212
1.6	0.05	yadI	3	20.55	0.527
1.6	0.05	AG1	3	20.55	0.656
1.6	0.05	ptsI	3	20.8	0.48
1.6	0.05	clcB	3	20.8	0.264
1.6	0.05	ycaM	3	20.8	0.212
1.6	0.05	yadI	3	20.8	0.526
1.6	0.05	AG1	3	20.8	0.654
1.6	0.05	ptsI	3	21.05	0.485
1.6	0.05	clcB	3	21.05	0.263
1.6	0.05	ycaM	3	21.05	0.212
1.6	0.05	yadI	3	21.05	0.528
1.6	0.05	AG1	3	21.05	0.712
1.6	0.05	ptsI	3	21.3	0.488
1.6	0.05	clcB	3	21.3	0.263
1.6	0.05	ycaM	3	21.3	0.211
1.6	0.05	yadI	3	21.3	0.531

1.6	0.05	AG1	3	21.3	0.718
1.6	0.05	ptsI	3	21.55	0.49
1.6	0.05	clcB	3	21.55	0.264
1.6	0.05	ycaM	3	21.55	0.212
1.6	0.05	yadI	3	21.55	0.531
1.6	0.05	AG1	3	21.55	0.749
1.6	0.05	ptsI	3	21.8	0.493
1.6	0.05	clcB	3	21.8	0.263
1.6	0.05	ycaM	3	21.8	0.212
1.6	0.05	yadI	3	21.8	0.536
1.6	0.05	AG1	3	21.8	0.759
1.6	0.05	ptsI	3	22.05	0.495
1.6	0.05	clcB	3	22.05	0.264
1.6	0.05	ycaM	3	22.05	0.213
1.6	0.05	yadI	3	22.05	0.537
1.6	0.05	AG1	3	22.05	0.76
1.6	0.05	ptsI	3	22.3	0.5
1.6	0.05	clcB	3	22.3	0.264
1.6	0.05	ycaM	3	22.3	0.212
1.6	0.05	yadI	3	22.3	0.539
1.6	0.05	AG1	3	22.3	0.762
1.6	0.05	ptsI	3	22.55	0.502
1.6	0.05	clcB	3	22.55	0.265
1.6	0.05	ycaM	3	22.55	0.212
1.6	0.05	yadI	3	22.55	0.542
1.6	0.05	AG1	3	22.55	0.771
1.6	0.05	ptsI	3	22.8	0.502
1.6	0.05	clcB	3	22.8	0.265
1.6	0.05	ycaM	3	22.8	0.212
1.6	0.05	yadI	3	22.8	0.543
1.6	0.05	AG1	3	22.8	0.777
1.6	0.05	ptsI	3	23.05	0.502
1.6	0.05	clcB	3	23.05	0.264
1.6	0.05	ycaM	3	23.05	0.212
1.6	0.05	yadI	3	23.05	0.545
1.6	0.05	AG1	3	23.05	0.776
1.6	0.05	ptsI	3	23.3	0.502
1.6	0.05	clcB	3	23.3	0.265
1.6	0.05	ycaM	3	23.3	0.213
1.6	0.05	yadI	3	23.3	0.547
1.6	0.05	AG1	3	23.3	0.779
1.6	0.05	ptsI	3	23.55	0.503
1.6	0.05	clcB	3	23.55	0.264
1.6	0.05	ycaM	3	23.55	0.212
1.6	0.05	yadI	3	23.55	0.545
1.6	0.05	AG1	3	23.55	0.762
1.6	0.05	ptsI	3	23.8	0.505
1.6	0.05	clcB	3	23.8	0.266
1.6	0.05	ycaM	3	23.8	0.212
1.6	0.05	yadI	3	23.8	0.548
1.6	0.05	AG1	3	23.8	0.776
1.6	0.05	ptsI	3	24.05	0.512
1.6	0.05	clcB	3	24.05	0.267

1.6	0.05	ycaM	3	24.05	0.212
1.6	0.05	yadI	3	24.05	0.552
1.6	0.05	AG1	3	24.05	0.774
1.6	0.05	ptsI	3	24.3	0.513
1.6	0.05	clcB	3	24.3	0.266
1.6	0.05	ycaM	3	24.3	0.211
1.6	0.05	yadI	3	24.3	0.554
1.6	0.05	AG1	3	24.3	0.795
1.6	0.05	ptsI	4	0	0.211
1.6	0.05	clcB	4	0	0.191
1.6	0.05	ycaM	4	0	0.214
1.6	0.05	yadI	4	0	0.2
1.6	0.05	AG1	4	0	0.211
1.6	0.05	ptsI	4	0.25	0.205
1.6	0.05	clcB	4	0.25	0.188
1.6	0.05	ycaM	4	0.25	0.205
1.6	0.05	yadI	4	0.25	0.205
1.6	0.05	AG1	4	0.25	0.205
1.6	0.05	ptsI	4	0.5	0.207
1.6	0.05	clcB	4	0.5	0.19
1.6	0.05	ycaM	4	0.5	0.206
1.6	0.05	yadI	4	0.5	0.206
1.6	0.05	AG1	4	0.5	0.205
1.6	0.05	ptsI	4	0.75	0.208
1.6	0.05	clcB	4	0.75	0.191
1.6	0.05	ycaM	4	0.75	0.207
1.6	0.05	yadI	4	0.75	0.209
1.6	0.05	AG1	4	0.75	0.21
1.6	0.05	ptsI	4	1	0.214
1.6	0.05	clcB	4	1	0.195
1.6	0.05	ycaM	4	1	0.211
1.6	0.05	yadI	4	1	0.21
1.6	0.05	AG1	4	1	0.216
1.6	0.05	ptsI	4	1.25	0.221
1.6	0.05	clcB	4	1.25	0.201
1.6	0.05	ycaM	4	1.25	0.217
1.6	0.05	yadI	4	1.25	0.214
1.6	0.05	AG1	4	1.25	0.225
1.6	0.05	ptsI	4	1.5	0.222
1.6	0.05	clcB	4	1.5	0.197
1.6	0.05	ycaM	4	1.5	0.216
1.6	0.05	yadI	4	1.5	0.213
1.6	0.05	AG1	4	1.5	0.22
1.6	0.05	ptsI	4	1.75	0.229
1.6	0.05	clcB	4	1.75	0.205
1.6	0.05	ycaM	4	1.75	0.225
1.6	0.05	yadI	4	1.75	0.217
1.6	0.05	AG1	4	1.75	0.226
1.6	0.05	ptsI	4	2	0.24
1.6	0.05	clcB	4	2	0.212
1.6	0.05	ycaM	4	2	0.232
1.6	0.05	yadI	4	2	0.226
1.6	0.05	AG1	4	2	0.236

1.6	0.05	ptsI	4	2.25	0.25
1.6	0.05	clcB	4	2.25	0.222
1.6	0.05	ycaM	4	2.25	0.244
1.6	0.05	yadI	4	2.25	0.234
1.6	0.05	AG1	4	2.25	0.247
1.6	0.05	ptsI	4	2.5	0.252
1.6	0.05	clcB	4	2.5	0.221
1.6	0.05	ycaM	4	2.5	0.245
1.6	0.05	yadI	4	2.5	0.236
1.6	0.05	AG1	4	2.5	0.242
1.6	0.05	ptsI	4	2.75	0.257
1.6	0.05	clcB	4	2.75	0.228
1.6	0.05	ycaM	4	2.75	0.252
1.6	0.05	yadI	4	2.75	0.247
1.6	0.05	AG1	4	2.75	0.249
1.6	0.05	ptsI	4	3	0.27
1.6	0.05	clcB	4	3	0.243
1.6	0.05	ycaM	4	3	0.264
1.6	0.05	yadI	4	3	0.255
1.6	0.05	AG1	4	3	0.26
1.6	0.05	ptsI	4	3.25	0.283
1.6	0.05	clcB	4	3.25	0.25
1.6	0.05	ycaM	4	3.25	0.272
1.6	0.05	yadI	4	3.25	0.263
1.6	0.05	AG1	4	3.25	0.268
1.6	0.05	ptsI	4	3.5	0.299
1.6	0.05	clcB	4	3.5	0.268
1.6	0.05	ycaM	4	3.5	0.285
1.6	0.05	yadI	4	3.5	0.273
1.6	0.05	AG1	4	3.5	0.283
1.6	0.05	ptsI	4	3.75	0.311
1.6	0.05	clcB	4	3.75	0.272
1.6	0.05	ycaM	4	3.75	0.296
1.6	0.05	yadI	4	3.75	0.284
1.6	0.05	AG1	4	3.75	0.289
1.6	0.05	ptsI	4	4	0.329
1.6	0.05	clcB	4	4	0.301
1.6	0.05	ycaM	4	4	0.315
1.6	0.05	yadI	4	4	0.293
1.6	0.05	AG1	4	4	0.305
1.6	0.05	ptsI	4	4.35	0.346
1.6	0.05	clcB	4	4.35	0.355
1.6	0.05	ycaM	4	4.35	0.346
1.6	0.05	yadI	4	4.35	0.34
1.6	0.05	AG1	4	4.35	0.28
1.6	0.05	ptsI	4	4.6	0.339
1.6	0.05	clcB	4	4.6	0.352
1.6	0.05	ycaM	4	4.6	0.34
1.6	0.05	yadI	4	4.6	0.349
1.6	0.05	AG1	4	4.6	0.292
1.6	0.05	ptsI	4	4.85	0.35
1.6	0.05	clcB	4	4.85	0.358
1.6	0.05	ycaM	4	4.85	0.336

1.6	0.05	yadI	4	4.85	0.358
1.6	0.05	AG1	4	4.85	0.304
1.6	0.05	ptsI	4	5.1	0.36
1.6	0.05	clcB	4	5.1	0.358
1.6	0.05	ycaM	4	5.1	0.337
1.6	0.05	yadI	4	5.1	0.37
1.6	0.05	AG1	4	5.1	0.313
1.6	0.05	ptsI	4	5.35	0.373
1.6	0.05	clcB	4	5.35	0.364
1.6	0.05	ycaM	4	5.35	0.34
1.6	0.05	yadI	4	5.35	0.389
1.6	0.05	AG1	4	5.35	0.324
1.6	0.05	ptsI	4	5.6	0.38
1.6	0.05	clcB	4	5.6	0.355
1.6	0.05	ycaM	4	5.6	0.335
1.6	0.05	yadI	4	5.6	0.393
1.6	0.05	AG1	4	5.6	0.329
1.6	0.05	ptsI	4	5.85	0.387
1.6	0.05	clcB	4	5.85	0.336
1.6	0.05	ycaM	4	5.85	0.331
1.6	0.05	yadI	4	5.85	0.391
1.6	0.05	AG1	4	5.85	0.333
1.6	0.05	ptsI	4	6.1	0.401
1.6	0.05	clcB	4	6.1	0.341
1.6	0.05	ycaM	4	6.1	0.337
1.6	0.05	yadI	4	6.1	0.407
1.6	0.05	AG1	4	6.1	0.342
1.6	0.05	ptsI	4	6.35	0.41
1.6	0.05	clcB	4	6.35	0.35
1.6	0.05	ycaM	4	6.35	0.338
1.6	0.05	yadI	4	6.35	0.418
1.6	0.05	AG1	4	6.35	0.353
1.6	0.05	ptsI	4	6.6	0.419
1.6	0.05	clcB	4	6.6	0.355
1.6	0.05	ycaM	4	6.6	0.336
1.6	0.05	yadI	4	6.6	0.425
1.6	0.05	AG1	4	6.6	0.361
1.6	0.05	ptsI	4	6.85	0.428
1.6	0.05	clcB	4	6.85	0.361
1.6	0.05	ycaM	4	6.85	0.34
1.6	0.05	yadI	4	6.85	0.434
1.6	0.05	AG1	4	6.85	0.371
1.6	0.05	ptsI	4	7.1	0.436
1.6	0.05	clcB	4	7.1	0.365
1.6	0.05	ycaM	4	7.1	0.341
1.6	0.05	yadI	4	7.1	0.439
1.6	0.05	AG1	4	7.1	0.38
1.6	0.05	ptsI	4	7.35	0.447
1.6	0.05	clcB	4	7.35	0.37
1.6	0.05	ycaM	4	7.35	0.344
1.6	0.05	yadI	4	7.35	0.447
1.6	0.05	AG1	4	7.35	0.386
1.6	0.05	ptsI	4	7.6	0.456

1.6	0.05	clcB	4	7.6	0.375
1.6	0.05	ycaM	4	7.6	0.352
1.6	0.05	yadI	4	7.6	0.454
1.6	0.05	AG1	4	7.6	0.395
1.6	0.05	ptsI	4	7.85	0.456
1.6	0.05	clcB	4	7.85	0.361
1.6	0.05	ycaM	4	7.85	0.336
1.6	0.05	yadI	4	7.85	0.456
1.6	0.05	AG1	4	7.85	0.394
1.6	0.05	ptsI	4	8.1	0.469
1.6	0.05	clcB	4	8.1	0.366
1.6	0.05	ycaM	4	8.1	0.34
1.6	0.05	yadI	4	8.1	0.463
1.6	0.05	AG1	4	8.1	0.403
1.6	0.05	ptsI	4	8.35	0.476
1.6	0.05	clcB	4	8.35	0.371
1.6	0.05	ycaM	4	8.35	0.343
1.6	0.05	yadI	4	8.35	0.47
1.6	0.05	AG1	4	8.35	0.412
1.6	0.05	ptsI	4	8.83333	0.428
1.6	0.05	clcB	4	8.83333	0.295
1.6	0.05	ycaM	4	8.83333	0.28
1.6	0.05	yadI	4	8.83333	0.46
1.6	0.05	AG1	4	8.83333	0.45
1.6	0.05	ptsI	4	9.08333	0.427
1.6	0.05	clcB	4	9.08333	0.292
1.6	0.05	ycaM	4	9.08333	0.277
1.6	0.05	yadI	4	9.08333	0.459
1.6	0.05	AG1	4	9.08333	0.456
1.6	0.05	ptsI	4	9.33333	0.436
1.6	0.05	clcB	4	9.33333	0.29
1.6	0.05	ycaM	4	9.33333	0.278
1.6	0.05	yadI	4	9.33333	0.465
1.6	0.05	AG1	4	9.33333	0.467
1.6	0.05	ptsI	4	9.58333	0.444
1.6	0.05	clcB	4	9.58333	0.292
1.6	0.05	ycaM	4	9.58333	0.281
1.6	0.05	yadI	4	9.58333	0.475
1.6	0.05	AG1	4	9.58333	0.478
1.6	0.05	ptsI	4	9.83333	0.449
1.6	0.05	clcB	4	9.83333	0.294
1.6	0.05	ycaM	4	9.83333	0.28
1.6	0.05	yadI	4	9.83333	0.485
1.6	0.05	AG1	4	9.83333	0.488
1.6	0.05	ptsI	4	10.0833	0.454
1.6	0.05	clcB	4	10.0833	0.293
1.6	0.05	ycaM	4	10.0833	0.283
1.6	0.05	yadI	4	10.0833	0.491
1.6	0.05	AG1	4	10.0833	0.498
1.6	0.05	ptsI	4	10.3333	0.458
1.6	0.05	clcB	4	10.3333	0.294
1.6	0.05	ycaM	4	10.3333	0.285
1.6	0.05	yadI	4	10.3333	0.496

1.6	0.05	AG1	4	10.3333	0.503
1.6	0.05	ptsI	4	10.5833	0.464
1.6	0.05	clcB	4	10.5833	0.295
1.6	0.05	ycaM	4	10.5833	0.286
1.6	0.05	yadI	4	10.5833	0.5
1.6	0.05	AG1	4	10.5833	0.509
1.6	0.05	ptsI	4	10.8333	0.471
1.6	0.05	clcB	4	10.8333	0.294
1.6	0.05	ycaM	4	10.8333	0.285
1.6	0.05	yadI	4	10.8333	0.505
1.6	0.05	AG1	4	10.8333	0.512
1.6	0.05	ptsI	4	11.0833	0.475
1.6	0.05	clcB	4	11.0833	0.295
1.6	0.05	ycaM	4	11.0833	0.286
1.6	0.05	yadI	4	11.0833	0.508
1.6	0.05	AG1	4	11.0833	0.514
1.6	0.05	ptsI	4	11.3333	0.479
1.6	0.05	clcB	4	11.3333	0.294
1.6	0.05	ycaM	4	11.3333	0.285
1.6	0.05	yadI	4	11.3333	0.512
1.6	0.05	AG1	4	11.3333	0.52
1.6	0.05	ptsI	4	11.5833	0.481
1.6	0.05	clcB	4	11.5833	0.293
1.6	0.05	ycaM	4	11.5833	0.285
1.6	0.05	yadI	4	11.5833	0.514
1.6	0.05	AG1	4	11.5833	0.525
1.6	0.05	ptsI	4	11.8333	0.482
1.6	0.05	clcB	4	11.8333	0.292
1.6	0.05	ycaM	4	11.8333	0.283
1.6	0.05	yadI	4	11.8333	0.516
1.6	0.05	AG1	4	11.8333	0.522
1.6	0.05	ptsI	4	12.0833	0.483
1.6	0.05	clcB	4	12.0833	0.292
1.6	0.05	ycaM	4	12.0833	0.281
1.6	0.05	yadI	4	12.0833	0.519
1.6	0.05	AG1	4	12.0833	0.532
1.6	0.05	ptsI	4	12.3333	0.482
1.6	0.05	clcB	4	12.3333	0.291
1.6	0.05	ycaM	4	12.3333	0.281
1.6	0.05	yadI	4	12.3333	0.521
1.6	0.05	AG1	4	12.3333	0.532
1.6	0.05	ptsI	4	12.5833	0.483
1.6	0.05	clcB	4	12.5833	0.291
1.6	0.05	ycaM	4	12.5833	0.279
1.6	0.05	yadI	4	12.5833	0.527
1.6	0.05	AG1	4	12.5833	0.532
1.6	0.05	ptsI	4	12.8333	0.486
1.6	0.05	clcB	4	12.8333	0.29
1.6	0.05	ycaM	4	12.8333	0.282
1.6	0.05	yadI	4	12.8333	0.528
1.6	0.05	AG1	4	12.8333	0.541
1.6	0.05	ptsI	4	13.0833	0.488
1.6	0.05	clcB	4	13.0833	0.29

1.6	0.05	ycaM	4	13.0833	0.277
1.6	0.05	yadI	4	13.0833	0.532
1.6	0.05	AG1	4	13.0833	0.544
1.6	0.05	ptsI	4	13.3333	0.491
1.6	0.05	clcB	4	13.3333	0.288
1.6	0.05	ycaM	4	13.3333	0.276
1.6	0.05	yadI	4	13.3333	0.534
1.6	0.05	AG1	4	13.3333	0.541
1.6	0.05	ptsI	4	13.5833	0.494
1.6	0.05	clcB	4	13.5833	0.286
1.6	0.05	ycaM	4	13.5833	0.276
1.6	0.05	yadI	4	13.5833	0.532
1.6	0.05	AG1	4	13.5833	0.545
1.6	0.05	ptsI	4	13.8333	0.494
1.6	0.05	clcB	4	13.8333	0.288
1.6	0.05	ycaM	4	13.8333	0.276
1.6	0.05	yadI	4	13.8333	0.537
1.6	0.05	AG1	4	13.8333	0.55
1.6	0.05	ptsI	4	14.0833	0.495
1.6	0.05	clcB	4	14.0833	0.286
1.6	0.05	ycaM	4	14.0833	0.274
1.6	0.05	yadI	4	14.0833	0.538
1.6	0.05	AG1	4	14.0833	0.554
1.6	0.05	ptsI	4	14.3333	0.5
1.6	0.05	clcB	4	14.3333	0.285
1.6	0.05	ycaM	4	14.3333	0.274
1.6	0.05	yadI	4	14.3333	0.536
1.6	0.05	AG1	4	14.3333	0.552
1.6	0.05	ptsI	4	14.5833	0.504
1.6	0.05	clcB	4	14.5833	0.284
1.6	0.05	ycaM	4	14.5833	0.273
1.6	0.05	yadI	4	14.5833	0.541
1.6	0.05	AG1	4	14.5833	0.557
1.6	0.05	ptsI	4	14.8333	0.507
1.6	0.05	clcB	4	14.8333	0.286
1.6	0.05	ycaM	4	14.8333	0.274
1.6	0.05	yadI	4	14.8333	0.541
1.6	0.05	AG1	4	14.8333	0.561
1.6	0.05	ptsI	4	15.0833	0.507
1.6	0.05	clcB	4	15.0833	0.285
1.6	0.05	ycaM	4	15.0833	0.274
1.6	0.05	yadI	4	15.0833	0.544
1.6	0.05	AG1	4	15.0833	0.564
1.6	0.05	ptsI	4	15.3333	0.512
1.6	0.05	clcB	4	15.3333	0.284
1.6	0.05	ycaM	4	15.3333	0.272
1.6	0.05	yadI	4	15.3333	0.542
1.6	0.05	AG1	4	15.3333	0.561
1.6	0.05	ptsI	4	15.5833	0.512
1.6	0.05	clcB	4	15.5833	0.285
1.6	0.05	ycaM	4	15.5833	0.273
1.6	0.05	yadI	4	15.5833	0.542
1.6	0.05	AG1	4	15.5833	0.568

1.6	0.05	ptsI	4	15.8333	0.512
1.6	0.05	clcB	4	15.8333	0.283
1.6	0.05	ycaM	4	15.8333	0.272
1.6	0.05	yadI	4	15.8333	0.545
1.6	0.05	AG1	4	15.8333	0.567
1.6	0.05	ptsI	4	16.0833	0.514
1.6	0.05	clcB	4	16.0833	0.282
1.6	0.05	ycaM	4	16.0833	0.272
1.6	0.05	yadI	4	16.0833	0.544
1.6	0.05	AG1	4	16.0833	0.57
1.6	0.05	ptsI	4	16.3333	0.518
1.6	0.05	clcB	4	16.3333	0.281
1.6	0.05	ycaM	4	16.3333	0.272
1.6	0.05	yadI	4	16.3333	0.543
1.6	0.05	AG1	4	16.3333	0.568
1.6	0.05	ptsI	4	16.5833	0.515
1.6	0.05	clcB	4	16.5833	0.281
1.6	0.05	ycaM	4	16.5833	0.271
1.6	0.05	yadI	4	16.5833	0.542
1.6	0.05	AG1	4	16.5833	0.57
1.6	0.05	ptsI	4	16.8333	0.515
1.6	0.05	clcB	4	16.8333	0.281
1.6	0.05	ycaM	4	16.8333	0.271
1.6	0.05	yadI	4	16.8333	0.543
1.6	0.05	AG1	4	16.8333	0.573
1.6	0.05	ptsI	4	17.0833	0.52
1.6	0.05	clcB	4	17.0833	0.281
1.6	0.05	ycaM	4	17.0833	0.271
1.6	0.05	yadI	4	17.0833	0.545
1.6	0.05	AG1	4	17.0833	0.572
1.6	0.05	ptsI	4	17.3333	0.523
1.6	0.05	clcB	4	17.3333	0.28
1.6	0.05	ycaM	4	17.3333	0.27
1.6	0.05	yadI	4	17.3333	0.543
1.6	0.05	AG1	4	17.3333	0.573
1.6	0.05	ptsI	4	17.5833	0.517
1.6	0.05	clcB	4	17.5833	0.28
1.6	0.05	ycaM	4	17.5833	0.271
1.6	0.05	yadI	4	17.5833	0.544
1.6	0.05	AG1	4	17.5833	0.573
1.6	0.05	ptsI	4	17.8333	0.52
1.6	0.05	clcB	4	17.8333	0.28
1.6	0.05	ycaM	4	17.8333	0.271
1.6	0.05	yadI	4	17.8333	0.544
1.6	0.05	AG1	4	17.8333	0.573
1.6	0.05	ptsI	4	18.0833	0.523
1.6	0.05	clcB	4	18.0833	0.28
1.6	0.05	ycaM	4	18.0833	0.27
1.6	0.05	yadI	4	18.0833	0.544
1.6	0.05	AG1	4	18.0833	0.576
1.6	0.05	ptsI	4	18.3333	0.522
1.6	0.05	clcB	4	18.3333	0.28
1.6	0.05	ycaM	4	18.3333	0.27

1.6	0.05	yadI	4	18.3333	0.544
1.6	0.05	AG1	4	18.3333	0.578
1.6	0.05	ptsI	4	18.5833	0.523
1.6	0.05	clcB	4	18.5833	0.28
1.6	0.05	ycaM	4	18.5833	0.269
1.6	0.05	yadI	4	18.5833	0.542
1.6	0.05	AG1	4	18.5833	0.577
1.6	0.05	ptsI	4	18.8333	0.525
1.6	0.05	clcB	4	18.8333	0.28
1.6	0.05	ycaM	4	18.8333	0.268
1.6	0.05	yadI	4	18.8333	0.54
1.6	0.05	AG1	4	18.8333	0.574
1.6	0.05	ptsI	4	19.0833	0.526
1.6	0.05	clcB	4	19.0833	0.279
1.6	0.05	ycaM	4	19.0833	0.267
1.6	0.05	yadI	4	19.0833	0.54
1.6	0.05	AG1	4	19.0833	0.582
1.6	0.05	ptsI	4	19.3333	0.529
1.6	0.05	clcB	4	19.3333	0.279
1.6	0.05	ycaM	4	19.3333	0.266
1.6	0.05	yadI	4	19.3333	0.541
1.6	0.05	AG1	4	19.3333	0.581
1.6	0.05	ptsI	4	19.5833	0.529
1.6	0.05	clcB	4	19.5833	0.278
1.6	0.05	ycaM	4	19.5833	0.267
1.6	0.05	yadI	4	19.5833	0.541
1.6	0.05	AG1	4	19.5833	0.587
1.6	0.05	ptsI	4	19.8333	0.529
1.6	0.05	clcB	4	19.8333	0.278
1.6	0.05	ycaM	4	19.8333	0.266
1.6	0.05	yadI	4	19.8333	0.541
1.6	0.05	AG1	4	19.8333	0.584
1.6	0.05	ptsI	4	20.0833	0.531
1.6	0.05	clcB	4	20.0833	0.279
1.6	0.05	ycaM	4	20.0833	0.267
1.6	0.05	yadI	4	20.0833	0.544
1.6	0.05	AG1	4	20.0833	0.588
1.6	0.05	ptsI	4	20.3333	0.527
1.6	0.05	clcB	4	20.3333	0.278
1.6	0.05	ycaM	4	20.3333	0.265
1.6	0.05	yadI	4	20.3333	0.539
1.6	0.05	AG1	4	20.3333	0.588
1.6	0.05	ptsI	4	20.5833	0.535
1.6	0.05	clcB	4	20.5833	0.277
1.6	0.05	ycaM	4	20.5833	0.267
1.6	0.05	yadI	4	20.5833	0.545
1.6	0.05	AG1	4	20.5833	0.591
1.6	0.05	ptsI	4	20.8333	0.536
1.6	0.05	clcB	4	20.8333	0.277
1.6	0.05	ycaM	4	20.8333	0.266
1.6	0.05	yadI	4	20.8333	0.547
1.6	0.05	AG1	4	20.8333	0.592
1.6	0.05	ptsI	4	21.0833	0.534

1.6	0.05	clcB	4	21.0833	0.277
1.6	0.05	ycaM	4	21.0833	0.266
1.6	0.05	yadI	4	21.0833	0.543
1.6	0.05	AG1	4	21.0833	0.593
1.6	0.05	ptsI	4	21.3333	0.537
1.6	0.05	clcB	4	21.3333	0.276
1.6	0.05	ycaM	4	21.3333	0.265
1.6	0.05	yadI	4	21.3333	0.546
1.6	0.05	AG1	4	21.3333	0.592
1.6	0.05	ptsI	4	21.5833	0.537
1.6	0.05	clcB	4	21.5833	0.276
1.6	0.05	ycaM	4	21.5833	0.266
1.6	0.05	yadI	4	21.5833	0.546
1.6	0.05	AG1	4	21.5833	0.594
1.6	0.05	ptsI	4	21.8333	0.541
1.6	0.05	clcB	4	21.8333	0.277
1.6	0.05	ycaM	4	21.8333	0.265
1.6	0.05	yadI	4	21.8333	0.547
1.6	0.05	AG1	4	21.8333	0.597
1.6	0.05	ptsI	4	22.0833	0.539
1.6	0.05	clcB	4	22.0833	0.276
1.6	0.05	ycaM	4	22.0833	0.266
1.6	0.05	yadI	4	22.0833	0.546
1.6	0.05	AG1	4	22.0833	0.596
1.6	0.05	ptsI	4	22.3333	0.543
1.6	0.05	clcB	4	22.3333	0.276
1.6	0.05	ycaM	4	22.3333	0.265
1.6	0.05	yadI	4	22.3333	0.547
1.6	0.05	AG1	4	22.3333	0.601
1.6	0.05	ptsI	4	22.5833	0.542
1.6	0.05	clcB	4	22.5833	0.276
1.6	0.05	ycaM	4	22.5833	0.265
1.6	0.05	yadI	4	22.5833	0.549
1.6	0.05	AG1	4	22.5833	0.603
1.6	0.05	ptsI	4	22.8333	0.542
1.6	0.05	clcB	4	22.8333	0.276
1.6	0.05	ycaM	4	22.8333	0.264
1.6	0.05	yadI	4	22.8333	0.551
1.6	0.05	AG1	4	22.8333	0.605
1.6	0.05	ptsI	4	23.0833	0.546
1.6	0.05	clcB	4	23.0833	0.275
1.6	0.05	ycaM	4	23.0833	0.264
1.6	0.05	yadI	4	23.0833	0.553
1.6	0.05	AG1	4	23.0833	0.606
1.6	0.05	ptsI	4	23.3333	0.544
1.6	0.05	clcB	4	23.3333	0.277
1.6	0.05	ycaM	4	23.3333	0.264
1.6	0.05	yadI	4	23.3333	0.551
1.6	0.05	AG1	4	23.3333	0.606
1.6	0.05	ptsI	4	23.5833	0.542
1.6	0.05	clcB	4	23.5833	0.276
1.6	0.05	ycaM	4	23.5833	0.264
1.6	0.05	yadI	4	23.5833	0.552

1.6	0.05	AG1	4	23.5833	0.607
1.6	0.05	ptsI	4	23.8333	0.543
1.6	0.05	clcB	4	23.8333	0.276
1.6	0.05	ycaM	4	23.8333	0.263
1.6	0.05	yadI	4	23.8333	0.549
1.6	0.05	AG1	4	23.8333	0.608
1.6	0.05	ptsI	4	24.0833	0.542
1.6	0.05	clcB	4	24.0833	0.275
1.6	0.05	ycaM	4	24.0833	0.262
1.6	0.05	yadI	4	24.0833	0.551
1.6	0.05	AG1	4	24.0833	0.61
1.6	0.05	ptsI	5	0	0.168
1.6	0.05	clcB	5	0	0.167
1.6	0.05	ycaM	5	0	0.167
1.6	0.05	yadI	5	0	0.176
1.6	0.05	AG1	5	0	0.167
1.6	0.05	ptsI	5	0.35	0.166
1.6	0.05	clcB	5	0.35	0.164
1.6	0.05	ycaM	5	0.35	0.173
1.6	0.05	yadI	5	0.35	0.173
1.6	0.05	AG1	5	0.35	0.164
1.6	0.05	ptsI	5	0.6	0.166
1.6	0.05	clcB	5	0.6	0.16
1.6	0.05	ycaM	5	0.6	0.158
1.6	0.05	yadI	5	0.6	0.171
1.6	0.05	AG1	5	0.6	0.162
1.6	0.05	ptsI	5	0.85	0.164
1.6	0.05	clcB	5	0.85	0.16
1.6	0.05	ycaM	5	0.85	0.158
1.6	0.05	yadI	5	0.85	0.172
1.6	0.05	AG1	5	0.85	0.163
1.6	0.05	ptsI	5	1.1	0.168
1.6	0.05	clcB	5	1.1	0.165
1.6	0.05	ycaM	5	1.1	0.163
1.6	0.05	yadI	5	1.1	0.174
1.6	0.05	AG1	5	1.1	0.164
1.6	0.05	ptsI	5	1.35	0.167
1.6	0.05	clcB	5	1.35	0.163
1.6	0.05	ycaM	5	1.35	0.161
1.6	0.05	yadI	5	1.35	0.177
1.6	0.05	AG1	5	1.35	0.165
1.6	0.05	ptsI	5	1.6	0.169
1.6	0.05	clcB	5	1.6	0.167
1.6	0.05	ycaM	5	1.6	0.166
1.6	0.05	yadI	5	1.6	0.179
1.6	0.05	AG1	5	1.6	0.168
1.6	0.05	ptsI	5	1.85	0.17
1.6	0.05	clcB	5	1.85	0.166
1.6	0.05	ycaM	5	1.85	0.171
1.6	0.05	yadI	5	1.85	0.183
1.6	0.05	AG1	5	1.85	0.171
1.6	0.05	ptsI	5	2.1	0.176
1.6	0.05	clcB	5	2.1	0.175

1.6	0.05	ycaM	5	2.1	0.175
1.6	0.05	yadI	5	2.1	0.193
1.6	0.05	AG1	5	2.1	0.176
1.6	0.05	ptsI	5	2.35	0.18
1.6	0.05	clcB	5	2.35	0.182
1.6	0.05	ycaM	5	2.35	0.184
1.6	0.05	yadI	5	2.35	0.199
1.6	0.05	AG1	5	2.35	0.182
1.6	0.05	ptsI	5	2.6	0.177
1.6	0.05	clcB	5	2.6	0.175
1.6	0.05	ycaM	5	2.6	0.177
1.6	0.05	yadI	5	2.6	0.2
1.6	0.05	AG1	5	2.6	0.18
1.6	0.05	ptsI	5	2.85	0.183
1.6	0.05	clcB	5	2.85	0.187
1.6	0.05	ycaM	5	2.85	0.19
1.6	0.05	yadI	5	2.85	0.207
1.6	0.05	AG1	5	2.85	0.188
1.6	0.05	ptsI	5	3.1	0.189
1.6	0.05	clcB	5	3.1	0.197
1.6	0.05	ycaM	5	3.1	0.202
1.6	0.05	yadI	5	3.1	0.214
1.6	0.05	AG1	5	3.1	0.197
1.6	0.05	ptsI	5	3.35	0.198
1.6	0.05	clcB	5	3.35	0.205
1.6	0.05	ycaM	5	3.35	0.216
1.6	0.05	yadI	5	3.35	0.224
1.6	0.05	AG1	5	3.35	0.205
1.6	0.05	ptsI	5	3.6	0.211
1.6	0.05	clcB	5	3.6	0.219
1.6	0.05	ycaM	5	3.6	0.232
1.6	0.05	yadI	5	3.6	0.236
1.6	0.05	AG1	5	3.6	0.215
1.6	0.05	ptsI	5	3.85	0.222
1.6	0.05	clcB	5	3.85	0.234
1.6	0.05	ycaM	5	3.85	0.247
1.6	0.05	yadI	5	3.85	0.248
1.6	0.05	AG1	5	3.85	0.222
1.6	0.05	ptsI	5	4.38333	0.223
1.6	0.05	clcB	5	4.38333	0.263
1.6	0.05	ycaM	5	4.38333	0.216
1.6	0.05	yadI	5	4.38333	0.287
1.6	0.05	AG1	5	4.38333	0.257
1.6	0.05	ptsI	5	4.63333	0.221
1.6	0.05	clcB	5	4.63333	0.24
1.6	0.05	ycaM	5	4.63333	0.22
1.6	0.05	yadI	5	4.63333	0.29
1.6	0.05	AG1	5	4.63333	0.26
1.6	0.05	ptsI	5	4.88333	0.23
1.6	0.05	clcB	5	4.88333	0.253
1.6	0.05	ycaM	5	4.88333	0.23
1.6	0.05	yadI	5	4.88333	0.312
1.6	0.05	AG1	5	4.88333	0.28

1.6	0.05	ptsI	5	5.13333	0.238
1.6	0.05	clcB	5	5.13333	0.262
1.6	0.05	ycaM	5	5.13333	0.235
1.6	0.05	yadI	5	5.13333	0.324
1.6	0.05	AG1	5	5.13333	0.291
1.6	0.05	ptsI	5	5.38333	0.25
1.6	0.05	clcB	5	5.38333	0.27
1.6	0.05	ycaM	5	5.38333	0.239
1.6	0.05	yadI	5	5.38333	0.336
1.6	0.05	AG1	5	5.38333	0.307
1.6	0.05	ptsI	5	5.63333	0.257
1.6	0.05	clcB	5	5.63333	0.281
1.6	0.05	ycaM	5	5.63333	0.241
1.6	0.05	yadI	5	5.63333	0.347
1.6	0.05	AG1	5	5.63333	0.32
1.6	0.05	ptsI	5	5.88333	0.27
1.6	0.05	clcB	5	5.88333	0.288
1.6	0.05	ycaM	5	5.88333	0.243
1.6	0.05	yadI	5	5.88333	0.351
1.6	0.05	AG1	5	5.88333	0.328
1.6	0.05	ptsI	5	6.13333	0.282
1.6	0.05	clcB	5	6.13333	0.3
1.6	0.05	ycaM	5	6.13333	0.246
1.6	0.05	yadI	5	6.13333	0.363
1.6	0.05	AG1	5	6.13333	0.341
1.6	0.05	ptsI	5	6.38333	0.297
1.6	0.05	clcB	5	6.38333	0.311
1.6	0.05	ycaM	5	6.38333	0.249
1.6	0.05	yadI	5	6.38333	0.374
1.6	0.05	AG1	5	6.38333	0.352
1.6	0.05	ptsI	5	6.63333	0.314
1.6	0.05	clcB	5	6.63333	0.321
1.6	0.05	ycaM	5	6.63333	0.251
1.6	0.05	yadI	5	6.63333	0.385
1.6	0.05	AG1	5	6.63333	0.364
1.6	0.05	ptsI	5	6.88333	0.33
1.6	0.05	clcB	5	6.88333	0.33
1.6	0.05	ycaM	5	6.88333	0.253
1.6	0.05	yadI	5	6.88333	0.396
1.6	0.05	AG1	5	6.88333	0.376
1.6	0.05	ptsI	5	7.13333	0.346
1.6	0.05	clcB	5	7.13333	0.337
1.6	0.05	ycaM	5	7.13333	0.253
1.6	0.05	yadI	5	7.13333	0.409
1.6	0.05	AG1	5	7.13333	0.388
1.6	0.05	ptsI	5	7.38333	0.362
1.6	0.05	clcB	5	7.38333	0.341
1.6	0.05	ycaM	5	7.38333	0.256
1.6	0.05	yadI	5	7.38333	0.415
1.6	0.05	AG1	5	7.38333	0.398
1.6	0.05	ptsI	5	7.63333	0.374
1.6	0.05	clcB	5	7.63333	0.346
1.6	0.05	ycaM	5	7.63333	0.256

1.6	0.05	yadI	5	7.63333	0.422
1.6	0.05	AG1	5	7.63333	0.408
1.6	0.05	ptsI	5	7.88333	0.391
1.6	0.05	clcB	5	7.88333	0.353
1.6	0.05	ycaM	5	7.88333	0.257
1.6	0.05	yadI	5	7.88333	0.427
1.6	0.05	AG1	5	7.88333	0.418
1.6	0.05	ptsI	5	8.13333	0.407
1.6	0.05	clcB	5	8.13333	0.355
1.6	0.05	ycaM	5	8.13333	0.259
1.6	0.05	yadI	5	8.13333	0.431
1.6	0.05	AG1	5	8.13333	0.429
1.6	0.05	ptsI	5	8.38333	0.424
1.6	0.05	clcB	5	8.38333	0.36
1.6	0.05	ycaM	5	8.38333	0.261
1.6	0.05	yadI	5	8.38333	0.44
1.6	0.05	AG1	5	8.38333	0.44
1.6	0.05	ptsI	5	8.88333	0.353
1.6	0.05	clcB	5	8.88333	0.282
1.6	0.05	ycaM	5	8.88333	0.262
1.6	0.05	yadI	5	8.88333	0.431
1.6	0.05	AG1	5	8.88333	0.435
1.6	0.05	ptsI	5	9.13333	0.359
1.6	0.05	clcB	5	9.13333	0.28
1.6	0.05	ycaM	5	9.13333	0.259
1.6	0.05	yadI	5	9.13333	0.424
1.6	0.05	AG1	5	9.13333	0.441
1.6	0.05	ptsI	5	9.38333	0.375
1.6	0.05	clcB	5	9.38333	0.282
1.6	0.05	ycaM	5	9.38333	0.258
1.6	0.05	yadI	5	9.38333	0.433
1.6	0.05	AG1	5	9.38333	0.459
1.6	0.05	ptsI	5	9.63333	0.38
1.6	0.05	clcB	5	9.63333	0.279
1.6	0.05	ycaM	5	9.63333	0.258
1.6	0.05	yadI	5	9.63333	0.441
1.6	0.05	AG1	5	9.63333	0.471
1.6	0.05	ptsI	5	9.88333	0.39
1.6	0.05	clcB	5	9.88333	0.28
1.6	0.05	ycaM	5	9.88333	0.259
1.6	0.05	yadI	5	9.88333	0.448
1.6	0.05	AG1	5	9.88333	0.479
1.6	0.05	ptsI	5	10.1333	0.396
1.6	0.05	clcB	5	10.1333	0.279
1.6	0.05	ycaM	5	10.1333	0.258
1.6	0.05	yadI	5	10.1333	0.455
1.6	0.05	AG1	5	10.1333	0.484
1.6	0.05	ptsI	5	10.3833	0.406
1.6	0.05	clcB	5	10.3833	0.278
1.6	0.05	ycaM	5	10.3833	0.259
1.6	0.05	yadI	5	10.3833	0.462
1.6	0.05	AG1	5	10.3833	0.492
1.6	0.05	ptsI	5	10.6333	0.41

1.6	0.05	clcB	5	10.6333	0.277
1.6	0.05	ycaM	5	10.6333	0.258
1.6	0.05	yadI	5	10.6333	0.466
1.6	0.05	AG1	5	10.6333	0.499
1.6	0.05	ptsI	5	10.8833	0.416
1.6	0.05	clcB	5	10.8833	0.279
1.6	0.05	ycaM	5	10.8833	0.258
1.6	0.05	yadI	5	10.8833	0.473
1.6	0.05	AG1	5	10.8833	0.507
1.6	0.05	ptsI	5	11.1333	0.421
1.6	0.05	clcB	5	11.1333	0.277
1.6	0.05	ycaM	5	11.1333	0.256
1.6	0.05	yadI	5	11.1333	0.476
1.6	0.05	AG1	5	11.1333	0.511
1.6	0.05	ptsI	5	11.3833	0.423
1.6	0.05	clcB	5	11.3833	0.278
1.6	0.05	ycaM	5	11.3833	0.26
1.6	0.05	yadI	5	11.3833	0.482
1.6	0.05	AG1	5	11.3833	0.518
1.6	0.05	ptsI	5	11.6333	0.428
1.6	0.05	clcB	5	11.6333	0.275
1.6	0.05	ycaM	5	11.6333	0.257
1.6	0.05	yadI	5	11.6333	0.488
1.6	0.05	AG1	5	11.6333	0.525
1.6	0.05	ptsI	5	11.8833	0.434
1.6	0.05	clcB	5	11.8833	0.277
1.6	0.05	ycaM	5	11.8833	0.257
1.6	0.05	yadI	5	11.8833	0.49
1.6	0.05	AG1	5	11.8833	0.531
1.6	0.05	ptsI	5	12.1333	0.439
1.6	0.05	clcB	5	12.1333	0.279
1.6	0.05	ycaM	5	12.1333	0.256
1.6	0.05	yadI	5	12.1333	0.494
1.6	0.05	AG1	5	12.1333	0.54
1.6	0.05	ptsI	5	12.3833	0.44
1.6	0.05	clcB	5	12.3833	0.278
1.6	0.05	ycaM	5	12.3833	0.252
1.6	0.05	yadI	5	12.3833	0.494
1.6	0.05	AG1	5	12.3833	0.536
1.6	0.05	ptsI	5	12.6333	0.446
1.6	0.05	clcB	5	12.6333	0.275
1.6	0.05	ycaM	5	12.6333	0.255
1.6	0.05	yadI	5	12.6333	0.503
1.6	0.05	AG1	5	12.6333	0.555
1.6	0.05	ptsI	5	12.8833	0.448
1.6	0.05	clcB	5	12.8833	0.276
1.6	0.05	ycaM	5	12.8833	0.252
1.6	0.05	yadI	5	12.8833	0.506
1.6	0.05	AG1	5	12.8833	0.556
1.6	0.05	ptsI	5	13.1333	0.453
1.6	0.05	clcB	5	13.1333	0.277
1.6	0.05	ycaM	5	13.1333	0.251
1.6	0.05	yadI	5	13.1333	0.51

1.6	0.05	AG1	5	13.1333	0.57
1.6	0.05	ptsI	5	13.3833	0.455
1.6	0.05	clcB	5	13.3833	0.275
1.6	0.05	ycaM	5	13.3833	0.251
1.6	0.05	yadI	5	13.3833	0.51
1.6	0.05	AG1	5	13.3833	0.573
1.6	0.05	ptsI	5	13.6333	0.455
1.6	0.05	clcB	5	13.6333	0.277
1.6	0.05	ycaM	5	13.6333	0.25
1.6	0.05	yadI	5	13.6333	0.514
1.6	0.05	AG1	5	13.6333	0.578
1.6	0.05	ptsI	5	13.8833	0.464
1.6	0.05	clcB	5	13.8833	0.274
1.6	0.05	ycaM	5	13.8833	0.248
1.6	0.05	yadI	5	13.8833	0.519
1.6	0.05	AG1	5	13.8833	0.583
1.6	0.05	ptsI	5	14.1333	0.467
1.6	0.05	clcB	5	14.1333	0.273
1.6	0.05	ycaM	5	14.1333	0.248
1.6	0.05	yadI	5	14.1333	0.522
1.6	0.05	AG1	5	14.1333	0.585
1.6	0.05	ptsI	5	14.3833	0.468
1.6	0.05	clcB	5	14.3833	0.273
1.6	0.05	ycaM	5	14.3833	0.248
1.6	0.05	yadI	5	14.3833	0.522
1.6	0.05	AG1	5	14.3833	0.594
1.6	0.05	ptsI	5	14.6333	0.471
1.6	0.05	clcB	5	14.6333	0.273
1.6	0.05	ycaM	5	14.6333	0.247
1.6	0.05	yadI	5	14.6333	0.524
1.6	0.05	AG1	5	14.6333	0.599
1.6	0.05	ptsI	5	14.8833	0.474
1.6	0.05	clcB	5	14.8833	0.271
1.6	0.05	ycaM	5	14.8833	0.246
1.6	0.05	yadI	5	14.8833	0.523
1.6	0.05	AG1	5	14.8833	0.608
1.6	0.05	ptsI	5	15.1333	0.477
1.6	0.05	clcB	5	15.1333	0.272
1.6	0.05	ycaM	5	15.1333	0.246
1.6	0.05	yadI	5	15.1333	0.527
1.6	0.05	AG1	5	15.1333	0.608
1.6	0.05	ptsI	5	15.3833	0.481
1.6	0.05	clcB	5	15.3833	0.271
1.6	0.05	ycaM	5	15.3833	0.246
1.6	0.05	yadI	5	15.3833	0.528
1.6	0.05	AG1	5	15.3833	0.616
1.6	0.05	ptsI	5	15.6333	0.479
1.6	0.05	clcB	5	15.6333	0.27
1.6	0.05	ycaM	5	15.6333	0.247
1.6	0.05	yadI	5	15.6333	0.528
1.6	0.05	AG1	5	15.6333	0.616
1.6	0.05	ptsI	5	15.8833	0.486
1.6	0.05	clcB	5	15.8833	0.27

1.6	0.05	ycaM	5	15.8833	0.247
1.6	0.05	yadI	5	15.8833	0.532
1.6	0.05	AG1	5	15.8833	0.622
1.6	0.05	ptsI	5	16.1333	0.49
1.6	0.05	clcB	5	16.1333	0.272
1.6	0.05	ycaM	5	16.1333	0.246
1.6	0.05	yadI	5	16.1333	0.533
1.6	0.05	AG1	5	16.1333	0.631
1.6	0.05	ptsI	5	16.3833	0.495
1.6	0.05	clcB	5	16.3833	0.271
1.6	0.05	ycaM	5	16.3833	0.247
1.6	0.05	yadI	5	16.3833	0.536
1.6	0.05	AG1	5	16.3833	0.629
1.6	0.05	ptsI	5	16.6333	0.499
1.6	0.05	clcB	5	16.6333	0.27
1.6	0.05	ycaM	5	16.6333	0.247
1.6	0.05	yadI	5	16.6333	0.536
1.6	0.05	AG1	5	16.6333	0.634
1.6	0.05	ptsI	5	16.8833	0.499
1.6	0.05	clcB	5	16.8833	0.27
1.6	0.05	ycaM	5	16.8833	0.247
1.6	0.05	yadI	5	16.8833	0.533
1.6	0.05	AG1	5	16.8833	0.635
1.6	0.05	ptsI	5	17.1333	0.49
1.6	0.05	clcB	5	17.1333	0.268
1.6	0.05	ycaM	5	17.1333	0.248
1.6	0.05	yadI	5	17.1333	0.533
1.6	0.05	AG1	5	17.1333	0.637
1.6	0.05	ptsI	5	17.3833	0.502
1.6	0.05	clcB	5	17.3833	0.27
1.6	0.05	ycaM	5	17.3833	0.248
1.6	0.05	yadI	5	17.3833	0.534
1.6	0.05	AG1	5	17.3833	0.636
1.6	0.05	ptsI	5	17.6333	0.504
1.6	0.05	clcB	5	17.6333	0.27
1.6	0.05	ycaM	5	17.6333	0.248
1.6	0.05	yadI	5	17.6333	0.534
1.6	0.05	AG1	5	17.6333	0.64
1.6	0.05	ptsI	5	17.8833	0.5
1.6	0.05	clcB	5	17.8833	0.269
1.6	0.05	ycaM	5	17.8833	0.247
1.6	0.05	yadI	5	17.8833	0.526
1.6	0.05	AG1	5	17.8833	0.634
1.6	0.05	ptsI	5	18.1333	0.511
1.6	0.05	clcB	5	18.1333	0.27
1.6	0.05	ycaM	5	18.1333	0.25
1.6	0.05	yadI	5	18.1333	0.53
1.6	0.05	AG1	5	18.1333	0.643
1.6	0.05	ptsI	5	18.3833	0.51
1.6	0.05	clcB	5	18.3833	0.269
1.6	0.05	ycaM	5	18.3833	0.25
1.6	0.05	yadI	5	18.3833	0.531
1.6	0.05	AG1	5	18.3833	0.643

1.6	0.05	ptsI	5	18.6333	0.511
1.6	0.05	clcB	5	18.6333	0.27
1.6	0.05	ycaM	5	18.6333	0.251
1.6	0.05	yadI	5	18.6333	0.527
1.6	0.05	AG1	5	18.6333	0.644
1.6	0.05	ptsI	5	18.8833	0.516
1.6	0.05	clcB	5	18.8833	0.27
1.6	0.05	ycaM	5	18.8833	0.251
1.6	0.05	yadI	5	18.8833	0.527
1.6	0.05	AG1	5	18.8833	0.644
1.6	0.05	ptsI	5	19.1333	0.514
1.6	0.05	clcB	5	19.1333	0.269
1.6	0.05	ycaM	5	19.1333	0.251
1.6	0.05	yadI	5	19.1333	0.526
1.6	0.05	AG1	5	19.1333	0.647
1.6	0.05	ptsI	5	19.3833	0.518
1.6	0.05	clcB	5	19.3833	0.27
1.6	0.05	ycaM	5	19.3833	0.252
1.6	0.05	yadI	5	19.3833	0.528
1.6	0.05	AG1	5	19.3833	0.652
1.6	0.05	ptsI	5	19.6333	0.521
1.6	0.05	clcB	5	19.6333	0.27
1.6	0.05	ycaM	5	19.6333	0.253
1.6	0.05	yadI	5	19.6333	0.525
1.6	0.05	AG1	5	19.6333	0.65
1.6	0.05	ptsI	5	19.8833	0.523
1.6	0.05	clcB	5	19.8833	0.269
1.6	0.05	ycaM	5	19.8833	0.252
1.6	0.05	yadI	5	19.8833	0.525
1.6	0.05	AG1	5	19.8833	0.65
1.6	0.05	ptsI	5	20.1333	0.521
1.6	0.05	clcB	5	20.1333	0.268
1.6	0.05	ycaM	5	20.1333	0.253
1.6	0.05	yadI	5	20.1333	0.528
1.6	0.05	AG1	5	20.1333	0.652
1.6	0.05	ptsI	5	20.3833	0.525
1.6	0.05	clcB	5	20.3833	0.268
1.6	0.05	ycaM	5	20.3833	0.254
1.6	0.05	yadI	5	20.3833	0.526
1.6	0.05	AG1	5	20.3833	0.652
1.6	0.05	ptsI	5	20.6333	0.52
1.6	0.05	clcB	5	20.6333	0.27
1.6	0.05	ycaM	5	20.6333	0.249
1.6	0.05	yadI	5	20.6333	0.527
1.6	0.05	AG1	5	20.6333	0.645
1.6	0.05	ptsI	5	20.8833	0.533
1.6	0.05	clcB	5	20.8833	0.267
1.6	0.05	ycaM	5	20.8833	0.253
1.6	0.05	yadI	5	20.8833	0.529
1.6	0.05	AG1	5	20.8833	0.654
1.6	0.05	ptsI	5	21.1333	0.532
1.6	0.05	clcB	5	21.1333	0.268
1.6	0.05	ycaM	5	21.1333	0.252

1.6	0.05	yadI	5	21.1333	0.53
1.6	0.05	AG1	5	21.1333	0.659
1.6	0.05	ptsI	5	21.3833	0.536
1.6	0.05	clcB	5	21.3833	0.267
1.6	0.05	ycaM	5	21.3833	0.253
1.6	0.05	yadI	5	21.3833	0.531
1.6	0.05	AG1	5	21.3833	0.659
1.6	0.05	ptsI	5	21.6333	0.538
1.6	0.05	clcB	5	21.6333	0.268
1.6	0.05	ycaM	5	21.6333	0.252
1.6	0.05	yadI	5	21.6333	0.53
1.6	0.05	AG1	5	21.6333	0.662
1.6	0.05	ptsI	5	21.8833	0.54
1.6	0.05	clcB	5	21.8833	0.268
1.6	0.05	ycaM	5	21.8833	0.252
1.6	0.05	yadI	5	21.8833	0.53
1.6	0.05	AG1	5	21.8833	0.666
1.6	0.05	ptsI	5	22.1333	0.539
1.6	0.05	clcB	5	22.1333	0.266
1.6	0.05	ycaM	5	22.1333	0.251
1.6	0.05	yadI	5	22.1333	0.526
1.6	0.05	AG1	5	22.1333	0.656
1.6	0.05	ptsI	5	22.3833	0.544
1.6	0.05	clcB	5	22.3833	0.267
1.6	0.05	ycaM	5	22.3833	0.252
1.6	0.05	yadI	5	22.3833	0.532
1.6	0.05	AG1	5	22.3833	0.671
1.6	0.05	ptsI	5	22.6333	0.55
1.6	0.05	clcB	5	22.6333	0.268
1.6	0.05	ycaM	5	22.6333	0.253
1.6	0.05	yadI	5	22.6333	0.534
1.6	0.05	AG1	5	22.6333	0.675
1.6	0.05	ptsI	5	22.8833	0.548
1.6	0.05	clcB	5	22.8833	0.267
1.6	0.05	ycaM	5	22.8833	0.252
1.6	0.05	yadI	5	22.8833	0.534
1.6	0.05	AG1	5	22.8833	0.677
1.6	0.05	ptsI	5	23.1333	0.552
1.6	0.05	clcB	5	23.1333	0.267
1.6	0.05	ycaM	5	23.1333	0.252
1.6	0.05	yadI	5	23.1333	0.536
1.6	0.05	AG1	5	23.1333	0.68
1.6	0.05	ptsI	5	23.3833	0.551
1.6	0.05	clcB	5	23.3833	0.267
1.6	0.05	ycaM	5	23.3833	0.251
1.6	0.05	yadI	5	23.3833	0.536
1.6	0.05	AG1	5	23.3833	0.682
1.6	0.05	ptsI	5	23.6333	0.551
1.6	0.05	clcB	5	23.6333	0.266
1.6	0.05	ycaM	5	23.6333	0.251
1.6	0.05	yadI	5	23.6333	0.534
1.6	0.05	AG1	5	23.6333	0.682
1.6	0.05	ptsI	5	23.8833	0.55

1.6	0.05	clcB	5	23.8833	0.266
1.6	0.05	ycaM	5	23.8833	0.249
1.6	0.05	yadI	5	23.8833	0.533
1.6	0.05	AG1	5	23.8833	0.672
1.6	0.05	ptsI	5	24.1333	0.555
1.6	0.05	clcB	5	24.1333	0.266
1.6	0.05	ycaM	5	24.1333	0.251
1.6	0.05	yadI	5	24.1333	0.537
1.6	0.05	AG1	5	24.1333	0.69
1.6	0.05	ptsI	5	24.3833	0.553
1.6	0.05	clcB	5	24.3833	0.267
1.6	0.05	ycaM	5	24.3833	0.249
1.6	0.05	yadI	5	24.3833	0.538
1.6	0.05	AG1	5	24.3833	0.688
3.2	0.05	ptsI	2	0	0.212
3.2	0.05	clcB	2	0	0.202
3.2	0.05	ycaM	2	0	0.212
3.2	0.05	yadI	2	0	1.942
3.2	0.05	AG1	2	0	0.233
3.2	0.05	ptsI	2	0.25	0.21
3.2	0.05	clcB	2	0.25	0.198
3.2	0.05	ycaM	2	0.25	0.209
3.2	0.05	yadI	2	0.25	1.93
3.2	0.05	AG1	2	0.25	0.229
3.2	0.05	ptsI	2	0.5	0.209
3.2	0.05	clcB	2	0.5	0.2
3.2	0.05	ycaM	2	0.5	0.21
3.2	0.05	yadI	2	0.5	1.9
3.2	0.05	AG1	2	0.5	0.232
3.2	0.05	ptsI	2	0.75	0.215
3.2	0.05	clcB	2	0.75	0.199
3.2	0.05	ycaM	2	0.75	0.215
3.2	0.05	yadI	2	0.75	1.896
3.2	0.05	AG1	2	0.75	0.235
3.2	0.05	ptsI	2	1	0.214
3.2	0.05	clcB	2	1	0.201
3.2	0.05	ycaM	2	1	0.214
3.2	0.05	yadI	2	1	1.851
3.2	0.05	AG1	2	1	0.242
3.2	0.05	ptsI	2	1.25	0.214
3.2	0.05	clcB	2	1.25	0.199
3.2	0.05	ycaM	2	1.25	0.216
3.2	0.05	yadI	2	1.25	1.782
3.2	0.05	AG1	2	1.25	0.236
3.2	0.05	ptsI	2	1.5	0.218
3.2	0.05	clcB	2	1.5	0.202
3.2	0.05	ycaM	2	1.5	0.217
3.2	0.05	yadI	2	1.5	1.692
3.2	0.05	AG1	2	1.5	0.242
3.2	0.05	ptsI	2	1.75	0.22
3.2	0.05	clcB	2	1.75	0.203
3.2	0.05	ycaM	2	1.75	0.222
3.2	0.05	yadI	2	1.75	1.495

3.2	0.05	AG1	2	1.75	0.248
3.2	0.05	ptsI	2	2	0.227
3.2	0.05	clcB	2	2	0.207
3.2	0.05	ycaM	2	2	0.229
3.2	0.05	yadI	2	2	1.418
3.2	0.05	AG1	2	2	0.257
3.2	0.05	ptsI	2	2.25	0.233
3.2	0.05	clcB	2	2.25	0.209
3.2	0.05	ycaM	2	2.25	0.233
3.2	0.05	yadI	2	2.25	1.288
3.2	0.05	AG1	2	2.25	0.264
3.2	0.05	ptsI	2	2.5	0.242
3.2	0.05	clcB	2	2.5	0.212
3.2	0.05	ycaM	2	2.5	0.242
3.2	0.05	yadI	2	2.5	1.254
3.2	0.05	AG1	2	2.5	0.274
3.2	0.05	ptsI	2	2.75	0.238
3.2	0.05	clcB	2	2.75	0.212
3.2	0.05	ycaM	2	2.75	0.244
3.2	0.05	yadI	2	2.75	1.162
3.2	0.05	AG1	2	2.75	0.261
3.2	0.05	ptsI	2	3	0.239
3.2	0.05	clcB	2	3	0.217
3.2	0.05	ycaM	2	3	0.247
3.2	0.05	yadI	2	3	0.907
3.2	0.05	AG1	2	3	0.267
3.2	0.05	ptsI	2	3.25	0.25
3.2	0.05	clcB	2	3.25	0.222
3.2	0.05	ycaM	2	3.25	0.254
3.2	0.05	yadI	2	3.25	1.06
3.2	0.05	AG1	2	3.25	0.277
3.2	0.05	ptsI	2	3.5	0.258
3.2	0.05	clcB	2	3.5	0.227
3.2	0.05	ycaM	2	3.5	0.262
3.2	0.05	yadI	2	3.5	0.936
3.2	0.05	AG1	2	3.5	0.286
3.2	0.05	ptsI	2	3.75	0.266
3.2	0.05	clcB	2	3.75	0.234
3.2	0.05	ycaM	2	3.75	0.268
3.2	0.05	yadI	2	3.75	0.838
3.2	0.05	AG1	2	3.75	0.298
3.2	0.05	ptsI	2	4	0.274
3.2	0.05	clcB	2	4	0.239
3.2	0.05	ycaM	2	4	0.277
3.2	0.05	yadI	2	4	0.814
3.2	0.05	AG1	2	4	0.307
3.2	0.05	ptsI	2	4.38333	0.302
3.2	0.05	clcB	2	4.38333	0.231
3.2	0.05	ycaM	2	4.38333	0.3
3.2	0.05	yadI	2	4.38333	0.4
3.2	0.05	AG1	2	4.38333	0.279
3.2	0.05	ptsI	2	4.63333	0.298
3.2	0.05	clcB	2	4.63333	0.238

3.2	0.05	ycaM	2	4.63333	0.297
3.2	0.05	yadI	2	4.63333	0.4
3.2	0.05	AG1	2	4.63333	0.286
3.2	0.05	ptsI	2	4.88333	0.302
3.2	0.05	clcB	2	4.88333	0.227
3.2	0.05	ycaM	2	4.88333	0.304
3.2	0.05	yadI	2	4.88333	0.418
3.2	0.05	AG1	2	4.88333	0.3
3.2	0.05	ptsI	2	5.13333	0.309
3.2	0.05	clcB	2	5.13333	0.232
3.2	0.05	ycaM	2	5.13333	0.309
3.2	0.05	yadI	2	5.13333	0.43
3.2	0.05	AG1	2	5.13333	0.308
3.2	0.05	ptsI	2	5.38333	0.316
3.2	0.05	clcB	2	5.38333	0.238
3.2	0.05	ycaM	2	5.38333	0.31
3.2	0.05	yadI	2	5.38333	0.445
3.2	0.05	AG1	2	5.38333	0.317
3.2	0.05	ptsI	2	5.63333	0.322
3.2	0.05	clcB	2	5.63333	0.244
3.2	0.05	ycaM	2	5.63333	0.312
3.2	0.05	yadI	2	5.63333	0.444
3.2	0.05	AG1	2	5.63333	0.325
3.2	0.05	ptsI	2	5.88333	0.328
3.2	0.05	clcB	2	5.88333	0.252
3.2	0.05	ycaM	2	5.88333	0.314
3.2	0.05	yadI	2	5.88333	0.451
3.2	0.05	AG1	2	5.88333	0.334
3.2	0.05	ptsI	2	6.13333	0.338
3.2	0.05	clcB	2	6.13333	0.258
3.2	0.05	ycaM	2	6.13333	0.317
3.2	0.05	yadI	2	6.13333	0.459
3.2	0.05	AG1	2	6.13333	0.342
3.2	0.05	ptsI	2	6.38333	0.345
3.2	0.05	clcB	2	6.38333	0.266
3.2	0.05	ycaM	2	6.38333	0.318
3.2	0.05	yadI	2	6.38333	0.47
3.2	0.05	AG1	2	6.38333	0.351
3.2	0.05	ptsI	2	6.63333	0.353
3.2	0.05	clcB	2	6.63333	0.271
3.2	0.05	ycaM	2	6.63333	0.321
3.2	0.05	yadI	2	6.63333	0.485
3.2	0.05	AG1	2	6.63333	0.362
3.2	0.05	ptsI	2	6.88333	0.363
3.2	0.05	clcB	2	6.88333	0.278
3.2	0.05	ycaM	2	6.88333	0.32
3.2	0.05	yadI	2	6.88333	0.5
3.2	0.05	AG1	2	6.88333	0.37
3.2	0.05	ptsI	2	7.13333	0.371
3.2	0.05	clcB	2	7.13333	0.282
3.2	0.05	ycaM	2	7.13333	0.327
3.2	0.05	yadI	2	7.13333	0.51
3.2	0.05	AG1	2	7.13333	0.379

3.2	0.05	ptsI	2	7.38333	0.378
3.2	0.05	clcB	2	7.38333	0.29
3.2	0.05	ycaM	2	7.38333	0.328
3.2	0.05	yadI	2	7.38333	0.521
3.2	0.05	AG1	2	7.38333	0.387
3.2	0.05	ptsI	2	7.63333	0.39
3.2	0.05	clcB	2	7.63333	0.298
3.2	0.05	ycaM	2	7.63333	0.327
3.2	0.05	yadI	2	7.63333	0.556
3.2	0.05	AG1	2	7.63333	0.393
3.2	0.05	ptsI	2	7.88333	0.398
3.2	0.05	clcB	2	7.88333	0.303
3.2	0.05	ycaM	2	7.88333	0.33
3.2	0.05	yadI	2	7.88333	0.58
3.2	0.05	AG1	2	7.88333	0.402
3.2	0.05	ptsI	2	8.13333	0.409
3.2	0.05	clcB	2	8.13333	0.311
3.2	0.05	ycaM	2	8.13333	0.333
3.2	0.05	yadI	2	8.13333	0.579
3.2	0.05	AG1	2	8.13333	0.411
3.2	0.05	ptsI	2	8.38333	0.418
3.2	0.05	clcB	2	8.38333	0.317
3.2	0.05	ycaM	2	8.38333	0.335
3.2	0.05	yadI	2	8.38333	0.608
3.2	0.05	AG1	2	8.38333	0.42
3.2	0.05	ptsI	2	8.91667	0.371
3.2	0.05	clcB	2	8.91667	0.3
3.2	0.05	ycaM	2	8.91667	0.326
3.2	0.05	yadI	2	8.91667	0.541
3.2	0.05	AG1	2	8.91667	0.469
3.2	0.05	ptsI	2	9.16667	0.381
3.2	0.05	clcB	2	9.16667	0.296
3.2	0.05	ycaM	2	9.16667	0.321
3.2	0.05	yadI	2	9.16667	0.57
3.2	0.05	AG1	2	9.16667	0.486
3.2	0.05	ptsI	2	9.41667	0.38
3.2	0.05	clcB	2	9.41667	0.295
3.2	0.05	ycaM	2	9.41667	0.317
3.2	0.05	yadI	2	9.41667	0.581
3.2	0.05	AG1	2	9.41667	0.486
3.2	0.05	ptsI	2	9.66667	0.385
3.2	0.05	clcB	2	9.66667	0.291
3.2	0.05	ycaM	2	9.66667	0.322
3.2	0.05	yadI	2	9.66667	0.593
3.2	0.05	AG1	2	9.66667	0.504
3.2	0.05	ptsI	2	9.91667	0.383
3.2	0.05	clcB	2	9.91667	0.289
3.2	0.05	ycaM	2	9.91667	0.316
3.2	0.05	yadI	2	9.91667	0.593
3.2	0.05	AG1	2	9.91667	0.499
3.2	0.05	ptsI	2	10.1667	0.385
3.2	0.05	clcB	2	10.1667	0.283
3.2	0.05	ycaM	2	10.1667	0.319

3.2	0.05	yadI	2	10.1667	0.596
3.2	0.05	AG1	2	10.1667	0.501
3.2	0.05	ptsI	2	10.4167	0.392
3.2	0.05	clcB	2	10.4167	0.287
3.2	0.05	ycaM	2	10.4167	0.32
3.2	0.05	yadI	2	10.4167	0.614
3.2	0.05	AG1	2	10.4167	0.515
3.2	0.05	ptsI	2	10.6667	0.395
3.2	0.05	clcB	2	10.6667	0.281
3.2	0.05	ycaM	2	10.6667	0.321
3.2	0.05	yadI	2	10.6667	0.614
3.2	0.05	AG1	2	10.6667	0.519
3.2	0.05	ptsI	2	10.9167	0.399
3.2	0.05	clcB	2	10.9167	0.282
3.2	0.05	ycaM	2	10.9167	0.322
3.2	0.05	yadI	2	10.9167	0.614
3.2	0.05	AG1	2	10.9167	0.524
3.2	0.05	ptsI	2	11.1667	0.405
3.2	0.05	clcB	2	11.1667	0.284
3.2	0.05	ycaM	2	11.1667	0.324
3.2	0.05	yadI	2	11.1667	0.63
3.2	0.05	AG1	2	11.1667	0.521
3.2	0.05	ptsI	2	11.4167	0.415
3.2	0.05	clcB	2	11.4167	0.28
3.2	0.05	ycaM	2	11.4167	0.328
3.2	0.05	yadI	2	11.4167	0.633
3.2	0.05	AG1	2	11.4167	0.519
3.2	0.05	ptsI	2	11.6667	0.414
3.2	0.05	clcB	2	11.6667	0.28
3.2	0.05	ycaM	2	11.6667	0.316
3.2	0.05	yadI	2	11.6667	0.645
3.2	0.05	AG1	2	11.6667	0.525
3.2	0.05	ptsI	2	11.9167	0.415
3.2	0.05	clcB	2	11.9167	0.28
3.2	0.05	ycaM	2	11.9167	0.321
3.2	0.05	yadI	2	11.9167	0.651
3.2	0.05	AG1	2	11.9167	0.529
3.2	0.05	ptsI	2	12.1667	0.424
3.2	0.05	clcB	2	12.1667	0.278
3.2	0.05	ycaM	2	12.1667	0.315
3.2	0.05	yadI	2	12.1667	0.658
3.2	0.05	AG1	2	12.1667	0.537
3.2	0.05	ptsI	2	12.4167	0.425
3.2	0.05	clcB	2	12.4167	0.279
3.2	0.05	ycaM	2	12.4167	0.305
3.2	0.05	yadI	2	12.4167	0.662
3.2	0.05	AG1	2	12.4167	0.538
3.2	0.05	ptsI	2	12.6667	0.424
3.2	0.05	clcB	2	12.6667	0.272
3.2	0.05	ycaM	2	12.6667	0.307
3.2	0.05	yadI	2	12.6667	0.664
3.2	0.05	AG1	2	12.6667	0.539
3.2	0.05	ptsI	2	12.9167	0.426

3.2	0.05	clcB	2	12.9167	0.272
3.2	0.05	ycaM	2	12.9167	0.303
3.2	0.05	yadI	2	12.9167	0.669
3.2	0.05	AG1	2	12.9167	0.542
3.2	0.05	ptsI	2	13.1667	0.427
3.2	0.05	clcB	2	13.1667	0.268
3.2	0.05	ycaM	2	13.1667	0.502
3.2	0.05	yadI	2	13.1667	0.675
3.2	0.05	AG1	2	13.1667	0.547
3.2	0.05	ptsI	2	13.4167	0.428
3.2	0.05	clcB	2	13.4167	0.267
3.2	0.05	ycaM	2	13.4167	0.487
3.2	0.05	yadI	2	13.4167	0.686
3.2	0.05	AG1	2	13.4167	0.549
3.2	0.05	ptsI	2	13.6667	0.43
3.2	0.05	clcB	2	13.6667	0.264
3.2	0.05	ycaM	2	13.6667	0.466
3.2	0.05	yadI	2	13.6667	0.69
3.2	0.05	AG1	2	13.6667	0.555
3.2	0.05	ptsI	2	13.9167	0.429
3.2	0.05	clcB	2	13.9167	0.264
3.2	0.05	ycaM	2	13.9167	0.456
3.2	0.05	yadI	2	13.9167	0.696
3.2	0.05	AG1	2	13.9167	0.559
3.2	0.05	ptsI	2	14.1667	0.431
3.2	0.05	clcB	2	14.1667	0.262
3.2	0.05	ycaM	2	14.1667	0.418
3.2	0.05	yadI	2	14.1667	0.699
3.2	0.05	AG1	2	14.1667	0.562
3.2	0.05	ptsI	2	14.4167	0.432
3.2	0.05	clcB	2	14.4167	0.26
3.2	0.05	ycaM	2	14.4167	0.384
3.2	0.05	yadI	2	14.4167	0.712
3.2	0.05	AG1	2	14.4167	0.568
3.2	0.05	ptsI	2	14.6667	0.432
3.2	0.05	clcB	2	14.6667	0.26
3.2	0.05	ycaM	2	14.6667	0.39
3.2	0.05	yadI	2	14.6667	0.717
3.2	0.05	AG1	2	14.6667	0.576
3.2	0.05	ptsI	2	14.9167	0.433
3.2	0.05	clcB	2	14.9167	0.259
3.2	0.05	ycaM	2	14.9167	0.39
3.2	0.05	yadI	2	14.9167	0.724
3.2	0.05	AG1	2	14.9167	0.575
3.2	0.05	ptsI	2	15.1667	0.436
3.2	0.05	clcB	2	15.1667	0.257
3.2	0.05	ycaM	2	15.1667	0.436
3.2	0.05	yadI	2	15.1667	0.693
3.2	0.05	AG1	2	15.1667	0.592
3.2	0.05	ptsI	2	15.4167	0.437
3.2	0.05	clcB	2	15.4167	0.257
3.2	0.05	ycaM	2	15.4167	0.376
3.2	0.05	yadI	2	15.4167	0.741

3.2	0.05	AG1	2	15.4167	0.582
3.2	0.05	ptsI	2	15.6667	0.437
3.2	0.05	clcB	2	15.6667	0.257
3.2	0.05	ycaM	2	15.6667	0.381
3.2	0.05	yadI	2	15.6667	0.743
3.2	0.05	AG1	2	15.6667	0.586
3.2	0.05	ptsI	2	15.9167	0.442
3.2	0.05	clcB	2	15.9167	0.257
3.2	0.05	ycaM	2	15.9167	0.381
3.2	0.05	yadI	2	15.9167	0.751
3.2	0.05	AG1	2	15.9167	0.587
3.2	0.05	ptsI	2	16.1667	0.444
3.2	0.05	clcB	2	16.1667	0.255
3.2	0.05	ycaM	2	16.1667	0.375
3.2	0.05	yadI	2	16.1667	0.758
3.2	0.05	AG1	2	16.1667	0.594
3.2	0.05	ptsI	2	16.4167	0.443
3.2	0.05	clcB	2	16.4167	0.254
3.2	0.05	ycaM	2	16.4167	0.375
3.2	0.05	yadI	2	16.4167	0.756
3.2	0.05	AG1	2	16.4167	0.596
3.2	0.05	ptsI	2	16.6667	0.445
3.2	0.05	clcB	2	16.6667	0.253
3.2	0.05	ycaM	2	16.6667	0.376
3.2	0.05	yadI	2	16.6667	0.759
3.2	0.05	AG1	2	16.6667	0.601
3.2	0.05	ptsI	2	16.9167	0.445
3.2	0.05	clcB	2	16.9167	0.252
3.2	0.05	ycaM	2	16.9167	0.377
3.2	0.05	yadI	2	16.9167	0.774
3.2	0.05	AG1	2	16.9167	0.602
3.2	0.05	ptsI	2	17.1667	0.451
3.2	0.05	clcB	2	17.1667	0.253
3.2	0.05	ycaM	2	17.1667	0.372
3.2	0.05	yadI	2	17.1667	0.773
3.2	0.05	AG1	2	17.1667	0.608
3.2	0.05	ptsI	2	17.4167	0.451
3.2	0.05	clcB	2	17.4167	0.251
3.2	0.05	ycaM	2	17.4167	0.368
3.2	0.05	yadI	2	17.4167	0.783
3.2	0.05	AG1	2	17.4167	0.612
3.2	0.05	ptsI	2	17.6667	0.45
3.2	0.05	clcB	2	17.6667	0.249
3.2	0.05	ycaM	2	17.6667	0.357
3.2	0.05	yadI	2	17.6667	0.791
3.2	0.05	AG1	2	17.6667	0.615
3.2	0.05	ptsI	2	17.9167	0.451
3.2	0.05	clcB	2	17.9167	0.248
3.2	0.05	ycaM	2	17.9167	0.358
3.2	0.05	yadI	2	17.9167	0.787
3.2	0.05	AG1	2	17.9167	0.618
3.2	0.05	ptsI	2	18.1667	0.451
3.2	0.05	clcB	2	18.1667	0.247

3.2	0.05	ycaM	2	18.1667	0.362
3.2	0.05	yadI	2	18.1667	0.797
3.2	0.05	AG1	2	18.1667	0.623
3.2	0.05	ptsI	2	18.4167	0.452
3.2	0.05	clcB	2	18.4167	0.247
3.2	0.05	ycaM	2	18.4167	0.353
3.2	0.05	yadI	2	18.4167	0.81
3.2	0.05	AG1	2	18.4167	0.623
3.2	0.05	ptsI	2	18.6667	0.455
3.2	0.05	clcB	2	18.6667	0.247
3.2	0.05	ycaM	2	18.6667	0.348
3.2	0.05	yadI	2	18.6667	0.809
3.2	0.05	AG1	2	18.6667	0.629
3.2	0.05	ptsI	2	18.9167	0.453
3.2	0.05	clcB	2	18.9167	0.246
3.2	0.05	ycaM	2	18.9167	0.319
3.2	0.05	yadI	2	18.9167	0.88
3.2	0.05	AG1	2	18.9167	0.619
3.2	0.05	ptsI	2	19.1667	0.458
3.2	0.05	clcB	2	19.1667	0.243
3.2	0.05	ycaM	2	19.1667	0.345
3.2	0.05	yadI	2	19.1667	0.808
3.2	0.05	AG1	2	19.1667	0.634
3.2	0.05	ptsI	2	19.4167	0.457
3.2	0.05	clcB	2	19.4167	0.244
3.2	0.05	ycaM	2	19.4167	0.349
3.2	0.05	yadI	2	19.4167	0.813
3.2	0.05	AG1	2	19.4167	0.637
3.2	0.05	ptsI	2	19.6667	0.459
3.2	0.05	clcB	2	19.6667	0.242
3.2	0.05	ycaM	2	19.6667	0.348
3.2	0.05	yadI	2	19.6667	0.815
3.2	0.05	AG1	2	19.6667	0.641
3.2	0.05	ptsI	2	19.9167	0.462
3.2	0.05	clcB	2	19.9167	0.242
3.2	0.05	ycaM	2	19.9167	0.347
3.2	0.05	yadI	2	19.9167	0.819
3.2	0.05	AG1	2	19.9167	0.646
3.2	0.05	ptsI	2	20.1667	0.46
3.2	0.05	clcB	2	20.1667	0.242
3.2	0.05	ycaM	2	20.1667	0.344
3.2	0.05	yadI	2	20.1667	0.827
3.2	0.05	AG1	2	20.1667	0.65
3.2	0.05	ptsI	2	20.4167	0.462
3.2	0.05	clcB	2	20.4167	0.24
3.2	0.05	ycaM	2	20.4167	0.341
3.2	0.05	yadI	2	20.4167	0.826
3.2	0.05	AG1	2	20.4167	0.651
3.2	0.05	ptsI	2	20.6667	0.464
3.2	0.05	clcB	2	20.6667	0.24
3.2	0.05	ycaM	2	20.6667	0.34
3.2	0.05	yadI	2	20.6667	0.827
3.2	0.05	AG1	2	20.6667	0.658

3.2	0.05	ptsI	2	20.9167	0.467
3.2	0.05	clcB	2	20.9167	0.239
3.2	0.05	ycaM	2	20.9167	0.339
3.2	0.05	yadI	2	20.9167	0.829
3.2	0.05	AG1	2	20.9167	0.657
3.2	0.05	ptsI	2	21.1667	0.466
3.2	0.05	clcB	2	21.1667	0.238
3.2	0.05	ycaM	2	21.1667	0.336
3.2	0.05	yadI	2	21.1667	0.835
3.2	0.05	AG1	2	21.1667	0.662
3.2	0.05	ptsI	2	21.4167	0.466
3.2	0.05	clcB	2	21.4167	0.238
3.2	0.05	ycaM	2	21.4167	0.332
3.2	0.05	yadI	2	21.4167	0.872
3.2	0.05	AG1	2	21.4167	0.667
3.2	0.05	ptsI	2	21.6667	0.469
3.2	0.05	clcB	2	21.6667	0.238
3.2	0.05	ycaM	2	21.6667	0.325
3.2	0.05	yadI	2	21.6667	0.845
3.2	0.05	AG1	2	21.6667	0.675
3.2	0.05	ptsI	2	21.9167	0.468
3.2	0.05	clcB	2	21.9167	0.237
3.2	0.05	ycaM	2	21.9167	0.333
3.2	0.05	yadI	2	21.9167	0.845
3.2	0.05	AG1	2	21.9167	0.676
3.2	0.05	ptsI	2	22.1667	0.472
3.2	0.05	clcB	2	22.1667	0.235
3.2	0.05	ycaM	2	22.1667	0.335
3.2	0.05	yadI	2	22.1667	0.849
3.2	0.05	AG1	2	22.1667	0.683
3.2	0.05	ptsI	2	22.4167	0.47
3.2	0.05	clcB	2	22.4167	0.235
3.2	0.05	ycaM	2	22.4167	0.329
3.2	0.05	yadI	2	22.4167	0.853
3.2	0.05	AG1	2	22.4167	0.687
3.2	0.05	ptsI	2	22.6667	0.472
3.2	0.05	clcB	2	22.6667	0.233
3.2	0.05	ycaM	2	22.6667	0.331
3.2	0.05	yadI	2	22.6667	0.856
3.2	0.05	AG1	2	22.6667	0.689
3.2	0.05	ptsI	2	22.9167	0.474
3.2	0.05	clcB	2	22.9167	0.234
3.2	0.05	ycaM	2	22.9167	0.32
3.2	0.05	yadI	2	22.9167	0.853
3.2	0.05	AG1	2	22.9167	0.694
3.2	0.05	ptsI	2	23.1667	0.476
3.2	0.05	clcB	2	23.1667	0.233
3.2	0.05	ycaM	2	23.1667	0.323
3.2	0.05	yadI	2	23.1667	0.859
3.2	0.05	AG1	2	23.1667	0.698
3.2	0.05	ptsI	2	23.4167	0.479
3.2	0.05	clcB	2	23.4167	0.231
3.2	0.05	ycaM	2	23.4167	0.32

3.2	0.05	yadI	2	23.4167	0.857
3.2	0.05	AG1	2	23.4167	0.705
3.2	0.05	ptsI	2	23.6667	0.48
3.2	0.05	clcB	2	23.6667	0.23
3.2	0.05	ycaM	2	23.6667	0.325
3.2	0.05	yadI	2	23.6667	0.858
3.2	0.05	AG1	2	23.6667	0.71
3.2	0.05	ptsI	2	23.9167	0.479
3.2	0.05	clcB	2	23.9167	0.229
3.2	0.05	ycaM	2	23.9167	0.323
3.2	0.05	yadI	2	23.9167	0.865
3.2	0.05	AG1	2	23.9167	0.716
3.2	0.05	ptsI	2	24.1667	0.481
3.2	0.05	clcB	2	24.1667	0.229
3.2	0.05	ycaM	2	24.1667	0.322
3.2	0.05	yadI	2	24.1667	0.869
3.2	0.05	AG1	2	24.1667	0.722
3.2	0.05	ptsI	2	24.4167	0.485
3.2	0.05	clcB	2	24.4167	0.229
3.2	0.05	ycaM	2	24.4167	0.32
3.2	0.05	yadI	2	24.4167	0.874
3.2	0.05	AG1	2	24.4167	0.732
3.2	0.05	ptsI	2	24.6667	0.483
3.2	0.05	clcB	2	24.6667	0.229
3.2	0.05	ycaM	2	24.6667	0.32
3.2	0.05	yadI	2	24.6667	0.878
3.2	0.05	AG1	2	24.6667	0.739
3.2	0.05	ptsI	2	24.9167	0.483
3.2	0.05	clcB	2	24.9167	0.226
3.2	0.05	ycaM	2	24.9167	0.319
3.2	0.05	yadI	2	24.9167	0.876
3.2	0.05	AG1	2	24.9167	0.744
3.2	0.05	ptsI	2	25.1667	0.489
3.2	0.05	clcB	2	25.1667	0.226
3.2	0.05	ycaM	2	25.1667	0.316
3.2	0.05	yadI	2	25.1667	0.875
3.2	0.05	AG1	2	25.1667	0.752
3.2	0.05	ptsI	2	25.4167	0.488
3.2	0.05	clcB	2	25.4167	0.226
3.2	0.05	ycaM	2	25.4167	0.317
3.2	0.05	yadI	2	25.4167	0.875
3.2	0.05	AG1	2	25.4167	0.761
3.2	0.05	ptsI	2	25.6667	0.491
3.2	0.05	clcB	2	25.6667	0.225
3.2	0.05	ycaM	2	25.6667	0.314
3.2	0.05	yadI	2	25.6667	0.877
3.2	0.05	AG1	2	25.6667	0.764
3.2	0.05	ptsI	2	25.9167	0.493
3.2	0.05	clcB	2	25.9167	0.225
3.2	0.05	ycaM	2	25.9167	0.312
3.2	0.05	yadI	2	25.9167	0.885
3.2	0.05	AG1	2	25.9167	0.771
3.2	0.05	ptsI	2	26.1667	0.494

3.2	0.05	clcB	2	26.1667	0.224
3.2	0.05	ycaM	2	26.1667	0.305
3.2	0.05	yadI	2	26.1667	0.916
3.2	0.05	AG1	2	26.1667	0.777
3.2	0.05	ptsI	2	26.4167	0.495
3.2	0.05	clcB	2	26.4167	0.223
3.2	0.05	ycaM	2	26.4167	0.31
3.2	0.05	yadI	2	26.4167	0.89
3.2	0.05	AG1	2	26.4167	0.789
3.2	0.05	ptsI	3	0	0.168
3.2	0.05	clcB	3	0	0.161
3.2	0.05	ycaM	3	0	0.158
3.2	0.05	yadI	3	0	0.164
3.2	0.05	AG1	3	0	0.165
3.2	0.05	ptsI	3	0.25	0.156
3.2	0.05	clcB	3	0.25	0.159
3.2	0.05	ycaM	3	0.25	0.154
3.2	0.05	yadI	3	0.25	0.163
3.2	0.05	AG1	3	0.25	0.165
3.2	0.05	ptsI	3	0.5	0.155
3.2	0.05	clcB	3	0.5	0.158
3.2	0.05	ycaM	3	0.5	0.153
3.2	0.05	yadI	3	0.5	0.161
3.2	0.05	AG1	3	0.5	0.163
3.2	0.05	ptsI	3	0.75	0.155
3.2	0.05	clcB	3	0.75	0.158
3.2	0.05	ycaM	3	0.75	0.152
3.2	0.05	yadI	3	0.75	0.159
3.2	0.05	AG1	3	0.75	0.161
3.2	0.05	ptsI	3	1	0.157
3.2	0.05	clcB	3	1	0.158
3.2	0.05	ycaM	3	1	0.154
3.2	0.05	yadI	3	1	0.16
3.2	0.05	AG1	3	1	0.163
3.2	0.05	ptsI	3	1.25	0.157
3.2	0.05	clcB	3	1.25	0.158
3.2	0.05	ycaM	3	1.25	0.154
3.2	0.05	yadI	3	1.25	0.16
3.2	0.05	AG1	3	1.25	0.162
3.2	0.05	ptsI	3	1.5	0.159
3.2	0.05	clcB	3	1.5	0.157
3.2	0.05	ycaM	3	1.5	0.154
3.2	0.05	yadI	3	1.5	0.16
3.2	0.05	AG1	3	1.5	0.162
3.2	0.05	ptsI	3	1.75	0.16
3.2	0.05	clcB	3	1.75	0.158
3.2	0.05	ycaM	3	1.75	0.154
3.2	0.05	yadI	3	1.75	0.161
3.2	0.05	AG1	3	1.75	0.163
3.2	0.05	ptsI	3	2	0.161
3.2	0.05	clcB	3	2	0.159
3.2	0.05	ycaM	3	2	0.156
3.2	0.05	yadI	3	2	0.163

3.2	0.05	AG1	3	2	0.164
3.2	0.05	ptsI	3	2.25	0.163
3.2	0.05	clcB	3	2.25	0.16
3.2	0.05	ycaM	3	2.25	0.158
3.2	0.05	yadI	3	2.25	0.165
3.2	0.05	AG1	3	2.25	0.166
3.2	0.05	ptsI	3	2.5	0.165
3.2	0.05	clcB	3	2.5	0.163
3.2	0.05	ycaM	3	2.5	0.159
3.2	0.05	yadI	3	2.5	0.166
3.2	0.05	AG1	3	2.5	0.168
3.2	0.05	ptsI	3	2.75	0.172
3.2	0.05	clcB	3	2.75	0.165
3.2	0.05	ycaM	3	2.75	0.163
3.2	0.05	yadI	3	2.75	0.17
3.2	0.05	AG1	3	2.75	0.173
3.2	0.05	ptsI	3	3	0.167
3.2	0.05	clcB	3	3	0.165
3.2	0.05	ycaM	3	3	0.162
3.2	0.05	yadI	3	3	0.172
3.2	0.05	AG1	3	3	0.173
3.2	0.05	ptsI	3	3.25	0.17
3.2	0.05	clcB	3	3.25	0.166
3.2	0.05	ycaM	3	3.25	0.164
3.2	0.05	yadI	3	3.25	0.175
3.2	0.05	AG1	3	3.25	0.174
3.2	0.05	ptsI	3	3.5	0.176
3.2	0.05	clcB	3	3.5	0.169
3.2	0.05	ycaM	3	3.5	0.168
3.2	0.05	yadI	3	3.5	0.18
3.2	0.05	AG1	3	3.5	0.177
3.2	0.05	ptsI	3	3.75	0.18
3.2	0.05	clcB	3	3.75	0.171
3.2	0.05	ycaM	3	3.75	0.171
3.2	0.05	yadI	3	3.75	0.184
3.2	0.05	AG1	3	3.75	0.18
3.2	0.05	ptsI	3	4	0.184
3.2	0.05	clcB	3	4	0.174
3.2	0.05	ycaM	3	4	0.174
3.2	0.05	yadI	3	4	0.191
3.2	0.05	AG1	3	4	0.183
3.2	0.05	ptsI	3	4.41667	0.198
3.2	0.05	clcB	3	4.41667	0.2
3.2	0.05	ycaM	3	4.41667	0.174
3.2	0.05	yadI	3	4.41667	0.268
3.2	0.05	AG1	3	4.41667	0.199
3.2	0.05	ptsI	3	4.66667	0.188
3.2	0.05	clcB	3	4.66667	0.187
3.2	0.05	ycaM	3	4.66667	0.17
3.2	0.05	yadI	3	4.66667	0.27
3.2	0.05	AG1	3	4.66667	0.199
3.2	0.05	ptsI	3	4.91667	0.193
3.2	0.05	clcB	3	4.91667	0.189

3.2	0.05	ycaM	3	4.91667	0.174
3.2	0.05	yadI	3	4.91667	0.282
3.2	0.05	AG1	3	4.91667	0.207
3.2	0.05	ptsI	3	5.16667	0.195
3.2	0.05	clcB	3	5.16667	0.194
3.2	0.05	ycaM	3	5.16667	0.178
3.2	0.05	yadI	3	5.16667	0.294
3.2	0.05	AG1	3	5.16667	0.214
3.2	0.05	ptsI	3	5.41667	0.198
3.2	0.05	clcB	3	5.41667	0.2
3.2	0.05	ycaM	3	5.41667	0.184
3.2	0.05	yadI	3	5.41667	0.307
3.2	0.05	AG1	3	5.41667	0.221
3.2	0.05	ptsI	3	5.66667	0.204
3.2	0.05	clcB	3	5.66667	0.206
3.2	0.05	ycaM	3	5.66667	0.191
3.2	0.05	yadI	3	5.66667	0.321
3.2	0.05	AG1	3	5.66667	0.23
3.2	0.05	ptsI	3	5.91667	0.208
3.2	0.05	clcB	3	5.91667	0.205
3.2	0.05	ycaM	3	5.91667	0.197
3.2	0.05	yadI	3	5.91667	0.33
3.2	0.05	AG1	3	5.91667	0.235
3.2	0.05	ptsI	3	6.16667	0.215
3.2	0.05	clcB	3	6.16667	0.214
3.2	0.05	ycaM	3	6.16667	0.204
3.2	0.05	yadI	3	6.16667	0.336
3.2	0.05	AG1	3	6.16667	0.247
3.2	0.05	ptsI	3	6.41667	0.223
3.2	0.05	clcB	3	6.41667	0.223
3.2	0.05	ycaM	3	6.41667	0.215
3.2	0.05	yadI	3	6.41667	0.347
3.2	0.05	AG1	3	6.41667	0.254
3.2	0.05	ptsI	3	6.66667	0.234
3.2	0.05	clcB	3	6.66667	0.235
3.2	0.05	ycaM	3	6.66667	0.223
3.2	0.05	yadI	3	6.66667	0.357
3.2	0.05	AG1	3	6.66667	0.262
3.2	0.05	ptsI	3	6.91667	0.24
3.2	0.05	clcB	3	6.91667	0.245
3.2	0.05	ycaM	3	6.91667	0.23
3.2	0.05	yadI	3	6.91667	0.368
3.2	0.05	AG1	3	6.91667	0.267
3.2	0.05	ptsI	3	7.16667	0.25
3.2	0.05	clcB	3	7.16667	0.26
3.2	0.05	ycaM	3	7.16667	0.235
3.2	0.05	yadI	3	7.16667	0.379
3.2	0.05	AG1	3	7.16667	0.274
3.2	0.05	ptsI	3	7.41667	0.258
3.2	0.05	clcB	3	7.41667	0.273
3.2	0.05	ycaM	3	7.41667	0.239
3.2	0.05	yadI	3	7.41667	0.391
3.2	0.05	AG1	3	7.41667	0.282

3.2	0.05	ptsI	3	7.66667	0.266
3.2	0.05	clcB	3	7.66667	0.284
3.2	0.05	ycaM	3	7.66667	0.243
3.2	0.05	yadI	3	7.66667	0.403
3.2	0.05	AG1	3	7.66667	0.289
3.2	0.05	ptsI	3	7.91667	0.276
3.2	0.05	clcB	3	7.91667	0.291
3.2	0.05	ycaM	3	7.91667	0.246
3.2	0.05	yadI	3	7.91667	0.417
3.2	0.05	AG1	3	7.91667	0.297
3.2	0.05	ptsI	3	8.16667	0.288
3.2	0.05	clcB	3	8.16667	0.299
3.2	0.05	ycaM	3	8.16667	0.249
3.2	0.05	yadI	3	8.16667	0.425
3.2	0.05	AG1	3	8.16667	0.304
3.2	0.05	ptsI	3	8.41667	0.295
3.2	0.05	clcB	3	8.41667	0.308
3.2	0.05	ycaM	3	8.41667	0.252
3.2	0.05	yadI	3	8.41667	0.441
3.2	0.05	AG1	3	8.41667	0.31
3.2	0.05	ptsI	3	9.05	0.315
3.2	0.05	clcB	3	9.05	0.29
3.2	0.05	ycaM	3	9.05	0.257
3.2	0.05	yadI	3	9.05	0.428
3.2	0.05	AG1	3	9.05	0.354
3.2	0.05	ptsI	3	9.3	0.315
3.2	0.05	clcB	3	9.3	0.283
3.2	0.05	ycaM	3	9.3	0.252
3.2	0.05	yadI	3	9.3	0.446
3.2	0.05	AG1	3	9.3	0.363
3.2	0.05	ptsI	3	9.55	0.309
3.2	0.05	clcB	3	9.55	0.279
3.2	0.05	ycaM	3	9.55	0.25
3.2	0.05	yadI	3	9.55	0.461
3.2	0.05	AG1	3	9.55	0.371
3.2	0.05	ptsI	3	9.8	0.308
3.2	0.05	clcB	3	9.8	0.276
3.2	0.05	ycaM	3	9.8	0.25
3.2	0.05	yadI	3	9.8	0.466
3.2	0.05	AG1	3	9.8	0.367
3.2	0.05	ptsI	3	10.05	0.303
3.2	0.05	clcB	3	10.05	0.278
3.2	0.05	ycaM	3	10.05	0.249
3.2	0.05	yadI	3	10.05	0.47
3.2	0.05	AG1	3	10.05	0.369
3.2	0.05	ptsI	3	10.3	0.302
3.2	0.05	clcB	3	10.3	0.276
3.2	0.05	ycaM	3	10.3	0.246
3.2	0.05	yadI	3	10.3	0.477
3.2	0.05	AG1	3	10.3	0.368
3.2	0.05	ptsI	3	10.55	0.293
3.2	0.05	clcB	3	10.55	0.273
3.2	0.05	ycaM	3	10.55	0.241

3.2	0.05	yadI	3	10.55	0.484
3.2	0.05	AG1	3	10.55	0.371
3.2	0.05	ptsI	3	10.8	0.286
3.2	0.05	clcB	3	10.8	0.275
3.2	0.05	ycaM	3	10.8	0.242
3.2	0.05	yadI	3	10.8	0.493
3.2	0.05	AG1	3	10.8	0.375
3.2	0.05	ptsI	3	11.05	0.284
3.2	0.05	clcB	3	11.05	0.276
3.2	0.05	ycaM	3	11.05	0.242
3.2	0.05	yadI	3	11.05	0.492
3.2	0.05	AG1	3	11.05	0.372
3.2	0.05	ptsI	3	11.3	0.281
3.2	0.05	clcB	3	11.3	0.274
3.2	0.05	ycaM	3	11.3	0.245
3.2	0.05	yadI	3	11.3	0.496
3.2	0.05	AG1	3	11.3	0.376
3.2	0.05	ptsI	3	11.55	0.28
3.2	0.05	clcB	3	11.55	0.27
3.2	0.05	ycaM	3	11.55	0.247
3.2	0.05	yadI	3	11.55	0.498
3.2	0.05	AG1	3	11.55	0.375
3.2	0.05	ptsI	3	11.8	0.273
3.2	0.05	clcB	3	11.8	0.27
3.2	0.05	ycaM	3	11.8	0.245
3.2	0.05	yadI	3	11.8	0.505
3.2	0.05	AG1	3	11.8	0.376
3.2	0.05	ptsI	3	12.05	0.27
3.2	0.05	clcB	3	12.05	0.27
3.2	0.05	ycaM	3	12.05	0.241
3.2	0.05	yadI	3	12.05	0.505
3.2	0.05	AG1	3	12.05	0.377
3.2	0.05	ptsI	3	12.3	0.263
3.2	0.05	clcB	3	12.3	0.267
3.2	0.05	ycaM	3	12.3	0.244
3.2	0.05	yadI	3	12.3	0.503
3.2	0.05	AG1	3	12.3	0.379
3.2	0.05	ptsI	3	12.55	0.26
3.2	0.05	clcB	3	12.55	0.271
3.2	0.05	ycaM	3	12.55	0.242
3.2	0.05	yadI	3	12.55	0.51
3.2	0.05	AG1	3	12.55	0.378
3.2	0.05	ptsI	3	12.8	0.26
3.2	0.05	clcB	3	12.8	0.268
3.2	0.05	ycaM	3	12.8	0.241
3.2	0.05	yadI	3	12.8	0.513
3.2	0.05	AG1	3	12.8	0.375
3.2	0.05	ptsI	3	13.05	0.257
3.2	0.05	clcB	3	13.05	0.266
3.2	0.05	ycaM	3	13.05	0.242
3.2	0.05	yadI	3	13.05	0.516
3.2	0.05	AG1	3	13.05	0.375
3.2	0.05	ptsI	3	13.3	0.256

3.2	0.05	clcB	3	13.3	0.266
3.2	0.05	ycaM	3	13.3	0.241
3.2	0.05	yadI	3	13.3	0.52
3.2	0.05	AG1	3	13.3	0.382
3.2	0.05	ptsI	3	13.55	0.256
3.2	0.05	clcB	3	13.55	0.264
3.2	0.05	ycaM	3	13.55	0.24
3.2	0.05	yadI	3	13.55	0.524
3.2	0.05	AG1	3	13.55	0.379
3.2	0.05	ptsI	3	13.8	0.254
3.2	0.05	clcB	3	13.8	0.262
3.2	0.05	ycaM	3	13.8	0.239
3.2	0.05	yadI	3	13.8	0.524
3.2	0.05	AG1	3	13.8	0.382
3.2	0.05	ptsI	3	14.05	0.253
3.2	0.05	clcB	3	14.05	0.262
3.2	0.05	ycaM	3	14.05	0.238
3.2	0.05	yadI	3	14.05	0.531
3.2	0.05	AG1	3	14.05	0.382
3.2	0.05	ptsI	3	14.3	0.252
3.2	0.05	clcB	3	14.3	0.262
3.2	0.05	ycaM	3	14.3	0.236
3.2	0.05	yadI	3	14.3	0.534
3.2	0.05	AG1	3	14.3	0.384
3.2	0.05	ptsI	3	14.55	0.252
3.2	0.05	clcB	3	14.55	0.259
3.2	0.05	ycaM	3	14.55	0.238
3.2	0.05	yadI	3	14.55	0.541
3.2	0.05	AG1	3	14.55	0.384
3.2	0.05	ptsI	3	14.8	0.25
3.2	0.05	clcB	3	14.8	0.259
3.2	0.05	ycaM	3	14.8	0.236
3.2	0.05	yadI	3	14.8	0.547
3.2	0.05	AG1	3	14.8	0.387
3.2	0.05	ptsI	3	15.05	0.249
3.2	0.05	clcB	3	15.05	0.26
3.2	0.05	ycaM	3	15.05	0.235
3.2	0.05	yadI	3	15.05	0.551
3.2	0.05	AG1	3	15.05	0.389
3.2	0.05	ptsI	3	15.3	0.25
3.2	0.05	clcB	3	15.3	0.259
3.2	0.05	ycaM	3	15.3	0.234
3.2	0.05	yadI	3	15.3	0.556
3.2	0.05	AG1	3	15.3	0.389
3.2	0.05	ptsI	3	15.55	0.247
3.2	0.05	clcB	3	15.55	0.258
3.2	0.05	ycaM	3	15.55	0.233
3.2	0.05	yadI	3	15.55	0.556
3.2	0.05	AG1	3	15.55	0.389
3.2	0.05	ptsI	3	15.8	0.247
3.2	0.05	clcB	3	15.8	0.257
3.2	0.05	ycaM	3	15.8	0.232
3.2	0.05	yadI	3	15.8	0.561

3.2	0.05	AG1	3	15.8	0.389
3.2	0.05	ptsI	3	16.05	0.247
3.2	0.05	clcB	3	16.05	0.256
3.2	0.05	ycaM	3	16.05	0.234
3.2	0.05	yadI	3	16.05	0.564
3.2	0.05	AG1	3	16.05	0.39
3.2	0.05	ptsI	3	16.3	0.242
3.2	0.05	clcB	3	16.3	0.256
3.2	0.05	ycaM	3	16.3	0.229
3.2	0.05	yadI	3	16.3	0.567
3.2	0.05	AG1	3	16.3	0.389
3.2	0.05	ptsI	3	16.55	0.245
3.2	0.05	clcB	3	16.55	0.255
3.2	0.05	ycaM	3	16.55	0.228
3.2	0.05	yadI	3	16.55	0.571
3.2	0.05	AG1	3	16.55	0.391
3.2	0.05	ptsI	3	16.8	0.243
3.2	0.05	clcB	3	16.8	0.255
3.2	0.05	ycaM	3	16.8	0.228
3.2	0.05	yadI	3	16.8	0.573
3.2	0.05	AG1	3	16.8	0.392
3.2	0.05	ptsI	3	17.05	0.242
3.2	0.05	clcB	3	17.05	0.254
3.2	0.05	ycaM	3	17.05	0.227
3.2	0.05	yadI	3	17.05	0.578
3.2	0.05	AG1	3	17.05	0.394
3.2	0.05	ptsI	3	17.3	0.242
3.2	0.05	clcB	3	17.3	0.253
3.2	0.05	ycaM	3	17.3	0.228
3.2	0.05	yadI	3	17.3	0.584
3.2	0.05	AG1	3	17.3	0.398
3.2	0.05	ptsI	3	17.55	0.24
3.2	0.05	clcB	3	17.55	0.253
3.2	0.05	ycaM	3	17.55	0.226
3.2	0.05	yadI	3	17.55	0.586
3.2	0.05	AG1	3	17.55	0.398
3.2	0.05	ptsI	3	17.8	0.241
3.2	0.05	clcB	3	17.8	0.25
3.2	0.05	ycaM	3	17.8	0.225
3.2	0.05	yadI	3	17.8	0.591
3.2	0.05	AG1	3	17.8	0.403
3.2	0.05	ptsI	3	18.05	0.241
3.2	0.05	clcB	3	18.05	0.249
3.2	0.05	ycaM	3	18.05	0.224
3.2	0.05	yadI	3	18.05	0.595
3.2	0.05	AG1	3	18.05	0.403
3.2	0.05	ptsI	3	18.3	0.24
3.2	0.05	clcB	3	18.3	0.249
3.2	0.05	ycaM	3	18.3	0.223
3.2	0.05	yadI	3	18.3	0.603
3.2	0.05	AG1	3	18.3	0.406
3.2	0.05	ptsI	3	18.55	0.24
3.2	0.05	clcB	3	18.55	0.248

3.2	0.05	ycaM	3	18.55	0.223
3.2	0.05	yadI	3	18.55	0.603
3.2	0.05	AG1	3	18.55	0.41
3.2	0.05	ptsI	3	18.8	0.24
3.2	0.05	clcB	3	18.8	0.246
3.2	0.05	ycaM	3	18.8	0.222
3.2	0.05	yadI	3	18.8	0.611
3.2	0.05	AG1	3	18.8	0.406
3.2	0.05	ptsI	3	19.05	0.238
3.2	0.05	clcB	3	19.05	0.247
3.2	0.05	ycaM	3	19.05	0.22
3.2	0.05	yadI	3	19.05	0.61
3.2	0.05	AG1	3	19.05	0.408
3.2	0.05	ptsI	3	19.3	0.238
3.2	0.05	clcB	3	19.3	0.245
3.2	0.05	ycaM	3	19.3	0.22
3.2	0.05	yadI	3	19.3	0.616
3.2	0.05	AG1	3	19.3	0.413
3.2	0.05	ptsI	3	19.55	0.239
3.2	0.05	clcB	3	19.55	0.243
3.2	0.05	ycaM	3	19.55	0.219
3.2	0.05	yadI	3	19.55	0.619
3.2	0.05	AG1	3	19.55	0.413
3.2	0.05	ptsI	3	19.8	0.237
3.2	0.05	clcB	3	19.8	0.243
3.2	0.05	ycaM	3	19.8	0.218
3.2	0.05	yadI	3	19.8	0.625
3.2	0.05	AG1	3	19.8	0.414
3.2	0.05	ptsI	3	20.05	0.239
3.2	0.05	clcB	3	20.05	0.242
3.2	0.05	ycaM	3	20.05	0.217
3.2	0.05	yadI	3	20.05	0.632
3.2	0.05	AG1	3	20.05	0.417
3.2	0.05	ptsI	3	20.3	0.237
3.2	0.05	clcB	3	20.3	0.242
3.2	0.05	ycaM	3	20.3	0.216
3.2	0.05	yadI	3	20.3	0.634
3.2	0.05	AG1	3	20.3	0.418
3.2	0.05	ptsI	3	20.55	0.238
3.2	0.05	clcB	3	20.55	0.242
3.2	0.05	ycaM	3	20.55	0.216
3.2	0.05	yadI	3	20.55	0.639
3.2	0.05	AG1	3	20.55	0.422
3.2	0.05	ptsI	3	20.8	0.238
3.2	0.05	clcB	3	20.8	0.24
3.2	0.05	ycaM	3	20.8	0.215
3.2	0.05	yadI	3	20.8	0.644
3.2	0.05	AG1	3	20.8	0.422
3.2	0.05	ptsI	3	21.05	0.237
3.2	0.05	clcB	3	21.05	0.239
3.2	0.05	ycaM	3	21.05	0.213
3.2	0.05	yadI	3	21.05	0.645
3.2	0.05	AG1	3	21.05	0.423

3.2	0.05	ptsI	3	21.3	0.236
3.2	0.05	clcB	3	21.3	0.238
3.2	0.05	ycaM	3	21.3	0.212
3.2	0.05	yadI	3	21.3	0.65
3.2	0.05	AG1	3	21.3	0.425
3.2	0.05	ptsI	3	21.55	0.238
3.2	0.05	clcB	3	21.55	0.237
3.2	0.05	ycaM	3	21.55	0.211
3.2	0.05	yadI	3	21.55	0.652
3.2	0.05	AG1	3	21.55	0.426
3.2	0.05	ptsI	3	21.8	0.237
3.2	0.05	clcB	3	21.8	0.237
3.2	0.05	ycaM	3	21.8	0.21
3.2	0.05	yadI	3	21.8	0.654
3.2	0.05	AG1	3	21.8	0.432
3.2	0.05	ptsI	3	22.05	0.237
3.2	0.05	clcB	3	22.05	0.236
3.2	0.05	ycaM	3	22.05	0.209
3.2	0.05	yadI	3	22.05	0.659
3.2	0.05	AG1	3	22.05	0.43
3.2	0.05	ptsI	3	22.3	0.236
3.2	0.05	clcB	3	22.3	0.235
3.2	0.05	ycaM	3	22.3	0.208
3.2	0.05	yadI	3	22.3	0.661
3.2	0.05	AG1	3	22.3	0.434
3.2	0.05	ptsI	3	22.55	0.236
3.2	0.05	clcB	3	22.55	0.234
3.2	0.05	ycaM	3	22.55	0.207
3.2	0.05	yadI	3	22.55	0.67
3.2	0.05	AG1	3	22.55	0.436
3.2	0.05	ptsI	3	22.8	0.236
3.2	0.05	clcB	3	22.8	0.232
3.2	0.05	ycaM	3	22.8	0.206
3.2	0.05	yadI	3	22.8	0.67
3.2	0.05	AG1	3	22.8	0.436
3.2	0.05	ptsI	3	23.05	0.235
3.2	0.05	clcB	3	23.05	0.232
3.2	0.05	ycaM	3	23.05	0.206
3.2	0.05	yadI	3	23.05	0.677
3.2	0.05	AG1	3	23.05	0.438
3.2	0.05	ptsI	3	23.3	0.235
3.2	0.05	clcB	3	23.3	0.231
3.2	0.05	ycaM	3	23.3	0.204
3.2	0.05	yadI	3	23.3	0.685
3.2	0.05	AG1	3	23.3	0.441
3.2	0.05	ptsI	3	23.55	0.235
3.2	0.05	clcB	3	23.55	0.229
3.2	0.05	ycaM	3	23.55	0.202
3.2	0.05	yadI	3	23.55	0.687
3.2	0.05	AG1	3	23.55	0.442
3.2	0.05	ptsI	3	23.8	0.234
3.2	0.05	clcB	3	23.8	0.229
3.2	0.05	ycaM	3	23.8	0.202

3.2	0.05	yadI	3	23.8	0.688
3.2	0.05	AG1	3	23.8	0.444
3.2	0.05	ptsI	3	24.05	0.235
3.2	0.05	clcB	3	24.05	0.227
3.2	0.05	ycaM	3	24.05	0.201
3.2	0.05	yadI	3	24.05	0.697
3.2	0.05	AG1	3	24.05	0.445
3.2	0.05	ptsI	3	24.3	0.231
3.2	0.05	clcB	3	24.3	0.229
3.2	0.05	ycaM	3	24.3	0.199
3.2	0.05	yadI	3	24.3	0.699
3.2	0.05	AG1	3	24.3	0.443
3.2	0.05	ptsI	4	0	0.208
3.2	0.05	clcB	4	0	0.19
3.2	0.05	ycaM	4	0	0.223
3.2	0.05	yadI	4	0	0.221
3.2	0.05	AG1	4	0	0.203
3.2	0.05	ptsI	4	0.25	0.207
3.2	0.05	clcB	4	0.25	0.19
3.2	0.05	ycaM	4	0.25	0.209
3.2	0.05	yadI	4	0.25	0.222
3.2	0.05	AG1	4	0.25	0.199
3.2	0.05	ptsI	4	0.5	0.208
3.2	0.05	clcB	4	0.5	0.192
3.2	0.05	ycaM	4	0.5	0.215
3.2	0.05	yadI	4	0.5	0.224
3.2	0.05	AG1	4	0.5	0.202
3.2	0.05	ptsI	4	0.75	0.212
3.2	0.05	clcB	4	0.75	0.193
3.2	0.05	ycaM	4	0.75	0.215
3.2	0.05	yadI	4	0.75	0.228
3.2	0.05	AG1	4	0.75	0.204
3.2	0.05	ptsI	4	1	0.216
3.2	0.05	clcB	4	1	0.195
3.2	0.05	ycaM	4	1	0.22
3.2	0.05	yadI	4	1	0.23
3.2	0.05	AG1	4	1	0.21
3.2	0.05	ptsI	4	1.25	0.218
3.2	0.05	clcB	4	1.25	0.201
3.2	0.05	ycaM	4	1.25	0.225
3.2	0.05	yadI	4	1.25	0.234
3.2	0.05	AG1	4	1.25	0.215
3.2	0.05	ptsI	4	1.5	0.218
3.2	0.05	clcB	4	1.5	0.199
3.2	0.05	ycaM	4	1.5	0.221
3.2	0.05	yadI	4	1.5	0.233
3.2	0.05	AG1	4	1.5	0.208
3.2	0.05	ptsI	4	1.75	0.225
3.2	0.05	clcB	4	1.75	0.207
3.2	0.05	ycaM	4	1.75	0.23
3.2	0.05	yadI	4	1.75	0.242
3.2	0.05	AG1	4	1.75	0.215
3.2	0.05	ptsI	4	2	0.234

3.2	0.05	clcB	4	2	0.213
3.2	0.05	ycaM	4	2	0.239
3.2	0.05	yadI	4	2	0.25
3.2	0.05	AG1	4	2	0.223
3.2	0.05	ptsI	4	2.25	0.242
3.2	0.05	clcB	4	2.25	0.225
3.2	0.05	ycaM	4	2.25	0.252
3.2	0.05	yadI	4	2.25	0.261
3.2	0.05	AG1	4	2.25	0.233
3.2	0.05	ptsI	4	2.5	0.246
3.2	0.05	clcB	4	2.5	0.225
3.2	0.05	ycaM	4	2.5	0.25
3.2	0.05	yadI	4	2.5	0.258
3.2	0.05	AG1	4	2.5	0.23
3.2	0.05	ptsI	4	2.75	0.254
3.2	0.05	clcB	4	2.75	0.234
3.2	0.05	ycaM	4	2.75	0.255
3.2	0.05	yadI	4	2.75	0.265
3.2	0.05	AG1	4	2.75	0.237
3.2	0.05	ptsI	4	3	0.263
3.2	0.05	clcB	4	3	0.247
3.2	0.05	ycaM	4	3	0.274
3.2	0.05	yadI	4	3	0.276
3.2	0.05	AG1	4	3	0.244
3.2	0.05	ptsI	4	3.25	0.274
3.2	0.05	clcB	4	3.25	0.261
3.2	0.05	ycaM	4	3.25	0.284
3.2	0.05	yadI	4	3.25	0.286
3.2	0.05	AG1	4	3.25	0.254
3.2	0.05	ptsI	4	3.5	0.288
3.2	0.05	clcB	4	3.5	0.277
3.2	0.05	ycaM	4	3.5	0.301
3.2	0.05	yadI	4	3.5	0.301
3.2	0.05	AG1	4	3.5	0.266
3.2	0.05	ptsI	4	3.75	0.296
3.2	0.05	clcB	4	3.75	0.291
3.2	0.05	ycaM	4	3.75	0.311
3.2	0.05	yadI	4	3.75	0.308
3.2	0.05	AG1	4	3.75	0.276
3.2	0.05	ptsI	4	4	0.314
3.2	0.05	clcB	4	4	0.316
3.2	0.05	ycaM	4	4	0.341
3.2	0.05	yadI	4	4	0.32
3.2	0.05	AG1	4	4	0.287
3.2	0.05	ptsI	4	4.35	0.327
3.2	0.05	clcB	4	4.35	0.322
3.2	0.05	ycaM	4	4.35	0.338
3.2	0.05	yadI	4	4.35	0.366
3.2	0.05	AG1	4	4.35	0.262
3.2	0.05	ptsI	4	4.6	0.331
3.2	0.05	clcB	4	4.6	0.328
3.2	0.05	ycaM	4	4.6	0.341
3.2	0.05	yadI	4	4.6	0.383

3.2	0.05	AG1	4	4.6	0.277
3.2	0.05	ptsI	4	4.85	0.341
3.2	0.05	clcB	4	4.85	0.329
3.2	0.05	ycaM	4	4.85	0.339
3.2	0.05	yadI	4	4.85	0.402
3.2	0.05	AG1	4	4.85	0.29
3.2	0.05	ptsI	4	5.1	0.351
3.2	0.05	clcB	4	5.1	0.33
3.2	0.05	ycaM	4	5.1	0.338
3.2	0.05	yadI	4	5.1	0.411
3.2	0.05	AG1	4	5.1	0.297
3.2	0.05	ptsI	4	5.35	0.363
3.2	0.05	clcB	4	5.35	0.336
3.2	0.05	ycaM	4	5.35	0.344
3.2	0.05	yadI	4	5.35	0.427
3.2	0.05	AG1	4	5.35	0.307
3.2	0.05	ptsI	4	5.6	0.368
3.2	0.05	clcB	4	5.6	0.33
3.2	0.05	ycaM	4	5.6	0.338
3.2	0.05	yadI	4	5.6	0.457
3.2	0.05	AG1	4	5.6	0.312
3.2	0.05	ptsI	4	5.85	0.371
3.2	0.05	clcB	4	5.85	0.32
3.2	0.05	ycaM	4	5.85	0.331
3.2	0.05	yadI	4	5.85	0.446
3.2	0.05	AG1	4	5.85	0.317
3.2	0.05	ptsI	4	6.1	0.388
3.2	0.05	clcB	4	6.1	0.324
3.2	0.05	ycaM	4	6.1	0.336
3.2	0.05	yadI	4	6.1	0.46
3.2	0.05	AG1	4	6.1	0.328
3.2	0.05	ptsI	4	6.35	0.404
3.2	0.05	clcB	4	6.35	0.33
3.2	0.05	ycaM	4	6.35	0.337
3.2	0.05	yadI	4	6.35	0.482
3.2	0.05	AG1	4	6.35	0.338
3.2	0.05	ptsI	4	6.6	0.417
3.2	0.05	clcB	4	6.6	0.336
3.2	0.05	ycaM	4	6.6	0.342
3.2	0.05	yadI	4	6.6	0.474
3.2	0.05	AG1	4	6.6	0.347
3.2	0.05	ptsI	4	6.85	0.429
3.2	0.05	clcB	4	6.85	0.339
3.2	0.05	ycaM	4	6.85	0.345
3.2	0.05	yadI	4	6.85	0.482
3.2	0.05	AG1	4	6.85	0.355
3.2	0.05	ptsI	4	7.1	0.441
3.2	0.05	clcB	4	7.1	0.343
3.2	0.05	ycaM	4	7.1	0.346
3.2	0.05	yadI	4	7.1	0.489
3.2	0.05	AG1	4	7.1	0.363
3.2	0.05	ptsI	4	7.35	0.45
3.2	0.05	clcB	4	7.35	0.348

3.2	0.05	ycaM	4	7.35	0.352
3.2	0.05	yadI	4	7.35	0.499
3.2	0.05	AG1	4	7.35	0.372
3.2	0.05	ptsI	4	7.6	0.46
3.2	0.05	clcB	4	7.6	0.35
3.2	0.05	ycaM	4	7.6	0.37
3.2	0.05	yadI	4	7.6	0.506
3.2	0.05	AG1	4	7.6	0.379
3.2	0.05	ptsI	4	7.85	0.459
3.2	0.05	clcB	4	7.85	0.347
3.2	0.05	ycaM	4	7.85	0.338
3.2	0.05	yadI	4	7.85	0.507
3.2	0.05	AG1	4	7.85	0.381
3.2	0.05	ptsI	4	8.1	0.474
3.2	0.05	clcB	4	8.1	0.354
3.2	0.05	ycaM	4	8.1	0.346
3.2	0.05	yadI	4	8.1	0.524
3.2	0.05	AG1	4	8.1	0.39
3.2	0.05	ptsI	4	8.35	0.487
3.2	0.05	clcB	4	8.35	0.358
3.2	0.05	ycaM	4	8.35	0.357
3.2	0.05	yadI	4	8.35	0.526
3.2	0.05	AG1	4	8.35	0.4
3.2	0.05	ptsI	4	8.83333	0.424
3.2	0.05	clcB	4	8.83333	0.304
3.2	0.05	ycaM	4	8.83333	0.306
3.2	0.05	yadI	4	8.83333	0.511
3.2	0.05	AG1	4	8.83333	0.439
3.2	0.05	ptsI	4	9.08333	0.434
3.2	0.05	clcB	4	9.08333	0.303
3.2	0.05	ycaM	4	9.08333	0.305
3.2	0.05	yadI	4	9.08333	0.522
3.2	0.05	AG1	4	9.08333	0.468
3.2	0.05	ptsI	4	9.33333	0.446
3.2	0.05	clcB	4	9.33333	0.3
3.2	0.05	ycaM	4	9.33333	0.304
3.2	0.05	yadI	4	9.33333	0.539
3.2	0.05	AG1	4	9.33333	0.488
3.2	0.05	ptsI	4	9.58333	0.452
3.2	0.05	clcB	4	9.58333	0.301
3.2	0.05	ycaM	4	9.58333	0.307
3.2	0.05	yadI	4	9.58333	0.561
3.2	0.05	AG1	4	9.58333	0.492
3.2	0.05	ptsI	4	9.83333	0.452
3.2	0.05	clcB	4	9.83333	0.299
3.2	0.05	ycaM	4	9.83333	0.306
3.2	0.05	yadI	4	9.83333	0.572
3.2	0.05	AG1	4	9.83333	0.494
3.2	0.05	ptsI	4	10.0833	0.451
3.2	0.05	clcB	4	10.0833	0.298
3.2	0.05	ycaM	4	10.0833	0.307
3.2	0.05	yadI	4	10.0833	0.587
3.2	0.05	AG1	4	10.0833	0.5

3.2	0.05	ptsI	4	10.3333	0.455
3.2	0.05	clcB	4	10.3333	0.296
3.2	0.05	ycaM	4	10.3333	0.306
3.2	0.05	yadI	4	10.3333	0.555
3.2	0.05	AG1	4	10.3333	0.504
3.2	0.05	ptsI	4	10.5833	0.464
3.2	0.05	clcB	4	10.5833	0.297
3.2	0.05	ycaM	4	10.5833	0.304
3.2	0.05	yadI	4	10.5833	0.558
3.2	0.05	AG1	4	10.5833	0.505
3.2	0.05	ptsI	4	10.8333	0.467
3.2	0.05	clcB	4	10.8333	0.299
3.2	0.05	ycaM	4	10.8333	0.306
3.2	0.05	yadI	4	10.8333	0.578
3.2	0.05	AG1	4	10.8333	0.512
3.2	0.05	ptsI	4	11.0833	0.469
3.2	0.05	clcB	4	11.0833	0.298
3.2	0.05	ycaM	4	11.0833	0.307
3.2	0.05	yadI	4	11.0833	0.583
3.2	0.05	AG1	4	11.0833	0.518
3.2	0.05	ptsI	4	11.3333	0.475
3.2	0.05	clcB	4	11.3333	0.3
3.2	0.05	ycaM	4	11.3333	0.307
3.2	0.05	yadI	4	11.3333	0.592
3.2	0.05	AG1	4	11.3333	0.521
3.2	0.05	ptsI	4	11.5833	0.48
3.2	0.05	clcB	4	11.5833	0.3
3.2	0.05	ycaM	4	11.5833	0.308
3.2	0.05	yadI	4	11.5833	0.577
3.2	0.05	AG1	4	11.5833	0.527
3.2	0.05	ptsI	4	11.8333	0.486
3.2	0.05	clcB	4	11.8333	0.299
3.2	0.05	ycaM	4	11.8333	0.31
3.2	0.05	yadI	4	11.8333	0.583
3.2	0.05	AG1	4	11.8333	0.528
3.2	0.05	ptsI	4	12.0833	0.494
3.2	0.05	clcB	4	12.0833	0.3
3.2	0.05	ycaM	4	12.0833	0.31
3.2	0.05	yadI	4	12.0833	0.582
3.2	0.05	AG1	4	12.0833	0.532
3.2	0.05	ptsI	4	12.3333	0.496
3.2	0.05	clcB	4	12.3333	0.3
3.2	0.05	ycaM	4	12.3333	0.308
3.2	0.05	yadI	4	12.3333	0.595
3.2	0.05	AG1	4	12.3333	0.537
3.2	0.05	ptsI	4	12.5833	0.502
3.2	0.05	clcB	4	12.5833	0.302
3.2	0.05	ycaM	4	12.5833	0.308
3.2	0.05	yadI	4	12.5833	0.592
3.2	0.05	AG1	4	12.5833	0.539
3.2	0.05	ptsI	4	12.8333	0.509
3.2	0.05	clcB	4	12.8333	0.3
3.2	0.05	ycaM	4	12.8333	0.307

3.2	0.05	yadI	4	12.8333	0.618
3.2	0.05	AG1	4	12.8333	0.544
3.2	0.05	ptsI	4	13.0833	0.517
3.2	0.05	clcB	4	13.0833	0.301
3.2	0.05	ycaM	4	13.0833	0.307
3.2	0.05	yadI	4	13.0833	0.602
3.2	0.05	AG1	4	13.0833	0.546
3.2	0.05	ptsI	4	13.3333	0.519
3.2	0.05	clcB	4	13.3333	0.3
3.2	0.05	ycaM	4	13.3333	0.306
3.2	0.05	yadI	4	13.3333	0.636
3.2	0.05	AG1	4	13.3333	0.552
3.2	0.05	ptsI	4	13.5833	0.523
3.2	0.05	clcB	4	13.5833	0.301
3.2	0.05	ycaM	4	13.5833	0.306
3.2	0.05	yadI	4	13.5833	0.603
3.2	0.05	AG1	4	13.5833	0.555
3.2	0.05	ptsI	4	13.8333	0.532
3.2	0.05	clcB	4	13.8333	0.3
3.2	0.05	ycaM	4	13.8333	0.306
3.2	0.05	yadI	4	13.8333	0.61
3.2	0.05	AG1	4	13.8333	0.557
3.2	0.05	ptsI	4	14.0833	0.53
3.2	0.05	clcB	4	14.0833	0.3
3.2	0.05	ycaM	4	14.0833	0.306
3.2	0.05	yadI	4	14.0833	0.611
3.2	0.05	AG1	4	14.0833	0.561
3.2	0.05	ptsI	4	14.3333	0.539
3.2	0.05	clcB	4	14.3333	0.302
3.2	0.05	ycaM	4	14.3333	0.306
3.2	0.05	yadI	4	14.3333	0.615
3.2	0.05	AG1	4	14.3333	0.563
3.2	0.05	ptsI	4	14.5833	0.54
3.2	0.05	clcB	4	14.5833	0.301
3.2	0.05	ycaM	4	14.5833	0.305
3.2	0.05	yadI	4	14.5833	0.618
3.2	0.05	AG1	4	14.5833	0.566
3.2	0.05	ptsI	4	14.8333	0.542
3.2	0.05	clcB	4	14.8333	0.301
3.2	0.05	ycaM	4	14.8333	0.306
3.2	0.05	yadI	4	14.8333	0.625
3.2	0.05	AG1	4	14.8333	0.566
3.2	0.05	ptsI	4	15.0833	0.552
3.2	0.05	clcB	4	15.0833	0.3
3.2	0.05	ycaM	4	15.0833	0.307
3.2	0.05	yadI	4	15.0833	0.638
3.2	0.05	AG1	4	15.0833	0.573
3.2	0.05	ptsI	4	15.3333	0.553
3.2	0.05	clcB	4	15.3333	0.303
3.2	0.05	ycaM	4	15.3333	0.306
3.2	0.05	yadI	4	15.3333	0.626
3.2	0.05	AG1	4	15.3333	0.576
3.2	0.05	ptsI	4	15.5833	0.559

3.2	0.05	clcB	4	15.5833	0.302
3.2	0.05	ycaM	4	15.5833	0.305
3.2	0.05	yadI	4	15.5833	0.631
3.2	0.05	AG1	4	15.5833	0.581
3.2	0.05	ptsI	4	15.8333	0.562
3.2	0.05	clcB	4	15.8333	0.302
3.2	0.05	ycaM	4	15.8333	0.307
3.2	0.05	yadI	4	15.8333	0.634
3.2	0.05	AG1	4	15.8333	0.581
3.2	0.05	ptsI	4	16.0833	0.567
3.2	0.05	clcB	4	16.0833	0.303
3.2	0.05	ycaM	4	16.0833	0.306
3.2	0.05	yadI	4	16.0833	0.633
3.2	0.05	AG1	4	16.0833	0.585
3.2	0.05	ptsI	4	16.3333	0.572
3.2	0.05	clcB	4	16.3333	0.302
3.2	0.05	ycaM	4	16.3333	0.306
3.2	0.05	yadI	4	16.3333	0.64
3.2	0.05	AG1	4	16.3333	0.588
3.2	0.05	ptsI	4	16.5833	0.571
3.2	0.05	clcB	4	16.5833	0.302
3.2	0.05	ycaM	4	16.5833	0.306
3.2	0.05	yadI	4	16.5833	0.642
3.2	0.05	AG1	4	16.5833	0.589
3.2	0.05	ptsI	4	16.8333	0.577
3.2	0.05	clcB	4	16.8333	0.301
3.2	0.05	ycaM	4	16.8333	0.307
3.2	0.05	yadI	4	16.8333	0.644
3.2	0.05	AG1	4	16.8333	0.594
3.2	0.05	ptsI	4	17.0833	0.58
3.2	0.05	clcB	4	17.0833	0.302
3.2	0.05	ycaM	4	17.0833	0.307
3.2	0.05	yadI	4	17.0833	0.647
3.2	0.05	AG1	4	17.0833	0.595
3.2	0.05	ptsI	4	17.3333	0.586
3.2	0.05	clcB	4	17.3333	0.3
3.2	0.05	ycaM	4	17.3333	0.307
3.2	0.05	yadI	4	17.3333	0.649
3.2	0.05	AG1	4	17.3333	0.597
3.2	0.05	ptsI	4	17.5833	0.588
3.2	0.05	clcB	4	17.5833	0.301
3.2	0.05	ycaM	4	17.5833	0.305
3.2	0.05	yadI	4	17.5833	0.649
3.2	0.05	AG1	4	17.5833	0.601
3.2	0.05	ptsI	4	17.8333	0.593
3.2	0.05	clcB	4	17.8333	0.302
3.2	0.05	ycaM	4	17.8333	0.306
3.2	0.05	yadI	4	17.8333	0.65
3.2	0.05	AG1	4	17.8333	0.602
3.2	0.05	ptsI	4	18.0833	0.597
3.2	0.05	clcB	4	18.0833	0.302
3.2	0.05	ycaM	4	18.0833	0.306
3.2	0.05	yadI	4	18.0833	0.656

3.2	0.05	AG1	4	18.0833	0.604
3.2	0.05	ptsI	4	18.3333	0.599
3.2	0.05	clcB	4	18.3333	0.302
3.2	0.05	ycaM	4	18.3333	0.305
3.2	0.05	yadI	4	18.3333	0.656
3.2	0.05	AG1	4	18.3333	0.605
3.2	0.05	ptsI	4	18.5833	0.602
3.2	0.05	clcB	4	18.5833	0.301
3.2	0.05	ycaM	4	18.5833	0.305
3.2	0.05	yadI	4	18.5833	0.658
3.2	0.05	AG1	4	18.5833	0.608
3.2	0.05	ptsI	4	18.8333	0.609
3.2	0.05	clcB	4	18.8333	0.301
3.2	0.05	ycaM	4	18.8333	0.305
3.2	0.05	yadI	4	18.8333	0.66
3.2	0.05	AG1	4	18.8333	0.609
3.2	0.05	ptsI	4	19.0833	0.613
3.2	0.05	clcB	4	19.0833	0.301
3.2	0.05	ycaM	4	19.0833	0.304
3.2	0.05	yadI	4	19.0833	0.662
3.2	0.05	AG1	4	19.0833	0.613
3.2	0.05	ptsI	4	19.3333	0.618
3.2	0.05	clcB	4	19.3333	0.301
3.2	0.05	ycaM	4	19.3333	0.305
3.2	0.05	yadI	4	19.3333	0.666
3.2	0.05	AG1	4	19.3333	0.617
3.2	0.05	ptsI	4	19.5833	0.62
3.2	0.05	clcB	4	19.5833	0.302
3.2	0.05	ycaM	4	19.5833	0.305
3.2	0.05	yadI	4	19.5833	0.67
3.2	0.05	AG1	4	19.5833	0.619
3.2	0.05	ptsI	4	19.8333	0.625
3.2	0.05	clcB	4	19.8333	0.301
3.2	0.05	ycaM	4	19.8333	0.304
3.2	0.05	yadI	4	19.8333	0.672
3.2	0.05	AG1	4	19.8333	0.622
3.2	0.05	ptsI	4	20.0833	0.63
3.2	0.05	clcB	4	20.0833	0.3
3.2	0.05	ycaM	4	20.0833	0.303
3.2	0.05	yadI	4	20.0833	0.671
3.2	0.05	AG1	4	20.0833	0.62
3.2	0.05	ptsI	4	20.3333	0.631
3.2	0.05	clcB	4	20.3333	0.301
3.2	0.05	ycaM	4	20.3333	0.304
3.2	0.05	yadI	4	20.3333	0.675
3.2	0.05	AG1	4	20.3333	0.628
3.2	0.05	ptsI	4	20.5833	0.635
3.2	0.05	clcB	4	20.5833	0.301
3.2	0.05	ycaM	4	20.5833	0.304
3.2	0.05	yadI	4	20.5833	0.679
3.2	0.05	AG1	4	20.5833	0.633
3.2	0.05	ptsI	4	20.8333	0.638
3.2	0.05	clcB	4	20.8333	0.3

3.2	0.05	ycaM	4	20.8333	0.304
3.2	0.05	yadI	4	20.8333	0.682
3.2	0.05	AG1	4	20.8333	0.636
3.2	0.05	ptsI	4	21.0833	0.639
3.2	0.05	clcB	4	21.0833	0.3
3.2	0.05	ycaM	4	21.0833	0.303
3.2	0.05	yadI	4	21.0833	0.678
3.2	0.05	AG1	4	21.0833	0.638
3.2	0.05	ptsI	4	21.3333	0.643
3.2	0.05	clcB	4	21.3333	0.301
3.2	0.05	ycaM	4	21.3333	0.303
3.2	0.05	yadI	4	21.3333	0.683
3.2	0.05	AG1	4	21.3333	0.642
3.2	0.05	ptsI	4	21.5833	0.649
3.2	0.05	clcB	4	21.5833	0.299
3.2	0.05	ycaM	4	21.5833	0.302
3.2	0.05	yadI	4	21.5833	0.686
3.2	0.05	AG1	4	21.5833	0.644
3.2	0.05	ptsI	4	21.8333	0.651
3.2	0.05	clcB	4	21.8333	0.299
3.2	0.05	ycaM	4	21.8333	0.302
3.2	0.05	yadI	4	21.8333	0.689
3.2	0.05	AG1	4	21.8333	0.647
3.2	0.05	ptsI	4	22.0833	0.656
3.2	0.05	clcB	4	22.0833	0.3
3.2	0.05	ycaM	4	22.0833	0.301
3.2	0.05	yadI	4	22.0833	0.689
3.2	0.05	AG1	4	22.0833	0.648
3.2	0.05	ptsI	4	22.3333	0.661
3.2	0.05	clcB	4	22.3333	0.299
3.2	0.05	ycaM	4	22.3333	0.301
3.2	0.05	yadI	4	22.3333	0.692
3.2	0.05	AG1	4	22.3333	0.65
3.2	0.05	ptsI	4	22.5833	0.662
3.2	0.05	clcB	4	22.5833	0.298
3.2	0.05	ycaM	4	22.5833	0.3
3.2	0.05	yadI	4	22.5833	0.694
3.2	0.05	AG1	4	22.5833	0.65
3.2	0.05	ptsI	4	22.8333	0.668
3.2	0.05	clcB	4	22.8333	0.297
3.2	0.05	ycaM	4	22.8333	0.3
3.2	0.05	yadI	4	22.8333	0.694
3.2	0.05	AG1	4	22.8333	0.658
3.2	0.05	ptsI	4	23.0833	0.668
3.2	0.05	clcB	4	23.0833	0.296
3.2	0.05	ycaM	4	23.0833	0.3
3.2	0.05	yadI	4	23.0833	0.697
3.2	0.05	AG1	4	23.0833	0.656
3.2	0.05	ptsI	4	23.3333	0.674
3.2	0.05	clcB	4	23.3333	0.297
3.2	0.05	ycaM	4	23.3333	0.3
3.2	0.05	yadI	4	23.3333	0.701
3.2	0.05	AG1	4	23.3333	0.661

3.2	0.05	ptsI	4	23.5833	0.677
3.2	0.05	clcB	4	23.5833	0.296
3.2	0.05	ycaM	4	23.5833	0.3
3.2	0.05	yadI	4	23.5833	0.702
3.2	0.05	AG1	4	23.5833	0.663
3.2	0.05	ptsI	4	23.8333	0.681
3.2	0.05	clcB	4	23.8333	0.296
3.2	0.05	ycaM	4	23.8333	0.299
3.2	0.05	yadI	4	23.8333	0.703
3.2	0.05	AG1	4	23.8333	0.665
3.2	0.05	ptsI	4	24.0833	0.682
3.2	0.05	clcB	4	24.0833	0.296
3.2	0.05	ycaM	4	24.0833	0.297
3.2	0.05	yadI	4	24.0833	0.705
3.2	0.05	AG1	4	24.0833	0.667
3.2	0.05	ptsI	5	0	0.182
3.2	0.05	clcB	5	0	0.158
3.2	0.05	ycaM	5	0	0.154
3.2	0.05	yadI	5	0	0.164
3.2	0.05	AG1	5	0	0.155
3.2	0.05	ptsI	5	0.35	0.181
3.2	0.05	clcB	5	0.35	0.158
3.2	0.05	ycaM	5	0.35	0.152
3.2	0.05	yadI	5	0.35	0.163
3.2	0.05	AG1	5	0.35	0.157
3.2	0.05	ptsI	5	0.6	0.175
3.2	0.05	clcB	5	0.6	0.157
3.2	0.05	ycaM	5	0.6	0.152
3.2	0.05	yadI	5	0.6	0.165
3.2	0.05	AG1	5	0.6	0.156
3.2	0.05	ptsI	5	0.85	0.178
3.2	0.05	clcB	5	0.85	0.157
3.2	0.05	ycaM	5	0.85	0.152
3.2	0.05	yadI	5	0.85	0.165
3.2	0.05	AG1	5	0.85	0.155
3.2	0.05	ptsI	5	1.1	0.18
3.2	0.05	clcB	5	1.1	0.159
3.2	0.05	ycaM	5	1.1	0.154
3.2	0.05	yadI	5	1.1	0.168
3.2	0.05	AG1	5	1.1	0.158
3.2	0.05	ptsI	5	1.35	0.185
3.2	0.05	clcB	5	1.35	0.16
3.2	0.05	ycaM	5	1.35	0.155
3.2	0.05	yadI	5	1.35	0.169
3.2	0.05	AG1	5	1.35	0.157
3.2	0.05	ptsI	5	1.6	0.185
3.2	0.05	clcB	5	1.6	0.161
3.2	0.05	ycaM	5	1.6	0.158
3.2	0.05	yadI	5	1.6	0.171
3.2	0.05	AG1	5	1.6	0.16
3.2	0.05	ptsI	5	1.85	0.186
3.2	0.05	clcB	5	1.85	0.165
3.2	0.05	ycaM	5	1.85	0.16

3.2	0.05	yadI	5	1.85	0.174
3.2	0.05	AG1	5	1.85	0.161
3.2	0.05	ptsI	5	2.1	0.194
3.2	0.05	clcB	5	2.1	0.17
3.2	0.05	ycaM	5	2.1	0.167
3.2	0.05	yadI	5	2.1	0.18
3.2	0.05	AG1	5	2.1	0.166
3.2	0.05	ptsI	5	2.35	0.196
3.2	0.05	clcB	5	2.35	0.174
3.2	0.05	ycaM	5	2.35	0.17
3.2	0.05	yadI	5	2.35	0.186
3.2	0.05	AG1	5	2.35	0.169
3.2	0.05	ptsI	5	2.6	0.19
3.2	0.05	clcB	5	2.6	0.176
3.2	0.05	ycaM	5	2.6	0.17
3.2	0.05	yadI	5	2.6	0.185
3.2	0.05	AG1	5	2.6	0.168
3.2	0.05	ptsI	5	2.85	0.201
3.2	0.05	clcB	5	2.85	0.183
3.2	0.05	ycaM	5	2.85	0.179
3.2	0.05	yadI	5	2.85	0.194
3.2	0.05	AG1	5	2.85	0.175
3.2	0.05	ptsI	5	3.1	0.208
3.2	0.05	clcB	5	3.1	0.192
3.2	0.05	ycaM	5	3.1	0.188
3.2	0.05	yadI	5	3.1	0.202
3.2	0.05	AG1	5	3.1	0.181
3.2	0.05	ptsI	5	3.35	0.22
3.2	0.05	clcB	5	3.35	0.2
3.2	0.05	ycaM	5	3.35	0.2
3.2	0.05	yadI	5	3.35	0.217
3.2	0.05	AG1	5	3.35	0.187
3.2	0.05	ptsI	5	3.6	0.233
3.2	0.05	clcB	5	3.6	0.215
3.2	0.05	ycaM	5	3.6	0.217
3.2	0.05	yadI	5	3.6	0.239
3.2	0.05	AG1	5	3.6	0.197
3.2	0.05	ptsI	5	3.85	0.243
3.2	0.05	clcB	5	3.85	0.23
3.2	0.05	ycaM	5	3.85	0.23
3.2	0.05	yadI	5	3.85	0.269
3.2	0.05	AG1	5	3.85	0.208
3.2	0.05	ptsI	5	4.38333	0.246
3.2	0.05	clcB	5	4.38333	0.247
3.2	0.05	ycaM	5	4.38333	0.209
3.2	0.05	yadI	5	4.38333	0.294
3.2	0.05	AG1	5	4.38333	0.228
3.2	0.05	ptsI	5	4.63333	0.233
3.2	0.05	clcB	5	4.63333	0.245
3.2	0.05	ycaM	5	4.63333	0.211
3.2	0.05	yadI	5	4.63333	0.277
3.2	0.05	AG1	5	4.63333	0.216
3.2	0.05	ptsI	5	4.88333	0.239

3.2	0.05	clcB	5	4.88333	0.259
3.2	0.05	ycaM	5	4.88333	0.225
3.2	0.05	yadI	5	4.88333	0.3
3.2	0.05	AG1	5	4.88333	0.232
3.2	0.05	ptsI	5	5.13333	0.247
3.2	0.05	clcB	5	5.13333	0.269
3.2	0.05	ycaM	5	5.13333	0.234
3.2	0.05	yadI	5	5.13333	0.312
3.2	0.05	AG1	5	5.13333	0.244
3.2	0.05	ptsI	5	5.38333	0.256
3.2	0.05	clcB	5	5.38333	0.275
3.2	0.05	ycaM	5	5.38333	0.236
3.2	0.05	yadI	5	5.38333	0.327
3.2	0.05	AG1	5	5.38333	0.253
3.2	0.05	ptsI	5	5.63333	0.269
3.2	0.05	clcB	5	5.63333	0.285
3.2	0.05	ycaM	5	5.63333	0.239
3.2	0.05	yadI	5	5.63333	0.346
3.2	0.05	AG1	5	5.63333	0.263
3.2	0.05	ptsI	5	5.88333	0.283
3.2	0.05	clcB	5	5.88333	0.29
3.2	0.05	ycaM	5	5.88333	0.239
3.2	0.05	yadI	5	5.88333	0.364
3.2	0.05	AG1	5	5.88333	0.262
3.2	0.05	ptsI	5	6.13333	0.295
3.2	0.05	clcB	5	6.13333	0.301
3.2	0.05	ycaM	5	6.13333	0.244
3.2	0.05	yadI	5	6.13333	0.381
3.2	0.05	AG1	5	6.13333	0.27
3.2	0.05	ptsI	5	6.38333	0.308
3.2	0.05	clcB	5	6.38333	0.313
3.2	0.05	ycaM	5	6.38333	0.247
3.2	0.05	yadI	5	6.38333	0.397
3.2	0.05	AG1	5	6.38333	0.28
3.2	0.05	ptsI	5	6.63333	0.322
3.2	0.05	clcB	5	6.63333	0.322
3.2	0.05	ycaM	5	6.63333	0.252
3.2	0.05	yadI	5	6.63333	0.411
3.2	0.05	AG1	5	6.63333	0.29
3.2	0.05	ptsI	5	6.88333	0.331
3.2	0.05	clcB	5	6.88333	0.334
3.2	0.05	ycaM	5	6.88333	0.255
3.2	0.05	yadI	5	6.88333	0.434
3.2	0.05	AG1	5	6.88333	0.296
3.2	0.05	ptsI	5	7.13333	0.343
3.2	0.05	clcB	5	7.13333	0.337
3.2	0.05	ycaM	5	7.13333	0.257
3.2	0.05	yadI	5	7.13333	0.453
3.2	0.05	AG1	5	7.13333	0.307
3.2	0.05	ptsI	5	7.38333	0.356
3.2	0.05	clcB	5	7.38333	0.339
3.2	0.05	ycaM	5	7.38333	0.263
3.2	0.05	yadI	5	7.38333	0.471

3.2	0.05	AG1	5	7.38333	0.314
3.2	0.05	ptsI	5	7.63333	0.371
3.2	0.05	clcB	5	7.63333	0.343
3.2	0.05	ycaM	5	7.63333	0.265
3.2	0.05	yadI	5	7.63333	0.482
3.2	0.05	AG1	5	7.63333	0.324
3.2	0.05	ptsI	5	7.88333	0.382
3.2	0.05	clcB	5	7.88333	0.354
3.2	0.05	ycaM	5	7.88333	0.271
3.2	0.05	yadI	5	7.88333	0.492
3.2	0.05	AG1	5	7.88333	0.335
3.2	0.05	ptsI	5	8.13333	0.402
3.2	0.05	clcB	5	8.13333	0.355
3.2	0.05	ycaM	5	8.13333	0.272
3.2	0.05	yadI	5	8.13333	0.502
3.2	0.05	AG1	5	8.13333	0.343
3.2	0.05	ptsI	5	8.38333	0.415
3.2	0.05	clcB	5	8.38333	0.358
3.2	0.05	ycaM	5	8.38333	0.274
3.2	0.05	yadI	5	8.38333	0.512
3.2	0.05	AG1	5	8.38333	0.353
3.2	0.05	ptsI	5	8.88333	0.369
3.2	0.05	clcB	5	8.88333	0.311
3.2	0.05	ycaM	5	8.88333	0.264
3.2	0.05	yadI	5	8.88333	0.444
3.2	0.05	AG1	5	8.88333	0.367
3.2	0.05	ptsI	5	9.13333	0.39
3.2	0.05	clcB	5	9.13333	0.309
3.2	0.05	ycaM	5	9.13333	0.263
3.2	0.05	yadI	5	9.13333	0.448
3.2	0.05	AG1	5	9.13333	0.394
3.2	0.05	ptsI	5	9.38333	0.394
3.2	0.05	clcB	5	9.38333	0.306
3.2	0.05	ycaM	5	9.38333	0.261
3.2	0.05	yadI	5	9.38333	0.468
3.2	0.05	AG1	5	9.38333	0.422
3.2	0.05	ptsI	5	9.63333	0.398
3.2	0.05	clcB	5	9.63333	0.306
3.2	0.05	ycaM	5	9.63333	0.258
3.2	0.05	yadI	5	9.63333	0.476
3.2	0.05	AG1	5	9.63333	0.413
3.2	0.05	ptsI	5	9.88333	0.405
3.2	0.05	clcB	5	9.88333	0.306
3.2	0.05	ycaM	5	9.88333	0.258
3.2	0.05	yadI	5	9.88333	0.482
3.2	0.05	AG1	5	9.88333	0.419
3.2	0.05	ptsI	5	10.1333	0.407
3.2	0.05	clcB	5	10.1333	0.302
3.2	0.05	ycaM	5	10.1333	0.256
3.2	0.05	yadI	5	10.1333	0.485
3.2	0.05	AG1	5	10.1333	0.42
3.2	0.05	ptsI	5	10.3833	0.412
3.2	0.05	clcB	5	10.3833	0.304

3.2	0.05	ycaM	5	10.3833	0.255
3.2	0.05	yadI	5	10.3833	0.492
3.2	0.05	AG1	5	10.3833	0.428
3.2	0.05	ptsI	5	10.6333	0.414
3.2	0.05	clcB	5	10.6333	0.301
3.2	0.05	ycaM	5	10.6333	0.255
3.2	0.05	yadI	5	10.6333	0.49
3.2	0.05	AG1	5	10.6333	0.433
3.2	0.05	ptsI	5	10.8833	0.424
3.2	0.05	clcB	5	10.8833	0.304
3.2	0.05	ycaM	5	10.8833	0.256
3.2	0.05	yadI	5	10.8833	0.498
3.2	0.05	AG1	5	10.8833	0.435
3.2	0.05	ptsI	5	11.1333	0.425
3.2	0.05	clcB	5	11.1333	0.303
3.2	0.05	ycaM	5	11.1333	0.255
3.2	0.05	yadI	5	11.1333	0.502
3.2	0.05	AG1	5	11.1333	0.466
3.2	0.05	ptsI	5	11.3833	0.428
3.2	0.05	clcB	5	11.3833	0.301
3.2	0.05	ycaM	5	11.3833	0.255
3.2	0.05	yadI	5	11.3833	0.506
3.2	0.05	AG1	5	11.3833	0.454
3.2	0.05	ptsI	5	11.6333	0.433
3.2	0.05	clcB	5	11.6333	0.299
3.2	0.05	ycaM	5	11.6333	0.26
3.2	0.05	yadI	5	11.6333	0.513
3.2	0.05	AG1	5	11.6333	0.459
3.2	0.05	ptsI	5	11.8833	0.436
3.2	0.05	clcB	5	11.8833	0.302
3.2	0.05	ycaM	5	11.8833	0.26
3.2	0.05	yadI	5	11.8833	0.517
3.2	0.05	AG1	5	11.8833	0.451
3.2	0.05	ptsI	5	12.1333	0.44
3.2	0.05	clcB	5	12.1333	0.302
3.2	0.05	ycaM	5	12.1333	0.257
3.2	0.05	yadI	5	12.1333	0.519
3.2	0.05	AG1	5	12.1333	0.458
3.2	0.05	ptsI	5	12.3833	0.444
3.2	0.05	clcB	5	12.3833	0.304
3.2	0.05	ycaM	5	12.3833	0.255
3.2	0.05	yadI	5	12.3833	0.523
3.2	0.05	AG1	5	12.3833	0.456
3.2	0.05	ptsI	5	12.6333	0.452
3.2	0.05	clcB	5	12.6333	0.304
3.2	0.05	ycaM	5	12.6333	0.256
3.2	0.05	yadI	5	12.6333	0.531
3.2	0.05	AG1	5	12.6333	0.468
3.2	0.05	ptsI	5	12.8833	0.455
3.2	0.05	clcB	5	12.8833	0.301
3.2	0.05	ycaM	5	12.8833	0.259
3.2	0.05	yadI	5	12.8833	0.532
3.2	0.05	AG1	5	12.8833	0.472

3.2	0.05	ptsI	5	13.1333	0.46
3.2	0.05	clcB	5	13.1333	0.302
3.2	0.05	ycaM	5	13.1333	0.257
3.2	0.05	yadI	5	13.1333	0.542
3.2	0.05	AG1	5	13.1333	0.478
3.2	0.05	ptsI	5	13.3833	0.462
3.2	0.05	clcB	5	13.3833	0.302
3.2	0.05	ycaM	5	13.3833	0.258
3.2	0.05	yadI	5	13.3833	0.544
3.2	0.05	AG1	5	13.3833	0.483
3.2	0.05	ptsI	5	13.6333	0.466
3.2	0.05	clcB	5	13.6333	0.3
3.2	0.05	ycaM	5	13.6333	0.257
3.2	0.05	yadI	5	13.6333	0.548
3.2	0.05	AG1	5	13.6333	0.486
3.2	0.05	ptsI	5	13.8833	0.472
3.2	0.05	clcB	5	13.8833	0.301
3.2	0.05	ycaM	5	13.8833	0.257
3.2	0.05	yadI	5	13.8833	0.553
3.2	0.05	AG1	5	13.8833	0.489
3.2	0.05	ptsI	5	14.1333	0.466
3.2	0.05	clcB	5	14.1333	0.298
3.2	0.05	ycaM	5	14.1333	0.256
3.2	0.05	yadI	5	14.1333	0.556
3.2	0.05	AG1	5	14.1333	0.499
3.2	0.05	ptsI	5	14.3833	0.479
3.2	0.05	clcB	5	14.3833	0.3
3.2	0.05	ycaM	5	14.3833	0.255
3.2	0.05	yadI	5	14.3833	0.558
3.2	0.05	AG1	5	14.3833	0.5
3.2	0.05	ptsI	5	14.6333	0.485
3.2	0.05	clcB	5	14.6333	0.301
3.2	0.05	ycaM	5	14.6333	0.254
3.2	0.05	yadI	5	14.6333	0.561
3.2	0.05	AG1	5	14.6333	0.506
3.2	0.05	ptsI	5	14.8833	0.486
3.2	0.05	clcB	5	14.8833	0.3
3.2	0.05	ycaM	5	14.8833	0.255
3.2	0.05	yadI	5	14.8833	0.568
3.2	0.05	AG1	5	14.8833	0.511
3.2	0.05	ptsI	5	15.1333	0.496
3.2	0.05	clcB	5	15.1333	0.301
3.2	0.05	ycaM	5	15.1333	0.256
3.2	0.05	yadI	5	15.1333	0.572
3.2	0.05	AG1	5	15.1333	0.512
3.2	0.05	ptsI	5	15.3833	0.501
3.2	0.05	clcB	5	15.3833	0.3
3.2	0.05	ycaM	5	15.3833	0.256
3.2	0.05	yadI	5	15.3833	0.575
3.2	0.05	AG1	5	15.3833	0.518
3.2	0.05	ptsI	5	15.6333	0.503
3.2	0.05	clcB	5	15.6333	0.299
3.2	0.05	ycaM	5	15.6333	0.253

3.2	0.05	yadI	5	15.6333	0.575
3.2	0.05	AG1	5	15.6333	0.519
3.2	0.05	ptsI	5	15.8833	0.502
3.2	0.05	clcB	5	15.8833	0.3
3.2	0.05	ycaM	5	15.8833	0.253
3.2	0.05	yadI	5	15.8833	0.579
3.2	0.05	AG1	5	15.8833	0.523
3.2	0.05	ptsI	5	16.1333	0.51
3.2	0.05	clcB	5	16.1333	0.296
3.2	0.05	ycaM	5	16.1333	0.253
3.2	0.05	yadI	5	16.1333	0.575
3.2	0.05	AG1	5	16.1333	0.529
3.2	0.05	ptsI	5	16.3833	0.513
3.2	0.05	clcB	5	16.3833	0.299
3.2	0.05	ycaM	5	16.3833	0.255
3.2	0.05	yadI	5	16.3833	0.588
3.2	0.05	AG1	5	16.3833	0.533
3.2	0.05	ptsI	5	16.6333	0.525
3.2	0.05	clcB	5	16.6333	0.301
3.2	0.05	ycaM	5	16.6333	0.254
3.2	0.05	yadI	5	16.6333	0.591
3.2	0.05	AG1	5	16.6333	0.538
3.2	0.05	ptsI	5	16.8833	0.528
3.2	0.05	clcB	5	16.8833	0.301
3.2	0.05	ycaM	5	16.8833	0.254
3.2	0.05	yadI	5	16.8833	0.592
3.2	0.05	AG1	5	16.8833	0.538
3.2	0.05	ptsI	5	17.1333	0.532
3.2	0.05	clcB	5	17.1333	0.299
3.2	0.05	ycaM	5	17.1333	0.254
3.2	0.05	yadI	5	17.1333	0.591
3.2	0.05	AG1	5	17.1333	0.546
3.2	0.05	ptsI	5	17.3833	0.539
3.2	0.05	clcB	5	17.3833	0.3
3.2	0.05	ycaM	5	17.3833	0.254
3.2	0.05	yadI	5	17.3833	0.594
3.2	0.05	AG1	5	17.3833	0.546
3.2	0.05	ptsI	5	17.6333	0.542
3.2	0.05	clcB	5	17.6333	0.299
3.2	0.05	ycaM	5	17.6333	0.254
3.2	0.05	yadI	5	17.6333	0.598
3.2	0.05	AG1	5	17.6333	0.559
3.2	0.05	ptsI	5	17.8833	0.545
3.2	0.05	clcB	5	17.8833	0.3
3.2	0.05	ycaM	5	17.8833	0.253
3.2	0.05	yadI	5	17.8833	0.597
3.2	0.05	AG1	5	17.8833	0.554
3.2	0.05	ptsI	5	18.1333	0.553
3.2	0.05	clcB	5	18.1333	0.298
3.2	0.05	ycaM	5	18.1333	0.253
3.2	0.05	yadI	5	18.1333	0.603
3.2	0.05	AG1	5	18.1333	0.572
3.2	0.05	ptsI	5	18.3833	0.556

3.2	0.05	clcB	5	18.3833	0.298
3.2	0.05	ycaM	5	18.3833	0.253
3.2	0.05	yadI	5	18.3833	0.605
3.2	0.05	AG1	5	18.3833	0.576
3.2	0.05	ptsI	5	18.6333	0.559
3.2	0.05	clcB	5	18.6333	0.297
3.2	0.05	ycaM	5	18.6333	0.253
3.2	0.05	yadI	5	18.6333	0.605
3.2	0.05	AG1	5	18.6333	0.591
3.2	0.05	ptsI	5	18.8833	0.567
3.2	0.05	clcB	5	18.8833	0.297
3.2	0.05	ycaM	5	18.8833	0.253
3.2	0.05	yadI	5	18.8833	0.607
3.2	0.05	AG1	5	18.8833	0.597
3.2	0.05	ptsI	5	19.1333	0.569
3.2	0.05	clcB	5	19.1333	0.297
3.2	0.05	ycaM	5	19.1333	0.252
3.2	0.05	yadI	5	19.1333	0.608
3.2	0.05	AG1	5	19.1333	0.591
3.2	0.05	ptsI	5	19.3833	0.576
3.2	0.05	clcB	5	19.3833	0.297
3.2	0.05	ycaM	5	19.3833	0.252
3.2	0.05	yadI	5	19.3833	0.614
3.2	0.05	AG1	5	19.3833	0.614
3.2	0.05	ptsI	5	19.6333	0.584
3.2	0.05	clcB	5	19.6333	0.298
3.2	0.05	ycaM	5	19.6333	0.252
3.2	0.05	yadI	5	19.6333	0.608
3.2	0.05	AG1	5	19.6333	0.618
3.2	0.05	ptsI	5	19.8833	0.586
3.2	0.05	clcB	5	19.8833	0.297
3.2	0.05	ycaM	5	19.8833	0.252
3.2	0.05	yadI	5	19.8833	0.613
3.2	0.05	AG1	5	19.8833	0.589
3.2	0.05	ptsI	5	20.1333	0.589
3.2	0.05	clcB	5	20.1333	0.296
3.2	0.05	ycaM	5	20.1333	0.251
3.2	0.05	yadI	5	20.1333	0.613
3.2	0.05	AG1	5	20.1333	0.581
3.2	0.05	ptsI	5	20.3833	0.593
3.2	0.05	clcB	5	20.3833	0.296
3.2	0.05	ycaM	5	20.3833	0.251
3.2	0.05	yadI	5	20.3833	0.615
3.2	0.05	AG1	5	20.3833	0.579
3.2	0.05	ptsI	5	20.6333	0.596
3.2	0.05	clcB	5	20.6333	0.3
3.2	0.05	ycaM	5	20.6333	0.251
3.2	0.05	yadI	5	20.6333	0.614
3.2	0.05	AG1	5	20.6333	0.583
3.2	0.05	ptsI	5	20.8833	0.609
3.2	0.05	clcB	5	20.8833	0.296
3.2	0.05	ycaM	5	20.8833	0.252
3.2	0.05	yadI	5	20.8833	0.616

3.2	0.05	AG1	5	20.8833	0.583
3.2	0.05	ptsI	5	21.1333	0.608
3.2	0.05	clcB	5	21.1333	0.296
3.2	0.05	ycaM	5	21.1333	0.251
3.2	0.05	yadI	5	21.1333	0.616
3.2	0.05	AG1	5	21.1333	0.586
3.2	0.05	ptsI	5	21.3833	0.616
3.2	0.05	clcB	5	21.3833	0.296
3.2	0.05	ycaM	5	21.3833	0.25
3.2	0.05	yadI	5	21.3833	0.62
3.2	0.05	AG1	5	21.3833	0.589
3.2	0.05	ptsI	5	21.6333	0.619
3.2	0.05	clcB	5	21.6333	0.296
3.2	0.05	ycaM	5	21.6333	0.25
3.2	0.05	yadI	5	21.6333	0.625
3.2	0.05	AG1	5	21.6333	0.595
3.2	0.05	ptsI	5	21.8833	0.62
3.2	0.05	clcB	5	21.8833	0.295
3.2	0.05	ycaM	5	21.8833	0.25
3.2	0.05	yadI	5	21.8833	0.625
3.2	0.05	AG1	5	21.8833	0.596
3.2	0.05	ptsI	5	22.1333	0.626
3.2	0.05	clcB	5	22.1333	0.297
3.2	0.05	ycaM	5	22.1333	0.249
3.2	0.05	yadI	5	22.1333	0.625
3.2	0.05	AG1	5	22.1333	0.598
3.2	0.05	ptsI	5	22.3833	0.631
3.2	0.05	clcB	5	22.3833	0.294
3.2	0.05	ycaM	5	22.3833	0.249
3.2	0.05	yadI	5	22.3833	0.63
3.2	0.05	AG1	5	22.3833	0.602
3.2	0.05	ptsI	5	22.6333	0.635
3.2	0.05	clcB	5	22.6333	0.296
3.2	0.05	ycaM	5	22.6333	0.25
3.2	0.05	yadI	5	22.6333	0.632
3.2	0.05	AG1	5	22.6333	0.607
3.2	0.05	ptsI	5	22.8833	0.643
3.2	0.05	clcB	5	22.8833	0.295
3.2	0.05	ycaM	5	22.8833	0.249
3.2	0.05	yadI	5	22.8833	0.633
3.2	0.05	AG1	5	22.8833	0.608
3.2	0.05	ptsI	5	23.1333	0.644
3.2	0.05	clcB	5	23.1333	0.295
3.2	0.05	ycaM	5	23.1333	0.249
3.2	0.05	yadI	5	23.1333	0.632
3.2	0.05	AG1	5	23.1333	0.61
3.2	0.05	ptsI	5	23.3833	0.647
3.2	0.05	clcB	5	23.3833	0.295
3.2	0.05	ycaM	5	23.3833	0.248
3.2	0.05	yadI	5	23.3833	0.634
3.2	0.05	AG1	5	23.3833	0.615
3.2	0.05	ptsI	5	23.6333	0.649
3.2	0.05	clcB	5	23.6333	0.294

3.2	0.05	ycaM	5	23.6333	0.249
3.2	0.05	yadI	5	23.6333	0.633
3.2	0.05	AG1	5	23.6333	0.616
3.2	0.05	ptsI	5	23.8833	0.658
3.2	0.05	clcB	5	23.8833	0.296
3.2	0.05	ycaM	5	23.8833	0.248
3.2	0.05	yadI	5	23.8833	0.633
3.2	0.05	AG1	5	23.8833	0.617
3.2	0.05	ptsI	5	24.1333	0.661
3.2	0.05	clcB	5	24.1333	0.295
3.2	0.05	ycaM	5	24.1333	0.248
3.2	0.05	yadI	5	24.1333	0.637
3.2	0.05	AG1	5	24.1333	0.623
3.2	0.05	ptsI	5	24.3833	0.666
3.2	0.05	clcB	5	24.3833	0.294
3.2	0.05	ycaM	5	24.3833	0.248
3.2	0.05	yadI	5	24.3833	0.636
3.2	0.05	AG1	5	24.3833	0.621
0	0.1	ptsI	1	0	0.242
0	0.1	clcB	1	0	0.224
0	0.1	ycaM	1	0	0.232
0	0.1	yadI	1	0	0.25
0	0.1	AG1	1	0	0.241
0	0.1	ptsI	1	0.25	0.226
0	0.1	clcB	1	0.25	0.215
0	0.1	ycaM	1	0.25	0.221
0	0.1	yadI	1	0.25	0.236
0	0.1	AG1	1	0.25	0.229
0	0.1	ptsI	1	0.5	0.225
0	0.1	clcB	1	0.5	0.214
0	0.1	ycaM	1	0.5	0.217
0	0.1	yadI	1	0.5	0.236
0	0.1	AG1	1	0.5	0.227
0	0.1	ptsI	1	0.75	0.224
0	0.1	clcB	1	0.75	0.211
0	0.1	ycaM	1	0.75	0.216
0	0.1	yadI	1	0.75	0.231
0	0.1	AG1	1	0.75	0.223
0	0.1	ptsI	1	1	0.235
0	0.1	clcB	1	1	0.215
0	0.1	ycaM	1	1	0.216
0	0.1	yadI	1	1	0.237
0	0.1	AG1	1	1	0.227
0	0.1	ptsI	1	1.25	0.24
0	0.1	clcB	1	1.25	0.218
0	0.1	ycaM	1	1.25	0.22
0	0.1	yadI	1	1.25	0.24
0	0.1	AG1	1	1.25	0.228
0	0.1	ptsI	1	1.5	0.246
0	0.1	clcB	1	1.5	0.218
0	0.1	ycaM	1	1.5	0.22
0	0.1	yadI	1	1.5	0.243
0	0.1	AG1	1	1.5	0.234

0	0.1	ptsI	1	1.75	0.251
0	0.1	clcB	1	1.75	0.222
0	0.1	ycaM	1	1.75	0.224
0	0.1	yadI	1	1.75	0.25
0	0.1	AG1	1	1.75	0.234
0	0.1	ptsI	1	2	0.26
0	0.1	clcB	1	2	0.224
0	0.1	ycaM	1	2	0.224
0	0.1	yadI	1	2	0.253
0	0.1	AG1	1	2	0.239
0	0.1	ptsI	1	2.25	0.276
0	0.1	clcB	1	2.25	0.232
0	0.1	ycaM	1	2.25	0.233
0	0.1	yadI	1	2.25	0.262
0	0.1	AG1	1	2.25	0.243
0	0.1	ptsI	1	2.5	0.288
0	0.1	clcB	1	2.5	0.233
0	0.1	ycaM	1	2.5	0.232
0	0.1	yadI	1	2.5	0.266
0	0.1	AG1	1	2.5	0.244
0	0.1	ptsI	1	2.75	0.309
0	0.1	clcB	1	2.75	0.243
0	0.1	ycaM	1	2.75	0.243
0	0.1	yadI	1	2.75	0.278
0	0.1	AG1	1	2.75	0.253
0	0.1	ptsI	1	3	0.324
0	0.1	clcB	1	3	0.244
0	0.1	ycaM	1	3	0.247
0	0.1	yadI	1	3	0.285
0	0.1	AG1	1	3	0.261
0	0.1	ptsI	1	3.25	0.341
0	0.1	clcB	1	3.25	0.249
0	0.1	ycaM	1	3.25	0.253
0	0.1	yadI	1	3.25	0.296
0	0.1	AG1	1	3.25	0.268
0	0.1	ptsI	1	3.5	0.351
0	0.1	clcB	1	3.5	0.259
0	0.1	ycaM	1	3.5	0.263
0	0.1	yadI	1	3.5	0.311
0	0.1	AG1	1	3.5	0.277
0	0.1	ptsI	1	3.75	0.37
0	0.1	clcB	1	3.75	0.266
0	0.1	ycaM	1	3.75	0.272
0	0.1	yadI	1	3.75	0.326
0	0.1	AG1	1	3.75	0.29
0	0.1	ptsI	1	4	0.383
0	0.1	clcB	1	4	0.275
0	0.1	ycaM	1	4	0.278
0	0.1	yadI	1	4	0.333
0	0.1	AG1	1	4	0.301
0	0.1	ptsI	1	4.25	0.391
0	0.1	clcB	1	4.25	0.282
0	0.1	ycaM	1	4.25	0.29

0	0.1	yadI	1	4.25	0.348
0	0.1	AG1	1	4.25	0.311
0	0.1	ptsI	1	4.5	0.393
0	0.1	clcB	1	4.5	0.295
0	0.1	ycaM	1	4.5	0.298
0	0.1	yadI	1	4.5	0.362
0	0.1	AG1	1	4.5	0.319
0	0.1	ptsI	1	4.75	0.402
0	0.1	clcB	1	4.75	0.302
0	0.1	ycaM	1	4.75	0.306
0	0.1	yadI	1	4.75	0.375
0	0.1	AG1	1	4.75	0.334
0	0.1	ptsI	1	5	0.41
0	0.1	clcB	1	5	0.306
0	0.1	ycaM	1	5	0.311
0	0.1	yadI	1	5	0.386
0	0.1	AG1	1	5	0.342
0	0.1	ptsI	1	5.25	0.414
0	0.1	clcB	1	5.25	0.314
0	0.1	ycaM	1	5.25	0.32
0	0.1	yadI	1	5.25	0.401
0	0.1	AG1	1	5.25	0.352
0	0.1	ptsI	1	5.5	0.42
0	0.1	clcB	1	5.5	0.319
0	0.1	ycaM	1	5.5	0.32
0	0.1	yadI	1	5.5	0.41
0	0.1	AG1	1	5.5	0.36
0	0.1	ptsI	1	5.75	0.425
0	0.1	clcB	1	5.75	0.324
0	0.1	ycaM	1	5.75	0.328
0	0.1	yadI	1	5.75	0.418
0	0.1	AG1	1	5.75	0.37
0	0.1	ptsI	1	6	0.434
0	0.1	clcB	1	6	0.34
0	0.1	ycaM	1	6	0.347
0	0.1	yadI	1	6	0.434
0	0.1	AG1	1	6	0.387
0	0.1	ptsI	1	6.25	0.444
0	0.1	clcB	1	6.25	0.344
0	0.1	ycaM	1	6.25	0.356
0	0.1	yadI	1	6.25	0.444
0	0.1	AG1	1	6.25	0.398
0	0.1	ptsI	1	6.5	0.451
0	0.1	clcB	1	6.5	0.354
0	0.1	ycaM	1	6.5	0.369
0	0.1	yadI	1	6.5	0.467
0	0.1	AG1	1	6.5	0.416
0	0.1	ptsI	1	6.75	0.462
0	0.1	clcB	1	6.75	0.363
0	0.1	ycaM	1	6.75	0.378
0	0.1	yadI	1	6.75	0.483
0	0.1	AG1	1	6.75	0.423
0	0.1	ptsI	1	7	0.466

0	0.1	clcB	1	7	0.371
0	0.1	ycaM	1	7	0.381
0	0.1	yadI	1	7	0.493
0	0.1	AG1	1	7	0.432
0	0.1	ptsI	1	7.25	0.472
0	0.1	clcB	1	7.25	0.376
0	0.1	ycaM	1	7.25	0.386
0	0.1	yadI	1	7.25	0.504
0	0.1	AG1	1	7.25	0.439
0	0.1	ptsI	1	7.5	0.472
0	0.1	clcB	1	7.5	0.386
0	0.1	ycaM	1	7.5	0.395
0	0.1	yadI	1	7.5	0.514
0	0.1	AG1	1	7.5	0.449
0	0.1	ptsI	1	7.75	0.482
0	0.1	clcB	1	7.75	0.394
0	0.1	ycaM	1	7.75	0.408
0	0.1	yadI	1	7.75	0.532
0	0.1	AG1	1	7.75	0.461
0	0.1	ptsI	1	8	0.486
0	0.1	clcB	1	8	0.398
0	0.1	ycaM	1	8	0.41
0	0.1	yadI	1	8	0.54
0	0.1	AG1	1	8	0.47
0	0.1	ptsI	1	8.25	0.485
0	0.1	clcB	1	8.25	0.409
0	0.1	ycaM	1	8.25	0.416
0	0.1	yadI	1	8.25	0.55
0	0.1	AG1	1	8.25	0.483
0	0.1	ptsI	1	8.5	0.504
0	0.1	clcB	1	8.5	0.419
0	0.1	ycaM	1	8.5	0.422
0	0.1	yadI	1	8.5	0.563
0	0.1	AG1	1	8.5	0.495
0	0.1	ptsI	1	8.75	0.51
0	0.1	clcB	1	8.75	0.422
0	0.1	ycaM	1	8.75	0.425
0	0.1	yadI	1	8.75	0.572
0	0.1	AG1	1	8.75	0.503
0	0.1	ptsI	1	9	0.534
0	0.1	clcB	1	9	0.432
0	0.1	ycaM	1	9	0.433
0	0.1	yadI	1	9	0.586
0	0.1	AG1	1	9	0.519
0	0.1	ptsI	1	9.25	0.541
0	0.1	clcB	1	9.25	0.445
0	0.1	ycaM	1	9.25	0.447
0	0.1	yadI	1	9.25	0.589
0	0.1	AG1	1	9.25	0.54
0	0.1	ptsI	1	9.5	0.586
0	0.1	clcB	1	9.5	0.463
0	0.1	ycaM	1	9.5	0.462
0	0.1	yadI	1	9.5	0.634

0	0.1	AG1	1	9.5	0.574
0	0.1	ptsI	1	9.75	0.571
0	0.1	clcB	1	9.75	0.477
0	0.1	ycaM	1	9.75	0.474
0	0.1	yadI	1	9.75	0.647
0	0.1	AG1	1	9.75	0.577
0	0.1	ptsI	1	10	0.569
0	0.1	clcB	1	10	0.487
0	0.1	ycaM	1	10	0.484
0	0.1	yadI	1	10	0.636
0	0.1	AG1	1	10	0.575
0	0.1	ptsI	1	10.25	0.559
0	0.1	clcB	1	10.25	0.495
0	0.1	ycaM	1	10.25	0.493
0	0.1	yadI	1	10.25	0.649
0	0.1	AG1	1	10.25	0.582
0	0.1	ptsI	1	10.5	0.575
0	0.1	clcB	1	10.5	0.517
0	0.1	ycaM	1	10.5	0.503
0	0.1	yadI	1	10.5	0.657
0	0.1	AG1	1	10.5	0.595
0	0.1	ptsI	1	10.75	0.614
0	0.1	clcB	1	10.75	0.519
0	0.1	ycaM	1	10.75	0.513
0	0.1	yadI	1	10.75	0.669
0	0.1	AG1	1	10.75	0.598
0	0.1	ptsI	1	11	0.659
0	0.1	clcB	1	11	0.548
0	0.1	ycaM	1	11	0.531
0	0.1	yadI	1	11	0.72
0	0.1	AG1	1	11	0.643
0	0.1	ptsI	1	11.25	0.707
0	0.1	clcB	1	11.25	0.553
0	0.1	ycaM	1	11.25	0.525
0	0.1	yadI	1	11.25	0.725
0	0.1	AG1	1	11.25	0.642
0	0.1	ptsI	1	11.5	0.693
0	0.1	clcB	1	11.5	0.55
0	0.1	ycaM	1	11.5	0.544
0	0.1	yadI	1	11.5	0.772
0	0.1	AG1	1	11.5	0.674
0	0.1	ptsI	1	11.75	0.711
0	0.1	clcB	1	11.75	0.563
0	0.1	ycaM	1	11.75	0.552
0	0.1	yadI	1	11.75	0.828
0	0.1	AG1	1	11.75	0.723
0	0.1	ptsI	1	12	0.728
0	0.1	clcB	1	12	0.552
0	0.1	ycaM	1	12	0.553
0	0.1	yadI	1	12	0.834
0	0.1	AG1	1	12	0.722
0	0.1	ptsI	1	12.25	0.711
0	0.1	clcB	1	12.25	0.58

0	0.1	ycaM	1	12.25	0.564
0	0.1	yadI	1	12.25	0.888
0	0.1	AG1	1	12.25	0.741
0	0.1	ptsI	1	12.5	0.744
0	0.1	clcB	1	12.5	0.595
0	0.1	ycaM	1	12.5	0.562
0	0.1	yadI	1	12.5	0.906
0	0.1	AG1	1	12.5	0.748
0	0.1	ptsI	1	12.75	0.759
0	0.1	clcB	1	12.75	0.609
0	0.1	ycaM	1	12.75	0.564
0	0.1	yadI	1	12.75	0.912
0	0.1	AG1	1	12.75	0.766
0	0.1	ptsI	1	13	0.752
0	0.1	clcB	1	13	0.624
0	0.1	ycaM	1	13	0.573
0	0.1	yadI	1	13	0.917
0	0.1	AG1	1	13	0.788
0	0.1	ptsI	1	13.25	0.75
0	0.1	clcB	1	13.25	0.639
0	0.1	ycaM	1	13.25	0.594
0	0.1	yadI	1	13.25	0.946
0	0.1	AG1	1	13.25	0.797
0	0.1	ptsI	1	13.5	0.796
0	0.1	clcB	1	13.5	0.675
0	0.1	ycaM	1	13.5	0.622
0	0.1	yadI	1	13.5	0.952
0	0.1	AG1	1	13.5	0.811
0	0.1	ptsI	1	13.75	0.783
0	0.1	clcB	1	13.75	0.677
0	0.1	ycaM	1	13.75	0.631
0	0.1	yadI	1	13.75	0.943
0	0.1	AG1	1	13.75	0.822
0	0.1	ptsI	1	14	0.789
0	0.1	clcB	1	14	0.688
0	0.1	ycaM	1	14	0.666
0	0.1	yadI	1	14	0.947
0	0.1	AG1	1	14	0.847
0	0.1	ptsI	1	14.25	0.805
0	0.1	clcB	1	14.25	0.656
0	0.1	ycaM	1	14.25	0.653
0	0.1	yadI	1	14.25	0.952
0	0.1	AG1	1	14.25	0.842
0	0.1	ptsI	1	14.5	0.796
0	0.1	clcB	1	14.5	0.678
0	0.1	ycaM	1	14.5	0.693
0	0.1	yadI	1	14.5	0.956
0	0.1	AG1	1	14.5	0.844
0	0.1	ptsI	1	14.75	0.773
0	0.1	clcB	1	14.75	0.704
0	0.1	ycaM	1	14.75	0.72
0	0.1	yadI	1	14.75	0.973
0	0.1	AG1	1	14.75	0.847

0	0.1	ptsI	1	15	0.806
0	0.1	clcB	1	15	0.714
0	0.1	ycaM	1	15	0.718
0	0.1	yadI	1	15	0.964
0	0.1	AG1	1	15	0.851
0	0.1	ptsI	1	15.25	0.821
0	0.1	clcB	1	15.25	0.736
0	0.1	ycaM	1	15.25	0.736
0	0.1	yadI	1	15.25	0.958
0	0.1	AG1	1	15.25	0.877
0	0.1	ptsI	1	15.5	0.806
0	0.1	clcB	1	15.5	0.748
0	0.1	ycaM	1	15.5	0.69
0	0.1	yadI	1	15.5	0.954
0	0.1	AG1	1	15.5	0.855
0	0.1	ptsI	1	15.75	0.817
0	0.1	clcB	1	15.75	0.722
0	0.1	ycaM	1	15.75	0.705
0	0.1	yadI	1	15.75	0.947
0	0.1	AG1	1	15.75	0.85
0	0.1	ptsI	1	16	0.8
0	0.1	clcB	1	16	0.708
0	0.1	ycaM	1	16	0.725
0	0.1	yadI	1	16	0.948
0	0.1	AG1	1	16	0.876
0	0.1	ptsI	1	16.25	0.83
0	0.1	clcB	1	16.25	0.733
0	0.1	ycaM	1	16.25	0.738
0	0.1	yadI	1	16.25	0.943
0	0.1	AG1	1	16.25	0.896
0	0.1	ptsI	1	16.5	0.827
0	0.1	clcB	1	16.5	0.736
0	0.1	ycaM	1	16.5	0.739
0	0.1	yadI	1	16.5	0.94
0	0.1	AG1	1	16.5	0.866
0	0.1	ptsI	1	17.0667	0.808
0	0.1	clcB	1	17.0667	0.569
0	0.1	ycaM	1	17.0667	0.592
0	0.1	yadI	1	17.0667	0.669
0	0.1	AG1	1	17.0667	0.706
0	0.1	ptsI	1	17.3167	0.784
0	0.1	clcB	1	17.3167	0.522
0	0.1	ycaM	1	17.3167	0.542
0	0.1	yadI	1	17.3167	0.651
0	0.1	AG1	1	17.3167	0.714
0	0.1	ptsI	1	17.5667	0.793
0	0.1	clcB	1	17.5667	0.512
0	0.1	ycaM	1	17.5667	0.543
0	0.1	yadI	1	17.5667	0.66
0	0.1	AG1	1	17.5667	0.738
0	0.1	ptsI	1	17.8167	0.834
0	0.1	clcB	1	17.8167	0.515
0	0.1	ycaM	1	17.8167	0.545

0	0.1	yadI	1	17.8167	0.678
0	0.1	AG1	1	17.8167	0.797
0	0.1	ptsI	1	18.0667	0.819
0	0.1	clcB	1	18.0667	0.517
0	0.1	ycaM	1	18.0667	0.555
0	0.1	yadI	1	18.0667	0.692
0	0.1	AG1	1	18.0667	0.784
0	0.1	ptsI	1	18.3167	0.829
0	0.1	clcB	1	18.3167	0.526
0	0.1	ycaM	1	18.3167	0.569
0	0.1	yadI	1	18.3167	0.695
0	0.1	AG1	1	18.3167	0.818
0	0.1	ptsI	1	18.5667	0.837
0	0.1	clcB	1	18.5667	0.534
0	0.1	ycaM	1	18.5667	0.575
0	0.1	yadI	1	18.5667	0.706
0	0.1	AG1	1	18.5667	0.832
0	0.1	ptsI	1	18.8167	0.844
0	0.1	clcB	1	18.8167	0.538
0	0.1	ycaM	1	18.8167	0.578
0	0.1	yadI	1	18.8167	0.721
0	0.1	AG1	1	18.8167	0.845
0	0.1	ptsI	1	19.0667	0.847
0	0.1	clcB	1	19.0667	0.542
0	0.1	ycaM	1	19.0667	0.597
0	0.1	yadI	1	19.0667	0.743
0	0.1	AG1	1	19.0667	0.876
0	0.1	ptsI	1	19.3167	0.849
0	0.1	clcB	1	19.3167	0.55
0	0.1	ycaM	1	19.3167	0.578
0	0.1	yadI	1	19.3167	0.739
0	0.1	AG1	1	19.3167	0.853
0	0.1	ptsI	1	19.5667	0.856
0	0.1	clcB	1	19.5667	0.552
0	0.1	ycaM	1	19.5667	0.576
0	0.1	yadI	1	19.5667	0.741
0	0.1	AG1	1	19.5667	0.858
0	0.1	ptsI	1	19.8167	0.864
0	0.1	clcB	1	19.8167	0.551
0	0.1	ycaM	1	19.8167	0.576
0	0.1	yadI	1	19.8167	0.751
0	0.1	AG1	1	19.8167	0.863
0	0.1	ptsI	1	20.0667	0.863
0	0.1	clcB	1	20.0667	0.548
0	0.1	ycaM	1	20.0667	0.571
0	0.1	yadI	1	20.0667	0.759
0	0.1	AG1	1	20.0667	0.86
0	0.1	ptsI	1	20.3167	0.867
0	0.1	clcB	1	20.3167	0.54
0	0.1	ycaM	1	20.3167	0.574
0	0.1	yadI	1	20.3167	0.761
0	0.1	AG1	1	20.3167	0.864
0	0.1	ptsI	1	20.5667	0.851

0	0.1	clcB	1	20.5667	0.551
0	0.1	ycaM	1	20.5667	0.574
0	0.1	yadI	1	20.5667	0.771
0	0.1	AG1	1	20.5667	0.852
0	0.1	ptsI	1	20.8167	0.864
0	0.1	clcB	1	20.8167	0.56
0	0.1	ycaM	1	20.8167	0.567
0	0.1	yadI	1	20.8167	0.772
0	0.1	AG1	1	20.8167	0.86
0	0.1	ptsI	1	21.0667	0.877
0	0.1	clcB	1	21.0667	0.552
0	0.1	ycaM	1	21.0667	0.565
0	0.1	yadI	1	21.0667	0.774
0	0.1	AG1	1	21.0667	0.877
0	0.1	ptsI	1	21.3167	0.878
0	0.1	clcB	1	21.3167	0.562
0	0.1	ycaM	1	21.3167	0.563
0	0.1	yadI	1	21.3167	0.786
0	0.1	AG1	1	21.3167	0.884
0	0.1	ptsI	1	21.5667	0.882
0	0.1	clcB	1	21.5667	0.571
0	0.1	ycaM	1	21.5667	0.566
0	0.1	yadI	1	21.5667	0.794
0	0.1	AG1	1	21.5667	0.885
0	0.1	ptsI	1	21.8167	0.883
0	0.1	clcB	1	21.8167	0.562
0	0.1	ycaM	1	21.8167	0.558
0	0.1	yadI	1	21.8167	0.801
0	0.1	AG1	1	21.8167	0.897
0	0.1	ptsI	1	22.0667	0.894
0	0.1	clcB	1	22.0667	0.558
0	0.1	ycaM	1	22.0667	0.557
0	0.1	yadI	1	22.0667	0.796
0	0.1	AG1	1	22.0667	0.907
0	0.1	ptsI	1	22.9333	0.96
0	0.1	clcB	1	22.9333	0.527
0	0.1	ycaM	1	22.9333	0.639
0	0.1	yadI	1	22.9333	0.86
0	0.1	AG1	1	22.9333	1.037
0	0.1	ptsI	1	23.1833	0.954
0	0.1	clcB	1	23.1833	0.525
0	0.1	ycaM	1	23.1833	0.602
0	0.1	yadI	1	23.1833	0.86
0	0.1	AG1	1	23.1833	0.998
0	0.1	ptsI	1	23.4333	0.91
0	0.1	clcB	1	23.4333	0.528
0	0.1	ycaM	1	23.4333	0.605
0	0.1	yadI	1	23.4333	0.884
0	0.1	AG1	1	23.4333	0.975
0	0.1	ptsI	1	23.6833	0.904
0	0.1	clcB	1	23.6833	0.525
0	0.1	ycaM	1	23.6833	0.603
0	0.1	yadI	1	23.6833	0.898

0	0.1	AG1	1	23.6833	0.972
0	0.1	ptsI	1	23.9333	0.907
0	0.1	clcB	1	23.9333	0.527
0	0.1	ycaM	1	23.9333	0.603
0	0.1	yadI	1	23.9333	0.891
0	0.1	AG1	1	23.9333	0.958
0	0.1	ptsI	1	24.1833	0.905
0	0.1	clcB	1	24.1833	0.526
0	0.1	ycaM	1	24.1833	0.608
0	0.1	yadI	1	24.1833	0.898
0	0.1	AG1	1	24.1833	0.958
0	0.1	ptsI	1	24.4333	0.917
0	0.1	clcB	1	24.4333	0.524
0	0.1	ycaM	1	24.4333	0.6
0	0.1	yadI	1	24.4333	0.898
0	0.1	AG1	1	24.4333	0.96
0	0.1	ptsI	1	24.6833	0.906
0	0.1	clcB	1	24.6833	0.527
0	0.1	ycaM	1	24.6833	0.604
0	0.1	yadI	1	24.6833	0.906
0	0.1	AG1	1	24.6833	0.962
0	0.1	ptsI	1	24.9333	0.912
0	0.1	clcB	1	24.9333	0.521
0	0.1	ycaM	1	24.9333	0.608
0	0.1	yadI	1	24.9333	0.92
0	0.1	AG1	1	24.9333	0.969
0	0.1	ptsI	1	25.1833	0.91
0	0.1	clcB	1	25.1833	0.516
0	0.1	ycaM	1	25.1833	0.606
0	0.1	yadI	1	25.1833	0.91
0	0.1	AG1	1	25.1833	0.961
0	0.1	ptsI	1	25.4333	0.91
0	0.1	clcB	1	25.4333	0.523
0	0.1	ycaM	1	25.4333	0.609
0	0.1	yadI	1	25.4333	0.918
0	0.1	AG1	1	25.4333	0.962
0	0.1	ptsI	1	25.6833	0.907
0	0.1	clcB	1	25.6833	0.515
0	0.1	ycaM	1	25.6833	0.608
0	0.1	yadI	1	25.6833	0.917
0	0.1	AG1	1	25.6833	0.965
0	0.1	ptsI	1	25.9333	0.908
0	0.1	clcB	1	25.9333	0.514
0	0.1	ycaM	1	25.9333	0.605
0	0.1	yadI	1	25.9333	0.909
0	0.1	AG1	1	25.9333	0.966
0	0.1	ptsI	1	26.1833	0.915
0	0.1	clcB	1	26.1833	0.513
0	0.1	ycaM	1	26.1833	0.605
0	0.1	yadI	1	26.1833	0.922
0	0.1	AG1	1	26.1833	0.967
0	0.1	ptsI	1	26.4333	0.908
0	0.1	clcB	1	26.4333	0.518

0	0.1	ycaM	1	26.4333	0.609
0	0.1	yadI	1	26.4333	0.919
0	0.1	AG1	1	26.4333	0.966
0	0.1	ptsI	1	26.6833	0.904
0	0.1	clcB	1	26.6833	0.516
0	0.1	ycaM	1	26.6833	0.608
0	0.1	yadI	1	26.6833	0.93
0	0.1	AG1	1	26.6833	0.969
0	0.1	ptsI	1	26.9333	0.909
0	0.1	clcB	1	26.9333	0.511
0	0.1	ycaM	1	26.9333	0.604
0	0.1	yadI	1	26.9333	0.922
0	0.1	AG1	1	26.9333	0.969
0	0.1	ptsI	1	27.1833	0.914
0	0.1	clcB	1	27.1833	0.513
0	0.1	ycaM	1	27.1833	0.605
0	0.1	yadI	1	27.1833	0.926
0	0.1	AG1	1	27.1833	0.973
0	0.1	ptsI	1	27.4333	0.909
0	0.1	clcB	1	27.4333	0.511
0	0.1	ycaM	1	27.4333	0.606
0	0.1	yadI	1	27.4333	0.936
0	0.1	AG1	1	27.4333	0.968
0	0.1	ptsI	1	27.6833	0.91
0	0.1	clcB	1	27.6833	0.518
0	0.1	ycaM	1	27.6833	0.604
0	0.1	yadI	1	27.6833	0.94
0	0.1	AG1	1	27.6833	0.972
0	0.1	ptsI	1	27.9333	0.907
0	0.1	clcB	1	27.9333	0.508
0	0.1	ycaM	1	27.9333	0.595
0	0.1	yadI	1	27.9333	0.931
0	0.1	AG1	1	27.9333	0.968
0	0.1	ptsI	1	28.1833	0.91
0	0.1	clcB	1	28.1833	0.516
0	0.1	ycaM	1	28.1833	0.602
0	0.1	yadI	1	28.1833	0.942
0	0.1	AG1	1	28.1833	0.979
0	0.1	ptsI	1	28.4333	0.916
0	0.1	clcB	1	28.4333	0.515
0	0.1	ycaM	1	28.4333	0.6
0	0.1	yadI	1	28.4333	0.94
0	0.1	AG1	1	28.4333	0.977
0	0.1	ptsI	1	28.6833	0.917
0	0.1	clcB	1	28.6833	0.515
0	0.1	ycaM	1	28.6833	0.6
0	0.1	yadI	1	28.6833	0.943
0	0.1	AG1	1	28.6833	0.977
0	0.1	ptsI	1	28.9333	0.921
0	0.1	clcB	1	28.9333	0.52
0	0.1	ycaM	1	28.9333	0.603
0	0.1	yadI	1	28.9333	0.954
0	0.1	AG1	1	28.9333	0.983

0	0.1	ptsI	1	29.1833	0.917
0	0.1	clcB	1	29.1833	0.516
0	0.1	ycaM	1	29.1833	0.6
0	0.1	yadI	1	29.1833	0.956
0	0.1	AG1	1	29.1833	0.98
0	0.1	ptsI	1	29.4333	0.915
0	0.1	clcB	1	29.4333	0.518
0	0.1	ycaM	1	29.4333	0.597
0	0.1	yadI	1	29.4333	0.956
0	0.1	AG1	1	29.4333	0.981
0	0.1	ptsI	1	29.6833	0.924
0	0.1	clcB	1	29.6833	0.517
0	0.1	ycaM	1	29.6833	0.602
0	0.1	yadI	1	29.6833	0.958
0	0.1	AG1	1	29.6833	0.98
0	0.1	ptsI	1	29.9333	0.92
0	0.1	clcB	1	29.9333	0.518
0	0.1	ycaM	1	29.9333	0.6
0	0.1	yadI	1	29.9333	0.966
0	0.1	AG1	1	29.9333	0.98
0	0.1	ptsI	1	30.1833	0.926
0	0.1	clcB	1	30.1833	0.517
0	0.1	ycaM	1	30.1833	0.602
0	0.1	yadI	1	30.1833	0.966
0	0.1	AG1	1	30.1833	0.983
0	0.1	ptsI	1	30.4333	0.934
0	0.1	clcB	1	30.4333	0.521
0	0.1	ycaM	1	30.4333	0.6
0	0.1	yadI	1	30.4333	0.972
0	0.1	AG1	1	30.4333	0.984
0	0.1	ptsI	1	30.6833	0.93
0	0.1	clcB	1	30.6833	0.517
0	0.1	ycaM	1	30.6833	0.602
0	0.1	yadI	1	30.6833	0.978
0	0.1	AG1	1	30.6833	0.989
0	0.1	ptsI	1	30.9333	0.932
0	0.1	clcB	1	30.9333	0.522
0	0.1	ycaM	1	30.9333	0.602
0	0.1	yadI	1	30.9333	0.985
0	0.1	AG1	1	30.9333	0.986
0	0.1	ptsI	1	31.1833	0.935
0	0.1	clcB	1	31.1833	0.514
0	0.1	ycaM	1	31.1833	0.602
0	0.1	yadI	1	31.1833	0.986
0	0.1	AG1	1	31.1833	0.985
0	0.1	ptsI	1	31.4333	0.94
0	0.1	clcB	1	31.4333	0.521
0	0.1	ycaM	1	31.4333	0.602
0	0.1	yadI	1	31.4333	0.994
0	0.1	AG1	1	31.4333	0.988
0	0.1	ptsI	1	31.6833	0.94
0	0.1	clcB	1	31.6833	0.518
0	0.1	ycaM	1	31.6833	0.602

0	0.1	yadI	1	31.6833	1
0	0.1	AG1	1	31.6833	0.99
0	0.1	ptsI	1	31.9333	0.942
0	0.1	clcB	1	31.9333	0.521
0	0.1	ycaM	1	31.9333	0.603
0	0.1	yadI	1	31.9333	0.994
0	0.1	AG1	1	31.9333	0.99
0	0.1	ptsI	1	32.1833	0.941
0	0.1	clcB	1	32.1833	0.52
0	0.1	ycaM	1	32.1833	0.605
0	0.1	yadI	1	32.1833	1.003
0	0.1	AG1	1	32.1833	0.993
0	0.1	ptsI	1	32.4333	0.929
0	0.1	clcB	1	32.4333	0.503
0	0.1	ycaM	1	32.4333	0.596
0	0.1	yadI	1	32.4333	0.991
0	0.1	AG1	1	32.4333	0.994
0	0.1	ptsI	1	32.6833	0.938
0	0.1	clcB	1	32.6833	0.522
0	0.1	ycaM	1	32.6833	0.604
0	0.1	yadI	1	32.6833	1.013
0	0.1	AG1	1	32.6833	0.994
0	0.1	ptsI	1	32.9333	0.942
0	0.1	clcB	1	32.9333	0.516
0	0.1	ycaM	1	32.9333	0.604
0	0.1	yadI	1	32.9333	1.01
0	0.1	AG1	1	32.9333	0.994
0	0.1	ptsI	1	33.1833	0.932
0	0.1	clcB	1	33.1833	0.514
0	0.1	ycaM	1	33.1833	0.601
0	0.1	yadI	1	33.1833	1.013
0	0.1	AG1	1	33.1833	0.995
0	0.1	ptsI	1	33.4333	0.942
0	0.1	clcB	1	33.4333	0.525
0	0.1	ycaM	1	33.4333	0.608
0	0.1	yadI	1	33.4333	1.022
0	0.1	AG1	1	33.4333	0.999
0	0.1	ptsI	1	33.6833	0.941
0	0.1	clcB	1	33.6833	0.515
0	0.1	ycaM	1	33.6833	0.607
0	0.1	yadI	1	33.6833	1.013
0	0.1	AG1	1	33.6833	0.995
0	0.1	ptsI	1	33.9333	0.942
0	0.1	clcB	1	33.9333	0.523
0	0.1	ycaM	1	33.9333	0.607
0	0.1	yadI	1	33.9333	1.023
0	0.1	AG1	1	33.9333	1.001
0	0.1	ptsI	1	34.1833	0.946
0	0.1	clcB	1	34.1833	0.536
0	0.1	ycaM	1	34.1833	0.61
0	0.1	yadI	1	34.1833	1.033
0	0.1	AG1	1	34.1833	0.992
0	0.1	ptsI	1	34.4333	0.939

0	0.1	clcB	1	34.4333	0.527
0	0.1	ycaM	1	34.4333	0.611
0	0.1	yadI	1	34.4333	1.027
0	0.1	AG1	1	34.4333	1.004
0	0.1	ptsI	1	34.6833	0.944
0	0.1	clcB	1	34.6833	0.528
0	0.1	ycaM	1	34.6833	0.615
0	0.1	yadI	1	34.6833	1.037
0	0.1	AG1	1	34.6833	1.01
0	0.1	ptsI	1	34.9333	0.939
0	0.1	clcB	1	34.9333	0.528
0	0.1	ycaM	1	34.9333	0.611
0	0.1	yadI	1	34.9333	1.029
0	0.1	AG1	1	34.9333	1.011
0	0.1	ptsI	1	35.1833	0.939
0	0.1	clcB	1	35.1833	0.524
0	0.1	ycaM	1	35.1833	0.612
0	0.1	yadI	1	35.1833	1.033
0	0.1	AG1	1	35.1833	1.012
0	0.1	ptsI	1	35.4333	0.939
0	0.1	clcB	1	35.4333	0.528
0	0.1	ycaM	1	35.4333	0.613
0	0.1	yadI	1	35.4333	1.035
0	0.1	AG1	1	35.4333	1.016
0	0.1	ptsI	1	35.6833	0.942
0	0.1	clcB	1	35.6833	0.529
0	0.1	ycaM	1	35.6833	0.617
0	0.1	yadI	1	35.6833	1.036
0	0.1	AG1	1	35.6833	1.022
0	0.1	ptsI	1	35.9333	0.932
0	0.1	clcB	1	35.9333	0.522
0	0.1	ycaM	1	35.9333	0.611
0	0.1	yadI	1	35.9333	1.026
0	0.1	AG1	1	35.9333	1.02
0	0.1	ptsI	1	36.1833	0.943
0	0.1	clcB	1	36.1833	0.536
0	0.1	ycaM	1	36.1833	0.622
0	0.1	yadI	1	36.1833	1.04
0	0.1	AG1	1	36.1833	1.023
0	0.1	ptsI	1	36.4333	0.938
0	0.1	clcB	1	36.4333	0.525
0	0.1	ycaM	1	36.4333	0.617
0	0.1	yadI	1	36.4333	1.037
0	0.1	AG1	1	36.4333	1.027
0	0.1	ptsI	1	36.6833	0.937
0	0.1	clcB	1	36.6833	0.522
0	0.1	ycaM	1	36.6833	0.618
0	0.1	yadI	1	36.6833	1.039
0	0.1	AG1	1	36.6833	1.03
0.05	0.1	ptsI	1	0	0.241
0.05	0.1	clcB	1	0	0.242
0.05	0.1	ycaM	1	0	0.231
0.05	0.1	yadI	1	0	0.254

0.05	0.1	AG1	1	0	0.232
0.05	0.1	ptsI	1	0.25	0.236
0.05	0.1	clcB	1	0.25	0.238
0.05	0.1	ycaM	1	0.25	0.221
0.05	0.1	yadI	1	0.25	0.24
0.05	0.1	AG1	1	0.25	0.221
0.05	0.1	ptsI	1	0.5	0.234
0.05	0.1	clcB	1	0.5	0.236
0.05	0.1	ycaM	1	0.5	0.218
0.05	0.1	yadI	1	0.5	0.236
0.05	0.1	AG1	1	0.5	0.219
0.05	0.1	ptsI	1	0.75	0.232
0.05	0.1	clcB	1	0.75	0.232
0.05	0.1	ycaM	1	0.75	0.216
0.05	0.1	yadI	1	0.75	0.237
0.05	0.1	AG1	1	0.75	0.227
0.05	0.1	ptsI	1	1	0.234
0.05	0.1	clcB	1	1	0.232
0.05	0.1	ycaM	1	1	0.215
0.05	0.1	yadI	1	1	0.239
0.05	0.1	AG1	1	1	0.218
0.05	0.1	ptsI	1	1.25	0.23
0.05	0.1	clcB	1	1.25	0.23
0.05	0.1	ycaM	1	1.25	0.218
0.05	0.1	yadI	1	1.25	0.243
0.05	0.1	AG1	1	1.25	0.221
0.05	0.1	ptsI	1	1.5	0.237
0.05	0.1	clcB	1	1.5	0.234
0.05	0.1	ycaM	1	1.5	0.221
0.05	0.1	yadI	1	1.5	0.244
0.05	0.1	AG1	1	1.5	0.225
0.05	0.1	ptsI	1	1.75	0.239
0.05	0.1	clcB	1	1.75	0.234
0.05	0.1	ycaM	1	1.75	0.224
0.05	0.1	yadI	1	1.75	0.248
0.05	0.1	AG1	1	1.75	0.23
0.05	0.1	ptsI	1	2	0.246
0.05	0.1	clcB	1	2	0.24
0.05	0.1	ycaM	1	2	0.226
0.05	0.1	yadI	1	2	0.253
0.05	0.1	AG1	1	2	0.232
0.05	0.1	ptsI	1	2.25	0.251
0.05	0.1	clcB	1	2.25	0.239
0.05	0.1	ycaM	1	2.25	0.232
0.05	0.1	yadI	1	2.25	0.26
0.05	0.1	AG1	1	2.25	0.238
0.05	0.1	ptsI	1	2.5	0.256
0.05	0.1	clcB	1	2.5	0.243
0.05	0.1	ycaM	1	2.5	0.238
0.05	0.1	yadI	1	2.5	0.265
0.05	0.1	AG1	1	2.5	0.242
0.05	0.1	ptsI	1	2.75	0.26
0.05	0.1	clcB	1	2.75	0.248

0.05	0.1	ycaM	1	2.75	0.243
0.05	0.1	yadI	1	2.75	0.275
0.05	0.1	AG1	1	2.75	0.247
0.05	0.1	ptsI	1	3	0.272
0.05	0.1	clcB	1	3	0.254
0.05	0.1	ycaM	1	3	0.248
0.05	0.1	yadI	1	3	0.285
0.05	0.1	AG1	1	3	0.255
0.05	0.1	ptsI	1	3.25	0.277
0.05	0.1	clcB	1	3.25	0.256
0.05	0.1	ycaM	1	3.25	0.253
0.05	0.1	yadI	1	3.25	0.295
0.05	0.1	AG1	1	3.25	0.263
0.05	0.1	ptsI	1	3.5	0.285
0.05	0.1	clcB	1	3.5	0.26
0.05	0.1	ycaM	1	3.5	0.26
0.05	0.1	yadI	1	3.5	0.307
0.05	0.1	AG1	1	3.5	0.272
0.05	0.1	ptsI	1	3.75	0.297
0.05	0.1	clcB	1	3.75	0.267
0.05	0.1	ycaM	1	3.75	0.269
0.05	0.1	yadI	1	3.75	0.327
0.05	0.1	AG1	1	3.75	0.28
0.05	0.1	ptsI	1	4	0.303
0.05	0.1	clcB	1	4	0.272
0.05	0.1	ycaM	1	4	0.275
0.05	0.1	yadI	1	4	0.338
0.05	0.1	AG1	1	4	0.291
0.05	0.1	ptsI	1	4.25	0.316
0.05	0.1	clcB	1	4.25	0.281
0.05	0.1	ycaM	1	4.25	0.286
0.05	0.1	yadI	1	4.25	0.346
0.05	0.1	AG1	1	4.25	0.3
0.05	0.1	ptsI	1	4.5	0.327
0.05	0.1	clcB	1	4.5	0.29
0.05	0.1	ycaM	1	4.5	0.294
0.05	0.1	yadI	1	4.5	0.358
0.05	0.1	AG1	1	4.5	0.308
0.05	0.1	ptsI	1	4.75	0.338
0.05	0.1	clcB	1	4.75	0.297
0.05	0.1	ycaM	1	4.75	0.303
0.05	0.1	yadI	1	4.75	0.372
0.05	0.1	AG1	1	4.75	0.323
0.05	0.1	ptsI	1	5	0.355
0.05	0.1	clcB	1	5	0.305
0.05	0.1	ycaM	1	5	0.307
0.05	0.1	yadI	1	5	0.383
0.05	0.1	AG1	1	5	0.33
0.05	0.1	ptsI	1	5.25	0.358
0.05	0.1	clcB	1	5.25	0.306
0.05	0.1	ycaM	1	5.25	0.316
0.05	0.1	yadI	1	5.25	0.399
0.05	0.1	AG1	1	5.25	0.338

0.05	0.1	ptsI	1	5.5	0.375
0.05	0.1	clcB	1	5.5	0.317
0.05	0.1	ycaM	1	5.5	0.322
0.05	0.1	yadI	1	5.5	0.408
0.05	0.1	AG1	1	5.5	0.35
0.05	0.1	ptsI	1	5.75	0.377
0.05	0.1	clcB	1	5.75	0.323
0.05	0.1	ycaM	1	5.75	0.328
0.05	0.1	yadI	1	5.75	0.418
0.05	0.1	AG1	1	5.75	0.356
0.05	0.1	ptsI	1	6	0.379
0.05	0.1	clcB	1	6	0.332
0.05	0.1	ycaM	1	6	0.339
0.05	0.1	yadI	1	6	0.429
0.05	0.1	AG1	1	6	0.374
0.05	0.1	ptsI	1	6.25	0.384
0.05	0.1	clcB	1	6.25	0.335
0.05	0.1	ycaM	1	6.25	0.343
0.05	0.1	yadI	1	6.25	0.441
0.05	0.1	AG1	1	6.25	0.38
0.05	0.1	ptsI	1	6.5	0.397
0.05	0.1	clcB	1	6.5	0.348
0.05	0.1	ycaM	1	6.5	0.358
0.05	0.1	yadI	1	6.5	0.46
0.05	0.1	AG1	1	6.5	0.399
0.05	0.1	ptsI	1	6.75	0.414
0.05	0.1	clcB	1	6.75	0.352
0.05	0.1	ycaM	1	6.75	0.366
0.05	0.1	yadI	1	6.75	0.476
0.05	0.1	AG1	1	6.75	0.403
0.05	0.1	ptsI	1	7	0.424
0.05	0.1	clcB	1	7	0.36
0.05	0.1	ycaM	1	7	0.372
0.05	0.1	yadI	1	7	0.483
0.05	0.1	AG1	1	7	0.413
0.05	0.1	ptsI	1	7.25	0.431
0.05	0.1	clcB	1	7.25	0.368
0.05	0.1	ycaM	1	7.25	0.379
0.05	0.1	yadI	1	7.25	0.494
0.05	0.1	AG1	1	7.25	0.421
0.05	0.1	ptsI	1	7.5	0.44
0.05	0.1	clcB	1	7.5	0.371
0.05	0.1	ycaM	1	7.5	0.387
0.05	0.1	yadI	1	7.5	0.506
0.05	0.1	AG1	1	7.5	0.429
0.05	0.1	ptsI	1	7.75	0.446
0.05	0.1	clcB	1	7.75	0.378
0.05	0.1	ycaM	1	7.75	0.392
0.05	0.1	yadI	1	7.75	0.524
0.05	0.1	AG1	1	7.75	0.444
0.05	0.1	ptsI	1	8	0.458
0.05	0.1	clcB	1	8	0.387
0.05	0.1	ycaM	1	8	0.396

0.05	0.1	yadI	1	8	0.531
0.05	0.1	AG1	1	8	0.449
0.05	0.1	ptsI	1	8.25	0.466
0.05	0.1	clcB	1	8.25	0.389
0.05	0.1	ycaM	1	8.25	0.401
0.05	0.1	yadI	1	8.25	0.539
0.05	0.1	AG1	1	8.25	0.46
0.05	0.1	ptsI	1	8.5	0.475
0.05	0.1	clcB	1	8.5	0.397
0.05	0.1	ycaM	1	8.5	0.404
0.05	0.1	yadI	1	8.5	0.549
0.05	0.1	AG1	1	8.5	0.471
0.05	0.1	ptsI	1	8.75	0.488
0.05	0.1	clcB	1	8.75	0.406
0.05	0.1	ycaM	1	8.75	0.404
0.05	0.1	yadI	1	8.75	0.557
0.05	0.1	AG1	1	8.75	0.481
0.05	0.1	ptsI	1	9	0.499
0.05	0.1	clcB	1	9	0.411
0.05	0.1	ycaM	1	9	0.408
0.05	0.1	yadI	1	9	0.566
0.05	0.1	AG1	1	9	0.498
0.05	0.1	ptsI	1	9.25	0.52
0.05	0.1	clcB	1	9.25	0.415
0.05	0.1	ycaM	1	9.25	0.414
0.05	0.1	yadI	1	9.25	0.584
0.05	0.1	AG1	1	9.25	0.513
0.05	0.1	ptsI	1	9.5	0.569
0.05	0.1	clcB	1	9.5	0.424
0.05	0.1	ycaM	1	9.5	0.43
0.05	0.1	yadI	1	9.5	0.641
0.05	0.1	AG1	1	9.5	0.548
0.05	0.1	ptsI	1	9.75	0.556
0.05	0.1	clcB	1	9.75	0.424
0.05	0.1	ycaM	1	9.75	0.436
0.05	0.1	yadI	1	9.75	0.636
0.05	0.1	AG1	1	9.75	0.555
0.05	0.1	ptsI	1	10	0.546
0.05	0.1	clcB	1	10	0.429
0.05	0.1	ycaM	1	10	0.439
0.05	0.1	yadI	1	10	0.621
0.05	0.1	AG1	1	10	0.552
0.05	0.1	ptsI	1	10.25	0.558
0.05	0.1	clcB	1	10.25	0.437
0.05	0.1	ycaM	1	10.25	0.444
0.05	0.1	yadI	1	10.25	0.626
0.05	0.1	AG1	1	10.25	0.566
0.05	0.1	ptsI	1	10.5	0.597
0.05	0.1	clcB	1	10.5	0.45
0.05	0.1	ycaM	1	10.5	0.45
0.05	0.1	yadI	1	10.5	0.622
0.05	0.1	AG1	1	10.5	0.572
0.05	0.1	ptsI	1	10.75	0.597

0.05	0.1	clcB	1	10.75	0.456
0.05	0.1	ycaM	1	10.75	0.457
0.05	0.1	yadI	1	10.75	0.622
0.05	0.1	AG1	1	10.75	0.577
0.05	0.1	ptsI	1	11	0.636
0.05	0.1	clcB	1	11	0.473
0.05	0.1	ycaM	1	11	0.472
0.05	0.1	yadI	1	11	0.682
0.05	0.1	AG1	1	11	0.634
0.05	0.1	ptsI	1	11.25	0.666
0.05	0.1	clcB	1	11.25	0.488
0.05	0.1	ycaM	1	11.25	0.485
0.05	0.1	yadI	1	11.25	0.673
0.05	0.1	AG1	1	11.25	0.642
0.05	0.1	ptsI	1	11.5	0.623
0.05	0.1	clcB	1	11.5	0.49
0.05	0.1	ycaM	1	11.5	0.498
0.05	0.1	yadI	1	11.5	0.684
0.05	0.1	AG1	1	11.5	0.688
0.05	0.1	ptsI	1	11.75	0.638
0.05	0.1	clcB	1	11.75	0.5
0.05	0.1	ycaM	1	11.75	0.516
0.05	0.1	yadI	1	11.75	0.735
0.05	0.1	AG1	1	11.75	0.749
0.05	0.1	ptsI	1	12	0.626
0.05	0.1	clcB	1	12	0.505
0.05	0.1	ycaM	1	12	0.524
0.05	0.1	yadI	1	12	0.74
0.05	0.1	AG1	1	12	0.745
0.05	0.1	ptsI	1	12.25	0.604
0.05	0.1	clcB	1	12.25	0.516
0.05	0.1	ycaM	1	12.25	0.518
0.05	0.1	yadI	1	12.25	0.761
0.05	0.1	AG1	1	12.25	0.768
0.05	0.1	ptsI	1	12.5	0.626
0.05	0.1	clcB	1	12.5	0.519
0.05	0.1	ycaM	1	12.5	0.524
0.05	0.1	yadI	1	12.5	0.789
0.05	0.1	AG1	1	12.5	0.761
0.05	0.1	ptsI	1	12.75	0.617
0.05	0.1	clcB	1	12.75	0.526
0.05	0.1	ycaM	1	12.75	0.529
0.05	0.1	yadI	1	12.75	0.807
0.05	0.1	AG1	1	12.75	0.776
0.05	0.1	ptsI	1	13	0.631
0.05	0.1	clcB	1	13	0.539
0.05	0.1	ycaM	1	13	0.537
0.05	0.1	yadI	1	13	0.812
0.05	0.1	AG1	1	13	0.798
0.05	0.1	ptsI	1	13.25	0.594
0.05	0.1	clcB	1	13.25	0.528
0.05	0.1	ycaM	1	13.25	0.54
0.05	0.1	yadI	1	13.25	0.83

0.05	0.1	AG1	1	13.25	0.808
0.05	0.1	ptsI	1	13.5	0.617
0.05	0.1	clcB	1	13.5	0.524
0.05	0.1	ycaM	1	13.5	0.541
0.05	0.1	yadI	1	13.5	0.845
0.05	0.1	AG1	1	13.5	0.815
0.05	0.1	ptsI	1	13.75	0.608
0.05	0.1	clcB	1	13.75	0.534
0.05	0.1	ycaM	1	13.75	0.544
0.05	0.1	yadI	1	13.75	0.841
0.05	0.1	AG1	1	13.75	0.81
0.05	0.1	ptsI	1	14	0.643
0.05	0.1	clcB	1	14	0.559
0.05	0.1	ycaM	1	14	0.547
0.05	0.1	yadI	1	14	0.846
0.05	0.1	AG1	1	14	0.847
0.05	0.1	ptsI	1	14.25	0.687
0.05	0.1	clcB	1	14.25	0.576
0.05	0.1	ycaM	1	14.25	0.551
0.05	0.1	yadI	1	14.25	0.846
0.05	0.1	AG1	1	14.25	0.85
0.05	0.1	ptsI	1	14.5	0.676
0.05	0.1	clcB	1	14.5	0.578
0.05	0.1	ycaM	1	14.5	0.564
0.05	0.1	yadI	1	14.5	0.85
0.05	0.1	AG1	1	14.5	0.847
0.05	0.1	ptsI	1	14.75	0.706
0.05	0.1	clcB	1	14.75	0.585
0.05	0.1	ycaM	1	14.75	0.575
0.05	0.1	yadI	1	14.75	0.85
0.05	0.1	AG1	1	14.75	0.841
0.05	0.1	ptsI	1	15	0.749
0.05	0.1	clcB	1	15	0.619
0.05	0.1	ycaM	1	15	0.598
0.05	0.1	yadI	1	15	0.852
0.05	0.1	AG1	1	15	0.861
0.05	0.1	ptsI	1	15.25	0.721
0.05	0.1	clcB	1	15.25	0.656
0.05	0.1	ycaM	1	15.25	0.632
0.05	0.1	yadI	1	15.25	0.852
0.05	0.1	AG1	1	15.25	0.884
0.05	0.1	ptsI	1	15.5	0.705
0.05	0.1	clcB	1	15.5	0.661
0.05	0.1	ycaM	1	15.5	0.624
0.05	0.1	yadI	1	15.5	0.841
0.05	0.1	AG1	1	15.5	0.858
0.05	0.1	ptsI	1	15.75	0.741
0.05	0.1	clcB	1	15.75	0.642
0.05	0.1	ycaM	1	15.75	0.647
0.05	0.1	yadI	1	15.75	0.844
0.05	0.1	AG1	1	15.75	0.864
0.05	0.1	ptsI	1	16	0.746
0.05	0.1	clcB	1	16	0.646

0.05	0.1	ycaM	1	16	0.654
0.05	0.1	yadI	1	16	0.839
0.05	0.1	AG1	1	16	0.867
0.05	0.1	ptsI	1	16.25	0.777
0.05	0.1	clcB	1	16.25	0.615
0.05	0.1	ycaM	1	16.25	0.662
0.05	0.1	yadI	1	16.25	0.846
0.05	0.1	AG1	1	16.25	0.889
0.05	0.1	ptsI	1	16.5	0.752
0.05	0.1	clcB	1	16.5	0.596
0.05	0.1	ycaM	1	16.5	0.621
0.05	0.1	yadI	1	16.5	0.845
0.05	0.1	AG1	1	16.5	0.885
0.05	0.1	ptsI	1	17.0667	0.609
0.05	0.1	clcB	1	17.0667	0.496
0.05	0.1	ycaM	1	17.0667	0.481
0.05	0.1	yadI	1	17.0667	0.586
0.05	0.1	AG1	1	17.0667	0.872
0.05	0.1	ptsI	1	17.3167	0.575
0.05	0.1	clcB	1	17.3167	0.435
0.05	0.1	ycaM	1	17.3167	0.444
0.05	0.1	yadI	1	17.3167	0.562
0.05	0.1	AG1	1	17.3167	0.766
0.05	0.1	ptsI	1	17.5667	0.604
0.05	0.1	clcB	1	17.5667	0.433
0.05	0.1	ycaM	1	17.5667	0.443
0.05	0.1	yadI	1	17.5667	0.576
0.05	0.1	AG1	1	17.5667	0.762
0.05	0.1	ptsI	1	17.8167	0.607
0.05	0.1	clcB	1	17.8167	0.436
0.05	0.1	ycaM	1	17.8167	0.448
0.05	0.1	yadI	1	17.8167	0.587
0.05	0.1	AG1	1	17.8167	0.814
0.05	0.1	ptsI	1	18.0667	0.63
0.05	0.1	clcB	1	18.0667	0.435
0.05	0.1	ycaM	1	18.0667	0.449
0.05	0.1	yadI	1	18.0667	0.605
0.05	0.1	AG1	1	18.0667	0.791
0.05	0.1	ptsI	1	18.3167	0.648
0.05	0.1	clcB	1	18.3167	0.438
0.05	0.1	ycaM	1	18.3167	0.46
0.05	0.1	yadI	1	18.3167	0.611
0.05	0.1	AG1	1	18.3167	0.81
0.05	0.1	ptsI	1	18.5667	0.707
0.05	0.1	clcB	1	18.5667	0.446
0.05	0.1	ycaM	1	18.5667	0.452
0.05	0.1	yadI	1	18.5667	0.627
0.05	0.1	AG1	1	18.5667	0.825
0.05	0.1	ptsI	1	18.8167	0.725
0.05	0.1	clcB	1	18.8167	0.45
0.05	0.1	ycaM	1	18.8167	0.459
0.05	0.1	yadI	1	18.8167	0.636
0.05	0.1	AG1	1	18.8167	0.829

0.05	0.1	ptsI	1	19.0667	0.75
0.05	0.1	clcB	1	19.0667	0.452
0.05	0.1	ycaM	1	19.0667	0.463
0.05	0.1	yadI	1	19.0667	0.655
0.05	0.1	AG1	1	19.0667	0.857
0.05	0.1	ptsI	1	19.3167	0.743
0.05	0.1	clcB	1	19.3167	0.462
0.05	0.1	ycaM	1	19.3167	0.466
0.05	0.1	yadI	1	19.3167	0.653
0.05	0.1	AG1	1	19.3167	0.843
0.05	0.1	ptsI	1	19.5667	0.752
0.05	0.1	clcB	1	19.5667	0.465
0.05	0.1	ycaM	1	19.5667	0.469
0.05	0.1	yadI	1	19.5667	0.664
0.05	0.1	AG1	1	19.5667	0.848
0.05	0.1	ptsI	1	19.8167	0.748
0.05	0.1	clcB	1	19.8167	0.447
0.05	0.1	ycaM	1	19.8167	0.47
0.05	0.1	yadI	1	19.8167	0.668
0.05	0.1	AG1	1	19.8167	0.856
0.05	0.1	ptsI	1	20.0667	0.746
0.05	0.1	clcB	1	20.0667	0.443
0.05	0.1	ycaM	1	20.0667	0.473
0.05	0.1	yadI	1	20.0667	0.672
0.05	0.1	AG1	1	20.0667	0.866
0.05	0.1	ptsI	1	20.3167	0.75
0.05	0.1	clcB	1	20.3167	0.434
0.05	0.1	ycaM	1	20.3167	0.475
0.05	0.1	yadI	1	20.3167	0.676
0.05	0.1	AG1	1	20.3167	0.869
0.05	0.1	ptsI	1	20.5667	0.744
0.05	0.1	clcB	1	20.5667	0.428
0.05	0.1	ycaM	1	20.5667	0.474
0.05	0.1	yadI	1	20.5667	0.687
0.05	0.1	AG1	1	20.5667	0.868
0.05	0.1	ptsI	1	20.8167	0.75
0.05	0.1	clcB	1	20.8167	0.422
0.05	0.1	ycaM	1	20.8167	0.482
0.05	0.1	yadI	1	20.8167	0.688
0.05	0.1	AG1	1	20.8167	0.871
0.05	0.1	ptsI	1	21.0667	0.762
0.05	0.1	clcB	1	21.0667	0.421
0.05	0.1	ycaM	1	21.0667	0.482
0.05	0.1	yadI	1	21.0667	0.696
0.05	0.1	AG1	1	21.0667	0.888
0.05	0.1	ptsI	1	21.3167	0.767
0.05	0.1	clcB	1	21.3167	0.418
0.05	0.1	ycaM	1	21.3167	0.483
0.05	0.1	yadI	1	21.3167	0.71
0.05	0.1	AG1	1	21.3167	0.893
0.05	0.1	ptsI	1	21.5667	0.772
0.05	0.1	clcB	1	21.5667	0.427
0.05	0.1	ycaM	1	21.5667	0.489

0.05	0.1	yadI	1	21.5667	0.722
0.05	0.1	AG1	1	21.5667	0.89
0.05	0.1	ptsI	1	21.8167	0.78
0.05	0.1	clcB	1	21.8167	0.42
0.05	0.1	ycaM	1	21.8167	0.491
0.05	0.1	yadI	1	21.8167	0.729
0.05	0.1	AG1	1	21.8167	0.905
0.05	0.1	ptsI	1	22.0667	0.787
0.05	0.1	clcB	1	22.0667	0.42
0.05	0.1	ycaM	1	22.0667	0.491
0.05	0.1	yadI	1	22.0667	0.726
0.05	0.1	AG1	1	22.0667	0.908
0.05	0.1	ptsI	1	22.9333	0.78
0.05	0.1	clcB	1	22.9333	0.46
0.05	0.1	ycaM	1	22.9333	0.411
0.05	0.1	yadI	1	22.9333	0.745
0.05	0.1	AG1	1	22.9333	0.779
0.05	0.1	ptsI	1	23.1833	0.768
0.05	0.1	clcB	1	23.1833	0.441
0.05	0.1	ycaM	1	23.1833	0.402
0.05	0.1	yadI	1	23.1833	0.729
0.05	0.1	AG1	1	23.1833	0.762
0.05	0.1	ptsI	1	23.4333	0.746
0.05	0.1	clcB	1	23.4333	0.44
0.05	0.1	ycaM	1	23.4333	0.404
0.05	0.1	yadI	1	23.4333	0.738
0.05	0.1	AG1	1	23.4333	0.783
0.05	0.1	ptsI	1	23.6833	0.756
0.05	0.1	clcB	1	23.6833	0.441
0.05	0.1	ycaM	1	23.6833	0.406
0.05	0.1	yadI	1	23.6833	0.756
0.05	0.1	AG1	1	23.6833	0.815
0.05	0.1	ptsI	1	23.9333	0.735
0.05	0.1	clcB	1	23.9333	0.447
0.05	0.1	ycaM	1	23.9333	0.407
0.05	0.1	yadI	1	23.9333	0.757
0.05	0.1	AG1	1	23.9333	0.807
0.05	0.1	ptsI	1	24.1833	0.744
0.05	0.1	clcB	1	24.1833	0.436
0.05	0.1	ycaM	1	24.1833	0.406
0.05	0.1	yadI	1	24.1833	0.761
0.05	0.1	AG1	1	24.1833	0.816
0.05	0.1	ptsI	1	24.4333	0.773
0.05	0.1	clcB	1	24.4333	0.427
0.05	0.1	ycaM	1	24.4333	0.404
0.05	0.1	yadI	1	24.4333	0.767
0.05	0.1	AG1	1	24.4333	0.816
0.05	0.1	ptsI	1	24.6833	0.772
0.05	0.1	clcB	1	24.6833	0.43
0.05	0.1	ycaM	1	24.6833	0.405
0.05	0.1	yadI	1	24.6833	0.775
0.05	0.1	AG1	1	24.6833	0.818
0.05	0.1	ptsI	1	24.9333	0.783

0.05	0.1	clcB	1	24.9333	0.425
0.05	0.1	ycaM	1	24.9333	0.406
0.05	0.1	yadI	1	24.9333	0.782
0.05	0.1	AG1	1	24.9333	0.838
0.05	0.1	ptsI	1	25.1833	0.788
0.05	0.1	clcB	1	25.1833	0.426
0.05	0.1	ycaM	1	25.1833	0.408
0.05	0.1	yadI	1	25.1833	0.779
0.05	0.1	AG1	1	25.1833	0.826
0.05	0.1	ptsI	1	25.4333	0.802
0.05	0.1	clcB	1	25.4333	0.428
0.05	0.1	ycaM	1	25.4333	0.406
0.05	0.1	yadI	1	25.4333	0.787
0.05	0.1	AG1	1	25.4333	0.833
0.05	0.1	ptsI	1	25.6833	0.804
0.05	0.1	clcB	1	25.6833	0.429
0.05	0.1	ycaM	1	25.6833	0.409
0.05	0.1	yadI	1	25.6833	0.793
0.05	0.1	AG1	1	25.6833	0.83
0.05	0.1	ptsI	1	25.9333	0.813
0.05	0.1	clcB	1	25.9333	0.429
0.05	0.1	ycaM	1	25.9333	0.403
0.05	0.1	yadI	1	25.9333	0.794
0.05	0.1	AG1	1	25.9333	0.835
0.05	0.1	ptsI	1	26.1833	0.823
0.05	0.1	clcB	1	26.1833	0.43
0.05	0.1	ycaM	1	26.1833	0.406
0.05	0.1	yadI	1	26.1833	0.804
0.05	0.1	AG1	1	26.1833	0.844
0.05	0.1	ptsI	1	26.4333	0.818
0.05	0.1	clcB	1	26.4333	0.428
0.05	0.1	ycaM	1	26.4333	0.409
0.05	0.1	yadI	1	26.4333	0.804
0.05	0.1	AG1	1	26.4333	0.84
0.05	0.1	ptsI	1	26.6833	0.821
0.05	0.1	clcB	1	26.6833	0.426
0.05	0.1	ycaM	1	26.6833	0.411
0.05	0.1	yadI	1	26.6833	0.81
0.05	0.1	AG1	1	26.6833	0.846
0.05	0.1	ptsI	1	26.9333	0.826
0.05	0.1	clcB	1	26.9333	0.427
0.05	0.1	ycaM	1	26.9333	0.408
0.05	0.1	yadI	1	26.9333	0.81
0.05	0.1	AG1	1	26.9333	0.849
0.05	0.1	ptsI	1	27.1833	0.836
0.05	0.1	clcB	1	27.1833	0.434
0.05	0.1	ycaM	1	27.1833	0.409
0.05	0.1	yadI	1	27.1833	0.818
0.05	0.1	AG1	1	27.1833	0.854
0.05	0.1	ptsI	1	27.4333	0.826
0.05	0.1	clcB	1	27.4333	0.433
0.05	0.1	ycaM	1	27.4333	0.41
0.05	0.1	yadI	1	27.4333	0.821

0.05	0.1	AG1	1	27.4333	0.855
0.05	0.1	ptsI	1	27.6833	0.837
0.05	0.1	clcB	1	27.6833	0.435
0.05	0.1	ycaM	1	27.6833	0.41
0.05	0.1	yadI	1	27.6833	0.822
0.05	0.1	AG1	1	27.6833	0.862
0.05	0.1	ptsI	1	27.9333	0.832
0.05	0.1	clcB	1	27.9333	0.428
0.05	0.1	ycaM	1	27.9333	0.405
0.05	0.1	yadI	1	27.9333	0.818
0.05	0.1	AG1	1	27.9333	0.854
0.05	0.1	ptsI	1	28.1833	0.847
0.05	0.1	clcB	1	28.1833	0.434
0.05	0.1	ycaM	1	28.1833	0.411
0.05	0.1	yadI	1	28.1833	0.827
0.05	0.1	AG1	1	28.1833	0.871
0.05	0.1	ptsI	1	28.4333	0.844
0.05	0.1	clcB	1	28.4333	0.434
0.05	0.1	ycaM	1	28.4333	0.411
0.05	0.1	yadI	1	28.4333	0.832
0.05	0.1	AG1	1	28.4333	0.872
0.05	0.1	ptsI	1	28.6833	0.84
0.05	0.1	clcB	1	28.6833	0.44
0.05	0.1	ycaM	1	28.6833	0.41
0.05	0.1	yadI	1	28.6833	0.838
0.05	0.1	AG1	1	28.6833	0.871
0.05	0.1	ptsI	1	28.9333	0.851
0.05	0.1	clcB	1	28.9333	0.439
0.05	0.1	ycaM	1	28.9333	0.411
0.05	0.1	yadI	1	28.9333	0.841
0.05	0.1	AG1	1	28.9333	0.881
0.05	0.1	ptsI	1	29.1833	0.843
0.05	0.1	clcB	1	29.1833	0.438
0.05	0.1	ycaM	1	29.1833	0.412
0.05	0.1	yadI	1	29.1833	0.849
0.05	0.1	AG1	1	29.1833	0.879
0.05	0.1	ptsI	1	29.4333	0.849
0.05	0.1	clcB	1	29.4333	0.442
0.05	0.1	ycaM	1	29.4333	0.414
0.05	0.1	yadI	1	29.4333	0.846
0.05	0.1	AG1	1	29.4333	0.884
0.05	0.1	ptsI	1	29.6833	0.852
0.05	0.1	clcB	1	29.6833	0.443
0.05	0.1	ycaM	1	29.6833	0.415
0.05	0.1	yadI	1	29.6833	0.848
0.05	0.1	AG1	1	29.6833	0.888
0.05	0.1	ptsI	1	29.9333	0.858
0.05	0.1	clcB	1	29.9333	0.444
0.05	0.1	ycaM	1	29.9333	0.412
0.05	0.1	yadI	1	29.9333	0.852
0.05	0.1	AG1	1	29.9333	0.894
0.05	0.1	ptsI	1	30.1833	0.852
0.05	0.1	clcB	1	30.1833	0.446

0.05	0.1	ycaM	1	30.1833	0.415
0.05	0.1	yadI	1	30.1833	0.856
0.05	0.1	AG1	1	30.1833	0.896
0.05	0.1	ptsI	1	30.4333	0.857
0.05	0.1	clcB	1	30.4333	0.444
0.05	0.1	ycaM	1	30.4333	0.414
0.05	0.1	yadI	1	30.4333	0.858
0.05	0.1	AG1	1	30.4333	0.903
0.05	0.1	ptsI	1	30.6833	0.852
0.05	0.1	clcB	1	30.6833	0.446
0.05	0.1	ycaM	1	30.6833	0.418
0.05	0.1	yadI	1	30.6833	0.873
0.05	0.1	AG1	1	30.6833	0.904
0.05	0.1	ptsI	1	30.9333	0.858
0.05	0.1	clcB	1	30.9333	0.448
0.05	0.1	ycaM	1	30.9333	0.417
0.05	0.1	yadI	1	30.9333	0.874
0.05	0.1	AG1	1	30.9333	0.907
0.05	0.1	ptsI	1	31.1833	0.857
0.05	0.1	clcB	1	31.1833	0.445
0.05	0.1	ycaM	1	31.1833	0.42
0.05	0.1	yadI	1	31.1833	0.876
0.05	0.1	AG1	1	31.1833	0.911
0.05	0.1	ptsI	1	31.4333	0.866
0.05	0.1	clcB	1	31.4333	0.456
0.05	0.1	ycaM	1	31.4333	0.418
0.05	0.1	yadI	1	31.4333	0.871
0.05	0.1	AG1	1	31.4333	0.918
0.05	0.1	ptsI	1	31.6833	0.863
0.05	0.1	clcB	1	31.6833	0.461
0.05	0.1	ycaM	1	31.6833	0.42
0.05	0.1	yadI	1	31.6833	0.876
0.05	0.1	AG1	1	31.6833	0.926
0.05	0.1	ptsI	1	31.9333	0.867
0.05	0.1	clcB	1	31.9333	0.451
0.05	0.1	ycaM	1	31.9333	0.42
0.05	0.1	yadI	1	31.9333	0.875
0.05	0.1	AG1	1	31.9333	0.928
0.05	0.1	ptsI	1	32.1833	0.869
0.05	0.1	clcB	1	32.1833	0.457
0.05	0.1	ycaM	1	32.1833	0.422
0.05	0.1	yadI	1	32.1833	0.881
0.05	0.1	AG1	1	32.1833	0.933
0.05	0.1	ptsI	1	32.4333	0.864
0.05	0.1	clcB	1	32.4333	0.448
0.05	0.1	ycaM	1	32.4333	0.418
0.05	0.1	yadI	1	32.4333	0.879
0.05	0.1	AG1	1	32.4333	0.928
0.05	0.1	ptsI	1	32.6833	0.864
0.05	0.1	clcB	1	32.6833	0.461
0.05	0.1	ycaM	1	32.6833	0.432
0.05	0.1	yadI	1	32.6833	0.89
0.05	0.1	AG1	1	32.6833	0.944

0.05	0.1	ptsI	1	32.9333	0.866
0.05	0.1	clcB	1	32.9333	0.446
0.05	0.1	ycaM	1	32.9333	0.425
0.05	0.1	yadI	1	32.9333	0.89
0.05	0.1	AG1	1	32.9333	0.952
0.05	0.1	ptsI	1	33.1833	0.869
0.05	0.1	clcB	1	33.1833	0.448
0.05	0.1	ycaM	1	33.1833	0.423
0.05	0.1	yadI	1	33.1833	0.89
0.05	0.1	AG1	1	33.1833	0.95
0.05	0.1	ptsI	1	33.4333	0.872
0.05	0.1	clcB	1	33.4333	0.454
0.05	0.1	ycaM	1	33.4333	0.428
0.05	0.1	yadI	1	33.4333	0.888
0.05	0.1	AG1	1	33.4333	0.966
0.05	0.1	ptsI	1	33.6833	0.868
0.05	0.1	clcB	1	33.6833	0.458
0.05	0.1	ycaM	1	33.6833	0.425
0.05	0.1	yadI	1	33.6833	0.886
0.05	0.1	AG1	1	33.6833	0.965
0.05	0.1	ptsI	1	33.9333	0.872
0.05	0.1	clcB	1	33.9333	0.453
0.05	0.1	ycaM	1	33.9333	0.428
0.05	0.1	yadI	1	33.9333	0.896
0.05	0.1	AG1	1	33.9333	0.978
0.05	0.1	ptsI	1	34.1833	0.876
0.05	0.1	clcB	1	34.1833	0.451
0.05	0.1	ycaM	1	34.1833	0.434
0.05	0.1	yadI	1	34.1833	0.902
0.05	0.1	AG1	1	34.1833	0.986
0.05	0.1	ptsI	1	34.4333	0.871
0.05	0.1	clcB	1	34.4333	0.457
0.05	0.1	ycaM	1	34.4333	0.43
0.05	0.1	yadI	1	34.4333	0.903
0.05	0.1	AG1	1	34.4333	0.994
0.05	0.1	ptsI	1	34.6833	0.879
0.05	0.1	clcB	1	34.6833	0.461
0.05	0.1	ycaM	1	34.6833	0.433
0.05	0.1	yadI	1	34.6833	0.903
0.05	0.1	AG1	1	34.6833	1.008
0.05	0.1	ptsI	1	34.9333	0.881
0.05	0.1	clcB	1	34.9333	0.453
0.05	0.1	ycaM	1	34.9333	0.431
0.05	0.1	yadI	1	34.9333	0.908
0.05	0.1	AG1	1	34.9333	1.01
0.05	0.1	ptsI	1	35.1833	0.872
0.05	0.1	clcB	1	35.1833	0.455
0.05	0.1	ycaM	1	35.1833	0.432
0.05	0.1	yadI	1	35.1833	0.911
0.05	0.1	AG1	1	35.1833	1.015
0.05	0.1	ptsI	1	35.4333	0.876
0.05	0.1	clcB	1	35.4333	0.453
0.05	0.1	ycaM	1	35.4333	0.433

0.05	0.1	yadI	1	35.4333	0.913
0.05	0.1	AG1	1	35.4333	1.023
0.05	0.1	ptsI	1	35.6833	0.879
0.05	0.1	clcB	1	35.6833	0.463
0.05	0.1	ycaM	1	35.6833	0.438
0.05	0.1	yadI	1	35.6833	0.911
0.05	0.1	AG1	1	35.6833	1.035
0.05	0.1	ptsI	1	35.9333	0.881
0.05	0.1	clcB	1	35.9333	0.457
0.05	0.1	ycaM	1	35.9333	0.435
0.05	0.1	yadI	1	35.9333	0.912
0.05	0.1	AG1	1	35.9333	1.032
0.05	0.1	ptsI	1	36.1833	0.877
0.05	0.1	clcB	1	36.1833	0.46
0.05	0.1	ycaM	1	36.1833	0.446
0.05	0.1	yadI	1	36.1833	0.915
0.05	0.1	AG1	1	36.1833	1.047
0.05	0.1	ptsI	1	36.4333	0.885
0.05	0.1	clcB	1	36.4333	0.456
0.05	0.1	ycaM	1	36.4333	0.437
0.05	0.1	yadI	1	36.4333	0.918
0.05	0.1	AG1	1	36.4333	1.05
0.05	0.1	ptsI	1	36.6833	0.883
0.05	0.1	clcB	1	36.6833	0.463
0.05	0.1	ycaM	1	36.6833	0.435
0.05	0.1	yadI	1	36.6833	0.921
0.05	0.1	AG1	1	36.6833	1.06
0.1	0.1	ptsI	1	0	0.235
0.1	0.1	clcB	1	0	0.249
0.1	0.1	ycaM	1	0	0.228
0.1	0.1	yadI	1	0	0.242
0.1	0.1	AG1	1	0	0.235
0.1	0.1	ptsI	1	0.25	0.222
0.1	0.1	clcB	1	0.25	0.24
0.1	0.1	ycaM	1	0.25	0.212
0.1	0.1	yadI	1	0.25	0.234
0.1	0.1	AG1	1	0.25	0.224
0.1	0.1	ptsI	1	0.5	0.219
0.1	0.1	clcB	1	0.5	0.236
0.1	0.1	ycaM	1	0.5	0.209
0.1	0.1	yadI	1	0.5	0.231
0.1	0.1	AG1	1	0.5	0.22
0.1	0.1	ptsI	1	0.75	0.218
0.1	0.1	clcB	1	0.75	0.232
0.1	0.1	ycaM	1	0.75	0.207
0.1	0.1	yadI	1	0.75	0.236
0.1	0.1	AG1	1	0.75	0.221
0.1	0.1	ptsI	1	1	0.22
0.1	0.1	clcB	1	1	0.234
0.1	0.1	ycaM	1	1	0.207
0.1	0.1	yadI	1	1	0.235
0.1	0.1	AG1	1	1	0.221
0.1	0.1	ptsI	1	1.25	0.222

0.1	0.1	clcB	1	1.25	0.232
0.1	0.1	ycaM	1	1.25	0.206
0.1	0.1	yadI	1	1.25	0.238
0.1	0.1	AG1	1	1.25	0.227
0.1	0.1	ptsI	1	1.5	0.227
0.1	0.1	clcB	1	1.5	0.232
0.1	0.1	ycaM	1	1.5	0.207
0.1	0.1	yadI	1	1.5	0.241
0.1	0.1	AG1	1	1.5	0.227
0.1	0.1	ptsI	1	1.75	0.23
0.1	0.1	clcB	1	1.75	0.235
0.1	0.1	ycaM	1	1.75	0.211
0.1	0.1	yadI	1	1.75	0.247
0.1	0.1	AG1	1	1.75	0.232
0.1	0.1	ptsI	1	2	0.236
0.1	0.1	clcB	1	2	0.239
0.1	0.1	ycaM	1	2	0.215
0.1	0.1	yadI	1	2	0.251
0.1	0.1	AG1	1	2	0.235
0.1	0.1	ptsI	1	2.25	0.242
0.1	0.1	clcB	1	2.25	0.244
0.1	0.1	ycaM	1	2.25	0.218
0.1	0.1	yadI	1	2.25	0.259
0.1	0.1	AG1	1	2.25	0.239
0.1	0.1	ptsI	1	2.5	0.248
0.1	0.1	clcB	1	2.5	0.246
0.1	0.1	ycaM	1	2.5	0.222
0.1	0.1	yadI	1	2.5	0.267
0.1	0.1	AG1	1	2.5	0.246
0.1	0.1	ptsI	1	2.75	0.253
0.1	0.1	clcB	1	2.75	0.252
0.1	0.1	ycaM	1	2.75	0.228
0.1	0.1	yadI	1	2.75	0.273
0.1	0.1	AG1	1	2.75	0.255
0.1	0.1	ptsI	1	3	0.264
0.1	0.1	clcB	1	3	0.255
0.1	0.1	ycaM	1	3	0.234
0.1	0.1	yadI	1	3	0.286
0.1	0.1	AG1	1	3	0.258
0.1	0.1	ptsI	1	3.25	0.274
0.1	0.1	clcB	1	3.25	0.263
0.1	0.1	ycaM	1	3.25	0.242
0.1	0.1	yadI	1	3.25	0.293
0.1	0.1	AG1	1	3.25	0.264
0.1	0.1	ptsI	1	3.5	0.281
0.1	0.1	clcB	1	3.5	0.268
0.1	0.1	ycaM	1	3.5	0.249
0.1	0.1	yadI	1	3.5	0.304
0.1	0.1	AG1	1	3.5	0.274
0.1	0.1	ptsI	1	3.75	0.29
0.1	0.1	clcB	1	3.75	0.272
0.1	0.1	ycaM	1	3.75	0.252
0.1	0.1	yadI	1	3.75	0.318

0.1	0.1	AG1	1	3.75	0.29
0.1	0.1	ptsI	1	4	0.307
0.1	0.1	clcB	1	4	0.279
0.1	0.1	ycaM	1	4	0.265
0.1	0.1	yadI	1	4	0.332
0.1	0.1	AG1	1	4	0.3
0.1	0.1	ptsI	1	4.25	0.305
0.1	0.1	clcB	1	4.25	0.288
0.1	0.1	ycaM	1	4.25	0.269
0.1	0.1	yadI	1	4.25	0.339
0.1	0.1	AG1	1	4.25	0.31
0.1	0.1	ptsI	1	4.5	0.316
0.1	0.1	clcB	1	4.5	0.295
0.1	0.1	ycaM	1	4.5	0.276
0.1	0.1	yadI	1	4.5	0.35
0.1	0.1	AG1	1	4.5	0.319
0.1	0.1	ptsI	1	4.75	0.329
0.1	0.1	clcB	1	4.75	0.304
0.1	0.1	ycaM	1	4.75	0.28
0.1	0.1	yadI	1	4.75	0.362
0.1	0.1	AG1	1	4.75	0.334
0.1	0.1	ptsI	1	5	0.343
0.1	0.1	clcB	1	5	0.31
0.1	0.1	ycaM	1	5	0.286
0.1	0.1	yadI	1	5	0.367
0.1	0.1	AG1	1	5	0.34
0.1	0.1	ptsI	1	5.25	0.355
0.1	0.1	clcB	1	5.25	0.313
0.1	0.1	ycaM	1	5.25	0.291
0.1	0.1	yadI	1	5.25	0.38
0.1	0.1	AG1	1	5.25	0.351
0.1	0.1	ptsI	1	5.5	0.365
0.1	0.1	clcB	1	5.5	0.321
0.1	0.1	ycaM	1	5.5	0.301
0.1	0.1	yadI	1	5.5	0.39
0.1	0.1	AG1	1	5.5	0.362
0.1	0.1	ptsI	1	5.75	0.373
0.1	0.1	clcB	1	5.75	0.325
0.1	0.1	ycaM	1	5.75	0.306
0.1	0.1	yadI	1	5.75	0.398
0.1	0.1	AG1	1	5.75	0.371
0.1	0.1	ptsI	1	6	0.375
0.1	0.1	clcB	1	6	0.334
0.1	0.1	ycaM	1	6	0.311
0.1	0.1	yadI	1	6	0.409
0.1	0.1	AG1	1	6	0.387
0.1	0.1	ptsI	1	6.25	0.384
0.1	0.1	clcB	1	6.25	0.339
0.1	0.1	ycaM	1	6.25	0.318
0.1	0.1	yadI	1	6.25	0.42
0.1	0.1	AG1	1	6.25	0.397
0.1	0.1	ptsI	1	6.5	0.394
0.1	0.1	clcB	1	6.5	0.349

0.1	0.1	ycaM	1	6.5	0.325
0.1	0.1	yadI	1	6.5	0.436
0.1	0.1	AG1	1	6.5	0.415
0.1	0.1	ptsI	1	6.75	0.416
0.1	0.1	clcB	1	6.75	0.356
0.1	0.1	ycaM	1	6.75	0.333
0.1	0.1	yadI	1	6.75	0.444
0.1	0.1	AG1	1	6.75	0.421
0.1	0.1	ptsI	1	7	0.438
0.1	0.1	clcB	1	7	0.362
0.1	0.1	ycaM	1	7	0.341
0.1	0.1	yadI	1	7	0.45
0.1	0.1	AG1	1	7	0.427
0.1	0.1	ptsI	1	7.25	0.448
0.1	0.1	clcB	1	7.25	0.366
0.1	0.1	ycaM	1	7.25	0.349
0.1	0.1	yadI	1	7.25	0.461
0.1	0.1	AG1	1	7.25	0.437
0.1	0.1	ptsI	1	7.5	0.465
0.1	0.1	clcB	1	7.5	0.374
0.1	0.1	ycaM	1	7.5	0.355
0.1	0.1	yadI	1	7.5	0.473
0.1	0.1	AG1	1	7.5	0.444
0.1	0.1	ptsI	1	7.75	0.479
0.1	0.1	clcB	1	7.75	0.381
0.1	0.1	ycaM	1	7.75	0.362
0.1	0.1	yadI	1	7.75	0.484
0.1	0.1	AG1	1	7.75	0.458
0.1	0.1	ptsI	1	8	0.496
0.1	0.1	clcB	1	8	0.391
0.1	0.1	ycaM	1	8	0.367
0.1	0.1	yadI	1	8	0.488
0.1	0.1	AG1	1	8	0.466
0.1	0.1	ptsI	1	8.25	0.509
0.1	0.1	clcB	1	8.25	0.395
0.1	0.1	ycaM	1	8.25	0.371
0.1	0.1	yadI	1	8.25	0.5
0.1	0.1	AG1	1	8.25	0.478
0.1	0.1	ptsI	1	8.5	0.525
0.1	0.1	clcB	1	8.5	0.405
0.1	0.1	ycaM	1	8.5	0.372
0.1	0.1	yadI	1	8.5	0.51
0.1	0.1	AG1	1	8.5	0.492
0.1	0.1	ptsI	1	8.75	0.524
0.1	0.1	clcB	1	8.75	0.413
0.1	0.1	ycaM	1	8.75	0.374
0.1	0.1	yadI	1	8.75	0.518
0.1	0.1	AG1	1	8.75	0.502
0.1	0.1	ptsI	1	9	0.537
0.1	0.1	clcB	1	9	0.418
0.1	0.1	ycaM	1	9	0.376
0.1	0.1	yadI	1	9	0.524
0.1	0.1	AG1	1	9	0.513

0.1	0.1	ptsI	1	9.25	0.554
0.1	0.1	clcB	1	9.25	0.422
0.1	0.1	ycaM	1	9.25	0.384
0.1	0.1	yadI	1	9.25	0.524
0.1	0.1	AG1	1	9.25	0.536
0.1	0.1	ptsI	1	9.5	0.626
0.1	0.1	clcB	1	9.5	0.437
0.1	0.1	ycaM	1	9.5	0.392
0.1	0.1	yadI	1	9.5	0.538
0.1	0.1	AG1	1	9.5	0.58
0.1	0.1	ptsI	1	9.75	0.59
0.1	0.1	clcB	1	9.75	0.442
0.1	0.1	ycaM	1	9.75	0.398
0.1	0.1	yadI	1	9.75	0.546
0.1	0.1	AG1	1	9.75	0.587
0.1	0.1	ptsI	1	10	0.564
0.1	0.1	clcB	1	10	0.443
0.1	0.1	ycaM	1	10	0.402
0.1	0.1	yadI	1	10	0.552
0.1	0.1	AG1	1	10	0.583
0.1	0.1	ptsI	1	10.25	0.625
0.1	0.1	clcB	1	10.25	0.451
0.1	0.1	ycaM	1	10.25	0.407
0.1	0.1	yadI	1	10.25	0.562
0.1	0.1	AG1	1	10.25	0.593
0.1	0.1	ptsI	1	10.5	0.627
0.1	0.1	clcB	1	10.5	0.459
0.1	0.1	ycaM	1	10.5	0.42
0.1	0.1	yadI	1	10.5	0.567
0.1	0.1	AG1	1	10.5	0.603
0.1	0.1	ptsI	1	10.75	0.624
0.1	0.1	clcB	1	10.75	0.467
0.1	0.1	ycaM	1	10.75	0.423
0.1	0.1	yadI	1	10.75	0.576
0.1	0.1	AG1	1	10.75	0.61
0.1	0.1	ptsI	1	11	0.678
0.1	0.1	clcB	1	11	0.485
0.1	0.1	ycaM	1	11	0.444
0.1	0.1	yadI	1	11	0.591
0.1	0.1	AG1	1	11	0.697
0.1	0.1	ptsI	1	11.25	0.686
0.1	0.1	clcB	1	11.25	0.501
0.1	0.1	ycaM	1	11.25	0.444
0.1	0.1	yadI	1	11.25	0.597
0.1	0.1	AG1	1	11.25	0.675
0.1	0.1	ptsI	1	11.5	0.658
0.1	0.1	clcB	1	11.5	0.508
0.1	0.1	ycaM	1	11.5	0.454
0.1	0.1	yadI	1	11.5	0.61
0.1	0.1	AG1	1	11.5	0.738
0.1	0.1	ptsI	1	11.75	0.686
0.1	0.1	clcB	1	11.75	0.522
0.1	0.1	ycaM	1	11.75	0.459

0.1	0.1	yadI	1	11.75	0.634
0.1	0.1	AG1	1	11.75	0.793
0.1	0.1	ptsI	1	12	0.699
0.1	0.1	clcB	1	12	0.549
0.1	0.1	ycaM	1	12	0.472
0.1	0.1	yadI	1	12	0.642
0.1	0.1	AG1	1	12	0.784
0.1	0.1	ptsI	1	12.25	0.664
0.1	0.1	clcB	1	12.25	0.539
0.1	0.1	ycaM	1	12.25	0.462
0.1	0.1	yadI	1	12.25	0.635
0.1	0.1	AG1	1	12.25	0.828
0.1	0.1	ptsI	1	12.5	0.683
0.1	0.1	clcB	1	12.5	0.566
0.1	0.1	ycaM	1	12.5	0.469
0.1	0.1	yadI	1	12.5	0.637
0.1	0.1	AG1	1	12.5	0.814
0.1	0.1	ptsI	1	12.75	0.712
0.1	0.1	clcB	1	12.75	0.585
0.1	0.1	ycaM	1	12.75	0.473
0.1	0.1	yadI	1	12.75	0.64
0.1	0.1	AG1	1	12.75	0.822
0.1	0.1	ptsI	1	13	0.712
0.1	0.1	clcB	1	13	0.6
0.1	0.1	ycaM	1	13	0.481
0.1	0.1	yadI	1	13	0.647
0.1	0.1	AG1	1	13	0.834
0.1	0.1	ptsI	1	13.25	0.686
0.1	0.1	clcB	1	13.25	0.607
0.1	0.1	ycaM	1	13.25	0.482
0.1	0.1	yadI	1	13.25	0.648
0.1	0.1	AG1	1	13.25	0.852
0.1	0.1	ptsI	1	13.5	0.733
0.1	0.1	clcB	1	13.5	0.615
0.1	0.1	ycaM	1	13.5	0.497
0.1	0.1	yadI	1	13.5	0.663
0.1	0.1	AG1	1	13.5	0.854
0.1	0.1	ptsI	1	13.75	0.702
0.1	0.1	clcB	1	13.75	0.612
0.1	0.1	ycaM	1	13.75	0.501
0.1	0.1	yadI	1	13.75	0.661
0.1	0.1	AG1	1	13.75	0.853
0.1	0.1	ptsI	1	14	0.742
0.1	0.1	clcB	1	14	0.615
0.1	0.1	ycaM	1	14	0.517
0.1	0.1	yadI	1	14	0.666
0.1	0.1	AG1	1	14	0.875
0.1	0.1	ptsI	1	14.25	0.728
0.1	0.1	clcB	1	14.25	0.624
0.1	0.1	ycaM	1	14.25	0.534
0.1	0.1	yadI	1	14.25	0.673
0.1	0.1	AG1	1	14.25	0.887
0.1	0.1	ptsI	1	14.5	0.726

0.1	0.1	clcB	1	14.5	0.651
0.1	0.1	ycaM	1	14.5	0.558
0.1	0.1	yadI	1	14.5	0.678
0.1	0.1	AG1	1	14.5	0.882
0.1	0.1	ptsI	1	14.75	0.724
0.1	0.1	clcB	1	14.75	0.65
0.1	0.1	ycaM	1	14.75	0.561
0.1	0.1	yadI	1	14.75	0.675
0.1	0.1	AG1	1	14.75	0.9
0.1	0.1	ptsI	1	15	0.758
0.1	0.1	clcB	1	15	0.7
0.1	0.1	ycaM	1	15	0.588
0.1	0.1	yadI	1	15	0.679
0.1	0.1	AG1	1	15	0.901
0.1	0.1	ptsI	1	15.25	0.766
0.1	0.1	clcB	1	15.25	0.717
0.1	0.1	ycaM	1	15.25	0.612
0.1	0.1	yadI	1	15.25	0.68
0.1	0.1	AG1	1	15.25	0.912
0.1	0.1	ptsI	1	15.5	0.74
0.1	0.1	clcB	1	15.5	0.714
0.1	0.1	ycaM	1	15.5	0.601
0.1	0.1	yadI	1	15.5	0.686
0.1	0.1	AG1	1	15.5	0.899
0.1	0.1	ptsI	1	15.75	0.756
0.1	0.1	clcB	1	15.75	0.71
0.1	0.1	ycaM	1	15.75	0.615
0.1	0.1	yadI	1	15.75	0.69
0.1	0.1	AG1	1	15.75	0.892
0.1	0.1	ptsI	1	16	0.75
0.1	0.1	clcB	1	16	0.702
0.1	0.1	ycaM	1	16	0.611
0.1	0.1	yadI	1	16	0.697
0.1	0.1	AG1	1	16	0.908
0.1	0.1	ptsI	1	16.25	0.781
0.1	0.1	clcB	1	16.25	0.74
0.1	0.1	ycaM	1	16.25	0.619
0.1	0.1	yadI	1	16.25	0.704
0.1	0.1	AG1	1	16.25	0.917
0.1	0.1	ptsI	1	16.5	0.78
0.1	0.1	clcB	1	16.5	0.705
0.1	0.1	ycaM	1	16.5	0.608
0.1	0.1	yadI	1	16.5	0.711
0.1	0.1	AG1	1	16.5	0.909
0.1	0.1	ptsI	1	17.0667	0.67
0.1	0.1	clcB	1	17.0667	0.536
0.1	0.1	ycaM	1	17.0667	0.632
0.1	0.1	yadI	1	17.0667	0.842
0.1	0.1	AG1	1	17.0667	0.781
0.1	0.1	ptsI	1	17.3167	0.661
0.1	0.1	clcB	1	17.3167	0.49
0.1	0.1	ycaM	1	17.3167	0.593
0.1	0.1	yadI	1	17.3167	0.821

0.1	0.1	AG1	1	17.3167	0.747
0.1	0.1	ptsI	1	17.5667	0.661
0.1	0.1	clcB	1	17.5667	0.487
0.1	0.1	ycaM	1	17.5667	0.583
0.1	0.1	yadI	1	17.5667	0.826
0.1	0.1	AG1	1	17.5667	0.754
0.1	0.1	ptsI	1	17.8167	0.731
0.1	0.1	clcB	1	17.8167	0.49
0.1	0.1	ycaM	1	17.8167	0.584
0.1	0.1	yadI	1	17.8167	0.838
0.1	0.1	AG1	1	17.8167	0.807
0.1	0.1	ptsI	1	18.0667	0.714
0.1	0.1	clcB	1	18.0667	0.484
0.1	0.1	ycaM	1	18.0667	0.591
0.1	0.1	yadI	1	18.0667	0.853
0.1	0.1	AG1	1	18.0667	0.788
0.1	0.1	ptsI	1	18.3167	0.737
0.1	0.1	clcB	1	18.3167	0.488
0.1	0.1	ycaM	1	18.3167	0.576
0.1	0.1	yadI	1	18.3167	0.85
0.1	0.1	AG1	1	18.3167	0.802
0.1	0.1	ptsI	1	18.5667	0.78
0.1	0.1	clcB	1	18.5667	0.486
0.1	0.1	ycaM	1	18.5667	0.584
0.1	0.1	yadI	1	18.5667	0.86
0.1	0.1	AG1	1	18.5667	0.817
0.1	0.1	ptsI	1	18.8167	0.787
0.1	0.1	clcB	1	18.8167	0.49
0.1	0.1	ycaM	1	18.8167	0.576
0.1	0.1	yadI	1	18.8167	0.864
0.1	0.1	AG1	1	18.8167	0.823
0.1	0.1	ptsI	1	19.0667	0.812
0.1	0.1	clcB	1	19.0667	0.49
0.1	0.1	ycaM	1	19.0667	0.578
0.1	0.1	yadI	1	19.0667	0.875
0.1	0.1	AG1	1	19.0667	0.847
0.1	0.1	ptsI	1	19.3167	0.793
0.1	0.1	clcB	1	19.3167	0.489
0.1	0.1	ycaM	1	19.3167	0.582
0.1	0.1	yadI	1	19.3167	0.874
0.1	0.1	AG1	1	19.3167	0.834
0.1	0.1	ptsI	1	19.5667	0.813
0.1	0.1	clcB	1	19.5667	0.483
0.1	0.1	ycaM	1	19.5667	0.572
0.1	0.1	yadI	1	19.5667	0.881
0.1	0.1	AG1	1	19.5667	0.849
0.1	0.1	ptsI	1	19.8167	0.816
0.1	0.1	clcB	1	19.8167	0.477
0.1	0.1	ycaM	1	19.8167	0.575
0.1	0.1	yadI	1	19.8167	0.884
0.1	0.1	AG1	1	19.8167	0.84
0.1	0.1	ptsI	1	20.0667	0.818
0.1	0.1	clcB	1	20.0667	0.48

0.1	0.1	ycaM	1	20.0667	0.572
0.1	0.1	yadI	1	20.0667	0.888
0.1	0.1	AG1	1	20.0667	0.842
0.1	0.1	ptsI	1	20.3167	0.82
0.1	0.1	clcB	1	20.3167	0.473
0.1	0.1	ycaM	1	20.3167	0.574
0.1	0.1	yadI	1	20.3167	0.889
0.1	0.1	AG1	1	20.3167	0.841
0.1	0.1	ptsI	1	20.5667	0.807
0.1	0.1	clcB	1	20.5667	0.458
0.1	0.1	ycaM	1	20.5667	0.574
0.1	0.1	yadI	1	20.5667	0.896
0.1	0.1	AG1	1	20.5667	0.838
0.1	0.1	ptsI	1	20.8167	0.81
0.1	0.1	clcB	1	20.8167	0.456
0.1	0.1	ycaM	1	20.8167	0.57
0.1	0.1	yadI	1	20.8167	0.899
0.1	0.1	AG1	1	20.8167	0.845
0.1	0.1	ptsI	1	21.0667	0.828
0.1	0.1	clcB	1	21.0667	0.456
0.1	0.1	ycaM	1	21.0667	0.564
0.1	0.1	yadI	1	21.0667	0.898
0.1	0.1	AG1	1	21.0667	0.858
0.1	0.1	ptsI	1	21.3167	0.825
0.1	0.1	clcB	1	21.3167	0.451
0.1	0.1	ycaM	1	21.3167	0.572
0.1	0.1	yadI	1	21.3167	0.903
0.1	0.1	AG1	1	21.3167	0.856
0.1	0.1	ptsI	1	21.5667	0.827
0.1	0.1	clcB	1	21.5667	0.464
0.1	0.1	ycaM	1	21.5667	0.568
0.1	0.1	yadI	1	21.5667	0.912
0.1	0.1	AG1	1	21.5667	0.859
0.1	0.1	ptsI	1	21.8167	0.83
0.1	0.1	clcB	1	21.8167	0.46
0.1	0.1	ycaM	1	21.8167	0.569
0.1	0.1	yadI	1	21.8167	0.913
0.1	0.1	AG1	1	21.8167	0.869
0.1	0.1	ptsI	1	22.0667	0.838
0.1	0.1	clcB	1	22.0667	0.456
0.1	0.1	ycaM	1	22.0667	0.566
0.1	0.1	yadI	1	22.0667	0.914
0.1	0.1	AG1	1	22.0667	0.874
0.1	0.1	ptsI	1	22.9333	0.827
0.1	0.1	clcB	1	22.9333	0.488
0.1	0.1	ycaM	1	22.9333	0.41
0.1	0.1	yadI	1	22.9333	0.953
0.1	0.1	AG1	1	22.9333	0.865
0.1	0.1	ptsI	1	23.1833	0.798
0.1	0.1	clcB	1	23.1833	0.476
0.1	0.1	ycaM	1	23.1833	0.404
0.1	0.1	yadI	1	23.1833	0.933
0.1	0.1	AG1	1	23.1833	0.841

0.1	0.1	ptsI	1	23.4333	0.792
0.1	0.1	clcB	1	23.4333	0.473
0.1	0.1	ycaM	1	23.4333	0.406
0.1	0.1	yadI	1	23.4333	0.941
0.1	0.1	AG1	1	23.4333	0.847
0.1	0.1	ptsI	1	23.6833	0.807
0.1	0.1	clcB	1	23.6833	0.466
0.1	0.1	ycaM	1	23.6833	0.407
0.1	0.1	yadI	1	23.6833	0.953
0.1	0.1	AG1	1	23.6833	0.865
0.1	0.1	ptsI	1	23.9333	0.807
0.1	0.1	clcB	1	23.9333	0.472
0.1	0.1	ycaM	1	23.9333	0.41
0.1	0.1	yadI	1	23.9333	0.955
0.1	0.1	AG1	1	23.9333	0.867
0.1	0.1	ptsI	1	24.1833	0.808
0.1	0.1	clcB	1	24.1833	0.461
0.1	0.1	ycaM	1	24.1833	0.407
0.1	0.1	yadI	1	24.1833	0.964
0.1	0.1	AG1	1	24.1833	0.868
0.1	0.1	ptsI	1	24.4333	0.833
0.1	0.1	clcB	1	24.4333	0.461
0.1	0.1	ycaM	1	24.4333	0.408
0.1	0.1	yadI	1	24.4333	0.968
0.1	0.1	AG1	1	24.4333	0.868
0.1	0.1	ptsI	1	24.6833	0.831
0.1	0.1	clcB	1	24.6833	0.448
0.1	0.1	ycaM	1	24.6833	0.406
0.1	0.1	yadI	1	24.6833	0.974
0.1	0.1	AG1	1	24.6833	0.868
0.1	0.1	ptsI	1	24.9333	0.839
0.1	0.1	clcB	1	24.9333	0.502
0.1	0.1	ycaM	1	24.9333	0.408
0.1	0.1	yadI	1	24.9333	0.976
0.1	0.1	AG1	1	24.9333	0.876
0.1	0.1	ptsI	1	25.1833	0.837
0.1	0.1	clcB	1	25.1833	0.445
0.1	0.1	ycaM	1	25.1833	0.407
0.1	0.1	yadI	1	25.1833	0.976
0.1	0.1	AG1	1	25.1833	0.87
0.1	0.1	ptsI	1	25.4333	0.844
0.1	0.1	clcB	1	25.4333	0.452
0.1	0.1	ycaM	1	25.4333	0.409
0.1	0.1	yadI	1	25.4333	0.984
0.1	0.1	AG1	1	25.4333	0.876
0.1	0.1	ptsI	1	25.6833	0.84
0.1	0.1	clcB	1	25.6833	0.45
0.1	0.1	ycaM	1	25.6833	0.408
0.1	0.1	yadI	1	25.6833	0.988
0.1	0.1	AG1	1	25.6833	0.873
0.1	0.1	ptsI	1	25.9333	0.854
0.1	0.1	clcB	1	25.9333	0.449
0.1	0.1	ycaM	1	25.9333	0.41

0.1	0.1	yadI	1	25.9333	0.982
0.1	0.1	AG1	1	25.9333	0.881
0.1	0.1	ptsI	1	26.1833	0.855
0.1	0.1	clcB	1	26.1833	0.45
0.1	0.1	ycaM	1	26.1833	0.414
0.1	0.1	yadI	1	26.1833	0.998
0.1	0.1	AG1	1	26.1833	0.884
0.1	0.1	ptsI	1	26.4333	0.853
0.1	0.1	clcB	1	26.4333	0.447
0.1	0.1	ycaM	1	26.4333	0.414
0.1	0.1	yadI	1	26.4333	0.994
0.1	0.1	AG1	1	26.4333	0.877
0.1	0.1	ptsI	1	26.6833	0.856
0.1	0.1	clcB	1	26.6833	0.45
0.1	0.1	ycaM	1	26.6833	0.412
0.1	0.1	yadI	1	26.6833	1.005
0.1	0.1	AG1	1	26.6833	0.884
0.1	0.1	ptsI	1	26.9333	0.862
0.1	0.1	clcB	1	26.9333	0.449
0.1	0.1	ycaM	1	26.9333	0.414
0.1	0.1	yadI	1	26.9333	1
0.1	0.1	AG1	1	26.9333	0.883
0.1	0.1	ptsI	1	27.1833	0.866
0.1	0.1	clcB	1	27.1833	0.448
0.1	0.1	ycaM	1	27.1833	0.413
0.1	0.1	yadI	1	27.1833	1.005
0.1	0.1	AG1	1	27.1833	0.89
0.1	0.1	ptsI	1	27.4333	0.859
0.1	0.1	clcB	1	27.4333	0.448
0.1	0.1	ycaM	1	27.4333	0.412
0.1	0.1	yadI	1	27.4333	1.01
0.1	0.1	AG1	1	27.4333	0.887
0.1	0.1	ptsI	1	27.6833	0.867
0.1	0.1	clcB	1	27.6833	0.451
0.1	0.1	ycaM	1	27.6833	0.413
0.1	0.1	yadI	1	27.6833	1.011
0.1	0.1	AG1	1	27.6833	0.894
0.1	0.1	ptsI	1	27.9333	0.86
0.1	0.1	clcB	1	27.9333	0.444
0.1	0.1	ycaM	1	27.9333	0.403
0.1	0.1	yadI	1	27.9333	0.992
0.1	0.1	AG1	1	27.9333	0.892
0.1	0.1	ptsI	1	28.1833	0.88
0.1	0.1	clcB	1	28.1833	0.447
0.1	0.1	ycaM	1	28.1833	0.415
0.1	0.1	yadI	1	28.1833	1.013
0.1	0.1	AG1	1	28.1833	0.906
0.1	0.1	ptsI	1	28.4333	0.879
0.1	0.1	clcB	1	28.4333	0.449
0.1	0.1	ycaM	1	28.4333	0.416
0.1	0.1	yadI	1	28.4333	1.013
0.1	0.1	AG1	1	28.4333	0.905
0.1	0.1	ptsI	1	28.6833	0.873

0.1	0.1	clcB	1	28.6833	0.448
0.1	0.1	ycaM	1	28.6833	0.416
0.1	0.1	yadI	1	28.6833	1.018
0.1	0.1	AG1	1	28.6833	0.912
0.1	0.1	ptsI	1	28.9333	0.881
0.1	0.1	clcB	1	28.9333	0.449
0.1	0.1	ycaM	1	28.9333	0.417
0.1	0.1	yadI	1	28.9333	1.02
0.1	0.1	AG1	1	28.9333	0.918
0.1	0.1	ptsI	1	29.1833	0.87
0.1	0.1	clcB	1	29.1833	0.45
0.1	0.1	ycaM	1	29.1833	0.416
0.1	0.1	yadI	1	29.1833	1.025
0.1	0.1	AG1	1	29.1833	0.914
0.1	0.1	ptsI	1	29.4333	0.878
0.1	0.1	clcB	1	29.4333	0.451
0.1	0.1	ycaM	1	29.4333	0.418
0.1	0.1	yadI	1	29.4333	1.026
0.1	0.1	AG1	1	29.4333	0.919
0.1	0.1	ptsI	1	29.6833	0.876
0.1	0.1	clcB	1	29.6833	0.452
0.1	0.1	ycaM	1	29.6833	0.42
0.1	0.1	yadI	1	29.6833	1.029
0.1	0.1	AG1	1	29.6833	0.924
0.1	0.1	ptsI	1	29.9333	0.88
0.1	0.1	clcB	1	29.9333	0.453
0.1	0.1	ycaM	1	29.9333	0.418
0.1	0.1	yadI	1	29.9333	1.028
0.1	0.1	AG1	1	29.9333	0.931
0.1	0.1	ptsI	1	30.1833	0.884
0.1	0.1	clcB	1	30.1833	0.454
0.1	0.1	ycaM	1	30.1833	0.418
0.1	0.1	yadI	1	30.1833	1.03
0.1	0.1	AG1	1	30.1833	0.938
0.1	0.1	ptsI	1	30.4333	0.887
0.1	0.1	clcB	1	30.4333	0.451
0.1	0.1	ycaM	1	30.4333	0.419
0.1	0.1	yadI	1	30.4333	1.031
0.1	0.1	AG1	1	30.4333	0.944
0.1	0.1	ptsI	1	30.6833	0.877
0.1	0.1	clcB	1	30.6833	0.456
0.1	0.1	ycaM	1	30.6833	0.422
0.1	0.1	yadI	1	30.6833	1.041
0.1	0.1	AG1	1	30.6833	0.943
0.1	0.1	ptsI	1	30.9333	0.884
0.1	0.1	clcB	1	30.9333	0.458
0.1	0.1	ycaM	1	30.9333	0.422
0.1	0.1	yadI	1	30.9333	1.042
0.1	0.1	AG1	1	30.9333	0.951
0.1	0.1	ptsI	1	31.1833	0.88
0.1	0.1	clcB	1	31.1833	0.454
0.1	0.1	ycaM	1	31.1833	0.423
0.1	0.1	yadI	1	31.1833	1.042

0.1	0.1	AG1	1	31.1833	0.952
0.1	0.1	ptsI	1	31.4333	0.888
0.1	0.1	clcB	1	31.4333	0.454
0.1	0.1	ycaM	1	31.4333	0.426
0.1	0.1	yadI	1	31.4333	1.044
0.1	0.1	AG1	1	31.4333	0.962
0.1	0.1	ptsI	1	31.6833	0.886
0.1	0.1	clcB	1	31.6833	0.456
0.1	0.1	ycaM	1	31.6833	0.427
0.1	0.1	yadI	1	31.6833	1.046
0.1	0.1	AG1	1	31.6833	0.967
0.1	0.1	ptsI	1	31.9333	0.888
0.1	0.1	clcB	1	31.9333	0.459
0.1	0.1	ycaM	1	31.9333	0.423
0.1	0.1	yadI	1	31.9333	1.049
0.1	0.1	AG1	1	31.9333	0.972
0.1	0.1	ptsI	1	32.1833	0.891
0.1	0.1	clcB	1	32.1833	0.456
0.1	0.1	ycaM	1	32.1833	0.428
0.1	0.1	yadI	1	32.1833	1.048
0.1	0.1	AG1	1	32.1833	0.976
0.1	0.1	ptsI	1	32.4333	0.89
0.1	0.1	clcB	1	32.4333	0.451
0.1	0.1	ycaM	1	32.4333	0.417
0.1	0.1	yadI	1	32.4333	1.032
0.1	0.1	AG1	1	32.4333	0.982
0.1	0.1	ptsI	1	32.6833	0.89
0.1	0.1	clcB	1	32.6833	0.459
0.1	0.1	ycaM	1	32.6833	0.432
0.1	0.1	yadI	1	32.6833	1.055
0.1	0.1	AG1	1	32.6833	0.987
0.1	0.1	ptsI	1	32.9333	0.886
0.1	0.1	clcB	1	32.9333	0.455
0.1	0.1	ycaM	1	32.9333	0.427
0.1	0.1	yadI	1	32.9333	1.057
0.1	0.1	AG1	1	32.9333	0.995
0.1	0.1	ptsI	1	33.1833	0.886
0.1	0.1	clcB	1	33.1833	0.455
0.1	0.1	ycaM	1	33.1833	0.422
0.1	0.1	yadI	1	33.1833	1.049
0.1	0.1	AG1	1	33.1833	1.001
0.1	0.1	ptsI	1	33.4333	0.891
0.1	0.1	clcB	1	33.4333	0.46
0.1	0.1	ycaM	1	33.4333	0.43
0.1	0.1	yadI	1	33.4333	1.06
0.1	0.1	AG1	1	33.4333	1.013
0.1	0.1	ptsI	1	33.6833	0.886
0.1	0.1	clcB	1	33.6833	0.454
0.1	0.1	ycaM	1	33.6833	0.426
0.1	0.1	yadI	1	33.6833	1.046
0.1	0.1	AG1	1	33.6833	1.016
0.1	0.1	ptsI	1	33.9333	0.888
0.1	0.1	clcB	1	33.9333	0.461

0.1	0.1	ycaM	1	33.9333	0.432
0.1	0.1	yadI	1	33.9333	1.063
0.1	0.1	AG1	1	33.9333	1.026
0.1	0.1	ptsI	1	34.1833	0.881
0.1	0.1	clcB	1	34.1833	0.467
0.1	0.1	ycaM	1	34.1833	0.432
0.1	0.1	yadI	1	34.1833	1.069
0.1	0.1	AG1	1	34.1833	1.028
0.1	0.1	ptsI	1	34.4333	0.891
0.1	0.1	clcB	1	34.4333	0.465
0.1	0.1	ycaM	1	34.4333	0.433
0.1	0.1	yadI	1	34.4333	1.07
0.1	0.1	AG1	1	34.4333	1.042
0.1	0.1	ptsI	1	34.6833	0.892
0.1	0.1	clcB	1	34.6833	0.466
0.1	0.1	ycaM	1	34.6833	0.434
0.1	0.1	yadI	1	34.6833	1.066
0.1	0.1	AG1	1	34.6833	1.054
0.1	0.1	ptsI	1	34.9333	0.888
0.1	0.1	clcB	1	34.9333	0.47
0.1	0.1	ycaM	1	34.9333	0.433
0.1	0.1	yadI	1	34.9333	1.065
0.1	0.1	AG1	1	34.9333	1.056
0.1	0.1	ptsI	1	35.1833	0.886
0.1	0.1	clcB	1	35.1833	0.467
0.1	0.1	ycaM	1	35.1833	0.435
0.1	0.1	yadI	1	35.1833	1.067
0.1	0.1	AG1	1	35.1833	1.062
0.1	0.1	ptsI	1	35.4333	0.888
0.1	0.1	clcB	1	35.4333	0.466
0.1	0.1	ycaM	1	35.4333	0.435
0.1	0.1	yadI	1	35.4333	1.068
0.1	0.1	AG1	1	35.4333	1.072
0.1	0.1	ptsI	1	35.6833	0.888
0.1	0.1	clcB	1	35.6833	0.472
0.1	0.1	ycaM	1	35.6833	0.437
0.1	0.1	yadI	1	35.6833	1.069
0.1	0.1	AG1	1	35.6833	1.076
0.1	0.1	ptsI	1	35.9333	0.883
0.1	0.1	clcB	1	35.9333	0.467
0.1	0.1	ycaM	1	35.9333	0.43
0.1	0.1	yadI	1	35.9333	1.061
0.1	0.1	AG1	1	35.9333	1.083
0.1	0.1	ptsI	1	36.1833	0.889
0.1	0.1	clcB	1	36.1833	0.472
0.1	0.1	ycaM	1	36.1833	0.442
0.1	0.1	yadI	1	36.1833	1.073
0.1	0.1	AG1	1	36.1833	1.092
0.1	0.1	ptsI	1	36.4333	0.888
0.1	0.1	clcB	1	36.4333	0.471
0.1	0.1	ycaM	1	36.4333	0.44
0.1	0.1	yadI	1	36.4333	1.073
0.1	0.1	AG1	1	36.4333	1.099

0.1	0.1	ptsI	1	36.6833	0.885
0.1	0.1	clcB	1	36.6833	0.472
0.1	0.1	ycaM	1	36.6833	0.439
0.1	0.1	yadI	1	36.6833	1.076
0.1	0.1	AG1	1	36.6833	1.103
0.2	0.1	ptsI	1	0	0.234
0.2	0.1	clcB	1	0	0.25
0.2	0.1	ycaM	1	0	0.232
0.2	0.1	yadI	1	0	0.28
0.2	0.1	AG1	1	0	0.259
0.2	0.1	ptsI	1	0.25	0.224
0.2	0.1	clcB	1	0.25	0.238
0.2	0.1	ycaM	1	0.25	0.216
0.2	0.1	yadI	1	0.25	0.265
0.2	0.1	AG1	1	0.25	0.245
0.2	0.1	ptsI	1	0.5	0.224
0.2	0.1	clcB	1	0.5	0.232
0.2	0.1	ycaM	1	0.5	0.213
0.2	0.1	yadI	1	0.5	0.262
0.2	0.1	AG1	1	0.5	0.238
0.2	0.1	ptsI	1	0.75	0.223
0.2	0.1	clcB	1	0.75	0.231
0.2	0.1	ycaM	1	0.75	0.211
0.2	0.1	yadI	1	0.75	0.261
0.2	0.1	AG1	1	0.75	0.235
0.2	0.1	ptsI	1	1	0.223
0.2	0.1	clcB	1	1	0.231
0.2	0.1	ycaM	1	1	0.213
0.2	0.1	yadI	1	1	0.261
0.2	0.1	AG1	1	1	0.238
0.2	0.1	ptsI	1	1.25	0.225
0.2	0.1	clcB	1	1.25	0.233
0.2	0.1	ycaM	1	1.25	0.21
0.2	0.1	yadI	1	1.25	0.258
0.2	0.1	AG1	1	1.25	0.237
0.2	0.1	ptsI	1	1.5	0.229
0.2	0.1	clcB	1	1.5	0.234
0.2	0.1	ycaM	1	1.5	0.21
0.2	0.1	yadI	1	1.5	0.26
0.2	0.1	AG1	1	1.5	0.239
0.2	0.1	ptsI	1	1.75	0.233
0.2	0.1	clcB	1	1.75	0.235
0.2	0.1	ycaM	1	1.75	0.214
0.2	0.1	yadI	1	1.75	0.262
0.2	0.1	AG1	1	1.75	0.245
0.2	0.1	ptsI	1	2	0.242
0.2	0.1	clcB	1	2	0.237
0.2	0.1	ycaM	1	2	0.217
0.2	0.1	yadI	1	2	0.268
0.2	0.1	AG1	1	2	0.249
0.2	0.1	ptsI	1	2.25	0.247
0.2	0.1	clcB	1	2.25	0.24
0.2	0.1	ycaM	1	2.25	0.221

0.2	0.1	yadI	1	2.25	0.272
0.2	0.1	AG1	1	2.25	0.255
0.2	0.1	ptsI	1	2.5	0.258
0.2	0.1	clcB	1	2.5	0.248
0.2	0.1	ycaM	1	2.5	0.226
0.2	0.1	yadI	1	2.5	0.28
0.2	0.1	AG1	1	2.5	0.258
0.2	0.1	ptsI	1	2.75	0.263
0.2	0.1	clcB	1	2.75	0.255
0.2	0.1	ycaM	1	2.75	0.233
0.2	0.1	yadI	1	2.75	0.289
0.2	0.1	AG1	1	2.75	0.263
0.2	0.1	ptsI	1	3	0.278
0.2	0.1	clcB	1	3	0.259
0.2	0.1	ycaM	1	3	0.236
0.2	0.1	yadI	1	3	0.301
0.2	0.1	AG1	1	3	0.267
0.2	0.1	ptsI	1	3.25	0.291
0.2	0.1	clcB	1	3.25	0.265
0.2	0.1	ycaM	1	3.25	0.243
0.2	0.1	yadI	1	3.25	0.31
0.2	0.1	AG1	1	3.25	0.275
0.2	0.1	ptsI	1	3.5	0.308
0.2	0.1	clcB	1	3.5	0.27
0.2	0.1	ycaM	1	3.5	0.248
0.2	0.1	yadI	1	3.5	0.321
0.2	0.1	AG1	1	3.5	0.28
0.2	0.1	ptsI	1	3.75	0.327
0.2	0.1	clcB	1	3.75	0.278
0.2	0.1	ycaM	1	3.75	0.257
0.2	0.1	yadI	1	3.75	0.33
0.2	0.1	AG1	1	3.75	0.288
0.2	0.1	ptsI	1	4	0.354
0.2	0.1	clcB	1	4	0.288
0.2	0.1	ycaM	1	4	0.272
0.2	0.1	yadI	1	4	0.357
0.2	0.1	AG1	1	4	0.299
0.2	0.1	ptsI	1	4.25	0.363
0.2	0.1	clcB	1	4.25	0.292
0.2	0.1	ycaM	1	4.25	0.268
0.2	0.1	yadI	1	4.25	0.352
0.2	0.1	AG1	1	4.25	0.305
0.2	0.1	ptsI	1	4.5	0.375
0.2	0.1	clcB	1	4.5	0.3
0.2	0.1	ycaM	1	4.5	0.275
0.2	0.1	yadI	1	4.5	0.364
0.2	0.1	AG1	1	4.5	0.31
0.2	0.1	ptsI	1	4.75	0.38
0.2	0.1	clcB	1	4.75	0.31
0.2	0.1	ycaM	1	4.75	0.28
0.2	0.1	yadI	1	4.75	0.375
0.2	0.1	AG1	1	4.75	0.32
0.2	0.1	ptsI	1	5	0.402

0.2	0.1	clcB	1	5	0.317
0.2	0.1	ycaM	1	5	0.289
0.2	0.1	yadI	1	5	0.385
0.2	0.1	AG1	1	5	0.328
0.2	0.1	ptsI	1	5.25	0.411
0.2	0.1	clcB	1	5.25	0.326
0.2	0.1	ycaM	1	5.25	0.292
0.2	0.1	yadI	1	5.25	0.396
0.2	0.1	AG1	1	5.25	0.333
0.2	0.1	ptsI	1	5.5	0.42
0.2	0.1	clcB	1	5.5	0.328
0.2	0.1	ycaM	1	5.5	0.298
0.2	0.1	yadI	1	5.5	0.407
0.2	0.1	AG1	1	5.5	0.341
0.2	0.1	ptsI	1	5.75	0.43
0.2	0.1	clcB	1	5.75	0.335
0.2	0.1	ycaM	1	5.75	0.309
0.2	0.1	yadI	1	5.75	0.416
0.2	0.1	AG1	1	5.75	0.35
0.2	0.1	ptsI	1	6	0.418
0.2	0.1	clcB	1	6	0.346
0.2	0.1	ycaM	1	6	0.31
0.2	0.1	yadI	1	6	0.427
0.2	0.1	AG1	1	6	0.352
0.2	0.1	ptsI	1	6.25	0.425
0.2	0.1	clcB	1	6.25	0.352
0.2	0.1	ycaM	1	6.25	0.317
0.2	0.1	yadI	1	6.25	0.438
0.2	0.1	AG1	1	6.25	0.353
0.2	0.1	ptsI	1	6.5	0.435
0.2	0.1	clcB	1	6.5	0.365
0.2	0.1	ycaM	1	6.5	0.32
0.2	0.1	yadI	1	6.5	0.45
0.2	0.1	AG1	1	6.5	0.367
0.2	0.1	ptsI	1	6.75	0.456
0.2	0.1	clcB	1	6.75	0.369
0.2	0.1	ycaM	1	6.75	0.328
0.2	0.1	yadI	1	6.75	0.459
0.2	0.1	AG1	1	6.75	0.378
0.2	0.1	ptsI	1	7	0.474
0.2	0.1	clcB	1	7	0.375
0.2	0.1	ycaM	1	7	0.334
0.2	0.1	yadI	1	7	0.468
0.2	0.1	AG1	1	7	0.388
0.2	0.1	ptsI	1	7.25	0.491
0.2	0.1	clcB	1	7.25	0.384
0.2	0.1	ycaM	1	7.25	0.341
0.2	0.1	yadI	1	7.25	0.474
0.2	0.1	AG1	1	7.25	0.398
0.2	0.1	ptsI	1	7.5	0.507
0.2	0.1	clcB	1	7.5	0.389
0.2	0.1	ycaM	1	7.5	0.347
0.2	0.1	yadI	1	7.5	0.487

0.2	0.1	AG1	1	7.5	0.408
0.2	0.1	ptsI	1	7.75	0.51
0.2	0.1	clcB	1	7.75	0.403
0.2	0.1	ycaM	1	7.75	0.352
0.2	0.1	yadI	1	7.75	0.499
0.2	0.1	AG1	1	7.75	0.422
0.2	0.1	ptsI	1	8	0.524
0.2	0.1	clcB	1	8	0.405
0.2	0.1	ycaM	1	8	0.355
0.2	0.1	yadI	1	8	0.506
0.2	0.1	AG1	1	8	0.428
0.2	0.1	ptsI	1	8.25	0.534
0.2	0.1	clcB	1	8.25	0.411
0.2	0.1	ycaM	1	8.25	0.359
0.2	0.1	yadI	1	8.25	0.511
0.2	0.1	AG1	1	8.25	0.439
0.2	0.1	ptsI	1	8.5	0.538
0.2	0.1	clcB	1	8.5	0.419
0.2	0.1	ycaM	1	8.5	0.359
0.2	0.1	yadI	1	8.5	0.518
0.2	0.1	AG1	1	8.5	0.45
0.2	0.1	ptsI	1	8.75	0.536
0.2	0.1	clcB	1	8.75	0.424
0.2	0.1	ycaM	1	8.75	0.361
0.2	0.1	yadI	1	8.75	0.526
0.2	0.1	AG1	1	8.75	0.456
0.2	0.1	ptsI	1	9	0.539
0.2	0.1	clcB	1	9	0.428
0.2	0.1	ycaM	1	9	0.364
0.2	0.1	yadI	1	9	0.536
0.2	0.1	AG1	1	9	0.47
0.2	0.1	ptsI	1	9.25	0.524
0.2	0.1	clcB	1	9.25	0.438
0.2	0.1	ycaM	1	9.25	0.371
0.2	0.1	yadI	1	9.25	0.537
0.2	0.1	AG1	1	9.25	0.496
0.2	0.1	ptsI	1	9.5	0.566
0.2	0.1	clcB	1	9.5	0.449
0.2	0.1	ycaM	1	9.5	0.382
0.2	0.1	yadI	1	9.5	0.565
0.2	0.1	AG1	1	9.5	0.579
0.2	0.1	ptsI	1	9.75	0.579
0.2	0.1	clcB	1	9.75	0.45
0.2	0.1	ycaM	1	9.75	0.388
0.2	0.1	yadI	1	9.75	0.57
0.2	0.1	AG1	1	9.75	0.596
0.2	0.1	ptsI	1	10	0.54
0.2	0.1	clcB	1	10	0.454
0.2	0.1	ycaM	1	10	0.391
0.2	0.1	yadI	1	10	0.566
0.2	0.1	AG1	1	10	0.607
0.2	0.1	ptsI	1	10.25	0.591
0.2	0.1	clcB	1	10.25	0.462

0.2	0.1	ycaM	1	10.25	0.389
0.2	0.1	yadI	1	10.25	0.57
0.2	0.1	AG1	1	10.25	0.625
0.2	0.1	ptsI	1	10.5	0.631
0.2	0.1	clcB	1	10.5	0.477
0.2	0.1	ycaM	1	10.5	0.398
0.2	0.1	yadI	1	10.5	0.58
0.2	0.1	AG1	1	10.5	0.641
0.2	0.1	ptsI	1	10.75	0.597
0.2	0.1	clcB	1	10.75	0.482
0.2	0.1	ycaM	1	10.75	0.402
0.2	0.1	yadI	1	10.75	0.572
0.2	0.1	AG1	1	10.75	0.572
0.2	0.1	ptsI	1	11	0.617
0.2	0.1	clcB	1	11	0.509
0.2	0.1	ycaM	1	11	0.415
0.2	0.1	yadI	1	11	0.6
0.2	0.1	AG1	1	11	0.661
0.2	0.1	ptsI	1	11.25	0.663
0.2	0.1	clcB	1	11.25	0.514
0.2	0.1	ycaM	1	11.25	0.421
0.2	0.1	yadI	1	11.25	0.607
0.2	0.1	AG1	1	11.25	0.69
0.2	0.1	ptsI	1	11.5	0.632
0.2	0.1	clcB	1	11.5	0.516
0.2	0.1	ycaM	1	11.5	0.426
0.2	0.1	yadI	1	11.5	0.616
0.2	0.1	AG1	1	11.5	0.734
0.2	0.1	ptsI	1	11.75	0.638
0.2	0.1	clcB	1	11.75	0.533
0.2	0.1	ycaM	1	11.75	0.437
0.2	0.1	yadI	1	11.75	0.616
0.2	0.1	AG1	1	11.75	0.713
0.2	0.1	ptsI	1	12	0.678
0.2	0.1	clcB	1	12	0.553
0.2	0.1	ycaM	1	12	0.445
0.2	0.1	yadI	1	12	0.622
0.2	0.1	AG1	1	12	0.734
0.2	0.1	ptsI	1	12.25	0.641
0.2	0.1	clcB	1	12.25	0.551
0.2	0.1	ycaM	1	12.25	0.447
0.2	0.1	yadI	1	12.25	0.589
0.2	0.1	AG1	1	12.25	0.697
0.2	0.1	ptsI	1	12.5	0.68
0.2	0.1	clcB	1	12.5	0.568
0.2	0.1	ycaM	1	12.5	0.448
0.2	0.1	yadI	1	12.5	0.585
0.2	0.1	AG1	1	12.5	0.745
0.2	0.1	ptsI	1	12.75	0.694
0.2	0.1	clcB	1	12.75	0.596
0.2	0.1	ycaM	1	12.75	0.454
0.2	0.1	yadI	1	12.75	0.583
0.2	0.1	AG1	1	12.75	0.767

0.2	0.1	ptsI	1	13	0.7
0.2	0.1	clcB	1	13	0.62
0.2	0.1	ycaM	1	13	0.462
0.2	0.1	yadI	1	13	0.588
0.2	0.1	AG1	1	13	0.792
0.2	0.1	ptsI	1	13.25	0.687
0.2	0.1	clcB	1	13.25	0.628
0.2	0.1	ycaM	1	13.25	0.47
0.2	0.1	yadI	1	13.25	0.583
0.2	0.1	AG1	1	13.25	0.798
0.2	0.1	ptsI	1	13.5	0.718
0.2	0.1	clcB	1	13.5	0.631
0.2	0.1	ycaM	1	13.5	0.487
0.2	0.1	yadI	1	13.5	0.595
0.2	0.1	AG1	1	13.5	0.823
0.2	0.1	ptsI	1	13.75	0.681
0.2	0.1	clcB	1	13.75	0.616
0.2	0.1	ycaM	1	13.75	0.501
0.2	0.1	yadI	1	13.75	0.598
0.2	0.1	AG1	1	13.75	0.816
0.2	0.1	ptsI	1	14	0.736
0.2	0.1	clcB	1	14	0.627
0.2	0.1	ycaM	1	14	0.53
0.2	0.1	yadI	1	14	0.617
0.2	0.1	AG1	1	14	0.864
0.2	0.1	ptsI	1	14.25	0.703
0.2	0.1	clcB	1	14.25	0.669
0.2	0.1	ycaM	1	14.25	0.556
0.2	0.1	yadI	1	14.25	0.615
0.2	0.1	AG1	1	14.25	0.868
0.2	0.1	ptsI	1	14.5	0.679
0.2	0.1	clcB	1	14.5	0.673
0.2	0.1	ycaM	1	14.5	0.58
0.2	0.1	yadI	1	14.5	0.619
0.2	0.1	AG1	1	14.5	0.872
0.2	0.1	ptsI	1	14.75	0.698
0.2	0.1	clcB	1	14.75	0.68
0.2	0.1	ycaM	1	14.75	0.645
0.2	0.1	yadI	1	14.75	0.613
0.2	0.1	AG1	1	14.75	0.902
0.2	0.1	ptsI	1	15	0.754
0.2	0.1	clcB	1	15	0.697
0.2	0.1	ycaM	1	15	0.586
0.2	0.1	yadI	1	15	0.616
0.2	0.1	AG1	1	15	0.885
0.2	0.1	ptsI	1	15.25	0.763
0.2	0.1	clcB	1	15.25	0.712
0.2	0.1	ycaM	1	15.25	0.598
0.2	0.1	yadI	1	15.25	0.627
0.2	0.1	AG1	1	15.25	0.904
0.2	0.1	ptsI	1	15.5	0.758
0.2	0.1	clcB	1	15.5	0.7
0.2	0.1	ycaM	1	15.5	0.569

0.2	0.1	yadI	1	15.5	0.629
0.2	0.1	AG1	1	15.5	0.896
0.2	0.1	ptsI	1	15.75	0.741
0.2	0.1	clcB	1	15.75	0.694
0.2	0.1	ycaM	1	15.75	0.578
0.2	0.1	yadI	1	15.75	0.626
0.2	0.1	AG1	1	15.75	0.922
0.2	0.1	ptsI	1	16	0.74
0.2	0.1	clcB	1	16	0.693
0.2	0.1	ycaM	1	16	0.574
0.2	0.1	yadI	1	16	0.607
0.2	0.1	AG1	1	16	0.922
0.2	0.1	ptsI	1	16.25	0.798
0.2	0.1	clcB	1	16.25	0.714
0.2	0.1	ycaM	1	16.25	0.556
0.2	0.1	yadI	1	16.25	0.618
0.2	0.1	AG1	1	16.25	0.923
0.2	0.1	ptsI	1	16.5	0.794
0.2	0.1	clcB	1	16.5	0.693
0.2	0.1	ycaM	1	16.5	0.559
0.2	0.1	yadI	1	16.5	0.63
0.2	0.1	AG1	1	16.5	0.923
0.2	0.1	ptsI	1	17.0667	0.588
0.2	0.1	clcB	1	17.0667	0.52
0.2	0.1	ycaM	1	17.0667	0.498
0.2	0.1	yadI	1	17.0667	0.749
0.2	0.1	AG1	1	17.0667	0.787
0.2	0.1	ptsI	1	17.3167	0.642
0.2	0.1	clcB	1	17.3167	0.48
0.2	0.1	ycaM	1	17.3167	0.47
0.2	0.1	yadI	1	17.3167	0.729
0.2	0.1	AG1	1	17.3167	0.746
0.2	0.1	ptsI	1	17.5667	0.653
0.2	0.1	clcB	1	17.5667	0.474
0.2	0.1	ycaM	1	17.5667	0.469
0.2	0.1	yadI	1	17.5667	0.722
0.2	0.1	AG1	1	17.5667	0.757
0.2	0.1	ptsI	1	17.8167	0.726
0.2	0.1	clcB	1	17.8167	0.467
0.2	0.1	ycaM	1	17.8167	0.477
0.2	0.1	yadI	1	17.8167	0.732
0.2	0.1	AG1	1	17.8167	0.814
0.2	0.1	ptsI	1	18.0667	0.66
0.2	0.1	clcB	1	18.0667	0.471
0.2	0.1	ycaM	1	18.0667	0.474
0.2	0.1	yadI	1	18.0667	0.754
0.2	0.1	AG1	1	18.0667	0.785
0.2	0.1	ptsI	1	18.3167	0.685
0.2	0.1	clcB	1	18.3167	0.475
0.2	0.1	ycaM	1	18.3167	0.47
0.2	0.1	yadI	1	18.3167	0.745
0.2	0.1	AG1	1	18.3167	0.783
0.2	0.1	ptsI	1	18.5667	0.748

0.2	0.1	clcB	1	18.5667	0.481
0.2	0.1	ycaM	1	18.5667	0.471
0.2	0.1	yadI	1	18.5667	0.759
0.2	0.1	AG1	1	18.5667	0.821
0.2	0.1	ptsI	1	18.8167	0.767
0.2	0.1	clcB	1	18.8167	0.479
0.2	0.1	ycaM	1	18.8167	0.471
0.2	0.1	yadI	1	18.8167	0.772
0.2	0.1	AG1	1	18.8167	0.826
0.2	0.1	ptsI	1	19.0667	0.787
0.2	0.1	clcB	1	19.0667	0.48
0.2	0.1	ycaM	1	19.0667	0.478
0.2	0.1	yadI	1	19.0667	0.782
0.2	0.1	AG1	1	19.0667	0.851
0.2	0.1	ptsI	1	19.3167	0.77
0.2	0.1	clcB	1	19.3167	0.47
0.2	0.1	ycaM	1	19.3167	0.474
0.2	0.1	yadI	1	19.3167	0.781
0.2	0.1	AG1	1	19.3167	0.835
0.2	0.1	ptsI	1	19.5667	0.803
0.2	0.1	clcB	1	19.5667	0.486
0.2	0.1	ycaM	1	19.5667	0.473
0.2	0.1	yadI	1	19.5667	0.787
0.2	0.1	AG1	1	19.5667	0.858
0.2	0.1	ptsI	1	19.8167	0.802
0.2	0.1	clcB	1	19.8167	0.466
0.2	0.1	ycaM	1	19.8167	0.476
0.2	0.1	yadI	1	19.8167	0.79
0.2	0.1	AG1	1	19.8167	0.866
0.2	0.1	ptsI	1	20.0667	0.8
0.2	0.1	clcB	1	20.0667	0.463
0.2	0.1	ycaM	1	20.0667	0.474
0.2	0.1	yadI	1	20.0667	0.796
0.2	0.1	AG1	1	20.0667	0.871
0.2	0.1	ptsI	1	20.3167	0.807
0.2	0.1	clcB	1	20.3167	0.459
0.2	0.1	ycaM	1	20.3167	0.476
0.2	0.1	yadI	1	20.3167	0.799
0.2	0.1	AG1	1	20.3167	0.876
0.2	0.1	ptsI	1	20.5667	0.788
0.2	0.1	clcB	1	20.5667	0.446
0.2	0.1	ycaM	1	20.5667	0.469
0.2	0.1	yadI	1	20.5667	0.806
0.2	0.1	AG1	1	20.5667	0.865
0.2	0.1	ptsI	1	20.8167	0.797
0.2	0.1	clcB	1	20.8167	0.439
0.2	0.1	ycaM	1	20.8167	0.472
0.2	0.1	yadI	1	20.8167	0.809
0.2	0.1	AG1	1	20.8167	0.877
0.2	0.1	ptsI	1	21.0667	0.818
0.2	0.1	clcB	1	21.0667	0.448
0.2	0.1	ycaM	1	21.0667	0.466
0.2	0.1	yadI	1	21.0667	0.813

0.2	0.1	AG1	1	21.0667	0.886
0.2	0.1	ptsI	1	21.3167	0.817
0.2	0.1	clcB	1	21.3167	0.439
0.2	0.1	ycaM	1	21.3167	0.47
0.2	0.1	yadI	1	21.3167	0.815
0.2	0.1	AG1	1	21.3167	0.889
0.2	0.1	ptsI	1	21.5667	0.814
0.2	0.1	clcB	1	21.5667	0.44
0.2	0.1	ycaM	1	21.5667	0.468
0.2	0.1	yadI	1	21.5667	0.821
0.2	0.1	AG1	1	21.5667	0.889
0.2	0.1	ptsI	1	21.8167	0.819
0.2	0.1	clcB	1	21.8167	0.436
0.2	0.1	ycaM	1	21.8167	0.474
0.2	0.1	yadI	1	21.8167	0.822
0.2	0.1	AG1	1	21.8167	0.899
0.2	0.1	ptsI	1	22.0667	0.834
0.2	0.1	clcB	1	22.0667	0.442
0.2	0.1	ycaM	1	22.0667	0.47
0.2	0.1	yadI	1	22.0667	0.822
0.2	0.1	AG1	1	22.0667	0.902
0.2	0.1	ptsI	1	22.9333	0.827
0.2	0.1	clcB	1	22.9333	0.452
0.2	0.1	ycaM	1	22.9333	0.456
0.2	0.1	yadI	1	22.9333	0.83
0.2	0.1	AG1	1	22.9333	0.817
0.2	0.1	ptsI	1	23.1833	0.799
0.2	0.1	clcB	1	23.1833	0.447
0.2	0.1	ycaM	1	23.1833	0.449
0.2	0.1	yadI	1	23.1833	0.825
0.2	0.1	AG1	1	23.1833	0.778
0.2	0.1	ptsI	1	23.4333	0.791
0.2	0.1	clcB	1	23.4333	0.44
0.2	0.1	ycaM	1	23.4333	0.447
0.2	0.1	yadI	1	23.4333	0.83
0.2	0.1	AG1	1	23.4333	0.785
0.2	0.1	ptsI	1	23.6833	0.794
0.2	0.1	clcB	1	23.6833	0.44
0.2	0.1	ycaM	1	23.6833	0.449
0.2	0.1	yadI	1	23.6833	0.84
0.2	0.1	AG1	1	23.6833	0.826
0.2	0.1	ptsI	1	23.9333	0.819
0.2	0.1	clcB	1	23.9333	0.44
0.2	0.1	ycaM	1	23.9333	0.444
0.2	0.1	yadI	1	23.9333	0.844
0.2	0.1	AG1	1	23.9333	0.827
0.2	0.1	ptsI	1	24.1833	0.837
0.2	0.1	clcB	1	24.1833	0.438
0.2	0.1	ycaM	1	24.1833	0.443
0.2	0.1	yadI	1	24.1833	0.855
0.2	0.1	AG1	1	24.1833	0.842
0.2	0.1	ptsI	1	24.4333	0.838
0.2	0.1	clcB	1	24.4333	0.435

0.2	0.1	ycaM	1	24.4333	0.442
0.2	0.1	yadI	1	24.4333	0.864
0.2	0.1	AG1	1	24.4333	0.841
0.2	0.1	ptsI	1	24.6833	0.844
0.2	0.1	clcB	1	24.6833	0.435
0.2	0.1	ycaM	1	24.6833	0.443
0.2	0.1	yadI	1	24.6833	0.863
0.2	0.1	AG1	1	24.6833	0.842
0.2	0.1	ptsI	1	24.9333	0.828
0.2	0.1	clcB	1	24.9333	0.433
0.2	0.1	ycaM	1	24.9333	0.441
0.2	0.1	yadI	1	24.9333	0.873
0.2	0.1	AG1	1	24.9333	0.848
0.2	0.1	ptsI	1	25.1833	0.842
0.2	0.1	clcB	1	25.1833	0.428
0.2	0.1	ycaM	1	25.1833	0.44
0.2	0.1	yadI	1	25.1833	0.872
0.2	0.1	AG1	1	25.1833	0.842
0.2	0.1	ptsI	1	25.4333	0.852
0.2	0.1	clcB	1	25.4333	0.437
0.2	0.1	ycaM	1	25.4333	0.441
0.2	0.1	yadI	1	25.4333	0.879
0.2	0.1	AG1	1	25.4333	0.854
0.2	0.1	ptsI	1	25.6833	0.852
0.2	0.1	clcB	1	25.6833	0.432
0.2	0.1	ycaM	1	25.6833	0.444
0.2	0.1	yadI	1	25.6833	0.884
0.2	0.1	AG1	1	25.6833	0.852
0.2	0.1	ptsI	1	25.9333	0.858
0.2	0.1	clcB	1	25.9333	0.425
0.2	0.1	ycaM	1	25.9333	0.441
0.2	0.1	yadI	1	25.9333	0.868
0.2	0.1	AG1	1	25.9333	0.861
0.2	0.1	ptsI	1	26.1833	0.866
0.2	0.1	clcB	1	26.1833	0.43
0.2	0.1	ycaM	1	26.1833	0.443
0.2	0.1	yadI	1	26.1833	0.889
0.2	0.1	AG1	1	26.1833	0.86
0.2	0.1	ptsI	1	26.4333	0.859
0.2	0.1	clcB	1	26.4333	0.433
0.2	0.1	ycaM	1	26.4333	0.445
0.2	0.1	yadI	1	26.4333	0.89
0.2	0.1	AG1	1	26.4333	0.855
0.2	0.1	ptsI	1	26.6833	0.865
0.2	0.1	clcB	1	26.6833	0.433
0.2	0.1	ycaM	1	26.6833	0.446
0.2	0.1	yadI	1	26.6833	0.899
0.2	0.1	AG1	1	26.6833	0.862
0.2	0.1	ptsI	1	26.9333	0.867
0.2	0.1	clcB	1	26.9333	0.437
0.2	0.1	ycaM	1	26.9333	0.444
0.2	0.1	yadI	1	26.9333	0.899
0.2	0.1	AG1	1	26.9333	0.862

0.2	0.1	ptsI	1	27.1833	0.873
0.2	0.1	clcB	1	27.1833	0.436
0.2	0.1	ycaM	1	27.1833	0.445
0.2	0.1	yadI	1	27.1833	0.904
0.2	0.1	AG1	1	27.1833	0.87
0.2	0.1	ptsI	1	27.4333	0.868
0.2	0.1	clcB	1	27.4333	0.433
0.2	0.1	ycaM	1	27.4333	0.446
0.2	0.1	yadI	1	27.4333	0.907
0.2	0.1	AG1	1	27.4333	0.865
0.2	0.1	ptsI	1	27.6833	0.875
0.2	0.1	clcB	1	27.6833	0.435
0.2	0.1	ycaM	1	27.6833	0.444
0.2	0.1	yadI	1	27.6833	0.908
0.2	0.1	AG1	1	27.6833	0.868
0.2	0.1	ptsI	1	27.9333	0.877
0.2	0.1	clcB	1	27.9333	0.43
0.2	0.1	ycaM	1	27.9333	0.443
0.2	0.1	yadI	1	27.9333	0.883
0.2	0.1	AG1	1	27.9333	0.865
0.2	0.1	ptsI	1	28.1833	0.878
0.2	0.1	clcB	1	28.1833	0.437
0.2	0.1	ycaM	1	28.1833	0.445
0.2	0.1	yadI	1	28.1833	0.911
0.2	0.1	AG1	1	28.1833	0.884
0.2	0.1	ptsI	1	28.4333	0.875
0.2	0.1	clcB	1	28.4333	0.434
0.2	0.1	ycaM	1	28.4333	0.446
0.2	0.1	yadI	1	28.4333	0.913
0.2	0.1	AG1	1	28.4333	0.882
0.2	0.1	ptsI	1	28.6833	0.877
0.2	0.1	clcB	1	28.6833	0.438
0.2	0.1	ycaM	1	28.6833	0.447
0.2	0.1	yadI	1	28.6833	0.914
0.2	0.1	AG1	1	28.6833	0.888
0.2	0.1	ptsI	1	28.9333	0.88
0.2	0.1	clcB	1	28.9333	0.437
0.2	0.1	ycaM	1	28.9333	0.447
0.2	0.1	yadI	1	28.9333	0.917
0.2	0.1	AG1	1	28.9333	0.892
0.2	0.1	ptsI	1	29.1833	0.875
0.2	0.1	clcB	1	29.1833	0.438
0.2	0.1	ycaM	1	29.1833	0.447
0.2	0.1	yadI	1	29.1833	0.923
0.2	0.1	AG1	1	29.1833	0.891
0.2	0.1	ptsI	1	29.4333	0.883
0.2	0.1	clcB	1	29.4333	0.44
0.2	0.1	ycaM	1	29.4333	0.447
0.2	0.1	yadI	1	29.4333	0.923
0.2	0.1	AG1	1	29.4333	0.899
0.2	0.1	ptsI	1	29.6833	0.882
0.2	0.1	clcB	1	29.6833	0.444
0.2	0.1	ycaM	1	29.6833	0.448

0.2	0.1	yadI	1	29.6833	0.929
0.2	0.1	AG1	1	29.6833	0.901
0.2	0.1	ptsI	1	29.9333	0.886
0.2	0.1	clcB	1	29.9333	0.444
0.2	0.1	ycaM	1	29.9333	0.449
0.2	0.1	yadI	1	29.9333	0.928
0.2	0.1	AG1	1	29.9333	0.909
0.2	0.1	ptsI	1	30.1833	0.886
0.2	0.1	clcB	1	30.1833	0.445
0.2	0.1	ycaM	1	30.1833	0.448
0.2	0.1	yadI	1	30.1833	0.928
0.2	0.1	AG1	1	30.1833	0.916
0.2	0.1	ptsI	1	30.4333	0.886
0.2	0.1	clcB	1	30.4333	0.444
0.2	0.1	ycaM	1	30.4333	0.45
0.2	0.1	yadI	1	30.4333	0.93
0.2	0.1	AG1	1	30.4333	0.918
0.2	0.1	ptsI	1	30.6833	0.886
0.2	0.1	clcB	1	30.6833	0.445
0.2	0.1	ycaM	1	30.6833	0.451
0.2	0.1	yadI	1	30.6833	0.938
0.2	0.1	AG1	1	30.6833	0.92
0.2	0.1	ptsI	1	30.9333	0.889
0.2	0.1	clcB	1	30.9333	0.446
0.2	0.1	ycaM	1	30.9333	0.452
0.2	0.1	yadI	1	30.9333	0.936
0.2	0.1	AG1	1	30.9333	0.927
0.2	0.1	ptsI	1	31.1833	0.883
0.2	0.1	clcB	1	31.1833	0.442
0.2	0.1	ycaM	1	31.1833	0.452
0.2	0.1	yadI	1	31.1833	0.943
0.2	0.1	AG1	1	31.1833	0.927
0.2	0.1	ptsI	1	31.4333	0.894
0.2	0.1	clcB	1	31.4333	0.447
0.2	0.1	ycaM	1	31.4333	0.455
0.2	0.1	yadI	1	31.4333	0.948
0.2	0.1	AG1	1	31.4333	0.936
0.2	0.1	ptsI	1	31.6833	0.893
0.2	0.1	clcB	1	31.6833	0.446
0.2	0.1	ycaM	1	31.6833	0.457
0.2	0.1	yadI	1	31.6833	0.951
0.2	0.1	AG1	1	31.6833	0.945
0.2	0.1	ptsI	1	31.9333	0.894
0.2	0.1	clcB	1	31.9333	0.451
0.2	0.1	ycaM	1	31.9333	0.453
0.2	0.1	yadI	1	31.9333	0.95
0.2	0.1	AG1	1	31.9333	0.951
0.2	0.1	ptsI	1	32.1833	0.893
0.2	0.1	clcB	1	32.1833	0.451
0.2	0.1	ycaM	1	32.1833	0.46
0.2	0.1	yadI	1	32.1833	0.951
0.2	0.1	AG1	1	32.1833	0.959
0.2	0.1	ptsI	1	32.4333	0.89

0.2	0.1	clcB	1	32.4333	0.435
0.2	0.1	ycaM	1	32.4333	0.449
0.2	0.1	yadI	1	32.4333	0.924
0.2	0.1	AG1	1	32.4333	0.954
0.2	0.1	ptsI	1	32.6833	0.891
0.2	0.1	clcB	1	32.6833	0.45
0.2	0.1	ycaM	1	32.6833	0.46
0.2	0.1	yadI	1	32.6833	0.956
0.2	0.1	AG1	1	32.6833	0.969
0.2	0.1	ptsI	1	32.9333	0.891
0.2	0.1	clcB	1	32.9333	0.447
0.2	0.1	ycaM	1	32.9333	0.456
0.2	0.1	yadI	1	32.9333	0.96
0.2	0.1	AG1	1	32.9333	0.977
0.2	0.1	ptsI	1	33.1833	0.89
0.2	0.1	clcB	1	33.1833	0.442
0.2	0.1	ycaM	1	33.1833	0.456
0.2	0.1	yadI	1	33.1833	0.943
0.2	0.1	AG1	1	33.1833	0.978
0.2	0.1	ptsI	1	33.4333	0.892
0.2	0.1	clcB	1	33.4333	0.453
0.2	0.1	ycaM	1	33.4333	0.459
0.2	0.1	yadI	1	33.4333	0.964
0.2	0.1	AG1	1	33.4333	0.992
0.2	0.1	ptsI	1	33.6833	0.89
0.2	0.1	clcB	1	33.6833	0.449
0.2	0.1	ycaM	1	33.6833	0.46
0.2	0.1	yadI	1	33.6833	0.942
0.2	0.1	AG1	1	33.6833	0.992
0.2	0.1	ptsI	1	33.9333	0.889
0.2	0.1	clcB	1	33.9333	0.452
0.2	0.1	ycaM	1	33.9333	0.46
0.2	0.1	yadI	1	33.9333	0.963
0.2	0.1	AG1	1	33.9333	1.007
0.2	0.1	ptsI	1	34.1833	0.889
0.2	0.1	clcB	1	34.1833	0.461
0.2	0.1	ycaM	1	34.1833	0.458
0.2	0.1	yadI	1	34.1833	0.974
0.2	0.1	AG1	1	34.1833	1.006
0.2	0.1	ptsI	1	34.4333	0.891
0.2	0.1	clcB	1	34.4333	0.456
0.2	0.1	ycaM	1	34.4333	0.461
0.2	0.1	yadI	1	34.4333	0.965
0.2	0.1	AG1	1	34.4333	1.022
0.2	0.1	ptsI	1	34.6833	0.891
0.2	0.1	clcB	1	34.6833	0.46
0.2	0.1	ycaM	1	34.6833	0.464
0.2	0.1	yadI	1	34.6833	0.964
0.2	0.1	AG1	1	34.6833	1.03
0.2	0.1	ptsI	1	34.9333	0.891
0.2	0.1	clcB	1	34.9333	0.457
0.2	0.1	ycaM	1	34.9333	0.461
0.2	0.1	yadI	1	34.9333	0.966

0.2	0.1	AG1	1	34.9333	1.032
0.2	0.1	ptsI	1	35.1833	0.888
0.2	0.1	clcB	1	35.1833	0.458
0.2	0.1	ycaM	1	35.1833	0.462
0.2	0.1	yadI	1	35.1833	0.967
0.2	0.1	AG1	1	35.1833	1.042
0.2	0.1	ptsI	1	35.4333	0.893
0.2	0.1	clcB	1	35.4333	0.458
0.2	0.1	ycaM	1	35.4333	0.46
0.2	0.1	yadI	1	35.4333	0.969
0.2	0.1	AG1	1	35.4333	1.052
0.2	0.1	ptsI	1	35.6833	0.893
0.2	0.1	clcB	1	35.6833	0.462
0.2	0.1	ycaM	1	35.6833	0.465
0.2	0.1	yadI	1	35.6833	0.969
0.2	0.1	AG1	1	35.6833	1.061
0.2	0.1	ptsI	1	35.9333	0.893
0.2	0.1	clcB	1	35.9333	0.455
0.2	0.1	ycaM	1	35.9333	0.463
0.2	0.1	yadI	1	35.9333	0.952
0.2	0.1	AG1	1	35.9333	1.062
0.2	0.1	ptsI	1	36.1833	0.892
0.2	0.1	clcB	1	36.1833	0.463
0.2	0.1	ycaM	1	36.1833	0.47
0.2	0.1	yadI	1	36.1833	0.971
0.2	0.1	AG1	1	36.1833	1.078
0.2	0.1	ptsI	1	36.4333	0.89
0.2	0.1	clcB	1	36.4333	0.461
0.2	0.1	ycaM	1	36.4333	0.466
0.2	0.1	yadI	1	36.4333	0.97
0.2	0.1	AG1	1	36.4333	1.078
0.2	0.1	ptsI	1	36.6833	0.891
0.2	0.1	clcB	1	36.6833	0.46
0.2	0.1	ycaM	1	36.6833	0.466
0.2	0.1	yadI	1	36.6833	0.974
0.2	0.1	AG1	1	36.6833	1.083
0.4	0.1	ptsI	1	0	0.232
0.4	0.1	clcB	1	0	0.253
0.4	0.1	ycaM	1	0	0.24
0.4	0.1	yadI	1	0	0.263
0.4	0.1	AG1	1	0	0.254
0.4	0.1	ptsI	1	0.25	0.229
0.4	0.1	clcB	1	0.25	0.239
0.4	0.1	ycaM	1	0.25	0.23
0.4	0.1	yadI	1	0.25	0.252
0.4	0.1	AG1	1	0.25	0.241
0.4	0.1	ptsI	1	0.5	0.228
0.4	0.1	clcB	1	0.5	0.233
0.4	0.1	ycaM	1	0.5	0.225
0.4	0.1	yadI	1	0.5	0.243
0.4	0.1	AG1	1	0.5	0.237
0.4	0.1	ptsI	1	0.75	0.229
0.4	0.1	clcB	1	0.75	0.231

0.4	0.1	ycaM	1	0.75	0.223
0.4	0.1	yadI	1	0.75	0.243
0.4	0.1	AG1	1	0.75	0.236
0.4	0.1	ptsI	1	1	0.228
0.4	0.1	clcB	1	1	0.233
0.4	0.1	ycaM	1	1	0.225
0.4	0.1	yadI	1	1	0.244
0.4	0.1	AG1	1	1	0.235
0.4	0.1	ptsI	1	1.25	0.228
0.4	0.1	clcB	1	1.25	0.232
0.4	0.1	ycaM	1	1.25	0.225
0.4	0.1	yadI	1	1.25	0.246
0.4	0.1	AG1	1	1.25	0.236
0.4	0.1	ptsI	1	1.5	0.235
0.4	0.1	clcB	1	1.5	0.233
0.4	0.1	ycaM	1	1.5	0.227
0.4	0.1	yadI	1	1.5	0.251
0.4	0.1	AG1	1	1.5	0.239
0.4	0.1	ptsI	1	1.75	0.235
0.4	0.1	clcB	1	1.75	0.237
0.4	0.1	ycaM	1	1.75	0.231
0.4	0.1	yadI	1	1.75	0.252
0.4	0.1	AG1	1	1.75	0.241
0.4	0.1	ptsI	1	2	0.239
0.4	0.1	clcB	1	2	0.239
0.4	0.1	ycaM	1	2	0.234
0.4	0.1	yadI	1	2	0.257
0.4	0.1	AG1	1	2	0.246
0.4	0.1	ptsI	1	2.25	0.245
0.4	0.1	clcB	1	2.25	0.242
0.4	0.1	ycaM	1	2.25	0.24
0.4	0.1	yadI	1	2.25	0.264
0.4	0.1	AG1	1	2.25	0.249
0.4	0.1	ptsI	1	2.5	0.251
0.4	0.1	clcB	1	2.5	0.246
0.4	0.1	ycaM	1	2.5	0.246
0.4	0.1	yadI	1	2.5	0.268
0.4	0.1	AG1	1	2.5	0.254
0.4	0.1	ptsI	1	2.75	0.256
0.4	0.1	clcB	1	2.75	0.252
0.4	0.1	ycaM	1	2.75	0.253
0.4	0.1	yadI	1	2.75	0.276
0.4	0.1	AG1	1	2.75	0.26
0.4	0.1	ptsI	1	3	0.262
0.4	0.1	clcB	1	3	0.254
0.4	0.1	ycaM	1	3	0.257
0.4	0.1	yadI	1	3	0.283
0.4	0.1	AG1	1	3	0.268
0.4	0.1	ptsI	1	3.25	0.273
0.4	0.1	clcB	1	3.25	0.26
0.4	0.1	ycaM	1	3.25	0.266
0.4	0.1	yadI	1	3.25	0.295
0.4	0.1	AG1	1	3.25	0.276

0.4	0.1	ptsI	1	3.5	0.278
0.4	0.1	clcB	1	3.5	0.269
0.4	0.1	ycaM	1	3.5	0.272
0.4	0.1	yadI	1	3.5	0.302
0.4	0.1	AG1	1	3.5	0.278
0.4	0.1	ptsI	1	3.75	0.292
0.4	0.1	clcB	1	3.75	0.274
0.4	0.1	ycaM	1	3.75	0.276
0.4	0.1	yadI	1	3.75	0.31
0.4	0.1	AG1	1	3.75	0.283
0.4	0.1	ptsI	1	4	0.31
0.4	0.1	clcB	1	4	0.285
0.4	0.1	ycaM	1	4	0.287
0.4	0.1	yadI	1	4	0.325
0.4	0.1	AG1	1	4	0.292
0.4	0.1	ptsI	1	4.25	0.314
0.4	0.1	clcB	1	4.25	0.286
0.4	0.1	ycaM	1	4.25	0.29
0.4	0.1	yadI	1	4.25	0.327
0.4	0.1	AG1	1	4.25	0.299
0.4	0.1	ptsI	1	4.5	0.328
0.4	0.1	clcB	1	4.5	0.292
0.4	0.1	ycaM	1	4.5	0.296
0.4	0.1	yadI	1	4.5	0.338
0.4	0.1	AG1	1	4.5	0.305
0.4	0.1	ptsI	1	4.75	0.337
0.4	0.1	clcB	1	4.75	0.299
0.4	0.1	ycaM	1	4.75	0.301
0.4	0.1	yadI	1	4.75	0.342
0.4	0.1	AG1	1	4.75	0.309
0.4	0.1	ptsI	1	5	0.354
0.4	0.1	clcB	1	5	0.302
0.4	0.1	ycaM	1	5	0.304
0.4	0.1	yadI	1	5	0.356
0.4	0.1	AG1	1	5	0.317
0.4	0.1	ptsI	1	5.25	0.355
0.4	0.1	clcB	1	5.25	0.308
0.4	0.1	ycaM	1	5.25	0.309
0.4	0.1	yadI	1	5.25	0.363
0.4	0.1	AG1	1	5.25	0.324
0.4	0.1	ptsI	1	5.5	0.365
0.4	0.1	clcB	1	5.5	0.315
0.4	0.1	ycaM	1	5.5	0.315
0.4	0.1	yadI	1	5.5	0.375
0.4	0.1	AG1	1	5.5	0.332
0.4	0.1	ptsI	1	5.75	0.375
0.4	0.1	clcB	1	5.75	0.323
0.4	0.1	ycaM	1	5.75	0.322
0.4	0.1	yadI	1	5.75	0.385
0.4	0.1	AG1	1	5.75	0.342
0.4	0.1	ptsI	1	6	0.368
0.4	0.1	clcB	1	6	0.328
0.4	0.1	ycaM	1	6	0.329

0.4	0.1	yadI	1	6	0.388
0.4	0.1	AG1	1	6	0.341
0.4	0.1	ptsI	1	6.25	0.377
0.4	0.1	clcB	1	6.25	0.338
0.4	0.1	ycaM	1	6.25	0.328
0.4	0.1	yadI	1	6.25	0.382
0.4	0.1	AG1	1	6.25	0.346
0.4	0.1	ptsI	1	6.5	0.388
0.4	0.1	clcB	1	6.5	0.34
0.4	0.1	ycaM	1	6.5	0.336
0.4	0.1	yadI	1	6.5	0.383
0.4	0.1	AG1	1	6.5	0.353
0.4	0.1	ptsI	1	6.75	0.404
0.4	0.1	clcB	1	6.75	0.346
0.4	0.1	ycaM	1	6.75	0.342
0.4	0.1	yadI	1	6.75	0.393
0.4	0.1	AG1	1	6.75	0.363
0.4	0.1	ptsI	1	7	0.42
0.4	0.1	clcB	1	7	0.356
0.4	0.1	ycaM	1	7	0.349
0.4	0.1	yadI	1	7	0.404
0.4	0.1	AG1	1	7	0.374
0.4	0.1	ptsI	1	7.25	0.449
0.4	0.1	clcB	1	7.25	0.362
0.4	0.1	ycaM	1	7.25	0.354
0.4	0.1	yadI	1	7.25	0.409
0.4	0.1	AG1	1	7.25	0.382
0.4	0.1	ptsI	1	7.5	0.46
0.4	0.1	clcB	1	7.5	0.369
0.4	0.1	ycaM	1	7.5	0.359
0.4	0.1	yadI	1	7.5	0.418
0.4	0.1	AG1	1	7.5	0.392
0.4	0.1	ptsI	1	7.75	0.472
0.4	0.1	clcB	1	7.75	0.38
0.4	0.1	ycaM	1	7.75	0.368
0.4	0.1	yadI	1	7.75	0.426
0.4	0.1	AG1	1	7.75	0.403
0.4	0.1	ptsI	1	8	0.498
0.4	0.1	clcB	1	8	0.386
0.4	0.1	ycaM	1	8	0.367
0.4	0.1	yadI	1	8	0.432
0.4	0.1	AG1	1	8	0.409
0.4	0.1	ptsI	1	8.25	0.502
0.4	0.1	clcB	1	8.25	0.396
0.4	0.1	ycaM	1	8.25	0.368
0.4	0.1	yadI	1	8.25	0.437
0.4	0.1	AG1	1	8.25	0.42
0.4	0.1	ptsI	1	8.5	0.517
0.4	0.1	clcB	1	8.5	0.402
0.4	0.1	ycaM	1	8.5	0.37
0.4	0.1	yadI	1	8.5	0.443
0.4	0.1	AG1	1	8.5	0.426
0.4	0.1	ptsI	1	8.75	0.527

0.4	0.1	clcB	1	8.75	0.413
0.4	0.1	ycaM	1	8.75	0.373
0.4	0.1	yadI	1	8.75	0.448
0.4	0.1	AG1	1	8.75	0.435
0.4	0.1	ptsI	1	9	0.537
0.4	0.1	clcB	1	9	0.417
0.4	0.1	ycaM	1	9	0.373
0.4	0.1	yadI	1	9	0.458
0.4	0.1	AG1	1	9	0.443
0.4	0.1	ptsI	1	9.25	0.544
0.4	0.1	clcB	1	9.25	0.421
0.4	0.1	ycaM	1	9.25	0.376
0.4	0.1	yadI	1	9.25	0.446
0.4	0.1	AG1	1	9.25	0.458
0.4	0.1	ptsI	1	9.5	0.564
0.4	0.1	clcB	1	9.5	0.435
0.4	0.1	ycaM	1	9.5	0.383
0.4	0.1	yadI	1	9.5	0.458
0.4	0.1	AG1	1	9.5	0.494
0.4	0.1	ptsI	1	9.75	0.592
0.4	0.1	clcB	1	9.75	0.454
0.4	0.1	ycaM	1	9.75	0.39
0.4	0.1	yadI	1	9.75	0.465
0.4	0.1	AG1	1	9.75	0.512
0.4	0.1	ptsI	1	10	0.565
0.4	0.1	clcB	1	10	0.459
0.4	0.1	ycaM	1	10	0.392
0.4	0.1	yadI	1	10	0.454
0.4	0.1	AG1	1	10	0.522
0.4	0.1	ptsI	1	10.25	0.623
0.4	0.1	clcB	1	10.25	0.471
0.4	0.1	ycaM	1	10.25	0.399
0.4	0.1	yadI	1	10.25	0.464
0.4	0.1	AG1	1	10.25	0.535
0.4	0.1	ptsI	1	10.5	0.641
0.4	0.1	clcB	1	10.5	0.495
0.4	0.1	ycaM	1	10.5	0.401
0.4	0.1	yadI	1	10.5	0.475
0.4	0.1	AG1	1	10.5	0.549
0.4	0.1	ptsI	1	10.75	0.607
0.4	0.1	clcB	1	10.75	0.492
0.4	0.1	ycaM	1	10.75	0.404
0.4	0.1	yadI	1	10.75	0.456
0.4	0.1	AG1	1	10.75	0.546
0.4	0.1	ptsI	1	11	0.613
0.4	0.1	clcB	1	11	0.51
0.4	0.1	ycaM	1	11	0.412
0.4	0.1	yadI	1	11	0.47
0.4	0.1	AG1	1	11	0.582
0.4	0.1	ptsI	1	11.25	0.679
0.4	0.1	clcB	1	11.25	0.548
0.4	0.1	ycaM	1	11.25	0.411
0.4	0.1	yadI	1	11.25	0.473

0.4	0.1	AG1	1	11.25	0.595
0.4	0.1	ptsI	1	11.5	0.657
0.4	0.1	clcB	1	11.5	0.538
0.4	0.1	ycaM	1	11.5	0.419
0.4	0.1	yadI	1	11.5	0.476
0.4	0.1	AG1	1	11.5	0.624
0.4	0.1	ptsI	1	11.75	0.667
0.4	0.1	clcB	1	11.75	0.553
0.4	0.1	ycaM	1	11.75	0.419
0.4	0.1	yadI	1	11.75	0.47
0.4	0.1	AG1	1	11.75	0.63
0.4	0.1	ptsI	1	12	0.703
0.4	0.1	clcB	1	12	0.599
0.4	0.1	ycaM	1	12	0.422
0.4	0.1	yadI	1	12	0.476
0.4	0.1	AG1	1	12	0.66
0.4	0.1	ptsI	1	12.25	0.678
0.4	0.1	clcB	1	12.25	0.575
0.4	0.1	ycaM	1	12.25	0.422
0.4	0.1	yadI	1	12.25	0.469
0.4	0.1	AG1	1	12.25	0.618
0.4	0.1	ptsI	1	12.5	0.713
0.4	0.1	clcB	1	12.5	0.588
0.4	0.1	ycaM	1	12.5	0.423
0.4	0.1	yadI	1	12.5	0.461
0.4	0.1	AG1	1	12.5	0.63
0.4	0.1	ptsI	1	12.75	0.721
0.4	0.1	clcB	1	12.75	0.604
0.4	0.1	ycaM	1	12.75	0.429
0.4	0.1	yadI	1	12.75	0.47
0.4	0.1	AG1	1	12.75	0.643
0.4	0.1	ptsI	1	13	0.728
0.4	0.1	clcB	1	13	0.608
0.4	0.1	ycaM	1	13	0.443
0.4	0.1	yadI	1	13	0.485
0.4	0.1	AG1	1	13	0.69
0.4	0.1	ptsI	1	13.25	0.716
0.4	0.1	clcB	1	13.25	0.608
0.4	0.1	ycaM	1	13.25	0.439
0.4	0.1	yadI	1	13.25	0.48
0.4	0.1	AG1	1	13.25	0.698
0.4	0.1	ptsI	1	13.5	0.748
0.4	0.1	clcB	1	13.5	0.637
0.4	0.1	ycaM	1	13.5	0.452
0.4	0.1	yadI	1	13.5	0.481
0.4	0.1	AG1	1	13.5	0.732
0.4	0.1	ptsI	1	13.75	0.73
0.4	0.1	clcB	1	13.75	0.632
0.4	0.1	ycaM	1	13.75	0.455
0.4	0.1	yadI	1	13.75	0.477
0.4	0.1	AG1	1	13.75	0.744
0.4	0.1	ptsI	1	14	0.768
0.4	0.1	clcB	1	14	0.669

0.4	0.1	ycaM	1	14	0.467
0.4	0.1	yadI	1	14	0.482
0.4	0.1	AG1	1	14	0.787
0.4	0.1	ptsI	1	14.25	0.772
0.4	0.1	clcB	1	14.25	0.675
0.4	0.1	ycaM	1	14.25	0.471
0.4	0.1	yadI	1	14.25	0.49
0.4	0.1	AG1	1	14.25	0.802
0.4	0.1	ptsI	1	14.5	0.767
0.4	0.1	clcB	1	14.5	0.683
0.4	0.1	ycaM	1	14.5	0.486
0.4	0.1	yadI	1	14.5	0.5
0.4	0.1	AG1	1	14.5	0.812
0.4	0.1	ptsI	1	14.75	0.739
0.4	0.1	clcB	1	14.75	0.668
0.4	0.1	ycaM	1	14.75	0.489
0.4	0.1	yadI	1	14.75	0.504
0.4	0.1	AG1	1	14.75	0.808
0.4	0.1	ptsI	1	15	0.792
0.4	0.1	clcB	1	15	0.707
0.4	0.1	ycaM	1	15	0.504
0.4	0.1	yadI	1	15	0.518
0.4	0.1	AG1	1	15	0.818
0.4	0.1	ptsI	1	15.25	0.794
0.4	0.1	clcB	1	15.25	0.71
0.4	0.1	ycaM	1	15.25	0.543
0.4	0.1	yadI	1	15.25	0.523
0.4	0.1	AG1	1	15.25	0.845
0.4	0.1	ptsI	1	15.5	0.78
0.4	0.1	clcB	1	15.5	0.701
0.4	0.1	ycaM	1	15.5	0.542
0.4	0.1	yadI	1	15.5	0.526
0.4	0.1	AG1	1	15.5	0.832
0.4	0.1	ptsI	1	15.75	0.773
0.4	0.1	clcB	1	15.75	0.701
0.4	0.1	ycaM	1	15.75	0.536
0.4	0.1	yadI	1	15.75	0.513
0.4	0.1	AG1	1	15.75	0.839
0.4	0.1	ptsI	1	16	0.77
0.4	0.1	clcB	1	16	0.696
0.4	0.1	ycaM	1	16	0.529
0.4	0.1	yadI	1	16	0.514
0.4	0.1	AG1	1	16	0.833
0.4	0.1	ptsI	1	16.25	0.822
0.4	0.1	clcB	1	16.25	0.723
0.4	0.1	ycaM	1	16.25	0.534
0.4	0.1	yadI	1	16.25	0.527
0.4	0.1	AG1	1	16.25	0.85
0.4	0.1	ptsI	1	16.5	0.815
0.4	0.1	clcB	1	16.5	0.73
0.4	0.1	ycaM	1	16.5	0.569
0.4	0.1	yadI	1	16.5	0.537
0.4	0.1	AG1	1	16.5	0.864

0.4	0.1	ptsI	1	17.0667	0.629
0.4	0.1	clcB	1	17.0667	0.519
0.4	0.1	ycaM	1	17.0667	0.509
0.4	0.1	yadI	1	17.0667	0.645
0.4	0.1	AG1	1	17.0667	0.759
0.4	0.1	ptsI	1	17.3167	0.659
0.4	0.1	clcB	1	17.3167	0.481
0.4	0.1	ycaM	1	17.3167	0.498
0.4	0.1	yadI	1	17.3167	0.622
0.4	0.1	AG1	1	17.3167	0.756
0.4	0.1	ptsI	1	17.5667	0.654
0.4	0.1	clcB	1	17.5667	0.479
0.4	0.1	ycaM	1	17.5667	0.492
0.4	0.1	yadI	1	17.5667	0.624
0.4	0.1	AG1	1	17.5667	0.746
0.4	0.1	ptsI	1	17.8167	0.742
0.4	0.1	clcB	1	17.8167	0.483
0.4	0.1	ycaM	1	17.8167	0.49
0.4	0.1	yadI	1	17.8167	0.641
0.4	0.1	AG1	1	17.8167	0.773
0.4	0.1	ptsI	1	18.0667	0.69
0.4	0.1	clcB	1	18.0667	0.483
0.4	0.1	ycaM	1	18.0667	0.486
0.4	0.1	yadI	1	18.0667	0.652
0.4	0.1	AG1	1	18.0667	0.775
0.4	0.1	ptsI	1	18.3167	0.7
0.4	0.1	clcB	1	18.3167	0.479
0.4	0.1	ycaM	1	18.3167	0.479
0.4	0.1	yadI	1	18.3167	0.644
0.4	0.1	AG1	1	18.3167	0.794
0.4	0.1	ptsI	1	18.5667	0.764
0.4	0.1	clcB	1	18.5667	0.482
0.4	0.1	ycaM	1	18.5667	0.481
0.4	0.1	yadI	1	18.5667	0.659
0.4	0.1	AG1	1	18.5667	0.807
0.4	0.1	ptsI	1	18.8167	0.766
0.4	0.1	clcB	1	18.8167	0.486
0.4	0.1	ycaM	1	18.8167	0.48
0.4	0.1	yadI	1	18.8167	0.67
0.4	0.1	AG1	1	18.8167	0.818
0.4	0.1	ptsI	1	19.0667	0.783
0.4	0.1	clcB	1	19.0667	0.493
0.4	0.1	ycaM	1	19.0667	0.487
0.4	0.1	yadI	1	19.0667	0.686
0.4	0.1	AG1	1	19.0667	0.839
0.4	0.1	ptsI	1	19.3167	0.764
0.4	0.1	clcB	1	19.3167	0.486
0.4	0.1	ycaM	1	19.3167	0.482
0.4	0.1	yadI	1	19.3167	0.685
0.4	0.1	AG1	1	19.3167	0.833
0.4	0.1	ptsI	1	19.5667	0.784
0.4	0.1	clcB	1	19.5667	0.497
0.4	0.1	ycaM	1	19.5667	0.483

0.4	0.1	yadI	1	19.5667	0.693
0.4	0.1	AG1	1	19.5667	0.836
0.4	0.1	ptsI	1	19.8167	0.786
0.4	0.1	clcB	1	19.8167	0.491
0.4	0.1	ycaM	1	19.8167	0.489
0.4	0.1	yadI	1	19.8167	0.697
0.4	0.1	AG1	1	19.8167	0.838
0.4	0.1	ptsI	1	20.0667	0.786
0.4	0.1	clcB	1	20.0667	0.484
0.4	0.1	ycaM	1	20.0667	0.487
0.4	0.1	yadI	1	20.0667	0.705
0.4	0.1	AG1	1	20.0667	0.847
0.4	0.1	ptsI	1	20.3167	0.789
0.4	0.1	clcB	1	20.3167	0.487
0.4	0.1	ycaM	1	20.3167	0.484
0.4	0.1	yadI	1	20.3167	0.706
0.4	0.1	AG1	1	20.3167	0.85
0.4	0.1	ptsI	1	20.5667	0.778
0.4	0.1	clcB	1	20.5667	0.468
0.4	0.1	ycaM	1	20.5667	0.482
0.4	0.1	yadI	1	20.5667	0.705
0.4	0.1	AG1	1	20.5667	0.852
0.4	0.1	ptsI	1	20.8167	0.786
0.4	0.1	clcB	1	20.8167	0.472
0.4	0.1	ycaM	1	20.8167	0.48
0.4	0.1	yadI	1	20.8167	0.708
0.4	0.1	AG1	1	20.8167	0.85
0.4	0.1	ptsI	1	21.0667	0.806
0.4	0.1	clcB	1	21.0667	0.472
0.4	0.1	ycaM	1	21.0667	0.48
0.4	0.1	yadI	1	21.0667	0.715
0.4	0.1	AG1	1	21.0667	0.861
0.4	0.1	ptsI	1	21.3167	0.803
0.4	0.1	clcB	1	21.3167	0.484
0.4	0.1	ycaM	1	21.3167	0.482
0.4	0.1	yadI	1	21.3167	0.716
0.4	0.1	AG1	1	21.3167	0.857
0.4	0.1	ptsI	1	21.5667	0.801
0.4	0.1	clcB	1	21.5667	0.477
0.4	0.1	ycaM	1	21.5667	0.48
0.4	0.1	yadI	1	21.5667	0.716
0.4	0.1	AG1	1	21.5667	0.855
0.4	0.1	ptsI	1	21.8167	0.805
0.4	0.1	clcB	1	21.8167	0.462
0.4	0.1	ycaM	1	21.8167	0.483
0.4	0.1	yadI	1	21.8167	0.724
0.4	0.1	AG1	1	21.8167	0.865
0.4	0.1	ptsI	1	22.0667	0.823
0.4	0.1	clcB	1	22.0667	0.467
0.4	0.1	ycaM	1	22.0667	0.481
0.4	0.1	yadI	1	22.0667	0.726
0.4	0.1	AG1	1	22.0667	0.874
0.4	0.1	ptsI	1	22.9333	0.654

0.4	0.1	clcB	1	22.9333	0.384
0.4	0.1	ycaM	1	22.9333	0.374
0.4	0.1	yadI	1	22.9333	0.792
0.4	0.1	AG1	1	22.9333	0.687
0.4	0.1	ptsI	1	23.1833	0.722
0.4	0.1	clcB	1	23.1833	0.366
0.4	0.1	ycaM	1	23.1833	0.37
0.4	0.1	yadI	1	23.1833	0.77
0.4	0.1	AG1	1	23.1833	0.703
0.4	0.1	ptsI	1	23.4333	0.727
0.4	0.1	clcB	1	23.4333	0.365
0.4	0.1	ycaM	1	23.4333	0.367
0.4	0.1	yadI	1	23.4333	0.777
0.4	0.1	AG1	1	23.4333	0.718
0.4	0.1	ptsI	1	23.6833	0.769
0.4	0.1	clcB	1	23.6833	0.364
0.4	0.1	ycaM	1	23.6833	0.365
0.4	0.1	yadI	1	23.6833	0.779
0.4	0.1	AG1	1	23.6833	0.742
0.4	0.1	ptsI	1	23.9333	0.76
0.4	0.1	clcB	1	23.9333	0.368
0.4	0.1	ycaM	1	23.9333	0.365
0.4	0.1	yadI	1	23.9333	0.783
0.4	0.1	AG1	1	23.9333	0.763
0.4	0.1	ptsI	1	24.1833	0.798
0.4	0.1	clcB	1	24.1833	0.364
0.4	0.1	ycaM	1	24.1833	0.366
0.4	0.1	yadI	1	24.1833	0.788
0.4	0.1	AG1	1	24.1833	0.777
0.4	0.1	ptsI	1	24.4333	0.805
0.4	0.1	clcB	1	24.4333	0.367
0.4	0.1	ycaM	1	24.4333	0.368
0.4	0.1	yadI	1	24.4333	0.797
0.4	0.1	AG1	1	24.4333	0.778
0.4	0.1	ptsI	1	24.6833	0.804
0.4	0.1	clcB	1	24.6833	0.364
0.4	0.1	ycaM	1	24.6833	0.364
0.4	0.1	yadI	1	24.6833	0.8
0.4	0.1	AG1	1	24.6833	0.782
0.4	0.1	ptsI	1	24.9333	0.797
0.4	0.1	clcB	1	24.9333	0.364
0.4	0.1	ycaM	1	24.9333	0.366
0.4	0.1	yadI	1	24.9333	0.807
0.4	0.1	AG1	1	24.9333	0.797
0.4	0.1	ptsI	1	25.1833	0.799
0.4	0.1	clcB	1	25.1833	0.365
0.4	0.1	ycaM	1	25.1833	0.367
0.4	0.1	yadI	1	25.1833	0.812
0.4	0.1	AG1	1	25.1833	0.794
0.4	0.1	ptsI	1	25.4333	0.814
0.4	0.1	clcB	1	25.4333	0.366
0.4	0.1	ycaM	1	25.4333	0.366
0.4	0.1	yadI	1	25.4333	0.821

0.4	0.1	AG1	1	25.4333	0.803
0.4	0.1	ptsI	1	25.6833	0.812
0.4	0.1	clcB	1	25.6833	0.367
0.4	0.1	ycaM	1	25.6833	0.367
0.4	0.1	yadI	1	25.6833	0.823
0.4	0.1	AG1	1	25.6833	0.803
0.4	0.1	ptsI	1	25.9333	0.824
0.4	0.1	clcB	1	25.9333	0.369
0.4	0.1	ycaM	1	25.9333	0.363
0.4	0.1	yadI	1	25.9333	0.836
0.4	0.1	AG1	1	25.9333	0.81
0.4	0.1	ptsI	1	26.1833	0.826
0.4	0.1	clcB	1	26.1833	0.369
0.4	0.1	ycaM	1	26.1833	0.367
0.4	0.1	yadI	1	26.1833	0.835
0.4	0.1	AG1	1	26.1833	0.812
0.4	0.1	ptsI	1	26.4333	0.819
0.4	0.1	clcB	1	26.4333	0.37
0.4	0.1	ycaM	1	26.4333	0.367
0.4	0.1	yadI	1	26.4333	0.834
0.4	0.1	AG1	1	26.4333	0.81
0.4	0.1	ptsI	1	26.6833	0.823
0.4	0.1	clcB	1	26.6833	0.372
0.4	0.1	ycaM	1	26.6833	0.37
0.4	0.1	yadI	1	26.6833	0.841
0.4	0.1	AG1	1	26.6833	0.818
0.4	0.1	ptsI	1	26.9333	0.829
0.4	0.1	clcB	1	26.9333	0.374
0.4	0.1	ycaM	1	26.9333	0.371
0.4	0.1	yadI	1	26.9333	0.837
0.4	0.1	AG1	1	26.9333	0.817
0.4	0.1	ptsI	1	27.1833	0.836
0.4	0.1	clcB	1	27.1833	0.373
0.4	0.1	ycaM	1	27.1833	0.372
0.4	0.1	yadI	1	27.1833	0.84
0.4	0.1	AG1	1	27.1833	0.824
0.4	0.1	ptsI	1	27.4333	0.823
0.4	0.1	clcB	1	27.4333	0.372
0.4	0.1	ycaM	1	27.4333	0.372
0.4	0.1	yadI	1	27.4333	0.844
0.4	0.1	AG1	1	27.4333	0.817
0.4	0.1	ptsI	1	27.6833	0.833
0.4	0.1	clcB	1	27.6833	0.376
0.4	0.1	ycaM	1	27.6833	0.372
0.4	0.1	yadI	1	27.6833	0.846
0.4	0.1	AG1	1	27.6833	0.832
0.4	0.1	ptsI	1	27.9333	0.824
0.4	0.1	clcB	1	27.9333	0.375
0.4	0.1	ycaM	1	27.9333	0.377
0.4	0.1	yadI	1	27.9333	0.811
0.4	0.1	AG1	1	27.9333	0.828
0.4	0.1	ptsI	1	28.1833	0.841
0.4	0.1	clcB	1	28.1833	0.377

0.4	0.1	ycaM	1	28.1833	0.375
0.4	0.1	yadI	1	28.1833	0.852
0.4	0.1	AG1	1	28.1833	0.838
0.4	0.1	ptsI	1	28.4333	0.838
0.4	0.1	clcB	1	28.4333	0.378
0.4	0.1	ycaM	1	28.4333	0.376
0.4	0.1	yadI	1	28.4333	0.85
0.4	0.1	AG1	1	28.4333	0.838
0.4	0.1	ptsI	1	28.6833	0.842
0.4	0.1	clcB	1	28.6833	0.38
0.4	0.1	ycaM	1	28.6833	0.376
0.4	0.1	yadI	1	28.6833	0.854
0.4	0.1	AG1	1	28.6833	0.843
0.4	0.1	ptsI	1	28.9333	0.847
0.4	0.1	clcB	1	28.9333	0.38
0.4	0.1	ycaM	1	28.9333	0.376
0.4	0.1	yadI	1	28.9333	0.858
0.4	0.1	AG1	1	28.9333	0.849
0.4	0.1	ptsI	1	29.1833	0.838
0.4	0.1	clcB	1	29.1833	0.379
0.4	0.1	ycaM	1	29.1833	0.375
0.4	0.1	yadI	1	29.1833	0.857
0.4	0.1	AG1	1	29.1833	0.843
0.4	0.1	ptsI	1	29.4333	0.847
0.4	0.1	clcB	1	29.4333	0.383
0.4	0.1	ycaM	1	29.4333	0.376
0.4	0.1	yadI	1	29.4333	0.86
0.4	0.1	AG1	1	29.4333	0.857
0.4	0.1	ptsI	1	29.6833	0.845
0.4	0.1	clcB	1	29.6833	0.383
0.4	0.1	ycaM	1	29.6833	0.378
0.4	0.1	yadI	1	29.6833	0.862
0.4	0.1	AG1	1	29.6833	0.858
0.4	0.1	ptsI	1	29.9333	0.851
0.4	0.1	clcB	1	29.9333	0.386
0.4	0.1	ycaM	1	29.9333	0.377
0.4	0.1	yadI	1	29.9333	0.864
0.4	0.1	AG1	1	29.9333	0.868
0.4	0.1	ptsI	1	30.1833	0.857
0.4	0.1	clcB	1	30.1833	0.385
0.4	0.1	ycaM	1	30.1833	0.378
0.4	0.1	yadI	1	30.1833	0.867
0.4	0.1	AG1	1	30.1833	0.875
0.4	0.1	ptsI	1	30.4333	0.853
0.4	0.1	clcB	1	30.4333	0.387
0.4	0.1	ycaM	1	30.4333	0.38
0.4	0.1	yadI	1	30.4333	0.864
0.4	0.1	AG1	1	30.4333	0.878
0.4	0.1	ptsI	1	30.6833	0.848
0.4	0.1	clcB	1	30.6833	0.387
0.4	0.1	ycaM	1	30.6833	0.381
0.4	0.1	yadI	1	30.6833	0.876
0.4	0.1	AG1	1	30.6833	0.874

0.4	0.1	ptsI	1	30.9333	0.86
0.4	0.1	clcB	1	30.9333	0.387
0.4	0.1	ycaM	1	30.9333	0.384
0.4	0.1	yadI	1	30.9333	0.879
0.4	0.1	AG1	1	30.9333	0.884
0.4	0.1	ptsI	1	31.1833	0.853
0.4	0.1	clcB	1	31.1833	0.386
0.4	0.1	ycaM	1	31.1833	0.382
0.4	0.1	yadI	1	31.1833	0.88
0.4	0.1	AG1	1	31.1833	0.884
0.4	0.1	ptsI	1	31.4333	0.862
0.4	0.1	clcB	1	31.4333	0.393
0.4	0.1	ycaM	1	31.4333	0.384
0.4	0.1	yadI	1	31.4333	0.884
0.4	0.1	AG1	1	31.4333	0.9
0.4	0.1	ptsI	1	31.6833	0.864
0.4	0.1	clcB	1	31.6833	0.39
0.4	0.1	ycaM	1	31.6833	0.387
0.4	0.1	yadI	1	31.6833	0.888
0.4	0.1	AG1	1	31.6833	0.903
0.4	0.1	ptsI	1	31.9333	0.865
0.4	0.1	clcB	1	31.9333	0.396
0.4	0.1	ycaM	1	31.9333	0.386
0.4	0.1	yadI	1	31.9333	0.881
0.4	0.1	AG1	1	31.9333	0.909
0.4	0.1	ptsI	1	32.1833	0.864
0.4	0.1	clcB	1	32.1833	0.398
0.4	0.1	ycaM	1	32.1833	0.391
0.4	0.1	yadI	1	32.1833	0.885
0.4	0.1	AG1	1	32.1833	0.917
0.4	0.1	ptsI	1	32.4333	0.862
0.4	0.1	clcB	1	32.4333	0.393
0.4	0.1	ycaM	1	32.4333	0.387
0.4	0.1	yadI	1	32.4333	0.873
0.4	0.1	AG1	1	32.4333	0.913
0.4	0.1	ptsI	1	32.6833	0.864
0.4	0.1	clcB	1	32.6833	0.398
0.4	0.1	ycaM	1	32.6833	0.394
0.4	0.1	yadI	1	32.6833	0.894
0.4	0.1	AG1	1	32.6833	0.927
0.4	0.1	ptsI	1	32.9333	0.865
0.4	0.1	clcB	1	32.9333	0.396
0.4	0.1	ycaM	1	32.9333	0.393
0.4	0.1	yadI	1	32.9333	0.891
0.4	0.1	AG1	1	32.9333	0.937
0.4	0.1	ptsI	1	33.1833	0.862
0.4	0.1	clcB	1	33.1833	0.397
0.4	0.1	ycaM	1	33.1833	0.4
0.4	0.1	yadI	1	33.1833	0.879
0.4	0.1	AG1	1	33.1833	0.942
0.4	0.1	ptsI	1	33.4333	0.875
0.4	0.1	clcB	1	33.4333	0.403
0.4	0.1	ycaM	1	33.4333	0.398

0.4	0.1	yadI	1	33.4333	0.902
0.4	0.1	AG1	1	33.4333	0.955
0.4	0.1	ptsI	1	33.6833	0.866
0.4	0.1	clcB	1	33.6833	0.404
0.4	0.1	ycaM	1	33.6833	0.404
0.4	0.1	yadI	1	33.6833	0.881
0.4	0.1	AG1	1	33.6833	0.962
0.4	0.1	ptsI	1	33.9333	0.872
0.4	0.1	clcB	1	33.9333	0.404
0.4	0.1	ycaM	1	33.9333	0.4
0.4	0.1	yadI	1	33.9333	0.906
0.4	0.1	AG1	1	33.9333	0.974
0.4	0.1	ptsI	1	34.1833	0.864
0.4	0.1	clcB	1	34.1833	0.409
0.4	0.1	ycaM	1	34.1833	0.41
0.4	0.1	yadI	1	34.1833	0.901
0.4	0.1	AG1	1	34.1833	0.981
0.4	0.1	ptsI	1	34.4333	0.876
0.4	0.1	clcB	1	34.4333	0.406
0.4	0.1	ycaM	1	34.4333	0.405
0.4	0.1	yadI	1	34.4333	0.911
0.4	0.1	AG1	1	34.4333	0.992
0.4	0.1	ptsI	1	34.6833	0.879
0.4	0.1	clcB	1	34.6833	0.41
0.4	0.1	ycaM	1	34.6833	0.41
0.4	0.1	yadI	1	34.6833	0.91
0.4	0.1	AG1	1	34.6833	1.006
0.4	0.1	ptsI	1	34.9333	0.876
0.4	0.1	clcB	1	34.9333	0.407
0.4	0.1	ycaM	1	34.9333	0.408
0.4	0.1	yadI	1	34.9333	0.909
0.4	0.1	AG1	1	34.9333	1.014
0.4	0.1	ptsI	1	35.1833	0.878
0.4	0.1	clcB	1	35.1833	0.409
0.4	0.1	ycaM	1	35.1833	0.408
0.4	0.1	yadI	1	35.1833	0.911
0.4	0.1	AG1	1	35.1833	1.025
0.4	0.1	ptsI	1	35.4333	0.878
0.4	0.1	clcB	1	35.4333	0.408
0.4	0.1	ycaM	1	35.4333	0.409
0.4	0.1	yadI	1	35.4333	0.914
0.4	0.1	AG1	1	35.4333	1.032
0.4	0.1	ptsI	1	35.6833	0.876
0.4	0.1	clcB	1	35.6833	0.41
0.4	0.1	ycaM	1	35.6833	0.411
0.4	0.1	yadI	1	35.6833	0.916
0.4	0.1	AG1	1	35.6833	1.041
0.4	0.1	ptsI	1	35.9333	0.866
0.4	0.1	clcB	1	35.9333	0.41
0.4	0.1	ycaM	1	35.9333	0.414
0.4	0.1	yadI	1	35.9333	0.9
0.4	0.1	AG1	1	35.9333	1.041
0.4	0.1	ptsI	1	36.1833	0.882

0.4	0.1	clcB	1	36.1833	0.414
0.4	0.1	ycaM	1	36.1833	0.417
0.4	0.1	yadI	1	36.1833	0.918
0.4	0.1	AG1	1	36.1833	1.052
0.4	0.1	ptsI	1	36.4333	0.878
0.4	0.1	clcB	1	36.4333	0.413
0.4	0.1	ycaM	1	36.4333	0.418
0.4	0.1	yadI	1	36.4333	0.926
0.4	0.1	AG1	1	36.4333	1.066
0.4	0.1	ptsI	1	36.6833	0.88
0.4	0.1	clcB	1	36.6833	0.414
0.4	0.1	ycaM	1	36.6833	0.416
0.4	0.1	yadI	1	36.6833	0.924
0.4	0.1	AG1	1	36.6833	1.074
0.8	0.1	ptsI	1	0	0.223
0.8	0.1	clcB	1	0	0.257
0.8	0.1	ycaM	1	0	0.243
0.8	0.1	yadI	1	0	0.253
0.8	0.1	AG1	1	0	0.254
0.8	0.1	ptsI	1	0.25	0.222
0.8	0.1	clcB	1	0.25	0.242
0.8	0.1	ycaM	1	0.25	0.229
0.8	0.1	yadI	1	0.25	0.244
0.8	0.1	AG1	1	0.25	0.239
0.8	0.1	ptsI	1	0.5	0.219
0.8	0.1	clcB	1	0.5	0.239
0.8	0.1	ycaM	1	0.5	0.228
0.8	0.1	yadI	1	0.5	0.241
0.8	0.1	AG1	1	0.5	0.234
0.8	0.1	ptsI	1	0.75	0.219
0.8	0.1	clcB	1	0.75	0.237
0.8	0.1	ycaM	1	0.75	0.224
0.8	0.1	yadI	1	0.75	0.238
0.8	0.1	AG1	1	0.75	0.232
0.8	0.1	ptsI	1	1	0.22
0.8	0.1	clcB	1	1	0.238
0.8	0.1	ycaM	1	1	0.224
0.8	0.1	yadI	1	1	0.239
0.8	0.1	AG1	1	1	0.232
0.8	0.1	ptsI	1	1.25	0.22
0.8	0.1	clcB	1	1.25	0.24
0.8	0.1	ycaM	1	1.25	0.223
0.8	0.1	yadI	1	1.25	0.242
0.8	0.1	AG1	1	1.25	0.232
0.8	0.1	ptsI	1	1.5	0.222
0.8	0.1	clcB	1	1.5	0.238
0.8	0.1	ycaM	1	1.5	0.226
0.8	0.1	yadI	1	1.5	0.242
0.8	0.1	AG1	1	1.5	0.234
0.8	0.1	ptsI	1	1.75	0.226
0.8	0.1	clcB	1	1.75	0.24
0.8	0.1	ycaM	1	1.75	0.229
0.8	0.1	yadI	1	1.75	0.248

0.8	0.1	AG1	1	1.75	0.234
0.8	0.1	ptsI	1	2	0.231
0.8	0.1	clcB	1	2	0.243
0.8	0.1	ycaM	1	2	0.233
0.8	0.1	yadI	1	2	0.252
0.8	0.1	AG1	1	2	0.238
0.8	0.1	ptsI	1	2.25	0.236
0.8	0.1	clcB	1	2.25	0.248
0.8	0.1	ycaM	1	2.25	0.239
0.8	0.1	yadI	1	2.25	0.257
0.8	0.1	AG1	1	2.25	0.242
0.8	0.1	ptsI	1	2.5	0.241
0.8	0.1	clcB	1	2.5	0.252
0.8	0.1	ycaM	1	2.5	0.244
0.8	0.1	yadI	1	2.5	0.262
0.8	0.1	AG1	1	2.5	0.244
0.8	0.1	ptsI	1	2.75	0.248
0.8	0.1	clcB	1	2.75	0.256
0.8	0.1	ycaM	1	2.75	0.246
0.8	0.1	yadI	1	2.75	0.271
0.8	0.1	AG1	1	2.75	0.248
0.8	0.1	ptsI	1	3	0.255
0.8	0.1	clcB	1	3	0.261
0.8	0.1	ycaM	1	3	0.255
0.8	0.1	yadI	1	3	0.28
0.8	0.1	AG1	1	3	0.253
0.8	0.1	ptsI	1	3.25	0.263
0.8	0.1	clcB	1	3.25	0.265
0.8	0.1	ycaM	1	3.25	0.265
0.8	0.1	yadI	1	3.25	0.291
0.8	0.1	AG1	1	3.25	0.261
0.8	0.1	ptsI	1	3.5	0.273
0.8	0.1	clcB	1	3.5	0.27
0.8	0.1	ycaM	1	3.5	0.266
0.8	0.1	yadI	1	3.5	0.299
0.8	0.1	AG1	1	3.5	0.263
0.8	0.1	ptsI	1	3.75	0.279
0.8	0.1	clcB	1	3.75	0.272
0.8	0.1	ycaM	1	3.75	0.27
0.8	0.1	yadI	1	3.75	0.303
0.8	0.1	AG1	1	3.75	0.266
0.8	0.1	ptsI	1	4	0.296
0.8	0.1	clcB	1	4	0.283
0.8	0.1	ycaM	1	4	0.282
0.8	0.1	yadI	1	4	0.316
0.8	0.1	AG1	1	4	0.271
0.8	0.1	ptsI	1	4.25	0.289
0.8	0.1	clcB	1	4.25	0.286
0.8	0.1	ycaM	1	4.25	0.279
0.8	0.1	yadI	1	4.25	0.316
0.8	0.1	AG1	1	4.25	0.28
0.8	0.1	ptsI	1	4.5	0.297
0.8	0.1	clcB	1	4.5	0.292

0.8	0.1	ycaM	1	4.5	0.286
0.8	0.1	yadI	1	4.5	0.326
0.8	0.1	AG1	1	4.5	0.288
0.8	0.1	ptsI	1	4.75	0.303
0.8	0.1	clcB	1	4.75	0.298
0.8	0.1	ycaM	1	4.75	0.287
0.8	0.1	yadI	1	4.75	0.327
0.8	0.1	AG1	1	4.75	0.29
0.8	0.1	ptsI	1	5	0.309
0.8	0.1	clcB	1	5	0.303
0.8	0.1	ycaM	1	5	0.295
0.8	0.1	yadI	1	5	0.336
0.8	0.1	AG1	1	5	0.298
0.8	0.1	ptsI	1	5.25	0.315
0.8	0.1	clcB	1	5.25	0.31
0.8	0.1	ycaM	1	5.25	0.299
0.8	0.1	yadI	1	5.25	0.342
0.8	0.1	AG1	1	5.25	0.301
0.8	0.1	ptsI	1	5.5	0.325
0.8	0.1	clcB	1	5.5	0.314
0.8	0.1	ycaM	1	5.5	0.305
0.8	0.1	yadI	1	5.5	0.352
0.8	0.1	AG1	1	5.5	0.309
0.8	0.1	ptsI	1	5.75	0.334
0.8	0.1	clcB	1	5.75	0.322
0.8	0.1	ycaM	1	5.75	0.311
0.8	0.1	yadI	1	5.75	0.363
0.8	0.1	AG1	1	5.75	0.317
0.8	0.1	ptsI	1	6	0.339
0.8	0.1	clcB	1	6	0.325
0.8	0.1	ycaM	1	6	0.311
0.8	0.1	yadI	1	6	0.36
0.8	0.1	AG1	1	6	0.315
0.8	0.1	ptsI	1	6.25	0.344
0.8	0.1	clcB	1	6.25	0.332
0.8	0.1	ycaM	1	6.25	0.316
0.8	0.1	yadI	1	6.25	0.358
0.8	0.1	AG1	1	6.25	0.321
0.8	0.1	ptsI	1	6.5	0.35
0.8	0.1	clcB	1	6.5	0.337
0.8	0.1	ycaM	1	6.5	0.322
0.8	0.1	yadI	1	6.5	0.363
0.8	0.1	AG1	1	6.5	0.324
0.8	0.1	ptsI	1	6.75	0.358
0.8	0.1	clcB	1	6.75	0.344
0.8	0.1	ycaM	1	6.75	0.33
0.8	0.1	yadI	1	6.75	0.37
0.8	0.1	AG1	1	6.75	0.334
0.8	0.1	ptsI	1	7	0.369
0.8	0.1	clcB	1	7	0.351
0.8	0.1	ycaM	1	7	0.337
0.8	0.1	yadI	1	7	0.377
0.8	0.1	AG1	1	7	0.34

0.8	0.1	ptsI	1	7.25	0.377
0.8	0.1	clcB	1	7.25	0.353
0.8	0.1	ycaM	1	7.25	0.34
0.8	0.1	yadI	1	7.25	0.381
0.8	0.1	AG1	1	7.25	0.348
0.8	0.1	ptsI	1	7.5	0.388
0.8	0.1	clcB	1	7.5	0.36
0.8	0.1	ycaM	1	7.5	0.348
0.8	0.1	yadI	1	7.5	0.392
0.8	0.1	AG1	1	7.5	0.357
0.8	0.1	ptsI	1	7.75	0.402
0.8	0.1	clcB	1	7.75	0.373
0.8	0.1	ycaM	1	7.75	0.349
0.8	0.1	yadI	1	7.75	0.401
0.8	0.1	AG1	1	7.75	0.369
0.8	0.1	ptsI	1	8	0.415
0.8	0.1	clcB	1	8	0.379
0.8	0.1	ycaM	1	8	0.347
0.8	0.1	yadI	1	8	0.402
0.8	0.1	AG1	1	8	0.375
0.8	0.1	ptsI	1	8.25	0.436
0.8	0.1	clcB	1	8.25	0.382
0.8	0.1	ycaM	1	8.25	0.346
0.8	0.1	yadI	1	8.25	0.41
0.8	0.1	AG1	1	8.25	0.383
0.8	0.1	ptsI	1	8.5	0.449
0.8	0.1	clcB	1	8.5	0.394
0.8	0.1	ycaM	1	8.5	0.35
0.8	0.1	yadI	1	8.5	0.415
0.8	0.1	AG1	1	8.5	0.389
0.8	0.1	ptsI	1	8.75	0.462
0.8	0.1	clcB	1	8.75	0.401
0.8	0.1	ycaM	1	8.75	0.352
0.8	0.1	yadI	1	8.75	0.42
0.8	0.1	AG1	1	8.75	0.399
0.8	0.1	ptsI	1	9	0.475
0.8	0.1	clcB	1	9	0.406
0.8	0.1	ycaM	1	9	0.354
0.8	0.1	yadI	1	9	0.433
0.8	0.1	AG1	1	9	0.407
0.8	0.1	ptsI	1	9.25	0.489
0.8	0.1	clcB	1	9.25	0.41
0.8	0.1	ycaM	1	9.25	0.358
0.8	0.1	yadI	1	9.25	0.434
0.8	0.1	AG1	1	9.25	0.422
0.8	0.1	ptsI	1	9.5	0.502
0.8	0.1	clcB	1	9.5	0.42
0.8	0.1	ycaM	1	9.5	0.367
0.8	0.1	yadI	1	9.5	0.434
0.8	0.1	AG1	1	9.5	0.444
0.8	0.1	ptsI	1	9.75	0.538
0.8	0.1	clcB	1	9.75	0.432
0.8	0.1	ycaM	1	9.75	0.373

0.8	0.1	yadI	1	9.75	0.441
0.8	0.1	AG1	1	9.75	0.459
0.8	0.1	ptsI	1	10	0.485
0.8	0.1	clcB	1	10	0.432
0.8	0.1	ycaM	1	10	0.37
0.8	0.1	yadI	1	10	0.421
0.8	0.1	AG1	1	10	0.43
0.8	0.1	ptsI	1	10.25	0.517
0.8	0.1	clcB	1	10.25	0.437
0.8	0.1	ycaM	1	10.25	0.387
0.8	0.1	yadI	1	10.25	0.428
0.8	0.1	AG1	1	10.25	0.448
0.8	0.1	ptsI	1	10.5	0.542
0.8	0.1	clcB	1	10.5	0.441
0.8	0.1	ycaM	1	10.5	0.385
0.8	0.1	yadI	1	10.5	0.435
0.8	0.1	AG1	1	10.5	0.463
0.8	0.1	ptsI	1	10.75	0.522
0.8	0.1	clcB	1	10.75	0.448
0.8	0.1	ycaM	1	10.75	0.386
0.8	0.1	yadI	1	10.75	0.423
0.8	0.1	AG1	1	10.75	0.431
0.8	0.1	ptsI	1	11	0.521
0.8	0.1	clcB	1	11	0.467
0.8	0.1	ycaM	1	11	0.39
0.8	0.1	yadI	1	11	0.435
0.8	0.1	AG1	1	11	0.452
0.8	0.1	ptsI	1	11.25	0.595
0.8	0.1	clcB	1	11.25	0.493
0.8	0.1	ycaM	1	11.25	0.404
0.8	0.1	yadI	1	11.25	0.439
0.8	0.1	AG1	1	11.25	0.49
0.8	0.1	ptsI	1	11.5	0.577
0.8	0.1	clcB	1	11.5	0.491
0.8	0.1	ycaM	1	11.5	0.4
0.8	0.1	yadI	1	11.5	0.44
0.8	0.1	AG1	1	11.5	0.482
0.8	0.1	ptsI	1	11.75	0.579
0.8	0.1	clcB	1	11.75	0.499
0.8	0.1	ycaM	1	11.75	0.404
0.8	0.1	yadI	1	11.75	0.426
0.8	0.1	AG1	1	11.75	0.475
0.8	0.1	ptsI	1	12	0.646
0.8	0.1	clcB	1	12	0.546
0.8	0.1	ycaM	1	12	0.423
0.8	0.1	yadI	1	12	0.43
0.8	0.1	AG1	1	12	0.526
0.8	0.1	ptsI	1	12.25	0.6
0.8	0.1	clcB	1	12.25	0.525
0.8	0.1	ycaM	1	12.25	0.414
0.8	0.1	yadI	1	12.25	0.43
0.8	0.1	AG1	1	12.25	0.507
0.8	0.1	ptsI	1	12.5	0.641

0.8	0.1	clcB	1	12.5	0.553
0.8	0.1	ycaM	1	12.5	0.423
0.8	0.1	yadI	1	12.5	0.429
0.8	0.1	AG1	1	12.5	0.544
0.8	0.1	ptsI	1	12.75	0.658
0.8	0.1	clcB	1	12.75	0.583
0.8	0.1	ycaM	1	12.75	0.436
0.8	0.1	yadI	1	12.75	0.447
0.8	0.1	AG1	1	12.75	0.569
0.8	0.1	ptsI	1	13	0.668
0.8	0.1	clcB	1	13	0.598
0.8	0.1	ycaM	1	13	0.465
0.8	0.1	yadI	1	13	0.447
0.8	0.1	AG1	1	13	0.627
0.8	0.1	ptsI	1	13.25	0.657
0.8	0.1	clcB	1	13.25	0.582
0.8	0.1	ycaM	1	13.25	0.451
0.8	0.1	yadI	1	13.25	0.44
0.8	0.1	AG1	1	13.25	0.613
0.8	0.1	ptsI	1	13.5	0.698
0.8	0.1	clcB	1	13.5	0.615
0.8	0.1	ycaM	1	13.5	0.471
0.8	0.1	yadI	1	13.5	0.444
0.8	0.1	AG1	1	13.5	0.668
0.8	0.1	ptsI	1	13.75	0.669
0.8	0.1	clcB	1	13.75	0.602
0.8	0.1	ycaM	1	13.75	0.471
0.8	0.1	yadI	1	13.75	0.454
0.8	0.1	AG1	1	13.75	0.688
0.8	0.1	ptsI	1	14	0.709
0.8	0.1	clcB	1	14	0.626
0.8	0.1	ycaM	1	14	0.485
0.8	0.1	yadI	1	14	0.447
0.8	0.1	AG1	1	14	0.751
0.8	0.1	ptsI	1	14.25	0.716
0.8	0.1	clcB	1	14.25	0.655
0.8	0.1	ycaM	1	14.25	0.498
0.8	0.1	yadI	1	14.25	0.459
0.8	0.1	AG1	1	14.25	0.777
0.8	0.1	ptsI	1	14.5	0.713
0.8	0.1	clcB	1	14.5	0.666
0.8	0.1	ycaM	1	14.5	0.503
0.8	0.1	yadI	1	14.5	0.454
0.8	0.1	AG1	1	14.5	0.785
0.8	0.1	ptsI	1	14.75	0.703
0.8	0.1	clcB	1	14.75	0.646
0.8	0.1	ycaM	1	14.75	0.497
0.8	0.1	yadI	1	14.75	0.451
0.8	0.1	AG1	1	14.75	0.76
0.8	0.1	ptsI	1	15	0.738
0.8	0.1	clcB	1	15	0.681
0.8	0.1	ycaM	1	15	0.549
0.8	0.1	yadI	1	15	0.456

0.8	0.1	AG1	1	15	0.8
0.8	0.1	ptsI	1	15.25	0.746
0.8	0.1	clcB	1	15.25	0.701
0.8	0.1	ycaM	1	15.25	0.589
0.8	0.1	yadI	1	15.25	0.47
0.8	0.1	AG1	1	15.25	0.838
0.8	0.1	ptsI	1	15.5	0.727
0.8	0.1	clcB	1	15.5	0.683
0.8	0.1	ycaM	1	15.5	0.575
0.8	0.1	yadI	1	15.5	0.466
0.8	0.1	AG1	1	15.5	0.819
0.8	0.1	ptsI	1	15.75	0.728
0.8	0.1	clcB	1	15.75	0.694
0.8	0.1	ycaM	1	15.75	0.564
0.8	0.1	yadI	1	15.75	0.462
0.8	0.1	AG1	1	15.75	0.799
0.8	0.1	ptsI	1	16	0.708
0.8	0.1	clcB	1	16	0.675
0.8	0.1	ycaM	1	16	0.548
0.8	0.1	yadI	1	16	0.458
0.8	0.1	AG1	1	16	0.764
0.8	0.1	ptsI	1	16.25	0.759
0.8	0.1	clcB	1	16.25	0.725
0.8	0.1	ycaM	1	16.25	0.614
0.8	0.1	yadI	1	16.25	0.474
0.8	0.1	AG1	1	16.25	0.82
0.8	0.1	ptsI	1	16.5	0.766
0.8	0.1	clcB	1	16.5	0.725
0.8	0.1	ycaM	1	16.5	0.64
0.8	0.1	yadI	1	16.5	0.476
0.8	0.1	AG1	1	16.5	0.822
0.8	0.1	ptsI	1	17.0667	0.596
0.8	0.1	clcB	1	17.0667	0.493
0.8	0.1	ycaM	1	17.0667	0.517
0.8	0.1	yadI	1	17.0667	0.638
0.8	0.1	AG1	1	17.0667	0.855
0.8	0.1	ptsI	1	17.3167	0.639
0.8	0.1	clcB	1	17.3167	0.467
0.8	0.1	ycaM	1	17.3167	0.484
0.8	0.1	yadI	1	17.3167	0.63
0.8	0.1	AG1	1	17.3167	0.812
0.8	0.1	ptsI	1	17.5667	0.651
0.8	0.1	clcB	1	17.5667	0.46
0.8	0.1	ycaM	1	17.5667	0.476
0.8	0.1	yadI	1	17.5667	0.633
0.8	0.1	AG1	1	17.5667	0.821
0.8	0.1	ptsI	1	17.8167	0.64
0.8	0.1	clcB	1	17.8167	0.465
0.8	0.1	ycaM	1	17.8167	0.477
0.8	0.1	yadI	1	17.8167	0.644
0.8	0.1	AG1	1	17.8167	0.809
0.8	0.1	ptsI	1	18.0667	0.651
0.8	0.1	clcB	1	18.0667	0.464

0.8	0.1	ycaM	1	18.0667	0.471
0.8	0.1	yadI	1	18.0667	0.657
0.8	0.1	AG1	1	18.0667	0.814
0.8	0.1	ptsI	1	18.3167	0.675
0.8	0.1	clcB	1	18.3167	0.45
0.8	0.1	ycaM	1	18.3167	0.468
0.8	0.1	yadI	1	18.3167	0.649
0.8	0.1	AG1	1	18.3167	0.814
0.8	0.1	ptsI	1	18.5667	0.756
0.8	0.1	clcB	1	18.5667	0.453
0.8	0.1	ycaM	1	18.5667	0.469
0.8	0.1	yadI	1	18.5667	0.656
0.8	0.1	AG1	1	18.5667	0.843
0.8	0.1	ptsI	1	18.8167	0.761
0.8	0.1	clcB	1	18.8167	0.454
0.8	0.1	ycaM	1	18.8167	0.465
0.8	0.1	yadI	1	18.8167	0.667
0.8	0.1	AG1	1	18.8167	0.849
0.8	0.1	ptsI	1	19.0667	0.791
0.8	0.1	clcB	1	19.0667	0.46
0.8	0.1	ycaM	1	19.0667	0.466
0.8	0.1	yadI	1	19.0667	0.689
0.8	0.1	AG1	1	19.0667	0.87
0.8	0.1	ptsI	1	19.3167	0.768
0.8	0.1	clcB	1	19.3167	0.454
0.8	0.1	ycaM	1	19.3167	0.462
0.8	0.1	yadI	1	19.3167	0.683
0.8	0.1	AG1	1	19.3167	0.864
0.8	0.1	ptsI	1	19.5667	0.793
0.8	0.1	clcB	1	19.5667	0.465
0.8	0.1	ycaM	1	19.5667	0.467
0.8	0.1	yadI	1	19.5667	0.695
0.8	0.1	AG1	1	19.5667	0.88
0.8	0.1	ptsI	1	19.8167	0.798
0.8	0.1	clcB	1	19.8167	0.452
0.8	0.1	ycaM	1	19.8167	0.47
0.8	0.1	yadI	1	19.8167	0.697
0.8	0.1	AG1	1	19.8167	0.879
0.8	0.1	ptsI	1	20.0667	0.799
0.8	0.1	clcB	1	20.0667	0.452
0.8	0.1	ycaM	1	20.0667	0.473
0.8	0.1	yadI	1	20.0667	0.71
0.8	0.1	AG1	1	20.0667	0.882
0.8	0.1	ptsI	1	20.3167	0.804
0.8	0.1	clcB	1	20.3167	0.453
0.8	0.1	ycaM	1	20.3167	0.471
0.8	0.1	yadI	1	20.3167	0.714
0.8	0.1	AG1	1	20.3167	0.888
0.8	0.1	ptsI	1	20.5667	0.796
0.8	0.1	clcB	1	20.5667	0.431
0.8	0.1	ycaM	1	20.5667	0.469
0.8	0.1	yadI	1	20.5667	0.711
0.8	0.1	AG1	1	20.5667	0.885

0.8	0.1	ptsI	1	20.8167	0.798
0.8	0.1	clcB	1	20.8167	0.435
0.8	0.1	ycaM	1	20.8167	0.468
0.8	0.1	yadI	1	20.8167	0.713
0.8	0.1	AG1	1	20.8167	0.891
0.8	0.1	ptsI	1	21.0667	0.807
0.8	0.1	clcB	1	21.0667	0.428
0.8	0.1	ycaM	1	21.0667	0.464
0.8	0.1	yadI	1	21.0667	0.723
0.8	0.1	AG1	1	21.0667	0.899
0.8	0.1	ptsI	1	21.3167	0.806
0.8	0.1	clcB	1	21.3167	0.426
0.8	0.1	ycaM	1	21.3167	0.467
0.8	0.1	yadI	1	21.3167	0.729
0.8	0.1	AG1	1	21.3167	0.898
0.8	0.1	ptsI	1	21.5667	0.805
0.8	0.1	clcB	1	21.5667	0.423
0.8	0.1	ycaM	1	21.5667	0.469
0.8	0.1	yadI	1	21.5667	0.725
0.8	0.1	AG1	1	21.5667	0.902
0.8	0.1	ptsI	1	21.8167	0.811
0.8	0.1	clcB	1	21.8167	0.41
0.8	0.1	ycaM	1	21.8167	0.47
0.8	0.1	yadI	1	21.8167	0.73
0.8	0.1	AG1	1	21.8167	0.905
0.8	0.1	ptsI	1	22.0667	0.829
0.8	0.1	clcB	1	22.0667	0.418
0.8	0.1	ycaM	1	22.0667	0.466
0.8	0.1	yadI	1	22.0667	0.743
0.8	0.1	AG1	1	22.0667	0.911
0.8	0.1	ptsI	1	22.9333	0.82
0.8	0.1	clcB	1	22.9333	0.394
0.8	0.1	ycaM	1	22.9333	0.431
0.8	0.1	yadI	1	22.9333	0.74
0.8	0.1	AG1	1	22.9333	0.738
0.8	0.1	ptsI	1	23.1833	0.796
0.8	0.1	clcB	1	23.1833	0.378
0.8	0.1	ycaM	1	23.1833	0.42
0.8	0.1	yadI	1	23.1833	0.724
0.8	0.1	AG1	1	23.1833	0.728
0.8	0.1	ptsI	1	23.4333	0.771
0.8	0.1	clcB	1	23.4333	0.375
0.8	0.1	ycaM	1	23.4333	0.414
0.8	0.1	yadI	1	23.4333	0.728
0.8	0.1	AG1	1	23.4333	0.739
0.8	0.1	ptsI	1	23.6833	0.824
0.8	0.1	clcB	1	23.6833	0.372
0.8	0.1	ycaM	1	23.6833	0.415
0.8	0.1	yadI	1	23.6833	0.738
0.8	0.1	AG1	1	23.6833	0.745
0.8	0.1	ptsI	1	23.9333	0.832
0.8	0.1	clcB	1	23.9333	0.375
0.8	0.1	ycaM	1	23.9333	0.416

0.8	0.1	yadI	1	23.9333	0.738
0.8	0.1	AG1	1	23.9333	0.762
0.8	0.1	ptsI	1	24.1833	0.855
0.8	0.1	clcB	1	24.1833	0.377
0.8	0.1	ycaM	1	24.1833	0.416
0.8	0.1	yadI	1	24.1833	0.75
0.8	0.1	AG1	1	24.1833	0.788
0.8	0.1	ptsI	1	24.4333	0.853
0.8	0.1	clcB	1	24.4333	0.378
0.8	0.1	ycaM	1	24.4333	0.416
0.8	0.1	yadI	1	24.4333	0.758
0.8	0.1	AG1	1	24.4333	0.793
0.8	0.1	ptsI	1	24.6833	0.855
0.8	0.1	clcB	1	24.6833	0.382
0.8	0.1	ycaM	1	24.6833	0.412
0.8	0.1	yadI	1	24.6833	0.761
0.8	0.1	AG1	1	24.6833	0.794
0.8	0.1	ptsI	1	24.9333	0.839
0.8	0.1	clcB	1	24.9333	0.427
0.8	0.1	ycaM	1	24.9333	0.412
0.8	0.1	yadI	1	24.9333	0.765
0.8	0.1	AG1	1	24.9333	0.8
0.8	0.1	ptsI	1	25.1833	0.843
0.8	0.1	clcB	1	25.1833	0.443
0.8	0.1	ycaM	1	25.1833	0.413
0.8	0.1	yadI	1	25.1833	0.77
0.8	0.1	AG1	1	25.1833	0.795
0.8	0.1	ptsI	1	25.4333	0.861
0.8	0.1	clcB	1	25.4333	0.385
0.8	0.1	ycaM	1	25.4333	0.415
0.8	0.1	yadI	1	25.4333	0.777
0.8	0.1	AG1	1	25.4333	0.81
0.8	0.1	ptsI	1	25.6833	0.854
0.8	0.1	clcB	1	25.6833	0.414
0.8	0.1	ycaM	1	25.6833	0.412
0.8	0.1	yadI	1	25.6833	0.782
0.8	0.1	AG1	1	25.6833	0.797
0.8	0.1	ptsI	1	25.9333	0.856
0.8	0.1	clcB	1	25.9333	0.388
0.8	0.1	ycaM	1	25.9333	0.412
0.8	0.1	yadI	1	25.9333	0.788
0.8	0.1	AG1	1	25.9333	0.81
0.8	0.1	ptsI	1	26.1833	0.867
0.8	0.1	clcB	1	26.1833	0.393
0.8	0.1	ycaM	1	26.1833	0.416
0.8	0.1	yadI	1	26.1833	0.792
0.8	0.1	AG1	1	26.1833	0.812
0.8	0.1	ptsI	1	26.4333	0.867
0.8	0.1	clcB	1	26.4333	0.386
0.8	0.1	ycaM	1	26.4333	0.414
0.8	0.1	yadI	1	26.4333	0.792
0.8	0.1	AG1	1	26.4333	0.809
0.8	0.1	ptsI	1	26.6833	0.866

0.8	0.1	clcB	1	26.6833	0.383
0.8	0.1	ycaM	1	26.6833	0.415
0.8	0.1	yadI	1	26.6833	0.796
0.8	0.1	AG1	1	26.6833	0.813
0.8	0.1	ptsI	1	26.9333	0.871
0.8	0.1	clcB	1	26.9333	0.386
0.8	0.1	ycaM	1	26.9333	0.414
0.8	0.1	yadI	1	26.9333	0.799
0.8	0.1	AG1	1	26.9333	0.816
0.8	0.1	ptsI	1	27.1833	0.872
0.8	0.1	clcB	1	27.1833	0.382
0.8	0.1	ycaM	1	27.1833	0.417
0.8	0.1	yadI	1	27.1833	0.803
0.8	0.1	AG1	1	27.1833	0.822
0.8	0.1	ptsI	1	27.4333	0.859
0.8	0.1	clcB	1	27.4333	0.373
0.8	0.1	ycaM	1	27.4333	0.417
0.8	0.1	yadI	1	27.4333	0.802
0.8	0.1	AG1	1	27.4333	0.811
0.8	0.1	ptsI	1	27.6833	0.873
0.8	0.1	clcB	1	27.6833	0.376
0.8	0.1	ycaM	1	27.6833	0.417
0.8	0.1	yadI	1	27.6833	0.805
0.8	0.1	AG1	1	27.6833	0.816
0.8	0.1	ptsI	1	27.9333	0.871
0.8	0.1	clcB	1	27.9333	0.366
0.8	0.1	ycaM	1	27.9333	0.412
0.8	0.1	yadI	1	27.9333	0.79
0.8	0.1	AG1	1	27.9333	0.812
0.8	0.1	ptsI	1	28.1833	0.886
0.8	0.1	clcB	1	28.1833	0.385
0.8	0.1	ycaM	1	28.1833	0.417
0.8	0.1	yadI	1	28.1833	0.806
0.8	0.1	AG1	1	28.1833	0.825
0.8	0.1	ptsI	1	28.4333	0.878
0.8	0.1	clcB	1	28.4333	0.385
0.8	0.1	ycaM	1	28.4333	0.418
0.8	0.1	yadI	1	28.4333	0.812
0.8	0.1	AG1	1	28.4333	0.822
0.8	0.1	ptsI	1	28.6833	0.882
0.8	0.1	clcB	1	28.6833	0.386
0.8	0.1	ycaM	1	28.6833	0.418
0.8	0.1	yadI	1	28.6833	0.818
0.8	0.1	AG1	1	28.6833	0.824
0.8	0.1	ptsI	1	28.9333	0.888
0.8	0.1	clcB	1	28.9333	0.389
0.8	0.1	ycaM	1	28.9333	0.419
0.8	0.1	yadI	1	28.9333	0.815
0.8	0.1	AG1	1	28.9333	0.825
0.8	0.1	ptsI	1	29.1833	0.872
0.8	0.1	clcB	1	29.1833	0.387
0.8	0.1	ycaM	1	29.1833	0.418
0.8	0.1	yadI	1	29.1833	0.814

0.8	0.1	AG1	1	29.1833	0.818
0.8	0.1	ptsI	1	29.4333	0.885
0.8	0.1	clcB	1	29.4333	0.386
0.8	0.1	ycaM	1	29.4333	0.42
0.8	0.1	yadI	1	29.4333	0.821
0.8	0.1	AG1	1	29.4333	0.828
0.8	0.1	ptsI	1	29.6833	0.878
0.8	0.1	clcB	1	29.6833	0.388
0.8	0.1	ycaM	1	29.6833	0.42
0.8	0.1	yadI	1	29.6833	0.831
0.8	0.1	AG1	1	29.6833	0.827
0.8	0.1	ptsI	1	29.9333	0.887
0.8	0.1	clcB	1	29.9333	0.389
0.8	0.1	ycaM	1	29.9333	0.419
0.8	0.1	yadI	1	29.9333	0.826
0.8	0.1	AG1	1	29.9333	0.833
0.8	0.1	ptsI	1	30.1833	0.886
0.8	0.1	clcB	1	30.1833	0.39
0.8	0.1	ycaM	1	30.1833	0.42
0.8	0.1	yadI	1	30.1833	0.825
0.8	0.1	AG1	1	30.1833	0.835
0.8	0.1	ptsI	1	30.4333	0.885
0.8	0.1	clcB	1	30.4333	0.392
0.8	0.1	ycaM	1	30.4333	0.423
0.8	0.1	yadI	1	30.4333	0.831
0.8	0.1	AG1	1	30.4333	0.838
0.8	0.1	ptsI	1	30.6833	0.878
0.8	0.1	clcB	1	30.6833	0.393
0.8	0.1	ycaM	1	30.6833	0.422
0.8	0.1	yadI	1	30.6833	0.841
0.8	0.1	AG1	1	30.6833	0.836
0.8	0.1	ptsI	1	30.9333	0.888
0.8	0.1	clcB	1	30.9333	0.394
0.8	0.1	ycaM	1	30.9333	0.424
0.8	0.1	yadI	1	30.9333	0.843
0.8	0.1	AG1	1	30.9333	0.841
0.8	0.1	ptsI	1	31.1833	0.884
0.8	0.1	clcB	1	31.1833	0.394
0.8	0.1	ycaM	1	31.1833	0.423
0.8	0.1	yadI	1	31.1833	0.845
0.8	0.1	AG1	1	31.1833	0.839
0.8	0.1	ptsI	1	31.4333	0.892
0.8	0.1	clcB	1	31.4333	0.397
0.8	0.1	ycaM	1	31.4333	0.425
0.8	0.1	yadI	1	31.4333	0.851
0.8	0.1	AG1	1	31.4333	0.847
0.8	0.1	ptsI	1	31.6833	0.89
0.8	0.1	clcB	1	31.6833	0.398
0.8	0.1	ycaM	1	31.6833	0.424
0.8	0.1	yadI	1	31.6833	0.854
0.8	0.1	AG1	1	31.6833	0.847
0.8	0.1	ptsI	1	31.9333	0.894
0.8	0.1	clcB	1	31.9333	0.399

0.8	0.1	ycaM	1	31.9333	0.426
0.8	0.1	yadI	1	31.9333	0.848
0.8	0.1	AG1	1	31.9333	0.853
0.8	0.1	ptsI	1	32.1833	0.896
0.8	0.1	clcB	1	32.1833	0.4
0.8	0.1	ycaM	1	32.1833	0.427
0.8	0.1	yadI	1	32.1833	0.852
0.8	0.1	AG1	1	32.1833	0.857
0.8	0.1	ptsI	1	32.4333	0.891
0.8	0.1	clcB	1	32.4333	0.385
0.8	0.1	ycaM	1	32.4333	0.42
0.8	0.1	yadI	1	32.4333	0.838
0.8	0.1	AG1	1	32.4333	0.856
0.8	0.1	ptsI	1	32.6833	0.893
0.8	0.1	clcB	1	32.6833	0.406
0.8	0.1	ycaM	1	32.6833	0.428
0.8	0.1	yadI	1	32.6833	0.861
0.8	0.1	AG1	1	32.6833	0.861
0.8	0.1	ptsI	1	32.9333	0.892
0.8	0.1	clcB	1	32.9333	0.404
0.8	0.1	ycaM	1	32.9333	0.428
0.8	0.1	yadI	1	32.9333	0.863
0.8	0.1	AG1	1	32.9333	0.864
0.8	0.1	ptsI	1	33.1833	0.892
0.8	0.1	clcB	1	33.1833	0.397
0.8	0.1	ycaM	1	33.1833	0.425
0.8	0.1	yadI	1	33.1833	0.858
0.8	0.1	AG1	1	33.1833	0.863
0.8	0.1	ptsI	1	33.4333	0.897
0.8	0.1	clcB	1	33.4333	0.408
0.8	0.1	ycaM	1	33.4333	0.429
0.8	0.1	yadI	1	33.4333	0.874
0.8	0.1	AG1	1	33.4333	0.868
0.8	0.1	ptsI	1	33.6833	0.891
0.8	0.1	clcB	1	33.6833	0.402
0.8	0.1	ycaM	1	33.6833	0.427
0.8	0.1	yadI	1	33.6833	0.864
0.8	0.1	AG1	1	33.6833	0.864
0.8	0.1	ptsI	1	33.9333	0.892
0.8	0.1	clcB	1	33.9333	0.408
0.8	0.1	ycaM	1	33.9333	0.433
0.8	0.1	yadI	1	33.9333	0.876
0.8	0.1	AG1	1	33.9333	0.872
0.8	0.1	ptsI	1	34.1833	0.9
0.8	0.1	clcB	1	34.1833	0.416
0.8	0.1	ycaM	1	34.1833	0.434
0.8	0.1	yadI	1	34.1833	0.88
0.8	0.1	AG1	1	34.1833	0.875
0.8	0.1	ptsI	1	34.4333	0.895
0.8	0.1	clcB	1	34.4333	0.41
0.8	0.1	ycaM	1	34.4333	0.434
0.8	0.1	yadI	1	34.4333	0.881
0.8	0.1	AG1	1	34.4333	0.877

0.8	0.1	ptsI	1	34.6833	0.897
0.8	0.1	clcB	1	34.6833	0.413
0.8	0.1	ycaM	1	34.6833	0.434
0.8	0.1	yadI	1	34.6833	0.886
0.8	0.1	AG1	1	34.6833	0.878
0.8	0.1	ptsI	1	34.9333	0.898
0.8	0.1	clcB	1	34.9333	0.412
0.8	0.1	ycaM	1	34.9333	0.434
0.8	0.1	yadI	1	34.9333	0.884
0.8	0.1	AG1	1	34.9333	0.878
0.8	0.1	ptsI	1	35.1833	0.898
0.8	0.1	clcB	1	35.1833	0.411
0.8	0.1	ycaM	1	35.1833	0.434
0.8	0.1	yadI	1	35.1833	0.889
0.8	0.1	AG1	1	35.1833	0.878
0.8	0.1	ptsI	1	35.4333	0.898
0.8	0.1	clcB	1	35.4333	0.413
0.8	0.1	ycaM	1	35.4333	0.436
0.8	0.1	yadI	1	35.4333	0.895
0.8	0.1	AG1	1	35.4333	0.881
0.8	0.1	ptsI	1	35.6833	0.898
0.8	0.1	clcB	1	35.6833	0.416
0.8	0.1	ycaM	1	35.6833	0.437
0.8	0.1	yadI	1	35.6833	0.9
0.8	0.1	AG1	1	35.6833	0.882
0.8	0.1	ptsI	1	35.9333	0.893
0.8	0.1	clcB	1	35.9333	0.409
0.8	0.1	ycaM	1	35.9333	0.431
0.8	0.1	yadI	1	35.9333	0.889
0.8	0.1	AG1	1	35.9333	0.878
0.8	0.1	ptsI	1	36.1833	0.9
0.8	0.1	clcB	1	36.1833	0.421
0.8	0.1	ycaM	1	36.1833	0.44
0.8	0.1	yadI	1	36.1833	0.9
0.8	0.1	AG1	1	36.1833	0.881
0.8	0.1	ptsI	1	36.4333	0.896
0.8	0.1	clcB	1	36.4333	0.416
0.8	0.1	ycaM	1	36.4333	0.439
0.8	0.1	yadI	1	36.4333	0.909
0.8	0.1	AG1	1	36.4333	0.882
0.8	0.1	ptsI	1	36.6833	0.895
0.8	0.1	clcB	1	36.6833	0.419
0.8	0.1	ycaM	1	36.6833	0.44
0.8	0.1	yadI	1	36.6833	0.909
0.8	0.1	AG1	1	36.6833	0.883
1.6	0.1	ptsI	1	0	0.228
1.6	0.1	clcB	1	0	0.285
1.6	0.1	ycaM	1	0	0.258
1.6	0.1	yadI	1	0	0.289
1.6	0.1	AG1	1	0	0.236
1.6	0.1	ptsI	1	0.25	0.226
1.6	0.1	clcB	1	0.25	0.27
1.6	0.1	ycaM	1	0.25	0.243

1.6	0.1	yadI	1	0.25	0.271
1.6	0.1	AG1	1	0.25	0.228
1.6	0.1	ptsI	1	0.5	0.227
1.6	0.1	clcB	1	0.5	0.268
1.6	0.1	ycaM	1	0.5	0.236
1.6	0.1	yadI	1	0.5	0.271
1.6	0.1	AG1	1	0.5	0.227
1.6	0.1	ptsI	1	0.75	0.224
1.6	0.1	clcB	1	0.75	0.262
1.6	0.1	ycaM	1	0.75	0.233
1.6	0.1	yadI	1	0.75	0.27
1.6	0.1	AG1	1	0.75	0.228
1.6	0.1	ptsI	1	1	0.227
1.6	0.1	clcB	1	1	0.266
1.6	0.1	ycaM	1	1	0.232
1.6	0.1	yadI	1	1	0.268
1.6	0.1	AG1	1	1	0.23
1.6	0.1	ptsI	1	1.25	0.231
1.6	0.1	clcB	1	1.25	0.257
1.6	0.1	ycaM	1	1.25	0.228
1.6	0.1	yadI	1	1.25	0.267
1.6	0.1	AG1	1	1.25	0.231
1.6	0.1	ptsI	1	1.5	0.232
1.6	0.1	clcB	1	1.5	0.262
1.6	0.1	ycaM	1	1.5	0.231
1.6	0.1	yadI	1	1.5	0.271
1.6	0.1	AG1	1	1.5	0.228
1.6	0.1	ptsI	1	1.75	0.24
1.6	0.1	clcB	1	1.75	0.266
1.6	0.1	ycaM	1	1.75	0.232
1.6	0.1	yadI	1	1.75	0.273
1.6	0.1	AG1	1	1.75	0.233
1.6	0.1	ptsI	1	2	0.245
1.6	0.1	clcB	1	2	0.272
1.6	0.1	ycaM	1	2	0.238
1.6	0.1	yadI	1	2	0.278
1.6	0.1	AG1	1	2	0.234
1.6	0.1	ptsI	1	2.25	0.255
1.6	0.1	clcB	1	2.25	0.278
1.6	0.1	ycaM	1	2.25	0.238
1.6	0.1	yadI	1	2.25	0.28
1.6	0.1	AG1	1	2.25	0.241
1.6	0.1	ptsI	1	2.5	0.261
1.6	0.1	clcB	1	2.5	0.284
1.6	0.1	ycaM	1	2.5	0.248
1.6	0.1	yadI	1	2.5	0.291
1.6	0.1	AG1	1	2.5	0.241
1.6	0.1	ptsI	1	2.75	0.272
1.6	0.1	clcB	1	2.75	0.293
1.6	0.1	ycaM	1	2.75	0.247
1.6	0.1	yadI	1	2.75	0.293
1.6	0.1	AG1	1	2.75	0.248
1.6	0.1	ptsI	1	3	0.282

1.6	0.1	clcB	1	3	0.299
1.6	0.1	ycaM	1	3	0.258
1.6	0.1	yadI	1	3	0.304
1.6	0.1	AG1	1	3	0.251
1.6	0.1	ptsI	1	3.25	0.294
1.6	0.1	clcB	1	3.25	0.308
1.6	0.1	ycaM	1	3.25	0.266
1.6	0.1	yadI	1	3.25	0.316
1.6	0.1	AG1	1	3.25	0.257
1.6	0.1	ptsI	1	3.5	0.306
1.6	0.1	clcB	1	3.5	0.318
1.6	0.1	ycaM	1	3.5	0.268
1.6	0.1	yadI	1	3.5	0.32
1.6	0.1	AG1	1	3.5	0.264
1.6	0.1	ptsI	1	3.75	0.314
1.6	0.1	clcB	1	3.75	0.321
1.6	0.1	ycaM	1	3.75	0.265
1.6	0.1	yadI	1	3.75	0.328
1.6	0.1	AG1	1	3.75	0.264
1.6	0.1	ptsI	1	4	0.33
1.6	0.1	clcB	1	4	0.339
1.6	0.1	ycaM	1	4	0.28
1.6	0.1	yadI	1	4	0.343
1.6	0.1	AG1	1	4	0.273
1.6	0.1	ptsI	1	4.25	0.336
1.6	0.1	clcB	1	4.25	0.342
1.6	0.1	ycaM	1	4.25	0.278
1.6	0.1	yadI	1	4.25	0.343
1.6	0.1	AG1	1	4.25	0.274
1.6	0.1	ptsI	1	4.5	0.348
1.6	0.1	clcB	1	4.5	0.356
1.6	0.1	ycaM	1	4.5	0.286
1.6	0.1	yadI	1	4.5	0.354
1.6	0.1	AG1	1	4.5	0.279
1.6	0.1	ptsI	1	4.75	0.344
1.6	0.1	clcB	1	4.75	0.353
1.6	0.1	ycaM	1	4.75	0.282
1.6	0.1	yadI	1	4.75	0.356
1.6	0.1	AG1	1	4.75	0.281
1.6	0.1	ptsI	1	5	0.36
1.6	0.1	clcB	1	5	0.37
1.6	0.1	ycaM	1	5	0.294
1.6	0.1	yadI	1	5	0.368
1.6	0.1	AG1	1	5	0.287
1.6	0.1	ptsI	1	5.25	0.365
1.6	0.1	clcB	1	5.25	0.378
1.6	0.1	ycaM	1	5.25	0.293
1.6	0.1	yadI	1	5.25	0.372
1.6	0.1	AG1	1	5.25	0.288
1.6	0.1	ptsI	1	5.5	0.376
1.6	0.1	clcB	1	5.5	0.399
1.6	0.1	ycaM	1	5.5	0.306
1.6	0.1	yadI	1	5.5	0.387

1.6	0.1	AG1	1	5.5	0.295
1.6	0.1	ptsI	1	5.75	0.385
1.6	0.1	clcB	1	5.75	0.408
1.6	0.1	ycaM	1	5.75	0.308
1.6	0.1	yadI	1	5.75	0.394
1.6	0.1	AG1	1	5.75	0.304
1.6	0.1	ptsI	1	6	0.373
1.6	0.1	clcB	1	6	0.401
1.6	0.1	ycaM	1	6	0.304
1.6	0.1	yadI	1	6	0.383
1.6	0.1	AG1	1	6	0.309
1.6	0.1	ptsI	1	6.25	0.378
1.6	0.1	clcB	1	6.25	0.401
1.6	0.1	ycaM	1	6.25	0.307
1.6	0.1	yadI	1	6.25	0.386
1.6	0.1	AG1	1	6.25	0.304
1.6	0.1	ptsI	1	6.5	0.388
1.6	0.1	clcB	1	6.5	0.4
1.6	0.1	ycaM	1	6.5	0.308
1.6	0.1	yadI	1	6.5	0.388
1.6	0.1	AG1	1	6.5	0.311
1.6	0.1	ptsI	1	6.75	0.411
1.6	0.1	clcB	1	6.75	0.429
1.6	0.1	ycaM	1	6.75	0.322
1.6	0.1	yadI	1	6.75	0.4
1.6	0.1	AG1	1	6.75	0.316
1.6	0.1	ptsI	1	7	0.426
1.6	0.1	clcB	1	7	0.446
1.6	0.1	ycaM	1	7	0.328
1.6	0.1	yadI	1	7	0.406
1.6	0.1	AG1	1	7	0.327
1.6	0.1	ptsI	1	7.25	0.44
1.6	0.1	clcB	1	7.25	0.459
1.6	0.1	ycaM	1	7.25	0.339
1.6	0.1	yadI	1	7.25	0.416
1.6	0.1	AG1	1	7.25	0.33
1.6	0.1	ptsI	1	7.5	0.456
1.6	0.1	clcB	1	7.5	0.468
1.6	0.1	ycaM	1	7.5	0.344
1.6	0.1	yadI	1	7.5	0.421
1.6	0.1	AG1	1	7.5	0.345
1.6	0.1	ptsI	1	7.75	0.468
1.6	0.1	clcB	1	7.75	0.465
1.6	0.1	ycaM	1	7.75	0.35
1.6	0.1	yadI	1	7.75	0.428
1.6	0.1	AG1	1	7.75	0.363
1.6	0.1	ptsI	1	8	0.48
1.6	0.1	clcB	1	8	0.489
1.6	0.1	ycaM	1	8	0.355
1.6	0.1	yadI	1	8	0.438
1.6	0.1	AG1	1	8	0.359
1.6	0.1	ptsI	1	8.25	0.488
1.6	0.1	clcB	1	8.25	0.493

1.6	0.1	ycaM	1	8.25	0.352
1.6	0.1	yadI	1	8.25	0.442
1.6	0.1	AG1	1	8.25	0.378
1.6	0.1	ptsI	1	8.5	0.498
1.6	0.1	clcB	1	8.5	0.502
1.6	0.1	ycaM	1	8.5	0.357
1.6	0.1	yadI	1	8.5	0.448
1.6	0.1	AG1	1	8.5	0.381
1.6	0.1	ptsI	1	8.75	0.508
1.6	0.1	clcB	1	8.75	0.503
1.6	0.1	ycaM	1	8.75	0.36
1.6	0.1	yadI	1	8.75	0.456
1.6	0.1	AG1	1	8.75	0.393
1.6	0.1	ptsI	1	9	0.511
1.6	0.1	clcB	1	9	0.513
1.6	0.1	ycaM	1	9	0.369
1.6	0.1	yadI	1	9	0.46
1.6	0.1	AG1	1	9	0.389
1.6	0.1	ptsI	1	9.25	0.522
1.6	0.1	clcB	1	9.25	0.513
1.6	0.1	ycaM	1	9.25	0.371
1.6	0.1	yadI	1	9.25	0.457
1.6	0.1	AG1	1	9.25	0.409
1.6	0.1	ptsI	1	9.5	0.544
1.6	0.1	clcB	1	9.5	0.515
1.6	0.1	ycaM	1	9.5	0.373
1.6	0.1	yadI	1	9.5	0.456
1.6	0.1	AG1	1	9.5	0.418
1.6	0.1	ptsI	1	9.75	0.552
1.6	0.1	clcB	1	9.75	0.555
1.6	0.1	ycaM	1	9.75	0.388
1.6	0.1	yadI	1	9.75	0.474
1.6	0.1	AG1	1	9.75	0.436
1.6	0.1	ptsI	1	10	0.503
1.6	0.1	clcB	1	10	0.514
1.6	0.1	ycaM	1	10	0.382
1.6	0.1	yadI	1	10	0.452
1.6	0.1	AG1	1	10	0.408
1.6	0.1	ptsI	1	10.25	0.539
1.6	0.1	clcB	1	10.25	0.546
1.6	0.1	ycaM	1	10.25	0.388
1.6	0.1	yadI	1	10.25	0.461
1.6	0.1	AG1	1	10.25	0.418
1.6	0.1	ptsI	1	10.5	0.581
1.6	0.1	clcB	1	10.5	0.551
1.6	0.1	ycaM	1	10.5	0.402
1.6	0.1	yadI	1	10.5	0.474
1.6	0.1	AG1	1	10.5	0.44
1.6	0.1	ptsI	1	10.75	0.529
1.6	0.1	clcB	1	10.75	0.516
1.6	0.1	ycaM	1	10.75	0.397
1.6	0.1	yadI	1	10.75	0.453
1.6	0.1	AG1	1	10.75	0.426

1.6	0.1	ptsI	1	11	0.526
1.6	0.1	clcB	1	11	0.492
1.6	0.1	ycaM	1	11	0.398
1.6	0.1	yadI	1	11	0.454
1.6	0.1	AG1	1	11	0.442
1.6	0.1	ptsI	1	11.25	0.577
1.6	0.1	clcB	1	11.25	0.543
1.6	0.1	ycaM	1	11.25	0.423
1.6	0.1	yadI	1	11.25	0.465
1.6	0.1	AG1	1	11.25	0.48
1.6	0.1	ptsI	1	11.5	0.572
1.6	0.1	clcB	1	11.5	0.511
1.6	0.1	ycaM	1	11.5	0.42
1.6	0.1	yadI	1	11.5	0.465
1.6	0.1	AG1	1	11.5	0.47
1.6	0.1	ptsI	1	11.75	0.565
1.6	0.1	clcB	1	11.75	0.501
1.6	0.1	ycaM	1	11.75	0.414
1.6	0.1	yadI	1	11.75	0.451
1.6	0.1	AG1	1	11.75	0.465
1.6	0.1	ptsI	1	12	0.601
1.6	0.1	clcB	1	12	0.546
1.6	0.1	ycaM	1	12	0.443
1.6	0.1	yadI	1	12	0.46
1.6	0.1	AG1	1	12	0.516
1.6	0.1	ptsI	1	12.25	0.579
1.6	0.1	clcB	1	12.25	0.503
1.6	0.1	ycaM	1	12.25	0.426
1.6	0.1	yadI	1	12.25	0.454
1.6	0.1	AG1	1	12.25	0.492
1.6	0.1	ptsI	1	12.5	0.612
1.6	0.1	clcB	1	12.5	0.535
1.6	0.1	ycaM	1	12.5	0.438
1.6	0.1	yadI	1	12.5	0.453
1.6	0.1	AG1	1	12.5	0.541
1.6	0.1	ptsI	1	12.75	0.616
1.6	0.1	clcB	1	12.75	0.556
1.6	0.1	ycaM	1	12.75	0.474
1.6	0.1	yadI	1	12.75	0.463
1.6	0.1	AG1	1	12.75	0.553
1.6	0.1	ptsI	1	13	0.644
1.6	0.1	clcB	1	13	0.564
1.6	0.1	ycaM	1	13	0.506
1.6	0.1	yadI	1	13	0.474
1.6	0.1	AG1	1	13	0.614
1.6	0.1	ptsI	1	13.25	0.625
1.6	0.1	clcB	1	13.25	0.563
1.6	0.1	ycaM	1	13.25	0.478
1.6	0.1	yadI	1	13.25	0.466
1.6	0.1	AG1	1	13.25	0.594
1.6	0.1	ptsI	1	13.5	0.663
1.6	0.1	clcB	1	13.5	0.589
1.6	0.1	ycaM	1	13.5	0.507

1.6	0.1	yadI	1	13.5	0.483
1.6	0.1	AG1	1	13.5	0.685
1.6	0.1	ptsI	1	13.75	0.64
1.6	0.1	clcB	1	13.75	0.608
1.6	0.1	ycaM	1	13.75	0.487
1.6	0.1	yadI	1	13.75	0.484
1.6	0.1	AG1	1	13.75	0.666
1.6	0.1	ptsI	1	14	0.671
1.6	0.1	clcB	1	14	0.638
1.6	0.1	ycaM	1	14	0.522
1.6	0.1	yadI	1	14	0.486
1.6	0.1	AG1	1	14	0.744
1.6	0.1	ptsI	1	14.25	0.668
1.6	0.1	clcB	1	14.25	0.645
1.6	0.1	ycaM	1	14.25	0.545
1.6	0.1	yadI	1	14.25	0.494
1.6	0.1	AG1	1	14.25	0.771
1.6	0.1	ptsI	1	14.5	0.683
1.6	0.1	clcB	1	14.5	0.652
1.6	0.1	ycaM	1	14.5	0.561
1.6	0.1	yadI	1	14.5	0.504
1.6	0.1	AG1	1	14.5	0.79
1.6	0.1	ptsI	1	14.75	0.657
1.6	0.1	clcB	1	14.75	0.624
1.6	0.1	ycaM	1	14.75	0.537
1.6	0.1	yadI	1	14.75	0.495
1.6	0.1	AG1	1	14.75	0.758
1.6	0.1	ptsI	1	15	0.701
1.6	0.1	clcB	1	15	0.662
1.6	0.1	ycaM	1	15	0.567
1.6	0.1	yadI	1	15	0.514
1.6	0.1	AG1	1	15	0.823
1.6	0.1	ptsI	1	15.25	0.716
1.6	0.1	clcB	1	15.25	0.663
1.6	0.1	ycaM	1	15.25	0.583
1.6	0.1	yadI	1	15.25	0.522
1.6	0.1	AG1	1	15.25	0.831
1.6	0.1	ptsI	1	15.5	0.698
1.6	0.1	clcB	1	15.5	0.674
1.6	0.1	ycaM	1	15.5	0.581
1.6	0.1	yadI	1	15.5	0.52
1.6	0.1	AG1	1	15.5	0.822
1.6	0.1	ptsI	1	15.75	0.683
1.6	0.1	clcB	1	15.75	0.661
1.6	0.1	ycaM	1	15.75	0.551
1.6	0.1	yadI	1	15.75	0.506
1.6	0.1	AG1	1	15.75	0.799
1.6	0.1	ptsI	1	16	0.672
1.6	0.1	clcB	1	16	0.666
1.6	0.1	ycaM	1	16	0.538
1.6	0.1	yadI	1	16	0.498
1.6	0.1	AG1	1	16	0.755
1.6	0.1	ptsI	1	16.25	0.732

1.6	0.1	clcB	1	16.25	0.7
1.6	0.1	ycaM	1	16.25	0.581
1.6	0.1	yadI	1	16.25	0.512
1.6	0.1	AG1	1	16.25	0.819
1.6	0.1	ptsI	1	16.5	0.725
1.6	0.1	clcB	1	16.5	0.712
1.6	0.1	ycaM	1	16.5	0.598
1.6	0.1	yadI	1	16.5	0.52
1.6	0.1	AG1	1	16.5	0.834
1.6	0.1	ptsI	1	17.0667	0.746
1.6	0.1	clcB	1	17.0667	0.52
1.6	0.1	ycaM	1	17.0667	0.507
1.6	0.1	yadI	1	17.0667	0.673
1.6	0.1	AG1	1	17.0667	0.851
1.6	0.1	ptsI	1	17.3167	0.68
1.6	0.1	clcB	1	17.3167	0.493
1.6	0.1	ycaM	1	17.3167	0.468
1.6	0.1	yadI	1	17.3167	0.651
1.6	0.1	AG1	1	17.3167	0.817
1.6	0.1	ptsI	1	17.5667	0.669
1.6	0.1	clcB	1	17.5667	0.482
1.6	0.1	ycaM	1	17.5667	0.458
1.6	0.1	yadI	1	17.5667	0.652
1.6	0.1	AG1	1	17.5667	0.831
1.6	0.1	ptsI	1	17.8167	0.745
1.6	0.1	clcB	1	17.8167	0.484
1.6	0.1	ycaM	1	17.8167	0.46
1.6	0.1	yadI	1	17.8167	0.658
1.6	0.1	AG1	1	17.8167	0.849
1.6	0.1	ptsI	1	18.0667	0.67
1.6	0.1	clcB	1	18.0667	0.481
1.6	0.1	ycaM	1	18.0667	0.453
1.6	0.1	yadI	1	18.0667	0.678
1.6	0.1	AG1	1	18.0667	0.824
1.6	0.1	ptsI	1	18.3167	0.682
1.6	0.1	clcB	1	18.3167	0.48
1.6	0.1	ycaM	1	18.3167	0.444
1.6	0.1	yadI	1	18.3167	0.663
1.6	0.1	AG1	1	18.3167	0.798
1.6	0.1	ptsI	1	18.5667	0.757
1.6	0.1	clcB	1	18.5667	0.477
1.6	0.1	ycaM	1	18.5667	0.446
1.6	0.1	yadI	1	18.5667	0.677
1.6	0.1	AG1	1	18.5667	0.827
1.6	0.1	ptsI	1	18.8167	0.745
1.6	0.1	clcB	1	18.8167	0.472
1.6	0.1	ycaM	1	18.8167	0.448
1.6	0.1	yadI	1	18.8167	0.69
1.6	0.1	AG1	1	18.8167	0.832
1.6	0.1	ptsI	1	19.0667	0.751
1.6	0.1	clcB	1	19.0667	0.472
1.6	0.1	ycaM	1	19.0667	0.452
1.6	0.1	yadI	1	19.0667	0.703

1.6	0.1	AG1	1	19.0667	0.851
1.6	0.1	ptsI	1	19.3167	0.74
1.6	0.1	clcB	1	19.3167	0.469
1.6	0.1	ycaM	1	19.3167	0.447
1.6	0.1	yadI	1	19.3167	0.706
1.6	0.1	AG1	1	19.3167	0.844
1.6	0.1	ptsI	1	19.5667	0.757
1.6	0.1	clcB	1	19.5667	0.467
1.6	0.1	ycaM	1	19.5667	0.451
1.6	0.1	yadI	1	19.5667	0.714
1.6	0.1	AG1	1	19.5667	0.859
1.6	0.1	ptsI	1	19.8167	0.765
1.6	0.1	clcB	1	19.8167	0.466
1.6	0.1	ycaM	1	19.8167	0.451
1.6	0.1	yadI	1	19.8167	0.72
1.6	0.1	AG1	1	19.8167	0.861
1.6	0.1	ptsI	1	20.0667	0.761
1.6	0.1	clcB	1	20.0667	0.465
1.6	0.1	ycaM	1	20.0667	0.451
1.6	0.1	yadI	1	20.0667	0.728
1.6	0.1	AG1	1	20.0667	0.865
1.6	0.1	ptsI	1	20.3167	0.768
1.6	0.1	clcB	1	20.3167	0.463
1.6	0.1	ycaM	1	20.3167	0.452
1.6	0.1	yadI	1	20.3167	0.734
1.6	0.1	AG1	1	20.3167	0.872
1.6	0.1	ptsI	1	20.5667	0.761
1.6	0.1	clcB	1	20.5667	0.461
1.6	0.1	ycaM	1	20.5667	0.445
1.6	0.1	yadI	1	20.5667	0.732
1.6	0.1	AG1	1	20.5667	0.867
1.6	0.1	ptsI	1	20.8167	0.767
1.6	0.1	clcB	1	20.8167	0.458
1.6	0.1	ycaM	1	20.8167	0.449
1.6	0.1	yadI	1	20.8167	0.737
1.6	0.1	AG1	1	20.8167	0.868
1.6	0.1	ptsI	1	21.0667	0.774
1.6	0.1	clcB	1	21.0667	0.456
1.6	0.1	ycaM	1	21.0667	0.447
1.6	0.1	yadI	1	21.0667	0.743
1.6	0.1	AG1	1	21.0667	0.879
1.6	0.1	ptsI	1	21.3167	0.775
1.6	0.1	clcB	1	21.3167	0.463
1.6	0.1	ycaM	1	21.3167	0.451
1.6	0.1	yadI	1	21.3167	0.744
1.6	0.1	AG1	1	21.3167	0.882
1.6	0.1	ptsI	1	21.5667	0.772
1.6	0.1	clcB	1	21.5667	0.454
1.6	0.1	ycaM	1	21.5667	0.444
1.6	0.1	yadI	1	21.5667	0.738
1.6	0.1	AG1	1	21.5667	0.886
1.6	0.1	ptsI	1	21.8167	0.776
1.6	0.1	clcB	1	21.8167	0.452

1.6	0.1	ycaM	1	21.8167	0.448
1.6	0.1	yadI	1	21.8167	0.749
1.6	0.1	AG1	1	21.8167	0.886
1.6	0.1	ptsI	1	22.0667	0.785
1.6	0.1	clcB	1	22.0667	0.454
1.6	0.1	ycaM	1	22.0667	0.445
1.6	0.1	yadI	1	22.0667	0.749
1.6	0.1	AG1	1	22.0667	0.893
1.6	0.1	ptsI	1	22.9333	0.811
1.6	0.1	clcB	1	22.9333	0.47
1.6	0.1	ycaM	1	22.9333	0.398
1.6	0.1	yadI	1	22.9333	0.776
1.6	0.1	AG1	1	22.9333	0.872
1.6	0.1	ptsI	1	23.1833	0.772
1.6	0.1	clcB	1	23.1833	0.456
1.6	0.1	ycaM	1	23.1833	0.384
1.6	0.1	yadI	1	23.1833	0.759
1.6	0.1	AG1	1	23.1833	0.844
1.6	0.1	ptsI	1	23.4333	0.748
1.6	0.1	clcB	1	23.4333	0.449
1.6	0.1	ycaM	1	23.4333	0.378
1.6	0.1	yadI	1	23.4333	0.768
1.6	0.1	AG1	1	23.4333	0.833
1.6	0.1	ptsI	1	23.6833	0.786
1.6	0.1	clcB	1	23.6833	0.449
1.6	0.1	ycaM	1	23.6833	0.384
1.6	0.1	yadI	1	23.6833	0.779
1.6	0.1	AG1	1	23.6833	0.84
1.6	0.1	ptsI	1	23.9333	0.789
1.6	0.1	clcB	1	23.9333	0.449
1.6	0.1	ycaM	1	23.9333	0.381
1.6	0.1	yadI	1	23.9333	0.785
1.6	0.1	AG1	1	23.9333	0.843
1.6	0.1	ptsI	1	24.1833	0.808
1.6	0.1	clcB	1	24.1833	0.444
1.6	0.1	ycaM	1	24.1833	0.383
1.6	0.1	yadI	1	24.1833	0.79
1.6	0.1	AG1	1	24.1833	0.847
1.6	0.1	ptsI	1	24.4333	0.818
1.6	0.1	clcB	1	24.4333	0.44
1.6	0.1	ycaM	1	24.4333	0.384
1.6	0.1	yadI	1	24.4333	0.795
1.6	0.1	AG1	1	24.4333	0.853
1.6	0.1	ptsI	1	24.6833	0.816
1.6	0.1	clcB	1	24.6833	0.44
1.6	0.1	ycaM	1	24.6833	0.384
1.6	0.1	yadI	1	24.6833	0.798
1.6	0.1	AG1	1	24.6833	0.849
1.6	0.1	ptsI	1	24.9333	0.815
1.6	0.1	clcB	1	24.9333	0.435
1.6	0.1	ycaM	1	24.9333	0.383
1.6	0.1	yadI	1	24.9333	0.798
1.6	0.1	AG1	1	24.9333	0.846

1.6	0.1	ptsI	1	25.1833	0.815
1.6	0.1	clcB	1	25.1833	0.437
1.6	0.1	ycaM	1	25.1833	0.383
1.6	0.1	yadI	1	25.1833	0.805
1.6	0.1	AG1	1	25.1833	0.85
1.6	0.1	ptsI	1	25.4333	0.819
1.6	0.1	clcB	1	25.4333	0.441
1.6	0.1	ycaM	1	25.4333	0.386
1.6	0.1	yadI	1	25.4333	0.813
1.6	0.1	AG1	1	25.4333	0.859
1.6	0.1	ptsI	1	25.6833	0.822
1.6	0.1	clcB	1	25.6833	0.441
1.6	0.1	ycaM	1	25.6833	0.387
1.6	0.1	yadI	1	25.6833	0.812
1.6	0.1	AG1	1	25.6833	0.858
1.6	0.1	ptsI	1	25.9333	0.822
1.6	0.1	clcB	1	25.9333	0.441
1.6	0.1	ycaM	1	25.9333	0.386
1.6	0.1	yadI	1	25.9333	0.808
1.6	0.1	AG1	1	25.9333	0.863
1.6	0.1	ptsI	1	26.1833	0.839
1.6	0.1	clcB	1	26.1833	0.445
1.6	0.1	ycaM	1	26.1833	0.39
1.6	0.1	yadI	1	26.1833	0.816
1.6	0.1	AG1	1	26.1833	0.868
1.6	0.1	ptsI	1	26.4333	0.831
1.6	0.1	clcB	1	26.4333	0.446
1.6	0.1	ycaM	1	26.4333	0.39
1.6	0.1	yadI	1	26.4333	0.816
1.6	0.1	AG1	1	26.4333	0.863
1.6	0.1	ptsI	1	26.6833	0.836
1.6	0.1	clcB	1	26.6833	0.448
1.6	0.1	ycaM	1	26.6833	0.39
1.6	0.1	yadI	1	26.6833	0.818
1.6	0.1	AG1	1	26.6833	0.869
1.6	0.1	ptsI	1	26.9333	0.836
1.6	0.1	clcB	1	26.9333	0.448
1.6	0.1	ycaM	1	26.9333	0.391
1.6	0.1	yadI	1	26.9333	0.822
1.6	0.1	AG1	1	26.9333	0.87
1.6	0.1	ptsI	1	27.1833	0.844
1.6	0.1	clcB	1	27.1833	0.45
1.6	0.1	ycaM	1	27.1833	0.394
1.6	0.1	yadI	1	27.1833	0.823
1.6	0.1	AG1	1	27.1833	0.872
1.6	0.1	ptsI	1	27.4333	0.835
1.6	0.1	clcB	1	27.4333	0.451
1.6	0.1	ycaM	1	27.4333	0.392
1.6	0.1	yadI	1	27.4333	0.818
1.6	0.1	AG1	1	27.4333	0.87
1.6	0.1	ptsI	1	27.6833	0.842
1.6	0.1	clcB	1	27.6833	0.45
1.6	0.1	ycaM	1	27.6833	0.394

1.6	0.1	yadI	1	27.6833	0.82
1.6	0.1	AG1	1	27.6833	0.878
1.6	0.1	ptsI	1	27.9333	0.84
1.6	0.1	clcB	1	27.9333	0.448
1.6	0.1	ycaM	1	27.9333	0.389
1.6	0.1	yadI	1	27.9333	0.814
1.6	0.1	AG1	1	27.9333	0.868
1.6	0.1	ptsI	1	28.1833	0.846
1.6	0.1	clcB	1	28.1833	0.45
1.6	0.1	ycaM	1	28.1833	0.394
1.6	0.1	yadI	1	28.1833	0.824
1.6	0.1	AG1	1	28.1833	0.888
1.6	0.1	ptsI	1	28.4333	0.846
1.6	0.1	clcB	1	28.4333	0.454
1.6	0.1	ycaM	1	28.4333	0.396
1.6	0.1	yadI	1	28.4333	0.826
1.6	0.1	AG1	1	28.4333	0.887
1.6	0.1	ptsI	1	28.6833	0.846
1.6	0.1	clcB	1	28.6833	0.454
1.6	0.1	ycaM	1	28.6833	0.395
1.6	0.1	yadI	1	28.6833	0.822
1.6	0.1	AG1	1	28.6833	0.892
1.6	0.1	ptsI	1	28.9333	0.849
1.6	0.1	clcB	1	28.9333	0.457
1.6	0.1	ycaM	1	28.9333	0.393
1.6	0.1	yadI	1	28.9333	0.828
1.6	0.1	AG1	1	28.9333	0.893
1.6	0.1	ptsI	1	29.1833	0.847
1.6	0.1	clcB	1	29.1833	0.454
1.6	0.1	ycaM	1	29.1833	0.396
1.6	0.1	yadI	1	29.1833	0.827
1.6	0.1	AG1	1	29.1833	0.892
1.6	0.1	ptsI	1	29.4333	0.857
1.6	0.1	clcB	1	29.4333	0.458
1.6	0.1	ycaM	1	29.4333	0.399
1.6	0.1	yadI	1	29.4333	0.831
1.6	0.1	AG1	1	29.4333	0.903
1.6	0.1	ptsI	1	29.6833	0.853
1.6	0.1	clcB	1	29.6833	0.458
1.6	0.1	ycaM	1	29.6833	0.396
1.6	0.1	yadI	1	29.6833	0.833
1.6	0.1	AG1	1	29.6833	0.902
1.6	0.1	ptsI	1	29.9333	0.855
1.6	0.1	clcB	1	29.9333	0.459
1.6	0.1	ycaM	1	29.9333	0.397
1.6	0.1	yadI	1	29.9333	0.832
1.6	0.1	AG1	1	29.9333	0.907
1.6	0.1	ptsI	1	30.1833	0.863
1.6	0.1	clcB	1	30.1833	0.457
1.6	0.1	ycaM	1	30.1833	0.4
1.6	0.1	yadI	1	30.1833	0.83
1.6	0.1	AG1	1	30.1833	0.913
1.6	0.1	ptsI	1	30.4333	0.86

1.6	0.1	clcB	1	30.4333	0.461
1.6	0.1	ycaM	1	30.4333	0.401
1.6	0.1	yadI	1	30.4333	0.831
1.6	0.1	AG1	1	30.4333	0.914
1.6	0.1	ptsI	1	30.6833	0.863
1.6	0.1	clcB	1	30.6833	0.46
1.6	0.1	ycaM	1	30.6833	0.403
1.6	0.1	yadI	1	30.6833	0.838
1.6	0.1	AG1	1	30.6833	0.916
1.6	0.1	ptsI	1	30.9333	0.868
1.6	0.1	clcB	1	30.9333	0.462
1.6	0.1	ycaM	1	30.9333	0.402
1.6	0.1	yadI	1	30.9333	0.836
1.6	0.1	AG1	1	30.9333	0.928
1.6	0.1	ptsI	1	31.1833	0.871
1.6	0.1	clcB	1	31.1833	0.461
1.6	0.1	ycaM	1	31.1833	0.402
1.6	0.1	yadI	1	31.1833	0.842
1.6	0.1	AG1	1	31.1833	0.926
1.6	0.1	ptsI	1	31.4333	0.872
1.6	0.1	clcB	1	31.4333	0.462
1.6	0.1	ycaM	1	31.4333	0.403
1.6	0.1	yadI	1	31.4333	0.842
1.6	0.1	AG1	1	31.4333	0.934
1.6	0.1	ptsI	1	31.6833	0.874
1.6	0.1	clcB	1	31.6833	0.461
1.6	0.1	ycaM	1	31.6833	0.404
1.6	0.1	yadI	1	31.6833	0.844
1.6	0.1	AG1	1	31.6833	0.943
1.6	0.1	ptsI	1	31.9333	0.877
1.6	0.1	clcB	1	31.9333	0.465
1.6	0.1	ycaM	1	31.9333	0.405
1.6	0.1	yadI	1	31.9333	0.848
1.6	0.1	AG1	1	31.9333	0.944
1.6	0.1	ptsI	1	32.1833	0.879
1.6	0.1	clcB	1	32.1833	0.465
1.6	0.1	ycaM	1	32.1833	0.404
1.6	0.1	yadI	1	32.1833	0.84
1.6	0.1	AG1	1	32.1833	0.95
1.6	0.1	ptsI	1	32.4333	0.878
1.6	0.1	clcB	1	32.4333	0.461
1.6	0.1	ycaM	1	32.4333	0.403
1.6	0.1	yadI	1	32.4333	0.831
1.6	0.1	AG1	1	32.4333	0.946
1.6	0.1	ptsI	1	32.6833	0.883
1.6	0.1	clcB	1	32.6833	0.467
1.6	0.1	ycaM	1	32.6833	0.41
1.6	0.1	yadI	1	32.6833	0.845
1.6	0.1	AG1	1	32.6833	0.961
1.6	0.1	ptsI	1	32.9333	0.881
1.6	0.1	clcB	1	32.9333	0.466
1.6	0.1	ycaM	1	32.9333	0.408
1.6	0.1	yadI	1	32.9333	0.845

1.6	0.1	AG1	1	32.9333	0.969
1.6	0.1	ptsI	1	33.1833	0.888
1.6	0.1	clcB	1	33.1833	0.464
1.6	0.1	ycaM	1	33.1833	0.41
1.6	0.1	yadI	1	33.1833	0.847
1.6	0.1	AG1	1	33.1833	0.963
1.6	0.1	ptsI	1	33.4333	0.885
1.6	0.1	clcB	1	33.4333	0.466
1.6	0.1	ycaM	1	33.4333	0.413
1.6	0.1	yadI	1	33.4333	0.847
1.6	0.1	AG1	1	33.4333	0.981
1.6	0.1	ptsI	1	33.6833	0.887
1.6	0.1	clcB	1	33.6833	0.467
1.6	0.1	ycaM	1	33.6833	0.414
1.6	0.1	yadI	1	33.6833	0.847
1.6	0.1	AG1	1	33.6833	0.981
1.6	0.1	ptsI	1	33.9333	0.883
1.6	0.1	clcB	1	33.9333	0.469
1.6	0.1	ycaM	1	33.9333	0.414
1.6	0.1	yadI	1	33.9333	0.849
1.6	0.1	AG1	1	33.9333	0.992
1.6	0.1	ptsI	1	34.1833	0.886
1.6	0.1	clcB	1	34.1833	0.473
1.6	0.1	ycaM	1	34.1833	0.419
1.6	0.1	yadI	1	34.1833	0.855
1.6	0.1	AG1	1	34.1833	0.994
1.6	0.1	ptsI	1	34.4333	0.884
1.6	0.1	clcB	1	34.4333	0.47
1.6	0.1	ycaM	1	34.4333	0.42
1.6	0.1	yadI	1	34.4333	0.852
1.6	0.1	AG1	1	34.4333	1.002
1.6	0.1	ptsI	1	34.6833	0.887
1.6	0.1	clcB	1	34.6833	0.474
1.6	0.1	ycaM	1	34.6833	0.417
1.6	0.1	yadI	1	34.6833	0.851
1.6	0.1	AG1	1	34.6833	1.003
1.6	0.1	ptsI	1	34.9333	0.883
1.6	0.1	clcB	1	34.9333	0.474
1.6	0.1	ycaM	1	34.9333	0.419
1.6	0.1	yadI	1	34.9333	0.854
1.6	0.1	AG1	1	34.9333	1.008
1.6	0.1	ptsI	1	35.1833	0.885
1.6	0.1	clcB	1	35.1833	0.474
1.6	0.1	ycaM	1	35.1833	0.417
1.6	0.1	yadI	1	35.1833	0.858
1.6	0.1	AG1	1	35.1833	1.015
1.6	0.1	ptsI	1	35.4333	0.885
1.6	0.1	clcB	1	35.4333	0.474
1.6	0.1	ycaM	1	35.4333	0.42
1.6	0.1	yadI	1	35.4333	0.858
1.6	0.1	AG1	1	35.4333	1.019
1.6	0.1	ptsI	1	35.6833	0.886
1.6	0.1	clcB	1	35.6833	0.479

1.6	0.1	ycaM	1	35.6833	0.42
1.6	0.1	yadI	1	35.6833	0.854
1.6	0.1	AG1	1	35.6833	1.022
1.6	0.1	ptsI	1	35.9333	0.885
1.6	0.1	clcB	1	35.9333	0.474
1.6	0.1	ycaM	1	35.9333	0.418
1.6	0.1	yadI	1	35.9333	0.856
1.6	0.1	AG1	1	35.9333	1.018
1.6	0.1	ptsI	1	36.1833	0.884
1.6	0.1	clcB	1	36.1833	0.482
1.6	0.1	ycaM	1	36.1833	0.422
1.6	0.1	yadI	1	36.1833	0.858
1.6	0.1	AG1	1	36.1833	1.028
1.6	0.1	ptsI	1	36.4333	0.882
1.6	0.1	clcB	1	36.4333	0.479
1.6	0.1	ycaM	1	36.4333	0.424
1.6	0.1	yadI	1	36.4333	0.862
1.6	0.1	AG1	1	36.4333	1.034
1.6	0.1	ptsI	1	36.6833	0.883
1.6	0.1	clcB	1	36.6833	0.481
1.6	0.1	ycaM	1	36.6833	0.426
1.6	0.1	yadI	1	36.6833	0.863
1.6	0.1	AG1	1	36.6833	1.039
3.2	0.1	ptsI	1	0	0.252
3.2	0.1	clcB	1	0	0.55
3.2	0.1	ycaM	1	0	0.228
3.2	0.1	yadI	1	0	0.275
3.2	0.1	AG1	1	0	0.244
3.2	0.1	ptsI	1	0.25	0.24
3.2	0.1	clcB	1	0.25	0.508
3.2	0.1	ycaM	1	0.25	0.218
3.2	0.1	yadI	1	0.25	0.257
3.2	0.1	AG1	1	0.25	0.234
3.2	0.1	ptsI	1	0.5	0.238
3.2	0.1	clcB	1	0.5	0.494
3.2	0.1	ycaM	1	0.5	0.215
3.2	0.1	yadI	1	0.5	0.254
3.2	0.1	AG1	1	0.5	0.231
3.2	0.1	ptsI	1	0.75	0.236
3.2	0.1	clcB	1	0.75	0.492
3.2	0.1	ycaM	1	0.75	0.213
3.2	0.1	yadI	1	0.75	0.252
3.2	0.1	AG1	1	0.75	0.228
3.2	0.1	ptsI	1	1	0.239
3.2	0.1	clcB	1	1	0.497
3.2	0.1	ycaM	1	1	0.212
3.2	0.1	yadI	1	1	0.255
3.2	0.1	AG1	1	1	0.232
3.2	0.1	ptsI	1	1.25	0.244
3.2	0.1	clcB	1	1.25	0.499
3.2	0.1	ycaM	1	1.25	0.214
3.2	0.1	yadI	1	1.25	0.254
3.2	0.1	AG1	1	1.25	0.227

3.2	0.1	ptsI	1	1.5	0.245
3.2	0.1	clcB	1	1.5	0.502
3.2	0.1	ycaM	1	1.5	0.215
3.2	0.1	yadI	1	1.5	0.258
3.2	0.1	AG1	1	1.5	0.232
3.2	0.1	ptsI	1	1.75	0.252
3.2	0.1	clcB	1	1.75	0.51
3.2	0.1	ycaM	1	1.75	0.218
3.2	0.1	yadI	1	1.75	0.261
3.2	0.1	AG1	1	1.75	0.232
3.2	0.1	ptsI	1	2	0.257
3.2	0.1	clcB	1	2	0.522
3.2	0.1	ycaM	1	2	0.223
3.2	0.1	yadI	1	2	0.265
3.2	0.1	AG1	1	2	0.238
3.2	0.1	ptsI	1	2.25	0.267
3.2	0.1	clcB	1	2.25	0.528
3.2	0.1	ycaM	1	2.25	0.229
3.2	0.1	yadI	1	2.25	0.271
3.2	0.1	AG1	1	2.25	0.242
3.2	0.1	ptsI	1	2.5	0.272
3.2	0.1	clcB	1	2.5	0.517
3.2	0.1	ycaM	1	2.5	0.231
3.2	0.1	yadI	1	2.5	0.275
3.2	0.1	AG1	1	2.5	0.245
3.2	0.1	ptsI	1	2.75	0.278
3.2	0.1	clcB	1	2.75	0.526
3.2	0.1	ycaM	1	2.75	0.238
3.2	0.1	yadI	1	2.75	0.278
3.2	0.1	AG1	1	2.75	0.248
3.2	0.1	ptsI	1	3	0.286
3.2	0.1	clcB	1	3	0.531
3.2	0.1	ycaM	1	3	0.244
3.2	0.1	yadI	1	3	0.285
3.2	0.1	AG1	1	3	0.253
3.2	0.1	ptsI	1	3.25	0.296
3.2	0.1	clcB	1	3.25	0.546
3.2	0.1	ycaM	1	3.25	0.255
3.2	0.1	yadI	1	3.25	0.296
3.2	0.1	AG1	1	3.25	0.26
3.2	0.1	ptsI	1	3.5	0.304
3.2	0.1	clcB	1	3.5	0.561
3.2	0.1	ycaM	1	3.5	0.257
3.2	0.1	yadI	1	3.5	0.306
3.2	0.1	AG1	1	3.5	0.265
3.2	0.1	ptsI	1	3.75	0.309
3.2	0.1	clcB	1	3.75	0.57
3.2	0.1	ycaM	1	3.75	0.256
3.2	0.1	yadI	1	3.75	0.316
3.2	0.1	AG1	1	3.75	0.262
3.2	0.1	ptsI	1	4	0.328
3.2	0.1	clcB	1	4	0.58
3.2	0.1	ycaM	1	4	0.266

3.2	0.1	yadI	1	4	0.33
3.2	0.1	AG1	1	4	0.275
3.2	0.1	ptsI	1	4.25	0.326
3.2	0.1	clcB	1	4.25	0.624
3.2	0.1	ycaM	1	4.25	0.269
3.2	0.1	yadI	1	4.25	0.333
3.2	0.1	AG1	1	4.25	0.276
3.2	0.1	ptsI	1	4.5	0.341
3.2	0.1	clcB	1	4.5	0.64
3.2	0.1	ycaM	1	4.5	0.278
3.2	0.1	yadI	1	4.5	0.345
3.2	0.1	AG1	1	4.5	0.285
3.2	0.1	ptsI	1	4.75	0.338
3.2	0.1	clcB	1	4.75	0.655
3.2	0.1	ycaM	1	4.75	0.278
3.2	0.1	yadI	1	4.75	0.349
3.2	0.1	AG1	1	4.75	0.283
3.2	0.1	ptsI	1	5	0.351
3.2	0.1	clcB	1	5	0.686
3.2	0.1	ycaM	1	5	0.281
3.2	0.1	yadI	1	5	0.358
3.2	0.1	AG1	1	5	0.294
3.2	0.1	ptsI	1	5.25	0.356
3.2	0.1	clcB	1	5.25	0.715
3.2	0.1	ycaM	1	5.25	0.292
3.2	0.1	yadI	1	5.25	0.369
3.2	0.1	AG1	1	5.25	0.294
3.2	0.1	ptsI	1	5.5	0.37
3.2	0.1	clcB	1	5.5	0.738
3.2	0.1	ycaM	1	5.5	0.299
3.2	0.1	yadI	1	5.5	0.382
3.2	0.1	AG1	1	5.5	0.304
3.2	0.1	ptsI	1	5.75	0.386
3.2	0.1	clcB	1	5.75	0.774
3.2	0.1	ycaM	1	5.75	0.314
3.2	0.1	yadI	1	5.75	0.394
3.2	0.1	AG1	1	5.75	0.31
3.2	0.1	ptsI	1	6	0.375
3.2	0.1	clcB	1	6	0.807
3.2	0.1	ycaM	1	6	0.31
3.2	0.1	yadI	1	6	0.396
3.2	0.1	AG1	1	6	0.306
3.2	0.1	ptsI	1	6.25	0.379
3.2	0.1	clcB	1	6.25	0.844
3.2	0.1	ycaM	1	6.25	0.306
3.2	0.1	yadI	1	6.25	0.394
3.2	0.1	AG1	1	6.25	0.306
3.2	0.1	ptsI	1	6.5	0.382
3.2	0.1	clcB	1	6.5	0.868
3.2	0.1	ycaM	1	6.5	0.312
3.2	0.1	yadI	1	6.5	0.397
3.2	0.1	AG1	1	6.5	0.312
3.2	0.1	ptsI	1	6.75	0.406

3.2	0.1	clcB	1	6.75	0.861
3.2	0.1	ycaM	1	6.75	0.323
3.2	0.1	yadI	1	6.75	0.409
3.2	0.1	AG1	1	6.75	0.322
3.2	0.1	ptsI	1	7	0.428
3.2	0.1	clcB	1	7	0.811
3.2	0.1	ycaM	1	7	0.337
3.2	0.1	yadI	1	7	0.419
3.2	0.1	AG1	1	7	0.327
3.2	0.1	ptsI	1	7.25	0.441
3.2	0.1	clcB	1	7.25	0.734
3.2	0.1	ycaM	1	7.25	0.348
3.2	0.1	yadI	1	7.25	0.431
3.2	0.1	AG1	1	7.25	0.334
3.2	0.1	ptsI	1	7.5	0.46
3.2	0.1	clcB	1	7.5	0.617
3.2	0.1	ycaM	1	7.5	0.366
3.2	0.1	yadI	1	7.5	0.449
3.2	0.1	AG1	1	7.5	0.344
3.2	0.1	ptsI	1	7.75	0.473
3.2	0.1	clcB	1	7.75	0.498
3.2	0.1	ycaM	1	7.75	0.374
3.2	0.1	yadI	1	7.75	0.458
3.2	0.1	AG1	1	7.75	0.352
3.2	0.1	ptsI	1	8	0.492
3.2	0.1	clcB	1	8	0.388
3.2	0.1	ycaM	1	8	0.377
3.2	0.1	yadI	1	8	0.464
3.2	0.1	AG1	1	8	0.36
3.2	0.1	ptsI	1	8.25	0.507
3.2	0.1	clcB	1	8.25	0.388
3.2	0.1	ycaM	1	8.25	0.388
3.2	0.1	yadI	1	8.25	0.479
3.2	0.1	AG1	1	8.25	0.368
3.2	0.1	ptsI	1	8.5	0.523
3.2	0.1	clcB	1	8.5	0.405
3.2	0.1	ycaM	1	8.5	0.398
3.2	0.1	yadI	1	8.5	0.497
3.2	0.1	AG1	1	8.5	0.374
3.2	0.1	ptsI	1	8.75	0.534
3.2	0.1	clcB	1	8.75	0.421
3.2	0.1	ycaM	1	8.75	0.396
3.2	0.1	yadI	1	8.75	0.504
3.2	0.1	AG1	1	8.75	0.38
3.2	0.1	ptsI	1	9	0.541
3.2	0.1	clcB	1	9	0.435
3.2	0.1	ycaM	1	9	0.407
3.2	0.1	yadI	1	9	0.513
3.2	0.1	AG1	1	9	0.388
3.2	0.1	ptsI	1	9.25	0.535
3.2	0.1	clcB	1	9.25	0.454
3.2	0.1	ycaM	1	9.25	0.401
3.2	0.1	yadI	1	9.25	0.519

3.2	0.1	AG1	1	9.25	0.4
3.2	0.1	ptsI	1	9.5	0.535
3.2	0.1	clcB	1	9.5	0.446
3.2	0.1	ycaM	1	9.5	0.407
3.2	0.1	yadI	1	9.5	0.51
3.2	0.1	AG1	1	9.5	0.403
3.2	0.1	ptsI	1	9.75	0.586
3.2	0.1	clcB	1	9.75	0.499
3.2	0.1	ycaM	1	9.75	0.434
3.2	0.1	yadI	1	9.75	0.542
3.2	0.1	AG1	1	9.75	0.426
3.2	0.1	ptsI	1	10	0.495
3.2	0.1	clcB	1	10	0.456
3.2	0.1	ycaM	1	10	0.422
3.2	0.1	yadI	1	10	0.507
3.2	0.1	AG1	1	10	0.423
3.2	0.1	ptsI	1	10.25	0.554
3.2	0.1	clcB	1	10.25	0.497
3.2	0.1	ycaM	1	10.25	0.437
3.2	0.1	yadI	1	10.25	0.522
3.2	0.1	AG1	1	10.25	0.44
3.2	0.1	ptsI	1	10.5	0.581
3.2	0.1	clcB	1	10.5	0.52
3.2	0.1	ycaM	1	10.5	0.458
3.2	0.1	yadI	1	10.5	0.538
3.2	0.1	AG1	1	10.5	0.457
3.2	0.1	ptsI	1	10.75	0.507
3.2	0.1	clcB	1	10.75	0.475
3.2	0.1	ycaM	1	10.75	0.447
3.2	0.1	yadI	1	10.75	0.516
3.2	0.1	AG1	1	10.75	0.448
3.2	0.1	ptsI	1	11	0.528
3.2	0.1	clcB	1	11	0.454
3.2	0.1	ycaM	1	11	0.452
3.2	0.1	yadI	1	11	0.523
3.2	0.1	AG1	1	11	0.448
3.2	0.1	ptsI	1	11.25	0.61
3.2	0.1	clcB	1	11.25	0.528
3.2	0.1	ycaM	1	11.25	0.488
3.2	0.1	yadI	1	11.25	0.555
3.2	0.1	AG1	1	11.25	0.505
3.2	0.1	ptsI	1	11.5	0.586
3.2	0.1	clcB	1	11.5	0.497
3.2	0.1	ycaM	1	11.5	0.494
3.2	0.1	yadI	1	11.5	0.528
3.2	0.1	AG1	1	11.5	0.489
3.2	0.1	ptsI	1	11.75	0.572
3.2	0.1	clcB	1	11.75	0.498
3.2	0.1	ycaM	1	11.75	0.491
3.2	0.1	yadI	1	11.75	0.528
3.2	0.1	AG1	1	11.75	0.488
3.2	0.1	ptsI	1	12	0.674
3.2	0.1	clcB	1	12	0.58

3.2	0.1	ycaM	1	12	0.533
3.2	0.1	yadI	1	12	0.539
3.2	0.1	AG1	1	12	0.558
3.2	0.1	ptsI	1	12.25	0.606
3.2	0.1	clcB	1	12.25	0.519
3.2	0.1	ycaM	1	12.25	0.512
3.2	0.1	yadI	1	12.25	0.527
3.2	0.1	AG1	1	12.25	0.516
3.2	0.1	ptsI	1	12.5	0.661
3.2	0.1	clcB	1	12.5	0.555
3.2	0.1	ycaM	1	12.5	0.537
3.2	0.1	yadI	1	12.5	0.53
3.2	0.1	AG1	1	12.5	0.551
3.2	0.1	ptsI	1	12.75	0.697
3.2	0.1	clcB	1	12.75	0.623
3.2	0.1	ycaM	1	12.75	0.574
3.2	0.1	yadI	1	12.75	0.558
3.2	0.1	AG1	1	12.75	0.596
3.2	0.1	ptsI	1	13	0.715
3.2	0.1	clcB	1	13	0.64
3.2	0.1	ycaM	1	13	0.595
3.2	0.1	yadI	1	13	0.586
3.2	0.1	AG1	1	13	0.658
3.2	0.1	ptsI	1	13.25	0.664
3.2	0.1	clcB	1	13.25	0.625
3.2	0.1	ycaM	1	13.25	0.575
3.2	0.1	yadI	1	13.25	0.557
3.2	0.1	AG1	1	13.25	0.644
3.2	0.1	ptsI	1	13.5	0.737
3.2	0.1	clcB	1	13.5	0.677
3.2	0.1	ycaM	1	13.5	0.624
3.2	0.1	yadI	1	13.5	0.591
3.2	0.1	AG1	1	13.5	0.702
3.2	0.1	ptsI	1	13.75	0.687
3.2	0.1	clcB	1	13.75	0.653
3.2	0.1	ycaM	1	13.75	0.603
3.2	0.1	yadI	1	13.75	0.577
3.2	0.1	AG1	1	13.75	0.688
3.2	0.1	ptsI	1	14	0.772
3.2	0.1	clcB	1	14	0.691
3.2	0.1	ycaM	1	14	0.635
3.2	0.1	yadI	1	14	0.602
3.2	0.1	AG1	1	14	0.747
3.2	0.1	ptsI	1	14.25	0.782
3.2	0.1	clcB	1	14.25	0.705
3.2	0.1	ycaM	1	14.25	0.666
3.2	0.1	yadI	1	14.25	0.612
3.2	0.1	AG1	1	14.25	0.764
3.2	0.1	ptsI	1	14.5	0.776
3.2	0.1	clcB	1	14.5	0.705
3.2	0.1	ycaM	1	14.5	0.68
3.2	0.1	yadI	1	14.5	0.622
3.2	0.1	AG1	1	14.5	0.763

3.2	0.1	ptsI	1	14.75	0.732
3.2	0.1	clcB	1	14.75	0.658
3.2	0.1	ycaM	1	14.75	0.654
3.2	0.1	yadI	1	14.75	0.602
3.2	0.1	AG1	1	14.75	0.757
3.2	0.1	ptsI	1	15	0.785
3.2	0.1	clcB	1	15	0.703
3.2	0.1	ycaM	1	15	0.683
3.2	0.1	yadI	1	15	0.631
3.2	0.1	AG1	1	15	0.793
3.2	0.1	ptsI	1	15.25	0.795
3.2	0.1	clcB	1	15.25	0.718
3.2	0.1	ycaM	1	15.25	0.702
3.2	0.1	yadI	1	15.25	0.646
3.2	0.1	AG1	1	15.25	0.806
3.2	0.1	ptsI	1	15.5	0.786
3.2	0.1	clcB	1	15.5	0.711
3.2	0.1	ycaM	1	15.5	0.686
3.2	0.1	yadI	1	15.5	0.634
3.2	0.1	AG1	1	15.5	0.783
3.2	0.1	ptsI	1	15.75	0.761
3.2	0.1	clcB	1	15.75	0.677
3.2	0.1	ycaM	1	15.75	0.677
3.2	0.1	yadI	1	15.75	0.621
3.2	0.1	AG1	1	15.75	0.799
3.2	0.1	ptsI	1	16	0.729
3.2	0.1	clcB	1	16	0.664
3.2	0.1	ycaM	1	16	0.66
3.2	0.1	yadI	1	16	0.616
3.2	0.1	AG1	1	16	0.78
3.2	0.1	ptsI	1	16.25	0.779
3.2	0.1	clcB	1	16.25	0.699
3.2	0.1	ycaM	1	16.25	0.692
3.2	0.1	yadI	1	16.25	0.642
3.2	0.1	AG1	1	16.25	0.814
3.2	0.1	ptsI	1	16.5	0.793
3.2	0.1	clcB	1	16.5	0.708
3.2	0.1	ycaM	1	16.5	0.702
3.2	0.1	yadI	1	16.5	0.662
3.2	0.1	AG1	1	16.5	0.827
3.2	0.1	ptsI	1	17.0667	0.646
3.2	0.1	clcB	1	17.0667	0.596
3.2	0.1	ycaM	1	17.0667	0.6
3.2	0.1	yadI	1	17.0667	0.663
3.2	0.1	AG1	1	17.0667	0.837
3.2	0.1	ptsI	1	17.3167	0.641
3.2	0.1	clcB	1	17.3167	0.576
3.2	0.1	ycaM	1	17.3167	0.551
3.2	0.1	yadI	1	17.3167	0.645
3.2	0.1	AG1	1	17.3167	0.79
3.2	0.1	ptsI	1	17.5667	0.642
3.2	0.1	clcB	1	17.5667	0.564
3.2	0.1	ycaM	1	17.5667	0.539

3.2	0.1	yadI	1	17.5667	0.656
3.2	0.1	AG1	1	17.5667	0.779
3.2	0.1	ptsI	1	17.8167	0.649
3.2	0.1	clcB	1	17.8167	0.552
3.2	0.1	ycaM	1	17.8167	0.537
3.2	0.1	yadI	1	17.8167	0.676
3.2	0.1	AG1	1	17.8167	0.797
3.2	0.1	ptsI	1	18.0667	0.654
3.2	0.1	clcB	1	18.0667	0.557
3.2	0.1	ycaM	1	18.0667	0.529
3.2	0.1	yadI	1	18.0667	0.7
3.2	0.1	AG1	1	18.0667	0.772
3.2	0.1	ptsI	1	18.3167	0.65
3.2	0.1	clcB	1	18.3167	0.537
3.2	0.1	ycaM	1	18.3167	0.521
3.2	0.1	yadI	1	18.3167	0.672
3.2	0.1	AG1	1	18.3167	0.772
3.2	0.1	ptsI	1	18.5667	0.671
3.2	0.1	clcB	1	18.5667	0.546
3.2	0.1	ycaM	1	18.5667	0.519
3.2	0.1	yadI	1	18.5667	0.703
3.2	0.1	AG1	1	18.5667	0.776
3.2	0.1	ptsI	1	18.8167	0.678
3.2	0.1	clcB	1	18.8167	0.539
3.2	0.1	ycaM	1	18.8167	0.519
3.2	0.1	yadI	1	18.8167	0.725
3.2	0.1	AG1	1	18.8167	0.788
3.2	0.1	ptsI	1	19.0667	0.702
3.2	0.1	clcB	1	19.0667	0.536
3.2	0.1	ycaM	1	19.0667	0.519
3.2	0.1	yadI	1	19.0667	0.742
3.2	0.1	AG1	1	19.0667	0.809
3.2	0.1	ptsI	1	19.3167	0.689
3.2	0.1	clcB	1	19.3167	0.537
3.2	0.1	ycaM	1	19.3167	0.511
3.2	0.1	yadI	1	19.3167	0.747
3.2	0.1	AG1	1	19.3167	0.797
3.2	0.1	ptsI	1	19.5667	0.72
3.2	0.1	clcB	1	19.5667	0.539
3.2	0.1	ycaM	1	19.5667	0.511
3.2	0.1	yadI	1	19.5667	0.758
3.2	0.1	AG1	1	19.5667	0.81
3.2	0.1	ptsI	1	19.8167	0.73
3.2	0.1	clcB	1	19.8167	0.533
3.2	0.1	ycaM	1	19.8167	0.509
3.2	0.1	yadI	1	19.8167	0.767
3.2	0.1	AG1	1	19.8167	0.813
3.2	0.1	ptsI	1	20.0667	0.743
3.2	0.1	clcB	1	20.0667	0.534
3.2	0.1	ycaM	1	20.0667	0.512
3.2	0.1	yadI	1	20.0667	0.773
3.2	0.1	AG1	1	20.0667	0.821
3.2	0.1	ptsI	1	20.3167	0.756

3.2	0.1	clcB	1	20.3167	0.532
3.2	0.1	ycaM	1	20.3167	0.512
3.2	0.1	yadI	1	20.3167	0.785
3.2	0.1	AG1	1	20.3167	0.834
3.2	0.1	ptsI	1	20.5667	0.738
3.2	0.1	clcB	1	20.5667	0.531
3.2	0.1	ycaM	1	20.5667	0.515
3.2	0.1	yadI	1	20.5667	0.77
3.2	0.1	AG1	1	20.5667	0.823
3.2	0.1	ptsI	1	20.8167	0.755
3.2	0.1	clcB	1	20.8167	0.534
3.2	0.1	ycaM	1	20.8167	0.512
3.2	0.1	yadI	1	20.8167	0.785
3.2	0.1	AG1	1	20.8167	0.825
3.2	0.1	ptsI	1	21.0667	0.772
3.2	0.1	clcB	1	21.0667	0.533
3.2	0.1	ycaM	1	21.0667	0.513
3.2	0.1	yadI	1	21.0667	0.797
3.2	0.1	AG1	1	21.0667	0.842
3.2	0.1	ptsI	1	21.3167	0.783
3.2	0.1	clcB	1	21.3167	0.536
3.2	0.1	ycaM	1	21.3167	0.514
3.2	0.1	yadI	1	21.3167	0.799
3.2	0.1	AG1	1	21.3167	0.843
3.2	0.1	ptsI	1	21.5667	0.776
3.2	0.1	clcB	1	21.5667	0.535
3.2	0.1	ycaM	1	21.5667	0.514
3.2	0.1	yadI	1	21.5667	0.792
3.2	0.1	AG1	1	21.5667	0.845
3.2	0.1	ptsI	1	21.8167	0.792
3.2	0.1	clcB	1	21.8167	0.536
3.2	0.1	ycaM	1	21.8167	0.516
3.2	0.1	yadI	1	21.8167	0.814
3.2	0.1	AG1	1	21.8167	0.849
3.2	0.1	ptsI	1	22.0667	0.797
3.2	0.1	clcB	1	22.0667	0.536
3.2	0.1	ycaM	1	22.0667	0.514
3.2	0.1	yadI	1	22.0667	0.82
3.2	0.1	AG1	1	22.0667	0.863
3.2	0.1	ptsI	1	22.9333	0.898
3.2	0.1	clcB	1	22.9333	0.472
3.2	0.1	ycaM	1	22.9333	0.523
3.2	0.1	yadI	1	22.9333	0.83
3.2	0.1	AG1	1	22.9333	0.985
3.2	0.1	ptsI	1	23.1833	0.848
3.2	0.1	clcB	1	23.1833	0.465
3.2	0.1	ycaM	1	23.1833	0.513
3.2	0.1	yadI	1	23.1833	0.816
3.2	0.1	AG1	1	23.1833	0.946
3.2	0.1	ptsI	1	23.4333	0.81
3.2	0.1	clcB	1	23.4333	0.462
3.2	0.1	ycaM	1	23.4333	0.518
3.2	0.1	yadI	1	23.4333	0.826

3.2	0.1	AG1	1	23.4333	0.932
3.2	0.1	ptsI	1	23.6833	0.822
3.2	0.1	clcB	1	23.6833	0.44
3.2	0.1	ycaM	1	23.6833	0.517
3.2	0.1	yadI	1	23.6833	0.842
3.2	0.1	AG1	1	23.6833	0.94
3.2	0.1	ptsI	1	23.9333	0.824
3.2	0.1	clcB	1	23.9333	0.446
3.2	0.1	ycaM	1	23.9333	0.516
3.2	0.1	yadI	1	23.9333	0.854
3.2	0.1	AG1	1	23.9333	0.943
3.2	0.1	ptsI	1	24.1833	0.821
3.2	0.1	clcB	1	24.1833	0.446
3.2	0.1	ycaM	1	24.1833	0.516
3.2	0.1	yadI	1	24.1833	0.862
3.2	0.1	AG1	1	24.1833	0.946
3.2	0.1	ptsI	1	24.4333	0.825
3.2	0.1	clcB	1	24.4333	0.449
3.2	0.1	ycaM	1	24.4333	0.514
3.2	0.1	yadI	1	24.4333	0.859
3.2	0.1	AG1	1	24.4333	0.946
3.2	0.1	ptsI	1	24.6833	0.826
3.2	0.1	clcB	1	24.6833	0.448
3.2	0.1	ycaM	1	24.6833	0.514
3.2	0.1	yadI	1	24.6833	0.858
3.2	0.1	AG1	1	24.6833	0.946
3.2	0.1	ptsI	1	24.9333	0.828
3.2	0.1	clcB	1	24.9333	0.442
3.2	0.1	ycaM	1	24.9333	0.515
3.2	0.1	yadI	1	24.9333	0.861
3.2	0.1	AG1	1	24.9333	0.945
3.2	0.1	ptsI	1	25.1833	0.838
3.2	0.1	clcB	1	25.1833	0.443
3.2	0.1	ycaM	1	25.1833	0.516
3.2	0.1	yadI	1	25.1833	0.865
3.2	0.1	AG1	1	25.1833	0.952
3.2	0.1	ptsI	1	25.4333	0.849
3.2	0.1	clcB	1	25.4333	0.446
3.2	0.1	ycaM	1	25.4333	0.514
3.2	0.1	yadI	1	25.4333	0.862
3.2	0.1	AG1	1	25.4333	0.954
3.2	0.1	ptsI	1	25.6833	0.847
3.2	0.1	clcB	1	25.6833	0.447
3.2	0.1	ycaM	1	25.6833	0.513
3.2	0.1	yadI	1	25.6833	0.864
3.2	0.1	AG1	1	25.6833	0.958
3.2	0.1	ptsI	1	25.9333	0.85
3.2	0.1	clcB	1	25.9333	0.449
3.2	0.1	ycaM	1	25.9333	0.505
3.2	0.1	yadI	1	25.9333	0.854
3.2	0.1	AG1	1	25.9333	0.956
3.2	0.1	ptsI	1	26.1833	0.855
3.2	0.1	clcB	1	26.1833	0.449

3.2	0.1	ycaM	1	26.1833	0.512
3.2	0.1	yadI	1	26.1833	0.864
3.2	0.1	AG1	1	26.1833	0.967
3.2	0.1	ptsI	1	26.4333	0.856
3.2	0.1	clcB	1	26.4333	0.45
3.2	0.1	ycaM	1	26.4333	0.51
3.2	0.1	yadI	1	26.4333	0.861
3.2	0.1	AG1	1	26.4333	0.962
3.2	0.1	ptsI	1	26.6833	0.858
3.2	0.1	clcB	1	26.6833	0.45
3.2	0.1	ycaM	1	26.6833	0.509
3.2	0.1	yadI	1	26.6833	0.862
3.2	0.1	AG1	1	26.6833	0.96
3.2	0.1	ptsI	1	26.9333	0.853
3.2	0.1	clcB	1	26.9333	0.448
3.2	0.1	ycaM	1	26.9333	0.509
3.2	0.1	yadI	1	26.9333	0.86
3.2	0.1	AG1	1	26.9333	0.961
3.2	0.1	ptsI	1	27.1833	0.862
3.2	0.1	clcB	1	27.1833	0.453
3.2	0.1	ycaM	1	27.1833	0.508
3.2	0.1	yadI	1	27.1833	0.863
3.2	0.1	AG1	1	27.1833	0.956
3.2	0.1	ptsI	1	27.4333	0.868
3.2	0.1	clcB	1	27.4333	0.451
3.2	0.1	ycaM	1	27.4333	0.507
3.2	0.1	yadI	1	27.4333	0.867
3.2	0.1	AG1	1	27.4333	0.954
3.2	0.1	ptsI	1	27.6833	0.868
3.2	0.1	clcB	1	27.6833	0.452
3.2	0.1	ycaM	1	27.6833	0.504
3.2	0.1	yadI	1	27.6833	0.877
3.2	0.1	AG1	1	27.6833	0.958
3.2	0.1	ptsI	1	27.9333	0.864
3.2	0.1	clcB	1	27.9333	0.448
3.2	0.1	ycaM	1	27.9333	0.506
3.2	0.1	yadI	1	27.9333	0.88
3.2	0.1	AG1	1	27.9333	0.959
3.2	0.1	ptsI	1	28.1833	0.875
3.2	0.1	clcB	1	28.1833	0.451
3.2	0.1	ycaM	1	28.1833	0.507
3.2	0.1	yadI	1	28.1833	0.881
3.2	0.1	AG1	1	28.1833	0.963
3.2	0.1	ptsI	1	28.4333	0.88
3.2	0.1	clcB	1	28.4333	0.454
3.2	0.1	ycaM	1	28.4333	0.507
3.2	0.1	yadI	1	28.4333	0.886
3.2	0.1	AG1	1	28.4333	0.964
3.2	0.1	ptsI	1	28.6833	0.885
3.2	0.1	clcB	1	28.6833	0.453
3.2	0.1	ycaM	1	28.6833	0.506
3.2	0.1	yadI	1	28.6833	0.893
3.2	0.1	AG1	1	28.6833	0.968

3.2	0.1	ptsI	1	28.9333	0.884
3.2	0.1	clcB	1	28.9333	0.45
3.2	0.1	ycaM	1	28.9333	0.51
3.2	0.1	yadI	1	28.9333	0.891
3.2	0.1	AG1	1	28.9333	0.965
3.2	0.1	ptsI	1	29.1833	0.883
3.2	0.1	clcB	1	29.1833	0.45
3.2	0.1	ycaM	1	29.1833	0.508
3.2	0.1	yadI	1	29.1833	0.898
3.2	0.1	AG1	1	29.1833	0.969
3.2	0.1	ptsI	1	29.4333	0.891
3.2	0.1	clcB	1	29.4333	0.452
3.2	0.1	ycaM	1	29.4333	0.509
3.2	0.1	yadI	1	29.4333	0.904
3.2	0.1	AG1	1	29.4333	0.974
3.2	0.1	ptsI	1	29.6833	0.892
3.2	0.1	clcB	1	29.6833	0.45
3.2	0.1	ycaM	1	29.6833	0.505
3.2	0.1	yadI	1	29.6833	0.907
3.2	0.1	AG1	1	29.6833	0.976
3.2	0.1	ptsI	1	29.9333	0.891
3.2	0.1	clcB	1	29.9333	0.449
3.2	0.1	ycaM	1	29.9333	0.511
3.2	0.1	yadI	1	29.9333	0.906
3.2	0.1	AG1	1	29.9333	0.977
3.2	0.1	ptsI	1	30.1833	0.897
3.2	0.1	clcB	1	30.1833	0.448
3.2	0.1	ycaM	1	30.1833	0.508
3.2	0.1	yadI	1	30.1833	0.909
3.2	0.1	AG1	1	30.1833	0.981
3.2	0.1	ptsI	1	30.4333	0.897
3.2	0.1	clcB	1	30.4333	0.446
3.2	0.1	ycaM	1	30.4333	0.506
3.2	0.1	yadI	1	30.4333	0.914
3.2	0.1	AG1	1	30.4333	0.982
3.2	0.1	ptsI	1	30.6833	0.902
3.2	0.1	clcB	1	30.6833	0.443
3.2	0.1	ycaM	1	30.6833	0.508
3.2	0.1	yadI	1	30.6833	0.909
3.2	0.1	AG1	1	30.6833	0.99
3.2	0.1	ptsI	1	30.9333	0.896
3.2	0.1	clcB	1	30.9333	0.442
3.2	0.1	ycaM	1	30.9333	0.506
3.2	0.1	yadI	1	30.9333	0.915
3.2	0.1	AG1	1	30.9333	0.997
3.2	0.1	ptsI	1	31.1833	0.896
3.2	0.1	clcB	1	31.1833	0.44
3.2	0.1	ycaM	1	31.1833	0.505
3.2	0.1	yadI	1	31.1833	0.912
3.2	0.1	AG1	1	31.1833	1.002
3.2	0.1	ptsI	1	31.4333	0.89
3.2	0.1	clcB	1	31.4333	0.439
3.2	0.1	ycaM	1	31.4333	0.506

3.2	0.1	yadI	1	31.4333	0.921
3.2	0.1	AG1	1	31.4333	1.004
3.2	0.1	ptsI	1	31.6833	0.891
3.2	0.1	clcB	1	31.6833	0.441
3.2	0.1	ycaM	1	31.6833	0.507
3.2	0.1	yadI	1	31.6833	0.925
3.2	0.1	AG1	1	31.6833	1.012
3.2	0.1	ptsI	1	31.9333	0.888
3.2	0.1	clcB	1	31.9333	0.438
3.2	0.1	ycaM	1	31.9333	0.506
3.2	0.1	yadI	1	31.9333	0.929
3.2	0.1	AG1	1	31.9333	1.02
3.2	0.1	ptsI	1	32.1833	0.891
3.2	0.1	clcB	1	32.1833	0.436
3.2	0.1	ycaM	1	32.1833	0.507
3.2	0.1	yadI	1	32.1833	0.936
3.2	0.1	AG1	1	32.1833	1.023
3.2	0.1	ptsI	1	32.4333	0.892
3.2	0.1	clcB	1	32.4333	0.431
3.2	0.1	ycaM	1	32.4333	0.507
3.2	0.1	yadI	1	32.4333	0.931
3.2	0.1	AG1	1	32.4333	1.034
3.2	0.1	ptsI	1	32.6833	0.89
3.2	0.1	clcB	1	32.6833	0.439
3.2	0.1	ycaM	1	32.6833	0.507
3.2	0.1	yadI	1	32.6833	0.948
3.2	0.1	AG1	1	32.6833	1.032
3.2	0.1	ptsI	1	32.9333	0.895
3.2	0.1	clcB	1	32.9333	0.435
3.2	0.1	ycaM	1	32.9333	0.509
3.2	0.1	yadI	1	32.9333	0.947
3.2	0.1	AG1	1	32.9333	1.035
3.2	0.1	ptsI	1	33.1833	0.892
3.2	0.1	clcB	1	33.1833	0.431
3.2	0.1	ycaM	1	33.1833	0.508
3.2	0.1	yadI	1	33.1833	0.953
3.2	0.1	AG1	1	33.1833	1.04
3.2	0.1	ptsI	1	33.4333	0.89
3.2	0.1	clcB	1	33.4333	0.434
3.2	0.1	ycaM	1	33.4333	0.508
3.2	0.1	yadI	1	33.4333	0.964
3.2	0.1	AG1	1	33.4333	1.048
3.2	0.1	ptsI	1	33.6833	0.89
3.2	0.1	clcB	1	33.6833	0.432
3.2	0.1	ycaM	1	33.6833	0.506
3.2	0.1	yadI	1	33.6833	0.978
3.2	0.1	AG1	1	33.6833	1.055
3.2	0.1	ptsI	1	33.9333	0.89
3.2	0.1	clcB	1	33.9333	0.44
3.2	0.1	ycaM	1	33.9333	0.507
3.2	0.1	yadI	1	33.9333	0.99
3.2	0.1	AG1	1	33.9333	1.06
3.2	0.1	ptsI	1	34.1833	0.889

3.2	0.1	clcB	1	34.1833	0.437
3.2	0.1	ycaM	1	34.1833	0.512
3.2	0.1	yadI	1	34.1833	1.008
3.2	0.1	AG1	1	34.1833	1.072
3.2	0.1	ptsI	1	34.4333	0.887
3.2	0.1	clcB	1	34.4333	0.436
3.2	0.1	ycaM	1	34.4333	0.509
3.2	0.1	yadI	1	34.4333	1.015
3.2	0.1	AG1	1	34.4333	1.079
3.2	0.1	ptsI	1	34.6833	0.886
3.2	0.1	clcB	1	34.6833	0.434
3.2	0.1	ycaM	1	34.6833	0.512
3.2	0.1	yadI	1	34.6833	1.023
3.2	0.1	AG1	1	34.6833	1.092
3.2	0.1	ptsI	1	34.9333	0.887
3.2	0.1	clcB	1	34.9333	0.436
3.2	0.1	ycaM	1	34.9333	0.506
3.2	0.1	yadI	1	34.9333	1.039
3.2	0.1	AG1	1	34.9333	1.099
3.2	0.1	ptsI	1	35.1833	0.891
3.2	0.1	clcB	1	35.1833	0.438
3.2	0.1	ycaM	1	35.1833	0.508
3.2	0.1	yadI	1	35.1833	1.048
3.2	0.1	AG1	1	35.1833	1.109
3.2	0.1	ptsI	1	35.4333	0.894
3.2	0.1	clcB	1	35.4333	0.438
3.2	0.1	ycaM	1	35.4333	0.509
3.2	0.1	yadI	1	35.4333	1.058
3.2	0.1	AG1	1	35.4333	1.118
3.2	0.1	ptsI	1	35.6833	0.889
3.2	0.1	clcB	1	35.6833	0.438
3.2	0.1	ycaM	1	35.6833	0.511
3.2	0.1	yadI	1	35.6833	1.069
3.2	0.1	AG1	1	35.6833	1.127
3.2	0.1	ptsI	1	35.9333	0.892
3.2	0.1	clcB	1	35.9333	0.438
3.2	0.1	ycaM	1	35.9333	0.511
3.2	0.1	yadI	1	35.9333	1.078
3.2	0.1	AG1	1	35.9333	1.134
3.2	0.1	ptsI	1	36.1833	0.888
3.2	0.1	clcB	1	36.1833	0.441
3.2	0.1	ycaM	1	36.1833	0.519
3.2	0.1	yadI	1	36.1833	1.083
3.2	0.1	AG1	1	36.1833	1.145
3.2	0.1	ptsI	1	36.4333	0.887
3.2	0.1	clcB	1	36.4333	0.442
3.2	0.1	ycaM	1	36.4333	0.517
3.2	0.1	yadI	1	36.4333	1.095
3.2	0.1	AG1	1	36.4333	1.153
3.2	0.1	ptsI	1	36.6833	0.89
3.2	0.1	clcB	1	36.6833	0.446
3.2	0.1	ycaM	1	36.6833	0.512
3.2	0.1	yadI	1	36.6833	1.101

3.2	0.1	AG1	1	36.6833	1.159

Table S3: Survival in larvae exposed to *E. coli* KO strains

Time	n.risk	n.event	n.censor	lower	upper	Strain	Colistin	surv
0	150	0	30	1	1	BW25113	No	1
24	120	16	14	0.807931	0.929673	BW25113	No	0.866667
48	90	21	9	0.581205	0.759606	BW25113	No	0.664444
72	60	23	7	0.322237	0.521007	BW25113	No	0.409741
96	30	24	6	0.038519	0.17434	BW25113	No	0.081948
0	150	0	30	1	1	BW25113	Yes	1
24	120	7	23	0.900653	0.984548	BW25113	Yes	0.941667
48	90	9	21	0.78077	0.919933	BW25113	Yes	0.8475
72	60	10	20	0.614137	0.812179	BW25113	Yes	0.70625
96	30	10	20	0.352639	0.628642	BW25113	Yes	0.470833
0	150	0	30	1	1	clcB	No	1
24	120	15	15	0.817784	0.936219	clcB	No	0.875
48	90	17	13	0.629171	0.800586	clcB	No	0.709722
72	60	19	11	0.393037	0.598423	clcB	No	0.484977
96	30	21	9	0.081004	0.261323	clcB	No	0.145493
0	150	0	30	1	1	clcB	Yes	1
24	120	12	18	0.847893	0.955309	clcB	Yes	0.9
48	90	18	12	0.639043	0.811213	clcB	Yes	0.72
72	60	18	12	0.410942	0.618131	clcB	Yes	0.504
96	30	22	8	0.071757	0.25173	clcB	Yes	0.1344
0	150	0	30	1	1	ptsI	No	1
24	120	14	16	0.827724	0.942679	ptsI	No	0.883333
48	90	17	13	0.636079	0.807046	ptsI	No	0.716481
72	60	19	11	0.397107	0.603626	ptsI	No	0.489596
96	30	24	6	0.046454	0.2064	ptsI	No	0.097919
0	150	0	30	1	1	ptsI	Yes	1
24	120	14	16	0.827724	0.942679	ptsI	Yes	0.883333
48	90	19	11	0.614908	0.789716	ptsI	Yes	0.696852
72	60	20	10	0.373452	0.577914	ptsI	Yes	0.464568
96	30	22	8	0.065829	0.233142	ptsI	Yes	0.123885
0	150	0	30	1	1	ycaM	No	1
24	120	13	17	0.837757	0.949046	ycaM	No	0.891667
48	90	18	12	0.632249	0.804816	ycaM	No	0.713333
72	60	18	12	0.406806	0.612905	ycaM	No	0.499333
96	30	19	11	0.109614	0.305814	ycaM	No	0.183089
0	150	0	30	1	1	ycaM	Yes	1
24	120	16	14	0.807931	0.929673	ycaM	Yes	0.866667
48	90	23	7	0.560943	0.742079	ycaM	Yes	0.645185
72	60	23	7	0.311824	0.507645	ycaM	Yes	0.397864
96	30	24	6	0.037362	0.169473	ycaM	Yes	0.079573
